# AKAP79 Orchestrates a Cyclic AMP Signalosome Adjacent to Orai1 Ca^2+^ Channels

**DOI:** 10.1093/function/zqab036

**Published:** 2021-07-29

**Authors:** Pulak Kar, Pradeep Barak, Anna Zerio, Yu-Ping Lin, Amy J Parekh, Val J Watts, Dermot M F Cooper, Manuela Zaccolo, Holger Kramer, Anant B Parekh

**Affiliations:** Department of Physiology, Anatomy and Genetics, University of Oxford, Parks Road, Oxford, OX1 3PT, UK; Department of Physiology, Anatomy and Genetics, University of Oxford, Parks Road, Oxford, OX1 3PT, UK; Department of Physiology, Anatomy and Genetics, University of Oxford, Parks Road, Oxford, OX1 3PT, UK; Department of Physiology, Anatomy and Genetics, University of Oxford, Parks Road, Oxford, OX1 3PT, UK; NIEHS/NIH, 111 TW Alexander Drive, Durham, NC 27709, USA; Stoke Mandeville Hospital, Mandeville Road, Aylesbury, HP21 8AL, UK; Department of Medicinal Chemistry and Molecular Pharmacology, Purdue Institute of Drug Discovery, Purdue Institute of Neuroscience, Purdue University, West Lafayette, IN 47907, USA; Department of Pharmacology, Tennis Court Road, Cambridge CB2 1PD, UK; Department of Physiology, Anatomy and Genetics, University of Oxford, Parks Road, Oxford, OX1 3PT, UK; Proteomics and Metabolomics Centre, Medical Research Council, London Institute of Medical Sciences, London, W12 0NN, UK; Department of Physiology, Anatomy and Genetics, University of Oxford, Parks Road, Oxford, OX1 3PT, UK; NIEHS/NIH, 111 TW Alexander Drive, Durham, NC 27709, USA

**Keywords:** Store-operated calcium channels, calcium nanodomain, AKAP79, cyclic AMP, phosphodiesterase, adenylyl cyclase 8, signalosome

## Abstract

To ensure specificity of response, eukaryotic cells often restrict signalling molecules to sub-cellular regions. The Ca^2+^ nanodomain is a spatially confined signal that arises near open Ca^2+^ channels. Ca^2+^ nanodomains near store-operated Orai1 channels stimulate the protein phosphatase calcineurin, which activates the transcription factor NFAT1, and both enzyme and target are initially attached to the plasma membrane through the scaffolding protein AKAP79. Here, we show that a cAMP signalling nexus also forms adjacent to Orai1. Protein kinase A and phosphodiesterase 4, an enzyme that rapidly breaks down cAMP, both associate with AKAP79 and realign close to Orai1 after stimulation. PCR and mass spectrometry failed to show expression of Ca^2+^-activated adenylyl cyclase 8 in HEK293 cells, whereas the enzyme was observed in neuronal cell lines. FRET and biochemical measurements of bulk cAMP and protein kinase A activity consistently failed to show an increase in adenylyl cyclase activity following even a large rise in cytosolic Ca^2+^. Furthermore, expression of AKAP79-CUTie, a cAMP FRET sensor tethered to AKAP79, did not report a rise in cAMP after stimulation, despite AKAP79 association with Orai1. Hence, HEK293 cells do not express functional active Ca^2+^-activated adenylyl cyclases including adenylyl cyclase 8. Our results show that two ancient second messengers are independently generated in nanodomains close to Orai1 Ca^2+^ channels.

## Graphical Abstract

**Graphical Abstract:**
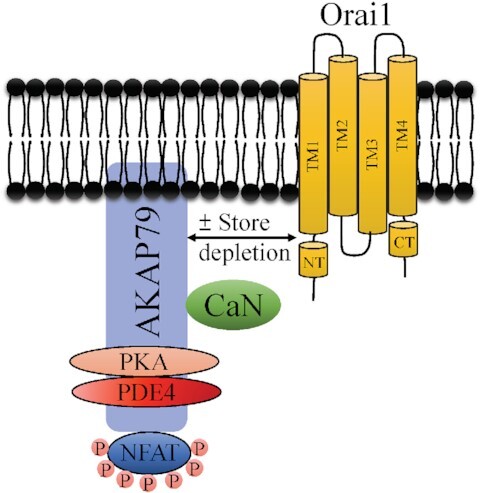

## Introduction

Increased biological complexity is accompanied by augmented spatial and temporal organization of biochemical processes. In many fundamental metabolic pathways, enzymes are not spatially segregated in the cytoplasm but physically interact to form functional multi-enzyme complexes in which the products of one reaction are readily accessible to the next enzyme in the cascade. In the protozoa *Trypanosoma brucei*, a membrane-bound organelle, the glycosome, houses all the glycolytic enzymes.^1^ In mammalian cells, membrane-bound proteins including Band3 and caveolin-1 provide nucleation sites for glycolytic enzyme complexes,^2,3^ favoring compartmentalised metabolism.

Corralling enzymes is not restricted to metabolic complexes. A kinase anchoring proteins (AKAPs) also provide a conserved and effective means for constraining enzymes close to their respective targets.^4^ AKAP79 is the prototypical AKAP and regulates important physiological processes including long-term synaptic depression,^5^ insulin secretion,^6^ and activation of the Ca^2+^-dependent transcription factor NFAT.^7^ Through its intrinsic ability to bind to protein kinases, phosphatases, and other signal transducers, AKAP79 orchestrates the formation of a local signalosome. An amphipathic α-helix on the C-terminus of AKAP79 binds directly to the regulatory RII subunits of protein kinase A.^8^ AKAP79 also has distinct binding sites for the Ca^2+^-activated protein phosphatase calcineurin and protein kinase C,^9^ calmodulin,^10^ and the Ca^2+^-dependent nuclear factor of activated T cells (NFAT) transcription factors.^11–13^ AKAP79 associates with the plasma membrane and thereby brings these signalling molecules close to cell-surface receptors and ion channels. A well-established example of ion channel regulation by AKAP79 has been provided by studies on CaV1.2 Ca^2+^ channels. AKAP79 binds to the C-terminus of the Ca^2+^ channel via a modified leucine-zipper interaction, juxtaposing protein kinase A and calcineurin with the channel.^14^ Stimulation of protein kinase A tethered to AKAP was thought to phosphorylate the channel to increase channel open probability, although this now seems to be indirect via regulation of the monomeric G protein Rad rather than through direct effects on the channel.^15^ As local Ca^2+^ builds up, calcineurin, also located on AKAP79, is activated and suppresses the enhancing effects of protein kinase A, leading to a form of Ca^2+^-dependent inactivation of the channels.^16^ The dualistic nature of the regulation of Cav1.2 by AKAP79 is therefore sequential; calcineurin-dependent dephosphorylation occurs after protein kinase A-mediated phosphorylation.

AKAP79 also interacts with non-voltage activated store-operated Ca^2+^ channels to activate selectively the Ca^2+^-dependent NFAT transcription factors.^7,13,17^ Store-operated Ca^2+^ channels are opened by depletion of intracellular Ca^2+^ stores,^18^ which typically occurs following engagement of cell surface receptors that stimulate phospholipase C to generate the second messenger inositol trisphosphate.^19^ The ensuing store depletion activates the endoplasmic reticulum (ER) Ca^2+^ sensors stromal interaction molecule 1 (STIM1) and STIM2, which then migrate to junctional ER closely apposed to the plasma membrane, where they gate open store-operated Ca^2+^ release-activated Ca^2+^ (CRAC) channels formed by the Orai1 integral membrane protein.^20^ Store depletion leads to association of AKAP79 with the N-terminus of Orai1,^7,13^ enabling Ca^2+^ nanodomains near the open CRAC channels to selectively activate calcineurin and thereby impart a private line of communication between the channels and NFAT1.^13^ Under non-stimulated conditions, NFAT1 is extensively phosphorylated and barred from the nucleus but dephosphorylation by active calcineurin results in exposure of a nuclear localization sequence, enabling NFAT to migrate into the nucleus to regulate expression of various chemokines and cytokines that shape an immune response.^21^

The interaction of AKAP79 and Orai1 will place protein kinase A close to the Ca^2+^ channel. Protein kinase A is activated by cAMP, two molecules of which bind to each of two regulatory subunits on the holoenzyme. cAMP is produced following stimulation of adenylyl cyclases (abbreviated in the literature to either ADCYs or ACs), of which there are ten known isoforms.^22^ Canonical activation of adenylyl cyclases is accomplished through the Gs heterotrimeric G protein. However, adenylyl cyclase isoforms 1, 3, and 8 are also activated directly by Ca^2+^-calmodulin.^22^ An extensive series of experiments by Cooper and colleagues have elegantly demonstrated that local Ca^2+^ entry through CRAC channels regulates cAMP production,^23^ and their demonstration that overexpressed adenylyl cyclase 8 (AC8) binds to the N-terminus of recombinant Orai1 channels in HEK293 cells^24^ provides a mechanistic explanation for the coupling between store-operated Ca^2+^ entry and adenylyl cyclase stimulation. It has recently been proposed that Ca^2+^ entry through Orai1 channels activates endogenous AC8 in HEK cells, resulting in stimulation of protein kinase A tethered to AKAP79.^25^ Protein kinase A has been suggested to then trigger Ca^2+^-dependent inactivation of Orai1 channels.^25^

Activity of cAMP is terminated by degradation to AMP by phosphodiesterases,^26^ a superfamily of enzymes with over 50 isoforms. Growing evidence suggests that the AKAP79-protein kinase A signalosome is also associated with a phosphodiesterase, ensuring locally produced cAMP is confined to the subcellular nanodomain.^27^ Whether a phosphodiesterase is associated with AKAP79 is unclear.

Here, we demonstrate that protein kinase A, calcineurin, and NFAT all associate with AKAP79 and relocate adjacent to Orai1 channels after stimulation. We also find that phosphodiesterase 4 (PDE4) is part of the AKAP79 signalosome and associates with AKAP79 in a manner dependent on protein kinase A. However, we do not find any molecular or functional evidence for expression of AC8 in HEK293 cells nor do our data support the view that store-operated Ca^2+^ entry activates endogenous Ca^2+^-sensitive adenylyl cyclases in these cells. Our data show that two ancient signalling pathways, Ca^2+^-calcineurin and cAMP-protein kinase A, are assembled close to Orai1 channels and in a manner wholly dependent on the presence of AKAP79. AKAP79 therefore orchestrates two independent signal transduction pathways in close vicinity of the same Ca^2+^ channel.

## Materials and Methods

### Cell Culture

HEK293 were purchased from ATCC (via the UK supplier LGC) and were cultured in Dulbecco's modified Eagle's medium (DMEM) (Thermo Scientific). Media were supplemented with 10% fetal bovine serum and 1% penicillin-streptomycin. Generation and maintenance of HEK293 cells in which adenylyl cyclases 3 and 6 had been knocked out using CRISPR/Cas technology is described in.^28^

### Plasmid Constructs and Transfection

Plasmids: cDNA Constructs-Orai1 and STIM1 were purchased from Origene. Orai1 was myc-tagged as previously described.^29^ NFAT1-GFP was provided by Dr Paul Worley (Johns Hopkins, USA). AKAP79-YFP was kindly provided by Dr Mark Dell'Acqua (Colorado). AKAP79-PKAmutant-YFP, lacking the protein kinase A binding domain, was synthesized by Mutagenex. HEK293 cells were transfected with Lipofectamine 2000 (Invitrogen), and then incubated in media without penicillin-streptomycin. Experiments were then carried out 24 to 48 hours after transfection.

SiRNA target sequences for AKAP79:

**Table:** 

Control siRNA- top	5′- cggTAAGGCTATGAAGAGATACctcgagGTATCTCTTCATAGCCTT Atttttg-3′
Human AKAP79 Dharmacon	GAGAUCAGCAGAAGGUAGUAA

### Co-Immunoprecipitation and Western Blotting

Twenty four hours after transfection, HEK293 cells were treated with 2 *μ*m thapsigargin in Ca^2+^-free external solution for 10 min and then lysed in 50 mM Tris-HCl (pH 7.5), 150 mM NaCl, 1% Triton X-100, 100 *μ*m sodium orthovanadate (Na_3_VO_4_), 100 *μ*m phenylmethanesulfonyl fluoride (PMSF), and 1X protease inhibitor cocktail (Sigma) with 4 mM EGTA for 10 min. Lysates were spun at 12 000 × g for 10 min, and the supernatant (1 mg protein) was used for immunoprecipitation reaction (anti-GFP agarose beads) at 4°C. After washing four times with ice cold lysis buffer, followed by resuspension in 2X SDS sample buffer, samples were heated at 95°C for 5 min and resolved by 10% SDS–PAGE and transferred to the nitrocellulose membranes. Membranes were blocked with 5% non-fat dry milk in PBS plus 0.1% Tween 20 (PBST) buffer for 1 hour at room temperature. Membranes were washed with PBST three times and then incubated with appropriate primary antibodies for 24 hour at 4°C. Total ERK2 (Santa Cruz Biotechnology), protein kinase A (Anti-PKA beta) (Abcam), AKAP79 (BD Transduction Laboratories), ADCY8 (AC8; Proteintech), and GFP (Cell signaling) primary antibodies were used at dilutions of 1:5000 (ERK2), 1:2000 (AC8), 1:1000 (PKA, AKAP79 and GFP). The membranes were then washed with PBST again and incubated with 1:2500 dilutions of peroxidase-linked anti-rabbit (Santa Cruz Biotechnology) or anti mouse IgG (BD Bioscience) for 1 hour at room temperature. After washing with PBST, the bands were detected by an enhanced chemiluminescence ECL-plus Western blotting detection system (GE Healthcare). Blots were analyzed by Image J software.

### FRET Measurements for cAMP

cAMP production in HEK cells was measured using H187 cAMP FRET probes packed in Adeno-associated Virus.^30^ FRET measurements were performed on an Olympus BX51 × 1 upright microscope equipped with 40X water immersion objective and OBS megaview CCD camera. DV2 beam splitter was used to split donor emission (CFP-480/30) and acceptor emission (YFP- 535/40) channels using a dichroic mirror at 505 wavelength. The donor and acceptor channels were projected onto separate halves of the camera and the two halves were aligned using pixel by pixel alignment using the patterned slide provided by Olympus and alignment was also reconfirmed using ImageJ. Analysis of ratiometric FRET (FRET = intensity in CFP channel/intensity in YFP channel) and FRET kinetics was calculated after subtracting background intensity using the FRET module provided with the commercial xcellence rt software by Olympus. Images were recorded with 100 ms of exposure using the cellR excitation system fitted with CFP excitation filter at 0.1 Hertz with no binning. All the FRET measurements were normalised to the initial FRET value to compare the cAMP production across experiments and aggregate data are plotted as mean ± SEM, unless indicated otherwise.

### AKAP79-cAMP FRET Probe

HEK 293 cells were infected with adenovirus encoding for the AKAP79-CUTie FRET biosensor^31^ at multiplicity of infection (MOI) of 100. After 2 hours of incubation, the medium containing the virus was removed and replaced with fresh medium. Cells were imaged 18–24 hour after infection. FRET imaging was performed as described before.^31^ Briefly, HEK293 cells expressing the FRET sensor were imaged using an epifluorescence inverted microscope (Olympus IX71) equipped with an oil immersion objective, coolSNAP HQ^2^ monochrome camera (Photometrics), and a dichroic beam-splitter, for simultaneous acquisition of both YFP and CFP emissions (Dual-view simultaneous-imaging system, DV2 MAG Biosystems, Photometrics, Tucson, AZ, USA). The FRET filter settings used were: CFP excitation filter ET436/20x, dichroic mirror 455 (Chroma Technology) in the microscope filter cube; dichroic mirror 495/24x, YFP emission filter ET535 nm, and CFP emission filter ET470 nm (Chroma Technology) in the beam splitter. Images were acquired at 10 s intervals and processed through MetaFluor software (Meta imaging series 7.1, Molecular Devices). FRET changes were calculated as the background subtracted YFP/CFP emission intensities (R) and values expressed as % FRET change. This is equivalent to the percentage of R/R0, where R is the ratio of the CFP and YFP intensities at time t and R0 is the average ratio of the first eight frames preceding the addition of thapsigargin. The signal was saturated by addition of 25 *μ*m forskolin and 100 *μ*m IBMX.

### Proximity Ligation Assay

Proximity ligation was carried out as described.^13^ HEK293 cells were transfected with AKAP-CUTie, Orai1-myc and untagged STIM1 and plated on coverslips. After 24 hour, cells were treated with thapsigargin (2 *μ*m) for 15 min. The Duolink™ In Situ Kit (Millipore Sigma) was used to probe interaction between AKAP79 and Orai1 according to the manufacturer's instructions in fixed cells. Slides were mounted using Duolink™ In Situ mounting medium with DAPI. Fluorescence was measured using a Zeiss LSM 880 microscope and puncta number were analyzed with Imaris software.

### Measurement of cAMP

Cyclic AMP was measured in cell lysates using an ELISA-based assay from Abcam, following the manufacturer's instructions. The broad cAMP phosphodiesterase inhibitor IBMX was present throughout the assay. Forskolin was applied to cells 10 min before lysis. Thapsigargin (2 *μ*m) was applied to cells for between 4 and 15 min. No increase in cAMP levels were detected at either time point.

### Protein Kinase A Activity Assay

HEK293 cells were stimulated with thapsigargin (2 *μ*m), forskolin (50 *μ*m), or ionomycin (5 *μ*m) for 20 min. Cells were then lysed and protein kinase A activity was detected using a colorimetric activity kit (Invitrogen), following the supplier's instructions. Protein kinase A substrate was phosphorylated by the kinase in the presence of ATP. Absorbance was read at 450 nm using a microplate reader.

### Mass Spectrometry

#### Sample Preparation and Trypsin Digestion

Samples were processed by Single-pot, solid-phase-enhanced sample preparation (SP3) as described previously.^32,33^ This was followed by peptide desalting using C18 spin tips (Glygen Corp).

#### Liquid Chromatography-Tandem Mass Spectrometry (LC-MS/MS) Analysis

Dried protein digests were dissolved in 0.1% trifluoroacetic acid by shaking (1000rpm,  5 min) for 5min and sonication on an ultrasonic water bath (5 min), followed by centrifugation (20 000 g,  5°C, 5 min). Liquid chromatography-tandem mass spectrometry (LC-MS/MS) analysis was carried out using an Ultimate 3000 RSLC nano liquid chromatography system (Thermo Scientific) coupled to a Q-Exactive mass spectrometer (Thermo Scientific) via an EASY spray source (Thermo Scientific). Total protein digests were injected and loaded onto a trapping column (Acclaim PepMap 100 C18, 100 *μ*m × 2 cm) for desalting and concentration at 8 *μ*L/min in 2% acetonitrile, 0.1% trifluoroacetic acid. Peptides were then eluted on-line to an analytical column (Acclaim Pepmap RSLC C18, 75 *μ*m × 75 cm) at a flow rate of 200 nL/min. Peptides were separated using a 120 min gradient, 4–25% of buffer B for 90 min followed by 25–45% buffer B for another 30 min (buffer B: 80% acetonitrile, 0.1% FA) and subsequent column conditioning and equilibration. Eluted peptides were analysed by the mass spectrometer operating in positive polarity using a data-dependent acquisition mode. Ions for fragmentation were determined from an initial MS1 survey scan at 70 000 resolution, followed by HCD (Higher Energy Collision Induced Dissociation) of the top 12 most abundant ions at 17 500 resolution. MS1 and MS2 scan AGC targets were set to 3e6 and 5e4 for maximum injection times of 50 and 50 ms, respectively. A survey scan m/z range of 400–1800 was used, normalised collision energy set to 27%, charge exclusion enabled with unassigned and + 1 charge states rejected and a minimal AGC target of 1e3.

#### Raw Data Processing

Data was processed using the MaxQuant software platform (v1.6.2.3),^34,35^ with database searches carried out by the Andromeda search engine against the Swissprot *homo sapiens* database (version 20 210 418, number of entries: 20 626). A reverse decoy database approach was used at a 1% false discovery rate (FDR) for peptide spectrum matches. Search parameters were: Maximum missed cleavages set to 2, fixed modification of cysteine carbamidomethylation and variable modifications of methionine oxidation and protein N-terminal acetylation. Label-free quantification was enabled with an LFQ minimum ratio count of 1. “Match between runs” function was used with match and alignment time limits of 0.7 and 20 min, respectively.

### Statistical Analysis

All results were expressed as means ± SEM. Two-tailed Student t test was used to compare differences between two groups in all the experiments, using GraphPad Prism and statistical significance was set at a P value of < 0.05. In all the graphs, *, **, and *** means *P* value < 0.05, 0.01, and 0.001, respectively.

## Results

### AKAP79 Co-Ordinates a **cAMP** Signalosome That Relocates Close to Orai1 Ca^2+^ Channels upon Stimulation

**Figure 1.**
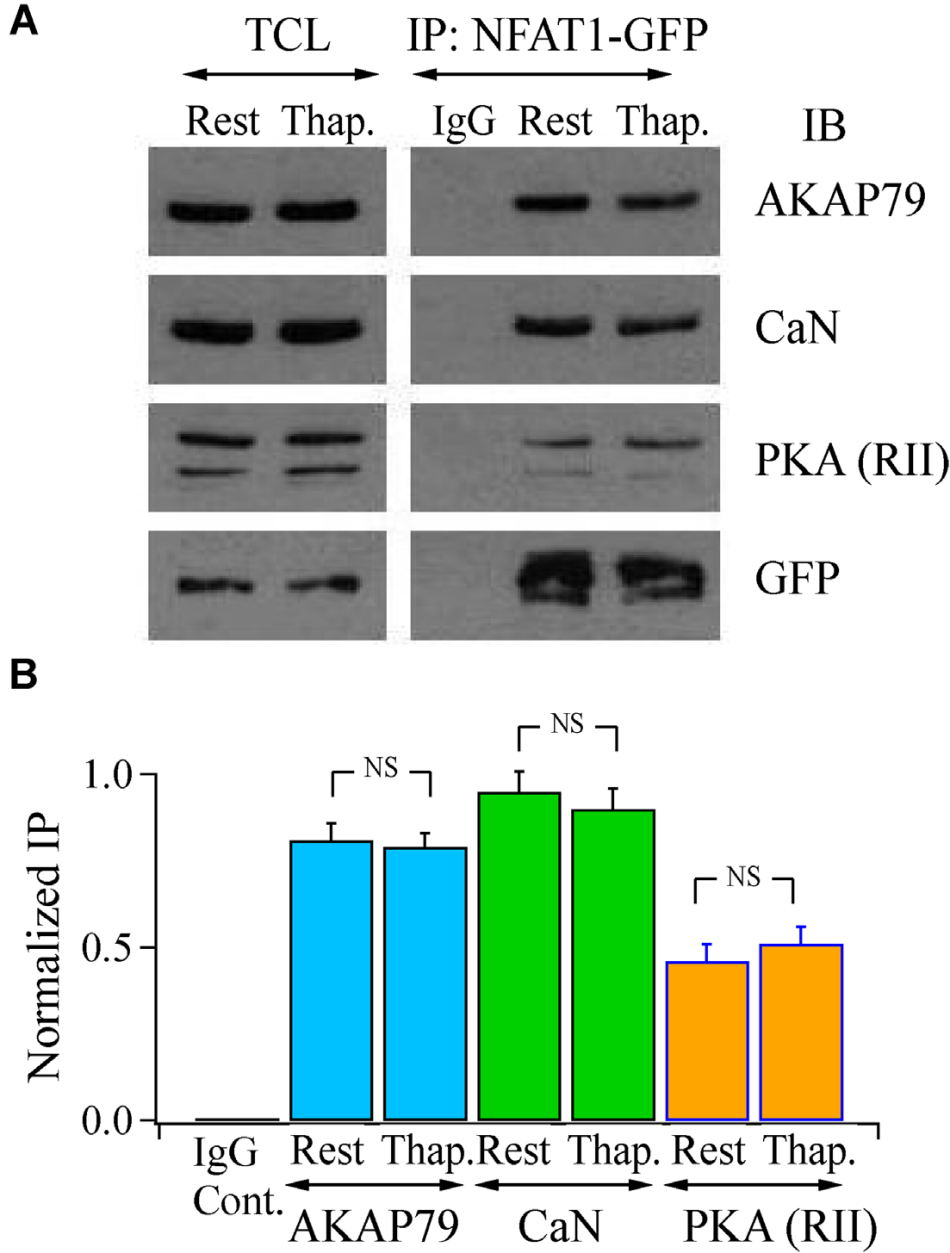
AKAP79 binds calcineurin, protein kinase A and NFAT1. (A) Pull down of NFAT1-GFP revealed the presence of calcineurin, the regulatory subunit of protein kinase A, denoted PKA (RII), and AKAP79. The signalosome remained intact after store depletion with thapsigargin (2 *μ*m, applied in Ca^2+^-free external solution). (B) Aggregate data from three independent experiments as in Panel A are shown.

Previous reports have shown that calcineurin, protein kinase A, and NFAT1 bind to AKAP79 but these studies have been conducted on different cell types or under different conditions.^7–9,11^ To address whether the phosphatase and protein kinase both associate with NFAT1 in the same cells through interaction with AKAP79 under identical conditions, we pulled down NFAT1-GFP and immuno-blotted for endogenous calcineurin, endogenous RII subunits of protein kinase A and endogenous AKAP79. NFAT1, calcineurin, and protein kinase A were all associated with AKAP79 at rest (Figure 1A). After activation of Orai1 channels following store depletion with the SERCA pump blocker thapsigargin, which was applied in the absence of external Ca^2+^ to prevent NFAT dephosphorylation and dissociation from AKAP79, the AKAP79-calcineurin-protein kinase A complex remained intact (Figure 1A and B).

Growing evidence suggests cAMP is a confined messenger, being constrained to nanodomains through breakdown by localized cAMP phosphodiesterases (PDEs).^27^ The dominant PDE in HEK cells is PDE4.^36^ We asked whether PDE4 also associated with AKAP79, thereby producing a cAMP signalosome adjacent to Orai1 channels after store depletion.

**Figure 2.**
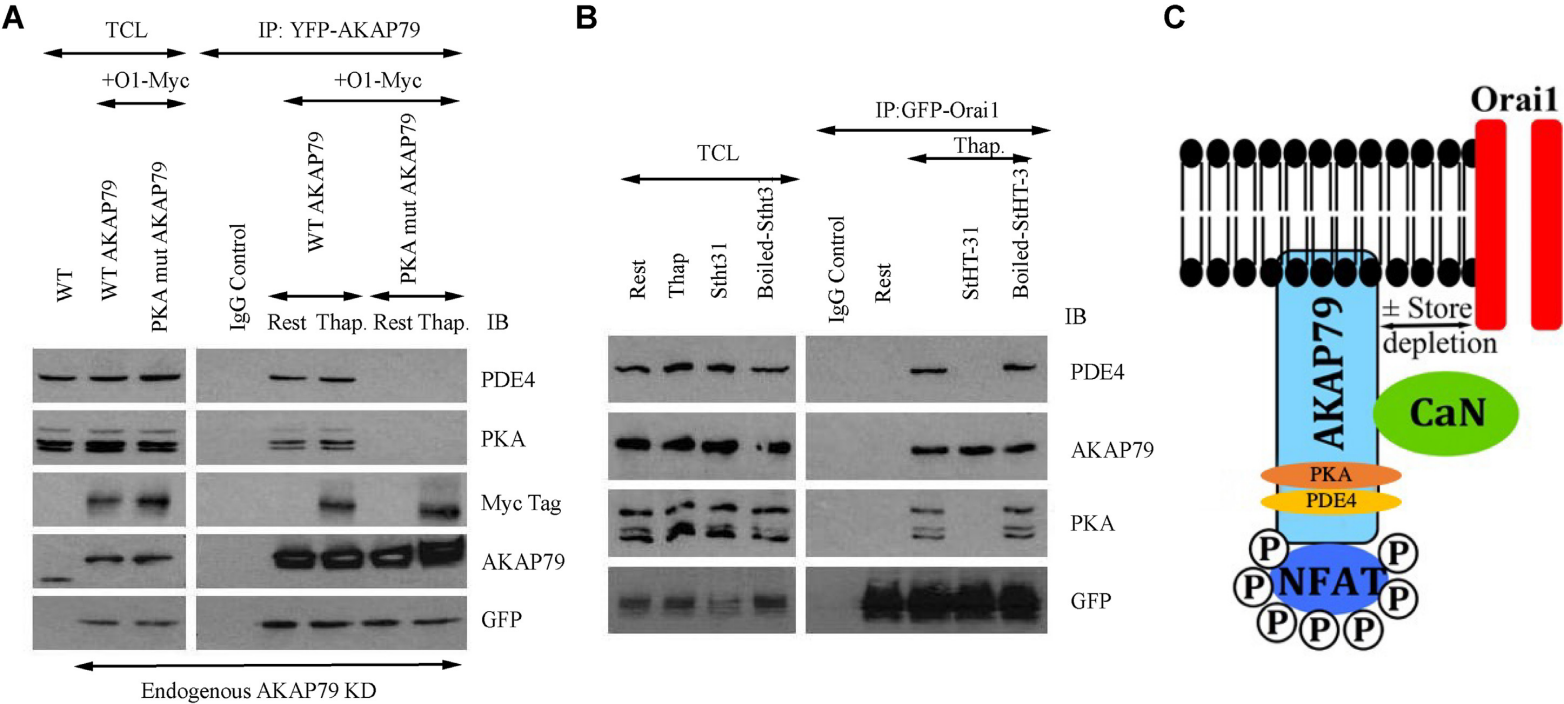
PDE4 associates with AKAP79 in a protein kinase-dependent manner. (A) Pulldown of AKAP79-YFP revealed the presence of PDE4 and protein kinase A under resting conditions as well as myc-Orai1 after stimulation with thapsigargin. PDE4 and protein kinase A failed to associate with AKAP79 when a mutant AKAP protein was expressed that lacked the protein kinase A binding site (PKA mutant AKAP79). WT denotes wild type cells. AKAP79-YFP and PKA mutant AKAP79-YFP were expressed in cells in which endogenous AKAP79 had been knocked down using an siRNA-based approach. The row labelled AKAP79 reflects bands obtained with an anti-AKAP79 antibody. The GFP row shows the extent of YFP-tagged AKAP79 expression. (B) Immunoblots compare the association of endogenous PDE4, protein kinase A, and AKAP79 with GFP-tagged Orai1 in non-stimulated cells (Rest) and then after exposure to thapsigargin for 8 min. Cells were pre-treated with StHT-31 or boiled peptide for ∼15 min prior to stimulation with thapsigargin. In Panels A and B, rest and Thap groups were obtained in the presence of 2 mM external Ca^2+^-containing solution. (C) Cartoon depicts the presence of NFAT, calcineurin, protein kinase A (PKA) and PDE4 on AKAP79 and that the signalosome can interact with Orai1 following store depletion in a reversible manner. We do not know if PKA is present as the heterotetramer or as a single heterodimer. Previous work^9^ demonstrates that both regulatory and catalytic subunits of PKA are associated with AKAP79.

In wild type cells, total cell lysate contained PDE4 (Figure 2A, left hand column labelled WT). We knocked down endogenous AKAP79 and then 24 hour later, co-expressed AKAP79-YFP and myc-tagged Orai1. We pulled down AKAP79-YFP with an anti-GFP antibody and then immunoblotted the samples with an antibody directed against endogenous AKAP79. This approach enabled us to confirm effective knockdown of endogenous AKAP79 in the pulldown samples (Figure 2A). In resting cells, pulldown of AKAP79-YFP revealed the presence of protein kinase A and PDE4 but not myc-tagged Orai1 (Figure 2A). However, after store depletion with thapsigargin, the complex additionally included Orai1 (Figure 2A). The PDE4 family can be subcategorized into long, short, and super-short isoforms. Based on the molecular weight of the PDE4 isoform we find associated with AKAP79, it is a member of the long family containing PDE4D3 and PDE4D5.^36^

Therefore under identical conditions, AKAP79 associates with calcineurin, NFAT, protein kinase A and PDE4 (Figure 2C).

### A Conformational Coupling-Type Switch Directs Protein Kinase A and PDE4 Association with AKAP79

We asked whether PDE4 association with AKAP79 was dependent on the presence of protein kinase A. Both protein kinase A and PDE4 failed to co-immunoprecipitate with mutant AKAP79 that lacked the protein kinase A binding site (Figure 2A), suggesting PDE4 association with AKAP79 was linked to the protein kinase A site. To test this further, we exposed cells to a membrane-permeable peptide which dislodges protein kinase A from AKAP79. The stearated membrane-permeable 24 amino acid peptide HT-31 (denoted StHT-31) inhibits the interaction between the regulatory RII subunits of cAMP-dependent protein kinase and AKAP79^37^ by dissociating protein kinase A from an amphipathic helix located between residues 392 and 405 on AKAP79. If protein kinase A tethered to AKAP79 was required for PDE4 binding, as suggested by the data in Figure 2A, a prediction would be that pre-treatment with St-HT31 should displace protein kinase A along with PDE4. This was indeed the case; in cells pre-exposed to StHT-31, neither protein kinase A nor PDE4 remained associated with AKAP79 (Figure 2B). St-HT31 was ineffective in preventing protein kinase A or PDE4 association with AKAP79 when the peptide was denatured by prior boiling (Figures 2B).

Calcineurin and NFAT dissociate from AKAP79 following Ca^2+^ entry through CRAC channels.^7^ We asked whether protein kinase A and PDE4 also escaped from AKAP79 after Ca^2+^ influx or stayed associated with the scaffold protein. Pull down of AKAP79 after opening of Orai1 channels in the presence of external Ca^2+^ revealed that protein kinase A and PDE4 both remained on Orai1 (Figure 2A), and at levels undiminished from the resting state. Therefore, protein kinase A and PDE4 are retained on AKAP79 following Ca^2+^ entry.

### AC8 is not Expressed at Detectable Molecular or Functional Levels in HEK 293 Cells

**Figure 3.**
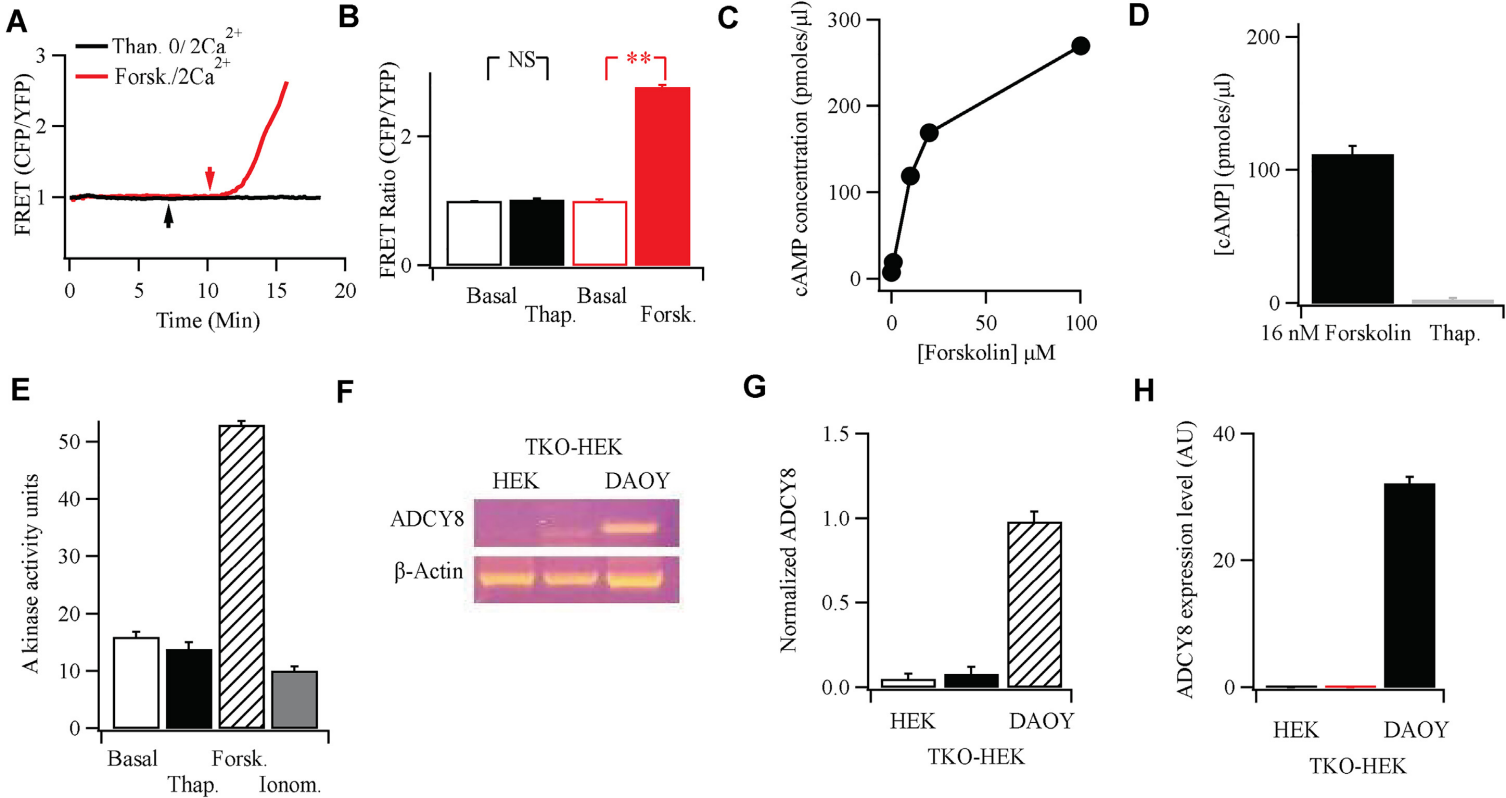
Functional and molecular evidence against endogenous Ca^2+^-activated adenylyl cyclase 8 in HEK cells. (A) FRET measurements show no rise in cAMP following Ca^2+^ readmission (2 mM; arrow) to cells bathed in thapsigargin (2 *μ*m) in Ca^2+^-free solution. Stimulation with forskolin (50 *μ*m) gave a substantial FRET signal. Upward deflection indicates an increase in cAMP. (B) Aggregate data are compared from experiments as in Panel A. Basal denotes FRET signal under non-stimulated conditions, averaged for 60 s prior to stimulation. Thapsigargin data were measured after readmission of external Ca^2+^. (C) Forskolin evokes a dose-dependent increase in cAMP, measured using an ELISA-based system. (D) Thapsigargin fails to increase cAMP, detected using ELISA. IBMX was present prior to and during thapsigargin stimulation. Thapsigargin was applied at 2 *μ*m for 10 min. The thapsigargin response is compared with the effect seen with a sub-maximal dose of forskolin (16 nM). (E) Protein kinase A activity is compared for the conditions shown. Basal denotes activity in the absence of stimulation (cells exposed to DMSO solvent control), thapsigargin (2 *μ*m), forskolin (50 *μ*m), and ionomycin (5 *μ*m) were all applied in 2 mM Ca^2+^-containing external solution for 10 min prior to measurement of enzyme activity. F, RT-PCR reveals expression of AC8 in the DAOY neuronal cell line, but not in either wild type HEK293 cells or HEK293 cells in which all three Orai genes had been knocked out.^13^ (G) Aggregate data from three independent experiments are summarised in the bar chart. (H) qPCR was used to detect AC8 in the various cell types shown.

AKAP79 brings protein kinase A and PDE4D3/D5 close to the Orai1 channel upon stimulation (Figure 2). To complete the triumvirate for cAMP signaling, we sought to identify the adenylyl cyclase. In HEK293 cells, it has been proposed that endogenous AC8, a Ca^2+^-activated isoform, is tethered to the N-terminus of Orai1 and is stimulated by local Ca^2+^ entry through the channels.^25^ The local production of cAMP was then suggested to activate protein kinase A, tethered to AKAP79, to cause Ca^2+^-dependent fast inactivation of CRAC channels.^25^ To see whether Ca^2+^-activated AC8 was functional in HEK293 cells, we directly measured cAMP production following CRAC channel activation using a fourth generation EPaC FRET probe.^30^ Exposure to forskolin led to a robust increase in cAMP but activation of CRAC channels with thapsigargin failed to generate any detectable rise, either in the absence or presence of external Ca^2+^ (Figure 3A and B). To test this further, we measured cAMP levels using an ELISA-based detection system. Exposure to forskolin resulted in a concentration-dependent increase in cAMP (Figure 3C). However, stimulation with thapsigargin in the presence of external Ca^2+^ failed to increase cAMP levels, even when cells had been pre-treated with the broad phosphodiesterase inhibitor IBMX to increase detection (Figure 3D). If cAMP was produced following CRAC channel opening, then this should be reflected through an increase in protein kinase A activity. Whereas forskolin stimulated protein kinase A activity within 4 min of stimulation, thapsigargin failed to evoke an increase up to 15 min of exposure (the longest period we measured; Figure 3E). A large global rise in cytosolic Ca^2+^, evoked by stimulation with the Ca^2+^ ionophore ionomycin, also failed to increase protein kinase A activity (Figure 3E).

**Figure 4.**
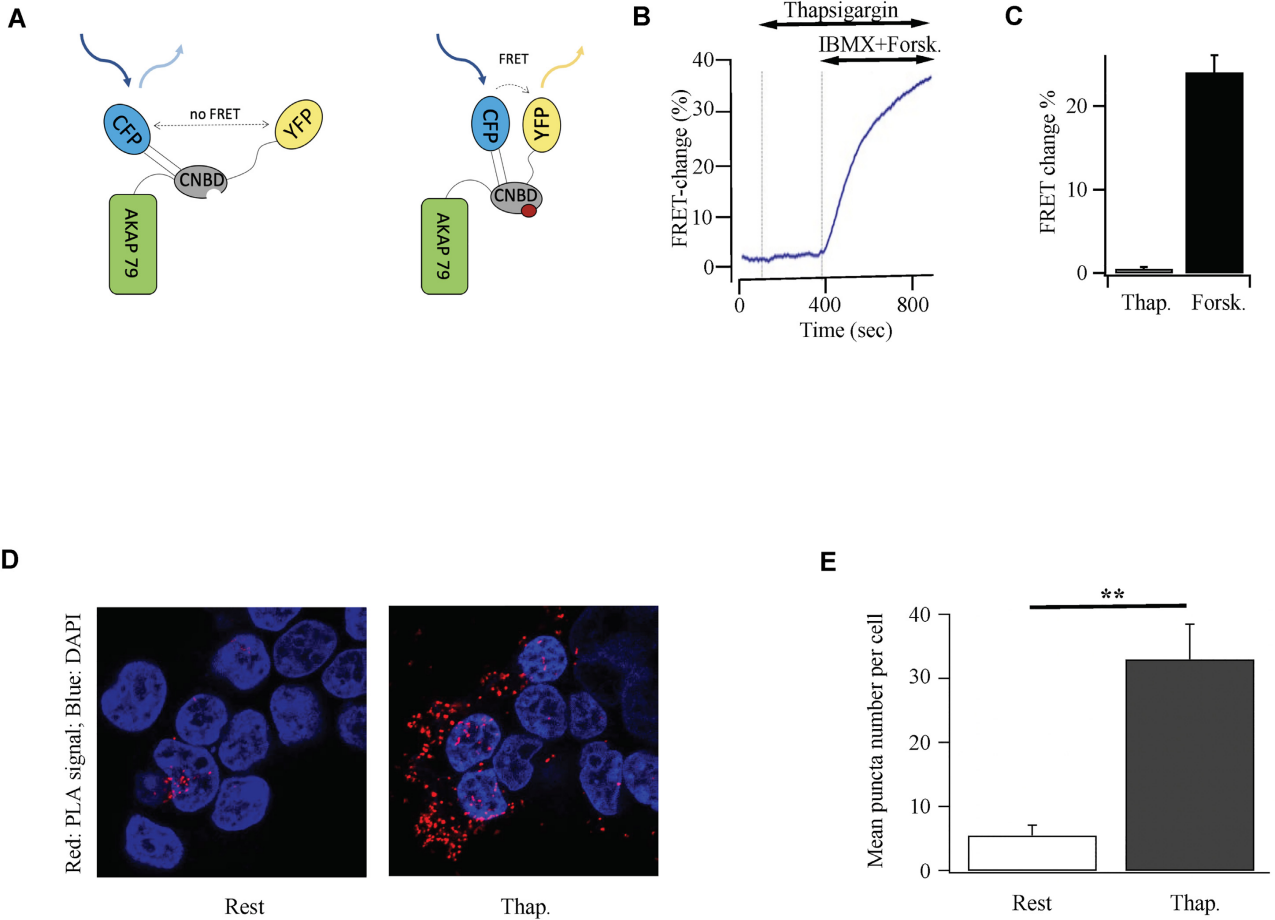
Store-operated Ca^2+^ entry does not generate a detectable cAMP rise near adjacent AKAP79. (A) Cartoon depicts the principle underlying FRET between CFP and YFP for plasma-membrane targeted AKAP79-CUTie. CNBD denotes cyclic nucleotide binding domain. (B) Stimulation with thapsigargin failed to generate a FRET signal from AKAP79-CUTie whereas a robust response was seen following stimulation with forskolin and IBMX. (C) Aggregate data from 6 cells from 3 independent experiments are shown. (D) Proximity ligation assay shows increased association of AKAP79-CUTie with Orai1-myc after stimulation in HEK293 cells. Images compare proximity of AKAP79-CUTie and Orai1-myc in HEK293 cells at rest and then after 15 min exposure to thapsigargin. Nuclei were stained with DAPI. (E) Aggregate data from experiments as in Panel B are compared (>12 cells per bar).

We considered the possibility that cAMP was rapidly broken down near the cell surface and therefore was undetectable in our bulk cytoplasmic (Figure 3A and B) and cell population (Figure 3C and D) measurements. We therefore took advantage of AKAP79-CUTie, a cAMP FRET probe which is attached directly to AKAP79 and localizes to the plasma membrane^31^ (Figure 4A). Since AKAP79 co-localises with Orai1 after store depletion,^7,13,17^ AKAP79-CUTie should be located close to the Ca^2+^ channel and therefore detect cAMP potentially produced by juxtaposed AC8 following store-operated Ca^2+^ entry. However, no FRET increase was detected following stimulation with thapsigargin (Figure 4B and C). Importantly, stimulation with forskolin increased the FRET signal (Figure 4B and C), demonstrating the probe was able to measure cAMP near the channels.

We used a proximity ligation assay to evaluate whether Orai1 colocalised with AKAP79-CUTie after stimulation. In this assay, proximity of two proteins is reflected in the presence of discrete spots. In resting cells, little overlap of AKAP79-CUTie and Orai1-myc was detected (Figure 4D). However, after stimulation with thapsigargin, significantly more spots were observed (Figure 4E), reflecting increased co-localization between AKAP79-CUTie and Orai1-myc. AKAP79-CUTie is located close to Orai1 after stimulation and therefore is ideally positioned to detect any cAMP generated by AC8 tethered to the N-terminus of the channels.

Due to the absence of any functional AC8, we asked whether the enzyme was expressed in HEK293 cells. We conducted both RT-PCR (Figure 3F and G) and qPCR (Figure 3H) but both methods failed to detect any AC8. However, the same primers revealed prominent expression in the human neuronal cell line DAOY (Figure 3F–H) and human glioblastoma U87-MG cells. AC8 was also undetectable in a HEK 293 cell line in which all three Orai genes had been knocked out^13^ (Figure 3F and G).

### Mass Spectrometry Analysis Fails to Detect AC8 in HEK293 Cells

**Figure 5.**
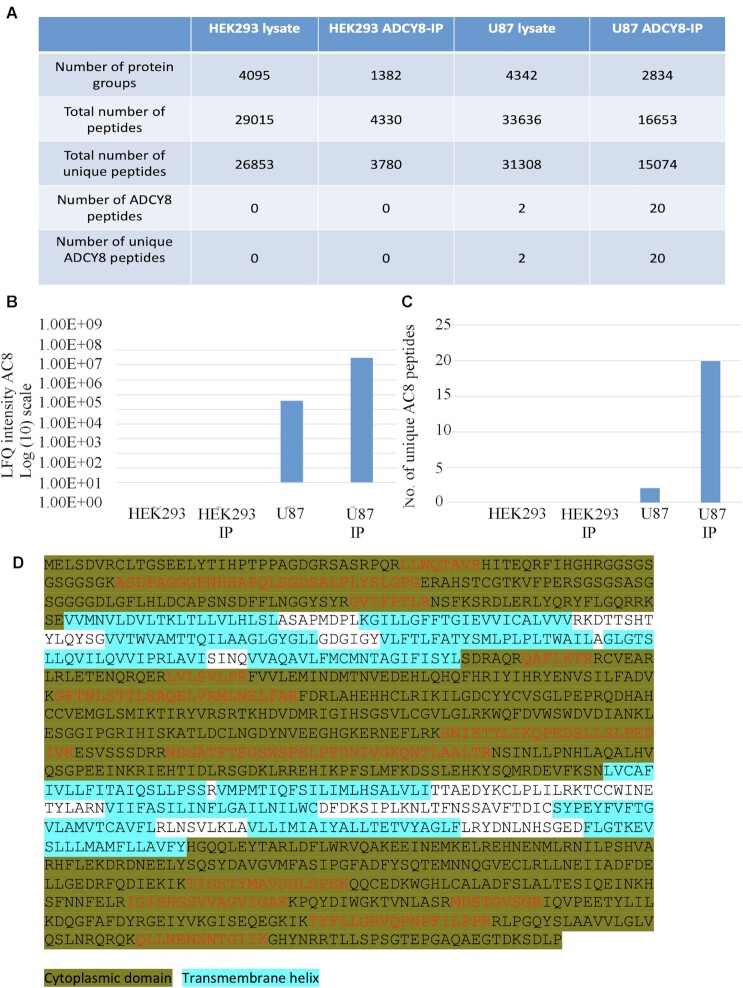
Identification of human AC8 (ADCY8; Swissprot ID: P40145; ADCY8_HUMAN Adenylate cyclase type 8) by liquid chromatography-tandem mass spectrometry (LC-MS/MS) analysis in U87 cells but not HEK293 cells. (A) Summary of proteomic data sets generated by trypsin digestion and LC-MS/MS analysis of HEK293, HEK293 cells in which immuno-precipitation with anti-AC8 antibody had been carried out, U87 cells and U87 samples which had been obtained by immuno-precipitation. Shown are the number of protein groups quantified, total number of peptides identified, total number of unique peptides identified, number of AC8 peptides identified and number of unique AC8 peptides identified. All results were generated in MaxQuant (Andromeda search algorithm) and are displayed at a FDR of 1% (FDR 0.01), which was obtained through a target-decoy database search approach. The AC8-IP heading in the table denotes immune-precipitated AC8. (B) Quantified protein raw intensity as generated in MaxQuant software are compared for the different conditions. (C) The number of peptides specific to ADCY8 (AC8) using trypsin digestion are shown. (D) Data base searching (MASCOT algorithm) following isolation from U87 cell lysate by immunoprecipitation identified human ADCY8 with a protein score of 348 (FDR: 1%). Sequence coverage overview showing tryptic peptides detected in red font. Highlighted regions show cytoplasmic domains and transmembrane helices, respectively. The overall sequence coverage achieved is 17%; sequence coverage within the cytoplasmic domains is 24%.

Because AC8 was reported using western blot in HEK293 cells,^25^ we used mass spectrometry to assess whether the protein was expressed in this cell type. Analysis of HEK 293 total cell lysate failed to reveal any AC8, whereas the protein was detectable in lysate from U87-MG glioblastoma cells run in the same way (Figure 5A). To increase the likelihood of detecting AC8, should it be present, we pulled down endogenous AC8 from HEK293 cells using the same polyclonal antibody against AC8 that was used in the earlier study.^25^ However, no AC8 was detected in the immuno-precipitate (Figure 5A and B). Pull down with the same antibody using U87-MG cells revealed the presence of AC8 (Figure 5B) and numerous peptides were detected that arose from enzymatic digestion of AC8 cytoplasmic domains (Figure 5C and D). We compared enrichment in the pulldown from U87 cells relative to the input (U87 lysate digest). The log(2) fold change of normalised LFQ intensity was 9.8 for AC8 (IP/lysate), showing that pulldown augmented the presence of AC8. Nevertheless, the protein was still undetectable in HEK293 cells even after following a protocol that would lead to enrichment, should AC8 be present. The mass spectrometry data show that AC8 is undetectable in HEK293 cells when profiled to a depth of proteome coverage of over 4000 proteins. A comprehensive list of all proteins detected under the different conditions is shown in Supplemental Material.

### Identification of the Adenylyl Cyclase in HEK293 Cells

**Figure 6.**
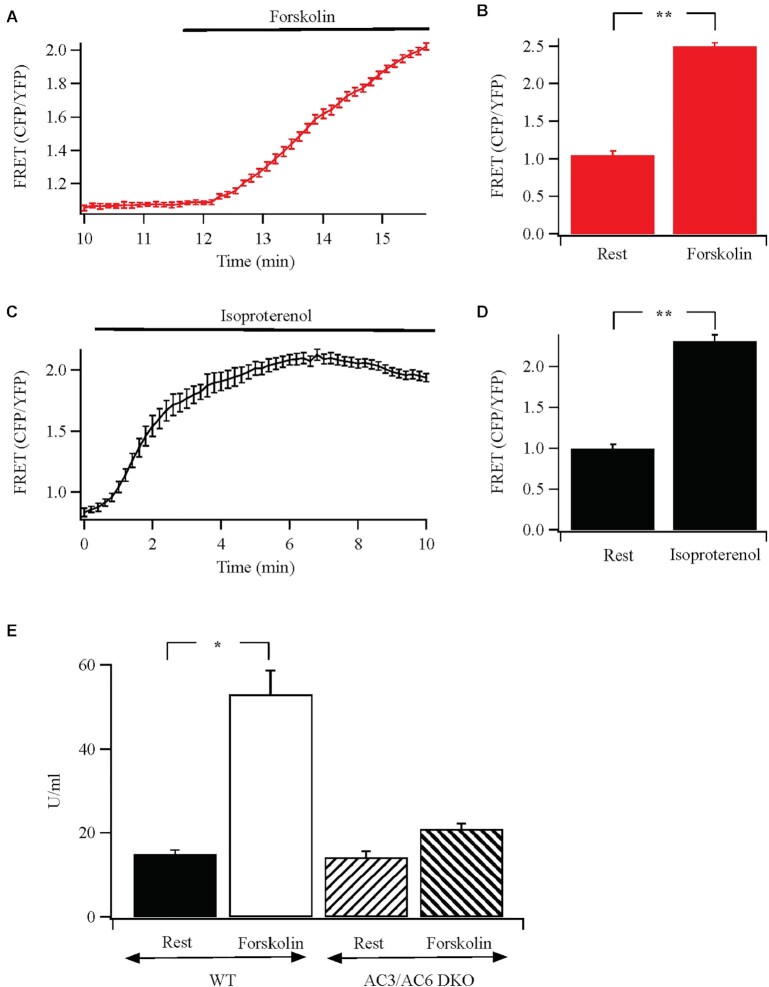
Identifying the forskolin-sensitive adenylyl cyclase in HEK293 cells. (A) Stimulation with forskolin evokes an increase in cAMP, measured with the H187 FRET probe. (B) Aggregate data are compared (each bar is the mean of between 8 and 11 cells). Rest denotes the resting condition prior to forskolin treatment. (C) Activation of β1 adrenoceptors with 100 *μ*m isoproterenol produces a robust increase in cAMP. (D) Aggregate data from 7–10 cells are compared. (E) Forskolin-stimulated protein kinase A activity is almost completely suppressed in AC3/AC6 double knockout (DKO) cells.

We designed experiments to identify the adenylyl cyclase in HEK293 cells that could generate cAMP adjacent to protein kinase A tethered to AKAP79. Adenylyl cyclase 2, 3, 5, 6, 8, and 9 can associate with AKAP79 but the isoforms are regulated differently in HEK293 cells.^38^ Exposure of tethered AC8 or AC9 to forskolin and Gαs failed to increase enzyme activity.^38^ Stimulation with forskolin (Figure 6A and B) or recombinant β1 receptors with isoproterenol (Figure 6C and D), which couple to Gαs, both increased cAMP levels and to similar extents in HEK293 cells, arguing against a major role for either AC8 and AC9. The dominant isoforms in HEK293 cells are AC3 and AC6, and CRISPR/Cas knockout of these proteins completely abolishes cAMP production to forskolin.^28^ Knockout of AC6 alone was found to reduce forskolin-stimulated adenylyl cyclase activity by ∼85%, demonstrating this isoform plays the major role in cAMP production.^28^ We therefore used the AC3/AC6 double knockout to see whether forskolin was still able to stimulate cAMP-dependent protein kinase A. Compared with wild type cells, where a robust increase in protein kinase A activity was observed following exposure to forskolin, the increase was considerably smaller in the AC3/AC6 double KO cells (reduced by >80%; Figure 6E). These results are in excellent agreement with Soto-Velasquez et al.^28^ and show that the dominant forskolin-sensitive membrane adenylyl cyclases are AC6 and AC3.

## Discussion

AKAP79 choreographs formation of a signalosome at the plasma membrane that enables Ca^2+^ nanodomains near open Orai1 Ca^2+^ channels to drive activation of the NFAT transcription factors selectively and with high fidelity.^7,13,39^ Through multiple protein–protein interactions, AKAP79 positions calcineurin and NFAT adjacent to the N-terminus of Orai1 after store depletion, placing the signalling nexus in the realm of the local Ca^2+^ signal. Ca^2+^ entry through Orai1 channels stimulates calcineurin tethered to AKAP79, resulting in dephosphorylation and activation of proximal NFAT molecules.^7,13,39^

Our new data demonstrate that a cAMP signalosome also forms adjacent to Orai Ca^2+^ channels after stimulation, juxtapositioning protein kinase A and PDE4D3/D5 adjacent to the Ca^2+^ nanodomain that builds up rapidly when the channels open. Orai1 therefore sits at the centre of a signalling nexus involving two ancient second messenger pathways, Ca^2+^ and cAMP, opening up new mechanisms to tightly control downstream targets through a local interplay between Ca^2+^, Ca^2+^-calcineurin, and protein kinase A.

In addition to protein kinase A and PDE, a typical cAMP signalosome requires an adenylyl cyclase isoform to generate cAMP and a receptor to activate the adenylyl cyclase. G protein-coupled receptors have been found to associate with AKAP79 in HEK293 cells, including the relaxin peptide receptor^40^ and the muscarinic type 3 receptor.^41^ It is noteworthy in this context that β1 adrenoceptors have also been found to associate with AKAP79 in HEK293 cells^42^ and we have observed co-localization between the recombinant receptor and AKAP79 in the absence and presence of isoproterenol (data not shown). This tight coupling where receptor, adenylate cyclase (see below) and PDE4 are all clustered together should lead to local transient increases in cAMP. The sustained increase in cAMP production we have found following stimulation of the β1 receptors likely reflects the presence of some overexpressed receptors located away from AKAP79 and therefore generating cAMP in the absence of juxtaposed PDE4. It is tempting to speculate that the AKAP79: receptor ratio might contribute to the extent of local cAMP signaling, with a high ratio favoring the generation of cAMP nanodomains and a low ratio leading to a more global rise.

Ten isoforms of AC have been found, nine of which (ACs1-9) are transmembrane proteins.^38^ The nine membrane-associated ACs are regulated by forskolin and the canonical Gs pathway to varying extents but they are differentially regulated by cytosolic Ca^2+^. ACs 1, 3, and 8 are activated by Ca^2+^-calmodulin, ACs 5 and 6 are inhibited by Ca^2+^ and ACs 2, 4, and 7 are insensitive to Ca^2+^.^22^ AC8 has a very restricted tissue distribution. Analysis of mRNA expression from the Human Protein Atlas shows that it is almost exclusively expressed in certain regions of the brain, epididymis, testis, Fallopian tube, and placenta. Protein expression follows a similar profile, although there are concerns with the specificity of anti-AC8 antibodies (see below). Strikingly, AC8 is absent in various immune cells including T and B lymphocytes, monocytes, and dendritic cells, which all show fast Ca^2+^-dependent inactivation of CRAC channels.

Recently, it has been reported that AC8 is endogenously expressed in HEK293 cells and is activated by Ca^2+^ entry through CRAC channels to stimulate protein kinase A tethered to AKAP79.^25^ The kinase was then suggested to phosphorylate Orai1 and cause fast Ca^2+^-dependent inactivation of the channels.^25^ However, a substantial body of evidence from multiple groups over several years has consistently failed to detect endogenous AC8 in HEK293 cells.^43^ These include the complete absence of AC8 mRNA using PCR,^28,44–47^ the absence of cAMP production even in the presence of PDE inhibition and a large rise in cytosolic Ca^2+^, measured using sensitive FRET probes^24^ or biochemical assays^23,28,48,49^ and the absence of detectable AC8 protein. Commercial antibodies are not thought specific enough to discriminate between various AC isoforms and are plagued by off-target effects.^50–52^ Antoni et al. reported contradictory data with commercial adenylyl cyclase isoform-specific antibodies even between different batches of the same antibody from the same commercial supplier.^50^ To circumvent this, Cooper and colleagues generated a polyclonal antibody against human AC8. Whereas the antibody detected overexpressed protein, endogenous enzyme was undetectable in HEK293 cells.^49^ Furthermore, mass spectrometry studies on HEK293 cells have failed to detect any AC8, despite broad coverage of >10 000 proteins and detection of AC8 in other cell types.^53^ Instead, the mass spectrometry analysis identified the presence of ACs 3, 5, 6, and 9 in HEK293 cells. Our data are in excellent agreement with these studies. We failed to detect the presence of AC8 using RT-PCR or qPCR or functionally either through Ca^2+^-dependent cAMP production or an increase in protein kinase A activity following Ca^2+^ entry through CRAC channels. We also were unable to detect an increase in cAMP in stimulated cells using AKAP79-CUTie,^31^ a cAMP FRET probe targeted to AKAP79, which associated with Orai1 after store depletion. Our proximity ligation assay data show that AKAP79-CUTie and Orai1-myc re-align to within 50 nm of one another after stimulation, the typical distance for ligation assay-dependent signals. Our mass spectrometry analyses on pull down samples of AC8 with a commercial antibody used in the earlier study^25^ or in total cell lysate were also unable to detect AC8 in HEK293 cells, although the protein was identified in a neuronal cell line that expressed mRNA for AC8. Zhang et al.^25^ reported the robust expression of AC8 based on western blots with one commercial antibody, which we find does not detect AC8 in HEK293 cells. Expression of recombinant human AC8 in HEK293 cells produces a diffuse protein band of ∼165 KDa, which is reduced to ∼125 kDa after deglycosylation.^49^ However, the anti-AC8 antibody used by Zhang et al.^25^ detected a protein band with molecular weight of ∼145 KDa. Zhang et al.^25^ also did not measure AC8 mRNA levels, did not show that store-operated Ca^2+^ entry increased either cAMP production or protein kinase A activity or confirm the presence of AC8 using alternative approaches, such as mass spectrometry analysis.

We cannot rule out the possibility that a very small amount of AC8 is expressed in HEK293, beyond the levels of detection of the various methods we and numerous other groups have used. In this context, it is noteworthy that the regulatory subunit in the protein kinase A holenzyme has a very similar K_D_ value for cAMP as AKAP79-CUTie, both ∼ 7 *μ*m.^31,54^ The fact that we could not detect a cAMP increase near the AKAP79-CUTie FRET probe, which co-localizes with Orai1 after stimulation, demonstrates, should Ca^2+^-dependent cAMP production occur only in a nanodomain near Orai1 channels, it is nevertheless insufficient to activate local protein kinase A and therefore would not evoke a functional response.

Strong evidence favors a major role for AC6 as the dominant isoform in HEK293 cells that is regulated by forskolin.^28^ Knockout of AC6 inhibited cAMP production induced by forskolin by ∼85% and the combined knockout of AC3 and AC6 nearly abolished forskolin- and agonist-evoked cAMP production.^28^ Consistent with this view, we found that activation of protein kinase A by forskolin was suppressed by >80% in AC3/AC6 double knockout cells. Interestingly, AC6 has been found to associate with AKAP79,^38^ placing it adjacent to protein kinase A and PDE4D3/D5. AC6 activity is inhibited by Ca^2+^ entry through store-operated channels so the enzyme would not be activated directly by Ca^2+^ entry, although its activity can be potentiated in a Ca^2+/^calmodulin- dependent fashion.^22^ Store depletion can activate AC activity through a process that involves STIM1.^55^ In NCM460 colonic epithelial cells, the store-operated adenylyl cyclase is AC3^56^ and in B16 cells, STIM1 has been found to physically interact with AC6.^57^

What could be the functional role of protein kinase A when the AKAP79 complex associates with Orai1 after store depletion? NFAT is phosphorylated by protein kinase A on three serine residues, which prevents dephosphorylation by calcineurin and subsequent nuclear migration.^58^ By placing the transcription factor and its negative regulator in close apposition, the cell can ensure that NFAT is maintained in the off-state. On the other hand, this mechanism would be turned off when Ca^2+^ entry through Orai1 channels occurs because AC6 is strongly inhibited by store-operated Ca^2+^ entry.^22^ Such an arrangement will help ensure that the actions of calcineurin to dephosphorylate (and thereby activate) NFAT are not circumvented by concurrent activation of a cAMP-generating G protein-coupled receptor.

The conventional view holds that active protein kinase A catalytic subunits, once released from their regulatory partners, are free to diffuse in the cytosol and phosphorylate a range of targets. This concept has been challenged by Scott and colleagues who demonstrated that the protein kinase A holoenzyme is retained on AKAP79 in the presence of cAMP and thus the active kinase remains immobilised, phosphorylating targets only within an arc of 150–250 angstroms.^59^ Our finding that the regulatory subunit of protein kinase A remains associated with AKAP79 following stimulation is consistent with the model of Smith et al.^59^ In addition, our demonstration that PDE4D3/D5 also associates with AKAP79 in a protein kinase A-dependent manner adds a further mechanism to ensure spatially confined cAMP signalling. Co-localisation of PDE4D3/D5 with protein kinase A on AKAP79 will ensure only cAMP generated locally will be able to activate protein kinase A. With a sizeable fraction of cAMP emanating from AC6, possibly tethered to AKAP79, cyclic AMP spill over to distal sites will be prevented by local PDE4D3/D5. Conversely, distally generated cAMP will be impeded from reaching protein kinase A by the high local PDE activity. The combination of spatially confined cAMP nanodomains around AKAP79 with retention of active protein kinase A on the scaffolding protein ensures the kinase only operates on a nanodomain scale.

## Supplementary Material

zqab036_Supplemental_File

## Data Availability

The data underlying this article will be shared on reasonable request to the corresponding author.
