# Supplementary material for: AKAP79 Orchestrates a Cyclic AMP Signalosome Adjacent to Orai1 Ca2+ Channels
Source: Function (Oxf). 2021 Jul 29;2(5):zqab036. doi: 10.1093/function/zqab036 (PMC8394516; doi:10.1093/function/zqab036)
Supplement: zqab036_Supplemental_File [file zqab036_supplemental_file.pdf]

Supplementary Table_1

Protein identification and quantification data from the MaxQuant analysis of LC-MS/MS runs of tryptic digests from ADCY8 immunoprecipitates from HEK293 and U-87 cells, as well as the corresponding input material (cell lysates). Shown are significant protein identifications with 1% false discovery rate (target-decoy search, FDR 0.01). Quantification data shown as protein raw intensities ('Intensity' columns) and normalized label-free quantification intensities, as described in reference [1] ('LFQ intensity' columns).

[1] Cox, J., Hein, M. Y., Luber, C. A., Paron, I., Nagaraj, N. & Mann, M. (2014) Accurate proteome-wide label-free quantification by delayed normalization and maximal peptide ratio extraction, termed MaxLFQ, *Molecular and Cellular Proteomics.* **13**, 2513-2526.

Protein IDs	Majority protein IDs	Protein names	Gene names	Fasta headers	Peptides	Razor + unique peptides	Unique peptides	Peptides HEK293_ADCY8-IP	Peptides HEK293_lysate	Peptides U87_ADCY8-IP	Peptides U87_lysate	Razor + unique peptides HEK293_ADCY8-IP	Razor + unique peptides HEK293_lysate	Razor + unique peptides U87_ADCY8-IP	Razor + unique peptides U87_lysate	Unique peptides HEK293_ADCY8-IP	Unique peptides HEK293_lysate	Unique peptides U87_ADCY8-IP	Unique peptides U87_lysate	Sequence coverage [%]	Mol. weight [kDa]	Q-value	Score	Intensity	Intensity HEK293_ADCY8-IP	Intensity HEK293_lysate	Intensity U87_ADCY8-IP	Intensity U87_lysate	LFQ intensity HEK293_ADCY8-IP	LFQ intensity HEK293_lysate	LFQ intensity U87_ADCY8-IP	LFQ intensity U87_lysate
P04406	P04406	Glyceraldehyde-3-phosphate dehydrogenase	GAPDH	sp|P04406	25	25	25	10	21	21	25	10	21	21	25	10	21	21	25	85.1	36.053	0	323.31	2.828E+11	3.68E+09	6.47E+10	3.277E+09	2.11E+11	3.16E+09	4.19E+09	1.266E+09	7.99E+09
P63261	P63261	Actin, cytoplasmic 2;Actin, cytoplasmic 2, N-termi	ACTG1	sp|P63261	26	26	1	14	23	26	25	14	23	26	25	1	1	1	1	89.6	41.792	0	323.31	2.255E+11	3.3E+09	7.04E+10	1.073E+10	1.41E+11	1.87E+09	6.19E+09	5.629E+09	7.37E+09
P14618;P3	P14618	Pyruvate kinase PKM	PKM	sp|P14618	45	45	45	11	41	29	45	11	41	29	45	11	41	29	45	76.6	57.936	0	323.31	2.043E+11	2.62E+08	2.6E+10	754850000	1.77E+11	2.37E+08	2.21E+09	453110000	6.89E+09
P35579;RE	P35579	Myosin-9	MYH9	sp|P35579	156	156	134	58	111	125	155	58	111	125	155	52	94	108	134	63.5	226.53	0	323.31	1.703E+11	4.3E+09	1.02E+10	4.282E+09	1.51E+11	4.02E+09	9.91E+08	3.262E+09	5.52E+09
P06733	P06733	Alpha-enolase	ENO1	sp|P06733	30	30	28	8	30	27	30	8	30	27	30	7	28	25	28	71	47.168	0	323.31	1.599E+11	3.24E+08	3.7E+10	588650000	1.22E+11	3.77E+08	2.96E+09	376650000	4.7E+09
P68363;Q9	P68363	Tubulin alpha-1B chain	TUBA1B	sp|P68363	27	27	0	10	27	23	27	10	27	23	27	0	0	0	0	69.6	50.151	0	323.31	1.345E+11	1.21E+09	3.74E+10	3.348E+09	9.25E+10	1.22E+09	2.88E+09	2.631E+09	3.59E+09
P68104;Q5	P68104;Q5	Elongation factor 1-alpha 1;Putative elongation fa	EEF1A1;EE	sp|P68104	25	25	13	9	23	19	24	9	23	19	24	3	13	9	13	65.2	50.14	0	235.2	1.274E+11	2.82E+09	3.63E+10	1.53E+09	8.68E+10	1.17E+09	2.7E+09	1.174E+09	4.53E+09
P07900;Q1	P07900	Heat shock protein HSP 90-alpha	HSP90AA1	sp|P07900	51	51	32	16	48	33	51	16	48	33	51	10	29	17	32	65.7	84.659	0	323.31	1.158E+11	1.95E+08	2.76E+10	908370000	8.71E+10	2.92E+08	2.32E+09	296540000	3.52E+09
P04083	P04083	Annexin A1	ANXA1	sp|P04083	27	27	27	1	10	19	27	1	10	19	27	1	10	19	27	68.2	38.714	0	312.27	1.146E+11	916260	1.16E+08	243060000	1.14E+11	880450	4885600	174320000	4.64E+09
P04350	P04350	Tubulin beta-4A chain	TUBB4A	sp|P04350	25	25	2	12	23	19	22	12	23	19	22	1	2	0	0	67.1	49.585	0	323.31	1.144E+11	5.22E+08	3.21E+10	2.824E+09	7.89E+10	7.34E+08	2.09E+09	1.535E+09	3.26E+09
P13639	P13639	Elongation factor 2	EEF2	sp|P13639	58	58	57	12	50	36	58	12	50	36	58	12	49	35	57	68.6	95.337	0	323.31	1.124E+11	1.71E+08	1.82E+10	435710000	9.35E+10	1.51E+08	1.93E+09	324770000	3.34E+09
P10809	P10809	60 kDa heat shock protein, mitochondrial	HSPD1	sp|P10809	45	45	45	11	44	30	44	11	44	30	44	11	44	30	44	77.7	61.054	0	323.31	9.791E+10	4.14E+08	4.47E+10	824510000	5.2E+10	3.6E+08	3.59E+09	622680000	1.92E+09
P00338	P00338	L-lactate dehydrogenase A chain	LDHA	sp|P00338	29	29	27	8	29	17	29	8	29	17	29	8	27	16	27	76.5	36.688	0	323.31	9.252E+10	93914000	1.89E+10	155540000	7.34E+10	93698000	1.6E+09	134030000	2.91E+09
P11142	P11142	Heat shock cognate 71 kDa protein	HSPA8	sp|P11142	42	41	28	15	39	36	41	14	38	35	40	12	25	22	27	71.8	70.897	0	323.31	8.872E+10	4.16E+08	2.13E+10	4.092E+09	6.29E+10	5.88E+08	1.67E+09	2.454E+09	2.45E+09
P60174	P60174	Triosephosphate isomerase	TPI1	sp|P60174	17	17	17	1	16	10	16	1	16	10	16	1	16	10	16	82.7	26.669	0	276.03	8.067E+10	67867000	3.31E+10	105980000	4.74E+10	74772000	2.67E+09	79793000	1.79E+09
P11021	P11021	78 kDa glucose-regulated protein	HSPA5	sp|P11021	48	48	47	31	45	43	44	31	45	43	44	30	44	42	43	63.3	72.332	0	323.31	7.681E+10	7.76E+09	3.08E+10	5.585E+09	3.26E+10	4.4E+09	3.79E+09	5.31E+09	1.9E+09
Q00610;P5	Q00610	Clathrin heavy chain 1	CLTC	sp|Q0061	83	83	83	23	74	57	82	23	74	57	82	23	74	57	82	59.6	191.61	0	323.31	7.501E+10	6.3E+08	9.65E+09	662790000	6.41E+10	7.81E+08	8.84E+08	475210000	2.29E+09
P07355;A6	P07355;A6	Annexin A2;Putative annexin A2-like protein	ANXA2;AN	sp|P07355	31	31	31	2	21	27	31	2	21	27	31	2	21	27	31	75.8	38.604	0	256.77	6.979E+10	1818200	8.64E+08	792300000	6.81E+10	4147100	32001000	170760000	3.18E+09
P23528	P23528	Cofilin-1	CFL1	sp|P23528	19	19	14	4	17	16	18	4	17	16	18	1	12	11	14	81.3	18.502	0	187.87	6.881E+10	68589000	1.8E+10	829150000	4.99E+10	99548000	1.81E+09	459340000	1.86E+09
Q14204	Q14204	Cytoplasmic dynein 1 heavy chain 1	DYNC1H1	sp|Q1420	216	216	216	21	143	153	213	21	143	153	213	21	143	153	213	53.7	532.4	0	323.31	6.145E+10	89619000	3.69E+09	1.303E+09	5.64E+10	1.26E+08	3.56E+08	961640000	2.14E+09
P07195;Q6	P07195	L-lactate dehydrogenase B chain	LDHB	sp|P07195	21	19	19	7	21	11	21	7	19	10	19	7	19	10	19	51.5	36.638	0	207.07	6.087E+10	1.29E+08	2.31E+10	29927000	3.76E+10	1.97E+08	2.03E+09	41638000	1.36E+09
P08238;Q5	P08238	Heat shock protein HSP 90-beta	HSP90AB1	sp|P08238	50	31	28	12	49	36	49	6	30	20	30	5	27	17	27	72.5	83.263	0	323.31	6.076E+10	70848000	1.86E+10	478360000	4.16E+10	80539000	1.54E+09	191080000	1.64E+09
P04075	P04075	Fructose-bisphosphate aldolase A	ALDOA	sp|P04075	31	31	27	11	31	20	29	11	31	20	29	11	27	20	25	85.4	39.42	0	323.31	5.843E+10	1.32E+08	2.47E+10	309790000	3.33E+10	1.29E+08	2.17E+09	90913000	1.35E+09
P08758	P08758	Annexin A5	ANXA5	sp|P08758	26	26	26	0	25	15	25	0	25	15	25	0	25	15	25	75	35.936	0	323.31	5.775E+10	0	7.82E+09	94396000	4.98E+10	0	8.06E+08	93398000	1.88E+09
Q09666	Q09666	Neuroblast differentiation-associated protein AHI	AHNAK	sp|Q0966	240	240	240	4	20	172	236	4	20	172	236	4	20	172	236	66	629.09	0	323.31	5.59E+10	41922000	89261000	1.73E+09	5.4E+10	19620000	5700400	1.123E+09	2.25E+09
P06576	P06576	ATP synthase subunit beta, mitochondrial	ATP5B	sp|P06576	29	29	29	11	29	16	28	11	29	16	28	11	29	16	28	80.2	56.559	0	323.31	5.514E+10	2.45E+08	2.38E+10	328530000	3.07E+10	4.28E+08	1.89E+09	288700000	1.13E+09
P62937;P0	P62937	Peptidyl-prolyl cis-trans isomerase A;Peptidyl-pro	PPIA	sp|P62937	12	12	12	6	12	11	12	6	12	11	12	6	12	11	12	71.5	18.012	0	147.93	5.398E+10	1.4E+08	2.16E+10	109950000	3.21E+10	33803000	1.96E+09	75997000	1.28E+09
Q02224	Q02224	Centromere-associated protein E	CENPE	sp|Q0222	3	3	3	1	0	2	2	1	0	2	2	1	0	2	2	1.8	316.41	0.000245	3.5783	5.317E+10	5.31E+10	0	81283000	13249000	5.1E+10	0	254430000	8.75E+08
P04040	P04040	Catalase	CAT	sp|P04040	10	10	10	1	10	9	9	1	10	9	9	1	10	9	9	23.7	59.755	0	41.566	5.266E+10	5.2E+10	2.84E+08	214460000	1.98E+08	4.96E+10	1.59E+08	1.963E+09	4.38E+08
P06748	P06748	Nucleophosmin	NPM1	sp|P06748	17	17	17	4	16	16	17	4	16	16	17	4	16	16	17	55.8	32.575	0	323.31	5.208E+10	4.76E+09	2.13E+10	2.982E+09	2.3E+10	5.73E+09	1.24E+09	1.75E+09	6.8E+08
P21333	P21333	Filamin-A	FLNA	sp|P21333	96	87	84	5	68	78	94	3	64	70	85	3	63	69	82	49.8	280.74	0	323.31	4.907E+10	5957900	1.64E+09	1.122E+09	4.63E+10	7242200	1.61E+08	759240000	1.86E+09
P12277;P0	P12277	Creatine kinase B-type	CKB	sp|P12277	24	24	24	3	24	2	1	3	24	2	1	3	24	2	1	82.4	42.644	0	323.31	4.855E+10	3.57E+08	4.82E+10	1672700	24675000	11466000	4.47E+09	643820	611720
Q14315	Q14315	Filamin-C	FLNC	sp|Q1431	99	99	88	4	26	76	96	4	26	76	96	1	21	67	85	47.2	291.02	0	323.31	4.838E+10	25249000	4.64E+08	921720000	4.7E+10	28106000	47154000	696550000	1.8E+09
P07437;A6	P07437	Tubulin beta chain	TUBB	sp|P07437	23	5	4	16	23	22	23	5	5	5	5	4	4	4	4	61.9	49.67	0	145.96	4.728E+10	7.13E+08	1.37E+10	1.023E+09	3.18E+10	6.87E+08	1.12E+09	732450000	1.24E+09
Q9Y490;Q9	Q9Y490	Talin-1	TLN1	sp|Q9Y49	104	104	104	4	80	46	104	4	80	46	104	4	80	46	104	54.1	269.76	0	323.31	4.636E+10	1.06E+08	3.74E+09	143420000	4.24E+10	1.77E+08	3.51E+08	111480000	1.61E+09
P07942	P07942	Laminin subunit beta-1	LAMB1	sp|P07942	5	5	5	1	3	1	4	1	3	1	4	1	3	1	4	4.5	198.04	0	23.532	4.635E+10	11883000	39372000	4.612E+10	1.8E+08	0	3153200	0	7712100
P60842	P60842	Eukaryotic initiation factor 4A-I	EIF4A1	sp|P60842	26	26	14	14	22	20	25	14	22	20	25	9	12	12	14	68	46.153	0	222.25	4.519E+10	7.66E+08	9E+09	293040000	3.51E+10	6.85E+08	7.47E+08	213750000	1.51E+09
P38646	P38646	Stress-70 protein, mitochondrial	HSPA9	sp|P38646	45	45	45	26	39	43	40	26	39	43	40	26	39	43	40	63.5	73.68	0	323.31	4.481E+10	3.5E+09	1.25E+10	6.605E+09	2.22E+10	3.71E+09	1.01E+09	3.946E+09	7.34E+08
P00558;P0	P00558	Phosphoglycerate kinase 1	PGK1	sp|P00558	32	32	32	6	32	14	30	6	32	14	30	6	32	14	30	78.2	44.614	0	311.28	4.354E+10	47512000	1.62E+10	88106000	2.72E+10	87198000	1.31E+09	32949000	1.1E+09
P14625;Q5	P14625	Endoplasmin	HSP90B1	sp|P14625	50	48	48	8	47	23	49	8	45	21	47	8	45	21	47	61.6	92.468	0	323.31	4.314E+10	22196000	1.21E+10	197550000	3.08E+10	39527000	1.1E+09	59556000	1.23E+09
P19338	P19338	Nucleolin	NCL	sp|P19338	38	38	38	14	36	34	36	14	36	34	36	14	36	34	36	43.8	76.613	0	323.31	4.079E+10	1.41E+09	1.77E+10	2.673E+09	1.9E+10	9.92E+08	1.61E+09	2.067E+09	7.77E+08
P43490	P43490	Nicotinamide phosphoribosyltransferase	NAMPT	sp|P43490	26	26	26	1	20	22	26	1	20	22	26	1	20	22	26	63.1	55.52	0	323.31	3.982E+10	394390	1.18E+09	635410000	3.8E+10	474380	1.33E+08	478040000	1.47E+09
P07737;CC	P07737	Profilin-1	PFN1	sp|P07737	10	10	10	0	9	6	10	0	9	6	10	0	9	6	10	83.6	15.054	0	84.299	3.953E+10	0	1.32E+10	58821000	2.63E+10	0	1.06E+09	39348000	1.08E+09
P68371	P68371	Tubulin beta-4B chain	TUBB4B	sp|P68371	25	6	1	14	25	23	25	4	6	6	6	1	1	1	1	66.1	49.83	0	206.58	3.904E+10	1.16E+09	9.95E+09	901570000	2.7E+10	7.15E+08	1.08E+09	828910000	9.37E+08
P78371	P78371	T-complex protein 1 subunit beta	CCT2	sp|P78371	34	34	34	11	33	20	33	11	33	20	33	11	33	20	33	71.6	57.488	0	323.31	3.661E+10	2.63E+08	1.77E+10	195070000	1.85E+10	3.36E+08	1.36E+09	142100000	8.11E+08
P61978	P61978	Heterogeneous nuclear ribonucleoprotein K	HNRNPK	sp|P61978	27	27	27	15	27	22	26	15	27	22	26	15	27	22	26	63.9	50.976	0	283.34	3.646E+10	5.91E+08	1.69E+10	491100000	1.85E+10	7.96E+08	1.23E+09	463060000	6E+08
P62258	P62258	14-3-3 protein epsilon	YWHAE	sp|P62258	23	23	20	10	23	15	23	10	23	15	23	9	20	12	20	75.7	29.174	0	136.11	3.56E+10	84199000	1.26E+10	206050000	2.27E+10	1.61E+08	9.17E+08	176960000	9.86E+08
O43707	O43707	Alpha-actinin-4	ACTN4	sp|O43707	53	53	38	2	45	48	50	2	45	48	50	2	33	34	35	67.3	104.85	0	323.31	3.557E+10	1073800	6.02E+09	1.759E+09	2.78E+10	596630	4.73E+08	1.563E+09	7.76E+08
P53396	P53396	ATP-citrate synthase	ACLY	sp|P53396	53	53	53	4	48	24	52	4	48	24	52	4	48	24	52	55.1	120.84	0	323.31	3.427E+10	2325200	5.63E+09	65290000	2.86E+10	2143300	5.08E+08	27168000	1.13E+09
P26641	P26641	Elongation factor 1-gamma	EEF1G	sp|P26641	30	30	30	8	27	20	30	8	27	20	30	8	27	20	30	73.7	50.118	0	285.68	3.381E+10	1.55E+08	8.26E+09	331410000	2.51E+10	2.17E+08	7.06E+08	238630000	9.34E+08
P27797	P27797	Calreticulin	CALR	sp|P27797	23	23	23	2	21	12	22	2	21	12	22	2	21	12	22	72.7	48.141	0	323.31	3.09E+10	21451000	7.39E+09	76036000	2.34E+10	18967000	6.13E+08	92189000	9.29E+08
Q9BUF5	Q9BUF5	Tubulin beta-6 chain	TUBB6	sp|Q9BUF	24	14	10	8	20	21	24	3	11	12	14	2	8	9	10	65.2	49.857	0	283.59	3.079E+10	2387800	3.71E+09	826220000	2.62E+10	2786500	1.12E+08	775670000	9.18E+08
P25705	P25705	ATP synthase subunit alpha, mitochondrial	ATP5A1	sp|P25705	30	30	30	9	30	24	30	9	30	24	30	9	30	24	30	57.3	59.75	0	322.7	3.074E+10	1.49E+08	1.19E+10	311340000	1.84E+10	1.77E+08	1.07E+09	223770000	6.57E+08
P49327	P49327	Fatty acid synthase;[Acyl-carrier-protein] S-acetyl	FASN	sp|P49327	93	93	93	22	87	35	85	22	87	35	85	22	87	35	85	49.5	273.42	0	323.31	3.037E+10	1.34E+08	1.24E+10	123060000	1.77E+10	1.77E+08	1.12E+09	101830000	5.91E+08
P01857;P0	P01857;P0	Ig gamma-1 chain C region;Ig gamma-3 chain C re	IGHG1;IGH	sp|P01857	7	7	6	3	1	7	1	3	1	7	1	2	1	6	1	26.7	36.105	0	24.085	3.028E+10	3.83E+08	23278000	2.984E+10	28670000	83628000	325950	1.957E+10	250530
P48643	P48643	T-complex protein 1 subunit epsilon	CCT5	sp|P48643	34	34	34	5	33	20	34	5	33	20	34	5	33	20	34	67.3	59.67	0	323.31	2.94E+10	9632700	1.07E+10	137980000	1.85E+10	7327200	1.04E+09	89172000	5.88E+08
P35908	P35908	Keratin, type II cytoskeletal 2 epidermal	KRT2	sp|P35908	53	49	1	17	43	53	36	15	39	49	33	1	1	1	1	83.6	65.432	0	323.31	2.912E+10	3.52E+08	9.38E+09	1.557E+10	3.81E+09	3.46E+08	7.87E+08	1.039E+10	1.46E+08
P78527	P78527	DNA-dependent protein kinase catalytic subunit	PRKDC	sp|P78527	133	133	133	18	121	102	125	18	121	102	125	18	121	102	125	39.4	469.08	0	323.31	2.851E+10	51050000	6.33E+09	740810000	2.14E+10	54978000	5.79E+08	548990000	7.91E+08
Q06830	Q06830	Peroxiredoxin-1	PRDX1	sp|Q0683	17	17	13	8	17	14	15	8	17	14	15	7	13	10	12	66.8	22.11	0	76.788	2.814E+10	69108000	1.7E+10	210550000	1.09E+10	84570000	1.33E+09	138610000	4.37E+08
P07237	P07237	Protein disulfide-isomerase	P4HB	sp|P07237	32	32	32	7	30	21	32	7	30	21	32	7	30	21	32	60.8	57.116	0	323.31	2.8E+10	8746800	6.1E+09	125910000	2.18E+10	5408000	4.82E+08	102700000	9.01E+08
P30101	P30101	Protein disulfide-isomerase A3	PDIA3	sp|P30101	36	36	36	7	35	16	34	7	35	16	34	7	35	16	34	68.7	56.782	0	323.31	2.708E+10	11718000	1.05E+10	69352000	1.65E+10	8300900	9.35E+08	28125000	6.32E+08
P63104	P63104	14-3-3 protein zeta/delta	YWHAZ	sp|P63104	20	17	14	6	19	16	20	5	16	13	17	5	13	12	14	75.9	27.745	0	321.49	2.642E+10	48135000	7.08E+09	152030000	1.91E+10	55802000	6.7E+08	108460000	7.17E+08
P55072	P55072	Transitional endoplasmic reticulum ATPase	VCP	sp|P55072	43	43	43	5	37	29	43	5	37	29	43	5	37	29	43	64.3	89.321	0	323.31	2.639E+10	1.71E+08	7.85E+09	346950000	1.8E+10	1.91E+08	6.57E+08	271620000	6.98E+08
P49368	P49368	T-complex protein 1 subunit gamma	CCT3	sp|P49368	33	33	33	3	31	14	30	3	31	14	30	3	31	14	30	63.5	60.533	0	244.93	2.618E+10	1.05E+08	8.88E+09	128950000	1.71E+10	87214000	7.06E+08	184660000	6.85E+08
P18669;P1	P18669	Phosphoglycerate mutase 1	PGAM1	sp|P18669	19	19	19	2	18	9	19	2	18	9	19	2	18	9	19	78.3	28.804	0	217.55	2.602E+10	4051600	1.04E+10	73588000	1.56E+10	3017800	9.2E+08	10904000	6.63E+08
P62081	P62081	40S ribosomal protein S7	RPS7	sp|P62081	14	14	14	9	12	12	14	9	12	12	14	9	12	12	14	62.9	22.127	0	167.56	2.549E+10	1.39E+09	4.85E+09	1.527E+09	1.77E+10	1.03E+09	4.99E+08	900160000	6.95E+08
Q14974	Q14974	Importin subunit beta-1	KPNB1	sp|Q1497	29	29	29	6	28	19	27	6	28	19	27	6	28	19	27	41.1	97.169	0	315.21	2.508E+10	1.17E+08	7.54E+09	117600000	1.73E+10	1.51E+08	6.71E+08	92031000	6.29E+08
P12956	P12956	X-ray repair cross-complementing protein 6	XRCC6	sp|P12956	39	39	39	12	36	27	34	12	36	27	34	12	36	27	34	59.1	69.842	0	276.4	2.501E+10	4.05E+08	7.71E+09	357450000	1.65E+10	4.12E+08	6.78E+08	258730000	6.36E+08
P22314	P22314	Ubiquitin-like modifier-activating enzyme 1	UBA1	sp|P22314	41	41	41	3	39	13	38	3	39	13	38	3	39	13	38	53.1	117.85	0	323.31	2.496E+10	43544000	1.22E+10	52795000	1.26E+10	23310000	8.01E+08	21025000	7.99E+08
P40926	P40926	Malate dehydrogenase, mitochondrial	MDH2	sp|P40926	17	17	17	4	17	5	17	4	17	5	17	4	17	5	17	61.8	35.503	0	131.44	2.487E+10	1.68E+08	1.63E+10	19288000	8.33E+09	1.47E+08	1.36E+09	18013000	3.25E+08
Q15149	Q15149	Plectin	PLEC	sp|Q1514	226	226	218	11	20	211	199	11	20	211	199	10	18	203	192	54.7	531.78	0	323.31	2.414E+10	91647000	1.24E+08	4.635E+09	1.93E+10	14398000	5448800	2.614E+09	1.35E+09
P22392;O6	P22392;O6	Nucleoside diphosphate kinase B;Putative nucleo	NME2;NM	sp|P22392	13	13	7	3	12	8	13	3	12	8	13	1	7	6	7	79.6	17.298	0	89.874	2.413E+10	71446000	9.8E+09	58300000	1.42E+10	1.33E+08	7.36E+08	50130000	5.88E+08
P05388;Q8	P05388;Q8	60S acidic ribosomal protein P0;60S acidic ribosor	RPLP0;RPL	sp|P05388	15	15	15	10	15	14	13	10	15	14	13	10	15	14	13	50.8	34.273	0	94.276	2.39E+10	8.68E+08	6.69E+09	1.932E+09	1.44E+10	1.02E+09	5.06E+08	1.299E+09	5.02E+08
Q14764	Q14764	Major vault protein	MVP	sp|Q1476	48	48	48	0	2	39	47	0	2	39	47	0	2	39	47	63.6	99.326	0	323.31	2.223E+10	0	10780000	482070000	2.17E+10	0	1196500	318170000	8.89E+08
P46940	P46940	Ras GTPase-activating-like protein IQGAP1	IQGAP1	sp|P46940	72	72	70	3	47	37	70	3	47	37	70	3	45	36	69	58.5	189.25	0	323.31	2.149E+10	976680	1.06E+09	176970000	2.02E+10	1194900	1.04E+08	180700000	7.58E+08

P50991	P50991	T-complex protein 1 subunit delta	CCT4	sp|P50991	25	25	25	3	23	14	23	3	23	14	23	3	23	14	23	57	57.924	0	280.52	2.055E+10	1.15E+08	6.26E+09	89038000	1.41E+10	1.43E+08	5.41E+08	83728000	5.21E+08
P11586	P11586	C-1-tetrahydrofolate synthase, cytoplasmic;Meth	MTHFD1	sp|P11586	48	48	48	8	43	24	44	8	43	24	44	8	43	24	44	65.2	101.56	0	323.31	2.048E+10	24430000	5.74E+09	147570000	1.46E+10	25675000	4.66E+08	121090000	6.01E+08
P46821	P46821	Microtubule-associated protein 1B;MAP1B heavy	MAP1B	sp|P46821	87	87	86	2	52	79	85	2	52	79	85	2	51	78	84	52.4	270.63	0	323.31	2.04E+10	2886000	7.74E+08	875420000	1.87E+10	2343800	73154000	604060000	7.22E+08
P42704;Q9	P42704	Leucine-rich PPR motif-containing protein, mitoch	LRPPRC	sp|P42704	62	62	62	7	56	35	60	7	56	35	60	7	56	35	60	48.1	157.9	0	323.31	2.009E+10	15386000	5.53E+09	141040000	1.44E+10	17255000	4.7E+08	130050000	5.09E+08
P50395	P50395	Rab GDP dissociation inhibitor beta	GDI2	sp|P50395	28	28	20	2	27	10	28	2	27	10	28	1	19	7	20	67.9	50.663	0	274.86	1.994E+10	2760500	5.64E+09	27058000	1.43E+10	2075100	5.17E+08	8388000	5.12E+08
P06744;CC	P06744	Glucose-6-phosphate isomerase	GPI	sp|P06744	27	27	27	2	26	5	25	2	26	5	25	2	26	5	25	60.6	63.146	0	304.47	1.993E+10	4557600	8.84E+09	24328000	1.11E+10	1393400	7.92E+08	4642600	3.66E+08
P17987	P17987	T-complex protein 1 subunit alpha	TCP1	sp|P17987	27	27	27	13	26	18	26	13	26	18	26	13	26	18	26	62.6	60.343	0	275.15	1.97E+10	1.77E+08	6.71E+09	98898000	1.27E+10	1.67E+08	5.35E+08	85893000	5.45E+08
P39023	P39023	60S ribosomal protein L3	RPL3	sp|P39023	17	17	17	9	15	17	15	9	15	17	15	9	15	17	15	47.6	46.108	0	173.44	1.946E+10	9.92E+08	4.6E+09	1.462E+09	1.24E+10	7.07E+08	4.69E+08	1.192E+09	4.74E+08
P13010	P13010	X-ray repair cross-complementing protein 5	XRCC5	sp|P13010	37	37	37	10	30	20	32	10	30	20	32	10	30	20	32	66.8	82.704	0	323.31	1.928E+10	1.32E+08	5.38E+09	237740000	1.35E+10	3.62E+08	3.78E+08	117530000	4.54E+08
P29401	P29401	Transketolase	TKT	sp|P29401	31	31	31	3	26	14	30	3	26	14	30	3	26	14	30	53.8	67.877	0	226.89	1.925E+10	1465900	4.32E+09	41130000	1.49E+10	1008400	4.11E+08	13526000	5.85E+08
P19105;O1	P19105;O1	Myosin regulatory light chain 12A;Myosin regulat	MYL12A;M	sp|P19105	9	9	4	7	7	8	9	7	7	8	9	4	2	3	4	51.5	19.794	0	274.87	1.918E+10	3.03E+08	2.27E+09	651920000	1.6E+10	4.4E+08	2.23E+08	384630000	5.08E+08
P05141;Q9	P05141	ADP/ATP translocase 2;ADP/ATP translocase 2, N	SLC25A5	sp|P05141	15	15	8	8	15	14	15	8	15	14	15	4	8	7	8	40.6	32.852	0	57.206	1.874E+10	1.36E+09	8.71E+09	511330000	8.16E+09	4.88E+08	1.15E+09	534530000	5.95E+08
P09211	P09211	Glutathione S-transferase P	GSTP1	sp|P09211	11	11	11	7	9	10	10	7	9	10	10	7	9	10	10	66.2	23.356	0	323.31	1.821E+10	2.04E+09	7.17E+09	218260000	8.77E+09	2.38E+09	4.06E+08	125040000	1.84E+08
P40227;Q9	P40227	T-complex protein 1 subunit zeta	CCT6A	sp|P40227	24	24	24	5	23	19	24	5	23	19	24	5	23	19	24	49.9	58.024	0	140.51	1.809E+10	33482000	5.68E+09	128940000	1.23E+10	45638000	5.2E+08	92317000	4.36E+08
Q99832	Q99832	T-complex protein 1 subunit eta	CCT7	sp|Q99832	31	31	31	3	29	15	30	3	29	15	30	3	29	15	30	69.2	59.366	0	293.76	1.805E+10	14090000	5.17E+09	77556000	1.28E+10	11134000	5.53E+08	43477000	4.22E+08
Q8IVF2	Q8IVF2	Protein AHNAK2	AHNAK2	sp|Q8IVF2	125	125	125	2	3	73	123	2	3	73	123	2	3	73	123	47.2	616.62	0	323.31	1.783E+10	41570000	29287000	530660000	1.72E+10	22606000	4804800	126880000	9.55E+08
Q14697	Q14697	Neutral alpha-glucosidase AB	GANAB	sp|Q14697	41	41	41	0	37	15	38	0	37	15	38	0	37	15	38	53.7	106.87	0	323.31	1.779E+10	0	4.64E+09	44225000	1.31E+10	0	4.06E+08	36792000	5.17E+08
Q02878	Q02878	60S ribosomal protein L6	RPL6	sp|Q02878	20	20	20	11	20	19	19	11	20	19	19	11	20	19	19	54.5	32.728	0	180.67	1.762E+10	4.96E+08	4.5E+09	1.243E+09	1.14E+10	6.57E+08	3.33E+08	778860000	3.24E+08
P26038	P26038	Moesin	MSN	sp|P26038	44	44	33	3	38	32	44	3	38	32	44	1	28	25	33	70.2	67.819	0	312.89	1.745E+10	11384000	2.14E+09	319950000	1.5E+10	15682000	2.07E+08	118950000	6.54E+08
P55060	P55060	Exportin-2	CSE1L	sp|P55060	46	46	46	5	40	22	43	5	40	22	43	5	40	22	43	54	110.42	0	323.31	1.732E+10	5039600	5.06E+09	130550000	1.21E+10	4790100	4.36E+08	106050000	4.58E+08
P09651;A0	P09651;A0	Heterogeneous nuclear ribonucleoprotein A1;Het	HNRNPA1;	sp|P09651	18	17	17	7	16	18	15	7	15	17	14	7	15	17	14	42.5	38.746	0	230.92	1.73E+10	6.22E+08	3.98E+09	1.51E+09	1.12E+10	3.49E+08	5.13E+08	966710000	6.04E+08
P30041	P30041	Peroxiredoxin-6	PRDX6	sp|P30041	18	18	18	0	17	4	17	0	17	4	17	0	17	4	17	72.3	25.035	0	243.27	1.716E+10	0	9.33E+09	9119000	7.82E+09	0	7.76E+08	5877100	3.44E+08
P36578	P36578	60S ribosomal protein L4	RPL4	sp|P36578	28	28	28	9	26	26	25	9	26	26	25	9	26	26	25	56.7	47.697	0	123.06	1.709E+10	4.16E+08	5.56E+09	1.664E+09	9.45E+09	3.92E+08	4.65E+08	1.053E+09	4.3E+08
P50990	P50990	T-complex protein 1 subunit theta	CCT8	sp|P50990	36	36	36	9	31	18	35	9	31	18	35	9	31	18	35	69.3	59.62	0	165	1.683E+10	1.28E+08	6.25E+09	98342000	1.04E+10	1.78E+08	5.26E+08	55257000	3.92E+08
Q9BZH6	Q9BZH6	WD repeat-containing protein 11	WDR11	sp|Q9BZH6	11	11	11	3	7	8	10	3	7	8	10	3	7	8	10	11.8	136.68	0	28.299	1.681E+10	1.5E+10	98851000	1.333E+09	4.13E+08	9.56E+09	62557000	4.103E+09	2.16E+09
P18124	P18124	60S ribosomal protein L7	RPL7	sp|P18124	19	19	19	12	18	16	18	12	18	16	18	12	18	16	18	55.6	29.225	0	75.765	1.65E+10	6.05E+08	4.51E+09	1.855E+09	9.53E+09	7.32E+08	3.1E+08	1.166E+09	3.07E+08
O43852	O43852	Calumenin	CALU	sp|O43852	24	24	24	0	24	19	23	0	24	19	23	0	24	19	23	79	37.106	0	323.31	1.64E+10	0	1.42E+09	671160000	1.43E+10	0	1.43E+08	509680000	5.12E+08
P55209	P55209	Nucleosome assembly protein 1-like 1	NAP1L1	sp|P55209	14	14	13	4	14	12	14	4	14	12	14	3	13	11	13	57	45.374	0	295.24	1.631E+10	3.06E+08	5.04E+09	281150000	1.07E+10	3.66E+08	4.08E+08	220060000	3.69E+08
P34932	P34932	Heat shock 70 kDa protein 4	HSPA4	sp|P34932	38	38	35	4	33	18	38	4	33	18	38	3	30	18	35	51.9	94.33	0	217.05	1.613E+10	7620400	5.32E+09	85837000	1.07E+10	4827900	3.93E+08	37922000	5.33E+08
O00410	O00410	Importin-5	IPO5	sp|O00410	36	36	34	3	28	11	35	3	28	11	35	3	26	9	33	44.3	123.63	0	282.87	1.608E+10	42578000	3.35E+09	37593000	1.26E+10	34131000	3.24E+08	40656000	4.83E+08
P63241;Q6	P63241;Q6	Eukaryotic translation initiation factor 5A-1;Eukar	EIF5A;EIF5	sp|P63241	15	15	15	2	12	8	14	2	12	8	14	2	12	8	14	72.7	16.832	0	256.02	1.593E+10	1.14E+08	6.89E+09	105980000	8.82E+09	1.27E+08	5.63E+08	71944000	3.71E+08
P13667	P13667	Protein disulfide-isomerase A4	PDIA4	sp|P13667	42	42	42	4	39	7	42	4	39	7	42	4	39	7	42	57.1	72.932	0	260.75	1.553E+10	11228000	6.92E+09	269530000	8.34E+09	9779400	5.51E+08	32321000	5.24E+08
P26599	P26599	Polypyrimidine tract-binding protein 1	PTBP1	sp|P26599	21	21	19	7	21	18	19	7	21	18	19	6	19	16	18	62.5	57.221	0	323.31	1.549E+10	1.09E+09	7.77E+09	353480000	6.28E+09	5.97E+08	8.4E+08	418330000	3.25E+08
P84077;P6	P84077;P6	ADP-ribosylation factor 1;ADP-ribosylation factor	ARF1;ARF3	sp|P84077	9	4	4	1	9	6	9	1	4	2	4	1	4	2	4	54.7	20.697	0	39.034	1.547E+10	1.02E+10	1.79E+09	11992000	3.49E+09	7.75E+09	5.5E+08	220550000	1.95E+09
Q14152	Q14152	Eukaryotic translation initiation factor 3 subunit A	EIF3A	sp|Q14152	68	68	68	34	62	54	61	34	62	54	61	34	62	54	61	50.6	166.57	0	323.31	1.543E+10	2.27E+09	3.75E+09	344320000	9.06E+09	1.85E+09	4.47E+08	346320000	5.57E+08
P31939	P31939	Bifunctional purine biosynthesis protein PURH;Ph	ATIC	sp|P31939	34	34	34	3	33	5	31	3	33	5	31	3	33	5	31	69.6	64.615	0	277.23	1.541E+10	21101000	3.61E+09	6841400	1.18E+10	10591000	3.34E+08	3489100	4.72E+08
P68032;P6	P68032;P6	Actin, alpha cardiac muscle 1;Actin, gamma-enter	ACTC1;ACT	sp|P68032	15	2	2	9	13	14	14	1	1	1	2	1	1	1	2	42.7	42.019	0	19.198	1.501E+10	10284000	9.52E+09	961070000	4.52E+09	55443000	8.3E+08	466700000	2.47E+08
Q04637;RE	Q04637	Eukaryotic translation initiation factor 4 gamma 1	EIF4G1	sp|Q04637	48	48	46	12	39	35	46	12	39	35	46	11	37	33	44	36.4	175.49	0	270.54	1.483E+10	2.08E+08	2.66E+09	305240000	1.17E+10	2.99E+08	2.46E+08	190690000	3.72E+08
P23396	P23396	40S ribosomal protein S3	RPS3	sp|P23396	20	20	20	10	20	16	18	10	20	16	18	10	20	16	18	75.3	26.688	0	161.57	1.483E+10	3.66E+08	4.51E+09	608040000	9.35E+09	6.11E+08	2.99E+08	363450000	2.76E+08
O75390	O75390	Citrate synthase, mitochondrial	CS	sp|O75390	20	20	20	0	20	5	20	0	20	5	20	0	20	5	20	57.9	51.712	0	284.99	1.426E+10	0	6.08E+09	17198000	8.16E+09	0	5.01E+08	15700000	3.41E+08
P24534	P24534	Elongation factor 1-beta	EEF1B2	sp|P24534	9	9	9	3	9	7	9	3	9	7	9	3	9	7	9	59.6	24.763	0	90.231	1.41E+10	2.35E+08	5.94E+09	209300000	7.71E+09	1.28E+08	5.68E+08	137770000	3.49E+08
Q15365;P5	Q15365	Poly(rC)-binding protein 1	PCBP1	sp|Q15365	17	17	12	2	16	10	16	2	16	10	16	1	11	6	11	74.2	37.497	0	323.31	1.4E+10	7960600	3.44E+09	72259000	1.05E+10	5314600	2.68E+08	35850000	3.83E+08
Q15084	Q15084	Protein disulfide-isomerase A6	PDIA6	sp|Q15084	19	19	19	4	19	9	19	4	19	9	19	4	19	9	19	48.9	48.121	0	228.19	1.397E+10	5.25E+08	6.7E+09	23472000	6.72E+09	3.69E+08	7.62E+08	21840000	2.31E+08
P31948	P31948	Stress-induced-phosphoprotein 1	STIP1	sp|P31948	44	44	44	4	42	11	42	4	42	11	42	4	42	11	42	65	62.639	0	214.95	1.365E+10	32292000	5.57E+09	18172000	8.03E+09	18779000	5.05E+08	10227000	3.32E+08
Q13200	Q13200	26S proteasome non-ATPase regulatory subunit 2	PSMD2	sp|Q13200	45	45	45	8	33	27	42	8	33	27	42	8	33	27	42	64.5	100.2	0	307.99	1.359E+10	63195000	1.78E+09	175360000	1.16E+10	99133000	1.61E+08	88988000	4.41E+08
P08670;P1	P08670	Vimentin	VIM	sp|P08670	43	43	41	16	30	42	37	16	30	42	37	16	29	40	37	76.4	53.651	0	290.91	1.338E+10	3.92E+08	1.2E+09	4.304E+09	7.49E+09	2.67E+08	1.14E+08	2.91E+09	3.53E+08
Q7KZF4	Q7KZF4	Staphylococcal nuclease domain-containing prote	SND1	sp|Q7KZF4	36	36	36	7	31	17	35	7	31	17	35	7	31	17	35	48	102	0	323.31	1.317E+10	53327000	2.66E+09	65622000	1.04E+10	1.08E+08	2.24E+08	39739000	3.58E+08
P18206	P18206	Vinculin	VCL	sp|P18206	45	45	45	4	37	15	44	4	37	15	44	4	37	15	44	48.8	123.8	0	323.31	1.316E+10	2224100	1.91E+09	26986000	1.12E+10	1630100	1.43E+08	13753000	4.9E+08
P23526	P23526	Adenosylhomocysteinase	AHCY	sp|P23526	18	18	18	3	18	7	18	3	18	7	18	3	18	7	18	40.7	47.716	0	83.627	1.277E+10	6555600	3.7E+09	20458000	9.03E+09	5353200	3.1E+08	8943700	3.87E+08
P22626	P22626	Heterogeneous nuclear ribonucleoproteins A2/B1	HNRNPA2B	sp|P22626	23	23	22	9	22	18	22	9	22	18	22	9	21	17	21	70	37.429	0	233.11	1.253E+10	6.73E+08	5.2E+09	698570000	5.95E+09	9.12E+08	3.18E+08	350780000	1.92E+08
P62277	P62277	40S ribosomal protein S13	RPS13	sp|P62277	13	13	13	9	9	13	13	9	9	13	13	9	9	13	13	62.9	17.222	0	39.696	1.246E+10	1.36E+09	1.81E+09	530370000	8.77E+09	3.68E+08	4.04E+08	976200000	4.83E+08
P31943	P31943	Heterogeneous nuclear ribonucleoprotein H;Hete	HNRNPH1	sp|P31943	20	20	12	6	19	17	19	6	19	17	19	2	11	9	11	59.5	49.229	0	173.04	1.243E+10	3.19E+08	5.43E+09	837550000	5.85E+09	4.17E+08	4.48E+08	458770000	2.16E+08
P29692	P29692	Elongation factor 1-delta	EEF1D	sp|P29692	11	11	11	9	10	9	11	9	10	9	11	9	10	9	11	53	31.121	0	101.66	1.226E+10	4.97E+08	2.56E+09	200270000	9E+09	2.28E+08	2.62E+08	163000000	5.34E+08
P19367;Q2	P19367	Hexokinase-1	HK1	sp|P19367	41	41	37	1	31	20	41	1	31	20	41	1	27	16	37	46.3	102.48	0	323.31	1.217E+10	1802400	1.26E+09	61218000	1.08E+10	2393700	1.17E+08	22046000	4.57E+08
P27824	P27824	Calnexin	CANX	sp|P27824	31	31	31	3	29	11	29	3	29	11	29	3	29	11	29	47.1	67.567	0	151.47	1.215E+10	66753000	5E+09	50609000	7.03E+09	96833000	4.2E+08	29505000	2.82E+08
Q99623	Q99623	Prohibitin-2	PHB2	sp|Q99623	17	17	17	9	16	13	16	9	16	13	16	9	16	13	16	67.2	33.296	0	76.218	1.203E+10	3.43E+08	3.86E+09	209620000	7.62E+09	1.44E+08	4.38E+08	170280000	3.82E+08
Q08211	Q08211	ATP-dependent RNA helicase A	DHX9	sp|Q08211	47	47	47	19	39	42	43	19	39	42	43	19	39	42	43	45.4	140.96	0	308.5	1.191E+10	6.5E+08	3.32E+09	724030000	7.21E+09	4.93E+08	2.69E+08	678280000	2.75E+08
P52565	P52565	Rho GDP-dissociation inhibitor 1	ARHGDIA	sp|P52565	10	10	10	0	9	6	10	0	9	6	10	0	9	6	10	75	23.207	0	133.69	1.175E+10	0	3.07E+09	25644000	8.66E+09	0	2.97E+08	19693000	3.25E+08
P17655	P17655	Calpain-2 catalytic subunit	CAPN2	sp|P17655	27	27	27	2	13	11	27	2	13	11	27	2	13	11	27	49.7	79.994	0	323.31	1.174E+10	54482000	3.03E+08	104700000	1.13E+10	34325000	28088000	79589000	4.63E+08
P04844	P04844	Dolichyl-diphosphooligosaccharide--protein glyco	RPN2	sp|P04844	20	20	20	3	17	15	20	3	17	15	20	3	17	15	20	47.1	69.283	0	323.31	1.173E+10	2.19E+08	1.21E+09	155030000	1.01E+10	75774000	1.78E+08	297860000	2.94E+08
P09874	P09874	Poly [ADP-ribose] polymerase 1	PARP1	sp|P09874	54	54	54	24	54	26	37	24	54	26	37	24	54	26	37	58.8	113.08	0	323.31	1.171E+10	1.49E+09	8.08E+09	67839000	2.07E+09	1.38E+09	7.65E+08	78565000	1.01E+08
P63244	P63244	Guanine nucleotide-binding protein subunit beta-	GNB2L1	sp|P63244	18	18	18	8	16	11	18	8	16	11	18	8	16	11	18	67.2	35.076	0	162.17	1.17E+10	5.07E+08	4.28E+09	136130000	6.77E+09	6.52E+08	2.44E+08	77132000	1.84E+08
Q01105;P0	Q01105	Protein SET	SET	sp|Q01105	10	10	10	1	9	4	9	1	9	4	9	1	9	4	9	42.4	33.488	0	151.12	1.15E+10	3517100	4.07E+09	22965000	7.4E+09	6958400	3.84E+08	13638000	2.38E+08
Q99613;B5	Q99613;B5	Eukaryotic translation initiation factor 3 subunit C	EIF3C;EIF3	sp|Q99613	32	32	32	17	28	20	31	17	28	20	31	17	28	20	31	41.6	105.34	0	278.81	1.141E+10	2.7E+09	2.64E+09	120570000	5.96E+09	2.23E+09	3.74E+08	257220000	3.96E+08
P46783;Q9	P46783	40S ribosomal protein S10	RPS10	sp|P46783	13	13	13	4	11	11	12	4	11	11	12	4	11	11	12	63.6	18.898	0	42.515	1.126E+10	1.94E+08	4.41E+09	291110000	6.36E+09	4.25E+08	2.4E+08	224060000	1.51E+08
P35232	P35232	Prohibitin	PHB	sp|P35232	18	18	18	8	15	11	15	8	15	11	15	8	15	11	15	79	29.804	0	130.36	1.123E+10	8.99E+08	3.42E+09	89791000	6.82E+09	7.19E+08	4.06E+08	85928000	3.25E+08
Q00839	Q00839	Heterogeneous nuclear ribonucleoprotein U	HNRNPU	sp|Q00839	25	25	25	11	25	23	25	11	25	23	25	11	25	23	25	31.9	90.583	0	193.91	1.122E+10	3.52E+08	3.85E+09	518340000	6.49E+09	3.61E+08	3.19E+08	330800000	2.21E+08
P49411	P49411	Elongation factor Tu, mitochondrial	TUFM	sp|P49411	26	26	26	7	23	14	24	7	23	14	24	7	23	14	24	69.5	49.541	0	136.26	1.116E+10	22990000	4.68E+09	87063000	6.36E+09	38547000	3.93E+08	67794000	2.49E+08
P13797	P13797	Plastin-3	PLS3	sp|P13797	28	28	22	1	26	7	27	1	26	7	27	0	20	5	21	49.2	70.81	0	323.31	1.108E+10	18631000	6.6E+09	11192000	4.45E+09	12224000	6.18E+08	7870000	1.48E+08
P61247	P61247	40S ribosomal protein S3a	RPS3A	sp|P61247	19	19	19	9	18	19	17	9	18	19	17	9	18	19	17	61	29.945	0	159.18	1.106E+10	1.82E+08	3.7E+09	696560000	6.49E+09	2.66E+08	2.88E+08	411990000	2.56E+08
O43175	O43175	D-3-phosphoglycerate dehydrogenase	PHGDH	sp|O43175	22	22	22	4	21	11	20	4	21	11	20	4	21	11	20	52.2	56.65	0	244.67	1.101E+10	21400000	6.6E+09	59065000	4.33E+09	32565000	5.61E+08	35896000	1.85E+08
P62826	P62826	GTP-binding nuclear protein Ran	RAN	sp|P62826	14	14	14	3	13	8	12	3	13	8	12	3	13	8	12	44.4	24.423	0	59.256	1.095E+10	68680000	4.1E+09	52853000	6.73E+09	63187000	3.52E+08	31505000	2.94E+08
P37802	P37802	Transgelin-2	TAGLN2	sp|P37802	15	15	14	1	14	6	15	1	14	6	15	1	13	5	14	79.9	22.391	0	123.54	1.087E+10	529120	3.05E+09	16036000	7.81E+09	604890	2.65E+08	5282600	3.14E+08
P15880	P15880	40S ribosomal protein S2	RPS2	sp|P15880	14	14	14	7	13	12	13	7	13	12	13	7	13	12	13	49.1	31.324	0	91.46	1.087E+10	62018000	2.76E+09	497860000	7.55E+09	2.8E+08	2.01E+08	255920000	1.9E+08
P05023;P1	P05023	Sodium/potassium-transporting ATPase subunit a	ATP1A1	sp|P05023	40	40	32	2	37	26	37	2	37	26	37	1	29	19	30	45.3	112.89	0	323.31	1.087E+10	1311300	2.18E+09	225220000	8.45E+09	741350	2.09E+08	127540000	3.49E+08
P07814	P07814	Bifunctional glutamate/proline--tRNA ligase;Gluta	EPRS	sp|P07814	63	63	63	18	61	43	57	18	61	43	57	18	61	43	57	52.8	170.59	0	323.31	1.083E+10	5.4E+08	4.35E+09	284840000	5.65E+09	4.8E+08	3.92E+08	232500000	2.32E+08
Q01518	Q01518	Adenylyl cyclase-associated protein 1	CAP1	sp|Q01518	26	26	25	1	22	14	26	1	22	14	26	1	21	13	25	62.3	51.901	0	192.54	1.082E+10	1639000	1.84E+09	63671000	8.91E+09	1802300	1.78E+08	14285000	3.74E+08
Q15181	Q15181	Inorganic pyrophosphatase	PPA1	sp|Q15181	21	21	21	1	21	4	16	1	21	4	16	1	21	4	16	73.7	32.66	0	154.37	1.062E+10	4864200	6.88E+09	6070800	3.73E+09	3863500	6.23E+08	2122100	96857000
P33993	P33993	DNA replication licensing factor MCM7	MCM7	sp|P33993	34	34	34	4	29	24	34	4	29	24	34	4	29	24	34	56.1	81.307	0	290.98	1.058E+10	1.67E+08	9.59E+08	144080000	9.31E+09	1.28E+08	1.11E+08	115060000	3.75E+08
P52272	P52272	Heterogeneous nuclear ribonucleoprotein M	HNRNPM	sp|P52272	39	39	39	18	33	38	39	18	33	38	39	18	33	38	39	66.7	77.515	0	263.67	1.056E+10	2.88E+08	3.25E+09	1.148E+09	5.87E+09	3.38E+08	2.89E+08	738790000	2.01E+08
P62249	P62249	40S ribosomal protein S16	RPS16	sp|P62249	13	13	13	6	13	11	13	6	13	11	13	6	13	11	13	64.4	16.445	0	38.454	1.055E+10	3.21E+08	3.49E+09	437510000	6.3E+09	3.67E+08	2.61E+08	290870000	2.4E+08
P62820;P5	P62820	Ras-related protein Rab-1A	RAB1A	sp|P62820	12	12	6	1	11	6	12	1	11	6	12	0	5	2	6	69.3	22.677	0	147.57	1.047E+10	355080	2.76E+09	40942000	7.68E+09	488960	2.7E+08	8055500	3E+08
Q07021	Q07021	Complement component 1 Q subcomponent-binc	C1QBP	sp|Q07021	10	10	10	4	10	9	8	4	10	9	8	4	10	9	8	62.8	31.362	0	176.97	1.026E+10	1.37E+09	6.25E+09	252650000	2.39E+09	1.65E+09	3.39E+08	103910000	59174000
P04843	P04843	Dolichyl-diphosphooligosaccharide--protein glyco	RPN1	sp|P04843	26	26	26	3	21	17	26	3	21	17	26	3	21	17	26	56.7	68.569	0	196.19	1.022E+10	2623300	2.12E+09	145800000	7.96E+09	4044100	2.09E+08	60440000	3.23E+08
P27708	P27708	CAD protein;Glutamine-dependent carbamoyl-ph	CAD	sp|P27708	59	59	57	12	54	28	54	12	54	28	54	11	52	27	53	40	242.98	0	323.31	1.021E+10	1.96E+08	2.98E+09	129960000	6.9E+09	1.02E+08	3.07E+08	123340000	2.82E+08
Q92616	Q92616	Translational activator GCN1	GCN1L1	sp|Q92616	78	78	78	6	69	42	75	6	69	42	75	6	69	42	75	38.5	292.75	0	323.31	1.004E+10	42120000	3.15E+09	140900000	6.71E+09	16361000	2.92E+08	189800000	1.84E+08
P31946	P31946	14-3-3 protein beta/alpha;14-3-3 protein beta/alp	YWHAB	sp|P31946	15	8	8	4	15	12	15	2	8	6	8	2	8	6	8	60.2	28.082	0	220.48	1.001E+10	2943900	3.21E+09	51526000	6.74E+09	5396500	2.83E+08	23642000	2.59E+08
Q13509	Q13509	Tubulin beta-3 chain	TUBB3	sp|Q13509	21	5	5	11	18	20	21	1	3	5	5	1	3	5	5	55.6	50.432	0	122.65	9.973E+09	1204000	1.13E+08	142460000	9.72E+09	7974200	6557900	58414000	4.2E+08
Q16555	Q16555	Dihydropyrimidinase-related protein 2	DPYSL2	sp|Q16555	27	27	23	1	21	13	27	1	21	13	27	1	17	10	23	68.9	62.293	0	237.06	9.799E+09	1098900	6.69E+08	59138000	9.07E+09	2668400	23525000	23079000	4.04E+08

P12268	P12268	Inosine-5-monophosphate dehydrogenase 2	IMPDH2	sp|P12268	25	25	22	6	23	17	24	6	23	17	24	6	20	15	21	53.9	55.804	0	222.3	9.798E+09	69376000	3E+09	87444000	6.64E+09	95417000	2.78E+08	40325000	2.52E+08
O75083	O75083	WD repeat-containing protein 1	WDR1	sp|O7508	29	29	29	0	20	12	29	0	20	12	29	0	20	12	29	67.8	66.193	0	190.63	9.679E+09	0	6.18E+08	46654000	9.01E+09	0	62904000	40511000	3.5E+08
P22234	P22234	Multifunctional protein ADE2;Phosphoribosylami	PAICS	sp|P22234	22	22	22	2	22	10	22	2	22	10	22	2	22	10	22	47.1	47.079	0	142.51	9.656E+09	2524800	5.42E+09	32301000	4.2E+09	4164100	4.66E+08	18627000	1.79E+08
Q9P2J5	Q9P2J5	Leucine--tRNA ligase, cytoplasmic	LARS	sp|Q9P2J5	40	40	40	4	38	26	37	4	38	26	37	4	38	26	37	42.4	134.46	0	260.57	9.655E+09	34386000	3.5E+09	144550000	5.97E+09	48996000	3.09E+08	116390000	2.12E+08
O95373	O95373	Importin-7	IPO7	sp|O9537	23	23	23	0	20	16	23	0	20	16	23	0	20	16	23	26.4	119.52	0	270.48	9.644E+09	0	1.08E+09	105100000	8.46E+09	0	1.12E+08	95894000	3E+08
P12814;P3	P12814	Alpha-actinin-1	ACTN1	sp|P12814	50	36	36	1	34	44	49	1	23	31	35	1	23	31	35	65.2	103.06	0	323.31	9.639E+09	103470	8.4E+08	726260000	8.07E+09	1134500	22076000	770640000	78263000
Q6NSI4	Q6NSI4	Uncharacterized protein CXorf57	CXorf57	sp|Q6NSI4	2	2	2	0	1	0	1	0	1	0	1	0	1	0	1	4.2	97.553	0.003915	1.9572	9.631E+09	0	2787900	0	9.63E+09	0	245750	0	0
P53621	P53621	Coatomer subunit alpha;Xenin;Proxenin	COPA	sp|P53621	35	35	35	3	31	25	32	3	31	25	32	3	31	25	32	37.1	138.34	0	252.1	9.566E+09	7336400	1.97E+09	185150000	7.4E+09	10108000	2.07E+08	81225000	3.11E+08
Q96QH2	Q96QH2	PML-RARA-regulated adapter molecule 1	PRAM1	sp|Q96QH	2	2	2	1	0	1	1	1	0	1	1	1	0	1	1	3.6	73.968	0.001175	3.0392	9.546E+09	9.53E+09	0	9500300	5869200	9.47E+09	0	6384100	0
Q86VP6	Q86VP6	Cullin-associated NEDD8-dissociated protein 1	CAND1	sp|Q86VP	29	29	25	3	26	13	29	3	26	13	29	3	23	10	25	29.3	136.37	0	142.19	9.525E+09	10046000	2.55E+09	29144000	6.94E+09	4280000	2.78E+08	15621000	2.41E+08
P62241	P62241	40S ribosomal protein S8	RPS8	sp|P62241	11	11	11	8	11	10	9	8	11	10	9	8	11	10	9	51.9	24.205	0	82.603	9.492E+09	2.68E+08	3.08E+09	596610000	5.55E+09	2.4E+08	2.81E+08	433670000	1.96E+08
P02786;Q9	P02786	Transferrin receptor protein 1;Transferrin recepto	TFRC	sp|P02786	31	31	31	0	23	16	31	0	23	16	31	0	23	16	31	50.4	84.87	0	176.9	9.354E+09	0	7.07E+08	63072000	8.58E+09	0	66570000	62240000	3.27E+08
Q13263	Q13263	Transcription intermediary factor 1-beta	TRIM28	sp|Q1326	28	28	28	5	28	22	27	5	28	22	27	5	28	22	27	52.1	88.549	0	198.52	9.309E+09	1.21E+08	3.45E+09	283250000	5.46E+09	1.8E+08	2.55E+08	170730000	2.14E+08
Q9NQC3	Q9NQC3	Reticulon-4	RTN4	sp|Q9NQC	14	14	14	2	7	7	14	2	7	7	14	2	7	7	14	23.2	129.93	0	140.29	9.273E+09	2745300	1.05E+08	119480000	9.04E+09	7189200	10776000	48300000	3.95E+08
P00918	P00918	Carbonic anhydrase 2	CA2	sp|P00918	16	16	16	3	16	1	2	3	16	1	2	3	16	1	2	63.5	29.246	0	101.99	9.259E+09	4084800	9.23E+09	1094200	25468000	553080	7.24E+08	234660	280060
P41252	P41252	Isoleucine--tRNA ligase, cytoplasmic	IARS	sp|P41252	43	43	43	13	41	24	40	13	41	24	40	13	41	24	40	43.2	144.5	0	323.31	9.194E+09	5.03E+08	3.58E+09	185770000	4.93E+09	3.6E+08	3.49E+08	227740000	2.04E+08
P52209	P52209	6-phosphogluconate dehydrogenase, decarboxyla	PGD	sp|P52209	19	19	19	0	16	5	18	0	16	5	18	0	16	5	18	47.2	53.139	0	209.06	9.187E+09	0	1.9E+09	12380000	7.28E+09	0	1.69E+08	7447400	2.94E+08
P62191	P62191	26S protease regulatory subunit 4	PSMC1	sp|P62191	21	21	21	3	19	15	20	3	19	15	20	3	19	15	20	57.3	49.184	0	119.96	9.102E+09	3216000	1.74E+09	128560000	7.23E+09	1936600	1.32E+08	163370000	2.41E+08
O14980	O14980	Exportin-1	XPO1	sp|O1498	35	35	35	1	30	20	34	1	30	20	34	1	30	20	34	41.9	123.38	0	232.27	9.044E+09	953980	2.94E+09	120990000	5.98E+09	5717200	4.19E+08	49110000	1.08E+08
P08865	P08865	40S ribosomal protein SA	RPSA	sp|P08865	12	12	12	8	11	9	11	8	11	9	11	8	11	9	11	45.8	32.854	0	189.79	9.036E+09	1.86E+09	2.78E+09	83472000	4.31E+09	1.93E+09	1.46E+08	87693000	1.54E+08
P78347;Q8	P78347	General transcription factor II-I	GTF2I	sp|P78347	47	47	47	29	37	45	31	29	37	45	31	29	37	45	31	53.1	112.42	0	267.67	9.015E+09	1.61E+09	1.85E+09	3.341E+09	2.21E+09	1.8E+09	1.51E+08	1.957E+09	1.01E+08
P55884	P55884	Eukaryotic translation initiation factor 3 subunit B	EIF3B	sp|P55884	37	37	37	19	33	24	36	19	33	24	36	19	33	24	36	57.4	92.48	0	277.96	8.996E+09	1.91E+09	1.93E+09	161430000	4.99E+09	1.89E+09	1.6E+08	149040000	1.98E+08
P23246	P23246	Splicing factor, proline- and glutamine-rich	SFPQ	sp|P23246	28	28	27	19	26	25	25	19	26	25	25	19	25	24	25	47.8	76.149	0	177.82	8.996E+09	8.09E+08	4.07E+09	805360000	3.31E+09	8.87E+08	3.15E+08	507100000	1.36E+08
P16152	P16152	Carbonyl reductase [NADPH] 1	CBR1	sp|P16152	15	15	13	11	14	15	15	11	14	15	15	9	12	13	13	70.4	30.375	0	135.85	8.961E+09	1.23E+09	2.32E+09	2.231E+09	3.17E+09	1.53E+09	2.29E+08	1.171E+09	1.36E+08
P40925	P40925	Malate dehydrogenase, cytoplasmic	MDH1	sp|P40925	11	11	11	0	10	6	11	0	10	6	11	0	10	6	11	38.9	36.426	0	87.197	8.917E+09	0	3.08E+09	12832000	5.83E+09	0	2.87E+08	11402000	2.05E+08
P17980	P17980	26S protease regulatory subunit 6A	PSMC3	sp|P17980	24	24	24	3	24	16	23	3	24	16	23	3	24	16	23	64.5	49.203	0	252.96	8.852E+09	1554000	3.39E+09	125410000	5.33E+09	1148500	3.77E+08	28991000	1.88E+08
P46781	P46781	40S ribosomal protein S9	RPS9	sp|P46781	17	17	17	11	16	13	17	11	16	13	17	11	16	13	17	58.8	22.591	0	42.909	8.704E+09	5.26E+08	2.48E+09	415640000	5.29E+09	1.93E+08	2.83E+08	496730000	2.43E+08
P15121;C9	P15121	Aldose reductase	AKR1B1	sp|P15121	12	12	12	0	9	7	12	0	9	7	12	0	9	7	12	45.6	35.853	0	116.29	8.691E+09	0	5.15E+08	22917000	8.15E+09	0	63698000	14798000	3.11E+08
P62424	P62424	60S ribosomal protein L7a	RPL7A	sp|P62424	17	17	17	11	15	17	15	11	15	17	15	11	15	17	15	45.1	29.995	0	69.944	8.67E+09	4.48E+08	2.26E+09	1.011E+09	4.95E+09	5.74E+08	1.93E+08	539940000	2.08E+08
P99999;CC	P99999	Cytochrome c	CYCS	sp|P99999	9	9	9	0	9	4	9	0	9	4	9	0	9	4	9	62.9	11.749	0	65.878	8.665E+09	0	3.79E+09	13561000	4.86E+09	0	3.14E+08	8744800	2.09E+08
P08708	P08708	40S ribosomal protein S17	RPS17	sp|P08708	10	10	10	2	9	7	10	2	9	7	10	2	9	7	10	65.9	15.55	0	92.92	8.64E+09	27861000	4.31E+09	321900000	3.98E+09	29627000	2.87E+08	281020000	1.83E+08
P67809	P67809	Nuclease-sensitive element-binding protein 1	YBX1	sp|P67809	18	18	12	10	17	17	18	10	17	17	18	4	11	11	12	71.6	35.924	0	142.02	8.578E+09	3.32E+08	2.06E+09	1.236E+09	4.95E+09	3.2E+08	1.87E+08	843080000	1.7E+08
P30086	P30086	Phosphatidylethanolamine-binding protein 1;Hip	PEBP1	sp|P30086	12	12	12	0	12	4	11	0	12	4	11	0	12	4	11	80.7	21.057	0	130.84	8.565E+09	0	4.26E+09	14513000	4.29E+09	0	3.94E+08	13624000	1.51E+08
P40939	P40939	Trifunctional enzyme subunit alpha, mitochondria	HADHA	sp|P40939	27	27	27	7	23	16	26	7	23	16	26	7	23	16	26	46.4	82.999	0	298.04	8.541E+09	36999000	2.05E+09	41730000	6.42E+09	81694000	1.92E+08	40039000	1.95E+08
P0DMV9;P	P0DMV9;P	Heat shock 70 kDa protein 1B;Heat shock 70 kDa	HSPA1B;H	sp|P0DMV	25	21	11	12	25	22	23	12	21	18	19	7	11	9	10	46.8	70.051	0	174.36	8.492E+09	6.43E+08	4.85E+09	251080000	2.75E+09	5.56E+08	4.25E+08	197340000	1.07E+08
P43686	P43686	26S protease regulatory subunit 6B	PSMC4	sp|P43686	27	27	27	5	26	15	24	5	26	15	24	5	26	15	24	73.7	47.366	0	223.33	8.491E+09	92368000	2.69E+09	76569000	5.63E+09	1.29E+08	2.19E+08	63696000	2.02E+08
P35580;P1	P35580	Myosin-10	MYH10	sp|P35580	86	69	64	47	69	18	20	42	57	5	4	38	52	5	4	49.5	229	0	323.31	8.469E+09	5.13E+09	3.26E+09	18396000	57254000	4.56E+09	8.22E+08	38205000	2401100
P22102	P22102	Trifunctional purine biosynthetic protein adenosi	GART	sp|P22102	33	33	33	3	32	5	30	3	32	5	30	3	32	5	30	40.6	107.77	0	225.11	8.464E+09	2497700	4.11E+09	35855000	4.32E+09	1362400	3.71E+08	9436900	1.72E+08
P49588	P49588	Alanine--tRNA ligase, cytoplasmic	AARS	sp|P49588	40	40	40	2	40	6	40	2	40	6	40	2	40	6	40	52.7	106.81	0	323.31	8.462E+09	1239100	2.48E+09	21894000	5.96E+09	743290	2.13E+08	782410	2.57E+08
Q9H4M9	Q9H4M9	EH domain-containing protein 1	EHD1	sp|Q9H4M	29	29	20	0	17	12	28	0	17	12	28	0	12	8	20	69.5	60.626	0	185.17	8.361E+09	0	4.3E+08	55776000	7.88E+09	0	41146000	65500000	2.81E+08
O00299	O00299	Chloride intracellular channel protein 1	CLIC1	sp|O0029	11	11	11	0	9	3	11	0	9	3	11	0	9	3	11	60.6	26.922	0	166.21	8.35E+09	0	1.63E+09	11707000	6.71E+09	0	1.37E+08	14353000	2.73E+08
O75369	O75369	Filamin-B	FLNB	sp|O7536	87	75	75	3	62	36	86	1	56	27	74	1	56	27	74	45.5	278.16	0	323.31	8.305E+09	631000	1.3E+09	80016000	6.93E+09	7390400	2.3E+08	43257000	1.71E+08
Q15366	Q15366	Poly(rC)-binding protein 2	PCBP2	sp|Q1536	17	12	12	2	17	11	16	1	12	7	11	1	12	7	11	53.2	38.58	0	133.48	8.295E+09	1419200	2.23E+09	59674000	6E+09	1815200	1.93E+08	22621000	2E+08
P30153	P30153	Serine/threonine-protein phosphatase 2A 65 kDa	PPP2R1A	sp|P30153	20	20	15	3	14	13	20	3	14	13	20	3	11	10	15	41.4	65.308	0	157.37	8.295E+09	12927000	1.19E+09	132720000	6.95E+09	18710000	1.53E+08	51611000	2.69E+08
P52597	P52597	Heterogeneous nuclear ribonucleoprotein F;Hete	HNRNPF	sp|P52597	12	10	10	7	11	11	12	5	9	9	10	5	9	9	10	48.2	45.671	0	212.16	8.29E+09	2.63E+08	2.54E+09	509020000	4.98E+09	3.2E+08	2E+08	321630000	1.61E+08
P15531	P15531	Nucleoside diphosphate kinase A	NME1	sp|P15531	13	7	7	4	11	5	13	2	6	3	7	2	6	3	7	86.2	17.149	0	69.933	8.223E+09	13436000	3.74E+09	7169300	4.46E+09	13131000	2.87E+08	5525800	2.23E+08
P62333	P62333	26S protease regulatory subunit 10B	PSMC6	sp|P62333	15	15	15	0	14	11	15	0	14	11	15	0	14	11	15	49.1	44.172	0	195.89	8.175E+09	0	2.67E+09	73946000	5.43E+09	0	2.29E+08	76941000	1.89E+08
P60228	P60228	Eukaryotic translation initiation factor 3 subunit E	EIF3E	sp|P60228	20	20	20	12	20	16	20	12	20	16	20	12	20	16	20	51.9	52.22	0	109.06	8.167E+09	4.24E+08	2.16E+09	134460000	5.45E+09	5.17E+08	1.41E+08	105790000	1.47E+08
P36776	P36776	Lon protease homolog, mitochondrial	LONP1	sp|P36776	30	30	30	2	24	12	28	2	24	12	28	2	24	12	28	44.1	106.49	0	323.31	8.144E+09	2154800	1.55E+09	43861000	6.55E+09	2592300	1.51E+08	19073000	2.63E+08
Q99714	Q99714	3-hydroxyacyl-CoA dehydrogenase type-2	HSD17B10	sp|Q9971	15	15	15	4	14	3	13	4	14	3	13	4	14	3	13	83.9	26.923	0	208.74	8.081E+09	54156000	4.21E+09	12507000	3.81E+09	32326000	3.92E+08	8365800	1.48E+08
Q07020	Q07020	60S ribosomal protein L18	RPL18	sp|Q0702	10	10	10	5	9	10	10	5	9	10	10	5	9	10	10	43.1	21.634	0	65.195	8.067E+09	72510000	2.33E+09	1.069E+09	4.6E+09	2.53E+08	1.65E+08	587890000	1.6E+08
A8K2U0	A8K2U0	Alpha-2-macroglobulin-like protein 1	A2ML1	sp|A8K2U	4	4	4	0	0	4	0	0	0	4	0	0	0	4	0	3.2	161.1	0	4.9258	8.067E+09	0	0	8.067E+09	0	0	0	5.421E+09	0
P13693;Q5	P13693	Translationally-controlled tumor protein	TPT1	sp|P13693	8	8	8	0	8	5	8	0	8	5	8	0	8	5	8	57	19.595	0	95.322	8.013E+09	0	9.79E+08	15388000	7.02E+09	0	87775000	15296000	2.82E+08
P33992	P33992	DNA replication licensing factor MCM5	MCM5	sp|P33992	33	33	33	0	25	17	33	0	25	17	33	0	25	17	33	53.8	82.285	0	286.75	7.97E+09	0	6.55E+08	113690000	7.2E+09	0	68854000	91685000	2.66E+08
Q96AG4	Q96AG4	Leucine-rich repeat-containing protein 59	LRRC59	sp|Q96AG	14	14	14	5	12	12	13	5	12	12	13	5	12	12	13	49.5	34.93	0	166	7.91E+09	4.18E+08	2.26E+09	419420000	4.82E+09	6.08E+08	97903000	299530000	86662000
Q8WUM4	Q8WUM4	Programmed cell death 6-interacting protein	PDCD6IP	sp|Q8WU	34	34	34	1	30	15	33	1	30	15	33	1	30	15	33	47.6	96.022	0	263.07	7.906E+09	853690	2.34E+09	48217000	5.52E+09	1087000	2.08E+08	9747500	2.4E+08
O95433	O95433	Activator of 90 kDa heat shock protein ATPase ho	AHSA1	sp|O9543	20	20	20	0	18	7	19	0	18	7	19	0	18	7	19	65.1	38.274	0	157.83	7.859E+09	0	1.93E+09	21047000	5.91E+09	0	1.59E+08	22522000	2.31E+08
O60506	O60506	Heterogeneous nuclear ribonucleoprotein Q	SYNCRIP	sp|O6050	26	26	22	9	21	22	25	9	21	22	25	7	17	19	21	53.8	69.602	0	320.14	7.764E+09	3.25E+08	1.71E+09	400370000	5.33E+09	2.69E+08	1.59E+08	345630000	1.83E+08
O00571;O	O00571;O	ATP-dependent RNA helicase DDX3X;ATP-depend	DDX3X;DD	sp|O0057	32	32	31	11	27	28	29	11	27	28	29	10	26	27	28	56.8	73.243	0	286.34	7.758E+09	8.12E+08	1.75E+09	589470000	4.61E+09	6.04E+08	2.11E+08	503750000	1.92E+08
P68036	P68036	Ubiquitin-conjugating enzyme E2 L3	UBE2L3	sp|P68036	8	8	4	1	8	1	7	1	8	1	7	0	4	1	4	68.8	17.861	0	113.75	7.738E+09	880790	3.89E+09	3240700	3.84E+09	441060	3.39E+08	806460	1.63E+08
P12004	P12004	Proliferating cell nuclear antigen	PCNA	sp|P12004	14	14	14	2	14	6	14	2	14	6	14	2	14	6	14	70.1	28.768	0	151.51	7.717E+09	54203000	3.81E+09	29849000	3.83E+09	34243000	3.4E+08	14796000	1.56E+08
O94808	O94808	Glutamine--fructose-6-phosphate aminotransfera	GFPT2	sp|O9480	31	31	26	1	10	24	30	1	10	24	30	0	5	19	26	52.8	76.93	0	211.53	7.714E+09	175310	2.2E+08	234080000	7.26E+09	295370	25801000	126440000	3.22E+08
Q9Y265	Q9Y265	RuvB-like 1	RUVBL1	sp|Q9Y26	23	23	23	4	21	18	21	4	21	18	21	4	21	18	21	62.3	50.227	0	171.38	7.703E+09	1.74E+08	3.19E+09	207530000	4.13E+09	1.07E+08	3.19E+08	182730000	1.46E+08
P30050	P30050	60S ribosomal protein L12	RPL12	sp|P30050	8	8	8	3	7	8	8	3	7	8	8	3	7	8	8	63.6	17.818	0	317.27	7.702E+09	1.49E+09	1.79E+09	839550000	3.58E+09	1.44E+09	1.42E+08	553200000	1.45E+08
Q13310;P0	Q13310	Polyadenylate-binding protein 4	PABPC4	sp|Q1331	30	30	19	11	23	24	26	11	23	24	26	9	14	14	16	50.3	70.782	0	239.22	7.68E+09	1.08E+08	1.91E+09	595530000	5.07E+09	1.7E+08	1.78E+08	347330000	1.79E+08
P62269	P62269	40S ribosomal protein S18	RPS18	sp|P62269	14	14	14	9	13	14	11	9	13	14	11	9	13	14	11	66.4	17.718	0	56.751	7.677E+09	1.18E+09	2.23E+09	478670000	3.79E+09	1.01E+09	2.19E+08	385790000	2.13E+08
P25205	P25205	DNA replication licensing factor MCM3	MCM3	sp|P25205	35	35	35	2	28	27	35	2	28	27	35	2	28	27	35	55.9	90.98	0	323.31	7.633E+09	2678500	1.02E+09	149270000	6.46E+09	5337500	46303000	117270000	2.85E+08
P61313	P61313	60S ribosomal protein L15	RPL15	sp|P61313	14	14	14	8	13	13	13	8	13	13	13	8	13	13	13	50	24.146	0	61.216	7.591E+09	2.07E+08	2.2E+09	658310000	4.52E+09	2.87E+08	1.41E+08	423790000	1.44E+08
P26373	P26373	60S ribosomal protein L13	RPL13	sp|P26373	12	12	12	7	11	12	11	7	11	12	11	7	11	12	11	50.7	24.261	0	87.32	7.588E+09	1.69E+08	2.29E+09	848110000	4.29E+09	2.26E+08	1.7E+08	537970000	1.52E+08
P07954	P07954	Fumarate hydratase, mitochondrial	FH	sp|P07954	16	16	16	0	15	0	16	0	15	0	16	0	15	0	16	50.4	54.636	0	149.62	7.554E+09	0	2.64E+09	0	4.91E+09	0	2.39E+08	0	1.96E+08
O00232	O00232	26S proteasome non-ATPase regulatory subunit 1	PSMD12	sp|O0023	20	20	20	2	17	13	18	2	17	13	18	2	17	13	18	49.6	52.904	0	133.99	7.525E+09	817370	1.96E+09	78542000	5.49E+09	3994700	73756000	32890000	3.41E+08
P53618	P53618	Coatomer subunit beta	COPB1	sp|P53618	30	30	30	4	25	16	29	4	25	16	29	4	25	16	29	43	107.14	0	170.28	7.497E+09	8.69E+08	1.56E+09	66684000	5E+09	1.78E+08	1.79E+08	138170000	7.54E+08
Q9Y4L1;Q5	Q9Y4L1	Hypoxia up-regulated protein 1	HYOU1	sp|Q9Y4L1	39	39	39	4	38	2	29	4	38	2	29	4	38	2	29	55.4	111.33	0	311.78	7.467E+09	1964400	4.92E+09	849460	2.55E+09	770530	4.1E+08	273480	1.29E+08
P62195	P62195	26S protease regulatory subunit 8	PSMC5	sp|P62195	20	20	19	3	19	15	19	3	19	15	19	2	18	14	18	60.1	45.626	0	251.98	7.439E+09	3471700	2.46E+09	87117000	4.89E+09	2834700	2.16E+08	62189000	1.88E+08
P05455	P05455	Lupus La protein	SSB	sp|P05455	28	28	28	5	28	13	25	5	28	13	25	5	28	13	25	57.6	46.836	0	120.21	7.417E+09	2.09E+08	4.14E+09	59613000	3.01E+09	2.18E+08	3.38E+08	54398000	1.21E+08
Q96TA1	Q96TA1	Niban-like protein 1	FAM129B	sp|Q96TA	23	23	23	1	11	8	23	1	11	8	23	1	11	8	23	48.5	84.137	0	236.95	7.402E+09	96225000	1.98E+08	18662000	7.09E+09	47147000	48072000	29933000	2.88E+08
P63010	P63010	AP-2 complex subunit beta	AP2B1	sp|P63010	32	32	21	0	26	16	29	0	26	16	29	0	18	10	20	41.4	104.55	0	323.31	7.397E+09	0	8.03E+08	54478000	6.54E+09	0	78125000	63095000	2.34E+08
P62701;Q8	P62701	40S ribosomal protein S4, X isoform	RPS4X	sp|P62701	14	14	14	9	12	13	13	9	12	13	13	9	12	13	13	52.1	29.597	0	63.993	7.362E+09	4.14E+08	2.19E+09	512730000	4.25E+09	6.85E+08	1.22E+08	193440000	1.24E+08
Q9Y262	Q9Y262	Eukaryotic translation initiation factor 3 subunit L	EIF3L	sp|Q9Y26	27	27	27	15	24	19	26	15	24	19	26	15	24	19	26	51.8	66.726	0	179.16	7.312E+09	1.74E+09	1.2E+09	115700000	4.26E+09	1.28E+09	2.6E+08	214860000	3.41E+08
P02545	P02545	Prelamin-A/C;Lamin-A/C	LMNA	sp|P02545	39	39	39	1	20	35	39	1	20	35	39	1	20	35	39	60.4	74.139	0	312.32	7.284E+09	8.52E+08	1.32E+08	635690000	5.66E+09	4.69E+08	30984000	803910000	2.11E+08
P84085	P84085	ADP-ribosylation factor 5	ARF5	sp|P84085	10	10	5	1	10	4	10	1	10	4	10	1	5	0	5	56.7	20.529	0	57.938	7.25E+09	504430	2.07E+09	24337000	5.15E+09	2385500	3E+08	11827000	89758000
P35998	P35998	26S protease regulatory subunit 7	PSMC2	sp|P35998	30	30	30	3	29	21	30	3	29	21	30	3	29	21	30	67.2	48.633	0	208.37	7.249E+09	11841000	1.83E+09	126030000	5.28E+09	10719000	1.41E+08	88960000	2.3E+08
Q01813	Q01813	ATP-dependent 6-phosphofructokinase, platelet t	PFKP	sp|Q0181	31	31	27	2	23	18	28	2	23	18	28	0	19	14	25	43.6	85.595	0	225.49	7.242E+09	1166500	6.99E+08	115330000	6.43E+09	3477600	28989000	40156000	3.29E+08
P41250	P41250	Glycine--tRNA ligase	GARS	sp|P41250	27	27	27	3	24	5	25	3	24	5	25	3	24	5	25	50.6	83.165	0	323.31	7.214E+09	23840000	2.11E+09	8792700	5.07E+09	13414000	1.97E+08	4916500	1.97E+08
P21796	P21796	Voltage-dependent anion-selective channel prote	VDAC1	sp|P21796	18	18	18	4	18	9	17	4	18	9	17	4	18	9	17	83.4	30.772	0	144.15	7.212E+09	72475000	2.72E+09	46070000	4.37E+09	1.07E+08	1.8E+08	31637000	1.69E+08
Q99460	Q99460	26S proteasome non-ATPase regulatory subunit 1	PSMD1	sp|Q9946	30	30	30	7	27	21	29	7	27	21	29	7	27	21	29	42.2	105.84	0	237.19	7.21E+09	10992000	1.86E+09	77275000	5.26E+09	4749200	2.45E+08	32939000	1.6E+08
Q99880;Q	Q99880;Q	Histone H2B type 1-L;Histone H2B type 1-M;Histo	HIST1H2BL	sp|Q9988	8	8	2	3	7	5	8	3	7	5	8	0	2	2	2	53.2	13.952	0	32.206	7.155E+09	93668000	1.42E+09	705120000	4.93E+09	85433000	1.29E+08	468270000	1.32E+08
Q9Y3I0	Q9Y3I0	tRNA-splicing ligase RtcB homolog	RTCB	sp|Q9Y3I0	25	25	25	4	24	22	24	4	24	22	24	4	24	22	24	58.8	55.21	0	166.44	7.139E+09	12893000	1.97E+09	464730000	4.69E+09	10300000	1.7E+08	360620000	1.43E+08
P33176	P33176	Kinesin-1 heavy chain	KIF5B	sp|P33176	42	42	34	2	31	19	42	2	31	19	42	2	24	13	34	57.3	109.68	0	323.31	7.131E+09	1466900	1E+09	51121000	6.08E+09	2377200	85326000	54544000	2.27E+08

P05198	P05198	Eukaryotic translation initiation factor 2 subunit 1	EIF2S1	sp|P05198	18	18	18	7	15	12	18	7	15	12	18	7	15	12	18	60.6	36.112	0	110.76	7.071E+09	2.9E+08	1.02E+09	94067000	5.66E+09	3.26E+08	73292000	95886000	1.79E+08
P33991	P33991	DNA replication licensing factor MCM4	MCM4	sp|P33991	30	30	30	1	19	11	30	1	19	11	30	1	19	11	30	49.4	96.557	0	258.11	7.06E+09	279000	5.61E+08	46178000	6.45E+09	898710	17429000	15827000	3.1E+08
P55786;A6	P55786	Puromycin-sensitive aminopeptidase	NPEPPS	sp|P55786	33	33	33	0	31	13	29	0	31	13	29	0	31	13	29	46.8	103.28	0	262.04	7.054E+09	0	3.62E+09	34989000	3.4E+09	0	2.98E+08	31571000	1.53E+08
P14868	P14868	Aspartate--tRNA ligase, cytoplasmic	DARS	sp|P14868	30	30	30	10	30	21	29	10	30	21	29	10	30	21	29	65.7	57.136	0	130.49	7.016E+09	1.19E+08	2.39E+09	135130000	4.38E+09	1.89E+08	1.78E+08	100050000	1.29E+08
P23284	P23284	Peptidyl-prolyl cis-trans isomerase B	PPIB	sp|P23284	13	13	13	1	12	10	13	1	12	10	13	1	12	10	13	51.9	23.742	0	71.2	7.013E+09	1621200	1.87E+09	76561000	5.07E+09	3961300	71980000	50854000	2.66E+08
P08133	P08133	Annexin A6	ANXA6	sp|P08133	29	29	29	0	20	4	28	0	20	4	28	0	20	4	28	55	75.872	0	323.31	6.974E+09	0	6.61E+08	7495400	6.31E+09	0	59813000	6335500	2.56E+08
P54577	P54577	Tyrosine--tRNA ligase, cytoplasmic;Tyrosine--tRN	YARS	sp|P54577	29	29	29	3	29	0	23	3	29	0	23	3	29	0	23	58.7	59.143	0	120.41	6.953E+09	3738100	3.55E+09	0	3.39E+09	2369800	2.78E+08	0	1.64E+08
P27348	P27348	14-3-3 protein theta	YWHAQ	sp|P27348	18	13	12	4	17	13	18	3	12	9	13	3	11	8	12	54.7	27.764	0	311.01	6.911E+09	32663000	1.54E+09	43622000	5.29E+09	64649000	1.27E+08	19982000	1.96E+08
P46060	P46060	Ran GTPase-activating protein 1	RANGAP1	sp|P46060	25	25	25	5	19	15	24	5	19	15	24	5	19	15	24	57.4	63.541	0	235.73	6.803E+09	24463000	7.55E+08	53464000	5.97E+09	44212000	60718000	38717000	2.14E+08
Q04837	Q04837	Single-stranded DNA-binding protein, mitochondi	SSBP1	sp|Q04837	9	9	9	0	9	8	9	0	9	8	9	0	9	8	9	58.8	17.259	0	86.791	6.797E+09	0	2.71E+09	230710000	3.85E+09	0	2.17E+08	148770000	1.41E+08
P45974	P45974	Ubiquitin carboxyl-terminal hydrolase 5	USP5	sp|P45974	27	27	26	0	25	5	26	0	25	5	26	0	24	5	25	48.5	95.785	0	216.02	6.731E+09	0	1.57E+09	14356000	5.15E+09	0	1.42E+08	12314000	2.05E+08
Q92598	Q92598	Heat shock protein 105 kDa	HSPH1	sp|Q92598	28	26	25	3	25	15	27	2	23	15	25	2	22	15	24	43.4	96.864	0	255.83	6.705E+09	7398900	1.43E+09	40551000	5.22E+09	19806000	1.22E+08	27831000	2.06E+08
P62244	P62244	40S ribosomal protein S15a	RPS15A	sp|P62244	7	7	7	5	6	7	7	5	6	7	7	5	6	7	7	59.2	14.839	0	66.383	6.676E+09	7.41E+08	1.63E+09	468590000	3.84E+09	3.93E+08	2.08E+08	541740000	1.7E+08
Q14103	Q14103	Heterogeneous nuclear ribonucleoprotein D0	HNRNPD	sp|Q14103	13	13	12	5	12	10	12	5	12	10	12	4	11	9	11	37.2	38.434	0	122.37	6.653E+09	2.48E+08	2.36E+09	183120000	3.86E+09	2.32E+08	1.52E+08	126970000	2.26E+08
P46777	P46777	60S ribosomal protein L5	RPL5	sp|P46777	15	15	15	7	13	14	14	7	13	14	14	7	13	14	14	48.1	34.362	0	48.604	6.617E+09	1.87E+08	2E+09	685210000	3.74E+09	1.68E+08	1.64E+08	492050000	1.43E+08
O43242	O43242	26S proteasome non-ATPase regulatory subunit 3	PSMD3	sp|O43242	29	29	29	9	23	13	29	9	23	13	29	9	23	13	29	58.6	60.977	0	169.36	6.594E+09	39409000	1.55E+09	41430000	4.96E+09	9081900	1.89E+08	34961000	1.74E+08
P43487	P43487	Ran-specific GTPase-activating protein	RANBP1	sp|P43487	8	8	8	1	8	2	7	1	8	2	7	1	8	2	7	64.7	23.31	0	53.627	6.556E+09	2190500	3.51E+09	3875900	3.04E+09	1060900	3.11E+08	1190700	1.11E+08
P54136	P54136	Arginine--tRNA ligase, cytoplasmic	RARS	sp|P54136	34	34	34	9	34	19	32	9	34	19	32	9	34	19	32	56.8	75.378	0	131.59	6.552E+09	55170000	2.15E+09	128700000	4.22E+09	1.25E+08	1.43E+08	101660000	1.27E+08
P34897	P34897	Serine hydroxymethyltransferase, mitochondrial	SHMT2	sp|P34897	22	22	21	4	21	6	20	4	21	6	20	4	20	5	19	56	55.992	0	201.96	6.545E+09	5442700	2.88E+09	17068000	3.64E+09	7809700	3.14E+08	7780100	90123000
P67936	P67936	Tropomyosin alpha-4 chain	TPM4	sp|P67936	21	21	10	5	19	16	21	5	19	16	21	3	9	7	10	57.7	28.521	0	107.98	6.52E+09	7679200	1.4E+09	123250000	4.99E+09	4469600	75202000	131000000	2.07E+08
O75533	O75533	Splicing factor 3B subunit 1	SF3B1	sp|O75533	45	45	45	10	42	31	43	10	42	31	43	10	42	31	43	49.9	145.83	0	323.31	6.509E+09	34109000	2.41E+09	177400000	3.89E+09	35256000	2.11E+08	138180000	1.36E+08
P49736	P49736	DNA replication licensing factor MCM2	MCM2	sp|P49736	30	30	30	0	19	16	29	0	19	16	29	0	19	16	29	47.7	101.89	0	323.31	6.489E+09	0	6.56E+08	50556000	5.78E+09	0	69624000	46932000	2.07E+08
Q92499	Q92499	ATP-dependent RNA helicase DDX1	DDX1	sp|Q92499	28	28	28	7	25	23	25	7	25	23	25	7	25	23	25	45.1	82.431	0	323.31	6.46E+09	1.75E+08	1.39E+09	318520000	4.57E+09	2.03E+08	1.2E+08	229110000	1.42E+08
P09972	P09972	Fructose-bisphosphate aldolase C	ALDOC	sp|P09972	19	15	15	0	17	2	15	0	13	2	11	0	13	2	11	56.6	39.455	0	125.24	6.455E+09	0	2.03E+09	9321900	4.41E+09	0	1.08E+08	6240000	1.74E+08
Q99798	Q99798	Aconitate hydratase, mitochondrial	ACO2	sp|Q99798	30	30	30	0	27	1	29	0	27	1	29	0	27	1	29	48.1	85.424	0	244.95	6.428E+09	0	2.12E+09	359950	4.3E+09	0	40705000	1627800	3.18E+08
Q9Y230	Q9Y230	RuvB-like 2	RUVBL2	sp|Q9Y230	30	30	30	11	29	24	28	11	29	24	28	11	29	24	28	67.4	51.156	0	165.62	6.419E+09	73744000	2.92E+09	274850000	3.15E+09	1.82E+08	1.86E+08	179400000	95456000
P62753	P62753	40S ribosomal protein S6	RPS6	sp|P62753	11	11	11	2	9	10	10	2	9	10	10	2	9	10	10	33.3	28.68	0	45.898	6.378E+09	11536000	2.27E+09	451070000	3.64E+09	21096000	2.23E+08	232130000	1.65E+08
Q15233	Q15233	Non-POU domain-containing octamer-binding pr	NONO	sp|Q15233	24	23	22	12	23	19	18	12	22	18	18	12	21	17	17	55.6	54.231	0	151.97	6.375E+09	4.33E+08	3.04E+09	487600000	2.41E+09	3.96E+08	2.64E+08	361550000	1.06E+08
P25787	P25787	Proteasome subunit alpha type-2	PSMA2	sp|P25787	11	11	11	1	10	6	10	1	10	6	10	1	10	6	10	57.3	25.898	0	124.66	6.36E+09	2173300	1.73E+09	49520000	4.58E+09	0	1.25E+08	51576000	1.92E+08
P26639;A2	P26639	Threonine--tRNA ligase, cytoplasmic	TARS	sp|P26639	27	27	27	1	25	6	25	1	25	6	25	1	25	6	25	36.2	83.434	0	133.69	6.358E+09	1043600	2.01E+09	14102000	4.33E+09	3120800	2.72E+08	5217500	84335000
Q15019	Q15019	Septin-2	Sep-02	sp|Q15019	12	12	12	1	12	9	11	1	12	9	11	1	12	9	11	52.6	41.487	0	169.82	6.328E+09	616670	1.3E+09	55567000	4.97E+09	3794800	1.99E+08	71628000	77380000
P11940;Q9	P11940	Polyadenylate-binding protein 1	PABPC1	sp|P11940	29	19	18	17	23	25	28	15	15	16	19	14	14	15	18	55	70.67	0	110.04	6.306E+09	2.75E+08	1.02E+09	542310000	4.47E+09	1.57E+08	1.17E+08	438770000	1.85E+08
P17812	P17812	CTP synthase 1	CTPS1	sp|P17812	21	21	18	3	21	5	20	3	21	5	20	3	18	4	18	44.3	66.69	0	165.15	6.296E+09	6315100	1.99E+09	9355800	4.29E+09	11172000	2.1E+08	6273300	1.38E+08
Q00341	Q00341	Vigilin	HDLBP	sp|Q00341	45	45	45	2	33	28	44	2	33	28	44	2	33	28	44	44.2	141.45	0	235.65	6.246E+09	3333200	9.99E+08	145220000	5.1E+09	6410200	1.1E+08	58039000	2.22E+08
Q86UP2;Q	Q86UP2	Kinectin	KTN1	sp|Q86UP2	57	57	57	4	44	40	55	4	44	40	55	4	44	40	55	49.7	156.27	0	270.65	6.198E+09	1902600	1.3E+09	233070000	4.66E+09	1581700	1.04E+08	189850000	1.69E+08
P50454	P50454	Serpin H1	SERPINH1	sp|P50454	22	22	22	1	17	13	22	1	17	13	22	1	17	13	22	65.3	46.44	0	137.07	6.178E+09	412840	7.16E+08	87788000	5.37E+09	1597100	25177000	28025000	2.87E+08
P04181	P04181	Ornithine aminotransferase, mitochondrial;Ornitl	OAT	sp|P04181	17	17	17	1	16	1	15	1	16	1	15	1	16	1	15	56.7	48.534	0	110.07	6.178E+09	971010	3.93E+09	1605600	2.24E+09	467370	3.4E+08	501400	95119000
P39656	P39656	Dolichyl-diphosphooligosaccharide--protein glyco	DDOST	sp|P39656	14	14	14	2	11	10	14	2	11	10	14	2	11	10	14	49.3	50.8	0	157.09	6.111E+09	995040	7.73E+08	108790000	5.23E+09	996850	1.06E+08	70468000	1.76E+08
P62829	P62829	60S ribosomal protein L23	RPL23	sp|P62829	8	8	8	1	7	8	6	1	7	8	6	1	7	8	6	64.3	14.865	0	38.609	6.107E+09	8.28E+08	2.05E+09	421060000	2.8E+09	9.55E+08	1.4E+08	218550000	93508000
O00231	O00231	26S proteasome non-ATPase regulatory subunit 1	PSMD11	sp|O00231	26	26	26	8	24	15	22	8	24	15	22	8	24	15	22	61.6	47.463	0	137.96	6.042E+09	83649000	2.17E+09	72458000	3.71E+09	1.34E+08	1.27E+08	64613000	1.51E+08
P61163	P61163	Alpha-centractin	ACTR1A	sp|P61163	12	12	5	2	12	7	11	2	12	7	11	1	5	3	5	48.9	42.613	0	215.88	6.007E+09	1.23E+08	2.32E+09	71635000	3.49E+09	9763900	1.19E+08	35618000	3.38E+08
Q04828;P1	Q04828	Aldo-keto reductase family 1 member C1	AKR1C1	sp|Q04828	9	9	0	0	0	2	9	0	0	2	9	0	0	0	0	46.7	36.788	0	104.01	6.001E+09	0	0	8514200	5.99E+09	0	0	5721500	2.43E+08
P62906	P62906	60S ribosomal protein L10a	RPL10A	sp|P62906	11	11	11	5	10	9	10	5	10	9	10	5	10	9	10	41.9	24.831	0	33.111	5.98E+09	1.55E+08	1.67E+09	829010000	3.33E+09	83140000	1.44E+08	576050000	1.92E+08
P32119	P32119	Peroxiredoxin-2	PRDX2	sp|P32119	10	9	9	4	10	8	10	3	9	7	9	3	9	7	9	54.5	21.892	0	25.81	5.967E+09	5903800	4.66E+09	47322000	1.25E+09	11000000	3.85E+08	52771000	50561000
Q99497	Q99497	Protein deglycase DJ-1	PARK7	sp|Q99497	15	15	15	0	14	7	15	0	14	7	15	0	14	7	15	70.4	19.891	0	109.53	5.959E+09	0	1.85E+09	34473000	4.08E+09	0	1.65E+08	21546000	1.56E+08
Q14566	Q14566	DNA replication licensing factor MCM6	MCM6	sp|Q14566	30	30	30	1	26	11	28	1	26	11	28	1	26	11	28	40.7	92.888	0	278.84	5.937E+09	20214000	6.65E+08	50661000	5.2E+09	20632000	45062000	36619000	2.24E+08
P62899	P62899	60S ribosomal protein L31	RPL31	sp|P62899	7	7	7	3	5	7	6	3	5	7	6	3	5	7	6	47.2	14.463	0	32.999	5.922E+09	6.37E+08	1.48E+09	852030000	2.96E+09	4.07E+08	1.67E+08	725360000	1.57E+08
P17174	P17174	Aspartate aminotransferase, cytoplasmic	GOT1	sp|P17174	20	20	20	1	19	5	19	1	19	5	19	1	19	5	19	61.7	46.247	0	234.75	5.906E+09	1755900	3.23E+09	6438000	2.67E+09	1020300	2.8E+08	1305500	1.18E+08
Q12905	Q12905	Interleukin enhancer-binding factor 2	ILF2	sp|Q12905	15	15	15	8	12	13	15	8	12	13	15	8	12	13	15	55.4	43.062	0	176.46	5.884E+09	1.56E+08	2.16E+09	201500000	3.37E+09	1.66E+08	2.24E+08	119360000	1.1E+08
O14818;Q8	O14818	Proteasome subunit alpha type-7	PSMA7	sp|O14818	15	15	15	2	15	12	13	2	15	12	13	2	15	12	13	54.8	27.887	0	150.26	5.864E+09	2815600	2.04E+09	111910000	3.71E+09	3063200	1.73E+08	78953000	1.54E+08
Q15293	Q15293	Reticulocalbin-1	RCN1	sp|Q15293	21	21	21	1	14	19	21	1	14	19	21	1	14	19	21	74.6	38.89	0	229.65	5.85E+09	451150	1.22E+09	271810000	4.36E+09	580600	1.11E+08	162730000	1.75E+08
Q14247	Q14247	Src substrate cortactin	CTTN	sp|Q14247	27	27	27	1	24	17	27	1	24	17	27	1	24	17	27	53.3	61.585	0	171.41	5.813E+09	594910	7.44E+08	74831000	4.99E+09	2052800	28486000	26935000	2.63E+08
Q9UL46	Q9UL46	Proteasome activator complex subunit 2	PSME2	sp|Q9UL46	11	11	11	0	10	7	11	0	10	7	11	0	10	7	11	62.3	27.401	0	195.55	5.77E+09	0	6.27E+08	31700000	5.11E+09	0	76528000	36492000	1.73E+08
P62847	P62847	40S ribosomal protein S24	RPS24	sp|P62847	5	5	5	3	5	5	5	3	5	5	5	3	5	5	5	35.3	15.423	0	18.454	5.757E+09	2.75E+08	1.88E+09	286660000	3.31E+09	2.37E+08	1.62E+08	210280000	1.43E+08
P62841	P62841	40S ribosomal protein S15	RPS15	sp|P62841	9	9	9	4	7	8	9	4	7	8	9	4	7	8	9	70.3	17.04	0	64.172	5.716E+09	1.73E+09	1.15E+09	271750000	2.56E+09	1.23E+09	1.54E+08	410290000	1.4E+08
P41567	P41567	Eukaryotic translation initiation factor 1	EIF1	sp|P41567	7	7	2	2	7	2	6	2	7	2	6	1	2	0	2	65.5	12.732	0	92.215	5.709E+09	76376000	1.33E+09	4130800	4.3E+09	55198000	1.1E+08	3766000	2.02E+08
Q16531	Q16531	DNA damage-binding protein 1	DDB1	sp|Q16531	37	37	37	2	34	17	36	2	34	17	36	2	34	17	36	35.1	126.97	0	169.52	5.704E+09	8123800	1.46E+09	43584000	4.2E+09	8781100	1.39E+08	29291000	1.62E+08
Q07065	Q07065	Cytoskeleton-associated protein 4	CKAP4	sp|Q07065	35	35	35	9	32	29	34	9	32	29	34	9	32	29	34	66.3	66.022	0	250.24	5.679E+09	35038000	1.25E+09	518910000	3.88E+09	44851000	91671000	389300000	1.19E+08
P49755	P49755	Transmembrane emp24 domain-containing prote	TMED10	sp|P49755	7	7	7	1	7	5	7	1	7	5	7	1	7	5	7	33.3	24.976	0	96.698	5.648E+09	1687200	1.6E+09	23214000	4.03E+09	1022100	1.4E+08	7578900	1.75E+08
P00491	P00491	Purine nucleoside phosphorylase	PNP	sp|P00491	15	15	15	0	15	4	14	0	15	4	14	0	15	4	14	61.2	32.118	0	180.01	5.641E+09	0	1.99E+09	7836300	3.65E+09	0	1.71E+08	6498600	1.23E+08
P07910;A0	P07910;A0	Heterogeneous nuclear ribonucleoproteins C1/C2	HNRNPC;H	sp|P07910	12	12	12	10	11	12	12	10	11	12	12	10	11	12	12	38.9	33.67	0	47.462	5.595E+09	2.46E+09	1.39E+09	510680000	1.23E+09	1.86E+09	2.53E+08	769790000	88342000
O75643	O75643	U5 small nuclear ribonucleoprotein 200 kDa helic	SNRNP200	sp|O75643	55	55	55	8	52	26	45	8	52	26	45	8	52	26	45	34.1	244.5	0	323.31	5.588E+09	2.19E+08	2.16E+09	103410000	3.1E+09	2.18E+08	1.64E+08	119770000	1.01E+08
P27816	P27816	Microtubule-associated protein 4	MAP4	sp|P27816	42	42	42	2	30	26	41	2	30	26	41	2	30	26	41	45.9	121	0	255.44	5.575E+09	1654400	7.33E+08	101140000	4.74E+09	2811000	82211000	39263000	2.05E+08
P61981	P61981	14-3-3 protein gamma;14-3-3 protein gamma, N-t	YWHAG	sp|P61981	17	13	12	4	14	8	17	3	10	5	13	2	9	4	12	72.1	28.302	0	215.72	5.555E+09	4704700	1.59E+09	34117000	3.92E+09	4549200	1.34E+08	35327000	1.54E+08
O00560	O00560	Syntenin-1	SDCBP	sp|O00560	12	12	12	1	7	9	11	1	7	9	11	1	7	9	11	56.4	32.444	0	254.52	5.529E+09	615550	2.37E+08	425350000	4.87E+09	641630	8646000	292670000	1.71E+08
P05556	P05556	Integrin beta-1	ITGB1	sp|P05556	20	20	20	1	9	7	20	1	9	7	20	1	9	7	20	31.8	88.414	0	133.29	5.518E+09	1906000	1.09E+08	24263000	5.38E+09	2117300	8202200	14582000	2.23E+08
P62917	P62917	60S ribosomal protein L8	RPL8	sp|P62917	14	14	14	6	11	11	12	6	11	11	12	6	11	11	12	54.5	28.024	0	58.028	5.478E+09	3.05E+08	1.66E+09	694260000	2.81E+09	4.72E+08	90942000	383350000	81226000
Q9UQ80	Q9UQ80	Proliferation-associated protein 2G4	PA2G4	sp|Q9UQ80	16	16	16	4	16	7	14	4	16	7	14	4	16	7	14	43.7	43.786	0	82.698	5.475E+09	11599000	2.54E+09	19132000	2.9E+09	13957000	2.14E+08	17259000	1.22E+08
P04792	P04792	Heat shock protein beta-1	HSPB1	sp|P04792	15	15	15	1	9	14	15	1	9	14	15	1	9	14	15	83.4	22.782	0	173.56	5.474E+09	987390	1.37E+08	465660000	4.87E+09	2523200	6530700	413780000	92464000
Q15691	Q15691	Microtubule-associated protein RP/EB family mer	MAPRE1	sp|Q15691	16	16	15	3	14	5	16	3	14	5	16	3	13	5	15	71.6	29.999	0	111.12	5.456E+09	1352500	1.92E+09	10571000	3.52E+09	1124700	1.63E+08	4607600	1.31E+08
Q96AE4	Q96AE4	Far upstream element-binding protein 1	FUBP1	sp|Q96AE4	30	30	26	4	29	2	24	4	29	2	24	3	25	2	21	56.8	67.56	0	138.12	5.44E+09	5099600	3.79E+09	4771600	1.64E+09	2484300	3.15E+08	1191100	79069000
Q16719	Q16719	Kynureninase	KYNU	sp|Q16719	16	16	16	0	0	2	16	0	0	2	16	0	0	2	16	44.7	52.351	0	136.46	5.437E+09	0	0	5155600	5.43E+09	0	0	3613300	2.18E+08
P78344	P78344	Eukaryotic translation initiation factor 4 gamma 2	EIF4G2	sp|P78344	35	35	35	7	29	15	34	7	29	15	34	7	29	15	34	46.3	102.36	0	201.14	5.43E+09	48297000	1.11E+09	39957000	4.23E+09	51258000	91401000	46797000	1.51E+08
P49915	P49915	GMP synthase [glutamine-hydrolyzing]	GMPS	sp|P49915	28	28	28	1	23	4	26	1	23	4	26	1	23	4	26	55.1	76.715	0	223.45	5.426E+09	14806000	1.27E+09	4072900	4.13E+09	10871000	1.55E+08	1011700	1.18E+08
Q9HB71	Q9HB71	Calcyclin-binding protein	CACYBP	sp|Q9HB71	13	13	13	0	13	6	12	0	13	6	12	0	13	6	12	56.6	26.21	0	117.19	5.409E+09	0	1.88E+09	10106000	3.52E+09	0	1.62E+08	14526000	1.38E+08
Q6P2Q9	Q6P2Q9	Pre-mRNA-processing-splicing factor 8	PRPF8	sp|Q6P2Q9	61	61	61	8	58	45	55	8	58	45	55	8	58	45	55	33.4	273.6	0	323.31	5.404E+09	8788200	2.04E+09	231610000	3.12E+09	6080600	2.07E+08	153010000	1.05E+08
P22695	P22695	Cytochrome b-c1 complex subunit 2, mitochondri	UQCRC2	sp|P22695	18	18	18	3	17	14	16	3	17	14	16	3	17	14	16	53.6	48.442	0	156.6	5.404E+09	51616000	1.63E+09	103000000	3.62E+09	39813000	1.19E+08	101510000	1.31E+08
Q9NTK5	Q9NTK5	Obg-like ATPase 1	OLA1	sp|Q9NTK5	19	19	19	0	17	7	18	0	17	7	18	0	17	7	18	51.8	44.743	0	68.65	5.392E+09	0	1.15E+09	14932000	4.23E+09	0	1.33E+08	15036000	1.38E+08
P51149	P51149	Ras-related protein Rab-7a	RAB7A	sp|P51149	12	12	12	4	11	5	12	4	11	5	12	4	11	5	12	59.4	23.489	0	103.25	5.376E+09	10364000	9.13E+08	13784000	4.44E+09	8443500	72052000	10273000	1.92E+08
P38606	P38606	V-type proton ATPase catalytic subunit A	ATP6V1A	sp|P38606	29	29	29	1	22	11	28	1	22	11	28	1	22	11	28	63	68.303	0	268.51	5.357E+09	301590	7.75E+08	29964000	4.55E+09	1054300	28744000	9991600	2.33E+08
P50502;Q8	P50502;Q8	Hsc70-interacting protein;Putative protein FAM1(	ST13;ST13I	sp|P50502	10	10	10	2	10	2	10	2	10	2	10	2	10	2	10	36.9	41.331	0	30.863	5.354E+09	427570	2.87E+09	3174600	2.48E+09	326620	2.73E+08	896470	82681000
Q96QK1	Q96QK1	Vacuolar protein sorting-associated protein 35	VPS35	sp|Q96QK1	27	27	27	0	21	5	26	0	21	5	26	0	21	5	26	36.7	91.706	0	206.05	5.34E+09	0	1.15E+09	17603000	4.18E+09	0	1.03E+08	14289000	1.63E+08
O76003	O76003	Glutaredoxin-3	GLRX3	sp|O76003	17	17	17	2	14	2	16	2	14	2	16	2	14	2	16	58.5	37.432	0	233.57	5.337E+09	37847000	1.8E+09	9459900	3.49E+09	39624000	1.51E+08	5720100	1.39E+08
P16615;O1	P16615	Sarcoplasmic/endoplasmic reticulum calcium ATP	ATP2A2	sp|P16615	23	23	23	6	21	15	23	6	21	15	23	6	21	15	23	27	114.76	0	178.92	5.335E+09	9269700	1.14E+09	100040000	4.09E+09	7265900	78248000	122680000	1.37E+08
P20290	P20290	Transcription factor BTF3	BTF3	sp|P20290	10	10	9	1	10	6	10	1	10	6	10	1	9	6	9	63.1	22.168	0	236.27	5.331E+09	2685900	1.48E+09	216030000	3.63E+09	13424000	1.94E+08	115680000	1.02E+08
P52907	P52907	F-actin-capping protein subunit alpha-1	CAPZA1	sp|P52907	12	12	9	1	11	11	12	1	11	11	12	0	8	8	9	61.9	32.922	0	275.13	5.323E+09	4586300	9.45E+08	289300000	4.08E+09	4883000	83234000	192420000	1.62E+08
Q99878;Q9	Q99878;Q9	Histone H2A type 1-J;Histone H2A type 1-H;Histor	HIST1H2AJ	sp|Q99878	4	4	2	2	3	4	3	2	3	4	3	1	1	2	1	35.9	13.936	0	17.319	5.304E+09	1.43E+09	1.31E+09	347600000	2.22E+09	1.29E+09	1.34E+08	327030000	1.19E+08
E9PAV3;Q9	E9PAV3	Nascent polypeptide-associated complex subunit	NACA	sp|E9PAV3	5	5	5	2	5	4	5	2	5	4	5	2	5	4	5	4.3	205.42	0	101.28	5.279E+09	7087500	1.85E+09	245670000	3.17E+09	9823900	1.43E+08	189210000	1.23E+08
P11413	P11413	Glucose-6-phosphate 1-dehydrogenase	G6PD	sp|P11413	23	23	23	1	18	4	23	1	18	4	23	1	18	4	23	51.5	59.256	0	82.824	5.248E+09	1647600	7.42E+08	6956900	4.5E+09	1237300	62360000	1832000	1.9E+08

P61158;Q9	P61158	Actin-related protein 3	ACTR3	sp|P61158	17	17	17	1	13	7	16	1	13	7	16	1	13	7	16	52.2	47.371	0	139.92	5.246E+09	682040	9.26E+08	31026000	4.29E+09	797140	83355000	8823600	1.87E+08
P78417	P78417	Glutathione S-transferase omega-1	GSTO1	sp|P78417	17	17	17	1	17	0	17	1	17	0	17	1	17	0	17	57.3	27.566	0	83.524	5.237E+09	686120	1.05E+09	0	4.19E+09	779180	92588000	0	1.72E+08
P62263	P62263	40S ribosomal protein S14	RPS14	sp|P62263	8	8	8	4	8	6	8	4	8	6	8	4	8	6	8	39.7	16.273	0	74.849	5.188E+09	1.1E+08	1.9E+09	291390000	2.88E+09	1.41E+08	1.29E+08	210450000	1.03E+08
Q9BSJ8	Q9BSJ8	Extended synaptotagmin-1	ESYT1	sp|Q9BSJ8	32	32	32	1	25	6	32	1	25	6	32	1	25	6	32	42.1	122.85	0	260.48	5.149E+09	3159300	9.4E+08	20606000	4.19E+09	10001000	1.09E+08	13173000	1.4E+08
P61353	P61353	60S ribosomal protein L27	RPL27	sp|P61353	7	7	7	6	5	7	5	6	5	7	5	6	5	7	5	45.6	15.798	0	40.179	5.148E+09	1.73E+08	1.19E+09	879080000	2.91E+09	1.39E+08	1.08E+08	589960000	1.44E+08
Q16543	Q16543	Hsp90 co-chaperone Cdc37;Hsp90 co-chaperone (	CDC37	sp|Q16543	11	11	11	0	10	5	11	0	10	5	11	0	10	5	11	36.2	44.468	0	228.12	5.135E+09	0	1.63E+09	18174000	3.49E+09	0	1.39E+08	22249000	1.35E+08
P28070	P28070	Proteasome subunit beta type-4	PSMB4	sp|P28070	7	7	7	3	7	7	7	3	7	7	7	3	7	7	7	45.5	29.204	0	101.18	5.123E+09	8189500	1.95E+09	95226000	3.07E+09	18124000	1.86E+08	46178000	1.02E+08
P39019	P39019	40S ribosomal protein S19	RPS19	sp|P39019	13	13	13	4	12	12	12	4	12	12	12	4	12	12	12	57.9	16.06	0	66.607	5.118E+09	2.27E+08	1.79E+09	416350000	2.69E+09	2.58E+08	1.52E+08	252310000	1.13E+08
P62750	P62750	60S ribosomal protein L23a	RPL23A	sp|P62750	13	13	13	8	11	13	11	8	11	13	11	8	11	13	11	56.4	17.695	0	92.112	5.112E+09	8.03E+08	9.68E+08	663460000	2.68E+09	5.02E+08	2.03E+08	581130000	1.55E+08
P62913	P62913	60S ribosomal protein L11	RPL11	sp|P62913	8	8	8	4	7	7	8	4	7	7	8	4	7	7	8	42.7	20.252	0	49.642	5.103E+09	97667000	1.41E+09	671690000	2.93E+09	96526000	1.34E+08	426800000	1.31E+08
Q13813	Q13813	Spectrin alpha chain, non-erythrocytic 1	SPTAN1	sp|Q13813	88	88	88	5	76	79	65	5	76	79	65	5	76	79	65	47.3	284.54	0	323.31	5.045E+09	5339200	2.41E+09	886550000	1.74E+09	3274500	1.86E+08	636770000	56545000
Q8NC51	Q8NC51	Plasminogen activator inhibitor 1 RNA-binding pr	SERBP1	sp|Q8NC5	22	22	22	6	21	14	20	6	21	14	20	6	21	14	20	56.9	44.965	0	97.274	5.04E+09	1.47E+08	2.68E+09	190100000	2.02E+09	2.77E+08	1.71E+08	79192000	60903000
P00505	P00505	Aspartate aminotransferase, mitochondrial	GOT2	sp|P00505	21	21	21	0	21	6	17	0	21	6	17	0	21	6	17	49.3	47.517	0	93.548	5.033E+09	0	2.66E+09	7309300	2.36E+09	0	2.42E+08	6445800	81162000
P00492	P00492	Hypoxanthine-guanine phosphoribosyltransferase	HPRT1	sp|P00492	9	9	9	0	9	4	9	0	9	4	9	0	9	4	9	50	24.579	0	32.982	5.031E+09	0	3.21E+09	7210300	1.81E+09	0	2.86E+08	7150400	69155000
Q9UNM6	Q9UNM6	26S proteasome non-ATPase regulatory subunit 1	PSMD13	sp|Q9UNM	16	16	16	3	14	10	16	3	14	10	16	3	14	10	16	52.7	42.945	0	160.2	5.02E+09	76025000	1.49E+09	35468000	3.41E+09	60046000	1.26E+08	34463000	1.47E+08
P14314	P14314	Glucosidase 2 subunit beta	PRKCSH	sp|P14314	19	19	19	2	19	3	18	2	19	3	18	2	19	3	18	33.1	59.425	0	87.444	5.008E+09	16654000	1.45E+09	5167200	3.53E+09	3463300	1.04E+08	2548700	1.84E+08
P13804	P13804	Electron transfer flavoprotein subunit alpha, mitc	ETFA	sp|P13804	9	9	9	1	9	1	9	1	9	1	9	1	9	1	9	41.1	35.079	0	68.86	4.986E+09	27151000	2.21E+09	387250	2.75E+09	9517200	2.02E+08	454750	1.22E+08
P17066;P4	P17066	Heat shock 70 kDa protein 6	HSPA6	sp|P17066	10	1	1	3	10	10	10	1	1	1	1	1	1	1	1	14.5	71.027	0	4.0122	4.967E+09	69508000	1.76E+09	261450000	2.87E+09	69508000	1.55E+08	175690000	1.18E+08
P17844	P17844	Probable ATP-dependent RNA helicase DDX5	DDX5	sp|P17844	30	29	22	13	26	28	28	12	25	27	27	7	18	20	20	50	69.147	0	113.28	4.942E+09	2.36E+08	1.24E+09	588800000	2.87E+09	1.17E+08	1.24E+08	482540000	1.22E+08
Q7L2H7	Q7L2H7	Eukaryotic translation initiation factor 3 subunit M	EIF3M	sp|Q7L2H7	13	13	13	5	10	8	12	5	10	8	12	5	10	8	12	42	42.502	0	122.66	4.94E+09	1.8E+09	9.64E+08	53260000	2.13E+09	1.73E+09	85906000	47224000	87395000
Q12931	Q12931	Heat shock protein 75 kDa, mitochondrial	TRAP1	sp|Q12931	26	26	26	3	25	2	22	3	25	2	22	3	25	2	22	40.2	80.109	0	136.53	4.923E+09	6500700	3.5E+09	12788000	1.4E+09	3401300	3.38E+08	2096300	37541000
P60709;Q5	P60709	Actin, cytoplasmic 1;Actin, cytoplasmic 1, N-termi	ACTB	sp|P60709	26	1	1	13	23	26	25	0	1	1	1	0	1	1	1	89.6	41.736	0	50.607	4.922E+09	0	1.46E+09	148970000	3.31E+09	0	77779000	48487000	81244000
P09104	P09104	Gamma-enolase	ENO2	sp|P09104	13	11	11	1	12	3	13	0	10	1	11	0	10	1	11	55.3	47.268	0	264.32	4.918E+09	0	6.62E+08	4780400	4.25E+09	0	1.07E+08	17436000	1.05E+08
P48047	P48047	ATP synthase subunit O, mitochondrial	ATP5O	sp|P48047	10	10	10	5	10	6	10	5	10	6	10	5	10	6	10	62	23.277	0	76.721	4.917E+09	14594000	1.92E+09	29862000	2.95E+09	28412000	1.72E+08	22389000	77369000
P46782	P46782	40S ribosomal protein S5;40S ribosomal protein S	RPS5	sp|P46782	13	13	13	6	11	11	11	6	11	11	11	6	11	11	11	49	22.876	0	64.061	4.905E+09	1.64E+08	1.73E+09	251280000	2.76E+09	2.33E+08	1.06E+08	166170000	82778000
P18621	P18621	60S ribosomal protein L17	RPL17	sp|P18621	10	10	10	6	9	8	9	6	9	8	9	6	9	8	9	51.6	21.397	0	48.619	4.905E+09	3.31E+08	1.59E+09	473880000	2.51E+09	3.51E+08	1.12E+08	332090000	86073000
P20618	P20618	Proteasome subunit beta type-1	PSMB1	sp|P20618	8	8	8	0	7	5	7	0	7	5	7	0	7	5	7	44	26.489	0	104.72	4.893E+09	0	1.88E+09	65178000	2.94E+09	0	1.53E+08	55557000	1.23E+08
O75821	O75821	Eukaryotic translation initiation factor 3 subunit G	EIF3G	sp|O75821	14	14	14	7	12	9	13	7	12	9	13	7	12	9	13	50	35.611	0	100.01	4.885E+09	6.53E+08	9.78E+08	107300000	3.15E+09	6.15E+08	1.19E+08	100070000	1.06E+08
P52788	P52788	Spermine synthase	SMS	sp|P52788	14	14	14	0	12	2	14	0	12	2	14	0	12	2	14	57.4	41.268	0	274.4	4.883E+09	0	6E+08	3161500	4.28E+09	0	54969000	1290800	1.75E+08
P47897	P47897	Glutamine--tRNA ligase	QARS	sp|P47897	33	33	33	7	24	20	33	7	24	20	33	7	24	20	33	50.2	87.798	0	184.51	4.875E+09	14503000	9.3E+08	140670000	3.79E+09	12361000	71442000	127230000	1.3E+08
P26640	P26640	Valine--tRNA ligase	VARS	sp|P26640	37	37	37	5	34	15	37	5	34	15	37	5	34	15	37	41.3	140.47	0	204.03	4.847E+09	17111000	1.52E+09	28348000	3.29E+09	30635000	1.32E+08	18810000	1.23E+08
Q13838	Q13838	Spliceosome RNA helicase DDX39B	DDX39B	sp|Q13838	18	18	9	5	18	5	12	5	18	5	12	0	9	0	4	49.8	48.991	0	93.572	4.81E+09	54186000	2.79E+09	19659000	1.95E+09	87439000	2.07E+08	10189000	85080000
P07339	P07339	Cathepsin D;Cathepsin D light chain;Cathepsin D h	CTSD	sp|P07339	13	13	13	1	10	12	11	1	10	12	11	1	10	12	11	37.6	44.552	0	75.872	4.752E+09	10399000	4.97E+08	262090000	3.98E+09	14157000	34702000	196960000	1.46E+08
Q12797	Q12797	Aspartyl/asparaginyl beta-hydroxylase	ASPH	sp|Q12797	24	24	24	0	12	22	22	0	12	22	22	0	12	22	22	41.7	85.862	0	323.31	4.745E+09	0	2.41E+08	612780000	3.89E+09	0	30318000	429340000	1.32E+08
Q9NZM1;C	Q9NZM1	Myoferlin	MYOF	sp|Q9NZM	56	56	56	2	2	17	56	2	2	17	56	2	2	17	56	34	234.71	0	307.21	4.735E+09	3516100	9894400	50995000	4.67E+09	2252300	1121800	8567100	2.19E+08
Q9NTJ5	Q9NTJ5	Phosphatidylinositide phosphatase SAC1	SACM1L	sp|Q9NTJ5	10	10	10	1	6	1	9	1	6	1	9	1	6	1	9	24.5	66.966	0	131.35	4.729E+09	4.29E+09	72101000	11150000	3.52E+08	3.85E+09	1.46E+08	264020000	61306000
Q99873;Q9	Q99873	Protein arginine N-methyltransferase 1	PRMT1	sp|Q99873	21	21	21	13	15	18	12	13	15	18	12	13	15	18	12	55.5	42.461	0	75.704	4.722E+09	1.28E+09	8.03E+08	1.268E+09	1.37E+09	1.09E+09	73180000	941220000	72364000
Q02952	Q02952	A-kinase anchor protein 12	AKAP12	sp|Q02952	47	47	47	1	31	20	47	1	31	20	47	1	31	20	47	44.8	191.48	0	323.31	4.704E+09	227480	4.67E+08	62280000	4.17E+09	425580	45132000	48809000	1.59E+08
Q02790	Q02790	Peptidyl-prolyl cis-trans isomerase FKBP4;Peptidy	FKBP4	sp|Q02790	29	29	29	0	28	1	24	0	28	1	24	0	28	1	24	67.1	51.804	0	190.69	4.696E+09	0	2.86E+09	717840	1.83E+09	0	3.04E+08	1285200	16873000
P46776	P46776	60S ribosomal protein L27a	RPL27A	sp|P46776	7	7	7	2	6	7	7	2	6	7	7	2	6	7	7	41.2	16.561	0	23.078	4.693E+09	4672900	1.46E+09	448670000	2.78E+09	3136500	1.71E+08	278930000	96716000
Q9ULV4	Q9ULV4	Coronin-1C	CORO1C	sp|Q9ULV4	19	19	19	0	14	18	15	0	14	18	15	0	14	18	15	38	53.248	0	160.21	4.689E+09	0	6.75E+08	921950000	3.09E+09	0	55144000	635010000	99682000
P24752	P24752	Acetyl-CoA acetyltransferase, mitochondrial	ACAT1	sp|P24752	20	20	20	5	20	7	17	5	20	7	17	5	20	7	17	60	45.199	0	149.54	4.656E+09	3171600	3.25E+09	8991500	1.39E+09	5252400	2.81E+08	7722300	42981000
Q13435	Q13435	Splicing factor 3B subunit 2	SF3B2	sp|Q13435	30	30	30	6	27	21	29	6	27	21	29	6	27	21	29	44	100.23	0	197.02	4.647E+09	2.44E+08	1.44E+09	195620000	2.76E+09	91648000	1.47E+08	204240000	1.64E+08
P83731	P83731	60S ribosomal protein L24	RPL24	sp|P83731	8	8	8	3	8	6	6	3	8	6	6	3	8	6	6	41.4	17.779	0	20.258	4.644E+09	3.55E+08	1.76E+09	476380000	2.05E+09	2.7E+08	1.89E+08	351650000	68187000
P49321	P49321	Nuclear autoantigenic sperm protein	NASP	sp|P49321	24	24	24	3	24	4	23	3	24	4	23	3	24	4	23	56.7	85.237	0	272.92	4.607E+09	4750500	1.49E+09	21071000	3.09E+09	2044200	1.36E+08	6652900	1.31E+08
Q15056	Q15056	Eukaryotic translation initiation factor 4H	EIF4H	sp|Q15056	9	9	9	1	9	4	9	1	9	4	9	1	9	4	9	41.9	27.385	0	61.463	4.547E+09	651350	2.04E+09	10987000	2.49E+09	853760	2.01E+08	3765500	84043000
P27635;Q9	P27635	60S ribosomal protein L10	RPL10	sp|P27635	9	9	9	5	9	7	9	5	9	7	9	5	9	7	9	39.3	24.604	0	100.37	4.509E+09	1.47E+08	1.42E+09	366240000	2.57E+09	2.05E+08	1.08E+08	228900000	77103000
Q00325	Q00325	Phosphate carrier protein, mitochondrial	SLC25A3	sp|Q00325	15	15	15	2	14	12	13	2	14	12	13	2	14	12	13	34	40.094	0	102.74	4.509E+09	7362800	1.33E+09	127490000	3.05E+09	8200000	1.07E+08	80300000	1.25E+08
Q9NVI7	Q9NVI7	ATPase family AAA domain-containing protein 3A	ATAD3A	sp|Q9NVI7	29	29	8	2	19	23	28	2	19	23	28	0	5	7	8	45	71.368	0	169.47	4.493E+09	7037900	6.98E+08	545400000	3.24E+09	7255000	54509000	359460000	1.29E+08
P62854;Q5	P62854;Q5	40S ribosomal protein S26;Putative 40S ribosoma	RPS26;RPS	sp|P62854	3	3	3	1	3	3	3	1	3	3	3	1	3	3	3	27	13.015	0	10.299	4.491E+09	9379300	2.26E+09	320110000	1.9E+09	20590000	1.91E+08	203420000	84847000
Q13347	Q13347	Eukaryotic translation initiation factor 3 subunit I	EIF3I	sp|Q13347	17	17	17	13	16	12	16	13	16	12	16	13	16	12	16	68.9	36.501	0	168.72	4.481E+09	4.7E+08	8.78E+08	89186000	3.04E+09	3.98E+08	1.01E+08	85655000	1.41E+08
Q15008	Q15008	26S proteasome non-ATPase regulatory subunit 6	PSMD6	sp|Q15008	23	23	23	3	22	11	23	3	22	11	23	3	22	11	23	55.5	45.531	0	109.56	4.478E+09	5648500	1.06E+09	23627000	3.39E+09	3299200	92304000	19196000	1.39E+08
P00387	P00387	NADH-cytochrome b5 reductase 3;NADH-cytochro	CYB5R3	sp|P00387	12	12	12	1	11	11	12	1	11	11	12	1	11	11	12	45.5	34.234	0	202.93	4.473E+09	71942000	1.08E+09	65141000	3.26E+09	62105000	95473000	57046000	1.3E+08
P49591	P49591	Serine--tRNA ligase, cytoplasmic	SARS	sp|P49591	17	17	17	0	16	3	14	0	16	3	14	0	16	3	14	37.9	58.777	0	165.49	4.458E+09	0	2.17E+09	4842000	2.28E+09	0	1.52E+08	2108300	1.32E+08
Q92945	Q92945	Far upstream element-binding protein 2	KHSRP	sp|Q92945	27	24	24	4	26	5	22	4	23	5	20	4	23	5	20	55.1	73.114	0	125	4.442E+09	5657100	2.37E+09	7358700	2.06E+09	2740800	1.83E+08	2740300	1.11E+08
P50914	P50914	60S ribosomal protein L14	RPL14	sp|P50914	5	5	5	2	4	4	5	2	4	4	5	2	4	4	5	24.7	23.432	0	20.666	4.442E+09	1.2E+09	1.01E+09	353310000	1.87E+09	1.3E+09	64157000	191250000	49359000
Q14203	Q14203	Dynactin subunit 1	DCTN1	sp|Q14203	37	37	37	5	31	14	35	5	31	14	35	5	31	14	35	42.1	141.69	0	323.31	4.44E+09	95841000	1.5E+09	39697000	2.8E+09	90846000	1.19E+08	55180000	1.04E+08
P40429;Q6	P40429	60S ribosomal protein L13a	RPL13A	sp|P40429	12	12	12	5	10	11	12	5	10	11	12	5	10	11	12	46.3	23.577	0	26.318	4.411E+09	1.8E+08	1.31E+09	348560000	2.57E+09	1.28E+08	1.01E+08	268800000	1.3E+08
P39748	P39748	Flap endonuclease 1	FEN1	sp|P39748	11	11	11	0	10	2	10	0	10	2	10	0	10	2	10	40	42.592	0	156.57	4.392E+09	0	1.52E+09	4871200	2.87E+09	0	1.27E+08	7483200	1.18E+08
Q14839	Q14839	Chromodomain-helicase-DNA-binding protein 4	CHD4	sp|Q14839	41	41	31	0	39	16	34	0	39	16	34	0	29	10	25	32.4	218	0	292.59	4.387E+09	0	1.52E+09	61852000	2.8E+09	0	1.26E+08	71754000	92444000
P23381	P23381	Tryptophan--tRNA ligase, cytoplasmic;T1-TrpRS;T	WARS	sp|P23381	19	19	19	0	18	2	19	0	18	2	19	0	18	2	19	52.2	53.165	0	223.43	4.377E+09	0	1.64E+09	2497100	2.73E+09	0	1.49E+08	1856600	1.04E+08
P07384	P07384	Calpain-1 catalytic subunit	CAPN1	sp|P07384	20	20	20	0	12	8	19	0	12	8	19	0	12	8	19	29.8	81.889	0	82.953	4.366E+09	0	8.87E+08	34207000	3.45E+09	0	57544000	64911000	1.2E+08
P51148	P51148	Ras-related protein Rab-5C	RAB5C	sp|P51148	11	11	8	1	11	3	9	1	11	3	9	1	8	2	7	65.7	23.482	0	129.47	4.365E+09	1689900	1.46E+09	13211000	2.89E+09	1414100	1.05E+08	5176500	1.45E+08
P32969	P32969	60S ribosomal protein L9	RPL9	sp|P32969	8	8	8	4	6	8	8	4	6	8	8	4	6	8	8	61.5	21.863	0	83.403	4.339E+09	1.04E+09	7.41E+08	342480000	2.22E+09	1.12E+09	52559000	168910000	80527000
P56192	P56192	Methionine--tRNA ligase, cytoplasmic	MARS	sp|P56192	22	22	22	4	21	12	21	4	21	12	21	4	21	12	21	33.1	101.11	0	178.11	4.332E+09	38014000	2.14E+09	56881000	2.1E+09	20156000	1.82E+08	40379000	1.02E+08
O15144	O15144	Actin-related protein 2/3 complex subunit 2	ARPC2	sp|O15144	16	16	16	2	11	5	16	2	11	5	16	2	11	5	16	65	34.333	0	148.6	4.326E+09	1194200	2.97E+08	8468000	4.02E+09	944360	21815000	3401800	1.62E+08
Q16881	Q16881	Thioredoxin reductase 1, cytoplasmic	TXNRD1	sp|Q16881	18	18	18	1	15	4	18	1	15	4	18	1	15	4	18	42.8	70.905	0	228.91	4.306E+09	44293	7.13E+08	10646000	3.58E+09	204520	14505000	2234400	2E+08
P22061	P22061	Protein-L-isoaspartate(D-aspartate) O-methyltran	PCMT1	sp|P22061	10	10	10	6	9	10	8	6	9	10	8	6	9	10	8	55.5	24.636	0	80.899	4.282E+09	1.78E+09	1.34E+09	167160000	9.93E+08	1.59E+09	1.69E+08	232310000	59997000
P31930	P31930	Cytochrome b-c1 complex subunit 1, mitochondri	UQCRC1	sp|P31930	15	15	15	2	15	2	13	2	15	2	13	2	15	2	13	40.8	52.645	0	168.95	4.279E+09	410160	1.37E+09	8004400	2.9E+09	259620	1.29E+08	1704200	1.15E+08
Q92841	Q92841	Probable ATP-dependent RNA helicase DDX17	DDX17	sp|Q92841	27	19	19	14	27	23	26	8	19	15	18	8	19	15	18	41.7	80.272	0	82.952	4.257E+09	3.22E+08	2E+09	280370000	1.66E+09	2.21E+08	1.62E+08	250940000	66826000
O15372	O15372	Eukaryotic translation initiation factor 3 subunit H	EIF3H	sp|O15372	15	15	15	8	11	8	13	8	11	8	13	8	11	8	13	47.4	39.93	0	135.94	4.246E+09	4.86E+08	6.57E+08	72665000	3.03E+09	4.83E+08	69702000	74007000	90810000
P61604	P61604	10 kDa heat shock protein, mitochondrial	HSPE1	sp|P61604	9	9	9	2	8	4	8	2	8	4	8	2	8	4	8	70.6	10.932	0	22.388	4.234E+09	6124200	2.18E+09	7470400	2.04E+09	5214600	1.95E+08	5659100	80548000
P46778	P46778	60S ribosomal protein L21	RPL21	sp|P46778	9	9	9	3	8	7	9	3	8	7	9	3	8	7	9	43.1	18.565	0	35.742	4.223E+09	69365000	1.24E+09	375580000	2.54E+09	96484000	63529000	256570000	68792000
Q9Y678	Q9Y678	Coatomer subunit gamma-1	COPG1	sp|Q9Y678	21	21	20	1	14	11	21	1	14	11	21	1	13	10	20	34.4	97.717	0	192.31	4.188E+09	1494100	6.86E+08	35455000	3.46E+09	2399800	67533000	20513000	1.37E+08
P51858	P51858	Hepatoma-derived growth factor	HDGF	sp|P51858	14	14	13	0	14	9	13	0	14	9	13	0	13	9	12	64.6	26.788	0	161.26	4.186E+09	0	1.55E+09	57410000	2.58E+09	0	1.51E+08	28156000	1.02E+08
P00403	P00403	Cytochrome c oxidase subunit 2	MT-CO2	sp|P00403	6	6	6	1	6	4	6	1	6	4	6	1	6	4	6	32.2	25.565	0	19.238	4.155E+09	1158900	1.28E+09	16929000	2.86E+09	4225000	1.2E+08	17187000	1.01E+08
P78559	P78559	Microtubule-associated protein 1A;MAP1A heavy	MAP1A	sp|P78559	62	61	61	0	4	43	59	0	3	42	58	0	3	42	58	35.1	305.48	0	323.31	4.141E+09	0	8576400	245760000	3.89E+09	0	3560800	57313000	2.64E+08
P62857	P62857	40S ribosomal protein S28	RPS28	sp|P62857	3	3	3	2	2	2	3	2	2	2	3	2	2	2	3	46.4	7.8409	0	26.736	4.091E+09	4.32E+08	1.53E+09	243990000	1.88E+09	4.16E+08	1.46E+08	165380000	82007000
P10599	P10599	Thioredoxin	TXN	sp|P10599	6	6	6	3	6	5	6	3	6	5	6	3	6	5	6	51.4	11.737	0	17.85	4.082E+09	8.05E+08	1.78E+09	76449000	1.42E+09	2.18E+08	4.89E+08	182550000	1.82E+08
Q27J81	Q27J81	Inverted formin-2	INF2	sp|Q27J81	23	23	23	0	11	18	23	0	11	18	23	0	11	18	23	32	135.62	0	323.31	4.077E+09	0	1.12E+08	129120000	3.84E+09	0	16394000	141500000	96494000
Q6NZI2	Q6NZI2	Polymerase I and transcript release factor	PTRF	sp|Q6NZI2	15	15	15	0	3	14	14	0	3	14	14	0	3	14	14	45.1	43.476	0	145.44	4.066E+09	0	9551100	509640000	3.55E+09	0	828620	338430000	1.23E+08
Q9UHB9	Q9UHB9	Signal recognition particle subunit SRP68	SRP68	sp|Q9UHB	24	24	24	3	21	9	21	3	21	9	21	3	21	9	21	42.4	70.729	0	131.16	4.058E+09	10738000	1.27E+09	33609000	2.75E+09	14007000	1.11E+08	29525000	1.03E+08
P36871	P36871	Phosphoglucomutase-1	PGM1	sp|P36871	29	29	29	1	25	3	26	1	25	3	26	1	25	3	26	66.9	61.448	0	111.64	4.057E+09	4550000	1.07E+09	7203900	2.97E+09	0	94607000	4816600	1.16E+08
P41091;Q2	P41091;Q2	Eukaryotic translation initiation factor 2 subunit 3	EIF2S3;EIF:	sp|P41091	16	16	16	9	13	13	15	9	13	13	15	9	13	13	15	48.5	51.109	0	77.24	4.057E+09	4.81E+08	6.22E+08	102900000	2.85E+09	3.87E+08	1.09E+08	94064000	1.3E+08
O15371	O15371	Eukaryotic translation initiation factor 3 subunit D	EIF3D	sp|O15371	18	18	18	10	12	12	16	10	12	12	16	10	12	12	16	44.2	63.972	0	150.98	4.05E+09	7.08E+08	1.05E+09	140370000	2.16E+09	6.51E+08	96266000	97611000	1.12E+08
Q16658	Q16658	Fascin	FSCN1	sp|Q16658	23	23	23	0	17	4	23	0	17	4	23	0	17	4	23	60.2	54.529	0	246.74	4.039E+09	0	3.21E+08	12510000	3.71E+09	0	27763000	12180000	1.49E+08
Q9Y3F4	Q9Y3F4	Serine-threonine kinase receptor-associated prot	STRAP	sp|Q9Y3F4	18	18	18	4	17	10	18	4	17	10	18	4	17	10	18	62.9	38.438	0	106.86	4.026E+09	54434000	1.18E+09	70831000	2.72E+09	82837000	93920000	39458000	98728000
P11177	P11177	Pyruvate dehydrogenase E1 component subunit b	PDHB	sp|P11177	11	11	11	2	11	3	11	2	11	3	11	2	11	3	11	39.3	39.233	0	46.391	4.025E+09	8778300	2.35E+09	4091000	1.66E+09	3990400	2.04E+08	2081400	76582000
P55084	P55084	Trifunctional enzyme subunit beta, mitochondrial	HADHB	sp|P55084	21	21	21	4	17	11	20	4	17	11	20	4	17	11	20	57.2	51.294	0	205.76	4.022E+09	19739000	1.07E+09	35113000	2.89E+09	21203000	1.1E+08	18187000	1.08E+08

P33316	P33316	Deoxyuridine 5-triphosphate nucleotidohydrolase	DUT	sp|P33316	8	8	8	0	8	3	8	0	8	3	8	0	8	3	8	38.1	26.563	0	58.75	4.022E+09	0	1.85E+09	13485000	2.16E+09	0	1.6E+08	11300000	89553000
Q12906;Q	Q12906	Interleukin enhancer-binding factor 3	ILF3	sp|Q12906	27	27	27	14	25	22	22	14	25	22	22	14	25	22	22	36.9	95.337	0	149.91	4.019E+09	4.8E+08	1.49E+09	262920000	1.79E+09	1.68E+08	2.08E+08	353430000	1.31E+08
P35637	P35637	RNA-binding protein FUS	FUS	sp|P35637	10	10	8	5	9	7	10	5	9	7	10	4	7	6	8	22.1	53.425	0	108.64	4.006E+09	59659000	1.24E+09	309860000	2.39E+09	93000000	89748000	212070000	79934000
P53999	P53999	Activated RNA polymerase II transcriptional coact	SUB1	sp|P53999	9	9	9	4	7	5	9	4	7	5	9	4	7	5	9	45.7	14.395	0	50.916	4.001E+09	4.93E+08	1.54E+09	29633000	1.94E+09	4.18E+08	1.81E+08	36007000	89801000
P09429;B2	P09429;B2	High mobility group protein B1;Putative high mob	HMGB1;HI	sp|P09429	12	12	8	4	12	1	10	4	12	1	10	4	8	1	7	40.5	24.893	0	50.241	3.998E+09	32878000	2.24E+09	2073400	1.72E+09	24146000	1.92E+08	2145100	74329000
O95782	O95782	AP-2 complex subunit alpha-1	AP2A1	sp|O95782	24	24	19	0	13	11	24	0	13	11	24	0	9	8	19	30.6	107.54	0	128.83	3.991E+09	0	3.5E+08	43073000	3.6E+09	0	39448000	41795000	1.25E+08
P26368	P26368	Splicing factor U2AF 65 kDa subunit	U2AF2	sp|P26368	11	11	11	1	7	8	10	1	7	8	10	1	7	8	10	38.9	53.5	0	100.32	3.977E+09	19071000	1.36E+09	144920000	2.45E+09	19762000	1.26E+08	104010000	87724000
O00429	O00429	Dynamin-1-like protein	DNM1L	sp|O00429	26	26	26	0	22	6	26	0	22	6	26	0	22	6	26	52.9	81.876	0	323.31	3.955E+09	0	1.12E+09	7863100	2.83E+09	0	1.06E+08	4211400	1.1E+08
P14866	P14866	Heterogeneous nuclear ribonucleoprotein L	HNRNPL	sp|P14866	19	19	19	6	16	12	17	6	16	12	17	6	16	12	17	44.3	64.132	0	130.37	3.926E+09	6.42E+08	1.05E+09	147720000	2.09E+09	3.23E+08	1.15E+08	339850000	1.38E+08
P55010	P55010	Eukaryotic translation initiation factor 5	EIF5	sp|P55010	17	17	17	4	14	10	17	4	14	10	17	4	14	10	17	40.8	49.222	0	66.68	3.912E+09	6909100	9.27E+08	30617000	2.95E+09	9256000	92861000	22937000	1.05E+08
Q09028	Q09028	Histone-binding protein RBBP4	RBBP4	sp|Q09028	14	14	7	4	13	8	13	4	13	8	13	2	7	4	6	44.7	47.655	0	67.615	3.909E+09	40421000	1.82E+09	62031000	1.99E+09	78640000	1.17E+08	39410000	88829000
Q07960	Q07960	Rho GTPase-activating protein 1	ARHGAP1	sp|Q07960	16	16	16	2	14	4	16	2	14	4	16	2	14	4	16	47.2	50.435	0	95.367	3.902E+09	20352000	4.03E+08	8910800	3.47E+09	12766000	33454000	7065600	1.51E+08
P52292	P52292	Importin subunit alpha-1	KPNA2	sp|P52292	16	16	16	2	15	13	16	2	15	13	16	2	15	13	16	47.6	57.861	0	135.52	3.897E+09	5302800	1.23E+09	98697000	2.56E+09	8719000	1.09E+08	59104000	1.08E+08
Q9Y266	Q9Y266	Nuclear migration protein nudC	NUDC	sp|Q9Y266	22	22	22	0	21	8	20	0	21	8	20	0	21	8	20	62.5	38.242	0	71.153	3.886E+09	0	1.06E+09	28354000	2.79E+09	0	1.01E+08	16558000	1.09E+08
P42766	P42766	60S ribosomal protein L35	RPL35	sp|P42766	5	5	5	3	4	4	5	3	4	4	5	3	4	4	5	26.8	14.551	0	26.832	3.87E+09	1.44E+08	1.02E+09	464860000	2.24E+09	1.47E+08	80135000	318780000	67840000
P62851	P62851	40S ribosomal protein S25	RPS25	sp|P62851	5	5	5	2	5	5	5	2	5	5	5	2	5	5	5	29.6	13.742	0	12.849	3.848E+09	73506000	1.53E+09	265500000	1.98E+09	61420000	1.3E+08	184380000	84663000
P46379	P46379	Large proline-rich protein BAG6	BAG6	sp|P46379	31	31	31	14	12	30	18	14	12	30	18	14	12	30	18	40.2	119.41	0	166.69	3.846E+09	6.09E+08	1.41E+08	2.47E+09	6.26E+08	9.35E+08	12526000	1.331E+09	23767000
P51991	P51991	Heterogeneous nuclear ribonucleoprotein A3	HNRNPA3	sp|P51991	16	16	16	6	12	12	14	6	12	12	14	6	12	12	14	39.7	39.594	0	85.677	3.838E+09	1.01E+09	8.12E+08	271100000	1.75E+09	7.48E+08	99143000	391160000	91149000
Q8NBS9	Q8NBS9	Thioredoxin domain-containing protein 5	TXNDC5	sp|Q8NBS9	13	13	13	1	13	4	12	1	13	4	12	1	13	4	12	35.6	47.628	0	72.491	3.814E+09	1188500	2.28E+09	10901000	1.52E+09	1756500	2.32E+08	3779700	34807000
Q9NR30	Q9NR30	Nucleolar RNA helicase 2	DDX21	sp|Q9NR30	28	28	26	3	20	24	26	3	20	24	26	3	18	22	24	42.1	87.343	0	145.3	3.812E+09	49272000	5.36E+08	357730000	2.87E+09	76382000	34389000	241900000	87603000
P62266	P62266	40S ribosomal protein S23	RPS23	sp|P62266	8	8	8	3	8	8	7	3	8	8	7	3	8	8	7	49.7	15.807	0	30.109	3.811E+09	1.23E+09	7.95E+08	190920000	1.59E+09	1.15E+09	80911000	178130000	83493000
P09382	P09382	Galectin-1	LGALS1	sp|P09382	8	8	8	0	3	8	8	0	3	8	8	0	3	8	8	71.9	14.716	0	40.622	3.803E+09	0	18589000	142560000	3.64E+09	0	2808500	65946000	1.78E+08
Q13501	Q13501	Sequestosome-1	SQSTM1	sp|Q13501	15	15	15	1	7	11	15	1	7	11	15	1	7	11	15	58.2	47.687	0	257.63	3.799E+09	1366400	93151000	209450000	3.5E+09	2591400	5875700	159210000	1.26E+08
P47895	P47895	Aldehyde dehydrogenase family 1 member A3	ALDH1A3	sp|P47895	22	22	20	0	3	5	22	0	3	5	22	0	1	4	20	46.1	56.108	0	105.89	3.767E+09	0	44561000	8350000	3.71E+09	0	2505700	2994900	1.55E+08
P61289	P61289	Proteasome activator complex subunit 3	PSME3	sp|P61289	15	15	15	0	12	4	14	0	12	4	14	0	12	4	14	62.2	29.506	0	66.257	3.762E+09	0	1.18E+09	14900000	2.56E+09	0	1.12E+08	14564000	91651000
P60660	P60660	Myosin light polypeptide 6	MYL6	sp|P60660	7	7	5	5	4	6	7	5	4	6	7	4	3	4	5	47.7	16.93	0	21.131	3.753E+09	3.04E+08	2.18E+08	53289000	3.18E+09	3.13E+08	18837000	37198000	1.2E+08
P84098	P84098	60S ribosomal protein L19	RPL19	sp|P84098	9	9	9	2	7	8	8	2	7	8	8	2	7	8	8	36.2	23.466	0	32.733	3.749E+09	1540800	9.48E+08	636970000	2.16E+09	4822700	1.03E+08	338770000	1.14E+08
Q15029	Q15029	116 kDa U5 small nuclear ribonucleoprotein comp	EFTUD2	sp|Q15029	27	26	26	5	26	24	26	5	25	23	25	5	25	23	25	39	109.43	0	192.97	3.743E+09	11245000	1.38E+09	147670000	2.21E+09	16388000	1.1E+08	124630000	71567000
P37108	P37108	Signal recognition particle 14 kDa protein	SRP14	sp|P37108	9	9	9	3	8	7	8	3	8	7	8	3	8	7	8	50.7	14.57	0	36.738	3.741E+09	2.83E+08	1.67E+09	139820000	1.65E+09	1.43E+08	1.22E+08	170140000	71842000
P12111	P12111	Collagen alpha-3(VI) chain	COL6A3	sp|P12111	51	51	51	1	2	9	51	1	2	9	51	1	2	9	51	23.6	343.67	0	323.31	3.735E+09	20530000	3247200	14613000	3.7E+09	4749200	560780	3147700	1.74E+08
P61221	P61221	ATP-binding cassette sub-family E member 1	ABCE1	sp|P61221	14	14	14	6	14	4	14	6	14	4	14	6	14	4	14	34.1	67.314	0	178.9	3.726E+09	51489000	1.12E+09	10822000	2.54E+09	50334000	1.1E+08	11230000	81925000
Q58FF8	Q58FF8	Putative heat shock protein HSP 90-beta 2	HSP90AB2	sp|Q58FF8	9	1	1	5	9	6	9	1	1	1	1	1	1	1	1	16.8	44.348	0	16.794	3.706E+09	2.68E+08	6.65E+08	15155000	2.76E+09	2.08E+08	1.12E+08	19535000	1.11E+08
Q9H9B4	Q9H9B4	Sideroflexin-1	SFXN1	sp|Q9H9B4	13	13	13	2	13	10	11	2	13	10	11	2	13	10	11	55	35.619	0	69.07	3.699E+09	33529000	1.79E+09	71840000	1.81E+09	46894000	1.44E+08	42979000	66043000
P49720	P49720	Proteasome subunit beta type-3	PSMB3	sp|P49720	8	8	8	0	6	5	8	0	6	5	8	0	6	5	8	44.9	22.949	0	41.807	3.679E+09	0	1.43E+09	43092000	2.21E+09	0	1.04E+08	37890000	96980000
Q9NYU2;Q	Q9NYU2	UDP-glucose:glycoprotein glucosyltransferase 1	UGGT1	sp|Q9NYU	31	31	31	0	29	6	29	0	29	6	29	0	29	6	29	29.6	177.19	0	193.12	3.67E+09	0	6.91E+08	20797000	2.96E+09	0	74047000	12699000	1.1E+08
P00367;P4	P00367;P4	Glutamate dehydrogenase 1, mitochondrial;Gluta	GLUD1;GLU	sp|P00367	24	24	24	0	24	9	20	0	24	9	20	0	24	9	20	53.2	61.397	0	88.493	3.664E+09	0	1.41E+09	15770000	2.24E+09	0	1.04E+08	16202000	80837000
Q16891	Q16891	MICOS complex subunit MIC60	IMMT	sp|Q16891	30	30	30	8	29	19	25	8	29	19	25	8	29	19	25	48.2	83.677	0	189.72	3.645E+09	28434000	2.3E+09	64413000	1.25E+09	29749000	1.67E+08	59903000	63585000
Q15046	Q15046	Lysine--tRNA ligase	KARS	sp|Q15046	31	31	31	5	27	20	28	5	27	20	28	5	27	20	28	59.8	68.047	0	141.9	3.634E+09	32280000	1.13E+09	162700000	2.3E+09	47183000	92873000	90252000	93268000
Q15393	Q15393	Splicing factor 3B subunit 3	SF3B3	sp|Q15393	29	29	29	7	26	18	28	7	26	18	28	7	26	18	28	34.1	135.58	0	244.62	3.625E+09	2.11E+08	1.39E+09	73792000	1.95E+09	2.28E+08	1.07E+08	58498000	69357000
P04080	P04080	Cystatin-B	CSTB	sp|P04080	5	5	5	0	5	2	5	0	5	2	5	0	5	2	5	77.6	11.139	0	38.376	3.623E+09	0	5.77E+08	6266300	3.04E+09	0	63596000	4676600	1.11E+08
P28838	P28838	Cytosol aminopeptidase	LAP3	sp|P28838	24	24	24	1	22	4	23	1	22	4	23	1	22	4	23	49.7	56.166	0	103.9	3.612E+09	758070	1.35E+09	4322500	2.25E+09	1290800	1.32E+08	757450	80086000
P61160	P61160	Actin-related protein 2	ACTR2	sp|P61160	12	12	12	0	12	5	12	0	12	5	12	0	12	5	12	47	44.76	0	101.6	3.611E+09	0	5.31E+08	15049000	3.06E+09	0	50362000	14452000	1.17E+08
O60763	O60763	General vesicular transport factor p115	USO1	sp|O60763	28	28	28	3	25	8	28	3	25	8	28	3	25	8	28	45	107.89	0	183.51	3.61E+09	8482600	1.1E+09	15866000	2.49E+09	7502300	1.04E+08	6627800	96808000
P12081	P12081	Histidine--tRNA ligase, cytoplasmic	HARS	sp|P12081	18	18	12	0	16	6	17	0	16	6	17	0	10	4	12	41.8	57.41	0	178.4	3.604E+09	0	7.26E+08	6466500	2.87E+09	0	77313000	11685000	97419000
Q7L1Q6	Q7L1Q6	Basic leucine zipper and W2 domain-containing p	BZW1	sp|Q7L1Q6	21	21	20	0	20	8	21	0	20	8	21	0	19	8	20	60.9	48.043	0	115.63	3.586E+09	0	9.23E+08	17770000	2.64E+09	0	73178000	20175000	1.05E+08
P43243	P43243	Matrin-3	MATR3	sp|P43243	25	25	25	10	23	24	23	10	23	24	23	10	23	24	23	39.2	94.622	0	131.08	3.584E+09	2.27E+08	9.87E+08	762450000	1.61E+09	4.17E+08	52647000	359120000	48701000
O60664	O60664	Perilipin-3	PLIN3	sp|O60664	18	18	18	1	9	11	17	1	9	11	17	1	9	11	17	62.4	47.074	0	246.88	3.562E+09	5573000	2.05E+08	129100000	3.22E+09	6455800	17247000	84277000	1.33E+08
Q8TEX9	Q8TEX9	Importin-4	IPO4	sp|Q8TEX9	18	18	18	0	17	3	18	0	17	3	18	0	17	3	18	23.7	118.71	0	230.16	3.558E+09	0	1E+09	6777300	2.55E+09	0	89668000	4414000	1.04E+08
Q15436	Q15436	Protein transport protein Sec23A	SEC23A	sp|Q15436	21	21	19	1	15	2	20	1	15	2	20	1	13	2	18	39	86.16	0	144.15	3.532E+09	66874000	5.07E+08	11490000	2.95E+09	61661000	57459000	14621000	1.06E+08
P37837	P37837	Transaldolase	TALDO1	sp|P37837	15	15	15	1	15	6	14	1	15	6	14	1	15	6	14	42.4	37.54	0	44.913	3.515E+09	999190	1.75E+09	8598000	1.75E+09	1142800	1.39E+08	4175300	83619000
Q9NR31	Q9NR31	GTP-binding protein SAR1a	SAR1A	sp|Q9NR31	11	11	6	0	9	2	11	0	9	2	11	0	4	0	6	65.2	22.367	0	89.717	3.509E+09	0	9.15E+08	3733500	2.59E+09	0	80750000	3686700	1.05E+08
O15067	O15067	Phosphoribosylformylglycinamidine synthase	PFAS	sp|O15067	24	24	24	2	20	0	23	2	20	0	23	2	20	0	23	28.1	144.73	0	165.95	3.507E+09	3864800	9.26E+08	0	2.58E+09	2425900	79874000	0	99755000
P25786	P25786	Proteasome subunit alpha type-1	PSMA1	sp|P25786	14	14	14	0	14	12	12	0	14	12	12	0	14	12	12	56.7	29.555	0	134.94	3.47E+09	0	1.47E+09	83269000	1.91E+09	0	1.24E+08	53477000	85534000
P45880	P45880	Voltage-dependent anion-selective channel prote	VDAC2	sp|P45880	13	13	13	2	13	5	12	2	13	5	12	2	13	5	12	52.4	31.566	0	104.46	3.459E+09	2500700	1.95E+09	40004000	1.47E+09	2775500	1.78E+08	15968000	63491000
P50570	P50570	Dynamin-2	DNM2	sp|P50570	25	25	16	0	19	3	23	0	19	3	23	0	12	0	15	34.9	98.063	0	169.41	3.451E+09	0	6.61E+08	5410500	2.78E+09	0	67136000	4410000	1.02E+08
O43399	O43399	Tumor protein D54	TPD52L2	sp|O43399	13	13	13	0	11	10	13	0	11	10	13	0	11	10	13	76.2	22.237	0	150.31	3.442E+09	0	4.03E+08	121230000	2.92E+09	0	61905000	84143000	84401000
Q9Y285	Q9Y285	Phenylalanine--tRNA ligase alpha subunit	FARSA	sp|Q9Y285	18	18	18	1	15	7	17	1	15	7	17	1	15	7	17	49.4	57.563	0	182	3.432E+09	3051400	1.47E+09	35054000	1.92E+09	3798500	1.38E+08	21333000	72426000
P16070	P16070	CD44 antigen	CD44	sp|P16070	8	8	8	1	1	6	8	1	1	6	8	1	1	6	8	10.8	81.537	0	27.199	3.429E+09	633030	6035100	26371000	3.4E+09	411750	540250	5303700	1.38E+08
P30044	P30044	Peroxiredoxin-5, mitochondrial	PRDX5	sp|P30044	9	9	9	0	9	2	8	0	9	2	8	0	9	2	8	48.1	22.086	0	54.808	3.426E+09	0	1.2E+09	2112400	2.23E+09	0	1.16E+08	2058800	79799000
Q9UBT2	Q9UBT2	SUMO-activating enzyme subunit 2	UBA2	sp|Q9UBT	22	22	22	0	20	3	21	0	20	3	21	0	20	3	21	44.2	71.223	0	318.7	3.422E+09	0	1.41E+09	10909000	2E+09	0	1.29E+08	3981800	80607000
P35606	P35606	Coatomer subunit beta	COPB2	sp|P35606	25	25	25	3	21	15	25	3	21	15	25	3	21	15	25	41.2	102.49	0	165.46	3.417E+09	2591900	9.61E+08	57269000	2.4E+09	4869500	78751000	26549000	1.12E+08
Q07954	Q07954	Prolow-density lipoprotein receptor-related prote	LRP1	sp|Q07954	46	46	46	0	3	13	46	0	3	13	46	0	3	13	46	13.8	504.6	0	205.44	3.399E+09	0	14844000	34684000	3.35E+09	0	2229900	10823000	1.48E+08
P27694	P27694	Replication protein A 70 kDa DNA-binding subunit	RPA1	sp|P27694	22	22	22	8	16	9	13	8	16	9	13	8	16	9	13	45.9	68.137	0	159.28	3.376E+09	1.59E+09	9.15E+08	48924000	8.2E+08	1.59E+09	69510000	26820000	30604000
O00159	O00159	Unconventional myosin-Ic	MYO1C	sp|O00159	31	31	31	3	14	22	31	3	14	22	31	3	14	22	31	39.6	121.68	0	180.54	3.372E+09	11515000	1.03E+08	163160000	3.09E+09	39641000	10294000	82529000	1.24E+08
P48444	P48444	Coatomer subunit delta	ARCN1	sp|P48444	17	17	17	3	15	11	17	3	15	11	17	3	15	11	17	40.7	57.21	0	53.202	3.371E+09	3899200	6.94E+08	41198000	2.63E+09	4300400	74680000	15253000	1.07E+08
Q9BXJ9	Q9BXJ9	N-alpha-acetyltransferase 15, NatA auxiliary subu	NAA15	sp|Q9BXJ9	25	25	22	1	23	7	22	1	23	7	22	0	20	5	20	35.2	101.27	0	173.76	3.332E+09	7771600	1.07E+09	13778000	2.24E+09	6331100	1.14E+08	7071400	76014000
Q02818	Q02818	Nucleobindin-1	NUCB1	sp|Q02818	3	3	3	0	0	1	3	0	0	1	3	0	0	1	3	9.8	53.879	0	7.8709	3.332E+09	0	0	3.263E+09	69319000	0	0	0	2850800
Q7Z6Z7	Q7Z6Z7	E3 ubiquitin-protein ligase HUWE1	HUWE1	sp|Q7Z6Z7	58	58	58	1	54	2	45	1	54	2	45	1	54	2	45	22.6	481.89	0	323.31	3.315E+09	1199000	1.8E+09	2729000	1.51E+09	4106800	1.41E+08	3250000	74025000
P46779	P46779	60S ribosomal protein L28	RPL28	sp|P46779	8	8	8	1	8	7	7	1	8	7	7	1	8	7	7	42.3	15.747	0	30.331	3.312E+09	9469600	6.9E+08	359220000	2.25E+09	7178400	37811000	246630000	77319000
P30566	P30566	Adenylosuccinate lyase	ADSL	sp|P30566	13	13	13	0	12	1	13	0	12	1	13	0	12	1	13	39.9	54.889	0	75.58	3.312E+09	0	1.14E+09	328050	2.17E+09	0	1.1E+08	505940	79937000
P62304	P62304	Small nuclear ribonucleoprotein E	SNRPE	sp|P62304	3	3	3	0	3	2	2	0	3	2	2	0	3	2	2	38	10.803	0	9.3015	3.29E+09	0	1.98E+09	51966000	1.25E+09	0	1.67E+08	51423000	43071000
Q9BZQ8	Q9BZQ8	Protein Niban	FAM129A	sp|Q9BZQ8	20	20	20	0	2	7	20	0	2	7	20	0	2	7	20	31.1	103.13	0	142.27	3.284E+09	0	6939800	25913000	3.25E+09	0	1384300	14162000	1.36E+08
Q99536	Q99536	Synaptic vesicle membrane protein VAT-1 homolo	VAT1	sp|Q99536	16	16	16	1	13	2	15	1	13	2	15	1	13	2	15	46.8	41.92	0	115.65	3.276E+09	983470	7.86E+08	2806100	2.49E+09	923970	60731000	1582000	1.11E+08
P54709	P54709	Sodium/potassium-transporting ATPase subunit b	ATP1B3	sp|P54709	10	10	10	0	9	2	9	0	9	2	9	0	9	2	9	36.6	31.512	0	49.387	3.26E+09	0	1.21E+09	10810000	2.04E+09	0	87251000	9717800	89257000
O95831	O95831	Apoptosis-inducing factor 1, mitochondrial	AIFM1	sp|O95831	22	22	22	2	22	12	16	2	22	12	16	2	22	12	16	48.5	66.9	0	84.288	3.25E+09	26746000	2.19E+09	40512000	9.95E+08	23819000	1.9E+08	28485000	45145000
Q9P258	Q9P258	Protein RCC2	RCC2	sp|Q9P258	12	12	12	3	12	9	10	3	12	9	10	3	12	9	10	29.9	56.084	0	51.209	3.25E+09	1.69E+09	7.04E+08	19739000	8.34E+08	9.13E+08	4.65E+08	148030000	2.7E+08
Q92973	Q92973	Transportin-1	TNPO1	sp|Q92973	20	20	16	2	16	8	20	2	16	8	20	1	13	6	16	33.4	102.35	0	115.97	3.247E+09	12565000	6.55E+08	19971000	2.56E+09	2329000	1.08E+08	7595000	61425000
Q96HE7;Q	Q96HE7	ERO1-like protein alpha	ERO1L	sp|Q96HE7	11	11	11	1	8	1	11	1	8	1	11	1	8	1	11	36.3	54.392	0	124.05	3.228E+09	811210	4.51E+08	279540	2.78E+09	2652700	1.29E+08	1276500	21355000
Q01082;P1	Q01082	Spectrin beta chain, non-erythrocytic 1	SPTBN1	sp|Q01082	65	65	61	2	57	53	53	2	57	53	53	2	54	51	50	40.4	274.61	0	299.72	3.212E+09	8259700	1.29E+09	529720000	1.39E+09	12758000	83751000	273460000	1.65E+08
P27695	P27695	DNA-(apurinic or apyrimidinic site) lyase;DNA-(ap	APEX1	sp|P27695	15	15	15	0	12	5	14	0	12	5	14	0	12	5	14	53.8	35.554	0	179.46	3.206E+09	0	1.23E+09	10877000	1.96E+09	0	1E+08	19014000	77347000
O43143	O43143	Pre-mRNA-splicing factor ATP-dependent RNA he	DHX15	sp|O43143	21	21	20	5	18	16	17	5	18	16	17	4	17	15	16	27.2	90.932	0	91.511	3.198E+09	17610000	9.87E+08	121420000	2.07E+09	35502000	68126000	97592000	70226000
P25398	P25398	40S ribosomal protein S12	RPS12	sp|P25398	4	4	4	2	4	3	3	2	4	3	3	2	4	3	3	37.1	14.515	0	40.225	3.19E+09	1.89E+08	1.47E+09	149950000	1.38E+09	2.67E+08	92112000	75644000	41144000
Q9P287	Q9P287	BRCA2 and CDKN1A-interacting protein	BCCIP	sp|Q9P287	8	8	8	0	8	0	7	0	8	0	7	0	8	0	7	36.3	35.979	0	51.276	3.182E+09	0	1.55E+09	0	1.63E+09	0	1.25E+08	0	70481000
Q9UHX1	Q9UHX1	Poly(U)-binding-splicing factor PUF60	PUF60	sp|Q9UHX	15	15	15	3	15	12	13	3	15	12	13	3	15	12	13	40.3	59.875	0	103.17	3.175E+09	9997800	1.2E+09	96720000	1.87E+09	14068000	1.24E+08	53084000	64886000
P26006	P26006	Integrin alpha-3;Integrin alpha-3 heavy chain;Inte	ITGA3	sp|P26006	17	17	17	1	2	12	16	1	2	12	16	1	2	12	16	19.6	116.61	0	96.482	3.172E+09	129060	21748000	54386000	3.1E+09	0	3068900	13007000	1.38E+08
P49748	P49748	Very long-chain specific acyl-CoA dehydrogenase,	ACADVL	sp|P49748	24	24	24	0	14	3	23	0	14	3	23	0	14	3	23	45.5	70.389	0	175.61	3.157E+09	0	3.52E+08	5554200	2.8E+09	0	22730000	7553700	1.19E+08
Q9P2E9	Q9P2E9	Ribosome-binding protein 1	RRBP1	sp|Q9P2E9	46	46	45	8	40	34	41	8	40	34	41	8	40	33	41	51.8	152.45	0	181.25	3.145E+09	26293000	9.42E+08	188040000	1.99E+09	26893000	93095000	115770000	78045000
Q9Y696	Q9Y696	Chloride intracellular channel protein 4	CLIC4	sp|Q9Y696	9	9	9	0	8	2	9	0	8	2	9	0	8	2	9	55.7	28.772	0	70.927	3.143E+09	0	4.38E+08	4874100	2.7E+09	0	27973000	7761100	96264000
Q5VYK3	Q5VYK3	Proteasome-associated protein ECM29 homolog	ECM29	sp|Q5VYK3	30	30	30	0	29	8	28	0	29	8	28	0	29	8	28	23.4	204.29	0	152.41	3.122E+09	0	1.14E+09	11664000	1.97E+09	0	1.08E+08	5228700	75562000

P36957	P36957	Dihydrolipoyllysine-residue succinyltransferase cc	DLST	sp|P36957	9	9	9	7	8	6	9	7	8	6	9	7	8	6	9	21	48.755	0	101.57	3.121E+09	2.73E+08	9.53E+08	98238000	1.8E+09	1.59E+08	1.12E+08	123520000	1.03E+08
P51665	P51665	26S proteasome non-ATPase regulatory subunit 7	PSMD7	sp|P51665	10	10	10	2	10	9	10	2	10	9	10	2	10	9	10	41.7	37.025	0	114.88	3.118E+09	123920	6.79E+08	42629000	2.4E+09	1455100	1.27E+08	16914000	38523000
P42677	P42677	40S ribosomal protein S27	RPS27	sp|P42677	6	6	3	2	5	5	5	2	5	5	5	2	3	3	2	41.7	9.461	0	23.588	3.118E+09	40578000	9.75E+08	118950000	1.98E+09	35732000	58951000	126420000	52159000
P18085	P18085	ADP-ribosylation factor 4	ARF4	sp|P18085	8	4	4	1	8	7	8	1	4	4	4	1	4	4	4	52.8	20.511	0	20.153	3.103E+09	1799900	5.94E+08	39233000	2.47E+09	4069800	27474000	24248000	1.26E+08
Q04917	Q04917	14-3-3 protein eta	YWHAH	sp|Q04917	16	12	12	4	16	9	15	3	12	6	11	3	12	6	11	61	28.218	0	211.48	3.102E+09	4164100	9.84E+08	16677000	2.1E+09	4072500	77514000	8161200	95833000
P28072	P28072	Proteasome subunit beta type-6	PSMB6	sp|P28072	7	7	7	1	6	4	6	1	6	4	6	1	6	4	6	51.5	25.357	0	105.39	3.1E+09	1572500	8.73E+08	27964000	2.2E+09	2398100	70876000	29090000	83271000
Q9Y617	Q9Y617	Phosphoserine aminotransferase	PSAT1	sp|Q9Y617	15	15	15	1	15	0	11	1	15	0	11	1	15	0	11	50.8	40.422	0	53.519	3.099E+09	2118400	2.16E+09	0	9.37E+08	3253000	2.1E+08	0	15743000
Q14008	Q14008	Cytoskeleton-associated protein 5	CKAP5	sp|Q14008	39	39	39	3	32	15	38	3	32	15	38	3	32	15	38	26.3	225.49	0	164.35	3.098E+09	1787300	8.22E+08	42118000	2.23E+09	9313700	92735000	37448000	54190000
P14550	P14550	Alcohol dehydrogenase [NADP(+)]	AKR1A1	sp|P14550	9	9	9	0	9	1	8	0	9	1	8	0	9	1	8	28.9	36.573	0	29.111	3.088E+09	0	5.11E+08	2873100	2.57E+09	0	78485000	2310900	72039000
Q13283	Q13283	Ras GTPase-activating protein-binding protein 1	G3BP1	sp|Q13283	16	16	14	2	14	12	14	2	14	12	14	1	13	11	13	45.3	52.164	0	135.49	3.085E+09	91349	7.64E+08	372190000	1.95E+09	211770	15686000	264660000	1.01E+08
Q16181;Q(	Q16181	Septin-7	Sep-07	sp|Q16181	14	14	14	2	14	9	14	2	14	9	14	2	14	9	14	37.3	50.679	0	100.23	3.075E+09	669910	9.71E+08	49248000	2.05E+09	891200	83840000	12735000	87223000
Q15631	Q15631	Translin	TSN	sp|Q15631	13	13	13	2	13	3	10	2	13	3	10	2	13	3	10	59.6	26.183	0	62.476	3.06E+09	2008900	1.14E+09	4585600	1.92E+09	845710	1.08E+08	1398400	73965000
P35613	P35613	Basigin	BSG	sp|P35613	12	12	12	3	12	4	9	3	12	4	9	3	12	4	9	42.6	42.2	0	74.9	3.045E+09	5455300	1.83E+09	7995500	1.2E+09	2899500	1.62E+08	3762600	47243000
Q9NSD9	Q9NSD9	Phenylalanine--tRNA ligase beta subunit	FARSB	sp|Q9NSD	20	20	20	1	19	10	19	1	19	10	19	1	19	10	19	40.9	66.115	0	99.12	3.042E+09	2180600	9.8E+08	33501000	2.03E+09	3962900	88437000	23923000	78070000
Q6PKG0;Q	Q6PKG0	La-related protein 1	LARP1	sp|Q6PKG	33	33	33	4	27	27	28	4	27	27	28	4	27	27	28	47.1	123.51	0	165.91	3.036E+09	14060000	1E+09	242330000	1.78E+09	16529000	82832000	169700000	69255000
P06396	P06396	Gelsolin	GSN	sp|P06396	22	22	11	3	14	12	22	3	14	12	22	1	5	3	11	47.8	85.696	0	189.54	3.036E+09	8035600	1.97E+08	58257000	2.77E+09	15440000	20388000	38100000	1.05E+08
P55036	P55036	26S proteasome non-ATPase regulatory subunit 4	PSMD4	sp|P55036	14	14	10	0	14	10	14	0	14	10	14	0	10	6	10	39.5	40.736	0	150.84	3.036E+09	0	1.28E+09	64473000	1.69E+09	0	1.09E+08	38969000	67064000
P54819	P54819	Adenylate kinase 2, mitochondrial;Adenylate kina	AK2	sp|P54819	13	13	13	1	13	1	13	1	13	1	13	1	13	1	13	67.4	26.477	0	75.054	3.032E+09	2562300	2.17E+09	1283300	8.58E+08	1362400	1.96E+08	861280	15897000
Q6IBS0	Q6IBS0	Twinfilin-2	TWF2	sp|Q6IBS0	15	14	14	1	10	5	15	1	9	4	14	1	9	4	14	52.1	39.548	0	110.23	3.029E+09	612390	2.54E+08	9017500	2.77E+09	0	36444000	3954200	1.02E+08
P28074	P28074	Proteasome subunit beta type-5	PSMB5	sp|P28074	10	10	10	1	9	9	10	1	9	9	10	1	9	9	10	44.9	28.48	0	93.283	3.029E+09	774650	1.11E+09	56756000	1.86E+09	4717100	60974000	19809000	1.28E+08
P61224;A6	P61224;A6	Ras-related protein Rap-1b;Ras-related protein Ra	RAP1B	sp|P61224	9	9	3	1	9	2	9	1	9	2	9	0	3	0	3	64.1	20.825	0	100.33	3.023E+09	424410	4.4E+08	18045000	2.56E+09	7987600	40907000	24271000	76971000
P40121	P40121	Macrophage-capping protein	CAPG	sp|P40121	9	9	9	0	1	1	9	0	1	1	9	0	1	1	9	35.6	38.498	0	105.2	3.009E+09	0	2850000	4767100	3E+09	0	191180	531310	1.08E+08
P60866	P60866	40S ribosomal protein S20	RPS20	sp|P60866	5	5	5	4	4	3	4	4	4	3	4	4	4	3	4	31.1	13.373	0	20.319	3.004E+09	81641000	1.1E+09	155710000	1.67E+09	66935000	99018000	123560000	61911000
P68400;Q8	P68400;Q8	Casein kinase II subunit alpha;Casein kinase II sub	CSNK2A1;(	sp|P68400	12	12	12	0	12	10	11	0	12	10	11	0	12	10	11	44.5	45.143	0	97.278	2.97E+09	0	1.22E+09	171540000	1.58E+09	0	87427000	138430000	58832000
Q07666;Q5	Q07666	KH domain-containing, RNA-binding, signal transc	KHDRBS1	sp|Q07666	10	10	10	1	9	8	8	1	9	8	8	1	9	8	8	28.7	48.227	0	51.612	2.969E+09	8354000	1.59E+09	106350000	1.26E+09	11185000	1.6E+08	50496000	49440000
O60841	O60841	Eukaryotic translation initiation factor 5B	EIF5B	sp|O60841	31	31	31	2	29	16	27	2	29	16	27	2	29	16	27	33.8	138.83	0	211.23	2.965E+09	4039700	1.15E+09	50328000	1.76E+09	2489500	92744000	23782000	91877000
Q9UJZ1	Q9UJZ1	Stomatin-like protein 2, mitochondrial	STOML2	sp|Q9UJZ1	13	13	13	2	13	6	13	2	13	6	13	2	13	6	13	48.9	38.534	0	132.68	2.951E+09	5005500	1.18E+09	13074000	1.76E+09	4662200	88000000	11870000	69807000
P18077	P18077	60S ribosomal protein L35a	RPL35A	sp|P18077	11	11	11	3	10	9	11	3	10	9	11	3	10	9	11	57.3	12.538	0	25.746	2.948E+09	19375000	6.99E+08	355920000	1.87E+09	28610000	52822000	230200000	77498000
Q93009	Q93009	Ubiquitin carboxyl-terminal hydrolase 7	USP7	sp|Q93009	26	26	26	1	24	13	25	1	24	13	25	1	24	13	25	34.1	128.3	0	145.89	2.946E+09	24240000	8.41E+08	44351000	2.04E+09	34420000	70468000	41655000	65376000
P00441	P00441	Superoxide dismutase [Cu-Zn]	SOD1	sp|P00441	8	8	8	0	8	2	6	0	8	2	6	0	8	2	6	76.6	15.936	0	57.496	2.919E+09	0	2.53E+09	3043800	3.89E+08	0	2.1E+08	3004100	24781000
P50213	P50213	Isocitrate dehydrogenase [NAD] subunit alpha, m	IDH3A	sp|P50213	10	10	10	0	9	4	7	0	9	4	7	0	9	4	7	31.1	39.591	0	81.39	2.907E+09	0	1.31E+09	4230900	1.6E+09	0	1.09E+08	13230000	61888000
Q14444	Q14444	Caprin-1	CAPRIN1	sp|Q14444	12	12	12	6	11	12	12	6	11	12	12	6	11	12	12	26	78.365	0	69.484	2.902E+09	60742000	6.47E+08	357660000	1.84E+09	65849000	40024000	259300000	52044000
Q99829	Q99829	Copine-1	CPNE1	sp|Q99829	13	13	13	3	8	6	13	3	8	6	13	3	8	6	13	37.6	59.058	0	148.64	2.896E+09	20870000	1.42E+08	26972000	2.71E+09	54544000	10425000	7098100	90139000
P28066	P28066	Proteasome subunit alpha type-5	PSMA5	sp|P28066	10	10	10	1	8	3	9	1	8	3	9	1	8	3	9	41.9	26.411	0	49.509	2.894E+09	6430700	1.03E+09	23381000	1.84E+09	10213000	77991000	22491000	77516000
P30048	P30048	Thioredoxin-dependent peroxide reductase, mito	PRDX3	sp|P30048	10	10	10	1	10	3	9	1	10	3	9	1	10	3	9	43.4	27.692	0	45.065	2.892E+09	6509100	1.65E+09	3784800	1.23E+09	4040100	1.07E+08	2087700	50175000
P09936	P09936	Ubiquitin carboxyl-terminal hydrolase isozyme L1	UCHL1	sp|P09936	9	9	9	0	7	0	8	0	7	0	8	0	7	0	8	51.6	24.824	0	36.922	2.887E+09	0	5.12E+08	0	2.38E+09	0	64780000	0	78013000
P30084	P30084	Enoyl-CoA hydratase, mitochondrial	ECHS1	sp|P30084	12	12	12	1	11	3	9	1	11	3	9	1	11	3	9	46.9	31.387	0	88.93	2.874E+09	1320500	1.38E+09	25443000	1.47E+09	0	1.23E+08	31431000	44666000
Q6DD88	Q6DD88	Atlastin-3	ATL3	sp|Q6DD8	18	18	18	0	8	5	18	0	8	5	18	0	8	5	18	42.7	60.541	0	152.61	2.858E+09	0	1.68E+08	6691200	2.68E+09	0	16821000	6964000	1.05E+08
P20810	P20810	Calpastatin	CAST	sp|P20810	20	20	20	0	12	1	20	0	12	1	20	0	12	1	20	46	76.572	0	149.05	2.858E+09	0	2.1E+08	2666900	2.65E+09	0	24409000	3102600	1.01E+08
P62987;P0	P62987;P0	Ubiquitin-60S ribosomal protein L40;Ubiquitin;60	UBA52;UB	sp|P62987	5	5	1	1	5	5	5	1	5	5	5	0	1	1	1	44.5	14.728	0	46.801	2.857E+09	4301800	9.39E+08	349020000	1.57E+09	5370600	80409000	246010000	51858000
P06737	P06737	Glycogen phosphorylase, liver form	PYGL	sp|P06737	35	35	29	0	32	2	30	0	32	2	30	0	26	0	24	51.2	97.147	0	162.54	2.834E+09	0	1.04E+09	1901700	1.79E+09	0	1.05E+08	1233300	58210000
O75874	O75874	Isocitrate dehydrogenase [NADP] cytoplasmic	IDH1	sp|O75874	16	15	15	0	13	9	16	0	12	8	15	0	12	8	15	45.9	46.659	0	70.447	2.827E+09	0	5.51E+08	13877000	2.26E+09	0	55572000	15099000	80235000
P59998	P59998	Actin-related protein 2/3 complex subunit 4	ARPC4	sp|P59998	8	8	8	0	7	5	7	0	7	5	7	0	7	5	7	60.1	19.667	0	67.594	2.825E+09	0	3.73E+08	78957000	2.37E+09	0	44234000	18197000	1.21E+08
P62495	P62495	Eukaryotic peptide chain release factor subunit 1	ETF1	sp|P62495	15	15	15	1	13	5	15	1	13	5	15	1	13	5	15	50.6	49.03	0	94.911	2.821E+09	49069000	6.75E+08	11631000	2.08E+09	36677000	58034000	15310000	91163000
Q16836	Q16836	Hydroxyacyl-coenzyme A dehydrogenase, mitoch	HADH	sp|Q16836	9	9	9	0	9	1	9	0	9	1	9	0	9	1	9	49	34.293	0	101.81	2.802E+09	0	1.21E+09	9793100	1.58E+09	0	1.29E+08	2453000	46435000
Q9H299	Q9H299	SH3 domain-binding glutamic acid-rich-like protei	SH3BGRL3	sp|Q9H299	4	4	4	0	1	1	4	0	1	1	4	0	1	1	4	32.3	10.438	0	40.056	2.792E+09	0	19243000	3561200	2.77E+09	0	1724800	2433500	1.14E+08
P16949	P16949	Stathmin	STMN1	sp|P16949	8	8	6	0	8	2	6	0	8	2	6	0	6	1	4	42.3	17.302	0	51.533	2.775E+09	0	1.46E+09	1945500	1.32E+09	0	1.29E+08	1544700	53498000
Q08945	Q08945	FACT complex subunit SSRP1	SSRP1	sp|Q08945	21	21	21	2	20	13	16	2	20	13	16	2	20	13	16	35.4	81.074	0	147.93	2.775E+09	22498000	2E+09	94698000	6.59E+08	29516000	1.63E+08	60202000	33967000
Q86V81	Q86V81	THO complex subunit 4	ALYREF	sp|Q86V81	10	10	10	6	10	10	9	6	10	10	9	6	10	10	9	46.7	26.888	0	84.96	2.775E+09	1.92E+08	1.35E+09	177190000	1.05E+09	2.26E+08	98843000	95293000	35429000
P09960	P09960	Leukotriene A-4 hydrolase	LTA4H	sp|P09960	21	21	21	1	21	0	16	1	21	0	16	1	21	0	16	44.5	69.284	0	91.137	2.772E+09	711980	1.89E+09	0	8.79E+08	848300	1.69E+08	0	33019000
Q9UHD8	Q9UHD8	Septin-9	Sep-09	sp|Q9UHD	22	22	22	2	15	13	21	2	15	13	21	2	15	13	21	48.5	65.401	0	123.18	2.763E+09	1537700	3.04E+08	40990000	2.42E+09	2466500	35131000	17328000	96609000
Q8N1F8	Q8N1F8	Serine/threonine-protein kinase 11-interacting pr	STK11IP	sp|Q8N1F8	2	2	2	0	1	1	2	0	1	1	2	0	1	1	2	4	120.26	0.001141	2.5741	2.756E+09	0	13105000	836880000	1.91E+09	0	15858000	349880000	2.76E+08
P62805	P62805	Histone H4	HIST1H4A	sp|P62805	9	9	9	5	7	6	8	5	7	6	8	5	7	6	8	60.2	11.367	0	27.52	2.755E+09	1.65E+08	8.19E+08	299220000	1.47E+09	1.37E+08	61715000	239430000	55663000
P63173	P63173	60S ribosomal protein L38	RPL38	sp|P63173	6	6	6	2	6	6	6	2	6	6	6	2	6	6	6	52.9	8.2178	0	16.389	2.754E+09	5.9E+08	5.4E+08	141620000	1.48E+09	6.15E+08	48130000	86672000	38331000
P43304	P43304	Glycerol-3-phosphate dehydrogenase, mitochond	GPD2	sp|P43304	21	21	21	1	17	5	21	1	17	5	21	1	17	5	21	38.5	80.852	0	126.97	2.753E+09	35755000	3.86E+08	21113000	2.31E+09	48784000	28337000	14198000	87636000
Q9UJS0	Q9UJS0	Calcium-binding mitochondrial carrier protein Ara	SLC25A13	sp|Q9UJS0	21	16	16	1	19	18	18	1	14	13	13	1	14	13	13	42.8	74.175	0	131.69	2.749E+09	549160	7.75E+08	98269000	1.88E+09	828350	70247000	58407000	82536000
Q99733	Q99733	Nucleosome assembly protein 1-like 4	NAP1L4	sp|Q99733	12	11	11	2	12	8	12	1	11	7	11	1	11	7	11	47.2	42.823	0	179.16	2.747E+09	1074400	9.02E+08	37521000	1.81E+09	1533600	92005000	11988000	74553000
P48637	P48637	Glutathione synthetase	GSS	sp|P48637	17	17	17	0	14	4	17	0	14	4	17	0	14	4	17	46.6	52.384	0	149.09	2.745E+09	0	6.85E+08	5342900	2.06E+09	0	55964000	1651200	90248000
P46459	P46459	Vesicle-fusing ATPase	NSF	sp|P46459	25	25	25	2	22	6	25	2	22	6	25	2	22	6	25	39.2	82.593	0	139.04	2.724E+09	2469300	9E+08	6189000	1.82E+09	4474000	97844000	5353200	52963000
P54886	P54886	Delta-1-pyrroline-5-carboxylate synthase;Glutama	ALDH18A1	sp|P54886	23	23	23	5	20	6	19	5	20	6	19	5	20	6	19	36	87.301	0	114.84	2.718E+09	1.17E+08	1.43E+09	9311200	1.16E+09	1.37E+08	1.03E+08	4353200	52111000
Q92900	Q92900	Regulator of nonsense transcripts 1	UPF1	sp|Q92900	32	32	32	6	31	26	27	6	31	26	27	6	31	26	27	38.2	124.34	0	127.15	2.714E+09	10181000	9.13E+08	196000000	1.6E+09	12175000	82505000	112250000	79225000
P62714;P6	P62714;P6	Serine/threonine-protein phosphatase 2A catalyt	PPP2CB;PF	sp|P62714	7	7	7	0	6	4	7	0	6	4	7	0	6	4	7	32.7	35.575	0	37.416	2.709E+09	0	6.81E+08	45278000	1.98E+09	0	55369000	26921000	64646000
P31689	P31689	DnaJ homolog subfamily A member 1	DNAJA1	sp|P31689	15	15	15	2	12	12	14	2	12	12	14	2	12	12	14	44.8	44.868	0	168.92	2.7E+09	13189000	4.83E+08	121910000	2.08E+09	5894700	31334000	122040000	49855000
P26196	P26196	Probable ATP-dependent RNA helicase DDX6	DDX6	sp|P26196	14	14	14	5	11	10	14	5	11	10	14	5	11	10	14	48	54.416	0	195.33	2.696E+09	30760000	5.2E+08	98236000	2.05E+09	44163000	47778000	73701000	57202000
A0AVT1	A0AVT1	Ubiquitin-like modifier-activating enzyme 6	UBA6	sp|A0AVT1	27	27	27	1	22	2	24	1	22	2	24	1	22	2	24	32.3	117.97	0	100.6	2.688E+09	40208	4.17E+08	5292400	2.27E+09	159370	12947000	1060700	1.19E+08
O00764	O00764	Pyridoxal kinase	PDXK	sp|O00764	11	11	11	0	11	1	11	0	11	1	11	0	11	1	11	45.8	35.102	0	157.73	2.674E+09	0	6.71E+08	1744700	2E+09	0	61243000	1296000	80077000
P21589	P21589	5-nucleotidase	NT5E	sp|P21589	15	15	15	0	1	14	15	0	1	14	15	0	1	14	15	38.9	63.367	0	118.59	2.672E+09	0	14111000	131680000	2.53E+09	0	4139900	41862000	1.44E+08
Q9BQG0	Q9BQG0	Myb-binding protein 1A	MYBBP1A	sp|Q9BQG	30	30	30	4	26	28	26	4	26	28	26	4	26	28	26	25.5	148.85	0	128.43	2.636E+09	82216000	7.04E+08	178410000	1.67E+09	1.18E+08	42579000	124640000	48104000
P15311	P15311	Ezrin	EZR	sp|P15311	28	18	16	4	24	14	26	2	15	8	16	2	13	7	15	45.4	69.412	0	56.293	2.636E+09	1216100	8.79E+08	21742000	1.73E+09	1259400	76251000	11154000	70264000
P15170	P15170	Eukaryotic peptide chain release factor GTP-bindi	GSPT1	sp|P15170	15	15	7	0	14	2	14	0	14	2	14	0	6	1	6	37.9	55.755	0	101.87	2.632E+09	0	5.87E+08	9158000	2.04E+09	0	57888000	6247600	77416000
P60900	P60900	Proteasome subunit alpha type-6	PSMA6	sp|P60900	7	7	7	1	7	5	7	1	7	5	7	1	7	5	7	35.4	27.399	0	38.52	2.622E+09	1545000	8.27E+08	48838000	1.75E+09	6724300	98764000	23885000	49630000
Q15363	Q15363	Transmembrane emp24 domain-containing prote	TMED2	sp|Q15363	6	6	6	1	6	4	6	1	6	4	6	1	6	4	6	34.3	22.761	0	72.807	2.621E+09	2955100	4.39E+08	15822000	2.16E+09	21619000	53816000	12267000	51263000
O75396	O75396	Vesicle-trafficking protein SEC22b	SEC22B	sp|O75396	11	11	11	0	11	6	11	0	11	6	11	0	11	6	11	46	24.593	0	58.451	2.616E+09	0	4.71E+08	18810000	2.13E+09	0	44763000	19953000	76904000
Q9NZI8	Q9NZI8	Insulin-like growth factor 2 mRNA-binding proteir	IGF2BP1	sp|Q9NZI8	19	19	17	11	19	3	3	11	19	3	3	9	17	1	1	38.5	63.48	0	90.678	2.614E+09	7.62E+08	1.64E+09	23904000	1.84E+08	7.34E+08	1.58E+08	20510000	11794000
Q01130;Q5	Q01130	Serine/arginine-rich splicing factor 2	SRSF2	sp|Q01130	7	7	7	2	7	7	7	2	7	7	7	2	7	7	7	34.4	25.476	0	83.98	2.607E+09	2009400	1.29E+09	161870000	1.15E+09	3081600	1.12E+08	113330000	43273000
P25788	P25788	Proteasome subunit alpha type-3	PSMA3	sp|P25788	11	11	11	0	10	8	11	0	10	8	11	0	10	8	11	45.9	28.433	0	34.394	2.605E+09	0	9.58E+08	27857000	1.62E+09	0	79523000	28396000	59977000
Q15637	Q15637	Splicing factor 1	SF1	sp|Q15637	12	12	12	1	12	3	10	1	12	3	10	1	12	3	10	30.2	68.329	0	121.67	2.589E+09	1466400	1.03E+09	12053000	1.54E+09	0	89794000	15795000	55118000
P61586	P61586	Transforming protein RhoA	RHOA	sp|P61586	6	6	3	0	6	3	6	0	6	3	6	0	3	2	3	45.1	21.768	0	38.134	2.585E+09	0	7.22E+08	9432300	1.85E+09	0	70360000	7508100	66796000
Q13185	Q13185	Chromobox protein homolog 3	CBX3	sp|Q13185	7	7	6	2	7	4	7	2	7	4	7	1	6	3	6	51.9	20.811	0	25.843	2.579E+09	59598000	9.3E+08	190040000	1.4E+09	70538000	90710000	61745000	83350000
P47756	P47756	F-actin-capping protein subunit beta	CAPZB	sp|P47756	14	14	14	1	13	9	14	1	13	9	14	1	13	9	14	55.2	31.35	0	71.775	2.577E+09	5241800	3.87E+08	103710000	2.08E+09	21671000	49773000	53820000	69384000
Q9HC38	Q9HC38	Glyoxalase domain-containing protein 4	GLOD4	sp|Q9HC38	10	10	10	0	9	1	9	0	9	1	9	0	9	1	9	47.6	34.793	0	110.68	2.575E+09	0	1.4E+09	1551100	1.17E+09	0	1.45E+08	2499500	14165000
P61019;Q8	P61019	Ras-related protein Rab-2A	RAB2A	sp|P61019	9	9	9	1	8	4	9	1	8	4	9	1	8	4	9	54.2	23.545	0	53.259	2.573E+09	13405000	4.14E+08	17141000	2.13E+09	11275000	38157000	8742700	82502000
O15260	O15260	Surfeit locus protein 4	SURF4	sp|O15260	5	5	5	0	5	5	5	0	5	5	5	0	5	5	5	22.7	30.394	0	57.934	2.573E+09	0	3.69E+08	78459000	2.13E+09	0	29954000	57890000	81319000
O75915	O75915	PRA1 family protein 3	ARL6IP5	sp|O75915	5	5	5	0	4	2	5	0	4	2	5	0	4	2	5	25	21.614	0	253.63	2.568E+09	0	3.64E+08	7246600	2.2E+09	0	36965000	8791600	81548000
Q12904	Q12904	Aminoacyl tRNA synthase complex-interacting mu	AIMP1	sp|Q12904	12	12	12	4	12	9	12	4	12	9	12	4	12	9	12	56.4	34.352	0	69.487	2.561E+09	1.25E+08	8.59E+08	117950000	1.46E+09	1.05E+08	73239000	108990000	46501000
O75165	O75165	DnaJ homolog subfamily C member 13	DNAJC13	sp|O75165	35	35	35	0	18	17	34	0	18	17	34	0	18	17	34	21.3	254.41	0	158.66	2.56E+09	0	1.3E+08	62783000	2.37E+09	0	15153000	71290000	64568000
O75534	O75534	Cold shock domain-containing protein E1	CSDE1	sp|O75534	25	25	25	1	16	18	25	1	16	18	25	1	16	18	25	37.2	88.884	0	201.39	2.559E+09	931090	4.07E+08	113410000	2.04E+09	2590800	45672000	31278000	1.11E+08
Q9UBE0	Q9UBE0	SUMO-activating enzyme subunit 1;SUMO-activat	SAE1	sp|Q9UBE	14	14	14	0	13	0	13	0	13	0	13	0	13	0	13	52.3	38.449	0	130.26	2.551E+09	0	9.64E+08	0	1.59E+09	0	1.03E+08	0	45264000
P04179	P04179	Superoxide dismutase [Mn], mitochondrial	SOD2	sp|P04179	7	7	7	0	5	1	7	0	5	1	7	0	5	1	7	38.7	24.75	0	41.176	2.546E+09	0	4.78E+08	3176000	2.07E+09	0	81695000	7781700	39707000

Q06323	Q06323	Proteasome activator complex subunit 1	PSME1	sp|Q06323	11	11	11	0	10	5	11	0	10	5	11	0	10	5	11	44.6	28.723	0	41.01	2.531E+09	0	6.28E+08	12174000	1.89E+09	0	56956000	12448000	68300000
Q53H12	Q53H12	Acylglycerol kinase, mitochondrial	AGK	sp|Q53H12	10	10	10	3	9	7	9	3	9	7	9	3	9	7	9	36	47.137	0	77.365	2.526E+09	44164000	1.07E+09	53886000	1.36E+09	64770000	85894000	27013000	52675000
O14617	O14617	AP-3 complex subunit delta-1	AP3D1	sp|O14617	24	24	24	1	20	12	22	1	20	12	22	1	20	12	22	27.5	130.16	0	175.82	2.524E+09	697500	5.1E+08	35095000	1.98E+09	0	43706000	39562000	66638000
P11766	P11766	Alcohol dehydrogenase class-3	ADH5	sp|P11766	8	8	8	0	8	1	7	0	8	1	7	0	8	1	7	22.7	39.724	0	35.578	2.522E+09	0	6.16E+08	743660	1.91E+09	0	16509000	1554500	1.15E+08
Q15942	Q15942	Zyxin	ZYX	sp|Q15942	13	13	13	0	7	4	13	0	7	4	13	0	7	4	13	39.5	61.277	0	213.15	2.511E+09	0	1.53E+08	7564400	2.35E+09	0	17340000	7207200	90675000
P62136	P62136	Serine/threonine-protein phosphatase PP1-alpha	PPP1CA	sp|P62136	12	12	6	4	12	7	12	4	12	7	12	2	6	4	6	46.4	37.512	0	66.508	2.508E+09	28390000	6.71E+08	47273000	1.76E+09	24666000	71433000	36073000	55280000
Q92614	Q92614	Unconventional myosin-XVIIIa	MYO18A	sp|Q92614	35	35	35	3	6	33	29	3	6	33	29	3	6	33	29	23.8	233.11	0	149.42	2.503E+09	1.34E+08	90304000	238590000	2.04E+09	65399000	6086500	230440000	81774000
P62280	P62280	40S ribosomal protein S11	RPS11	sp|P62280	10	10	10	5	10	9	9	5	10	9	9	5	10	9	9	55.7	18.431	0	31.868	2.503E+09	98376000	9.86E+08	201020000	1.22E+09	87724000	72764000	156210000	47483000
Q9Y6N5	Q9Y6N5	Sulfide:quinone oxidoreductase, mitochondrial	SQRDL	sp|Q9Y6N5	15	15	15	0	0	14	13	0	0	14	13	0	0	14	13	44.9	49.96	0	114.68	2.501E+09	0	0	149660000	2.35E+09	0	0	101980000	95307000
Q16851	Q16851	UTP--glucose-1-phosphate uridylyltransferase	UGP2	sp|Q16851	15	15	15	0	12	4	15	0	12	4	15	0	12	4	15	42.7	56.94	0	185.46	2.5E+09	0	2.14E+08	4474500	2.28E+09	0	19104000	8160700	88438000
Q9H0U4;Q	Q9H0U4	Ras-related protein Rab-1B	RAB1B	sp|Q9H0U4	10	4	4	2	10	7	10	1	4	3	4	1	4	3	4	66.2	22.171	0	70.059	2.498E+09	990600	5.61E+08	6399600	1.93E+09	997580	53671000	4292000	67867000
O75489	O75489	NADH dehydrogenase [ubiquinone] iron-sulfur pr	NDUFS3	sp|O75489	12	12	12	0	11	6	12	0	11	6	12	0	11	6	12	54.9	30.241	0	86.314	2.482E+09	0	1.1E+09	31770000	1.35E+09	0	89541000	30260000	54211000
Q9UMS4	Q9UMS4	Pre-mRNA-processing factor 19	PRPF19	sp|Q9UMS	12	12	12	3	11	8	11	3	11	8	11	3	11	8	11	34.5	55.18	0	80.065	2.471E+09	28904000	6.68E+08	112230000	1.66E+09	56162000	48184000	60795000	45522000
O00303	O00303	Eukaryotic translation initiation factor 3 subunit F	EIF3F	sp|O00303	13	13	13	9	11	7	12	9	11	7	12	9	11	7	12	49.6	37.563	0	97.538	2.47E+09	5.83E+08	3.82E+08	22041000	1.48E+09	5.42E+08	50533000	25151000	74883000
Q04446	Q04446	1,4-alpha-glucan-branching enzyme	GBE1	sp|Q04446	18	18	18	1	8	0	18	1	8	0	18	1	8	0	18	42.7	80.473	0	119.55	2.463E+09	60161000	94956000	0	2.31E+09	42854000	24133000	0	96440000
P55809;Q9	P55809	Succinyl-CoA:3-ketoacid coenzyme A transferase	OXCT1	sp|P55809	14	14	14	1	12	2	14	1	12	2	14	1	12	2	14	40.4	56.157	0	195.94	2.458E+09	1149000	8.69E+08	8209400	1.58E+09	3850600	50078000	9471900	82578000
P20340;Q1	P20340	Ras-related protein Rab-6A	RAB6A	sp|P20340	8	8	5	1	8	1	6	1	8	1	6	0	5	0	4	39.9	23.593	0	39.011	2.454E+09	2015500	7.71E+08	11455000	1.67E+09	4738800	97345000	4774400	28494000
Q13148	Q13148	TAR DNA-binding protein 43	TARDBP	sp|Q13148	9	9	9	3	9	6	9	3	9	6	9	3	9	6	9	35	44.739	0	59.83	2.45E+09	4143200	1.09E+09	23031000	1.33E+09	4057800	1.05E+08	11312000	48287000
P61011	P61011	Signal recognition particle 54 kDa protein	SRP54	sp|P61011	16	16	16	0	14	3	15	0	14	3	15	0	14	3	15	37.5	55.704	0	97.382	2.449E+09	0	8.11E+08	3302900	1.64E+09	0	74621000	1616400	64696000
P28331	P28331	NADH-ubiquinone oxidoreductase 75 kDa subunit	NDUFS1	sp|P28331	23	23	23	3	22	11	19	3	22	11	19	3	22	11	19	42.1	79.467	0	121.5	2.448E+09	2346200	1.19E+09	34711000	1.22E+09	2386500	99290000	14409000	64319000
O94979;Q9	O94979	Protein transport protein Sec31A	SEC31A	sp|O94979	24	24	24	0	18	1	24	0	18	1	24	0	18	1	24	26	133.01	0	113.89	2.436E+09	0	3.61E+08	1558400	2.07E+09	0	38041000	0	76441000
P36542	P36542	ATP synthase subunit gamma, mitochondrial	ATP5C1	sp|P36542	11	11	11	2	11	7	10	2	11	7	10	2	11	7	10	45	32.996	0	49.224	2.433E+09	4527900	1.01E+09	44750000	1.38E+09	6250900	95367000	21559000	56847000
P54578	P54578	Ubiquitin carboxyl-terminal hydrolase 14	USP14	sp|P54578	18	18	18	0	16	4	17	0	16	4	17	0	16	4	17	49.6	56.068	0	115.4	2.43E+09	0	5.63E+08	5703400	1.86E+09	0	49190000	5281500	75425000
P12236	P12236	ADP/ATP translocase 3;ADP/ATP translocase 3, N	SLC25A6	sp|P12236	12	5	3	6	12	10	9	2	5	3	2	1	3	3	2	38.9	32.866	0	21.116	2.428E+09	1.77E+08	1.23E+09	51180000	9.72E+08	1.72E+08	27172000	35126000	45355000
O60701	O60701	UDP-glucose 6-dehydrogenase	UGDH	sp|O60701	17	17	17	1	8	5	17	1	8	5	17	1	8	5	17	45.3	55.023	0	94.431	2.427E+09	1811600	87114000	5543100	2.33E+09	2151400	9062300	3452500	94479000
P30043	P30043	Flavin reductase (NADPH)	BLVRB	sp|P30043	8	8	8	0	6	1	8	0	6	1	8	0	6	1	8	48.1	22.119	0	41.491	2.424E+09	0	2.33E+08	812420	2.19E+09	0	23197000	634740	87307000
P21281;P1	P21281	V-type proton ATPase subunit B, brain isoform	ATP6V1B2	sp|P21281	16	16	16	0	13	4	15	0	13	4	15	0	13	4	15	52.1	56.5	0	146.87	2.418E+09	0	5.81E+08	4949100	1.83E+09	0	36686000	4522300	76613000
Q9BTT0	Q9BTT0	Acidic leucine-rich nuclear phosphoprotein 32 fan	ANP32E	sp|Q9BTT0	4	4	4	0	4	0	4	0	4	0	4	0	4	0	4	12.3	30.692	0	39.165	2.414E+09	0	8.98E+08	0	1.52E+09	0	79472000	0	62048000
P06753	P06753	Tropomyosin alpha-3 chain	TPM3	sp|P06753	16	7	7	5	14	13	16	4	6	6	7	4	6	6	7	43.2	32.95	0	23.57	2.414E+09	13351000	9.61E+08	100260000	1.34E+09	13486000	67473000	74893000	63111000
P11216	P11216	Glycogen phosphorylase, brain form	PYGB	sp|P11216	29	24	22	2	25	5	25	2	20	3	20	2	19	3	18	41.2	96.695	0	107.95	2.41E+09	10699000	4.55E+08	5663100	1.94E+09	6596600	48030000	1848700	77738000
O94905	O94905	Erlin-2	ERLIN2	sp|O94905	12	12	10	2	12	11	12	2	12	11	12	2	10	9	10	38.6	37.839	0	125.43	2.405E+09	9855300	7.95E+08	103420000	1.5E+09	26746000	78875000	61748000	43622000
P56134	P56134	ATP synthase subunit f, mitochondrial	ATP5J2	sp|P56134	3	3	3	1	3	3	2	1	3	3	2	1	3	3	2	39.4	10.918	0	17.508	2.404E+09	94141000	1.06E+09	44466000	1.21E+09	1.16E+08	84649000	26185000	38984000
P49207	P49207	60S ribosomal protein L34	RPL34	sp|P49207	7	7	7	1	7	7	6	1	7	7	6	1	7	7	6	37.6	13.293	0	15.002	2.397E+09	5774200	7.35E+08	357320000	1.3E+09	7234900	63127000	237310000	54327000
P38117	P38117	Electron transfer flavoprotein subunit beta	ETFB	sp|P38117	14	14	14	0	13	4	13	0	13	4	13	0	13	4	13	45.9	27.843	0	35.344	2.395E+09	0	1.19E+09	5369800	1.2E+09	0	1.07E+08	5304400	44869000
Q02218;Q9	Q02218	2-oxoglutarate dehydrogenase, mitochondrial	OGDH	sp|Q02218	30	30	30	6	20	12	30	6	20	12	30	6	20	12	30	40.4	115.93	0	145.49	2.393E+09	43406000	3.36E+08	23308000	1.99E+09	42378000	29673000	31659000	63432000
Q7L576	Q7L576	Cytoplasmic FMR1-interacting protein 1	CYFIP1	sp|Q7L576	25	25	14	2	18	3	23	2	18	3	23	2	10	1	12	25.5	145.18	0	106.44	2.39E+09	887840	3.06E+08	3828000	2.08E+09	1147900	22687000	1366300	90630000
P01834	P01834	Ig kappa chain C region	IGKC	sp|P01834	4	4	4	3	1	2	0	3	1	2	0	3	1	2	0	65.4	11.765	0	20.001	2.388E+09	2.35E+09	2341500	40306000	0	2.31E+09	596050	58163000	0
Q13409	Q13409	Cytoplasmic dynein 1 intermediate chain 2	DYNC1I2	sp|Q13409	16	16	16	2	6	10	16	2	6	10	16	2	6	10	16	35.1	71.456	0	220.68	2.388E+09	4813000	74837000	68457000	2.24E+09	11795000	8391500	46458000	82864000
Q8WVM8	Q8WVM8	Sec1 family domain-containing protein 1	SCFD1	sp|Q8WVM	16	16	16	1	13	2	15	1	13	2	15	1	13	2	15	39.7	72.379	0	239.93	2.376E+09	1192200	9.69E+08	27448000	1.38E+09	2968700	1.18E+08	11132000	30089000
Q8N163	Q8N163	Cell cycle and apoptosis regulator protein 2	CCAR2	sp|Q8N163	24	24	24	4	18	13	21	4	18	13	21	4	18	13	21	35.3	102.9	0	74.097	2.375E+09	34542000	6.12E+08	93706000	1.64E+09	36515000	45893000	78634000	56698000
P61254	P61254	60S ribosomal protein L26	RPL26	sp|P61254	11	11	3	5	9	8	9	5	9	8	9	1	2	1	2	47.6	17.258	0	24.3	2.37E+09	36913000	5.53E+08	343650000	1.44E+09	44899000	47827000	218040000	52278000
P39687;O9	P39687	Acidic leucine-rich nuclear phosphoprotein 32 fan	ANP32A	sp|P39687	9	9	7	0	9	3	8	0	9	3	8	0	7	3	6	40.2	28.585	0	65.423	2.368E+09	0	1.16E+09	6105900	1.2E+09	0	99521000	4746300	44041000
Q9P0L0	Q9P0L0	Vesicle-associated membrane protein-associated	VAPA	sp|Q9P0L0	13	13	12	1	12	12	13	1	12	12	13	1	11	11	12	55.4	27.893	0	69.528	2.359E+09	117750	5.39E+08	62749000	1.76E+09	272290	62583000	12841000	86329000
P05387	P05387	60S acidic ribosomal protein P2	RPLP2	sp|P05387	8	8	8	6	7	7	6	6	7	7	6	6	7	7	6	80.9	11.665	0	60.091	2.359E+09	5.99E+08	6.92E+08	298590000	7.69E+08	5.65E+08	63626000	219600000	40957000
Q15435	Q15435	Protein phosphatase 1 regulatory subunit 7	PPP1R7	sp|Q15435	16	16	16	2	14	0	16	2	14	0	16	2	14	0	16	66.9	41.564	0	167.15	2.358E+09	628160	5.33E+08	0	1.82E+09	0	54570000	0	67468000
Q06210	Q06210	Glutamine--fructose-6-phosphate aminotransfera	GFPT1	sp|Q06210	19	14	14	3	16	10	16	2	11	5	12	2	11	5	12	34.2	78.806	0	57.379	2.354E+09	2676900	6.78E+08	23390000	1.65E+09	4650900	36071000	15652000	89644000
Q14166	Q14166	Tubulin--tyrosine ligase-like protein 12	TTLL12	sp|Q14166	19	19	19	2	19	0	14	2	19	0	14	2	19	0	14	39.9	74.403	0	90.726	2.342E+09	1119800	1.41E+09	0	9.34E+08	1245300	1.21E+08	0	40449000
P84103	P84103	Serine/arginine-rich splicing factor 3	SRSF3	sp|P84103	7	7	6	3	7	6	5	3	7	6	5	3	6	5	4	39.6	19.329	0	37.606	2.338E+09	1.61E+08	1.23E+09	147250000	8.01E+08	1.84E+08	78193000	99407000	36409000
Q14195;Q1	Q14195	Dihydropyrimidinase-related protein 3	DPYSL3	sp|Q14195	16	13	11	3	11	3	16	3	8	1	13	3	6	1	11	43	61.963	0	78.444	2.334E+09	3.11E+08	2.16E+08	2026300	1.81E+09	1.39E+08	1.06E+08	8737400	1.5E+08
P07741	P07741	Adenine phosphoribosyltransferase	APRT	sp|P07741	8	8	8	0	8	1	8	0	8	1	8	0	8	1	8	66.7	19.608	0	79.716	2.333E+09	0	1.45E+09	472000	8.87E+08	0	1.28E+08	386980	35412000
P21399	P21399	Cytoplasmic aconitate hydratase	ACO1	sp|P21399	19	19	19	1	17	0	19	1	17	0	19	1	17	0	19	30.7	98.398	0	157.64	2.327E+09	28485000	4.55E+08	0	1.84E+09	28859000	32560000	0	79220000
P62318	P62318	Small nuclear ribonucleoprotein Sm D3	SNRPD3	sp|P62318	4	4	4	2	4	2	4	2	4	2	4	2	4	2	4	47.6	13.916	0	33.275	2.324E+09	54043000	1.37E+09	72261000	8.24E+08	39947000	1.14E+08	60591000	42242000
O75937	O75937	DnaJ homolog subfamily C member 8	DNAJC8	sp|O75937	9	9	9	1	9	2	7	1	9	2	7	1	9	2	7	45.1	29.841	0	63.773	2.324E+09	1554500	1.31E+09	3286500	1.01E+09	1072400	98861000	1334100	54190000
Q9UDY2	Q9UDY2	Tight junction protein ZO-2	TJP2	sp|Q9UDY	44	44	44	0	13	42	34	0	13	42	34	0	13	42	34	51.8	133.96	0	181.43	2.318E+09	0	57818000	990870000	1.27E+09	0	10964000	641550000	58463000
P24539	P24539	ATP synthase F(0) complex subunit B1, mitochonc	ATP5F1	sp|P24539	14	14	14	1	13	8	13	1	13	8	13	1	13	8	13	56.2	28.908	0	165.83	2.312E+09	10201000	1.24E+09	19973000	1.04E+09	5954400	96551000	13820000	58481000
Q15459	Q15459	Splicing factor 3A subunit 1	SF3A1	sp|Q15459	24	24	24	5	22	15	21	5	22	15	21	5	22	15	21	36.6	88.885	0	118.64	2.293E+09	55065000	1.04E+09	38561000	1.16E+09	28443000	1.23E+08	28656000	34472000
Q5T4S7	Q5T4S7	E3 ubiquitin-protein ligase UBR4	UBR4	sp|Q5T4S7	49	49	49	2	38	11	47	2	38	11	47	2	38	11	47	15.8	573.83	0	251.79	2.29E+09	4131800	5.47E+08	26732000	1.71E+09	6769000	39719000	24107000	70125000
Q9NY33	Q9NY33	Dipeptidyl peptidase 3	DPP3	sp|Q9NY33	18	18	18	0	14	1	18	0	14	1	18	0	14	1	18	40.4	82.588	0	221.88	2.286E+09	0	4.27E+08	2491900	1.86E+09	0	16404000	3366700	95895000
Q07866	Q07866	Kinesin light chain 1	KLC1	sp|Q07866	21	21	15	1	13	9	20	1	13	9	20	0	8	6	15	44	65.309	0	138.41	2.275E+09	485030	2.36E+08	24329000	2.01E+09	0	29617000	26162000	63905000
P25789	P25789	Proteasome subunit alpha type-4	PSMA4	sp|P25789	13	13	13	3	11	8	12	3	11	8	12	3	11	8	12	54.4	29.483	0	52.13	2.268E+09	21414000	7.38E+08	38681000	1.47E+09	21914000	77512000	24122000	46471000
Q9BR76;A9	Q9BR76	Coronin-1B	CORO1B	sp|Q9BR76	12	12	12	1	3	8	12	1	3	8	12	1	3	8	12	31.5	54.234	0	60.147	2.267E+09	1.07E+08	58782000	54394000	2.05E+09	83946000	15856000	40822000	91880000
O76094	O76094	Signal recognition particle subunit SRP72	SRP72	sp|O76094	20	20	20	3	18	7	19	3	18	7	19	3	18	7	19	41.3	74.605	0	126.58	2.266E+09	5344300	7.37E+08	18982000	1.51E+09	4208400	62958000	12101000	63021000
Q9Y5B9	Q9Y5B9	FACT complex subunit SPT16	SUPT16H	sp|Q9Y5B9	29	29	29	3	29	13	20	3	29	13	20	3	29	13	20	30.1	119.91	0	107.21	2.262E+09	9211500	1.67E+09	53075000	5.26E+08	11163000	1.38E+08	34717000	29326000
Q08J23	Q08J23	tRNA (cytosine(34)-C(5))-methyltransferase	NSUN2	sp|Q08J23	24	24	24	2	23	11	19	2	23	11	19	2	23	11	19	39	86.47	0	116.26	2.259E+09	23495000	1.06E+09	26344000	1.15E+09	5715700	1.23E+08	20304000	32114000
Q13418	Q13418	Integrin-linked protein kinase	ILK	sp|Q13418	11	11	11	0	9	6	11	0	9	6	11	0	9	6	11	26.8	51.419	0	42.889	2.258E+09	0	1.98E+08	31931000	2.03E+09	0	14992000	24250000	83045000
P48735	P48735	Isocitrate dehydrogenase [NADP], mitochondrial	IDH2	sp|P48735	17	17	16	0	17	3	15	0	17	3	15	0	16	2	14	35.2	50.909	0	155.27	2.251E+09	0	6.59E+08	4380700	1.59E+09	0	68218000	2381800	55705000
P09525	P09525	Annexin A4	ANXA4	sp|P09525	14	14	14	0	8	4	14	0	8	4	14	0	8	4	14	47.6	35.882	0	132.27	2.248E+09	0	1.34E+08	8920300	2.11E+09	0	11975000	15484000	76904000
P30085	P30085	UMP-CMP kinase	CMPK1	sp|P30085	10	10	10	0	10	1	10	0	10	1	10	0	10	1	10	61.7	22.222	0	81.916	2.244E+09	0	7.51E+08	731300	1.49E+09	0	24459000	2485600	1.01E+08
P46063	P46063	ATP-dependent DNA helicase Q1	RECQL	sp|P46063	20	20	20	0	15	12	19	0	15	12	19	0	15	12	19	40.5	73.457	0	120.86	2.237E+09	0	2.48E+08	48063000	1.94E+09	0	22969000	36994000	74048000
P30040	P30040	Endoplasmic reticulum resident protein 29	ERP29	sp|P30040	8	8	8	0	8	2	8	0	8	2	8	0	8	2	8	35.6	28.993	0	35.457	2.232E+09	0	8.25E+08	13191000	1.39E+09	0	69680000	11110000	57824000
P08559;P2	P08559	Pyruvate dehydrogenase E1 component subunit a	PDHA1	sp|P08559	17	17	17	4	17	4	14	4	17	4	14	4	17	4	14	42.1	43.295	0	65.594	2.225E+09	73350000	1.24E+09	4265800	9.08E+08	59842000	1.13E+08	5772100	44067000
P62316	P62316	Small nuclear ribonucleoprotein Sm D2	SNRPD2	sp|P62316	6	6	6	1	6	5	6	1	6	5	6	1	6	5	6	44.9	13.527	0	29.898	2.223E+09	1225200	1.19E+09	70257000	9.64E+08	1891300	1.09E+08	65844000	16475000
Q15907;P6	Q15907;P6	Ras-related protein Rab-11B;Ras-related protein I	RAB11B;RA	sp|Q15907	8	8	8	0	8	5	7	0	8	5	7	0	8	5	7	44	24.488	0	93.28	2.22E+09	0	7.74E+08	10189000	1.44E+09	0	61356000	8181700	64590000
Q9Y5S9	Q9Y5S9	RNA-binding protein 8A	RBM8A	sp|Q9Y5S9	6	6	6	1	6	5	6	1	6	5	6	1	6	5	6	55.2	19.889	0	68.65	2.214E+09	2987600	1.29E+09	42824000	8.83E+08	3916100	1.15E+08	15168000	46271000
P51114	P51114	Fragile X mental retardation syndrome-related pr	FXR1	sp|P51114	21	21	19	3	12	18	17	3	12	18	17	2	11	16	15	44.9	69.72	0	100.95	2.212E+09	3794200	1.66E+08	624820000	1.42E+09	3148000	9699200	423010000	51757000
Q96I99	Q96I99	Succinyl-CoA ligase [GDP-forming] subunit beta, n	SUCLG2	sp|Q96I99	18	18	18	0	14	3	17	0	14	3	17	0	14	3	17	41.4	46.51	0	84.371	2.209E+09	0	6.77E+08	2464400	1.53E+09	0	40852000	3018000	74583000
P51572	P51572	B-cell receptor-associated protein 31	BCAP31	sp|P51572	12	12	12	4	12	10	12	4	12	10	12	4	12	10	12	38.2	27.991	0	35.086	2.206E+09	16531000	1.11E+09	40873000	1.04E+09	21684000	93593000	21805000	47261000
P08237	P08237	ATP-dependent 6-phosphofructokinase, muscle ty	PFKM	sp|P08237	24	22	21	1	19	9	23	1	17	7	22	0	16	6	21	36.8	85.182	0	117.77	2.205E+09	3270800	5.68E+08	21150000	1.61E+09	7667700	52092000	10962000	63138000
Q99729	Q99729	Heterogeneous nuclear ribonucleoprotein A/B	HNRNPAB	sp|Q99729	11	11	11	2	11	10	10	2	11	10	10	2	11	10	10	26.2	36.224	0	50.36	2.197E+09	838710	1.06E+09	76193000	1.06E+09	1667400	94421000	45679000	46540000
Q92688	Q92688	Acidic leucine-rich nuclear phosphoprotein 32 fan	ANP32B	sp|Q92688	7	5	5	1	7	3	7	1	5	3	5	1	5	3	5	29.1	28.787	0	13.647	2.191E+09	272000	1.02E+09	4489400	1.17E+09	1148500	47674000	2262700	75945000
Q86W92;C	Q86W92	Liprin-beta-1	PPFIBP1	sp|Q86W9	25	25	25	0	1	20	24	0	1	20	24	0	1	20	24	31.8	114.02	0	123.95	2.189E+09	0	8231900	156550000	2.02E+09	0	3601200	45314000	1.4E+08
Q96FW1	Q96FW1	Ubiquitin thioesterase OTUB1	OTUB1	sp|Q96FW	10	10	10	0	7	4	9	0	7	4	9	0	7	4	9	50.6	31.284	0	66.583	2.188E+09	0	5.31E+08	6869100	1.65E+09	0	58876000	6532300	53893000
P0DP25;P0	P0DP25;P0DP24;P0DP23			sp|P0DP25	6	6	6	0	5	5	6	0	5	5	6	0	5	5	6	55.7	16.837	0	72.424	2.176E+09	0	3.81E+08	92779000	1.7E+09	0	29511000	52151000	72164000
Q92597	Q92597	Protein NDRG1	NDRG1	sp|Q92597	10	10	10	0	7	0	10	0	7	0	10	0	7	0	10	44.2	42.835	0	71.916	2.174E+09	0	3.4E+08	0	1.83E+09	0	40792000	0	64574000
P51659	P51659	Peroxisomal multifunctional enzyme type 2;(3R)-I	HSD17B4	sp|P51659	17	17	17	3	17	7	16	3	17	7	16	3	17	7	16	35.6	79.685	0	93.795	2.172E+09	10230000	9.57E+08	23131000	1.18E+09	10863000	75030000	23172000	46856000
Q15021;RE	Q15021	Condensin complex subunit 1	NCAPD2	sp|Q15021	29	29	29	0	21	11	29	0	21	11	29	0	21	11	29	29.4	157.18	0	197.87	2.171E+09	0	3.26E+08	22310000	1.82E+09	0	30448000	26252000	62015000
O14979	O14979	Heterogeneous nuclear ribonucleoprotein D-like	HNRNPDL	sp|O14979	8	7	7	2	8	8	8	1	7	7	7	1	7	7	7	17.1	46.437	0	59.463	2.164E+09	1065600	5.29E+08	112650000	1.52E+09	1290000	55636000	79829000	49206000
Q92974	Q92974	Rho guanine nucleotide exchange factor 2	ARHGEF2	sp|Q92974	23	23	23	6	14	23	21	6	14	23	21	6	14	23	21	31.3	111.54	0	137.45	2.159E+09	37724000	1.8E+08	294740000	1.65E+09	8144000	37703000	190980000	76337000
O14744	O14744	Protein arginine N-methyltransferase 5;Protein ar	PRMT5	sp|O14744	15	15	15	0	15	4	12	0	15	4	12	0	15	4	12	29.7	72.683	0	66.292	2.154E+09	0	9.84E+08	9579400	1.16E+09	0	76417000	14266000	50214000

P49721	P49721	Proteasome subunit beta type-2	PSMB2	sp|P49721	7	7	7	2	6	4	7	2	6	4	7	2	6	4	7	45.8	22.836	0	31.565	2.154E+09	2097300	7.19E+08	17125000	1.42E+09	4601000	56383000	13665000	48800000
Q9UKK9	Q9UKK9	ADP-sugar pyrophosphatase	NUDT5	sp|Q9UKK	10	10	10	0	9	4	8	0	9	4	8	0	9	4	8	51.6	24.327	0	45.767	2.154E+09	0	8.75E+08	5277400	1.27E+09	0	75663000	6891700	50506000
Q96CX2;Q(	Q96CX2	BTB/POZ domain-containing protein KCTD12	KCTD12	sp|Q96CX	14	14	14	6	9	14	12	6	9	14	12	6	9	14	12	51.4	35.7	0	104.59	2.153E+09	2.19E+08	1.83E+08	688230000	1.06E+09	3.68E+08	10425000	339440000	24142000
Q14847	Q14847	LIM and SH3 domain protein 1	LASP1	sp|Q14847	14	14	14	1	12	9	14	1	12	9	14	1	12	9	14	55.6	29.717	0	51.321	2.142E+09	135960	2.04E+08	21606000	1.92E+09	401630	13183000	5546500	87805000
Q6P5R6	Q6P5R6	60S ribosomal protein L22-like 1	RPL22L1	sp|Q6P5R	4	4	4	2	3	4	3	2	3	4	3	2	3	4	3	51.6	14.606	0	61.879	2.142E+09	23988000	2.23E+08	214110000	1.68E+09	51105000	35681000	133390000	36455000
P62879	P62879	Guanine nucleotide-binding protein G(I)/G(S)/G(T	GNB2	sp|P62879	9	9	1	1	6	6	9	1	6	6	9	0	0	0	1	31.2	37.331	0	123.33	2.134E+09	35069000	2.86E+08	30518000	1.78E+09	34743000	27573000	23895000	67853000
Q14108	Q14108	Lysosome membrane protein 2	SCARB2	sp|Q1410	10	10	10	0	7	3	9	0	7	3	9	0	7	3	9	29.7	54.29	0	86.075	2.129E+09	0	1.6E+08	1871000	1.97E+09	0	11825000	2270900	75410000
P35268	P35268	60S ribosomal protein L22	RPL22	sp|P35268	5	5	5	2	5	4	4	2	5	4	4	2	5	4	4	52.3	14.787	0	42.754	2.128E+09	75605000	9.47E+08	376820000	7.28E+08	60233000	82859000	240890000	58276000
O75822	O75822	Eukaryotic translation initiation factor 3 subunit J	EIF3J	sp|O7582	12	12	12	4	12	3	12	4	12	3	12	4	12	3	12	53.5	29.062	0	99.622	2.127E+09	1.71E+08	7.24E+08	12581000	1.22E+09	2.42E+08	29015000	5020300	17524000
P20042	P20042	Eukaryotic translation initiation factor 2 subunit 2	EIF2S2	sp|P20042	15	15	15	5	12	11	14	5	12	11	14	5	12	11	14	47.4	38.388	0	109.06	2.126E+09	1.31E+08	7.32E+08	88482000	1.17E+09	1.05E+08	71021000	86201000	39231000
P07858	P07858	Cathepsin B;Cathepsin B light chain;Cathepsin B h	CTSB	sp|P07858	8	8	8	0	1	3	8	0	1	3	8	0	1	3	8	38.9	37.821	0	56.805	2.124E+09	0	5459000	43851000	2.07E+09	0	741530	21495000	80258000
O15355	O15355	Protein phosphatase 1G	PPM1G	sp|O1535	17	17	17	1	17	6	14	1	17	6	14	1	17	6	14	45.8	59.271	0	112.53	2.117E+09	1370900	9.03E+08	7385500	1.21E+09	1392000	73626000	5615600	49013000
A6NJG6	A6NJG6	Arginine-fifty homeobox	ARGFX	sp|A6NJG	1	1	1	1	0	0	0	1	0	0	0	1	0	0	0	8.6	35.616	0.002446	2.2173	2.113E+09	2.11E+09	0	0	0	2.11E+09	0	0	0
O60488	O60488	Long-chain-fatty-acid--CoA ligase 4	ACSL4	sp|O6048	17	17	16	1	11	6	17	1	11	6	17	1	10	5	16	33.8	79.187	0	78.646	2.113E+09	940870	2.01E+08	33873000	1.88E+09	1915600	9095100	14305000	92591000
P09622	P09622	Dihydrolipoyl dehydrogenase, mitochondrial	DLD	sp|P09622	11	11	11	1	8	3	11	1	8	3	11	1	8	3	11	27.5	54.177	0	40.569	2.112E+09	695090	9.19E+08	5871000	1.19E+09	550480	86653000	2145700	45120000
P06132	P06132	Uroporphyrinogen decarboxylase	UROD	sp|P06132	9	9	9	0	8	1	9	0	8	1	9	0	8	1	9	45.5	40.786	0	80.297	2.109E+09	0	3.78E+08	518300	1.73E+09	0	39420000	399490	65048000
O00425	O00425	Insulin-like growth factor 2 mRNA-binding proteir	IGF2BP3	sp|O0042	17	15	15	6	12	14	14	4	10	12	12	4	10	12	12	35.6	63.704	0	76.337	2.101E+09	96931000	3.96E+08	201620000	1.41E+09	1.3E+08	31924000	102680000	44557000
P61758	P61758	Prefoldin subunit 3	VBP1	sp|P61758	10	10	10	1	10	1	10	1	10	1	10	1	10	1	10	49.2	22.626	0	60.734	2.1E+09	18227000	9.16E+08	805930	1.17E+09	11030000	1.05E+08	1924700	18419000
P13798	P13798	Acylamino-acid-releasing enzyme	APEH	sp|P13798	16	16	16	0	14	4	15	0	14	4	15	0	14	4	15	29.9	81.224	0	76.988	2.097E+09	0	5.5E+08	4286000	1.54E+09	0	42462000	7006900	63250000
Q15404	Q15404	Ras suppressor protein 1	RSU1	sp|Q1540	12	12	12	1	12	8	11	1	12	8	11	1	12	8	11	57.4	31.54	0	138.44	2.096E+09	671200	3.79E+08	25578000	1.69E+09	1042400	38683000	9888800	71177000
P11310	P11310	Medium-chain specific acyl-CoA dehydrogenase, r	ACADM	sp|P11310	17	17	17	1	17	3	15	1	17	3	15	1	17	3	15	47.5	46.588	0	147.2	2.09E+09	1004800	1.61E+09	2846500	4.73E+08	760000	1.35E+08	500630	18545000
P55735	P55735	Protein SEC13 homolog	SEC13	sp|P55735	2	2	2	0	2	2	2	0	2	2	2	0	2	2	2	11.5	35.54	0	46.722	2.086E+09	0	3.82E+08	22365000	1.68E+09	0	37470000	13822000	59010000
Q15257	Q15257	Serine/threonine-protein phosphatase 2A activat	PPP2R4	sp|Q1525	11	11	11	0	11	0	10	0	11	0	10	0	11	0	10	53.4	40.667	0	94.807	2.073E+09	0	7.78E+08	0	1.3E+09	0	67842000	0	53998000
Q9Y5L0	Q9Y5L0	Transportin-3	TNPO3	sp|Q9Y5L	14	14	14	0	11	5	14	0	11	5	14	0	11	5	14	23.8	104.2	0	79.831	2.073E+09	0	2.76E+08	9065100	1.79E+09	0	30085000	14197000	58616000
Q15738	Q15738	Sterol-4-alpha-carboxylate 3-dehydrogenase, dec	NSDHL	sp|Q1573	9	9	9	0	8	1	9	0	8	1	9	0	8	1	9	34.9	41.9	0	95.78	2.071E+09	0	6.22E+08	7805000	1.44E+09	0	40234000	10006000	62721000
Q96CS3	Q96CS3	FAS-associated factor 2	FAF2	sp|Q96CS	9	9	9	2	8	6	8	2	8	6	8	2	8	6	8	29.9	52.623	0	61.852	2.07E+09	1959200	5.77E+08	36119000	1.46E+09	6969700	48643000	33090000	47715000
O43809	O43809	Cleavage and polyadenylation specificity factor su	NUDT21	sp|O4380	11	11	11	5	11	6	9	5	11	6	9	5	11	6	9	57.7	26.227	0	79.728	2.069E+09	10984000	1.16E+09	35279000	8.59E+08	20011000	82676000	32885000	36999000
P12270	P12270	Nucleoprotein TPR	TPR	sp|P12270	41	41	41	4	35	20	33	4	35	20	33	4	35	20	33	24.4	267.29	0	186.42	2.062E+09	11293000	8.08E+08	42021000	1.2E+09	6898400	87204000	26979000	38004000
Q53GQ0	Q53GQ0	Very-long-chain 3-oxoacyl-CoA reductase	HSD17B12	sp|Q53GQ	10	10	10	2	10	5	10	2	10	5	10	2	10	5	10	49.7	34.324	0	43.626	2.059E+09	75122000	6.15E+08	30009000	1.34E+09	79658000	39143000	44650000	40996000
Q12907	Q12907	Vesicular integral-membrane protein VIP36	LMAN2	sp|Q1290	10	10	10	0	6	1	9	0	6	1	9	0	6	1	9	37.9	40.228	0	172	2.058E+09	0	3.98E+08	663720	1.66E+09	0	12360000	1211600	90189000
O15145	O15145	Actin-related protein 2/3 complex subunit 3	ARPC3	sp|O1514	8	8	8	1	5	3	8	1	5	3	8	1	5	3	8	53.4	20.546	0	30.599	2.052E+09	640990	2.1E+08	6204100	1.84E+09	1075000	12881000	3667400	81171000
P38919	P38919	Eukaryotic initiation factor 4A-III;Eukaryotic initiat	EIF4A3	sp|P38919	17	14	14	7	16	9	16	6	14	7	13	6	14	7	13	38.4	46.871	0	56.96	2.052E+09	2.18E+08	9.43E+08	20571000	8.7E+08	1.69E+08	1.01E+08	34734000	46130000
O43390	O43390	Heterogeneous nuclear ribonucleoprotein R	HNRNPR	sp|O4339	20	16	16	4	18	15	20	2	14	12	16	2	14	12	16	40.3	70.942	0	114.8	2.05E+09	1E+08	4.31E+08	131320000	1.39E+09	1.19E+08	33379000	85266000	45868000
O75828	O75828	Carbonyl reductase [NADPH] 3	CBR3	sp|O7582	10	8	7	4	6	3	8	2	4	1	6	2	4	1	6	48.7	30.85	0	92.807	2.042E+09	1.52E+09	1.62E+08	81796000	2.81E+08	3.58E+08	1.28E+08	1.052E+09	59884000
P47914	P47914	60S ribosomal protein L29	RPL29	sp|P47914	4	4	4	2	3	2	4	2	3	2	4	2	3	2	4	25.2	17.752	0	9.6257	2.036E+09	1.88E+08	6.91E+08	226570000	9.3E+08	1.12E+08	79709000	196370000	51915000
P07686	P07686	Beta-hexosaminidase subunit beta;Beta-hexosam	HEXB	sp|P07686	12	12	12	1	9	6	12	1	9	6	12	1	9	6	12	29.1	63.111	0	90.393	2.035E+09	6480500	4.2E+08	34093000	1.57E+09	9682200	22896000	29850000	68472000
Q9Y2L1	Q9Y2L1	Exosome complex exonuclease RRP44	DIS3	sp|Q9Y2L1	20	20	20	2	17	6	19	2	17	6	19	2	17	6	19	31.1	109	0	95.867	2.033E+09	2788000	7.51E+08	8453300	1.27E+09	6057700	46915000	8053100	65468000
P13073	P13073	Cytochrome c oxidase subunit 4 isoform 1, mitoch	COX4I1	sp|P13073	7	7	7	3	7	2	6	3	7	2	6	3	7	2	6	42.6	19.576	0	16.348	2.03E+09	22648000	1.2E+09	2763300	8.04E+08	15875000	1.06E+08	3838200	37753000
Q14739	Q14739	Lamin-B receptor	LBR	sp|Q1473	11	11	11	1	11	5	8	1	11	5	8	1	11	5	8	21.5	70.702	0	77.493	2.02E+09	3121500	1.71E+09	13657000	2.93E+08	9653900	1.16E+08	27519000	20081000
P31150	P31150	Rab GDP dissociation inhibitor alpha	GDI1	sp|P31150	20	12	12	1	20	3	19	0	12	0	11	0	12	0	11	56.8	50.582	0	241.15	2.018E+09	0	7.86E+08	0	1.23E+09	0	65720000	0	54199000
O96019	O96019	Actin-like protein 6A	ACTL6A	sp|O9601	9	9	9	1	8	6	7	1	8	6	7	1	8	6	7	34.5	47.46	0	99.055	2.01E+09	999470	3.35E+08	32728000	1.64E+09	5847800	51381000	22557000	31049000
P42224	P42224	Signal transducer and activator of transcription 1-	STAT1	sp|P42224	21	21	21	0	13	5	18	0	13	5	18	0	13	5	18	36.5	87.334	0	125.51	2.005E+09	0	1.68E+08	14052000	1.82E+09	0	13636000	11885000	72364000
P07099	P07099	Epoxide hydrolase 1	EPHX1	sp|P07099	11	11	11	0	8	1	10	0	8	1	10	0	8	1	10	26.2	52.948	0	25.738	2.001E+09	0	3.16E+08	643350	1.68E+09	0	26128000	4534200	66861000
O00151	O00151	PDZ and LIM domain protein 1	PDLIM1	sp|O00151	12	12	12	1	10	4	11	1	10	4	11	1	10	4	11	57.1	36.071	0	109.34	1.997E+09	6589700	6.92E+08	12260000	1.29E+09	11279000	77944000	6379900	32882000
Q15819	Q15819	Ubiquitin-conjugating enzyme E2 variant 2	UBE2V2	sp|Q1581	8	8	3	1	8	2	7	1	8	2	7	0	3	0	2	64.1	16.363	0	39.852	1.996E+09	161100	9.74E+08	11418000	1.01E+09	533000	1.13E+08	3347300	16592000
P43246	P43246	DNA mismatch repair protein Msh2	MSH2	sp|P43246	20	20	20	2	19	6	20	2	19	6	20	2	19	6	20	32.9	104.74	0	95.024	1.992E+09	1738000	3.18E+08	17990000	1.65E+09	3931900	15696000	18543000	71722000
P10768	P10768	S-formylglutathione hydrolase	ESD	sp|P10768	6	6	6	0	6	2	6	0	6	2	6	0	6	2	6	38.7	31.462	0	51.815	1.984E+09	0	8.33E+08	1909100	1.15E+09	0	48035000	2952800	67417000
P61201	P61201	COP9 signalosome complex subunit 2	COPS2	sp|P61201	15	15	15	0	11	3	15	0	11	3	15	0	11	3	15	37.2	51.596	0	97.268	1.978E+09	0	4.31E+08	4593700	1.54E+09	0	50660000	2932300	50905000
P16403	P16403	Histone H1.2	HIST1H1C	sp|P16403	10	8	2	4	10	8	10	3	8	7	8	0	2	1	2	36.6	21.364	0	21.078	1.97E+09	75469000	5.13E+08	275130000	1.11E+09	1.07E+08	39010000	167910000	36779000
P61026	P61026	Ras-related protein Rab-10	RAB10	sp|P61026	10	9	8	0	8	4	9	0	7	3	8	0	6	2	7	45	22.541	0	31.378	1.961E+09	0	3.64E+08	6196200	1.59E+09	0	41310000	5414200	54913000
Q13151	Q13151	Heterogeneous nuclear ribonucleoprotein A0	HNRNPA0	sp|Q1315	8	8	8	3	7	7	8	3	7	7	8	3	7	7	8	32.5	30.84	0	85.29	1.95E+09	4.06E+08	5.01E+08	121930000	9.21E+08	4.18E+08	37715000	74097000	29381000
Q15185	Q15185	Prostaglandin E synthase 3	PTGES3	sp|Q1518	6	6	6	1	5	1	5	1	5	1	5	1	5	1	5	38.8	18.697	0	29.058	1.949E+09	1006300	7.29E+08	1982800	1.22E+09	1510000	81005000	898260	26408000
P36405	P36405	ADP-ribosylation factor-like protein 3	ARL3	sp|P36405	8	8	8	0	8	1	7	0	8	1	7	0	8	1	7	57.7	20.455	0	78.601	1.948E+09	0	7.61E+08	1043700	1.19E+09	0	1.01E+08	1929800	13273000
P10644	P10644	cAMP-dependent protein kinase type I-alpha regu	PRKAR1A	sp|P10644	13	13	10	0	10	7	12	0	10	7	12	0	9	7	9	37	42.981	0	56.365	1.94E+09	0	1.75E+08	34762000	1.73E+09	0	13880000	25593000	69242000
P63220	P63220	40S ribosomal protein S21	RPS21	sp|P63220	5	5	5	4	5	5	5	4	5	5	5	4	5	5	5	53	9.1113	0	89.994	1.94E+09	89240000	7.97E+08	53768000	1E+09	90521000	59925000	38880000	43522000
Q9UKY7	Q9UKY7	Protein CDV3 homolog	CDV3	sp|Q9UKY	12	12	12	1	12	5	12	1	12	5	12	1	12	5	12	72.1	27.335	0	92.125	1.939E+09	1920700	5.37E+08	7565200	1.39E+09	4318100	64085000	5678700	37505000
Q9NVA2	Q9NVA2	Septin-11	Sep-11	sp|Q9NVA	14	14	9	3	13	6	14	3	13	6	14	2	8	2	9	35.7	49.398	0	68.585	1.934E+09	5212100	5.93E+08	18772000	1.32E+09	7516700	56124000	13460000	47156000
P08243	P08243	Asparagine synthetase [glutamine-hydrolyzing]	ASNS	sp|P08243	15	15	15	0	14	2	12	0	14	2	12	0	14	2	12	36.5	64.369	0	80.067	1.932E+09	0	1.06E+09	3237300	8.72E+08	0	92491000	3481200	35236000
P53992	P53992	Protein transport protein Sec24C	SEC24C	sp|P53992	18	18	18	1	16	1	18	1	16	1	18	1	16	1	18	22.3	118.32	0	73.711	1.932E+09	365560	3.48E+08	191510	1.58E+09	512410	4825800	304030	90641000
Q9NTJ3	Q9NTJ3	Structural maintenance of chromosomes protein	SMC4	sp|Q9NTJ	34	34	34	4	23	23	30	4	23	23	30	4	23	23	30	34.4	147.18	0	226.67	1.932E+09	4457300	3.29E+08	56782000	1.54E+09	3348800	36758000	45891000	49023000
Q6FI81	Q6FI81	Anamorsin	CIAPIN1	sp|Q6FI81	8	8	8	0	8	1	8	0	8	1	8	0	8	1	8	34.3	33.582	0	64.203	1.928E+09	0	9.87E+08	199310000	7.42E+08	0	86647000	135600000	29176000
O43776	O43776	Asparagine--tRNA ligase, cytoplasmic	NARS	sp|O4377	15	15	15	0	14	5	11	0	14	5	11	0	14	5	11	31.6	62.942	0	62.774	1.925E+09	0	7.59E+08	8883600	1.16E+09	0	71781000	9359200	39296000
O00487	O00487	26S proteasome non-ATPase regulatory subunit 1	PSMD14	sp|O0048	10	10	10	1	9	6	8	1	9	6	8	1	9	6	8	52.3	34.577	0	45.296	1.924E+09	4664500	8.47E+08	24362000	1.05E+09	6819700	63532000	17572000	50862000
Q13557	Q13557	Calcium/calmodulin-dependent protein kinase tyı	CAMK2D	sp|Q1355	9	9	4	1	6	6	9	1	6	6	9	0	3	2	4	30.3	56.369	0	140.19	1.917E+09	428960	1.09E+08	61738000	1.75E+09	2687400	9486900	14478000	90205000
P49023	P49023	Paxillin	PXN	sp|P49023	10	10	10	1	1	3	10	1	1	3	10	1	1	3	10	29.6	64.505	0	99.931	1.915E+09	16358000	31516000	13826000	1.85E+09	7741400	3190200	7018100	86689000
Q9H845	Q9H845	Acyl-CoA dehydrogenase family member 9, mitoc	ACAD9	sp|Q9H84	17	17	17	1	12	7	16	1	12	7	16	1	12	7	16	34	68.76	0	79.412	1.915E+09	3.99E+08	2.81E+08	7266000	1.23E+09	2.51E+08	77545000	31498000	1.19E+08
O96008	O96008	Mitochondrial import receptor subunit TOM40 hc	TOMM40	sp|O9600	6	6	6	0	6	0	5	0	6	0	5	0	6	0	5	26	37.893	0	84.533	1.914E+09	0	8.3E+08	0	1.08E+09	0	64959000	0	46986000
Q02809	Q02809	Procollagen-lysine,2-oxoglutarate 5-dioxygenase	PLOD1	sp|Q0280	11	11	11	3	6	6	11	3	6	6	11	3	6	6	11	22.1	83.549	0	46.596	1.909E+09	1.13E+09	51826000	22057000	7.04E+08	8.65E+08	47502000	180990000	85838000
Q9H2G2	Q9H2G2	STE20-like serine/threonine-protein kinase	SLK	sp|Q9H2G	25	25	24	2	23	5	21	2	23	5	21	1	22	5	20	35.4	142.69	0	125.84	1.897E+09	3850200	6.35E+08	2823600	1.25E+09	555870	30189000	685270	81907000
P54727	P54727	UV excision repair protein RAD23 homolog B	RAD23B	sp|P54727	10	10	9	1	10	3	10	1	10	3	10	1	9	2	9	42.1	43.171	0	121.66	1.892E+09	337530	7.57E+08	2826200	1.13E+09	921050	86313000	1900600	23562000
Q15813	Q15813	Tubulin-specific chaperone E	TBCE	sp|Q1581	15	15	14	0	15	0	11	0	15	0	11	0	14	0	11	39.1	59.345	0	118.27	1.89E+09	0	6.98E+08	0	1.19E+09	0	68941000	0	41340000
P49419	P49419	Alpha-aminoadipic semialdehyde dehydrogenase	ALDH7A1	sp|P49419	15	15	15	1	15	1	9	1	15	1	9	1	15	1	9	36.2	58.486	0	52.59	1.889E+09	1736500	1.34E+09	788950	5.42E+08	1586800	1.24E+08	565660	5305400
P23588	P23588	Eukaryotic translation initiation factor 4B	EIF4B	sp|P23588	16	16	16	4	14	10	16	4	14	10	16	4	14	10	16	33.9	69.15	0	216.14	1.883E+09	80879000	3.47E+08	37756000	1.42E+09	88717000	30628000	24478000	51318000
P48556	P48556	26S proteasome non-ATPase regulatory subunit 8	PSMD8	sp|P48556	13	13	13	2	10	7	13	2	10	7	13	2	10	7	13	39.7	39.611	0	56.996	1.882E+09	1618400	6.18E+08	16631000	1.25E+09	1675300	55900000	9892700	50870000
Q13620	Q13620	Cullin-4B	CUL4B	sp|Q1362	24	24	17	0	21	2	23	0	21	2	23	0	15	0	16	34	103.98	0	143.29	1.882E+09	0	5.07E+08	2562800	1.37E+09	0	40252000	3027800	55368000
P52789;P5	P52789	Hexokinase-2	HK2	sp|P52789	22	18	18	1	16	13	21	1	12	9	17	1	12	9	17	28.6	102.38	0	117.55	1.881E+09	173720	1.68E+08	25300000	1.69E+09	2407400	45757000	17416000	35819000
O43681	O43681	ATPase ASNA1	ASNA1	sp|O43681	8	8	8	0	8	2	8	0	8	2	8	0	8	2	8	33	38.792	0	68.414	1.872E+09	0	3.89E+08	3511400	1.48E+09	0	25374000	3857800	68257000
Q12849	Q12849	G-rich sequence factor 1	GRSF1	sp|Q1284	11	11	11	0	10	8	10	0	10	8	10	0	10	8	10	38.1	53.126	0	89.325	1.857E+09	0	4.05E+08	120140000	1.33E+09	0	33831000	96587000	40794000
Q15758	Q15758	Neutral amino acid transporter B(0)	SLC1A5	sp|Q1575	7	7	7	0	7	1	7	0	7	1	7	0	7	1	7	15.2	56.598	0	33.555	1.837E+09	0	8.23E+08	2347900	1.01E+09	0	65826000	2286600	47603000
P13861	P13861	cAMP-dependent protein kinase type II-alpha reg	PRKAR2A	sp|P13861	18	18	17	2	18	5	16	2	18	5	16	2	17	5	15	55.7	45.518	0	101.53	1.83E+09	718480	6.78E+08	9775800	1.14E+09	624950	63153000	3419400	46795000
Q05193	Q05193	Dynamin-1	DNM1	sp|Q0519	23	17	15	0	8	4	23	0	4	1	17	0	3	1	15	41.6	97.407	0	130.96	1.824E+09	0	10526000	807900	1.81E+09	0	377640	288870	75330000
Q9BZZ5	Q9BZZ5	Apoptosis inhibitor 5	API5	sp|Q9BZZ	12	12	12	1	12	1	10	1	12	1	10	1	12	1	10	24.6	59.004	0	75.215	1.818E+09	464630	7.74E+08	5740400	1.04E+09	444260	74868000	2587400	37337000
P53004	P53004	Biliverdin reductase A	BLVRA	sp|P53004	10	10	10	1	9	0	9	1	9	0	9	1	9	0	9	41.2	33.428	0	52.109	1.816E+09	327490	5E+08	0	1.32E+09	1149400	14218000	0	83133000
Q6P2E9	Q6P2E9	Enhancer of mRNA-decapping protein 4	EDC4	sp|Q6P2E	23	23	23	1	23	6	18	1	23	6	18	1	23	6	18	28.8	151.66	0	182.16	1.815E+09	255310	9.02E+08	19867000	8.92E+08	1066300	1.01E+08	8025100	19419000
P08195	P08195	4F2 cell-surface antigen heavy chain	SLC3A2	sp|P08195	16	16	16	0	16	3	10	0	16	3	10	0	16	3	10	36	67.993	0	98.504	1.812E+09	0	1.14E+09	4977200	6.68E+08	0	1.03E+08	3114000	25423000
Q9Y295	Q9Y295	Developmentally-regulated GTP-binding protein 1	DRG1	sp|Q9Y29	12	12	12	2	9	7	12	2	9	7	12	2	9	7	12	40.3	40.542	0	92.385	1.812E+09	15817000	4.6E+08	53760000	1.28E+09	28577000	39244000	36142000	41264000
Q15785	Q15785	Mitochondrial import receptor subunit TOM34	TOMM34	sp|Q1578	16	16	16	0	11	1	16	0	11	1	16	0	11	1	16	59.5	34.559	0	78.546	1.807E+09	0	3.17E+08	661230	1.49E+09	0	29818000	540780	56774000
P00966	P00966	Argininosuccinate synthase	ASS1	sp|P00966	8	8	8	0	8	1	8	0	8	1	8	0	8	1	8	28.2	46.53	0	159.49	1.803E+09	0	1.99E+08	493980	1.6E+09	0	4440500	637730	78752000
Q7Z406	Q7Z406	Myosin-14	MYH14	sp|Q7Z40	13	6	6	6	10	7	8	4	4	1	1	4	4	1	1	6.5	227.87	0	18.259	1.801E+09	83563000	1.38E+08	68762000	1.51E+09	3383600	7865300	82254000	1.11E+08

O95347	O95347	Structural maintenance of chromosomes protein	SMC2	sp|O95347	28	28	28	3	23	13	25	3	23	13	25	3	23	13	25	29.3	135.65	0	77.632	1.8E+09	5061000	4.21E+08	31397000	1.34E+09	2788600	38842000	33705000	41018000
Q12874	Q12874	Splicing factor 3A subunit 3	SF3A3	sp|Q12874	17	17	17	3	17	6	14	3	17	6	14	3	17	6	14	43.1	58.848	0	131.85	1.8E+09	11939000	9.39E+08	20626000	8.28E+08	9109700	75421000	26270000	31846000
P31153;Q(	P31153	S-adenosylmethionine synthase isoform type-2	MAT2A	sp|P31153	10	10	10	2	9	7	10	2	9	7	10	2	9	7	10	28.1	43.66	0	40.621	1.792E+09	3509000	3.94E+08	17659000	1.38E+09	8901200	45522000	10674000	41635000
Q9UBQ7	Q9UBQ7	Glyoxylate reductase/hydroxypyruvate reductase	GRHPR	sp|Q9UBQ	8	8	8	0	7	0	8	0	7	0	8	0	7	0	8	37.5	35.668	0	87.018	1.783E+09	0	8.1E+08	0	9.73E+08	0	66698000	0	44740000
Q9NR45	Q9NR45	Sialic acid synthase	NANS	sp|Q9NR4	11	11	11	0	9	1	8	0	9	1	8	0	9	1	8	44	40.307	0	69.3	1.781E+09	0	1.13E+09	1402500	6.54E+08	0	1E+08	1199100	24062000
O75153	O75153	Clustered mitochondria protein homolog	CLUH	sp|O7515	20	20	20	1	16	0	18	1	16	0	18	1	16	0	18	20.1	146.67	0	50.268	1.781E+09	1.09E+09	1.9E+08	0	5.04E+08	9.91E+08	1.01E+08	0	31142000
Q9UQ35	Q9UQ35	Serine/arginine repetitive matrix protein 2	SRRM2	sp|Q9UQ3	25	25	25	16	17	13	12	16	17	13	12	16	17	13	12	14.1	299.61	0	130.63	1.778E+09	1.09E+09	3.6E+08	158150000	1.69E+08	7.18E+08	87891000	409130000	20611000
Q02978	Q02978	Mitochondrial 2-oxoglutarate/malate carrier prot	SLC25A11	sp|Q0297	15	15	15	2	14	8	12	2	14	8	12	2	14	8	12	51.6	34.061	0	69.592	1.777E+09	23763000	6.26E+08	48002000	1.08E+09	23197000	48018000	38154000	44227000
Q9ULC4;A(	Q9ULC4;A(	Malignant T-cell-amplified sequence 1	MCTS1	sp|Q9ULC	7	7	7	0	7	1	7	0	7	1	7	0	7	1	7	56.9	20.555	0	36.011	1.775E+09	0	6.77E+08	913200	1.1E+09	0	58180000	635200	44522000
Q7L2E3	Q7L2E3	Putative ATP-dependent RNA helicase DHX30	DHX30	sp|Q7L2E3	27	27	27	5	20	22	22	5	20	22	22	5	20	22	22	32.4	133.94	0	140.59	1.773E+09	68267000	4.25E+08	251880000	1.03E+09	32788000	42852000	183810000	57805000
O75746	O75746	Calcium-binding mitochondrial carrier protein Ara	SLC25A12	sp|O7574	22	22	17	0	15	16	22	0	15	16	22	0	10	11	17	41.4	74.761	0	119.69	1.77E+09	0	2.11E+08	51609000	1.51E+09	0	19139000	53583000	42528000
Q07955	Q07955	Serine/arginine-rich splicing factor 1	SRSF1	sp|Q0795	12	12	12	1	12	11	12	1	12	11	12	1	12	11	12	44.8	27.744	0	36.802	1.768E+09	1119300	9.13E+08	94681000	7.59E+08	1811400	91312000	69880000	13234000
P23921	P23921	Ribonucleoside-diphosphate reductase large subu	RRM1	sp|P23921	20	20	20	1	17	2	20	1	17	2	20	1	17	2	20	34.8	90.069	0	106.51	1.75E+09	1.32E+08	4.36E+08	1855700	1.18E+09	87448000	75409000	2940200	54199000
O43837	O43837	Isocitrate dehydrogenase [NAD] subunit beta, mit	IDH3B	sp|O43837	12	12	12	0	8	3	11	0	8	3	11	0	8	3	11	41	42.183	0	72.276	1.75E+09	0	6.43E+08	8506300	1.1E+09	0	49590000	6884200	51082000
O94826	O94826	Mitochondrial import receptor subunit TOM70	TOMM70A	sp|O9482	13	13	13	1	12	5	13	1	12	5	13	1	12	5	13	35.4	67.454	0	97.636	1.737E+09	389310	4.74E+08	8797300	1.25E+09	499640	41849000	4084900	53239000
P06493	P06493	Cyclin-dependent kinase 1	CDK1	sp|P06493	13	13	12	1	12	9	8	1	12	9	8	1	11	8	7	55.9	34.095	0	64.053	1.724E+09	45414000	1.06E+09	34794000	5.83E+08	49706000	84641000	26043000	25893000
Q96KP4	Q96KP4	Cytosolic non-specific dipeptidase	CNDP2	sp|Q96KP	15	15	15	0	14	1	14	0	14	1	14	0	14	1	14	42.1	52.878	0	71.183	1.721E+09	0	5.62E+08	154650	1.16E+09	0	82992000	833090	13470000
P30837	P30837	Aldehyde dehydrogenase X, mitochondrial	ALDH1B1	sp|P30837	13	12	12	0	13	0	11	0	12	0	10	0	12	0	10	36.8	57.206	0	95.626	1.716E+09	0	9.86E+08	0	7.3E+08	0	81669000	0	28050000
P98179	P98179	RNA-binding protein 3	RBM3	sp|P98179	5	5	5	0	5	3	5	0	5	3	5	0	5	3	5	52.2	17.17	0	124.8	1.708E+09	0	4.8E+08	14405000	1.21E+09	0	39658000	22212000	40021000
P40222	P40222	Alpha-taxilin	TXLNA	sp|P40222	19	19	19	1	18	9	15	1	18	9	15	1	18	9	15	46.7	61.89	0	96.188	1.707E+09	808910	7.65E+08	27277000	9.13E+08	4578900	38401000	21810000	59372000
Q8TCT9	Q8TCT9	Minor histocompatibility antigen H13	HM13	sp|Q8TCT	8	8	8	1	7	2	8	1	7	2	8	1	7	2	8	24.1	41.488	0	118.84	1.703E+09	394460	3.35E+08	8647300	1.36E+09	510620	25471000	7335900	58300000
Q9Y281	Q9Y281	Cofilin-2	CFL2	sp|Q9Y281	9	4	4	3	9	9	8	0	4	4	4	0	4	4	4	56.6	18.736	0	52.946	1.703E+09	0	5.3E+08	133560000	1.04E+09	0	35782000	77202000	38439000
P04632;Q9	P04632	Calpain small subunit 1	CAPNS1	sp|P04632	8	8	8	1	4	2	8	1	4	2	8	1	4	2	8	63.4	28.315	0	124.95	1.701E+09	1371500	32141000	3135800	1.66E+09	2665100	2345200	1164800	68600000
P27361;P3	P27361	Mitogen-activated protein kinase 3	MAPK3	sp|P27361	13	13	10	4	12	1	13	4	12	1	13	2	9	0	10	48.3	43.135	0	46.359	1.699E+09	27807000	4.37E+08	3806000	1.23E+09	39461000	29510000	3142600	47332000
P60891;P2	P60891	Ribose-phosphate pyrophosphokinase 1	PRPS1	sp|P60891	11	11	5	2	10	4	8	2	10	4	8	2	5	2	3	44.3	34.834	0	37.743	1.699E+09	3253600	9.78E+08	9494100	7.08E+08	4022300	89823000	7437300	22591000
P55145	P55145	Mesencephalic astrocyte-derived neurotrophic fa	MANF	sp|P55145	9	9	9	0	9	2	9	0	9	2	9	0	9	2	9	41.2	20.7	0	40.179	1.698E+09	0	6.62E+08	1262700	1.03E+09	0	74993000	995210	24962000
O75436	O75436	Vacuolar protein sorting-associated protein 26A	VPS26A	sp|O7543	12	12	11	0	12	4	12	0	12	4	12	0	11	4	11	47.1	38.169	0	50.962	1.694E+09	0	3.51E+08	7996300	1.33E+09	0	28963000	7912600	54340000
P06730;A6	P06730	Eukaryotic translation initiation factor 4E	EIF4E	sp|P06730	8	8	8	2	8	5	8	2	8	5	8	2	8	5	8	41.9	25.097	0	27.952	1.683E+09	7605000	7.41E+08	43580000	8.91E+08	15027000	58760000	25475000	39551000
P47985;P0	P47985;P0	Cytochrome b-c1 complex subunit Rieske, mitoch	UQCRFS1;I	sp|P47985	7	7	7	0	7	4	7	0	7	4	7	0	7	4	7	30.3	29.668	0	30.813	1.667E+09	0	6.41E+08	20290000	1.01E+09	0	52860000	18217000	40415000
Q9H3U1	Q9H3U1	Protein unc-45 homolog A	UNC45A	sp|Q9H3U	17	17	17	1	16	14	15	1	16	14	15	1	16	14	15	22.4	103.08	0	58.197	1.657E+09	1408700	4.58E+08	51555000	1.15E+09	4908300	23187000	26885000	68570000
P30520;Q8	P30520	Adenylosuccinate synthetase isozyme 2	ADSS	sp|P30520	15	15	15	1	12	1	14	1	12	1	14	1	12	1	14	39.3	50.097	0	72.18	1.655E+09	660920	5.94E+08	899070	1.06E+09	1062300	33807000	493580	61448000
P62910	P62910	60S ribosomal protein L32	RPL32	sp|P62910	8	8	8	2	7	7	8	2	7	7	8	2	7	7	8	40.7	15.86	0	25.536	1.652E+09	15403000	3.74E+08	168990000	1.09E+09	32083000	40824000	92362000	41262000
P56537	P56537	Eukaryotic translation initiation factor 6	EIF6	sp|P56537	5	5	5	1	4	3	5	1	4	3	5	1	4	3	5	35.1	26.599	0	99.492	1.649E+09	3141800	2.35E+08	25351000	1.39E+09	8936900	11332000	15095000	62512000
Q14498;Q8	Q14498	RNA-binding protein 39	RBM39	sp|Q1449	13	13	13	5	10	12	12	5	10	12	12	5	10	12	12	30.6	59.379	0	90.027	1.645E+09	1.56E+08	4.38E+08	134100000	9.17E+08	2.03E+08	26080000	67516000	24348000
P68366	P68366	Tubulin alpha-4A chain	TUBA4A	sp|P68366	24	4	4	6	20	21	23	0	0	4	3	0	0	4	3	58.3	49.924	0	31.775	1.64E+09	0	0	50742000	1.59E+09	0	0	41609000	57835000
Q14157	Q14157	Ubiquitin-associated protein 2-like	UBAP2L	sp|Q1415	18	18	18	0	18	9	17	0	18	9	17	0	18	9	17	30.9	114.53	0	79.765	1.633E+09	0	6.27E+08	30243000	9.76E+08	0	48301000	29407000	34823000
P31040	P31040	Succinate dehydrogenase [ubiquinone] flavoprote	SDHA	sp|P31040	14	14	14	0	11	0	11	0	11	0	11	0	11	0	11	33.1	72.691	0	95.742	1.631E+09	0	6.35E+08	0	9.97E+08	0	55491000	0	39863000
P51571	P51571	Translocon-associated protein subunit delta	SSR4	sp|P51571	5	5	5	0	5	4	5	0	5	4	5	0	5	4	5	36.4	18.998	0	27.661	1.63E+09	0	6.63E+08	10605000	9.56E+08	0	45802000	16804000	42318000
O75347	O75347	Tubulin-specific chaperone A	TBCA	sp|O75347	8	8	8	1	8	0	8	1	8	0	8	1	8	0	8	54.6	12.855	0	57.039	1.629E+09	465230	8.23E+08	0	8.05E+08	0	67387000	0	37941000
P30419	P30419	Glycylpeptide N-tetradecanoyltransferase 1	NMT1	sp|P30419	10	10	8	0	10	6	9	0	10	6	9	0	8	5	7	31.5	56.806	0	107.53	1.623E+09	0	5.91E+08	11432000	1.02E+09	0	52774000	13814000	35154000
Q9HAV4	Q9HAV4	Exportin-5	XPO5	sp|Q9HAV	19	19	19	0	19	1	15	0	19	1	15	0	19	1	15	22	136.31	0	77.835	1.623E+09	0	1.08E+09	1258800	5.39E+08	0	1.06E+08	1886300	10369000
P35237	P35237	Serpin B6	SERPINB6	sp|P35237	12	12	11	0	11	1	12	0	11	1	12	0	10	1	11	47.6	42.621	0	64.772	1.618E+09	0	4.84E+08	2935700	1.13E+09	0	42946000	2290400	45906000
P42166	P42166	Lamina-associated polypeptide 2, isoform alpha;T	TMPO	sp|P42166	18	18	18	2	17	15	14	2	17	15	14	2	17	15	14	43.4	75.491	0	142.99	1.617E+09	5064900	9.32E+08	98571000	5.82E+08	4548000	77365000	78675000	16722000
O43615	O43615	Mitochondrial import inner membrane translocas	TIMM44	sp|O4361	14	14	14	0	12	8	12	0	12	8	12	0	12	8	12	35.8	51.355	0	75.167	1.615E+09	0	4.56E+08	57485000	1.1E+09	0	48899000	47374000	27868000
O15460	O15460	Prolyl 4-hydroxylase subunit alpha-2	P4HA2	sp|O1546	16	16	16	1	8	4	16	1	8	4	16	1	8	4	16	43	60.901	0	116.65	1.614E+09	22605000	65366000	9070500	1.52E+09	12010000	13137000	9090400	62596000
O75306	O75306	NADH dehydrogenase [ubiquinone] iron-sulfur pr	NDUFS2	sp|O7530	14	14	14	0	11	4	11	0	11	4	11	0	11	4	11	47.7	52.545	0	89.656	1.612E+09	0	7.92E+08	12236000	8.08E+08	0	72284000	4134800	32760000
Q08257	Q08257	Quinone oxidoreductase	CRYZ	sp|Q08257	10	10	10	0	7	2	10	0	7	2	10	0	7	2	10	49.2	35.206	0	76.838	1.604E+09	0	1.39E+08	2701600	1.46E+09	0	12870000	2311600	59023000
P63000;P6	P63000;P6	Ras-related C3 botulinum toxin substrate 1;Ras-re	RAC1;RAC3	sp|P63000	8	8	4	0	7	3	7	0	7	3	7	0	3	0	3	44.3	21.45	0	29.423	1.6E+09	0	3.51E+08	7567700	1.24E+09	0	23200000	11509000	52363000
P0DN79;P3	P0DN79;P3	Cystathionine beta-synthase	CBS	sp|P0DN7	15	15	15	0	15	0	0	0	15	0	0	0	15	0	0	46.5	60.586	0	86.021	1.598E+09	0	1.6E+09	0	0	0	1.41E+08	0	0
Q13162	Q13162	Peroxiredoxin-4	PRDX4	sp|Q1316	13	10	10	0	13	6	11	0	10	3	9	0	10	3	9	55.7	30.54	0	44.629	1.595E+09	0	1.24E+09	6819100	3.51E+08	0	1.02E+08	11828000	11357000
Q13155	Q13155	Aminoacyl tRNA synthase complex-interacting mu	AIMP2	sp|Q1315	10	10	10	1	9	5	7	1	9	5	7	1	9	5	7	43.8	35.348	0	56.554	1.591E+09	1156200	8.46E+08	33409000	7.11E+08	2875900	91225000	17392000	15926000
Q3ZCQ8	Q3ZCQ8	Mitochondrial import inner membrane translocas	TIMM50	sp|Q3ZCQ	7	7	7	2	7	7	7	2	7	7	7	2	7	7	7	26.3	39.646	0	90.88	1.588E+09	1.09E+08	5.12E+08	44799000	9.21E+08	1.47E+08	23803000	19421000	32182000
O43592	O43592	Exportin-T	XPOT	sp|O4359	16	16	16	1	10	8	15	1	10	8	15	1	10	8	15	22.5	109.96	0	75.005	1.586E+09	656850	3.45E+08	30739000	1.21E+09	1025300	38137000	11128000	51146000
P16435	P16435	NADPH--cytochrome P450 reductase	POR	sp|P16435	16	16	16	0	9	0	16	0	9	0	16	0	9	0	16	32.6	76.689	0	132.29	1.582E+09	0	2.17E+08	0	1.36E+09	0	27109000	0	48130000
P51610	P51610	Host cell factor 1;HCF N-terminal chain 1;HCF N-te	HCFC1	sp|P51610	23	23	23	0	19	4	21	0	19	4	21	0	19	4	21	19.7	208.73	0	95.339	1.579E+09	0	8.41E+08	5318500	7.32E+08	0	62420000	2524400	41080000
Q16795	Q16795	NADH dehydrogenase [ubiquinone] 1 alpha subcc	NDUFA9	sp|Q1679	13	13	13	1	12	7	11	1	12	7	11	1	12	7	11	44	42.509	0	48.619	1.577E+09	1070200	7.34E+08	22593000	8.19E+08	2983900	74312000	11674000	25683000
Q9NUU7;C	Q9NUU7	ATP-dependent RNA helicase DDX19A	DDX19A	sp|Q9NUU	14	14	3	1	14	0	12	1	14	0	12	0	3	0	3	34.5	53.974	0	88.705	1.573E+09	1695100	7.55E+08	0	8.17E+08	2034600	65893000	0	32934000
Q9UQE7	Q9UQE7	Structural maintenance of chromosomes protein	SMC3	sp|Q9UQE	20	20	20	3	20	6	17	3	20	6	17	3	20	6	17	21.9	141.54	0	145.84	1.569E+09	2839300	9.59E+08	18398000	5.88E+08	1871300	78716000	11586000	31778000
Q16666;Q(	Q16666	Gamma-interferon-inducible protein 16	IFI16	sp|Q1666	17	17	17	0	1	14	17	0	1	14	17	0	1	14	17	36.4	88.255	0	64.974	1.562E+09	0	1730100	91744000	1.47E+09	0	181640	65781000	55265000
P14735	P14735	Insulin-degrading enzyme	IDE	sp|P14735	25	25	25	3	22	9	20	3	22	9	20	3	22	9	20	30.3	117.97	0	66.984	1.558E+09	42361000	6.42E+08	27775000	8.46E+08	50160000	53009000	23625000	25576000
Q99598	Q99598	Translin-associated protein X	TSNAX	sp|Q9959	7	7	7	1	7	0	6	1	7	0	6	1	7	0	6	32.4	33.112	0	62.305	1.555E+09	15156000	5E+08	0	1.04E+09	65892000	26353000	0	8945600
P46109	P46109	Crk-like protein	CRKL	sp|P46109	11	11	11	0	11	2	9	0	11	2	9	0	11	2	9	47.5	33.777	0	43.321	1.551E+09	0	8.92E+08	2265600	6.57E+08	0	78220000	1693000	27279000
Q9UG63	Q9UG63	ATP-binding cassette sub-family F member 2	ABCF2	sp|Q9UG6	15	15	15	2	11	14	14	2	11	14	14	2	11	14	14	26.6	71.289	0	43.298	1.547E+09	3451000	1.66E+08	79571000	1.3E+09	7961100	15370000	36024000	65545000
O15118	O15118	Niemann-Pick C1 protein	NPC1	sp|O1511	8	8	8	1	3	2	8	1	3	2	8	1	3	2	8	8.1	142.17	0	25.773	1.545E+09	266730	61809000	4624400	1.48E+09	481210	5444400	2031900	61671000
Q8WXX5	Q8WXX5	DnaJ homolog subfamily C member 9	DNAJC9	sp|Q8WX	11	11	11	2	9	10	10	2	9	10	10	2	9	10	10	45.8	29.909	0	139.58	1.543E+09	1262800	5.63E+08	121960000	8.57E+08	1902900	74814000	42570000	47418000
P15559	P15559	NAD(P)H dehydrogenase [quinone] 1	NQO1	sp|P15559	12	12	12	0	2	1	12	0	2	1	12	0	2	1	12	41.6	30.867	0	109.06	1.539E+09	0	5262400	1317600	1.53E+09	0	379010	523640	63455000
O60271	O60271	C-Jun-amino-terminal kinase-interacting protein 4	SPAG9	sp|O60271	30	30	26	2	25	1	26	2	25	1	26	2	23	1	22	32.9	146.2	0	152.9	1.539E+09	4717200	5.01E+08	5598200	1.03E+09	5321200	39941000	0	45811000
P23258;Q9	P23258;Q9	Tubulin gamma-1 chain;Tubulin gamma-2 chain	TUBG1;TU	sp|P23258	10	10	10	1	10	7	9	1	10	7	9	1	10	7	9	36.6	51.169	0	71.555	1.538E+09	1554600	3.17E+08	38673000	1.18E+09	6469400	42482000	25855000	28568000
Q9UPN3	Q9UPN3	Microtubule-actin cross-linking factor 1, isoforms	MACF1	sp|Q9UPN	46	45	44	2	20	17	45	2	19	16	44	2	19	15	43	9.2	838.3	0	167.23	1.535E+09	1378100	89622000	53665000	1.39E+09	3261900	8665800	33512000	56713000
Q16576	Q16576	Histone-binding protein RBBP7	RBBP7	sp|Q1657	13	6	6	5	11	5	13	3	5	1	6	3	5	1	6	40.7	47.82	0	49.428	1.535E+09	44568000	6.82E+08	1147100	8.07E+08	17524000	80821000	1368600	38939000
O00148	O00148	ATP-dependent RNA helicase DDX39A	DDX39A	sp|O0014	17	8	8	5	15	8	16	0	6	3	8	0	6	3	8	44.7	49.129	0	64.402	1.535E+09	0	2.84E+08	14722000	1.24E+09	0	22794000	17454000	45490000
P0DN76;Q(	P0DN76;Q(	Splicing factor U2AF 35 kDa subunit	U2AF1	sp|P0DN7	8	8	8	1	7	6	7	1	7	6	7	1	7	6	7	46.7	27.872	0	54.732	1.519E+09	3011700	6.87E+08	90671000	7.38E+08	0	37764000	47404000	24399000
P43307	P43307	Translocon-associated protein subunit alpha	SSR1	sp|P43307	5	5	5	0	5	3	4	0	5	3	4	0	5	3	4	28.3	32.235	0	67.228	1.518E+09	0	7.15E+08	38738000	7.64E+08	0	62305000	31052000	27133000
P18031	P18031	Tyrosine-protein phosphatase non-receptor type	PTPN1	sp|P18031	11	11	11	2	10	4	10	2	10	4	10	2	10	4	10	34	49.966	0	55.017	1.517E+09	19404000	3.37E+08	9137900	1.15E+09	17814000	26567000	13779000	44448000
Q93008;O(	Q93008	Probable ubiquitin carboxyl-terminal hydrolase FA	USP9X	sp|Q9300	30	30	30	3	24	5	24	3	24	5	24	3	24	5	24	16.5	290.46	0	95.334	1.516E+09	2168600	6.47E+08	7820700	8.59E+08	1693500	49991000	2600800	37721000
P63151;Q6	P63151	Serine/threonine-protein phosphatase 2A 55 kDa	PPP2R2A	sp|P63151	12	12	12	2	11	5	12	2	11	5	12	2	11	5	12	40.9	51.691	0	111.33	1.515E+09	8911500	3.1E+08	16121000	1.18E+09	11056000	29534000	13699000	40333000
P26583	P26583	High mobility group protein B2	HMGB2	sp|P26583	10	9	9	2	10	3	10	2	9	3	9	2	9	3	9	38.3	24.033	0	28.478	1.512E+09	7051300	8.55E+08	2644300	6.47E+08	9166600	81685000	2566000	17053000
P19623	P19623	Spermidine synthase	SRM	sp|P19623	11	11	11	0	11	0	11	0	11	0	11	0	11	0	11	48	33.824	0	94.986	1.511E+09	0	4.54E+08	0	1.06E+09	0	38660000	0	44330000
P05091;P0	P05091	Aldehyde dehydrogenase, mitochondrial	ALDH2	sp|P05091	14	13	13	1	14	0	6	1	13	0	5	1	13	0	5	37.1	56.381	0	55.661	1.503E+09	1.28E+08	1.3E+09	0	72096000	74296000	1.56E+08	0	15943000
Q9NQW7;(	Q9NQW7	Xaa-Pro aminopeptidase 1	XPNPEP1	sp|Q9NQ\	14	14	14	1	12	1	13	1	12	1	13	1	12	1	13	29.5	69.917	0	53.016	1.498E+09	96261000	5.57E+08	392570	8.45E+08	46719000	1.2E+08	1562200	10619000
Q02543	Q02543	60S ribosomal protein L18a	RPL18A	sp|Q0254	9	9	9	4	6	8	8	4	6	8	8	4	6	8	8	48.3	20.762	0	34.834	1.496E+09	1.05E+08	4.36E+08	128720000	8.26E+08	1.42E+08	29126000	62900000	23039000
Q13247	Q13247	Serine/arginine-rich splicing factor 6	SRSF6	sp|Q13247	12	12	8	5	11	11	11	5	11	11	11	4	7	7	7	31.1	39.586	0	27.461	1.494E+09	52092000	5.65E+08	109770000	7.67E+08	53210000	41376000	72694000	27508000
Q14019	Q14019	Coactosin-like protein	COTL1	sp|Q1401	8	8	8	3	6	0	7	3	6	0	7	3	6	0	7	58.5	15.945	0	48.621	1.492E+09	52116000	1.24E+08	0	1.32E+09	38305000	17939000	0	60917000
Q05682;RE	Q05682	Caldesmon	CALD1	sp|Q0568	19	19	19	1	10	6	19	1	10	6	19	1	10	6	19	28.8	93.23	0	85.376	1.492E+09	646520	1.04E+08	16143000	1.37E+09	1009700	9037900	4794600	59031000
Q13126	Q13126	S-methyl-5-thioadenosine phosphorylase	MTAP	sp|Q1312	10	10	10	0	10	0	0	0	10	0	0	0	10	0	0	49.8	31.236	0	86.342	1.489E+09	0	1.49E+09	0	0	0	1.31E+08	0	0
Q9BPX3	Q9BPX3	Condensin complex subunit 3	NCAPG	sp|Q9BPX	18	18	18	0	13	7	18	0	13	7	18	0	13	7	18	20.2	114.33	0	65.147	1.487E+09	0	3.48E+08	12075000	1.13E+09	0	29811000	11509000	43798000
Q8TAT6	Q8TAT6	Nuclear protein localization protein 4 homolog	NPLOC4	sp|Q8TAT	13	13	13	0	10	5	12	0	10	5	12	0	10	5	12	35.4	68.119	0	60.163	1.486E+09	0	3.08E+08	15884000	1.16E+09	0	31240000	14088000	40277000
O95757	O95757	Heat shock 70 kDa protein 4L	HSPA4L	sp|O95757	21	18	18	5	21	3	17	4	18	3	14	4	18	3	14	34.3	94.511	0	109.64	1.486E+09	54997000	8.86E+08	4906900	5.4E+08	53706000	75948000	7158800	20286000
P46926	P46926	Glucosamine-6-phosphate isomerase 1	GNPDA1	sp|P46926	11	11	8	0	8	1	10	0	8	1	10	0	6	1	8	59.2	32.668	0	65.067	1.481E+09	0	3.16E+08	1461300	1.16E+09	0	30916000	1207200	44561000

Q14914	Q14914	Prostaglandin reductase 1	PTGR1	sp|Q14914	6	6	6	0	0	0	6	0	0	0	6	0	0	0	6	29.8	35.869	0	100.52	1.48E+09	0	0	0	1.48E+09	0	0	0	60872000
Q5T9A4	Q5T9A4	ATPase family AAA domain-containing protein 3B	ATAD3B	sp|Q5T9A4	24	8	6	3	15	20	23	1	3	8	8	1	2	6	6	38.6	72.572	0	129.07	1.48E+09	8261900	65348000	211410000	1.19E+09	7424200	8077200	146580000	43143000
Q8N766	Q8N766	ER membrane protein complex subunit 1	EMC1	sp|Q8N766	21	21	21	1	17	3	21	1	17	3	21	1	17	3	21	29.6	111.76	0	80.763	1.478E+09	298340	2.71E+08	3085400	1.2E+09	394650	21264000	1711900	50060000
Q13177;O7	Q13177	Serine/threonine-protein kinase PAK 2;PAK-2p27;	PAK2	sp|Q13177	16	16	13	0	14	1	16	0	14	1	16	0	11	1	13	42.6	58.042	0	42.588	1.475E+09	0	3.62E+08	1722800	1.11E+09	0	12550000	2884300	57370000
P54920;Q9	P54920	Alpha-soluble NSF attachment protein	NAPA	sp|P54920	10	10	10	0	9	2	10	0	9	2	10	0	9	2	10	39	33.232	0	80.871	1.475E+09	0	2.05E+08	2550600	1.27E+09	0	13675000	2728900	51202000
P04899	P04899	Guanine nucleotide-binding protein G(i) subunit a	GNAI2	sp|P04899	9	9	6	0	5	3	9	0	5	3	9	0	4	1	6	34.1	40.45	0	34.98	1.473E+09	0	1.34E+08	19845000	1.32E+09	0	9197700	17401000	52810000
P08754	P08754	Guanine nucleotide-binding protein G(k) subunit a	GNAI3	sp|P08754	8	5	5	0	3	3	8	0	2	1	5	0	2	1	5	32.8	40.532	0	59.561	1.473E+09	0	41630000	4286800	1.43E+09	0	8996600	5671100	49975000
P15924	P15924	Desmoplakin	DSP	sp|P15924	56	56	56	7	31	55	12	7	31	55	12	7	31	55	12	22.2	331.77	0	160.05	1.471E+09	5135400	3.78E+08	962560000	1.25E+08	6442500	23373000	637050000	5935100
O95299	O95299	NADH dehydrogenase [ubiquinone] 1 alpha subco	NDUFA10	sp|O95299	6	6	6	1	6	2	6	1	6	2	6	1	6	2	6	21.7	40.75	0	66.744	1.47E+09	1726700	6.74E+08	2688400	7.91E+08	8058700	47596000	4007600	35815000
P13473	P13473	Lysosome-associated membrane glycoprotein 2	LAMP2	sp|P13473	3	3	3	0	3	2	3	0	3	2	3	0	3	2	3	7.1	44.96	0	13.916	1.466E+09	0	5.49E+08	5398900	9.11E+08	0	45662000	4033600	39096000
Q86XP3	Q86XP3	ATP-dependent RNA helicase DDX42	DDX42	sp|Q86XP3	17	17	17	0	13	8	14	0	13	8	14	0	13	8	14	31.8	102.97	0	114.81	1.464E+09	0	4.15E+08	14419000	1.03E+09	0	34140000	28666000	26009000
Q16401	Q16401	26S proteasome non-ATPase regulatory subunit 5	PSMD5	sp|Q16401	14	14	14	0	1	2	14	0	1	2	14	0	1	2	14	40.5	56.195	0	178.11	1.463E+09	0	5505000	1760500	1.46E+09	0	297150	463960	60778000
Q56VL3	Q56VL3	OCIA domain-containing protein 2	OCIAD2	sp|Q56VL3	6	6	6	0	1	4	6	0	1	4	6	0	1	4	6	45.5	16.953	0	18.493	1.463E+09	0	2885900	10015000	1.45E+09	0	465560	6624500	59106000
Q12792	Q12792	Twinfilin-1	TWF1	sp|Q12792	15	15	14	1	11	4	15	1	11	4	15	1	10	3	14	61.1	40.282	0	201.69	1.458E+09	681550	1.96E+08	5976700	1.26E+09	702160	17706000	2375000	52827000
O75663	O75663	TIP41-like protein	TIPRL	sp|O75663	10	10	10	1	8	1	9	1	8	1	9	1	8	1	9	56.2	31.444	0	61.223	1.456E+09	43196000	5.04E+08	544400	9.09E+08	22969000	24133000	3346900	74870000
Q7L014	Q7L014	Probable ATP-dependent RNA helicase DDX46	DDX46	sp|Q7L014	24	24	24	5	23	7	17	5	23	7	17	5	23	7	17	27.7	117.36	0	76.047	1.456E+09	10879000	7.74E+08	12919000	6.58E+08	12962000	70317000	9817600	21075000
O43491	O43491	Band 4.1-like protein 2	EPB41L2	sp|O43491	27	27	26	0	26	15	22	0	26	15	22	0	25	14	22	41.9	112.59	0	100.37	1.451E+09	0	6.59E+08	43093000	7.5E+08	0	54974000	33956000	28918000
P62314	P62314	Small nuclear ribonucleoprotein Sm D1	SNRPD1	sp|P62314	4	4	4	2	4	2	3	2	4	2	3	2	4	2	3	45.4	13.281	0	22.208	1.448E+09	5494300	5.51E+08	40060000	8.51E+08	20200000	39957000	22645000	33193000
Q14683	Q14683	Structural maintenance of chromosomes protein	SMC1A	sp|Q14683	37	37	37	5	36	14	22	5	36	14	22	5	36	14	22	33.1	143.23	0	105.05	1.446E+09	11502000	1.05E+09	28441000	3.52E+08	17721000	75138000	28257000	16133000
Q92544	Q92544	Transmembrane 9 superfamily member 4	TM9SF4	sp|Q92544	7	7	7	0	5	2	7	0	5	2	7	0	5	2	7	13.9	74.518	0	41.086	1.445E+09	0	1.31E+08	5232100	1.31E+09	0	13840000	6324300	48735000
Q10567	Q10567	AP-1 complex subunit beta-1	AP1B1	sp|Q10567	23	12	12	0	17	6	21	0	9	0	12	0	9	0	12	30.6	104.64	0	66.959	1.445E+09	0	3.09E+08	0	1.14E+09	0	30963000	0	43011000
Q9Y383	Q9Y383	Putative RNA-binding protein Luc7-like 2	LUC7L2	sp|Q9Y383	13	13	8	4	11	8	11	4	11	8	11	3	7	5	7	29.3	46.513	0	35.848	1.445E+09	56559000	7.61E+08	75836000	5.52E+08	82176000	57348000	34724000	18120000
O60716	O60716	Catenin delta-1	CTNND1	sp|O60716	18	18	18	0	15	14	16	0	15	14	16	0	15	14	16	29.1	108.17	0	112.34	1.439E+09	0	2.35E+08	76282000	1.13E+09	0	22516000	56517000	39243000
Q5JRX3	Q5JRX3	Presequence protease, mitochondrial	PITRM1	sp|Q5JRX3	21	21	21	0	17	1	17	0	17	1	17	0	17	1	17	27.7	117.41	0	109.71	1.432E+09	0	4.09E+08	3603400	1.02E+09	0	24908000	5988800	49486000
Q96I24	Q96I24	Far upstream element-binding protein 3	FUBP3	sp|Q96I24	21	18	18	3	20	8	16	2	17	8	14	2	17	8	14	56.3	61.64	0	70.211	1.418E+09	1825200	7.31E+08	32271000	6.53E+08	1929300	55500000	34867000	22510000
Q13011	Q13011	Delta(3,5)-Delta(2,4)-dienoyl-CoA isomerase, mitc	ECH1	sp|Q13011	12	12	12	3	11	3	11	3	11	3	11	3	11	3	11	53.4	35.816	0	163.19	1.417E+09	52009000	6E+08	4780700	7.6E+08	37507000	65466000	6588700	29825000
Q14257	Q14257	Reticulocalbin-2	RCN2	sp|Q14257	9	9	9	0	9	8	8	0	9	8	8	0	9	8	8	37.9	36.876	0	127.81	1.417E+09	0	6.26E+08	117810000	6.73E+08	0	54820000	77664000	29554000
Q9Y520	Q9Y520	Protein PRRC2C	PRRC2C	sp|Q9Y520	38	38	37	8	25	31	33	8	25	31	33	8	24	30	32	20.3	316.91	0	112.31	1.413E+09	58027000	2.43E+08	375060000	7.37E+08	45402000	41974000	241600000	30148000
O95747	O95747	Serine/threonine-protein kinase OSR1	OXSR1	sp|O95747	10	10	10	0	9	1	10	0	9	1	10	0	9	1	10	25	58.022	0	48.264	1.413E+09	0	3.14E+08	861740	1.1E+09	0	27865000	881250	44652000
O14929	O14929	Histone acetyltransferase type B catalytic subunit	HAT1	sp|O14929	9	9	9	0	9	0	6	0	9	0	6	0	9	0	6	29.4	49.512	0	39.629	1.407E+09	0	5.53E+08	0	8.55E+08	0	53550000	0	30313000
P10515	P10515	Dihydrolipoyllysine-residue acetyltransferase com	DLAT	sp|P10515	10	10	10	1	10	1	6	1	10	1	6	1	10	1	6	21	68.996	0	43.179	1.405E+09	38652000	1.16E+09	1189200	2.08E+08	27688000	1.16E+08	1462300	3524600
P11279	P11279	Lysosome-associated membrane glycoprotein 1	LAMP1	sp|P11279	5	5	5	1	2	2	5	1	2	2	5	1	2	2	5	11.8	44.882	0	20.564	1.405E+09	930550	1.04E+08	6577000	1.29E+09	8265600	7944400	14535000	35592000
O60313	O60313	Dynamin-like 120 kDa protein, mitochondrial;Dyn	OPA1	sp|O60313	20	20	20	2	13	11	19	2	13	11	19	2	13	11	19	25.9	111.63	0	116.96	1.399E+09	1684700	2.15E+08	23797000	1.16E+09	3244200	13783000	14317000	52914000
P26358	P26358	DNA (cytosine-5)-methyltransferase 1	DNMT1	sp|P26358	23	23	23	0	21	11	23	0	21	11	23	0	21	11	23	16.6	183.16	0	81.291	1.396E+09	0	2.91E+08	23305000	1.08E+09	0	25996000	23024000	36799000
P31947	P31947	14-3-3 protein sigma	SFN	sp|P31947	7	3	3	1	5	6	5	0	1	3	1	0	1	3	1	27.4	27.774	0	7.0699	1.395E+09	0	5.4E+08	19809000	8.35E+08	0	52210000	5159700	37760000
P60983;O6	P60983	Glia maturation factor beta	GMFB	sp|P60983	6	6	6	0	6	1	6	0	6	1	6	0	6	1	6	53.5	16.713	0	94.477	1.394E+09	0	1.9E+08	294860	1.2E+09	0	4867700	466020	61109000
O95399	O95399	Urotensin-2	UTS2	sp|O95399	1	1	1	1	1	1	1	1	1	1	1	1	1	1	1	6.5	14.295	0.001131	2.4452	1.392E+09	41652000	6.06E+08	112300000	6.31E+08	41652000	53457000	75468000	25969000
Q9UNS2	Q9UNS2	COP9 signalosome complex subunit 3	COPS3	sp|Q9UNS	10	10	10	0	10	2	9	0	10	2	9	0	10	2	9	32.2	47.873	0	46.673	1.39E+09	0	5.5E+08	3492600	8.36E+08	0	42666000	3948900	38563000
Q13098	Q13098	COP9 signalosome complex subunit 1	GPS1	sp|Q13098	9	9	9	0	8	1	9	0	8	1	9	0	8	1	9	25.3	55.536	0	50.295	1.389E+09	0	4.41E+08	1703700	9.46E+08	0	40409000	1455600	36661000
P49753;Q8	P49753;Q8	Acyl-coenzyme A thioesterase 2, mitochondrial;A	ACOT2;AC	sp|P49753	11	11	11	0	10	0	9	0	10	0	9	0	10	0	9	36.4	53.218	0	98.295	1.388E+09	0	4.61E+08	0	9.27E+08	0	45459000	0	33318000
O15042	O15042	U2 snRNP-associated SURP motif-containing prot	U2SURP	sp|O15042	22	22	22	2	22	17	19	2	22	17	19	2	22	17	19	32	118.29	0	87.995	1.388E+09	26625000	7.59E+08	86002000	5.16E+08	21181000	64060000	52786000	34521000
Q9NTX5	Q9NTX5	Ethylmalonyl-CoA decarboxylase	ECHDC1	sp|Q9NTX	8	8	8	0	6	1	8	0	6	1	8	0	6	1	8	36.5	33.698	0	108.6	1.383E+09	0	4.98E+08	1060800	8.83E+08	0	46108000	812540	34030000
O95202	O95202	LETM1 and EF-hand domain-containing protein 1,	LETM1	sp|O95202	18	18	18	1	17	3	12	1	17	3	12	1	17	3	12	36.1	83.353	0	60.786	1.381E+09	135500	6.94E+08	6844800	6.8E+08	410720	82726000	2959200	7760400
O43324	O43324	Eukaryotic translation elongation factor 1 epsilon-	EEF1E1	sp|O43324	5	5	5	4	5	3	4	4	5	3	4	4	5	3	4	38.5	19.81	0	40.243	1.38E+09	1.99E+08	7.91E+08	20567000	3.7E+08	1.42E+08	87043000	27827000	24927000
Q96EP5	Q96EP5	DAZ-associated protein 1	DAZAP1	sp|Q96EP5	4	4	4	0	4	4	4	0	4	4	4	0	4	4	4	17	43.383	0	50.342	1.375E+09	0	7.06E+08	22884000	6.46E+08	0	65495000	14335000	24315000
P11172	P11172	Uridine 5-monophosphate synthase;Orotate phos	UMPS	sp|P11172	11	11	11	0	11	5	9	0	11	5	9	0	11	5	9	34.2	52.221	0	32.121	1.375E+09	0	7.48E+08	5687200	6.21E+08	0	71315000	5262000	18718000
P78345	P78345	Ribonuclease P protein subunit p38	RPP38	sp|P78345	4	4	4	1	3	2	3	1	3	2	3	1	3	2	3	21.2	31.834	0	11.482	1.374E+09	1408900	6.8E+08	51314000	6.41E+08	5712500	39705000	57074000	19730000
O60749	O60749	Sorting nexin-2	SNX2	sp|O60749	15	15	13	0	13	2	15	0	13	2	15	0	11	1	13	34.3	58.47	0	41.921	1.373E+09	0	4.8E+08	3828900	8.89E+08	0	25981000	6530400	47611000
P48147	P48147	Prolyl endopeptidase	PREP	sp|P48147	18	18	18	2	18	0	16	2	18	0	16	2	18	0	16	32	80.699	0	90.172	1.372E+09	1051900	6.84E+08	0	6.87E+08	943760	57489000	0	31146000
P08621	P08621	U1 small nuclear ribonucleoprotein 70 kDa	SNRNP70	sp|P08621	13	13	13	5	13	12	13	5	13	12	13	5	13	12	13	34.1	51.556	0	85.244	1.369E+09	16085000	6.18E+08	60140000	6.75E+08	35698000	46628000	24279000	25335000
O00154	O00154	Cytosolic acyl coenzyme A thioester hydrolase	ACOT7	sp|O00154	7	7	7	1	7	5	7	1	7	5	7	1	7	5	7	25.5	41.796	0	47.493	1.366E+09	450270	3.62E+08	17797000	9.85E+08	595270	30404000	5747000	48128000
P42765	P42765	3-ketoacyl-CoA thiolase, mitochondrial	ACAA2	sp|P42765	12	12	12	1	11	1	10	1	11	1	10	1	11	1	10	50.1	41.924	0	90.777	1.365E+09	509750	6.42E+08	276430	7.22E+08	427960	35332000	585030	48622000
P62888	P62888	60S ribosomal protein L30	RPL30	sp|P62888	7	7	7	1	4	6	7	1	4	6	7	1	4	6	7	53	12.784	0	39.476	1.364E+09	2815700	1.3E+08	258290000	9.73E+08	13043000	25162000	127850000	61817000
Q9UI12	Q9UI12	V-type proton ATPase subunit H	ATP6V1H	sp|Q9UI12	8	8	8	1	7	5	8	1	7	5	8	1	7	5	8	27.5	55.882	0	78.676	1.364E+09	1118500	2.9E+08	23176000	1.05E+09	3876900	15200000	14171000	52187000
Q9HDC9	Q9HDC9	Adipocyte plasma membrane-associated protein	APMAP	sp|Q9HDC	12	12	12	2	11	3	11	2	11	3	11	2	11	3	11	43	46.48	0	71.943	1.362E+09	4555500	4.44E+08	3573800	9.1E+08	1282900	41831000	1858500	37704000
P23919	P23919	Thymidylate kinase	DTYMK	sp|P23919	13	13	13	0	9	3	12	0	9	3	12	0	9	3	12	64.6	23.819	0	35.403	1.358E+09	0	2.99E+08	2560000	1.06E+09	0	34279000	1223600	32644000
Q9Y613	Q9Y613	FH1/FH2 domain-containing protein 1	FHOD1	sp|Q9Y613	20	20	20	0	15	15	20	0	15	15	20	0	15	15	20	24	126.55	0	113.53	1.354E+09	0	1.43E+08	75175000	1.14E+09	0	11341000	60511000	37901000
Q02750	Q02750	Dual specificity mitogen-activated protein kinase	MAP2K1	sp|Q02750	12	8	8	0	9	2	12	0	6	1	8	0	6	1	8	48.3	43.439	0	45.218	1.345E+09	0	1.73E+08	969080	1.17E+09	0	29201000	8822200	25481000
O60884	O60884	DnaJ homolog subfamily A member 2	DNAJA2	sp|O60884	13	13	13	2	10	10	13	2	10	10	13	2	10	10	13	39.1	45.745	0	60.679	1.342E+09	14503000	2.71E+08	44947000	1.01E+09	25222000	19751000	26126000	39118000
O75439	O75439	Mitochondrial-processing peptidase subunit beta	PMPCB	sp|O75439	13	13	13	0	12	7	10	0	12	7	10	0	12	7	10	33.7	54.366	0	117.05	1.34E+09	0	4.71E+08	41463000	8.28E+08	0	41489000	35179000	26745000
Q9BXS5;Q9	Q9BXS5	AP-1 complex subunit mu-1	AP1M1	sp|Q9BXS5	13	13	13	0	11	3	13	0	11	3	13	0	11	3	13	44.4	48.586	0	43.715	1.339E+09	0	3.77E+08	7265000	9.55E+08	0	23055000	7539100	46794000
P16989;Q9	P16989	Y-box-binding protein 3	YBX3	sp|P16989	16	10	10	12	16	16	14	6	10	10	8	6	10	10	8	58.1	40.089	0	39.805	1.338E+09	29439000	4.49E+08	200020000	6.6E+08	42835000	34557000	124020000	29166000
P04818	P04818	Thymidylate synthase	TYMS	sp|P04818	9	9	9	0	8	1	9	0	8	1	9	0	8	1	9	40.6	35.716	0	61.302	1.338E+09	0	2.67E+08	737440	1.07E+09	0	23344000	717620	43980000
O43684	O43684	Mitotic checkpoint protein BUB3	BUB3	sp|O43684	10	10	10	2	10	4	9	2	10	4	9	2	10	4	9	32.6	37.154	0	33.282	1.333E+09	4312000	3.87E+08	16998000	9.24E+08	5649100	35607000	11074000	35555000
Q13442	Q13442	28 kDa heat- and acid-stable phosphoprotein	PDAP1	sp|Q13442	7	7	7	3	6	3	6	3	6	3	6	3	6	3	6	27.6	20.63	0	20.342	1.327E+09	5292600	5.45E+08	11020000	7.66E+08	5547200	47096000	7233000	27280000
Q96CW1	Q96CW1	AP-2 complex subunit mu	AP2M1	sp|Q96CW	11	11	11	0	8	6	11	0	8	6	11	0	8	6	11	32.2	49.654	0	46.267	1.323E+09	0	98824000	27308000	1.2E+09	0	10833000	24912000	39361000
Q99615	Q99615	DnaJ homolog subfamily C member 7	DNAJC7	sp|Q99615	17	17	17	0	14	2	16	0	14	2	16	0	14	2	16	38.9	56.44	0	59.948	1.322E+09	0	5.44E+08	2493300	7.76E+08	0	40661000	1659100	39184000
P42285	P42285	Superkiller viralicidic activity 2-like 2	SKIV2L2	sp|P42285	16	16	16	3	13	7	15	3	13	7	15	3	13	7	15	22.6	117.8	0	73.418	1.322E+09	7973500	3.3E+08	36063000	9.47E+08	14080000	32716000	19828000	33676000
O14579	O14579	Coatomer subunit epsilon	COPE	sp|O14579	9	9	9	0	5	2	9	0	5	2	9	0	5	2	9	56.2	34.482	0	92.311	1.321E+09	0	1.31E+08	21769000	1.17E+09	0	13419000	22469000	38327000
Q86UE8	Q86UE8	Serine/threonine-protein kinase tousled-like 2	TLK2	sp|Q86UE8	2	2	2	0	2	0	2	0	2	0	2	0	2	0	2	3.9	87.66	0.004329	1.8854	1.319E+09	0	6.06E+08	0	7.13E+08	0	51582000	0	27091000
Q9BRX8	Q9BRX8	Redox-regulatory protein FAM213A	FAM213A	sp|Q9BRX8	8	8	8	0	8	0	6	0	8	0	6	0	8	0	6	30.1	25.764	0	18.351	1.318E+09	0	5.02E+08	0	8.16E+08	0	40660000	0	37132000
P08648	P08648	Integrin alpha-5;Integrin alpha-5 heavy chain;Inte	ITGA5	sp|P08648	12	12	12	0	0	2	12	0	0	2	12	0	0	2	12	17.2	114.54	0	84.127	1.314E+09	0	0	9624700	1.3E+09	0	0	6705800	48941000
Q86UE4	Q86UE4	Protein LYRIC	MTDH	sp|Q86UE4	20	20	20	6	18	16	16	6	18	16	16	6	18	16	16	52.1	63.836	0	130.77	1.313E+09	13711000	6.17E+08	104150000	5.78E+08	15180000	53140000	72000000	21501000
Q96AC1	Q96AC1	Fermitin family homolog 2	FERMT2	sp|Q96AC1	15	15	14	0	10	1	15	0	10	1	15	0	9	1	14	31.6	77.86	0	68.46	1.312E+09	0	1.55E+08	2901700	1.15E+09	0	10632000	3585300	48854000
Q6YN16	Q6YN16	Hydroxysteroid dehydrogenase-like protein 2	HSDL2	sp|Q6YN16	8	8	8	0	7	2	7	0	7	2	7	0	7	2	7	29.9	45.394	0	68.985	1.307E+09	0	7.87E+08	2610300	5.17E+08	0	64251000	4245100	23916000
P47755	P47755	F-actin-capping protein subunit alpha-2	CAPZA2	sp|P47755	11	8	8	1	10	9	11	0	7	6	8	0	7	6	8	58.7	32.949	0	110.64	1.3E+09	0	2.37E+08	42360000	1.02E+09	0	18171000	31980000	41162000
Q9HB07	Q9HB07	UPF0160 protein MYG1, mitochondrial	C12orf10	sp|Q9HB07	10	10	10	0	9	0	8	0	9	0	8	0	9	0	8	30.1	42.477	0	38.611	1.298E+09	0	3.42E+08	0	9.56E+08	0	31967000	0	37520000
Q9NSE4	Q9NSE4	Isoleucine--tRNA ligase, mitochondrial	IARS2	sp|Q9NSE4	21	21	21	1	17	5	21	1	17	5	21	1	17	5	21	27.7	113.79	0	59.861	1.296E+09	4186000	4.12E+08	5938600	8.74E+08	6015500	37549000	3982400	32911000
P35080	P35080	Profilin-2	PFN2	sp|P35080	7	7	7	1	7	3	7	1	7	3	7	1	7	3	7	62.1	15.046	0	36.971	1.292E+09	946600	3.57E+08	14906000	9.19E+08	1124000	25953000	10679000	38247000
Q9H1E3	Q9H1E3	Nuclear ubiquitous casein and cyclin-dependent k	NUCKS1	sp|Q9H1E3	6	6	6	1	6	2	6	1	6	2	6	1	6	2	6	18.9	27.296	0	27.062	1.291E+09	2970000	7.91E+08	1846900	4.95E+08	5585300	53679000	2621900	32412000
Q96P70	Q96P70	Importin-9	IPO9	sp|Q96P70	12	12	12	1	10	4	11	1	10	4	11	1	10	4	11	14.8	115.96	0	77.074	1.291E+09	150380	3.76E+08	9408200	9.05E+08	289690	37072000	4857000	34631000
Q14258	Q14258	E3 ubiquitin/ISG15 ligase TRIM25	TRIM25	sp|Q14258	12	12	12	2	9	7	10	2	9	7	10	2	9	7	10	24.9	70.973	0	38.588	1.287E+09	2629600	2.78E+08	91219000	9.16E+08	3812800	33237000	43942000	45092000
O94925	O94925	Glutaminase kidney isoform, mitochondrial	GLS	sp|O94925	12	12	12	1	11	7	12	1	11	7	12	1	11	7	12	27.4	73.46	0	117.11	1.286E+09	654000	2.23E+08	25002000	1.04E+09	2186700	13099000	15150000	49338000
O75131;Q9	O75131	Copine-3	CPNE3	sp|O75131	13	13	12	0	13	4	12	0	13	4	12	0	12	3	11	27	60.13	0	38.764	1.285E+09	0	5.1E+08	2352900	7.73E+08	0	37432000	1001000	39887000
Q9BWD1	Q9BWD1	Acetyl-CoA acetyltransferase, cytosolic	ACAT2	sp|Q9BWD	11	11	11	0	11	0	6	0	11	0	6	0	11	0	6	45.8	41.35	0	119.95	1.284E+09	0	1.08E+09	0	2.04E+08	0	86162000	0	10578000
A5YKK6	A5YKK6	CCR4-NOT transcription complex subunit 1	CNOT1	sp|A5YKK6	28	28	28	3	23	18	27	3	23	18	27	3	23	18	27	15.5	266.94	0	106.52	1.282E+09	3747200	3.31E+08	61602000	8.86E+08	5134800	26691000	48379000	30523000
O43747	O43747	AP-1 complex subunit gamma-1	AP1G1	sp|O43747	12	12	12	0	10	1	10	0	10	1	10	0	10	1	10	24.3	91.35	0	80.35	1.28E+09	0	3.35E+08	114400	9.45E+08	0	30891000	101050	32132000
Q15645	Q15645	Pachytene checkpoint protein 2 homolog	TRIP13	sp|Q15645	15	15	15	0	12	5	14	0	12	5	14	0	12	5	14	42.1	48.55	0	45.305	1.278E+09	0	2.88E+08	11855000	9.78E+08	0	28668000	12326000	32560000
Q7Z417	Q7Z417	Nuclear fragile X mental retardation-interacting p	NUFIP2	sp|Q7Z417	17	17	17	4	15	16	15	4	15	16	15	4	15	16	15	38.1	76.12	0	50.775	1.277E+09	4.07E+08	1.96E+08	181070000	4.93E+08	73397000	1.73E+08	244690000	75254000

P15927	P15927	Replication protein A 32 kDa subunit	RPA2	sp|P15927	8	8	8	2	8	4	6	2	8	4	6	2	8	4	6	55.9	29.247	0	38.873	1.275E+09	18623000	6.13E+08	15931000	6.27E+08	20146000	52072000	13727000	23187000
Q9BVK6	Q9BVK6	Transmembrane emp24 domain-containing prote	TMED9	sp|Q9BVK	6	6	5	0	5	2	6	0	5	2	6	0	4	2	5	29.4	27.277	0	68.802	1.273E+09	0	4.46E+08	5371300	8.22E+08	0	37435000	2987900	28747000
Q04760	Q04760	Lactoylglutathione lyase	GLO1	sp|Q0476	6	6	6	0	6	0	4	0	6	0	4	0	6	0	4	38	20.777	0	31.194	1.272E+09	0	1.12E+09	0	1.48E+08	0	95577000	0	9542000
O60762	O60762	Dolichol-phosphate mannosyltransferase subunit	DPM1	sp|O60762	8	8	8	0	7	7	8	0	7	7	8	0	7	7	8	40	29.634	0	30.132	1.27E+09	0	3.56E+08	55562000	8.58E+08	0	31524000	42493000	28914000
Q96HY6	Q96HY6	DDRGK domain-containing protein 1	DDRGK1	sp|Q96HY	8	8	8	0	7	8	8	0	7	8	8	0	7	8	8	51.9	35.61	0	122.52	1.269E+09	0	4.9E+08	50120000	7.29E+08	0	39216000	41631000	25264000
Q01844	Q01844	RNA-binding protein EWS	EWSR1	sp|Q01844	7	7	7	2	7	5	6	2	7	5	6	2	7	5	6	16	68.477	0	70.334	1.267E+09	2867100	5.54E+08	36530000	6.73E+08	4935800	45679000	23491000	28815000
Q9Y4Z0	Q9Y4Z0	U6 snRNA-associated Sm-like protein LSm4	LSM4	sp|Q9Y4Z	4	4	4	0	4	1	3	0	4	1	3	0	4	1	3	25.9	15.35	0	12.383	1.263E+09	0	6.75E+08	2785100	5.85E+08	0	70395000	3096200	11975000
O60610	O60610	Protein diaphanous homolog 1	DIAPH1	sp|O6061	21	21	21	0	18	3	21	0	18	3	21	0	18	3	21	19.7	141.35	0	100.15	1.262E+09	0	4.01E+08	1667800	8.6E+08	0	40174000	933820	30698000
P62308;A8	P62308;A8	Small nuclear ribonucleoprotein G;Putative small	SNRPG;SN	sp|P62308	4	4	4	1	4	3	4	1	4	3	4	1	4	3	4	67.1	8.496	0	10.533	1.257E+09	27428000	6.33E+08	22200000	5.74E+08	51111000	40254000	10109000	20297000
P38159;Q9	P38159;Q9	RNA-binding motif protein, X chromosome;RNA-b	RBMX;RBN	sp|P38159	13	13	12	4	13	13	13	4	13	13	13	4	12	12	12	34	42.331	0	41.746	1.257E+09	1.03E+08	5.55E+08	118240000	4.81E+08	60127000	66111000	98041000	26943000
P61764	P61764	Syntaxin-binding protein 1	STXBP1	sp|P61764	12	12	12	0	8	1	12	0	8	1	12	0	8	1	12	31	67.568	0	60.235	1.256E+09	0	83491000	35700000	1.14E+09	0	13419000	21854000	42826000
Q9Y5K5	Q9Y5K5	Ubiquitin carboxyl-terminal hydrolase isozyme L5	UCHL5	sp|Q9Y5K5	14	14	14	0	14	1	10	0	14	1	10	0	14	1	10	62.9	37.606	0	95.98	1.255E+09	0	8.51E+08	753310	4.04E+08	0	85296000	844040	5124800
P17858	P17858	ATP-dependent 6-phosphofructokinase, liver type	PFKL	sp|P17858	20	15	15	6	16	10	17	3	11	5	13	3	11	5	13	31.2	85.018	0	62.321	1.244E+09	2345300	3.11E+08	13628000	9.17E+08	1813600	32538000	8864800	31698000
Q13617	Q13617	Cullin-2	CUL2	sp|Q13617	16	16	16	0	11	1	16	0	11	1	16	0	11	1	16	24.2	86.982	0	49.752	1.243E+09	0	2.4E+08	263430	1E+09	0	7642000	799310	54124000
Q9Y2A7	Q9Y2A7	Nck-associated protein 1	NCKAP1	sp|Q9Y2A7	21	21	21	0	13	4	20	0	13	4	20	0	13	4	20	24.1	128.79	0	134.29	1.242E+09	0	1.5E+08	4888500	1.09E+09	0	16538000	5261300	39430000
P36507;A0	P36507	Dual specificity mitogen-activated protein kinase I	MAP2K2	sp|P36507	13	13	9	0	11	3	13	0	11	3	13	0	8	2	9	50.5	44.424	0	107.2	1.24E+09	0	2.32E+08	1549800	1.01E+09	0	18553000	1354200	41143000
Q9BQA1	Q9BQA1	Methylosome protein 50	WDR77	sp|Q9BQA	8	8	8	0	8	4	5	0	8	4	5	0	8	4	5	43.9	36.724	0	30.565	1.239E+09	0	6.78E+08	12176000	5.49E+08	0	52089000	9970100	28485000
Q9Y237	Q9Y237	Peptidyl-prolyl cis-trans isomerase NIMA-interact	PIN4	sp|Q9Y237	5	5	5	0	5	0	5	0	5	0	5	0	5	0	5	55.7	13.81	0	16.415	1.235E+09	0	8.37E+08	0	3.98E+08	0	68472000	0	21697000
Q15155	Q15155	Nodal modulator 1	NOMO1	sp|Q15155	20	20	1	0	17	1	18	0	17	1	18	0	1	0	1	24	134.32	0	60.715	1.235E+09	0	3.93E+08	505760	8.42E+08	0	36017000	448470	33108000
O15143	O15143	Actin-related protein 2/3 complex subunit 1B	ARPC1B	sp|O15143	9	9	9	0	6	3	9	0	6	3	9	0	6	3	9	32.8	40.949	0	26.545	1.234E+09	0	84168000	4363000	1.15E+09	0	7809900	4068100	42180000
P04439	P04439	HLA class I histocompatibility antigen, A-3 alpha cl	HLA-A	sp|P04439	4	4	3	1	2	1	4	1	2	1	4	1	1	0	3	15.1	40.84	0	44.066	1.234E+09	81694000	44911000	6149500	1.1E+09	84257000	2912600	9493300	38394000
P49792;A6	P49792	E3 SUMO-protein ligase RanBP2	RANBP2	sp|P49792	28	28	28	5	18	15	25	5	18	15	25	5	18	15	25	15	358.2	0	78.399	1.231E+09	11416000	2E+08	42074000	9.78E+08	10513000	15345000	44668000	26987000
O76021	O76021	Ribosomal L1 domain-containing protein 1	RSL1D1	sp|O76021	14	14	14	2	3	11	14	2	3	11	14	2	3	11	14	35.1	54.972	0	50.852	1.229E+09	2069800	51642000	131820000	1.04E+09	384540	4320200	110100000	22278000
Q8IY67	Q8IY67	Ribonucleoprotein PTB-binding 1	RAVER1	sp|Q8IY67	7	7	7	0	6	1	5	0	6	1	5	0	6	1	5	22.1	63.876	0	109.5	1.227E+09	0	7.17E+08	622640	5.1E+08	0	63577000	0	20605000
Q9BS26	Q9BS26	Endoplasmic reticulum resident protein 44	ERP44	sp|Q9BS2	12	12	12	1	12	1	7	1	12	1	7	1	12	1	7	44.6	46.971	0	78.229	1.227E+09	216560	7.86E+08	492140	4.4E+08	936120	41476000	404800	25269000
P61106	P61106	Ras-related protein Rab-14	RAB14	sp|P61106	9	9	9	0	8	3	9	0	8	3	9	0	8	3	9	49.8	23.897	0	35.824	1.226E+09	0	3.41E+08	6440000	8.78E+08	0	26516000	4844800	39164000
P62306	P62306	Small nuclear ribonucleoprotein F	SNRPF	sp|P62306	3	3	3	1	3	1	3	1	3	1	3	1	3	1	3	39.5	9.7251	0	12.228	1.225E+09	8162700	5.14E+08	1518700	7.02E+08	45339000	8686300	3149000	3188500
P10155	P10155	60 kDa SS-A/Ro ribonucleoprotein	TROVE2	sp|P10155	11	11	11	1	10	2	9	1	10	2	9	1	10	2	9	29.7	60.67	0	68.269	1.224E+09	14078000	3.21E+08	6829700	8.82E+08	11371000	31460000	3570300	34372000
Q15717;Q1	Q15717	ELAV-like protein 1	ELAVL1	sp|Q15717	13	13	13	3	11	12	11	3	11	12	11	3	11	12	11	49.4	36.091	0	76.726	1.224E+09	73411000	4.1E+08	121580000	6.19E+08	1.05E+08	28240000	60622000	19671000
Q8N1F7	Q8N1F7	Nuclear pore complex protein Nup93	NUP93	sp|Q8N1F7	23	23	23	2	22	13	20	2	22	13	20	2	22	13	20	28.1	93.487	0	62.22	1.222E+09	2628600	2.39E+08	35334000	9.45E+08	2695100	19028000	31582000	33007000
Q6PIU2	Q6PIU2	Neutral cholesterol ester hydrolase 1	NCEH1	sp|Q6PIU2	11	11	11	0	0	0	11	0	0	0	11	0	0	0	11	35.3	45.807	0	38.238	1.222E+09	0	0	0	1.22E+09	0	0	0	50248000
P00568;Q9	P00568	Adenylate kinase isoenzyme 1	AK1	sp|P00568	8	8	8	0	4	0	8	0	4	0	8	0	4	0	8	44.8	21.635	0	38.518	1.222E+09	0	2.37E+08	0	9.85E+08	0	20532000	0	40836000
P48739	P48739	Phosphatidylinositol transfer protein beta isoforn	PITPNB	sp|P48739	13	13	13	0	10	1	12	0	10	1	12	0	10	1	12	50.2	31.54	0	36.877	1.217E+09	0	4.68E+08	952580	7.48E+08	0	45275000	678200	26730000
P09012	P09012	U1 small nuclear ribonucleoprotein A	SNRPA	sp|P09012	11	11	9	1	9	7	8	1	9	7	8	1	8	5	7	42.9	31.279	0	55.05	1.216E+09	9793300	5.61E+08	25330000	6.2E+08	9502300	51133000	10314000	24257000
O95816	O95816	BAG family molecular chaperone regulator 2	BAG2	sp|O9581	6	6	6	3	6	6	5	3	6	6	5	3	6	6	5	37	23.772	0	79.048	1.215E+09	67140000	2.82E+08	35590000	8.3E+08	60451000	30766000	35922000	22910000
P11387;Q9	P11387	DNA topoisomerase 1	TOP1	sp|P11387	18	18	18	1	15	16	14	1	15	16	14	1	15	16	14	24.3	90.725	0	37.556	1.214E+09	616710	2.95E+08	152160000	7.66E+08	6264500	18879000	79102000	56173000
O00469	O00469	Procollagen-lysine,2-oxoglutarate 5-dioxygenase	PLOD2	sp|O0046	15	15	15	0	6	3	15	0	6	3	15	0	6	3	15	27.4	84.685	0	105.89	1.211E+09	0	34644000	13050000	1.16E+09	0	3611600	2157300	53623000
Q9BY44	Q9BY44	Eukaryotic translation initiation factor 2A;Eukaryc	EIF2A	sp|Q9BY4	21	21	21	5	16	5	20	5	16	5	20	5	16	5	20	52.6	64.989	0	83.747	1.209E+09	8917200	3.11E+08	5878300	8.83E+08	14600000	18781000	10003000	31714000
Q96RP9	Q96RP9	Elongation factor G, mitochondrial	GFM1	sp|Q96RP	17	17	17	1	12	0	17	1	12	0	17	1	12	0	17	33.8	83.471	0	71.078	1.206E+09	3247500	2.9E+08	0	9.12E+08	5756400	17919000	0	42684000
Q13428	Q13428	Treacle protein	TCOF1	sp|Q1342	27	27	27	3	25	21	22	3	25	21	22	3	25	21	22	21.6	152.1	0	78.847	1.206E+09	8199200	6.71E+08	73192000	4.54E+08	5202200	48648000	56324000	24988000
O60493	O60493	Sorting nexin-3	SNX3	sp|O6049	11	11	9	0	9	2	10	0	9	2	10	0	7	2	9	69.8	18.762	0	42.309	1.203E+09	0	2.56E+08	730690	9.46E+08	0	23128000	571120	38257000
Q00688	Q00688	Peptidyl-prolyl cis-trans isomerase FKBP3	FKBP3	sp|Q0068	8	8	8	1	6	2	8	1	6	2	8	1	6	2	8	33	25.177	0	21.333	1.2E+09	2300900	4.13E+08	1833400	7.84E+08	4989900	36683000	2271500	28177000
Q96KG9	Q96KG9	N-terminal kinase-like protein	SCYL1	sp|Q96KG	19	19	19	5	10	17	15	5	10	17	15	5	10	17	15	38.5	89.63	0	140.24	1.199E+09	2.24E+08	96574000	286920000	5.91E+08	2.41E+08	10303000	177130000	20464000
P60953	P60953	Cell division control protein 42 homolog	CDC42	sp|P60953	5	5	5	1	3	2	5	1	3	2	5	1	3	2	5	31.4	21.258	0	18.644	1.196E+09	1448600	2.32E+08	4698100	9.58E+08	2032300	13752000	3558800	45109000
P49257	P49257	Protein ERGIC-53	LMAN1	sp|P49257	15	15	15	0	12	2	12	0	12	2	12	0	12	2	12	39.2	57.548	0	77.795	1.195E+09	0	3.1E+08	6388600	8.79E+08	0	21890000	7875000	35777000
P15586	P15586	N-acetylglucosamine-6-sulfatase	GNS	sp|P15586	8	8	8	0	3	0	8	0	3	0	8	0	3	0	8	23.6	62.081	0	46.748	1.189E+09	0	66735000	0	1.12E+09	0	8829400	0	43213000
O75694	O75694	Nuclear pore complex protein Nup155	NUP155	sp|O7569	19	19	19	2	17	4	16	2	17	4	16	2	17	4	16	20.1	155.2	0	70.451	1.189E+09	12050000	2.48E+08	6792100	9.23E+08	12445000	25245000	7101200	30706000
P00374;Q8	P00374	Dihydrofolate reductase	DHFR	sp|P00374	8	8	8	1	8	1	8	1	8	1	8	1	8	1	8	47.6	21.452	0	33.172	1.187E+09	379550	5.37E+08	149630	6.5E+08	317100	22473000	316400	48552000
Q9NT62	Q9NT62	Ubiquitin-like-conjugating enzyme ATG3	ATG3	sp|Q9NT6	5	5	5	0	5	1	5	0	5	1	5	0	5	1	5	27.4	35.864	0	63.427	1.185E+09	0	2.42E+08	900620	9.42E+08	0	7759200	1353200	51601000
P40616	P40616	ADP-ribosylation factor-like protein 1	ARL1	sp|P40616	5	5	5	0	4	2	5	0	4	2	5	0	4	2	5	43.6	20.417	0	138.25	1.185E+09	0	1.78E+08	13254000	9.93E+08	0	14308000	16670000	34491000
Q03001	Q03001	Dystonin	DST	sp|Q03001	46	45	45	0	11	23	41	0	11	22	40	0	11	22	40	9.1	860.65	0	166.72	1.18E+09	0	42852000	62446000	1.07E+09	0	6595600	46717000	36610000
P51812;Q1	P51812	Ribosomal protein S6 kinase alpha-3	RPS6KA3	sp|P51812	17	17	10	0	13	1	17	0	13	1	17	0	9	0	10	28.9	83.735	0	57.6	1.173E+09	0	2.34E+08	1261000	9.38E+08	0	11435000	2172000	46443000
P49903	P49903	Selenide, water dikinase 1	SEPHS1	sp|P49903	7	7	7	1	7	0	5	1	7	0	5	1	7	0	5	32.4	42.91	0	44.092	1.17E+09	5.04E+08	5.09E+08	0	1.57E+08	4.36E+08	50618000	0	68500000
Q16698	Q16698	2,4-dienoyl-CoA reductase, mitochondrial	DECR1	sp|Q1669	9	9	9	1	8	7	8	1	8	7	8	1	8	7	8	34.9	36.067	0	44.901	1.162E+09	2697100	2.86E+08	79240000	7.94E+08	4752100	22381000	58886000	27806000
P21980	P21980	Protein-glutamine gamma-glutamyltransferase 2	TGM2	sp|P21980	14	14	14	0	1	6	14	0	1	6	14	0	1	6	14	31.4	77.328	0	101.76	1.162E+09	0	776790	13930000	1.15E+09	0	220320	11881000	44508000
Q03135;P5	Q03135	Caveolin-1	CAV1	sp|Q0313	7	7	7	0	3	7	7	0	3	7	7	0	3	7	7	45.5	20.471	0	38.829	1.157E+09	0	11332000	101420000	1.04E+09	0	1795200	68798000	41504000
Q96A33	Q96A33	Coiled-coil domain-containing protein 47	CCDC47	sp|Q96A3	11	11	11	1	10	5	10	1	10	5	10	1	10	5	10	31.5	55.873	0	105.99	1.154E+09	259630	4.62E+08	22643000	6.69E+08	812020	48915000	9870000	24143000
Q99436	Q99436	Proteasome subunit beta type-7	PSMB7	sp|Q9943	7	7	7	1	6	5	6	1	6	5	6	1	6	5	6	26.7	29.965	0	83.581	1.153E+09	914720	4.29E+08	27415000	6.96E+08	3527200	30228000	9210800	42771000
Q9NRV9	Q9NRV9	Heme-binding protein 1	HEBP1	sp|Q9NRV	7	7	7	0	7	0	7	0	7	0	7	0	7	0	7	56.6	21.097	0	59.997	1.152E+09	0	2.37E+08	0	9.15E+08	0	20894000	0	37633000
Q9H2U2	Q9H2U2	Inorganic pyrophosphatase 2, mitochondrial	PPA2	sp|Q9H2U	11	11	11	0	8	1	10	0	8	1	10	0	8	1	10	39.2	37.92	0	36.001	1.152E+09	0	2.54E+08	445380	8.97E+08	0	45322000	1572100	12712000
Q3KQV9	Q3KQV9	UDP-N-acetylhexosamine pyrophosphorylase-like	UAP1L1	sp|Q3KQV	11	11	10	0	6	2	11	0	6	2	11	0	5	2	10	23.7	57.03	0	64.787	1.15E+09	0	80434000	5820400	1.06E+09	0	7346300	3901500	43486000
Q12965;O(	Q12965	Unconventional myosin-Ie	MYO1E	sp|Q1296	16	16	16	0	5	2	16	0	5	2	16	0	5	2	16	20.3	127.06	0	136.08	1.146E+09	0	41390000	866780	1.1E+09	0	2343100	676460	46605000
Q9BXP5	Q9BXP5	Serrate RNA effector molecule homolog	SRRT	sp|Q9BXP	14	14	14	1	12	6	13	1	12	6	13	1	12	6	13	22.8	100.67	0	51.405	1.146E+09	920950	5.83E+08	14394000	5.48E+08	4261200	29202000	11570000	39457000
Q9UJ70	Q9UJ70	N-acetyl-D-glucosamine kinase	NAGK	sp|Q9UJ7	9	9	9	0	4	4	9	0	4	4	9	0	4	4	9	42.2	37.375	0	41.706	1.142E+09	0	65178000	15635000	1.06E+09	0	7668700	15456000	36774000
O00116	O00116	Alkyldihydroxyacetonephosphate synthase, perox	AGPS	sp|O0011	12	12	12	0	11	1	12	0	11	1	12	0	11	1	12	28.9	72.911	0	61.812	1.141E+09	0	2.68E+08	1919900	8.71E+08	0	27961000	1778400	31017000
Q9H0D6	Q9H0D6	5-3 exoribonuclease 2	XRN2	sp|Q9H0D	17	17	17	1	14	15	15	1	14	15	15	1	14	15	15	26	108.58	0	64.719	1.138E+09	28605000	3.57E+08	126020000	6.26E+08	35249000	26211000	89050000	20020000
P0DPI2;A0	P0DPI2;A0A0B4J2D5			sp|P0DPI2	8	8	8	1	8	0	8	1	8	0	8	1	8	0	8	40.7	28.17	0	48.956	1.138E+09	3830200	8.33E+08	0	3.01E+08	4126300	78269000	0	7231700
P17252;P0	P17252	Protein kinase C alpha type	PRKCA	sp|P17252	11	11	11	2	4	0	11	2	4	0	11	2	4	0	11	21.7	76.749	0	75.652	1.137E+09	1558600	56443000	0	1.08E+09	1553300	3357300	0	44928000
P21964	P21964	Catechol O-methyltransferase	COMT	sp|P21964	6	6	6	0	4	2	6	0	4	2	6	0	4	2	6	24	30.037	0	21.386	1.136E+09	0	1.55E+08	3370000	9.77E+08	0	16361000	7446000	32322000
Q9UJU6	Q9UJU6	Drebrin-like protein	DBNL	sp|Q9UJU	13	13	13	1	11	2	13	1	11	2	13	1	11	2	13	39.8	48.207	0	101.03	1.136E+09	2238000	3.68E+08	2252300	7.63E+08	2078100	31674000	298050	33530000
P09601	P09601	Heme oxygenase 1	HMOX1	sp|P09601	11	11	11	0	4	1	11	0	4	1	11	0	4	1	11	59	32.818	0	94.906	1.131E+09	0	15679000	1878400	1.11E+09	0	1758900	1074400	45615000
Q9BSD7	Q9BSD7	Cancer-related nucleoside-triphosphatase	NTPCR	sp|Q9BSD	7	7	7	1	7	3	6	1	7	3	6	1	7	3	6	53.2	20.713	0	33.622	1.13E+09	12855000	6.31E+08	11902000	4.74E+08	20253000	45873000	13037000	13236000
P50416	P50416	Carnitine O-palmitoyltransferase 1, liver isoform	CPT1A	sp|P50416	19	19	19	0	5	13	19	0	5	13	19	0	5	13	19	30.9	88.367	0	113.55	1.128E+09	0	17553000	43433000	1.07E+09	0	4215400	20659000	49754000
Q9C0C9	Q9C0C9	E2/E3 hybrid ubiquitin-protein ligase UBE2O	UBE2O	sp|Q9C0C	17	17	17	1	17	1	12	1	17	1	12	1	17	1	12	21.1	141.29	0	78.72	1.126E+09	1997400	7.47E+08	1572900	3.76E+08	9271200	63811000	1646600	9602300
P55263	P55263	Adenosine kinase	ADK	sp|P55263	12	12	12	0	9	1	10	0	9	1	10	0	9	1	10	39.2	40.545	0	116.15	1.126E+09	0	3.55E+08	4148400	7.67E+08	0	41132000	5549100	18931000
Q13057	Q13057	Bifunctional coenzyme A synthase;Phosphopante	COASY	sp|Q13057	9	9	9	0	7	1	9	0	7	1	9	0	7	1	9	27.7	62.328	0	58.708	1.125E+09	0	2.54E+08	1981800	8.69E+08	0	10974000	2589800	45892000
Q9P035	Q9P035	Very-long-chain (3R)-3-hydroxyacyl-CoA dehydrat	HACD3	sp|Q9P03	9	9	9	1	8	6	8	1	8	6	8	1	8	6	8	30.7	43.159	0	32.908	1.124E+09	2681200	4.34E+08	59654000	6.27E+08	11025000	29488000	45071000	21257000
O95292	O95292	Vesicle-associated membrane protein-associated	VAPB	sp|O95292	11	10	10	2	11	4	11	2	10	3	10	2	10	3	10	53.9	27.228	0	42.196	1.12E+09	2024200	3.93E+08	10026000	7.15E+08	2032900	35203000	6195600	29274000
Q9Y2S7	Q9Y2S7	Polymerase delta-interacting protein 2	POLDIP2	sp|Q9Y2S7	9	9	9	0	7	4	9	0	7	4	9	0	7	4	9	35.1	42.033	0	21.636	1.114E+09	0	2.84E+08	22804000	8.08E+08	0	32231000	11494000	29817000
Q9BUJ2	Q9BUJ2	Heterogeneous nuclear ribonucleoprotein U-like	HNRNPUL1	sp|Q9BUJ	17	17	17	4	16	14	15	4	16	14	15	4	16	14	15	28.5	95.737	0	64.889	1.112E+09	15074000	3.87E+08	76882000	6.33E+08	26543000	35193000	45103000	20066000
Q14C86	Q14C86	GTPase-activating protein and VPS9 domain-conta	GAPVD1	sp|Q14C8	22	22	22	2	20	2	18	2	20	2	18	2	20	2	18	23.5	164.98	0	73.577	1.111E+09	1839400	7.07E+08	1746000	4E+08	1303400	67386000	1094800	10858000
Q04323	Q04323	UBX domain-containing protein 1	UBXN1	sp|Q0432	9	9	9	0	8	1	8	0	8	1	8	0	8	1	8	55.2	33.325	0	31.399	1.111E+09	0	4.79E+08	1905000	6.3E+08	0	22935000	4038200	42412000
Q13867	Q13867	Bleomycin hydrolase	BLMH	sp|Q13867	12	12	12	0	11	10	7	0	11	10	7	0	11	10	7	37.1	52.562	0	58.881	1.11E+09	0	7.15E+08	124650000	2.71E+08	0	51240000	93640000	12482000
Q9NVJ2	Q9NVJ2	ADP-ribosylation factor-like protein 8B	ARL8B	sp|Q9NVJ	6	6	3	0	4	2	6	0	4	2	6	0	2	1	3	39.8	21.539	0	100.2	1.109E+09	0	64390000	4341900	1.04E+09	0	11017000	6272600	34083000
Q9ULA0	Q9ULA0	Aspartyl aminopeptidase	DNPEP	sp|Q9ULA	9	9	9	0	3	5	9	0	3	5	9	0	3	5	9	25.8	53.41	0	55.166	1.107E+09	0	22119000	9626300	1.07E+09	0	8029700	7713700	36880000
P04062	P04062	Glucosylceramidase	GBA	sp|P04062	8	8	8	1	6	6	8	1	6	6	8	1	6	6	8	22	59.716	0	49.369	1.104E+09	3.11E+08	72068000	15196000	7.06E+08	2.7E+08	20370000	12354000	53335000
Q8N806	Q8N806	Putative E3 ubiquitin-protein ligase UBR7	UBR7	sp|Q8N80	10	10	10	4	8	10	7	4	8	10	7	4	8	10	7	25.6	47.998	0	97.683	1.104E+09	3.72E+08	1.36E+08	386500000	2.1E+08	4.26E+08	11086000	202710000	11556000
P29144	P29144	Tripeptidyl-peptidase 2	TPP2	sp|P29144	20	20	20	1	14	4	19	1	14	4	19	1	14	4	19	23.5	138.35	0	138.31	1.104E+09	352170	1.64E+08	3899600	9.36E+08	243360	20030000	1691500	33325000
O94973	O94973	AP-2 complex subunit alpha-2	AP2A2	sp|O9497	16	11	11	1	12	3	16	1	8	0	11	1	8	0	11	25.9	103.96	0	77.794	1.101E+09	42542000	1.47E+08	0	9.12E+08	59669000	8100300	0	25241000

Q9Y3B4	Q9Y3B4	Splicing factor 3B subunit 6	SF3B6	sp|Q9Y3B4	3	3	3	0	3	2	2	0	3	2	2	0	3	2	2	26.4	14.585	0	9.757	1.1E+09	0	5.92E+08	41347000	4.67E+08	0	51479000	29232000	18473000
P20020;Q1	P20020	Plasma membrane calcium-transporting ATPase 1	ATP2B1	sp|P20020	15	15	7	0	12	2	14	0	12	2	14	0	5	0	6	18.4	134.68	0	81.296	1.1E+09	0	1.89E+08	1275500	9.09E+08	0	13711000	1437900	39771000
Q96PE2	Q96PE2	Rho guanine nucleotide exchange factor 17	ARHGEF17	sp|Q96PE2	2	2	2	1	0	2	0	1	0	2	0	1	0	2	0	1.3	221.67	0.002869	2.1312	1.1E+09	1.1E+08	0	989910000	0	1.19E+08	0	655840000	0
Q9H7Z7	Q9H7Z7	Prostaglandin E synthase 2;Prostaglandin E syntha	PTGES2	sp|Q9H7Z7	11	11	11	0	11	0	10	0	11	0	10	0	11	0	10	44	41.943	0	91.855	1.098E+09	0	3.63E+08	0	7.35E+08	0	33906000	0	28321000
Q6WCQ1	Q6WCQ1	Myosin phosphatase Rho-interacting protein	MPRIP	sp|Q6WCQ	21	21	21	1	9	21	17	1	9	21	17	1	9	21	17	28.3	116.53	0	93.62	1.097E+09	12598000	64363000	238830000	7.81E+08	8737200	8434100	175490000	18243000
P35249	P35249	Replication factor C subunit 4	RFC4	sp|P35249	9	9	9	1	9	7	9	1	9	7	9	1	9	7	9	35.5	39.681	0	88.55	1.094E+09	359590	3.34E+08	42809000	7.17E+08	791320	38630000	13995000	34630000
O15511	O15511	Actin-related protein 2/3 complex subunit 5	ARPC5	sp|O15511	8	8	6	0	7	2	7	0	7	2	7	0	5	2	6	64.9	16.32	0	23.579	1.093E+09	0	2.09E+08	4371000	8.8E+08	0	11410000	5206200	36236000
Q9UBF2	Q9UBF2	Coatomer subunit gamma-2	COPG2	sp|Q9UBF2	17	16	16	1	14	6	16	1	13	5	15	1	13	5	15	27.1	97.621	0	76.345	1.093E+09	884700	2.89E+08	8281100	7.95E+08	843610	29962000	2119100	31696000
P09661	P09661	U2 small nuclear ribonucleoprotein A	SNRPA1	sp|P09661	10	10	10	4	10	5	8	4	10	5	8	4	10	5	8	44.3	28.415	0	53.215	1.092E+09	31927000	5.24E+08	28970000	5.06E+08	29477000	45481000	25188000	17615000
Q13045	Q13045	Protein flightless-1 homolog	FLII	sp|Q13045	15	15	15	0	7	13	13	0	7	13	13	0	7	13	13	19	144.75	0	120.09	1.09E+09	0	2.28E+08	64131000	7.98E+08	0	17404000	43162000	35474000
Q14558	Q14558	Phosphoribosyl pyrophosphate synthase-associat	PRPSAP1	sp|Q14558	10	10	9	0	8	3	9	0	8	3	9	0	7	2	8	40.2	39.393	0	66.684	1.089E+09	0	4.59E+08	5701200	6.25E+08	0	27698000	17860000	20076000
Q9Y2B0	Q9Y2B0	Protein canopy homolog 2	CNPY2	sp|Q9Y2B0	5	5	5	1	5	1	5	1	5	1	5	1	5	1	5	37.9	20.652	0	19.999	1.089E+09	1740900	3.75E+08	999060	7.11E+08	2662900	31646000	2011700	21720000
Q8TEQ6	Q8TEQ6	Gem-associated protein 5	GEMIN5	sp|Q8TEQ6	20	20	20	0	18	9	19	0	18	9	19	0	18	9	19	18.5	168.59	0	68.705	1.089E+09	0	3.22E+08	37610000	7.29E+08	0	28002000	27653000	27981000
Q5SW79;C	Q5SW79	Centrosomal protein of 170 kDa	CEP170	sp|Q5SW7	24	24	24	5	19	19	22	5	19	19	22	5	19	19	22	26.5	175.29	0	81.826	1.088E+09	10599000	2.48E+08	87825000	7.42E+08	8749000	10095000	80676000	22461000
Q9BWM7	Q9BWM7	Sideroflexin-3	SFXN3	sp|Q9BWM	10	10	10	1	3	6	9	1	3	6	9	1	3	6	9	38	35.503	0	34.789	1.088E+09	232620	1.34E+08	49173000	9.04E+08	1437000	8339900	16938000	55553000
Q92538	Q92538	Golgi-specific brefeldin A-resistance guanine nucl	GBF1	sp|Q92538	22	22	22	0	12	6	21	0	12	6	21	0	12	6	21	18.5	206.44	0	107.6	1.087E+09	0	1.29E+08	13716000	9.44E+08	0	17030000	7184900	35195000
Q8TC12;Q	Q8TC12	Retinol dehydrogenase 11	RDH11	sp|Q8TC12	8	8	8	1	5	2	8	1	5	2	8	1	5	2	8	28.9	35.386	0	33.73	1.085E+09	956780	1.54E+08	2004300	9.28E+08	1092400	11791000	2085700	39076000
Q14011	Q14011	Cold-inducible RNA-binding protein	CIRBP	sp|Q14011	5	5	5	0	5	5	5	0	5	5	5	0	5	5	5	38.4	18.648	0	76.762	1.075E+09	0	7.44E+08	23560000	3.07E+08	0	47003000	27652000	19401000
P21291	P21291	Cysteine and glycine-rich protein 1	CSRP1	sp|P21291	6	6	6	1	0	3	6	1	0	3	6	1	0	3	6	50.8	20.567	0	59.474	1.071E+09	8557900	0	14166000	1.05E+09	7874800	0	10536000	42780000
Q8TBC4	Q8TBC4	NEDD8-activating enzyme E1 catalytic subunit	UBA3	sp|Q8TBC4	7	7	7	0	7	0	5	0	7	0	5	0	7	0	5	26.3	51.852	0	67.052	1.071E+09	0	5.2E+08	0	5.5E+08	0	43968000	0	24523000
Q13547	Q13547	Histone deacetylase 1	HDAC1	sp|Q13547	9	9	5	1	8	3	7	1	8	3	7	0	4	1	4	32.2	55.102	0	48.407	1.069E+09	364980	5.38E+08	13191000	5.18E+08	975700	60524000	6768900	9658100
Q8WWM7	Q8WWM7	Ataxin-2-like protein	ATXN2L	sp|Q8WW	19	19	19	3	15	13	18	3	15	13	18	3	15	13	18	27.5	113.37	0	67.327	1.068E+09	5817500	2.56E+08	36880000	7.7E+08	5529000	20793000	21083000	37389000
Q14980	Q14980	Nuclear mitotic apparatus protein 1	NUMA1	sp|Q14980	26	26	26	1	15	16	26	1	15	16	26	1	15	16	26	19.1	238.26	0	116.55	1.067E+09	1567700	1.16E+08	41091000	9.09E+08	3728800	13306000	25933000	33394000
P29966	P29966	Myristoylated alanine-rich C-kinase substrate	MARCKS	sp|P29966	9	9	9	1	8	5	9	1	8	5	9	1	8	5	9	44	31.554	0	40.341	1.064E+09	1385400	3.49E+08	12290000	7.01E+08	1276400	29862000	7553300	26359000
P52888	P52888	Thimet oligopeptidase	THOP1	sp|P52888	14	14	14	0	12	0	14	0	12	0	14	0	12	0	14	21.8	78.839	0	58.531	1.059E+09	0	2.86E+08	0	7.73E+08	0	26387000	0	30578000
Q9Y3U8	Q9Y3U8	60S ribosomal protein L36	RPL36	sp|Q9Y3U8	6	6	6	0	5	5	5	0	5	5	5	0	5	5	5	37.1	12.254	0	12.723	1.056E+09	0	3.06E+08	193520000	5.57E+08	0	29852000	120920000	26333000
Q92734	Q92734	Protein TFG	TFG	sp|Q92734	8	8	8	1	8	1	8	1	8	1	8	1	8	1	8	28.5	43.447	0	85.079	1.056E+09	32328000	4.09E+08	2812700	6.11E+08	20143000	39456000	3034400	32786000
Q9Y4W6	Q9Y4W6	AFG3-like protein 2	AFG3L2	sp|Q9Y4W	20	20	19	1	20	7	17	1	20	7	17	1	19	6	16	30.4	88.583	0	98.991	1.053E+09	618670	5.73E+08	16364000	4.63E+08	993310	51999000	8399000	19782000
Q9HCJ6	Q9HCJ6	Synaptic vesicle membrane protein VAT-1 homolo	VAT1L	sp|Q9HCJ6	11	11	11	0	0	1	11	0	0	1	11	0	0	1	11	34.8	45.899	0	77.451	1.053E+09	0	0	1237500	1.05E+09	0	0	884130	43191000
P14174	P14174	Macrophage migration inhibitory factor	MIF	sp|P14174	2	2	2	0	2	0	2	0	2	0	2	0	2	0	2	27.8	12.476	0	18.43	1.05E+09	0	7.88E+08	0	2.62E+08	0	70568000	0	8290200
P47813;O1	P47813;O1	Eukaryotic translation initiation factor 1A, X-chror	EIF1AX;EIF	sp|P47813	5	5	5	0	5	2	5	0	5	2	5	0	5	2	5	36.1	16.46	0	24.058	1.048E+09	0	4.88E+08	24614000	5.35E+08	0	43077000	23458000	15039000
P15153	P15153	Ras-related C3 botulinum toxin substrate 2	RAC2	sp|P15153	6	2	2	0	4	5	6	0	0	2	2	0	0	2	2	32.3	21.429	0	8.783	1.047E+09	0	0	6253300	1.04E+09	0	0	4979200	42031000
Q96T76	Q96T76	MMS19 nucleotide excision repair protein homol	MMS19	sp|Q96T76	15	15	15	0	14	6	12	0	14	6	12	0	14	6	12	20.3	113.29	0	63.724	1.046E+09	0	3.63E+08	15571000	6.67E+08	0	29913000	15742000	24243000
O14786	O14786	Neuropilin-1	NRP1	sp|O14786	16	16	16	1	1	3	16	1	1	3	16	1	1	3	16	28.2	103.13	0	71.516	1.045E+09	312560	1820000	8177600	1.04E+09	199710	143080	932700	47261000
P35610	P35610	Sterol O-acyltransferase 1	SOAT1	sp|P35610	4	4	4	0	3	2	4	0	3	2	4	0	3	2	4	9.8	64.734	0	94.66	1.045E+09	0	78777000	21747000	9.44E+08	0	6688000	19836000	33857000
P20337	P20337	Ras-related protein Rab-3B	RAB3B	sp|P20337	5	4	4	0	1	3	5	0	1	3	4	0	1	3	4	40.6	24.758	0	58.488	1.043E+09	0	14397000	10957000	1.02E+09	0	1583600	4882000	43445000
P49821	P49821	NADH dehydrogenase [ubiquinone] flavoprotein :	NDUFV1	sp|P49821	9	9	9	0	8	3	8	0	8	3	8	0	8	3	8	26.1	50.817	0	33.163	1.04E+09	0	3.16E+08	10713000	7.14E+08	0	27689000	6593400	30092000
P08574	P08574	Cytochrome c1, heme protein, mitochondrial	CYC1	sp|P08574	6	6	6	0	6	4	6	0	6	4	6	0	6	4	6	28	35.422	0	32.415	1.038E+09	0	3.54E+08	23367000	6.6E+08	0	29345000	16127000	27808000
Q9Y224	Q9Y224	UPF0568 protein C14orf166	C14orf166	sp|Q9Y224	8	8	8	4	6	6	7	4	6	6	7	4	6	6	7	43.9	28.068	0	30.455	1.036E+09	2.05E+08	2.06E+08	87891000	5.37E+08	2.28E+08	14613000	46521000	13651000
Q6UB35	Q6UB35	Monofunctional C1-tetrahydrofolate synthase, mi	MTHFD1L	sp|Q6UB35	14	14	14	0	13	2	12	0	13	2	12	0	13	2	12	18.1	105.79	0	52.721	1.035E+09	0	4.88E+08	2329100	5.44E+08	0	41688000	1486200	23799000
Q9Y4K1	Q9Y4K1	Absent in melanoma 1 protein	AIM1	sp|Q9Y4K1	25	25	25	0	1	12	25	0	1	12	25	0	1	12	25	23.4	188.67	0	144.4	1.033E+09	0	4179300	44551000	9.84E+08	0	1295200	14751000	54730000
P36543;Q	P36543	V-type proton ATPase subunit E 1	ATP6V1E1	sp|P36543	10	10	10	1	7	1	7	1	7	1	7	1	7	1	7	40.7	26.145	0	29.811	1.03E+09	6213100	1.91E+08	511620	8.32E+08	0	21831000	0	29216000
Q96A72	Q96A72	Protein mago nashi homolog 2	MAGOHB	sp|Q96A72	7	7	1	4	7	5	7	4	7	5	7	0	1	1	1	53.4	17.276	0	45.948	1.025E+09	37199000	4.81E+08	20155000	4.86E+08	46959000	32332000	16800000	14921000
Q9UKF6	Q9UKF6	Cleavage and polyadenylation specificity factor su	CPSF3	sp|Q9UKF6	9	9	9	0	8	3	9	0	8	3	9	0	8	3	9	25.4	77.485	0	68.781	1.023E+09	0	2.29E+08	6738600	7.88E+08	0	17259000	4509500	35333000
Q14669	Q14669	E3 ubiquitin-protein ligase TRIP12	TRIP12	sp|Q14669	18	18	18	1	8	13	17	1	8	13	17	1	8	13	17	13.1	220.43	0	66.621	1.022E+09	897030	73940000	40190000	9.07E+08	2026200	5109300	25536000	39061000
Q99685	Q99685	Monoglyceride lipase	MGLL	sp|Q99685	7	7	7	0	0	0	7	0	0	0	7	0	0	0	7	33.7	33.261	0	53.979	1.021E+09	0	0	0	1.02E+09	0	0	0	42001000
Q9NZL9	Q9NZL9	Methionine adenosyltransferase 2 subunit beta	MAT2B	sp|Q9NZL9	9	9	9	1	9	1	9	1	9	1	9	1	9	1	9	38	37.551	0	86.438	1.021E+09	9209500	3.67E+08	441920	6.44E+08	7165500	50882000	1286700	9016600
Q9Y5M8	Q9Y5M8	Signal recognition particle receptor subunit beta	SRPRB	sp|Q9Y5M	8	8	8	1	8	3	8	1	8	3	8	1	8	3	8	38.7	29.702	0	85.272	1.021E+09	923390	5.76E+08	7505300	4.36E+08	1458000	53040000	4775700	15426000
P51570	P51570	Galactokinase	GALK1	sp|P51570	9	9	9	0	9	2	7	0	9	2	7	0	9	2	7	28.3	42.272	0	37.067	1.018E+09	0	4.23E+08	4552900	5.91E+08	0	49196000	2108000	13328000
O75964	O75964	ATP synthase subunit g, mitochondrial	ATP5L	sp|O75964	5	5	5	1	5	3	5	1	5	3	5	1	5	3	5	46.6	11.428	0	19.489	1.018E+09	28469000	4.5E+08	9557300	5.3E+08	39166000	33648000	4705200	18811000
P61081	P61081	NEDD8-conjugating enzyme Ubc12	UBE2M	sp|P61081	8	8	8	0	8	1	8	0	8	1	8	0	8	1	8	40.4	20.9	0	46.002	1.016E+09	0	4.76E+08	295020	5.4E+08	0	16065000	1193700	47128000
Q92905	Q92905	COP9 signalosome complex subunit 5	COPS5	sp|Q92905	13	13	13	0	12	3	12	0	12	3	12	0	12	3	12	52.4	37.578	0	73.005	1.015E+09	0	4.45E+08	3112500	5.67E+08	0	34333000	2314200	28003000
Q9C0C2	Q9C0C2	182 kDa tankyrase-1-binding protein	TNKS1BP1	sp|Q9C0C2	26	26	26	1	7	15	24	1	7	15	24	1	7	15	24	27.2	181.79	0	155.51	1.015E+09	200150	31211000	97668000	8.86E+08	878750	1758800	44015000	58367000
O75521	O75521	Enoyl-CoA delta isomerase 2, mitochondrial	ECI2	sp|O75521	9	9	9	0	9	1	8	0	9	1	8	0	9	1	8	30.2	43.585	0	45.502	1.013E+09	0	4.73E+08	602180	5.39E+08	0	27050000	2300600	34962000
P51970	P51970	NADH dehydrogenase [ubiquinone] 1 alpha subcc	NDUFA8	sp|P51970	5	5	5	2	5	3	4	2	5	3	4	2	5	3	4	37.8	20.105	0	33.846	1.013E+09	3154400	4.93E+08	7733400	5.1E+08	5870300	49738000	5219700	11901000
Q9BTW9	Q9BTW9	Tubulin-specific chaperone D	TBCD	sp|Q9BTW	18	18	18	0	14	3	18	0	14	3	18	0	14	3	18	20.7	132.6	0	94.265	1.011E+09	0	1.86E+08	3932900	8.22E+08	0	14401000	4883500	33523000
P61923	P61923	Coatomer subunit zeta-1	COPZ1	sp|P61923	6	6	6	0	5	1	6	0	5	1	6	0	5	1	6	41.8	20.198	0	23.097	1.009E+09	0	1.26E+08	1065200	8.82E+08	0	14125000	1410600	32568000
Q01780	Q01780	Exosome component 10	EXOSC10	sp|Q01780	14	14	14	1	11	13	13	1	11	13	13	1	11	13	13	21.4	100.83	0	49.015	1.008E+09	3694500	2.38E+08	77471000	6.89E+08	13715000	19081000	47325000	24607000
Q9BV73	Q9BV73	Centrosome-associated protein CEP250	CEP250	sp|Q9BV73	6	6	6	0	1	0	6	0	1	0	6	0	1	0	6	3.9	281.13	0	9.7017	1.008E+09	0	2.01E+08	0	8.07E+08	0	17798000	0	33105000
O14828	O14828	Secretory carrier-associated membrane protein 3	SCAMP3	sp|O14828	8	8	8	0	8	3	8	0	8	3	8	0	8	3	8	38.9	38.287	0	50.286	1.007E+09	0	2.55E+08	10040000	7.42E+08	0	17048000	15348000	27035000
P51398	P51398	28S ribosomal protein S29, mitochondrial	DAP3	sp|P51398	10	10	10	1	9	3	10	1	9	3	10	1	9	3	10	32.9	45.566	0	39.524	1.006E+09	13153000	4.29E+08	12519000	5.52E+08	25594000	26149000	16446000	13840000
Q9NQG5	Q9NQG5	Regulation of nuclear pre-mRNA domain-containi	RPRD1B	sp|Q9NQG	9	6	6	0	8	2	8	0	6	2	5	0	6	2	5	35.3	36.899	0	22.725	1.003E+09	0	3.53E+08	4311100	6.46E+08	0	31952000	10056000	18554000
O95793	O95793	Double-stranded RNA-binding protein Staufen ho	STAU1	sp|O95793	19	19	17	6	13	18	16	6	13	18	16	6	11	16	14	42.6	63.182	0	68.269	999300000	68578000	1.71E+08	183600000	5.77E+08	73605000	16664000	117280000	23155000
P27169	P27169	Serum paraoxonase/arylesterase 1	PON1	sp|P27169	3	3	3	0	2	3	1	0	2	3	1	0	2	3	1	10.4	39.731	0	10.768	998990000	0	6.18E+08	35384000	3.45E+08	0	10902000	55678000	25897000
Q9UBS4	Q9UBS4	DnaJ homolog subfamily B member 11	DNAJB11	sp|Q9UBS4	12	12	12	0	10	8	12	0	10	8	12	0	10	8	12	36	40.513	0	56.516	998410000	0	2.93E+08	24229000	6.82E+08	0	25907000	20610000	23589000
Q13618	Q13618	Cullin-3	CUL3	sp|Q13618	11	11	11	0	10	0	10	0	10	0	10	0	10	0	10	20.2	88.929	0	52.304	996970000	0	2.31E+08	0	7.66E+08	0	25957000	0	25900000
O95352	O95352	Ubiquitin-like modifier-activating enzyme ATG7	ATG7	sp|O95352	15	15	15	0	6	0	15	0	6	0	15	0	6	0	15	27.6	77.959	0	64.17	995700000	0	46740000	0	9.49E+08	0	9707900	0	32951000
Q15125	Q15125	3-beta-hydroxysteroid-Delta(8),Delta(7)-isomeras	EBP	sp|Q15125	3	3	3	0	3	2	3	0	3	2	3	0	3	2	3	16.5	26.352	0	13.34	994480000	0	2.43E+08	9838500	7.42E+08	0	24590000	4866700	27661000
Q86Y56	Q86Y56	Dynein assembly factor 5, axonemal	DNAAF5	sp|Q86Y56	18	18	18	0	17	4	15	0	17	4	15	0	17	4	15	30.2	93.52	0	118.47	993780000	0	4.38E+08	21191000	5.35E+08	0	35433000	14813000	24573000
O43264	O43264	Centromere/kinetochore protein zw10 homolog	ZW10	sp|O43264	10	10	10	0	10	3	8	0	10	3	8	0	10	3	8	17.6	88.828	0	52.403	993530000	0	4.55E+08	15162000	5.24E+08	0	36768000	19806000	15232000
Q9Y2Q3	Q9Y2Q3	Glutathione S-transferase kappa 1	GSTK1	sp|Q9Y2Q3	6	6	6	0	4	3	6	0	4	3	6	0	4	3	6	39.8	25.497	0	36.047	991040000	0	1.7E+08	5517100	8.16E+08	0	21455000	4558900	26218000
Q8TCJ2	Q8TCJ2	Dolichyl-diphosphooligosaccharide--protein glyco	STT3B	sp|Q8TCJ2	12	12	11	0	5	8	12	0	5	8	12	0	4	7	11	14.6	93.673	0	34.575	990060000	0	55624000	30626000	9.04E+08	0	6097200	22678000	33879000
P20073	P20073	Annexin A7	ANXA7	sp|P20073	11	11	11	0	10	3	11	0	10	3	11	0	10	3	11	24.2	52.739	0	37.876	990030000	0	2.72E+08	7628400	7.1E+08	0	24665000	9044800	24614000
Q9H857	Q9H857	5-nucleotidase domain-containing protein 2	NT5DC2	sp|Q9H857	10	10	10	0	6	4	10	0	6	4	10	0	6	4	10	28.7	60.718	0	55.42	985060000	0	1.15E+08	12521000	8.58E+08	0	10322000	17363000	25791000
P13674	P13674	Prolyl 4-hydroxylase subunit alpha-1	P4HA1	sp|P13674	12	12	12	1	9	2	9	1	9	2	9	1	9	2	9	39	61.049	0	94.875	984730000	6007100	3.11E+08	5301400	6.62E+08	7467600	26168000	4035200	26380000
Q8IYD1	Q8IYD1	Eukaryotic peptide chain release factor GTP-bindi	GSPT2	sp|Q8IYD1	11	3	3	0	9	1	11	0	1	0	3	0	1	0	3	25.6	68.882	0	13.17	984070000	0	91618000	0	8.92E+08	0	8364500	0	36414000
P10620	P10620	Microsomal glutathione S-transferase 1	MGST1	sp|P10620	4	4	4	0	1	1	4	0	1	1	4	0	1	1	4	27.1	17.598	0	27.413	982570000	0	11917000	488780	9.7E+08	0	396870	221920	40349000
P05120	P05120	Plasminogen activator inhibitor 2	SERPINB2	sp|P05120	10	10	10	0	1	3	10	0	1	3	10	0	1	3	10	33.3	46.596	0	89.385	982320000	0	5560400	8841600	9.68E+08	0	0	7687200	36524000
Q9NRW7	Q9NRW7	Vacuolar protein sorting-associated protein 45	VPS45	sp|Q9NRW	15	15	15	0	9	1	15	0	9	1	15	0	9	1	15	30.4	65.076	0	98.35	982200000	0	99751000	1254000	8.81E+08	0	6221600	1364600	38289000
Q92621	Q92621	Nuclear pore complex protein Nup205	NUP205	sp|Q92621	19	19	19	2	7	4	19	2	7	4	19	2	7	4	19	13.5	227.92	0	85.825	982040000	10010000	67779000	8125300	8.96E+08	7876400	7385200	12562000	30475000
O60684;O	O60684;O1	Importin subunit alpha-7;Importin subunit alpha-	KPNA6;KPI	sp|O60684	12	12	8	2	8	4	11	2	8	4	11	1	6	3	7	24.6	60.029	0	121.6	981460000	4449000	3.02E+08	26723000	6.48E+08	8825600	33876000	10097000	22883000
Q92552	Q92552	28S ribosomal protein S27, mitochondrial	MRPS27	sp|Q92552	10	10	10	2	9	4	8	2	9	4	8	2	9	4	8	25.1	47.611	0	22.478	980460000	18386000	6.69E+08	4786000	2.88E+08	19641000	48832000	13523000	10441000
P28482	P28482	Mitogen-activated protein kinase 1	MAPK1	sp|P28482	10	7	7	4	8	1	10	2	5	0	7	2	5	0	7	40.3	41.389	0	70.892	979640000	16693000	3.02E+08	0	6.61E+08	20574000	22976000	0	26960000
Q9NUQ9;C	Q9NUQ9	Protein FAM49B	FAM49B	sp|Q9NUQ	8	8	8	0	8	0	7	0	8	0	7	0	8	0	7	31.5	36.748	0	34.593	977310000	0	5.17E+08	0	4.6E+08	0	45551000	0	18961000
P36551	P36551	Oxygen-dependent coproporphyrinogen-III oxida	CPOX	sp|P36551	9	9	9	0	9	1	8	0	9	1	8	0	9	1	8	24.2	50.151	0	34.44	977030000	0	3.81E+08	552650	5.95E+08	0	47493000	1234400	9723500
Q5GLZ8	Q5GLZ8	Probable E3 ubiquitin-protein ligase HERC4	HERC4	sp|Q5GLZ8	15	15	15	0	3	1	15	0	3	1	15	0	3	1	15	21	118.56	0	75.47	975740000	0	10164000	575220	9.65E+08	0	463550	270330	39644000
O43795	O43795	Unconventional myosin-Ib	MYO1B	sp|O43795	15	15	14	1	9	9	15	1	9	9	15	1	8	8	14	16.7	131.98	0	54.554	974380000	436190	1.03E+08	26432000	8.45E+08	7831000	7738300	22553000	23877000
Q9BT78	Q9BT78	COP9 signalosome complex subunit 4	COPS4	sp|Q9BT78	11	11	11	1	11	1	11	1	11	1	11	1	11	1	11	38.7	46.268	0	75.421	973670000	602460	2.12E+08	2965000	7.58E+08	1093300	5706100	1988200	43658000
O14773	O14773	Tripeptidyl-peptidase 1	TPP1	sp|O14773	8	8	8	0	7	6	7	0	7	6	7	0	7	6	7	21.7	61.247	0	56.756	972960000	0	1.45E+08	22803000	8.05E+08	0	14555000	20484000	26106000

Q05519	Q05519	Serine/arginine-rich splicing factor 11	SRSF11	sp|Q05519	7	7	7	2	6	3	5	2	6	3	5	2	6	3	5	24.4	53.542	0	19.201	971000000	25664000	5.8E+08	24306000	3.41E+08	60482000	27810000	10246000	8608000
Q9UNF1;Q	Q9UNF1	Melanoma-associated antigen D2	MAGED2	sp|Q9UNF	14	14	13	0	12	7	14	0	12	7	14	0	11	6	13	37.3	64.953	0	88.289	970340000	0	1.66E+08	17252000	7.87E+08	0	16121000	19239000	23251000
Q7L5N1	Q7L5N1	COP9 signalosome complex subunit 6	COPS6	sp|Q7L5N1	9	9	9	0	7	1	9	0	7	1	9	0	7	1	9	31.5	36.163	0	44.043	970130000	0	2.42E+08	407050	7.28E+08	0	39194000	1357300	10966000
O00203;Q	O00203	AP-3 complex subunit beta-1	AP3B1	sp|O00203	18	18	18	2	15	7	14	2	15	7	14	2	15	7	14	17.9	121.32	0	75.201	969820000	1761000	2.53E+08	28599000	6.87E+08	2625100	38301000	11891000	18683000
P43034	P43034	Platelet-activating factor acetylhydrolase IB subur	PAFAH1B1	sp|P43034	11	11	11	0	11	4	11	0	11	4	11	0	11	4	11	28.8	46.637	0	41.265	968990000	0	2.5E+08	5703900	7.13E+08	0	21342000	7204300	26667000
P54687	P54687	Branched-chain-amino-acid aminotransferase, cyt	BCAT1	sp|P54687	8	8	8	0	6	0	7	0	6	0	7	0	6	0	7	23.3	42.966	0	79.803	967410000	0	2.09E+08	0	7.58E+08	0	19375000	0	30246000
Q96HC4	Q96HC4	PDZ and LIM domain protein 5	PDLIM5	sp|Q96HC4	14	14	14	0	8	7	13	0	8	7	13	0	8	7	13	33.7	63.944	0	60.733	965670000	0	51008000	14640000	9E+08	0	8764300	10614000	31970000
P25685	P25685	DnaJ homolog subfamily B member 1	DNAJB1	sp|P25685	11	11	11	0	11	4	11	0	11	4	11	0	11	4	11	42.1	38.044	0	36.833	963510000	0	2.54E+08	4897400	7.04E+08	0	22218000	7406300	25046000
P43897	P43897	Elongation factor Ts, mitochondrial	TSFM	sp|P43897	6	6	6	0	5	0	6	0	5	0	6	0	5	0	6	29.8	35.39	0	80.717	963280000	0	4.44E+08	0	5.19E+08	0	39861000	0	20627000
Q96K17	Q96K17	Transcription factor BTF3 homolog 4	BTF3L4	sp|Q96K17	8	7	7	0	8	1	6	0	7	1	5	0	7	1	5	60.1	17.27	0	63.392	961250000	0	5.27E+08	20477000	4.14E+08	0	38450000	15283000	23478000
O95163	O95163	Elongator complex protein 1	IKBKAP	sp|O95163	19	19	19	1	19	2	15	1	19	2	15	1	19	2	15	22.4	150.25	0	75.71	960260000	1853000	5.18E+08	6930700	4.33E+08	2240100	47682000	3069900	17010000
Q14696	Q14696	LDLR chaperone MESD	MESDC2	sp|Q14696	5	5	5	0	5	0	5	0	5	0	5	0	5	0	5	33.3	26.076	0	55.538	959920000	0	2.96E+08	0	6.64E+08	0	25951000	0	25752000
Q99459	Q99459	Cell division cycle 5-like protein	CDC5L	sp|Q99459	18	18	18	1	16	17	12	1	16	17	12	1	16	17	12	31.7	92.25	0	64.841	959710000	832180	3.74E+08	90035000	4.95E+08	3276600	20733000	79478000	11146000
Q9BZX2	Q9BZX2	Uridine-cytidine kinase 2	UCK2	sp|Q9BZX2	9	9	8	0	8	4	8	0	8	4	8	0	7	3	7	60.9	29.299	0	38.698	955610000	0	3.61E+08	18925000	5.76E+08	0	25871000	17581000	24739000
Q99961;Q	Q99961	Endophilin-A2	SH3GL1	sp|Q99961	9	9	9	2	9	0	8	2	9	0	8	2	9	0	8	36.4	41.489	0	43.091	955200000	663580	4.56E+08	0	4.98E+08	1805400	46945000	0	8110100
Q9H4A4	Q9H4A4	Aminopeptidase B	RNPEP	sp|Q9H4A4	15	15	15	1	15	1	11	1	15	1	11	1	15	1	11	28.5	72.595	0	70.921	946840000	1431900	5.72E+08	1274600	3.73E+08	1438000	61563000	1112200	3880600
Q9UBB4	Q9UBB4	Ataxin-10	ATXN10	sp|Q9UBB4	11	11	11	0	11	1	10	0	11	1	10	0	11	1	10	28.8	53.488	0	50.011	946180000	0	3.2E+08	1222600	6.25E+08	0	27203000	913320	24506000
Q9NP72	Q9NP72	Ras-related protein Rab-18	RAB18	sp|Q9NP72	8	8	8	1	8	4	8	1	8	4	8	1	8	4	8	47.6	22.977	0	33.273	945160000	291830	1.97E+08	9314300	7.39E+08	1822700	27071000	5608700	19784000
Q8NE71	Q8NE71	ATP-binding cassette sub-family F member 1	ABCF1	sp|Q8NE71	16	16	16	3	15	10	15	3	15	10	15	3	15	10	15	26.4	95.925	0	62.487	942500000	16698000	4.79E+08	23912000	4.23E+08	22360000	37609000	19219000	11777000
Q7L5D6	Q7L5D6	Golgi to ER traffic protein 4 homolog	GET4	sp|Q7L5D6	10	10	10	5	7	10	6	5	7	10	6	5	7	10	6	29.1	36.504	0	36.362	941620000	46426000	1.34E+08	497560000	2.64E+08	76708000	10454000	302400000	10478000
P51153	P51153	Ras-related protein Rab-13	RAB13	sp|P51153	9	7	6	0	3	5	9	0	1	3	7	0	1	3	6	48.8	22.774	0	44.445	938640000	0	20215000	28448000	8.9E+08	0	2742400	7628800	47128000
Q15006	Q15006	ER membrane protein complex subunit 2	EMC2	sp|Q15006	9	9	9	1	8	3	8	1	8	3	8	1	8	3	8	47.1	34.833	0	121.53	936910000	16436000	2.2E+08	3517300	6.97E+08	0	28614000	2412400	19396000
P26885	P26885	Peptidyl-prolyl cis-trans isomerase FKBP2	FKBP2	sp|P26885	6	6	6	1	6	1	6	1	6	1	6	1	6	1	6	57.7	15.649	0	36.758	936440000	1097700	3.74E+08	2149400	5.59E+08	888970	28984000	1067400	27577000
P21283	P21283	V-type proton ATPase subunit C 1	ATP6V1C1	sp|P21283	14	14	14	0	10	2	12	0	10	2	12	0	10	2	12	47.9	43.941	0	48.852	935790000	0	1.96E+08	2639300	7.37E+08	0	10260000	3000100	35839000
Q9Y6Y8	Q9Y6Y8	SEC23-interacting protein	SEC23IP	sp|Q9Y6Y8	14	14	14	1	12	0	12	1	12	0	12	1	12	0	12	17.9	111.08	0	39.249	933440000	332850	3.31E+08	0	6.02E+08	1132200	44601000	0	8552200
O75844	O75844	CAAX prenyl protease 1 homolog	ZMPSTE24	sp|O75844	11	11	11	0	8	0	11	0	8	0	11	0	8	0	11	32.4	54.812	0	64.202	933020000	0	3.24E+08	0	6.09E+08	0	30259000	0	23343000
Q8IWE2	Q8IWE2	Protein NOXP20	FAM114A1	sp|Q8IWE2	8	8	8	0	0	1	8	0	0	1	8	0	0	1	8	22.7	60.741	0	71.866	930990000	0	0	711030	9.3E+08	0	0	477810	38258000
Q14978	Q14978	Nucleolar and coiled-body phosphoprotein 1	NOLC1	sp|Q14978	16	16	16	4	14	14	12	4	14	14	12	4	14	14	12	24.3	73.602	0	47.983	922850000	37508000	4.58E+08	86735000	3.4E+08	45703000	40725000	48176000	14717000
Q9H3K6	Q9H3K6	BolA-like protein 2	BOLA2	sp|Q9H3K6	4	4	4	2	4	1	4	2	4	1	4	2	4	1	4	50	10.116	0	33.287	922480000	2482500	4.18E+08	1031700	5.01E+08	3948500	38455000	1036400	17184000
Q16629	Q16629	Serine/arginine-rich splicing factor 7	SRSF7	sp|Q16629	5	4	4	1	3	4	5	1	2	3	4	1	2	3	4	22.3	27.366	0	14.827	921840000	3359700	3.31E+08	81821000	5.05E+08	13864000	25959000	44028000	24487000
P08397	P08397	Porphobilinogen deaminase	HMBS	sp|P08397	8	8	8	0	8	0	7	0	8	0	7	0	8	0	7	27.7	39.33	0	46.566	920680000	0	4.09E+08	0	5.11E+08	0	38083000	0	16706000
P05386	P05386	60S acidic ribosomal protein P1	RPLP1	sp|P05386	1	1	1	0	1	1	1	0	1	1	1	0	1	1	1	14	11.514	0	5.6563	920250000	0	4.27E+08	57283000	4.35E+08	0	37212000	39467000	17407000
Q9NS69	Q9NS69	Mitochondrial import receptor subunit TOM22 hc	TOMM22	sp|Q9NS69	6	6	6	1	4	2	6	1	4	2	6	1	4	2	6	66.2	15.521	0	51.599	919630000	2959800	2.08E+08	7761000	7.01E+08	3880700	21929000	3923700	25597000
Q9H444;P	Q9H444	Charged multivesicular body protein 4b	CHMP4B	sp|Q9H444	6	6	6	1	5	4	6	1	5	4	6	1	5	4	6	29	24.95	0	23.223	918940000	366380	2.22E+08	14685000	6.82E+08	1557200	30358000	8112500	13353000
Q9P2R3	Q9P2R3	Rabankyrin-5	ANKFY1	sp|Q9P2R3	16	16	16	0	12	1	16	0	12	1	16	0	12	1	16	21.2	128.4	0	66.17	918880000	0	1.54E+08	1514500	7.63E+08	0	7465200	2093900	36439000
Q9NTZ6	Q9NTZ6	RNA-binding protein 12	RBM12	sp|Q9NTZ6	14	14	14	0	14	1	14	0	14	1	14	0	14	1	14	17.7	97.394	0	33.9	917440000	0	3.45E+08	2176200	5.7E+08	0	20735000	5336000	28648000
O95232	O95232	Luc7-like protein 3	LUC7L3	sp|O95232	8	8	8	2	8	4	6	2	8	4	6	2	8	4	6	24.3	51.466	0	44.128	914350000	13287000	3.05E+08	20460000	5.75E+08	28312000	20030000	19238000	9028000
Q9UNZ2	Q9UNZ2	NSFL1 cofactor p47	NSFL1C	sp|Q9UNZ	13	13	13	0	11	1	13	0	11	1	13	0	11	1	13	41.6	40.572	0	91.907	912730000	0	3.54E+08	1279200	5.58E+08	0	28979000	1109400	21667000
Q9Y6G9	Q9Y6G9	Cytoplasmic dynein 1 light intermediate chain 1	DYNC1LI1	sp|Q9Y6G9	14	14	14	1	13	9	14	1	13	9	14	1	13	9	14	33.7	56.578	0	55.671	912260000	217470	1.91E+08	24467000	6.96E+08	1070500	8918300	11397000	40780000
P11717	P11717	Cation-independent mannose-6-phosphate recep	IGF2R	sp|P11717	21	21	21	1	9	6	21	1	9	6	21	1	9	6	21	10.9	274.37	0	103.71	911840000	101470	1.05E+08	11297000	7.95E+08	331860	2757600	3776100	42817000
Q9BYD3	Q9BYD3	39S ribosomal protein L4, mitochondrial	MRPL4	sp|Q9BYD3	7	7	7	0	5	2	7	0	5	2	7	0	5	2	7	41.8	34.919	0	74.727	911790000	0	2.53E+08	9678700	6.49E+08	0	21420000	12826000	21242000
Q9GZT3	Q9GZT3	SRA stem-loop-interacting RNA-binding protein, r	SLIRP	sp|Q9GZT3	6	6	6	3	6	5	5	3	6	5	5	3	6	5	5	53.2	12.349	0	20.75	911780000	16701000	5.26E+08	18900000	3.5E+08	23371000	38881000	9472100	18234000
Q14376	Q14376	UDP-glucose 4-epimerase	GALE	sp|Q14376	6	6	6	0	4	0	6	0	4	0	6	0	4	0	6	24.1	38.281	0	50.242	907950000	0	52958000	0	8.55E+08	0	8032800	0	31797000
Q9Y316	Q9Y316	Protein MEMO1	MEMO1	sp|Q9Y316	8	8	8	0	6	3	8	0	6	3	8	0	6	3	8	49.2	33.733	0	141.95	907900000	0	1.18E+08	6403400	7.83E+08	0	11368000	10794000	24778000
Q16186	Q16186	Proteasomal ubiquitin receptor ADRM1	ADRM1	sp|Q16186	6	6	6	0	5	4	6	0	5	4	6	0	5	4	6	14.7	42.153	0	22.021	906140000	0	3.53E+08	14882000	5.38E+08	0	33699000	10137000	19430000
Q00535	Q00535	Cyclin-dependent-like kinase 5	CDK5	sp|Q00535	8	7	7	1	8	2	8	1	7	1	7	1	7	1	7	33.2	33.304	0	16.3	904870000	855060	2.31E+08	1846500	6.71E+08	1345900	15104000	1036900	32595000
P60903	P60903	Protein S100-A10	S100A10	sp|P60903	3	3	3	0	1	2	3	0	1	2	3	0	1	2	3	45.4	11.203	0	9.9633	904620000	0	5345300	11808000	8.87E+08	0	677350	2871600	41355000
Q00796	Q00796	Sorbitol dehydrogenase	SORD	sp|Q00796	5	5	5	0	5	0	5	0	5	0	5	0	5	0	5	21.3	38.324	0	41.386	903380000	0	2.66E+08	0	6.38E+08	0	23615000	0	23783000
P12955	P12955	Xaa-Pro dipeptidase	PEPD	sp|P12955	12	12	12	1	9	3	11	1	9	3	11	1	9	3	11	30.8	54.548	0	96.638	902380000	450260	2.46E+08	18941000	6.37E+08	649660	24334000	5146000	30908000
P41227;Q	P41227	N-alpha-acetyltransferase 10	NAA10	sp|P41227	12	12	12	0	9	3	11	0	9	3	11	0	9	3	11	58.3	26.458	0	39.952	901260000	0	3.83E+08	5575900	5.12E+08	0	32370000	5637500	20596000
Q9Y6E2	Q9Y6E2	Basic leucine zipper and W2 domain-containing pi	BZW2	sp|Q9Y6E2	11	10	10	0	10	6	11	0	9	6	10	0	9	6	10	23.6	48.162	0	27.365	900260000	0	2.22E+08	9118400	6.69E+08	0	26688000	4864300	19451000
Q9UL15	Q9UL15	BAG family molecular chaperone regulator 5	BAG5	sp|Q9UL15	9	9	9	0	9	2	8	0	9	2	8	0	9	2	8	30.4	51.199	0	44.613	898050000	0	2.61E+08	3217200	6.34E+08	0	23148000	10629000	17448000
P21912	P21912	Succinate dehydrogenase [ubiquinone] iron-sulfu	SDHB	sp|P21912	8	8	8	0	8	1	6	0	8	1	6	0	8	1	6	31.4	31.629	0	18.418	897290000	0	4.75E+08	415660	4.22E+08	0	40505000	294500	18560000
Q9NPQ8;Q	Q9NPQ8	Synembryn-A	RIC8A	sp|Q9NPQ	13	13	13	0	10	5	12	0	10	5	12	0	10	5	12	33.5	59.709	0	111.64	895440000	0	2.13E+08	17975000	6.65E+08	0	11594000	15754000	30832000
P62330	P62330	ADP-ribosylation factor 6	ARF6	sp|P62330	4	4	4	0	4	1	4	0	4	1	4	0	4	1	4	33.1	20.082	0	23.04	894040000	0	1.78E+08	3103400	7.13E+08	0	16624000	4194900	23690000
Q5H9R7	Q5H9R7	Serine/threonine-protein phosphatase 6 regulato	PPP6R3	sp|Q5H9R7	12	12	12	0	11	4	11	0	11	4	11	0	11	4	11	17.5	97.668	0	109.16	894000000	0	2.36E+08	6861100	6.52E+08	0	18966000	10251000	21825000
P60981	P60981	Destrin	DSTN	sp|P60981	5	4	4	1	5	2	5	1	4	1	4	1	4	1	4	34.5	18.506	0	17.138	893400000	2149800	2.01E+08	7816500	6.83E+08	2415900	17568000	5902800	27290000
Q96EY7	Q96EY7	Pentatricopeptide repeat domain-containing prot	PTCD3	sp|Q96EY7	11	11	11	0	9	9	8	0	9	9	8	0	9	9	8	20.6	78.549	0	44.037	890960000	0	4.14E+08	18688000	4.58E+08	0	34420000	16394000	16645000
Q12979	Q12979	Active breakpoint cluster region-related protein	ABR	sp|Q12979	11	11	10	1	4	2	11	1	4	2	11	1	3	2	10	20.7	97.597	0	51.498	887870000	88516000	20238000	63313000	7.16E+08	64304000	14325000	44582000	39073000
Q13724	Q13724	Mannosyl-oligosaccharide glucosidase	MOGS	sp|Q13724	15	15	15	0	12	7	11	0	12	7	11	0	12	7	11	26.2	91.916	0	58.869	886930000	0	3.21E+08	26220000	5.39E+08	0	21275000	27470000	19385000
P62273	P62273	40S ribosomal protein S29	RPS29	sp|P62273	2	2	2	1	2	2	2	1	2	2	2	1	2	2	2	33.9	6.6767	0	6.1368	885610000	1.5E+08	2.33E+08	87383000	4.15E+08	2.37E+08	1244000	2558200	4736000
P52701	P52701	DNA mismatch repair protein Msh6	MSH6	sp|P52701	14	14	14	0	10	8	14	0	10	8	14	0	10	8	14	15.8	152.78	0	39.764	883280000	0	1.33E+08	30316000	7.2E+08	0	11995000	29549000	20152000
Q92890	Q92890	Ubiquitin fusion degradation protein 1 homolog	UFD1L	sp|Q92890	8	8	8	0	7	2	8	0	7	2	8	0	7	2	8	35.8	34.5	0	55.249	881570000	0	2.06E+08	1855600	6.74E+08	0	21116000	436570	25567000
Q92820	Q92820	Gamma-glutamyl hydrolase	GGH	sp|Q92820	9	9	9	0	6	4	9	0	6	4	9	0	6	4	9	35.2	35.964	0	53.641	878470000	0	63333000	26556000	7.89E+08	0	12225000	20150000	23483000
P30405	P30405	Peptidyl-prolyl cis-trans isomerase F, mitochondri	PPIF	sp|P30405	7	7	6	0	7	0	4	0	7	0	4	0	6	0	4	44.9	22.04	0	67.989	877660000	0	5.44E+08	0	3.33E+08	0	31470000	0	9122000
Q9BPW8	Q9BPW8	Protein NipSnap homolog 1	NIPSNAP1	sp|Q9BPW	9	9	7	0	6	5	8	0	6	5	8	0	4	4	7	40.1	33.31	0	33.473	875720000	0	2.11E+08	53823000	6.11E+08	0	27926000	36739000	15230000
P49770	P49770	Translation initiation factor eIF-2B subunit beta	EIF2B2	sp|P49770	9	9	9	0	7	4	9	0	7	4	9	0	7	4	9	29.3	38.989	0	38.565	869490000	0	2.04E+08	4062000	6.62E+08	0	13318000	11355000	23236000
P23368	P23368	NAD-dependent malic enzyme, mitochondrial	ME2	sp|P23368	8	8	8	0	8	1	7	0	8	1	7	0	8	1	7	22.9	65.443	0	45.024	868270000	0	3.53E+08	6706400	5.08E+08	0	37778000	6818100	11963000
O60711	O60711	Leupaxin	LPXN	sp|O60711	3	3	3	0	0	2	3	0	0	2	3	0	0	2	3	15.3	43.332	0	33.191	865400000	0	0	15019000	8.5E+08	0	0	13110000	31955000
Q6Y7W6	Q6Y7W6	PERQ amino acid-rich with GYF domain-containin	GIGYF2	sp|Q6Y7W	20	20	20	2	14	5	16	2	14	5	16	2	14	5	16	25.3	150.07	0	122.79	862790000	1049700	2.45E+08	14974000	6.02E+08	1279500	24665000	9938500	21560000
P82979	P82979	SAP domain-containing ribonucleoprotein	SARNP	sp|P82979	7	7	7	0	6	5	7	0	6	5	7	0	6	5	7	38.6	23.671	0	26.257	862100000	0	5.28E+08	7755100	3.26E+08	0	46008000	7800900	11366000
P0DOY3;P(	P0DOY3;P(	Ig lambda-6 chain C region;Ig lambda-7 chain C re	IGLC6;IGLC	sp|P0DOY3	4	4	2	2	1	4	0	2	1	4	0	1	1	2	0	55.7	11.265	0	11.601	861550000	8.13E+08	841240	47699000	0	6.5E+08	9505900	185760000	0
Q96EM0	Q96EM0	Trans-3-hydroxy-L-proline dehydratase	L3HYPDH	sp|Q96EM	9	9	9	0	1	0	9	0	1	0	9	0	1	0	9	37.9	38.137	0	28.199	859470000	0	2926300	0	8.57E+08	0	262300	0	35221000
P53597	P53597	Succinyl-CoA ligase [ADP/GDP-forming] subunit al	SUCLG1	sp|P53597	7	7	7	1	7	4	7	1	7	4	7	1	7	4	7	28.9	36.249	0	76.264	854560000	1330400	2.63E+08	10975000	5.8E+08	2390800	24028000	5863600	21972000
Q9H3N1	Q9H3N1	Thioredoxin-related transmembrane protein 1	TMX1	sp|Q9H3N	9	9	9	0	9	1	8	0	9	1	8	0	9	1	8	27.9	31.791	0	21.907	853690000	0	3.52E+08	624850	5.02E+08	0	26936000	624190	24472000
Q96G03	Q96G03	Phosphoglucomutase-2	PGM2	sp|Q96G03	10	10	10	0	9	1	9	0	9	1	9	0	9	1	9	19.6	68.283	0	54.534	852710000	0	3.89E+08	344760	4.64E+08	0	25289000	617600	27660000
P50552	P50552	Vasodilator-stimulated phosphoprotein	VASP	sp|P50552	10	10	10	0	6	1	10	0	6	1	10	0	6	1	10	31.8	39.829	0	66.892	852320000	0	75365000	707580	7.76E+08	0	3610600	754460	33824000
Q9Y3E5	Q9Y3E5	Peptidyl-tRNA hydrolase 2, mitochondrial	PTRH2	sp|Q9Y3E5	4	4	4	0	3	0	4	0	3	0	4	0	3	0	4	35.2	19.193	0	38.431	850160000	0	2.8E+08	0	5.7E+08	0	27124000	0	20999000
Q13616	Q13616	Cullin-1	CUL1	sp|Q13616	18	18	18	2	13	0	16	2	13	0	16	2	13	0	16	31.1	89.677	0	87.966	850000000	1871700	1.92E+08	0	6.56E+08	1232100	22332000	0	22238000
Q9H0A0	Q9H0A0	N-acetyltransferase 10	NAT10	sp|Q9H0A0	19	19	19	3	7	19	12	3	7	19	12	3	7	19	12	28.4	115.73	0	75.174	848800000	6975400	70137000	200760000	5.71E+08	21426000	7534800	114820000	27317000
P40763	P40763	Signal transducer and activator of transcription 3	STAT3	sp|P40763	12	12	12	0	8	0	12	0	8	0	12	0	8	0	12	25.2	88.067	0	85.764	844330000	0	1.45E+08	0	7E+08	0	16420000	0	24895000
Q96P16	Q96P16	Regulation of nuclear pre-mRNA domain-containi	RPRD1A	sp|Q96P16	11	11	8	0	10	1	8	0	10	1	8	0	8	1	5	40.4	35.719	0	34.915	844270000	0	5.58E+08	784000	2.85E+08	0	57978000	717870	2776700
Q14232	Q14232	Translation initiation factor eIF-2B subunit alpha	EIF2B1	sp|Q14232	9	9	9	0	9	1	8	0	9	1	8	0	9	1	8	34.8	33.712	0	25.72	844020000	0	3.28E+08	1243900	5.15E+08	0	28978000	952580	20998000
Q8TAQ2	Q8TAQ2	SWI/SNF complex subunit SMARCC2	SMARCC2	sp|Q8TAQ	13	13	9	0	11	6	12	0	11	6	12	0	8	5	9	17.8	132.88	0	77.068	839300000	0	2.12E+08	16672000	6.11E+08	0	14078000	21159000	18485000
Q96CT7	Q96CT7	Coiled-coil domain-containing protein 124	CCDC124	sp|Q96CT7	11	11	11	2	11	8	9	2	11	8	9	2	11	8	9	46.6	25.835	0	77.69	838180000	2423000	4.73E+08	39708000	3.23E+08	2013100	33793000	39762000	8529100
Q06124	Q06124	Tyrosine-protein phosphatase non-receptor type	PTPN11	sp|Q06124	9	9	9	0	7	2	8	0	7	2	8	0	7	2	8	24.6	68.01	0	64.981	836950000	0	1.55E+08	1216400	6.81E+08	0	28288000	2581200	11588000
O14880	O14880	Microsomal glutathione S-transferase 3	MGST3	sp|O14880	3	3	3	0	3	3	3	0	3	3	3	0	3	3	3	18.4	16.516	0	11.69	836680000	0	2.32E+08	6312900	5.98E+08	0	19768000	5560100	23993000
Q14320;Q	Q14320	Protein FAM50A	FAM50A	sp|Q14320	7	7	7	0	7	1	6	0	7	1	6	0	7	1	6	24.2	40.241	0	22.208	836330000	0	2.21E+08	652690	6.15E+08	0	22470000	547320	22178000
Q9UHI6	Q9UHI6	Probable ATP-dependent RNA helicase DDX20	DDX20	sp|Q9UHI6	13	13	13	2	11	8	11	2	11	8	11	2	11	8	11	26.2	92.239	0	97.517	835820000	1821600	2.81E+08	22039000	5.31E+08	4455900	21113000	15461000	22229000

P67812	P67812	Signal peptidase complex catalytic subunit SEC11/	SEC11A	sp|P67812	5	5	5	0	5	3	5	0	5	3	5	0	5	3	5	20.7	20.625	0	26.495	834860000	0	2.09E+08	4936300	6.21E+08	0	18028000	4476700	24751000
P50402	P50402	Emerin	EMD	sp|P50402	11	11	11	3	11	11	9	3	11	11	9	3	11	11	9	63.8	28.994	0	72.941	833910000	9612100	4.84E+08	78376000	2.62E+08	12774000	29973000	64203000	8775000
O95573	O95573	Long-chain-fatty-acid--CoA ligase 3	ACSL3	sp|O95573	14	13	13	1	4	7	14	1	3	6	13	1	3	6	13	31.5	80.419	0	67.204	832180000	2395600	36471000	19680000	7.74E+08	2674200	4079600	10800000	33097000
P31350	P31350	Ribonucleoside-diphosphate reductase subunit M	RRM2	sp|P31350	6	6	5	0	4	0	6	0	4	0	6	0	3	0	5	24.7	44.877	0	31.283	829910000	0	1.18E+08	0	7.12E+08	0	12043000	0	27654000
P46087	P46087	Probable 28S rRNA (cytosine(4447)-C(5))-methylt	NOP2	sp|P46087	20	20	20	1	14	18	17	1	14	18	17	1	14	18	17	33.5	89.301	0	52.095	829490000	5044000	1.37E+08	208120000	4.8E+08	6845000	13020000	133120000	23687000
O00442	O00442	RNA 3-terminal phosphate cyclase	RTCA	sp|O00442	8	8	8	0	6	1	8	0	6	1	8	0	6	1	8	30.6	39.336	0	42.24	829450000	0	1.39E+08	1499900	6.89E+08	0	24161000	4139600	13311000
O75348;O	O75348;O	V-type proton ATPase subunit G 1;V-type proton /	ATP6V1G1	sp|O75348	4	4	4	0	3	1	3	0	3	1	3	0	3	1	3	30.5	13.757	0	68.781	828740000	0	2.39E+08	1287400	5.89E+08	0	24916000	5581900	15614000
O00194	O00194	Ras-related protein Rab-27B	RAB27B	sp|O00194	6	6	4	0	0	1	6	0	0	1	6	0	0	1	4	30.3	24.608	0	23.519	828430000	0	0	1342900	8.27E+08	0	0	902420	34014000
Q9H3P7	Q9H3P7	Golgi resident protein GCP60	ACBD3	sp|Q9H3P7	10	10	10	0	10	0	9	0	10	0	9	0	10	0	9	32	60.593	0	100.18	828020000	0	3.2E+08	0	5.08E+08	0	24282000	0	21243000
Q92783	Q92783	Signal transducing adapter molecule 1	STAM	sp|Q92783	9	9	8	1	8	1	9	1	8	1	9	1	7	0	8	27.4	59.179	0	63.967	826190000	494390	1.73E+08	735740	6.52E+08	632270	3785100	632290	38013000
O75947	O75947	ATP synthase subunit d, mitochondrial	ATP5H	sp|O75947	8	8	8	2	8	2	8	2	8	2	8	2	8	2	8	64	18.491	0	26.355	824030000	1846300	3.85E+08	11635000	4.26E+08	4098600	32520000	6010300	18284000
Q9BSH4	Q9BSH4	Translational activator of cytochrome c oxidase 1	TACO1	sp|Q9BSH4	6	6	6	0	6	1	5	0	6	1	5	0	6	1	5	31.6	32.477	0	32.189	823850000	0	2.48E+08	2181300	5.74E+08	0	12462000	3098100	31345000
Q9BTV4	Q9BTV4	Transmembrane protein 43	TMEM43	sp|Q9BTV4	10	10	10	1	4	7	9	1	4	7	9	1	4	7	9	38.8	44.875	0	62.067	823780000	1438600	65988000	28798000	7.28E+08	4016400	5831600	11156000	35525000
P51452	P51452	Dual specificity protein phosphatase 3	DUSP3	sp|P51452	6	6	6	0	6	2	6	0	6	2	6	0	6	2	6	41.6	20.478	0	32.236	822690000	0	2.44E+08	1800900	5.77E+08	0	26270000	1479500	18675000
Q12765	Q12765	Secernin-1	SCRN1	sp|Q12765	8	8	8	0	8	1	7	0	8	1	7	0	8	1	7	24.4	46.382	0	31.382	822210000	0	1.63E+08	403860	6.59E+08	0	6822400	735810	34190000
O75717	O75717	WD repeat and HMG-box DNA-binding protein 1	WDHD1	sp|O75717	18	18	18	1	12	2	17	1	12	2	17	1	12	2	17	24.2	125.97	0	111.93	822040000	1140900	1.39E+08	2908200	6.79E+08	1334800	8899900	1667900	31354000
Q8IYT4	Q8IYT4	Katanin p60 ATPase-containing subunit A-like 2	KATNAL2	sp|Q8IYT4	1	1	1	0	1	1	1	0	1	1	1	0	1	1	1	2.2	61.252	0.000485	3.4349	818470000	0	2.44E+08	13990000	5.61E+08	0	21483000	9401300	23062000
P09417	P09417	Dihydropteridine reductase	QDPR	sp|P09417	7	7	7	0	6	0	7	0	6	0	7	0	6	0	7	41.8	25.789	0	37.43	817150000	0	2.73E+08	0	5.45E+08	0	24787000	0	21635000
O95394	O95394	Phosphoacetylglucosamine mutase	PGM3	sp|O95394	11	11	11	0	9	0	9	0	9	0	9	0	9	0	9	32.3	59.851	0	123.59	816260000	0	4.87E+08	0	3.29E+08	0	44358000	0	12119000
Q63HN8	Q63HN8	E3 ubiquitin-protein ligase RNF213	RNF213	sp|Q63HN	20	20	20	0	8	8	19	0	8	8	19	0	8	8	19	6.4	591.4	0	98.862	815560000	0	49889000	18904000	7.47E+08	0	4833500	29274000	13705000
O14964	O14964	Hepatocyte growth factor-regulated tyrosine kina	HGS	sp|O14964	12	12	12	0	8	3	11	0	8	3	11	0	8	3	11	21.5	86.191	0	54.706	815260000	0	1.5E+08	1906000	6.63E+08	0	12477000	2089800	26449000
P53007	P53007	Tricarboxylate transport protein, mitochondrial	SLC25A1	sp|P53007	10	10	10	1	7	8	10	1	7	8	10	1	7	8	10	39.2	34.012	0	23.836	815120000	2064900	2.29E+08	41059000	5.43E+08	6039900	15982000	21098000	29007000
P16401	P16401	Histone H1.5	HIST1H1B	sp|P16401	10	10	8	1	2	8	10	1	2	8	10	0	0	7	8	35	22.58	0	23.498	814940000	72082	46989000	103640000	6.64E+08	4255900	11695000	63172000	22051000
Q969H8	Q969H8	Myeloid-derived growth factor	MYDGF	sp|Q969H8	4	4	4	0	4	0	4	0	4	0	4	0	4	0	4	27.2	18.795	0	13.138	814330000	0	2.47E+08	0	5.67E+08	0	20610000	0	24505000
Q15388	Q15388	Mitochondrial import receptor subunit TOM20 hc	TOMM20	sp|Q15388	4	4	4	1	4	3	3	1	4	3	3	1	4	3	3	32.4	16.298	0	26.072	813860000	3057300	4.52E+08	59394000	3E+08	9418200	28203000	42077000	15420000
Q8IYB3	Q8IYB3	Serine/arginine repetitive matrix protein 1	SRRM1	sp|Q8IYB3	7	7	7	4	6	4	4	4	6	4	4	4	6	4	4	11.5	102.33	0	33.828	813750000	64157000	5.85E+08	30359000	1.34E+08	65955000	36139000	30399000	9141600
O75477	O75477	Erlin-1	ERLIN1	sp|O75477	12	10	10	1	9	7	11	1	7	5	9	1	7	5	9	38.5	39.171	0	43.779	811730000	5963800	1.03E+08	28644000	6.74E+08	8322200	11969000	19092000	22605000
Q13217	Q13217	DnaJ homolog subfamily C member 3	DNAJC3	sp|Q13217	12	12	12	1	10	1	9	1	10	1	9	1	10	1	9	39.1	57.579	0	140.29	811060000	529870	3.13E+08	516690	4.97E+08	366650	17699000	594090	30239000
Q9NZL4	Q9NZL4	Hsp70-binding protein 1	HSPBP1	sp|Q9NZL4	9	9	9	0	6	0	9	0	6	0	9	0	6	0	9	39.3	39.302	0	42.714	810110000	0	1.21E+08	0	6.9E+08	0	15016000	0	23967000
P61619	P61619	Protein transport protein Sec61 subunit alpha iso	SEC61A1	sp|P61619	7	7	4	2	7	5	7	2	7	5	7	2	4	3	4	17.9	52.264	0	26.854	809340000	2757100	1.53E+08	20769000	6.32E+08	6228200	13323000	14138000	22487000
Q8WXF1	Q8WXF1	Paraspeckle component 1	PSPC1	sp|Q8WXF	13	12	12	2	13	7	9	2	12	6	8	2	12	6	8	30.6	58.743	0	57.052	806580000	4310900	5.43E+08	28730000	2.31E+08	6451000	46643000	17710000	10140000
Q71U36;P(	Q71U36;P(	Tubulin alpha-1A chain;Tubulin alpha-3E chain	TUBA1A;TI	sp|Q71U3	27	2	1	10	27	22	27	0	2	1	2	0	1	0	1	69.6	50.135	0	7.3523	806080000	0	2.52E+08	11251000	5.43E+08	0	20617000	11107000	20383000
Q9UM00	Q9UM00	Transmembrane and coiled-coil domain-containir	TMCO1	sp|Q9UM0	4	4	4	0	2	4	3	0	2	4	3	0	2	4	3	20.1	27.079	0	13.71	805420000	0	1.09E+08	25372000	6.71E+08	0	9877600	16466000	26437000
P13489	P13489	Ribonuclease inhibitor	RNH1	sp|P13489	7	7	7	0	4	2	7	0	4	2	7	0	4	2	7	23.6	49.973	0	61.791	804630000	0	1.1E+08	7753500	6.87E+08	0	11315000	5754000	22002000
P49189	P49189	4-trimethylaminobutyraldehyde dehydrogenase	ALDH9A1	sp|P49189	13	13	13	0	12	2	12	0	12	2	12	0	12	2	12	29.6	53.801	0	52.794	804540000	0	4.54E+08	1801100	3.48E+08	0	47189000	769290	7628400
Q9NX46	Q9NX46	Poly(ADP-ribose) glycohydrolase ARH3	ADPRHL2	sp|Q9NX46	8	8	8	1	8	0	7	1	8	0	7	1	8	0	7	31.7	38.946	0	41.975	804080000	1064300	3.22E+08	0	4.81E+08	1515900	29297000	0	13911000
Q9BV20	Q9BV20	Methylthioribose-1-phosphate isomerase	MRI1	sp|Q9BV20	10	10	10	0	10	0	6	0	10	0	6	0	10	0	6	44.2	39.149	0	34.086	802890000	0	6.62E+08	0	1.41E+08	0	53710000	0	8366600
P19474	P19474	E3 ubiquitin-protein ligase TRIM21	TRIM21	sp|P19474	15	15	15	2	1	15	4	2	1	15	4	2	1	15	4	34.5	54.169	0	74.866	800570000	1381800	8667200	764750000	25770000	281680	209450	504410000	297170
Q9HCE1	Q9HCE1	Putative helicase MOV-10	MOV10	sp|Q9HCE1	16	16	16	4	15	9	15	4	15	9	15	4	15	9	15	18.6	113.67	0	47.897	800250000	1.48E+08	2.55E+08	37514000	3.6E+08	1.34E+08	17783000	48657000	9713000
Q9HC35	Q9HC35	Echinoderm microtubule-associated protein-like 4	EML4	sp|Q9HC35	14	14	14	2	11	1	14	2	11	1	14	2	11	1	14	20.2	108.91	0	33.559	799120000	3150000	2.19E+08	1571300	5.76E+08	2992400	18777000	1319400	24063000
Q9NX58	Q9NX58	Cell growth-regulating nucleolar protein	LYAR	sp|Q9NX58	12	12	12	1	9	11	9	1	9	11	9	1	9	11	9	43	43.614	0	73.728	796950000	25732000	2.68E+08	103970000	4E+08	31273000	22363000	72025000	9963700
Q92696	Q92696	Geranylgeranyl transferase type-2 subunit alpha	RABGGTA	sp|Q92696	5	5	5	0	5	1	4	0	5	1	4	0	5	1	4	10.2	65.071	0	45.874	794020000	0	95934000	4136500	6.94E+08	0	10287000	9629400	19859000
Q9Y5X3	Q9Y5X3	Sorting nexin-5	SNX5	sp|Q9Y5X3	11	11	11	1	11	0	11	1	11	0	11	1	11	0	11	29.5	46.816	0	38.2	792320000	926780	2.65E+08	0	5.27E+08	0	23699000	0	21300000
P14324	P14324	Farnesyl pyrophosphate synthase	FDPS	sp|P14324	5	5	5	0	4	1	5	0	4	1	5	0	4	1	5	17.2	48.275	0	43.088	792170000	0	3.78E+08	485070	4.14E+08	0	41774000	981960	6069600
Q6NVY1	Q6NVY1	3-hydroxyisobutyryl-CoA hydrolase, mitochondria	HIBCH	sp|Q6NVY1	10	10	10	2	10	0	6	2	10	0	6	2	10	0	6	29.8	43.482	0	39.461	785790000	1529400	4.77E+08	0	3.07E+08	1733600	39593000	0	14898000
Q9UHY1	Q9UHY1	Nuclear receptor-binding protein	NRBP1	sp|Q9UHY	7	7	7	0	3	0	7	0	3	0	7	0	3	0	7	23.6	59.844	0	69.168	783290000	0	1.24E+08	0	6.59E+08	0	15159000	0	22886000
Q9NUJ1	Q9NUJ1	Mycophenolic acid acyl-glucuronide esterase, mit	ABHD10	sp|Q9NUJ1	8	8	8	0	8	1	7	0	8	1	7	0	8	1	7	32.4	33.932	0	33.378	782830000	0	5.03E+08	8226100	2.72E+08	0	44070000	7672200	9281400
Q9BUL8	Q9BUL8	Programmed cell death protein 10	PDCD10	sp|Q9BUL8	6	6	6	0	6	1	4	0	6	1	4	0	6	1	4	33	24.701	0	80.53	782200000	0	3.54E+08	1414700	4.26E+08	0	31762000	1343900	16619000
P00390	P00390	Glutathione reductase, mitochondrial	GSR	sp|P00390	7	7	7	0	6	2	7	0	6	2	7	0	6	2	7	22	56.256	0	58.037	781040000	0	2.15E+08	5374900	5.6E+08	0	18656000	4802000	22186000
Q9BUQ8	Q9BUQ8	Probable ATP-dependent RNA helicase DDX23	DDX23	sp|Q9BUQ	15	15	15	0	14	14	11	0	14	14	11	0	14	14	11	22	95.581	0	30.84	779590000	0	2.73E+08	43385000	4.63E+08	0	22435000	32938000	16918000
Q16643	Q16643	Drebrin	DBN1	sp|Q16643	11	11	11	1	11	7	2	1	11	7	2	1	11	7	2	25.9	71.428	0	55.556	779510000	1.53E+08	5.53E+08	47453000	25988000	1.29E+08	76170000	21863000	7196600
Q13564	Q13564	NEDD8-activating enzyme E1 regulatory subunit	NAE1	sp|Q13564	12	12	12	0	9	0	11	0	9	0	11	0	9	0	11	35.6	60.246	0	107.04	778560000	0	3.76E+08	0	4.02E+08	0	31920000	0	17790000
P31949	P31949	Protein S100-A11;Protein S100-A11, N-terminally	S100A11	sp|P31949	2	2	2	0	0	1	2	0	0	1	2	0	0	1	2	23.8	11.74	0	7.248	778390000	0	0	10424000	7.68E+08	0	0	7692500	30895000
P84243;Q7	P84243;Q7	Histone H3.3;Histone H3.2;Histone H3.1t;Histone	H3F3A;HIS	sp|P84243	4	4	4	2	4	3	4	2	4	3	4	2	4	3	4	24.3	15.328	0	6.9965	777340000	60953000	1.79E+08	101490000	4.36E+08	68766000	13489000	65673000	14935000
Q8WWY3	Q8WWY3	U4/U6 small nuclear ribonucleoprotein Prp31	PRPF31	sp|Q8WW	9	9	9	2	8	6	7	2	8	6	7	2	8	6	7	30.3	55.455	0	51.887	776620000	1433300	2.79E+08	21885000	4.74E+08	1367600	25635000	10783000	22464000
O60443	O60443	Non-syndromic hearing impairment protein 5	DFNA5	sp|O60443	10	10	10	0	1	2	10	0	1	2	10	0	1	2	10	30.2	54.554	0	96.875	776300000	0	3149800	2138800	7.71E+08	0	224790	511440	32687000
Q9H993	Q9H993	Protein-glutamate O-methyltransferase	ARMT1	sp|Q9H993	10	10	10	1	10	0	8	1	10	0	8	1	10	0	8	38.3	51.172	0	69.1	775450000	32605000	3.73E+08	0	3.7E+08	28023000	30689000	0	21992000
Q7Z2W4	Q7Z2W4	Zinc finger CCCH-type antiviral protein 1	ZC3HAV1	sp|Q7Z2W	13	13	13	2	7	11	13	2	7	11	13	2	7	11	13	22.7	101.43	0	80.698	775250000	2732400	90139000	67471000	6.15E+08	2077000	8781100	51483000	18228000
Q9BQ52	Q9BQ52	Zinc phosphodiesterase ELAC protein 2	ELAC2	sp|Q9BQ52	12	12	12	2	9	1	11	2	9	1	11	2	9	1	11	21.4	92.218	0	55.476	774340000	2.09E+08	2.75E+08	1268700	2.9E+08	1.24E+08	57594000	7190500	57403000
Q99986	Q99986	Serine/threonine-protein kinase VRK1	VRK1	sp|Q99986	7	7	7	0	7	7	7	0	7	7	7	0	7	7	7	24.7	45.476	0	41.353	771730000	0	1.92E+08	27724000	5.52E+08	0	17640000	17940000	22687000
Q9GZY8	Q9GZY8	Mitochondrial fission factor	MFF	sp|Q9GZY8	6	6	6	1	1	0	6	1	1	0	6	1	1	0	6	34.5	38.464	0	32.054	771480000	31616000	5910700	0	7.34E+08	12847000	1649200	0	47825000
Q9UHY7	Q9UHY7	Enolase-phosphatase E1	ENOPH1	sp|Q9UHY	3	3	3	0	2	0	3	0	2	0	3	0	2	0	3	20.7	28.932	0	10.931	771210000	0	3.25E+08	0	4.47E+08	0	26503000	0	20473000
P49006	P49006	MARCKS-related protein	MARCKSL1	sp|P49006	4	4	4	0	4	0	1	0	4	0	1	0	4	0	1	51.3	19.529	0	20.575	770110000	0	7.64E+08	0	5658300	0	63329000	0	232700
O43719	O43719	HIV Tat-specific factor 1	HTATSF1	sp|O43719	12	12	12	1	12	1	9	1	12	1	9	1	12	1	9	20.5	85.852	0	31.892	768340000	5728100	4.98E+08	116970	2.64E+08	2252200	55288000	263820	2796600
Q15417	Q15417	Calponin-3	CNN3	sp|Q15417	11	11	10	0	10	2	9	0	10	2	9	0	9	1	8	53.2	36.413	0	44.083	768310000	0	4.57E+08	4484900	3.07E+08	0	39954000	3949300	12027000
Q9Y6C9	Q9Y6C9	Mitochondrial carrier homolog 2	MTCH2	sp|Q9Y6C9	8	8	8	0	5	2	8	0	5	2	8	0	5	2	8	35.3	33.331	0	22.706	766960000	0	1.94E+08	10713000	5.62E+08	0	17970000	10360000	19102000
Q15654	Q15654	Thyroid receptor-interacting protein 6	TRIP6	sp|Q15654	6	6	6	0	4	3	6	0	4	3	6	0	4	3	6	29	50.287	0	60.494	766270000	0	27363000	8355200	7.31E+08	0	4538400	11476000	16431000
O43768	O43768	Alpha-endosulfine	ENSA	sp|O43768	9	9	8	0	9	0	8	0	9	0	8	0	8	0	7	60.3	13.389	0	21.707	766190000	0	5.59E+08	0	2.07E+08	0	47493000	0	10314000
Q9BQE3	Q9BQE3	Tubulin alpha-1C chain	TUBA1C	sp|Q9BQE3	25	2	2	10	24	21	25	0	1	2	2	0	1	2	2	65.7	49.895	0	33.985	765790000	0	78378000	51418000	6.36E+08	0	18300000	26965000	21519000
Q15126	Q15126	Phosphomevalonate kinase	PMVK	sp|Q15126	7	7	7	0	5	0	7	0	5	0	7	0	5	0	7	37.5	21.995	0	22.461	765470000	0	1E+08	0	6.65E+08	0	11775000	0	24414000
Q9Y450	Q9Y450	HBS1-like protein	HBS1L	sp|Q9Y450	14	14	14	2	11	4	14	2	11	4	14	2	11	4	14	32.2	75.472	0	51.228	764520000	4106100	1.73E+08	8356900	5.79E+08	3661600	14111000	4499700	26519000
Q9H488	Q9H488	GDP-fucose protein O-fucosyltransferase 1	POFUT1	sp|Q9H488	5	5	5	0	4	0	5	0	4	0	5	0	4	0	5	22.2	43.955	0	42.187	764430000	0	1.2E+08	0	6.45E+08	0	9778100	0	27289000
Q9UDW1	Q9UDW1	Cytochrome b-c1 complex subunit 9	UQCR10	sp|Q9UDW	3	3	3	1	3	2	3	1	3	2	3	1	3	2	3	50.8	7.3084	0	13.342	762950000	0	2.49E+08	16898000	4.97E+08	0	21409000	12312000	20012000
Q4J6C6	Q4J6C6	Prolyl endopeptidase-like	PREPL	sp|Q4J6C6	9	9	9	0	6	0	9	0	6	0	9	0	6	0	9	21.3	83.926	0	55.899	761340000	0	1.17E+08	0	6.44E+08	0	12440000	0	24380000
Q9P289;O(	Q9P289	Serine/threonine-protein kinase 26	STK26	sp|Q9P289	11	11	5	0	10	2	8	0	10	2	8	0	5	0	2	31	46.528	0	44.123	761070000	0	3.85E+08	3027500	3.73E+08	0	37402000	1588200	12326000
O60869	O60869	Endothelial differentiation-related factor 1	EDF1	sp|O60869	6	6	6	0	6	1	6	0	6	1	6	0	6	1	6	43.9	16.368	0	22.599	760850000	0	3.73E+08	953580	3.87E+08	0	32791000	687620	15932000
Q9BZE1	Q9BZE1	39S ribosomal protein L37, mitochondrial	MRPL37	sp|Q9BZE1	9	9	9	0	8	7	9	0	8	7	9	0	8	7	9	28.6	48.117	0	28.674	760070000	0	2.45E+08	24503000	4.9E+08	0	19775000	19569000	18904000
P80723	P80723	Brain acid soluble protein 1	BASP1	sp|P80723	7	7	7	0	3	5	7	0	3	5	7	0	3	5	7	57.3	22.693	0	41.272	759190000	0	15819000	16432000	7.27E+08	0	3545800	7413100	31373000
Q9NZ01	Q9NZ01	Very-long-chain enoyl-CoA reductase	TECR	sp|Q9NZ01	10	10	10	4	5	8	10	4	5	8	10	4	5	8	10	28.2	36.034	0	22.472	758950000	9773500	1.21E+08	30740000	5.98E+08	13352000	14063000	19556000	18691000
Q9UBW8	Q9UBW8	COP9 signalosome complex subunit 7a	COPS7A	sp|Q9UBW	8	8	8	1	6	3	8	1	6	3	8	1	6	3	8	37.8	30.276	0	95.53	758500000	1877500	2.12E+08	2694600	5.42E+08	1619100	18807000	1772000	22484000
O15258	O15258	Protein RER1	RER1	sp|O15258	5	5	5	0	3	3	5	0	3	3	5	0	3	3	5	32.7	22.958	0	23.672	757860000	0	26942000	18986000	7.12E+08	0	4846500	13745000	24611000
P35241	P35241	Radixin	RDX	sp|P35241	27	14	14	4	24	12	26	2	12	4	14	2	12	4	14	41.3	68.563	0	39.417	757130000	1177700	3.56E+08	7571100	3.93E+08	2663600	34421000	5951500	10741000
Q15437	Q15437	Protein transport protein Sec23B	SEC23B	sp|Q15437	13	11	11	0	10	2	12	0	8	2	10	0	8	2	10	23.7	86.478	0	138.48	754660000	0	1.49E+08	3915600	6.02E+08	0	19617000	1627400	19252000
P22307	P22307	Non-specific lipid-transfer protein	SCP2	sp|P22307	14	14	14	2	14	1	9	2	14	1	9	2	14	1	9	24.1	58.993	0	51.747	753750000	1775900	3.62E+08	2253000	3.88E+08	1726000	29958000	1295700	18142000
P51116	P51116	Fragile X mental retardation syndrome-related pr	FXR2	sp|P51116	14	12	11	3	10	13	10	2	9	11	8	2	8	10	7	38	74.222	0	87.125	750390000	18504000	2.2E+08	183080000	3.29E+08	23908000	19186000	119160000	11630000
Q9BVC6	Q9BVC6	Transmembrane protein 109	TMEM109	sp|Q9BVC	2	2	2	0	2	2	1	0	2	2	1	0	2	2	1	9.1	26.21	0	4.8315	749950000	0	3.41E+08	13511000	3.96E+08	0	32769000	8676800	13947000
P28161	P28161	Glutathione S-transferase Mu 2	GSTM2	sp|P28161	9	8	5	5	8	1	6	4	7	1	5	3	4	0	2	47.7	25.744	0	30.386	749940000	73229000	4.08E+08	3185000	2.66E+08	51463000	49860000	5976600	14935000
Q9NYK5	Q9NYK5	39S ribosomal protein L39, mitochondrial	MRPL39	sp|Q9NYK5	7	7	7	0	5	3	7	0	5	3	7	0	5	3	7	24.6	38.711	0	24.688	748490000	0	2.58E+08	14600000	4.76E+08	0	22117000	14576000	15408000
Q9NV70	Q9NV70	Exocyst complex component 1	EXOC1	sp|Q9NV7	10	10	10	0	5	3	10	0	5	3	10	0	5	3	10	15.7	101.98	0	40.821	746440000	0	45321000	10933000	6.9E+08	0	7303400	3920800	28502000

Q9Y277	Q9Y277	Voltage-dependent anion-selective channel prote	VDAC3	sp|Q9Y277	8	8	8	1	8	2	6	1	8	2	6	1	8	2	6	36.7	30.658	0	23.889	745490000	2892500	6.17E+08	5327800	1.2E+08	2452800	51843000	3644500	5054400
Q13084	Q13084	39S ribosomal protein L28, mitochondrial	MRPL28	sp|Q13084	11	11	11	1	9	3	9	1	9	3	9	1	9	3	9	59.4	30.156	0	48.036	745430000	2147300	1.98E+08	14867000	5.3E+08	6951600	20973000	10800000	12704000
Q9P0J0	Q9P0J0	NADH dehydrogenase [ubiquinone] 1 alpha subcc	NDUFA13	sp|Q9P0J0	5	5	5	1	5	3	5	1	5	3	5	1	5	3	5	43.1	16.698	0	18.044	745000000	532150	2.14E+08	11344000	5.19E+08	830010	22312000	4128300	21113000
Q9NX40	Q9NX40	OCIA domain-containing protein 1	OCIAD1	sp|Q9NX40	6	6	6	0	6	1	5	0	6	1	5	0	6	1	5	24.1	27.626	0	19.479	744330000	0	2.06E+08	1524400	5.37E+08	0	14739000	1509600	25013000
Q13526;O	Q13526	Peptidyl-prolyl cis-trans isomerase NIMA-interact	PIN1	sp|Q13526	5	5	5	1	5	1	4	1	5	1	4	1	5	1	4	42.3	18.243	0	34.207	744220000	2.19E+08	3.1E+08	212530	2.14E+08	1.23E+08	98351000	3128900	29243000
Q9ULE6	Q9ULE6	Paladin	PALD1	sp|Q9ULE6	5	5	5	5	2	1	1	5	2	1	1	5	2	1	1	8.4	96.753	0	31.627	742540000	2.26E+08	1.37E+08	4144100	3.75E+08	2.41E+08	7027000	2528300	5950300
P20645	P20645	Cation-dependent mannose-6-phosphate recepto	M6PR	sp|P20645	4	4	4	0	4	0	4	0	4	0	4	0	4	0	4	22	30.993	0	12.612	742080000	0	1.7E+08	0	5.72E+08	0	15952000	0	22573000
Q13907	Q13907	Isopentenyl-diphosphate Delta-isomerase 1	IDI1	sp|Q13907	7	7	7	0	7	1	7	0	7	1	7	0	7	1	7	36.6	26.319	0	49.274	741040000	0	5.18E+08	291300	2.22E+08	0	52218000	350570	2465900
O60256	O60256	Phosphoribosyl pyrophosphate synthase-associat	PRPSAP2	sp|O60256	10	9	9	0	9	3	10	0	8	2	9	0	8	2	9	37.4	40.925	0	49.504	739940000	0	2.92E+08	2142100	4.45E+08	0	14058000	4775100	26700000
Q13423	Q13423	NAD(P) transhydrogenase, mitochondrial	NNT	sp|Q13423	13	13	13	1	13	1	8	1	13	1	8	1	13	1	8	17.5	113.89	0	44.971	739320000	1053100	4.19E+08	165990	3.19E+08	1862700	43073000	0	6174800
Q9NRF9	Q9NRF9	DNA polymerase epsilon subunit 3	POLE3	sp|Q9NRF9	5	5	5	0	4	2	5	0	4	2	5	0	4	2	5	36.7	16.859	0	41.073	739170000	0	3.33E+08	4889500	4.01E+08	0	34706000	3143500	11296000
P49643	P49643	DNA primase large subunit	PRIM2	sp|P49643	11	11	11	0	9	0	10	0	9	0	10	0	9	0	10	27.1	58.805	0	23.098	739000000	0	3.27E+08	0	4.12E+08	0	30091000	0	15659000
O43598	O43598	2-deoxynucleoside 5-phosphate N-hydrolase 1	DNPH1	sp|O43598	5	5	5	0	5	1	2	0	5	1	2	0	5	1	2	55.2	19.108	0	46.069	736260000	0	6.84E+08	5137500	47590000	0	61573000	2105400	1983700
Q9UN86	Q9UN86	Ras GTPase-activating protein-binding protein 2	G3BP2	sp|Q9UN86	9	7	7	3	6	7	8	2	5	6	7	2	5	6	7	23.9	54.12	0	41.063	735880000	19051000	87880000	159210000	4.7E+08	65833000	10171000	65098000	10293000
Q16537;Q	Q16537	Serine/threonine-protein phosphatase 2A 56 kDa	PPP2R5E	sp|Q16537	10	10	7	0	4	2	10	0	4	2	10	0	3	1	7	24.8	54.699	0	33.127	735650000	0	27780000	7310400	7.01E+08	0	3783000	9809800	22580000
O94874	O94874	E3 UFM1-protein ligase 1	UFL1	sp|O94874	14	14	14	0	8	9	13	0	8	9	13	0	8	9	13	23.3	89.594	0	79.115	733860000	0	1.14E+08	18721000	6.02E+08	0	11684000	17458000	18187000
P17301	P17301	Integrin alpha-2	ITGA2	sp|P17301	16	16	16	0	5	1	16	0	5	1	16	0	5	1	16	21.2	129.29	0	66.891	730190000	0	17282000	1708200	7.11E+08	0	1332700	894610	29693000
P35250	P35250	Replication factor C subunit 2	RFC2	sp|P35250	11	11	11	0	11	6	8	0	11	6	8	0	11	6	8	45.2	39.157	0	106.29	729740000	0	3.06E+08	17948000	4.06E+08	0	21343000	19222000	15153000
Q9Y333	Q9Y333	U6 snRNA-associated Sm-like protein LSm2	LSM2	sp|Q9Y333	3	3	3	0	3	0	2	0	3	0	2	0	3	0	2	48.4	10.834	0	13.06	729540000	0	3.6E+08	0	3.69E+08	0	30082000	0	16870000
Q29RF7	Q29RF7	Sister chromatid cohesion protein PDS5 homolog	PDS5A	sp|Q29RF7	21	21	20	0	17	4	20	0	17	4	20	0	16	4	19	22.4	150.83	0	68.86	729270000	0	2.31E+08	10259000	4.88E+08	0	17943000	6076800	23318000
Q53EU6	Q53EU6	Glycerol-3-phosphate acyltransferase 3	AGPAT9	sp|Q53EU6	12	12	12	0	0	7	12	0	0	7	12	0	0	7	12	26.7	48.705	0	65.893	729180000	0	0	28113000	7.01E+08	0	0	27268000	20456000
Q8TCS8	Q8TCS8	Polyribonucleotide nucleotidyltransferase 1, mito	PNPT1	sp|Q8TCS8	16	16	16	0	15	5	13	0	15	5	13	0	15	5	13	27.6	85.95	0	63.932	728700000	0	3.45E+08	7178900	3.76E+08	0	32792000	4221000	12709000
Q8NI35	Q8NI35	InaD-like protein	INADL	sp|Q8NI35	1	1	1	0	1	1	1	0	1	1	1	0	1	1	1	0.7	196.37	0.00453	1.8274	726730000	0	5.89E+08	7791800	1.3E+08	0	50696000	6591500	5213500
P61956;Q6	P61956	Small ubiquitin-related modifier 2	SUMO2	sp|P61956	3	3	3	1	3	0	3	1	3	0	3	1	3	0	3	27.4	10.871	0	15.332	726360000	7985200	4.51E+08	0	2.67E+08	9121800	38159000	0	11478000
Q9HA77	Q9HA77	Probable cysteine--tRNA ligase, mitochondrial	CARS2	sp|Q9HA77	7	7	7	1	5	1	6	1	5	1	6	1	5	1	6	18.8	62.223	0	23.958	726290000	310260	1.45E+08	5478300	5.76E+08	938780	3080600	3233800	33169000
Q99426	Q99426	Tubulin-folding cofactor B	TBCB	sp|Q99426	6	6	6	0	6	0	6	0	6	0	6	0	6	0	6	32.8	27.325	0	21.803	724240000	0	1.94E+08	0	5.3E+08	0	16680000	0	22238000
Q8WU90	Q8WU90	Zinc finger CCCH domain-containing protein 15	ZC3H15	sp|Q8WU9	8	8	8	2	7	7	8	2	7	7	8	2	7	7	8	26.3	48.602	0	40.68	723550000	15590000	1.97E+08	25842000	4.85E+08	19779000	14318000	19643000	16555000
Q10471;Q8	Q10471	Polypeptide N-acetylgalactosaminyltransferase 2;	GALNT2	sp|Q10471	11	11	11	1	8	6	11	1	8	6	11	1	8	6	11	24	64.732	0	44.032	722040000	301310	72592000	16069000	6.33E+08	8540000	10366000	8085800	16542000
P49593	P49593	Protein phosphatase 1F	PPM1F	sp|P49593	11	11	11	0	8	1	9	0	8	1	9	0	8	1	9	42.5	49.83	0	45.833	721680000	0	1.78E+08	1526900	5.42E+08	0	26048000	3089900	9872300
P24941;QC	P24941	Cyclin-dependent kinase 2	CDK2	sp|P24941	8	7	7	0	8	3	7	0	7	2	6	0	7	2	6	38.9	33.929	0	35.693	720310000	0	4.51E+08	4203500	2.65E+08	0	38032000	6445800	8751200
Q9BV86	Q9BV86	N-terminal Xaa-Pro-Lys N-methyltransferase 1;N-t	NTMT1	sp|Q9BV86	9	9	9	0	9	0	6	0	9	0	6	0	9	0	6	53.8	25.387	0	27.884	719410000	0	4.74E+08	0	2.46E+08	0	38310000	0	13425000
P61077	P61077	Ubiquitin-conjugating enzyme E2 D3	UBE2D3	sp|P61077	3	3	1	0	2	1	3	0	2	1	3	0	1	0	1	24.5	16.687	0	4.8906	719010000	0	2.36E+08	2053900	4.81E+08	0	21698000	1569400	18698000
Q08AM6	Q08AM6	Protein VAC14 homolog	VAC14	sp|Q08AM	14	14	14	1	9	10	13	1	9	10	13	1	9	10	13	22.5	87.972	0	45.192	716810000	3999900	82885000	43303000	5.87E+08	0	9950500	28390000	22191000
Q9H3S7	Q9H3S7	Tyrosine-protein phosphatase non-receptor type	PTPN23	sp|Q9H3S7	19	19	19	0	13	1	19	0	13	1	19	0	13	1	19	21.1	178.97	0	70.753	715300000	0	97580000	1371200	6.16E+08	0	9862100	1250900	23758000
Q96HS1	Q96HS1	Serine/threonine-protein phosphatase PGAM5, m	PGAM5	sp|Q96HS1	13	13	13	8	12	9	8	8	12	9	8	8	12	9	8	54	32.004	0	32.463	714960000	1.44E+08	3.64E+08	36132000	1.71E+08	1.37E+08	31920000	27103000	10864000
Q52LJ0	Q52LJ0	Protein FAM98B	FAM98B	sp|Q52LJ0	10	10	7	1	8	4	8	1	8	4	8	1	6	2	6	33.5	45.547	0	73.831	714250000	957690	2.17E+08	21426000	4.75E+08	2709600	29047000	11268000	10982000
Q9NZ08	Q9NZ08	Endoplasmic reticulum aminopeptidase 1	ERAP1	sp|Q9NZ08	10	10	10	1	0	0	10	1	0	0	10	1	0	0	10	14.3	107.23	0	33.581	710780000	162960	0	0	7.11E+08	162960	0	0	29224000
Q9UNL2	Q9UNL2	Translocon-associated protein subunit gamma	SSR3	sp|Q9UNL2	1	1	1	0	1	1	1	0	1	1	1	0	1	1	1	7.6	21.08	0	44.648	710150000	0	2.42E+08	28110000	4.4E+08	0	17602000	15257000	14930000
Q99439	Q99439	Calponin-2	CNN2	sp|Q99439	5	4	4	0	3	3	5	0	2	2	4	0	2	2	4	20.4	33.697	0	17.458	708990000	0	76994000	6076700	6.26E+08	0	10188000	8704800	17719000
Q9NP74	Q9NP74	Palmdelphin	PALMD	sp|Q9NP74	11	11	11	0	0	4	11	0	0	4	11	0	0	4	11	34.3	62.757	0	33.048	707870000	0	0	7584500	7E+08	0	0	7838100	26058000
P08962	P08962	CD63 antigen	CD63	sp|P08962	1	1	1	0	0	0	1	0	0	0	1	0	0	0	1	4.2	25.636	0	12.305	706900000	0	0	0	7.07E+08	0	0	0	23414000
O95104	O95104	Splicing factor, arginine/serine-rich 15	SCAF4	sp|O95104	8	8	5	0	8	2	8	0	8	2	8	0	5	1	5	12.2	125.87	0	32.274	705060000	0	3.28E+08	10547000	3.67E+08	0	27103000	9455500	14495000
Q9P0K7	Q9P0K7	Ankycorbin	RAI14	sp|Q9P0K7	25	25	25	1	8	22	20	1	8	22	20	1	8	22	20	31.3	110.04	0	79.595	705020000	728100	40215000	161590000	5.02E+08	4345200	4929800	79802000	42079000
Q13868	Q13868	Exosome complex component RRP4	EXOSC2	sp|Q13868	9	9	9	1	8	9	8	1	8	9	8	1	8	9	8	46.1	32.789	0	55.492	704830000	4254200	3.17E+08	61741000	3.22E+08	8679600	21294000	43327000	12810000
Q9BVP2	Q9BVP2	Guanine nucleotide-binding protein-like 3	GNL3	sp|Q9BVP2	12	12	12	1	9	4	7	1	9	4	7	1	9	4	7	29.1	61.992	0	51.062	704740000	1.87E+08	3.49E+08	12100000	1.57E+08	0	26483000	13905000	4934800
Q9UGP8	Q9UGP8	Translocation protein SEC63 homolog	SEC63	sp|Q9UGP	10	10	10	0	9	3	10	0	9	3	10	0	9	3	10	19.7	87.996	0	44.85	703700000	0	1.82E+08	5806600	5.16E+08	0	14311000	7891200	18970000
P20936	P20936	Ras GTPase-activating protein 1	RASA1	sp|P20936	10	10	10	0	1	0	10	0	1	0	10	0	1	0	10	13	116.4	0	53.677	703500000	0	4033700	0	6.99E+08	0	355570	0	28766000
P07711	P07711	Cathepsin L1;Cathepsin L1 heavy chain;Cathepsin	CTSL	sp|P07711	5	5	5	1	0	1	5	1	0	1	5	1	0	1	5	20.7	37.564	0	21.849	703190000	20853000	0	1563000	6.81E+08	22758000	0	5107600	22035000
O43847	O43847	Nardilysin	NRD1	sp|O43847	15	15	15	1	12	3	14	1	12	3	14	1	12	3	14	17.9	131.7	0	64.08	701660000	985100	1.41E+08	8179900	5.51E+08	1308400	11526000	2937300	24951000
Q9GZZ1	Q9GZZ1	N-alpha-acetyltransferase 50	NAA50	sp|Q9GZZ1	11	11	11	1	8	8	8	1	8	8	8	1	8	8	8	49.1	19.398	0	27.656	701550000	36906000	2.63E+08	50389000	3.51E+08	61079000	16514000	19641000	11181000
P35270	P35270	Sepiapterin reductase	SPR	sp|P35270	8	8	8	0	8	0	7	0	8	0	7	0	8	0	7	41	28.048	0	44.482	701150000	0	3.8E+08	0	3.21E+08	0	27478000	0	19210000
A1L0T0	A1L0T0	Acetolactate synthase-like protein	ILVBL	sp|A1L0T0	11	11	11	0	6	2	10	0	6	2	10	0	6	2	10	29.6	67.867	0	101.3	700610000	0	1.45E+08	7182600	5.49E+08	0	11435000	7684300	21032000
O14737	O14737	Programmed cell death protein 5	PDCD5	sp|O14737	5	5	5	0	5	0	5	0	5	0	5	0	5	0	5	44.8	14.285	0	18.162	700040000	0	4.54E+08	0	2.46E+08	0	38811000	0	9903500
P60468	P60468	Protein transport protein Sec61 subunit beta	SEC61B	sp|P60468	3	3	3	0	3	3	3	0	3	3	3	0	3	3	3	37.5	9.9743	0	11.619	699400000	0	2.6E+08	25508000	4.14E+08	0	23069000	17291000	16740000
Q9Y6B6	Q9Y6B6	GTP-binding protein SAR1b	SAR1B	sp|Q9Y6B6	9	4	4	0	9	3	9	0	4	1	4	0	4	1	4	38.9	22.41	0	41.248	699040000	0	1.14E+08	285580	5.85E+08	0	10059000	276850	23937000
Q96C19;Q	Q96C19	EF-hand domain-containing protein D2	EFHD2	sp|Q96C19	7	7	7	1	6	1	7	1	6	1	7	1	6	1	7	35.4	26.697	0	26.443	697240000	11631000	1.22E+08	2404100	5.61E+08	6910200	12942000	2497700	24735000
Q9BYC5	Q9BYC5	Alpha-(1,6)-fucosyltransferase	FUT8	sp|Q9BYC5	12	12	12	0	1	9	12	0	1	9	12	0	1	9	12	25.9	66.515	0	63.965	696830000	0	663840	29461000	6.67E+08	0	279020	5725600	41270000
Q9Y2Z4	Q9Y2Z4	Tyrosine--tRNA ligase, mitochondrial	YARS2	sp|Q9Y2Z4	9	9	9	0	9	0	6	0	9	0	6	0	9	0	6	26	53.198	0	59.949	695400000	0	4.69E+08	0	2.26E+08	0	39986000	0	10672000
P49959	P49959	Double-strand break repair protein MRE11A	MRE11A	sp|P49959	8	8	8	0	8	3	5	0	8	3	5	0	8	3	5	15.7	80.592	0	28.504	694270000	0	4.69E+08	7378300	2.18E+08	0	28349000	15112000	11793000
Q9Y2D5	Q9Y2D5	A-kinase anchor protein 2	AKAP2	sp|Q9Y2D5	16	16	16	0	3	8	16	0	3	8	16	0	3	8	16	29.5	94.659	0	126.95	694120000	0	17340000	25411000	6.51E+08	0	1833300	20141000	23419000
Q9NVD7	Q9NVD7	Alpha-parvin	PARVA	sp|Q9NVD	8	8	6	0	4	1	8	0	4	1	8	0	4	1	6	30.1	42.243	0	94.267	693800000	0	31778000	1685800	6.6E+08	0	2567300	2509600	26014000
Q9UNF0	Q9UNF0	Protein kinase C and casein kinase substrate in ne	PACSIN2	sp|Q9UNF	9	9	9	0	9	4	7	0	9	4	7	0	9	4	7	24.5	55.738	0	30.034	693090000	0	2.33E+08	11139000	4.49E+08	0	18788000	9032300	18660000
Q12888	Q12888	Tumor suppressor p53-binding protein 1	TP53BP1	sp|Q12888	22	22	22	1	9	5	22	1	9	5	22	1	9	5	22	18.1	213.57	0	77.457	692970000	1099600	67949000	12536000	6.11E+08	1684400	7250500	8037700	23684000
P60059	P60059	Protein transport protein Sec61 subunit gamma	SEC61G	sp|P60059	2	2	2	0	2	2	2	0	2	2	2	0	2	2	2	36.8	7.7412	0	12.034	691370000	0	2.68E+08	11558000	4.12E+08	0	24360000	8557600	15399000
Q9GZZ9	Q9GZZ9	Ubiquitin-like modifier-activating enzyme 5	UBA5	sp|Q9GZZ9	6	6	6	0	6	0	4	0	6	0	4	0	6	0	4	29.2	44.863	0	59.985	690780000	0	2.92E+08	0	3.99E+08	0	23160000	0	18989000
P19784	P19784	Casein kinase II subunit alpha	CSNK2A2	sp|P19784	11	11	11	2	10	7	8	2	10	7	8	2	10	7	8	45.1	41.213	0	48.274	689940000	13607000	3.02E+08	36847000	3.37E+08	15994000	31263000	21025000	10595000
Q9BSJ2	Q9BSJ2	Gamma-tubulin complex component 2	TUBGCP2	sp|Q9BSJ2	14	14	14	0	9	10	13	0	9	10	13	0	9	10	13	21	102.53	0	59.001	689920000	0	98322000	29318000	5.62E+08	0	13643000	18945000	18905000
Q8NBJ5	Q8NBJ5	Procollagen galactosyltransferase 1	COLGALT1	sp|Q8NBJ5	14	14	14	0	8	1	14	0	8	1	14	0	8	1	14	22.5	71.635	0	41.473	689010000	0	41777000	1101600	6.46E+08	0	3232300	1046400	26717000
Q9NZB2	Q9NZB2	Constitutive coactivator of PPAR-gamma-like prot	FAM120A	sp|Q9NZB2	17	17	17	7	13	14	15	7	13	14	15	7	13	14	15	21.8	121.89	0	68.715	688420000	75556000	1.57E+08	71699000	3.84E+08	64466000	22337000	50687000	15884000
O94776	O94776	Metastasis-associated protein MTA2	MTA2	sp|O94776	14	14	13	3	13	7	14	3	13	7	14	3	12	7	13	25.6	75.022	0	40.938	688070000	2203800	2.76E+08	14056000	3.96E+08	4786000	20641000	10929000	15884000
P61088;Q5	P61088;Q5	Ubiquitin-conjugating enzyme E2 N;Putative ubiq	UBE2N;UB	sp|P61088	6	6	6	1	6	1	5	1	6	1	5	1	6	1	5	52	17.138	0	26.116	687430000	991260	4.76E+08	519840	2.1E+08	925500	45943000	376170	3448300
Q9Y2Z0	Q9Y2Z0	Suppressor of G2 allele of SKP1 homolog	SUGT1	sp|Q9Y2Z0	7	7	7	0	7	0	5	0	7	0	5	0	7	0	5	26	41.024	0	47.727	686790000	0	4.44E+08	0	2.43E+08	0	38236000	0	10878000
Q14527	Q14527	Helicase-like transcription factor	HLTF	sp|Q14527	12	12	12	0	10	9	12	0	10	9	12	0	10	9	12	16.7	113.93	0	49.116	685690000	0	1.9E+08	40653000	4.55E+08	0	19502000	28572000	14698000
Q8IX12	Q8IX12	Cell division cycle and apoptosis regulator protein	CCAR1	sp|Q8IX12	17	17	17	2	17	9	13	2	17	9	13	2	17	9	13	17.8	132.82	0	41.924	685230000	2457700	3.96E+08	20300000	2.66E+08	2354400	37436000	15020000	7154900
Q08379;RE	Q08379	Golgin subfamily A member 2	GOLGA2	sp|Q08379	13	13	13	1	13	4	9	1	13	4	9	1	13	4	9	19.3	113.08	0	65.093	684240000	303950	2.46E+08	12394000	4.25E+08	1087800	29834000	9665400	7245700
Q9BQ67	Q9BQ67	Glutamate-rich WD repeat-containing protein 1	GRWD1	sp|Q9BQ67	9	9	9	3	8	4	3	3	8	4	3	3	8	4	3	30.5	49.419	0	31.592	683310000	35229000	4.23E+08	36897000	1.88E+08	17563000	33417000	33226000	20858000
P08240	P08240	Signal recognition particle receptor subunit alpha	SRPR	sp|P08240	12	12	12	0	11	4	11	0	11	4	11	0	11	4	11	23.5	69.81	0	52.949	678140000	0	1.62E+08	5865300	5.11E+08	0	13541000	3776600	21871000
P53041;E7	P53041	Serine/threonine-protein phosphatase 5	PPP5C	sp|P53041	15	15	15	0	12	0	11	0	12	0	11	0	12	0	11	34.9	56.878	0	117.26	677340000	0	3.98E+08	0	2.79E+08	0	34303000	0	12282000
P52306	P52306	Rap1 GTPase-GDP dissociation stimulator 1	RAP1GDS1	sp|P52306	8	8	8	0	2	0	8	0	2	0	8	0	2	0	8	18.5	66.316	0	55.674	676760000	0	17020000	0	6.6E+08	0	1500300	0	27132000
P82673	P82673	28S ribosomal protein S35, mitochondrial	MRPS35	sp|P82673	8	8	8	0	8	4	5	0	8	4	5	0	8	4	5	35.6	36.844	0	23.78	674810000	0	4.3E+08	18510000	2.26E+08	0	24833000	15978000	18845000
P06454	P06454	Prothymosin alpha;Prothymosin alpha, N-termina	PTMA	sp|P06454	3	3	3	1	3	1	3	1	3	1	3	1	3	1	3	25.2	12.203	0	12.655	673670000	13296000	1.98E+08	4923000	4.57E+08	13581000	17900000	3379100	17751000
Q15836	Q15836	Vesicle-associated membrane protein 3	VAMP3	sp|Q15836	6	6	4	1	5	2	5	1	5	2	5	0	3	2	3	53	11.309	0	52.593	673180000	639830	1.36E+08	751330	5.36E+08	315310	12859000	167980	21819000
Q14643	Q14643	Inositol 1,4,5-trisphosphate receptor type 1	ITPR1	sp|Q14643	17	17	15	1	2	5	16	1	2	5	16	1	0	4	14	8.6	313.93	0	83.009	671700000	3539300	3656300	11132000	6.53E+08	2195500	662530	4354400	31000000
Q9BWF3	Q9BWF3	RNA-binding protein 4	RBM4	sp|Q9BWF	10	10	3	3	9	7	7	3	9	7	7	1	3	2	2	38.7	40.313	0	102.95	671560000	4274100	2.69E+08	27333000	3.71E+08	5849300	25217000	16533000	14030000
Q9HAV7	Q9HAV7	GrpE protein homolog 1, mitochondrial	GRPEL1	sp|Q9HAV	6	6	6	1	5	3	6	1	5	3	6	1	5	3	6	33.2	24.279	0	14.122	671310000	313920	3.32E+08	6489000	3.32E+08	1475200	21585000	4983400	19580000
P81605	P81605	Dermcidin;Survival-promoting peptide;DCD-1	DCD	sp|P81605	5	5	5	1	3	5	5	1	3	5	5	1	3	5	5	42.7	11.284	0	29.221	670380000	5668900	1.6E+08	371290000	1.33E+08	23871000	18200000	228270000	4435600
Q5RKV6	Q5RKV6	Exosome complex component MTR3	EXOSC6	sp|Q5RKV6	6	6	6	1	6	6	5	1	6	6	5	1	6	6	5	29.8	28.235	0	40.051	670340000	1601400	3.59E+08	44950000	2.65E+08	8236500	21917000	34547000	9647200
Q15003	Q15003	Condensin complex subunit 2	NCAPH	sp|Q15003	14	14	14	0	9	4	13	0	9	4	13	0	9	4	13	26.3	82.562	0	62.751	669940000	0	1.74E+08	11607000	4.85E+08	0	14475000	8713600	19210000
P19525	P19525	Interferon-induced, double-stranded RNA-activat	EIF2AK2	sp|P19525	9	9	9	0	9	6	8	0	9	6	8	0	9	6	8	18.3	62.094	0	24.359	669730000	0	2.29E+08	33139000	4.07E+08	0	16028000	26603000	16593000

Q53EL6	Q53EL6	Programmed cell death protein 4	PDCD4	sp|Q53EL6	8	8	8	4	7	1	0	4	7	1	0	4	7	1	0	23.9	51.735	0	46.26	667700000	1.87E+08	4.79E+08	2130500	0	54675000	1.66E+08	9967200	0
P82933	P82933	28S ribosomal protein S9, mitochondrial	MRPS9	sp|P82933	10	10	10	1	8	5	8	1	8	5	8	1	8	5	8	33.6	45.834	0	33.992	666090000	2351000	3.78E+08	31302000	2.55E+08	4139200	33792000	13188000	16040000
Q9UHD2	Q9UHD2	Serine/threonine-protein kinase TBK1	TBK1	sp|Q9UHD	16	16	16	6	6	13	10	6	6	13	10	6	6	13	10	32.1	83.641	0	83.512	664530000	84715000	48720000	211950000	3.19E+08	85543000	8298400	134670000	16055000
Q96C36	Q96C36	Pyrroline-5-carboxylate reductase 2	PYCR2	sp|Q96C3	7	7	7	0	7	4	5	0	7	4	5	0	7	4	5	25.9	33.637	0	31.579	664190000	0	4.11E+08	6019400	2.48E+08	0	34025000	7813500	8582900
O15347	O15347	High mobility group protein B3	HMGB3	sp|O15347	5	5	5	0	5	0	4	0	5	0	4	0	5	0	4	18.5	22.98	0	18.311	664180000	0	4.47E+08	0	2.17E+08	0	39918000	0	8430300
P12532;P1	P12532	Creatine kinase U-type, mitochondrial	CKMT1A	sp|P12532	8	8	8	0	8	1	0	0	8	1	0	0	8	1	0	34.1	47.036	0	137.64	663290000	0	6.61E+08	2337400	0	0	58263000	1570700	0
O94903	O94903	Proline synthase co-transcribed bacterial homolo	PROSC	sp|O94903	5	5	5	0	5	1	2	0	5	1	2	0	5	1	2	25.5	30.344	0	23.676	662610000	0	4.68E+08	555120	1.94E+08	0	46264000	558560	2797200
Q14CX7	Q14CX7	N-alpha-acetyltransferase 25, NatB auxiliary subu	NAA25	sp|Q14CX	13	13	13	0	10	0	10	0	10	0	10	0	10	0	10	14.8	112.29	0	36.959	661860000	0	1.6E+08	0	5.02E+08	0	16163000	0	18576000
Q32MZ4	Q32MZ4	Leucine-rich repeat flightless-interacting protein	LRRFIP1	sp|Q32MZ	10	10	9	0	9	4	10	0	9	4	10	0	8	3	9	18.7	89.252	0	53.472	658090000	0	1.92E+08	11485000	4.55E+08	0	15217000	13453000	14648000
O14925;Q	O14925	Mitochondrial import inner membrane transloca	TIMM23	sp|O14925	7	7	7	0	7	3	7	0	7	3	7	0	7	3	7	61.7	21.943	0	71.402	656790000	0	2.05E+08	7254700	4.45E+08	0	16417000	11580000	13223000
Q969Q0	Q969Q0	60S ribosomal protein L36a-like	RPL36AL	sp|Q969Q	7	7	2	0	6	7	6	0	6	7	6	0	2	2	1	45.3	12.469	0	19.239	656190000	0	2.28E+08	66584000	3.62E+08	0	20450000	43820000	15422000
P48449	P48449	Lanosterol synthase	LSS	sp|P48449	12	12	12	0	12	0	11	0	12	0	11	0	12	0	11	22.3	83.308	0	37.632	655180000	0	2.07E+08	0	4.48E+08	0	16720000	0	19970000
P57088	P57088	Transmembrane protein 33	TMEM33	sp|P57088	4	4	4	0	4	3	4	0	4	3	4	0	4	3	4	16.2	27.978	0	22.007	652860000	0	1.61E+08	21965000	4.7E+08	0	12808000	16675000	18771000
P78346	P78346	Ribonuclease P protein subunit p30	RPP30	sp|P78346	8	8	8	0	7	4	8	0	7	4	8	0	7	4	8	39.9	29.321	0	30.694	652530000	0	2.19E+08	5425900	4.29E+08	0	20812000	9563400	10161000
Q92665	Q92665	28S ribosomal protein S31, mitochondrial	MRPS31	sp|Q92665	9	9	9	1	9	6	7	1	9	6	7	1	9	6	7	37.5	45.318	0	44.725	651400000	1306900	4.19E+08	13257000	2.18E+08	2145600	35311000	10826000	7808800
Q5SSJ5	Q5SSJ5	Heterochromatin protein 1-binding protein 3	HP1BP3	sp|Q5SSJ5	12	12	12	1	6	9	10	1	6	9	10	1	6	9	10	26.6	61.206	0	30.279	651250000	272740	30223000	75855000	5.45E+08	1147900	1718400	42260000	31194000
Q00577	Q00577	Transcriptional activator protein Pur-alpha	PURA	sp|Q00577	6	6	6	0	5	4	4	0	5	4	4	0	5	4	4	32.3	34.91	0	93.412	650860000	0	2.68E+08	86672000	2.97E+08	0	18936000	63656000	11437000
P62993	P62993	Growth factor receptor-bound protein 2	GRB2	sp|P62993	7	7	7	0	7	3	7	0	7	3	7	0	7	3	7	41.9	25.206	0	25.15	649820000	0	2.55E+08	5314400	3.9E+08	0	22939000	5206900	13911000
Q92879	Q92879	CUGBP Elav-like family member 1	CELF1	sp|Q92879	9	9	9	0	7	2	7	0	7	2	7	0	7	2	7	27.8	52.063	0	39.834	649190000	0	2.33E+08	5095000	4.11E+08	0	11679000	1527100	26891000
Q9H5K3	Q9H5K3	Protein O-mannose kinase	POMK	sp|Q9H5K	2	2	2	0	2	0	2	0	2	0	2	0	2	0	2	7.4	40.05	0	7.0596	649040000	0	48807000	0	6E+08	0	5791400	0	23196000
Q658P3	Q658P3	Metalloreductase STEAP3	STEAP3	sp|Q658P	4	4	4	0	3	2	4	0	3	2	4	0	3	2	4	13.7	54.6	0	14.243	647670000	0	18331000	16952000	6.12E+08	0	2371500	19644000	16177000
P55039	P55039	Developmentally-regulated GTP-binding protein	DRG2	sp|P55039	7	7	7	2	4	0	7	2	4	0	7	2	4	0	7	25.3	40.746	0	20.49	647350000	10804000	1.15E+08	0	5.22E+08	17038000	13000000	0	12355000
Q08752	Q08752	Peptidyl-prolyl cis-trans isomerase D	PPID	sp|Q08752	11	11	11	0	10	0	11	0	10	0	11	0	10	0	11	38.1	40.763	0	40.586	645220000	0	2.65E+08	0	3.81E+08	0	23345000	0	15511000
Q96A35	Q96A35	39S ribosomal protein L24, mitochondrial	MRPL24	sp|Q96A3	3	3	3	0	3	3	3	0	3	3	3	0	3	3	3	23.1	24.915	0	10.724	644630000	0	1.79E+08	25672000	4.4E+08	0	14304000	20238000	16576000
P13807;P5	P13807	Glycogen [starch] synthase, muscle	GYS1	sp|P13807	11	11	11	0	10	2	8	0	10	2	8	0	10	2	8	22.1	83.785	0	24.062	644430000	0	2.71E+08	4042900	3.7E+08	0	30527000	3018600	8245300
Q9HCC0	Q9HCC0	Methylcrotonoyl-CoA carboxylase beta chain, mit	MCCC2	sp|Q9HCC	10	10	10	1	10	4	9	1	10	4	9	1	10	4	9	27.4	61.332	0	36.06	644180000	487540	3.3E+08	4437900	3.09E+08	931500	34117000	3067600	6648600
Q96AY3	Q96AY3	Peptidyl-prolyl cis-trans isomerase FKBP10	FKBP10	sp|Q96AY	10	10	10	0	6	2	10	0	6	2	10	0	6	2	10	17.4	64.244	0	25.811	641940000	0	28602000	1900700	6.11E+08	0	2553600	768490	25622000
P35222	P35222	Catenin beta-1	CTNNB1	sp|P35222	13	13	11	0	11	3	13	0	11	3	13	0	9	1	11	24.2	85.496	0	75.795	640890000	0	2.2E+08	13942000	4.07E+08	0	17337000	13976000	14168000
P46977	P46977	Dolichyl-diphosphooligosaccharide--protein glyco	STT3A	sp|P46977	8	7	7	0	8	6	7	0	7	5	6	0	7	5	6	11.5	80.529	0	38.413	640840000	0	2.46E+08	20102000	3.74E+08	0	17952000	15822000	16849000
Q99720	Q99720	Sigma non-opioid intracellular receptor 1	SIGMAR1	sp|Q9972	4	4	4	0	4	2	4	0	4	2	4	0	4	2	4	29.6	25.127	0	47.83	636840000	0	2.64E+08	2330900	3.71E+08	0	20493000	5188900	14387000
Q9Y2V2	Q9Y2V2	Calcium-regulated heat stable protein 1	CARHSP1	sp|Q9Y2V	5	5	5	0	4	1	5	0	4	1	5	0	4	1	5	64.6	15.892	0	38.749	636030000	0	1.33E+08	1544300	5.01E+08	0	12091000	1249900	20065000
P28340	P28340	DNA polymerase delta catalytic subunit	POLD1	sp|P28340	15	15	15	0	10	3	12	0	10	3	12	0	10	3	12	21.3	123.63	0	60.881	635050000	0	2.37E+08	5877100	3.92E+08	0	20666000	2290800	18019000
P23193;Q1	P23193	Transcription elongation factor A protein 1	TCEA1	sp|P23193	13	13	13	0	13	1	12	0	13	1	12	0	13	1	12	49.2	33.969	0	44.454	634660000	0	2.35E+08	10357000	3.89E+08	0	17079000	11629000	14982000
Q15020	Q15020	Squamous cell carcinoma antigen recognized by T	SART3	sp|Q1502	12	12	12	0	11	5	11	0	11	5	11	0	11	5	11	16.7	109.93	0	40.193	633770000	0	2.55E+08	5388700	3.73E+08	0	18346000	2862300	20247000
O75962;O	O75962	Triple functional domain protein	TRIO	sp|O75962	19	19	19	0	1	8	18	0	1	8	18	0	1	8	18	10.9	346.9	0	57.949	633220000	0	2594300	18891000	6.12E+08	0	754830	6152400	31174000
Q13596	Q13596	Sorting nexin-1	SNX1	sp|Q13596	12	10	10	0	8	3	12	0	6	2	10	0	6	2	10	29.5	59.069	0	43.013	632300000	0	1.18E+08	4389300	5.1E+08	0	9831600	4030200	20444000
P17096	P17096	High mobility group protein HMG-I/HMG-Y	HMGA1	sp|P17096	5	5	5	1	4	4	5	1	4	4	5	1	4	4	5	38.3	11.676	0	21.833	631800000	749730	73558000	38393000	5.19E+08	1287500	8652300	30851000	13590000
Q6NYC1	Q6NYC1	Bifunctional arginine demethylase and lysyl-hydr	JMJD6	sp|Q6NYC	6	6	6	1	6	1	4	1	6	1	4	1	6	1	4	22.8	46.461	0	27.2	631670000	1125200	2.62E+08	6915600	3.61E+08	0	20679000	8609600	13339000
P30519	P30519	Heme oxygenase 2	HMOX2	sp|P30519	8	8	8	0	7	0	5	0	7	0	5	0	7	0	5	41.1	36.032	0	51.647	631530000	0	4.52E+08	0	1.79E+08	0	36892000	0	10339000
Q07157	Q07157	Tight junction protein ZO-1	TJP1	sp|Q07157	26	26	26	1	10	25	20	1	10	25	20	1	10	25	20	22.5	195.46	0	69.417	631030000	810120	57144000	230470000	3.43E+08	1094300	5204500	161390000	7120000
Q9NX63	Q9NX63	MICOS complex subunit MIC19	CHCHD3	sp|Q9NX6	7	7	7	0	6	4	6	0	6	4	6	0	6	4	6	23.3	26.152	0	27.419	630570000	0	3.57E+08	14643000	2.59E+08	0	29983000	11700000	10294000
Q9BV36	Q9BV36	Melanophilin	MLPH	sp|Q9BV3	13	13	13	0	0	6	13	0	0	6	13	0	0	6	13	33.8	65.948	0	80.823	629360000	0	0	32602000	5.97E+08	0	0	24708000	21742000
O00193	O00193	Small acidic protein	SMAP	sp|O00193	5	5	5	0	5	1	5	0	5	1	5	0	5	1	5	30.1	20.332	0	56.838	629130000	0	3.26E+08	140040	3.03E+08	0	11312000	704830	29254000
Q7Z2W7	Q7Z2W7	Transient receptor potential cation channel subfa	TRPM8	sp|Q7Z2W	1	1	1	0	0	1	1	0	0	1	1	0	0	1	1	0.7	127.68	0.007036	1.7018	629030000	0	0	624010000	5018700	0	0	412870000	206400
P51532;P5	P51532	Transcription activator BRG1	SMARCA4	sp|P51532	16	16	16	2	14	6	15	2	14	6	15	2	14	6	15	13.8	184.64	0	53.576	628490000	1654700	1.65E+08	8838800	4.53E+08	1412300	17712000	7901300	13392000
P18754	P18754	Regulator of chromosome condensation	RCC1	sp|P18754	10	10	10	0	9	1	9	0	9	1	9	0	9	1	9	36.3	44.969	0	58.905	628420000	0	2.81E+08	1160300	3.47E+08	0	25289000	990580	13486000
O60832	O60832	H/ACA ribonucleoprotein complex subunit 4	DKC1	sp|O60832	8	8	8	0	7	8	7	0	7	8	7	0	7	8	7	20.8	57.673	0	41.416	627870000	0	2.36E+08	47030000	3.44E+08	0	15836000	37344000	13430000
Q9UNH7	Q9UNH7	Sorting nexin-6;Sorting nexin-6, N-terminally proc	SNX6	sp|Q9UNH	9	9	9	0	7	2	9	0	7	2	9	0	7	2	9	20.2	46.648	0	25.316	627370000	0	1.93E+08	4894000	4.3E+08	0	12450000	4128200	21364000
Q5JTH9	Q5JTH9	RRP12-like protein	RRP12	sp|Q5JTH9	16	16	15	1	12	11	15	1	12	11	15	1	11	11	14	16.4	143.7	0	48.909	625930000	5735300	1.56E+08	45809000	4.18E+08	7755300	11355000	34830000	13554000
Q8N1G4	Q8N1G4	Leucine-rich repeat-containing protein 47	LRRC47	sp|Q8N1G	11	11	11	1	10	1	8	1	10	1	8	1	10	1	8	28.8	63.472	0	39.261	624940000	790850	3.4E+08	573670	2.83E+08	653610	36104000	527770	5531400
Q96A65	Q96A65	Exocyst complex component 4	EXOC4	sp|Q96A6	18	18	18	1	8	9	17	1	8	9	17	1	8	9	17	27.6	110.5	0	71.629	624150000	423770	65152000	25624000	5.33E+08	1702900	6298500	13629000	23673000
Q9NZT1	Q9NZT1	Calmodulin-like protein 5	CALML5	sp|Q9NZT	11	11	11	1	4	11	3	1	4	11	3	1	4	11	3	79.5	15.892	0	73.183	624150000	384310	30608000	541720000	51434000	942170	3940700	318730000	1063200
O43583	O43583	Density-regulated protein	DENR	sp|O43583	5	5	5	0	5	0	5	0	5	0	5	0	5	0	5	27.3	22.092	0	15.725	622950000	0	3.21E+08	0	3.02E+08	0	28148000	0	12571000
Q9UHD1	Q9UHD1	Cysteine and histidine-rich domain-containing pro	CHORDC1	sp|Q9UHD	9	9	9	0	9	0	8	0	9	0	8	0	9	0	8	39.5	37.489	0	33.154	622640000	0	3.14E+08	0	3.09E+08	0	26129000	0	14236000
Q15642	Q15642	Cdc42-interacting protein 4	TRIP10	sp|Q15642	9	9	9	1	6	3	9	1	6	3	9	1	6	3	9	22.3	68.351	0	103.97	622610000	867640	40721000	4364400	5.77E+08	1036700	2754600	2704400	24610000
Q66K74	Q66K74	Microtubule-associated protein 1S;MAP1S heavy	MAP1S	sp|Q66K7	8	8	8	0	5	3	8	0	5	3	8	0	5	3	8	12.9	112.21	0	60.952	622560000	0	2.43E+08	4480300	3.75E+08	0	10871000	11545000	17460000
Q92520	Q92520	Protein FAM3C	FAM3C	sp|Q9252	6	6	6	0	6	1	6	0	6	1	6	0	6	1	6	34.4	24.68	0	23.693	621480000	0	1.62E+08	454000	4.59E+08	0	14485000	1067100	17918000
Q6PI48	Q6PI48	Aspartate--tRNA ligase, mitochondrial	DARS2	sp|Q6PI48	9	9	9	0	8	0	9	0	8	0	9	0	8	0	9	21.6	73.562	0	40.172	620090000	0	3.15E+08	0	3.05E+08	0	29518000	0	10815000
Q9BXW7	Q9BXW7	Cat eye syndrome critical region protein 5	CECR5	sp|Q9BXW	7	7	7	0	7	0	6	0	7	0	6	0	7	0	6	28.8	46.321	0	89.721	619970000	0	4.78E+08	0	1.42E+08	0	41015000	0	6968100
P14406	P14406	Cytochrome c oxidase subunit 7A2, mitochondrial	COX7A2	sp|P14406	2	2	2	0	2	1	2	0	2	1	2	0	2	1	2	27.7	9.3959	0	5.1941	619690000	0	2.36E+08	3488800	3.81E+08	0	20623000	3007400	15136000
Q9Y570	Q9Y570	Protein phosphatase methylesterase 1	PPME1	sp|Q9Y57	11	11	11	0	11	2	9	0	11	2	9	0	11	2	9	34.5	42.315	0	89.016	619340000	0	3.41E+08	1276200	2.77E+08	0	31694000	1249500	9350000
Q9BZE4	Q9BZE4	Nucleolar GTP-binding protein 1	GTPBP4	sp|Q9BZE	13	13	13	3	10	10	8	3	10	10	8	3	10	10	8	27	73.964	0	46.929	619090000	11433000	3.43E+08	48169000	2.17E+08	10697000	31896000	34200000	6127100
O00505	O00505	Importin subunit alpha-4	KPNA3	sp|O00505	8	8	4	1	6	3	6	1	6	3	6	0	3	2	3	19.4	57.81	0	36.92	618830000	7350400	1.69E+08	4731600	4.38E+08	6205600	15247000	5471300	16482000
Q7Z2W9	Q7Z2W9	39S ribosomal protein L21, mitochondrial	MRPL21	sp|Q7Z2W	5	5	5	1	4	1	4	1	4	1	4	1	4	1	4	34.1	22.814	0	20.436	618800000	333910	2.15E+08	2850600	4.01E+08	0	18097000	2393600	16841000
O60502	O60502	Protein O-GlcNAcase	MGEA5	sp|O60502	12	12	12	0	9	1	12	0	9	1	12	0	9	1	12	21.2	102.91	0	48.076	617710000	0	1.59E+08	1971700	4.57E+08	0	9018900	2737000	22351000
Q8WW12	Q8WW12	PEST proteolytic signal-containing nuclear protei	PCNP	sp|Q8WW	7	7	7	1	7	2	7	1	7	2	7	1	7	2	7	44.9	18.925	0	38.667	617310000	304370	2.33E+08	1336500	3.83E+08	262720	22731000	643670	13827000
O00178	O00178	GTP-binding protein 1	GTPBP1	sp|O00178	10	10	10	2	10	4	9	2	10	4	9	2	10	4	9	23.2	72.453	0	35.39	615700000	99379000	3.15E+08	16797000	1.84E+08	1.2E+08	11157000	13568000	1808900
Q06203	Q06203	Amidophosphoribosyltransferase	PPAT	sp|Q06203	9	9	9	0	9	0	5	0	9	0	5	0	9	0	5	27.3	57.398	0	47.604	614120000	0	3.78E+08	0	2.36E+08	0	30254000	0	12762000
P00533	P00533	Epidermal growth factor receptor	EGFR	sp|P00533	11	11	11	0	1	4	11	0	1	4	11	0	1	4	11	13.6	134.28	0	40.127	613290000	0	15421000	7865200	5.9E+08	0	1880600	4863200	24165000
Q9UGV2	Q9UGV2	Protein NDRG3	NDRG3	sp|Q9UGV	6	6	6	0	5	0	6	0	5	0	6	0	5	0	6	30.9	41.408	0	76.331	613280000	0	1.29E+08	0	4.84E+08	0	14308000	0	16989000
P61513	P61513	60S ribosomal protein L37a	RPL37A	sp|P61513	3	3	3	1	3	3	3	1	3	3	3	1	3	3	3	29.3	10.275	0	20.49	613010000	31274000	1.48E+08	109010000	3.25E+08	37115000	10049000	68411000	9624100
P01111	P01111	GTPase NRas	NRAS	sp|P01111	6	6	0	0	4	0	6	0	4	0	6	0	0	0	0	34.4	21.229	0	48.269	612830000	0	1.16E+08	0	4.97E+08	0	10108000	0	18053000
Q5VTR2	Q5VTR2	E3 ubiquitin-protein ligase BRE1A	RNF20	sp|Q5VTR	18	18	17	3	16	3	14	3	16	3	14	3	15	3	13	26.4	113.66	0	44.667	612800000	4156300	1.82E+08	5687100	4.21E+08	1214900	14407000	4768300	20944000
P19387	P19387	DNA-directed RNA polymerase II subunit RPB3	POLR2C	sp|P19387	6	6	6	0	5	1	5	0	5	1	5	0	5	1	5	32.7	31.441	0	41.612	610600000	0	1.72E+08	6409300	4.32E+08	0	12537000	6600300	18112000
Q96EK6	Q96EK6	Glucosamine 6-phosphate N-acetyltransferase	GNPNAT1	sp|Q96EK	3	3	3	0	3	0	3	0	3	0	3	0	3	0	3	33.7	20.749	0	22.521	609860000	0	2.56E+08	0	3.54E+08	0	21187000	0	15940000
P11166	P11166	Solute carrier family 2, facilitated glucose transpo	SLC2A1	sp|P11166	5	5	5	1	5	2	5	1	5	2	5	1	5	2	5	10	54.083	0	17.62	608160000	295090	2.64E+08	8365700	3.35E+08	1730800	29238000	3444000	8590500
Q9Y5K8	Q9Y5K8	V-type proton ATPase subunit D	ATP6V1D	sp|Q9Y5K	3	3	3	1	3	0	3	1	3	0	3	1	3	0	3	27.1	28.262	0	34.258	608050000	97181000	1.08E+08	0	4.03E+08	1.11E+08	4011700	0	8216500
Q2TAA2	Q2TAA2	Isoamyl acetate-hydrolyzing esterase 1 homolog	IAH1	sp|Q2TAA	4	4	4	0	4	1	3	0	4	1	3	0	4	1	3	21	27.598	0	12.106	607880000	0	1.49E+08	615590	4.58E+08	0	18670000	2526500	11214000
P61020	P61020	Ras-related protein Rab-5B	RAB5B	sp|P61020	8	5	5	1	8	2	6	1	5	1	4	1	5	1	4	52.6	23.707	0	63.589	607700000	197240	1.77E+08	1045900	4.29E+08	1249800	21196000	5565100	6169900
Q59GN2;P	Q59GN2;P	Putative 60S ribosomal protein L39-like 5;60S ribo	RPL39P5;R	sp|Q59GN	2	2	2	1	2	2	2	1	2	2	2	1	2	2	2	19.6	6.3225	0	5.7197	607150000	55256000	1.37E+08	50906000	3.64E+08	20502000	15726000	35273000	18804000
O75400	O75400	Pre-mRNA-processing factor 40 homolog A	PRPF40A	sp|O7540	11	11	11	2	11	6	8	2	11	6	8	2	11	6	8	14.3	108.8	0	32.647	606700000	3601400	3.49E+08	21648000	2.32E+08	5600900	25845000	20399000	6631200
O00170	O00170	AH receptor-interacting protein	AIP	sp|O0017	8	8	8	0	8	0	8	0	8	0	8	0	8	0	8	33.3	37.636	0	32.94	605310000	0	2.1E+08	0	3.96E+08	0	18682000	0	16065000
Q9UBQ5	Q9UBQ5	Eukaryotic translation initiation factor 3 subunit K	EIF3K	sp|Q9UBQ	5	5	5	2	5	2	5	2	5	2	5	2	5	2	5	25.2	25.059	0	18.307	603760000	34353000	1.25E+08	8136000	4.36E+08	42824000	9263500	4698600	11994000
Q01085	Q01085	Nucleolysin TIAR	TIAL1	sp|Q01085	7	7	5	1	7	2	6	1	7	2	6	0	5	1	4	25.1	41.59	0	16.426	603170000	480370	2.09E+08	10351000	3.84E+08	1794700	8522400	2309000	28978000
O95861	O95861	3(2),5-bisphosphate nucleotidase 1	BPNT1	sp|O95861	11	11	11	0	11	0	5	0	11	0	5	0	11	0	5	48.7	33.392	0	49.05	602410000	0	4.33E+08	0	1.69E+08	0	36334000	0	8810100
Q13492;O	Q13492	Phosphatidylinositol-binding clathrin assembly pr	PICALM	sp|Q13492	6	6	6	0	4	1	6	0	4	1	6	0	4	1	6	13.8	70.754	0	45.473	601810000	0	99847000	245970	5.02E+08	0	5409800	1023000	21919000
Q9Y3D9	Q9Y3D9	28S ribosomal protein S23, mitochondrial	MRPS23	sp|Q9Y3D	9	9	9	3	8	5	7	3	8	5	7	3	8	5	7	60	21.77	0	25.5	601650000	1643400	3.2E+08	25148000	2.55E+08	1089100	29476000	15785000	10880000
Q96PK6	Q96PK6	RNA-binding protein 14	RBM14	sp|Q96PK	13	13	13	2	11	12	12	2	11	12	12	2	11	12	12	26.9	69.491	0	44.785	601180000	2920400	1.43E+08	82089000	3.73E+08	3248300	10049000	52744000	19650000
P35659	P35659	Protein DEK	DEK	sp|P35659	7	7	7	1	6	5	5	1	6	5	5	1	6	5	5	17.3	42.674	0	15.985	600070000	36987000	3.6E+08	13497000	1.9E+08	0	29344000	10510000	8740000

P49589	P49589	Cysteine--tRNA ligase, cytoplasmic	CARS	sp|P49589	11	11	11	1	9	2	10	1	9	2	10	1	9	2	10	16.8	85.472	0	60.933	598850000	1745300	1.8E+08	3255600	4.14E+08	2714100	11146000	3705400	19269000
Q9NYL9;Q	Q9NYL9	Tropomodulin-3	TMOD3	sp|Q9NYL	9	9	9	0	8	9	6	0	8	9	6	0	8	9	6	36.6	39.594	0	42.829	598610000	0	1.74E+08	83401000	3.42E+08	0	15982000	53602000	15811000
Q15382	Q15382	GTP-binding protein Rheb	RHEB	sp|Q15382	7	7	7	0	5	1	7	0	5	1	7	0	5	1	7	29.3	20.497	0	13.695	597380000	0	28004000	862920	5.69E+08	0	1615400	616190	24197000
Q99805	Q99805	Transmembrane 9 superfamily member 2	TM9SF2	sp|Q99805	7	7	7	0	7	4	7	0	7	4	7	0	7	4	7	11.2	75.775	0	30.074	596700000	0	1.14E+08	13716000	4.69E+08	0	9961400	9046600	18613000
Q9H4A6	Q9H4A6	Golgi phosphoprotein 3	GOLPH3	sp|Q9H4A	4	4	3	0	4	0	4	0	4	0	4	0	3	0	3	24.2	33.81	0	24.621	595150000	0	94623000	0	5.01E+08	0	9066600	0	19859000
P54105	P54105	Methylosome subunit pICln	CLNS1A	sp|P54105	5	5	5	0	4	0	4	0	4	0	4	0	4	0	4	43.5	26.215	0	49.942	594880000	0	3.83E+08	0	2.12E+08	0	36236000	0	6243400
Q15005	Q15005	Signal peptidase complex subunit 2	SPCS2	sp|Q15005	7	7	7	0	7	0	6	0	7	0	6	0	7	0	6	39.8	25.003	0	48.611	593310000	0	2.28E+08	0	3.66E+08	0	20849000	0	14258000
O00767	O00767	Acyl-CoA desaturase	SCD	sp|O00767	4	4	4	0	2	2	4	0	2	2	4	0	2	2	4	22.6	41.522	0	12.294	593100000	0	28356000	12723000	5.52E+08	0	3844700	15062000	14845000
Q9Y4I1;Q9	Q9Y4I1	Unconventional myosin-Va	MYO5A	sp|Q9Y4I1	19	19	19	2	6	3	19	2	6	3	19	2	6	3	19	13	215.4	0	70.396	592000000	64518000	13509000	6158300	5.08E+08	41359000	8695100	10475000	30202000
O60783	O60783	28S ribosomal protein S14, mitochondrial	MRPS14	sp|O60783	2	2	2	1	2	2	2	1	2	2	2	1	2	2	2	21.9	15.139	0	13.894	591540000	1329600	48630000	497440000	44136000	28828000	21099000	288640000	3142300
Q9NUQ6	Q9NUQ6	SPATS2-like protein	SPATS2L	sp|Q9NUC	11	11	11	0	4	8	11	0	4	8	11	0	4	8	11	26.7	61.728	0	32.547	591420000	0	9482200	97838000	4.84E+08	0	623860	58271000	27597000
Q15165	Q15165	Serum paraoxonase/arylesterase 2	PON2	sp|Q15165	7	7	7	0	1	0	7	0	1	0	7	0	1	0	7	40.1	39.38	0	50.672	590890000	0	10963000	0	5.8E+08	0	997020	0	20881000
P20700	P20700	Lamin-B1	LMNB1	sp|P20700	17	17	16	2	15	3	9	2	15	3	9	2	14	2	8	40.3	66.408	0	80.014	590050000	1719900	4.31E+08	7156800	1.5E+08	968620	43228000	2599600	3901300
Q9Y3Z3	Q9Y3Z3	Deoxynucleoside triphosphate triphosphohydrola	SAMHD1	sp|Q9Y3Z3	16	16	16	1	16	1	9	1	16	1	9	1	16	1	9	35.3	72.2	0	44.122	589020000	2219100	3.54E+08	674520	2.33E+08	0	36271000	903870	4009500
Q14789	Q14789	Golgin subfamily B member 1	GOLGB1	sp|Q14789	22	22	22	1	18	4	16	1	18	4	16	1	18	4	16	11.3	376.01	0	62.755	588940000	8762900	2.16E+08	5023100	3.6E+08	0	16481000	5296800	15386000
P35573	P35573	Glycogen debranching enzyme;4-alpha-glucanotr	AGL	sp|P35573	11	11	11	0	11	0	11	0	11	0	11	0	11	0	11	9.9	174.76	0	49.61	588330000	0	2.26E+08	0	3.63E+08	0	20138000	0	14674000
P09669	P09669	Cytochrome c oxidase subunit 6C	COX6C	sp|P09669	5	5	5	0	5	5	5	0	5	5	5	0	5	5	5	37.3	8.7813	0	11.347	588050000	0	3.19E+08	11541000	2.57E+08	0	27781000	7606400	11092000
P31937	P31937	3-hydroxyisobutyrate dehydrogenase, mitochond	HIBADH	sp|P31937	9	9	9	0	9	0	7	0	9	0	7	0	9	0	7	40.5	35.329	0	68.669	587950000	0	4.19E+08	0	1.69E+08	0	33954000	0	9930300
P36404	P36404	ADP-ribosylation factor-like protein 2	ARL2	sp|P36404	6	6	6	0	6	2	6	0	6	2	6	0	6	2	6	40.2	20.878	0	32.53	587170000	0	2.08E+08	2571300	3.77E+08	0	18724000	2519500	14310000
Q5TFE4	Q5TFE4	5-nucleotidase domain-containing protein 1	NT5DC1	sp|Q5TFE4	6	6	6	0	6	0	5	0	6	0	5	0	6	0	5	22.4	51.844	0	25.005	586900000	0	2.86E+08	0	3.01E+08	0	19083000	0	18511000
P34949	P34949	Mannose-6-phosphate isomerase	MPI	sp|P34949	8	8	8	0	8	0	6	0	8	0	6	0	8	0	6	27.7	46.655	0	55.642	586480000	0	4.97E+08	0	89234000	0	38040000	0	9461600
P55795	P55795	Heterogeneous nuclear ribonucleoprotein H2	HNRNPH2	sp|P55795	13	6	6	4	12	11	12	1	5	4	5	1	5	4	5	36.1	49.263	0	64.383	585410000	44467000	2.39E+08	42745000	2.59E+08	56041000	17897000	20740000	10245000
Q58FF6	Q58FF6	Putative heat shock protein HSP 90-beta 4	HSP90AB4	sp|Q58FF6	7	1	1	3	7	5	7	0	1	0	1	0	1	0	1	10.1	58.264	0.000246	3.6023	583960000	0	2.47E+08	0	3.37E+08	0	21730000	0	13878000
O15498	O15498	Synaptobrevin homolog YKT6	YKT6	sp|O15498	8	8	8	1	6	1	8	1	6	1	8	1	6	1	8	45.5	22.417	0	20.735	583660000	1388700	97296000	1757100	4.83E+08	1117700	9833200	1003200	18027000
Q9Y5K6	Q9Y5K6	CD2-associated protein	CD2AP	sp|Q9Y5K6	8	8	8	0	6	1	8	0	6	1	8	0	6	1	8	20.7	71.45	0	58.82	583460000	0	1.53E+08	1570800	4.29E+08	0	12197000	7955200	12014000
O95336	O95336	6-phosphogluconolactonase	PGLS	sp|O95336	6	6	6	0	3	0	6	0	3	0	6	0	3	0	6	39.1	27.547	0	59.327	581600000	0	20245000	0	5.61E+08	0	3696900	0	21174000
P05121	P05121	Plasminogen activator inhibitor 1	SERPINE1	sp|P05121	5	5	5	0	0	2	5	0	0	2	5	0	0	2	5	21.6	45.059	0	22.366	581490000	0	0	16791000	5.65E+08	0	0	9795500	24711000
Q8N8S7;Q	Q8N8S7	Protein enabled homolog	ENAH	sp|Q8N8S	13	13	13	0	13	0	12	0	13	0	12	0	13	0	12	31.1	66.509	0	60.096	581280000	0	2.68E+08	0	3.13E+08	0	23365000	0	12432000
P82650	P82650	28S ribosomal protein S22, mitochondrial	MRPS22	sp|P82650	13	13	13	3	13	2	6	3	13	2	6	3	13	2	6	37.8	41.28	0	49.011	581160000	6514700	4.66E+08	5228600	1.04E+08	6321700	40004000	3494300	5138600
Q2NL82	Q2NL82	Pre-rRNA-processing protein TSR1 homolog	TSR1	sp|Q2NL82	11	11	11	1	11	11	8	1	11	11	8	1	11	11	8	16.9	91.809	0	27.174	581040000	920240	3.15E+08	36993000	2.28E+08	2460000	31330000	14466000	14676000
Q15428	Q15428	Splicing factor 3A subunit 2	SF3A2	sp|Q15428	4	4	4	1	4	0	4	1	4	0	4	1	4	0	4	14.9	49.255	0	33.774	579850000	2178900	3.11E+08	0	2.67E+08	3986400	27442000	0	9136300
Q9UHB6	Q9UHB6	LIM domain and actin-binding protein 1	LIMA1	sp|Q9UHB	9	9	9	0	6	9	8	0	6	9	8	0	6	9	8	18.6	85.225	0	31.816	579770000	0	1.14E+08	150330000	3.15E+08	0	18165000	80199000	25699000
P22033	P22033	Methylmalonyl-CoA mutase, mitochondrial	MUT	sp|P22033	12	12	12	1	10	2	6	1	10	2	6	1	10	2	6	21.3	83.134	0	30.371	578980000	1.01E+08	2.66E+08	6442400	2.06E+08	0	32406000	848120	2949900
Q16539	Q16539	Mitogen-activated protein kinase 14	MAPK14	sp|Q16539	9	9	9	0	9	0	6	0	9	0	6	0	9	0	6	42.2	41.293	0	67.138	578690000	0	1.51E+08	0	4.28E+08	0	17156000	0	13726000
P11498	P11498	Pyruvate carboxylase, mitochondrial	PC	sp|P11498	11	11	11	0	3	0	11	0	3	0	11	0	3	0	11	13.8	129.63	0	62.269	578430000	0	23603000	0	5.55E+08	0	5441300	0	19457000
Q96N67;Q	Q96N67	Dedicator of cytokinesis protein 7	DOCK7	sp|Q96N6	17	17	16	3	11	11	16	3	11	11	16	3	10	10	15	12.3	242.56	0	62.555	578360000	2113500	1.28E+08	19171000	4.29E+08	4187400	11655000	12824000	15245000
Q3ZCM7	Q3ZCM7	Tubulin beta-8 chain	TUBB8	sp|Q3ZCM	11	2	2	5	10	8	11	0	1	0	2	0	1	0	2	24.5	49.775	0	23.998	578350000	0	2.3E+08	0	3.48E+08	0	21638000	0	12984000
Q8TDN6	Q8TDN6	Ribosome biogenesis protein BRX1 homolog	BRIX1	sp|Q8TDN	10	10	10	1	6	10	10	1	6	10	10	1	6	10	10	36	41.401	0	32.03	577480000	150230	56735000	51415000	4.69E+08	653650	5147500	47311000	5885700
Q13561	Q13561	Dynactin subunit 2	DCTN2	sp|Q13561	8	8	8	1	7	3	7	1	7	3	7	1	7	3	7	31.9	44.23	0	28.004	576820000	3703500	2.45E+08	8891800	3.19E+08	5229900	19147000	11368000	8646800
Q10713	Q10713	Mitochondrial-processing peptidase subunit alph	PMPCA	sp|Q10713	13	13	13	1	13	1	10	1	13	1	10	1	13	1	10	26.5	58.252	0	40.041	576760000	415310	3.5E+08	639750	2.26E+08	0	27266000	0	11256000
Q9NYL2	Q9NYL2	Mitogen-activated protein kinase kinase kinase M	ZAK	sp|Q9NYL	5	5	5	0	3	3	5	0	3	3	5	0	3	3	5	8.8	91.154	0	65.781	576330000	0	17944000	6382500	5.52E+08	0	2505400	4201300	21866000
Q9UH65	Q9UH65	Switch-associated protein 70	SWAP70	sp|Q9UH6	11	11	11	0	4	0	10	0	4	0	10	0	4	0	10	29.4	68.997	0	202.38	575680000	0	30197000	0	5.45E+08	0	2019400	0	23076000
Q8N3U4;Q	Q8N3U4	Cohesin subunit SA-2	STAG2	sp|Q8N3U	8	8	8	1	8	0	3	1	8	0	3	1	8	0	3	9.7	141.32	0	28.371	574540000	3.77E+08	1.57E+08	0	40343000	3.3E+08	26399000	0	35981000
P62995	P62995	Transformer-2 protein homolog beta	TRA2B	sp|P62995	6	6	5	1	6	5	6	1	6	5	6	1	5	5	5	22.9	33.665	0	21.013	573130000	0	2.82E+08	46696000	2.44E+08	0	23394000	33110000	9346300
Q9NRN7	Q9NRN7	L-aminoadipate-semialdehyde dehydrogenase-ph	AASDHPPT	sp|Q9NRN	7	7	7	0	7	0	6	0	7	0	6	0	7	0	6	29.1	35.776	0	30.756	572390000	0	3.64E+08	0	2.08E+08	0	31417000	0	9259600
P11234	P11234	Ras-related protein Ral-B	RALB	sp|P11234	8	8	4	1	6	4	8	1	6	4	8	0	3	2	4	45.6	23.408	0	28.895	572140000	564100	1.3E+08	6129900	4.35E+08	3286400	11830000	5458600	13486000
P25325	P25325	3-mercaptopyruvate sulfurtransferase	MPST	sp|P25325	5	5	5	0	5	0	2	0	5	0	2	0	5	0	2	29.6	33.178	0	54.526	571740000	0	4.7E+08	0	1.01E+08	0	39921000	0	5716200
P35611	P35611	Alpha-adducin	ADD1	sp|P35611	10	10	10	1	9	3	9	1	9	3	9	1	9	3	9	19.8	80.954	0	35.61	571720000	1478500	1.69E+08	6559400	3.94E+08	2996800	17172000	4402500	12466000
Q92888	Q92888	Rho guanine nucleotide exchange factor 1	ARHGEF1	sp|Q92888	16	16	16	0	10	3	15	0	10	3	15	0	10	3	15	22.9	102.43	0	65.385	570410000	0	62218000	2297800	5.06E+08	0	6426400	3503900	17904000
P49458	P49458	Signal recognition particle 9 kDa protein	SRP9	sp|P49458	4	4	4	1	4	3	2	1	4	3	2	1	4	3	2	40.7	10.112	0	15.695	568690000	7906600	3.13E+08	32762000	2.15E+08	11555000	23367000	18659000	12768000
Q6IN85	Q6IN85	Serine/threonine-protein phosphatase 4 regulato	SMEK1	sp|Q6IN85	13	13	10	0	10	1	13	0	10	1	13	0	8	0	10	23.8	95.367	0	57.429	567910000	0	1.32E+08	300120	4.35E+08	0	4508000	577780	24685000
Q9Y6M7	Q9Y6M7	Sodium bicarbonate cotransporter 3	SLC4A7	sp|Q9Y6M	6	6	6	0	2	5	6	0	2	5	6	0	2	5	6	7.4	136.04	0	25.611	566650000	0	37008000	32663000	4.97E+08	0	6046300	14359000	25245000
P17612;P2	P17612;P2	cAMP-dependent protein kinase catalytic subunit	PRKACA;PI	sp|P17612	10	10	10	0	8	1	8	0	8	1	8	0	8	1	8	34.5	40.589	0	24.507	565780000	0	1.78E+08	775690	3.87E+08	0	20643000	1441600	10065000
Q68EM7	Q68EM7	Rho GTPase-activating protein 17	ARHGAP17	sp|Q68EM	11	11	11	0	7	0	10	0	7	0	10	0	7	0	10	26.9	95.436	0	76.817	563800000	0	1.05E+08	0	4.59E+08	0	9953900	0	18158000
O00499;Q	O00499	Myc box-dependent-interacting protein 1	BIN1	sp|O00499	10	10	9	0	2	1	10	0	2	1	10	0	2	1	9	25.3	64.699	0	41.021	563560000	0	30647000	1271800	5.32E+08	0	671980	771840	23976000
O43396	O43396	Thioredoxin-like protein 1	TXNL1	sp|O43396	7	7	7	0	7	1	6	0	7	1	6	0	7	1	6	34.3	32.251	0	58.803	563030000	0	2.14E+08	1552000	3.48E+08	0	24060000	2106900	8027300
Q96M27	Q96M27	Protein PRRC1	PRRC1	sp|Q96M2	6	6	6	0	5	2	5	0	5	2	5	0	5	2	5	15.3	46.701	0	14.743	562590000	0	1.71E+08	2705700	3.89E+08	0	10100000	7701500	15086000
Q9NVS9	Q9NVS9	Pyridoxine-5-phosphate oxidase	PNPO	sp|Q9NVS	7	7	7	0	6	0	7	0	6	0	7	0	6	0	7	34.5	29.988	0	27.816	561290000	0	2.3E+08	0	3.32E+08	0	21279000	0	12602000
Q96IX5	Q96IX5	Up-regulated during skeletal muscle growth prote	USMG5	sp|Q96IX5	3	3	3	0	3	2	3	0	3	2	3	0	3	2	3	44.8	6.4575	0	19.054	560880000	0	2.43E+08	6144600	3.12E+08	0	20002000	5479500	12890000
O43708	O43708	Maleylacetoacetate isomerase	GSTZ1	sp|O43708	3	3	3	0	2	1	3	0	2	1	3	0	2	1	3	21.8	24.212	0	17.328	560620000	0	1.02E+08	3795000	4.55E+08	0	8155300	3285700	18820000
Q15555	Q15555	Microtubule-associated protein RP/EB family mer	MAPRE2	sp|Q15555	8	8	8	0	7	7	8	0	7	7	8	0	7	7	8	37.6	37.031	0	19.362	558680000	0	1.71E+08	18868000	3.69E+08	0	15650000	12649000	14614000
O15160	O15160	DNA-directed RNA polymerases I and III subunit R	POLR1C	sp|O15160	10	10	10	0	10	4	7	0	10	4	7	0	10	4	7	46.8	39.249	0	44.701	558130000	0	3.38E+08	13330000	2.06E+08	0	24529000	15082000	7665000
Q13619	Q13619	Cullin-4A	CUL4A	sp|Q13619	15	8	8	1	8	2	15	1	2	0	8	1	2	0	8	22.3	87.679	0	28.292	556820000	83190	16752000	0	5.4E+08	105480	542610	0	23119000
Q9P032	Q9P032	NADH dehydrogenase [ubiquinone] 1 alpha subcc	NDUFAF4	sp|Q9P032	5	5	5	0	5	3	3	0	5	3	3	0	5	3	3	30.3	20.266	0	14.624	556780000	0	2.41E+08	4721700	3.11E+08	0	21626000	5668100	8016400
Q9BRR6	Q9BRR6	ADP-dependent glucokinase	ADPGK	sp|Q9BRR	5	5	5	0	3	0	5	0	3	0	5	0	3	0	5	18.5	54.088	0	30.038	555550000	0	49066000	0	5.06E+08	0	5609600	0	19545000
Q9NQH7	Q9NQH7	Probable Xaa-Pro aminopeptidase 3	XPNPEP3	sp|Q9NQH	5	5	5	0	5	0	4	0	5	0	4	0	5	0	4	16.4	57.033	0	41.245	555220000	0	2.89E+08	0	2.66E+08	0	26288000	0	10140000
Q93045	Q93045	Stathmin-2	STMN2	sp|Q93045	3	1	1	0	3	2	3	0	1	1	1	0	1	1	1	11.2	20.828	0.000947	3.1239	554940000	0	2.93E+08	253690	2.61E+08	0	25849000	170480	10752000
P61086	P61086	Ubiquitin-conjugating enzyme E2 K	UBE2K	sp|P61086	8	8	8	0	8	0	6	0	8	0	6	0	8	0	6	46.5	22.406	0	63.317	553990000	0	3.88E+08	0	1.66E+08	0	31114000	0	9908500
Q13243	Q13243	Serine/arginine-rich splicing factor 5	SRSF5	sp|Q13243	8	7	7	2	7	7	5	1	6	6	4	1	6	6	4	25.4	31.263	0	36.973	553920000	10272000	3.34E+08	47672000	1.62E+08	18487000	31266000	20650000	7992300
P36873	P36873	Serine/threonine-protein phosphatase PP1-gamn	PPP1CC	sp|P36873	8	2	2	2	8	5	8	0	2	2	2	0	2	2	2	29.7	36.983	0	13.432	553900000	0	2.37E+08	10231000	3.07E+08	0	22212000	7955600	10188000
P11908	P11908	Ribose-phosphate pyrophosphokinase 2	PRPS2	sp|P11908	9	3	3	0	8	4	8	0	3	2	3	0	3	2	3	40.3	34.769	0	27.812	553760000	0	2.63E+08	2900800	2.88E+08	0	22430000	4280600	10260000
Q9UKG1	Q9UKG1	DCC-interacting protein 13-alpha	APPL1	sp|Q9UKG	10	10	10	0	1	9	10	0	1	9	10	0	1	9	10	28.1	79.663	0	30.506	550860000	0	13385000	69104000	4.68E+08	0	2591800	57193000	7094500
P78406	P78406	mRNA export factor	RAE1	sp|P78406	7	7	7	0	5	3	6	0	5	3	6	0	5	3	6	34.5	40.968	0	37.115	550240000	0	1.42E+08	15372000	3.93E+08	0	16787000	4638700	17499000
Q9ULT8	Q9ULT8	E3 ubiquitin-protein ligase HECTD1	HECTD1	sp|Q9ULT8	20	20	20	0	11	2	20	0	11	2	20	0	11	2	20	10.7	289.38	0	58.145	549720000	0	65705000	3105800	4.81E+08	0	4829200	3712000	19116000
Q9BW92	Q9BW92	Threonine--tRNA ligase, mitochondrial	TARS2	sp|Q9BW9	9	9	9	1	8	3	9	1	8	3	9	1	8	3	9	15	81.035	0	27.175	549220000	345170	1.97E+08	5231200	3.47E+08	1012400	24976000	2812200	6687100
Q9HB90;Q	Q9HB90;Q	Ras-related GTP-binding protein C;Ras-related GT	RRAGC;RR	sp|Q9HB9	7	7	7	0	6	0	7	0	6	0	7	0	6	0	7	22.6	44.223	0	28.981	548950000	0	94210000	0	4.55E+08	0	9418900	0	17587000
O14908	O14908	PDZ domain-containing protein GIPC1	GIPC1	sp|O14908	11	11	11	0	8	0	10	0	8	0	10	0	8	0	10	41.7	36.049	0	57.705	545630000	0	1.3E+08	0	4.16E+08	0	12570000	0	15977000
O43237	O43237	Cytoplasmic dynein 1 light intermediate chain 2	DYNC1LI2	sp|O43237	13	13	13	0	7	9	13	0	7	9	13	0	7	9	13	36.4	54.099	0	50.554	544630000	0	37229000	25356000	4.82E+08	0	4923200	17976000	17246000
Q8IXM3	Q8IXM3	39S ribosomal protein L41, mitochondrial	MRPL41	sp|Q8IXM	5	5	5	1	5	4	4	1	5	4	4	1	5	4	4	54.7	15.383	0	21.952	544360000	1386600	2.65E+08	25812000	2.52E+08	3744100	23507000	15268000	9927000
P56545	P56545	C-terminal-binding protein 2	CTBP2	sp|P56545	8	8	7	0	7	1	6	0	7	1	6	0	6	1	5	27.2	48.944	0	50.813	540190000	0	2.03E+08	371870	3.37E+08	0	26636000	688560	4653700
P30260	P30260	Cell division cycle protein 27 homolog	CDC27	sp|P30260	14	14	14	1	9	14	10	1	9	14	10	1	9	14	10	25.5	91.866	0	58.473	539910000	651220	1.37E+08	164050000	2.38E+08	1015700	14334000	109800000	7616000
Q9H8S9;Q	Q9H8S9;Q	MOB kinase activator 1A;MOB kinase activator 1E	MOB1A;M	sp|Q9H8S	4	4	4	0	4	0	4	0	4	0	4	0	4	0	4	19.4	25.079	0	12.331	539610000	0	1.58E+08	0	3.82E+08	0	15722000	0	13879000
P62140	P62140	Serine/threonine-protein phosphatase PP1-beta (	PPP1CB	sp|P62140	10	5	5	2	9	5	8	0	4	3	3	0	4	3	3	44	37.186	0	78.153	539550000	0	1.55E+08	23866000	3.61E+08	0	14062000	16048000	14424000
Q86U42	Q86U42	Polyadenylate-binding protein 2	PABPN1	sp|Q86U4	5	5	5	1	5	4	4	1	5	4	4	1	5	4	4	33.7	32.749	0	28.494	539110000	819410	2.64E+08	36783000	2.38E+08	2332000	31775000	15992000	7304500
Q9H6Z4	Q9H6Z4	Ran-binding protein 3	RANBP3	sp|Q9H6Z	9	9	9	0	9	0	8	0	9	0	8	0	9	0	8	19.4	60.209	0	31.437	538930000	0	2.38E+08	0	3.01E+08	0	21461000	0	11914000
P09543	P09543	2,3-cyclic-nucleotide 3-phosphodiesterase	CNP	sp|P09543	7	7	7	0	5	4	7	0	5	4	7	0	5	4	7	19.5	47.578	0	32.842	538610000	0	1.58E+08	10928000	3.7E+08	0	12413000	10247000	13812000
Q6P587	Q6P587	Acylpyruvase FAHD1, mitochondrial	FAHD1	sp|Q6P587	4	4	4	0	4	0	4	0	4	0	4	0	4	0	4	27.7	24.843	0	33.32	538140000	0	2.15E+08	0	3.24E+08	0	18765000	0	13457000
Q8NBX0	Q8NBX0	Saccharopine dehydrogenase-like oxidoreductase	SCCPDH	sp|Q8NBX	5	5	5	0	5	2	5	0	5	2	5	0	5	2	5	17	47.151	0	23.7	537070000	0	1.03E+08	4042600	4.3E+08	0	7510700	6113500	15869000
Q9HBI1	Q9HBI1	Beta-parvin	PARVB	sp|Q9HBI1	7	5	5	0	2	3	7	0	2	3	5	0	2	3	5	28.3	41.714	0	34.479	535790000	0	12919000	11074000	5.12E+08	0	1982300	10557000	17089000

P46939	P46939	Utrophin	UTRN	sp|P46939	19	19	19	3	13	4	16	3	13	4	16	3	13	4	16	7.9	394.46	0	74.933	535060000	6743500	1.24E+08	5273700	3.99E+08	1665600	3816400	1700200	30450000
Q9GZT8;A(	Q9GZT8	NIF3-like protein 1	NIF3L1	sp|Q9GZT	6	6	6	0	6	0	6	0	6	0	6	0	6	0	6	21.8	41.968	0	25.067	534540000	0	2.29E+08	0	3.05E+08	0	19855000	0	12905000
Q9Y3A5	Q9Y3A5	Ribosome maturation protein SBDS	SBDS	sp|Q9Y3A	9	9	9	1	8	1	7	1	8	1	7	1	8	1	7	34.8	28.763	0	21.072	534290000	4635000	2.23E+08	411430	3.06E+08	3349800	28256000	818130	4747500
Q8NBJ7	Q8NBJ7	Sulfatase-modifying factor 2	SUMF2	sp|Q8NBJ	7	7	7	0	7	1	7	0	7	1	7	0	7	1	7	29.9	33.843	0	23.157	533440000	0	2.32E+08	1395100	3E+08	0	24996000	2054800	6668100
Q53GS9	Q53GS9	U4/U6.U5 tri-snRNP-associated protein 2	USP39	sp|Q53GS	8	8	8	2	7	7	7	2	7	7	7	2	7	7	7	19.5	65.38	0	33.009	533050000	20855000	1.31E+08	28879000	3.52E+08	33406000	11365000	11755000	9775600
Q8N3F8	Q8N3F8	MICAL-like protein 1	MICALL1	sp|Q8N3F	10	10	10	0	6	1	10	0	6	1	10	0	6	1	10	22.7	93.44	0	41.842	532840000	0	49213000	551230	4.83E+08	0	2540300	586610	21449000
Q96F86	Q96F86	Enhancer of mRNA-decapping protein 3	EDC3	sp|Q96F8	9	9	9	0	9	5	8	0	9	5	8	0	9	5	8	23.8	56.077	0	21.753	531400000	0	1.77E+08	8971600	3.45E+08	0	14075000	11147000	10615000
P10301	P10301	Ras-related protein R-Ras	RRAS	sp|P10301	5	5	3	0	1	0	5	0	1	0	5	0	0	0	3	27.1	23.48	0	18.823	531200000	0	12641000	0	5.19E+08	0	2490700	0	19950000
O43670	O43670	BUB3-interacting and GLEBS motif-containing pro	ZNF207	sp|O4367	6	6	6	1	6	3	6	1	6	3	6	1	6	3	6	15.5	50.75	0	32.111	531130000	2613600	2.14E+08	10916000	3.04E+08	3919900	20978000	6477700	9905200
Q2M389	Q2M389	WASH complex subunit 7	KIAA1033	sp|Q2M38	13	13	13	0	4	0	13	0	4	0	13	0	4	0	13	17.3	136.4	0	79.186	530400000	0	47552000	0	4.83E+08	0	770060	0	23279000
Q8NBF2	Q8NBF2	NHL repeat-containing protein 2	NHLRC2	sp|Q8NBF	9	9	9	1	9	0	8	1	9	0	8	1	9	0	8	21.5	79.443	0	41.444	530190000	5116900	2.4E+08	0	2.85E+08	6811500	21178000	0	9280000
Q92797	Q92797	Symplekin	SYMPK	sp|Q9279	12	12	12	0	11	3	10	0	11	3	10	0	11	3	10	11.8	141.15	0	40.337	529540000	0	2.3E+08	3074800	2.96E+08	0	25434000	1953300	7153700
Q8N5K1	Q8N5K1	CDGSH iron-sulfur domain-containing protein 2	CISD2	sp|Q8N5K	5	5	5	0	4	5	5	0	4	5	5	0	4	5	5	31.9	15.278	0	13.621	529050000	0	2.14E+08	10646000	3.05E+08	0	18905000	7799200	11811000
Q9Y3A6	Q9Y3A6	Transmembrane emp24 domain-containing prote	TMED5	sp|Q9Y3A	5	5	5	1	5	0	4	1	5	0	4	1	5	0	4	27.1	26.005	0	34.326	528590000	7081100	2.68E+08	0	2.54E+08	7058200	25718000	0	8336400
Q8NCA5	Q8NCA5	Protein FAM98A	FAM98A	sp|Q8NCA	7	4	4	2	5	6	6	2	3	4	4	2	3	4	4	18.1	55.272	0	33.493	527470000	70211000	1.09E+08	60871000	2.87E+08	9857400	12314000	96576000	13803000
Q9UQB8	Q9UQB8	Brain-specific angiogenesis inhibitor 1-associated	BAIAP2	sp|Q9UQB	12	12	12	0	8	8	12	0	8	8	12	0	8	8	12	29.3	60.867	0	26.379	526930000	0	98789000	24098000	4.04E+08	0	9087900	20330000	12101000
Q6NUK1	Q6NUK1	Calcium-binding mitochondrial carrier protein SCa	SLC25A24	sp|Q6NUK	12	12	12	0	10	3	9	0	10	3	9	0	10	3	9	31.4	53.354	0	45.204	525930000	0	2.42E+08	3439600	2.8E+08	0	18977000	5422700	10781000
P21266	P21266	Glutathione S-transferase Mu 3	GSTM3	sp|P21266	10	10	9	3	9	0	1	3	9	0	1	2	8	0	0	47.1	26.559	0	44.586	524820000	25065000	4.83E+08	0	16411000	13285000	53746000	0	1314300
P22087;A6	P22087	rRNA 2-O-methyltransferase fibrillarin	FBL	sp|P22087	9	9	9	0	6	8	8	0	6	8	8	0	6	8	8	43	33.784	0	47.734	524280000	0	31074000	81115000	4.12E+08	0	5402400	50422000	17627000
Q9NQT5	Q9NQT5	Exosome complex component RRP40	EXOSC3	sp|Q9NQT	5	5	5	0	2	4	5	0	2	4	5	0	2	4	5	39.6	29.572	0	45.261	523530000	0	1.56E+08	33044000	3.34E+08	0	15279000	25851000	8597900
P41208;Q1	P41208	Centrin-2	CETN2	sp|P41208	5	5	5	0	4	3	4	0	4	3	4	0	4	3	4	48.8	19.738	0	103.14	523270000	0	2.17E+08	18615000	2.88E+08	0	17580000	11176000	14698000
P53701	P53701	Cytochrome c-type heme lyase	HCCS	sp|P53701	10	10	10	1	10	0	8	1	10	0	8	1	10	0	8	52.6	30.601	0	42.898	523010000	6028900	3.79E+08	0	1.38E+08	12819000	29602000	0	2678100
Q9H2M9	Q9H2M9	Rab3 GTPase-activating protein non-catalytic sub	RAB3GAP2	sp|Q9H2M	14	14	14	0	10	2	14	0	10	2	14	0	10	2	14	13.6	155.98	0	54.619	522760000	0	89551000	866060	4.32E+08	0	9008400	698980	16549000
O95400	O95400	CD2 antigen cytoplasmic tail-binding protein 2	CD2BP2	sp|O9540	7	7	7	1	7	6	4	1	7	6	4	1	7	6	4	26.4	37.646	0	26.951	522400000	1207400	3.07E+08	45489000	1.69E+08	4944000	20263000	33272000	7284700
Q86X55	Q86X55	Histone-arginine methyltransferase CARM1	CARM1	sp|Q86X5	7	7	7	0	7	0	5	0	7	0	5	0	7	0	5	19.4	65.853	0	32.367	522240000	0	2.15E+08	0	3.07E+08	0	14802000	0	13973000
Q9Y4P3	Q9Y4P3	Transducin beta-like protein 2	TBL2	sp|Q9Y4P	7	7	7	3	5	5	7	3	5	5	7	3	5	5	7	23.5	49.797	0	30.046	521540000	9301800	75014000	36234000	4.01E+08	17093000	4884300	22724000	12053000
P13995	P13995	Bifunctional methylenetetrahydrofolate dehydrog	MTHFD2	sp|P13995	7	7	7	0	6	3	6	0	6	3	6	0	6	3	6	29.1	37.895	0	18.984	519920000	0	3.51E+08	1559100	1.67E+08	0	35466000	672810	2737700
P50995;P2	P50995	Annexin A11	ANXA11	sp|P50995	7	7	7	1	5	2	7	1	5	2	7	1	5	2	7	13.1	54.389	0	18.646	519530000	217780	1.61E+08	1595100	3.57E+08	1926900	6289300	2156700	19777000
Q8WUX9	Q8WUX9	Charged multivesicular body protein 7	CHMP7	sp|Q8WU	6	6	6	1	3	0	6	1	3	0	6	1	3	0	6	21.2	50.91	0	35.6	518150000	1.53E+08	29285000	0	3.36E+08	1.22E+08	31000000	0	16087000
Q16630	Q16630	Cleavage and polyadenylation specificity factor su	CPSF6	sp|Q1663	7	7	7	2	7	5	6	2	7	5	6	2	7	5	6	13.8	59.209	0	32.408	518040000	4058800	2.92E+08	12707000	2.09E+08	5311400	22060000	8747400	10831000
P30533	P30533	Alpha-2-macroglobulin receptor-associated prote	LRPAP1	sp|P30533	11	11	11	1	10	8	9	1	10	8	9	1	10	8	9	38.1	41.465	0	32.604	517300000	450210	2.04E+08	15726000	2.97E+08	2271800	8730400	11117000	19093000
P41240;P4	P41240	Tyrosine-protein kinase CSK	CSK	sp|P41240	11	11	11	1	11	0	10	1	11	0	10	1	11	0	10	28	50.704	0	28.659	516070000	88621	2.42E+08	0	2.74E+08	365610	28526000	0	3800000
A0A0B4J1Y	A0A0B4J1Y9;A0A0B4J1V6;A0A0B4J1V0		IGHV3-72;I	sp|A0A0B	1	1	1	1	0	1	0	1	0	1	0	1	0	1	0	5.9	13.203	0.004543	1.8751	515680000	7648500	0	508030000	0	7648500	0	340830000	0
Q9BRX2	Q9BRX2	Protein pelota homolog	PELO	sp|Q9BRX	8	8	8	1	8	3	8	1	8	3	8	1	8	3	8	27.3	43.359	0	97.745	514190000	901090	1.24E+08	13444000	3.75E+08	1453300	11338000	10235000	13317000
Q9BPX5	Q9BPX5	Actin-related protein 2/3 complex subunit 5-like p	ARPC5L	sp|Q9BPX	5	3	3	0	4	1	4	0	2	1	3	0	2	1	3	49	16.941	0	73.525	512640000	0	98834000	2028700	4.12E+08	0	6971700	4537200	15501000
Q9GZQ8;A	Q9GZQ8;A	Microtubule-associated proteins 1A/1B light chair	MAP1LC3B	sp|Q9GZQ	2	2	2	0	2	2	2	0	2	2	2	0	2	2	2	16.8	14.688	0	5.2283	512260000	0	40791000	5862700	4.66E+08	0	3943500	3872000	18868000
O94906	O94906	Pre-mRNA-processing factor 6	PRPF6	sp|O9490	18	18	18	4	14	14	14	4	14	14	14	4	14	14	14	20	106.92	0	70.457	512140000	3311600	1.58E+08	41014000	3.1E+08	4141200	13654000	26065000	12450000
Q00765	Q00765	Receptor expression-enhancing protein 5	REEP5	sp|Q0076	5	5	5	0	5	2	4	0	5	2	4	0	5	2	4	21.7	21.493	0	11.458	510290000	0	2.07E+08	1748000	3.01E+08	0	16249000	1493900	14097000
Q9Y376;Q	Q9Y376	Calcium-binding protein 39	CAB39	sp|Q9Y37	9	9	9	0	8	0	9	0	8	0	9	0	8	0	9	24.3	39.869	0	26.875	510150000	0	2.48E+08	0	2.62E+08	0	18209000	0	14425000
Q9H9A6	Q9H9A6	Leucine-rich repeat-containing protein 40	LRRC40	sp|Q9H9A	12	12	12	0	10	0	11	0	10	0	11	0	10	0	11	25.6	68.249	0	36.395	508930000	0	2.39E+08	0	2.7E+08	0	19821000	0	11898000
Q9C0B1	Q9C0B1	Alpha-ketoglutarate-dependent dioxygenase FTO	FTO	sp|Q9C0B	8	8	8	0	8	0	8	0	8	0	8	0	8	0	8	22	58.281	0	31.305	508920000	0	1.74E+08	0	3.35E+08	0	13958000	0	14745000
Q7L0Y3	Q7L0Y3	Mitochondrial ribonuclease P protein 1	TRMT10C	sp|Q7L0Y3	8	8	8	1	7	3	8	1	7	3	8	1	7	3	8	31.5	47.346	0	44.675	508700000	0	1.23E+08	7875800	3.78E+08	0	10021000	8092900	13570000
Q13085;O(	Q13085	Acetyl-CoA carboxylase 1;Biotin carboxylase	ACACA	sp|Q1308	16	16	16	0	14	2	11	0	14	2	11	0	14	2	11	9.6	265.55	0	59.448	508400000	0	2.41E+08	5049900	2.62E+08	0	13462000	1414900	20551000
Q01970	Q01970	1-phosphatidylinositol 4,5-bisphosphate phospho	PLCB3	sp|Q0197	10	10	10	0	4	6	10	0	4	6	10	0	4	6	10	13.5	138.8	0	39.811	507450000	0	12829000	11760000	4.83E+08	0	3521200	12120000	12490000
Q9NWV4	Q9NWV4	UPF0587 protein C1orf123	C1orf123	sp|Q9NW	4	4	4	0	4	1	3	0	4	1	3	0	4	1	3	40	18.048	0	31.42	507210000	0	2.12E+08	956770	2.95E+08	0	17587000	1481300	12351000
O75531	O75531	Barrier-to-autointegration factor;Barrier-to-autoi	BANF1	sp|O75531	2	2	2	1	1	1	1	1	1	1	1	1	1	1	1	40.4	10.058	0	4.5017	506540000	1.64E+08	1.15E+08	6915400	2.2E+08	0	10108000	4216200	9537800
P16930	P16930	Fumarylacetoacetase	FAH	sp|P16930	9	9	9	0	4	0	9	0	4	0	9	0	4	0	9	30.1	46.374	0	30.934	506400000	0	73779000	0	4.33E+08	0	8271000	0	16024000
Q16637	Q16637	Survival motor neuron protein	SMN1	sp|Q1663	6	6	6	1	6	2	4	1	6	2	4	1	6	2	4	23.5	31.848	0	19.561	505760000	2695300	3.29E+08	7653400	1.66E+08	4151600	23394000	9758000	6380400
Q641Q2;Q	Q641Q2;Q	WASH complex subunit FAM21A;WASH complex	FAM21A;F.	sp|Q641Q	13	13	13	0	8	0	13	0	8	0	13	0	8	0	13	17.2	147.18	0	131.47	505730000	0	92072000	0	4.14E+08	0	10950000	0	14178000
O75607	O75607	Nucleoplasmin-3	NPM3	sp|O7560	3	3	3	1	3	2	3	1	3	2	3	1	3	2	3	29.2	19.343	0	75.564	505630000	1242300	3E+08	9430000	1.95E+08	5546700	23414000	6669600	6399900
Q86VS8	Q86VS8	Protein Hook homolog 3	HOOK3	sp|Q86VS	10	10	10	0	6	1	9	0	6	1	9	0	6	1	9	22.4	83.125	0	52.58	505230000	0	1.18E+08	1598900	3.85E+08	0	7269400	1994200	18087000
Q14318	Q14318	Peptidyl-prolyl cis-trans isomerase FKBP8	FKBP8	sp|Q1431	6	6	6	0	6	1	5	0	6	1	5	0	6	1	5	18.7	44.561	0	20.389	504510000	0	1.41E+08	4024600	3.6E+08	0	9889800	3809100	16209000
O14654	O14654	Insulin receptor substrate 4	IRS4	sp|O1465	19	19	19	9	19	1	1	9	19	1	1	9	19	1	1	24.1	133.77	0	80.738	504230000	34838000	4.59E+08	975590	9007600	29277000	44491000	684540	467860
P62873;P1	P62873	Guanine nucleotide-binding protein G(I)/G(S)/G(T	GNB1	sp|P62873	7	1	1	1	6	4	7	0	1	0	1	0	1	0	1	25.3	37.377	0	33.041	503770000	0	87513000	0	4.16E+08	0	7714100	0	17119000
Q92620	Q92620	Pre-mRNA-splicing factor ATP-dependent RNA he	DHX38	sp|Q9262	13	13	12	0	11	1	12	0	11	1	12	0	10	1	11	13.5	140.5	0	40.894	502660000	0	1.09E+08	6803900	3.87E+08	0	8492000	5453500	16135000
Q8N183	Q8N183	Mimitin, mitochondrial	NDUFAF2	sp|Q8N18	7	7	7	0	7	1	6	0	7	1	6	0	7	1	6	56.8	19.856	0	35.667	502500000	0	2.91E+08	1265800	2.11E+08	0	28745000	1409900	4976500
Q9Y6H1;Q	Q9Y6H1;Q	Coiled-coil-helix-coiled-coil-helix domain-containi	CHCHD2;C	sp|Q9Y6H	3	3	3	2	3	3	3	2	3	3	3	2	3	3	3	27.2	15.512	0	27.25	501520000	37147000	2.74E+08	31453000	1.59E+08	24539000	21859000	34720000	7831100
Q9UIA9	Q9UIA9	Exportin-7	XPO7	sp|Q9UIA	14	14	13	0	11	2	11	0	11	2	11	0	10	2	10	16.8	123.91	0	41.319	500690000	0	1.82E+08	2596100	3.16E+08	0	23678000	885580	6240700
Q969X5	Q969X5	Endoplasmic reticulum-Golgi intermediate compa	ERGIC1	sp|Q969X	8	8	8	0	6	4	8	0	6	4	8	0	6	4	8	39	32.592	0	49.004	500180000	0	40472000	7767400	4.52E+08	0	6018300	7521100	13834000
Q92878	Q92878	DNA repair protein RAD50	RAD50	sp|Q9287	10	10	10	0	8	1	9	0	8	1	9	0	8	1	9	9.6	153.89	0	43.541	498830000	0	2.27E+08	1661100	2.7E+08	0	19708000	0	11400000
Q1KMD3	Q1KMD3	Heterogeneous nuclear ribonucleoprotein U-like	HNRNPUL2	sp|Q1KMD	9	9	9	1	4	5	9	1	4	5	9	1	4	5	9	16.7	85.104	0	39.992	498660000	1162400	64034000	24394000	4.09E+08	4144000	7434600	14955000	13489000
P40145	P40145	Adenylate cyclase type 8	ADCY8	sp|P40145	20	20	20	0	0	20	2	0	0	20	2	0	0	20	2	20.5	140.12	0	58.668	497780000	0	0	489230000	8554900	0	0	308440000	351830
O95456	O95456	Proteasome assembly chaperone 1	PSMG1	sp|O9545	5	5	5	0	5	1	5	0	5	1	5	0	5	1	5	18.8	32.854	0	18.963	497360000	0	1.47E+08	1229100	3.49E+08	0	10605000	1498600	16025000
Q7Z478	Q7Z478	ATP-dependent RNA helicase DHX29	DHX29	sp|Q7Z47	15	15	15	2	9	9	14	2	9	9	14	2	9	9	14	16.7	155.23	0	46.621	496930000	3519500	97704000	24738000	3.71E+08	9270200	7533000	16707000	10502000
P51553	P51553	Isocitrate dehydrogenase [NAD] subunit gamma,	IDH3G	sp|P51553	7	7	7	0	5	0	6	0	5	0	6	0	5	0	6	25.2	42.794	0	68.76	496490000	0	1.74E+08	0	3.23E+08	0	17007000	0	9759600
P53990	P53990	IST1 homolog	IST1	sp|P53990	8	8	8	0	8	2	6	0	8	2	6	0	8	2	6	40.7	39.75	0	33.698	495620000	0	2.64E+08	2657300	2.29E+08	0	21501000	5143400	7830400
Q5JVF3	Q5JVF3	PCI domain-containing protein 2	PCID2	sp|Q5JVF3	7	7	7	0	4	4	7	0	4	4	7	0	4	4	7	23.8	46.029	0	41.019	495190000	0	46085000	6930500	4.42E+08	0	6856000	8140600	11908000
Q9NZ32	Q9NZ32	Actin-related protein 10	ACTR10	sp|Q9NZ3	7	7	7	0	6	1	7	0	6	1	7	0	6	1	7	25.7	46.306	0	53.796	495030000	0	1.73E+08	2794700	3.19E+08	0	10882000	3499400	15876000
P28288	P28288	ATP-binding cassette sub-family D member 3	ABCD3	sp|P28288	7	7	7	0	7	3	4	0	7	3	4	0	7	3	4	13.7	75.475	0	29.034	494610000	0	3.4E+08	7709000	1.47E+08	0	29326000	5843600	6000100
Q9UHG3	Q9UHG3	Prenylcysteine oxidase 1	PCYOX1	sp|Q9UHG	9	9	9	1	6	2	9	1	6	2	9	1	6	2	9	28.1	56.639	0	31.928	492910000	10505000	71185000	3301500	4.08E+08	7961900	7502300	6657900	13652000
P32322	P32322	Pyrroline-5-carboxylate reductase 1, mitochondri	PYCR1	sp|P32322	9	9	9	1	8	1	6	1	8	1	6	1	8	1	6	38.6	33.36	0	28.8	492320000	1763500	4.04E+08	178100	86624000	608400	39366000	158490	902810
Q92804	Q92804	TATA-binding protein-associated factor 2N	TAF15	sp|Q9280	6	4	4	3	4	5	5	2	2	4	3	2	2	4	3	15.5	61.829	0	26.189	491530000	24127000	1.11E+08	145400000	2.11E+08	71378000	7930200	54755000	6244300
Q9Y305	Q9Y305	Acyl-coenzyme A thioesterase 9, mitochondrial	ACOT9	sp|Q9Y30	11	11	10	1	6	3	9	1	6	3	9	1	6	3	9	32.1	49.901	0	37.701	491360000	336560	73051000	19279000	3.99E+08	699980	10792000	7152800	17483000
Q9H2D6	Q9H2D6	TRIO and F-actin-binding protein	TRIOBP	sp|Q9H2D	6	6	6	1	4	5	5	1	4	5	5	1	4	5	5	3.4	261.37	0	25.698	490920000	2360700	1.42E+08	14901000	3.31E+08	8609500	6490500	11140000	10943000
Q9Y3B7	Q9Y3B7	39S ribosomal protein L11, mitochondrial	MRPL11	sp|Q9Y3B	7	7	7	0	5	6	6	0	5	6	6	0	5	6	6	40.1	20.683	0	25.247	489980000	0	1.79E+08	34807000	2.76E+08	0	17299000	22357000	10880000
P01859	P01859	Ig gamma-2 chain C region	IGHG2	sp|P01859	4	3	3	4	0	4	0	3	0	3	0	3	0	3	0	14.7	35.9	0	14.501	489920000	4.6E+08	0	30043000	0	3.21E+08	0	56376000	0
Q13573	Q13573	SNW domain-containing protein 1	SNW1	sp|Q1357	9	9	9	1	8	8	6	1	8	8	6	1	8	8	6	29.5	61.494	0	48.319	489040000	3341200	2.41E+08	66581000	1.78E+08	9103800	21215000	39747000	6595600
Q8N983	Q8N983	39S ribosomal protein L43, mitochondrial	MRPL43	sp|Q8N98	5	5	5	1	4	1	5	1	4	1	5	1	4	1	5	24.2	23.431	0	11.72	487470000	340160	1.3E+08	1520800	3.56E+08	1762200	15608000	4049000	5870800
Q6P1M0	Q6P1M0	Long-chain fatty acid transport protein 4	SLC27A4	sp|Q6P1M	9	9	9	0	4	2	9	0	4	2	9	0	4	2	9	19.1	72.063	0	31.716	486420000	0	1.01E+08	6719600	3.79E+08	0	9144000	7965100	11888000
Q96Q11	Q96Q11	CCA tRNA nucleotidyltransferase 1, mitochondrial	TRNT1	sp|Q96Q1	8	8	8	0	7	0	8	0	7	0	8	0	7	0	8	22.6	50.127	0	29.114	486290000	0	84803000	0	4.01E+08	0	8937600	0	15049000
Q9NR12	Q9NR12	PDZ and LIM domain protein 7	PDLIM7	sp|Q9NR1	8	8	8	1	2	6	8	1	2	6	8	1	2	6	8	31.3	49.844	0	37.864	485970000	4002300	18427000	39798000	4.24E+08	0	3229600	23482000	18488000
Q16204	Q16204	Coiled-coil domain-containing protein 6	CCDC6	sp|Q1620	11	11	11	0	10	0	10	0	10	0	10	0	10	0	10	34	53.29	0	38.741	485600000	0	2.41E+08	0	2.45E+08	0	21114000	0	10171000
P14678;P6	P14678;P6	Small nuclear ribonucleoprotein-associated prote	SNRPB;SNI	sp|P14678	5	5	5	2	4	4	4	2	4	4	4	2	4	4	4	18.8	24.61	0	21.26	485590000	22896000	2.82E+08	13546000	1.67E+08	24800000	18641000	10165000	10116000
Q9Y4C2	Q9Y4C2	TRPM8 channel-associated factor 1	TCAF1	sp|Q9Y4C	11	11	11	0	7	5	11	0	7	5	11	0	7	5	11	15.6	102.12	0	48.472	485220000	0	19858000	11659000	4.54E+08	0	3176800	9803500	15263000
P46108	P46108	Adapter molecule crk	CRK	sp|P46108	7	7	7	0	6	1	7	0	6	1	7	0	6	1	7	36.2	33.83	0	23.126	484610000	0	1.44E+08	1263000	3.39E+08	0	10026000	4813500	12664000
P53367	P53367	Arfaptin-1	ARFIP1	sp|P53367	8	8	8	1	6	1	8	1	6	1	8	1	6	1	8	33	41.738	0	33.934	484560000	1155700	1.02E+08	4350900	3.77E+08	4229400	6660700	5568800	11910000
Q13637	Q13637	Ras-related protein Rab-32	RAB32	sp|Q1363	7	7	6	0	0	1	7	0	0	1	7	0	0	1	6	45.8	24.997	0	29.095	483930000	0	0	1042400	4.83E+08	0	0	700520	17916000
Q15036	Q15036	Sorting nexin-17	SNX17	sp|Q1503	8	8	8	0	3	1	8	0	3	1	8	0	3	1	8	25.5	52.901	0	85.76	483420000	0	43557000	1654400	4.38E+08	0	5756200	0	16105000
P40937	P40937	Replication factor C subunit 5	RFC5	sp|P40937	7	7	7	1	4	4	6	1	4	4	6	1	4	4	6	31.5	38.496	0	31.442	483420000	8840200	1.13E+08	17284000	3.44E+08	18456000	6725500	13846000	5549200

O75116	O75116	Rho-associated protein kinase 2	ROCK2	sp|O7511	17	17	15	0	14	1	15	0	14	1	15	0	12	1	13	18.7	160.9	0	60.949	482810000	0	1.41E+08	1294100	3.4E+08	0	6345200	1938600	19042000
Q8N3D4	Q8N3D4	EH domain-binding protein 1-like protein 1	EHBP1L1	sp|Q8N3D	16	16	16	0	1	4	16	0	1	4	16	0	1	4	16	21	161.85	0	84.275	481250000	0	8503800	28940000	4.44E+08	0	1996100	10859000	25594000
Q8IY81	Q8IY81	pre-rRNA processing protein FTSJ3	FTSJ3	sp|Q8IY81	13	13	13	1	9	10	8	1	9	10	8	1	9	10	8	24.1	96.557	0	44.456	479070000	578120	1.71E+08	101570000	2.06E+08	1245900	8385400	76062000	6662900
Q9H1A4	Q9H1A4	Anaphase-promoting complex subunit 1	ANAPC1	sp|Q9H1A	17	17	17	0	9	17	11	0	9	17	11	0	9	17	11	11.9	216.5	0	50.314	477610000	0	64269000	153340000	2.6E+08	0	9851000	97853000	11697000
Q9UI26	Q9UI26	Importin-11	IPO11	sp|Q9UI26	10	10	10	0	10	0	9	0	10	0	9	0	10	0	9	12.5	112.53	0	37.099	477470000	0	1.14E+08	0	3.63E+08	0	10099000	0	14899000
Q9UJA5	Q9UJA5	tRNA (adenine(58)-N(1))-methyltransferase non-c	TRMT6	sp|Q9UJA	11	11	11	1	7	4	11	1	7	4	11	1	7	4	11	36.8	55.799	0	39.926	476320000	3251200	1.19E+08	6112300	3.48E+08	8959400	9265500	5930800	8018900
Q09161	Q09161	Nuclear cap-binding protein subunit 1	NCBP1	sp|Q09161	8	8	8	1	8	2	7	1	8	2	7	1	8	2	7	14.1	91.838	0	20.562	476290000	27993000	2.05E+08	16196000	2.27E+08	34401000	18372000	3495400	10002000
Q16822	Q16822	Phosphoenolpyruvate carboxykinase [GTP], mitoc	PCK2	sp|Q16822	7	7	7	0	7	0	6	0	7	0	6	0	7	0	6	14.1	70.698	0	31.281	476160000	0	2.79E+08	0	1.97E+08	0	24583000	0	8119400
P31942	P31942	Heterogeneous nuclear ribonucleoprotein H3	HNRNPH3	sp|P31942	9	9	9	0	8	7	9	0	8	7	9	0	8	7	9	35.8	36.926	0	59.899	475640000	0	2.02E+08	47148000	2.26E+08	0	21438000	25342000	12041000
Q8N6T3	Q8N6T3	ADP-ribosylation factor GTPase-activating protein	ARFGAP1	sp|Q8N6T	8	8	8	0	5	0	7	0	5	0	7	0	5	0	7	29.6	44.667	0	55.371	474780000	0	64218000	0	4.11E+08	0	6864600	0	15681000
Q969V3	Q969V3	Nicalin	NCLN	sp|Q969V	12	12	12	0	10	2	10	0	10	2	10	0	10	2	10	31.3	62.974	0	43.116	474270000	0	1.07E+08	4816600	3.63E+08	0	8032900	5257800	14271000
Q9H7D7	Q9H7D7	WD repeat-containing protein 26	WDR26	sp|Q9H7D	11	11	11	0	10	3	10	0	10	3	10	0	10	3	10	19.7	72.123	0	25.102	472650000	0	2.05E+08	7391800	2.61E+08	0	14911000	4688300	14126000
Q9Y673	Q9Y673	Dolichyl-phosphate beta-glucosyltransferase	ALG5	sp|Q9Y673	4	4	4	0	4	1	2	0	4	1	2	0	4	1	2	18.8	36.946	0	17.158	472590000	0	1.3E+08	1791400	3.41E+08	0	6575500	0	18907000
Q9H9J2	Q9H9J2	39S ribosomal protein L44, mitochondrial	MRPL44	sp|Q9H9J2	8	8	8	0	8	4	7	0	8	4	7	0	8	4	7	32.8	37.535	0	23.321	472510000	0	1.88E+08	8855700	2.75E+08	0	13623000	8614500	11635000
P30622	P30622	CAP-Gly domain-containing linker protein 1	CLIP1	sp|P30622	18	18	16	0	10	3	17	0	10	3	17	0	9	2	15	16	162.24	0	69.576	470880000	0	54690000	3582200	4.13E+08	0	3590400	5017600	15589000
P49756	P49756	RNA-binding protein 25	RBM25	sp|P49756	8	8	8	3	8	5	8	3	8	5	8	3	8	5	8	13.2	100.18	0	23.17	470160000	24303000	1.25E+08	16721000	3.05E+08	28564000	10309000	13828000	6346300
Q9UII2	Q9UII2	ATPase inhibitor, mitochondrial	ATPIF1	sp|Q9UII2	2	2	2	0	1	2	2	0	1	2	2	0	1	2	2	17	12.249	0	4.4932	469840000	0	2.45E+08	5960700	2.19E+08	0	26760000	2374100	5455500
P06703	P06703	Protein S100-A6	S100A6	sp|P06703	1	1	1	0	1	1	1	0	1	1	1	0	1	1	1	8.9	10.18	0.001163	2.8485	469620000	0	7617100	3192000	4.59E+08	0	601410	1074900	20009000
Q4V328	Q4V328	GRIP1-associated protein 1	GRIPAP1	sp|Q4V328	12	12	12	0	12	0	7	0	12	0	7	0	12	0	7	25.1	96.004	0	27.276	469100000	0	2.74E+08	0	1.95E+08	0	21329000	0	10841000
Q9BYD1	Q9BYD1	39S ribosomal protein L13, mitochondrial	MRPL13	sp|Q9BYD	5	5	5	0	5	1	4	0	5	1	4	0	5	1	4	35.4	20.692	0	23.639	468660000	0	1.85E+08	1707500	2.82E+08	0	12305000	3994100	12765000
O95983	O95983	Methyl-CpG-binding domain protein 3	MBD3	sp|O95983	7	7	7	0	7	0	7	0	7	0	7	0	7	0	7	26.8	32.844	0	26.713	467690000	0	2.36E+08	0	2.32E+08	0	23251000	0	7085600
P35221	P35221	Catenin alpha-1	CTNNA1	sp|P35221	11	11	8	0	10	6	8	0	10	6	8	0	7	4	5	21.4	100.07	0	139.96	467260000	0	1.43E+08	10075000	3.14E+08	0	10317000	7576900	14404000
Q9NXG2	Q9NXG2	THUMP domain-containing protein 1	THUMPD1	sp|Q9NXG	7	7	7	0	6	1	6	0	6	1	6	0	6	1	6	29.2	39.315	0	24.522	466900000	0	1.79E+08	2145600	2.86E+08	0	13133000	3046100	12792000
Q9NPD3	Q9NPD3	Exosome complex component RRP41	EXOSC4	sp|Q9NPD	6	6	6	1	6	4	4	1	6	4	4	1	6	4	4	29.8	26.383	0	27.328	466830000	704700	2.37E+08	31624000	1.98E+08	0	16116000	22589000	11537000
Q7Z4H8	Q7Z4H8	KDEL motif-containing protein 2	KDELC2	sp|Q7Z4H	3	3	3	0	3	0	3	0	3	0	3	0	3	0	3	7.5	58.572	0	16.706	465650000	0	83346000	0	3.82E+08	0	8871200	0	14198000
O15269	O15269	Serine palmitoyltransferase 1	SPTLC1	sp|O1526	7	7	7	0	3	4	5	0	3	4	5	0	3	4	5	20.1	52.743	0	17.161	465650000	0	1.12E+08	18109000	3.36E+08	0	9538900	12754000	13527000
Q9P0I2	Q9P0I2	ER membrane protein complex subunit 3	EMC3	sp|Q9P0I2	5	5	5	1	5	0	5	1	5	0	5	1	5	0	5	25.7	29.952	0	57.95	465210000	553340	1.15E+08	0	3.49E+08	1112100	4558400	0	19409000
P50281	P50281	Matrix metalloproteinase-14	MMP14	sp|P50281	4	4	4	0	0	4	4	0	0	4	4	0	0	4	4	10.1	65.893	0	16.931	465020000	0	0	19564000	4.45E+08	0	0	11955000	19512000
Q96A26	Q96A26	Protein FAM162A	FAM162A	sp|Q96A2	5	5	5	1	5	3	3	1	5	3	3	1	5	3	3	35.1	17.342	0	72.977	464820000	1176000	3.03E+08	3607900	1.58E+08	1403000	24940000	3073000	7328000
O15173	O15173	Membrane-associated progesterone receptor cor	PGRMC2	sp|O1517	6	6	6	1	6	0	4	1	6	0	4	1	6	0	4	30.5	23.818	0	31.744	464440000	1144100	3.04E+08	0	1.6E+08	1914600	27133000	0	4974000
Q96DH6	Q96DH6	RNA-binding protein Musashi homolog 2	MSI2	sp|Q96DH	6	6	6	0	6	1	3	0	6	1	3	0	6	1	3	25.6	35.196	0	19.138	463510000	0	3.32E+08	723080	1.31E+08	0	30828000	893070	3388100
Q9NUQ8	Q9NUQ8	ATP-binding cassette sub-family F member 3	ABCF3	sp|Q9NUC	8	8	8	0	5	2	7	0	5	2	7	0	5	2	7	15.9	79.744	0	26.174	463290000	0	57675000	1570500	4.04E+08	0	6426100	0	15275000
Q14738	Q14738	Serine/threonine-protein phosphatase 2A 56 kDa	PPP2R5D	sp|Q1473	8	8	6	0	7	1	8	0	7	1	8	0	5	0	6	20.8	69.991	0	21.563	462860000	0	1.12E+08	5264400	3.46E+08	0	9512600	5060200	13037000
P41214	P41214	Eukaryotic translation initiation factor 2D	EIF2D	sp|P41214	12	12	12	0	12	2	9	0	12	2	9	0	12	2	9	32	64.706	0	30.484	462520000	0	2.27E+08	1123100	2.34E+08	0	18257000	690890	11462000
O60264;P2	O60264	SWI/SNF-related matrix-associated actin-depend	SMARCA5	sp|O6026	17	17	17	0	15	10	15	0	15	10	15	0	15	10	15	17.3	121.9	0	36.302	461740000	0	1.55E+08	19274000	2.88E+08	0	10138000	18286000	10003000
O00273	O00273	DNA fragmentation factor subunit alpha	DFFA	sp|O0027	8	8	8	0	7	1	7	0	7	1	7	0	7	1	7	37.2	36.521	0	30.643	460810000	0	1.75E+08	1058600	2.85E+08	0	17407000	0	9740200
Q9H6T3	Q9H6T3	RNA polymerase II-associated protein 3	RPAP3	sp|Q9H6T	10	10	10	1	7	4	9	1	7	4	9	1	7	4	9	23.5	75.718	0	40.18	460720000	1059600	1.11E+08	7152200	3.41E+08	2483900	11399000	5119000	10708000
Q9BQS8	Q9BQS8	FYVE and coiled-coil domain-containing protein 1	FYCO1	sp|Q9BQS	26	26	26	5	3	25	14	5	3	25	14	5	3	25	14	24.6	166.98	0	75.692	460050000	35609000	25110000	151840000	2.48E+08	33906000	2249100	108590000	5286000
P18564	P18564	Integrin beta-6	ITGB6	sp|P18564	1	1	1	0	1	1	1	0	1	1	1	0	1	1	1	1.1	85.935	0.003292	2.0834	459750000	0	12990000	3558500	4.43E+08	0	1145000	2391300	18227000
O00629	O00629	Importin subunit alpha-3	KPNA4	sp|O0062	8	4	4	1	6	1	7	0	3	0	4	0	3	0	4	21.5	57.886	0	58.059	458400000	0	1.45E+08	0	3.13E+08	0	12002000	0	13677000
Q9Y5Y2	Q9Y5Y2	Cytosolic Fe-S cluster assembly factor NUBP2	NUBP2	sp|Q9Y5Y	3	3	3	0	3	0	3	0	3	0	3	0	3	0	3	21.4	28.825	0	20.867	457800000	0	1.92E+08	0	2.66E+08	0	14009000	0	13838000
Q8IZP0	Q8IZP0	Abl interactor 1	ABI1	sp|Q8IZP0	10	10	8	0	9	3	8	0	9	3	8	0	7	2	6	26	55.08	0	32.894	456600000	0	1.91E+08	5491100	2.6E+08	0	17639000	1581500	12000000
Q9HC07	Q9HC07	Transmembrane protein 165	TMEM165	sp|Q9HC0	4	4	4	0	3	3	4	0	3	3	4	0	3	3	4	19.4	34.905	0	53.046	454410000	0	56163000	19879000	3.78E+08	0	4110800	11834000	13475000
O75251	O75251	NADH dehydrogenase [ubiquinone] iron-sulfur pr	NDUFS7	sp|O75251	4	4	4	1	4	1	4	1	4	1	4	1	4	1	4	15.5	23.563	0	12.447	453570000	551060	1.88E+08	3269100	2.62E+08	0	13373000	4600900	9819400
Q9UMY4	Q9UMY4	Sorting nexin-12	SNX12	sp|Q9UMY	8	6	6	0	7	0	6	0	5	0	5	0	5	0	5	62.3	18.884	0	19.739	453290000	0	1.9E+08	0	2.64E+08	0	18080000	0	9478500
Q9NP79	Q9NP79	Vacuolar protein sorting-associated protein VTA1	VTA1	sp|Q9NP7	6	6	6	0	6	0	3	0	6	0	3	0	6	0	3	22.8	33.879	0	27.281	451380000	0	2.98E+08	0	1.54E+08	0	26503000	0	6063700
Q9NPH2	Q9NPH2	Inositol-3-phosphate synthase 1	ISYNA1	sp|Q9NPH	9	9	9	0	9	0	1	0	9	0	1	0	9	0	1	24.2	61.067	0	47.124	451080000	0	4.45E+08	0	6522600	0	39188000	0	268250
Q5MNZ9	Q5MNZ9	WD repeat domain phosphoinositide-interacting	WIPI1	sp|Q5MN	3	3	3	0	1	1	3	0	1	1	3	0	1	1	3	18.2	48.673	0	11.035	450750000	0	74355000	217350000	1.59E+08	0	13498000	128990000	16671000
P49354	P49354	Protein farnesyltransferase/geranylgeranyltransfe	FNTA	sp|P49354	11	11	11	0	8	2	8	0	8	2	8	0	8	2	8	36.9	44.408	0	89.021	450250000	0	2.06E+08	4453800	2.4E+08	0	11549000	4933700	14516000
Q9UIJ7	Q9UIJ7	GTP:AMP phosphotransferase AK3, mitochondria	AK3	sp|Q9UIJ7	7	7	7	0	4	0	7	0	4	0	7	0	4	0	7	31.3	25.565	0	48.934	449490000	0	66809000	0	3.83E+08	0	7509600	0	14118000
Q13033	Q13033	Striatin-3	STRN3	sp|Q1303	10	10	9	0	7	3	9	0	7	3	9	0	6	2	8	21.2	87.208	0	84.499	449430000	0	88059000	3347300	3.58E+08	0	10552000	969710	13214000
Q9Y6D5	Q9Y6D5	Brefeldin A-inhibited guanine nucleotide-exchang	ARFGEF2	sp|Q9Y6D	15	15	11	1	6	4	14	1	6	4	14	1	4	3	10	12.7	202.04	0	36.625	449220000	567570	27094000	5907900	4.16E+08	1099300	1915300	1994500	19011000
Q9H307	Q9H307	Pinin	PNN	sp|Q9H30	9	9	9	0	7	9	4	0	7	9	4	0	7	9	4	15.1	81.627	0	34.809	448800000	0	1.7E+08	174200000	1.04E+08	0	24205000	104270000	7882100
O96005	O96005	Cleft lip and palate transmembrane protein 1	CLPTM1	sp|O9600	9	9	9	0	4	1	9	0	4	1	9	0	4	1	9	22	76.096	0	64.011	448310000	0	26804000	3228600	4.18E+08	0	3327500	2181900	15996000
Q9BTE3	Q9BTE3	Mini-chromosome maintenance complex-binding	MCMBP	sp|Q9BTE	12	12	12	0	12	1	8	0	12	1	8	0	12	1	8	26.2	72.979	0	46.96	448110000	0	2.92E+08	958970	1.55E+08	0	26417000	1066300	5281700
P07203	P07203	Glutathione peroxidase 1	GPX1	sp|P07203	6	6	6	0	6	0	6	0	6	0	6	0	6	0	6	31	22.088	0	18.444	447610000	0	44847000	0	4.03E+08	0	5648700	0	14869000
P52594	P52594	Arf-GAP domain and FG repeat-containing protei	AGFG1	sp|P52594	5	5	5	0	3	0	5	0	3	0	5	0	3	0	5	15.7	58.259	0	31.825	446850000	0	50192000	0	3.97E+08	0	7243300	0	13494000
P24666	P24666	Low molecular weight phosphotyrosine protein p	ACP1	sp|P24666	5	5	5	1	4	2	5	1	4	2	5	1	4	2	5	39.2	18.042	0	52.537	446340000	1105400	2.27E+08	2814600	2.16E+08	3324400	18313000	2810400	7179900
P42025	P42025	Beta-centractin	ACTR1B	sp|P42025	9	2	2	2	9	5	7	1	2	1	1	1	2	1	1	30.1	42.293	0	10.433	445620000	36181000	2.17E+08	775370	1.91E+08	30269000	16311000	6707200	7379100
O14974	O14974	Protein phosphatase 1 regulatory subunit 12A	PPP1R12A	sp|O1497	16	16	16	0	13	15	14	0	13	15	14	0	13	15	14	22.4	115.28	0	35.87	445400000	0	1.19E+08	90278000	2.36E+08	0	8758100	62981000	7751900
Q6P996;Q	Q6P996	Pyridoxal-dependent decarboxylase domain-cont	PDXDC1	sp|Q6P99	13	13	13	1	9	2	12	1	9	2	12	1	9	2	12	26.5	86.706	0	58.053	444390000	216090	91906000	4172900	3.48E+08	1212200	13341000	4031700	6852200
Q8IY17	Q8IY17	Neuropathy target esterase	PNPLA6	sp|Q8IY17	9	9	9	0	0	4	9	0	0	4	9	0	0	4	9	12.4	150.95	0	63.482	444200000	0	0	21017000	4.23E+08	0	0	17597000	13929000
Q15427	Q15427	Splicing factor 3B subunit 4	SF3B4	sp|Q1542	4	4	4	0	4	0	3	0	4	0	3	0	4	0	3	17.7	44.385	0	29.018	443590000	0	1.29E+08	0	3.15E+08	0	8200600	0	16111000
Q8NCW5	Q8NCW5	NAD(P)H-hydrate epimerase	APOA1BP	sp|Q8NCV	4	4	4	0	4	1	4	0	4	1	4	0	4	1	4	18.8	31.674	0	16.417	443210000	0	2.58E+08	349410	1.85E+08	0	25714000	1179100	3666200
Q16513	Q16513	Serine/threonine-protein kinase N2	PKN2	sp|Q1651	14	14	14	0	13	0	9	0	13	0	9	0	13	0	9	23.7	112.03	0	78.234	442750000	0	2.19E+08	0	2.24E+08	0	19115000	0	9403700
Q9Y5S2	Q9Y5S2	Serine/threonine-protein kinase MRCK beta	CDC42BPB	sp|Q9Y5S	13	13	12	0	5	4	13	0	5	4	13	0	5	3	12	11.2	194.31	0	48.666	441920000	0	17067000	5324500	4.2E+08	0	2167700	6381600	13786000
Q9UNN8	Q9UNN8	Endothelial protein C receptor	PROCR	sp|Q9UNN	1	1	1	0	0	0	1	0	0	0	1	0	0	0	1	7.1	26.671	0	7.4695	441270000	0	0	0	4.41E+08	0	0	0	18147000
Q14165	Q14165	Malectin	MLEC	sp|Q1416	5	5	5	0	4	0	5	0	4	0	5	0	4	0	5	17.8	32.233	0	9.6764	441100000	0	90306000	0	3.51E+08	0	9274500	0	13113000
Q96IJ6	Q96IJ6	Mannose-1-phosphate guanyltransferase alpha	GMPPA	sp|Q96IJ6	4	4	4	0	3	2	4	0	3	2	4	0	3	2	4	15.2	46.291	0	17.98	440950000	0	1.22E+08	7289500	3.11E+08	0	9387000	8783800	10310000
P35269	P35269	General transcription factor IIF subunit 1	GTF2F1	sp|P35269	9	9	9	0	8	3	8	0	8	3	8	0	8	3	8	21.1	58.24	0	53.866	440630000	0	2.08E+08	3936400	2.28E+08	0	14986000	4533600	10876000
P30046;A6	P30046;A6	D-dopachrome decarboxylase;D-dopachrome de	DDT;DDTL	sp|P30046	3	3	3	0	3	0	2	0	3	0	2	0	3	0	2	27.1	12.712	0	33.495	439940000	0	3.53E+08	0	87390000	0	28044000	0	6627000
Q7KZ85	Q7KZ85	Transcription elongation factor SPT6	SUPT6H	sp|Q7KZ8	11	11	11	1	8	0	11	1	8	0	11	1	8	0	11	9.7	199.07	0	70.101	439470000	827490	99387000	0	3.39E+08	3137700	11937000	0	8466100
Q13404	Q13404	Ubiquitin-conjugating enzyme E2 variant 1	UBE2V1	sp|Q1340	7	2	2	1	7	2	7	0	2	0	2	0	2	0	2	55.1	16.495	0	7.3384	439120000	0	1.6E+08	0	2.8E+08	0	10993000	0	9347900
Q14677	Q14677	Clathrin interactor 1	CLINT1	sp|Q1467	13	13	13	0	11	2	9	0	11	2	9	0	11	2	9	24.6	68.259	0	36.24	438760000	0	2.03E+08	9294700	2.27E+08	0	21779000	3385100	8285200
Q15024	Q15024	Exosome complex component RRP42	EXOSC7	sp|Q1502	6	6	6	0	6	5	4	0	6	5	4	0	6	5	4	22.3	31.821	0	34.788	438630000	0	1.72E+08	36304000	2.3E+08	0	12470000	25281000	11275000
Q8N7H5	Q8N7H5	RNA polymerase II-associated factor 1 homolog	PAF1	sp|Q8N7H	11	11	11	0	11	8	8	0	11	8	8	0	11	8	8	26.2	59.975	0	36.912	437170000	0	2.11E+08	33065000	1.93E+08	0	15927000	21997000	10831000
O15121	O15121	Sphingolipid delta(4)-desaturase DES1	DEGS1	sp|O15121	3	3	3	0	2	0	3	0	2	0	3	0	2	0	3	10.5	37.866	0	10.724	436980000	0	65036000	0	3.72E+08	0	5735300	0	15294000
O95801	O95801	Tetratricopeptide repeat protein 4	TTC4	sp|O95801	7	7	7	1	7	2	7	1	7	2	7	1	7	2	7	22.2	44.678	0	21.033	436710000	2330300	1.37E+08	1502800	2.96E+08	1960800	10768000	1420600	13441000
Q9BVA1	Q9BVA1	Tubulin beta-2B chain	TUBB2B	sp|Q9BVA	23	4	1	12	23	19	22	2	4	1	3	1	1	0	0	61.8	49.953	0	28.346	436410000	22135000	3.45E+08	5392600	63742000	31724000	21454000	3407300	2218500
Q9UH99	Q9UH99	SUN domain-containing protein 2	SUN2	sp|Q9UH9	8	8	8	1	0	2	8	1	0	2	8	1	0	2	8	23	80.31	0	48.81	435990000	6390000	0	16718000	4.13E+08	8002700	0	4455500	22147000
Q86UX7	Q86UX7	Fermitin family homolog 3	FERMT3	sp|Q86UX	10	10	10	0	1	2	10	0	1	2	10	0	1	2	10	23.7	75.952	0	39.51	435780000	0	7623500	16331000	4.12E+08	0	0	0	16937000
Q9UBV2	Q9UBV2	Protein sel-1 homolog 1	SEL1L	sp|Q9UBV	9	9	9	0	3	0	9	0	3	0	9	0	3	0	9	24.3	88.754	0	129.72	435100000	0	13713000	0	4.21E+08	0	2602100	0	15936000
P03886	P03886	NADH-ubiquinone oxidoreductase chain 1	MT-ND1	sp|P03886	3	3	3	0	3	0	3	0	3	0	3	0	3	0	3	8.8	35.66	0	12.291	433410000	0	1.98E+08	0	2.36E+08	0	12579000	0	14535000
Q96N66	Q96N66	Lysophospholipid acyltransferase 7	MBOAT7	sp|Q96N6	5	5	5	0	2	2	5	0	2	2	5	0	2	2	5	16.9	52.764	0	31.05	432750000	0	12969000	5422800	4.14E+08	0	1543700	5322700	14962000
Q9Y4E8	Q9Y4E8	Ubiquitin carboxyl-terminal hydrolase 15	USP15	sp|Q9Y4E	12	12	12	0	11	0	11	0	11	0	11	0	11	0	11	17.1	112.42	0	36.77	432640000	0	1.68E+08	0	2.65E+08	0	12981000	0	12715000
Q9UIC8	Q9UIC8	Leucine carboxyl methyltransferase 1	LCMT1	sp|Q9UIC	7	7	7	0	7	1	7	0	7	1	7	0	7	1	7	23.7	38.379	0	21.923	432390000	0	1.43E+08	1673900	2.88E+08	0	11846000	4577300	9136500
P63172	P63172	Dynein light chain Tctex-type 1	DYNLT1	sp|P63172	2	2	2	0	2	2	2	0	2	2	2	0	2	2	2	30.1	12.452	0	7.3223	432260000	0	60840000	12365000	3.59E+08	0	4749900	6443000	17246000
Q5T5P2	Q5T5P2	Sickle tail protein homolog	KIAA1217	sp|Q5T5P	20	20	20	3	1	17	15	3	1	17	15	3	1	17	15	13.9	214.11	0	41.702	431770000	729150	1.02E+08	79641000	2.5E+08	572830	0	53879000	10072000
O00186	O00186	Syntaxin-binding protein 3	STXBP3	sp|O0018	7	7	7	0	7	1	7	0	7	1	7	0	7	1	7	13.5	67.764	0	21.949	431360000	0	93096000	290260	3.38E+08	0	7136300	345040	14819000

Q8IZ83	Q8IZ83	Aldehyde dehydrogenase family 16 member A1	ALDH16A1	sp|Q8IZ83	9	9	9	0	9	1	4	0	9	1	4	0	9	1	4	18.2	85.126	0	29.949	431350000	0	2.79E+08	771210	1.51E+08	0	20012000	0	10831000
P61009	P61009	Signal peptidase complex subunit 3	SPCS3	sp|P61009	2	2	2	0	2	1	2	0	2	1	2	0	2	1	2	12.8	20.313	0	11.933	431310000	0	1.75E+08	2675000	2.54E+08	0	11204000	2431700	7873200
P19388	P19388	DNA-directed RNA polymerases I, II, and III subun	POLR2E	sp|P19388	3	3	3	0	3	3	2	0	3	3	2	0	3	3	2	16.7	24.551	0	17.667	430560000	0	1.38E+08	12213000	2.8E+08	0	10127000	12219000	9548800
Q9H0A8	Q9H0A8	COMM domain-containing protein 4	COMMD4	sp|Q9H0A	5	5	5	0	2	0	5	0	2	0	5	0	2	0	5	33.7	21.764	0	9.4591	429890000	0	7059500	0	4.23E+08	0	2862500	0	15149000
Q99816	Q99816	Tumor susceptibility gene 101 protein	TSG101	sp|Q9981	7	7	7	0	6	3	7	0	6	3	7	0	6	3	7	24.9	43.944	0	24.884	429540000	0	1.19E+08	4466400	3.06E+08	0	9825000	4324300	11941000
Q9UI09	Q9UI09	NADH dehydrogenase [ubiquinone] 1 alpha subco	NDUFA12	sp|Q9UI0	8	8	8	1	6	1	6	1	6	1	6	1	6	1	6	67.6	17.114	0	22.845	429480000	1847900	1.38E+08	820330	2.89E+08	0	12319000	865500	11388000
O95197	O95197	Reticulon-3	RTN3	sp|O9519	3	3	3	1	3	2	3	1	3	2	3	1	3	2	3	5.6	112.61	0	13.119	429120000	588460	1.82E+08	3087000	2.44E+08	1029300	15594000	1015200	11072000
Q13630	Q13630	GDP-L-fucose synthase	TSTA3	sp|Q1363	6	6	6	0	5	0	6	0	5	0	6	0	5	0	6	22.1	35.892	0	15.569	428510000	0	1.22E+08	0	3.06E+08	0	11260000	0	12116000
Q9NYP7	Q9NYP7	Elongation of very long chain fatty acids protein 5	ELOVL5	sp|Q9NYP	2	2	2	0	1	1	2	0	1	1	2	0	1	1	2	13.4	35.293	0	14.113	428300000	0	53668000	12809000	3.62E+08	0	4986400	9072700	13552000
Q9H9Q2	Q9H9Q2	COP9 signalosome complex subunit 7b	COPS7B	sp|Q9H9Q	5	5	5	0	5	0	5	0	5	0	5	0	5	0	5	27.3	29.622	0	30.823	428110000	0	1.71E+08	0	2.57E+08	0	15750000	0	9900400
O96000	O96000	NADH dehydrogenase [ubiquinone] 1 beta subcor	NDUFB10	sp|O9600	5	5	5	0	5	2	5	0	5	2	5	0	5	2	5	37.2	20.776	0	14.704	428090000	0	1.83E+08	1501200	2.44E+08	0	17386000	3559000	6189200
O94915	O94915	Protein furry homolog-like	FRYL	sp|O9491	15	15	15	0	2	1	15	0	2	1	15	0	2	1	15	7	339.59	0	47.522	427850000	0	6906000	595960	4.2E+08	0	371040	300950	17624000
Q8IWB9	Q8IWB9	Testis-expressed sequence 2 protein	TEX2	sp|Q8IWB	15	15	15	4	2	15	4	4	2	15	4	4	2	15	4	20.7	125.3	0	42.244	427770000	2270600	3373200	376120000	46000000	328810	148160	256280000	451410
O95218	O95218	Zinc finger Ran-binding domain-containing protei	ZRANB2	sp|O9521	8	8	8	0	5	8	6	0	5	8	6	0	5	8	6	27.6	37.404	0	36.92	427660000	0	81570000	217580000	1.29E+08	0	11583000	140620000	6482800
Q9UKD2	Q9UKD2	mRNA turnover protein 4 homolog	MRTO4	sp|Q9UKD	6	6	6	0	5	5	5	0	5	5	5	0	5	5	5	32.2	27.56	0	32.047	426980000	0	1.2E+08	19128000	2.88E+08	0	8642400	16407000	10226000
O14562	O14562	Ubiquitin domain-containing protein UBFD1	UBFD1	sp|O1456	5	5	5	0	4	0	4	0	4	0	4	0	4	0	4	27.2	33.382	0	19.897	426770000	0	1.36E+08	0	2.91E+08	0	14253000	0	9699200
Q9H3K2	Q9H3K2	Growth hormone-inducible transmembrane prot	GHITM	sp|Q9H3K	6	6	6	1	6	0	5	1	6	0	5	1	6	0	5	15.1	37.205	0	19.57	426500000	338310	2.27E+08	0	2E+08	483040	20681000	0	7356900
Q9NWY4;A	Q9NWY4	UPF0609 protein C4orf27	C4orf27	sp|Q9NWY	4	4	4	0	4	0	4	0	4	0	4	0	4	0	4	14.7	39.436	0	10.661	426340000	0	1.15E+08	0	3.12E+08	0	10351000	0	12578000
Q96EK5	Q96EK5	KIF1-binding protein	KIAA1279	sp|Q96EK	8	8	8	0	6	0	7	0	6	0	7	0	6	0	7	24	71.813	0	48.93	426270000	0	1.35E+08	0	2.91E+08	0	12925000	0	10956000
O43252	O43252	Bifunctional 3-phosphoadenosine 5-phosphosulfa	PAPSS1	sp|O4325	9	9	9	0	5	0	9	0	5	0	9	0	5	0	9	18.9	70.832	0	21.245	425650000	0	69589000	0	3.56E+08	0	8824500	0	11953000
Q5XKP0	Q5XKP0	Protein QIL1	QIL1	sp|Q5XKP	3	3	3	1	3	2	3	1	3	2	3	1	3	2	3	41.5	13.087	0	29.036	424740000	6357200	79353000	7907200	3.31E+08	9748900	6497500	7333300	8703800
P45954	P45954	Short/branched chain specific acyl-CoA dehydrog	ACADSB	sp|P45954	8	8	8	1	8	0	6	1	8	0	6	1	8	0	6	28.5	47.485	0	24.412	424130000	72889000	2.86E+08	0	65388000	0	24109000	0	3777700
Q13158	Q13158	FAS-associated death domain protein	FADD	sp|Q1315	3	3	3	0	2	0	3	0	2	0	3	0	2	0	3	20.7	23.279	0	32.122	420110000	0	30133000	0	3.9E+08	0	4677700	0	14017000
Q9NRF8	Q9NRF8	CTP synthase 2	CTPS2	sp|Q9NRF	9	6	6	1	9	1	7	1	6	0	5	1	6	0	5	23.9	65.677	0	20.902	419820000	148670	2.21E+08	0	1.99E+08	302990	25504000	0	1989900
Q5VW32	Q5VW32	BRO1 domain-containing protein BROX	BROX	sp|Q5VW3	5	5	5	0	4	0	5	0	4	0	5	0	4	0	5	23.6	46.476	0	15.998	419510000	0	1.82E+08	0	2.37E+08	0	16872000	0	8961600
O00743	O00743	Serine/threonine-protein phosphatase 6 catalytic	PPP6C	sp|O0074	6	6	6	0	5	3	6	0	5	3	6	0	5	3	6	24.3	35.144	0	19.594	419040000	0	1.44E+08	8794400	2.67E+08	0	9759200	7568700	11875000
O75122	O75122	CLIP-associating protein 2	CLASP2	sp|O7512	14	14	12	0	7	6	14	0	7	6	14	0	5	5	12	17	141.06	0	51.45	418420000	0	80565000	10513000	3.27E+08	0	8147600	10606000	8874500
Q9P1F3	Q9P1F3	Costars family protein ABRACL	ABRACL	sp|Q9P1F	1	1	1	0	1	0	1	0	1	0	1	0	1	0	1	19.8	9.0564	0	6.5244	418290000	0	1.22E+08	0	2.96E+08	0	10761000	0	12182000
Q53T59	Q53T59	HCLS1-binding protein 3	HS1BP3	sp|Q53T5	5	5	5	0	4	0	5	0	4	0	5	0	4	0	5	23.5	42.78	0	59.702	417600000	0	81238000	0	3.36E+08	0	8609800	0	12384000
Q99538	Q99538	Legumain	LGMN	sp|Q9953	2	2	2	0	1	1	2	0	1	1	2	0	1	1	2	6.5	49.411	0	7.497	417410000	0	33040000	2037400	3.82E+08	0	6473400	4438400	9093300
Q96BY6;Q	Q96BY6	Dedicator of cytokinesis protein 10	DOCK10	sp|Q96BY	17	17	17	1	0	1	17	1	0	1	17	1	0	1	17	11.3	249.53	0	106.53	417390000	455100	0	441070	4.17E+08	271810	0	219350	17389000
Q9Y3B8	Q9Y3B8	Oligoribonuclease, mitochondrial	REXO2	sp|Q9Y3B	3	3	3	0	3	1	2	0	3	1	2	0	3	1	2	15.2	26.832	0	11.197	417390000	0	1.77E+08	924900	2.39E+08	0	16699000	2795800	6579700
P18858	P18858	DNA ligase 1	LIG1	sp|P18858	14	14	14	0	13	0	11	0	13	0	11	0	13	0	11	22.2	101.73	0	34.498	417240000	0	2.23E+08	0	1.94E+08	0	18481000	0	9183000
Q08AF3;Q	Q08AF3	Schlafen family member 5	SLFN5	sp|Q08AF	12	12	12	0	1	7	12	0	1	7	12	0	1	7	12	15.7	101.05	0	28.996	415960000	0	870850	25745000	3.89E+08	0	347370	5692600	27350000
O43290	O43290	U4/U6.U5 tri-snRNP-associated protein 1	SART1	sp|O4329	14	14	14	1	10	11	9	1	10	11	9	1	10	11	9	28	90.254	0	40.032	415690000	2069700	98877000	68816000	2.46E+08	2897900	7637200	49122000	7486400
Q01469	Q01469	Fatty acid-binding protein, epidermal	FABP5	sp|Q0146	3	3	3	0	3	3	3	0	3	3	3	0	3	3	3	20.7	15.164	0	8.4771	415110000	0	2.04E+08	35435000	1.76E+08	0	21014000	20335000	7497700
Q9GZS3	Q9GZS3	WD repeat-containing protein 61;WD repeat-cont	WDR61	sp|Q9GZS	4	4	4	0	4	1	3	0	4	1	3	0	4	1	3	28.2	33.58	0	45.313	414510000	0	2.26E+08	6405500	1.82E+08	0	14602000	5985700	11109000
Q9P2I0	Q9P2I0	Cleavage and polyadenylation specificity factor su	CPSF2	sp|Q9P2I0	9	9	9	1	3	1	9	1	3	1	9	1	3	1	9	21	88.486	0	40.456	414220000	1308600	25800000	896240	3.86E+08	1322100	2742700	896930	15107000
Q8WYA6	Q8WYA6	Beta-catenin-like protein 1	CTNNBL1	sp|Q8WYA	9	9	9	0	7	2	9	0	7	2	9	0	7	2	9	18.7	65.173	0	50.827	413770000	0	1.68E+08	8915800	2.37E+08	0	10455000	10283000	9801300
Q9Y2X3	Q9Y2X3	Nucleolar protein 58	NOP58	sp|Q9Y2X	10	10	10	1	6	8	9	1	6	8	9	1	6	8	9	25.7	59.578	0	29.311	413700000	1124800	32960000	43577000	3.36E+08	1606800	3421000	35536000	6570000
Q6ZRP7	Q6ZRP7	Sulfhydryl oxidase 2	QSOX2	sp|Q6ZRP	5	5	5	0	4	0	5	0	4	0	5	0	4	0	5	8.6	77.528	0	26.281	413670000	0	29474000	0	3.84E+08	0	3221400	0	15177000
Q13671	Q13671	Ras and Rab interactor 1	RIN1	sp|Q13671	11	11	11	0	1	6	11	0	1	6	11	0	1	6	11	20.3	84.098	0	43.16	413180000	0	4185800	8571700	4E+08	0	0	7439900	14788000
Q08378	Q08378	Golgin subfamily A member 3	GOLGA3	sp|Q0837	20	20	20	1	15	2	18	1	15	2	18	1	15	2	18	18.5	167.35	0	61.15	413040000	493820	1.29E+08	2231400	2.81E+08	1261000	4775600	2197400	16706000
Q9H2W6	Q9H2W6	39S ribosomal protein L46, mitochondrial	MRPL46	sp|Q9H2W	7	7	7	3	6	0	6	3	6	0	6	3	6	0	6	39.1	31.705	0	40.257	412190000	11936000	1.79E+08	0	2.21E+08	13122000	16404000	0	7287500
P12110	P12110	Collagen alpha-2(VI) chain	COL6A2	sp|P12110	10	10	10	0	0	2	10	0	0	2	10	0	0	2	10	13.7	108.58	0	33.348	411710000	0	0	2120200	4.1E+08	0	0	1424800	16845000
Q9BYD2	Q9BYD2	39S ribosomal protein L9, mitochondrial	MRPL9	sp|Q9BYD	4	4	4	0	3	3	4	0	3	3	4	0	3	3	4	17.6	30.243	0	36.298	410630000	0	1.11E+08	6679700	2.93E+08	0	8786700	7350200	10164000
Q7Z2Z2	Q7Z2Z2	Elongation factor Tu GTP-binding domain-contain	EFTUD1	sp|Q7Z2Z	9	9	9	0	7	0	9	0	7	0	9	0	7	0	9	13.5	125.43	0	22.728	410540000	0	1.23E+08	0	2.87E+08	0	11058000	0	11628000
Q02413	Q02413	Desmoglein-1	DSG1	sp|Q0241	14	14	14	1	5	14	5	1	5	14	5	1	5	14	5	19	113.75	0	57.851	409370000	688100	75300000	300560000	32820000	1903500	5219100	187210000	1899400
P20339	P20339	Ras-related protein Rab-5A	RAB5A	sp|P20339	7	4	4	0	7	1	6	0	4	0	4	0	4	0	4	46	23.658	0	7.0692	407780000	0	1.38E+08	0	2.7E+08	0	12689000	0	10570000
A0FGR8	A0FGR8	Extended synaptotagmin-2	ESYT2	sp|A0FGR	9	9	9	0	7	4	8	0	7	4	8	0	7	4	8	17.5	102.36	0	73.582	407720000	0	92134000	6361100	3.09E+08	0	5908500	8723300	10481000
Q86TI2	Q86TI2	Dipeptidyl peptidase 9	DPP9	sp|Q86TI2	12	12	12	0	6	0	12	0	6	0	12	0	6	0	12	18.1	98.262	0	50.722	407340000	0	35850000	0	3.71E+08	0	6322400	0	12115000
Q96JB5	Q96JB5	CDK5 regulatory subunit-associated protein 3	CDK5RAP3	sp|Q96JB5	8	8	8	0	8	2	7	0	8	2	7	0	8	2	7	19.6	56.92	0	80.793	407180000	0	1.49E+08	2133900	2.56E+08	0	15864000	1290600	7961500
O95777	O95777	U6 snRNA-associated Sm-like protein LSm8	LSM8	sp|O9577	4	4	4	0	4	0	2	0	4	0	2	0	4	0	2	62.5	10.403	0	28.072	406190000	0	2.06E+08	0	2E+08	0	16151000	0	10255000
Q96G23	Q96G23	Ceramide synthase 2	CERS2	sp|Q96G2	5	5	5	0	3	2	5	0	3	2	5	0	3	2	5	13.4	44.876	0	16.299	404470000	0	41157000	13174000	3.5E+08	0	3509300	10832000	12539000
Q9H223	Q9H223	EH domain-containing protein 4	EHD4	sp|Q9H22	8	6	6	1	8	2	6	1	6	2	5	1	6	2	5	18.7	61.174	0	24.203	404350000	1874600	1.32E+08	4383300	2.66E+08	3747400	6677100	4272200	12696000
Q96CN7	Q96CN7	Isochorismatase domain-containing protein 1	ISOC1	sp|Q96CN	7	7	7	3	7	1	4	3	7	1	4	3	7	1	4	33.9	32.236	0	24.4	403880000	19736000	2.37E+08	968940	1.46E+08	21277000	17350000	1804400	6847600
Q9BRA2	Q9BRA2	Thioredoxin domain-containing protein 17	TXNDC17	sp|Q9BRA	4	4	4	0	4	0	4	0	4	0	4	0	4	0	4	38.2	13.941	0	13.134	403860000	0	1.79E+08	0	2.25E+08	0	15738000	0	9284700
Q06787	Q06787	Fragile X mental retardation protein 1	FMR1	sp|Q0678	12	10	10	0	10	10	8	0	9	8	6	0	9	8	6	26.9	71.174	0	27.247	403840000	0	1.37E+08	88938000	1.78E+08	0	13263000	59477000	6412800
O14745	O14745	Na(+)/H(+) exchange regulatory cofactor NHE-RF1	SLC9A3R1	sp|O1474	6	6	6	0	6	1	6	0	6	1	6	0	6	1	6	23.5	38.868	0	21.901	403760000	0	1.7E+08	387890	2.33E+08	0	16307000	465350	8067100
O95819;Q	O95819	Mitogen-activated protein kinase kinase kinase ki	MAP4K4	sp|O9581	16	16	12	2	7	10	16	2	7	10	16	1	5	8	12	16.9	142.1	0	44.209	402610000	15098000	51394000	22577000	3.14E+08	21042000	2828100	14620000	9205100
O00264	O00264	Membrane-associated progesterone receptor cor	PGRMC1	sp|O0026	6	6	6	0	6	1	4	0	6	1	4	0	6	1	4	46.2	21.671	0	67.004	402490000	0	2.25E+08	680510	1.76E+08	0	17600000	3114600	6870400
Q69YQ0	Q69YQ0	Cytospin-A	SPECC1L	sp|Q69YQ	21	21	21	2	9	21	10	2	9	21	10	2	9	21	10	28.5	124.6	0	55.285	401500000	5234200	53349000	248010000	94899000	4345700	4784500	162660000	8711000
O60739	O60739	Eukaryotic translation initiation factor 1b	EIF1B	sp|O6073	7	2	2	1	7	2	6	0	2	0	2	0	2	0	2	65.5	12.823	0	61.346	400990000	0	1.92E+08	0	2.09E+08	0	17029000	0	8478300
P09110	P09110	3-ketoacyl-CoA thiolase, peroxisomal	ACAA1	sp|P09110	4	4	4	0	3	0	4	0	3	0	4	0	3	0	4	20.5	44.292	0	16.532	400750000	0	86226000	0	3.15E+08	0	8665700	0	11870000
Q6UW68	Q6UW68	Transmembrane protein 205	TMEM205	sp|Q6UW	2	2	2	0	2	1	2	0	2	1	2	0	2	1	2	15.9	21.198	0	11.328	400720000	0	85884000	1233000	3.14E+08	0	7147200	2004500	12145000
O94822	O94822	E3 ubiquitin-protein ligase listerin	LTN1	sp|O9482	12	12	12	0	4	3	12	0	4	3	12	0	4	3	12	11.3	200.55	0	51.609	400680000	0	16838000	10442000	3.73E+08	0	3875800	5330500	14651000
P48960	P48960	CD97 antigen;CD97 antigen subunit alpha;CD97 a	CD97	sp|P48960	6	6	6	0	1	1	6	0	1	1	6	0	1	1	6	10.4	91.868	0	44.572	397850000	0	2191500	2414300	3.93E+08	0	199780	578960	17209000
Q96S44	Q96S44	TP53-regulating kinase	TP53RK	sp|Q96S4	6	6	6	0	4	2	6	0	4	2	6	0	4	2	6	30.8	28.16	0	20.377	397330000	0	89747000	3216000	3.04E+08	0	6804900	2036600	13748000
O15027	O15027	Protein transport protein Sec16A	SEC16A	sp|O1502	17	17	17	1	15	0	15	1	15	0	15	1	15	0	15	12.3	251.89	0	68.958	397210000	1732000	1.76E+08	0	2.19E+08	2755800	18211000	0	5316800
P19174	P19174	1-phosphatidylinositol 4,5-bisphosphate phospho	PLCG1	sp|P19174	10	10	10	0	6	1	10	0	6	1	10	0	6	1	10	12.6	148.53	0	33.381	397030000	0	1.39E+08	344830	2.57E+08	0	12341000	289240	10468000
Q6IAN0	Q6IAN0	Dehydrogenase/reductase SDR family member 7E	DHRS7B	sp|Q6IAN	5	5	5	1	4	2	4	1	4	2	4	1	4	2	4	21.2	35.119	0	17.051	395880000	675560	2.25E+08	4867400	1.65E+08	2079900	20877000	3613100	4024800
O43865	O43865	Putative adenosylhomocysteinase 2	AHCYL1	sp|O4386	7	7	4	0	7	1	6	0	7	1	6	0	4	0	3	15.8	58.951	0	21.952	395110000	0	1.17E+08	655390	2.77E+08	0	10141000	439930	11607000
P48729;Q8	P48729	Casein kinase I isoform alpha	CSNK1A1	sp|P48729	9	9	9	3	5	9	8	3	5	9	8	3	5	9	8	29.7	38.914	0	24.849	395000000	1668900	37568000	71405000	2.84E+08	1486800	2502400	52705000	7964700
Q13190	Q13190	Syntaxin-5	STX5	sp|Q1319	6	6	6	1	4	3	6	1	4	3	6	1	4	3	6	25.4	39.672	0	20.649	394730000	1523800	91073000	9069300	2.93E+08	3288900	6316300	5066700	13027000
P30825;P5	P30825	High affinity cationic amino acid transporter 1	SLC7A1	sp|P30825	3	3	3	0	3	0	3	0	3	0	3	0	3	0	3	6.8	67.638	0	27.356	393680000	0	1.11E+08	0	2.83E+08	0	10663000	0	10745000
Q9UBX3	Q9UBX3	Mitochondrial dicarboxylate carrier	SLC25A10	sp|Q9UBX	4	4	4	0	4	1	3	0	4	1	3	0	4	1	3	18.8	31.282	0	23.83	393440000	0	2.46E+08	2598000	1.45E+08	0	21617000	3330200	4424900
Q9NZ45	Q9NZ45	CDGSH iron-sulfur domain-containing protein 1	CISD1	sp|Q9NZ4	3	3	3	1	3	2	1	1	3	2	1	1	3	2	1	34.3	12.199	0	11.372	392380000	1647000	2.83E+08	3170800	1.05E+08	7441600	18943000	2966900	3673800
Q8TAE8	Q8TAE8	Growth arrest and DNA damage-inducible proteir	GADD45GI	sp|Q8TAE	5	5	5	0	5	4	5	0	5	4	5	0	5	4	5	30.6	25.384	0	22.448	391660000	0	2.02E+08	20527000	1.69E+08	0	14091000	16040000	8423900
Q9BXL7	Q9BXL7	Caspase recruitment domain-containing protein 1	CARD11	sp|Q9BXL	1	1	1	1	0	0	0	1	0	0	0	1	0	0	0	1.8	133.28	0.001566	2.3076	391560000	3.92E+08	0	0	0	3.92E+08	0	0	0
O95340	O95340	Bifunctional 3-phosphoadenosine 5-phosphosulfa	PAPSS2	sp|O9534	8	8	8	0	1	0	8	0	1	0	8	0	1	0	8	17.8	69.5	0	27.225	391300000	0	3156800	0	3.88E+08	0	278270	0	15963000
Q7Z4V5	Q7Z4V5	Hepatoma-derived growth factor-related protein	HDGFRP2	sp|Q7Z4V	6	5	4	0	5	4	6	0	4	4	5	0	4	4	4	11.2	74.316	0	16.535	390340000	0	1.27E+08	17740000	2.46E+08	0	14900000	10751000	7557700
P22059;Q	P22059	Oxysterol-binding protein 1	OSBP	sp|P22059	9	9	9	0	8	1	8	0	8	1	8	0	8	1	8	22.6	89.42	0	27.661	389920000	0	1.46E+08	537280	2.43E+08	0	10458000	2531200	10265000
P35244	P35244	Replication protein A 14 kDa subunit	RPA3	sp|P35244	5	5	5	2	4	1	3	2	4	1	3	2	4	1	3	61.2	13.569	0	34.285	389430000	50795000	1.25E+08	1989900	2.12E+08	50695000	10906000	1769700	8476000
Q9Y3E0	Q9Y3E0	Vesicle transport protein GOT1B	GOLT1B	sp|Q9Y3E	3	3	3	0	2	1	3	0	2	1	3	0	2	1	3	17.4	15.425	0	12.276	388560000	0	15822000	730070	3.72E+08	0	1925300	2118600	13140000
P01130	P01130	Low-density lipoprotein receptor	LDLR	sp|P01130	6	6	6	0	0	2	6	0	0	2	6	0	0	2	6	11.3	95.375	0	70.074	388310000	0	0	4829800	3.83E+08	0	0	4251800	14765000
Q9Y5X1	Q9Y5X1	Sorting nexin-9	SNX9	sp|Q9Y5X1	6	6	6	0	4	1	6	0	4	1	6	0	4	1	6	19.8	66.591	0	54.276	388290000	0	87592000	888910	3E+08	0	3350700	1971000	15327000
Q8WVY7	Q8WVY7	Ubiquitin-like domain-containing CTD phosphatas	UBLCP1	sp|Q8WVY	9	9	9	0	8	0	6	0	8	0	6	0	8	0	6	38.4	36.804	0	57.759	388120000	0	1.17E+08	0	2.71E+08	0	11075000	0	10400000
P01911;P7	P01911	HLA class II histocompatibility antigen, DRB1-15 b	HLA-DRB1	sp|P01911	6	6	4	0	1	3	6	0	1	3	6	0	1	2	4	38.3	29.966	0	32.291	387830000	0	1950200	8783000	3.77E+08	0	313930	1682100	19586000
P42345	P42345	Serine/threonine-protein kinase mTOR	MTOR	sp|P42345	11	11	11	0	7	4	11	0	7	4	11	0	7	4	11	7.3	288.89	0	49.375	387280000	0	66115000	9736300	3.11E+08	0	7926300	9847900	7404200

P14927	P14927	Cytochrome b-c1 complex subunit 7	UQCRB	sp|P14927	4	4	4	1	4	1	3	1	4	1	3	1	4	1	3	38.7	13.53	0	17.006	386690000	2306100	2.28E+08	897370	1.55E+08	2912200	21775000	811030	3904900
P33121	P33121	Long-chain-fatty-acid--CoA ligase 1	ACSL1	sp|P33121	11	11	11	0	6	1	11	0	6	1	11	0	6	1	11	17.9	77.942	0	34.758	386230000	0	43752000	500810	3.42E+08	0	3570000	640300	14047000
Q96E11	Q96E11	Ribosome-recycling factor, mitochondrial	MRRF	sp|Q96E11	5	5	5	0	5	1	4	0	5	1	4	0	5	1	4	26.7	29.277	0	33.361	385970000	0	1.81E+08	10129000	1.95E+08	0	10731000	14192000	5852800
P52732	P52732	Kinesin-like protein KIF11	KIF11	sp|P52732	9	9	9	2	9	0	6	2	9	0	6	2	9	0	6	10.9	119.16	0	21.766	384080000	6011500	2.33E+08	0	1.45E+08	7850900	16562000	0	8095800
Q8IX04	Q8IX04	Ubiquitin-conjugating enzyme E2 variant 3	UEVLD	sp|Q8IX04	6	6	6	0	2	1	6	0	2	1	6	0	2	1	6	15.3	52.263	0	7.6721	383990000	0	1.53E+08	987950	2.3E+08	0	16301000	1387200	5908800
Q9Y315	Q9Y315	Deoxyribose-phosphate aldolase	DERA	sp|Q9Y315	5	5	5	2	5	3	5	2	5	3	5	2	5	3	5	23.6	35.23	0	24.499	383270000	73076000	1.1E+08	9815000	1.91E+08	63868000	13130000	11855000	8328000
Q96GA7	Q96GA7	Serine dehydratase-like	SDSL	sp|Q96GA	4	4	4	1	3	0	4	1	3	0	4	1	3	0	4	20.1	34.674	0	43.711	382970000	819060	31393000	0	3.51E+08	5667000	2165500	0	10179000
Q96DB5	Q96DB5	Regulator of microtubule dynamics protein 1	RMDN1	sp|Q96DB	8	8	8	0	8	0	6	0	8	0	6	0	8	0	6	27.7	35.808	0	47.437	381910000	0	1.9E+08	0	1.92E+08	0	13793000	0	10831000
Q01581;P5	Q01581	Hydroxymethylglutaryl-CoA synthase, cytoplasmi	HMGCS1	sp|Q01581	10	10	10	0	8	0	10	0	8	0	10	0	8	0	10	27.5	57.293	0	31.538	381280000	0	1.09E+08	0	2.72E+08	0	10762000	0	10045000
P30740	P30740	Leukocyte elastase inhibitor	SERPINB1	sp|P30740	8	8	8	0	7	0	8	0	7	0	8	0	7	0	8	30.3	42.741	0	40.571	380330000	0	95361000	0	2.85E+08	0	8758200	0	11367000
O76031	O76031	ATP-dependent Clp protease ATP-binding subunit	CLPX	sp|O76031	11	11	11	1	9	1	10	1	9	1	10	1	9	1	10	25.3	69.223	0	33.292	379990000	1955500	1.42E+08	1804000	2.35E+08	3345000	11356000	1727300	8872100
Q9BTU6	Q9BTU6	Phosphatidylinositol 4-kinase type 2-alpha	PI4K2A	sp|Q9BTU	14	14	14	0	7	0	12	0	7	0	12	0	7	0	12	42	54.022	0	33.066	379690000	0	60325000	0	3.19E+08	0	6187000	0	12265000
Q5JWF2;P1	Q5JWF2	Guanine nucleotide-binding protein G(s) subunit a	GNAS	sp|Q5JWF	8	7	7	1	7	6	7	1	6	5	6	1	6	5	6	8.6	111.02	0	21.656	379450000	13488000	98338000	15090000	2.53E+08	11613000	14194000	7595000	9280500
Q9Y371	Q9Y371	Endophilin-B1	SH3GLB1	sp|Q9Y371	6	6	6	0	4	0	5	0	4	0	5	0	4	0	5	24.7	40.796	0	32.548	379160000	0	1.73E+08	0	2.06E+08	0	16787000	0	6958700
Q9NW13	Q9NW13	RNA-binding protein 28	RBM28	sp|Q9NW1	12	12	12	0	7	10	10	0	7	10	10	0	7	10	10	20.6	85.737	0	35.535	378510000	0	44986000	59384000	2.74E+08	0	5106200	40002000	10037000
P46976	P46976	Glycogenin-1	GYG1	sp|P46976	6	6	6	0	5	3	6	0	5	3	6	0	5	3	6	16.3	39.383	0	22.995	378460000	0	1.47E+08	31475000	2E+08	0	12964000	22096000	7258100
Q9Y5P6	Q9Y5P6	Mannose-1-phosphate guanyltransferase beta	GMPPB	sp|Q9Y5P	7	7	7	0	3	0	7	0	3	0	7	0	3	0	7	30	39.834	0	22.401	378390000	0	87304000	0	2.91E+08	0	8975800	0	10691000
P50579	P50579	Methionine aminopeptidase 2	METAP2	sp|P50579	7	7	7	0	7	2	6	0	7	2	6	0	7	2	6	22.8	52.891	0	54.617	378070000	0	2.28E+08	1491000	1.48E+08	0	16925000	3616600	4236900
Q5T6V5	Q5T6V5	UPF0553 protein C9orf64	C9orf64	sp|Q5T6V	8	8	8	0	7	0	7	0	7	0	7	0	7	0	7	25.2	39.028	0	20.09	378040000	0	1.33E+08	0	2.45E+08	0	12284000	0	9537000
Q8WX92	Q8WX92	Negative elongation factor B	NELFB	sp|Q8WX9	7	7	7	0	6	1	4	0	6	1	4	0	6	1	4	18.3	65.697	0	20.183	378020000	0	1.49E+08	573690	2.28E+08	0	18139000	911440	3886700
Q9UI30	Q9UI30	Multifunctional methyltransferase subunit TRM11	TRMT112	sp|Q9UI30	4	4	4	0	3	2	4	0	3	2	4	0	3	2	4	37.6	14.199	0	23.958	377670000	0	51623000	4257600	3.22E+08	0	4782200	4731200	11132000
Q92609	Q92609	TBC1 domain family member 5	TBC1D5	sp|Q9260	9	9	9	0	4	4	9	0	4	4	9	0	4	4	9	22.8	89.003	0	52.441	377400000	0	14625000	6245600	3.57E+08	0	3773400	5027900	11347000
Q9H8Y8;Q	Q9H8Y8	Golgi reassembly-stacking protein 2	GORASP2	sp|Q9H8Y	6	6	6	0	6	0	5	0	6	0	5	0	6	0	5	22.8	47.145	0	21.039	376450000	0	1.45E+08	0	2.32E+08	0	13197000	0	9092500
Q4KMP7	Q4KMP7	TBC1 domain family member 10B	TBC1D10B	sp|Q4KMP	10	10	10	0	8	8	10	0	8	8	10	0	8	8	10	23.8	87.198	0	28.295	375920000	0	99929000	32365000	2.44E+08	0	8631100	22335000	9610900
P0C0L4;P0	P0C0L4;P0	Complement C4-A;Complement C4 beta chain;Co	C4A;C4B	sp|P0C0L4	7	4	4	3	0	5	2	3	0	4	0	3	0	4	0	5.6	192.78	0	27.27	375840000	2.15E+08	0	160500000	0	1.7E+08	0	152800000	0
Q13042	Q13042	Cell division cycle protein 16 homolog	CDC16	sp|Q13042	12	12	12	0	2	12	3	0	2	12	3	0	2	12	3	34.2	71.655	0	38.079	375580000	0	12958000	302680000	59947000	0	812820	199390000	1718700
P50583	P50583	Bis(5-nucleosyl)-tetraphosphatase [asymmetrical]	NUDT2	sp|P50583	2	2	2	0	2	0	2	0	2	0	2	0	2	0	2	23.1	16.829	0	15.028	375090000	0	44126000	0	3.31E+08	0	5524100	0	11977000
Q5UIP0	Q5UIP0	Telomere-associated protein RIF1	RIF1	sp|Q5UIP	11	11	11	0	10	5	6	0	10	5	6	0	10	5	6	6.3	274.46	0	30.283	374590000	0	1.66E+08	26551000	1.82E+08	0	9831000	23639000	6480500
Q8NFH4	Q8NFH4	Nucleoporin Nup37	NUP37	sp|Q8NFH	4	4	4	0	3	0	4	0	3	0	4	0	3	0	4	14.1	36.707	0	12.829	373640000	0	26769000	0	3.47E+08	0	3679400	0	12945000
P26440	P26440	Isovaleryl-CoA dehydrogenase, mitochondrial	IVD	sp|P26440	9	9	9	0	8	1	8	0	8	1	8	0	8	1	8	31.9	46.65	0	55.463	373500000	0	1.51E+08	1000300	2.21E+08	0	12172000	0	10258000
Q32P41	Q32P41	tRNA (guanine(37)-N1)-methyltransferase	TRMT5	sp|Q32P41	7	7	7	0	4	0	7	0	4	0	7	0	4	0	7	22.2	58.246	0	34.835	373420000	0	48879000	0	3.25E+08	0	6890800	0	10765000
O43488;Q8	O43488	Aflatoxin B1 aldehyde reductase member 2	AKR7A2	sp|O43488	6	6	6	0	6	0	4	0	6	0	4	0	6	0	4	14.5	39.589	0	22.507	373060000	0	1.26E+08	0	2.47E+08	0	11204000	0	10067000
Q9BTE7;Q	Q9BTE7	DCN1-like protein 5	DCUN1D5	sp|Q9BTE	7	7	7	0	7	1	5	0	7	1	5	0	7	1	5	32.5	27.508	0	29.17	372960000	0	2.51E+08	3967200	1.18E+08	0	22215000	3882500	3568200
P68402	P68402	Platelet-activating factor acetylhydrolase IB subur	PAFAH1B2	sp|P68402	2	2	2	0	2	0	2	0	2	0	2	0	2	0	2	12.2	25.569	0	23.664	372230000	0	2.78E+08	0	93796000	0	24928000	0	3472900
P10619	P10619	Lysosomal protective protein;Lysosomal protectiv	CTSA	sp|P10619	4	4	4	0	3	3	4	0	3	3	4	0	3	3	4	9.4	54.465	0	9.9129	371910000	0	18353000	14089000	3.39E+08	0	1947700	9618300	13481000
P30876	P30876	DNA-directed RNA polymerase II subunit RPB2	POLR2B	sp|P30876	12	12	12	0	6	8	11	0	6	8	11	0	6	8	11	13.4	133.9	0	42.242	371910000	0	48894000	18031000	3.05E+08	0	5034600	14889000	9046300
A6ND36	A6ND36	Protein FAM83G	FAM83G	sp|A6ND3	15	15	15	3	0	15	3	3	0	15	3	3	0	15	3	20.8	90.834	0	36.366	371690000	1.94E+08	0	155690000	21664000	67497000	0	222230000	5648400
Q8N335	Q8N335	Glycerol-3-phosphate dehydrogenase 1-like prote	GPD1L	sp|Q8N33	7	7	7	0	6	0	5	0	6	0	5	0	6	0	5	26.5	38.418	0	15.586	371640000	0	1.18E+08	0	2.53E+08	0	13232000	0	7616600
Q9P003	Q9P003	Protein cornichon homolog 4	CNIH4	sp|Q9P00	1	1	1	0	1	1	1	0	1	1	1	0	1	1	1	14.4	16.093	0	9.7147	371040000	0	1.01E+08	4629600	2.65E+08	0	8939600	3111100	10898000
Q8IWX8	Q8IWX8	Calcium homeostasis endoplasmic reticulum prot	CHERP	sp|Q8IWX	10	10	10	0	10	4	6	0	10	4	6	0	10	4	6	15.7	103.7	0	26.172	370580000	0	2.23E+08	15861000	1.32E+08	0	13072000	15047000	6230100
Q13439	Q13439	Golgin subfamily A member 4	GOLGA4	sp|Q1343	18	18	18	1	4	1	18	1	4	1	18	1	4	1	18	10.4	261.14	0	60.103	370350000	3885700	16167000	472310	3.5E+08	1846300	1681800	527690	15959000
Q9UL63	Q9UL63	Muskelin	MKLN1	sp|Q9UL6	8	8	8	1	2	2	8	1	2	2	8	1	2	2	8	19.5	84.767	0	85.541	370160000	1528200	19521000	3991400	3.45E+08	1303500	800110	2833100	15188000
P46734	P46734	Dual specificity mitogen-activated protein kinase	MAP2K3	sp|P46734	6	6	5	0	4	1	6	0	4	1	6	0	3	1	5	23.1	39.318	0	23.96	370110000	0	37627000	652940	3.32E+08	0	2286800	624310	14491000
Q9BVG4	Q9BVG4	Protein PBDC1	PBDC1	sp|Q9BVG	6	6	6	0	5	2	6	0	5	2	6	0	5	2	6	42.9	26.056	0	28.307	370110000	0	98796000	855040	2.7E+08	0	11718000	1460300	7227400
Q00169	Q00169	Phosphatidylinositol transfer protein alpha isofor	PITPNA	sp|Q0016	6	6	6	0	6	0	5	0	6	0	5	0	6	0	5	29.3	31.806	0	57.193	368940000	0	1.61E+08	0	2.08E+08	0	12764000	0	9997500
Q9NXR7	Q9NXR7	BRCA1-A complex subunit BRE	BRE	sp|Q9NXR	5	5	5	0	5	0	4	0	5	0	4	0	5	0	4	23	43.551	0	39.811	368930000	0	1.56E+08	0	2.13E+08	0	13539000	0	8982100
Q9P015	Q9P015	39S ribosomal protein L15, mitochondrial	MRPL15	sp|Q9P01	6	6	6	1	4	3	6	1	4	3	6	1	4	3	6	26.7	33.419	0	12.256	368060000	1041800	97653000	9910900	2.59E+08	5269200	7906400	6663800	7141000
Q9Y2W1	Q9Y2W1	Thyroid hormone receptor-associated protein 3	THRAP3	sp|Q9Y2W	10	10	10	4	8	8	7	4	8	8	7	4	8	8	7	12.3	108.66	0	29.904	367810000	20535000	1.47E+08	42933000	1.58E+08	36065000	8169000	20372000	4184100
Q7Z739	Q7Z739	YTH domain-containing family protein 3	YTHDF3	sp|Q7Z73	10	10	6	0	7	6	9	0	7	6	9	0	4	3	5	29.1	63.86	0	32.349	367350000	0	75526000	17480000	2.74E+08	0	8332400	13636000	7718400
Q96KP1	Q96KP1	Exocyst complex component 2	EXOC2	sp|Q96KP	9	9	9	0	7	5	8	0	7	5	8	0	7	5	8	11.7	104.07	0	20.814	366170000	0	67455000	11639000	2.87E+08	0	6142600	9957600	9473400
O95249	O95249	Golgi SNAP receptor complex member 1	GOSR1	sp|O9524	5	5	5	1	4	1	4	1	4	1	4	1	4	1	4	26.8	28.612	0	17.877	366040000	1.53E+08	55539000	1501200	1.56E+08	1.24E+08	20546000	11336000	10072000
Q15031	Q15031	Probable leucine--tRNA ligase, mitochondrial	LARS2	sp|Q15031	11	11	11	0	11	0	10	0	11	0	10	0	11	0	10	16.6	101.97	0	28.052	365540000	0	1.51E+08	0	2.14E+08	0	12087000	0	10062000
Q9UJY4	Q9UJY4	ADP-ribosylation factor-binding protein GGA2	GGA2	sp|Q9UJY4	2	2	2	1	1	1	0	1	1	1	0	1	1	1	0	3.9	67.15	0.00117	2.9577	365370000	3.45E+08	9755500	10122000	0	0	0	6802200	0
Q5VTU8;P	Q5VTU8;P	ATP synthase subunit epsilon-like protein, mitoch	ATP5EP2;A	sp|Q5VTU	2	2	2	0	2	1	2	0	2	1	2	0	2	1	2	31.4	5.8068	0.000484	3.4203	365130000	0	1.93E+08	3483200	1.69E+08	0	16405000	4574500	5288800
Q96GK7;Q	Q96GK7;Q	Fumarylacetoacetate hydrolase domain-containin	FAHD2A;F	sp|Q96GK	6	6	6	0	6	0	6	0	6	0	6	0	6	0	6	27.7	34.596	0	30.095	365020000	0	93333000	0	2.72E+08	0	7634100	0	11766000
O00541	O00541	Pescadillo homolog	PES1	sp|O00541	12	12	12	1	10	9	11	1	10	9	11	1	10	9	11	20.4	68.002	0	24.541	364750000	1485700	1.13E+08	29894000	2.2E+08	5212900	7939200	16374000	11085000
P27144	P27144	Adenylate kinase 4, mitochondrial	AK4	sp|P27144	6	6	6	1	6	2	6	1	6	2	6	1	6	2	6	43.5	25.268	0	30.68	364720000	17652000	1.68E+08	1061900	1.78E+08	23925000	11556000	0	4302500
Q5T653	Q5T653	39S ribosomal protein L2, mitochondrial	MRPL2	sp|Q5T65	3	3	3	0	3	3	3	0	3	3	3	0	3	3	3	17.7	33.3	0	41.921	364650000	0	1.24E+08	7628400	2.33E+08	0	11918000	6552700	7153200
Q9UHA4	Q9UHA4	Ragulator complex protein LAMTOR3	LAMTOR3	sp|Q9UHA	2	2	2	0	2	0	2	0	2	0	2	0	2	0	2	30.6	13.623	0	25.638	364600000	0	1.07E+08	0	2.57E+08	0	9394500	0	10645000
Q9NR50	Q9NR50	Translation initiation factor eIF-2B subunit gamm	EIF2B3	sp|Q9NR5	7	7	7	2	7	6	6	2	7	6	6	2	7	6	6	20.8	50.24	0	20.387	364450000	10814000	1.35E+08	13073000	2.06E+08	6016900	18505000	8089500	7338000
P57737	P57737	Coronin-7	CORO7	sp|P57737	6	6	6	0	6	1	4	0	6	1	4	0	6	1	4	9.8	100.6	0	32.926	363520000	0	2.03E+08	489260	1.6E+08	0	20133000	1629400	3024000
Q16850	Q16850	Lanosterol 14-alpha demethylase	CYP51A1	sp|Q1685	11	11	11	0	11	0	7	0	11	0	7	0	11	0	7	27.8	56.805	0	32.945	362850000	0	2.25E+08	0	1.38E+08	0	17968000	0	7527800
Q6UW02	Q6UW02	Cytochrome P450 20A1	CYP20A1	sp|Q6UW	6	6	6	0	4	0	6	0	4	0	6	0	4	0	6	19.7	52.432	0	30.751	361710000	0	70932000	0	2.91E+08	0	6992700	0	11218000
Q92882	Q92882	Osteoclast-stimulating factor 1	OSTF1	sp|Q9288	3	3	3	0	3	0	3	0	3	0	3	0	3	0	3	26.2	23.787	0	22.708	361410000	0	1.63E+08	0	1.98E+08	0	20030000	0	2515200
O00267	O00267	Transcription elongation factor SPT5	SUPT5H	sp|O0026	10	10	10	1	10	1	7	1	10	1	7	1	10	1	7	14.3	121	0	35.275	360960000	618070	1.49E+08	1121900	2.1E+08	1436900	15380000	1339500	4983500
Q9HD45	Q9HD45	Transmembrane 9 superfamily member 3	TM9SF3	sp|Q9HD4	6	6	6	1	3	3	6	1	3	3	6	1	3	3	6	14.4	67.887	0	67.026	360840000	391710	24868000	11467000	3.24E+08	898670	3430100	5140400	14150000
O60678	O60678	Protein arginine N-methyltransferase 3	PRMT3	sp|O6067	5	5	5	0	5	1	4	0	5	1	4	0	5	1	4	12.6	59.902	0	15.353	360690000	0	1.82E+08	897900	1.78E+08	0	8716200	0	14615000
Q9UNE7	Q9UNE7	E3 ubiquitin-protein ligase CHIP	STUB1	sp|Q9UNE	7	7	7	2	7	1	4	2	7	1	4	2	7	1	4	24.1	34.856	0	15.249	360080000	3835000	2.44E+08	17287000	95270000	1490300	23812000	0	2664600
O15397	O15397	Importin-8	IPO8	sp|O1539	8	8	8	0	6	1	8	0	6	1	8	0	6	1	8	11.6	119.94	0	28.194	359510000	0	82070000	1671800	2.76E+08	0	7362500	1455500	10881000
O14967	O14967	Calmegin	CLGN	sp|O1496	7	7	7	0	3	0	7	0	3	0	7	0	3	0	7	17	70.038	0	25.486	359490000	0	50203000	0	3.09E+08	0	4972000	0	12173000
Q9UHV9	Q9UHV9	Prefoldin subunit 2	PFDN2	sp|Q9UHV	5	5	5	1	5	4	4	1	5	4	4	1	5	4	4	32.5	16.648	0	34.263	359220000	3154900	2.12E+08	5812900	1.38E+08	3656500	18275000	3483900	6018400
Q9HCN8	Q9HCN8	Stromal cell-derived factor 2-like protein 1	SDF2L1	sp|Q9HCN	2	2	2	0	2	1	2	0	2	1	2	0	2	1	2	19.9	23.598	0	26.538	359200000	0	2.05E+08	2286400	1.52E+08	0	18118000	1847500	5879000
P05166	P05166	Propionyl-CoA carboxylase beta chain, mitochond	PCCB	sp|P05166	3	3	3	0	2	0	3	0	2	0	3	0	2	0	3	8	58.215	0	6.5484	358800000	0	29434000	0	3.29E+08	0	8431100	0	7708900
Q8NHH9	Q8NHH9	Atlastin-2	ATL2	sp|Q8NHH	3	3	3	0	3	0	3	0	3	0	3	0	3	0	3	7.4	66.228	0	20.174	358730000	0	73439000	0	2.85E+08	0	9489000	0	8717200
P49841	P49841	Glycogen synthase kinase-3 beta	GSK3B	sp|P49841	6	6	5	0	4	5	6	0	4	5	6	0	3	5	5	25.7	46.744	0	27.337	358430000	0	46122000	10494000	3.02E+08	0	7194900	7118700	9216400
Q9UJW0	Q9UJW0	Dynactin subunit 4	DCTN4	sp|Q9UJW	5	5	5	0	5	2	4	0	5	2	4	0	5	2	4	21.5	52.337	0	67.986	358390000	0	1.34E+08	4016100	2.2E+08	0	10507000	4749500	8322400
P35658	P35658	Nuclear pore complex protein Nup214	NUP214	sp|P35658	12	12	12	2	4	3	12	2	4	3	12	2	4	3	12	9.4	213.62	0	72.532	358270000	1618100	16846000	3636800	3.36E+08	1376600	2184900	1551700	14259000
Q8TD19	Q8TD19	Serine/threonine-protein kinase Nek9	NEK9	sp|Q8TD1	9	9	9	0	8	0	8	0	8	0	8	0	8	0	8	15.1	107.17	0	28.496	358260000	0	86044000	0	2.72E+08	0	6249200	0	12531000
Q969Z0	Q969Z0	Protein TBRG4	TBRG4	sp|Q969Z	11	11	11	0	9	2	10	0	9	2	10	0	9	2	10	23.5	70.737	0	27.012	358160000	0	1.08E+08	3175600	2.47E+08	0	7805700	2829600	11197000
O95139	O95139	NADH dehydrogenase [ubiquinone] 1 beta subcor	NDUFB6	sp|O9513	3	3	3	0	3	3	3	0	3	3	3	0	3	3	3	27.3	15.489	0	5.5878	358090000	0	1.34E+08	13356000	2.11E+08	0	10980000	8122800	10342000
P84095	P84095	Rho-related GTP-binding protein RhoG	RHOG	sp|P84095	7	7	7	0	4	0	7	0	4	0	7	0	4	0	7	44.5	21.308	0	37.471	357960000	0	66424000	0	2.92E+08	0	6556100	0	11289000
O00471	O00471	Exocyst complex component 5	EXOC5	sp|O00471	10	10	10	0	8	1	10	0	8	1	10	0	8	1	10	17.2	81.852	0	35.164	357890000	0	72808000	1457300	2.84E+08	0	4869100	1692800	12500000
P05204;Q1	P05204;Q1	Non-histone chromosomal protein HMG-17;High	HMGN2;HI	sp|P05204	2	2	2	1	2	2	2	1	2	2	2	1	2	2	2	14.4	9.3926	0	4.5686	357670000	1135600	1.02E+08	5355100	2.5E+08	824230	11571000	2042000	9514000
Q9NVP1	Q9NVP1	ATP-dependent RNA helicase DDX18	DDX18	sp|Q9NVP	4	4	4	0	2	1	4	0	2	1	4	0	2	1	4	7.6	75.406	0	7.3469	356890000	0	1.41E+08	10531000	2.05E+08	0	4344600	18987000	4625200
Q9NVH2	Q9NVH2	Integrator complex subunit 7	INTS7	sp|Q9NVH	5	5	5	0	3	1	5	0	3	1	5	0	3	1	5	5.9	106.83	0	7.8449	356710000	0	97660000	34042000	2.25E+08	0	7623700	24036000	9078600
Q92615	Q92615	La-related protein 4B	LARP4B	sp|Q9261	11	11	11	1	11	5	7	1	11	5	7	1	11	5	7	20.5	80.551	0	26.553	355910000	9713500	2.28E+08	12053000	1.07E+08	0	14600000	11141000	6803700
Q15043	Q15043	Zinc transporter ZIP14	SLC39A14	sp|Q1504	3	3	3	0	1	2	3	0	1	2	3	0	1	2	3	9.1	54.212	0	19.283	354730000	0	4355400	7849500	3.43E+08	0	1814300	3694700	14000000
Q4VC31	Q4VC31	Coiled-coil domain-containing protein 58	CCDC58	sp|Q4VC3	6	6	6	0	6	0	5	0	6	0	5	0	6	0	5	52.8	16.62	0	20.92	354620000	0	1.78E+08	0	1.77E+08	0	15524000	0	7412900
Q86Y82	Q86Y82	Syntaxin-12	STX12	sp|Q86Y8	5	5	5	0	5	0	4	0	5	0	4	0	5	0	4	29	31.642	0	28.952	354440000	0	1.38E+08	0	2.17E+08	0	11831000	0	9217000

Q9BZF1	Q9BZF1	Oxysterol-binding protein-related protein 8	OSBPL8	sp|Q9BZF1	6	6	6	0	6	2	6	0	6	2	6	0	6	2	6	9.9	101.19	0	22.588	354000000	0	97571000	4093100	2.52E+08	0	8229700	3610400	9889000
Q14181	Q14181	DNA polymerase alpha subunit B	POLA2	sp|Q14181	5	5	5	0	5	0	3	0	5	0	3	0	5	0	3	12.2	65.947	0	18.478	353870000	0	1.8E+08	0	1.74E+08	0	14830000	0	8172600
Q9BV44	Q9BV44	THUMP domain-containing protein 3	THUMPD3	sp|Q9BV4	5	5	5	0	2	1	5	0	2	1	5	0	2	1	5	12.8	57.002	0	18.802	353860000	0	27583000	2669600	3.24E+08	0	3764400	2593500	11176000
P63167;Q9	P63167;Q9	Dynein light chain 1, cytoplasmic;Dynein light chai	DYNLL1;DY	sp|P63167	2	2	2	1	2	2	2	1	2	2	2	1	2	2	2	37.1	10.366	0	23.171	353500000	17588000	49024000	7900200	2.79E+08	27922000	2106900	2917900	5744700
Q8NI27	Q8NI27	THO complex subunit 2	THOC2	sp|Q8NI27	10	10	10	0	10	4	5	0	10	4	5	0	10	4	5	6.5	182.77	0	19.901	352960000	0	2E+08	6930100	1.46E+08	0	16335000	6470100	5509700
Q9Y312	Q9Y312	Protein AAR2 homolog	AAR2	sp|Q9Y312	5	5	5	0	5	2	3	0	5	2	3	0	5	2	3	21.4	43.472	0	25.276	352830000	0	91458000	7335500	2.54E+08	0	5475400	5848300	12115000
Q9NRY5	Q9NRY5	Protein FAM114A2	FAM114A2	sp|Q9NRY	6	6	6	0	5	0	5	0	5	0	5	0	5	0	5	18.6	55.468	0	43.147	352800000	0	86239000	0	2.67E+08	0	8904500	0	9660000
Q9BRK5	Q9BRK5	45 kDa calcium-binding protein	SDF4	sp|Q9BRK	5	5	5	0	4	4	5	0	4	4	5	0	4	4	5	21.3	41.806	0	14.766	352710000	0	35022000	16799000	3.01E+08	0	8485900	8449900	9814800
Q9Y3L5	Q9Y3L5	Ras-related protein Rap-2c	RAP2C	sp|Q9Y3L5	3	3	2	0	2	0	3	0	2	0	3	0	1	0	2	25.7	20.745	0	128.56	352440000	0	44728000	0	3.08E+08	0	8212600	0	8385000
O75223	O75223	Gamma-glutamylcyclotransferase	GGCT	sp|O75223	5	5	5	1	4	4	4	1	4	4	4	1	4	4	4	38.3	21.007	0	33.713	352380000	4992400	1.86E+08	48795000	1.12E+08	7259600	20199000	25933000	5427700
P63279	P63279	SUMO-conjugating enzyme UBC9	UBE2I	sp|P63279	4	4	4	0	4	0	4	0	4	0	4	0	4	0	4	22.8	18.007	0	10.026	352000000	0	2.1E+08	0	1.42E+08	0	18834000	0	5533400
Q2TAL8	Q2TAL8	Glutamine-rich protein 1	QRICH1	sp|Q2TAL	7	7	7	0	7	0	6	0	7	0	6	0	7	0	6	16.4	86.435	0	24.804	351510000	0	1.46E+08	0	2.06E+08	0	11174000	0	10144000
Q9NQ50	Q9NQ50	39S ribosomal protein L40, mitochondrial	MRPL40	sp|Q9NQ5	3	3	3	0	3	2	2	0	3	2	2	0	3	2	2	25.7	24.49	0	20.081	351320000	0	1.92E+08	11249000	1.48E+08	0	12840000	6182800	10305000
P24928	P24928	DNA-directed RNA polymerase II subunit RPB1	POLR2A	sp|P24928	14	14	14	2	5	2	14	2	5	2	14	2	5	2	14	12.3	217.17	0	40.883	350190000	1638100	28080000	1779400	3.19E+08	1245100	2079100	1063800	14028000
Q5T8P6	Q5T8P6	RNA-binding protein 26	RBM26	sp|Q5T8P	12	12	11	4	11	5	9	4	11	5	9	3	10	4	8	12.9	113.6	0	28.408	349310000	6202400	1.92E+08	6320100	1.44E+08	6186200	17805000	4490400	4862900
Q9Y6E0	Q9Y6E0	Serine/threonine-protein kinase 24;Serine/threor	STK24	sp|Q9Y6E	10	4	4	1	9	3	10	1	4	1	4	1	4	1	4	21.2	49.307	0	14.511	348100000	4828400	58188000	907360	2.84E+08	8869900	1345600	1232300	8126400
Q01433	Q01433	AMP deaminase 2	AMPD2	sp|Q01433	9	9	9	0	5	7	9	0	5	7	9	0	5	7	9	12.4	100.69	0	26.497	347780000	0	71468000	15920000	2.6E+08	0	5993600	14365000	7161700
Q969G5	Q969G5	Protein kinase C delta-binding protein	PRKCDBP	sp|Q969G	8	8	8	0	1	6	8	0	1	6	8	0	1	6	8	31	27.701	0	33.237	347540000	0	2399700	37286000	3.08E+08	0	1085100	13129000	23714000
P61006;Q9	P61006	Ras-related protein Rab-8A	RAB8A	sp|P61006	6	4	3	0	4	2	6	0	3	1	4	0	3	1	3	35.7	23.668	0	35.242	346850000	0	1.3E+08	1586800	2.16E+08	0	8847900	2929800	9588700
Q7Z4W1	Q7Z4W1	L-xylulose reductase	DCXR	sp|Q7Z4W	6	6	6	0	6	0	4	0	6	0	4	0	6	0	4	31.1	25.913	0	44.618	346600000	0	2.51E+08	0	95521000	0	20236000	0	5824800
Q16880	Q16880	2-hydroxyacylsphingosine 1-beta-galactosyltransf	UGT8	sp|Q1688	4	4	4	0	1	3	4	0	1	3	4	0	1	3	4	8.9	61.437	0	17.443	346440000	0	2772900	7767800	3.36E+08	0	1202600	3648300	14427000
Q9BRJ2	Q9BRJ2	39S ribosomal protein L45, mitochondrial	MRPL45	sp|Q9BRJ2	5	5	5	0	3	3	4	0	3	3	4	0	3	3	4	26.5	35.351	0	13.516	345630000	0	78756000	8351700	2.59E+08	0	9340200	4720700	9125700
Q71RC2	Q71RC2	La-related protein 4	LARP4	sp|Q71RC	6	6	6	1	5	4	6	1	5	4	6	1	5	4	6	14.2	80.595	0	33.377	345160000	1596700	78154000	19095000	2.46E+08	5992700	4693900	12442000	8318900
Q96GC9	Q96GC9	Vacuole membrane protein 1	VMP1	sp|Q96GC	2	2	2	0	2	1	2	0	2	1	2	0	2	1	2	9.1	46.237	0	42.151	344770000	0	88290000	9581300	2.47E+08	0	7655900	7676600	9042800
O43447	O43447	Peptidyl-prolyl cis-trans isomerase H	PPIH	sp|O43447	6	6	6	0	6	1	4	0	6	1	4	0	6	1	4	37.9	19.208	0	26.319	344680000	0	2.34E+08	906550	1.1E+08	0	19055000	1614300	5068000
Q15042	Q15042	Rab3 GTPase-activating protein catalytic subunit	RAB3GAP1	sp|Q15042	10	10	10	0	3	0	10	0	3	0	10	0	3	0	10	16.9	110.52	0	42.028	344660000	0	33671000	0	3.11E+08	0	6788300	0	8969300
A6NHR9	A6NHR9	Structural maintenance of chromosomes flexible	SMCHD1	sp|A6NHR	12	12	12	4	12	11	6	4	12	11	6	4	12	11	6	8.1	226.37	0	52.369	344230000	15755000	1.85E+08	41667000	1.02E+08	29355000	10201000	21659000	3027600
Q8N0X7	Q8N0X7	Spartin	SPG20	sp|Q8N0X	10	10	10	0	10	4	9	0	10	4	9	0	10	4	9	19.1	72.832	0	25.91	344060000	0	1.52E+08	3005300	1.89E+08	0	16822000	1632900	4759700
O95571	O95571	Persulfide dioxygenase ETHE1, mitochondrial	ETHE1	sp|O95571	4	4	4	0	2	0	4	0	2	0	4	0	2	0	4	24.4	27.873	0	25.691	344030000	0	7449000	0	3.37E+08	0	563950	0	13935000
P19404	P19404	NADH dehydrogenase [ubiquinone] flavoprotein	NDUFV2	sp|P19404	4	4	4	0	4	0	4	0	4	0	4	0	4	0	4	21.7	27.391	0	10.852	342880000	0	1.68E+08	0	1.74E+08	0	13981000	0	8038200
Q14919	Q14919	Dr1-associated corepressor	DRAP1	sp|Q1491	4	4	4	0	4	1	3	0	4	1	3	0	4	1	3	26.3	22.35	0	13.155	342740000	0	1.53E+08	1845200	1.88E+08	0	13531000	3236900	5670500
O43432	O43432	Eukaryotic translation initiation factor 4 gamma 3	EIF4G3	sp|O43432	8	6	6	2	7	3	8	1	5	1	6	1	5	1	6	6.6	176.65	0	21.707	342700000	193920	52863000	4108900	2.86E+08	417180	2476400	1574200	14890000
P15407	P15407	Fos-related antigen 1	FOSL1	sp|P15407	2	2	2	1	1	1	2	1	1	1	2	1	1	1	2	11.1	29.413	0	28.027	342520000	65721000	3840600	8446800	2.65E+08	69988000	5023200	5299800	2303200
O60568	O60568	Procollagen-lysine,2-oxoglutarate 5-dioxygenase	PLOD3	sp|O6056	6	6	6	0	4	3	6	0	4	3	6	0	4	3	6	8.1	84.784	0	15.392	342480000	0	27273000	3083400	3.12E+08	0	2115300	2804500	11547000
O14776;Q5	O14776	Transcription elongation regulator 1	TCERG1	sp|O1477	13	13	13	0	11	2	13	0	11	2	13	0	11	2	13	12.8	123.9	0	28.489	342100000	0	1.38E+08	1837000	2.02E+08	0	13572000	3089700	5050300
Q5JRA6	Q5JRA6	Melanoma inhibitory activity protein 3	MIA3	sp|Q5JRA	15	15	15	0	11	0	11	0	11	0	11	0	11	0	11	15.7	213.7	0	42.179	342020000	0	1.14E+08	0	2.28E+08	0	12151000	0	7293100
Q9Y394	Q9Y394	Dehydrogenase/reductase SDR family member 7	DHRS7	sp|Q9Y39	7	7	7	0	4	1	6	0	4	1	6	0	4	1	6	32.2	38.298	0	50.518	341320000	0	48311000	2024900	2.91E+08	0	3700900	3431200	10454000
Q9H936;Q	Q9H936;Q	Mitochondrial glutamate carrier 1;Mitochondrial	SLC25A22;	sp|Q9H93	8	8	8	0	8	6	7	0	8	6	7	0	8	6	7	27.6	34.47	0	20.978	340620000	0	1.07E+08	14758000	2.19E+08	0	6699000	12905000	8740300
Q6UVK1	Q6UVK1	Chondroitin sulfate proteoglycan 4	CSPG4	sp|Q6UVK	13	13	13	1	1	0	13	1	1	0	13	1	1	0	13	9	250.53	0	50.133	340510000	547000	4652300	0	3.35E+08	796650	0	0	13540000
Q69YN2	Q69YN2	CWF19-like protein 1	CWF19L1	sp|Q69YN	10	10	10	1	9	0	10	1	9	0	10	1	9	0	10	19.5	60.618	0	23.794	340410000	211140	1.66E+08	0	1.74E+08	651070	18424000	0	2949500
Q969G3	Q969G3	SWI/SNF-related matrix-associated actin-depende	SMARCE1	sp|Q969G	7	7	7	0	5	2	6	0	5	2	6	0	5	2	6	29.9	46.649	0	22.245	339020000	0	95215000	3564200	2.4E+08	0	7867800	3034900	9765600
O95486	O95486	Protein transport protein Sec24A	SEC24A	sp|O9548	8	8	8	1	5	0	8	1	5	0	8	1	5	0	8	10.7	119.75	0	28.019	338010000	276550	55977000	0	2.82E+08	577410	2608800	0	13612000
Q9H910	Q9H910	Hematological and neurological expressed 1-like	HN1L	sp|Q9H91	6	6	6	0	6	0	5	0	6	0	5	0	6	0	5	51.1	20.063	0	29.609	337480000	0	2.32E+08	0	1.06E+08	0	19820000	0	4946300
Q32P28	Q32P28	Prolyl 3-hydroxylase 1	LEPRE1	sp|Q32P2	7	7	7	0	6	0	6	0	6	0	6	0	6	0	6	12.9	83.393	0	50.302	336350000	0	1.04E+08	0	2.32E+08	0	9033200	0	9711600
P56385	P56385	ATP synthase subunit e, mitochondrial	ATP5I	sp|P56385	4	4	4	0	4	2	3	0	4	2	3	0	4	2	3	42	7.9331	0	6.7693	336080000	0	1.81E+08	1182200	1.54E+08	0	16212000	2365600	4485200
Q3LXA3	Q3LXA3	Bifunctional ATP-dependent dihydroxyacetone ki	DAK	sp|Q3LXA	7	7	7	0	6	0	7	0	6	0	7	0	6	0	7	20.5	58.946	0	21.616	335930000	0	1.02E+08	0	2.34E+08	0	8064500	0	10533000
Q9UBP6	Q9UBP6	tRNA (guanine-N(7)-)-methyltransferase	METTL1	sp|Q9UBP	6	6	6	0	6	0	5	0	6	0	5	0	6	0	5	36.2	31.471	0	19.111	335640000	0	1.74E+08	0	1.61E+08	0	14052000	0	7950200
Q9Y2W2	Q9Y2W2	WW domain-binding protein 11	WBP11	sp|Q9Y2W	8	8	8	1	7	2	7	1	7	2	7	1	7	2	7	17.6	69.997	0	27.712	335590000	540740	1.85E+08	3301800	1.47E+08	1613200	12571000	1100200	9800800
O75323	O75323	Protein NipSnap homolog 2	GBAS	sp|O7532	8	6	6	0	8	4	6	0	6	3	5	0	6	3	5	26.9	33.742	0	24.137	334890000	0	2.24E+08	9632800	1.01E+08	0	16035000	8649600	4843400
Q3MHD2	Q3MHD2	Protein LSM12 homolog	LSM12	sp|Q3MH	3	3	3	0	3	3	3	0	3	3	3	0	3	3	3	18.5	21.701	0	10.039	333910000	0	1.2E+08	24803000	1.89E+08	0	10325000	17147000	7559900
P27707	P27707	Deoxycytidine kinase	DCK	sp|P27707	4	4	4	0	2	0	4	0	2	0	4	0	2	0	4	25.4	30.518	0	21.966	333710000	0	14932000	0	3.19E+08	0	2992700	0	11433000
Q5JTZ9	Q5JTZ9	Alanine--tRNA ligase, mitochondrial	AARS2	sp|Q5JTZ9	12	12	12	1	11	0	8	1	11	0	8	1	11	0	8	19.3	107.34	0	47.297	333550000	525450	1.7E+08	0	1.63E+08	2354400	10081000	0	9795300
Q0VDF9	Q0VDF9	Heat shock 70 kDa protein 14	HSPA14	sp|Q0VDF	6	6	6	0	6	1	6	0	6	1	6	0	6	1	6	15.3	54.794	0	14.133	332490000	0	1.36E+08	3785400	1.93E+08	0	12654000	0	7250600
P82675	P82675	28S ribosomal protein S5, mitochondrial	MRPS5	sp|P82675	4	4	4	0	3	1	4	0	3	1	4	0	3	1	4	12.3	48.006	0	9.862	332360000	0	86676000	2361100	2.43E+08	0	7637900	7361100	4234800
P49406	P49406	39S ribosomal protein L19, mitochondrial	MRPL19	sp|P49406	5	5	5	0	5	4	5	0	5	4	5	0	5	4	5	22.6	33.535	0	15.719	332080000	0	66080000	7405900	2.59E+08	0	6339900	4604400	10492000
Q9Y679	Q9Y679	Ancient ubiquitous protein 1	AUP1	sp|Q9Y67	7	7	7	0	4	2	7	0	4	2	7	0	4	2	7	26.6	45.786	0	30.098	331850000	0	44551000	8963600	2.78E+08	0	5736200	7538900	8122300
Q5JTV8	Q5JTV8	Torsin-1A-interacting protein 1	TOR1AIP1	sp|Q5JTV8	9	9	9	1	7	4	7	1	7	4	7	1	7	4	7	22.3	66.248	0	21.828	331850000	1155100	1.47E+08	8392600	1.75E+08	1821400	16276000	3894000	4983400
P31944	P31944	Caspase-14;Caspase-14 subunit p17, mature form	CASP14	sp|P31944	14	14	14	1	4	14	1	1	4	14	1	1	4	14	1	53.7	27.679	0	56.933	331480000	5863000	43849000	271210000	10550000	1541500	1432100	189020000	419360
P82970	P82970	High mobility group nucleosome-binding domain-	HMGN5	sp|P82970	5	5	5	0	5	0	2	0	5	0	2	0	5	0	2	21.3	31.524	0	54.887	331260000	0	3.08E+08	0	23502000	0	26657000	0	1438600
Q7Z3B4	Q7Z3B4	Nucleoporin p54	NUP54	sp|Q7Z3B	10	10	10	0	9	2	8	0	9	2	8	0	9	2	8	25.6	55.435	0	27.938	330960000	0	1.38E+08	1314400	1.91E+08	0	10555000	964240	9418100
Q8NF37	Q8NF37	Lysophosphatidylcholine acyltransferase 1	LPCAT1	sp|Q8NF3	4	4	4	0	4	0	3	0	4	0	3	0	4	0	3	9.6	59.151	0	23.106	330770000	0	40072000	0	2.91E+08	0	8906000	0	6581500
Q13478	Q13478	Interleukin-18 receptor 1	IL18R1	sp|Q1347	1	1	1	0	0	0	1	0	0	0	1	0	0	0	1	1.3	62.303	0.006413	1.7245	330730000	0	0	0	3.31E+08	0	0	0	13601000
Q9UHQ9	Q9UHQ9	NADH-cytochrome b5 reductase 1	CYB5R1	sp|Q9UH	6	6	6	0	4	0	6	0	4	0	6	0	4	0	6	24.9	34.094	0	23.915	330250000	0	60716000	0	2.7E+08	0	7703700	0	8733300
Q96SB4	Q96SB4	SRSF protein kinase 1	SRPK1	sp|Q96SB	7	7	6	4	4	4	5	4	4	4	5	3	3	3	4	16.9	74.324	0	17.416	330160000	1.41E+08	65467000	15634000	1.08E+08	1.25E+08	13927000	13600000	9824100
Q9Y547	Q9Y547	Intraflagellar transport protein 25 homolog	HSPB11	sp|Q9Y54	2	2	2	0	2	1	2	0	2	1	2	0	2	1	2	25.7	16.297	0	26.853	330070000	0	1.02E+08	1225600	2.27E+08	0	8424500	1469600	9240400
Q92947	Q92947	Glutaryl-CoA dehydrogenase, mitochondrial	GCDH	sp|Q9294	6	6	6	0	5	1	5	0	5	1	5	0	5	1	5	26.3	48.127	0	58.288	330040000	0	1.2E+08	970820	2.09E+08	0	10585000	1375600	7890500
Q9BRJ6	Q9BRJ6	Uncharacterized protein C7orf50	C7orf50	sp|Q9BRJ6	5	5	5	0	4	5	4	0	4	5	4	0	4	5	4	39.7	22.083	0	16.767	330040000	0	1.11E+08	64783000	1.54E+08	0	9027200	44022000	6618500
Q14155	Q14155	Rho guanine nucleotide exchange factor 7	ARHGEF7	sp|Q1415	6	6	5	0	4	1	5	0	4	1	5	0	3	1	4	12.5	90.011	0	25.223	329780000	0	52248000	2955900	2.75E+08	0	5954200	0	9520700
P00846	P00846	ATP synthase subunit a	MT-ATP6	sp|P00846	2	2	2	1	2	1	2	1	2	1	2	1	2	1	2	8.4	24.817	0	4.5554	329710000	507220	1.5E+08	4035000	1.75E+08	544780	12716000	2912300	7468500
P49902	P49902	Cytosolic purine 5-nucleotidase	NT5C2	sp|P49902	4	4	4	0	2	0	4	0	2	0	4	0	2	0	4	9.4	64.969	0	13.379	329010000	0	14838000	0	3.14E+08	0	5578800	0	8649800
Q9Y5A9	Q9Y5A9	YTH domain-containing family protein 2	YTHDF2	sp|Q9Y5A	9	7	6	1	8	7	9	1	6	5	7	1	6	5	6	17.6	62.333	0	25.562	328710000	3232100	74809000	29659000	2.21E+08	4007900	2092100	26962000	5784400
Q96TA2	Q96TA2	ATP-dependent zinc metalloprotease YME1L1	YME1L1	sp|Q96TA	8	8	8	0	6	8	7	0	6	8	7	0	6	8	7	15.7	86.454	0	35.011	328400000	0	63683000	26772000	2.38E+08	0	7892200	16368000	9130200
Q9UEW8	Q9UEW8	STE20/SPS1-related proline-alanine-rich protein k	STK39	sp|Q9UEW	6	6	6	0	3	2	6	0	3	2	6	0	3	2	6	20.2	59.473	0	36.211	327540000	0	26049000	5452300	2.96E+08	0	3719500	5226000	9189600
Q8TC07	Q8TC07	TBC1 domain family member 15	TBC1D15	sp|Q8TC0	6	6	6	0	6	2	5	0	6	2	5	0	6	2	5	11.7	79.49	0	37.517	327520000	0	1.82E+08	4205900	1.41E+08	0	19634000	1303500	3760300
Q05048	Q05048	Cleavage stimulation factor subunit 1	CSTF1	sp|Q0504	6	6	6	0	5	2	6	0	5	2	6	0	5	2	6	20.2	48.357	0	28.772	327190000	0	79915000	4187800	2.43E+08	0	6189000	4767200	8899300
P28799	P28799	Granulins;Acrogranin;Paragranulin;Granulin-1;Gr	GRN	sp|P28799	9	9	9	3	2	9	4	3	2	9	4	3	2	9	4	14	63.544	0	20.969	326620000	5890100	4956900	283830000	31942000	1843900	336530	195490000	706150
P49366	P49366	Deoxyhypusine synthase	DHPS	sp|P49366	5	5	5	0	5	0	5	0	5	0	5	0	5	0	5	23.3	40.97	0	48.211	326590000	0	82221000	0	2.44E+08	0	6868000	0	10429000
O15126	O15126	Secretory carrier-associated membrane protein 1	SCAMP1	sp|O1512	5	5	5	0	5	0	4	0	5	0	4	0	5	0	4	29.6	37.92	0	31.534	326310000	0	1.18E+08	0	2.09E+08	0	9010900	0	9948500
Q9BYT8	Q9BYT8	Neurolysin, mitochondrial	NLN	sp|Q9BYT	6	6	6	0	6	0	6	0	6	0	6	0	6	0	6	11.5	80.651	0	22.633	325950000	0	1.2E+08	0	2.06E+08	0	9545900	0	9489100
Q6L8Q7	Q6L8Q7	2,5-phosphodiesterase 12	PDE12	sp|Q6L8Q	6	6	6	0	5	0	4	0	5	0	4	0	5	0	4	16.9	67.351	0	42.785	325190000	0	1.58E+08	0	1.68E+08	0	11543000	0	9237500
P80303	P80303	Nucleobindin-2;Nesfatin-1	NUCB2	sp|P80303	7	7	7	0	6	0	5	0	6	0	5	0	6	0	5	25.7	50.222	0	30.113	324740000	0	1.37E+08	0	1.88E+08	0	14583000	0	5199300
Q9NZT2	Q9NZT2	Opioid growth factor receptor	OGFR	sp|Q9NZT	12	12	12	0	8	0	11	0	8	0	11	0	8	0	11	33.5	73.324	0	30.868	324590000	0	61654000	0	2.63E+08	0	6794900	0	9453500
P62877	P62877	E3 ubiquitin-protein ligase RBX1;E3 ubiquitin-prot	RBX1	sp|P62877	2	2	2	0	2	2	2	0	2	2	2	0	2	2	2	17.6	12.274	0	13.986	324230000	0	1.4E+08	1504900	1.82E+08	0	10629000	1121900	7427400
Q15386	Q15386	Ubiquitin-protein ligase E3C	UBE3C	sp|Q1538	8	8	8	0	4	2	8	0	4	2	8	0	4	2	8	8.4	123.92	0	36.532	323820000	0	19579000	2417400	3.02E+08	0	2538400	3884100	9340600
Q14684	Q14684	Ribosomal RNA processing protein 1 homolog B	RRP1B	sp|Q1468	11	11	11	2	10	10	9	2	10	10	9	2	10	10	9	24.1	84.427	0	26.626	323790000	3373500	1.17E+08	55260000	1.49E+08	4641600	13887000	32021000	6342100
P22612	P22612	cAMP-dependent protein kinase catalytic subunit	PRKACG	sp|P22612	1	1	1	0	1	1	1	0	1	1	1	0	1	1	1	6.8	40.434	0	19.186	323640000	0	65920000	3589300	2.54E+08	0	5810800	2412000	10451000
Q8IYI6	Q8IYI6	Exocyst complex component 8	EXOC8	sp|Q8IYI6|	7	7	7	0	6	0	7	0	6	0	7	0	6	0	7	11.9	81.798	0	22.105	322950000	0	83861000	0	2.39E+08	0	9271300	0	7953800
Q9UQ88;P	Q9UQ88;P	Cyclin-dependent kinase 11A;Cyclin-dependent ki	CDK11A;C	sp|Q9UQ8	8	8	8	0	7	3	8	0	7	3	8	0	7	3	8	10.9	91.361	0	20.746	322870000	0	94900000	5594700	2.22E+08	0	5537300	9885100	5847700
P49642	P49642	DNA primase small subunit	PRIM1	sp|P49642	5	5	5	0	5	0	4	0	5	0	4	0	5	0	4	14.3	49.901	0	10.606	322510000	0	1.33E+08	0	1.89E+08	0	12817000	0	6709400

Q13438	Q13438	Protein OS-9	OS9	sp|Q1343	4	4	4	0	3	2	4	0	3	2	4	0	3	2	4	6.6	75.561	0	5.8272	322080000	0	15555000	1479300	3.05E+08	0	3310800	4142300	7457400
Q9BYD6	Q9BYD6	39S ribosomal protein L1, mitochondrial	MRPL1	sp|Q9BYD	7	7	7	1	7	5	6	1	7	5	6	1	7	5	6	26.5	36.908	0	15.573	321840000	1686100	1.72E+08	9320500	1.38E+08	2903800	13375000	5742800	5871500
Q9UBM7	Q9UBM7	7-dehydrocholesterol reductase	DHCR7	sp|Q9UBM	6	6	6	0	5	3	6	0	5	3	6	0	5	3	6	13.5	54.489	0	23.612	321540000	0	1.36E+08	4816200	1.81E+08	0	9606000	6573400	6468800
O95487	O95487	Protein transport protein Sec24B	SEC24B	sp|O95487	9	9	9	0	6	0	9	0	6	0	9	0	6	0	9	11.8	137.42	0	87.373	321350000	0	29449000	0	2.92E+08	0	4225600	0	10375000
Q08380	Q08380	Galectin-3-binding protein	LGALS3BP	sp|Q0838	7	7	7	0	7	6	7	0	7	6	7	0	7	6	7	15.2	65.33	0	21.059	321180000	0	56302000	18281000	2.47E+08	0	4495900	14610000	8283300
Q6NXE6	Q6NXE6	Armadillo repeat-containing protein 6	ARMC6	sp|Q6NXE	5	5	5	0	3	1	5	0	3	1	5	0	3	1	5	17.8	54.141	0	49.715	319360000	0	64879000	546550	2.54E+08	0	8212200	0	7949800
Q9H2P9	Q9H2P9	Diphthine synthase	DPH5	sp|Q9H2P	5	5	5	1	5	0	3	1	5	0	3	1	5	0	3	27.7	31.651	0	49.616	319050000	790430	1.09E+08	0	2.09E+08	3255500	8748900	0	7010600
O15270	O15270	Serine palmitoyltransferase 2	SPTLC2	sp|O1527	9	9	9	1	5	2	8	1	5	2	8	1	5	2	8	29.4	62.924	0	62.427	318320000	3804200	67569000	6091800	2.41E+08	4398000	4709900	4853700	9797700
Q13459;B2	Q13459	Unconventional myosin-IXb	MYO9B	sp|Q1345	18	18	18	0	3	6	18	0	3	6	18	0	3	6	18	12.3	243.4	0	32.894	318310000	0	16664000	4776100	2.97E+08	0	1427300	7237100	8222800
P98082	P98082	Disabled homolog 2	DAB2	sp|P98082	9	9	9	0	0	0	9	0	0	0	9	0	0	0	9	20.4	82.447	0	23.909	318230000	0	0	0	3.18E+08	0	0	0	13087000
Q96B97	Q96B97	SH3 domain-containing kinase-binding protein 1	SH3KBP1	sp|Q96B9	4	4	4	0	0	0	4	0	0	0	4	0	0	0	4	9.5	73.125	0	21.918	318010000	0	0	0	3.18E+08	0	0	0	13078000
Q9HD20	Q9HD20	Manganese-transporting ATPase 13A1	ATP13A1	sp|Q9HD2	11	11	11	0	6	1	10	0	6	1	10	0	6	1	10	11.5	132.95	0	31.433	317340000	0	46450000	44008000	2.27E+08	0	4942800	0	8482500
Q15643	Q15643	Thyroid receptor-interacting protein 11	TRIP11	sp|Q1564	16	16	16	0	3	3	16	0	3	3	16	0	3	3	16	11.8	227.58	0	41.693	316530000	0	11601000	4723600	3E+08	0	1385300	2401500	12756000
O00401	O00401	Neural Wiskott-Aldrich syndrome protein	WASL	sp|O00401	7	7	7	0	5	0	6	0	5	0	6	0	5	0	6	25.9	54.826	0	65.661	316260000	0	38039000	0	2.78E+08	0	4443700	0	10352000
Q12846	Q12846	Syntaxin-4	STX4	sp|Q1284	6	6	6	0	4	0	6	0	4	0	6	0	4	0	6	38.4	34.18	0	45.492	315240000	0	52837000	0	2.62E+08	0	6155600	0	9293500
O00567	O00567	Nucleolar protein 56	NOP56	sp|O0056	9	9	9	0	9	6	8	0	9	6	8	0	9	6	8	25.1	66.049	0	50.286	314910000	0	81960000	30089000	2.03E+08	0	5400300	23372000	7015000
Q16799	Q16799	Reticulon-1	RTN1	sp|Q1679	10	10	10	1	1	2	10	1	1	2	10	1	1	2	10	18	83.617	0	27.551	313620000	1001100	70617000	1665900	2.4E+08	856030	7486600	1019600	8867000
Q9Y2X7	Q9Y2X7	ARF GTPase-activating protein GIT1	GIT1	sp|Q9Y2X	4	3	3	0	4	1	4	0	3	1	3	0	3	1	3	9.1	84.34	0	8.1039	313050000	0	66900000	3624500	2.43E+08	0	5314600	2944600	10048000
Q9HD33	Q9HD33	39S ribosomal protein L47, mitochondrial	MRPL47	sp|Q9HD3	6	6	6	0	6	5	5	0	6	5	5	0	6	5	5	20.8	29.45	0	16.401	312860000	0	1.33E+08	16204000	1.64E+08	0	9545300	12497000	7298000
Q13131	Q13131	5-AMP-activated protein kinase catalytic subunit a	PRKAA1	sp|Q13131	6	6	5	0	3	3	6	0	3	3	6	0	2	3	5	14.5	64.009	0	69.225	312440000	0	55558000	5055600	2.52E+08	0	5179500	4982700	8489000
Q13510	Q13510	Acid ceramidase;Acid ceramidase subunit alpha;A	ASAH1	sp|Q1351	2	2	2	0	2	2	2	0	2	2	2	0	2	2	2	8.6	44.659	0	21.069	312150000	0	42741000	25495000	2.44E+08	0	3865600	18436000	8629700
Q68E01	Q68E01	Integrator complex subunit 3	INTS3	sp|Q68E01	5	5	5	0	4	1	5	0	4	1	5	0	4	1	5	10.3	118.07	0	46.548	311470000	0	42047000	3058000	2.66E+08	0	4347800	2238300	10130000
O43674	O43674	NADH dehydrogenase [ubiquinone] 1 beta subcor	NDUFB5	sp|O4367	4	4	4	0	4	1	3	0	4	1	3	0	4	1	3	19	21.75	0	11.698	311020000	0	97114000	7620600	2.06E+08	0	7329700	6910100	7925300
Q96QD8	Q96QD8	Sodium-coupled neutral amino acid transporter 2	SLC38A2	sp|Q96QD	4	4	4	0	2	1	4	0	2	1	4	0	2	1	4	15.4	56.025	0	17.934	310880000	0	89683000	3358700	2.18E+08	0	7759100	2575100	8787000
O75340	O75340	Programmed cell death protein 6	PDCD6	sp|O7534	4	4	4	0	3	2	4	0	3	2	4	0	3	2	4	21.5	21.868	0	11.816	310280000	0	92182000	4948400	2.13E+08	0	8627800	5069300	6520000
Q8TF05	Q8TF05	Serine/threonine-protein phosphatase 4 regulato	PPP4R1	sp|Q8TF0	9	9	9	1	8	0	9	1	8	0	9	1	8	0	9	12.2	107	0	23.949	309600000	664020	49183000	0	2.6E+08	0	5690000	0	9328200
Q4G0J3	Q4G0J3	La-related protein 7	LARP7	sp|Q4G0J3	10	10	10	0	10	6	6	0	10	6	6	0	10	6	6	20.4	66.898	0	24.423	308580000	0	1.31E+08	23155000	1.55E+08	0	10486000	16678000	6272800
Q9Y512	Q9Y512	Sorting and assembly machinery component 50 h	SAMM50	sp|Q9Y51	7	7	7	0	7	2	6	0	7	2	6	0	7	2	6	23	51.976	0	15.919	308540000	0	1.51E+08	3310700	1.54E+08	0	13936000	2671100	4922900
Q9NQ88	Q9NQ88	Fructose-2,6-bisphosphatase TIGAR	TIGAR	sp|Q9NQ8	3	3	3	0	3	0	3	0	3	0	3	0	3	0	3	13	30.062	0	12.599	308080000	0	1.21E+08	0	1.87E+08	0	10218000	0	8132400
Q9H9T3	Q9H9T3	Elongator complex protein 3	ELP3	sp|Q9H9T	5	5	5	0	3	0	5	0	3	0	5	0	3	0	5	14.4	62.258	0	27.478	307860000	0	48830000	0	2.59E+08	0	5708900	0	9248000
P50851;Q8	P50851	Lipopolysaccharide-responsive and beige-like anc	LRBA	sp|P50851	18	18	18	2	15	2	16	2	15	2	16	2	15	2	16	8.9	319.1	0	44.812	307710000	7091100	90913000	1500300	2.08E+08	4418700	11417000	686250	8153700
P14923	P14923	Junction plakoglobin	JUP	sp|P14923	13	11	11	0	11	13	7	0	9	11	5	0	9	11	5	20.8	81.744	0	33.27	305790000	0	94695000	191430000	19667000	0	3306900	130680000	1244700
Q8IXB1	Q8IXB1	DnaJ homolog subfamily C member 10	DNAJC10	sp|Q8IXB1	8	8	8	2	3	3	8	2	3	3	8	2	3	3	8	14	91.079	0	30.32	305500000	8260900	32471000	14118000	2.51E+08	12800000	2484100	9720000	5914300
P11047;Q9	P11047	Laminin subunit gamma-1	LAMC1	sp|P11047	11	11	11	1	5	1	11	1	5	1	11	1	5	1	11	9.3	177.6	0	36.771	305470000	34323000	58203000	1596000	2.11E+08	21763000	9625500	3847100	13982000
Q9Y4P1	Q9Y4P1	Cysteine protease ATG4B	ATG4B	sp|Q9Y4P1	4	4	4	1	3	1	3	1	3	1	3	1	3	1	3	17.6	44.294	0	52.792	305210000	227850	59649000	7462000	2.38E+08	2879500	6578800	3969700	6854800
Q9Y496	Q9Y496	Kinesin-like protein KIF3A	KIF3A	sp|Q9Y49	10	10	8	1	8	1	9	1	8	1	9	1	6	1	7	14.4	80.04	0	22.908	304400000	907440	48246000	1056000	2.54E+08	0	3913500	4991000	6511600
Q9BW91	Q9BW91	ADP-ribose pyrophosphatase, mitochondrial	NUDT9	sp|Q9BW	6	6	6	0	4	0	5	0	4	0	5	0	4	0	5	30	39.125	0	31.245	304270000	0	53618000	0	2.51E+08	0	5745500	0	9289000
Q13330	Q13330	Metastasis-associated protein MTA1	MTA1	sp|Q1333	10	10	9	1	8	4	8	1	8	4	8	1	7	4	8	18.6	80.785	0	22.841	304200000	172860	1.05E+08	6942800	1.92E+08	802230	5707500	3710000	11792000
Q86U38	Q86U38	Nucleolar protein 9	NOP9	sp|Q86U3	5	5	5	0	0	3	5	0	0	3	5	0	0	3	5	9.9	69.437	0	11.923	303900000	0	0	9668700	2.94E+08	0	0	10860000	7738300
Q9H832	Q9H832	Ubiquitin-conjugating enzyme E2 Z	UBE2Z	sp|Q9H83	6	6	6	0	3	0	6	0	3	0	6	0	3	0	6	16.9	38.21	0	27.753	303490000	0	55488000	0	2.48E+08	0	6240400	0	8850200
Q9UPN7	Q9UPN7	Serine/threonine-protein phosphatase 6 regulato	PPP6R1	sp|Q9UPN	6	6	6	0	4	2	6	0	4	2	6	0	4	2	6	14.5	96.723	0	50.913	303470000	0	48479000	3107000	2.52E+08	0	3927800	1764300	11028000
Q9NVI1	Q9NVI1	Fanconi anemia group I protein	FANCI	sp|Q9NVI1	12	12	12	0	12	1	7	0	12	1	7	0	12	1	7	12.4	149.32	0	24.635	303170000	0	2.15E+08	734360	87532000	0	15618000	0	6925200
Q9UI10	Q9UI10	Translation initiation factor eIF-2B subunit delta	EIF2B4	sp|Q9UI10	6	6	6	0	2	4	6	0	2	4	6	0	2	4	6	16.6	57.557	0	27.311	302810000	0	26920000	7540300	2.68E+08	0	5397700	6174300	6904000
Q99848	Q99848	Probable rRNA-processing protein EBP2	EBNA1BP2	sp|Q9984	9	9	9	0	7	9	7	0	7	9	7	0	7	9	7	36.3	34.852	0	19.783	302800000	0	91508000	69451000	1.42E+08	0	8286900	42108000	5795000
Q9NPA0	Q9NPA0	ER membrane protein complex subunit 7	EMC7	sp|Q9NPA	3	3	3	0	3	0	3	0	3	0	3	0	3	0	3	19.4	26.47	0	25.786	302710000	0	82621000	0	2.2E+08	0	6680800	0	9653500
Q9BSF4	Q9BSF4	Uncharacterized protein C19orf52	C19orf52	sp|Q9BSF	4	4	4	0	2	1	4	0	2	1	4	0	2	1	4	23.8	29.233	0	29.565	302400000	0	55010000	654410	2.47E+08	0	3041000	756070	11639000
O60826	O60826	Coiled-coil domain-containing protein 22	CCDC22	sp|O6082	7	7	7	0	7	0	6	0	7	0	6	0	7	0	6	17.4	70.755	0	20.122	302120000	0	1E+08	0	2.02E+08	0	9363600	0	7767100
Q14956	Q14956	Transmembrane glycoprotein NMB	GPNMB	sp|Q1495	3	3	3	0	0	2	3	0	0	2	3	0	0	2	3	7.2	63.922	0	25.149	301740000	0	0	26561000	2.75E+08	0	0	17891000	11275000
Q8N3C0	Q8N3C0	Activating signal cointegrator 1 complex subunit 3	ASCC3	sp|Q8N3C	14	14	14	1	8	7	11	1	8	7	11	1	8	7	11	8.7	251.46	0	29.699	301510000	1587500	86687000	17863000	1.95E+08	0	8354300	12063000	7262400
Q96JJ3;Q9	Q96JJ3	Engulfment and cell motility protein 2	ELMO2	sp|Q96JJ3	9	9	9	0	4	2	9	0	4	2	9	0	4	2	9	21.1	82.614	0	37.473	301460000	0	29972000	2283700	2.69E+08	0	3078200	2047100	10123000
Q8WUM0	Q8WUM0	Nuclear pore complex protein Nup133	NUP133	sp|Q8WU	7	7	7	0	5	1	6	0	5	1	6	0	5	1	6	9.9	128.98	0	51.727	301040000	0	54330000	2966100	2.44E+08	0	4234800	2527900	10044000
P46934	P46934	E3 ubiquitin-protein ligase NEDD4	NEDD4	sp|P46934	8	8	6	0	0	1	8	0	0	1	8	0	0	1	6	8.6	149.11	0	35.79	300400000	0	0	18411000	2.82E+08	0	0	12372000	11597000
Q9GZR7	Q9GZR7	ATP-dependent RNA helicase DDX24	DDX24	sp|Q9GZR	14	14	14	1	2	10	12	1	2	10	12	1	2	10	12	26.7	96.331	0	35.301	299840000	0	11938000	57611000	2.3E+08	0	5511800	24896000	16943000
Q96RQ3	Q96RQ3	Methylcrotonoyl-CoA carboxylase subunit alpha,	MCCC1	sp|Q96RQ	10	10	10	0	8	0	9	0	8	0	9	0	8	0	9	26.1	80.472	0	49.797	298770000	0	88613000	0	2.1E+08	0	8453700	0	8000300
Q9UKX7	Q9UKX7	Nuclear pore complex protein Nup50	NUP50	sp|Q9UKX	9	9	9	1	9	4	5	1	9	4	5	1	9	4	5	28	50.144	0	23.25	298380000	2492200	1.47E+08	14800000	1.34E+08	8172600	7630400	10766000	3961200
Q13144	Q13144	Translation initiation factor eIF-2B subunit epsilor	EIF2B5	sp|Q1314	5	5	5	0	5	2	5	0	5	2	5	0	5	2	5	10.3	80.379	0	13.198	298330000	0	1.08E+08	7401000	1.82E+08	0	8924000	6294900	6820600
O94760;O	O94760	N(G),N(G)-dimethylarginine dimethylaminohydro	DDAH1	sp|O9476	6	6	6	0	6	0	0	0	6	0	0	0	6	0	0	20	31.121	0	14.863	298310000	0	2.98E+08	0	0	0	26295000	0	0
Q9BY32	Q9BY32	Inosine triphosphate pyrophosphatase	ITPA	sp|Q9BY3	4	4	4	0	4	1	3	0	4	1	3	0	4	1	3	21.1	21.445	0	18.375	297250000	0	1.81E+08	1095100	1.15E+08	0	15172000	1816800	4454300
Q13257	Q13257	Mitotic spindle assembly checkpoint protein MAD	MAD2L1	sp|Q13257	6	6	6	0	6	1	6	0	6	1	6	0	6	1	6	28.3	23.51	0	14.7	296840000	0	1.52E+08	1498300	1.44E+08	0	13674000	2554100	4049700
P32455;Q	P32455	Interferon-induced guanylate-binding protein 1	GBP1	sp|P32455	6	6	4	0	1	1	6	0	1	1	6	0	1	1	4	12.8	67.93	0	23.779	296530000	0	932230	3687900	2.92E+08	0	0	2511300	11792000
Q5VIR6	Q5VIR6	Vacuolar protein sorting-associated protein 53 ho	VPS53	sp|Q5VIR6	8	8	8	0	5	2	8	0	5	2	8	0	5	2	8	15.9	94.404	0	36.147	296350000	0	45610000	4562800	2.46E+08	0	4904000	4932600	7374500
Q9NU22	Q9NU22	Midasin	MDN1	sp|Q9NU2	19	19	19	2	19	2	7	2	19	2	7	2	19	2	7	4.3	632.81	0	48.618	295710000	5999700	2.13E+08	7294700	69692000	9206900	16862000	3761900	2688100
Q92922	Q92922	SWI/SNF complex subunit SMARCC1	SMARCC1	sp|Q9292	11	7	7	0	10	2	7	0	7	1	4	0	7	1	4	16.4	122.87	0	42.418	295370000	0	98338000	233150	1.97E+08	0	12825000	624170	3469500
P29373;P2	P29373	Cellular retinoic acid-binding protein 2	CRABP2	sp|P29373	5	5	5	0	0	2	5	0	0	2	5	0	0	2	5	29	15.693	0	11.032	295050000	0	0	1127900	2.94E+08	0	0	2325100	10520000
Q92747	Q92747	Actin-related protein 2/3 complex subunit 1A	ARPC1A	sp|Q92747	6	6	6	0	4	0	6	0	4	0	6	0	4	0	6	22.2	41.569	0	19.12	294980000	0	36254000	0	2.59E+08	0	4972400	0	8863800
Q13451	Q13451	Peptidyl-prolyl cis-trans isomerase FKBP5;Peptidy	FKBP5	sp|Q13451	11	11	11	0	11	0	4	0	11	0	4	0	11	0	4	28.9	51.212	0	24.174	294790000	0	2.36E+08	0	58448000	0	19705000	0	3531700
Q96PZ0	Q96PZ0	Pseudouridylate synthase 7 homolog	PUS7	sp|Q96PZ	7	7	7	0	7	0	3	0	7	0	3	0	7	0	3	18.8	75.035	0	29.368	294240000	0	2.53E+08	0	40826000	0	17212000	0	6805500
Q9UKK3	Q9UKK3	Poly [ADP-ribose] polymerase 4	PARP4	sp|Q9UKK	8	8	8	0	1	4	8	0	1	4	8	0	1	4	8	5.5	192.59	0	25.049	294110000	0	33663000	4864800	2.56E+08	0	2891700	3529500	10326000
Q16706	Q16706	Alpha-mannosidase 2	MAN2A1	sp|Q1670	7	7	7	0	1	0	7	0	1	0	7	0	1	0	7	6.9	131.14	0	63.86	293320000	0	0	0	2.93E+08	0	0	0	12063000
Q86TB9	Q86TB9	Protein PAT1 homolog 1	PATL1	sp|Q86TB	7	7	7	0	3	3	7	0	3	3	7	0	3	3	7	14.5	86.849	0	39.049	293090000	0	72326000	6108600	2.15E+08	0	7328700	6397600	5582000
Q9HBM1	Q9HBM1	Kinetochore protein Spc25	SPC25	sp|Q9HBM	4	4	4	0	3	1	4	0	3	1	4	0	3	1	4	23.2	26.152	0	48.805	293010000	0	96423000	1237800	1.95E+08	0	7406900	0	9126600
Q9BWU0	Q9BWU0	Kanadaptin	SLC4A1AP	sp|Q9BWU	8	8	8	2	6	3	7	2	6	3	7	2	6	3	7	11.1	88.813	0	33.834	292880000	11612000	1.28E+08	6109100	1.47E+08	18570000	3694300	5958700	4835600
Q9ULC3	Q9ULC3	Ras-related protein Rab-23	RAB23	sp|Q9ULC	8	8	8	0	5	0	7	0	5	0	7	0	5	0	7	38.4	26.659	0	30.484	291190000	0	61113000	0	2.3E+08	0	5658600	0	9190600
Q9NQ48	Q9NQ48	Leucine zipper transcription factor-like protein 1	LZTFL1	sp|Q9NQ4	6	6	6	1	6	1	4	1	6	1	4	1	6	1	4	26.8	34.592	0	29.135	291170000	20817000	1.42E+08	2337500	1.26E+08	14704000	14009000	4039100	7327700
Q969T9	Q969T9	WW domain-binding protein 2	WBP2	sp|Q969T	6	6	6	0	4	0	6	0	4	0	6	0	4	0	6	33	28.087	0	14.078	291160000	0	68868000	0	2.22E+08	0	7464900	0	7747700
Q658Y4;P	Q658Y4	Protein FAM91A1	FAM91A1	sp|Q658Y	6	6	6	0	1	2	6	0	1	2	6	0	1	2	6	14.4	93.908	0	28.295	291070000	0	3977900	8800900	2.78E+08	0	897080	5151800	11661000
Q96PD2	Q96PD2	Discoidin, CUB and LCCL domain-containing prote	DCBLD2	sp|Q96PD	4	4	4	0	0	0	4	0	0	0	4	0	0	0	4	8.4	85.034	0	34.209	290810000	0	0	0	2.91E+08	0	0	0	11960000
O43172	O43172	U4/U6 small nuclear ribonucleoprotein Prp4	PRPF4	sp|O4317	6	6	6	1	5	1	6	1	5	1	6	1	5	1	6	17.4	58.449	0	20.274	290800000	1252500	1.02E+08	2792800	1.85E+08	3015800	7373300	2880300	6444900
Q9GZP4	Q9GZP4	PITH domain-containing protein 1	PITHD1	sp|Q9GZP	5	5	5	0	5	1	5	0	5	1	5	0	5	1	5	37.4	24.178	0	11.153	290560000	0	87934000	3739500	1.99E+08	0	6151000	4624300	7668200
O43464;P8	O43464	Serine protease HTRA2, mitochondrial	HTRA2	sp|O4346	4	4	4	1	4	2	4	1	4	2	4	1	4	2	4	12.9	48.84	0	9.5483	289920000	61944000	76849000	10469000	1.41E+08	67561000	2735400	9376500	1864900
P53985	P53985	Monocarboxylate transporter 1	SLC16A1	sp|P53985	2	2	2	0	2	2	1	0	2	2	1	0	2	2	1	5.6	53.944	0	7.0336	289720000	0	1.98E+08	4810600	87288000	0	14235000	4596700	5410600
Q8NEZ5	Q8NEZ5	F-box only protein 22	FBXO22	sp|Q8NEZ	6	6	6	2	5	3	3	2	5	3	3	2	5	3	3	18.4	44.508	0	28.187	289590000	6096700	1.32E+08	9422100	1.42E+08	9464300	7422400	6779300	6248600
O75909	O75909	Cyclin-K	CCNK	sp|O7590	3	3	3	0	3	2	3	0	3	2	3	0	3	2	3	6.7	64.239	0	20.576	289580000	0	91767000	1149400	1.97E+08	0	5624800	4486600	6838100
P11802;Q	P11802	Cyclin-dependent kinase 4	CDK4	sp|P11802	6	5	5	0	5	2	6	0	4	1	5	0	4	1	5	27.7	33.729	0	15.56	289160000	0	1.1E+08	1369400	1.78E+08	0	9794700	1541900	6581300
P49585;Q	P49585	Choline-phosphate cytidylyltransferase A	PCYT1A	sp|P49585	7	7	7	0	4	5	7	0	4	5	7	0	4	5	7	24.5	41.731	0	21.854	289150000	0	34638000	29421000	2.25E+08	0	3554300	18920000	9606200
O75175	O75175	CCR4-NOT transcription complex subunit 3	CNOT3	sp|O7517	8	8	8	0	7	6	7	0	7	6	7	0	7	6	7	14.9	81.871	0	23.701	289130000	0	96277000	24554000	1.68E+08	0	6776800	17766000	6156600
Q96A49	Q96A49	Synapse-associated protein 1	SYAP1	sp|Q96A4	5	5	5	0	5	0	5	0	5	0	5	0	5	0	5	22.4	39.933	0	38.233	288860000	0	57530000	0	2.31E+08	0	5855200	0	8729400
Q8N684	Q8N684	Cleavage and polyadenylation specificity factor su	CPSF7	sp|Q8N68	8	8	8	3	5	5	5	3	5	5	5	3	5	5	5	21.2	52.049	0	24.875	288720000	66087000	1.28E+08	11585000	83334000	56000000	14818000	9869700	7869400
O43156	O43156	TELO2-interacting protein 1 homolog	TTI1	sp|O4315	6	6	6	0	2	1	6	0	2	1	6	0	2	1	6	9.2	122.07	0	66.612	288310000	0	15215000	1329400	2.72E+08	0	1039900	936720	11434000

Q92530	Q92530	Proteasome inhibitor PI31 subunit	PSMF1	sp|Q9253(	3	3	3	0	2	0	2	0	2	0	2	0	2	0	2	14.4	29.816	0	9.9497	288040000	0	1.16E+08	0	1.72E+08	0	7120900	0	10189000
Q3YEC7	Q3YEC7	Rab-like protein 6	RABL6	sp|Q3YEC7	8	8	8	0	8	0	6	0	8	0	6	0	8	0	6	16.9	79.548	0	20.548	287960000	0	2.22E+08	0	66318000	0	16821000	0	5443600
Q96C86	Q96C86	m7GpppX diphosphatase	DCPS	sp|Q96C86	6	6	6	0	6	0	4	0	6	0	4	0	6	0	4	24.9	38.608	0	23.289	287560000	0	2.51E+08	0	36803000	0	20347000	0	3270800
Q8WW59	Q8WW59	SPRY domain-containing protein 4	SPRYD4	sp|Q8WW	2	2	2	0	2	0	2	0	2	0	2	0	2	0	2	22.2	23.128	0	10.024	287300000	0	1.5E+08	0	1.37E+08	0	12056000	0	6809100
Q8IVD9	Q8IVD9	NudC domain-containing protein 3	NUDCD3	sp|Q8IVD9	10	10	10	0	8	1	9	0	8	1	9	0	8	1	9	43.2	40.822	0	58.928	287140000	0	75278000	3686600	2.08E+08	0	5457300	3184500	9032700
O14976	O14976	Cyclin-G-associated kinase	GAK	sp|O14976	7	7	7	0	1	1	7	0	1	1	7	0	1	1	7	10.2	143.19	0	33.248	285730000	0	4397900	2074700	2.79E+08	0	380620	745000	12141000
Q7Z2T5	Q7Z2T5	TRMT1-like protein	TRMT1L	sp|Q7Z2T5	10	10	10	0	9	3	8	0	9	3	8	0	9	3	8	23.5	81.746	0	26.56	285680000	0	1.01E+08	13076000	1.72E+08	0	6407600	13085000	5250700
Q16762	Q16762	Thiosulfate sulfurtransferase	TST	sp|Q16762	4	4	4	0	4	0	1	0	4	0	1	0	4	0	1	21.5	33.429	0	40.012	285650000	0	2.56E+08	0	29721000	0	22429000	0	1353200
Q3KQU3	Q3KQU3	MAP7 domain-containing protein 1	MAP7D1	sp|Q3KQU	12	12	12	0	2	9	11	0	2	9	11	0	2	9	11	19.1	92.819	0	27.023	285250000	0	9638000	41265000	2.34E+08	0	1303700	29945000	6968800
Q9UBQ0	Q9UBQ0	Vacuolar protein sorting-associated protein 29	VPS29	sp|Q9UBQ	3	3	3	0	2	0	3	0	2	0	3	0	2	0	3	23.6	20.505	0	62.376	285100000	0	78118000	0	2.07E+08	0	8252200	0	7145900
Q8WVJ2	Q8WVJ2	NudC domain-containing protein 2	NUDCD2	sp|Q8WVJ	3	3	3	0	3	1	3	0	3	1	3	0	3	1	3	28	17.676	0	85.466	285100000	0	1.44E+08	868410	1.4E+08	0	11818000	2849100	4371000
Q9UBC2	Q9UBC2	Epidermal growth factor receptor substrate 15-lik	EPS15L1	sp|Q9UBC	11	11	11	0	10	0	9	0	10	0	9	0	10	0	9	16	94.254	0	45.342	284970000	0	1.03E+08	0	1.82E+08	0	9515500	0	7042900
P37235;P8	P37235	Hippocalcin-like protein 1	HPCAL1	sp|P37235	4	4	4	0	0	0	4	0	0	0	4	0	0	0	4	23.3	22.313	0	11.831	284780000	0	0	0	2.85E+08	0	0	0	11712000
Q6P179	Q6P179	Endoplasmic reticulum aminopeptidase 2	ERAP2	sp|Q6P179	8	8	8	0	1	0	8	0	1	0	8	0	1	0	8	12.9	110.46	0	53.835	284620000	0	3656400	0	2.81E+08	0	322300	0	11555000
Q9Y508	Q9Y508	E3 ubiquitin-protein ligase RNF114	RNF114	sp|Q9Y508	4	4	4	0	4	2	4	0	4	2	4	0	4	2	4	26.8	25.694	0	40.523	284150000	0	70608000	3248200	2.1E+08	0	5769000	2585100	8701200
O95273	O95273	Cyclin-D1-binding protein 1	CCNDBP1	sp|O95273	1	1	1	0	0	0	1	0	0	0	1	0	0	0	1	2.5	40.262	0.009843	1.51	283670000	0	0	0	2.84E+08	0	0	0	11666000
Q2M2I8	Q2M2I8	AP2-associated protein kinase 1	AAK1	sp|Q2M2I8	10	10	10	0	2	1	10	0	2	1	10	0	2	1	10	23.3	103.88	0	23.943	283270000	0	11891000	1082600	2.7E+08	0	1331100	1082800	10478000
Q06265	Q06265	Exosome complex component RRP45	EXOSC9	sp|Q06265	5	5	5	0	5	3	5	0	5	3	5	0	5	3	5	14.4	48.948	0	15.387	283010000	0	1.17E+08	12712000	1.54E+08	0	8981200	10601000	5557000
O00206	O00206	Toll-like receptor 4	TLR4	sp|O00206	3	3	3	0	2	3	2	0	2	3	2	0	2	3	2	4.4	95.679	0	6.3206	282320000	0	44460000	25806000	2.12E+08	0	4002400	20456000	5522400
Q12756	Q12756	Kinesin-like protein KIF1A	KIF1A	sp|Q12756	10	10	8	0	10	3	3	0	10	3	3	0	8	1	1	8.9	191.06	0	33.221	282290000	0	2.13E+08	5110800	64333000	0	9427900	8062400	7351700
Q92769	Q92769	Histone deacetylase 2	HDAC2	sp|Q92769	9	5	4	1	8	4	6	0	4	2	3	0	4	2	3	23.8	55.364	0	33.11	280440000	0	78649000	5981200	1.96E+08	0	5804600	5505300	7695000
P51151;Q9	P51151	Ras-related protein Rab-9A	RAB9A	sp|P51151	5	5	5	0	3	0	5	0	3	0	5	0	3	0	5	37.3	22.837	0	17.322	280370000	0	57710000	0	2.23E+08	0	7922600	0	6321700
P53680	P53680	AP-2 complex subunit sigma	AP2S1	sp|P53680	4	4	4	0	3	2	4	0	3	2	4	0	3	2	4	31	17.018	0	10.063	279760000	0	29758000	3929500	2.46E+08	0	3814300	3572700	7996700
Q5T1M5	Q5T1M5	FK506-binding protein 15	FKBP15	sp|Q5T1M	11	11	11	0	11	5	10	0	11	5	10	0	11	5	10	15.3	133.63	0	42.422	279200000	0	1.16E+08	3899600	1.6E+08	0	8078000	6618600	4680700
Q9NZN4	Q9NZN4	EH domain-containing protein 2	EHD2	sp|Q9NZN	8	8	8	0	0	2	8	0	0	2	8	0	0	2	8	23	61.161	0	44.113	278050000	0	0	2028100	2.76E+08	0	0	3541500	9173100
Q15397	Q15397	Pumilio domain-containing protein KIAA0020	KIAA0020	sp|Q15397	5	5	5	0	0	3	5	0	0	3	5	0	0	3	5	11.1	73.584	0	11.278	277990000	0	0	33176000	2.45E+08	0	0	21992000	10370000
P08579	P08579	U2 small nuclear ribonucleoprotein B	SNRPB2	sp|P08579	6	4	4	0	4	4	5	0	3	2	4	0	3	2	4	30.7	25.486	0	17.891	277830000	0	1.09E+08	7797600	1.61E+08	0	11584000	4221200	5680100
Q04206	Q04206	Transcription factor p65	RELA	sp|Q04206	5	5	5	0	5	1	5	0	5	1	5	0	5	1	5	12.2	60.218	0	16.34	277340000	0	80509000	478180	1.96E+08	0	7631300	0	7540400
O95168	O95168	NADH dehydrogenase [ubiquinone] 1 beta subcor	NDUFB4	sp|O95168	4	4	4	0	3	3	3	0	3	3	3	0	3	3	3	41.1	15.208	0	16.385	276960000	0	1.06E+08	9338300	1.62E+08	0	10066000	6394400	5782600
Q86WJ1	Q86WJ1	Chromodomain-helicase-DNA-binding protein 1-li	CHD1L	sp|Q86WJ	8	8	8	0	8	2	5	0	8	2	5	0	8	2	5	11.4	101	0	32.836	276820000	0	1.56E+08	1953900	1.19E+08	0	8000800	8494700	3450500
Q96KB5	Q96KB5	Lymphokine-activated killer T-cell-originated prot	PBK	sp|Q96KB5	4	4	4	0	4	1	4	0	4	1	4	0	4	1	4	11.8	36.085	0	6.0155	276310000	0	78670000	601790	1.97E+08	0	3458500	855320	11128000
P25311	P25311	Zinc-alpha-2-glycoprotein	AZGP1	sp|P25311	11	11	11	0	1	11	1	0	1	11	1	0	1	11	1	43	34.258	0	35.801	275930000	0	18197000	255570000	2160300	0	282780	173090000	66551
P13984	P13984	General transcription factor IIF subunit 2	GTF2F2	sp|P13984	9	9	9	1	7	0	8	1	7	0	8	1	7	0	8	34.1	28.38	0	24.756	275730000	218650	66450000	0	2.09E+08	1689400	8219800	0	4764700
Q9P0V9	Q9P0V9	Septin-10	Sep-10	sp|Q9P0V9	6	6	6	0	0	2	6	0	0	2	6	0	0	2	6	19.8	52.592	0	36.169	275630000	0	0	1977400	2.74E+08	0	0	268580	12314000
Q9UPT5	Q9UPT5	Exocyst complex component 7	EXOC7	sp|Q9UPT5	8	8	8	0	5	2	6	0	5	2	6	0	5	2	6	13.2	83.381	0	21.334	275330000	0	57561000	5155100	2.13E+08	0	5129300	4584700	7568100
P10321;P0	P10321;P0	HLA class I histocompatibility antigen, Cw-7 alpha	HLA-C;HLA	sp|P10321	3	2	1	0	3	1	2	0	2	0	1	0	1	0	1	13.9	40.648	0	8.685	274980000	0	30172000	0	2.45E+08	0	1281600	0	11446000
Q8IWB7	Q8IWB7	WD repeat and FYVE domain-containing protein 1	WDFY1	sp|Q8IWB	4	4	4	0	1	0	4	0	1	0	4	0	1	0	4	12.9	46.323	0	10.535	274940000	0	6759900	0	2.68E+08	0	595880	0	11029000
Q9BXF6	Q9BXF6	Rab11 family-interacting protein 5	RAB11FIP5	sp|Q9BXF6	11	11	11	0	2	9	11	0	2	9	11	0	2	9	11	27.3	70.414	0	25.523	274350000	0	8105100	26333000	2.4E+08	0	2193900	11510000	14572000
Q92575	Q92575	UBX domain-containing protein 4	UBXN4	sp|Q92575	5	5	5	0	5	1	5	0	5	1	5	0	5	1	5	20.5	56.777	0	15.319	274270000	0	72735000	1344900	2E+08	0	4003800	1482400	10062000
O95630	O95630	STAM-binding protein	STAMBP	sp|O95630	4	4	4	0	3	0	4	0	3	0	4	0	3	0	4	12.3	48.076	0	14.331	274070000	0	67961000	0	2.06E+08	0	6351500	0	8115500
Q9NS87	Q9NS87	Kinesin-like protein KIF15	KIF15	sp|Q9NS87	6	6	6	0	1	0	6	0	1	0	6	0	1	0	6	5.5	160.16	0	19.003	274040000	0	31961000	0	2.42E+08	0	3689300	0	9083600
Q9NP97;Q	Q9NP97	Dynein light chain roadblock-type 1	DYNLRB1	sp|Q9NP97	3	3	3	0	0	1	3	0	0	1	3	0	0	1	3	51	10.921	0	11.577	273770000	0	0	4782200	2.69E+08	0	0	4468600	9807200
Q9Y6M9	Q9Y6M9	NADH dehydrogenase [ubiquinone] 1 beta subcor	NDUFB9	sp|Q9Y6M	5	5	5	0	5	0	4	0	5	0	4	0	5	0	4	35.8	21.831	0	19.322	273630000	0	1.32E+08	0	1.41E+08	0	11598000	0	5880100
Q9Y676	Q9Y676	28S ribosomal protein S18b, mitochondrial	MRPS18B	sp|Q9Y676	5	5	5	0	5	2	3	0	5	2	3	0	5	2	3	39.1	29.395	0	16.815	273460000	0	2.1E+08	4155400	59111000	0	14930000	5056800	3765600
O75352	O75352	Mannose-P-dolichol utilization defect 1 protein	MPDU1	sp|O75352	1	1	1	0	1	0	1	0	1	0	1	0	1	0	1	4	26.638	0.000944	3.0787	273270000	0	56475000	0	2.17E+08	0	4978200	0	8915700
Q14694	Q14694	Ubiquitin carboxyl-terminal hydrolase 10	USP10	sp|Q14694	10	10	10	1	5	7	7	1	5	7	7	1	5	7	7	21.1	87.133	0	35.082	273210000	1002900	71209000	40623000	1.6E+08	1599900	6315900	27866000	5392400
Q9NQR4	Q9NQR4	Omega-amidase NIT2	NIT2	sp|Q9NQR	5	5	5	0	5	0	5	0	5	0	5	0	5	0	5	27.2	30.608	0	29.818	272180000	0	1E+08	0	1.72E+08	0	9230500	0	6674300
Q16222	Q16222	UDP-N-acetylhexosamine pyrophosphorylase;UDI	UAP1	sp|Q16222	7	6	6	0	7	1	6	0	6	1	5	0	6	1	5	17.6	58.768	0	31.24	272040000	0	1.3E+08	2357800	1.4E+08	0	11028000	2260000	5508900
O43676	O43676	NADH dehydrogenase [ubiquinone] 1 beta subcor	NDUFB3	sp|O43676	4	4	4	1	4	0	3	1	4	0	3	1	4	0	3	35.7	11.402	0	7.8336	272020000	400420	1.42E+08	0	1.29E+08	5440300	8252200	0	4566400
Q63ZY3;Q1	Q63ZY3	KN motif and ankyrin repeat domain-containing p	KANK2	sp|Q63ZY3	10	10	10	0	0	6	10	0	0	6	10	0	0	6	10	16.3	91.173	0	38.557	271930000	0	0	12283000	2.6E+08	0	0	12589000	6343100
Q7Z4H3	Q7Z4H3	HD domain-containing protein 2	HDDC2	sp|Q7Z4H3	8	8	8	1	8	1	4	1	8	1	4	1	8	1	4	45.1	23.39	0	72.829	271770000	10822000	2.34E+08	877340	25752000	6753800	22783000	0	2999200
Q9HCN4	Q9HCN4	GPN-loop GTPase 1	GPN1	sp|Q9HCN	5	5	5	0	3	0	5	0	3	0	5	0	3	0	5	25.9	41.74	0	36.908	271740000	0	38829000	0	2.33E+08	0	5143600	0	7857600
O14787	O14787	Transportin-2	TNPO2	sp|O14787	12	8	8	1	8	2	11	0	5	0	7	0	5	0	7	22	101.39	0	20.105	271670000	0	78698000	0	1.93E+08	0	8467300	0	6405900
Q15424;Q1	Q15424;Q1	Scaffold attachment factor B1;Scaffold attachmer	SAFB;SAFB	sp|Q15424	7	7	7	1	6	2	4	1	6	2	4	1	6	2	4	12.1	102.64	0	28.223	271230000	3628400	1.24E+08	8705200	1.35E+08	6894100	7822300	6920800	4309300
Q92522	Q92522	Histone H1x	H1FX	sp|Q92522	3	3	3	0	3	3	2	0	3	3	2	0	3	3	2	18.8	22.487	0	14.108	271100000	0	1.67E+08	18907000	84891000	0	13750000	12310000	4883600
Q9UJX5	Q9UJX5	Anaphase-promoting complex subunit 4	ANAPC4	sp|Q9UJX5	8	8	8	0	4	8	7	0	4	8	7	0	4	8	7	18.9	92.115	0	35.854	271080000	0	32747000	75581000	1.63E+08	0	6161500	45568000	8640200
Q9NUV7	Q9NUV7	Serine palmitoyltransferase 3	SPTLC3	sp|Q9NUV	7	7	7	0	0	3	7	0	0	3	7	0	0	3	7	18.3	62.049	0	42.525	270800000	0	0	10173000	2.61E+08	0	0	8294500	9259800
Q9H9P8	Q9H9P8	L-2-hydroxyglutarate dehydrogenase, mitochondi	L2HGDH	sp|Q9H9P8	8	8	8	0	6	0	6	0	6	0	6	0	6	0	6	23.3	50.315	0	35.157	270410000	0	1.16E+08	0	1.54E+08	0	11593000	0	4980900
Q9UKM9	Q9UKM9	RNA-binding protein Raly	RALY	sp|Q9UKM	8	8	8	2	4	8	6	2	4	8	6	2	4	8	6	33.3	32.463	0	27.812	270190000	5043100	33942000	88331000	1.43E+08	8927800	3639200	56659000	4043700
Q96P47;Q9	Q96P47	Arf-GAP with GTPase, ANK repeat and PH domain	AGAP3	sp|Q96P47	4	4	4	0	2	3	4	0	2	3	4	0	2	3	4	7.5	95.043	0	17.39	270170000	0	7454700	8104300	2.55E+08	0	2470600	8868500	5235000
Q96DV4	Q96DV4	39S ribosomal protein L38, mitochondrial	MRPL38	sp|Q96DV4	5	5	5	0	4	3	4	0	4	3	4	0	4	3	4	16.6	44.596	0	28.242	269710000	0	51929000	7795600	2.1E+08	0	5308900	5860800	7282200
P35251	P35251	Replication factor C subunit 1	RFC1	sp|P35251	6	6	6	1	6	2	6	1	6	2	6	1	6	2	6	9.2	128.25	0	11.668	269590000	1007600	97780000	9851000	1.61E+08	0	7068800	8464000	6325400
Q9NR56;Q	Q9NR56	Muscleblind-like protein 1	MBNL1	sp|Q9NR56	4	4	4	1	3	1	3	1	3	1	3	1	3	1	3	18	41.817	0	38.517	269530000	859000	98912000	3724600	1.66E+08	3546200	4639000	4472500	5026200
Q9Y320	Q9Y320	Thioredoxin-related transmembrane protein 2	TMX2	sp|Q9Y320	4	4	4	0	4	0	4	0	4	0	4	0	4	0	4	22	34.037	0	12.294	269410000	0	70729000	0	1.99E+08	0	7197800	0	6583800
P15374	P15374	Ubiquitin carboxyl-terminal hydrolase isozyme L3	UCHL3	sp|P15374	5	5	5	0	4	0	5	0	4	0	5	0	4	0	5	27.8	26.182	0	25.296	269340000	0	63288000	0	2.06E+08	0	5654400	0	8398200
Q5VWZ2	Q5VWZ2	Lysophospholipase-like protein 1	LYPLAL1	sp|Q5VWZ	6	6	6	0	6	2	5	0	6	2	5	0	6	2	5	28.3	26.316	0	20.168	269010000	0	1.07E+08	2675200	1.6E+08	0	9825200	2903400	5038200
Q9P0S9	Q9P0S9	Transmembrane protein 14C	TMEM14C	sp|Q9P0S9	3	3	3	0	2	0	3	0	2	0	3	0	2	0	3	50	11.564	0	11.074	268900000	0	57133000	0	2.12E+08	0	5428700	0	8316400
O14949	O14949	Cytochrome b-c1 complex subunit 8	UQCRQ	sp|O14949	4	4	4	0	4	4	4	0	4	4	4	0	4	4	4	37.8	9.9062	0	7.7716	268150000	0	62064000	10007000	1.96E+08	0	5968800	8085500	6205000
Q15075	Q15075	Early endosome antigen 1	EEA1	sp|Q15075	16	16	16	3	11	11	11	3	11	11	11	3	11	11	11	15.1	162.46	0	36.863	267880000	3008400	54042000	32605000	1.78E+08	3840000	6099600	20900000	6173100
Q9UN37	Q9UN37	Vacuolar protein sorting-associated protein 4A	VPS4A	sp|Q9UN3	6	6	3	0	6	4	5	0	6	4	5	0	3	1	2	15.3	48.897	0	11.758	267460000	0	1.06E+08	10054000	1.51E+08	0	8874900	6951400	6509200
Q7Z460	Q7Z460	CLIP-associating protein 1	CLASP1	sp|Q7Z460	10	8	8	0	7	4	10	0	5	3	8	0	5	3	8	9	169.45	0	25.087	267260000	0	43212000	4525400	2.2E+08	0	4696900	878810	10302000
Q9BZG1	Q9BZG1	Ras-related protein Rab-34	RAB34	sp|Q9BZG1	6	6	6	0	3	1	6	0	3	1	6	0	3	1	6	31.7	29.044	0	20.873	266300000	0	19634000	1125900	2.46E+08	0	1607700	1405200	9572500
Q9UH62	Q9UH62	Armadillo repeat-containing X-linked protein 3	ARMCX3	sp|Q9UH6	5	5	5	1	4	3	5	1	4	3	5	1	4	3	5	15.8	42.5	0	13.854	266080000	245550	84468000	7180400	1.74E+08	0	7720900	5293800	6419800
P62942	P62942	Peptidyl-prolyl cis-trans isomerase FKBP1A	FKBP1A	sp|P62942	2	2	2	0	2	0	2	0	2	0	2	0	2	0	2	25	11.951	0	16.184	265980000	0	1.7E+08	0	96376000	0	14940000	0	3973300
Q96S66	Q96S66	Chloride channel CLIC-like protein 1	CLCC1	sp|Q96S66	9	9	9	1	7	0	9	1	7	0	9	1	7	0	9	22.9	62.022	0	24.674	264440000	1238800	77679000	0	1.86E+08	0	8169600	0	6307600
Q16740	Q16740	ATP-dependent Clp protease proteolytic subunit,	CLPP	sp|Q16740	5	5	5	0	4	0	5	0	4	0	5	0	4	0	5	30	30.18	0	27.716	264170000	0	1.06E+08	0	1.58E+08	0	8867600	0	6983100
Q02252	Q02252	Methylmalonate-semialdehyde dehydrogenase [a	ALDH6A1	sp|Q02252	9	9	9	0	8	0	7	0	8	0	7	0	8	0	7	23.4	57.839	0	29.006	263720000	0	1.49E+08	0	1.15E+08	0	12124000	0	5730400
Q9NXH9	Q9NXH9	tRNA (guanine(26)-N(2))-dimethyltransferase	TRMT1	sp|Q9NXH	6	6	6	0	4	1	6	0	4	1	6	0	4	1	6	13.5	72.233	0	23.452	263570000	0	38609000	400530	2.25E+08	0	5889100	2175800	4842800
Q12996	Q12996	Cleavage stimulation factor subunit 3	CSTF3	sp|Q12996	5	5	5	0	3	0	5	0	3	0	5	0	3	0	5	13.2	82.921	0	19.716	262780000	0	40764000	0	2.22E+08	0	6720300	0	6003700
Q8NFQ8	Q8NFQ8	Torsin-1A-interacting protein 2	TOR1AIP2	sp|Q8NFQ	10	10	10	0	10	1	9	0	10	1	9	0	10	1	9	30.6	51.263	0	23.626	262390000	0	75945000	1323000	1.85E+08	0	3956900	1770100	9469700
Q99575	Q99575	Ribonucleases P/MRP protein subunit POP1	POP1	sp|Q99575	7	7	7	2	6	6	7	2	6	6	7	2	6	6	7	9.2	114.71	0	14.365	262160000	17118000	86624000	13751000	1.45E+08	14431000	8923600	7823400	8766500
Q9NVV0	Q9NVV0	Trimeric intracellular cation channel type B	TMEM38B	sp|Q9NVV	1	1	1	1	1	0	1	1	1	0	1	1	1	0	1	7.6	32.509	0	12.016	261880000	2E+08	24999000	0	37019000	2E+08	1935600	0	1140100
Q9H4A3;Q	Q9H4A3	Serine/threonine-protein kinase WNK1	WNK1	sp|Q9H4A3	10	10	9	0	8	2	10	0	8	2	10	0	7	2	9	6.1	250.79	0	29.704	261730000	0	67373000	905570	1.93E+08	0	4364000	977640	9161500
Q92917	Q92917	G patch domain and KOW motifs-containing prote	GPKOW	sp|Q92917	7	7	7	0	5	0	5	0	5	0	5	0	5	0	5	28.8	52.228	0	33.239	261350000	0	1.31E+08	0	1.3E+08	0	12595000	0	4313900
Q6PD62	Q6PD62	RNA polymerase-associated protein CTR9 homolc	CTR9	sp|Q6PD62	7	7	7	0	6	1	4	0	6	1	4	0	6	1	4	9.3	133.5	0	27.671	261010000	0	78998000	1301400	1.81E+08	0	7929100	0	6466300
Q96GA3	Q96GA3	Protein LTV1 homolog	LTV1	sp|Q96GA3	3	3	3	0	2	1	2	0	2	1	2	0	2	1	2	9.9	54.854	0	101.75	260980000	0	55654000	6478200	1.99E+08	0	4362700	5797600	7276600
Q8IZL8	Q8IZL8	Proline-, glutamic acid- and leucine-rich protein 1	PELP1	sp|Q8IZL8	8	8	8	1	8	1	2	1	8	1	2	1	8	1	2	11.2	119.7	0	26.288	260620000	575170	2.19E+08	784490	40272000	431990	20111000	413580	1104900
P83916	P83916	Chromobox protein homolog 1	CBX1	sp|P83916	5	4	4	2	4	5	3	1	3	4	2	1	3	4	2	28.6	21.418	0	17.306	259210000	5292600	53783000	156460000	43674000	17403000	5095200	91475000	2993700
Q969S3	Q969S3	Zinc finger protein 622	ZNF622	sp|Q969S3	5	5	5	0	4	4	5	0	4	4	5	0	4	4	5	16.1	54.271	0	23.82	258970000	0	61681000	12800000	1.84E+08	0	4909000	8686900	8029900

P48507	P48507	Glutamate--cysteine ligase regulatory subunit	GCLM	sp|P48507	4	4	4	0	3	0	4	0	3	0	4	0	3	0	4	26.3	30.727	0	17.694	258870000	0	52675000	0	2.06E+08	0	5912100	0	7211200
Q9NYY8	Q9NYY8	FAST kinase domain-containing protein 2	FASTKD2	sp|Q9NYY	6	6	6	0	1	2	6	0	1	2	6	0	1	2	6	11.7	81.462	0	28.028	258590000	0	6644200	5163700	2.47E+08	0	831180	2633700	10740000
Q9Y5X2	Q9Y5X2	Sorting nexin-8	SNX8	sp|Q9Y5X	4	4	4	0	0	4	4	0	0	4	4	0	0	4	4	13.8	52.569	0	16.658	257890000	0	0	19755000	2.38E+08	0	0	14572000	8497500
Q9NR19	Q9NR19	Acetyl-coenzyme A synthetase, cytoplasmic	ACSS2	sp|Q9NR1	5	5	5	0	1	0	5	0	1	0	5	0	1	0	5	11.8	78.579	0	10.542	257820000	0	1365900	0	2.56E+08	0	415200	0	10252000
Q8IX01	Q8IX01	SURP and G-patch domain-containing protein 2	SUGP2	sp|Q8IX01	4	4	4	0	1	3	4	0	1	3	4	0	1	3	4	4.2	120.21	0	7.0253	257790000	0	2173200	8604000	2.47E+08	0	1791800	12393000	1946800
Q9UKV3	Q9UKV3	Apoptotic chromatin condensation inducer in the	ACIN1	sp|Q9UKV	12	12	12	2	9	7	9	2	9	7	9	2	9	7	9	13.6	151.86	0	26.939	257290000	4403000	1.26E+08	13448000	1.14E+08	4694400	9867000	10803000	3838400
Q13043	Q13043	Serine/threonine-protein kinase 4;Serine/threoni	STK4	sp|Q13043	6	6	5	0	6	0	4	0	6	0	4	0	5	0	3	17	55.63	0	17.671	256110000	0	1.02E+08	0	1.55E+08	0	7071600	0	8237100
Q9BRT2	Q9BRT2	Ubiquinol-cytochrome-c reductase complex asser	UQCC2	sp|Q9BRT	3	3	3	0	3	2	2	0	3	2	2	0	3	2	2	28.6	14.875	0	8.3984	255830000	0	93015000	4524700	1.58E+08	0	6471400	3965800	7312500
Q9BQ61	Q9BQ61	Uncharacterized protein C19orf43	C19orf43	sp|Q9BQ6	6	6	6	1	5	3	5	1	5	3	5	1	5	3	5	60.2	18.419	0	19.371	255520000	10353000	1.12E+08	9147300	1.24E+08	10979000	6649000	9144300	4709000
Q8WTS6	Q8WTS6	Histone-lysine N-methyltransferase SETD7	SETD7	sp|Q8WTS	5	5	5	0	5	1	4	0	5	1	4	0	5	1	4	19.7	40.72	0	22.176	255440000	0	78877000	10112000	1.66E+08	0	6645800	7658700	6289500
Q9GZP9	Q9GZP9	Derlin-2	DERL2	sp|Q9GZP	2	2	2	0	1	0	2	0	1	0	2	0	1	0	2	17.2	27.567	0	56.326	255390000	0	70646000	0	1.85E+08	0	6227400	0	7597600
Q13363	Q13363	C-terminal-binding protein 1	CTBP1	sp|Q13363	5	4	4	0	5	0	5	0	4	0	4	0	4	0	4	17.3	47.535	0	24.143	254810000	0	79109000	0	1.76E+08	0	7775500	0	6349500
P56556	P56556	NADH dehydrogenase [ubiquinone] 1 alpha subco	NDUFA6	sp|P56556	5	5	5	1	5	4	4	1	5	4	4	1	5	4	4	39.1	15.136	0	7.7489	253750000	1792800	1.42E+08	3978700	1.06E+08	3544400	12490000	2575100	2753400
Q9Y697	Q9Y697	Cysteine desulfurase, mitochondrial	NFS1	sp|Q9Y697	5	5	5	0	4	0	5	0	4	0	5	0	4	0	5	14	50.195	0	13.839	253100000	0	38872000	0	2.14E+08	0	6509000	0	5727800
Q14160	Q14160	Protein scribble homolog	SCRIB	sp|Q14160	10	10	9	1	7	1	9	1	7	1	9	1	6	1	8	10.3	174.88	0	43.368	253010000	1041400	1.04E+08	3243600	1.45E+08	1794700	9952900	0	4396400
O75145;O7	O75145	Liprin-alpha-3	PPFIA3	sp|O75145	15	15	13	11	3	11	1	11	3	11	1	11	2	9	0	15.6	133.49	0	39.06	252740000	1.4E+08	21631000	65224000	26145000	94797000	2669200	83695000	5390700
Q96KR1	Q96KR1	Zinc finger RNA-binding protein	ZFR	sp|Q96KR	11	11	11	1	7	6	9	1	7	6	9	1	7	6	9	21.1	117.01	0	41.328	252560000	594420	38183000	22010000	1.92E+08	2646700	3617300	11475000	8898000
P82664	P82664	28S ribosomal protein S10, mitochondrial	MRPS10	sp|P82664	3	3	3	0	3	2	3	0	3	2	3	0	3	2	3	22.4	22.999	0	11.13	251960000	0	1.34E+08	16908000	1.01E+08	0	13279000	8089500	5954000
P55265	P55265	Double-stranded RNA-specific adenosine deamin	ADAR	sp|P55265	8	8	8	1	6	7	7	1	6	7	7	1	6	7	7	8.7	136.06	0	24.153	251870000	1535400	89542000	32839000	1.28E+08	2063400	8801100	22304000	3589900
A6NDG6	A6NDG6	Phosphoglycolate phosphatase	PGP	sp|A6NDG	5	5	5	0	4	1	4	0	4	1	4	0	4	1	4	29	34.006	0	15.02	251790000	0	1.62E+08	847140	89408000	0	14441000	1123600	2920500
P19022;P5	P19022	Cadherin-2	CDH2	sp|P19022	6	6	6	0	5	1	3	0	5	1	3	0	5	1	3	15.1	99.808	0	39.895	250970000	0	1.61E+08	2241500	87350000	0	10959000	3246600	5118200
Q9BRP1	Q9BRP1	Programmed cell death protein 2-like	PDCD2L	sp|Q9BRP	5	5	5	0	4	1	5	0	4	1	5	0	4	1	5	20.4	39.416	0	20.038	250780000	0	92558000	1213700	1.57E+08	0	9415400	1918500	4097600
Q8TDD1	Q8TDD1	ATP-dependent RNA helicase DDX54	DDX54	sp|Q8TDD	8	8	8	2	6	5	6	2	6	5	6	2	6	5	6	12.9	98.594	0	13.831	249900000	4210000	74657000	13945000	1.57E+08	7186500	4170000	10807000	4459100
Q96ST3	Q96ST3	Paired amphipathic helix protein Sin3a	SIN3A	sp|Q96ST3	8	8	8	2	6	1	7	2	6	1	7	2	6	1	7	10.1	145.17	0	30.709	249840000	2197100	45483000	734970	2.01E+08	4538800	5777400	1847300	2820300
P51648;P3	P51648	Fatty aldehyde dehydrogenase	ALDH3A2	sp|P51648	5	5	4	0	5	4	5	0	5	4	5	0	4	4	4	11.1	54.847	0	10.936	248660000	0	69718000	8191800	1.71E+08	0	7050800	5655300	5966500
P11182	P11182	Lipoamide acyltransferase component of branche	DBT	sp|P11182	7	7	7	1	6	3	2	1	6	3	2	1	6	3	2	20.7	53.486	0	15.801	248550000	37485000	1.34E+08	10031000	66938000	0	7524200	10167000	3622800
Q15276	Q15276	Rab GTPase-binding effector protein 1	RABEP1	sp|Q15276	7	7	7	0	7	1	5	0	7	1	5	0	7	1	5	11	99.289	0	24.463	247920000	0	95854000	419830	1.52E+08	0	5231900	3196600	6539400
Q53H82	Q53H82	Beta-lactamase-like protein 2	LACTB2	sp|Q53H8	5	5	5	1	5	2	5	1	5	2	5	1	5	2	5	25.3	32.805	0	16.995	247420000	989080	1E+08	2693600	1.43E+08	0	8157800	2723700	4275700
O15091	O15091	Mitochondrial ribonuclease P protein 3	KIAA0391	sp|O15091	4	4	4	0	3	0	4	0	3	0	4	0	3	0	4	8.6	67.315	0	15.603	246830000	0	75587000	0	1.71E+08	0	7734800	0	5970800
P56211	P56211	cAMP-regulated phosphoprotein 19	ARPP19	sp|P56211	3	2	2	0	3	0	3	0	2	0	2	0	2	0	2	50	12.323	0	10.306	246370000	0	79162000	0	1.67E+08	0	6225500	0	6190300
Q96B26	Q96B26	Exosome complex component RRP43	EXOSC8	sp|Q96B2	4	4	4	3	4	4	4	3	4	4	4	3	4	4	4	24.6	30.039	0	20.64	246300000	19429000	81558000	22636000	1.23E+08	29999000	5079000	8269300	3527400
Q9BTC0	Q9BTC0	Death-inducer obliterator 1	DIDO1	sp|Q9BTC	9	9	9	0	4	4	9	0	4	4	9	0	4	4	9	8.6	243.87	0	32.92	245970000	0	30391000	11767000	2.04E+08	0	3981100	8038300	6948800
Q9NY27	Q9NY27	Serine/threonine-protein phosphatase 4 regulato	PPP4R2	sp|Q9NY2	5	5	5	0	5	0	4	0	5	0	4	0	5	0	4	19.2	46.898	0	18.683	245710000	0	86336000	0	1.59E+08	0	7865100	0	6299700
Q14671;Q8	Q14671	Pumilio homolog 1	PUM1	sp|Q14671	9	9	9	0	8	2	8	0	8	2	8	0	8	2	8	9.6	126.47	0	32.202	245600000	0	92868000	1392200	1.51E+08	0	7327600	4110900	3907100
Q9Y6I3	Q9Y6I3	Epsin-1	EPN1	sp|Q9Y6I3	5	5	4	0	5	0	5	0	5	0	5	0	4	0	4	10.2	60.293	0	7.3387	245280000	0	89188000	0	1.56E+08	0	6706300	0	7575100
P78330	P78330	Phosphoserine phosphatase	PSPH	sp|P78330	5	5	5	0	5	0	2	0	5	0	2	0	5	0	2	39.6	25.007	0	25.336	245210000	0	1.92E+08	0	52827000	0	13952000	0	4287800
Q9H2J4	Q9H2J4	Phosducin-like protein 3	PDCL3	sp|Q9H2J4	5	5	5	0	3	0	5	0	3	0	5	0	3	0	5	31.4	27.614	0	66.55	245100000	0	54045000	0	1.91E+08	0	5984100	0	6637000
Q9H0R6	Q9H0R6	Glutamyl-tRNA(Gln) amidotransferase subunit A,	QRSL1	sp|Q9H0R	4	4	4	0	4	0	3	0	4	0	3	0	4	0	3	10.6	57.46	0	11.757	245000000	0	64021000	0	1.81E+08	0	4309400	0	8776800
Q04941	Q04941	Proteolipid protein 2	PLP2	sp|Q04941	1	1	1	1	0	0	1	1	0	0	1	1	0	0	1	8.6	16.691	0.001159	2.8106	244800000	174750	0	0	2.45E+08	174750	0	0	10060000
Q6XZF7	Q6XZF7	Dynamin-binding protein	DNMBP	sp|Q6XZF7	9	9	9	0	0	0	9	0	0	0	9	0	0	0	9	9.3	177.35	0	41.852	244660000	0	0	0	2.45E+08	0	0	0	10062000
Q9UJX3	Q9UJX3	Anaphase-promoting complex subunit 7	ANAPC7	sp|Q9UJX3	11	11	11	0	9	10	6	0	9	10	6	0	9	10	6	23	66.855	0	31.08	244660000	0	87190000	53232000	1.04E+08	0	7240200	34917000	5587100
Q9NRX4	Q9NRX4	14 kDa phosphohistidine phosphatase	PHPT1	sp|Q9NRX	4	4	4	0	4	0	4	0	4	0	4	0	4	0	4	44.8	13.832	0	15.692	244650000	0	1.34E+08	0	1.1E+08	0	11075000	0	5307700
Q9UPT8	Q9UPT8	Zinc finger CCCH domain-containing protein 4	ZC3H4	sp|Q9UPT	8	8	8	1	7	4	5	1	7	4	5	1	7	4	5	14	140.26	0	18.025	244620000	3762500	1.3E+08	10489000	1E+08	8299300	6438300	7346700	4328600
Q15629	Q15629	Translocating chain-associated membrane protei	TRAM1	sp|Q1562	3	3	3	0	3	3	3	0	3	3	3	0	3	3	3	10.2	43.071	0	23.431	244500000	0	65350000	13003000	1.66E+08	0	3486400	6152500	4995900
Q13242	Q13242	Serine/arginine-rich splicing factor 9	SRSF9	sp|Q13242	8	8	8	0	7	5	5	0	7	5	5	0	7	5	5	37.6	25.542	0	16.242	243730000	0	1.86E+08	11315000	46827000	0	12795000	10151000	2943500
O43169	O43169	Cytochrome b5 type B	CYB5B	sp|O4316	3	3	3	0	2	0	3	0	2	0	3	0	2	0	3	40.7	16.694	0	30.186	243160000	0	61079000	0	1.82E+08	0	6693700	0	6178400
Q9NZD8	Q9NZD8	Maspardin	SPG21	sp|Q9NZD	4	4	4	0	3	0	4	0	3	0	4	0	3	0	4	22.4	34.96	0	35.697	242990000	0	19512000	0	2.23E+08	0	2856700	0	8054000
O94855	O94855	Protein transport protein Sec24D	SEC24D	sp|O9485	7	7	7	0	5	0	7	0	5	0	7	0	5	0	7	7.9	113.01	0	15.049	242630000	0	25646000	0	2.17E+08	0	3537400	0	7647100
Q8WTT2	Q8WTT2	Nucleolar complex protein 3 homolog	NOC3L	sp|Q8WTT	4	4	4	0	4	3	4	0	4	3	4	0	4	3	4	6.8	92.547	0	12.438	242540000	0	65714000	13223000	1.64E+08	0	5110900	11443000	4852800
P05089	P05089	Arginase-1	ARG1	sp|P05089	7	7	7	0	5	7	0	0	5	7	0	0	5	7	0	29.2	34.735	0	24.769	242350000	0	67944000	174410000	0	0	6633000	116560000	0
Q8NA56	Q8NA56	Tetratricopeptide repeat protein 29	TTC29	sp|Q8NA5	1	1	1	0	1	0	1	0	1	0	1	0	1	0	1	2.5	55.081	0.006408	1.7182	242190000	0	1.42E+08	0	1E+08	0	12307000	0	4335400
O95071	O95071	E3 ubiquitin-protein ligase UBR5	UBR5	sp|O95071	12	12	12	0	10	3	10	0	10	3	10	0	10	3	10	5.5	309.35	0	28.991	241830000	0	64733000	2342900	1.75E+08	0	8510500	2389000	3568000
O00217	O00217	NADH dehydrogenase [ubiquinone] iron-sulfur pr	NDUFS8	sp|O00217	4	4	4	0	3	1	4	0	3	1	4	0	3	1	4	20.5	23.705	0	11.244	241430000	0	65866000	447010	1.75E+08	0	5067800	1943000	6297500
Q9Y6Y0	Q9Y6Y0	Influenza virus NS1A-binding protein	IVNS1ABP	sp|Q9Y6Y	4	4	4	0	2	0	4	0	2	0	4	0	2	0	4	13.6	71.729	0	52.013	241230000	0	28482000	0	2.13E+08	0	4412400	0	6847400
Q8TBA6	Q8TBA6	Golgin subfamily A member 5	GOLGA5	sp|Q8TBA	10	10	10	0	5	8	8	0	5	8	8	0	5	8	8	18.5	83.023	0	42.907	240620000	0	48087000	23228000	1.69E+08	0	5207000	14874000	6729900
Q9UPU5	Q9UPU5	Ubiquitin carboxyl-terminal hydrolase 24	USP24	sp|Q9UPU	13	13	13	1	12	0	9	1	12	0	9	1	12	0	9	6.6	294.36	0	42.673	240270000	1097300	1.09E+08	0	1.3E+08	1651000	7934900	0	6481000
P10586;P2	P10586	Receptor-type tyrosine-protein phosphatase F	PTPRF	sp|P10586	12	12	12	0	12	1	1	0	12	1	1	0	12	1	1	9.7	212.88	0	40.467	240210000	0	2.35E+08	1252700	4344000	0	21228000	323980	149250
Q0VDG4	Q0VDG4	Secernin-3	SCRN3	sp|Q0VDG	4	4	4	1	2	0	4	1	2	0	4	1	2	0	4	13.7	48.544	0	14.794	240080000	63877000	35843000	0	1.4E+08	0	5499300	0	3432400
Q96RQ1	Q96RQ1	Endoplasmic reticulum-Golgi intermediate compa	ERGIC2	sp|Q96RQ	4	4	4	0	3	1	4	0	3	1	4	0	3	1	4	15.1	42.548	0	17.66	239990000	0	32471000	2496100	2.05E+08	0	2271900	1678100	9021400
Q08209	Q08209	Serine/threonine-protein phosphatase 2B catalyt	PPP3CA	sp|Q0820	9	9	4	0	8	0	7	0	8	0	7	0	3	0	3	20.9	58.687	0	16.921	239840000	0	1.08E+08	0	1.32E+08	0	7869900	0	7065300
Q9H2U1	Q9H2U1	ATP-dependent RNA helicase DHX36	DHX36	sp|Q9H2U	9	9	8	1	4	5	8	1	4	5	8	0	4	4	7	12.5	114.76	0	18.045	239660000	609420	18522000	19317000	2.01E+08	1625400	2239200	10093000	9540900
Q12929	Q12929	Epidermal growth factor receptor kinase substrat	EPS8	sp|Q1292	10	10	10	0	1	1	10	0	1	1	10	0	1	1	10	20.4	91.88	0	42.654	239130000	0	3624900	2975100	2.33E+08	0	360390	0	9522000
P60033	P60033	CD81 antigen	CD81	sp|P60033	1	1	1	0	1	0	1	0	1	0	1	0	1	0	1	8.5	25.809	0	56.879	239040000	0	1.74E+08	0	65207000	0	13052000	0	2786500
Q7Z4Q2	Q7Z4Q2	HEAT repeat-containing protein 3	HEATR3	sp|Q7Z4Q	4	4	4	0	3	1	2	0	3	1	2	0	3	1	2	9.7	74.582	0	13.194	238770000	0	1.18E+08	3197000	1.18E+08	0	8617000	4332300	4436000
P61129	P61129	Zinc finger CCCH domain-containing protein 6	ZC3H6	sp|P61129	1	1	1	0	1	0	1	0	1	0	1	0	1	0	1	0.6	131.67	0.004732	1.8055	238630000	0	1.17E+08	0	1.21E+08	0	10349000	0	4985500
Q13136;Q	Q13136	Liprin-alpha-1	PPFIA1	sp|Q13136	12	10	10	0	7	5	10	0	6	3	9	0	6	3	9	15	135.78	0	32.048	236950000	0	71950000	7736900	1.57E+08	0	4907300	8951000	4150600
Q96ST2	Q96ST2	Protein IWS1 homolog	IWS1	sp|Q96ST2	6	6	6	0	5	1	5	0	5	1	5	0	5	1	5	9.2	91.954	0	20.319	236920000	0	83627000	1996400	1.51E+08	0	4872100	4335200	5728200
Q9BW83	Q9BW83	Intraflagellar transport protein 27 homolog	IFT27	sp|Q9BW8	3	3	3	0	3	0	3	0	3	0	3	0	3	0	3	24.7	20.48	0	14.783	236860000	0	88974000	0	1.48E+08	0	7948500	0	5976200
Q8IYB8	Q8IYB8	ATP-dependent RNA helicase SUPV3L1, mitochon	SUPV3L1	sp|Q8IYB8	8	8	8	1	8	0	5	1	8	0	5	1	8	0	5	13.4	87.99	0	19.857	236800000	164250	1.56E+08	0	80538000	0	12755000	0	4316600
Q2TAY7	Q2TAY7	WD40 repeat-containing protein SMU1;WD40 rep	SMU1	sp|Q2TAY	8	8	8	0	7	3	7	0	7	3	7	0	7	3	7	22.6	57.543	0	25.623	236510000	0	78097000	4216200	1.54E+08	0	7560100	4329900	4168900
P23434	P23434	Glycine cleavage system H protein, mitochondrial	GCSH	sp|P23434	1	1	1	0	1	1	1	0	1	1	1	0	1	1	1	11.6	18.884	0	15.007	236380000	0	1.16E+08	3191000	1.17E+08	0	6839100	5720500	3776300
Q96L92	Q96L92	Sorting nexin-27	SNX27	sp|Q96L92	6	6	6	1	6	0	5	1	6	0	5	1	6	0	5	15	61.264	0	27.209	236290000	208590	69820000	0	1.66E+08	0	6457500	0	6534400
O95817	O95817	BAG family molecular chaperone regulator 3	BAG3	sp|O95817	10	10	10	0	5	3	10	0	5	3	10	0	5	3	10	31	61.594	0	23.775	235720000	0	18850000	1536300	2.15E+08	0	2194300	2737600	6617800
O95155	O95155	Ubiquitin conjugation factor E4 B	UBE4B	sp|O9515	7	7	7	0	1	0	7	0	1	0	7	0	1	0	7	10.9	146.18	0	47.668	235710000	0	2239500	0	2.33E+08	0	197410	0	9601500
Q96SU4	Q96SU4	Oxysterol-binding protein-related protein 9	OSBPL9	sp|Q96SU	8	8	8	0	3	1	8	0	3	1	8	0	3	1	8	12.4	83.184	0	21.664	235090000	0	43074000	653690	1.91E+08	0	7074000	1328900	3703100
Q70UQ0	Q70UQ0	Inhibitor of nuclear factor kappa-B kinase-interact	IKBIP	sp|Q70UQ	4	4	4	1	4	2	2	1	4	2	2	1	4	2	2	12.6	39.309	0	9.6395	235090000	1.01E+08	65406000	2599000	65805000	0	3280200	3917500	1754600
Q96I25	Q96I25	Splicing factor 45	RBM17	sp|Q96I25	4	4	4	1	4	4	3	1	4	4	3	1	4	4	3	12	44.961	0	7.1308	234770000	1679100	1.27E+08	20163000	86140000	3328100	11556000	12084000	2979500
Q96EE3	Q96EE3	Nucleoporin SEH1	SEH1L	sp|Q96EE3	3	3	3	0	2	1	3	0	2	1	3	0	2	1	3	14.7	39.648	0	33.237	234430000	0	43985000	754560	1.9E+08	0	3508200	1395800	7281300
Q96J02;Q9	Q96J02	E3 ubiquitin-protein ligase Itchy homolog	ITCH	sp|Q96J02	6	6	6	0	5	0	5	0	5	0	5	0	5	0	5	11.7	102.8	0	24.58	233930000	0	29496000	0	2.04E+08	0	2982800	0	8024900
O95563	O95563	Mitochondrial pyruvate carrier 2	MPC2	sp|O9556	4	4	4	0	3	0	4	0	3	0	4	0	3	0	4	26.8	14.279	0	8.8254	233880000	0	93246000	0	1.41E+08	0	6342900	0	5305900
Q14653	Q14653	Interferon regulatory factor 3	IRF3	sp|Q1465	6	6	6	0	5	0	5	0	5	0	5	0	5	0	5	28.1	47.219	0	37.855	233790000	0	64859000	0	1.69E+08	0	5513900	0	7151000
Q96T88	Q96T88	E3 ubiquitin-protein ligase UHRF1	UHRF1	sp|Q96T8	7	7	7	0	6	4	6	0	6	4	6	0	6	4	6	11	89.813	0	12.851	233600000	0	55719000	13692000	1.64E+08	0	4756900	10144000	5964300
P17302	P17302	Gap junction alpha-1 protein	GJA1	sp|P17302	9	9	9	1	0	8	5	1	0	8	5	1	0	8	5	28.8	43.008	0	33.046	233450000	264550	0	81948000	1.51E+08	560380	0	58878000	2114600
Q99543	Q99543	DnaJ homolog subfamily C member 2;DnaJ homol	DNAJC2	sp|Q9954	6	6	6	1	6	2	5	1	6	2	5	1	6	2	5	11.4	71.996	0	48.013	233340000	856510	1.11E+08	2515000	1.19E+08	1100300	9662500	1911300	4531500
Q8NFV4	Q8NFV4	Alpha/beta hydrolase domain-containing protein	ABHD11	sp|Q8NFV	4	4	4	0	0	0	4	0	0	0	4	0	0	0	4	27.6	34.69	0	29.326	231790000	0	0	0	2.32E+08	0	0	0	9532500
Q96GD0;Q	Q96GD0	Pyridoxal phosphate phosphatase	PDXP	sp|Q96GD	7	7	7	0	7	1	3	0	7	1	3	0	7	1	3	40.2	31.698	0	28.76	231700000	0	1.99E+08	1608300	30613000	0	17733000	886250	1305100
Q9NYB0	Q9NYB0	Telomeric repeat-binding factor 2-interacting pro	TERF2IP	sp|Q9NYB	4	4	4	0	3	1	4	0	3	1	4	0	3	1	4	19.3	44.259	0	23.165	231540000	0	78494000	2459900	1.51E+08	0	5319100	2853500	5159900
Q9Y3D6	Q9Y3D6	Mitochondrial fission 1 protein	FIS1	sp|Q9Y3D	1	1	1	0	1	0	1	0	1	0	1	0	1	0	1	7.2	16.937	0.000945	3.0888	231220000	0	61447000	0	1.7E+08	0	5416500	0	6981900
O94992	O94992	Protein HEXIM1	HEXIM1	sp|O94992	3	3	3	0	3	3	2	0	3	3	2	0	3	3	2	13.9	40.623	0	7.0407	230180000	0	1.4E+08	13309000	77302000	0	11451000	9534500	3439900

Q86TS9	Q86TS9	39S ribosomal protein L52, mitochondrial	MRPL52	sp|Q86TS9	2	2	2	0	2	1	2	0	2	1	2	0	2	1	2	41.5	13.664	0	33.643	230170000	0	1.14E+08	3107400	1.13E+08	0	8343600	3872000	4590400
Q5TC12	Q5TC12	ATP synthase mitochondrial F1 complex assembly	ATPAF1	sp|Q5TC12	6	6	6	0	6	0	4	0	6	0	4	0	6	0	4	24.1	36.436	0	20.354	229600000	0	94948000	0	1.35E+08	0	10041000	0	3865700
A0A0G2JM	A0A0G2JM	HLA class II histocompatibility antigen, DR alpha c	HLA-DRA;H	tr|A0A0G2	2	2	2	0	1	0	2	0	1	0	2	0	1	0	2	11.4	28.621	0	5.1899	229500000	0	1748000	0	2.28E+08	0	0	0	8214600
P55196	P55196	Afadin	MLLT4	sp|P55196	6	6	6	0	5	4	5	0	5	4	5	0	5	4	5	5.8	206.8	0	33.447	229250000	0	50825000	5834500	1.73E+08	0	4326200	6043000	5129700
Q14254	Q14254	Flotillin-2	FLOT2	sp|Q14254	6	6	6	5	2	1	2	5	2	1	2	5	2	1	2	21.3	47.064	0	22.999	229050000	1.91E+08	10976000	1808000	25528000	1.91E+08	970540	422320	1302200
Q9BTE6	Q9BTE6	Alanyl-tRNA editing protein Aarsd1	AARSD1	sp|Q9BTE6	7	7	7	0	5	1	6	0	5	1	6	0	5	1	6	24.5	45.479	0	21.381	229040000	0	1.11E+08	450040	1.18E+08	0	5611500	1279700	8013100
Q08170	Q08170	Serine/arginine-rich splicing factor 4	SRSF4	sp|Q08170	8	4	4	2	8	7	8	1	4	3	4	1	4	3	4	15.4	56.678	0	7.3591	228970000	128510	1.8E+08	5089500	43754000	1431000	12874000	2483700	1754600
Q9NUY8	Q9NUY8	TBC1 domain family member 23	TBC1D23	sp|Q9NUY	3	3	3	0	0	0	3	0	0	0	3	0	0	0	3	7.7	78.321	0	11.274	228790000	0	0	0	2.29E+08	0	0	0	9409100
Q9H553	Q9H553	Alpha-1,3/1,6-mannosyltransferase ALG2	ALG2	sp|Q9H553	5	5	5	0	1	1	5	0	1	1	5	0	1	1	5	19.2	47.091	0	17.537	228400000	0	8396100	1246600	2.19E+08	0	535390	569590	9469100
P42892	P42892	Endothelin-converting enzyme 1	ECE1	sp|P42892	6	6	6	0	0	0	6	0	0	0	6	0	0	0	6	11.4	87.163	0	31.662	228320000	0	0	0	2.28E+08	0	0	0	9389800
P22570	P22570	NADPH:adrenodoxin oxidoreductase, mitochondr	FDXR	sp|P22570	5	5	5	0	4	0	4	0	4	0	4	0	4	0	4	13.6	53.836	0	13.553	228140000	0	54048000	0	1.74E+08	0	4613700	0	7310500
Q9H074	Q9H074	Polyadenylate-binding protein-interacting proteir	PAIP1	sp|Q9H074	5	5	5	0	4	0	5	0	4	0	5	0	4	0	5	12.7	53.524	0	8.9849	228050000	0	61129000	0	1.67E+08	0	6523700	0	5729600
O75940	O75940	Survival of motor neuron-related-splicing factor 3	SMNDC1	sp|O75940	4	4	4	0	3	3	3	0	3	3	3	0	3	3	3	29.4	26.711	0	16.264	228050000	0	1.22E+08	10962000	95576000	0	9776400	7847300	4384500
Q9NRX1	Q9NRX1	RNA-binding protein PNO1	PNO1	sp|Q9NRX	3	3	3	0	2	1	3	0	2	1	3	0	2	1	3	16.7	27.924	0	10.052	227950000	0	41561000	6035500	1.8E+08	0	4835700	4467400	5833600
P82930	P82930	28S ribosomal protein S34, mitochondrial	MRPS34	sp|P82930	6	6	6	2	6	4	5	2	6	4	5	2	6	4	5	36.2	25.65	0	17.278	227780000	18162000	1.39E+08	6173100	64317000	13191000	14886000	6611000	2532100
Q8NHP8	Q8NHP8	Putative phospholipase B-like 2;Putative phospho	PLBD2	sp|Q8NHP	5	5	5	0	2	0	5	0	2	0	5	0	2	0	5	10.7	65.471	0	15.981	227360000	0	11458000	0	2.16E+08	0	3705900	0	6183100
Q9H078	Q9H078	Caseinolytic peptidase B protein homolog	CLPB	sp|Q9H078	9	9	9	2	7	1	5	2	7	1	5	2	7	1	5	17.7	78.728	0	62.652	227110000	8681300	1.27E+08	1327000	89944000	6350400	10233000	3392000	4505800
A6NHL2	A6NHL2	Tubulin alpha chain-like 3	TUBAL3	sp|A6NHL2	4	1	1	1	4	4	4	0	1	1	1	0	1	1	1	8.3	49.908	0	17.12	227070000	0	87689000	3883900	1.36E+08	0	8405900	3049800	4456400
Q8NFW8	Q8NFW8	N-acylneuraminate cytidylyltransferase	CMAS	sp|Q8NFW	4	4	4	0	4	2	4	0	4	2	4	0	4	2	4	12.9	48.379	0	25.543	226690000	0	54121000	15709000	1.57E+08	0	4519200	11331000	5927500
O14578	O14578	Citron Rho-interacting kinase	CIT	sp|O14578	10	10	10	0	2	4	10	0	2	4	10	0	2	4	10	6.3	231.43	0	28.405	226400000	0	1884800	7140900	2.17E+08	0	2302900	6396300	5205200
Q8WUA2	Q8WUA2	Peptidyl-prolyl cis-trans isomerase-like 4	PPIL4	sp|Q8WUA	6	6	6	0	6	4	4	0	6	4	4	0	6	4	4	15.4	57.224	0	26.586	225970000	0	1.08E+08	13343000	1.04E+08	0	8463900	8843300	5497800
O75616	O75616	GTPase Era, mitochondrial	ERAL1	sp|O75616	4	4	4	0	4	1	4	0	4	1	4	0	4	1	4	11.7	48.349	0	15.039	225930000	0	75000000	1911800	1.49E+08	0	3961100	3038900	7024200
Q9NQX3	Q9NQX3	Gephyrin;Molybdopterin adenylyltransferase;Mo	GPHN	sp|Q9NQX	5	5	5	0	4	1	5	0	4	1	5	0	4	1	5	10.7	79.748	0	24.277	225790000	0	63651000	114790	1.62E+08	0	7488400	970890	3891800
O75326	O75326	Semaphorin-7A	SEMA7A	sp|O75326	6	6	6	0	0	1	6	0	0	1	6	0	0	1	6	11.9	74.823	0	27.856	225510000	0	0	2833300	2.23E+08	0	0	2932800	8129100
Q7L523;Q5	Q7L523;Q5	Ras-related GTP-binding protein A;Ras-related GT	RRAGA;RR	sp|Q7L523	4	4	4	0	3	1	4	0	3	1	4	0	3	1	4	13.7	36.566	0	9.4497	225050000	0	43757000	1232500	1.8E+08	0	4199500	2728700	5162100
Q9ULX6	Q9ULX6	A-kinase anchor protein 8-like	AKAP8L	sp|Q9ULX6	6	6	6	0	6	3	4	0	6	3	4	0	6	3	4	13.5	71.639	0	40.482	225010000	0	1.6E+08	5065600	59512000	0	11854000	5209200	2930400
Q9BXK5	Q9BXK5	Bcl-2-like protein 13	BCL2L13	sp|Q9BXK5	4	4	4	0	2	0	4	0	2	0	4	0	2	0	4	13.6	52.723	0	10.979	224980000	0	21560000	0	2.03E+08	0	4942900	0	5323400
Q4KWH8	Q4KWH8	1-phosphatidylinositol 4,5-bisphosphate phospho	PLCH1	sp|Q4KWH	1	1	1	0	1	0	1	0	1	0	1	0	1	0	1	0.5	189.22	0.003082	2.101	224580000	0	59384000	0	1.65E+08	0	5234600	0	5178300
Q92990	Q92990	Glomulin	GLMN	sp|Q92990	4	4	4	0	4	0	4	0	4	0	4	0	4	0	4	7.9	68.207	0	13.824	224310000	0	99978000	0	1.24E+08	0	10031000	0	3895100
O75592	O75592	E3 ubiquitin-protein ligase MYCBP2	MYCBP2	sp|O75592	9	9	9	1	6	5	8	1	6	5	8	1	6	5	8	2.7	513.63	0	11.548	224190000	1289700	60203000	20332000	1.42E+08	4150000	4357300	13568000	4038800
Q9BY77	Q9BY77	Polymerase delta-interacting protein 3	POLDIP3	sp|Q9BY77	10	10	10	4	8	5	5	4	8	5	5	4	8	5	5	33.5	46.089	0	38.087	224000000	42026000	1.2E+08	6833600	55219000	34405000	10427000	11810000	2763900
Q9HA64	Q9HA64	Ketosamine-3-kinase	FN3KRP	sp|Q9HA64	6	6	6	0	6	4	6	0	6	4	6	0	6	4	6	24.6	34.412	0	18.999	223840000	0	86264000	6658300	1.31E+08	0	7318900	4293100	5850400
O60645	O60645	Exocyst complex component 3	EXOC3	sp|O60645	7	7	7	0	3	2	7	0	3	2	7	0	3	2	7	12.2	85.566	0	22.821	223830000	0	23649000	4152800	1.96E+08	0	3185400	3712100	6039800
P26232	P26232	Catenin alpha-2	CTNNA2	sp|P26232	4	1	1	0	4	2	4	0	1	0	1	0	1	0	1	5.5	105.31	0	14.936	223800000	0	70414000	0	1.53E+08	0	6746400	0	5768600
P29323;P5	P29323	Ephrin type-B receptor 2	EPHB2	sp|P29323	8	8	8	0	0	1	8	0	0	1	8	0	0	1	8	10	117.49	0	20.672	223440000	0	0	51634000	1.72E+08	0	0	34838000	6925800
P56378	P56378	6.8 kDa mitochondrial proteolipid	MP68	sp|P56378	3	3	3	0	2	0	3	0	2	0	3	0	2	0	3	48.3	6.662	0.000246	3.6573	222930000	0	1.1E+08	0	1.12E+08	0	11397000	0	2964100
P98175	P98175	RNA-binding protein 10	RBM10	sp|P98175	8	8	8	2	7	5	7	2	7	5	7	2	7	5	7	15.4	103.53	0	22.812	222850000	4338500	75987000	18045000	1.24E+08	5680400	7541700	12417000	2643100
Q7Z7A4	Q7Z7A4	PX domain-containing protein kinase-like protein	PXK	sp|Q7Z7A4	5	5	5	0	1	0	5	0	1	0	5	0	1	0	5	14.4	64.949	0	14.334	222730000	0	6661700	0	2.16E+08	0	0	0	8886100
Q8NB90	Q8NB90	Spermatogenesis-associated protein 5	SPATA5	sp|Q8NB90	8	7	7	1	7	6	8	0	6	5	7	0	6	5	7	13.4	97.903	0	19.864	222620000	0	71725000	19604000	1.31E+08	0	5927000	13576000	5392200
Q9H814	Q9H814	Phosphorylated adapter RNA export protein	PHAX	sp|Q9H814	8	8	8	0	8	4	6	0	8	4	6	0	8	4	6	30.7	44.402	0	16.872	222510000	0	93281000	7009900	1.22E+08	0	5599900	8201000	4158800
Q8TCE6;Q(	Q8TCE6;Q(	Protein FAM45A;Protein FAM45B	FAM45A;F.	sp|Q8TCE6	4	4	4	0	2	1	4	0	2	1	4	0	2	1	4	18.5	40.513	0	23.956	222500000	0	44108000	1330100	1.77E+08	0	2730500	2142800	7190300
Q96EY8	Q96EY8	Cob(I)yrinic acid a,c-diamide adenosyltransferase,	MMAB	sp|Q96EY8	4	4	4	0	4	0	4	0	4	0	4	0	4	0	4	20	27.388	0	9.4084	222440000	0	1.4E+08	0	82422000	0	12001000	0	3731500
Q70E73	Q70E73	Ras-associated and pleckstrin homology domains-	RAPH1	sp|Q70E73	8	8	8	0	2	1	8	0	2	1	8	0	2	1	8	12.3	135.25	0	33.73	222330000	0	5364900	548050	2.16E+08	0	621400	439450	8680800
O14777	O14777	Kinetochore protein NDC80 homolog	NDC80	sp|O14777	10	10	10	0	4	0	10	0	4	0	10	0	4	0	10	19.8	73.912	0	19.477	221780000	0	17802000	0	2.04E+08	0	3622300	0	6335800
Q5T6F2	Q5T6F2	Ubiquitin-associated protein 2	UBAP2	sp|Q5T6F2	10	10	10	0	8	0	9	0	8	0	9	0	8	0	9	18.4	117.11	0	54.339	221630000	0	55393000	0	1.66E+08	0	5485400	0	6213900
P52630	P52630	Signal transducer and activator of transcription 2	STAT2	sp|P52630	4	4	4	0	2	1	4	0	2	1	4	0	2	1	4	9.3	97.915	0	41.502	221270000	0	16737000	2638200	2.02E+08	0	2132600	2096900	7321900
Q8IVL6	Q8IVL6	Prolyl 3-hydroxylase 3	LEPREL2	sp|Q8IVL6	6	6	6	0	2	0	6	0	2	0	6	0	2	0	6	14.7	81.836	0	37.633	221040000	0	26881000	0	1.94E+08	0	4366200	0	5988100
Q14161	Q14161	ARF GTPase-activating protein GIT2	GIT2	sp|Q14161	4	4	3	0	3	1	4	0	3	1	4	0	2	1	3	9.6	84.542	0	21.157	220850000	0	40243000	2473400	1.78E+08	0	2947100	5694900	3893500
Q9BQ39	Q9BQ39	ATP-dependent RNA helicase DDX50	DDX50	sp|Q9BQ39	7	5	5	1	7	7	7	1	5	5	5	1	5	5	5	13.3	82.564	0	14.608	220820000	451240	45560000	21581000	1.53E+08	2942400	2898900	15639000	3791000
Q9NXF1	Q9NXF1	Testis-expressed sequence 10 protein	TEX10	sp|Q9NXF1	7	7	7	0	7	1	1	0	7	1	1	0	7	1	1	9.7	105.67	0	22.799	220760000	0	1.94E+08	355000	26136000	0	17793000	206510	438360
Q96D46	Q96D46	60S ribosomal export protein NMD3	NMD3	sp|Q96D46	4	4	4	0	4	1	4	0	4	1	4	0	4	1	4	13.5	57.603	0	15.097	220660000	0	37193000	1412400	1.82E+08	0	2466300	4374300	4874200
Q9Y3C6	Q9Y3C6	Peptidyl-prolyl cis-trans isomerase-like 1	PPIL1	sp|Q9Y3C6	5	5	5	0	5	2	3	0	5	2	3	0	5	2	3	31.3	18.237	0	10.658	220480000	0	1.9E+08	7727700	22615000	0	13625000	5992400	2718700
P11171	P11171	Protein 4.1	EPB41	sp|P11171	13	13	12	0	13	1	2	0	13	1	2	0	12	1	2	24.2	97.016	0	23.091	220480000	0	2.09E+08	75189	11080000	0	18394000	0	514010
Q9Y606	Q9Y606	tRNA pseudouridine synthase A, mitochondrial	PUS1	sp|Q9Y606	4	4	4	0	4	0	3	0	4	0	3	0	4	0	3	11.5	47.47	0	17.032	220300000	0	1.39E+08	0	81341000	0	9315600	0	6279000
Q14240	Q14240	Eukaryotic initiation factor 4A-II;Eukaryotic initiati	EIF4A2	sp|Q14240	15	3	3	6	12	8	14	1	2	0	3	1	2	0	3	42.8	46.402	0	50.357	219370000	4470100	30927000	0	1.84E+08	6685600	3381700	0	4695000
Q12769	Q12769	Nuclear pore complex protein Nup160	NUP160	sp|Q12769	6	6	6	1	1	0	5	1	1	0	5	1	1	0	5	6.5	162.12	0	32.501	219330000	9570500	5354500	0	2.04E+08	0	2189700	0	6688500
P07947;P0	P07947;P0	Tyrosine-protein kinase Yes;Tyrosine-protein kina	YES1;FYN	sp|P07947	4	2	2	1	4	1	4	1	2	0	2	1	2	0	2	8.5	60.801	0	10.641	219140000	378580	67488000	0	1.51E+08	1455600	3814300	0	7278900
P14635	P14635	G2/mitotic-specific cyclin-B1	CCNB1	sp|P14635	5	5	5	0	3	1	4	0	3	1	4	0	3	1	4	17.8	48.337	0	8.3548	218510000	0	1.06E+08	802510	1.12E+08	0	5796900	3633600	5028700
Q6UWE0	Q6UWE0	E3 ubiquitin-protein ligase LRSAM1	LRSAM1	sp|Q6UWE	7	7	7	0	5	1	7	0	5	1	7	0	5	1	7	14.8	83.593	0	33.974	217520000	0	41556000	564960	1.75E+08	0	2581800	649110	8025400
Q12972	Q12972	Nuclear inhibitor of protein phosphatase 1;Activa	PPP1R8	sp|Q12972	4	4	4	0	4	0	2	0	4	0	2	0	4	0	2	21.4	38.478	0	21.307	216890000	0	1.57E+08	0	59853000	0	12007000	0	4296900
O60784	O60784	Target of Myb protein 1	TOM1	sp|O60784	6	6	6	0	2	0	6	0	2	0	6	0	2	0	6	20.9	53.818	0	21.03	216430000	0	8930700	0	2.08E+08	0	1979100	0	7341800
Q9H267	Q9H267	Vacuolar protein sorting-associated protein 33B	VPS33B	sp|Q9H267	6	6	6	0	3	0	6	0	3	0	6	0	3	0	6	11.8	70.584	0	16.197	216340000	0	34249000	0	1.82E+08	0	3995600	0	6512100
Q8N0U8	Q8N0U8	Vitamin K epoxide reductase complex subunit 1-li	VKORC1L1	sp|Q8N0U	2	2	2	1	1	0	2	1	1	0	2	1	1	0	2	11.9	19.835	0	4.2409	216150000	2858000	48553000	0	1.65E+08	4921600	4597200	0	4393900
Q6RW13	Q6RW13	Type-1 angiotensin II receptor-associated protein	AGTRAP	sp|Q6RW1	1	1	1	0	1	1	1	0	1	1	1	0	1	1	1	13.8	17.419	0	21.806	215630000	0	25976000	7076800	1.83E+08	0	2556600	3743300	8254000
P54132	P54132	Bloom syndrome protein	BLM	sp|P54132	11	11	11	4	4	11	1	4	4	11	1	4	4	11	1	9.2	159	0	24.059	215420000	28779000	21547000	43560000	1.22E+08	42046000	1110700	14500000	7291900
Q9UFN0	Q9UFN0	Protein NipSnap homolog 3A	NIPSNAP3/	sp|Q9UFN	5	5	5	0	5	0	3	0	5	0	3	0	5	0	3	32.8	28.466	0	31.534	215400000	0	1.5E+08	0	65393000	0	11985000	0	3927700
Q99653	Q99653	Calcineurin B homologous protein 1	CHP1	sp|Q99653	5	5	5	0	1	4	5	0	1	4	5	0	1	4	5	29.7	22.456	0	30.494	215150000	0	4644700	11416000	1.99E+08	0	1838500	5371000	8688700
P83881	P83881	60S ribosomal protein L36a	RPL36A	sp|P83881	7	2	2	0	6	7	7	0	2	2	2	0	2	2	2	45.3	12.441	0	4.3043	215150000	0	38060000	49094000	1.28E+08	0	427560	39689000	1492700
Q676U5	Q676U5	Autophagy-related protein 16-1	ATG16L1	sp|Q676U5	7	7	7	0	5	0	7	0	5	0	7	0	5	0	7	17.6	68.264	0	32.015	214720000	0	30987000	0	1.84E+08	0	5359400	0	4928000
P56589	P56589	Peroxisomal biogenesis factor 3	PEX3	sp|P56589	2	2	2	0	2	0	2	0	2	0	2	0	2	0	2	11.8	42.139	0	8.7752	214710000	0	45712000	0	1.69E+08	0	4438300	0	6541400
Q9P2R7	Q9P2R7	Succinyl-CoA ligase [ADP-forming] subunit beta, n	SUCLA2	sp|Q9P2R7	5	5	5	0	5	0	2	0	5	0	2	0	5	0	2	17.5	50.317	0	17.583	214530000	0	1.62E+08	0	52752000	0	11362000	0	5068800
O14662	O14662	Syntaxin-16	STX16	sp|O14662	5	5	5	0	4	1	5	0	4	1	5	0	4	1	5	22.5	37.031	0	14.563	214470000	0	43664000	622490	1.7E+08	0	3377200	1415400	6473600
Q07812	Q07812	Apoptosis regulator BAX	BAX	sp|Q07812	5	5	5	1	4	0	4	1	4	0	4	1	4	0	4	35.9	21.184	0	15.322	214440000	1714900	59702000	0	1.53E+08	5568300	3355800	0	4346700
A4D1E9	A4D1E9	GTP-binding protein 10	GTPBP10	sp|A4D1E9	6	6	6	0	4	4	6	0	4	4	6	0	4	4	6	18.3	42.932	0	20.851	214310000	0	17673000	14058000	1.83E+08	0	4201000	9312000	5000300
Q5SRE5	Q5SRE5	Nucleoporin NUP188 homolog	NUP188	sp|Q5SRE5	5	5	5	0	4	0	5	0	4	0	5	0	4	0	5	4.5	196.04	0	10.729	214170000	0	31814000	0	1.82E+08	0	4115700	0	6188100
Q9BXB4;Q(	Q9BXB4	Oxysterol-binding protein-related protein 11	OSBPL11	sp|Q9BXB4	5	5	5	0	5	0	4	0	5	0	4	0	5	0	4	14.3	83.642	0	21.075	214090000	0	73373000	0	1.41E+08	0	6046600	0	6208200
P62633	P62633	Cellular nucleic acid-binding protein	CNBP	sp|P62633	4	4	4	0	3	2	4	0	3	2	4	0	3	2	4	28.2	19.463	0	11.15	213870000	0	48038000	5442000	1.6E+08	0	8032000	2742200	3713300
Q71UM5	Q71UM5	40S ribosomal protein S27-like	RPS27L	sp|Q71UM	4	1	1	1	3	3	4	1	1	1	1	1	1	1	1	39.3	9.4771	0	8.3273	213860000	1266700	49846000	15699000	1.47E+08	3084100	4128600	9559900	5275100
Q6P9B6	Q6P9B6	TLD domain-containing protein 1	TLDC1	sp|Q6P9B6	4	4	4	0	3	1	4	0	3	1	4	0	3	1	4	14.9	50.993	0	12.981	213800000	0	42500000	3769000	1.68E+08	0	3503600	4698000	4967400
Q9P2T1;P3	Q9P2T1	GMP reductase 2	GMPR2	sp|Q9P2T1	6	6	6	0	3	0	5	0	3	0	5	0	3	0	5	28.2	37.874	0	40.938	213780000	0	85376000	0	1.28E+08	0	8623900	0	4182600
Q96K76	Q96K76	Ubiquitin carboxyl-terminal hydrolase 47	USP47	sp|Q96K76	10	10	10	1	5	0	10	1	5	0	10	1	5	0	10	11.7	157.31	0	28.251	213650000	1180500	36475000	0	1.76E+08	0	4790800	0	5662300
Q96RS6	Q96RS6	NudC domain-containing protein 1	NUDCD1	sp|Q96RS6	7	7	7	0	6	0	3	0	6	0	3	0	6	0	3	18.4	66.755	0	40.216	213580000	0	1.07E+08	0	1.07E+08	0	5629600	0	8179900
Q13636	Q13636	Ras-related protein Rab-31	RAB31	sp|Q13636	6	6	5	0	0	0	6	0	0	0	6	0	0	0	5	39.7	21.569	0	19.174	213370000	0	0	0	2.13E+08	0	0	0	8775000
Q8N6R0	Q8N6R0	Methyltransferase-like protein 13	METTL13	sp|Q8N6R0	4	4	4	0	4	2	3	0	4	2	3	0	4	2	3	7.9	78.767	0	16.039	213270000	0	63363000	2000800	1.48E+08	0	4601100	4288200	4123200
Q02241	Q02241	Kinesin-like protein KIF23	KIF23	sp|Q02241	8	8	8	0	3	6	8	0	3	6	8	0	3	6	8	12.1	110.06	0	19.265	213200000	0	20061000	16311000	1.77E+08	0	3206000	11625000	5170200
Q5T0F9	Q5T0F9	Coiled-coil and C2 domain-containing protein 1B	CC2D1B	sp|Q5T0F9	4	4	4	0	2	0	4	0	2	0	4	0	2	0	4	10	94.223	0	36.787	213090000	0	15270000	0	1.98E+08	0	1895200	0	7586200
Q9BQC3	Q9BQC3	Diphthamide biosynthesis protein 2	DPH2	sp|Q9BQC	6	6	6	1	6	0	4	1	6	0	4	1	6	0	4	20.4	52.082	0	17.327	212590000	9350200	1.05E+08	0	97919000	0	10212000	0	3098900
Q9BZK7;O(	Q9BZK7	F-box-like/WD repeat-containing protein TBL1XR1	TBL1XR1	sp|Q9BZK7	7	7	7	0	4	1	7	0	4	1	7	0	4	1	7	20.2	55.594	0	35.089	212050000	0	33848000	402830	1.78E+08	0	4412600	2017800	4136000
P37268	P37268	Squalene synthase	FDFT1	sp|P37268	5	5	5	0	2	1	5	0	2	1	5	0	2	1	5	12.9	48.115	0	17.507	211630000	0	15713000	166510	1.96E+08	0	749590	166500	8631300

Q96C90	Q96C90	Protein phosphatase 1 regulatory subunit 14B	PPP1R14B	sp|Q96C9(	3	3	3	1	3	0	2	1	3	0	2	1	3	0	2	43.5	15.911	0	25.301	211130000	3933000	84337000	0	1.23E+08	6806600	3858900	0	5754400
Q5T2E6	Q5T2E6	UPF0668 protein C10orf76	C10orf76	sp|Q5T2E6	5	5	5	0	2	0	5	0	2	0	5	0	2	0	5	12.8	78.71	0	36.69	210900000	0	4870000	0	2.06E+08	0	743480	0	8159000
Q9C0D9	Q9C0D9	Ethanolaminephosphotransferase 1	EPT1	sp|Q9C0D9	2	2	2	0	1	1	2	0	1	1	2	0	1	1	2	9.8	45.228	0	14.995	210790000	0	34470000	5406500	1.71E+08	0	4460700	5333500	3906200
Q9BWH6	Q9BWH6	RNA polymerase II-associated protein 1	RPAP1	sp|Q9BWH	8	8	8	0	6	0	8	0	6	0	8	0	6	0	8	9.6	152.75	0	21.922	210770000	0	45868000	0	1.65E+08	0	4490800	0	6334000
Q9ULX3	Q9ULX3	RNA-binding protein NOB1	NOB1	sp|Q9ULX3	5	5	5	1	3	4	3	1	3	4	3	1	3	4	3	17.7	46.674	0	17.62	210630000	36188000	33256000	14763000	1.26E+08	29308000	7255800	7651300	10024000
P29992;O9	P29992	Guanine nucleotide-binding protein subunit alpha	GNA11	sp|P29992	6	6	5	0	3	1	5	0	3	1	5	0	2	0	4	24.8	42.123	0	18.946	210320000	0	61650000	1163000	1.48E+08	0	0	0	5776200
Q8WWC4	Q8WWC4	Uncharacterized protein C2orf47, mitochondrial	C2orf47	sp|Q8WW	2	2	2	0	2	0	2	0	2	0	2	0	2	0	2	7.2	32.544	0	5.4616	210280000	0	86662000	0	1.24E+08	0	7031700	0	5691400
Q92466	Q92466	DNA damage-binding protein 2	DDB2	sp|Q92466	3	3	3	0	1	0	3	0	1	0	3	0	1	0	3	11.7	47.863	0	13.416	210240000	0	67857000	0	1.42E+08	0	7898600	0	3938700
Q9NUQ7	Q9NUQ7	Ufm1-specific protease 2	UFSP2	sp|Q9NUC	5	5	5	0	2	1	5	0	2	1	5	0	2	1	5	18.6	53.261	0	15.306	210180000	0	23605000	3548200	1.83E+08	0	3017300	3817200	5157600
O43148	O43148	mRNA cap guanine-N7 methyltransferase	RNMT	sp|O43148	6	6	6	0	4	2	6	0	4	2	6	0	4	2	6	18.3	54.843	0	16.95	210080000	0	16909000	5187100	1.88E+08	0	2616700	2446500	7644100
P48723	P48723	Heat shock 70 kDa protein 13	HSPA13	sp|P48723	5	5	5	0	5	0	4	0	5	0	4	0	5	0	4	12.7	51.927	0	12.193	210050000	0	97387000	0	1.13E+08	0	8154200	0	5063600
Q7L2J0	Q7L2J0	7SK snRNA methylphosphate capping enzyme	MEPCE	sp|Q7L2J0	4	4	4	1	4	3	3	1	4	3	3	1	4	3	3	12.3	74.354	0	13.111	209190000	612410	60961000	9129200	1.38E+08	4676100	3870300	5473300	3796600
O14530	O14530	Thioredoxin domain-containing protein 9	TXNDC9	sp|O1453(	4	4	4	0	3	1	4	0	3	1	4	0	3	1	4	22.1	26.534	0	33.686	209090000	0	18154000	2081800	1.89E+08	0	1798900	1293900	7673300
Q9Y2Q9	Q9Y2Q9	28S ribosomal protein S28, mitochondrial	MRPS28	sp|Q9Y2Q9	3	3	3	0	3	2	3	0	3	2	3	0	3	2	3	23.5	20.843	0	16.068	208530000	0	94844000	7506200	1.06E+08	0	5792700	6862500	5116000
Q9NP92	Q9NP92	28S ribosomal protein S30, mitochondrial	MRPS30	sp|Q9NP9	4	4	4	0	4	2	4	0	4	2	4	0	4	2	4	13	50.364	0	7.1401	207890000	0	50458000	4802500	1.53E+08	0	5015000	5126700	3810500
O75475	O75475	PC4 and SFRS1-interacting protein	PSIP1	sp|O75475	7	6	6	0	6	3	6	0	5	3	5	0	5	3	5	12.5	60.103	0	16.253	207600000	0	1.15E+08	4159500	88858000	0	7614600	6476400	2459000
P12109	P12109	Collagen alpha-1(VI) chain	COL6A1	sp|P12109	6	6	6	0	5	1	6	0	5	1	6	0	5	1	6	7.3	108.53	0	18.624	206900000	0	19601000	507830	1.87E+08	0	1710000	2574500	5466300
P61962	P61962	DDB1- and CUL4-associated factor 7	DCAF7	sp|P61962	5	5	5	0	5	5	1	0	5	5	1	0	5	5	1	17	38.926	0	13.094	206850000	0	1.53E+08	31125000	22457000	0	12076000	14203000	7263100
Q8TD55	Q8TD55	Pleckstrin homology domain-containing family O r	PLEKHO2	sp|Q8TD5	6	6	6	0	0	6	3	0	0	6	3	0	0	6	3	19.8	53.349	0	40.758	206790000	0	0	128440000	78346000	0	0	85327000	4207200
Q96EK9	Q96EK9	Protein KTI12 homolog	KTI12	sp|Q96EK9	7	7	7	0	4	1	7	0	4	1	7	0	4	1	7	25.7	38.615	0	23.073	206690000	0	20241000	534500	1.86E+08	0	1787500	497070	7504700
P23634	P23634	Plasma membrane calcium-transporting ATPase 4	ATP2B4	sp|P23634	15	7	7	1	9	3	15	1	2	1	7	1	2	1	7	16.3	137.92	0	14.039	206520000	3526600	5439700	10339000	1.87E+08	0	3026900	5950600	6149000
P02751	P02751	Fibronectin;Anastellin;Ugl-Y1;Ugl-Y2;Ugl-Y3	FN1	sp|P02751	10	10	10	2	0	5	9	2	0	5	9	2	0	5	9	7.2	272.32	0	33.607	206490000	6807200	0	18134000	1.82E+08	9027600	0	10176000	7255800
P51809	P51809	Vesicle-associated membrane protein 7	VAMP7	sp|P51809	3	3	3	0	3	0	3	0	3	0	3	0	3	0	3	18.2	24.935	0	9.1567	206460000	0	1.14E+08	0	92456000	0	9865400	0	3985800
Q9BPU6	Q9BPU6	Dihydropyrimidinase-related protein 5	DPYSL5	sp|Q9BPU	7	7	7	0	7	2	0	0	7	2	0	0	7	2	0	23.4	61.421	0	41.742	206220000	0	2.05E+08	1093000	0	0	18284000	532500	0
O75569	O75569	Interferon-inducible double-stranded RNA-depen	PRKRA	sp|O75569	6	6	6	2	6	3	3	2	6	3	3	2	6	3	3	29.1	34.404	0	22.166	206000000	9130200	95943000	15563000	85362000	10552000	5439100	12210000	3355300
P60520	P60520	Gamma-aminobutyric acid receptor-associated pr	GABARAPL	sp|P60520	4	4	4	0	4	0	4	0	4	0	4	0	4	0	4	29.9	13.667	0	10.976	205800000	0	1.04E+08	0	1.02E+08	0	9229300	0	4120800
Q13505	Q13505	Metaxin-1	MTX1	sp|Q13505	6	6	6	0	4	1	6	0	4	1	6	0	4	1	6	20	51.462	0	20.169	205580000	0	44579000	1233900	1.6E+08	0	3682600	3195700	4451000
Q6NZ67;Q(	Q6NZ67;Q(	Mitotic-spindle organizing protein 2B;Mitotic-spir	MZT2B;MZ	sp|Q6NZ6	5	5	5	0	4	1	4	0	4	1	4	0	4	1	4	56.3	16.225	0	14.072	205450000	0	77410000	3918100	1.24E+08	0	5712000	0	5669400
Q9NYF8	Q9NYF8	Bcl-2-associated transcription factor 1	BCLAF1	sp|Q9NYF8	10	10	10	2	10	6	6	2	10	6	6	2	10	6	6	12.7	106.12	0	15.808	205250000	7763200	1.15E+08	13277000	69067000	14724000	7021300	6202400	1727900
Q13325;Q5	Q13325	Interferon-induced protein with tetratricopeptide	IFIT5	sp|Q13325	3	3	3	0	3	0	3	0	3	0	3	0	3	0	3	6.2	55.846	0	18.392	205210000	0	48230000	0	1.57E+08	0	4344800	0	6362400
Q96JJ7	Q96JJ7	Protein disulfide-isomerase TMX3	TMX3	sp|Q96JJ7	4	4	4	0	4	1	4	0	4	1	4	0	4	1	4	16.3	51.871	0	11.487	205040000	0	39117000	1162100	1.65E+08	0	2474900	2024900	6505300
Q9BX40	Q9BX40	Protein LSM14 homolog B	LSM14B	sp|Q9BX4(	6	6	6	1	3	5	4	1	3	5	4	1	3	5	4	28.3	42.07	0	26.712	204730000	29545000	54747000	33442000	86997000	30267000	8728600	13020000	8406000
Q14997	Q14997	Proteasome activator complex subunit 4	PSME4	sp|Q14997	9	9	9	1	7	0	6	1	7	0	6	1	7	0	6	6.3	211.33	0	30.315	204530000	5946600	68172000	0	1.3E+08	6667000	4812100	0	5840100
P12235	P12235	ADP/ATP translocase 1	SLC25A4	sp|P12235	11	3	3	5	11	9	9	1	3	3	3	1	3	3	3	36.6	33.064	0	7.6727	204460000	293150	1.4E+08	9533600	54362000	1085900	15339000	3091300	1783700
Q9Y399	Q9Y399	28S ribosomal protein S2, mitochondrial	MRPS2	sp|Q9Y399	7	7	7	2	7	3	5	2	7	3	5	2	7	3	5	19.6	33.249	0	13.273	204230000	8917800	99990000	5420200	89898000	10025000	7074000	4007900	3964200
Q7Z7F7	Q7Z7F7	39S ribosomal protein L55, mitochondrial	MRPL55	sp|Q7Z7F7	2	2	2	0	2	1	2	0	2	1	2	0	2	1	2	18.8	15.128	0	5.1522	204160000	0	1.13E+08	1507600	89459000	0	7846300	3714400	3109400
O00291;O7	O00291	Huntingtin-interacting protein 1	HIP1	sp|O00291	6	6	6	0	1	0	6	0	1	0	6	0	1	0	6	8.8	116.22	0	76.248	203710000	0	8710200	0	1.95E+08	0	767790	0	8019700
P46199	P46199	Translation initiation factor IF-2, mitochondrial	MTIF2	sp|P46199	7	7	7	0	5	0	7	0	5	0	7	0	5	0	7	13.5	81.316	0	20.838	203520000	0	58454000	0	1.45E+08	0	5816800	0	5301900
Q9Y6A9	Q9Y6A9	Signal peptidase complex subunit 1	SPCS1	sp|Q9Y6A9	2	2	2	1	2	1	2	1	2	1	2	1	2	1	2	10.1	18.298	0	7.9797	203200000	1328800	67603000	1041400	1.33E+08	2421900	6322200	1757600	2965200
Q14137	Q14137	Ribosome biogenesis protein BOP1	BOP1	sp|Q14137	6	6	6	0	5	2	3	0	5	2	3	0	5	2	3	16	83.629	0	37.281	202860000	0	81210000	7341900	1.14E+08	0	4766900	7502100	4524200
Q05086	Q05086	Ubiquitin-protein ligase E3A	UBE3A	sp|Q05086	11	11	11	1	7	1	9	1	7	1	9	1	7	1	9	16.8	100.69	0	57.262	202570000	22911000	63160000	2222200	1.14E+08	16371000	7787800	4500600	6012900
Q9BQ95	Q9BQ95	Evolutionarily conserved signaling intermediate ir	ECSIT	sp|Q9BQ9	4	4	4	0	3	1	4	0	3	1	4	0	3	1	4	19.3	49.148	0	23.469	202390000	0	61300000	4082600	1.37E+08	0	5075200	4372600	4333600
Q92692	Q92692	Nectin-2	PVRL2	sp|Q92692	2	2	2	0	1	0	2	0	1	0	2	0	1	0	2	5.8	57.741	0	7.2769	202250000	0	19953000	0	1.82E+08	0	0	0	7497100
Q9H270	Q9H270	Vacuolar protein sorting-associated protein 11 ho	VPS11	sp|Q9H27(	4	4	4	0	3	1	4	0	3	1	4	0	3	1	4	7.1	107.84	0	42.995	201940000	0	45170000	573370	1.56E+08	0	4285600	3055700	3449400
P49418	P49418	Amphiphysin	AMPH	sp|P49418	7	6	6	0	0	0	7	0	0	0	6	0	0	0	6	21.3	76.256	0	19.619	201920000	0	0	0	2.02E+08	0	0	0	8304300
Q9BUB5	Q9BUB5	MAP kinase-interacting serine/threonine-protein	MKNK1	sp|Q9BUB	1	1	1	0	1	1	1	0	1	1	1	0	1	1	1	2.6	51.342	0.001164	2.8673	201220000	0	1.09E+08	20689000	71307000	0	10923000	13126000	2414500
Q9H0U3	Q9H0U3	Magnesium transporter protein 1	MAGT1	sp|Q9H0U	3	3	3	0	1	1	3	0	1	1	3	0	1	1	3	10.1	38.036	0	28.009	200940000	0	13203000	6046700	1.82E+08	0	2200800	6393300	3963800
Q6UXN9	Q6UXN9	WD repeat-containing protein 82	WDR82	sp|Q6UXN	7	7	7	1	5	3	6	1	5	3	6	1	5	3	6	28.4	35.079	0	18.946	200480000	887970	1.27E+08	2520900	70260000	2791000	4439000	4547000	4872700
Q8IUD2	Q8IUD2	ELKS/Rab6-interacting/CAST family member 1	ERC1	sp|Q8IUD2	9	9	9	1	3	3	8	1	3	3	8	1	3	3	8	14.6	128.08	0	25.284	200420000	33457000	16228000	12545000	1.38E+08	21947000	4476200	17536000	5041500
Q6P1J9	Q6P1J9	Parafibromin	CDC73	sp|Q6P1J9	8	8	8	0	7	2	8	0	7	2	8	0	7	2	8	16.6	60.576	0	19.602	200240000	0	71750000	985510	1.28E+08	0	6886600	0	4681700
Q7KZN9	Q7KZN9	Cytochrome c oxidase assembly protein COX15 hc	COX15	sp|Q7KZN9	4	4	4	0	3	1	4	0	3	1	4	0	3	1	4	13.9	46.03	0	12.426	200230000	0	25956000	983740	1.73E+08	0	4750500	0	4664100
O95716	O95716	Ras-related protein Rab-3D	RAB3D	sp|O95716	4	3	3	0	0	1	4	0	0	1	3	0	0	1	3	20.5	24.267	0	9.5138	200160000	0	0	977560	1.99E+08	0	0	1046300	7802300
O75368	O75368	SH3 domain-binding glutamic acid-rich-like protei	SH3BGRL	sp|O75368	4	4	4	0	4	0	2	0	4	0	2	0	4	0	2	61.4	12.774	0	15.171	199980000	0	98017000	0	1.02E+08	0	7729900	0	5103500
Q9Y2T2	Q9Y2T2	AP-3 complex subunit mu-1	AP3M1	sp|Q9Y2T2	4	4	4	0	3	2	3	0	3	2	3	0	3	2	3	12.7	46.939	0	6.531	199780000	0	48985000	2162700	1.49E+08	0	4297900	2988200	4597600
P62312	P62312	U6 snRNA-associated Sm-like protein LSm6	LSM6	sp|P62312	5	5	5	0	4	0	5	0	4	0	5	0	4	0	5	46.2	9.1275	0	8.5183	199740000	0	1.23E+08	0	76675000	0	8901600	0	4411400
Q9NUP9	Q9NUP9	Protein lin-7 homolog C	LIN7C	sp|Q9NUP	6	6	3	0	6	1	6	0	6	1	6	0	3	0	3	35	21.834	0	15.86	199710000	0	1.11E+08	197490	88259000	0	10830000	232150	2506600
O60828	O60828	Polyglutamine-binding protein 1	PQBP1	sp|O60828	4	4	4	0	4	3	3	0	4	3	3	0	4	3	3	29.4	30.472	0	18.349	199540000	0	1.02E+08	7347000	90089000	0	8889100	4999100	3754400
O15127	O15127	Secretory carrier-associated membrane protein 2	SCAMP2	sp|O15127	2	2	2	0	2	0	2	0	2	0	2	0	2	0	2	9.7	36.648	0	29.842	199460000	0	84024000	0	1.15E+08	0	7138100	0	5015900
O14920	O14920	Inhibitor of nuclear factor kappa-B kinase subunit	IKBKB	sp|O1492(	6	6	5	1	4	0	6	1	4	0	6	1	3	0	5	11	86.563	0	6.7347	198900000	83944000	21700000	0	93254000	73757000	12381000	0	3554900
Q9UJF2	Q9UJF2	Ras GTPase-activating protein nGAP	RASAL2	sp|Q9UJF2	5	5	5	0	2	4	5	0	2	4	5	0	2	4	5	7	128.56	0	15.774	198810000	0	6222000	10298000	1.82E+08	0	2877800	7030200	5057200
O43920	O43920	NADH dehydrogenase [ubiquinone] iron-sulfur pr	NDUFS5	sp|O4392(	4	4	4	1	4	1	4	1	4	1	4	1	4	1	4	34	12.517	0	6.6784	198650000	7393500	91897000	949820	98414000	6683700	3756400	6982300	2757300
Q13951	Q13951	Core-binding factor subunit beta	CBFB	sp|Q13951	2	2	2	0	2	1	2	0	2	1	2	0	2	1	2	14.3	21.508	0	7.4807	198250000	0	35301000	695420	1.62E+08	0	2565300	1652800	6034000
Q96D71;Q	Q96D71	RalBP1-associated Eps domain-containing protein	REPS1	sp|Q96D7	7	7	7	0	6	0	7	0	6	0	7	0	6	0	7	14.6	86.661	0	17.211	198200000	0	97250000	0	1.01E+08	0	8750200	0	3974100
P57678	P57678	Gem-associated protein 4	GEMIN4	sp|P57678	8	8	8	0	6	4	7	0	6	4	7	0	6	4	7	11	120.04	0	32.124	198180000	0	71553000	5310700	1.21E+08	0	4985200	6042900	3837400
O43709	O43709	Probable 18S rRNA (guanine-N(7))-methyltransfer	WBSCR22	sp|O43709	3	3	3	0	2	2	3	0	2	2	3	0	2	2	3	17.4	31.88	0	18.499	198070000	0	7655600	5146100	1.85E+08	0	1105800	6816200	3830100
Q12768	Q12768	WASH complex subunit strumpellin	KIAA0196	sp|Q12768	5	5	5	1	2	0	5	1	2	0	5	1	2	0	5	5.1	134.28	0	16.501	198030000	1089600	20511000	0	1.76E+08	2205400	2294800	0	5653400
Q16563	Q16563	Synaptophysin-like protein 1	SYPL1	sp|Q16563	2	2	2	0	2	0	2	0	2	0	2	0	2	0	2	13.9	28.565	0	12.44	198020000	0	46252000	0	1.52E+08	0	4444400	0	5874100
Q96DG6	Q96DG6	Carboxymethylenebutenolidase homolog	CMBL	sp|Q96DG	6	6	6	1	6	1	5	1	6	1	5	1	6	1	5	25.7	28.048	0	18.096	197870000	1229000	89921000	4495500	1.02E+08	0	5267900	5543500	4340000
P38571	P38571	Lysosomal acid lipase/cholesteryl ester hydrolase	LIPA	sp|P38571	3	3	3	0	0	0	3	0	0	0	3	0	0	0	3	13	45.418	0	24.978	197390000	0	0	0	1.97E+08	0	0	0	8117600
Q96QR8	Q96QR8	Transcriptional activator protein Pur-beta	PURB	sp|Q96QR	3	3	3	0	1	3	1	0	1	3	1	0	1	3	1	33.7	33.24	0	11.878	197350000	0	23544000	51814000	1.22E+08	0	3077000	31396000	7438000
Q96KA5	Q96KA5	Cleft lip and palate transmembrane protein 1-like	CLPTM1L	sp|Q96KA5	3	3	3	0	3	0	3	0	3	0	3	0	3	0	3	7.4	62.228	0	7.7248	197320000	0	30792000	0	1.67E+08	0	3852700	0	5710300
Q6UN15	Q6UN15	Pre-mRNA 3-end-processing factor FIP1	FIP1L1	sp|Q6UN1	6	6	6	0	5	4	3	0	5	4	3	0	5	4	3	15.3	66.526	0	21.718	197090000	0	1.16E+08	12770000	67860000	0	8017600	10523000	3097800
Q96PU8	Q96PU8	Protein quaking	QKI	sp|Q96PU	3	3	3	0	3	0	3	0	3	0	3	0	3	0	3	11.1	37.67	0	11.877	196460000	0	76319000	0	1.2E+08	0	6633300	0	5034900
P61225;P1	P61225	Ras-related protein Rap-2b	RAP2B	sp|P61225	3	2	2	0	2	0	3	0	1	0	2	0	1	0	2	29.5	20.504	0	7.3985	196160000	0	35694000	0	1.6E+08	0	3400000	0	6345700
Q14139	Q14139	Ubiquitin conjugation factor E4 A	UBE4A	sp|Q14139	2	2	2	0	2	0	2	0	2	0	2	0	2	0	2	3.6	122.56	0	5.6985	195860000	0	87682000	0	1.08E+08	0	7748400	0	4429600
Q16644	Q16644	MAP kinase-activated protein kinase 3	MAPKAPK3	sp|Q16644	6	6	4	0	5	0	5	0	5	0	5	0	3	0	3	21.7	42.987	0	8.5451	195850000	0	54653000	0	1.41E+08	0	4373300	0	6251000
P48059	P48059	LIM and senescent cell antigen-like-containing do	LIMS1	sp|P48059	1	1	1	0	0	1	1	0	0	1	1	0	0	1	1	3.7	37.251	0.001177	3.0577	194750000	0	0	5385700	1.89E+08	0	0	3619200	7787900
Q8N668	Q8N668	COMM domain-containing protein 1	COMMD1	sp|Q8N668	2	2	2	0	2	0	2	0	2	0	2	0	2	0	2	18.4	21.178	0	30.473	194470000	0	28526000	0	1.66E+08	0	4000900	0	5338300
Q9Y5P4	Q9Y5P4	Collagen type IV alpha-3-binding protein	COL4A3BP	sp|Q9Y5P4	5	5	5	0	2	0	5	0	2	0	5	0	2	0	5	15.5	70.834	0	53.908	194250000	0	25703000	0	1.69E+08	0	4357000	0	4840100
Q03701	Q03701	CCAAT/enhancer-binding protein zeta	CEBPZ	sp|Q03701	5	5	5	0	0	4	5	0	0	4	5	0	0	4	5	6.9	120.97	0	31.594	194210000	0	0	24745000	1.69E+08	0	0	19348000	4249800
Q8TCD5	Q8TCD5	5(3)-deoxyribonucleotidase, cytosolic type	NT5C	sp|Q8TCD5	3	3	3	0	3	0	3	0	3	0	3	0	3	0	3	29.4	23.382	0	21.785	193690000	0	55797000	0	1.38E+08	0	5165400	0	5424100
O43181	O43181	NADH dehydrogenase [ubiquinone] iron-sulfur pr	NDUFS4	sp|O43181	2	2	2	1	2	1	2	1	2	1	2	1	2	1	2	13.7	20.108	0	6.1855	193380000	157510	85507000	3761400	1.04E+08	249650	7707600	1588300	4952100
Q9BW27	Q9BW27	Nuclear pore complex protein Nup85	NUP85	sp|Q9BW2	6	6	6	0	3	1	5	0	3	1	5	0	3	1	5	13.6	75.019	0	18.782	193100000	0	20326000	1091600	1.72E+08	0	1798700	2475800	5311300
Q9Y2V7	Q9Y2V7	Conserved oligomeric Golgi complex subunit 6	COG6	sp|Q9Y2V7	7	7	7	0	3	2	5	0	3	2	5	0	3	2	5	20.1	73.278	0	11.293	192940000	0	39688000	3619900	1.5E+08	0	2692600	3317600	6074500
Q9BRP8	Q9BRP8	Partner of Y14 and mago	WIBG	sp|Q9BRP8	5	5	5	1	5	4	3	1	5	4	3	1	5	4	3	42.2	22.655	0	7.3648	192860000	1789000	93694000	7376200	90004000	2136900	8835800	4405000	3328600
O60343	O60343	TBC1 domain family member 4	TBC1D4	sp|O60343	11	11	11	0	9	1	7	0	9	1	7	0	9	1	7	11.6	146.56	0	22.41	192300000	0	1.33E+08	594410	59156000	0	11875000	613840	2027300
P04637	P04637	Cellular tumor antigen p53	TP53	sp|P04637	5	5	5	2	5	0	2	2	5	0	2	2	5	0	2	19.6	43.653	0	10.697	192030000	3562100	1.13E+08	0	75818000	5249200	6013300	0	5347800
O95602	O95602	DNA-directed RNA polymerase I subunit RPA1	POLR1A	sp|O95602	10	10	10	0	3	3	9	0	3	3	9	0	3	3	9	9.7	194.81	0	30.632	191990000	0	16867000	5599800	1.7E+08	0	1653600	4599000	5969000
P42126	P42126	Enoyl-CoA delta isomerase 1, mitochondrial	ECI1	sp|P42126	3	3	3	0	3	0	1	0	3	0	1	0	3	0	1	11.9	32.816	0	9.6337	191880000	0	1.76E+08	0	16189000	0	15025000	0	1128200

Q13425	Q13425	Beta-2-syntrophin	SNTB2	sp|Q13425	5	5	5	0	4	0	5	0	4	0	5	0	4	0	5	13.1	57.949	0	10.317	191740000	0	32675000	0	1.59E+08	0	2725300	0	6051000
Q9UKN8	Q9UKN8	General transcription factor 3C polypeptide 4	GTF3C4	sp|Q9UKN	9	9	9	0	9	4	6	0	9	4	6	0	9	4	6	18.4	91.981	0	16.626	191740000	0	1.18E+08	5489300	68497000	0	7763800	6126500	2995500
Q9Y2R9	Q9Y2R9	28S ribosomal protein S7, mitochondrial	MRPS7	sp|Q9Y2R9	6	6	6	2	4	5	4	2	4	5	4	2	4	5	4	27.3	28.134	0	15.306	191700000	10813000	88667000	18417000	73805000	11311000	6771600	12149000	3808800
P83111	P83111	Serine beta-lactamase-like protein LACTB, mitoch	LACTB	sp|P83111	6	6	6	0	1	6	4	0	1	6	4	0	1	6	4	15	60.693	0	16.356	191610000	0	2025900	71580000	1.18E+08	0	1872300	47958000	3302800
Q96CW5	Q96CW5	Gamma-tubulin complex component 3	TUBGCP3	sp|Q96CW	7	7	7	0	6	2	7	0	6	2	7	0	6	2	7	11.2	103.57	0	26.38	191260000	0	70904000	4782900	1.16E+08	0	4255100	4804200	5158100
Q8TEM1	Q8TEM1	Nuclear pore membrane glycoprotein 210	NUP210	sp|Q8TEM	5	5	5	0	5	1	1	0	5	1	1	0	5	1	1	3.3	205.11	0	9.1985	191260000	0	1.48E+08	9129400	33715000	0	15023000	3782500	1798200
P46013	P46013	Antigen KI-67	MKI67	sp|P46013	11	11	11	2	9	9	10	2	9	9	10	2	9	9	10	6.2	358.69	0	17.833	191130000	1660300	46012000	17692000	1.26E+08	1932800	3351700	13310000	4182600
P42330	P42330	Aldo-keto reductase family 1 member C3	AKR1C3	sp|P42330	7	3	3	0	0	1	7	0	0	0	3	0	0	0	3	33.1	36.853	0	15.272	190580000	0	0	0	1.91E+08	0	0	0	7837700
O94766	O94766	Galactosylgalactosylxylosylprotein 3-beta-glucuro	B3GAT3	sp|O94766	7	7	7	0	5	5	6	0	5	5	6	0	5	5	6	32.5	37.121	0	21.233	190410000	0	29553000	10969000	1.5E+08	0	2879500	7652500	5608500
Q8IXH7	Q8IXH7	Negative elongation factor C/D	NELFCD	sp|Q8IXH7	5	5	5	0	4	0	5	0	4	0	5	0	4	0	5	11.7	66.246	0	9.4982	189980000	0	65633000	0	1.24E+08	0	6730500	0	4168900
Q9H1I8	Q9H1I8	Activating signal cointegrator 1 complex subunit 2	ASCC2	sp|Q9H1I8	8	8	8	1	5	5	7	1	5	5	7	1	5	5	7	16.9	86.359	0	39.482	189960000	694550	65217000	16067000	1.08E+08	2042700	4591100	9253800	5793500
Q9HD42	Q9HD42	Charged multivesicular body protein 1a	CHMP1A	sp|Q9HD4	2	2	2	1	2	0	2	1	2	0	2	1	2	0	2	9.2	21.703	0	5.6132	189710000	148090	74553000	0	1.15E+08	344430	6261100	0	4844000
Q9NVR0	Q9NVR0	Kelch-like protein 11	KLHL11	sp|Q9NVR	9	9	9	2	2	8	3	2	2	8	3	2	2	8	3	17.9	80.147	0	25.278	189620000	20350000	30366000	87668000	51238000	7694100	3074200	69957000	3321500
Q9BU23	Q9BU23	Lipase maturation factor 2	LMF2	sp|Q9BU2	5	5	5	0	1	2	5	0	1	2	5	0	1	2	5	8.2	79.697	0	17.474	189310000	0	0	4555200	1.85E+08	0	0	6469200	4190000
P12931;Q9	P12931	Proto-oncogene tyrosine-protein kinase Src	SRC	sp|P12931	6	6	3	0	4	1	6	0	4	1	6	0	2	0	3	12.5	59.834	0	16.548	189270000	0	62944000	822550	1.26E+08	0	4827100	832760	5602600
P06280	P06280	Alpha-galactosidase A	GLA	sp|P06280	4	4	4	0	4	1	1	0	4	1	1	0	4	1	1	12.8	48.766	0	13.33	189240000	0	1.83E+08	2059800	3922900	0	17064000	474210	161900
Q96S59	Q96S59	Ran-binding protein 9	RANBP9	sp|Q96S59	5	5	5	0	5	2	2	0	5	2	2	0	5	2	2	11	77.846	0	14.211	189180000	0	96495000	8172400	84509000	0	7031800	5175100	5266400
Q16831	Q16831	Uridine phosphorylase 1	UPP1	sp|Q16831	6	6	6	0	1	2	6	0	1	2	6	0	1	2	6	22.9	33.934	0	10.689	188990000	0	1212100	1368600	1.86E+08	0	152500	606540	7933800
Q9Y263	Q9Y263	Phospholipase A-2-activating protein	PLAA	sp|Q9Y263	9	9	9	0	5	0	9	0	5	0	9	0	5	0	9	15.1	87.156	0	23.751	188960000	0	45619000	0	1.43E+08	0	4476400	0	5439800
Q9GZT4	Q9GZT4	Serine racemase	SRR	sp|Q9GZT4	6	6	6	0	3	0	6	0	3	0	6	0	3	0	6	30	36.566	0	20.487	188660000	0	12759000	0	1.76E+08	0	2488000	0	5870600
P43003	P43003	Excitatory amino acid transporter 1	SLC1A3	sp|P43003	3	3	3	0	0	0	3	0	0	0	3	0	0	0	3	8.9	59.572	0	8.2084	188540000	0	0	0	1.89E+08	0	0	0	7753700
P10909	P10909	Clusterin;Clusterin beta chain;Clusterin alpha chai	CLU	sp|P10909	4	4	4	0	1	3	3	0	1	3	3	0	1	3	3	14	52.494	0	21.277	188040000	0	4937900	15018000	1.68E+08	0	1628000	9151400	6660200
Q9BUI4	Q9BUI4	DNA-directed RNA polymerase III subunit RPC3	POLR3C	sp|Q9BUI4	4	4	4	0	3	0	3	0	3	0	3	0	3	0	3	12.2	60.611	0	12.462	187850000	0	43039000	0	1.45E+08	0	6373100	0	3376100
Q9UNI6	Q9UNI6	Dual specificity protein phosphatase 12	DUSP12	sp|Q9UNI6	3	3	3	0	3	0	3	0	3	0	3	0	3	0	3	13.8	37.687	0	9.3953	187690000	0	17093000	0	1.71E+08	0	2428100	0	6094500
P51003;Q9	P51003	Poly(A) polymerase alpha	PAPOLA	sp|P51003	7	7	7	0	4	0	5	0	4	0	5	0	4	0	5	13.3	82.842	0	43.804	187330000	0	58945000	0	1.28E+08	0	4568600	0	5907300
Q5T447	Q5T447	E3 ubiquitin-protein ligase HECTD3	HECTD3	sp|Q5T447	5	5	5	0	2	1	5	0	2	1	5	0	2	1	5	8.2	97.112	0	17.2	187220000	0	29759000	2232000	1.55E+08	0	3551800	0	5455100
Q13576	Q13576	Ras GTPase-activating-like protein IQGAP2	IQGAP2	sp|Q13576	6	5	5	1	6	0	1	1	5	0	1	1	5	0	1	5.1	180.58	0	20.103	186860000	2976500	62868000	0	1.21E+08	4808100	4437100	0	4249700
Q9Y6M1	Q9Y6M1	Insulin-like growth factor 2 mRNA-binding proteir	IGF2BP2	sp|Q9Y6M	8	7	7	2	5	7	6	1	4	6	5	1	4	6	5	17.5	66.121	0	17.163	186310000	2064800	49308000	22245000	1.13E+08	11275000	3442300	8347800	2930000
P40123	P40123	Adenylyl cyclase-associated protein 2	CAP2	sp|P40123	6	5	5	0	5	1	6	0	4	0	5	0	4	0	5	15.1	52.823	0	11.482	186070000	0	79764000	0	1.06E+08	0	7627000	0	3775900
Q6P1N9	Q6P1N9	Putative deoxyribonuclease TATDN1	TATDN1	sp|Q6P1N9	6	6	6	1	3	0	5	1	3	0	5	1	3	0	5	20.9	33.601	0	13.847	185780000	10539000	32223000	0	1.43E+08	11127000	5340700	0	2793800
P57740	P57740	Nuclear pore complex protein Nup107	NUP107	sp|P57740	5	5	5	0	2	0	5	0	2	0	5	0	2	0	5	8.8	106.37	0	44.266	185730000	0	12260000	0	1.73E+08	0	1677500	0	6537100
P78362;Q9	P78362	SRSF protein kinase 2;SRSF protein kinase 2 N-ter	SRPK2	sp|P78362	5	4	4	1	3	4	5	0	2	3	4	0	2	3	4	9.6	77.526	0	13.427	185520000	0	21788000	7553700	1.56E+08	0	3601000	4743700	5075000
Q86YP4	Q86YP4	Transcriptional repressor p66-alpha	GATAD2A	sp|Q86YP4	8	8	7	1	8	5	6	1	8	5	6	0	7	4	5	18.8	68.062	0	30.251	185400000	3492200	78455000	8484400	94970000	7014000	5543700	4726900	2730500
Q15286	Q15286	Ras-related protein Rab-35	RAB35	sp|Q15286	3	3	3	0	3	1	3	0	3	1	3	0	3	1	3	17.4	23.025	0	15.866	185210000	0	94846000	948850	89419000	0	6260700	3240500	3174300
Q9HAN9	Q9HAN9	Nicotinamide/nicotinic acid mononucleotide ader	NMNAT1	sp|Q9HAN	5	5	5	0	2	5	4	0	2	5	4	0	2	5	4	22.9	31.932	0	11.803	184810000	0	21837000	58170000	1.05E+08	0	2436800	38595000	4293100
Q6UXV4	Q6UXV4	MICOS complex subunit MIC27	APOOL	sp|Q6UXV4	8	8	8	0	4	0	8	0	4	0	8	0	4	0	8	43.3	29.159	0	23.219	183810000	0	29973000	0	1.54E+08	0	3441800	0	5527000
P49773	P49773	Histidine triad nucleotide-binding protein 1	HINT1	sp|P49773	3	3	3	2	3	1	2	2	3	1	2	2	3	1	2	30.2	13.802	0	14.851	183660000	71388000	86399000	1584700	24288000	54930000	15561000	5991200	3933800
Q86U44	Q86U44	N6-adenosine-methyltransferase 70 kDa subunit	METTL3	sp|Q86U44	6	6	6	0	6	1	4	0	6	1	4	0	6	1	4	20.7	64.473	0	17.455	183640000	0	71889000	2025700	1.1E+08	0	4731500	3519300	3960100
O95302;Q7	O95302	Peptidyl-prolyl cis-trans isomerase FKBP9	FKBP9	sp|O95302	6	6	6	0	1	0	6	0	1	0	6	0	1	0	6	16.8	63.083	0	18.423	183600000	0	3148500	0	1.8E+08	0	296410	0	7402400
Q7Z7H5	Q7Z7H5	Transmembrane emp24 domain-containing prote	TMED4	sp|Q7Z7H5	4	3	3	0	4	0	4	0	3	0	3	0	3	0	3	26	25.943	0	12.771	183390000	0	73809000	0	1.1E+08	0	6622100	0	4390600
Q9NRG9	Q9NRG9	Aladin	AAAS	sp|Q9NRG	4	4	4	0	4	2	4	0	4	2	4	0	4	2	4	10.4	59.573	0	14.277	183210000	0	35456000	2198500	1.46E+08	0	2651100	2377700	5560200
Q9NR09	Q9NR09	Baculoviral IAP repeat-containing protein 6	BIRC6	sp|Q9NR09	8	8	8	0	8	0	6	0	8	0	6	0	8	0	6	3.3	530.25	0	14.128	183210000	0	87711000	0	95501000	0	6880600	0	4778600
Q9P265	Q9P265	Disco-interacting protein 2 homolog B	DIP2B	sp|Q9P265	8	8	8	0	2	0	8	0	2	0	8	0	2	0	8	8.3	171.49	0	25.569	182660000	0	6223000	0	1.76E+08	0	548550	0	7256200
Q15054	Q15054	DNA polymerase delta subunit 3	POLD3	sp|Q15054	5	5	5	1	5	2	4	1	5	2	4	1	5	2	4	14.6	51.4	0	18.983	181890000	560870	1.1E+08	1217500	69921000	948720	7818800	2583100	2616900
P08473	P08473	Neprilysin	MME	sp|P08473	4	4	4	1	0	1	4	1	0	1	4	1	0	1	4	6.3	85.513	0	34.772	181880000	791120	0	827410	1.8E+08	795800	0	667160	7297700
Q13895	Q13895	Bystin	BYSL	sp|Q13895	7	7	7	0	6	3	7	0	6	3	7	0	6	3	7	21.5	49.601	0	25.92	181850000	0	54221000	4560400	1.23E+08	0	3615400	2563900	6725900
Q15018	Q15018	BRISC complex subunit Abro1	FAM175B	sp|Q15018	6	6	6	0	6	1	5	0	6	1	5	0	6	1	5	22.4	46.9	0	9.5163	181620000	0	76742000	2190800	1.03E+08	0	5182800	3754800	3522400
Q86XZ4	Q86XZ4	Spermatogenesis-associated serine-rich protein 2	SPATS2	sp|Q86XZ4	6	6	6	2	3	5	6	2	3	5	6	2	3	5	6	17.8	59.544	0	27.805	181590000	975370	7510200	27171000	1.46E+08	2118000	1290200	18728000	3761600
O95372	O95372	Acyl-protein thioesterase 2	LYPLA2	sp|O95372	2	2	2	0	0	0	2	0	0	0	2	0	0	0	2	22.1	24.737	0	39.213	181530000	0	0	0	1.82E+08	0	0	0	7465700
Q9Y4W2	Q9Y4W2	Ribosomal biogenesis protein LAS1L	LAS1L	sp|Q9Y4W	7	7	7	1	7	2	3	1	7	2	3	1	7	2	3	15.4	83.064	0	40.264	181270000	1177800	1.35E+08	2044400	43420000	2337100	9211300	2470100	2186500
Q96JB2	Q96JB2	Conserved oligomeric Golgi complex subunit 3	COG3	sp|Q96JB2	5	5	5	0	4	2	4	0	4	2	4	0	4	2	4	7.9	94.095	0	14.925	180810000	0	59159000	9070900	1.13E+08	0	4140000	7011900	4788500
P13796	P13796	Plastin-2	LCP1	sp|P13796	10	6	6	1	4	3	10	0	0	2	6	0	0	2	6	20.3	70.288	0	21.753	180620000	0	0	1284400	1.79E+08	0	0	2536300	5702100
P67870	P67870	Casein kinase II subunit beta	CSNK2B	sp|P67870	3	3	3	1	3	2	2	1	3	2	2	1	3	2	2	15.3	24.942	0	4.7294	180480000	2927700	1.15E+08	14844000	47902000	4939200	9405300	6950200	3698400
Q9BUN8	Q9BUN8	Derlin-1	DERL1	sp|Q9BUN	2	2	2	0	2	1	2	0	2	1	2	0	2	1	2	7.6	28.8	0	15.879	180430000	0	10517000	3384200	1.67E+08	0	2503800	3921400	3432100
P23786	P23786	Carnitine O-palmitoyltransferase 2, mitochondrial	CPT2	sp|P23786	9	9	9	0	8	0	7	0	8	0	7	0	8	0	7	18.1	73.776	0	13.132	180300000	0	1.02E+08	0	78337000	0	9217000	0	2992500
P29353	P29353	SHC-transforming protein 1	SHC1	sp|P29353	5	5	5	0	1	0	5	0	1	0	5	0	1	0	5	14.1	62.821	0	8.9692	180240000	0	10085000	0	1.7E+08	0	1894300	0	5992500
Q99614;RE	Q99614	Tetratricopeptide repeat protein 1	TTC1	sp|Q99614	6	6	6	0	6	0	6	0	6	0	6	0	6	0	6	27.1	33.526	0	20.216	180190000	0	86850000	0	93342000	0	7809200	0	3685300
Q8IWS0	Q8IWS0	PHD finger protein 6	PHF6	sp|Q8IWS0	4	4	4	1	4	3	4	1	4	3	4	1	4	3	4	13.7	41.29	0	11.218	179950000	1207000	1.11E+08	7331000	60141000	2227200	8556100	5765900	1866200
Q9BT17	Q9BT17	Mitochondrial ribosome-associated GTPase 1	MTG1	sp|Q9BT17	3	3	3	0	3	1	3	0	3	1	3	0	3	1	3	12	37.236	0	14.621	179930000	0	60414000	1029000	1.18E+08	0	3528700	1925800	5435200
P60510	P60510	Serine/threonine-protein phosphatase 4 catalytic	PPP4C	sp|P60510	3	3	3	0	3	0	2	0	3	0	2	0	3	0	2	13	35.08	0	14.149	179580000	0	91574000	0	88007000	0	7230200	0	4461200
P06756	P06756	Integrin alpha-V;Integrin alpha-V heavy chain;Inte	ITGAV	sp|P06756	6	6	6	0	0	0	6	0	0	0	6	0	0	0	6	10.8	116.04	0	44.915	179450000	0	0	0	1.79E+08	0	0	0	7379900
Q7Z3U7	Q7Z3U7	Protein MON2 homolog	MON2	sp|Q7Z3U7	5	5	5	1	2	3	4	1	2	3	4	1	2	3	4	4.5	190.36	0	10.383	178990000	702540	17270000	11745000	1.49E+08	2932000	2570100	5767900	4986800
Q13464	Q13464	Rho-associated protein kinase 1	ROCK1	sp|Q13464	8	6	6	0	7	1	8	0	5	1	6	0	5	1	6	6.9	158.17	0	13.525	178150000	0	46322000	88281	1.32E+08	0	2237900	872650	6449700
P16219	P16219	Short-chain specific acyl-CoA dehydrogenase, mit	ACADS	sp|P16219	4	4	4	0	4	0	3	0	4	0	3	0	4	0	3	16.7	44.297	0	9.1673	177800000	0	68265000	0	1.1E+08	0	8126400	0	2395700
P55769	P55769	NHP2-like protein 1;NHP2-like protein 1, N-termir	NHP2L1	sp|P55769	3	3	3	0	2	1	3	0	2	1	3	0	2	1	3	24.2	14.173	0	16.103	177550000	0	43454000	7930100	1.26E+08	0	3386900	6832000	4129100
Q5T440	Q5T440	Putative transferase CAF17, mitochondrial	IBA57	sp|Q5T440	4	4	4	0	4	0	1	0	4	0	1	0	4	0	1	18.5	38.155	0	18.177	177440000	0	1.76E+08	0	1884200	0	15475000	0	77489
O43633	O43633	Charged multivesicular body protein 2a	CHMP2A	sp|O43633	4	4	4	0	3	3	3	0	3	3	3	0	3	3	3	17.1	25.104	0	7.6725	177310000	0	43125000	16765000	1.17E+08	0	3165900	13199000	3531100
Q7LBC6	Q7LBC6	Lysine-specific demethylase 3B	KDM3B	sp|Q7LBC6	4	4	4	0	4	0	3	0	4	0	3	0	4	0	3	4.1	191.58	0	6.0722	177310000	0	69205000	0	1.08E+08	0	6392500	0	4153800
Q9Y3I1	Q9Y3I1	F-box only protein 7	FBXO7	sp|Q9Y3I1	3	3	3	0	2	0	3	0	2	0	3	0	2	0	3	9	58.502	0	14.607	177250000	0	37189000	0	1.4E+08	0	3800000	0	5238500
P57076	P57076	UPF0769 protein C21orf59	C21orf59	sp|P57076	3	3	3	0	3	0	2	0	3	0	2	0	3	0	2	20.3	33.224	0	45.086	177110000	0	1.18E+08	0	59211000	0	10451000	0	2376700
Q9NWV8	Q9NWV8	BRISC and BRCA1-A complex member 1	BABAM1	sp|Q9NWV	4	4	4	1	2	1	4	1	2	1	4	1	2	1	4	18.5	36.56	0	14.676	176990000	409180	40998000	910470	1.35E+08	918450	2322500	1240700	5691900
P29218	P29218	Inositol monophosphatase 1	IMPA1	sp|P29218	4	4	4	0	2	1	4	0	2	1	4	0	2	1	4	18.8	30.188	0	11.068	176990000	0	68481000	454560	1.08E+08	0	4387300	2728500	3669900
Q9NVA1	Q9NVA1	Ubiquinol-cytochrome-c reductase complex asser	UQCC1	sp|Q9NVA	3	3	3	0	3	1	3	0	3	1	3	0	3	1	3	13	34.6	0	11.884	176970000	0	67273000	850450	1.09E+08	0	6328800	1602900	3046100
Q6IA69	Q6IA69	Glutamine-dependent NAD(+) synthetase	NADSYN1	sp|Q6IA69	7	7	7	0	1	3	7	0	1	3	7	0	1	3	7	14.4	79.284	0	13.464	176670000	0	8386700	3016700	1.65E+08	0	1634200	1865800	6063300
Q9NX20	Q9NX20	39S ribosomal protein L16, mitochondrial	MRPL16	sp|Q9NX20	4	4	4	0	4	1	4	0	4	1	4	0	4	1	4	18.3	28.449	0	9.4057	176670000	0	71072000	6129600	99465000	0	6088900	5161900	3223700
Q92785	Q92785	Zinc finger protein ubi-d4	DPF2	sp|Q92785	5	5	5	0	5	2	3	0	5	2	3	0	5	2	3	16.1	44.155	0	12.985	176610000	0	69874000	3686100	1.03E+08	0	5535500	3622900	3716000
Q8IUF8	Q8IUF8	Bifunctional lysine-specific demethylase and histic	MINA	sp|Q8IUF8	5	5	5	0	2	3	5	0	2	3	5	0	2	3	5	19.8	52.8	0	39.871	175780000	0	14854000	8234100	1.53E+08	0	3548600	4869700	4703900
Q96G46	Q96G46	tRNA-dihydrouridine(47) synthase [NAD(P)(+)]-lik	DUS3L	sp|Q96G46	9	9	9	0	7	1	9	0	7	1	9	0	7	1	9	23.5	72.593	0	15.038	175510000	0	48116000	2630500	1.25E+08	0	2754200	3803600	4582400
Q9P2W9	Q9P2W9	Syntaxin-18	STX18	sp|Q9P2W	4	4	4	0	4	0	2	0	4	0	2	0	4	0	2	18.2	38.673	0	17.252	175020000	0	99737000	0	75285000	0	6159200	0	5728600
Q8WZ82	Q8WZ82	Ovarian cancer-associated gene 2 protein	OVCA2	sp|Q8WZ8	2	2	2	0	0	0	2	0	0	0	2	0	0	0	2	22	24.418	0.006417	1.7313	174640000	0	0	0	1.75E+08	0	0	0	7182000
Q8WU79	Q8WU79	Stromal membrane-associated protein 2	SMAP2	sp|Q8WU7	5	5	4	0	5	0	5	0	5	0	5	0	4	0	4	16.1	46.785	0	19.055	174360000	0	68402000	0	1.06E+08	0	6158700	0	4228300
O15228	O15228	Dihydroxyacetone phosphate acyltransferase	GNPAT	sp|O15228	4	4	4	0	2	1	3	0	2	1	3	0	2	1	3	8.8	77.187	0	17.551	174320000	0	20983000	4736500	1.49E+08	0	2335500	5153700	2938000
Q9BVS4	Q9BVS4	Serine/threonine-protein kinase RIO2	RIOK2	sp|Q9BVS4	3	3	3	1	0	2	3	1	0	2	3	1	0	2	3	10.9	63.282	0	7.6597	173680000	83400000	0	3864200	86415000	81194000	0	7431400	925320
Q9UJX4	Q9UJX4	Anaphase-promoting complex subunit 5	ANAPC5	sp|Q9UJX4	13	13	13	0	4	13	7	0	4	13	7	0	4	13	7	22.6	85.076	0	24.918	173370000	0	9550800	65312000	98510000	0	3691200	37526000	4854200
P82094	P82094	TATA element modulatory factor	TMF1	sp|P82094	4	4	4	0	2	0	4	0	2	0	4	0	2	0	4	5.7	122.84	0	20.959	173310000	0	23481000	0	1.5E+08	0	2738800	0	5492800
P14859;Q9	P14859	POU domain, class 2, transcription factor 1	POU2F1	sp|P14859	4	4	4	0	4	0	2	0	4	0	2	0	4	0	2	9.7	76.471	0	11.828	173180000	0	1.26E+08	0	46699000	0	9496700	0	3572900
O95219	O95219	Sorting nexin-4	SNX4	sp|O95219	6	6	6	0	5	0	5	0	5	0	5	0	5	0	5	17.8	51.908	0	17.62	173050000	0	79165000	0	93885000	0	6673600	0	3677500
Q86XL3	Q86XL3	Ankyrin repeat and LEM domain-containing prote	ANKLE2	sp|Q86XL3	6	6	6	0	2	1	6	0	2	1	6	0	2	1	6	12.2	104.11	0	12.283	173000000	0	17521000	537290	1.55E+08	0	1041900	430660	6805100
Q9Y2R5	Q9Y2R5	28S ribosomal protein S17, mitochondrial	MRPS17	sp|Q9Y2R5	3	3	3	2	3	2	3	2	3	2	3	2	3	2	3	36.2	14.502	0	6.1844	172280000	33408000	65727000	2865900	70279000	36337000	2424000	1873500	3383400

P07311	P07311	Acylphosphatase-1	ACYP1	sp|P07311	3	3	3	0	3	0	3	0	3	0	3	0	3	0	3	38.4	11.261	0	8.6982	171420000	0	88198000	0	83219000	0	7473800	0	3723200
Q96ER3	Q96ER3	Protein SAAL1	SAAL1	sp|Q96ER	5	5	5	0	4	0	5	0	4	0	5	0	4	0	5	13.3	53.557	0	12.609	171160000	0	62994000	0	1.08E+08	0	6040600	0	3960500
Q66PJ3	Q66PJ3	ADP-ribosylation factor-like protein 6-interacting	ARL6IP4	sp|Q66PJ3	3	3	3	0	2	3	2	0	2	3	2	0	2	3	2	8.1	44.915	0	8.2245	171120000	0	41094000	47178000	82847000	0	4025500	30582000	4125100
P49757	P49757	Protein numb homolog	NUMB	sp|P49757	4	4	4	0	1	2	4	0	1	2	4	0	1	2	4	11.7	70.803	0	12.322	170960000	0	3251600	4704500	1.63E+08	0	517540	2532600	7101500
Q8N6H7	Q8N6H7	ADP-ribosylation factor GTPase-activating protein	ARFGAP2	sp|Q8N6H	6	6	6	0	3	0	5	0	3	0	5	0	3	0	5	16.1	56.72	0	18.268	170790000	0	38858000	0	1.32E+08	0	4189800	0	4661200
Q9NRL2	Q9NRL2	Bromodomain adjacent to zinc finger domain pro	BAZ1A	sp|Q9NRL	2	2	2	1	0	1	0	1	0	1	0	1	0	1	0	1.9	178.7	0.00308	2.0978	170330000	1.23E+08	0	47410000	0	1.23E+08	0	0	0
Q5TZA2	Q5TZA2	Rootletin	CROCC	sp|Q5TZA	1	1	1	0	1	1	1	0	1	1	1	0	1	1	1	0.4	228.52	0.003282	2.0531	169810000	0	66194000	2927200	1.01E+08	0	6341100	2285600	3316300
Q9NQW6	Q9NQW6	Actin-binding protein anillin	ANLN	sp|Q9NQV	6	6	6	0	3	1	6	0	3	1	6	0	3	1	6	8.2	124.2	0	26.056	169360000	0	16319000	436740	1.53E+08	0	1526300	409630	6071900
P53582	P53582	Methionine aminopeptidase 1	METAP1	sp|P53582	4	4	4	0	3	1	3	0	3	1	3	0	3	1	3	14.5	43.215	0	9.2743	168640000	0	67405000	273020	1.01E+08	0	5179300	720100	4378000
P36969	P36969	Phospholipid hydroperoxide glutathione peroxida	GPX4	sp|P36969	4	4	4	0	4	1	3	0	4	1	3	0	4	1	3	27.9	22.174	0	9.3439	168500000	0	50722000	1605000	1.16E+08	0	4074500	1495500	4757400
O75880	O75880	Protein SCO1 homolog, mitochondrial	SCO1	sp|O7588	5	5	5	0	3	1	2	0	3	1	2	0	3	1	2	30.9	33.814	0	10	168330000	0	83128000	1115300	84091000	0	6603400	1473600	0
Q9H7B2	Q9H7B2	Ribosome production factor 2 homolog	RPF2	sp|Q9H7B	5	5	5	1	3	3	5	1	3	3	5	1	3	3	5	20.6	35.582	0	12.592	168080000	526990	31084000	13246000	1.23E+08	2300000	2768700	8539600	3627300
P63313	P63313	Thymosin beta-10	TMSB10	sp|P63313	1	1	1	1	1	0	1	1	1	0	1	1	1	0	1	31.8	5.0256	0	3.926	168030000	2279700	17913000	0	1.48E+08	2279700	1579000	0	6079800
Q04721	Q04721	Neurogenic locus notch homolog protein 2;Notch	NOTCH2	sp|Q04721	8	8	8	0	2	1	8	0	2	1	8	0	2	1	8	5.2	265.4	0	22.991	167750000	0	4106000	1604500	1.62E+08	0	1140400	0	5885500
Q9NR46	Q9NR46	Endophilin-B2	SH3GLB2	sp|Q9NR4	5	5	5	0	5	1	4	0	5	1	4	0	5	1	4	15.2	43.973	0	17.184	167710000	0	1.09E+08	1371400	56988000	0	9991900	997700	1915200
Q9BXR0	Q9BXR0	Queuine tRNA-ribosyltransferase	QTRT1	sp|Q9BXR	6	6	6	1	6	0	3	1	6	0	3	1	6	0	3	21.6	44.047	0	49.456	167390000	1759900	86597000	0	79033000	2397100	7402400	0	2844100
O14957	O14957	Cytochrome b-c1 complex subunit 10	UQCR11	sp|O14957	1	1	1	0	1	0	1	0	1	0	1	0	1	0	1	21.4	6.5696	0.001159	2.8092	167360000	0	58482000	0	1.09E+08	0	3524800	0	3116800
Q9NW15	Q9NW15	Anoctamin-10	ANO10	sp|Q9NW1	7	7	7	0	0	1	7	0	0	1	7	0	0	1	7	14.7	76.328	0	14.373	167200000	0	0	2753000	1.64E+08	0	0	2285400	6327500
Q5J8M3	Q5J8M3	ER membrane protein complex subunit 4	EMC4	sp|Q5J8M	3	3	3	0	3	1	3	0	3	1	3	0	3	1	3	22.4	20.086	0	8.747	167140000	0	49053000	2100500	1.16E+08	0	4057200	2233800	4214300
P45973	P45973	Chromobox protein homolog 5	CBX5	sp|P45973	3	3	3	0	2	1	3	0	2	1	3	0	2	1	3	17.8	22.225	0	6.3327	167090000	0	10007000	4907700	1.52E+08	0	0	5867100	3689200
Q9UBB6	Q9UBB6	Neurochondrin	NCDN	sp|Q9UBB	6	6	6	0	5	1	6	0	5	1	6	0	5	1	6	13	78.863	0	22.596	166960000	0	55217000	295500	1.11E+08	0	6174700	849640	2624700
Q9Y3D7	Q9Y3D7	Mitochondrial import inner membrane translocas	PAM16	sp|Q9Y3D	3	3	3	0	2	1	3	0	2	1	3	0	2	1	3	33.6	13.825	0	8.2977	166960000	0	55814000	1752200	1.09E+08	0	3342700	5381600	1872000
P80297;P0	P80297;P0	Metallothionein-1X;Metallothionein-2;Metallothi	MT1X;MT2	sp|P80297	2	2	1	0	0	0	2	0	0	0	2	0	0	0	1	32.8	6.0683	0	5.7197	166950000	0	0	0	1.67E+08	0	0	0	6865900
Q9UIQ6	Q9UIQ6	Leucyl-cystinyl aminopeptidase;Leucyl-cystinyl am	LNPEP	sp|Q9UIQ	6	6	6	0	0	1	6	0	0	1	6	0	0	1	6	6.8	117.35	0	10.09	166940000	0	0	774500	1.66E+08	0	0	0	6833800
Q9UGI8	Q9UGI8	Testin	TES	sp|Q9UGI	7	7	7	1	7	1	0	1	7	1	0	1	7	1	0	18.8	47.996	0	15.588	166800000	1478100	1.65E+08	263080	0	580940	15423000	200920	0
Q99595	Q99595	Mitochondrial import inner membrane translocas	TIMM17A	sp|Q9959	2	2	2	0	1	0	2	0	1	0	2	0	1	0	2	18.1	18.023	0	8.3489	166790000	0	55865000	0	1.11E+08	0	5301200	0	4184900
P49005	P49005	DNA polymerase delta subunit 2	POLD2	sp|P49005	5	5	5	0	4	0	5	0	4	0	5	0	4	0	5	13.9	51.289	0	11.401	166570000	0	71788000	0	94781000	0	6570100	0	3655900
Q13555	Q13555	Calcium/calmodulin-dependent protein kinase ty	CAMK2G	sp|Q1355	7	3	2	1	5	5	7	0	2	2	3	0	1	1	2	19.2	62.606	0	16.497	166000000	0	47830000	1848600	1.16E+08	0	3122800	1307000	5812200
Q9BZI7	Q9BZI7	Regulator of nonsense transcripts 3B	UPF3B	sp|Q9BZI7	4	4	4	1	4	2	3	1	4	2	3	1	4	2	3	13.7	57.761	0.000723	3.3575	165910000	2458800	1.34E+08	9440700	19711000	0	8766100	7440800	2786500
P11233	P11233	Ras-related protein Ral-A	RALA	sp|P11233	6	2	2	1	4	3	6	0	1	1	2	0	1	1	2	32.5	23.567	0.000248	3.7279	165820000	0	8736500	9395900	1.48E+08	0	0	6695100	5692400
Q8NBU5	Q8NBU5	ATPase family AAA domain-containing protein 1	ATAD1	sp|Q8NBU	6	6	6	0	6	0	4	0	6	0	4	0	6	0	4	25.8	40.744	0	13.796	165800000	0	92826000	0	72971000	0	8596000	0	2587400
O75718	O75718	Cartilage-associated protein	CRTAP	sp|O7571	5	5	5	0	5	0	4	0	5	0	4	0	5	0	4	11.5	46.561	0	10.501	165730000	0	52105000	0	1.14E+08	0	4018900	0	5247200
P31749	P31749	RAC-alpha serine/threonine-protein kinase	AKT1	sp|P31749	3	2	2	0	1	0	3	0	1	0	2	0	1	0	2	6.9	55.686	0	3.8897	165610000	0	2155400	0	1.63E+08	0	1472400	0	5439800
O43854	O43854	EGF-like repeat and discoidin I-like domain-contai	EDIL3	sp|O4385	7	7	7	0	1	7	5	0	1	7	5	0	1	7	5	19	53.764	0	24.665	165570000	0	19877000	25222000	1.2E+08	0	3955100	13963000	5737600
Q9H0B6	Q9H0B6	Kinesin light chain 2	KLC2	sp|Q9H0B	14	9	9	1	11	3	12	0	7	0	8	0	7	0	8	32	68.934	0	22.009	165430000	0	32408000	0	1.33E+08	0	3330800	0	4996600
Q6ZSR9	Q6ZSR9	Uncharacterized protein FLJ45252		sp|Q6ZSR	5	5	5	1	4	1	5	1	4	1	5	1	4	1	5	21.1	37.976	0	19.402	165230000	5939100	21669000	872400	1.37E+08	3474700	2176100	1091700	7317000
Q16540	Q16540	39S ribosomal protein L23, mitochondrial	MRPL23	sp|Q1654	3	3	3	0	3	3	3	0	3	3	3	0	3	3	3	19.6	17.781	0	8.9959	165100000	0	59377000	8920900	96801000	0	5938800	5666000	3605000
P26572	P26572	Alpha-1,3-mannosyl-glycoprotein 2-beta-N-acetyl	MGAT1	sp|P26572	3	3	3	0	1	1	3	0	1	1	3	0	1	1	3	10.6	50.878	0	12.312	165080000	0	3752500	1940600	1.59E+08	0	2309200	0	4576500
Q8N543	Q8N543	Prolyl 3-hydroxylase OGFOD1	OGFOD1	sp|Q8N54	4	4	4	0	2	0	4	0	2	0	4	0	2	0	4	9.8	63.245	0	21.412	164680000	0	15022000	0	1.5E+08	0	3253100	0	4225700
Q9UBI6	Q9UBI6	Guanine nucleotide-binding protein G(I)/G(S)/G((	GNG12	sp|Q9UBI	3	3	3	0	1	2	3	0	1	2	3	0	1	2	3	34.7	8.0061	0	6.8	164320000	0	4999200	5490300	1.54E+08	0	0	3903100	6112800
P35914	P35914	Hydroxymethylglutaryl-CoA lyase, mitochondrial	HMGCL	sp|P35914	4	4	4	0	2	1	4	0	2	1	4	0	2	1	4	17.5	34.36	0	26.64	164320000	0	41076000	2418800	1.21E+08	0	2470700	4198600	3546000
Q96I15	Q96I15	Selenocysteine lyase	SCLY	sp|Q96I15	4	4	4	0	3	0	4	0	3	0	4	0	3	0	4	14.8	48.148	0	8.2284	164150000	0	91435000	0	72717000	0	7642500	0	3407900
O95140	O95140	Mitofusin-2	MFN2	sp|O9514	9	9	8	0	8	0	8	0	8	0	8	0	7	0	7	15.6	86.401	0	23.175	164100000	0	44520000	0	1.2E+08	0	3462400	0	5379700
O15400	O15400	Syntaxin-7	STX7	sp|O1540	5	5	5	1	3	0	5	1	3	0	5	1	3	0	5	26.4	29.815	0	19.922	164070000	1907800	22435000	0	1.4E+08	3222200	2491100	0	3918500
Q969F1	Q969F1	General transcription factor 3C polypeptide 6	GTF3C6	sp|Q969F1	1	1	1	0	1	1	1	0	1	1	1	0	1	1	1	7	24.048	0.002873	2.1511	163910000	0	45709000	1849000	1.16E+08	0	4619600	1835400	3601800
Q9Y6I9	Q9Y6I9	Testis-expressed sequence 264 protein	TEX264	sp|Q9Y6I9	3	3	3	0	3	0	3	0	3	0	3	0	3	0	3	10.2	34.188	0	6.6662	163780000	0	31825000	0	1.32E+08	0	2864900	0	5367200
P31151;Q8	P31151	Protein S100-A7	S100A7	sp|P31151	4	4	4	0	1	4	0	0	1	4	0	0	1	4	0	42.6	11.471	0	7.0661	163760000	0	9244200	154510000	0	0	814870	103830000	0
O75792	O75792	Ribonuclease H2 subunit A	RNASEH2A	sp|O7579	5	5	5	0	5	1	4	0	5	1	4	0	5	1	4	22.1	33.395	0	8.907	163540000	0	85933000	3022500	74587000	0	7684800	0	2957500
Q9Y3A3	Q9Y3A3	MOB-like protein phocein	MOB4	sp|Q9Y3A	2	2	2	0	2	0	2	0	2	0	2	0	2	0	2	19.6	26.032	0	20.348	163530000	0	63200000	0	1E+08	0	5675400	0	4021900
Q9Y3B3	Q9Y3B3	Transmembrane emp24 domain-containing prote	TMED7	sp|Q9Y3B	1	1	1	1	1	1	1	1	1	1	1	1	1	1	1	4.5	25.171	0	12.329	163510000	227650	23454000	520970	1.39E+08	282420	2059600	376270	5656000
Q6S8J3;A5	Q6S8J3;A5	POTE ankyrin domain family member E;POTE ank	POTEE;PO	sp|Q6S8J3	11	1	0	7	10	10	11	0	1	0	1	0	0	0	0	9.5	121.36	0.004544	1.8818	163310000	0	50856000	0	1.12E+08	0	4482900	0	4624600
Q5T3I0	Q5T3I0	G patch domain-containing protein 4	GPATCH4	sp|Q5T3I0	6	6	6	2	6	4	4	2	6	4	4	2	6	4	4	20.4	50.381	0	23.134	163190000	4002800	71879000	15670000	71639000	7647800	3274400	10759000	2134500
P50748	P50748	Kinetochore-associated protein 1	KNTC1	sp|P50748	5	5	5	1	4	2	4	1	4	2	4	1	4	2	4	3	250.75	0	13.937	163140000	901380	39054000	1649800	1.22E+08	0	3189000	2005000	4355400
Q9Y3T9	Q9Y3T9	Nucleolar complex protein 2 homolog	NOC2L	sp|Q9Y3T	5	5	5	0	3	5	5	0	3	5	5	0	3	5	5	7.3	84.918	0	7.2162	163110000	0	26469000	14180000	1.22E+08	0	3616100	8982900	4299300
P52294	P52294	Importin subunit alpha-5;Importin subunit alpha-	KPNA1	sp|P52294	6	2	2	1	4	1	6	0	2	0	2	0	2	0	2	11.7	60.221	0	7.8777	163090000	0	49305000	0	1.14E+08	0	4241600	0	4784100
P43155	P43155	Carnitine O-acetyltransferase	CRAT	sp|P43155	7	7	7	0	7	1	7	0	7	1	7	0	7	1	7	12.8	70.857	0	17.152	162650000	0	46720000	1627300	1.14E+08	0	2958800	2157800	4796100
Q12789	Q12789	General transcription factor 3C polypeptide 1	GTF3C1	sp|Q1278	3	3	3	0	3	1	3	0	3	1	3	0	3	1	3	2.5	238.87	0	8.0318	162440000	0	90780000	505610	71150000	0	6623200	2431000	2213900
Q9BY43	Q9BY43	Charged multivesicular body protein 4a	CHMP4A	sp|Q9BY4	5	5	5	0	5	1	4	0	5	1	4	0	5	1	4	26.1	25.098	0	20.832	162390000	0	56393000	12300000	93698000	0	4442100	6986900	5660900
Q3SXM5	Q3SXM5	Inactive hydroxysteroid dehydrogenase-like prote	HSDL1	sp|Q3SXM	5	5	5	0	5	0	4	0	5	0	4	0	5	0	4	17.3	37.001	0	16.461	162330000	0	72239000	0	90091000	0	5382300	0	4690600
Q8NBQ5	Q8NBQ5	Estradiol 17-beta-dehydrogenase 11	HSD17B11	sp|Q8NBQ	5	5	5	0	4	1	5	0	4	1	5	0	4	1	5	19.7	32.935	0	13.764	161870000	0	31982000	459170	1.29E+08	0	3729300	0	4412800
Q15041	Q15041	ADP-ribosylation factor-like protein 6-interacting	ARL6IP1	sp|Q15041	2	2	2	0	2	0	2	0	2	0	2	0	2	0	2	8.9	23.362	0	4.7077	161710000	0	45249000	0	1.16E+08	0	3485900	0	5292300
Q86V21	Q86V21	Acetoacetyl-CoA synthetase	AACS	sp|Q86V2	5	5	5	0	3	1	5	0	3	1	5	0	3	1	5	8.8	75.143	0	17.043	161670000	0	26838000	521240	1.34E+08	0	2598900	610510	5030100
O60499	O60499	Syntaxin-10	STX10	sp|O6049	4	4	4	0	2	1	4	0	2	1	4	0	2	1	4	28.5	28.114	0	37.475	161540000	0	4504000	1547500	1.55E+08	0	854950	786390	6190200
Q93034	Q93034	Cullin-5	CUL5	sp|Q9303	8	8	8	0	8	0	4	0	8	0	4	0	8	0	4	11.5	90.954	0	17.116	161240000	0	1.06E+08	0	55497000	0	7730100	0	3873300
Q16774	Q16774	Guanylate kinase	GUK1	sp|Q1677	2	2	2	0	2	0	1	0	2	0	1	0	2	0	1	22.8	21.725	0	19.793	160980000	0	55524000	0	1.05E+08	0	4318300	0	4913100
Q9BV38	Q9BV38	WD repeat-containing protein 18	WDR18	sp|Q9BV3	6	6	6	3	6	2	4	3	6	2	4	3	6	2	4	16.2	47.405	0	15.992	160910000	4450100	1.19E+08	1966900	35686000	7629600	7100000	1796500	1185600
Q8IXK0;Q8	Q8IXK0	Polyhomeotic-like protein 2	PHC2	sp|Q8IXK0	5	5	5	0	4	3	5	0	4	3	5	0	4	3	5	5.9	90.712	0	6.5906	160860000	0	24589000	11393000	1.25E+08	0	4022200	7695900	3241300
Q96EY1	Q96EY1	DnaJ homolog subfamily A member 3, mitochond	DNAJA3	sp|Q96EY1	3	3	3	1	3	2	3	1	3	2	3	1	3	2	3	8.3	52.488	0	10.237	160860000	485510	76563000	4983300	78826000	1181600	6360800	4580600	1702100
Q6P1L8	Q6P1L8	39S ribosomal protein L14, mitochondrial	MRPL14	sp|Q6P1L	4	4	4	1	3	3	4	1	3	3	4	1	3	3	4	35.9	15.947	0	11.878	160760000	5151200	53296000	5397300	96920000	7061700	2152000	5199700	3048700
Q96IU4	Q96IU4	Alpha/beta hydrolase domain-containing protein	ABHD14B	sp|Q96IU4	2	2	2	1	2	1	1	1	2	1	1	1	2	1	1	17.1	22.345	0	21.801	160690000	32177000	1.1E+08	2176400	16496000	19743000	13230000	4209100	6818800
O60306	O60306	Intron-binding protein aquarius	AQR	sp|O6030	6	5	5	0	5	0	4	0	4	0	3	0	4	0	3	7	171.29	0	23.43	160570000	0	46633000	0	1.14E+08	0	4507400	0	4288900
Q9UJY5	Q9UJY5	ADP-ribosylation factor-binding protein GGA1	GGA1	sp|Q9UJY5	4	4	4	0	4	0	2	0	4	0	2	0	4	0	2	11.7	70.383	0	31.094	160570000	0	62620000	0	97952000	0	5270400	0	4277800
Q96FQ6	Q96FQ6	Protein S100-A16	S100A16	sp|Q96FQ	4	4	4	1	0	2	4	1	0	2	4	1	0	2	4	52.4	11.801	0	18.472	160490000	688930	0	5206500	1.55E+08	980070	0	2446000	7119500
Q6P6C2	Q6P6C2	RNA demethylase ALKBH5	ALKBH5	sp|Q6P6C	3	3	3	1	3	1	2	1	3	1	2	1	3	1	2	15.5	44.255	0	26.992	160360000	141950	84923000	5920400	69371000	1024100	7133700	3658500	2642900
P17931	P17931	Galectin-3	LGALS3	sp|P17931	5	5	5	0	1	1	5	0	1	1	5	0	1	1	5	22.8	26.152	0	9.7074	160130000	0	3790300	1480400	1.55E+08	0	315720	544800	6837300
Q9NV56	Q9NV56	MRG/MORF4L-binding protein	MRGBP	sp|Q9NV5	1	1	1	0	1	1	1	0	1	1	1	0	1	1	1	6.4	22.417	0.000715	3.2622	159840000	0	87414000	6718900	65703000	0	7705400	4515100	2702100
Q9Y3D3	Q9Y3D3	28S ribosomal protein S16, mitochondrial	MRPS16	sp|Q9Y3D	2	2	2	0	2	1	2	0	2	1	2	0	2	1	2	19	15.345	0	5.7485	159440000	0	95430000	1999500	62007000	0	7417500	2739900	2148400
Q9GZL7	Q9GZL7	Ribosome biogenesis protein WDR12	WDR12	sp|Q9GZL	5	5	5	0	4	1	4	0	4	1	4	0	4	1	4	19.9	47.707	0	19.244	158700000	0	55521000	2674900	1.01E+08	0	3998700	3886500	2939900
P33908	P33908	Mannosyl-oligosaccharide 1,2-alpha-mannosidase	MAN1A1	sp|P33908	2	2	2	0	1	0	2	0	1	0	2	0	1	0	2	6.4	72.968	0	5.8543	158690000	0	18645000	0	1.4E+08	0	1928600	0	5474400
Q9UK45	Q9UK45	U6 snRNA-associated Sm-like protein LSm7	LSM7	sp|Q9UK4	1	1	1	0	1	0	1	0	1	0	1	0	1	0	1	24.3	11.602	0	17.952	158490000	0	68154000	0	90335000	0	5603500	0	4119300
Q9BUB7	Q9BUB7	Transmembrane protein 70, mitochondrial	TMEM70	sp|Q9BUB	2	2	2	0	2	0	2	0	2	0	2	0	2	0	2	12.7	28.969	0	9.4893	158460000	0	28573000	0	1.3E+08	0	2522500	0	5338000
P62256	P62256	Ubiquitin-conjugating enzyme E2 H	UBE2H	sp|P62256	4	4	4	0	1	0	4	0	1	0	4	0	1	0	4	26.8	20.655	0	12.323	158430000	0	2294100	0	1.56E+08	0	423680	0	6199700
P10253	P10253	Lysosomal alpha-glucosidase;76 kDa lysosomal al	GAA	sp|P10253	9	9	9	0	8	0	1	0	8	0	1	0	8	0	1	15.4	105.32	0	33.075	158430000	0	1.28E+08	0	30803000	0	11250000	0	0
O95477	O95477	ATP-binding cassette sub-family A member 1	ABCA1	sp|O9547	9	9	9	0	0	2	9	0	0	2	9	0	0	2	9	5.1	254.3	0	14.337	158420000	0	0	1051700	1.57E+08	0	0	2980700	4198100
P56182	P56182	Ribosomal RNA processing protein 1 homolog A	RRP1	sp|P56182	3	3	3	0	2	3	3	0	2	3	3	0	2	3	3	12.4	52.839	0	11.015	158410000	0	50314000	17205000	90889000	0	5213000	11675000	2846900
A0A0G2JR	A0A0G2JRQ6			tr|A0A0G2	2	2	2	0	1	2	0	0	1	2	0	0	1	2	0	13.7	12.748	0.001148	2.6427	158410000	0	1741600	156670000	0	0	234940	105200000	0
Q99584	Q99584	Protein S100-A13	S100A13	sp|Q9958	3	3	3	1	2	2	3	1	2	2	3	1	2	2	3	33.7	11.471	0	8.8002	158360000	592310	33898000	1256700	1.23E+08	1109000	4359600	957700	3041200
Q96S52	Q96S52	GPI transamidase component PIG-S	PIGS	sp|Q96S5	6	6	6	0	3	0	5	0	3	0	5	0	3	0	5	14.4	61.655	0	14.048	158320000	0	18378000	0	1.4E+08	0	2581700	0	4793400
P98170	P98170	E3 ubiquitin-protein ligase XIAP	XIAP	sp|P98170	2	2	2	0	1	0	2	0	1	0	2	0	1	0	2	4.8	56.684	0	6.8041	157970000	0	2248700	0	1.56E+08	0	0	0	6403900

Q5VST9	Q5VST9	Obscurin	OBSCN	sp|Q5VST9	2	2	2	0	2	1	2	0	2	1	2	0	2	1	2	0.3	868.47	0.001128	2.4182	157910000	0	69049000	6162700	82702000	0	5185900	5664200	2779000
Q9UNP9	Q9UNP9	Peptidyl-prolyl cis-trans isomerase E	PPIE	sp|Q9UNP	6	5	5	0	6	0	2	0	5	0	2	0	5	0	2	31.6	33.43	0	15.247	157890000	0	1.34E+08	0	23897000	0	10275000	0	2519200
Q9UP83	Q9UP83	Conserved oligomeric Golgi complex subunit 5	COG5	sp|Q9UP83	6	6	6	0	4	2	6	0	4	2	6	0	4	2	6	8.7	92.742	0	20.801	157510000	0	19523000	1323100	1.37E+08	0	2681200	0	4660200
Q08188	Q08188	Protein-glutamine gamma-glutamyltransferase E;	TGM3	sp|Q08188	9	9	9	0	1	9	0	0	1	9	0	0	1	9	0	16	76.631	0	33.032	157500000	0	17700000	139800000	0	0	1815400	93688000	0
Q92541	Q92541	RNA polymerase-associated protein RTF1 homolo	RTF1	sp|Q92541	7	7	7	0	7	2	5	0	7	2	5	0	7	2	5	10.6	80.313	0	19.751	157290000	0	97007000	5829600	54454000	0	6212400	6003400	2492300
O43913	O43913	Origin recognition complex subunit 5	ORC5	sp|O43913	4	4	4	1	3	0	2	1	3	0	2	1	3	0	2	10.8	50.282	0	10.545	157220000	35085000	61601000	0	60534000	0	5430000	0	0
Q9NNW5	Q9NNW5	WD repeat-containing protein 6	WDR6	sp|Q9NNW	4	4	4	0	4	3	4	0	4	3	4	0	4	3	4	8.4	121.72	0	11.38	157180000	0	40540000	7358600	1.09E+08	0	2993900	6581200	3437700
Q9NQT8	Q9NQT8	Kinesin-like protein KIF13B	KIF13B	sp|Q9NQT	9	9	8	0	1	1	9	0	1	1	9	0	0	0	8	7.4	202.79	0	23.123	157010000	0	7844600	1837800	1.47E+08	0	890760	1590900	5503600
P09884	P09884	DNA polymerase alpha catalytic subunit	POLA1	sp|P09884	4	4	4	0	4	0	4	0	4	0	4	0	4	0	4	4	165.91	0	13.672	156870000	0	85977000	0	70894000	0	7541200	0	2953100
P51948	P51948	CDK-activating kinase assembly factor MAT1	MNAT1	sp|P51948	3	3	3	0	2	0	3	0	2	0	3	0	2	0	3	15.9	35.823	0	19.49	156660000	0	13713000	0	1.43E+08	0	3208500	0	3879100
Q9Y2D4	Q9Y2D4	Exocyst complex component 6B	EXOC6B	sp|Q9Y2D4	5	5	5	0	2	0	5	0	2	0	5	0	2	0	5	8.5	94.2	0	19.73	156520000	0	13201000	0	1.43E+08	0	1163700	0	5894000
Q9NWU5	Q9NWU5	39S ribosomal protein L22, mitochondrial	MRPL22	sp|Q9NWU	3	3	3	1	1	1	3	1	1	1	3	1	1	1	3	19.9	23.64	0	9.4272	155990000	3272000	32850000	3009600	1.17E+08	4297700	3383700	2363300	2951400
Q9HBH5	Q9HBH5	Retinol dehydrogenase 14	RDH14	sp|Q9HBH	5	5	5	0	4	0	5	0	4	0	5	0	4	0	5	22.6	36.864	0	16.032	155960000	0	41595000	0	1.14E+08	0	4608300	0	3761800
Q99570	Q99570	Phosphoinositide 3-kinase regulatory subunit 4	PIK3R4	sp|Q99570	4	4	4	0	4	1	2	0	4	1	2	0	4	1	2	3.8	153.1	0	12.903	155800000	0	31292000	3740400	1.21E+08	0	2693400	2589300	4955900
O43149	O43149	Zinc finger ZZ-type and EF-hand domain-containir	ZZEF1	sp|O43149	4	4	4	0	3	0	4	0	3	0	4	0	3	0	4	2	331.07	0	4.7922	155720000	0	62368000	0	93352000	0	4877500	0	4459400
Q14149	Q14149	MORC family CW-type zinc finger protein 3	MORC3	sp|Q14149	2	2	2	0	1	0	1	0	1	0	1	0	1	0	1	3.7	107.11	0	7.6237	155530000	0	95740000	0	59785000	0	0	0	2458700
O43819	O43819	Protein SCO2 homolog, mitochondrial	SCO2	sp|O43819	6	6	6	1	6	3	4	1	6	3	4	1	6	3	4	26.3	29.81	0	11.434	155470000	253730	83098000	2800800	69315000	1303700	5201100	2006500	3800200
Q16134	Q16134	Electron transfer flavoprotein-ubiquinone oxidore	ETFDH	sp|Q16134	3	3	3	0	2	0	2	0	2	0	2	0	2	0	2	6.3	68.495	0	9.9616	155170000	0	9865400	0	1.45E+08	0	0	0	5975600
Q9Y276	Q9Y276	Mitochondrial chaperone BCS1	BCS1L	sp|Q9Y276	3	3	3	0	3	0	3	0	3	0	3	0	3	0	3	12.6	47.534	0	12.417	154870000	0	45876000	0	1.09E+08	0	0	0	4482500
Q6YP21	Q6YP21	Kynurenine--oxoglutarate transaminase 3	CCBL2	sp|Q6YP21	6	6	6	0	6	0	2	0	6	0	2	0	6	0	2	20	51.4	0	13.419	154870000	0	1.32E+08	0	22575000	0	11662000	0	0
O94804	O94804	Serine/threonine-protein kinase 10	STK10	sp|O94804	10	9	9	1	3	1	10	0	2	1	9	0	2	1	9	13.5	112.13	0	25.972	154860000	0	6601600	379430	1.48E+08	0	490800	243700	6184000
Q9Y2I1	Q9Y2I1	Nischarin	NISCH	sp|Q9Y2I1	2	2	2	1	2	0	2	1	2	0	2	1	2	0	2	1.7	166.63	0.000249	3.754	154690000	93853000	50851000	0	9986900	81064000	11195000	0	6487900
Q8IZ81	Q8IZ81	ELMO domain-containing protein 2	ELMOD2	sp|Q8IZ81	4	4	4	0	3	0	4	0	3	0	4	0	3	0	4	14	34.96	0	6.9607	154590000	0	35194000	0	1.19E+08	0	3154500	0	4858000
Q9H974	Q9H974	Queuine tRNA-ribosyltransferase subunit QTRTD1	QTRTD1	sp|Q9H974	3	3	3	0	3	1	3	0	3	1	3	0	3	1	3	12.5	46.712	0	17.46	154480000	0	59297000	7944600	87244000	0	3995600	7497000	2661000
Q9Y241	Q9Y241	HIG1 domain family member 1A, mitochondrial	HIGD1A	sp|Q9Y241	1	1	1	0	1	1	1	0	1	1	1	0	1	1	1	19.4	10.143	0	3.8884	154430000	0	57637000	9353100	87444000	0	5080600	6285200	3596200
Q9BZV1	Q9BZV1	UBX domain-containing protein 6	UBXN6	sp|Q9BZV1	4	4	4	0	2	0	4	0	2	0	4	0	2	0	4	18.4	49.753	0	21.759	154140000	0	13510000	0	1.41E+08	0	1808600	0	5165700
P09234	P09234	U1 small nuclear ribonucleoprotein C	SNRPC	sp|P09234	2	2	2	1	2	1	1	1	2	1	1	1	2	1	1	13.2	17.394	0	8.5655	154140000	3034900	1.05E+08	5552600	40325000	2874200	9514000	3187000	2124700
P34896	P34896	Serine hydroxymethyltransferase, cytosolic	SHMT1	sp|P34896	6	5	5	0	4	1	6	0	3	0	5	0	3	0	5	17.6	53.082	0	13.502	153900000	0	63826000	0	90074000	0	5783400	0	3547200
Q6SZW1	Q6SZW1	Sterile alpha and TIR motif-containing protein 1	SARM1	sp|Q6SZW	6	6	6	1	5	1	4	1	5	1	4	1	5	1	4	12.7	79.387	0	20.935	153580000	410720	1.12E+08	1236200	39578000	2928700	4927300	2458500	2458900
Q15773	Q15773	Myeloid leukemia factor 2	MLF2	sp|Q15773	2	2	2	0	1	2	2	0	1	2	2	0	1	2	2	18.1	28.147	0	4.611	153350000	0	21979000	12031000	1.19E+08	0	3412200	7714500	3803400
Q99797	Q99797	Mitochondrial intermediate peptidase	MIPEP	sp|Q99797	5	5	5	0	5	0	5	0	5	0	5	0	5	0	5	8.4	80.64	0	8.2762	153320000	0	65335000	0	87990000	0	6009300	0	3368600
Q99567	Q99567	Nuclear pore complex protein Nup88	NUP88	sp|Q99567	4	4	4	0	2	1	4	0	2	1	4	0	2	1	4	9.7	83.541	0	28.577	153100000	0	21371000	1618700	1.3E+08	0	2002500	1392400	4927700
O60524	O60524	Nuclear export mediator factor NEMF	NEMF	sp|O60524	8	8	8	1	4	2	8	1	4	2	8	1	4	2	8	8.3	122.95	0	13.083	153030000	789150	19959000	6436000	1.26E+08	0	2840400	5267600	3151700
Q9NPF4	Q9NPF4	Probable tRNA N6-adenosine threonylcarbamoylt	OSGEP	sp|Q9NPF4	5	5	5	0	5	1	3	0	5	1	3	0	5	1	3	24.8	36.426	0	16.696	153000000	0	69949000	1505500	81544000	0	5147500	0	4372000
Q9Y4E6	Q9Y4E6	WD repeat-containing protein 7	WDR7	sp|Q9Y4E6	3	3	3	0	1	0	3	0	1	0	3	0	1	0	3	3.3	163.81	0.001157	2.7672	152960000	0	18111000	0	1.35E+08	0	1640100	0	5502200
Q9NRG0	Q9NRG0	Chromatin accessibility complex protein 1	CHRAC1	sp|Q9NRG	4	4	4	0	4	0	4	0	4	0	4	0	4	0	4	45	14.71	0	9.3095	152410000	0	84331000	0	68080000	0	7266900	0	2966600
Q15370	Q15370	Transcription elongation factor B polypeptide 2	TCEB2	sp|Q15370	4	4	4	1	3	2	4	1	3	2	4	1	3	2	4	39	13.133	0	11.488	152270000	3120400	38931000	2979600	1.07E+08	4224600	2825500	2585500	3329000
Q9NSC5	Q9NSC5	Homer protein homolog 3	HOMER3	sp|Q9NSC5	5	5	5	0	0	2	5	0	0	2	5	0	0	2	5	19.9	39.836	0	22.501	152230000	0	0	6649700	1.46E+08	0	0	5411000	5044500
Q00013	Q00013	55 kDa erythrocyte membrane protein	MPP1	sp|Q00013	4	4	4	0	2	0	4	0	2	0	4	0	2	0	4	15.5	52.296	0	23.2	152220000	0	35368000	0	1.17E+08	0	4560300	0	3363000
Q8WUH6	Q8WUH6	Transmembrane protein 263	TMEM263	sp|Q8WUH	3	3	3	0	3	0	3	0	3	0	3	0	3	0	3	40.5	11.748	0	5.9309	152190000	0	86892000	0	65299000	0	7838100	0	2506800
P42226	P42226	Signal transducer and activator of transcription 6	STAT6	sp|P42226	5	5	5	0	0	0	5	0	0	0	5	0	0	0	5	8.7	94.134	0	25.594	151950000	0	0	0	1.52E+08	0	0	0	6249100
Q96F07	Q96F07	Cytoplasmic FMR1-interacting protein 2	CYFIP2	sp|Q96F07	15	4	4	1	11	2	15	1	3	0	4	1	3	0	4	14.6	148.4	0	10.891	151720000	218430	33071000	0	1.18E+08	1510600	3984800	0	2508900
P48426	P48426	Phosphatidylinositol 5-phosphate 4-kinase type-2	PIP4K2A	sp|P48426	5	3	3	0	3	0	5	0	3	0	3	0	3	0	3	15.8	46.224	0	9.5716	151700000	0	29485000	0	1.22E+08	0	3291200	0	4334100
Q99627	Q99627	COP9 signalosome complex subunit 8	COPS8	sp|Q99627	2	2	2	0	2	0	2	0	2	0	2	0	2	0	2	17.2	23.225	0	6.316	151540000	0	52866000	0	98674000	0	4373500	0	4344700
P42566	P42566	Epidermal growth factor receptor substrate 15	EPS15	sp|P42566	7	7	7	0	6	0	5	0	6	0	5	0	6	0	5	11.8	98.655	0	28.62	151440000	0	43196000	0	1.08E+08	0	4230300	0	4028900
Q3SY69	Q3SY69	Mitochondrial 10-formyltetrahydrofolate dehydro	ALDH1L2	sp|Q3SY69	8	8	8	0	8	0	2	0	8	0	2	0	8	0	2	13.5	101.74	0	30.271	151350000	0	1.38E+08	0	13162000	0	11972000	0	750840
Q8N5M4	Q8N5M4	Tetratricopeptide repeat protein 9C	TTC9C	sp|Q8N5M	2	2	2	0	2	0	2	0	2	0	2	0	2	0	2	17	20.012	0	9.1132	151300000	0	52836000	0	98463000	0	4662800	0	4044000
P62834	P62834	Ras-related protein Rap-1A	RAP1A	sp|P62834	7	1	1	1	7	2	7	0	1	0	1	0	1	0	1	42.9	20.987	0.002226	2.2365	151240000	0	41787000	0	1.09E+08	0	3683500	0	4501500
P54652	P54652	Heat shock-related 70 kDa protein 2	HSPA2	sp|P54652	17	4	4	4	14	16	17	1	1	3	4	1	1	3	4	28.6	70.02	0	14.329	151020000	16145000	3414300	6087700	1.25E+08	15975000	1554000	2726500	5437900
Q96FX7	Q96FX7	tRNA (adenine(58)-N(1))-methyltransferase cataly	TRMT61A	sp|Q96FX7	3	3	3	1	2	1	3	1	2	1	3	1	2	1	3	14.2	31.381	0	12.486	151010000	359090	63733000	1432700	85489000	1645600	4164200	1688500	2957400
Q9Y6A5	Q9Y6A5	Transforming acidic coiled-coil-containing protein	TACC3	sp|Q9Y6A5	4	4	4	0	4	0	2	0	4	0	2	0	4	0	2	9.1	90.359	0	8.5102	150870000	0	1.32E+08	0	19345000	0	7301700	0	5088000
Q14690	Q14690	Protein RRP5 homolog	PDCD11	sp|Q14690	7	7	7	0	0	6	5	0	0	6	5	0	0	6	5	6.6	208.7	0	13.066	150570000	0	0	21035000	1.3E+08	0	0	15665000	3797400
Q9NRG1	Q9NRG1	Phosphoribosyltransferase domain-containing pro	PRTFDC1	sp|Q9NRG	2	2	2	0	2	0	2	0	2	0	2	0	2	0	2	15.6	25.673	0	6.608	150180000	0	56568000	0	93608000	0	5058900	0	3777200
O95453	O95453	Poly(A)-specific ribonuclease PARN	PARN	sp|O95453	5	5	5	1	3	1	5	1	3	1	5	1	3	1	5	9.1	73.45	0	6.9822	149900000	3723900	25988000	3061000	1.17E+08	3732900	3039900	5574700	541070
Q92896	Q92896	Golgi apparatus protein 1	GLG1	sp|Q92896	4	4	4	0	2	1	4	0	2	1	4	0	2	1	4	3.9	134.55	0	13.159	149830000	0	18703000	1223700	1.3E+08	0	2388200	1230500	4194400
Q9Y221	Q9Y221	60S ribosome subunit biogenesis protein NIP7 ho	NIP7	sp|Q9Y221	4	4	4	0	3	4	3	0	3	4	3	0	3	4	3	40	20.462	0	10.99	149830000	0	32746000	31851000	85232000	0	3777200	17424000	5855400
Q9HAU5	Q9HAU5	Regulator of nonsense transcripts 2	UPF2	sp|Q9HAU	9	9	9	0	6	8	4	0	6	8	4	0	6	8	4	9	147.81	0	17.681	149610000	0	50330000	36841000	62436000	0	5172400	21662000	4926600
Q8N8N7	Q8N8N7	Prostaglandin reductase 2	PTGR2	sp|Q8N8N	4	4	4	0	4	0	3	0	4	0	3	0	4	0	3	21.1	38.499	0	6.93	149520000	0	49520000	0	1E+08	0	4507700	0	3970200
Q9H4L5	Q9H4L5	Oxysterol-binding protein-related protein 3	OSBPL3	sp|Q9H4L5	6	6	6	1	2	5	6	1	2	5	6	1	2	5	6	10.7	101.22	0	17.691	149510000	599440	11097000	21477000	1.16E+08	2040700	2340700	6901000	9512000
Q9NZN8	Q9NZN8	CCR4-NOT transcription complex subunit 2	CNOT2	sp|Q9NZN	3	3	3	0	2	2	3	0	2	2	3	0	2	2	3	13.5	59.737	0	7.1957	149450000	0	33849000	9409400	1.06E+08	0	2679800	6469400	4524800
Q6VY07	Q6VY07	Phosphofurin acidic cluster sorting protein 1	PACS1	sp|Q6VY07	8	8	8	0	1	5	8	0	1	5	8	0	1	5	8	12.3	104.9	0	21.633	149400000	0	4762400	10290000	1.34E+08	0	0	8423800	4016100
Q9H0V9	Q9H0V9	VIP36-like protein	LMAN2L	sp|Q9H0V9	2	2	2	0	1	1	2	0	1	1	2	0	1	1	2	8.9	39.71	0	5.4639	149210000	0	35619000	2883500	1.11E+08	0	3020200	3888700	2721300
O75608	O75608	Acyl-protein thioesterase 1	LYPLA1	sp|O75608	3	3	3	0	3	0	3	0	3	0	3	0	3	0	3	13.9	24.669	0	6.6132	149200000	0	54765000	0	94436000	0	4747300	0	3964000
Q96PY5	Q96PY5	Formin-like protein 2	FMNL2	sp|Q96PY5	4	3	3	0	1	2	4	0	1	1	3	0	1	1	3	4.1	123.32	0	4.7562	148860000	0	95737000	1106900	52012000	0	7481800	2303500	1536600
Q9Y294	Q9Y294	Histone chaperone ASF1A	ASF1A	sp|Q9Y294	1	1	1	0	1	1	1	0	1	1	1	0	1	1	1	13.7	22.968	0	30.44	148540000	0	38853000	4091000	1.06E+08	0	3424900	2749100	4342900
Q9BV57	Q9BV57	1,2-dihydroxy-3-keto-5-methylthiopentene dioxy	ADI1	sp|Q9BV57	4	4	4	0	4	0	4	0	4	0	4	0	4	0	4	27.4	21.498	0	8.0648	148040000	0	63575000	0	84461000	0	5094600	0	3983000
Q9NUQ3;C	Q9NUQ3	Gamma-taxilin	TXLNG	sp|Q9NUC	7	7	7	0	7	3	5	0	7	3	5	0	7	3	5	16.1	60.585	0	21.594	148000000	0	1.01E+08	2032100	45154000	0	8370800	1670900	2067900
Q99661	Q99661	Kinesin-like protein KIF2C	KIF2C	sp|Q99661	6	6	5	0	5	3	5	0	5	3	5	0	4	2	4	11.4	81.312	0	13.781	147970000	0	66282000	7123700	74568000	0	5600800	5176500	2919100
Q5HYI8	Q5HYI8	Rab-like protein 3	RABL3	sp|Q5HYI8	3	3	3	1	3	3	3	1	3	3	3	1	3	3	3	12.7	26.422	0	6.8034	147920000	502310	42502000	4682200	1E+08	1642700	4059000	2531100	3284900
Q13017	Q13017	Rho GTPase-activating protein 5	ARHGAP5	sp|Q13017	3	3	3	0	2	0	3	0	2	0	3	0	2	0	3	4.1	172.46	0	9.4478	147900000	0	29396000	0	1.19E+08	0	2730800	0	4734100
O14653	O14653	Golgi SNAP receptor complex member 2	GOSR2	sp|O14653	3	3	3	0	1	0	3	0	1	0	3	0	1	0	3	19.3	24.775	0	14.988	147590000	0	13573000	0	1.34E+08	0	1970200	0	4737800
Q9NP77	Q9NP77	RNA polymerase II subunit A C-terminal domain p	SSU72	sp|Q9NP77	4	4	4	0	4	1	4	0	4	1	4	0	4	1	4	22.2	22.574	0	8.7499	147580000	0	51897000	2287800	93395000	0	2590300	5014600	2348100
Q8NEU8	Q8NEU8	DCC-interacting protein 13-beta	APPL2	sp|Q8NEU	6	6	6	0	0	2	6	0	0	2	6	0	0	2	6	13.7	74.493	0	11.652	147380000	0	0	1743300	1.46E+08	0	0	0	5989200
Q9Y5Q9	Q9Y5Q9	General transcription factor 3C polypeptide 3	GTF3C3	sp|Q9Y5Q9	5	5	5	2	5	4	4	2	5	4	4	2	5	4	4	6.5	101.27	0	10.464	147380000	6207300	64750000	7407400	69015000	10271000	3285700	4435500	1738700
Q8TEA8	Q8TEA8	D-tyrosyl-tRNA(Tyr) deacylase 1	DTD1	sp|Q8TEA8	2	2	2	0	1	1	2	0	1	1	2	0	1	1	2	20.1	23.423	0	31.858	147180000	0	34350000	1032400	1.12E+08	0	4826400	1105900	2387300
Q9NPL8	Q9NPL8	Complex I assembly factor TIMMDC1, mitochondr	TIMMDC1	sp|Q9NPL8	4	4	4	0	3	1	4	0	3	1	4	0	3	1	4	23.2	32.177	0	8.7298	147050000	0	36012000	518010	1.11E+08	0	2571500	2404000	3092200
O14548	O14548	Cytochrome c oxidase subunit 7A-related protein,	COX7A2L	sp|O14548	2	2	2	0	1	0	2	0	1	0	2	0	1	0	2	40.4	12.615	0	15.441	146880000	0	22943000	0	1.24E+08	0	2473700	0	4645700
Q8WXI9	Q8WXI9	Transcriptional repressor p66-beta	GATAD2B	sp|Q8WXI9	5	4	4	2	5	3	4	1	4	2	3	1	4	2	3	15.2	65.26	0	18.729	146720000	820630	89464000	2538700	53901000	1204300	7117000	2261600	2046500
P61964	P61964	WD repeat-containing protein 5	WDR5	sp|P61964	3	3	3	1	3	2	3	1	3	2	3	1	3	2	3	16.2	36.588	0	6.7531	146660000	4871500	60584000	5108500	76096000	6883600	4154700	3023000	2713100
Q14197	Q14197	Peptidyl-tRNA hydrolase ICT1, mitochondrial	ICT1	sp|Q14197	3	3	3	0	3	0	3	0	3	0	3	0	3	0	3	18.9	23.63	0	11.343	146440000	0	63506000	0	82930000	0	5088100	0	3920500
Q9H061	Q9H061	Transmembrane protein 126A	TMEM126A	sp|Q9H061	3	3	3	0	2	3	2	0	2	3	2	0	2	3	2	20	21.527	0	12.208	146330000	0	40318000	4434200	1.02E+08	0	3876600	2414100	4420400
P82909	P82909	28S ribosomal protein S36, mitochondrial	MRPS36	sp|P82909	3	3	3	1	3	2	2	1	3	2	2	1	3	2	2	44.7	11.466	0	17.051	146090000	4554200	59094000	6749200	75688000	4695600	4218300	4148600	4348900
Q14126	Q14126	Desmoglein-2	DSG2	sp|Q14126	5	5	5	0	3	0	4	0	3	0	4	0	3	0	4	7.2	122.29	0	12.277	145890000	0	30219000	0	1.16E+08	0	3792700	0	3628100
Q00059	Q00059	Transcription factor A, mitochondrial	TFAM	sp|Q00059	5	5	5	0	4	4	3	0	4	4	3	0	4	4	3	19.5	29.096	0	12.348	145540000	0	80166000	8574900	56801000	0	8140800	6078100	945990
P49356	P49356	Protein farnesyltransferase subunit beta	FNTB	sp|P49356	3	3	3	0	3	0	2	0	3	0	2	0	3	0	2	12.4	48.773	0.000945	3.099	145460000	0	47164000	0	98292000	0	3642100	0	4557600
P16278	P16278	Beta-galactosidase	GLB1	sp|P16278	3	3	3	1	3	0	2	1	3	0	2	1	3	0	2	5	76.074	0	7.7348	145460000	28226000	29661000	0	87576000	26303000	5131900	0	3007700
P53814	P53814	Smoothelin	SMTN	sp|P53814	10	10	10	0	0	8	9	0	0	8	9	0	0	8	9	14.7	99.058	0	30.816	145310000	0	0	16750000	1.29E+08	0	0	11201000	5343000
Q9NX74	Q9NX74	tRNA-dihydrouridine(20) synthase [NAD(P)+]-like	DUS2	sp|Q9NX74	4	4	4	0	3	0	4	0	3	0	4	0	3	0	4	10.8	55.05	0	6.3142	145180000	0	52781000	0	92403000	0	4826400	0	3626300
P12694	P12694	2-oxoisovalerate dehydrogenase subunit alpha, m	BCKDHA	sp|P12694	8	8	8	0	8	0	3	0	8	0	3	0	8	0	3	27.4	50.47	0	23.298	145140000	0	1.2E+08	0	25541000	0	9144400	0	2448500

A8MXV4	A8MXV4	Nucleoside diphosphate-linked moiety X motif 19	NUDT19	sp|A8MXV	5	5	5	0	4	1	4	0	4	1	4	0	4	1	4	21.9	42.233	0	15.443	144700000	0	75186000	1064800	68448000	0	3987200	3724300	2446500
Q6PJT7	Q6PJT7	Zinc finger CCCH domain-containing protein 14	ZC3H14	sp|Q6PJT7	5	5	5	1	4	1	5	1	4	1	5	1	4	1	5	9.1	82.875	0	11.427	144210000	662820	48011000	1797800	93738000	1564900	4044800	2200800	2147600
Q9H6R4	Q9H6R4	Nucleolar protein 6	NOL6	sp|Q9H6R	7	7	7	1	1	7	7	1	1	7	7	1	1	7	7	9.1	127.59	0	17.837	144160000	1110800	9861500	26119000	1.07E+08	3532000	1912000	14725000	3765800
Q8ND56	Q8ND56	Protein LSM14 homolog A	LSM14A	sp|Q8ND5	3	3	3	0	3	3	2	0	3	3	2	0	3	3	2	9.7	50.529	0	13.108	144070000	0	63342000	17340000	63388000	0	5138100	11185000	3520100
Q6UWP7	Q6UWP7	Lysocardiolipin acyltransferase 1	LCLAT1	sp|Q6UWF	4	4	4	1	3	0	4	1	3	0	4	1	3	0	4	11.1	48.92	0	7.5328	143920000	788760	40076000	0	1.03E+08	2720000	2765200	0	3074400
Q9Y3E7	Q9Y3E7	Charged multivesicular body protein 3	CHMP3	sp|Q9Y3E7	4	4	4	0	3	2	4	0	3	2	4	0	3	2	4	28.4	25.073	0	22.319	143890000	0	15596000	3281300	1.25E+08	0	1938100	2970900	3812200
Q9HD26	Q9HD26	Golgi-associated PDZ and coiled-coil motif-contair	GOPC	sp|Q9HD2	7	7	7	0	4	2	6	0	4	2	6	0	4	2	6	22.7	50.519	0	34.747	143850000	0	18397000	2316700	1.23E+08	0	3299100	2637900	2305700
Q92572	Q92572	AP-3 complex subunit sigma-1	AP3S1	sp|Q92572	4	4	4	0	3	1	4	0	3	1	4	0	3	1	4	22.3	21.732	0	11.743	143820000	0	39700000	2419300	1.02E+08	0	2743500	2528000	4036300
P36915	P36915	Guanine nucleotide-binding protein-like 1	GNL1	sp|P36915	4	4	4	0	4	1	3	0	4	1	3	0	4	1	3	8.4	68.66	0	14.488	143820000	0	93663000	2250200	47904000	0	7688600	1959400	2090500
Q92979	Q92979	Ribosomal RNA small subunit methyltransferase N	EMG1	sp|Q92979	3	3	3	1	3	1	2	1	3	1	2	1	3	1	2	16.8	26.72	0	8.5008	143800000	1060500	30714000	768360	1.11E+08	2952200	2133000	1827100	1947700
Q709C8	Q709C8	Vacuolar protein sorting-associated protein 13C	VPS13C	sp|Q709C8	9	9	9	0	0	1	9	0	0	1	9	0	0	1	9	3.5	422.39	0	22.457	143710000	0	0	459670	1.43E+08	0	0	388320	5811700
Q6PJG6	Q6PJG6	BRCA1-associated ATM activator 1	BRAT1	sp|Q6PJG6	6	6	6	0	2	1	6	0	2	1	6	0	2	1	6	9.7	88.118	0	25.018	143650000	0	26517000	1018800	1.16E+08	0	3022000	1728500	3046900
Q6PD74	Q6PD74	Alpha- and gamma-adaptin-binding protein p34	AAGAB	sp|Q6PD74	2	2	2	0	2	0	2	0	2	0	2	0	2	0	2	13	34.593	0	9.3017	143200000	0	71504000	0	71691000	0	6347100	0	2904200
Q71UI9;P0	Q71UI9;P0	Histone H2A.V;Histone H2A.Z	H2AFV;H2A	sp|Q71UI9	3	1	1	1	3	3	3	0	1	1	1	0	1	1	1	20.3	13.509	0.001569	2.3362	143080000	0	30904000	13737000	98438000	0	2724200	9057400	3725700
Q9H089	Q9H089	Large subunit GTPase 1 homolog	LSG1	sp|Q9H089	5	5	5	0	4	4	3	0	4	4	3	0	4	4	3	12.9	75.225	0	16.475	143070000	0	34381000	17800000	90890000	0	2147800	12535000	4047200
Q13595	Q13595	Transformer-2 protein homolog alpha	TRA2A	sp|Q13595	5	4	4	0	5	1	4	0	4	1	3	0	4	1	3	22.7	32.688	0	8.976	142960000	0	82938000	2279900	57745000	0	6583500	2577700	2056700
Q9UEE9	Q9UEE9	Craniofacial development protein 1	CFDP1	sp|Q9UEE9	4	4	4	0	4	0	2	0	4	0	2	0	4	0	2	20.1	33.592	0	7.7268	142930000	0	1.05E+08	0	38297000	0	8692900	0	2105100
Q9H490	Q9H490	Phosphatidylinositol glycan anchor biosynthesis cl	PIGU	sp|Q9H490	2	2	2	0	0	1	2	0	0	1	2	0	0	1	2	6.4	50.051	0	23.852	142880000	0	0	1101400	1.42E+08	0	0	1616000	4955000
O00625	O00625	Pirin	PIR	sp|O00625	3	3	3	0	2	0	3	0	2	0	3	0	2	0	3	15.5	32.113	0.000717	3.2852	142680000	0	1.1E+08	0	33036000	0	9664600	0	0
Q9H000	Q9H000	Probable E3 ubiquitin-protein ligase makorin-2	MKRN2	sp|Q9H000	4	4	4	1	3	2	4	1	3	2	4	1	3	2	4	16.1	46.94	0	4.172	142670000	2041900	42528000	638950	97459000	0	3149300	2932300	2104600
Q15121	Q15121	Astrocytic phosphoprotein PEA-15	PEA15	sp|Q15121	2	2	2	0	0	0	2	0	0	0	2	0	0	0	2	27.7	15.04	0	11.746	142470000	0	0	0	1.42E+08	0	0	0	5859000
Q9NZZ3	Q9NZZ3	Charged multivesicular body protein 5	CHMP5	sp|Q9NZZ3	1	1	1	0	1	1	0	0	1	1	0	0	1	1	0	8.2	24.57	0	4.3128	141930000	0	1.36E+08	5471200	0	0	12028000	3676600	0
Q96GC5	Q96GC5	39S ribosomal protein L48, mitochondrial	MRPL48	sp|Q96GC5	4	4	4	1	4	1	2	1	4	1	2	1	4	1	2	21.7	23.934	0	9.6158	141880000	770330	87834000	818160	52457000	0	4791400	2704000	2657200
Q96H79	Q96H79	Zinc finger CCCH-type antiviral protein 1-like	ZC3HAV1L	sp|Q96H79	5	5	5	0	5	1	3	0	5	1	3	0	5	1	3	32.3	32.962	0	29.531	141760000	0	1.15E+08	415450	25850000	0	8643900	1206400	1672400
P27487	P27487	Dipeptidyl peptidase 4;Dipeptidyl peptidase 4 me	DPP4	sp|P27487	1	1	1	1	0	0	0	1	0	0	0	1	0	0	0	2.7	88.278	0.009845	1.5106	141330000	1.41E+08	0	0	0	1.41E+08	0	0	0
Q9UGP4	Q9UGP4	LIM domain-containing protein 1	LIMD1	sp|Q9UGP	3	3	3	0	0	0	3	0	0	0	3	0	0	0	3	11.2	72.189	0	39.387	141060000	0	0	0	1.41E+08	0	0	0	5801000
Q15833	Q15833	Syntaxin-binding protein 2	STXBP2	sp|Q15833	5	5	5	0	5	0	1	0	5	0	1	0	5	0	1	12	66.452	0	12.633	140990000	0	85695000	0	55298000	0	7553900	0	2274200
Q9P016	Q9P016	Thymocyte nuclear protein 1	THYN1	sp|Q9P016	5	5	5	1	5	2	5	1	5	2	5	1	5	2	5	21.3	25.697	0	12.273	140960000	159550	90705000	1145800	48945000	232500	8581100	908660	1215700
P49750	P49750	YLP motif-containing protein 1	YLPM1	sp|P49750	7	7	7	1	6	3	7	1	6	3	7	1	6	3	7	6.1	241.64	0	16.971	140880000	1391000	32752000	7403100	99334000	2031500	2092800	6101300	2879400
O60573	O60573	Eukaryotic translation initiation factor 4E type 2	EIF4E2	sp|O60573	3	3	3	1	3	1	3	1	3	1	3	1	3	1	3	22.9	28.362	0	7.0676	140810000	882550	23996000	5920400	1.1E+08	2815700	1086500	5104100	2093500
Q9BTE1	Q9BTE1	Dynactin subunit 5	DCTN5	sp|Q9BTE1	2	2	2	0	2	2	2	0	2	2	2	0	2	2	2	11.5	20.126	0	5.8504	140700000	0	59012000	2683800	79003000	0	5185100	1824100	3245200
Q8WWV3	Q8WWV3	Reticulon-4-interacting protein 1, mitochondrial	RTN4IP1	sp|Q8WW	3	3	3	0	1	0	3	0	1	0	3	0	1	0	3	13.4	43.589	0	5.4989	140470000	0	2708700	0	1.38E+08	0	1257300	0	4646900
P06312	P06312	Ig kappa chain V-IV region	IGKV4-1	sp|P06312	3	3	3	1	0	2	1	1	0	2	1	1	0	2	1	19.8	13.38	0	9.7706	140440000	45613000	0	91418000	3407600	0	0	61285000	287450
Q9UKM7	Q9UKM7	Endoplasmic reticulum mannosyl-oligosaccharide	MAN1B1	sp|Q9UKM	5	5	5	0	1	3	5	0	1	3	5	0	1	3	5	11.6	79.579	0	47.293	140360000	0	3923100	4326000	1.32E+08	0	580590	2244000	5861400
Q9Y6K9	Q9Y6K9	NF-kappa-B essential modulator	IKBKG	sp|Q9Y6K9	5	5	5	2	5	1	3	2	5	1	3	2	5	1	3	16	48.197	0	30.25	140310000	1197600	75556000	1241300	62312000	1767300	5926800	1506500	2053900
Q9Y5Q8	Q9Y5Q8	General transcription factor 3C polypeptide 5	GTF3C5	sp|Q9Y5Q8	6	6	6	0	5	2	5	0	5	2	5	0	5	2	5	14.3	59.57	0	11.846	140260000	0	73045000	6772100	60439000	0	3819500	6860600	1721900
Q9H0C8	Q9H0C8	Integrin-linked kinase-associated serine/threonin	ILKAP	sp|Q9H0C8	6	6	6	0	5	1	5	0	5	1	5	0	5	1	5	16.8	42.906	0	13.191	140150000	0	58222000	899710	81025000	0	4825100	0	3639400
Q9BSV6	Q9BSV6	tRNA-splicing endonuclease subunit Sen34	TSEN34	sp|Q9BSV6	1	1	1	0	1	0	1	0	1	0	1	0	1	0	1	4.2	33.652	0	3.8872	139980000	0	20295000	0	1.2E+08	0	1789000	0	4922100
Q6IA86	Q6IA86	Elongator complex protein 2	ELP2	sp|Q6IA86	5	5	5	0	5	0	4	0	5	0	4	0	5	0	4	8.8	92.499	0	14.214	139910000	0	47735000	0	92178000	0	3822200	0	4176400
Q15102	Q15102	Platelet-activating factor acetylhydrolase IB subur	PAFAH1B3	sp|Q15102	4	4	4	0	2	0	4	0	2	0	4	0	2	0	4	27.3	25.734	0	25.205	139860000	0	35815000	0	1.04E+08	0	4348600	0	3087300
Q6IAA8	Q6IAA8	Ragulator complex protein LAMTOR1	LAMTOR1	sp|Q6IAA8	4	4	4	0	2	0	4	0	2	0	4	0	2	0	4	44.7	17.745	0	19.774	139740000	0	13026000	0	1.27E+08	0	912480	0	5447000
P52948	P52948	Nuclear pore complex protein Nup98-Nup96;Nucl	NUP98	sp|P52948	6	6	6	0	3	0	6	0	3	0	6	0	3	0	6	5.2	197.58	0	29.839	139650000	0	26342000	0	1.13E+08	0	2813700	0	4168300
Q05397	Q05397	Focal adhesion kinase 1	PTK2	sp|Q05397	8	8	8	0	0	0	8	0	0	0	8	0	0	0	8	11.5	119.23	0	26.119	139290000	0	0	0	1.39E+08	0	0	0	5728500
Q9NVH1	Q9NVH1	DnaJ homolog subfamily C member 11	DNAJC11	sp|Q9NVH	4	4	4	0	3	0	4	0	3	0	4	0	3	0	4	10.4	63.277	0	16.728	139200000	0	44652000	0	94552000	0	4214000	0	3610600
Q9UHJ6	Q9UHJ6	Sedoheptulokinase	SHPK	sp|Q9UHJ6	6	6	6	1	2	0	6	1	2	0	6	1	2	0	6	15.1	51.49	0	17.796	139140000	1070600	8514900	0	1.3E+08	0	1467200	0	4611200
Q9UJX2	Q9UJX2	Cell division cycle protein 23 homolog	CDC23	sp|Q9UJX2	10	10	10	2	3	10	5	2	3	10	5	2	3	10	5	17.9	68.833	0	26.322	138960000	1186600	5940100	93216000	38614000	509120	338210	64113000	978680
Q9NPD8	Q9NPD8	Ubiquitin-conjugating enzyme E2 T	UBE2T	sp|Q9NPD	4	4	4	0	4	0	3	0	4	0	3	0	4	0	3	25.9	22.521	0	11.967	138640000	0	78267000	0	60375000	0	5732800	0	3649200
O43617	O43617	Trafficking protein particle complex subunit 3	TRAPPC3	sp|O43617	4	4	4	0	3	0	4	0	3	0	4	0	3	0	4	24.4	20.274	0	9.4676	138550000	0	30738000	0	1.08E+08	0	3558300	0	3585200
Q96EY4	Q96EY4	Translation machinery-associated protein 16	TMA16	sp|Q96EY4	2	2	2	1	2	2	0	1	2	2	0	1	2	2	0	17.2	23.864	0.001563	2.2769	138460000	1027300	1.18E+08	19878000	0	4719500	10641000	9387500	0
P36406	P36406	E3 ubiquitin-protein ligase TRIM23	TRIM23	sp|P36406	1	1	1	0	1	0	1	0	1	0	1	0	1	0	1	1.7	64.066	0.000484	3.4191	138450000	0	29876000	0	1.09E+08	0	2633600	0	4465200
Q8N5M1	Q8N5M1	ATP synthase mitochondrial F1 complex assembly	ATPAF2	sp|Q8N5M	5	5	5	0	5	0	4	0	5	0	4	0	5	0	4	23.9	32.772	0	16.758	138430000	0	87121000	0	51310000	0	6382900	0	3406900
Q9Y608	Q9Y608	Leucine-rich repeat flightless-interacting protein 2	LRRFIP2	sp|Q9Y608	8	7	7	0	5	8	5	0	4	7	4	0	4	7	4	13.7	82.17	0	17.734	138350000	0	31573000	44126000	62652000	0	2484200	29102000	3426000
O75431	O75431	Metaxin-2	MTX2	sp|O75431	3	3	3	1	2	0	3	1	2	0	3	1	2	0	3	17.1	29.763	0	17.015	138200000	250780	21748000	0	1.16E+08	1401900	2949700	0	2595000
O43752	O43752	Syntaxin-6	STX6	sp|O43752	2	2	2	0	2	0	2	0	2	0	2	0	2	0	2	17.3	29.176	0	21.734	138160000	0	37537000	0	1.01E+08	0	3275000	0	4172200
Q9UEY8	Q9UEY8	Gamma-adducin	ADD3	sp|Q9UEY8	6	6	6	0	4	1	5	0	4	1	5	0	4	1	5	10.3	79.154	0	14.406	138000000	0	20905000	589790	1.17E+08	0	1813100	2351700	2865600
Q9BVI4	Q9BVI4	Nucleolar complex protein 4 homolog	NOC4L	sp|Q9BVI4	5	5	5	0	5	3	3	0	5	3	3	0	5	3	3	14.5	58.467	0	22.464	137830000	0	68899000	9041200	59890000	0	4674400	7635700	2301800
Q8TDB6	Q8TDB6	E3 ubiquitin-protein ligase DTX3L	DTX3L	sp|Q8TDB6	4	4	4	0	0	2	4	0	0	2	4	0	0	2	4	8.4	83.553	0	19.59	137680000	0	0	6750300	1.31E+08	0	0	6239100	3681400
P30536	P30536	Translocator protein	TSPO	sp|P30536	2	2	2	0	2	1	2	0	2	1	2	0	2	1	2	9.5	18.828	0.001136	2.5085	137650000	0	32167000	1873600	1.04E+08	0	2423800	2477700	3454200
P04183	P04183	Thymidine kinase, cytosolic	TK1	sp|P04183	2	2	2	0	1	0	2	0	1	0	2	0	1	0	2	13.2	25.468	0	4.5631	137370000	0	0	0	1.37E+08	0	0	0	5649600
Q9Y6W5	Q9Y6W5	Wiskott-Aldrich syndrome protein family member	WASF2	sp|Q9Y6W	3	3	3	0	3	1	2	0	3	1	2	0	3	1	2	7.6	54.283	0	39.061	137170000	0	24961000	2297800	1.1E+08	0	2196000	3082900	2985700
Q9UID3	Q9UID3	Vacuolar protein sorting-associated protein 51 ho	VPS51	sp|Q9UID3	6	6	6	0	3	0	5	0	3	0	5	0	3	0	5	11.3	86.041	0	32.772	136890000	0	12280000	0	1.25E+08	0	2126400	0	4080700
P45877	P45877	Peptidyl-prolyl cis-trans isomerase C	PPIC	sp|P45877	3	3	3	0	3	0	3	0	3	0	3	0	3	0	3	14.6	22.763	0	4.9458	136880000	0	36199000	0	1.01E+08	0	3052600	0	4279100
P49459	P49459	Ubiquitin-conjugating enzyme E2 A	UBE2A	sp|P49459	3	3	3	0	3	0	3	0	3	0	3	0	3	0	3	43.4	17.315	0	6.5402	136730000	0	62121000	0	74606000	0	5475900	0	0
Q9H4A5	Q9H4A5	Golgi phosphoprotein 3-like	GOLPH3L	sp|Q9H4A5	4	3	3	0	4	0	3	0	3	0	2	0	3	0	2	19.6	32.767	0	8.5339	136720000	0	62884000	0	73837000	0	5087700	0	3492100
O43395	O43395	U4/U6 small nuclear ribonucleoprotein Prp3	PRPF3	sp|O43395	7	7	7	0	4	0	7	0	4	0	7	0	4	0	7	15.7	77.528	0	25.656	136500000	0	34374000	0	1.02E+08	0	3562900	0	3667200
P20336	P20336	Ras-related protein Rab-3A	RAB3A	sp|P20336	3	2	2	0	2	0	2	0	2	0	1	0	2	0	1	17.7	24.984	0	6.0011	136460000	0	14333000	0	1.22E+08	0	971960	0	5314000
Q9UEU0	Q9UEU0	Vesicle transport through interaction with t-SNAR	VTI1B	sp|Q9UEU	4	4	4	1	1	2	3	1	1	2	3	1	1	2	3	25.9	26.688	0	15.614	136390000	27359000	9622200	6702400	92706000	25049000	3962100	5890200	1622000
O95182	O95182	NADH dehydrogenase [ubiquinone] 1 alpha subcc	NDUFA7	sp|O95182	3	3	3	1	3	0	3	1	3	0	3	1	3	0	3	32.7	12.551	0	10.174	136320000	2947700	63293000	0	70080000	2845300	6072300	0	2491400
Q9NXV2	Q9NXV2	BTB/POZ domain-containing protein KCTD5	KCTD5	sp|Q9NXV	2	2	2	0	1	0	2	0	1	0	2	0	1	0	2	20.5	26.092	0.000715	3.2428	136020000	0	21445000	0	1.15E+08	0	2011100	0	4591200
A0MZ66	A0MZ66	Shootin-1	KIAA1598	sp|A0MZ6	9	9	9	1	9	0	6	1	9	0	6	1	9	0	6	17.4	71.639	0	19.938	135980000	720280	1E+08	0	35164000	823740	8931500	0	1234200
Q8NCN5	Q8NCN5	Pyruvate dehydrogenase phosphatase regulatory	PDPR	sp|Q8NCN	5	5	5	0	3	0	4	0	3	0	4	0	3	0	4	7.3	99.363	0	22.526	135960000	0	14360000	0	1.22E+08	0	3960600	0	2306300
Q7Z3J2	Q7Z3J2	UPF0505 protein C16orf62	C16orf62	sp|Q7Z3J2	6	6	6	0	4	0	5	0	4	0	5	0	4	0	5	6.6	109.56	0	15.262	135780000	0	20291000	0	1.15E+08	0	2392900	0	4145400
Q53H96	Q53H96	Pyrroline-5-carboxylate reductase 3	PYCRL	sp|Q53H96	4	4	4	1	3	2	4	1	3	2	4	1	3	2	4	22.3	28.663	0	16.289	135600000	330750	42237000	2210500	90824000	1421700	2251500	1689800	3911500
O15382	O15382	Branched-chain-amino-acid aminotransferase, mi	BCAT2	sp|O15382	5	5	5	0	5	0	1	0	5	0	1	0	5	0	1	15.3	44.287	0	18.189	135370000	0	1.34E+08	0	1292400	0	11819000	0	0
Q9HCU5	Q9HCU5	Prolactin regulatory element-binding protein	PREB	sp|Q9HCU	3	3	3	0	3	3	1	0	3	3	1	0	3	3	1	13.7	45.468	0	14.509	135310000	0	71536000	6842300	56934000	0	5197800	3929000	4118500
O14656	O14656	Torsin-1A	TOR1A	sp|O14656	3	3	3	0	1	0	3	0	1	0	3	0	1	0	3	10.5	37.808	0	11.455	135230000	0	15515000	0	1.2E+08	0	2964600	0	3326400
Q96GQ5	Q96GQ5	RUS1 family protein C16orf58	C16orf58	sp|Q96GQ	2	2	2	0	0	0	2	0	0	0	2	0	0	0	2	7.7	51.017	0	9.2917	135200000	0	0	0	1.35E+08	0	0	0	5560000
Q9GZM5	Q9GZM5	Protein YIPF3;Protein YIPF3, 36 kDa form III	YIPF3	sp|Q9GZM	2	2	2	0	2	1	2	0	2	1	2	0	2	1	2	10.3	38.247	0	11.604	135020000	0	18402000	1656000	1.15E+08	0	1441200	1867100	4154700
Q15070	Q15070	Mitochondrial inner membrane protein OXA1L	OXA1L	sp|Q15070	3	3	3	0	2	3	3	0	2	3	3	0	2	3	3	6.4	48.547	0	5.8375	134400000	0	26498000	8169100	99737000	0	3193600	4847400	3886200
Q9C0E8	Q9C0E8	Protein lunapark	LNP	sp|Q9C0E8	3	3	3	0	3	2	3	0	3	2	3	0	3	2	3	10.7	47.739	0	5.2735	134220000	0	26121000	3164400	1.05E+08	0	2022100	2548700	4173600
Q9P253	Q9P253	Vacuolar protein sorting-associated protein 18 ho	VPS18	sp|Q9P253	3	3	3	0	1	0	3	0	1	0	3	0	1	0	3	4.1	110.18	0	10.307	134200000	0	18728000	0	1.15E+08	0	2431900	0	3967600
Q8N6M0	Q8N6M0	OTU domain-containing protein 6B	OTUD6B	sp|Q8N6M	3	3	3	0	2	1	3	0	2	1	3	0	2	1	3	15.4	33.812	0	24.46	134120000	0	6190900	2568500	1.25E+08	0	1092600	1943200	4391400
Q2NKX8	Q2NKX8	DNA excision repair protein ERCC-6-like	ERCC6L	sp|Q2NKX8	5	5	5	0	3	0	5	0	3	0	5	0	3	0	5	5	141.1	0	8.2435	134110000	0	21584000	0	1.13E+08	0	2563800	0	3966700
Q9Y3Y2	Q9Y3Y2	Chromatin target of PRMT1 protein	CHTOP	sp|Q9Y3Y2	3	3	3	2	3	3	2	2	3	3	2	2	3	3	2	14.5	26.396	0	9.5512	134110000	9736700	91078000	4787900	28504000	10071000	7683400	3620800	780000
O75351;Q6	O75351	Vacuolar protein sorting-associated protein 4B	VPS4B	sp|O75351	6	3	3	0	6	4	5	0	3	1	2	0	3	1	2	15.1	49.301	0	5.2355	134110000	0	1.11E+08	158050	23435000	0	5779600	1731200	3301400
Q9UL25	Q9UL25	Ras-related protein Rab-21	RAB21	sp|Q9UL25	4	4	4	0	3	0	2	0	3	0	2	0	3	0	2	23.6	24.347	0	14.218	133900000	0	40057000	0	93848000	0	3531000	0	0
O75150	O75150	E3 ubiquitin-protein ligase BRE1B	RNF40	sp|O75150	8	7	7	0	6	0	7	0	5	0	6	0	5	0	6	13.9	113.68	0	20.131	133810000	0	38104000	0	95710000	0	4572100	0	2722900
Q8WVC0	Q8WVC0	RNA polymerase-associated protein LEO1	LEO1	sp|Q8WVC	3	3	3	0	3	1	2	0	3	1	2	0	3	1	2	8.6	75.403	0	18.438	133780000	0	58265000	7692600	67821000	0	3923400	6442800	2728300
Q9UBT7	Q9UBT7	Alpha-catulin	CTNNAL1	sp|Q9UBT	2	2	2	0	0	1	2	0	0	1	2	0	0	1	2	4.8	81.895	0	14.492	133700000	0	0	1412600	1.32E+08	0	0	1089300	5300400

Q96S55	Q96S55	ATPase WRNIP1	WRNIP1	sp|Q96S55	6	6	6	4	5	0	0	4	5	0	0	4	5	0	0	12	72.132	0	20.304	133700000	75437000	58264000	0	0	74033000	6539400	0	0
Q8IVF7	Q8IVF7	Formin-like protein 3	FMNL3	sp|Q8IVF7	4	4	3	0	0	2	4	0	0	2	4	0	0	1	3	4.4	117.21	0	7.7604	133620000	0	0	12993000	1.21E+08	0	0	9620100	4072400
Q9BUT1	Q9BUT1	3-hydroxybutyrate dehydrogenase type 2	BDH2	sp|Q9BUT	5	5	5	0	5	0	3	0	5	0	3	0	5	0	3	28.6	26.724	0	15.85	133600000	0	92975000	0	40621000	0	7099300	0	2766900
Q9P260	Q9P260	LisH domain and HEAT repeat-containing protein	KIAA1468	sp|Q9P26	6	6	6	0	6	0	5	0	6	0	5	0	6	0	5	6.2	134.63	0	11.273	133450000	0	52020000	0	81434000	0	4363300	0	3571200
Q15287	Q15287	RNA-binding protein with serine-rich domain 1	RNPS1	sp|Q1528	3	3	3	1	2	3	2	1	2	3	2	1	2	3	2	11.5	34.208	0	10.818	133450000	7133200	82225000	11396000	32700000	7293200	8060700	6232500	1797900
P42356;A4	P42356	Phosphatidylinositol 4-kinase alpha	PI4KA	sp|P42356	7	7	7	0	1	0	7	0	1	0	7	0	1	0	7	3.8	236.83	0	13.573	133290000	0	7219900	0	1.26E+08	0	861250	0	4959800
Q13123	Q13123	Protein Red	IK	sp|Q1312	8	8	8	0	7	4	3	0	7	4	3	0	7	4	3	18.9	65.601	0	35.121	133170000	0	92237000	8768700	32166000	0	6937900	6245000	2163200
P10606	P10606	Cytochrome c oxidase subunit 5B, mitochondrial	COX5B	sp|P10606	5	5	5	0	5	0	3	0	5	0	3	0	5	0	3	25.6	13.696	0	11.034	133130000	0	88563000	0	44569000	0	6845900	0	2793700
Q9H1B7	Q9H1B7	Interferon regulatory factor 2-binding protein-like	IRF2BPL	sp|Q9H1B	7	7	7	0	1	0	7	0	1	0	7	0	1	0	7	14.2	82.658	0	23.146	133010000	0	3132600	0	1.3E+08	0	567450	0	5049800
P61966	P61966	AP-1 complex subunit sigma-1A	AP1S1	sp|P61966	3	3	3	0	2	1	3	0	2	1	3	0	2	1	3	22.2	18.733	0	48.607	132690000	0	19058000	1725200	1.12E+08	0	1706700	1258400	4476300
Q8TCG1	Q8TCG1	Protein CIP2A	KIAA1524	sp|Q8TCG	7	7	7	0	4	3	7	0	4	3	7	0	4	3	7	9.8	102.18	0	20.157	132680000	0	13059000	5157400	1.14E+08	0	2407100	3532100	3385300
Q96QC0	Q96QC0	Serine/threonine-protein phosphatase 1 regulato	PPP1R10	sp|Q96QC	6	6	6	0	5	3	5	0	5	3	5	0	5	3	5	6.8	99.057	0	10.815	132450000	0	46609000	4825200	81012000	0	4165500	3911600	2605600
Q9NS86	Q9NS86	LanC-like protein 2	LANCL2	sp|Q9NS8	3	3	3	0	3	0	3	0	3	0	3	0	3	0	3	10	50.854	0	8.6798	132230000	0	50223000	0	82010000	0	4250300	0	3549600
P27701	P27701	CD82 antigen	CD82	sp|P27701	1	1	1	0	0	0	1	0	0	0	1	0	0	0	1	5.2	29.625	0	3.9271	132170000	0	0	0	1.32E+08	0	0	0	5435600
Q6P161	Q6P161	39S ribosomal protein L54, mitochondrial	MRPL54	sp|Q6P16	1	1	1	0	1	1	0	0	1	1	0	0	1	1	0	18.8	15.819	0	22.035	132160000	0	1.2E+08	12485000	0	0	10549000	8389700	0
Q9BT73	Q9BT73	Proteasome assembly chaperone 3	PSMG3	sp|Q9BT7	4	4	4	0	4	0	2	0	4	0	2	0	4	0	2	50.8	13.104	0	13.759	132020000	0	84857000	0	47160000	0	6837400	0	2582200
O60231	O60231	Putative pre-mRNA-splicing factor ATP-dependen	DHX16	sp|O6023	8	7	7	1	7	1	6	0	6	0	5	0	6	0	5	9.5	119.26	0	19.272	131940000	0	67048000	0	64894000	0	5981200	0	2597800
P06702	P06702	Protein S100-A9	S100A9	sp|P06702	4	4	4	0	3	4	0	0	3	4	0	0	3	4	0	44.7	13.242	0	12.354	131930000	0	25126000	106800000	0	0	4666600	69319000	0
P49247	P49247	Ribose-5-phosphate isomerase	RPIA	sp|P49247	6	6	6	0	5	4	2	0	5	4	2	0	5	4	2	26	33.269	0	17.327	131680000	0	74201000	25074000	32401000	0	6612500	13167000	4944100
Q8NFH3	Q8NFH3	Nucleoporin Nup43	NUP43	sp|Q8NFH	4	4	4	0	1	1	4	0	1	1	4	0	1	1	4	18.9	42.15	0	12.091	131640000	0	6338500	1510000	1.24E+08	0	1472900	1302300	3889100
Q14651	Q14651	Plastin-1	PLS1	sp|Q1465	11	8	8	1	10	4	4	1	7	2	1	1	7	2	1	25.1	70.253	0	14.689	131500000	9651600	83686000	26996000	11169000	9924700	9932500	13607000	2165100
Q9NX47	Q9NX47	E3 ubiquitin-protein ligase MARCH5	Mar-05	sp|Q9NX4	3	3	3	0	1	0	3	0	1	0	3	0	1	0	3	14.7	31.231	0	8.8518	131280000	0	30763000	0	1.01E+08	0	3168500	0	3677000
Q9H0S4	Q9H0S4	Probable ATP-dependent RNA helicase DDX47	DDX47	sp|Q9H0S	7	7	7	0	5	5	5	0	5	5	5	0	5	5	5	20.9	50.646	0	16.071	131140000	0	37310000	15736000	78099000	0	3779900	9990800	3304700
Q9Y6N1	Q9Y6N1	Cytochrome c oxidase assembly protein COX11, n	COX11	sp|Q9Y6N	3	3	3	0	3	0	2	0	3	0	2	0	3	0	2	16.7	31.43	0	10.817	130710000	0	77832000	0	52876000	0	6502100	0	2533200
Q9UBU8	Q9UBU8	Mortality factor 4-like protein 1	MORF4L1	sp|Q9UBU	3	3	2	0	2	1	3	0	2	1	3	0	1	0	2	10.5	41.473	0	6.8325	130700000	0	22958000	1951300	1.06E+08	0	2919700	2216800	2548900
Q9BY42	Q9BY42	Protein RTF2 homolog	RTFDC1	sp|Q9BY4	2	2	2	0	2	0	2	0	2	0	2	0	2	0	2	9.5	33.886	0	8.2292	130700000	0	32534000	0	98166000	0	3766500	0	3138500
P25440	P25440	Bromodomain-containing protein 2	BRD2	sp|P25440	6	6	5	0	6	4	4	0	6	4	4	0	5	3	3	10.9	88.06	0	9.1456	130680000	0	82067000	7989200	40628000	0	6114000	6270300	1889400
P04424	P04424	Argininosuccinate lyase	ASL	sp|P04424	6	6	6	0	3	0	6	0	3	0	6	0	3	0	6	15.9	51.657	0	13.706	130650000	0	15730000	0	1.15E+08	0	1482300	0	4630500
O15427	O15427	Monocarboxylate transporter 4	SLC16A3	sp|O1542	4	4	4	0	1	1	4	0	1	1	4	0	1	1	4	9.7	49.469	0	11.506	130030000	0	1440200	1683200	1.27E+08	0	377620	1189000	4910700
O94966	O94966	Ubiquitin carboxyl-terminal hydrolase 19	USP19	sp|O9496	8	8	8	0	3	0	8	0	3	0	8	0	3	0	8	10.5	145.65	0	24.632	129760000	0	23060000	0	1.07E+08	0	2602400	0	3818400
P31751	P31751	RAC-beta serine/threonine-protein kinase	AKT2	sp|P31751	4	4	3	0	2	0	4	0	2	0	4	0	2	0	3	8.7	55.768	0	5.6482	129170000	0	8653600	0	1.21E+08	0	2626500	0	3092600
Q9NRX2	Q9NRX2	39S ribosomal protein L17, mitochondrial	MRPL17	sp|Q9NRX	2	2	2	0	2	1	2	0	2	1	2	0	2	1	2	13.1	20.05	0	10.496	129060000	0	52932000	4112900	72010000	0	3888600	4181300	2321300
Q8TBX8	Q8TBX8	Phosphatidylinositol 5-phosphate 4-kinase type-2	PIP4K2C	sp|Q8TBX	4	4	4	1	4	0	4	1	4	0	4	1	4	0	4	12.8	47.299	0	15.378	128950000	2070900	30661000	0	96219000	4658100	1577200	0	2495400
P51692;P4	P51692;P4	Signal transducer and activator of transcription 5	STAT5B;ST	sp|P51692	2	2	2	0	2	1	2	0	2	1	2	0	2	1	2	2.9	89.865	0	6.2641	128520000	0	75393000	2104400	51019000	0	5079500	1473600	1532400
Q9BYC8	Q9BYC8	39S ribosomal protein L32, mitochondrial	MRPL32	sp|Q9BYC	1	1	1	0	1	1	1	0	1	1	1	0	1	1	1	12.2	21.405	0	42.635	128500000	0	41983000	4216300	82299000	0	3776800	2934500	3207300
P55327	P55327	Tumor protein D52	TPD52	sp|P55327	3	3	3	0	3	1	0	0	3	1	0	0	3	1	0	34.4	24.327	0	8.6818	128350000	0	1.25E+08	2954900	0	0	10559000	2480600	0
Q9UNW1	Q9UNW1	Multiple inositol polyphosphate phosphatase 1	MINPP1	sp|Q9UNV	2	2	2	0	2	0	2	0	2	0	2	0	2	0	2	5.1	55.051	0.000248	3.7266	128230000	0	71357000	0	56876000	0	6187100	0	2442000
Q5VT52	Q5VT52	Regulation of nuclear pre-mRNA domain-containi	RPRD2	sp|Q5VT5	7	7	7	0	6	0	4	0	6	0	4	0	6	0	4	10.3	156.02	0	14.024	128180000	0	61802000	0	66381000	0	4711700	0	3466100
Q9UKS6	Q9UKS6	Protein kinase C and casein kinase substrate in ne	PACSIN3	sp|Q9UKS	3	3	3	0	2	3	1	0	2	3	1	0	2	3	1	9	48.486	0	4.5484	128130000	0	40259000	3017400	84853000	0	3739000	529750	3633500
P08581	P08581	Hepatocyte growth factor receptor	MET	sp|P08581	3	2	2	1	0	0	3	1	0	0	2	1	0	0	2	3.1	155.54	0	14.882	128100000	1155000	0	0	1.27E+08	4605100	0	0	1770400
Q15398	Q15398	Disks large-associated protein 5	DLGAP5	sp|Q1539	6	6	6	0	3	0	5	0	3	0	5	0	3	0	5	11.2	95.114	0	14.674	128080000	0	30963000	0	97116000	0	3358900	0	3364400
Q9H446	Q9H446	RWD domain-containing protein 1	RWDD1	sp|Q9H44	2	2	2	0	1	0	1	0	1	0	1	0	1	0	1	10.7	27.939	0	8.5128	127980000	0	4058400	0	1.24E+08	0	0	0	5096300
P04920	P04920	Anion exchange protein 2	SLC4A2	sp|P04920	4	4	4	0	0	1	4	0	0	1	4	0	0	1	4	5.6	137.01	0	11.066	127900000	0	0	2544100	1.25E+08	0	0	2597100	4267800
Q8WVV9	Q8WVV9	Heterogeneous nuclear ribonucleoprotein L-like	HNRNPLL	sp|Q8WV	4	4	4	0	4	3	3	0	4	3	3	0	4	3	3	10.1	60.082	0	10.814	127870000	0	80055000	3910800	43907000	0	4683100	2377300	3909600
Q147X3	Q147X3	N-alpha-acetyltransferase 30	NAA30	sp|Q147X	4	4	4	0	4	1	4	0	4	1	4	0	4	1	4	19.1	39.319	0	19.253	127810000	0	26906000	1025100	99882000	0	1856900	2530700	2780600
P40938	P40938	Replication factor C subunit 3	RFC3	sp|P40938	6	6	6	0	5	3	3	0	5	3	3	0	5	3	3	21.3	40.556	0	15.049	127420000	0	53696000	11893000	61827000	0	5096800	6902000	3269100
P42858	P42858	Huntingtin	HTT	sp|P42858	9	9	9	0	9	0	5	0	9	0	5	0	9	0	5	3.9	347.6	0	18.538	127370000	0	77397000	0	49975000	0	5622400	0	3255300
Q9UHR5	Q9UHR5	SAP30-binding protein	SAP30BP	sp|Q9UHR	3	3	3	0	3	2	3	0	3	2	3	0	3	2	3	12.3	33.87	0	8.9963	127220000	0	42793000	4004500	80420000	0	3353300	3194700	3222500
P78540	P78540	Arginase-2, mitochondrial	ARG2	sp|P78540	5	5	5	0	5	0	1	0	5	0	1	0	5	0	1	27.1	38.577	0	17.028	127180000	0	1.24E+08	0	3363200	0	10585000	0	138320
Q9UKJ3	Q9UKJ3	G patch domain-containing protein 8	GPATCH8	sp|Q9UKJ	9	9	9	1	9	4	5	1	9	4	5	1	9	4	5	9	164.2	0	32.776	127020000	11667000	74803000	7430900	33121000	14063000	4531200	5081600	940880
O00233	O00233	26S proteasome non-ATPase regulatory subunit 9	PSMD9	sp|O0023	5	5	5	0	2	0	4	0	2	0	4	0	2	0	4	31.4	24.682	0	14.349	126970000	0	35394000	0	91576000	0	2540200	0	4345900
Q01415	Q01415	N-acetylgalactosamine kinase	GALK2	sp|Q0141	3	3	3	0	2	0	3	0	2	0	3	0	2	0	3	9	50.378	0	12.794	126860000	0	13699000	0	1.13E+08	0	1895700	0	3965600
Q15646	Q15646	2-5-oligoadenylate synthase-like protein	OASL	sp|Q1564	6	6	6	0	0	4	5	0	0	4	5	0	0	4	5	17.5	59.225	0	24.456	126610000	0	0	17348000	1.09E+08	0	0	12947000	3204100
Q16763	Q16763	Ubiquitin-conjugating enzyme E2 S	UBE2S	sp|Q1676	4	4	4	0	3	0	4	0	3	0	4	0	3	0	4	28.8	23.845	0	11.617	126550000	0	29325000	0	97223000	0	2810500	0	3772800
Q9Y4J8;O6	Q9Y4J8	Dystrobrevin alpha	DTNA	sp|Q9Y4J8	5	5	5	0	5	2	5	0	5	2	5	0	5	2	5	10.8	83.9	0	27.295	126520000	0	29391000	1065600	96061000	0	1994600	1986000	3276900
P51159	P51159	Ras-related protein Rab-27A	RAB27A	sp|P51159	6	4	4	0	0	3	6	0	0	3	4	0	0	3	4	27.6	24.868	0	5.084	126500000	0	0	4048400	1.22E+08	0	0	3006100	4750300
P49916	P49916	DNA ligase 3	LIG3	sp|P49916	7	7	7	0	6	4	6	0	6	4	6	0	6	4	6	11.2	112.91	0	11.739	126370000	0	66534000	19306000	40533000	0	9164900	7344900	3995300
Q7L311	Q7L311	Armadillo repeat-containing X-linked protein 2	ARMCX2	sp|Q7L311	6	6	6	0	0	1	6	0	0	1	6	0	0	1	6	25	65.683	0	22.852	126350000	0	0	1536400	1.25E+08	0	0	1032400	5133100
P52434	P52434	DNA-directed RNA polymerases I, II, and III subun	POLR2H	sp|P52434	3	3	3	0	3	1	3	0	3	1	3	0	3	1	3	26.7	17.143	0	8.6535	126020000	0	42224000	2195300	81605000	0	3152200	2127700	3273400
Q8N9N8	Q8N9N8	Probable RNA-binding protein EIF1AD	EIF1AD	sp|Q8N9N	5	5	5	0	5	0	2	0	5	0	2	0	5	0	2	42.4	19.053	0	14.765	125860000	0	54901000	0	70962000	0	4433300	0	3324500
P24386	P24386	Rab proteins geranylgeranyltransferase compone	CHM	sp|P24386	3	3	3	0	3	0	2	0	3	0	2	0	3	0	2	8.6	73.475	0	24.279	125170000	0	38555000	0	86620000	0	3501400	0	3459400
Q9NVX2	Q9NVX2	Notchless protein homolog 1	NLE1	sp|Q9NVX	5	5	5	0	5	0	3	0	5	0	3	0	5	0	3	17.3	53.32	0	11.66	125140000	0	73383000	0	51758000	0	5311400	0	3285800
P27449	P27449	V-type proton ATPase 16 kDa proteolipid subunit	ATP6V0C	sp|P27449	1	1	1	0	1	1	1	0	1	1	1	0	1	1	1	11.6	15.736	0.000946	3.0999	125100000	0	47239000	3613300	74247000	0	4344100	2499100	2802500
A3KMH1	A3KMH1	von Willebrand factor A domain-containing prote	VWA8	sp|A3KMH	5	5	5	0	3	0	4	0	3	0	4	0	3	0	4	3.7	214.82	0	15.866	125090000	0	57205000	0	67881000	0	5199900	0	2634300
O75044;PC	O75044	SLIT-ROBO Rho GTPase-activating protein 2	SRGAP2	sp|O75044	10	10	9	0	2	10	7	0	2	10	7	0	2	9	6	11.3	120.87	0	61.147	124910000	0	9075800	45309000	70525000	0	998930	31274000	1875300
Q9UQ16	Q9UQ16	Dynamin-3	DNM3	sp|Q9UQ1	9	1	1	0	7	1	8	0	1	0	1	0	1	0	1	11.7	97.745	0.000722	3.3459	124900000	0	62362000	0	62539000	0	5497100	0	2571900
Q96T51;Q8	Q96T51	RUN and FYVE domain-containing protein 1	RUFY1	sp|Q96T51	6	6	5	0	5	4	5	0	5	4	5	0	4	4	4	8.2	79.817	0	14.971	124830000	0	36978000	7097100	80760000	0	2668300	5788000	2893800
O14735	O14735	CDP-diacylglycerol--inositol 3-phosphatidyltransfe	CDIPT	sp|O1473	2	2	2	0	1	0	2	0	1	0	2	0	1	0	2	10.8	23.539	0	5.7004	124730000	0	573890	0	1.24E+08	0	183270	0	4973400
Q5JSH3	Q5JSH3	WD repeat-containing protein 44	WDR44	sp|Q5JSH3	10	10	10	1	9	0	6	1	9	0	6	1	9	0	6	14.8	101.37	0	21.307	124540000	1223600	67982000	0	55333000	0	6053600	0	2214600
Q9NRK6;Q	Q9NRK6	ATP-binding cassette sub-family B member 10, m	ABCB10	sp|Q9NRK	7	7	6	1	6	0	5	1	6	0	5	1	5	0	4	11.4	79.147	0	17.288	124490000	132650	66456000	0	57902000	660880	6401500	0	1309500
Q9H9A5	Q9H9A5	CCR4-NOT transcription complex subunit 10	CNOT10	sp|Q9H9A	3	3	3	0	2	1	3	0	2	1	3	0	2	1	3	9.1	82.309	0	7.283	124390000	0	47905000	1709200	74772000	0	4754500	1528000	2163800
Q15139	Q15139	Serine/threonine-protein kinase D1	PRKD1	sp|Q1513	2	2	1	0	2	0	2	0	2	0	2	0	1	0	1	3.1	101.7	0	9.8204	124230000	0	14861000	0	1.09E+08	0	1409800	0	4398000
Q14691	Q14691	DNA replication complex GINS protein PSF1	GINS1	sp|Q1469	3	3	3	0	3	0	2	0	3	0	2	0	3	0	2	24	22.988	0	13.312	124230000	0	93775000	0	30455000	0	7983800	0	1534900
Q9BYI3	Q9BYI3	Hyccin	FAM126A	sp|Q9BYI3	4	4	4	0	0	1	4	0	0	1	4	0	0	1	4	8.6	57.625	0	9.9041	124130000	0	0	2409700	1.22E+08	0	0	2636900	3988100
Q9H269	Q9H269	Vacuolar protein sorting-associated protein 16 ho	VPS16	sp|Q9H26	4	4	4	1	2	1	4	1	2	1	4	1	2	1	4	6.1	94.693	0	17.276	124090000	1143300	14490000	1018100	1.07E+08	1817100	1707600	0	3314300
O60830	O60830	Mitochondrial import inner membrane transloca	TIMM17B	sp|O6083	3	3	3	0	3	0	2	0	3	0	2	0	3	0	2	25.6	18.273	0	20.145	123760000	0	1.03E+08	0	20661000	0	6557500	0	3380600
Q9NVT9	Q9NVT9	Armadillo repeat-containing protein 1	ARMC1	sp|Q9NVT	2	2	2	0	1	0	2	0	1	0	2	0	1	0	2	11.7	31.28	0	11.433	123630000	0	16346000	0	1.07E+08	0	2849100	0	3004100
Q8IWZ3	Q8IWZ3	Ankyrin repeat and KH domain-containing protein	ANKHD1	sp|Q8IWZ	6	6	4	1	4	1	4	1	4	1	4	1	2	1	2	3.7	269.45	0	19.572	123510000	268330	62934000	1063500	59245000	877650	4973400	1432300	1683700
Q13641	Q13641	Trophoblast glycoprotein	TPBG	sp|Q1364	3	3	3	0	0	0	3	0	0	0	3	0	0	0	3	10.2	46.031	0	14.469	123230000	0	0	0	1.23E+08	0	0	0	5068000
Q9NXR1;Q	Q9NXR1;Q	Nuclear distribution protein nudE homolog 1;Nuc	NDE1;NDE	sp|Q9NXR	2	2	2	0	2	0	2	0	2	0	2	0	2	0	2	9.3	37.72	0	23.547	123140000	0	20818000	0	1.02E+08	0	2248700	0	3794300
Q86TU7	Q86TU7	Histone-lysine N-methyltransferase setd3	SETD3	sp|Q86TU	5	5	5	0	4	0	4	0	4	0	4	0	4	0	4	13.3	67.256	0	15.454	123020000	0	52316000	0	70704000	0	4442700	0	3076700
A0A024R1	A0A024R1	Translation machinery-associated protein 7	hCG_2014	tr|A0A024	1	1	1	0	1	0	1	0	1	0	1	0	1	0	1	14.1	7.0913	0.002445	2.2146	122890000	0	63840000	0	59046000	0	5627400	0	2428300
Q9P270	Q9P270	SLAIN motif-containing protein 2	SLAIN2	sp|Q9P27	4	4	4	0	1	2	4	0	1	2	4	0	1	2	4	11.2	62.543	0	46.955	122740000	0	3889600	4621900	1.14E+08	0	1023000	4389000	2734400
O95881	O95881	Thioredoxin domain-containing protein 12	TXNDC12	sp|O9588	3	3	3	0	2	0	2	0	2	0	2	0	2	0	2	36.6	19.206	0	15.086	122570000	0	49706000	0	72864000	0	4747900	0	2630100
C4AMC7;C	C4AMC7;C	Putative WAS protein family homolog 3;WAS prot	WASH3P;V	sp|C4AMC	5	5	5	0	1	0	5	0	1	0	5	0	1	0	5	15.3	49.994	0	12.353	122430000	0	18832000	0	1.04E+08	0	0	0	4260500
Q8TDQ7	Q8TDQ7	Glucosamine-6-phosphate isomerase 2	GNPDA2	sp|Q8TDQ	6	3	3	0	4	0	5	0	2	0	3	0	2	0	3	28.3	31.084	0	9.479	122410000	0	26258000	0	96156000	0	2062600	0	4206500
O75976	O75976	Carboxypeptidase D	CPD	sp|O7597	6	6	6	0	1	0	6	0	1	0	6	0	1	0	6	5.5	152.93	0	17.144	122120000	0	9611600	0	1.13E+08	0	1253300	0	4220700
O94964	O94964	Protein SOGA1;N-terminal form;C-terminal 80 kD	SOGA1	sp|O9496	7	6	6	0	1	7	7	0	1	6	6	0	1	6	6	5.7	159.76	0	9.7457	121870000	0	3187900	19932000	98749000	0	1030900	14899000	1806800
O60885;Q1	O60885	Bromodomain-containing protein 4	BRD4	sp|O6088	5	4	4	0	5	2	5	0	4	1	4	0	4	1	4	5.6	152.22	0	10.742	121780000	0	53587000	673960	67520000	0	5024300	1023700	1905300

Q9BYV8	Q9BYV8	Centrosomal protein of 41 kDa	CEP41	sp|Q9BYV	3	3	3	0	1	0	3	0	1	0	3	0	1	0	3	8.6	41.368	0	5.733	121420000	0	21179000	0	1E+08	0	2285100	0	2142600
P40692	P40692	DNA mismatch repair protein Mlh1	MLH1	sp|P40692	4	4	4	0	0	0	4	0	0	0	4	0	0	0	4	9.3	84.6	0	24.492	121290000	0	0	0	1.21E+08	0	0	0	4988000
O95721	O95721	Synaptosomal-associated protein 29	SNAP29	sp|O95721	5	5	5	0	5	1	4	0	5	1	4	0	5	1	4	28.7	28.97	0	19.844	121260000	0	37624000	944900	82686000	0	2295700	2309800	2746600
Q8WVK2	Q8WVK2	U4/U6.U5 small nuclear ribonucleoprotein 27 kDa	SNRNP27	sp|Q8WVK	3	3	3	1	3	2	3	1	3	2	3	1	3	2	3	29	18.86	0	9.0826	121220000	4734200	57026000	5029100	54431000	6499300	3609700	3809700	1460400
Q13322	Q13322	Growth factor receptor-bound protein 10	GRB10	sp|Q13322	5	5	5	0	1	2	5	0	1	2	5	0	1	2	5	9.8	67.231	0	9.7049	121080000	0	12137000	2653700	1.06E+08	0	0	2206100	3948200
Q9BZE9	Q9BZE9	Tether containing UBX domain for GLUT4	ASPSCR1	sp|Q9BZE	5	5	5	0	5	0	2	0	5	0	2	0	5	0	2	18.8	60.182	0	6.8614	120840000	0	79813000	0	41024000	0	5329200	0	3393400
Q9BUK6;R	Q9BUK6	Protein misato homolog 1	MSTO1	sp|Q9BUK	6	6	6	0	5	2	4	0	5	2	4	0	5	2	4	13.9	61.835	0	21.567	120820000	0	40900000	2826700	77095000	0	2966200	1759600	3949700
P22830	P22830	Ferrochelatase, mitochondrial	FECH	sp|P22830	5	5	5	0	5	2	2	0	5	2	2	0	5	2	2	16.1	47.862	0	13.68	120800000	0	1.07E+08	2498400	11294000	0	8707000	1735700	1133400
Q13740	Q13740	CD166 antigen	ALCAM	sp|Q1374	2	2	2	0	0	0	2	0	0	0	2	0	0	0	2	5	65.102	0	13.933	120740000	0	0	0	1.21E+08	0	0	0	4965700
Q8WZ42	Q8WZ42	Titin	TTN	sp|Q8WZ4	3	3	3	0	1	1	3	0	1	1	3	0	1	1	3	0.1	3816	0.006003	1.762	120700000	0	24664000	6816800	89217000	0	2599600	4708900	3115600
Q8IUR7	Q8IUR7	Armadillo repeat-containing protein 8	ARMC8	sp|Q8IUR7	4	4	4	1	4	1	4	1	4	1	4	1	4	1	4	8	75.509	0	13.641	120390000	196610	50026000	1109700	69059000	0	4527500	0	2722300
Q9GZN8	Q9GZN8	UPF0687 protein C20orf27	C20orf27	sp|Q9GZN	1	1	1	0	1	0	1	0	1	0	1	0	1	0	1	8	19.291	0	5.1826	120370000	0	60648000	0	59721000	0	5346000	0	2456000
Q8TD16	Q8TD16	Protein bicaudal D homolog 2	BICD2	sp|Q8TD1	6	6	6	0	3	0	6	0	3	0	6	0	3	0	6	9	93.532	0	21.776	120090000	0	15522000	0	1.05E+08	0	2587300	0	3081200
O75496	O75496	Geminin	GMNN	sp|O7549	3	3	3	0	3	0	1	0	3	0	1	0	3	0	1	23.4	23.565	0	4.0702	120060000	0	1.16E+08	0	4126000	0	8083500	0	2305800
P61916	P61916	Epididymal secretory protein E1	NPC2	sp|P61916	2	2	2	0	1	0	2	0	1	0	2	0	1	0	2	17.2	16.57	0	6.9906	119960000	0	6917900	0	1.13E+08	0	1101300	0	4157500
Q9H0H5	Q9H0H5	Rac GTPase-activating protein 1	RACGAP1	sp|Q9H0H	6	6	6	0	5	4	6	0	5	4	6	0	5	4	6	14.4	71.026	0	11.055	119890000	0	28573000	11748000	79565000	0	2272100	8747400	2665900
Q9Y314	Q9Y314	Nitric oxide synthase-interacting protein	NOSIP	sp|Q9Y31	4	4	4	0	4	1	4	0	4	1	4	0	4	1	4	20.9	33.172	0	6.611	119660000	0	47889000	2767800	69003000	0	4514000	0	2545200
Q92599	Q92599	Septin-8	Sep-08	sp|Q9259	6	4	4	1	5	2	6	1	3	1	4	1	3	1	4	14.3	55.756	0	11.525	119650000	98169	37444000	822090	81285000	691940	3983500	951750	1666900
Q96CP2;Q4	Q96CP2	FLYWCH family member 2	FLYWCH2	sp|Q96CP	5	5	5	1	5	0	5	1	5	0	5	1	5	0	5	45.7	14.563	0	15.518	119520000	485320	93194000	0	25842000	434960	8633300	0	694830
Q92562	Q92562	Polyphosphoinositide phosphatase	FIG4	sp|Q9256	4	4	4	1	1	1	3	1	1	1	3	1	1	1	3	6.2	103.63	0	6.8773	119200000	0	52303000	7439700	59455000	0	5534100	4163300	2357600
Q9Y6D9	Q9Y6D9	Mitotic spindle assembly checkpoint protein MAD	MAD1L1	sp|Q9Y6D	6	6	6	0	6	2	3	0	6	2	3	0	6	2	3	11.3	83.066	0	20.122	119180000	0	70383000	1276600	47517000	0	4852600	1720200	2443400
E9PRG8	E9PRG8	Uncharacterized protein C11orf98	C11orf98	sp|E9PRG	2	2	2	0	1	2	1	0	1	2	1	0	1	2	1	21.1	14.234	0	4.1874	119080000	0	34026000	17939000	67116000	0	2999300	12055000	2760200
Q15650	Q15650	Activating signal cointegrator 1	TRIP4	sp|Q1565	5	5	5	0	5	3	5	0	5	3	5	0	5	3	5	11.2	66.145	0	7.7544	119060000	0	45283000	5825400	67948000	0	2828100	5340300	2136200
Q0VGL1	Q0VGL1	Ragulator complex protein LAMTOR4;Ragulator c	LAMTOR4	sp|Q0VGL	1	1	1	1	1	1	0	1	1	1	0	1	1	1	0	10.1	10.741	0.004331	1.8973	118980000	3567600	34291000	81123000	0	2233500	2055800	56815000	0
Q8TDZ2	Q8TDZ2	Protein-methionine sulfoxide oxidase MICAL1	MICAL1	sp|Q8TDZ	5	5	5	0	0	0	5	0	0	0	5	0	0	0	5	8.4	117.87	0	15.689	118880000	0	0	0	1.19E+08	0	0	0	4888900
P48634	P48634	Protein PRRC2A	PRRC2A	sp|P48634	5	4	4	0	3	5	4	0	2	4	3	0	2	4	3	4.7	228.86	0	7.3785	118760000	0	35546000	16411000	66806000	0	7021500	7009700	2877900
Q8WTW3	Q8WTW3	Conserved oligomeric Golgi complex subunit 1	COG1	sp|Q8WTV	5	5	5	0	3	0	5	0	3	0	5	0	3	0	5	7.7	108.98	0	13.323	118680000	0	25374000	0	93301000	0	2969500	0	3104300
P78318;A0	P78318	Immunoglobulin-binding protein 1	IGBP1	sp|P78318	3	3	3	0	2	0	3	0	2	0	3	0	2	0	3	14.5	39.221	0	18.071	118680000	0	25960000	0	92722000	0	3271300	0	2830300
Q16878	Q16878	Cysteine dioxygenase type 1	CDO1	sp|Q1687	1	1	1	0	1	1	1	0	1	1	1	0	1	1	1	3.5	22.972	0.003485	1.9784	118520000	0	41501000	866830	76149000	0	3658200	582500	3131700
Q9Y4K4	Q9Y4K4	Mitogen-activated protein kinase kinase kinase ki	MAP4K5	sp|Q9Y4K	7	7	7	0	2	0	7	0	2	0	7	0	2	0	7	13.9	95.023	0	18.211	118470000	0	10904000	0	1.08E+08	0	1524400	0	3860400
Q86UY8	Q86UY8	5-nucleotidase domain-containing protein 3	NT5DC3	sp|Q86UY	3	3	3	0	3	0	2	0	3	0	2	0	3	0	2	11.3	63.419	0	11.614	118460000	0	50329000	0	68127000	0	4289600	0	2948700
Q9P2N5	Q9P2N5	RNA-binding protein 27	RBM27	sp|Q9P2N	7	6	6	1	7	5	4	0	6	4	3	0	6	4	3	8.8	118.72	0	10.287	118370000	0	55642000	11407000	51322000	0	3589800	8059500	3031700
O43660	O43660	Pleiotropic regulator 1	PLRG1	sp|O4366	4	4	4	0	2	0	4	0	2	0	4	0	2	0	4	17.7	57.193	0	18.998	118260000	0	25093000	0	93164000	0	2414600	0	3628700
Q92636	Q92636	Protein FAN	NSMAF	sp|Q9263	2	2	2	0	0	0	2	0	0	0	2	0	0	0	2	5	104.37	0	8.8291	117950000	0	0	0	1.18E+08	0	0	0	4850800
O00139	O00139	Kinesin-like protein KIF2A	KIF2A	sp|O0013	5	4	4	0	4	2	5	0	3	1	4	0	3	1	4	11.9	79.954	0	11.638	117730000	0	41957000	5808400	69962000	0	3942100	4714800	1822000
Q9UM54	Q9UM54	Unconventional myosin-VI	MYO6	sp|Q9UM5	8	8	8	3	8	0	2	3	8	0	2	3	8	0	2	8.7	149.69	0	17.594	117670000	9203600	94847000	0	13624000	10688000	5810100	0	1626400
Q5VYS8	Q5VYS8	Terminal uridylyltransferase 7	ZCCHC6	sp|Q5VYS	6	6	6	0	0	5	4	0	0	5	4	0	0	5	4	6.4	171.23	0	11.007	117630000	0	0	18091000	99542000	0	0	10131000	6120500
O75794	O75794	Cell division cycle protein 123 homolog	CDC123	sp|O7579	3	3	3	0	3	0	3	0	3	0	3	0	3	0	3	11	39.134	0	7.6487	117610000	0	13201000	0	1.04E+08	0	1087100	0	4370500
Q5SY16	Q5SY16	Polynucleotide 5-hydroxyl-kinase NOL9	NOL9	sp|Q5SY1	5	5	5	0	5	1	0	0	5	1	0	0	5	1	0	12.4	79.322	0	25.541	117450000	0	1.14E+08	3602600	0	0	9336200	3120700	0
Q99470	Q99470	Stromal cell-derived factor 2	SDF2	sp|Q9947	3	3	3	0	2	1	3	0	2	1	3	0	2	1	3	25.1	23.026	0	10.157	117310000	0	13036000	2060100	1.02E+08	0	1342300	1667200	3727400
Q9UBN6	Q9UBN6	Tumor necrosis factor receptor superfamily mem	TNFRSF10I	sp|Q9UBN	1	1	1	0	0	0	1	0	0	0	1	0	0	0	1	7.5	41.823	0	22.246	117170000	0	0	0	1.17E+08	0	0	0	4818700
P09488;QC	P09488;QC	Glutathione S-transferase Mu 1;Glutathione S-tra	GSTM1;GS	sp|P09488	5	2	1	1	3	2	5	0	0	1	2	0	0	0	1	29.8	25.712	0	11.532	117030000	0	0	3848600	1.13E+08	0	0	2836200	4404600
O43294	O43294	Transforming growth factor beta-1-induced trans	TGFB1I1	sp|O4329	2	2	2	0	1	2	2	0	1	2	2	0	1	2	2	6.3	49.814	0	6.2067	116890000	0	23071000	9531400	84286000	0	2907400	5666800	3330800
O43678	O43678	NADH dehydrogenase [ubiquinone] 1 alpha subcc	NDUFA2	sp|O4367	2	2	2	0	2	0	2	0	2	0	2	0	2	0	2	31.3	10.921	0	11.139	116610000	0	73494000	0	43114000	0	5512900	0	2738600
O76071	O76071	Probable cytosolic iron-sulfur protein assembly pr	CIAO1	sp|O76071	3	3	3	0	2	1	3	0	2	1	3	0	2	1	3	7.7	37.84	0	6.5212	116490000	0	31814000	3235700	81437000	0	2744200	3346100	2237500
P00736;Q9	P00736	Complement C1r subcomponent;Complement C1	C1R	sp|P00736	4	4	4	1	1	4	2	1	1	4	2	1	1	4	2	7	80.118	0	12.211	116490000	1702300	7929600	87797000	19056000	569950	365220	60694000	554670
Q96C57	Q96C57	Uncharacterized protein C12orf43	C12orf43	sp|Q96C5	5	5	5	0	4	4	4	0	4	4	4	0	4	4	4	27.5	28.17	0	15.135	116370000	0	77530000	9630900	29210000	0	5261400	7396000	1849900
Q05639	Q05639	Elongation factor 1-alpha 2	EEF1A2	sp|Q0563	14	2	2	6	12	10	11	0	2	0	0	0	2	0	0	43.6	50.47	0	9.038	116070000	0	1.16E+08	0	0	0	10232000	0	0
Q96G28	Q96G28	Cilia- and flagella-associated protein 36	CFAP36	sp|Q96G2	4	4	4	0	4	0	3	0	4	0	3	0	4	0	3	20.5	39.446	0	5.8325	115840000	0	49530000	0	66313000	0	3194200	0	3899000
P55011	P55011	Solute carrier family 12 member 2	SLC12A2	sp|P55011	7	7	7	0	6	1	5	0	6	1	5	0	6	1	5	9.7	131.45	0	14.191	115700000	0	58173000	326540	57198000	0	3116300	2307400	2275800
Q93052	Q93052	Lipoma-preferred partner	LPP	sp|Q93052	5	5	5	0	1	0	5	0	1	0	5	0	1	0	5	17.8	65.746	0	13.224	115670000	0	4282400	0	1.11E+08	0	696400	0	4261800
P20839	P20839	Inosine-5-monophosphate dehydrogenase 1	IMPDH1	sp|P20839	9	6	6	0	9	2	3	0	6	0	0	0	6	0	0	24.3	55.405	0	14.728	115600000	0	1.16E+08	0	0	0	10190000	0	0
Q9H6S0	Q9H6S0	Probable ATP-dependent RNA helicase YTHDC2	YTHDC2	sp|Q9H6S	6	6	6	1	6	2	3	1	6	2	3	1	6	2	3	6.3	160.25	0	20.626	115520000	1208300	73448000	4000100	36862000	0	4459400	4630300	1588600
Q9UKA4	Q9UKA4	A-kinase anchor protein 11	AKAP11	sp|Q9UKA	7	7	7	0	0	3	7	0	0	3	7	0	0	3	7	6.8	210.51	0	20.277	115130000	0	0	3841200	1.11E+08	0	0	4056800	3101200
Q96JM3	Q96JM3	Chromosome alignment-maintaining phosphopro	CHAMP1	sp|Q96JM	3	3	3	0	2	2	3	0	2	2	3	0	2	2	3	5.2	89.098	0	10.765	115000000	0	10413000	3666800	1.01E+08	0	1734200	1985100	3813100
Q8IWW6	Q8IWW6	Rho GTPase-activating protein 12	ARHGAP12	sp|Q8IWW	5	5	5	0	0	1	5	0	0	1	5	0	0	1	5	11.7	96.253	0	11.568	114990000	0	0	256310	1.15E+08	0	0	172240	4718300
Q9NVG8	Q9NVG8	TBC1 domain family member 13	TBC1D13	sp|Q9NVG	4	4	4	0	4	0	3	0	4	0	3	0	4	0	3	11.8	46.553	0	5.5987	114900000	0	53059000	0	61841000	0	5168900	0	2051500
Q86SZ2	Q86SZ2	Trafficking protein particle complex subunit 6B	TRAPPC6B	sp|Q86SZ2	4	4	4	0	2	0	4	0	2	0	4	0	2	0	4	29.7	17.983	0	12.629	114700000	0	28425000	0	86279000	0	2484300	0	3569600
P40306	P40306	Proteasome subunit beta type-10	PSMB10	sp|P40306	1	1	1	0	0	0	1	0	0	0	1	0	0	0	1	7.3	28.936	0	9.8725	114680000	0	0	0	1.15E+08	0	0	0	4716400
Q8N122	Q8N122	Regulatory-associated protein of mTOR	RPTOR	sp|Q8N12	3	3	3	0	2	0	2	0	2	0	2	0	2	0	2	3.7	149.04	0	11.038	114340000	0	6797200	0	1.08E+08	0	0	0	4422800
Q7Z4L5	Q7Z4L5	Tetratricopeptide repeat protein 21B	TTC21B	sp|Q7Z4L5	1	1	1	1	0	1	1	1	0	1	1	1	0	1	1	0.5	150.94	0.007433	1.6628	114170000	38124000	0	38241000	37804000	38124000	0	25698000	1554700
Q6P1N0	Q6P1N0	Coiled-coil and C2 domain-containing protein 1A	CC2D1A	sp|Q6P1N	6	6	6	0	2	1	6	0	2	1	6	0	2	1	6	9.7	104.06	0	12.227	114160000	0	10930000	865580	1.02E+08	0	1719900	1791400	2243700
P29508;P4	P29508;P4	Serpin B3;Serpin B4	SERPINB3;	sp|P29508	10	10	10	0	1	9	5	0	1	9	5	0	1	9	5	31.3	44.564	0	20.572	113980000	0	1840500	56499000	55645000	0	351690	36058000	1720000
Q9UJV9	Q9UJV9	Probable ATP-dependent RNA helicase DDX41	DDX41	sp|Q9UJV	6	6	6	2	5	5	3	2	5	5	3	2	5	5	3	11.9	69.837	0	10.787	113950000	3207800	46222000	17571000	46953000	3987300	3903400	10223000	2906700
Q96P48	Q96P48	Arf-GAP with Rho-GAP domain, ANK repeat and P	ARAP1	sp|Q96P4	5	5	5	0	4	0	5	0	4	0	5	0	4	0	5	4.6	162.19	0	9.7919	113570000	0	20666000	0	92904000	0	1083200	0	4559200
Q9Y3P9	Q9Y3P9	Rab GTPase-activating protein 1	RABGAP1	sp|Q9Y3P	4	4	4	1	4	0	4	1	4	0	4	1	4	0	4	5.4	121.74	0	14.1	113520000	408600	25872000	0	87240000	636460	2047500	0	3592900
Q15678	Q15678	Tyrosine-protein phosphatase non-receptor type	PTPN14	sp|Q1567	4	4	4	0	1	4	4	0	1	4	4	0	1	4	4	6.2	135.26	0	14.159	113380000	0	3111800	15881000	94386000	0	3318700	7375200	4134400
P83436	P83436	Conserved oligomeric Golgi complex subunit 7	COG7	sp|P83436	4	4	4	0	3	0	3	0	3	0	3	0	3	0	3	8.1	86.343	0	16.041	113290000	0	28626000	0	84666000	0	2336000	0	3669200
P28062	P28062	Proteasome subunit beta type-8	PSMB8	sp|P28062	4	4	4	0	0	0	4	0	0	0	4	0	0	0	4	25.7	30.354	0	33.749	113040000	0	0	0	1.13E+08	0	0	0	4648800
P05106	P05106	Integrin beta-3	ITGB3	sp|P05106	5	5	5	0	0	0	5	0	0	0	5	0	0	0	5	8.8	87.057	0	16.288	113000000	0	0	0	1.13E+08	0	0	0	4647200
P82663	P82663	28S ribosomal protein S25, mitochondrial	MRPS25	sp|P82663	4	4	4	2	4	2	4	2	4	2	4	2	4	2	4	29.5	20.116	0	14.793	112850000	4803800	65767000	2340600	39939000	5237600	4749000	2469100	1360800
Q9BPZ3	Q9BPZ3	Polyadenylate-binding protein-interacting protein	PAIP2	sp|Q9BPZ	1	1	1	0	1	1	1	0	1	1	1	0	1	1	1	11	14.984	0	11.524	112790000	0	39511000	27135000	46142000	0	4462400	14481000	4671500
Q9P0J1	Q9P0J1	[Pyruvate dehydrogenase [acetyl-transferring]]-pl	PDP1	sp|Q9P0J1	6	6	6	0	2	0	6	0	2	0	6	0	2	0	6	19.6	61.053	0	31.808	112770000	0	24079000	0	88690000	0	2434400	0	3335500
Q7Z434	Q7Z434	Mitochondrial antiviral-signaling protein	MAVS	sp|Q7Z434	4	4	4	0	3	0	4	0	3	0	4	0	3	0	4	13.1	56.527	0	8.8668	112720000	0	25089000	0	87631000	0	3006800	0	2808600
O00330	O00330	Pyruvate dehydrogenase protein X component, m	PDHX	sp|O0033	5	5	5	0	5	0	2	0	5	0	2	0	5	0	2	12	54.122	0	13.329	112670000	0	1.04E+08	0	8901500	0	8290700	0	1222700
P19838	P19838	Nuclear factor NF-kappa-B p105 subunit;Nuclear f	NFKB1	sp|P19838	3	3	3	0	1	3	2	0	1	3	2	0	1	3	2	6	105.35	0	24.108	112560000	0	22061000	24251000	66249000	0	4490300	13571000	2904400
Q7Z422	Q7Z422	SUZ domain-containing protein 1	SZRD1	sp|Q7Z42	2	2	2	0	2	0	2	0	2	0	2	0	2	0	2	28.3	16.997	0	7.6585	112350000	0	28736000	0	83610000	0	2622600	0	3349000
Q9H3H3	Q9H3H3	UPF0696 protein C11orf68	C11orf68	sp|Q9H3H	4	4	4	0	1	0	4	0	1	0	4	0	1	0	4	17.1	31.43	0	8.0364	112260000	0	27756000	0	84500000	0	2706000	0	3215700
P05412	P05412	Transcription factor AP-1	JUN	sp|P05412	2	2	2	0	2	2	2	0	2	2	2	0	2	2	2	13.9	35.675	0	6.8407	112160000	0	19466000	10073000	82621000	0	1829100	7141100	2912300
P32929	P32929	Cystathionine gamma-lyase	CTH	sp|P32929	5	5	5	0	5	0	2	0	5	0	2	0	5	0	2	15.6	44.508	0	18.023	111910000	0	98034000	0	13874000	0	8213100	0	999140
P29083	P29083	General transcription factor IIE subunit 1	GTF2E1	sp|P29083	4	4	4	0	3	0	3	0	3	0	3	0	3	0	3	15.7	49.452	0	22.028	111870000	0	56375000	0	55494000	0	4642300	0	2609300
Q92600	Q92600	Cell differentiation protein RCD1 homolog	RQCD1	sp|Q9260	4	4	4	0	4	3	3	0	4	3	3	0	4	3	3	14	33.631	0	6.25	111750000	0	51683000	8199600	51870000	0	3799000	5719800	2680200
Q14344	Q14344	Guanine nucleotide-binding protein subunit alpha	GNA13	sp|Q1434	3	3	3	0	2	0	3	0	2	0	3	0	2	0	3	9.8	44.049	0	12.381	111710000	0	34642000	0	77068000	0	3484900	0	2738300
Q96BW5	Q96BW5	Phosphotriesterase-related protein	PTER	sp|Q96BW	3	3	3	0	3	0	0	0	3	0	0	0	3	0	0	16.3	39.017	0	4.3931	111480000	0	1.11E+08	0	0	0	9826600	0	0
P18583	P18583	Protein SON	SON	sp|P18583	5	5	5	0	5	0	2	0	5	0	2	0	5	0	2	3.6	263.83	0	9.6597	111440000	0	98001000	0	13442000	0	7530100	0	1324900
Q08554	Q08554	Desmocollin-1	DSC1	sp|Q0855	3	3	3	1	2	3	2	1	2	3	2	1	2	3	2	4.7	99.986	0	8.87	111390000	298750	13196000	90767000	7127000	177400	547620	61742000	282580
Q9UBB9	Q9UBB9	Tuftelin-interacting protein 11	TFIP11	sp|Q9UBB	3	3	3	0	1	0	3	0	1	0	3	0	1	0	3	5.1	96.819	0	4.4595	111360000	0	13069000	0	98289000	0	2348700	0	2845500
O75886	O75886	Signal transducing adapter molecule 2	STAM2	sp|O7588	5	4	4	0	3	2	4	0	2	1	3	0	2	1	3	11.8	58.164	0	14.475	111210000	0	13985000	4937900	92288000	0	1363700	4301500	2681300
Q9Y625;O7	Q9Y625;O7	Glypican-6;Secreted glypican-6;Glypican-4;Secret	GPC6;GPC	sp|Q9Y625	2	2	2	0	1	1	2	0	1	1	2	0	1	1	2	5.8	62.735	0	8.7513	111070000	0	23647000	3182500	84240000	0	3581600	2184000	1922000

P01889	P01889	HLA class I histocompatibility antigen, B-7 alpha cl	HLA-B	sp|P01889	3	2	2	0	2	1	2	0	1	1	2	0	1	1	2	13.3	40.46	0	13.689	110970000	0	11254000	1802300	97917000	0	1665900	1471900	3092300
Q9NW82	Q9NW82	WD repeat-containing protein 70	WDR70	sp|Q9NW8	8	8	8	0	7	1	4	0	7	1	4	0	7	1	4	16.8	73.2	0	17.258	110540000	0	90121000	492120	19925000	0	8016600	0	746830
Q15392	Q15392	Delta(24)-sterol reductase	DHCR24	sp|Q15392	4	4	4	0	4	1	4	0	4	1	4	0	4	1	4	6.6	60.101	0	7.4824	110280000	0	26929000	1122700	82226000	0	1888800	1367400	3253600
Q9UKL0	Q9UKL0	REST corepressor 1	RCOR1	sp|Q9UKL	5	5	4	0	5	1	2	0	5	1	2	0	4	1	2	15.3	53.327	0	11.565	109680000	0	63868000	1850400	43963000	0	3679000	2518300	2484100
O75955	O75955	Flotillin-1	FLOT1	sp|O75955	7	7	7	3	4	4	4	3	4	4	4	3	4	4	4	21.1	47.355	0	20.834	109560000	17651000	26539000	5826500	59543000	18203000	2814900	3559900	1776800
Q96K37	Q96K37	Solute carrier family 35 member E1	SLC35E1	sp|Q96K37	2	2	2	1	1	1	2	1	1	1	2	1	1	1	2	12	44.772	0	24.4	109410000	2583600	20081000	5190000	81560000	2606400	1859300	4022200	2707800
Q15334	Q15334	Lethal(2) giant larvae protein homolog 1	LLGL1	sp|Q15334	6	6	6	0	2	2	6	0	2	2	6	0	2	2	6	8.2	115.42	0	22.514	109390000	0	9852000	3506200	96034000	0	1343200	2033000	3797800
Q9Y426	Q9Y426	C2 domain-containing protein 2	C2CD2	sp|Q9Y426	7	7	7	0	0	3	7	0	0	3	7	0	0	3	7	19.4	75.532	0	28.457	109030000	0	0	4807300	1.04E+08	0	0	4693900	2822700
P61970	P61970	Nuclear transport factor 2	NUTF2	sp|P61970	2	2	2	0	1	1	2	0	1	1	2	0	1	1	2	26	14.478	0	5.8931	108670000	0	21146000	1388600	86136000	0	0	0	3542400
O15020	O15020	Spectrin beta chain, non-erythrocytic 2	SPTBN2	sp|O1502	11	7	7	1	10	3	3	1	7	1	0	1	7	1	0	7.9	271.32	0	16.47	108620000	1155500	1.02E+08	5168800	0	1624900	8547900	0	0
Q9H9E3	Q9H9E3	Conserved oligomeric Golgi complex subunit 4	COG4	sp|Q9H9E	5	5	5	0	4	4	4	0	4	4	4	0	4	4	4	8.4	89.082	0	19.084	108330000	0	30944000	10766000	66624000	0	2451400	7611500	2639500
O60637	O60637	Tetraspanin-3	TSPAN3	sp|O60637	2	2	2	0	1	0	2	0	1	0	2	0	1	0	2	8.3	28.017	0.001139	2.5535	108100000	0	16784000	0	91312000	0	3043600	0	2191200
Q6WKZ4	Q6WKZ4	Rab11 family-interacting protein 1	RAB11FIP1	sp|Q6WKZ	3	3	3	0	3	1	2	0	3	1	2	0	3	1	2	4.1	137.17	0	7.015	108000000	0	59767000	12661000	35569000	0	3407900	10453000	1378500
Q8N108	Q8N108	Mesoderm induction early response protein 1	MIER1	sp|Q8N10	2	2	2	0	2	0	1	0	2	0	1	0	2	0	1	6.2	57.983	0	5.386	107990000	0	53391000	0	54604000	0	4706300	0	0
Q9BQ70	Q9BQ70	Transcription factor 25	TCF25	sp|Q9BQ7	4	4	4	0	2	1	4	0	2	1	4	0	2	1	4	10.7	76.666	0	27.775	107690000	0	18844000	3523000	85322000	0	2623200	2411400	2502900
Q9NRZ9	Q9NRZ9	Lymphoid-specific helicase	HELLS	sp|Q9NRZ	7	7	7	0	5	4	6	0	5	4	6	0	5	4	6	9.8	97.073	0	15.106	107640000	0	37093000	5823900	64720000	0	2706100	4226800	2912100
O75127	O75127	Pentatricopeptide repeat-containing protein 1, m	PTCD1	sp|O75127	2	2	2	0	2	0	2	0	2	0	2	0	2	0	2	3.3	78.855	0	6.4494	107280000	0	36257000	0	71018000	0	3128600	0	2988100
Q9UL45	Q9UL45	Biogenesis of lysosome-related organelles comple	BLOC1S6	sp|Q9UL4	1	1	1	0	0	0	1	0	0	0	1	0	0	0	1	11	19.743	0.00135	2.3856	107090000	0	0	0	1.07E+08	0	0	0	4404200
Q9Y2I8	Q9Y2I8	WD repeat-containing protein 37	WDR37	sp|Q9Y2I8	3	3	3	0	2	3	2	0	2	3	2	0	2	3	2	8.1	54.665	0	9.3733	106850000	0	37779000	23760000	45316000	0	3938700	14711000	2511100
Q12840	Q12840	Kinesin heavy chain isoform 5A	KIF5A	sp|Q1284	10	3	3	1	7	6	10	1	0	0	3	1	0	0	3	12.3	117.38	0	9.1044	106390000	58155000	0	0	48231000	58466000	0	0	1672300
Q5JPH6	Q5JPH6	Probable glutamate--tRNA ligase, mitochondrial	EARS2	sp|Q5JPH	4	4	4	0	3	0	2	0	3	0	2	0	3	0	2	9	58.688	0	13.949	106330000	0	95746000	0	10586000	0	7034800	0	1840400
Q8N1G2	Q8N1G2	Cap-specific mRNA (nucleoside-2-O-)-methyltrans	CMTR1	sp|Q8N1G	6	6	6	0	6	0	5	0	6	0	5	0	6	0	5	6.9	95.32	0	13.309	106270000	0	63696000	0	42574000	0	5827900	0	1537700
Q9Y2J2	Q9Y2J2	Band 4.1-like protein 3;Band 4.1-like protein 3, N-	EPB41L3	sp|Q9Y2J2	10	8	8	0	10	1	0	0	8	0	0	0	8	0	0	17.8	120.68	0	16.669	106110000	0	1.06E+08	0	0	0	9353300	0	0
Q92844	Q92844	TRAF family member-associated NF-kappa-B activ	TANK	sp|Q9284	9	9	9	2	1	8	2	2	1	8	2	2	1	8	2	30.4	47.815	0	21.633	105820000	5180600	1668700	85775000	13194000	825430	139090	62190000	356350
Q9HBK9	Q9HBK9	Arsenite methyltransferase	AS3MT	sp|Q9HBK	4	4	4	0	4	0	3	0	4	0	3	0	4	0	3	11.7	41.747	0	11.159	105570000	0	57472000	0	48097000	0	4610200	0	2433900
P03891	P03891	NADH-ubiquinone oxidoreductase chain 2	MT-ND2	sp|P03891	1	1	1	0	1	1	1	0	1	1	1	0	1	1	1	2.6	38.96	0.001565	2.2953	105490000	0	29404000	1336400	74747000	0	2591900	898030	3074000
P82932	P82932	28S ribosomal protein S6, mitochondrial	MRPS6	sp|P82932	3	3	3	1	2	3	3	1	2	3	3	1	2	3	3	24	14.226	0	7.817	105440000	321600	51013000	7948600	46155000	1181500	6421100	2285300	2170000
Q7Z3E5	Q7Z3E5	LisH domain-containing protein ARMC9	ARMC9	sp|Q7Z3E	5	5	5	0	1	0	5	0	1	0	5	0	1	0	5	7.1	91.818	0	13.008	105040000	0	3585400	0	1.01E+08	0	316050	0	4172200
P56945	P56945	Breast cancer anti-estrogen resistance protein 1	BCAR1	sp|P56945	5	5	5	0	0	1	5	0	0	1	5	0	0	1	5	12	93.371	0	14.575	104870000	0	0	4667200	1E+08	0	0	3136300	4120700
P11388	P11388	DNA topoisomerase 2-alpha	TOP2A	sp|P11388	6	6	2	1	5	3	3	1	5	3	3	0	2	2	1	4.9	174.38	0	14.565	104730000	346330	43265000	10379000	50739000	0	2909700	8549200	1416400
P13591	P13591	Neural cell adhesion molecule 1	NCAM1	sp|P13591	4	4	4	0	4	0	3	0	4	0	3	0	4	0	3	8.2	94.573	0	28.018	104670000	0	63652000	0	41018000	0	5445900	0	1851900
Q9Y2U8	Q9Y2U8	Inner nuclear membrane protein Man1	LEMD3	sp|Q9Y2U	5	5	5	0	5	1	2	0	5	1	2	0	5	1	2	12.3	99.996	0	17.116	104580000	0	60247000	2242900	42093000	0	4460300	1908300	2180400
Q8NBN3	Q8NBN3	Transmembrane protein 87A	TMEM87A	sp|Q8NBN	1	1	1	0	1	0	1	0	1	0	1	0	1	0	1	3.8	63.429	0	10.425	104480000	0	17661000	0	86818000	0	1556800	0	3570500
P0CW20;P	P0CW20;P	LIM and senescent cell antigen-like-containing do	LIMS3L;LIM	sp|P0CW2	2	2	2	0	0	1	2	0	0	1	2	0	0	1	2	25.6	13.251	0	10.662	104330000	0	0	2286200	1.02E+08	0	0	1951800	2793100
P78316	P78316	Nucleolar protein 14	NOP14	sp|P78316	6	6	6	0	6	4	4	0	6	4	4	0	6	4	4	8.9	97.667	0	12.316	104310000	0	49217000	12878000	42214000	0	3423200	9344500	1960400
Q9H7D0	Q9H7D0	Dedicator of cytokinesis protein 5	DOCK5	sp|Q9H7D	4	4	4	0	0	1	4	0	0	1	4	0	0	1	4	3.5	215.31	0	9.0077	104290000	0	0	1266000	1.03E+08	0	0	2677700	2410100
Q13885	Q13885	Tubulin beta-2A chain	TUBB2A	sp|Q1388	23	1	1	11	23	20	23	0	1	1	1	0	1	1	1	61.8	49.906	0.000249	3.7546	104290000	0	56549000	2654100	45091000	0	4984800	1783500	1854400
Q6UW63	Q6UW63	KDEL motif-containing protein 1	KDELC1	sp|Q6UW	4	4	4	0	1	0	4	0	1	0	4	0	1	0	4	10.6	58.042	0	9.463	104270000	0	15019000	0	89248000	0	2050300	0	2944100
O95208	O95208	Epsin-2	EPN2	sp|O9520	6	6	6	0	3	0	6	0	3	0	6	0	3	0	6	9.4	68.481	0	14.933	104120000	0	12089000	0	92036000	0	1793600	0	3057000
Q5VTB9	Q5VTB9	E3 ubiquitin-protein ligase RNF220	RNF220	sp|Q5VTB	3	3	3	0	3	2	2	0	3	2	2	0	3	2	2	5.8	62.765	0	5.45	103990000	0	62132000	2219900	39635000	0	4027400	1992700	2578600
Q9BUR5	Q9BUR5	Apolipoprotein O	APOO	sp|Q9BUR	2	2	2	0	2	0	2	0	2	0	2	0	2	0	2	11.1	22.284	0	5.874	103650000	0	54845000	0	48810000	0	4691200	0	2150700
P16383	P16383	GC-rich sequence DNA-binding factor 2	GCFC2	sp|P16383	3	3	3	0	2	0	3	0	2	0	3	0	2	0	3	4.5	89.384	0.001176	3.0558	103570000	0	46477000	0	57092000	0	4483300	0	1961500
Q13445	Q13445	Transmembrane emp24 domain-containing prote	TMED1	sp|Q1344	4	4	4	1	4	0	3	1	4	0	3	1	4	0	3	20.7	25.206	0	16.2	103560000	236970	53034000	0	50288000	517190	4058500	0	2404300
P51580	P51580	Thiopurine S-methyltransferase	TPMT	sp|P51580	4	4	4	0	4	0	4	0	4	0	4	0	4	0	4	17.1	28.18	0	12.375	103450000	0	41597000	0	61856000	0	3749000	0	2461500
Q9BSR8	Q9BSR8	Protein YIPF4	YIPF4	sp|Q9BSR	2	2	2	0	1	2	2	0	1	2	2	0	1	2	2	7.4	27.082	0.001131	2.4517	103420000	0	16408000	3812500	83204000	0	1898000	2248000	3284200
A0A0C4DH	A0A0C4DH67;A0A0C4DH69;A0A075B6S5		IGKV1-8;IG	sp|A0A0C	1	1	1	0	0	1	0	0	0	1	0	0	0	1	0	13.9	12.537	0.000723	3.3592	103400000	0	0	103400000	0	0	0	69485000	0
Q9NYV4	Q9NYV4	Cyclin-dependent kinase 12	CDK12	sp|Q9NYV	4	3	3	0	4	2	4	0	3	1	3	0	3	1	3	4.9	164.15	0	5.1078	103350000	0	26921000	1363300	75070000	0	1992100	2251900	2132500
Q00403	Q00403	Transcription initiation factor IIB	GTF2B	sp|Q0040	4	4	4	0	4	0	2	0	4	0	2	0	4	0	2	22.2	34.833	0	18.546	103330000	0	81872000	0	21457000	0	6215400	0	1883900
O75600	O75600	2-amino-3-ketobutyrate coenzyme A ligase, mitoc	GCAT	sp|O7560	5	5	5	0	5	0	2	0	5	0	2	0	5	0	2	19.1	45.285	0	17.558	103300000	0	87996000	0	15304000	0	7108200	0	1277900
P50897	P50897	Palmitoyl-protein thioesterase 1	PPT1	sp|P50897	2	2	2	0	1	0	2	0	1	0	2	0	1	0	2	8.2	34.193	0	5.3204	103200000	0	23278000	0	79922000	0	2144200	0	3194600
O43663	O43663	Protein regulator of cytokinesis 1	PRC1	sp|O4366	3	3	3	0	2	2	2	0	2	2	2	0	2	2	2	5.8	71.606	0	8.7579	103160000	0	60642000	5008500	37510000	0	0	3397900	1510400
O75381	O75381	Peroxisomal membrane protein PEX14	PEX14	sp|O75381	4	4	4	1	4	1	3	1	4	1	3	1	4	1	3	18.8	41.236	0	7.4374	103090000	1407500	42239000	6819700	52626000	3189600	2232600	4728900	1726800
Q9UPN9	Q9UPN9	E3 ubiquitin-protein ligase TRIM33	TRIM33	sp|Q9UPN	6	6	6	0	6	0	5	0	6	0	5	0	6	0	5	6.5	122.53	0	17.354	102970000	0	45900000	0	57074000	0	3729300	0	2663900
P62328	P62328	Thymosin beta-4;Hematopoietic system regulator	TMSB4X	sp|P62328	2	2	2	0	0	0	2	0	0	0	2	0	0	0	2	31.8	5.0526	0	5.7052	102870000	0	0	0	1.03E+08	0	0	0	4230500
O00458	O00458	Interferon-related developmental regulator 1	IFRD1	sp|O0045	3	3	3	0	3	2	3	0	3	2	3	0	3	2	3	11.1	50.268	0	14.95	102870000	0	47049000	3946100	51878000	0	2799900	4437300	1695300
Q08722	Q08722	Leukocyte surface antigen CD47	CD47	sp|Q0872	2	2	2	0	2	0	1	0	2	0	1	0	2	0	1	5.9	35.213	0.000488	3.5133	102830000	0	34727000	0	68102000	0	2324700	0	3537100
P46736	P46736	Lys-63-specific deubiquitinase BRCC36	BRCC3	sp|P46736	3	3	3	0	2	2	2	0	2	2	2	0	2	2	2	19	36.072	0	11.272	102790000	0	66291000	1810500	34693000	0	3509500	2288900	2688500
P54725	P54725	UV excision repair protein RAD23 homolog A	RAD23A	sp|P54725	5	4	4	0	4	1	4	0	3	0	3	0	3	0	3	17.6	39.609	0	7.2238	102560000	0	31027000	0	71535000	0	2715100	0	2961800
Q9H7F0	Q9H7F0	Probable cation-transporting ATPase 13A3	ATP13A3	sp|Q9H7F	2	2	2	0	0	0	2	0	0	0	2	0	0	0	2	3	138.04	0	11.871	102450000	0	0	0	1.02E+08	0	0	0	4213400
P51784	P51784	Ubiquitin carboxyl-terminal hydrolase 11	USP11	sp|P51784	5	5	5	0	5	0	3	0	5	0	3	0	5	0	3	5.8	109.82	0	11.334	102450000	0	72950000	0	29503000	0	5438900	0	2204900
O43347	O43347	RNA-binding protein Musashi homolog 1	MSI1	sp|O43347	4	4	4	0	4	0	0	0	4	0	0	0	4	0	0	15.5	39.124	0	13.253	102010000	0	1.02E+08	0	0	0	8992400	0	0
Q8WY22	Q8WY22	BRI3-binding protein	BRI3BP	sp|Q8WY2	2	2	2	0	2	1	2	0	2	1	2	0	2	1	2	14.3	27.835	0	9.9142	101980000	0	62821000	1862600	37294000	0	4553300	2336300	1433400
Q9NV88	Q9NV88	Integrator complex subunit 9	INTS9	sp|Q9NV8	3	3	3	1	0	0	3	1	0	0	3	1	0	0	3	6.8	73.814	0	7.9983	101840000	3288200	0	0	98551000	5617000	0	0	1724200
Q6UX53	Q6UX53	Methyltransferase-like protein 7B	METTL7B	sp|Q6UX5	3	3	3	0	0	1	3	0	0	1	3	0	0	1	3	15.6	27.775	0	9.3419	101710000	0	0	695700	1.01E+08	0	0	1320100	3301700
Q6A1A2;O	Q6A1A2;O	Putative 3-phosphoinositide-dependent protein k	PDPK2P;PD	sp|Q6A1A	3	3	3	0	2	0	2	0	2	0	2	0	2	0	2	11.6	44.765	0	10.862	101660000	0	23608000	0	78051000	0	1809900	0	3481100
Q15554	Q15554	Telomeric repeat-binding factor 2	TERF2	sp|Q1555	5	5	5	1	5	1	4	1	5	1	4	1	5	1	4	17	59.593	0	12.465	101400000	568600	57720000	1858700	41250000	1264800	3786300	2283900	1267000
Q9BW60	Q9BW60	Elongation of very long chain fatty acids protein 1	ELOVL1	sp|Q9BW6	2	2	2	0	1	1	2	0	1	1	2	0	1	1	2	9	32.662	0	5.6176	101320000	0	11670000	7247500	82399000	0	1057800	4619300	1271500
Q9NX62	Q9NX62	Inositol monophosphatase 3	IMPAD1	sp|Q9NX6	2	2	2	0	1	0	2	0	1	0	2	0	1	0	2	7.2	38.681	0	6.292	101280000	0	11838000	0	89446000	0	1193600	0	3528400
Q6P3W7	Q6P3W7	SCY1-like protein 2	SCYL2	sp|Q6P3W	3	3	3	0	3	0	1	0	3	0	1	0	3	0	1	5.5	103.71	0.000719	3.3043	101250000	0	69756000	0	31489000	0	5146600	0	2297400
Q96FZ7	Q96FZ7	Charged multivesicular body protein 6	CHMP6	sp|Q96FZ7	3	3	3	0	3	0	3	0	3	0	3	0	3	0	3	23.9	23.485	0	12.414	101190000	0	42615000	0	58570000	0	3568200	0	2597000
Q9UBU9	Q9UBU9	Nuclear RNA export factor 1	NXF1	sp|Q9UBU	7	7	7	0	1	2	7	0	1	2	7	0	1	2	7	12.8	70.182	0	13.995	101180000	0	5221400	4487500	91468000	0	1245400	3820900	2171200
Q8N9F7	Q8N9F7	Glycerophosphodiester phosphodiesterase doma	GDPD1	sp|Q8N9F	2	2	2	0	2	0	0	0	2	0	0	0	2	0	0	11.1	36.167	0	9.4195	101090000	0	1.01E+08	0	0	0	8910900	0	0
P17480	P17480	Nucleolar transcription factor 1	UBTF	sp|P17480	7	7	7	2	6	4	2	2	6	4	2	2	6	4	2	11	89.405	0	13.995	100980000	6864400	77815000	6445800	9851300	7073500	7698700	2156700	1531400
O15446	O15446	DNA-directed RNA polymerase I subunit RPA34	CD3EAP	sp|O1544	3	3	3	0	3	1	2	0	3	1	2	0	3	1	2	13.9	54.985	0	16.969	100940000	0	41852000	9527500	49556000	0	2860900	7251200	2017500
O60934	O60934	Nibrin	NBN	sp|O6093	5	5	5	0	4	0	5	0	4	0	5	0	4	0	5	12.2	84.958	0	8.2277	100910000	0	42659000	0	58253000	0	3898100	0	2258000
P51965	P51965	Ubiquitin-conjugating enzyme E2 E1	UBE2E1	sp|P51965	3	3	2	0	2	0	2	0	2	0	2	0	1	0	1	25.4	21.404	0	7.4073	100710000	0	38540000	0	62173000	0	3128300	0	2825900
Q14627	Q14627	Interleukin-13 receptor subunit alpha-2	IL13RA2	sp|Q1462	1	1	1	0	0	0	1	0	0	0	1	0	0	0	1	2.9	44.176	0.00135	2.3885	100640000	0	0	0	1.01E+08	0	0	0	4138800
P51649	P51649	Succinate-semialdehyde dehydrogenase, mitocho	ALDH5A1	sp|P51649	5	5	5	0	5	0	1	0	5	0	1	0	5	0	1	9.5	57.214	0	10.228	100630000	0	82492000	0	18137000	0	7271600	0	0
Q92643	Q92643	GPI-anchor transamidase	PIGK	sp|Q9264	3	3	3	1	2	3	3	1	2	3	3	1	2	3	3	14.7	45.251	0	14.148	100510000	3323000	21270000	6742500	69172000	4595200	1835900	3261900	2880600
Q96I51	Q96I51	Williams-Beuren syndrome chromosomal region	WBSCR16	sp|Q96I51	3	3	3	0	2	0	3	0	2	0	3	0	2	0	3	15.3	49.996	0	18.487	100310000	0	18020000	0	82293000	0	2525700	0	2447100
Q8TB03	Q8TB03	Uncharacterized protein CXorf38	CXorf38	sp|Q8TB0	3	3	3	0	3	1	2	0	3	1	2	0	3	1	2	12.2	36.67	0	9.8734	99978000	0	42239000	2800700	54939000	0	3189900	2625400	2049400
Q7L4I2	Q7L4I2	Arginine/serine-rich coiled-coil protein 2	RSRC2	sp|Q7L4I2	4	4	4	0	4	3	2	0	4	3	2	0	4	3	2	12.7	50.559	0	18.711	99955000	0	50721000	21839000	27395000	0	7503100	8574300	4196300
Q10469	Q10469	Alpha-1,6-mannosyl-glycoprotein 2-beta-N-acetyl	MGAT2	sp|Q1046	3	3	3	0	3	1	3	0	3	1	3	0	3	1	3	7.6	51.55	0	8.1352	99790000	0	25875000	565210	73350000	0	1862300	1292800	2522100
Q15746	Q15746	Myosin light chain kinase, smooth muscle;Myosin	MYLK	sp|Q1574	2	2	2	0	2	2	1	0	2	2	1	0	2	2	1	1.1	210.71	0.000715	3.2582	99616000	0	42446000	5493800	51676000	0	3384600	3034300	2903800
P09001	P09001	39S ribosomal protein L3, mitochondrial	MRPL3	sp|P09001	2	2	2	0	2	0	1	0	2	0	1	0	2	0	1	7.8	38.632	0	5.1129	99514000	0	74899000	0	24615000	0	3190500	0	4424000
O96013	O96013	Serine/threonine-protein kinase PAK 4	PAK4	sp|O9601	3	3	3	0	3	0	3	0	3	0	3	0	3	0	3	10	64.071	0	15.915	99374000	0	22407000	0	76968000	0	1916600	0	2548500
O14618	O14618	Copper chaperone for superoxide dismutase	CCS	sp|O1461	3	3	3	0	3	0	3	0	3	0	3	0	3	0	3	10.2	29.04	0	6.9077	99228000	0	44120000	0	55107000	0	3793300	0	2362200
Q8WVF5	Q8WVF5	BTB/POZ domain-containing protein KCTD4	KCTD4	sp|Q8WVF	3	3	3	0	1	2	3	0	1	2	3	0	1	2	3	14.3	29.967	0	5.6048	99151000	0	1103300	8587600	89460000	0	238070	8395100	914070
Q96H20	Q96H20	Vacuolar-sorting protein SNF8	SNF8	sp|Q96H2	2	2	2	0	2	0	2	0	2	0	2	0	2	0	2	10.1	28.864	0	11.258	99069000	0	55279000	0	43789000	0	4872300	0	1801400

Q9Y3B2	Q9Y3B2	Exosome complex component CSL4	EXOSC1	sp|Q9Y3B2	3	3	3	0	3	3	3	0	3	3	3	0	3	3	3	18.5	21.452	0	5.5852	98952000	0	34388000	9137800	55426000	0	3055900	6131500	2263800
P61803	P61803	Dolichyl-diphosphooligosaccharide--protein glyco	DAD1	sp|P61803	2	2	2	0	0	0	2	0	0	0	2	0	0	0	2	15.9	12.497	0.001564	2.2816	98915000	0	0	0	98915000	0	0	0	4067900
P49790	P49790	Nuclear pore complex protein Nup153	NUP153	sp|P49790	5	5	5	0	5	3	4	0	5	3	4	0	5	3	4	4.9	153.94	0	17.172	98715000	0	47977000	3931000	46807000	0	3568000	4134600	1093100
Q7L5Y1	Q7L5Y1	Mitochondrial enolase superfamily member 1	ENOSF1	sp|Q7L5Y1	1	1	1	0	1	0	1	0	1	0	1	0	1	0	1	3.8	49.786	0	7.9043	98633000	0	27658000	0	70975000	0	2438000	0	2918900
Q8TAF3	Q8TAF3	WD repeat-containing protein 48	WDR48	sp|Q8TAF	4	4	4	0	4	1	3	0	4	1	3	0	4	1	3	10.5	76.21	0	6.3021	98615000	0	52771000	1417300	44426000	0	3967400	1547300	1916500
O95167	O95167	NADH dehydrogenase [ubiquinone] 1 alpha subcc	NDUFA3	sp|O95167	2	2	2	0	1	0	2	0	1	0	2	0	1	0	2	41.7	9.2787	0.001566	2.3048	98381000	0	27058000	0	71323000	0	2771500	0	2546900
Q9Y2R0	Q9Y2R0	Cytochrome c oxidase assembly factor 3 homolog	COA3	sp|Q9Y2R	3	3	3	0	3	0	3	0	3	0	3	0	3	0	3	30.2	11.731	0	9.6127	98363000	0	57719000	0	40644000	0	4993800	0	1765600
Q6UX04	Q6UX04	Peptidyl-prolyl cis-trans isomerase CWC27 homol	CWC27	sp|Q6UX0	1	1	1	0	1	0	1	0	1	0	1	0	1	0	1	7.2	53.846	0	11.916	98295000	0	24753000	0	73541000	0	2182000	0	3024400
Q86YN1	Q86YN1	Dolichyldiphosphatase 1	DOLPP1	sp|Q86YN	3	3	3	0	3	0	2	0	3	0	2	0	3	0	2	15.5	27.03	0	11.275	98295000	0	44947000	0	53348000	0	4074000	0	2082000
Q9BVA0	Q9BVA0	Katanin p80 WD40 repeat-containing subunit B1	KATNB1	sp|Q9BVA	5	5	5	0	5	0	2	0	5	0	2	0	5	0	2	14.5	72.333	0	9.7607	98145000	0	49765000	0	48380000	0	3878600	0	2497800
Q969U7	Q969U7	Proteasome assembly chaperone 2	PSMG2	sp|Q969U	3	3	3	0	3	0	3	0	3	0	3	0	3	0	3	10.6	29.396	0	7.1504	97794000	0	29245000	0	68549000	0	2551300	0	2845800
Q9BTX1	Q9BTX1	Nucleoporin NDC1	NDC1	sp|Q9BTX	4	4	4	0	1	0	4	0	1	0	4	0	1	0	4	9.2	76.304	0	16.426	97763000	0	3839100	0	93924000	0	575200	0	3625900
Q07864	Q07864	DNA polymerase epsilon catalytic subunit A	POLE	sp|Q07864	4	4	4	0	3	0	3	0	3	0	3	0	3	0	3	2.9	261.51	0	9.8734	97614000	0	46387000	0	51227000	0	2301300	0	3894400
Q9H788	Q9H788	SH2 domain-containing protein 4A	SH2D4A	sp|Q9H78	2	2	2	0	1	0	2	0	1	0	2	0	1	0	2	6.8	52.726	0.001172	2.9921	97596000	0	16040000	0	81556000	0	0	0	3354000
P09496	P09496	Clathrin light chain A	CLTA	sp|P09496	2	2	2	0	1	2	2	0	1	2	2	0	1	2	2	6.9	27.076	0	4.5578	97553000	0	13894000	16786000	66873000	0	1857200	10516000	2882100
Q96CV9	Q96CV9	Optineurin	OPTN	sp|Q96CV	3	3	3	0	1	0	3	0	1	0	3	0	1	0	3	6.4	65.921	0	4.2729	97525000	0	11292000	0	86233000	0	0	0	3546400
Q03252	Q03252	Lamin-B2	LMNB2	sp|Q03252	6	5	5	0	6	1	5	0	5	0	4	0	5	0	4	10.2	69.948	0	11.781	97463000	0	42539000	0	54923000	0	3789500	0	2219100
O75879	O75879	Glutamyl-tRNA(Gln) amidotransferase subunit B,	GATB	sp|O7587	4	4	4	0	3	0	4	0	3	0	4	0	3	0	4	15.3	61.863	0	30	97462000	0	28586000	0	68876000	0	2900800	0	2451600
Q8TB52	Q8TB52	F-box only protein 30	FBXO30	sp|Q8TB5	3	3	3	0	2	0	3	0	2	0	3	0	2	0	3	4.4	82.303	0	8.3992	97386000	0	7190500	0	90195000	0	1239300	0	3103900
Q8IZ07;Q6	Q8IZ07	Ankyrin repeat domain-containing protein 13A	ANKRD13A	sp|Q8IZ07	4	4	4	0	1	1	4	0	1	1	4	0	1	1	4	7.8	67.618	0	9.9823	97311000	0	10028000	940650	86343000	0	1095200	1543300	2428400
Q8WXE9	Q8WXE9	Stonin-2	STON2	sp|Q8WXE	3	3	3	0	0	0	3	0	0	0	3	0	0	0	3	5	101.16	0	15.714	97258000	0	0	0	97258000	0	0	0	3999800
Q7RTP6	Q7RTP6	Protein-methionine sulfoxide oxidase MICAL3	MICAL3	sp|Q7RTP	2	2	2	0	1	1	2	0	1	1	2	0	1	1	2	1.4	224.29	0	5.8645	96987000	0	23626000	11120000	62241000	0	2191500	7862900	2060500
O00423	O00423	Echinoderm microtubule-associated protein-like	EML1	sp|O0042	3	3	3	0	3	0	3	0	3	0	3	0	3	0	3	6.3	89.86	0	18.811	96816000	0	19480000	0	77335000	0	1658200	0	3239500
Q9UFW8	Q9UFW8	CGG triplet repeat-binding protein 1	CGGBP1	sp|Q9UFW	3	3	3	0	3	2	3	0	3	2	3	0	3	2	3	19.2	18.82	0	5.4181	96795000	0	37073000	1817300	57904000	0	3694900	1461700	1713900
O95260	O95260	Arginyl-tRNA--protein transferase 1	ATE1	sp|O9526	2	2	2	0	1	0	2	0	1	0	2	0	1	0	2	6.8	59.09	0.001175	3.0456	96780000	0	20832000	0	75948000	0	0	0	3123400
P48681	P48681	Nestin	NES	sp|P48681	4	4	4	1	1	2	4	1	1	2	4	1	1	2	4	2.5	177.44	0	6.9068	96651000	355510	7969200	6783800	81543000	1477700	969390	3843100	2679900
O75494	O75494	Serine/arginine-rich splicing factor 10	SRSF10	sp|O75494	2	2	2	0	2	1	1	0	2	1	1	0	2	1	1	8.8	31.3	0	6.1475	96612000	0	39197000	3365700	54049000	0	3455200	2261800	2222800
Q9Y6D6	Q9Y6D6	Brefeldin A-inhibited guanine nucleotide-exchang	ARFGEF1	sp|Q9Y6D	10	6	6	0	5	2	10	0	3	1	6	0	3	1	6	7.4	208.76	0	10.994	96572000	0	9958100	879610	85735000	0	1047200	707530	3240100
Q5TBB1	Q5TBB1	Ribonuclease H2 subunit B	RNASEH2B	sp|Q5TBB	3	3	3	0	3	0	3	0	3	0	3	0	3	0	3	12.8	35.138	0	25.224	96560000	0	58079000	0	38482000	0	5117600	0	1584500
Q9H0P0	Q9H0P0	Cytosolic 5-nucleotidase 3A	NT5C3A	sp|Q9H0P	4	4	4	0	4	0	3	0	4	0	3	0	4	0	3	18.8	37.948	0	35.351	96496000	0	33822000	0	62674000	0	3208500	0	2350300
Q8NCG7	Q8NCG7	Sn1-specific diacylglycerol lipase beta	DAGLB	sp|Q8NCG	3	3	3	0	3	0	3	0	3	0	3	0	3	0	3	6.4	73.731	0	10.277	96476000	0	26111000	0	70365000	0	2301200	0	2894200
A1A4S6	A1A4S6	Rho GTPase-activating protein 10	ARHGAP1C	sp|A1A4S	2	2	2	0	0	0	2	0	0	0	2	0	0	0	2	3.1	89.374	0	5.9881	96470000	0	0	0	96470000	0	0	0	3967400
P13716	P13716	Delta-aminolevulinic acid dehydratase	ALAD	sp|P13716	3	3	3	0	2	1	3	0	2	1	3	0	2	1	3	17.9	36.294	0	10.691	96300000	0	18730000	2430900	75139000	0	1794500	2428100	2152100
Q13523	Q13523	Serine/threonine-protein kinase PRP4 homolog	PRPF4B	sp|Q13523	5	5	5	0	4	1	3	0	4	1	3	0	4	1	3	7.6	116.99	0	26.104	96229000	0	46126000	2515400	47588000	0	2805600	2766000	2141700
Q9NYA1	Q9NYA1	Sphingosine kinase 1	SPHK1	sp|Q9NYA	4	4	4	0	1	1	4	0	1	1	4	0	1	1	4	8.1	42.517	0	6.9207	96197000	0	1754600	383120	94059000	0	371870	318210	3590300
Q92542	Q92542	Nicastrin	NCSTN	sp|Q92542	2	2	2	0	2	1	2	0	2	1	2	0	2	1	2	2.7	78.41	0.000714	3.2307	96031000	0	29189000	1519100	65323000	0	2051400	1784200	2444700
Q9BYN8	Q9BYN8	28S ribosomal protein S26, mitochondrial	MRPS26	sp|Q9BYN	4	4	4	1	4	1	1	1	4	1	1	1	4	1	1	23.9	24.211	0	8.5214	96019000	1750400	69827000	365840	24076000	1746700	5839500	654600	900830
Q7L592	Q7L592	NADH dehydrogenase [ubiquinone] complex I, as	NDUFAF7	sp|Q7L592	4	4	4	0	4	0	3	0	4	0	3	0	4	0	3	12.5	49.237	0	15.082	95927000	0	62763000	0	33163000	0	5532500	0	0
Q15628	Q15628	Tumor necrosis factor receptor type 1-associated	TRADD	sp|Q1562	3	3	3	0	2	0	3	0	2	0	3	0	2	0	3	12.5	34.247	0	11.849	95900000	0	4714000	0	91186000	0	1157700	0	3007900
Q96BP3	Q96BP3	Peptidylprolyl isomerase domain and WD repeat-	PPWD1	sp|Q96BP	5	5	5	0	4	0	3	0	4	0	3	0	4	0	3	9.8	73.574	0	12.604	95866000	0	52330000	0	43536000	0	3836400	0	2566900
Q9UDX4;O	Q9UDX4;O	SEC14-like protein 3;SEC14-like protein 2	SEC14L3;SI	sp|Q9UDX	1	1	1	0	0	0	1	0	0	0	1	0	0	0	1	5.5	46.048	0	7.6888	95841000	0	0	0	95841000	0	0	0	3941500
Q969N2	Q969N2	GPI transamidase component PIG-T	PIGT	sp|Q969N	2	2	2	1	0	1	2	1	0	1	2	1	0	1	2	5	65.699	0	5.1711	95806000	901230	0	717970	94186000	0	0	0	3873500
Q9BVM2	Q9BVM2	Protein DPCD	DPCD	sp|Q9BVM	4	4	4	0	4	0	2	0	4	0	2	0	4	0	2	17.2	23.239	0	6.0122	95688000	0	41093000	0	54594000	0	3237900	0	2629700
Q96EY5	Q96EY5	Multivesicular body subunit 12A	MVB12A	sp|Q96EY	2	2	2	0	2	0	1	0	2	0	1	0	2	0	1	15	28.783	0	6.9372	95628000	0	24510000	0	71118000	0	1676600	0	3408700
P08134;P6	P08134	Rho-related GTP-binding protein RhoC	RHOC	sp|P08134	5	2	2	0	4	2	5	0	1	1	2	0	1	1	2	36.3	22.006	0.0037	1.9648	95490000	0	5868400	249070	89373000	0	299620	170430	3890100
A1X283	A1X283	SH3 and PX domain-containing protein 2B	SH3PXD2B	sp|A1X283	3	3	3	0	2	0	3	0	2	0	3	0	2	0	3	6.4	101.58	0	9.7033	95420000	0	9402200	0	86018000	0	1212600	0	3153800
Q96ER9	Q96ER9	Coiled-coil domain-containing protein 51	CCDC51	sp|Q96ER	6	6	6	0	5	1	6	0	5	1	6	0	5	1	6	18	45.811	0	30.645	95265000	0	52196000	2448500	40620000	0	2849600	3863300	1204100
O15056	O15056	Synaptojanin-2	SYNJ2	sp|O1505	6	6	6	0	0	0	6	0	0	0	6	0	0	0	6	8.3	165.54	0	19.831	95262000	0	0	0	95262000	0	0	0	3917700
Q86WB0	Q86WB0	Nuclear-interacting partner of ALK	ZC3HC1	sp|Q86WE	4	4	4	0	3	0	3	0	3	0	3	0	3	0	3	14.3	55.261	0	15.267	95220000	0	25256000	0	69964000	0	2223900	0	2879800
Q13356	Q13356	Peptidyl-prolyl cis-trans isomerase-like 2	PPIL2	sp|Q1335	2	2	2	0	1	1	2	0	1	1	2	0	1	1	2	5.6	58.823	0	4.0937	95187000	0	35171000	803920	59212000	0	2593500	1739300	1742900
P52758	P52758	Ribonuclease UK114	HRSP12	sp|P52758	3	3	3	0	2	0	3	0	2	0	3	0	2	0	3	31.4	14.494	0	28.074	95169000	0	20785000	0	74384000	0	3246400	0	1644800
Q9Y3C8	Q9Y3C8	Ubiquitin-fold modifier-conjugating enzyme 1	UFC1	sp|Q9Y3C	2	2	2	0	2	0	2	0	2	0	2	0	2	0	2	11.4	19.458	0	4.257	95168000	0	29924000	0	65244000	0	3183700	0	2137200
Q8IWV7	Q8IWV7	E3 ubiquitin-protein ligase UBR1	UBR1	sp|Q8IWV	3	3	3	0	1	0	2	0	1	0	2	0	1	0	2	2.5	200.21	0	4.0288	95021000	0	79876000	0	15145000	0	0	0	622870
Q86X76	Q86X76	Nitrilase homolog 1	NIT1	sp|Q86X7	2	2	2	0	2	0	2	0	2	0	2	0	2	0	2	8.6	35.896	0	7.5991	95016000	0	29749000	0	65267000	0	2608200	0	2395900
Q99447	Q99447	Ethanolamine-phosphate cytidylyltransferase	PCYT2	sp|Q99447	4	4	4	0	2	0	3	0	2	0	3	0	2	0	3	18	43.835	0	22.128	94826000	0	17364000	0	77462000	0	0	0	3185700
Q96BJ3	Q96BJ3	Axin interactor, dorsalization-associated protein	AIDA	sp|Q96BJ3	3	3	3	0	3	0	2	0	3	0	2	0	3	0	2	19.3	35.023	0	6.5094	94780000	0	34119000	0	60661000	0	2355900	0	3146400
Q8NB37	Q8NB37	Parkinson disease 7 domain-containing protein 1	PDDC1	sp|Q8NB3	2	2	2	0	2	0	2	0	2	0	2	0	2	0	2	10.5	23.297	0	6.1949	94425000	0	31909000	0	62516000	0	2911300	0	2472500
Q7Z6B7;O	Q7Z6B7	SLIT-ROBO Rho GTPase-activating protein 1	SRGAP1	sp|Q7Z6B	7	6	6	0	1	7	4	0	1	6	3	0	1	6	3	10.1	124.26	0	14.442	94381000	0	7435900	31358000	55587000	0	705120	21490000	1819000
O95714;Q	O95714	E3 ubiquitin-protein ligase HERC2	HERC2	sp|O9571	7	7	7	2	1	2	6	2	1	2	6	2	1	2	6	2.3	527.22	0	12.738	94183000	31198000	7990800	1417800	53576000	23124000	6229400	3624900	2080900
Q13535	Q13535	Serine/threonine-protein kinase ATR	ATR	sp|Q1353	1	1	1	0	1	1	1	0	1	1	1	0	1	1	1	0.7	301.36	0	7.529	93981000	0	20589000	621650	72770000	0	1186400	569190	3469700
Q9NQT4	Q9NQT4	Exosome complex component RRP46	EXOSC5	sp|Q9NQT	1	1	1	0	1	1	1	0	1	1	1	0	1	1	1	5.1	25.249	0.000485	3.4526	93830000	0	37955000	6495100	49380000	0	3507200	3706000	2527900
Q53HC9	Q53HC9	Protein TSSC1	TSSC1	sp|Q53HC	4	4	4	0	2	0	4	0	2	0	4	0	2	0	4	19.1	43.603	0	7.5963	93741000	0	15817000	0	77924000	0	2227900	0	2371000
Q9BYC9	Q9BYC9	39S ribosomal protein L20, mitochondrial	MRPL20	sp|Q9BYC	3	3	3	0	2	2	3	0	2	2	3	0	2	2	3	21.5	17.442	0	5.6518	93487000	0	33539000	3850300	56098000	0	0	2645600	2248900
Q8ND24	Q8ND24	RING finger protein 214	RNF214	sp|Q8ND2	4	4	4	0	4	1	2	0	4	1	2	0	4	1	2	8.5	77.667	0	12.308	93484000	0	71551000	687620	21245000	0	5782200	880220	980560
Q9H9C1	Q9H9C1	Spermatogenesis-defective protein 39 homolog	VIPAS39	sp|Q9H9C	4	4	4	0	1	0	4	0	1	0	4	0	1	0	4	11.8	57.005	0	11.051	93410000	0	14365000	0	79045000	0	2685900	0	1831100
P00414	P00414	Cytochrome c oxidase subunit 3	MT-CO3	sp|P00414	1	1	1	0	1	0	1	0	1	0	1	0	1	0	1	5.4	29.95	0	5.0647	93362000	0	32930000	0	60432000	0	2934000	0	2454100
O75884	O75884	Putative hydrolase RBBP9	RBBP9	sp|O7588	3	3	3	0	1	0	3	0	1	0	3	0	1	0	3	23.1	21	0	6.1594	93254000	0	11928000	0	81326000	0	2661500	0	1734600
Q8IYS1	Q8IYS1	Peptidase M20 domain-containing protein 2	PM20D2	sp|Q8IYS1	3	3	3	0	3	0	1	0	3	0	1	0	3	0	1	10.8	47.776	0	14.167	93235000	0	70648000	0	22588000	0	5788200	0	1368200
O00411	O00411	DNA-directed RNA polymerase, mitochondrial	POLRMT	sp|O00411	6	6	6	1	5	4	2	1	5	4	2	1	5	4	2	8	138.62	0	44.827	93146000	4032000	42562000	12417000	34135000	7103200	2078200	6054000	2296200
Q05209	Q05209	Tyrosine-protein phosphatase non-receptor type	PTPN12	sp|Q0520	6	6	6	0	2	1	6	0	2	1	6	0	2	1	6	11.3	88.105	0	23.049	93081000	0	3526000	2815300	86739000	0	860100	1918200	2991600
P06865	P06865	Beta-hexosaminidase subunit alpha	HEXA	sp|P06865	3	3	3	0	2	0	2	0	2	0	2	0	2	0	2	6.4	60.702	0	6.2552	93004000	0	48563000	0	44440000	0	3753000	0	2355400
Q15526	Q15526	Surfeit locus protein 1	SURF1	sp|Q1552	3	3	3	0	2	0	3	0	2	0	3	0	2	0	3	12.3	33.331	0	7.9266	92939000	0	20768000	0	72171000	0	1892800	0	2905900
Q9H5N1	Q9H5N1	Rab GTPase-binding effector protein 2	RABEP2	sp|Q9H5N	1	1	1	0	1	0	1	0	1	0	1	0	1	0	1	2.1	63.542	0.007431	1.6572	92858000	0	15594000	0	77264000	0	1374600	0	3177500
P33527	P33527	Multidrug resistance-associated protein 1	ABCC1	sp|P33527	1	1	1	0	1	0	1	0	1	0	1	0	1	0	1	1.6	171.59	0	17.03	92755000	0	30340000	0	62415000	0	2674400	0	2566900
Q9H0E2	Q9H0E2	Toll-interacting protein	TOLLIP	sp|Q9H0E	2	2	2	0	2	1	2	0	2	1	2	0	2	1	2	9.9	30.281	0	5.6347	92495000	0	13848000	3540300	75107000	0	1018700	3091200	2578700
Q8TF66	Q8TF66	Leucine-rich repeat-containing protein 15	LRRC15	sp|Q8TF6	2	2	2	0	0	0	2	0	0	0	2	0	0	0	2	5.2	64.365	0	12.965	92388000	0	0	0	92388000	0	0	0	3799500
Q9HB40	Q9HB40	Retinoid-inducible serine carboxypeptidase	SCPEP1	sp|Q9HB4	2	2	2	0	2	1	2	0	2	1	2	0	2	1	2	5.1	50.83	0	5.1477	92351000	0	30299000	1136500	60915000	0	2272300	1008200	2659200
O15305	O15305	Phosphomannomutase 2	PMM2	sp|O1530	4	4	4	0	4	0	1	0	4	0	1	0	4	0	1	20.3	28.082	0	7.3593	92052000	0	80074000	0	11978000	0	6163000	0	1388100
P50613;Q	P50613	Cyclin-dependent kinase 7	CDK7	sp|P50613	4	4	4	0	1	1	4	0	1	1	4	0	1	1	4	15	39.038	0	7.9629	91912000	0	2850300	422580	88639000	0	0	1041700	2887600
Q9HBM6;C	Q9HBM6;C	Transcription initiation factor TFIID subunit 9B;Tra	TAF9B;TAF	sp|Q9HBM	4	4	4	0	4	0	2	0	4	0	2	0	4	0	2	15.9	27.622	0	7.7556	91895000	0	54855000	0	37040000	0	4752900	0	1605800
Q9HCS7	Q9HCS7	Pre-mRNA-splicing factor SYF1	XAB2	sp|Q9HCS	6	6	6	1	4	0	4	1	4	0	4	1	4	0	4	11.3	100.01	0	30.377	91792000	2044800	48795000	0	40952000	1834600	4349100	0	1846600
Q9BTZ2;PC	Q9BTZ2;PC	Dehydrogenase/reductase SDR family member 4;	DHRS4;DH	sp|Q9BTZ	3	3	3	1	3	0	3	1	3	0	3	1	3	0	3	11.2	29.537	0	4.5435	91507000	386680	26127000	0	64993000	1369300	1631600	0	2361600
O00483	O00483	Cytochrome c oxidase subunit NDUFA4	NDUFA4	sp|O0048	2	2	2	1	2	1	1	1	2	1	1	1	2	1	1	22.2	9.3697	0	4.4932	91204000	1226700	66261000	4370600	19346000	1476600	4943800	2284800	2095100
O14933	O14933	Ubiquitin/ISG15-conjugating enzyme E2 L6	UBE2L6	sp|O1493	2	2	2	0	1	0	2	0	1	0	2	0	1	0	2	15	17.769	0	5.4129	91153000	0	3461800	0	87692000	0	0	0	3606400
O15294	O15294	UDP-N-acetylglucosamine--peptide N-acetylgluco	OGT	sp|O1529	6	6	6	0	4	0	6	0	4	0	6	0	4	0	6	7.7	116.92	0	25.309	91055000	0	35112000	0	55943000	0	2722300	0	2673500
Q15843	Q15843	NEDD8	NEDD8	sp|Q1584	1	1	1	0	1	0	1	0	1	0	1	0	1	0	1	17.3	9.0714	0	4.4926	90901000	0	32172000	0	58729000	0	2695600	0	2555500
O14802	O14802	DNA-directed RNA polymerase III subunit RPC1	POLR3A	sp|O1480	2	2	2	0	2	0	1	0	2	0	1	0	2	0	1	1.7	155.64	0.001349	2.3803	90876000	0	26399000	0	64477000	0	2093800	0	2884900
Q9UKY1	Q9UKY1	Zinc fingers and homeoboxes protein 1	ZHX1	sp|Q9UKY	1	1	1	0	0	1	1	0	0	1	1	0	0	1	1	1.4	98.096	0.004741	1.819	90546000	0	0	3767500	86779000	0	0	2531700	3568800
Q9H5Q4	Q9H5Q4	Dimethyladenosine transferase 2, mitochondrial	TFB2M	sp|Q9H5Q	4	4	4	0	4	2	2	0	4	2	2	0	4	2	2	11.1	45.348	0	10.93	90429000	0	69171000	2720100	18538000	0	3801200	2849200	2037400

Q15050	Q15050	Ribosome biogenesis regulatory protein homolog	RRS1	sp|Q1505(	3	3	3	0	2	3	1	0	2	3	1	0	2	3	1	11.8	41.193	0	12.894	90407000	0	25391000	30543000	34473000	0	2806700	19034000	2339300
P78356	P78356	Phosphatidylinositol 5-phosphate 4-kinase type-2	PIP4K2B	sp|P78356	6	6	4	0	1	0	6	0	1	0	6	0	1	0	4	18	47.377	0	11.558	90266000	0	4771100	0	85495000	0	420570	0	3516000
Q9Y4R8	Q9Y4R8	Telomere length regulation protein TEL2 homolog	TELO2	sp|Q9Y4R8	5	5	5	0	3	2	4	0	3	2	4	0	3	2	4	8.2	91.746	0	18.972	90010000	0	10578000	497580	78934000	0	997660	612430	2903000
Q5VZE5	Q5VZE5	N-alpha-acetyltransferase 35, NatC auxiliary subu	NAA35	sp|Q5VZE5	2	2	2	0	2	0	2	0	2	0	2	0	2	0	2	4.7	83.638	0	11.664	89965000	0	14279000	0	75687000	0	975620	0	3395700
Q8TER5	Q8TER5	Rho guanine nucleotide exchange factor 40	ARHGEF40	sp|Q8TER5	9	9	9	0	0	4	9	0	0	4	9	0	0	4	9	8	164.66	0	17.11	89788000	0	0	6236800	83551000	0	0	5897900	1729300
Q8IYQ7	Q8IYQ7	Threonine synthase-like 1	THNSL1	sp|Q8IYQ7	4	4	4	0	4	0	3	0	4	0	3	0	4	0	3	7	83.069	0	12.499	89667000	0	41813000	0	47854000	0	3128500	0	2525200
O95671	O95671	N-acetylserotonin O-methyltransferase-like prote	ASMTL	sp|O95671	2	2	2	0	0	0	2	0	0	0	2	0	0	0	2	7.6	68.856	0	5.9171	89379000	0	0	0	89379000	0	0	0	3675800
O43813	O43813	LanC-like protein 1	LANCL1	sp|O43813	3	3	3	1	1	2	2	1	1	2	2	1	1	2	2	8	45.283	0	7.5777	89273000	1351400	16549000	6208700	65164000	1768700	2428900	3078300	2163300
P80217	P80217	Interferon-induced 35 kDa protein	IFI35	sp|P80217	3	3	3	0	1	0	3	0	1	0	3	0	1	0	3	13.3	31.546	0	9.6105	89159000	0	1480400	0	87679000	0	1153600	0	2582700
Q9UFC0	Q9UFC0	Leucine-rich repeat and WD repeat-containing pr	LRWD1	sp|Q9UFC(	3	3	3	0	3	0	2	0	3	0	2	0	3	0	2	6.2	70.86	0	9.9845	89072000	0	50790000	0	38282000	0	2979200	0	3072300
Q9NRP0	Q9NRP0	Oligosaccharyltransferase complex subunit OSTC	OSTC	sp|Q9NRP	1	1	1	0	1	0	1	0	1	0	1	0	1	0	1	8.1	16.829	0.000246	3.6322	88912000	0	34652000	0	54259000	0	3054600	0	2231400
Q8WUR7	Q8WUR7	UPF0235 protein C15orf40	C15orf40	sp|Q8WUI	2	2	2	0	2	1	2	0	2	1	2	0	2	1	2	13.7	16.353	0	7.1175	88847000	0	43211000	2659200	42977000	0	2222900	3636800	1503800
O75449	O75449	Katanin p60 ATPase-containing subunit A1	KATNA1	sp|O75449	4	4	4	0	3	0	4	0	3	0	4	0	3	0	4	10.4	55.964	0	9.0027	88811000	0	28491000	0	60319000	0	2869200	0	2123000
Q8IVF6;A2	Q8IVF6;A2	Ankyrin repeat domain-containing protein 18A;Ar	ANKRD18A	sp|Q8IVF6	2	2	2	0	1	1	2	0	1	1	2	0	1	1	2	2.6	115.6	0.004116	1.9076	88802000	0	28932000	6307600	53562000	0	3116800	4018200	1856800
Q12981	Q12981	Vesicle transport protein SEC20	BNIP1	sp|Q12981	3	3	3	0	3	1	2	0	3	1	2	0	3	1	2	17.1	26.132	0	14.666	88523000	0	55952000	2301100	30270000	0	3765300	2046200	1911700
Q9NUT2	Q9NUT2	ATP-binding cassette sub-family B member 8, mit	ABCB8	sp|Q9NUT	3	2	2	0	3	0	3	0	2	0	2	0	2	0	2	5.9	79.988	0	19.237	88515000	0	6212800	0	82303000	0	624090	0	3308300
Q15648	Q15648	Mediator of RNA polymerase II transcription subu	MED1	sp|Q15648	5	5	5	0	5	3	3	0	5	3	3	0	5	3	3	5.3	168.48	0	15.608	88365000	0	52430000	3212200	32723000	0	3608300	2528500	1989200
Q96DI7	Q96DI7	U5 small nuclear ribonucleoprotein 40 kDa protei	SNRNP40	sp|Q96DI7	4	4	4	1	4	3	2	1	4	3	2	1	4	3	2	18.5	39.31	0	11.288	88232000	2207000	37964000	5778700	42282000	3922500	1669000	3232400	2351700
Q10570	Q10570	Cleavage and polyadenylation specificity factor su	CPSF1	sp|Q1057(	5	5	5	0	5	1	4	0	5	1	4	0	5	1	4	5.7	160.88	0	16.47	88119000	0	41250000	1603000	45266000	0	3787600	1154400	1633000
Q96SZ6	Q96SZ6	CDK5 regulatory subunit-associated protein 1	CDK5RAP1	sp|Q96SZ6	3	3	3	0	2	0	3	0	2	0	3	0	2	0	3	7.2	67.688	0	26.084	88104000	0	36758000	0	51347000	0	3781400	0	1570400
O75629	O75629	Protein CREG1	CREG1	sp|O75629	1	1	1	0	1	1	0	0	1	1	0	0	1	1	0	9.5	24.074	0	4.3327	88056000	0	20122000	67933000	0	0	1773800	45651000	0
Q9H0W8	Q9H0W8	Protein SMG9	SMG9	sp|Q9H0W	5	5	5	0	2	0	5	0	2	0	5	0	2	0	5	20.6	57.65	0	13.33	87720000	0	12614000	0	75106000	0	2179300	0	2021400
O95239;Q2	O95239;Q2	Chromosome-associated kinesin KIF4A;Chromoso	KIF4A;KIF4	sp|O95239	7	6	6	1	7	3	5	1	6	3	4	1	6	3	4	6.3	139.88	0	11.604	87682000	889150	45756000	5199900	35836000	1428600	3472000	2529300	2460700
Q9UMR2	Q9UMR2	ATP-dependent RNA helicase DDX19B	DDX19B	sp|Q9UMI	13	2	2	1	13	0	9	0	2	0	0	0	2	0	0	36.3	53.926	0	32.675	87581000	0	87581000	0	0	0	7720200	0	0
Q8IV08	Q8IV08	Phospholipase D3	PLD3	sp|Q8IV08	2	2	2	0	1	1	2	0	1	1	2	0	1	1	2	4.1	54.705	0	5.1803	87501000	0	6476100	1410900	79614000	0	961290	0	2883800
P57105	P57105	Synaptojanin-2-binding protein	SYNJ2BP	sp|P57105	2	2	2	0	1	0	2	0	1	0	2	0	1	0	2	17.9	15.928	0	7.884	87431000	0	13443000	0	73987000	0	1923900	0	2303900
Q9C040	Q9C040	Tripartite motif-containing protein 2	TRIM2	sp|Q9C04(	3	3	3	1	0	0	3	1	0	0	3	1	0	0	3	5.1	81.529	0.000247	3.6579	87402000	832280	0	0	86570000	2767700	0	0	1624800
P18615	P18615	Negative elongation factor E	NELFE	sp|P18615	5	5	5	0	3	0	4	0	3	0	4	0	3	0	4	19.7	43.239	0	10.84	87370000	0	45716000	0	41654000	0	4005300	0	1737600
P84090	P84090	Enhancer of rudimentary homolog	ERH	sp|P84090	3	3	3	1	3	2	2	1	3	2	2	1	3	2	2	27.9	12.259	0	21.08	87359000	22576000	40888000	2111600	21784000	24238000	2706900	906660	643870
P61769	P61769	Beta-2-microglobulin;Beta-2-microglobulin form p	B2M	sp|P61769	2	2	2	0	1	0	2	0	1	0	2	0	1	0	2	16.8	13.714	0	5.6571	87301000	0	2369900	0	84931000	0	452920	0	3248800
O95772	O95772	MLN64 N-terminal domain homolog	STARD3NL	sp|O95772	2	2	2	0	2	1	2	0	2	1	2	0	2	1	2	14.1	26.654	0	10.272	87298000	0	18259000	5335400	63703000	0	1145800	4739200	1929700
Q9NW64	Q9NW64	Pre-mRNA-splicing factor RBM22	RBM22	sp|Q9NW6	4	4	4	0	4	3	3	0	4	3	3	0	4	3	3	10.5	46.895	0	4.5769	87189000	0	44547000	4419700	38222000	0	3312200	2087800	3068700
P00749	P00749	Urokinase-type plasminogen activator;Urokinase-	PLAU	sp|P00749	4	4	4	0	0	2	4	0	0	2	4	0	0	2	4	13.2	48.507	0	17.224	87085000	0	0	2035100	85050000	0	0	2415500	2449800
Q9UGJ0	Q9UGJ0	5-AMP-activated protein kinase subunit gamma-2	PRKAG2	sp|Q9UGJ(	1	1	1	0	0	0	1	0	0	0	1	0	0	0	1	3.2	63.065	0	7.4276	86938000	0	0	0	86938000	0	0	0	3575400
Q6GMV2	Q6GMV2	SET and MYND domain-containing protein 5	SMYD5	sp|Q6GM\	2	2	2	1	2	1	2	1	2	1	2	1	2	1	2	7.2	47.34	0	8.3395	86866000	25964000	15364000	1281400	44257000	25510000	791960	2633300	1064000
Q5M775	Q5M775	Cytospin-B	SPECC1	sp|Q5M77	7	7	7	0	1	7	4	0	1	7	4	0	1	7	4	8.8	118.58	0	13.38	86691000	0	18662000	32395000	35635000	0	1140400	22548000	1190900
Q9NXW2	Q9NXW2	DnaJ homolog subfamily B member 12	DNAJB12	sp|Q9NXW	4	4	4	0	1	4	4	0	1	4	4	0	1	4	4	16.5	41.859	0	8.4379	86619000	0	6668100	8511300	71440000	0	1510000	4752000	2983400
Q9HCK8;Q	Q9HCK8;Q	Chromodomain-helicase-DNA-binding protein 8;C	CHD8;CHD	sp|Q9HCK8	5	5	4	0	5	1	4	0	5	1	4	0	4	0	3	2.9	290.52	0	10.798	86603000	0	34099000	1328400	51175000	0	2432600	1729400	1841100
O43505	O43505	Beta-1,4-glucuronyltransferase 1	B4GAT1	sp|O43505	3	3	3	0	1	1	3	0	1	1	3	0	1	1	3	12.3	47.119	0	21.005	86443000	0	15234000	681070	70527000	0	2130700	1436300	1134000
O95470	O95470	Sphingosine-1-phosphate lyase 1	SGPL1	sp|O9547(	4	4	4	0	4	0	2	0	4	0	2	0	4	0	2	8.5	63.523	0	5.836	86381000	0	37014000	0	49366000	0	3070800	0	2222200
P42575	P42575	Caspase-2;Caspase-2 subunit p18;Caspase-2 subu	CASP2	sp|P42575	3	3	3	0	3	0	1	0	3	0	1	0	3	0	1	9.3	50.685	0	5.2861	86292000	0	56374000	0	29918000	0	4615800	0	1583900
Q92930	Q92930	Ras-related protein Rab-8B	RAB8B	sp|Q9293(	5	2	2	0	1	1	5	0	0	0	2	0	0	0	2	29	23.584	0	10.139	86241000	0	0	0	86241000	0	0	0	3546700
O14497	O14497	AT-rich interactive domain-containing protein 1A	ARID1A	sp|O14497	5	5	5	0	3	1	4	0	3	1	4	0	3	1	4	3.7	242.04	0	13.143	86199000	0	20853000	1131500	64215000	0	1369700	2021000	1848700
Q969M3	Q969M3	Protein YIPF5	YIPF5	sp|Q969M	2	2	2	0	1	1	2	0	1	1	2	0	1	1	2	18.7	27.989	0	6.2143	86174000	0	7571600	2157200	76445000	0	1029200	2235400	1996200
Q9UQN3	Q9UQN3	Charged multivesicular body protein 2b	CHMP2B	sp|Q9UQN	4	4	4	1	3	2	4	1	3	2	4	1	3	2	4	17.8	23.906	0	6.7976	86022000	49581	23439000	940190	61593000	155820	2461700	601650	2061500
Q8N2G8	Q8N2G8	GH3 domain-containing protein	GHDC	sp|Q8N2G	3	3	3	0	2	0	3	0	2	0	3	0	2	0	3	8.1	57.522	0	8.6652	85828000	0	21053000	0	64775000	0	1930200	0	2589500
P48509	P48509	CD151 antigen	CD151	sp|P48509	1	1	1	0	1	1	1	0	1	1	1	0	1	1	1	4	28.295	0.000947	3.1162	85623000	0	2602600	650930	82370000	0	235100	324630	3494600
Q9Y223	Q9Y223	Bifunctional UDP-N-acetylglucosamine 2-epimera	GNE	sp|Q9Y223	4	4	4	0	2	0	4	0	2	0	4	0	2	0	4	6.6	79.274	0	7.082	85535000	0	12152000	0	73383000	0	1198300	0	2890800
P0DSN7;A(	P0DSN7;A0A075B6S9		IGKV1D-37	sp|P0DSN7	1	1	1	0	0	1	0	0	0	1	0	0	0	1	0	13.7	12.688	0.000721	3.3435	85253000	0	0	85253000	0	0	0	54938000	0
P47929	P47929	Galectin-7	LGALS7	sp|P47929	4	4	4	0	0	4	0	0	0	4	0	0	0	4	0	38.2	15.075	0	10.889	85160000	0	0	85160000	0	0	0	55974000	0
Q8ND76	Q8ND76	Cyclin-Y	CCNY	sp|Q8ND7	1	1	1	0	1	0	1	0	1	0	1	0	1	0	1	4.4	39.336	0	5.464	85005000	0	11810000	0	73195000	0	1041000	0	3010200
Q7Z5K2	Q7Z5K2	Wings apart-like protein homolog	WAPAL	sp|Q7Z5K2	4	4	4	0	4	0	2	0	4	0	2	0	4	0	2	6.1	132.94	0	12.623	84931000	0	45629000	0	39301000	0	3425200	0	2213300
Q9NSK0	Q9NSK0	Kinesin light chain 4	KLC4	sp|Q9NSK(	4	2	2	1	3	0	1	1	1	0	0	1	1	0	0	7.1	68.639	0	5.0465	84892000	75473000	9420000	0	0	75473000	0	0	0
Q96J01	Q96J01	THO complex subunit 3	THOC3	sp|Q96J01	3	3	3	0	3	0	1	0	3	0	1	0	3	0	1	7.4	38.771	0	4.57	84841000	0	43663000	0	41178000	0	3371800	0	2170600
O43402	O43402	ER membrane protein complex subunit 8	EMC8	sp|O43402	2	2	2	0	2	0	1	0	2	0	1	0	2	0	1	11.9	23.773	0	10.201	84749000	0	50602000	0	34147000	0	3673100	0	2191700
Q86XI8	Q86XI8	Uncharacterized protein C19orf68	C19orf68	sp|Q86XI8	1	1	1	0	1	1	0	0	1	1	0	0	1	1	0	1.6	70.072	0.00965	1.5263	84623000	0	928000	83695000	0	0	81802	56243000	0
Q96BH1	Q96BH1	E3 ubiquitin-protein ligase RNF25	RNF25	sp|Q96BH	3	3	3	0	1	0	3	0	1	0	3	0	1	0	3	13.3	51.218	0	4.1297	84552000	0	4359500	0	80193000	0	1470600	0	2211700
Q7Z5L9	Q7Z5L9	Interferon regulatory factor 2-binding protein 2	IRF2BP2	sp|Q7Z5L9	3	3	3	0	3	0	1	0	3	0	1	0	3	0	1	9.9	61.024	0	32.034	84505000	0	48240000	0	36265000	0	3125900	0	2617800
Q8WUM9	Q8WUM9	Sodium-dependent phosphate transporter 1	SLC20A1	sp|Q8WUI	3	3	3	0	1	1	3	0	1	1	3	0	1	1	3	4	73.699	0	4.7977	84448000	0	2846400	2186200	79415000	0	641840	2467400	1876700
O43929;CC	O43929	Origin recognition complex subunit 4	ORC4	sp|O43929	3	3	3	0	3	0	3	0	3	0	3	0	3	0	3	8.7	50.377	0	6.7672	84038000	0	27717000	0	56321000	0	2573000	0	2186500
P22413	P22413	Ectonucleotide pyrophosphatase/phosphodiester	ENPP1	sp|P22413	3	3	3	0	1	0	3	0	1	0	3	0	1	0	3	5.8	104.92	0	10.887	83942000	0	8139200	0	75803000	0	1203400	0	2631600
Q16778;P3	Q16778;P3	Histone H2B type 2-E;Histone H2B type 1-B;Histor	HIST2H2BE	sp|Q16778	8	2	2	3	5	5	8	0	0	2	2	0	0	2	2	53.2	13.92	0.000943	3.0706	83871000	0	0	14224000	69646000	0	0	9683500	2739500
Q4G0N4	Q4G0N4	NAD kinase 2, mitochondrial	NADK2	sp|Q4G0N	5	5	5	0	5	0	4	0	5	0	4	0	5	0	4	15.8	49.432	0	13.05	83782000	0	43739000	0	40043000	0	3470000	0	2032400
P52298	P52298	Nuclear cap-binding protein subunit 2	NCBP2	sp|P52298	1	1	1	0	1	1	1	0	1	1	1	0	1	1	1	7.1	18.001	0.000245	3.5974	83777000	0	37111000	5811900	40854000	0	3271300	3905600	1680200
Q96Q15	Q96Q15	Serine/threonine-protein kinase SMG1	SMG1	sp|Q96Q1	7	7	7	0	3	0	6	0	3	0	6	0	3	0	6	3.4	410.5	0	7.4391	83693000	0	6181600	0	77512000	0	582750	0	3149900
Q96EV2	Q96EV2	RNA-binding protein 33	RBM33	sp|Q96EV2	3	3	3	1	3	1	3	1	3	1	3	1	3	1	3	4.1	129.98	0	7.1115	83618000	10364000	36766000	624450	35863000	14062000	694520	0	322840
Q7Z4G4	Q7Z4G4	tRNA (guanine(10)-N2)-methyltransferase homol	TRMT11	sp|Q7Z4G4	1	1	1	0	1	0	1	0	1	0	1	0	1	0	1	4.3	53.42	0	11.371	83497000	0	24465000	0	59032000	0	2156500	0	2427700
P05026	P05026	Sodium/potassium-transporting ATPase subunit b	ATP1B1	sp|P05026	2	2	2	0	2	0	2	0	2	0	2	0	2	0	2	7.3	35.061	0	4.0096	83448000	0	13834000	0	69615000	0	1031700	0	3050700
Q9HD15	Q9HD15	Steroid receptor RNA activator 1	SRA1	sp|Q9HD1	2	2	2	0	2	0	2	0	2	0	2	0	2	0	2	10.2	25.673	0.000245	3.5808	83281000	0	17233000	0	66048000	0	1540500	0	2694800
Q9BWS9	Q9BWS9	Chitinase domain-containing protein 1	CHID1	sp|Q9BWS	2	2	2	0	1	0	2	0	1	0	2	0	1	0	2	6.6	44.94	0	4.6289	83252000	0	22326000	0	60927000	0	0	0	2505600
O43815	O43815	Striatin	STRN	sp|O43815	4	3	3	0	4	1	3	0	3	0	2	0	3	0	2	8.2	86.131	0	9.1358	83225000	0	36990000	0	46235000	0	2816800	0	2345300
Q12959;Q9	Q12959	Disks large homolog 1	DLG1	sp|Q12959	4	4	4	0	3	1	3	0	3	1	3	0	3	1	3	4.8	100.45	0	8.8819	83219000	0	20446000	3032800	59740000	0	1042200	3295800	1959200
Q86SF2	Q86SF2	N-acetylgalactosaminyltransferase 7	GALNT7	sp|Q86SF2	5	5	5	0	1	0	5	0	1	0	5	0	1	0	5	9.9	75.388	0	8.9769	83200000	0	2867200	0	80333000	0	252740	0	3303700
P23025	P23025	DNA repair protein complementing XP-A cells	XPA	sp|P23025	2	2	2	0	2	0	1	0	2	0	1	0	2	0	1	13.6	31.368	0	7.1262	83175000	0	42916000	0	40259000	0	2648800	0	2789800
Q9BZF9	Q9BZF9	Uveal autoantigen with coiled-coil domains and a	UACA	sp|Q9BZF9	2	2	2	0	2	2	1	0	2	2	1	0	2	2	1	1.6	162.5	0.001138	2.5201	83085000	0	17678000	6570800	58836000	0	1472300	4021600	2899500
O15431	O15431	High affinity copper uptake protein 1	SLC31A1	sp|O15431	1	1	1	0	1	0	1	0	1	0	1	0	1	0	1	10	21.09	0	5.8693	83083000	0	21408000	0	61675000	0	1887100	0	2536400
Q9BZJ0	Q9BZJ0	Crooked neck-like protein 1	CRNKL1	sp|Q9BZJ0	1	1	1	0	1	0	1	0	1	0	1	0	1	0	1	1.5	100.45	0	10.626	83049000	0	19731000	0	63318000	0	0	0	2604000
P43353;P4	P43353;P4	Aldehyde dehydrogenase family 3 member B1;Al	ALDH3B1;/	sp|P43353	3	2	2	0	1	1	3	0	0	1	2	0	0	1	2	7.3	51.839	0.000951	3.1889	82997000	0	0	2299500	80698000	0	0	2439700	2424300
P23497	P23497	Nuclear autoantigen Sp-100	SP100	sp|P23497	7	4	4	0	4	0	6	0	1	0	4	0	1	0	4	11.9	100.42	0	14.033	82991000	0	5092500	0	77899000	0	924170	0	2728400
Q86Y39	Q86Y39	NADH dehydrogenase [ubiquinone] 1 alpha subcc	NDUFA11	sp|Q86Y39	2	2	2	0	2	1	2	0	2	1	2	0	2	1	2	18.4	14.852	0.000489	3.5337	82936000	0	21531000	428700	60977000	0	1737500	1127400	1828700
Q8N4V1	Q8N4V1	Membrane magnesium transporter 1	MMGT1	sp|Q8N4V	1	1	1	0	1	0	1	0	1	0	1	0	1	0	1	18.3	14.686	0	17.015	82853000	0	36361000	0	46492000	0	3205200	0	1912000
Q9UQ03	Q9UQ03	Coronin-2B	CORO2B	sp|Q9UQ0	5	5	5	0	1	5	3	0	1	5	3	0	1	5	3	10.8	54.953	0	13.99	82264000	0	2230000	42318000	37717000	0	190840	29392000	602550
O00165	O00165	HCLS1-associated protein X-1	HAX1	sp|O00165	3	3	3	0	3	1	1	0	3	1	1	0	3	1	1	24.7	31.62	0	13.544	82233000	0	60439000	3425400	18369000	0	3890900	3383500	1110400
Q9Y4P8	Q9Y4P8	WD repeat domain phosphoinositide-interacting	WIPI2	sp|Q9Y4P8	2	2	2	0	1	0	2	0	1	0	2	0	1	0	2	9.7	49.408	0	16.357	82186000	0	14647000	0	67539000	0	1880800	0	2187800
Q9UGR2	Q9UGR2	Zinc finger CCCH domain-containing protein 7B	ZC3H7B	sp|Q9UGR	2	2	2	0	0	1	2	0	0	1	2	0	0	1	2	4.3	109.86	0	10.073	82174000	0	0	2537800	79636000	0	0	3594600	1385900
Q8TAA9	Q8TAA9	Vang-like protein 1	VANGL1	sp|Q8TAA	7	7	7	0	1	7	2	0	1	7	2	0	1	7	2	22.9	59.974	0	26.246	82151000	0	1872000	51629000	28650000	0	219200	35049000	769430
P48163	P48163	NADP-dependent malic enzyme	ME1	sp|P48163	3	3	3	0	3	1	0	0	3	1	0	0	3	1	0	10.3	64.149	0	19.532	81929000	0	79989000	1939700	0	0	6015200	2339200	0
Q96PU5	Q96PU5	E3 ubiquitin-protein ligase NEDD4-like	NEDD4L	sp|Q96PU	4	2	2	0	0	0	4	0	0	0	2	0	0	0	2	3.8	111.93	0	5.6224	81576000	0	0	0	81576000	0	0	0	3354900
O75382	O75382	Tripartite motif-containing protein 3	TRIM3	sp|O75382	3	3	3	0	0	1	3	0	0	1	3	0	0	1	3	6.6	80.829	0	9.7906	81569000	0	0	3129100	78440000	0	0	2605700	2722900

Q9H201	Q9H201	Epsin-3	EPN3	sp|Q9H201	2	1	1	0	2	0	2	0	1	0	1	0	1	0	1	3.5	68.221	0.001138	2.5362	81546000	0	13108000	0	68438000	0	1155500	0	2814600
P09132	P09132	Signal recognition particle 19 kDa protein	SRP19	sp|P09132	3	3	3	1	2	1	3	1	2	1	3	1	2	1	3	19.4	16.156	0	12.712	81537000	385320	32704000	423710	48023000	489760	2535200	621730	1881200
Q8NBF6	Q8NBF6	Late secretory pathway protein AVL9 homolog	AVL9	sp|Q8NBF6	2	2	2	0	1	0	2	0	1	0	2	0	1	0	2	9.6	71.947	0	20.593	81496000	0	7209700	0	74287000	0	1455800	0	2234900
Q13362	Q13362	Serine/threonine-protein phosphatase 2A 56 kDa	PPP2R5C	sp|Q13362	6	2	2	0	3	2	6	0	0	0	2	0	0	0	2	19.5	61.06	0	74.484	81234000	0	0	0	81234000	0	0	0	3340800
P50750	P50750	Cyclin-dependent kinase 9	CDK9	sp|P50750	5	4	4	0	4	3	5	0	3	2	4	0	3	2	4	15.1	42.777	0	10.615	81233000	0	37411000	5145100	38676000	0	2536400	4029500	1780000
Q9UM22	Q9UM22	Mammalian ependymin-related protein 1	EPDR1	sp|Q9UM2	2	2	2	0	1	2	2	0	1	2	2	0	1	2	2	8.5	25.437	0	3.9837	81229000	0	4378700	4185000	72665000	0	0	4448600	1352100
Q969G6	Q969G6	Riboflavin kinase	RFK	sp|Q969G6	2	2	2	0	0	0	2	0	0	0	2	0	0	0	2	38.7	17.623	0	5.2823	81209000	0	0	0	81209000	0	0	0	3339800
Q9BVJ6;Q5	Q9BVJ6;Q5	U3 small nucleolar RNA-associated protein 14 hor	UTP14A;UT	sp|Q9BVJ6	2	2	2	0	1	2	2	0	1	2	2	0	1	2	2	3.1	87.977	0	7.0769	81051000	0	5435400	10566000	65049000	0	882400	7135800	2236700
O43251;Q9	O43251;Q9	RNA binding protein fox-1 homolog 2;RNA binding	RBFOX2;RE	sp|O43251	3	3	3	0	3	3	2	0	3	3	2	0	3	3	2	12.8	41.373	0	8.6581	81029000	0	43313000	13369000	24347000	0	3695800	8344800	1762900
O00461	O00461	Golgi integral membrane protein 4	GOLIM4	sp|O00461	4	4	4	1	2	2	3	1	2	2	3	1	2	2	3	6.8	81.879	0	28.489	80961000	5109600	14325000	2039900	59487000	3700100	1462800	1482500	1723100
Q86VM9	Q86VM9	Zinc finger CCCH domain-containing protein 18	ZC3H18	sp|Q86VM	4	4	4	0	4	3	3	0	4	3	3	0	4	3	3	6.7	106.38	0	6.6809	80775000	0	38670000	10447000	31657000	0	3290000	6257000	2184400
Q4G176	Q4G176	Acyl-CoA synthetase family member 3, mitochonc	ACSF3	sp|Q4G176	3	3	3	0	3	0	3	0	3	0	3	0	3	0	3	5.7	64.129	0	8.884	80713000	0	27228000	0	53485000	0	2397500	0	2202300
Q8NBI5	Q8NBI5	Solute carrier family 43 member 3	SLC43A3	sp|Q8NBI5	3	3	3	0	0	0	3	0	0	0	3	0	0	0	3	4.9	54.528	0	4.8847	80548000	0	0	0	80548000	0	0	0	3312600
Q13642	Q13642	Four and a half LIM domains protein 1	FHL1	sp|Q13642	2	2	2	1	2	0	1	1	2	0	1	1	2	0	1	7.1	36.263	0	5.8822	80439000	3288600	61353000	0	15798000	4273300	3767400	0	1305800
Q96DA6	Q96DA6	Mitochondrial import inner membrane translocas	DNAJC19	sp|Q96DA6	1	1	1	0	1	1	1	0	1	1	1	0	1	1	1	12.1	12.498	0	4.8051	80346000	0	38563000	4631200	37152000	0	3399300	3112100	1527900
Q8N4P3	Q8N4P3	Guanosine-3,5-bis(diphosphate) 3-pyrophosphoh	HDDC3	sp|Q8N4P3	3	3	3	0	3	0	1	0	3	0	1	0	3	0	1	24	20.329	0	18.497	80305000	0	63574000	0	16731000	0	4971400	0	1320700
O14981	O14981	TATA-binding protein-associated factor 172	BTAF1	sp|O14981	4	4	4	0	2	1	4	0	2	1	4	0	2	1	4	3.6	206.89	0	11.539	80294000	0	12691000	1002100	66601000	0	1292200	865700	2373200
Q4G0F5	Q4G0F5	Vacuolar protein sorting-associated protein 26B	VPS26B	sp|Q4G0F5	3	2	2	0	3	0	3	0	2	0	2	0	2	0	2	9.5	39.154	0	5.1504	80231000	0	28451000	0	51779000	0	2700600	0	1936800
Q13405	Q13405	39S ribosomal protein L49, mitochondrial	MRPL49	sp|Q13405	2	2	2	0	1	2	1	0	1	2	1	0	1	2	1	15.1	19.198	0	4.4582	79903000	0	22290000	1967400	55646000	0	1964800	1322100	2288500
Q6NYC8	Q6NYC8	Phostensin	PPP1R18	sp|Q6NYC8	3	3	3	0	0	0	3	0	0	0	3	0	0	0	3	8.2	67.942	0	18.848	79719000	0	0	0	79719000	0	0	0	3278500
Q14141	Q14141	Septin-6	Sep-06	sp|Q14141	10	5	5	1	10	4	5	0	5	0	0	0	5	0	0	26.3	49.716	0	15.025	79643000	0	79643000	0	0	0	7020400	0	0
Q6P158	Q6P158	Putative ATP-dependent RNA helicase DHX57	DHX57	sp|Q6P158	5	4	4	1	4	1	4	0	4	0	3	0	4	0	3	4.1	155.6	0.001153	2.7157	79457000	0	31403000	0	48054000	0	3399100	0	1345300
Q16877;P1	Q16877;P1	6-phosphofructo-2-kinase/fructose-2,6-bisphosph	PFKFB4;PF	sp|Q16877	3	3	2	1	0	0	2	1	0	0	2	0	0	0	2	8.1	54.039	0	5.8036	79420000	33723000	0	0	45696000	0	0	0	1879300
Q9Y4X5	Q9Y4X5	E3 ubiquitin-protein ligase ARIH1	ARIH1	sp|Q9Y4X5	4	4	4	0	3	0	3	0	3	0	3	0	3	0	3	11	64.117	0	29.331	79193000	0	44630000	0	34563000	0	4537600	0	817940
Q8TCC3	Q8TCC3	39S ribosomal protein L30, mitochondrial	MRPL30	sp|Q8TCC3	3	3	3	0	3	2	3	0	3	2	3	0	3	2	3	18	18.546	0	5.7212	79109000	0	29910000	2671100	46527000	0	2454800	2091500	1787100
Q86U90	Q86U90	YrdC domain-containing protein, mitochondrial	YRDC	sp|Q86U90	3	3	3	0	3	0	2	0	3	0	2	0	3	0	2	14	29.328	0	10.019	78935000	0	48412000	0	30523000	0	3884500	0	1638200
Q92504	Q92504	Zinc transporter SLC39A7	SLC39A7	sp|Q92504	1	1	1	1	1	0	1	1	1	0	1	1	1	0	1	3	50.117	0	4.9011	78807000	3174500	30350000	0	45282000	3174500	2675300	0	1862300
Q9BXW9	Q9BXW9	Fanconi anemia group D2 protein	FANCD2	sp|Q9BXW	5	5	5	1	5	1	3	1	5	1	3	1	5	1	3	4.9	164.13	0	9.8788	78644000	614240	48232000	457980	29340000	1926900	2797400	0	1348200
Q9UNN5	Q9UNN5	FAS-associated factor 1	FAF1	sp|Q9UNN	7	7	7	0	7	1	0	0	7	1	0	0	7	1	0	13.7	73.953	0	29.475	78610000	0	77309000	1300800	0	0	6814700	874100	0
Q9H2H8	Q9H2H8	Peptidyl-prolyl cis-trans isomerase-like 3	PPIL3	sp|Q9H2H8	2	2	2	0	1	1	2	0	1	1	2	0	1	1	2	16.1	18.154	0	8.6472	78558000	0	14473000	1292900	62792000	0	2002200	1363500	1361300
Q15154	Q15154	Pericentriolar material 1 protein	PCM1	sp|Q15154	5	5	5	1	1	1	5	1	1	1	5	1	1	1	5	3.2	228.54	0	10.567	78540000	23522000	3499700	2729700	48788000	24564000	322150	1915600	870170
P15309	P15309	Prostatic acid phosphatase;PAPf39	ACPP	sp|P15309	3	3	3	0	0	1	3	0	0	1	3	0	0	1	3	9.6	44.566	0	7.0107	78529000	0	0	684000	77845000	0	0	512820	3148200
Q8TED1	Q8TED1	Probable glutathione peroxidase 8	GPX8	sp|Q8TED1	2	2	2	0	2	1	2	0	2	1	2	0	2	1	2	10	23.881	0	4.7081	78499000	0	12410000	1320200	64769000	0	1013200	1443700	2187800
Q96AB3	Q96AB3	Isochorismatase domain-containing protein 2, mit	ISOC2	sp|Q96AB3	2	2	2	0	1	0	1	0	1	0	1	0	1	0	1	20	22.337	0	27.375	78440000	0	6657900	0	71782000	0	0	0	2952100
O75818	O75818	Ribonuclease P protein subunit p40	RPP40	sp|O75818	2	2	2	0	2	1	1	0	2	1	1	0	2	1	1	10.5	41.833	0	9.2079	78412000	0	51308000	3838500	23265000	0	3927900	2567400	1563700
Q13610	Q13610	Periodic tryptophan protein 1 homolog	PWP1	sp|Q13610	2	2	2	1	2	0	1	1	2	0	1	1	2	0	1	7.2	55.827	0.000485	3.447	78376000	39884000	20272000	0	18220000	40425000	1235100	0	759500
O94832	O94832	Unconventional myosin-Id	MYO1D	sp|O94832	5	5	5	2	4	2	4	2	4	2	4	2	4	2	4	5.2	116.2	0	8.2401	78182000	9368300	28118000	1491000	39205000	5328200	2910300	3757900	2464700
Q13188	Q13188	Serine/threonine-protein kinase 3;Serine/threoni	STK3	sp|Q13188	3	2	2	0	3	0	3	0	2	0	2	0	2	0	2	7.7	56.3	0	6.417	78173000	0	35737000	0	42435000	0	3193800	0	1701600
Q99700	Q99700	Ataxin-2	ATXN2	sp|Q99700	4	4	4	0	4	1	2	0	4	1	2	0	4	1	2	8	140.28	0	8.5879	77915000	0	46663000	685560	30567000	0	3015500	1291600	1523900
O75330	O75330	Hyaluronan mediated motility receptor	HMMR	sp|O75330	4	4	4	0	2	4	2	0	2	4	2	0	2	4	2	8.6	84.099	0	15.494	77731000	0	9572900	18970000	49188000	0	1585100	11816000	2214100
Q7LG56	Q7LG56	Ribonucleoside-diphosphate reductase subunit M	RRM2B	sp|Q7LG56	3	2	2	0	3	0	2	0	2	0	1	0	2	0	1	13.7	40.736	0	14.321	77539000	0	32063000	0	45476000	0	1846500	0	2850100
Q9ULH7	Q9ULH7	MKL/myocardin-like protein 2	MKL2	sp|Q9ULH7	3	3	2	0	3	0	3	0	3	0	3	0	2	0	2	5.9	118.13	0.002876	2.1578	77527000	0	23676000	0	53851000	0	2008600	0	2293100
P07738	P07738	Bisphosphoglycerate mutase	BPGM	sp|P07738	2	2	2	0	1	0	2	0	1	0	2	0	1	0	2	12.4	30.005	0	12.623	77387000	0	16800000	0	60587000	0	1556900	0	2415600
A6NCN2	A6NCN2	Putative keratin-87 protein	KRT87P	sp|A6NCN2	7	1	1	0	7	0	0	0	1	0	0	0	1	0	0	25.1	29.117	0.001159	2.8031	77219000	0	77219000	0	0	0	6806800	0	0
Q9UDT6	Q9UDT6	CAP-Gly domain-containing linker protein 2	CLIP2	sp|Q9UDT	8	6	6	0	1	4	8	0	0	3	6	0	0	3	6	14.1	115.84	0	18.941	77215000	0	0	2077300	75138000	0	0	2888900	1597200
Q4G148	Q4G148	Glucoside xylosyltransferase 1	GXYLT1	sp|Q4G148	3	3	3	0	2	0	3	0	2	0	3	0	2	0	3	8.2	50.566	0	6.4962	76886000	0	10410000	0	66476000	0	1099200	0	2552300
P18510	P18510	Interleukin-1 receptor antagonist protein	IL1RN	sp|P18510	2	2	2	0	0	2	2	0	0	2	2	0	0	2	2	21.5	20.055	0	49.421	76861000	0	0	4568700	72292000	0	0	3916300	2126900
Q99836	Q99836	Myeloid differentiation primary response protein	MYD88	sp|Q99836	3	3	3	0	0	0	3	0	0	0	3	0	0	0	3	17.9	33.233	0	5.79	76779000	0	0	0	76779000	0	0	0	3157600
P85037;Q0	P85037	Forkhead box protein K1	FOXK1	sp|P85037	4	4	4	0	4	0	2	0	4	0	2	0	4	0	2	8.3	75.456	0	8.5829	76750000	0	66726000	0	10024000	0	4908200	0	1385900
Q9P0M9	Q9P0M9	39S ribosomal protein L27, mitochondrial	MRPL27	sp|Q9P0M	2	2	2	0	1	1	2	0	1	1	2	0	1	1	2	16.9	16.073	0.009648	1.5263	76636000	0	22432000	2713200	51491000	0	2064200	2067800	1786200
Q12824	Q12824	SWI/SNF-related matrix-associated actin-depende	SMARCB1	sp|Q12824	3	3	3	0	2	1	2	0	2	1	2	0	2	1	2	11.4	44.141	0	6.1162	76485000	0	46863000	904020	28718000	0	3859700	879590	1180100
Q13049	Q13049	E3 ubiquitin-protein ligase TRIM32	TRIM32	sp|Q13049	2	2	2	0	0	1	2	0	0	1	2	0	0	1	2	3.5	71.988	0	5.8935	76439000	0	0	4055500	72384000	0	0	2965000	2737100
Q9H6V9	Q9H6V9	UPF0554 protein C2orf43	C2orf43	sp|Q9H6V9	2	2	2	0	2	0	2	0	2	0	2	0	2	0	2	7.4	37.318	0	4.4135	76402000	0	10539000	0	65863000	0	944730	0	2692900
O75380	O75380	NADH dehydrogenase [ubiquinone] iron-sulfur pr	NDUFS6	sp|O75380	3	3	3	0	3	1	2	0	3	1	2	0	3	1	2	31.5	13.711	0	5.551	76361000	0	42412000	440060	33509000	0	3430000	551560	1430800
Q8IWR0	Q8IWR0	Zinc finger CCCH domain-containing protein 7A	ZC3H7A	sp|Q8IWR0	2	2	2	0	2	2	2	0	2	2	2	0	2	2	2	3.4	110.54	0.001135	2.495	76355000	0	23579000	6734000	46042000	0	2585200	4022900	1889000
P35555;P3	P35555	Fibrillin-1	FBN1	sp|P35555	4	4	4	0	1	0	4	0	1	0	4	0	1	0	4	1.6	312.3	0	8.9125	76333000	0	5471500	0	70861000	0	691310	0	2705200
Q96RE7	Q96RE7	Nucleus accumbens-associated protein 1	NACC1	sp|Q96RE7	5	5	5	0	2	1	4	0	2	1	4	0	2	1	4	12	57.258	0	10.708	76208000	0	12181000	685940	63341000	0	1132700	943880	2063000
O75027	O75027	ATP-binding cassette sub-family B member 7, mit	ABCB7	sp|O75027	5	5	5	0	2	0	3	0	2	0	3	0	2	0	3	11.4	82.64	0	18.081	76093000	0	25007000	0	51086000	0	0	0	2100900
Q01650;Q9	Q01650;Q9	Large neutral amino acids transporter small subui	SLC7A5;SL	sp|Q01650	2	2	1	0	2	1	1	0	2	1	1	0	1	1	1	4.1	55.01	0	4.1239	76030000	0	47378000	706910	27945000	0	3083500	1284100	1433000
P37840	P37840	Alpha-synuclein	SNCA	sp|P37840	3	3	3	1	3	2	2	1	3	2	2	1	3	2	2	27.1	14.46	0	10.418	75892000	759830	56539000	5225800	13367000	1872200	4781300	2264600	887040
Q9Y2W6	Q9Y2W6	Tudor and KH domain-containing protein	TDRKH	sp|Q9Y2W	3	3	3	0	3	0	2	0	3	0	2	0	3	0	2	9.6	62.045	0	9.2409	75673000	0	36160000	0	39512000	0	2831200	0	1981300
Q53EZ4	Q53EZ4	Centrosomal protein of 55 kDa	CEP55	sp|Q53EZ4	4	4	4	0	2	4	4	0	2	4	4	0	2	4	4	11.4	54.178	0	8.1598	75609000	0	5760600	19758000	50091000	0	4023700	9392800	2428300
Q15582	Q15582	Transforming growth factor-beta-induced proteir	TGFBI	sp|Q15582	4	4	4	0	2	1	4	0	2	1	4	0	2	1	4	6.7	74.68	0	7.0296	75583000	0	4101300	1674800	69807000	0	1414700	1421500	1521600
O43567	O43567	E3 ubiquitin-protein ligase RNF13	RNF13	sp|O43567	1	1	1	0	1	0	1	0	1	0	1	0	1	0	1	6.3	42.813	0	8.1893	75537000	0	11053000	0	64484000	0	974330	0	2651900
Q9NRL3	Q9NRL3	Striatin-4	STRN4	sp|Q9NRL3	4	3	3	0	4	1	3	0	3	0	2	0	3	0	2	8.9	80.595	0	8.6865	75519000	0	45098000	0	30421000	0	3144200	0	2082300
Q9BRK4;Q9	Q9BRK4	Leucine zipper putative tumor suppressor 2	LZTS2	sp|Q9BRK4	7	7	7	0	0	7	1	0	0	7	1	0	0	7	1	15.5	72.759	0	20.427	75210000	0	0	53591000	21620000	0	0	36013000	889130
P27105	P27105	Erythrocyte band 7 integral membrane protein	STOM	sp|P27105	4	4	4	0	3	0	3	0	3	0	3	0	3	0	3	21.5	31.73	0	8.6168	75177000	0	27467000	0	47710000	0	2662400	0	1720900
Q8N999	Q8N999	Uncharacterized protein C12orf29	C12orf29	sp|Q8N999	2	2	2	0	1	1	2	0	1	1	2	0	1	1	2	7.4	37.49	0.001162	2.8395	75137000	0	17032000	6827500	51277000	0	1669900	5103100	1218800
Q7Z4T9	Q7Z4T9	Protein MAATS1	MAATS1	sp|Q7Z4T9	1	1	1	1	0	1	0	1	0	1	0	1	0	1	0	1	89.954	0.009662	1.5338	75135000	74516000	0	619420	0	74516000	0	416250	0
Q8NHQ9	Q8NHQ9	ATP-dependent RNA helicase DDX55	DDX55	sp|Q8NHQ	2	2	2	0	2	2	1	0	2	2	1	0	2	2	1	7.5	68.546	0	14.296	74998000	0	61718000	7793100	5487000	0	5123700	3836400	1942800
Q9BTD8	Q9BTD8	RNA-binding protein 42	RBM42	sp|Q9BTD8	2	2	2	0	2	0	2	0	2	0	2	0	2	0	2	6	50.413	0	6.1506	74980000	0	32090000	0	42890000	0	2823500	0	1769100
Q9NXV6	Q9NXV6	CDKN2A-interacting protein	CDKN2AIP	sp|Q9NXV6	7	7	7	0	4	7	5	0	4	7	5	0	4	7	5	17.2	61.124	0	18.637	74912000	0	21192000	12782000	40939000	0	3232200	6555400	2353500
O15344	O15344	E3 ubiquitin-protein ligase Midline-1	MID1	sp|O15344	4	4	4	0	3	1	4	0	3	1	4	0	3	1	4	10	75.25	0	13.906	74837000	0	18929000	2197000	53711000	0	1698900	1676200	1978700
P11441	P11441	Ubiquitin-like protein 4A	UBL4A	sp|P11441	3	3	3	2	3	1	2	2	3	1	2	2	3	1	2	22.9	17.776	0	8.1438	74810000	5693500	48728000	2785300	17604000	6539500	2513200	2786300	745510
P49768	P49768	Presenilin-1;Presenilin-1 NTF subunit;Presenilin-1	PSEN1	sp|P49768	4	4	2	0	1	1	4	0	1	1	4	0	0	0	2	13.5	52.667	0	18.372	74683000	0	6738600	488280	67456000	0	0	0	2774200
Q5K651	Q5K651	Sterile alpha motif domain-containing protein 9	SAMD9	sp|Q5K651	5	5	5	0	1	1	5	0	1	1	5	0	1	1	5	4.7	184.28	0	12.305	74682000	0	5499500	2080700	67102000	0	913290	2212900	1516400
Q53EP0	Q53EP0	Fibronectin type III domain-containing protein 3B	FNDC3B	sp|Q53EP0	4	4	4	0	1	1	4	0	1	1	4	0	1	1	4	5.1	132.89	0	17.561	74397000	0	425780	1169600	72802000	0	160870	736170	2920500
Q9Y6G3	Q9Y6G3	39S ribosomal protein L42, mitochondrial	MRPL42	sp|Q9Y6G3	2	2	2	0	2	0	2	0	2	0	2	0	2	0	2	21.8	16.661	0	4.2616	74212000	0	42212000	0	32000000	0	3722100	0	1314900
P14649	P14649	Myosin light chain 6B	MYL6B	sp|P14649	5	3	3	1	2	3	5	0	1	1	3	0	1	1	3	33.2	22.764	0	5.9759	73912000	0	9920900	1489100	62502000	0	0	0	2570400
Q9NP81	Q9NP81	Serine--tRNA ligase, mitochondrial	SARS2	sp|Q9NP81	5	5	5	0	5	0	2	0	5	0	2	0	5	0	2	14.3	58.282	0	11.464	73912000	0	38982000	0	34930000	0	3118900	0	1753800
Q9UJC3	Q9UJC3	Protein Hook homolog 1	HOOK1	sp|Q9UJC3	6	6	6	1	6	0	0	1	6	0	0	1	6	0	0	10.4	84.647	0	18.453	73872000	1530200	72341000	0	0	1530200	6376800	0	0
Q12800;Q9	Q12800;Q9	Alpha-globin transcription factor CP2;Upstream-b	TFCP2;UBF	sp|Q12800	2	2	2	0	2	1	1	0	2	1	1	0	2	1	1	6	57.255	0	11.511	73841000	0	45661000	2236600	25944000	0	3373800	1748200	1472900
Q96AX1	Q96AX1	Vacuolar protein sorting-associated protein 33A	VPS33A	sp|Q96AX1	3	3	3	0	0	0	3	0	0	0	3	0	0	0	3	7.7	67.61	0	10.303	73778000	0	0	0	73778000	0	0	0	3034200
Q9H1D9	Q9H1D9	DNA-directed RNA polymerase III subunit RPC6	POLR3F	sp|Q9H1D9	3	3	3	0	3	1	0	0	3	1	0	0	3	1	0	11.7	35.684	0	6.1459	73639000	0	72937000	701860	0	0	2402800	4498100	0
Q14192	Q14192	Four and a half LIM domains protein 2	FHL2	sp|Q14192	2	2	2	0	0	1	2	0	0	1	2	0	0	1	2	10	32.193	0	6.6718	73498000	0	0	3172100	70326000	0	0	2271800	2752100
Q9NVE7	Q9NVE7	Pantothenate kinase 4	PANK4	sp|Q9NVE7	5	5	5	0	3	1	4	0	3	1	4	0	3	1	4	8	85.99	0	14.463	73476000	0	27652000	2965500	42859000	0	2003100	2213700	1976000
P48651	P48651	Phosphatidylserine synthase 1	PTDSS1	sp|P48651	1	1	1	0	1	0	1	0	1	0	1	0	1	0	1	3	55.527	0	4.1551	73464000	0	14107000	0	59357000	0	1243500	0	2441100
P55042	P55042	GTP-binding protein RAD	RRAD	sp|P55042	2	2	2	0	0	2	2	0	0	2	2	0	0	2	2	7.8	33.245	0	4.9471	73327000	0	0	4362600	68965000	0	0	2963700	2804200
Q12974;Q9	Q12974;Q9	Protein tyrosine phosphatase type IVA 2;Protein t	PTP4A2;PT	sp|Q12974	3	3	3	0	3	0	1	0	3	0	1	0	3	0	1	18.6	19.127	0	6.1441	73256000	0	42224000	0	31032000	0	2699000	0	2299200

Q99747	Q99747	Gamma-soluble NSF attachment protein	NAPG	sp|Q99747	3	3	3	0	3	0	2	0	3	0	2	0	3	0	2	12.2	34.746	0	12.77	73197000	0	34355000	0	38842000	0	3000600	0	1625100
Q9NZN3	Q9NZN3	EH domain-containing protein 3	EHD3	sp|Q9NZN	12	3	2	0	6	4	10	0	1	0	2	0	0	0	2	29.5	60.886	0	4.9069	72911000	0	18422000	0	54489000	0	1623900	0	0
Q6NW29	Q6NW29	RWD domain-containing protein 4	RWDD4	sp|Q6NW2	2	2	2	0	2	0	2	0	2	0	2	0	2	0	2	10.1	21.25	0.00072	3.3262	72880000	0	23886000	0	48994000	0	2096700	0	2023700
P51617	P51617	Interleukin-1 receptor-associated kinase 1	IRAK1	sp|P51617	4	4	3	0	4	0	2	0	4	0	2	0	3	0	1	7.2	76.536	0	19.164	72695000	0	50054000	0	22640000	0	3736600	0	1606700
Q9BRZ2	Q9BRZ2	E3 ubiquitin-protein ligase TRIM56	TRIM56	sp|Q9BRZ	3	3	3	0	0	3	2	0	0	3	2	0	0	3	2	6.1	81.487	0	13.638	72639000	0	0	9339600	63299000	0	0	4985600	3893800
Q6NUQ4	Q6NUQ4	Transmembrane protein 214	TMEM214	sp|Q6NUC	2	2	2	0	1	1	2	0	1	1	2	0	1	1	2	3.5	77.15	0	7.4648	72558000	0	11202000	3460000	57896000	0	1162900	2738300	1792300
P36639	P36639	7,8-dihydro-8-oxoguanine triphosphatase	NUDT1	sp|P36639	2	2	2	0	2	0	0	0	2	0	0	0	2	0	0	14.2	22.519	0	5.165	72437000	0	72437000	0	0	0	6385300	0	0
Q9UBL3	Q9UBL3	Set1/Ash2 histone methyltransferase complex sul	ASH2L	sp|Q9UBL	2	2	2	0	2	0	1	0	2	0	1	0	2	0	1	5.6	68.722	0	12.904	72429000	0	61718000	0	10710000	0	4286000	0	1594900
O95166;Q	O95166;Q	Gamma-aminobutyric acid receptor-associated pr	GABARAP;	sp|O95166	2	2	2	0	1	0	2	0	1	0	2	0	1	0	2	23.9	13.918	0	5.1555	72330000	0	6142800	0	66187000	0	0	0	2722000
Q8TCU6	Q8TCU6	Phosphatidylinositol 3,4,5-trisphosphate-depende	PREX1	sp|Q8TCU	5	5	5	0	0	1	5	0	0	1	5	0	0	1	5	3.7	186.2	0	9.1456	72325000	0	0	658910	71666000	0	0	852280	2537800
Q9BUR4	Q9BUR4	Telomerase Cajal body protein 1	WRAP53	sp|Q9BUR	2	2	2	0	1	0	2	0	1	0	2	0	1	0	2	5.7	59.309	0	6.2347	72077000	0	4105500	0	67972000	0	1371600	0	1785600
P42696	P42696	RNA-binding protein 34	RBM34	sp|P42696	4	4	4	0	2	4	2	0	2	4	2	0	2	4	2	12.3	48.564	0	7.8294	72035000	0	9996500	18989000	43049000	0	1469400	12541000	1402400
O94842	O94842	TOX high mobility group box family member 4	TOX4	sp|O94842	3	3	1	1	2	1	2	1	2	1	2	0	0	0	1	6.4	66.194	0	12.384	72030000	934690	29130000	1577000	40389000	1540000	1639300	1639800	1404100
Q8N5M9	Q8N5M9	Protein jagunal homolog 1	JAGN1	sp|Q8N5M	1	1	1	0	1	1	1	0	1	1	1	0	1	1	1	6.6	21.125	0	5.1861	71983000	0	30448000	1930000	39605000	0	2694400	1349200	1566000
B9A064;P0	B9A064;P0	Immunoglobulin lambda-like polypeptide 5;Ig lam	IGLL5;IGLC	sp|B9A064	4	2	2	3	0	3	0	2	0	1	0	2	0	1	0	26.6	23.063	0.001145	2.6103	71964000	68274000	0	3689500	0	31399000	0	39355000	0
Q8WXD5	Q8WXD5	Gem-associated protein 6	GEMIN6	sp|Q8WXD	3	3	3	1	3	0	3	1	3	0	3	1	3	0	3	21.6	18.824	0	4.5074	71900000	900840	31297000	0	39703000	0	2658500	0	1733000
Q9HBL7	Q9HBL7	Plasminogen receptor (KT)	PLGRKT	sp|Q9HBL	1	1	1	0	0	1	1	0	0	1	1	0	0	1	1	8.8	17.201	0	4.5549	71877000	0	0	2722400	69154000	0	0	1835300	2838100
P08047	P08047	Transcription factor Sp1	SP1	sp|P08047	2	2	2	0	2	1	2	0	2	1	2	0	2	1	2	5.5	80.692	0	10.589	71856000	0	25630000	3479300	42746000	0	1925400	2908700	1521200
P18887	P18887	DNA repair protein XRCC1	XRCC1	sp|P18887	2	2	2	0	2	0	2	0	2	0	2	0	2	0	2	3.5	69.476	0	6.9196	71772000	0	18266000	0	53506000	0	1673100	0	2137500
Q9BWQ6	Q9BWQ6	Protein YIPF2	YIPF2	sp|Q9BWC	1	1	1	0	1	0	1	0	1	0	1	0	1	0	1	9.2	35.151	0	9.6654	71606000	0	20180000	0	51426000	0	1778900	0	2114900
P58107	P58107	Epiplakin	EPPK1	sp|P58107	8	1	1	1	2	8	7	0	1	1	1	0	1	1	1	9	555.65	0.001149	2.6469	71557000	0	26635000	13615000	31307000	0	0	0	1287500
Q9NZJ7	Q9NZJ7	Mitochondrial carrier homolog 1	MTCH1	sp|Q9NZJ7	1	1	1	0	1	1	1	0	1	1	1	0	1	1	1	2.8	41.544	0.000489	3.5382	71462000	0	20022000	1261000	50179000	0	1764900	847400	2063600
Q16718	Q16718	NADH dehydrogenase [ubiquinone] 1 alpha subcc	NDUFA5	sp|Q16718	1	1	1	0	1	0	1	0	1	0	1	0	1	0	1	8.6	13.459	0.000716	3.2665	71298000	0	29023000	0	42276000	0	2558300	0	1738600
Q04726;Q0	Q04726;Q0	Transducin-like enhancer protein 3;Transducin-lik	TLE3;TLE2;	sp|Q04726	3	3	3	0	3	0	2	0	3	0	2	0	3	0	2	4.8	83.416	0	7.1424	71289000	0	48136000	0	23153000	0	4192800	0	1002500
P04114	P04114	Apolipoprotein B-100;Apolipoprotein B-48	APOB	sp|P04114	3	2	2	1	1	0	2	1	1	0	1	1	1	0	1	0.7	515.6	0.001142	2.5774	71280000	55305	35959000	0	35266000	55305	0	0	1450300
Q5VTL8	Q5VTL8	Pre-mRNA-splicing factor 38B	PRPF38B	sp|Q5VTL8	2	2	2	1	2	1	2	1	2	1	2	1	2	1	2	3.7	64.467	0	4.5011	71214000	2431700	28864000	2570100	37348000	2518300	2595500	1654200	1471100
P04150	P04150	Glucocorticoid receptor	NR3C1	sp|P04150	4	4	4	1	0	1	4	1	0	1	4	1	0	1	4	7.7	85.658	0	7.1424	71182000	26393000	0	258760	44531000	24745000	0	2139500	1513500
Q8TB61	Q8TB61	Adenosine 3-phospho 5-phosphosulfate transpor	SLC35B2	sp|Q8TB6	4	4	4	0	2	3	4	0	2	3	4	0	2	3	4	8.3	47.514	0	4.6135	71155000	0	5019500	3191300	62944000	0	1196400	1706600	2272600
Q5SWX8	Q5SWX8	Protein odr-4 homolog	ODR4	sp|Q5SWX	2	2	2	0	2	0	2	0	2	0	2	0	2	0	2	4.6	51.103	0.000248	3.7045	71048000	0	17716000	0	53332000	0	1571000	0	2183900
P56377	P56377	AP-1 complex subunit sigma-2	AP1S2	sp|P56377	2	2	2	0	2	0	2	0	2	0	2	0	2	0	2	15.9	18.615	0	7.2883	71026000	0	35992000	0	35034000	0	3069800	0	1543700
Q9Y2H6	Q9Y2H6	Fibronectin type-III domain-containing protein 3A	FNDC3A	sp|Q9Y2H	3	3	3	0	1	0	3	0	1	0	3	0	1	0	3	4.5	131.85	0	12.866	70833000	0	3482800	0	67350000	0	1622300	0	1454600
P13498	P13498	Cytochrome b-245 light chain	CYBA	sp|P13498	2	2	2	0	2	1	2	0	2	1	2	0	2	1	2	8.7	21.012	0.001146	2.6194	70693000	0	25377000	759010	44557000	0	1579400	1294800	1705200
Q8N111	Q8N111	Cell cycle exit and neuronal differentiation protei	CEND1	sp|Q8N11	2	2	2	0	0	0	2	0	0	0	2	0	0	0	2	28.2	14.954	0	5.2047	70660000	0	0	0	70660000	0	0	0	2906000
P49840	P49840	Glycogen synthase kinase-3 alpha	GSK3A	sp|P49840	3	2	2	0	3	0	3	0	2	0	2	0	2	0	2	8.5	50.98	0	6.2315	70408000	0	28930000	0	41477000	0	2483000	0	1773000
Q9NPJ3	Q9NPJ3	Acyl-coenzyme A thioesterase 13;Acyl-coenzyme	ACOT13	sp|Q9NPJ	1	1	1	0	1	0	1	0	1	0	1	0	1	0	1	7.1	14.96	0.001129	2.4313	70371000	0	33938000	0	36433000	0	2991600	0	1498300
Q9Y4B6	Q9Y4B6	Protein VPRBP	VPRBP	sp|Q9Y4B	3	3	3	0	2	0	2	0	2	0	2	0	2	0	2	3.9	169.01	0	5.9377	70309000	0	22807000	0	47502000	0	0	0	1953600
Q8NB16	Q8NB16	Mixed lineage kinase domain-like protein	MLKL	sp|Q8NB1	2	2	2	0	1	1	2	0	1	1	2	0	1	1	2	6.8	54.478	0	14.835	69997000	0	18363000	669110	50964000	0	2472600	686830	1004900
Q8WUY1	Q8WUY1	Protein THEM6	THEM6	sp|Q8WU	3	3	3	0	3	1	2	0	3	1	2	0	3	1	2	17.3	23.865	0	7.6341	69957000	0	51165000	1064500	17728000	0	4149900	948710	855920
Q9UBD5	Q9UBD5	Origin recognition complex subunit 3	ORC3	sp|Q9UBD	2	2	2	0	2	1	2	0	2	1	2	0	2	1	2	3.8	82.253	0	7.8485	69871000	0	10467000	1403400	58000000	0	828030	1291100	2132000
Q99996	Q99996	A-kinase anchor protein 9	AKAP9	sp|Q9999	8	8	8	0	6	0	8	0	6	0	8	0	6	0	8	2.4	452.98	0	11.739	69838000	0	17806000	0	52031000	0	2067900	0	1641600
O15075	O15075	Serine/threonine-protein kinase DCLK1	DCLK1	sp|O1507	2	2	2	0	0	0	2	0	0	0	2	0	0	0	2	5.8	82.223	0	5.3286	69822000	0	0	0	69822000	0	0	0	2871500
P16402	P16402	Histone H1.3	HIST1H1D	sp|P16402	9	2	2	3	8	7	9	0	1	1	2	0	1	1	2	30.8	22.35	0	4.6878	69777000	0	5429000	118910	64229000	0	2502200	331420	366290
Q9BVQ7	Q9BVQ7	Spermatogenesis-associated protein 5-like protei	SPATA5L1	sp|Q9BVQ	3	3	3	1	3	1	2	1	3	1	2	1	3	1	2	7.3	80.709	0	11.659	69673000	102970	24641000	1585500	43343000	354110	785170	2988900	994860
O75419	O75419	Cell division control protein 45 homolog	CDC45	sp|O7541	3	3	3	0	3	0	2	0	3	0	2	0	3	0	2	8.3	65.568	0	12.965	69603000	0	50200000	0	19403000	0	4132100	0	1091000
P23511	P23511	Nuclear transcription factor Y subunit alpha	NFYA	sp|P23511	1	1	1	0	1	0	1	0	1	0	1	0	1	0	1	5.5	36.876	0	15.221	69564000	0	32653000	0	36911000	0	2878300	0	1518000
P49590	P49590	Probable histidine--tRNA ligase, mitochondrial	HARS2	sp|P49590	9	3	3	0	9	2	7	0	3	0	2	0	3	0	2	18.4	56.888	0	8.2924	69479000	0	15713000	0	53766000	0	937850	0	2658400
Q13488	Q13488	V-type proton ATPase 116 kDa subunit a isoform	TCIRG1	sp|Q1348	2	2	2	0	0	0	2	0	0	0	2	0	0	0	2	4.5	92.967	0	18.336	69444000	0	0	0	69444000	0	0	0	2855900
Q96I59	Q96I59	Probable asparagine--tRNA ligase, mitochondrial	NARS2	sp|Q96I59	2	2	2	0	2	0	2	0	2	0	2	0	2	0	2	4.8	54.089	0	7.7383	69422000	0	32679000	0	36742000	0	3020800	0	1370900
P41743	P41743	Protein kinase C iota type	PRKCI	sp|P41743	3	3	3	0	3	0	2	0	3	0	2	0	3	0	2	9.1	68.262	0	6.2322	69356000	0	30252000	0	39105000	0	1554900	0	2719900
O43493	O43493	Trans-Golgi network integral membrane protein	TGOLN2	sp|O4349	3	3	3	0	3	0	3	0	3	0	3	0	3	0	3	9.2	45.879	0	5.1091	68967000	0	10096000	0	58872000	0	786700	0	2524400
P62310	P62310	U6 snRNA-associated Sm-like protein LSm3	LSM3	sp|P62310	2	2	2	1	2	1	2	1	2	1	2	1	2	1	2	19.6	11.845	0.001151	2.684	68822000	231040	32852000	535830	35204000	346130	3405500	432110	750970
Q9BRT8;Q	Q9BRT8;Q	COBW domain-containing protein 1;COBW domai	CBWD1;CB	sp|Q9BRT	4	4	4	0	2	1	4	0	2	1	4	0	2	1	4	13.9	44.068	0	12.225	68803000	0	10626000	2012900	56165000	0	1170000	1802400	1626800
Q01658	Q01658	Protein Dr1	DR1	sp|Q0165	3	3	3	0	3	0	2	0	3	0	2	0	3	0	2	19.3	19.443	0.000485	3.4495	68760000	0	47245000	0	21515000	0	4446000	0	603410
Q8NI60	Q8NI60	Atypical kinase ADCK3, mitochondrial	ADCK3	sp|Q8NI60	2	2	2	0	2	0	1	0	2	0	1	0	2	0	1	4	71.949	0	5.8005	68683000	0	45159000	0	23523000	0	3488100	0	1460100
Q7Z7K0	Q7Z7K0	COX assembly mitochondrial protein homolog	CMC1	sp|Q7Z7K	2	2	2	0	2	1	1	0	2	1	1	0	2	1	1	17.9	12.49	0.001171	2.9682	68666000	0	52161000	3825700	12679000	0	4208300	2960500	0
Q7KZI7	Q7KZI7	Serine/threonine-protein kinase MARK2	MARK2	sp|Q7KZI7	4	4	4	0	3	3	4	0	3	3	4	0	3	3	4	8	87.91	0	9.7305	68647000	0	15298000	7733900	45615000	0	1477200	5341200	1603200
Q9H3Z4	Q9H3Z4	DnaJ homolog subfamily C member 5	DNAJC5	sp|Q9H3Z	2	2	2	0	2	0	2	0	2	0	2	0	2	0	2	7.6	22.149	0	8.8914	68482000	0	15099000	0	53383000	0	1565800	0	1960600
Q3V6T2	Q3V6T2	Girdin	CCDC88A	sp|Q3V6T	8	8	8	0	1	1	8	0	1	1	8	0	1	1	8	5.7	216.04	0	16.826	68424000	0	3571200	933960	63919000	0	301050	425080	2845000
Q2M1P5	Q2M1P5	Kinesin-like protein KIF7	KIF7	sp|Q2M1P	5	5	4	0	2	1	5	0	2	1	5	0	2	1	4	5.1	150.59	0	18.144	68399000	0	4146200	2986600	61266000	0	1362600	1696700	1832800
P49815	P49815	Tuberin	TSC2	sp|P49815	4	4	4	0	2	0	4	0	2	0	4	0	2	0	4	3.7	200.61	0	5.9077	68334000	0	13305000	0	55029000	0	1922500	0	1513500
Q9BTA9	Q9BTA9	WW domain-containing adapter protein with coil	WAC	sp|Q9BTA	3	3	3	0	3	2	2	0	3	2	2	0	3	2	2	9.9	70.723	0	9.7897	68332000	0	24389000	4040600	39903000	0	2182500	2630700	1692900
Q9BYG3	Q9BYG3	MKI67 FHA domain-interacting nucleolar phosph	NIFK	sp|Q9BYG	5	5	5	0	4	2	3	0	4	2	3	0	4	2	3	21.2	34.222	0	12.093	68258000	0	35409000	5956600	26893000	0	2581900	4182100	1466000
Q96HY7	Q96HY7	Probable 2-oxoglutarate dehydrogenase E1 comp	DHTKD1	sp|Q96HY	3	3	3	0	3	0	1	0	3	0	1	0	3	0	1	6	103.08	0	5.0346	68155000	0	65401000	0	2754200	0	5188600	0	689630
Q9UI42	Q9UI42	Carboxypeptidase A4	CPA4	sp|Q9UI42	3	3	3	0	0	1	3	0	0	1	3	0	0	1	3	11.4	47.351	0	13.251	68129000	0	0	6365700	61763000	0	0	6574200	243500
Q8NBJ4	Q8NBJ4	Golgi membrane protein 1	GOLM1	sp|Q8NBJ	5	5	5	0	5	0	2	0	5	0	2	0	5	0	2	16.7	45.333	0	12.615	68001000	0	51129000	0	16872000	0	3383000	0	1817800
Q13232	Q13232	Nucleoside diphosphate kinase 3	NME3	sp|Q1323	4	4	4	0	4	0	0	0	4	0	0	0	4	0	0	33.1	19.015	0	16.818	67936000	0	67936000	0	0	0	5988500	0	0
P24821	P24821	Tenascin	TNC	sp|P24821	3	3	3	0	0	0	3	0	0	0	3	0	0	0	3	2.3	240.85	0	20.425	67667000	0	0	0	67667000	0	0	0	2782900
Q9BZD4	Q9BZD4	Kinetochore protein Nuf2	NUF2	sp|Q9BZD	1	1	1	0	0	0	1	0	0	0	1	0	0	0	1	4.3	54.303	0	12.366	67452000	0	0	0	67452000	0	0	0	2774000
Q8IUR6	Q8IUR6	CREB3 regulatory factor	CREBRF	sp|Q8IUR	1	1	1	0	1	1	1	0	1	1	1	0	1	1	1	1.4	72.148	0.003283	2.057	67438000	0	17779000	35565000	14093000	0	1567200	23900000	579590
Q6YHK3	Q6YHK3	CD109 antigen	CD109	sp|Q6YHK	4	4	4	0	0	0	4	0	0	0	4	0	0	0	4	3.7	161.69	0	7.4452	67365000	0	0	0	67365000	0	0	0	2770400
P04233	P04233	HLA class II histocompatibility antigen gamma cha	CD74	sp|P04233	2	2	2	0	0	0	2	0	0	0	2	0	0	0	2	7.8	33.515	0.001149	2.6743	67339000	0	0	0	67339000	0	0	0	2769400
Q9P2D3	Q9P2D3	HEAT repeat-containing protein 5B	HEATR5B	sp|Q9P2D	2	2	2	0	1	0	2	0	1	0	2	0	1	0	2	2.3	224.3	0	14.748	67338000	0	6566200	0	60771000	0	748020	0	2330000
Q96BW9	Q96BW9	Phosphatidate cytidylyltransferase, mitochondria	TAMM41	sp|Q96BW	2	2	2	0	2	1	2	0	2	1	2	0	2	1	2	6.4	51.066	0	9.6482	67225000	0	27274000	4785400	35166000	0	1644000	4425900	996330
Q9Y2L5	Q9Y2L5	Trafficking protein particle complex subunit 8	TRAPPC8	sp|Q9Y2L5	4	4	4	0	2	0	4	0	2	0	4	0	2	0	4	3.3	161	0	12.823	67194000	0	13366000	0	53828000	0	1310400	0	2081500
Q5C9Z4	Q5C9Z4	Nucleolar MIF4G domain-containing protein 1	NOM1	sp|Q5C9Z	2	2	2	0	2	2	2	0	2	2	2	0	2	2	2	3.3	96.256	0	5.5154	67136000	0	17027000	7880500	42229000	0	1463800	4995900	2073600
O14874	O14874	[3-methyl-2-oxobutanoate dehydrogenase [lipoar	BCKDK	sp|O1487	3	3	3	0	2	2	1	0	2	2	1	0	2	2	1	9.7	46.36	0	5.8666	67094000	0	35015000	5508500	26570000	0	3086500	0	0
Q9NWB7	Q9NWB7	Intraflagellar transport protein 57 homolog	IFT57	sp|Q9NWE	2	2	2	0	0	1	2	0	0	1	2	0	0	1	2	8.9	49.108	0	11.906	66833000	0	0	438620	66394000	0	0	1588700	1436500
P78357	P78357	Contactin-associated protein 1	CNTNAP1	sp|P78357	4	4	4	0	0	2	4	0	0	2	4	0	0	2	4	5	156.26	0	13.2	66707000	0	0	3783500	62923000	0	0	3345100	1785200
Q8WVT3	Q8WVT3	Trafficking protein particle complex subunit 12	TRAPPC12	sp|Q8WVT	4	4	4	0	1	0	4	0	1	0	4	0	1	0	4	9.3	79.374	0	10.109	66667000	0	2153000	0	64514000	0	381760	0	2461200
Q8NBT2	Q8NBT2	Kinetochore protein Spc24	SPC24	sp|Q8NBT	3	3	3	0	3	0	3	0	3	0	3	0	3	0	3	26.9	22.478	0	27.827	66548000	0	10740000	0	55808000	0	1138900	0	2103000
Q5HYK3	Q5HYK3	2-methoxy-6-polyprenyl-1,4-benzoquinol methyla	COQ5	sp|Q5HYK	4	4	4	0	4	0	4	0	4	0	4	0	4	0	4	16.2	37.14	0	11.643	66527000	0	50982000	0	15545000	0	4282000	0	851270
P11169;Q8	P11169;Q8	Solute carrier family 2, facilitated glucose transpo	SLC2A3;SL	sp|P11169	1	1	1	0	0	0	1	0	0	0	1	0	0	0	1	1.8	53.924	0	3.9062	66437000	0	0	0	66437000	0	0	0	2380600
Q86VR2	Q86VR2	Protein FAM134C	FAM134C	sp|Q86VR	2	2	2	0	2	0	2	0	2	0	2	0	2	0	2	10.5	51.396	0	6.5423	66419000	0	20482000	0	45938000	0	1753800	0	1940900
Q5RI15	Q5RI15	Cytochrome c oxidase protein 20 homolog	COX20	sp|Q5RI15	1	1	1	0	1	1	1	0	1	1	1	0	1	1	1	10.2	13.291	0.001175	3.0293	66343000	0	3674600	878450	61790000	0	0	0	2541100
P30154	P30154	Serine/threonine-protein phosphatase 2A 65 kDa	PPP2R1B	sp|P30154	7	2	2	0	5	3	7	0	2	0	2	0	2	0	2	15.8	66.213	0	11.793	66107000	0	17220000	0	48887000	0	1519300	0	2009200
Q8NB46	Q8NB46	Serine/threonine-protein phosphatase 6 regulato	ANKRD52	sp|Q8NB4	2	2	2	0	1	0	2	0	1	0	2	0	1	0	2	2.8	115.08	0	5.3547	66015000	0	2187800	0	63827000	0	360340	0	2457500
Q96MW1	Q96MW1	Coiled-coil domain-containing protein 43	CCDC43	sp|Q96MV	3	3	3	0	2	0	2	0	2	0	2	0	2	0	2	19.6	25.248	0	9.0647	65993000	0	26601000	0	39392000	0	3044600	0	920250
Q5T5Y3	Q5T5Y3	Calmodulin-regulated spectrin-associated protein	CAMSAP1	sp|Q5T5Y	4	4	3	1	2	1	3	1	2	1	3	1	1	1	3	4.6	177.97	0	7.0964	65891000	15436000	7048300	1853900	41553000	0	1029900	1752600	793500
Q9C0F1	Q9C0F1	Centrosomal protein of 44 kDa	CEP44	sp|Q9C0F1	2	2	2	0	2	2	0	0	2	2	0	0	2	2	0	10	44.139	0	5.7357	65754000	0	60606000	5148300	0	0	1978100	6823900	0

Q68CQ7	Q68CQ7	Glycosyltransferase 8 domain-containing protein	GLT8D1	sp|Q68CQ	3	3	3	0	1	0	3	0	1	0	3	0	1	0	3	10.8	41.935	0	10.226	65621000	0	6313900	0	59307000	0	0	0	2439100
Q9Y3R5	Q9Y3R5	Protein dopey-2	DOPEY2	sp|Q9Y3R5	3	3	3	0	2	0	3	0	2	0	3	0	2	0	3	1.4	258.23	0	4.0196	65598000	0	12712000	0	52886000	0	1242800	0	2052700
Q86TP1	Q86TP1	Protein prune homolog	PRUNE	sp|Q86TP1	4	4	4	0	4	0	1	0	4	0	1	0	4	0	1	11.9	50.199	0	12.266	65360000	0	58582000	0	6778100	0	4775500	0	667230
P15291	P15291	Beta-1,4-galactosyltransferase 1;Lactose synthase	B4GALT1	sp|P15291	1	1	1	0	0	0	1	0	0	0	1	0	0	0	1	5	43.92	0	9.3908	65290000	0	0	0	65290000	0	0	0	2685100
Q9P013	Q9P013	Spliceosome-associated protein CWC15 homolog	CWC15	sp|Q9P013	2	2	2	0	2	0	1	0	2	0	1	0	2	0	1	15.7	26.624	0	5.4814	65233000	0	50178000	0	15055000	0	3182500	0	1859700
Q13546	Q13546	Receptor-interacting serine/threonine-protein kir	RIPK1	sp|Q13546	4	4	4	0	4	0	2	0	4	0	2	0	4	0	2	7.6	75.93	0	8.0752	65181000	0	40089000	0	25092000	0	2442200	0	2123600
P29317	P29317	Ephrin type-A receptor 2	EPHA2	sp|P29317	4	4	4	0	0	0	4	0	0	0	4	0	0	0	4	5	108.27	0	8.672	65029000	0	0	0	65029000	0	0	0	2674400
P52735	P52735	Guanine nucleotide exchange factor VAV2	VAV2	sp|P52735	3	3	3	0	2	0	3	0	2	0	3	0	2	0	3	5.4	101.29	0	11.255	64982000	0	10984000	0	53998000	0	1437000	0	1751900
Q92845	Q92845	Kinesin-associated protein 3	KIFAP3	sp|Q92845	1	1	1	0	1	0	1	0	1	0	1	0	1	0	1	2.5	91.204	0	12.411	64979000	0	2904200	0	62075000	0	438700	0	2370200
Q969T3	Q969T3	Sorting nexin-21	SNX21	sp|Q969T3	3	3	3	0	0	1	3	0	0	1	3	0	0	1	3	16.1	41.365	0	9.6346	64898000	0	0	1042500	63856000	0	0	937370	2389300
Q8IZ73	Q8IZ73	RNA pseudouridylate synthase domain-containing	RPUSD2	sp|Q8IZ73	2	2	2	0	2	1	2	0	2	1	2	0	2	1	2	4.6	61.311	0	6.6737	64827000	0	19085000	1375500	44366000	0	1678500	0	1828500
Q15052	Q15052	Rho guanine nucleotide exchange factor 6	ARHGEF6	sp|Q15052	4	3	3	0	2	1	4	0	1	1	3	0	1	1	3	8.4	87.498	0	4.7199	64811000	0	4010800	2367400	58433000	0	0	0	2403100
Q9UJ41	Q9UJ41	Rab5 GDP/GTP exchange factor	RABGEF1	sp|Q9UJ41	4	4	4	0	4	0	3	0	4	0	3	0	4	0	3	11.8	56.89	0	10.408	64695000	0	17819000	0	46876000	0	1459200	0	2039300
Q6NUM9	Q6NUM9	All-trans-retinol 13,14-reductase	RETSAT	sp|Q6NUM	1	1	1	0	0	0	1	0	0	0	1	0	0	0	1	2	66.819	0.000488	3.5135	64656000	0	0	0	64656000	0	0	0	2659000
P51808	P51808	Dynein light chain Tctex-type 3	DYNLT3	sp|P51808	1	1	1	0	0	0	1	0	0	0	1	0	0	0	1	29.3	13.062	0	6.3405	64319000	0	0	0	64319000	0	0	0	2645200
Q15067	Q15067	Peroxisomal acyl-coenzyme A oxidase 1	ACOX1	sp|Q15067	4	4	4	0	4	0	1	0	4	0	1	0	4	0	1	9.4	74.423	0	22.395	64215000	0	62141000	0	2074000	0	4658500	0	904480
Q16566	Q16566	Calcium/calmodulin-dependent protein kinase ty	CAMK4	sp|Q16566	3	3	3	0	1	1	3	0	1	1	3	0	1	1	3	9.7	51.925	0	9.5085	64102000	0	3188000	2322500	58592000	0	652600	1443300	2155400
Q99442	Q99442	Translocation protein SEC62	SEC62	sp|Q99442	3	3	3	0	1	2	3	0	1	2	3	0	1	2	3	7	45.861	0	5.1132	63872000	0	5775500	2715700	55381000	0	1271600	1634400	1705600
Q9NRW3	Q9NRW3	DNA dC->dU-editing enzyme APOBEC-3C	APOBEC3C	sp|Q9NRW	2	2	2	0	0	2	2	0	0	2	2	0	0	2	2	16.3	22.826	0.001169	2.9376	63858000	0	0	6301700	57556000	0	0	4170100	2431600
Q9BYJ9	Q9BYJ9	YTH domain-containing family protein 1	YTHDF1	sp|Q9BYJ9	6	2	2	1	4	4	6	1	2	2	2	1	2	2	2	17.5	60.873	0	5.6419	63845000	2421700	15174000	6416600	39833000	3043900	1906400	3411000	1348000
Q96SY0	Q96SY0	von Willebrand factor A domain-containing prote	VWA9	sp|Q96SY0	3	3	3	0	2	0	3	0	2	0	3	0	2	0	3	10	57.47	0	10.148	63783000	0	15554000	0	48228000	0	1420700	0	1933800
Q14573;Q:	Q14573	Inositol 1,4,5-trisphosphate receptor type 3	ITPR3	sp|Q14573	6	4	4	0	4	4	6	0	2	3	4	0	2	3	4	2.7	304.1	0	4.5333	63540000	0	14624000	2766900	46149000	0	2369100	1013400	1663900
P49069	P49069	Calcium signal-modulating cyclophilin ligand	CAMLG	sp|P49069	3	3	3	0	3	0	3	0	3	0	3	0	3	0	3	23.3	32.952	0	12.444	63477000	0	25020000	0	38457000	0	2309800	0	1477200
Q96EC8	Q96EC8	Protein YIPF6	YIPF6	sp|Q96EC8	2	2	2	0	2	1	2	0	2	1	2	0	2	1	2	11.9	26.256	0	6.9974	63437000	0	11926000	2360900	49150000	0	931240	1938800	1789100
Q9Y2Z9	Q9Y2Z9	Ubiquinone biosynthesis monooxygenase COQ6,	COQ6	sp|Q9Y2Z9	5	5	5	0	5	1	2	0	5	1	2	0	5	1	2	12.4	50.869	0	9.3709	63278000	0	46072000	1510500	15695000	0	3988000	0	718710
Q9UNX4	Q9UNX4	WD repeat-containing protein 3	WDR3	sp|Q9UNX	3	3	3	0	3	0	3	0	3	0	3	0	3	0	3	4	106.1	0	11.568	63250000	0	29988000	0	33263000	0	2724100	0	1287200
Q96GX9	Q96GX9	Methylthioribulose-1-phosphate dehydratase	APIP	sp|Q96GX9	2	2	2	0	2	0	1	0	2	0	1	0	2	0	1	9.1	27.125	0	5.296	63180000	0	32391000	0	30789000	0	2352800	0	1768700
Q8TBQ9	Q8TBQ9	Protein kish-A	TMEM167A	sp|Q8TBQ9	1	1	1	0	1	0	1	0	1	0	1	0	1	0	1	12.5	8.0598	0.003484	1.9777	63044000	0	17808000	0	45236000	0	1569700	0	1860400
Q7RTV0	Q7RTV0	PHD finger-like domain-containing protein 5A	PHF5A	sp|Q7RTV0	2	2	2	0	2	1	2	0	2	1	2	0	2	1	2	15.5	12.405	0	4.2142	62919000	0	34972000	1723700	26224000	0	2787700	1556100	912760
Q8IY37	Q8IY37	Probable ATP-dependent RNA helicase DHX37	DHX37	sp|Q8IY37	3	3	3	0	2	1	3	0	2	1	3	0	2	1	3	4	129.54	0	6.6329	62890000	0	30697000	2189500	30004000	0	736230	4428600	246290
Q7Z6M1	Q7Z6M1	Rab9 effector protein with kelch motifs	RABEPK	sp|Q7Z6M	4	4	4	0	3	0	3	0	3	0	3	0	3	0	3	19.1	40.564	0	6.9261	62886000	0	30149000	0	32737000	0	2806700	0	1197300
Q9BV79	Q9BV79	Trans-2-enoyl-CoA reductase, mitochondrial	MECR	sp|Q9BV79	2	2	2	0	2	0	2	0	2	0	2	0	2	0	2	5.6	40.461	0	4.9639	62878000	0	19597000	0	43281000	0	1922100	0	1585300
Q9BPY3	Q9BPY3	Protein FAM118B	FAM118B	sp|Q9BPY3	2	2	2	0	2	0	0	0	2	0	0	0	2	0	0	8	39.499	0	7.7007	62845000	0	62845000	0	0	0	5539700	0	0
O75152	O75152	Zinc finger CCCH domain-containing protein 11A	ZC3H11A	sp|O75152	5	5	4	2	5	0	2	2	5	0	2	2	4	0	1	10.5	89.13	0	8.5671	62657000	6302900	50295000	0	6059100	0	4118600	0	564040
Q6VMQ6	Q6VMQ6	Activating transcription factor 7-interacting prote	ATF7IP	sp|Q6VMQ	3	3	3	0	1	1	3	0	1	1	3	0	1	1	3	4.8	136.39	0	5.2048	62654000	0	9398900	4415400	48840000	0	1056900	3785100	962130
Q9UHQ4	Q9UHQ4	B-cell receptor-associated protein 29	BCAP29	sp|Q9UHQ	2	2	2	0	1	1	2	0	1	1	2	0	1	1	2	10	28.32	0	6.9714	62420000	0	2605000	935000	58880000	0	559940	640350	2079100
O95379	O95379	Tumor necrosis factor alpha-induced protein 8	TNFAIP8	sp|O95379	2	2	2	0	1	0	2	0	1	0	2	0	1	0	2	17.2	23.003	0	15.828	62230000	0	3305600	0	58925000	0	388970	0	2325700
O75691	O75691	Small subunit processome component 20 homolo	UTP20	sp|O75691	5	5	5	0	1	3	5	0	1	3	5	0	1	3	5	2.6	318.38	0	6.6831	62189000	0	2229200	4562600	55397000	0	1339000	2407500	1794200
Q7LGA3	Q7LGA3	Heparan sulfate 2-O-sulfotransferase 1	HS2ST1	sp|Q7LGA3	3	3	3	0	3	1	1	0	3	1	1	0	3	1	1	12.9	41.881	0	6.9986	61930000	0	46765000	2094500	13070000	0	3350400	1405800	1311100
Q9H900	Q9H900	Protein zwilch homolog	ZWILCH	sp|Q9H900	2	2	2	0	2	0	2	0	2	0	2	0	2	0	2	3.9	67.213	0	3.9908	61880000	0	24524000	0	37356000	0	2337600	0	1360500
Q9C035	Q9C035	Tripartite motif-containing protein 5	TRIM5	sp|Q9C035	2	2	2	0	1	0	2	0	1	0	2	0	1	0	2	6.5	56.338	0	18.583	61801000	0	18590000	0	43211000	0	1738100	0	1677700
P61421	P61421	V-type proton ATPase subunit d 1	ATP6V0D1	sp|P61421	1	1	1	0	1	1	1	0	1	1	1	0	1	1	1	3.1	40.329	0.00474	1.8148	61538000	0	9205500	1643100	50689000	0	819780	956260	2224200
O15066;O:	O15066	Kinesin-like protein KIF3B;Kinesin-like protein KIF	KIF3B	sp|O15066	6	5	5	0	2	1	6	0	1	1	5	0	1	1	5	10.8	85.124	0	6.6774	61433000	0	1637700	703530	59092000	0	579760	1049200	1418400
P19447	P19447	TFIIH basal transcription factor complex helicase	ERCC3	sp|P19447	3	3	3	0	3	2	2	0	3	2	2	0	3	2	2	7.9	89.277	0	4.276	61417000	0	38506000	3471500	19440000	0	1724000	3681500	1121100
Q70CQ2	Q70CQ2	Ubiquitin carboxyl-terminal hydrolase 34	USP34	sp|Q70CQ	2	2	2	0	2	1	2	0	2	1	2	0	2	1	2	0.7	404.23	0	3.922	61413000	0	18792000	1106200	41515000	0	0	0	1707300
Q53RT3	Q53RT3	Retroviral-like aspartic protease 1	ASPRV1	sp|Q53RT3	2	2	2	0	0	2	0	0	0	2	0	0	0	2	0	13.1	36.991	0	44.377	61342000	0	0	61342000	0	0	0	41222000	0
Q9Y282	Q9Y282	Endoplasmic reticulum-Golgi intermediate compa	ERGIC3	sp|Q9Y282	3	3	3	0	3	0	3	0	3	0	3	0	3	0	3	7.3	43.222	0	5.3358	61210000	0	11589000	0	49621000	0	1039300	0	2022900
Q86VI3	Q86VI3	Ras GTPase-activating-like protein IQGAP3	IQGAP3	sp|Q86VI3	5	3	3	0	2	1	4	0	0	0	3	0	0	0	3	4.8	184.7	0	13.593	61163000	0	0	0	61163000	0	0	0	2515300
Q5EBL4	Q5EBL4	RILP-like protein 1	RILPL1	sp|Q5EBL4	3	3	3	0	0	0	3	0	0	0	3	0	0	0	3	11.7	47.107	0	13.527	60913000	0	0	0	60913000	0	0	0	2505100
Q9GZS1	Q9GZS1	DNA-directed RNA polymerase I subunit RPA49	POLR1E	sp|Q9GZS1	3	3	3	0	2	1	3	0	2	1	3	0	2	1	3	7.4	47.26	0	6.8046	60779000	0	8623700	3257000	48898000	0	1010400	0	1760700
Q9NZC9	Q9NZC9	SWI/SNF-related matrix-associated actin-depende	SMARCAL1	sp|Q9NZC9	4	4	4	1	2	1	4	1	2	1	4	1	2	1	4	6	105.94	0	5.9709	60703000	3572400	7952800	1037600	48140000	3404300	914420	1743600	888140
Q96BM9	Q96BM9	ADP-ribosylation factor-like protein 8A	ARL8A	sp|Q96BM	4	1	1	0	3	1	4	0	1	0	1	0	1	0	1	31.2	21.416	0.003493	2.024	60648000	0	0	0	60648000	0	0	0	2494200
Q06587;Q	Q06587	E3 ubiquitin-protein ligase RING1	RING1	sp|Q06587	3	3	3	0	3	1	3	0	3	1	3	0	3	1	3	10.3	42.429	0	8.7918	60640000	0	38978000	5413100	16249000	0	3542900	3162100	1036800
Q8WUA7	Q8WUA7	TBC1 domain family member 22A	TBC1D22A	sp|Q8WUA	4	4	4	0	0	0	4	0	0	0	4	0	0	0	4	13	59.12	0	9.1663	60616000	0	0	0	60616000	0	0	0	2492900
Q9UQ53	Q9UQ53	Alpha-1,3-mannosyl-glycoprotein 4-beta-N-acetyl	MGAT4B	sp|Q9UQ5	2	2	2	0	0	0	2	0	0	0	2	0	0	0	2	5.7	63.198	0.001137	2.5176	60601000	0	0	0	60601000	0	0	0	2492300
P49754	P49754	Vacuolar protein sorting-associated protein 41 ho	VPS41	sp|P49754	2	2	2	0	0	0	2	0	0	0	2	0	0	0	2	2.2	98.565	0.001168	2.9345	60537000	0	0	0	60537000	0	0	0	2489600
Q9NQ55	Q9NQ55	Suppressor of SWI4 1 homolog	PPAN	sp|Q9NQ5	6	6	6	1	3	3	4	1	3	3	4	1	3	3	4	19.5	53.193	0	11.994	60524000	0	9613000	4077100	46834000	0	653860	3655400	1204000
P35813	P35813	Protein phosphatase 1A	PPM1A	sp|P35813	2	2	1	1	1	0	2	1	1	0	2	1	0	0	1	7.9	42.447	0	7.8961	60479000	627010	12825000	0	47027000	901000	1467800	0	1322700
Q6PID6	Q6PID6	Tetratricopeptide repeat protein 33	TTC33	sp|Q6PID6	2	2	2	0	2	0	1	0	2	0	1	0	2	0	1	17.9	29.411	0	6.1194	60392000	0	49936000	0	10456000	0	4007400	0	824400
Q9H2K8;Q	Q9H2K8	Serine/threonine-protein kinase TAO3	TAOK3	sp|Q9H2K8	5	5	4	0	1	1	5	0	1	1	5	0	0	1	4	6.9	105.4	0	9.7174	60317000	0	6600900	1609900	52106000	0	818920	1211000	1776700
Q9Y4C8	Q9Y4C8	Probable RNA-binding protein 19	RBM19	sp|Q9Y4C8	4	4	4	0	0	3	4	0	0	3	4	0	0	3	4	5.1	107.33	0	12.073	60235000	0	0	8255100	51980000	0	0	6197800	1487300
Q8N392	Q8N392	Rho GTPase-activating protein 18	ARHGAP18	sp|Q8N392	6	6	6	0	2	0	6	0	2	0	6	0	2	0	6	12.4	74.976	0	15.757	60151000	0	3761100	0	56390000	0	659180	0	1991400
Q96TC7	Q96TC7	Regulator of microtubule dynamics protein 3	RMDN3	sp|Q96TC7	2	2	2	0	2	0	2	0	2	0	2	0	2	0	2	7.2	52.118	0	6.6433	60147000	0	31510000	0	28637000	0	2804100	0	1151200
Q8WWI5	Q8WWI5	Choline transporter-like protein 1	SLC44A1	sp|Q8WW	2	2	2	0	2	0	2	0	2	0	2	0	2	0	2	4.7	73.301	0.001169	2.9443	60130000	0	15678000	0	44452000	0	0	0	1828100
Q7L7V1	Q7L7V1	Putative pre-mRNA-splicing factor ATP-dependen	DHX32	sp|Q7L7V1	1	1	1	0	0	0	1	0	0	0	1	0	0	0	1	1.6	84.418	0.000246	3.6309	60122000	0	0	0	60122000	0	0	0	2472600
Q9BY41	Q9BY41	Histone deacetylase 8	HDAC8	sp|Q9BY41	1	1	1	0	1	0	0	0	1	0	0	0	1	0	0	5	41.757	0	6.0226	60102000	0	60102000	0	0	0	5297900	0	0
Q9UGJ1	Q9UGJ1	Gamma-tubulin complex component 4	TUBGCP4	sp|Q9UGJ1	1	1	1	0	1	0	1	0	1	0	1	0	1	0	1	2.4	76.088	0.000249	3.7474	60093000	0	5986200	0	54107000	0	0	0	2225200
Q9H330	Q9H330	Transmembrane protein 245	TMEM245	sp|Q9H330	2	2	2	0	1	0	2	0	1	0	2	0	1	0	2	3.2	97.356	0	6.7678	60093000	0	15171000	0	44922000	0	1693700	0	1491000
Q14012;Q8	Q14012	Calcium/calmodulin-dependent protein kinase ty	CAMK1	sp|Q14012	4	4	4	0	3	0	3	0	3	0	3	0	3	0	3	13.2	41.337	0	4.0428	60062000	0	27703000	0	32359000	0	2557700	0	1215000
P05114	P05114	Non-histone chromosomal protein HMG-14	HMGN1	sp|P05114	1	1	1	0	1	0	1	0	1	0	1	0	1	0	1	34	10.659	0.001128	2.4179	60032000	0	33262000	0	26770000	0	1640300	0	680510
O15111	O15111	Inhibitor of nuclear factor kappa-B kinase subunit	CHUK	sp|O15111	4	3	3	0	4	0	4	0	3	0	3	0	3	0	3	6	84.639	0	5.6713	59892000	0	23671000	0	36221000	0	2020600	0	1555500
Q9Y5Z4	Q9Y5Z4	Heme-binding protein 2	HEBP2	sp|Q9Y5Z4	4	4	4	0	4	0	2	0	4	0	2	0	4	0	2	22.4	22.875	0	11.624	59825000	0	44730000	0	15095000	0	3462300	0	1101400
Q9Y4B5	Q9Y4B5	Microtubule cross-linking factor 1	MTCL1	sp|Q9Y4B5	14	14	13	0	1	14	3	0	1	14	3	0	1	13	2	8.8	209.52	0	17.624	59784000	0	1926600	47635000	10222000	0	110980	32275000	214560
Q9Y291	Q9Y291	28S ribosomal protein S33, mitochondrial	MRPS33	sp|Q9Y291	2	2	2	1	2	1	2	1	2	1	2	1	2	1	2	19.8	12.629	0.006011	1.7695	59758000	393880	34078000	653890	24632000	997460	1819800	1053500	979420
Q02880	Q02880	DNA topoisomerase 2-beta	TOP2B	sp|Q02880	6	2	2	1	5	2	4	0	2	1	2	0	2	1	2	4.4	183.26	0.000245	3.5905	59563000	0	32753000	2650300	24161000	0	2907000	1653800	1100900
Q7L266	Q7L266	Isoaspartyl peptidase/L-asparaginase;Isoaspartyl	ASRGL1	sp|Q7L266	3	3	3	0	3	0	0	0	3	0	0	0	3	0	0	15.3	32.054	0	11.046	59478000	0	59478000	0	0	0	5242900	0	0
Q5T160	Q5T160	Probable arginine--tRNA ligase, mitochondrial	RARS2	sp|Q5T160	1	1	1	0	1	0	1	0	1	0	1	0	1	0	1	2.1	65.505	0.000248	3.6892	59328000	0	10874000	0	48454000	0	958550	0	1992700
Q9NUW8	Q9NUW8	Tyrosyl-DNA phosphodiesterase 1	TDP1	sp|Q9NUW	2	2	2	0	1	0	2	0	1	0	2	0	1	0	2	4.6	68.42	0	5.3113	59213000	0	7102000	0	52111000	0	1268600	0	1500500
Q9Y587	Q9Y587	AP-4 complex subunit sigma-1	AP4S1	sp|Q9Y587	1	1	1	0	1	0	1	0	1	0	1	0	1	0	1	6.9	17.005	0.000943	3.0787	59033000	0	6803600	0	52230000	0	599730	0	2148000
P57081	P57081	tRNA (guanine-N(7)-)-methyltransferase non-cata	WDR4	sp|P57081	3	3	3	0	2	0	2	0	2	0	2	0	2	0	2	10.2	45.489	0.000719	3.3147	59014000	0	31327000	0	27687000	0	2545400	0	1354700
L0R819	L0R819		ASNSD1	sp|L0R819	3	3	3	0	3	3	2	0	3	3	2	0	3	3	2	49	11.25	0	10.958	59013000	0	22537000	5516600	30959000	0	1991000	3200600	1470000
Q9NXE4	Q9NXE4	Sphingomyelin phosphodiesterase 4	SMPD4	sp|Q9NXE4	3	3	3	0	3	0	3	0	3	0	3	0	3	0	3	4.4	97.809	0	6.0139	58946000	0	15502000	0	43444000	0	1331500	0	1821700
Q7Z7E8	Q7Z7E8	Ubiquitin-conjugating enzyme E2 Q1	UBE2Q1	sp|Q7Z7E8	2	2	2	0	2	1	2	0	2	1	2	0	2	1	2	5.5	46.126	0	5.7218	58872000	0	21507000	1799200	35566000	0	1921000	0	1437500
Q9P2B4	Q9P2B4	CTTNBP2 N-terminal-like protein	CTTNBP2NL	sp|Q9P2B4	4	4	4	1	0	0	4	1	0	0	4	1	0	0	4	10.5	70.157	0	9.2149	58474000	3633000	0	0	54841000	4959000	0	0	929320
Q9NRY4	Q9NRY4	Rho GTPase-activating protein 35	ARHGAP35	sp|Q9NRY4	4	4	4	0	2	0	4	0	2	0	4	0	2	0	4	3.1	170.51	0	13.03	58469000	0	10510000	0	47958000	0	1119700	0	1779100
Q8IWA4	Q8IWA4	Mitofusin-1	MFN1	sp|Q8IWA4	3	2	2	0	1	0	3	0	0	0	2	0	0	0	2	4.3	84.159	0	7.5719	58395000	0	0	0	58395000	0	0	0	2401500
Q9HC98;Q	Q9HC98;Q	Serine/threonine-protein kinase Nek6;Serine/thr	NEK6;NEK7	sp|Q9HC98	3	3	3	0	0	1	3	0	0	1	3	0	0	1	3	15	35.714	0	5.8129	58343000	0	0	2556700	55786000	0	0	2066900	1945500
Q9NZW5	Q9NZW5	MAGUK p55 subfamily member 6	MPP6	sp|Q9NZW	2	2	2	0	2	0	2	0	2	0	2	0	2	0	2	3.5	61.116	0.001132	2.4743	58301000	0	35066000	0	23236000	0	0	0	955590
O95677	O95677	Eyes absent homolog 4	EYA4	sp|O95677	1	1	1	0	1	0	1	0	1	0	1	0	1	0	1	2.5	69.504	0.001144	2.5976	58229000	0	17181000	0	41048000	0	1514500	0	1688100

Q92667	Q92667	A-kinase anchor protein 1, mitochondrial	AKAP1	sp|Q92667	6	6	6	0	6	0	1	0	6	0	1	0	6	0	1	11.6	97.34	0	9.0885	58215000	0	45202000	0	13012000	0	3984500	0	535140
O75391	O75391	Sperm-associated antigen 7	SPAG7	sp|O75391	3	3	3	0	3	0	0	0	3	0	0	0	3	0	0	19.8	26.034	0	9.9929	58208000	0	58208000	0	0	0	5131000	0	0
Q9Y4K3	Q9Y4K3	TNF receptor-associated factor 6	TRAF6	sp|Q9Y4K3	4	4	4	0	2	0	4	0	2	0	4	0	2	0	4	10.9	59.573	0	9.4981	58195000	0	5746400	0	52449000	0	1317700	0	1345800
Q8IVM0	Q8IVM0	Coiled-coil domain-containing protein 50	CCDC50	sp|Q8IVM0	4	4	4	0	1	0	4	0	1	0	4	0	1	0	4	22.9	35.822	0	21.942	58085000	0	2457900	0	55627000	0	0	0	2287700
O60341	O60341	Lysine-specific histone demethylase 1A	KDM1A	sp|O60341	5	5	5	1	4	0	3	1	4	0	3	1	4	0	3	9.5	92.902	0	9.5167	58035000	251520	24033000	0	33750000	586140	2073400	0	1098500
Q9UKZ1	Q9UKZ1	CCR4-NOT transcription complex subunit 11	CNOT11	sp|Q9UKZ1	2	2	2	0	1	0	2	0	1	0	2	0	1	0	2	6.3	55.215	0	5.7024	58026000	0	14435000	0	43590000	0	1427000	0	1638200
Q14728	Q14728	Major facilitator superfamily domain-containing p	MFSD10	sp|Q14728	1	1	1	0	1	1	1	0	1	1	1	0	1	1	1	2.9	48.339	0	3.8513	57950000	0	8698000	1461800	47791000	0	766720	982350	1965400
Q92791	Q92791	Synaptonemal complex protein SC65	LEPREL4	sp|Q92791	2	2	2	0	1	0	2	0	1	0	2	0	1	0	2	5	50.381	0.000487	3.4825	57913000	0	5454000	0	52459000	0	0	0	2157400
Q13823	Q13823	Nucleolar GTP-binding protein 2	GNL2	sp|Q13823	3	3	3	0	2	2	1	0	2	2	1	0	2	2	1	5.6	83.654	0	6.7372	57876000	0	40975000	4147900	12753000	0	3611900	0	0
Q15369	Q15369	Transcription elongation factor B polypeptide 1	TCEB1	sp|Q15369	1	1	1	0	1	0	1	0	1	0	1	0	1	0	1	17.9	12.473	0	11.223	57777000	0	13640000	0	44137000	0	1202400	0	1815200
Q6ZRS2	Q6ZRS2	Helicase SRCAP	SRCAP	sp|Q6ZRS2	2	2	2	0	2	0	1	0	2	0	1	0	2	0	1	0.6	343.55	0.001571	2.35	57692000	0	50326000	0	7366000	0	2296100	0	2443000
Q96DE0;Q	Q96DE0	U8 snoRNA-decapping enzyme	NUDT16	sp|Q96DE0	3	3	3	0	3	0	2	0	3	0	2	0	3	0	2	22.6	21.273	0	11.077	57519000	0	13651000	0	43868000	0	1219000	0	1788400
Q9NZ52	Q9NZ52	ADP-ribosylation factor-binding protein GGA3	GGA3	sp|Q9NZ52	3	3	3	0	2	0	3	0	2	0	3	0	2	0	3	6.4	78.314	0	6.8051	57473000	0	13964000	0	43509000	0	1413400	0	1606900
P01024	P01024	Complement C3;Complement C3 beta chain;C3-b	C3	sp|P01024	4	2	2	2	2	3	1	1	0	2	0	1	0	2	0	2.5	187.15	0.000716	3.2712	57438000	40429000	0	17009000	0	43098000	0	8760800	0
P23508	P23508	Colorectal mutant cancer protein	MCC	sp|P23508	3	3	3	0	0	2	3	0	0	2	3	0	0	2	3	6.8	93.026	0	9.6303	57362000	0	0	2910300	54451000	0	0	2173600	2021500
Q86WR0	Q86WR0	Coiled-coil domain-containing protein 25	CCDC25	sp|Q86WR	2	2	2	0	2	0	2	0	2	0	2	0	2	0	2	11.1	24.479	0.000945	3.0874	56916000	0	18451000	0	38465000	0	1486300	0	1722000
Q7Z3C6	Q7Z3C6	Autophagy-related protein 9A	ATG9A	sp|Q7Z3C6	3	3	3	0	3	1	2	0	3	1	2	0	3	1	2	6.2	94.446	0.000248	3.7294	56892000	0	26567000	2199500	28125000	0	1519700	2154300	1302600
P06400	P06400	Retinoblastoma-associated protein	RB1	sp|P06400	3	3	3	0	1	0	3	0	1	0	3	0	1	0	3	5.8	106.16	0	18.504	56848000	0	5277200	0	51571000	0	831550	0	1754500
Q9NWU1	Q9NWU1	3-oxoacyl-[acyl-carrier-protein] synthase, mitoch	OXSM	sp|Q9NWU	3	3	3	0	3	0	1	0	3	0	1	0	3	0	1	13.1	48.842	0	13.05	56836000	0	49976000	0	6860800	0	3731400	0	956090
Q9NWS0	Q9NWS0	PIH1 domain-containing protein 1	PIH1D1	sp|Q9NWS	2	2	2	0	1	0	2	0	1	0	2	0	1	0	2	10	32.363	0	5.2601	56769000	0	10192000	0	46577000	0	0	0	1915500
Q7L5L3	Q7L5L3	Glycerophosphodiester phosphodiesterase doma	GDPD3	sp|Q7L5L3	1	1	1	0	0	1	1	0	0	1	1	0	0	1	1	4.4	36.596	0	4.3399	56581000	0	0	9251400	47329000	0	0	6216900	1946400
P58546	P58546	Myotrophin	MTPN	sp|P58546	1	1	1	0	1	0	1	0	1	0	1	0	1	0	1	14.4	12.895	0	38.323	56507000	0	23455000	0	33052000	0	2067500	0	1359300
P15104	P15104	Glutamine synthetase	GLUL	sp|P15104	3	3	3	0	3	0	0	0	3	0	0	0	3	0	0	16.6	42.064	0	10.217	56467000	0	56467000	0	0	0	4977500	0	0
O95684	O95684	FGFR1 oncogene partner	FGFR1OP	sp|O95684	2	2	2	0	1	0	2	0	1	0	2	0	1	0	2	11.3	43.064	0	11.146	56345000	0	10600000	0	45745000	0	1078900	0	1736800
O95149	O95149	Snurportin-1	SNUPN	sp|O95149	1	1	1	0	1	0	1	0	1	0	1	0	1	0	1	7.5	41.142	0	35.561	56333000	0	8297600	0	48035000	0	731430	0	1975500
P47712	P47712	Cytosolic phospholipase A2;Phospholipase A2;Lys	PLA2G4A	sp|P47712	3	3	3	0	0	0	3	0	0	0	3	0	0	0	3	7.1	85.238	0	13.199	56287000	0	0	0	56287000	0	0	0	2314800
O95807	O95807	Transmembrane protein 50A	TMEM50A	sp|O95807	1	1	1	0	1	0	1	0	1	0	1	0	1	0	1	5.1	17.4	0.000484	3.4245	56251000	0	8802100	0	47449000	0	775900	0	1951400
Q15796	Q15796	Mothers against decapentaplegic homolog 2	SMAD2	sp|Q15796	2	2	1	0	2	1	1	0	2	1	1	0	1	0	0	5.1	52.306	0	4.3059	56231000	0	23655000	2517600	30058000	0	2171100	1532300	1309800
P42785	P42785	Lysosomal Pro-X carboxypeptidase	PRCP	sp|P42785	1	1	1	0	1	0	1	0	1	0	1	0	1	0	1	2.4	55.799	0.008441	1.5915	56133000	0	18402000	0	37731000	0	0	0	1551700
P04049	P04049	RAF proto-oncogene serine/threonine-protein kir	RAF1	sp|P04049	2	2	2	0	2	1	2	0	2	1	2	0	2	1	2	4.9	73.051	0	7.6289	56114000	0	12272000	822340	43019000	0	1125800	1081100	1196700
Q96F85	Q96F85	CB1 cannabinoid receptor-interacting protein 1	CNRIP1	sp|Q96F85	2	2	2	0	1	0	2	0	1	0	2	0	1	0	2	23.2	18.648	0	9.4025	56015000	0	0	0	56015000	0	0	0	2303600
O43677	O43677	NADH dehydrogenase [ubiquinone] 1 subunit C1,	NDUFC1	sp|O43677	1	1	1	0	0	1	0	0	0	1	0	0	0	1	0	10.5	8.7341	0.003086	2.1197	55906000	0	0	55906000	0	0	0	32565000	0
Q8TEY7	Q8TEY7	Ubiquitin carboxyl-terminal hydrolase 33	USP33	sp|Q8TEY7	2	2	2	0	1	1	1	0	1	1	1	0	1	1	1	4.2	106.73	0.000952	3.2039	55870000	0	16887000	27908000	11075000	0	1488600	18754000	0
Q6NZY4	Q6NZY4	Zinc finger CCHC domain-containing protein 8	ZCCHC8	sp|Q6NZY4	5	5	5	0	5	0	0	0	5	0	0	0	5	0	0	12.6	78.576	0	10.75	55729000	0	55729000	0	0	0	4912400	0	0
Q8WVC6	Q8WVC6	Dephospho-CoA kinase domain-containing protei	DCAKD	sp|Q8WVC	2	2	2	0	1	0	2	0	1	0	2	0	1	0	2	10	26.55	0	4.3392	55641000	0	10153000	0	45488000	0	1207700	0	1558000
Q9NTI5	Q9NTI5	Sister chromatid cohesion protein PDS5 homolog	PDS5B	sp|Q9NTI5	5	4	4	0	5	0	2	0	4	0	1	0	4	0	1	6.2	164.67	0	3.9644	55370000	0	38400000	0	16969000	0	3127300	0	955560
Q9UJ83	Q9UJ83	2-hydroxyacyl-CoA lyase 1	HACL1	sp|Q9UJ83	3	3	3	0	3	1	2	0	3	1	2	0	3	1	2	5.4	63.728	0	11.299	54762000	0	36965000	2718900	15078000	0	2401000	1859200	1445400
Q12893	Q12893	Transmembrane protein 115	TMEM115	sp|Q12893	1	1	1	0	1	0	1	0	1	0	1	0	1	0	1	5.1	38.197	0.001134	2.4823	54630000	0	13064000	0	41567000	0	1151500	0	1709500
O00592	O00592	Podocalyxin	PODXL	sp|O00592	2	2	2	0	2	0	1	0	2	0	1	0	2	0	1	3.9	58.635	0	4.478	54522000	0	42752000	0	11770000	0	3768600	0	0
O15013	O15013	Rho guanine nucleotide exchange factor 10	ARHGEF10	sp|O15013	4	4	4	0	0	0	4	0	0	0	4	0	0	0	4	6.7	151.61	0	16.176	54459000	0	0	0	54459000	0	0	0	2239700
P07602	P07602	Prosaposin;Saposin-A;Saposin-B-Val;Saposin-B;Sa	PSAP	sp|P07602	2	2	2	0	2	1	2	0	2	1	2	0	2	1	2	4.6	58.112	0	5.5324	54197000	0	13866000	2196100	38135000	0	672860	1333800	2259700
Q8NFF5	Q8NFF5	FAD synthase;Molybdenum cofactor biosynthesis	FLAD1	sp|Q8NFF5	4	4	4	0	4	0	2	0	4	0	2	0	4	0	2	10.9	65.265	0	14.043	54128000	0	17475000	0	36653000	0	1837500	0	1210300
Q9H1E5	Q9H1E5	Thioredoxin-related transmembrane protein 4	TMX4	sp|Q9H1E5	2	2	2	0	0	0	2	0	0	0	2	0	0	0	2	8	38.952	0	5.8273	54102000	0	0	0	54102000	0	0	0	2225000
P14373	P14373	Zinc finger protein RFP	TRIM27	sp|P14373	2	2	2	1	1	1	1	1	1	1	1	1	1	1	1	5.1	58.489	0	6.3683	54081000	39908000	4975000	2106800	7090700	0	339970	1528700	277230
O75190;Q8	O75190	DnaJ homolog subfamily B member 6	DNAJB6	sp|O75190	3	3	3	0	2	3	1	0	2	3	1	0	2	3	1	10.1	36.087	0	5.2099	54069000	0	19157000	9170600	25741000	0	1717400	4920400	2272200
Q13112	Q13112	Chromatin assembly factor 1 subunit B	CHAF1B	sp|Q13112	3	3	3	1	3	1	2	1	3	1	2	1	3	1	2	10	61.492	0	6.3331	53953000	1223400	27506000	586000	24638000	1461700	1264300	1346100	982990
Q86W56	Q86W56	Poly(ADP-ribose) glycohydrolase	PARG	sp|Q86W5	3	3	3	0	3	0	2	0	3	0	2	0	3	0	2	4.2	111.11	0	6.494	53950000	0	28667000	0	25283000	0	2468700	0	1098100
Q96JP5	Q96JP5	E3 ubiquitin-protein ligase ZFP91	ZFP91	sp|Q96JP5	2	2	2	0	2	2	2	0	2	2	2	0	2	2	2	6.5	63.444	0.001167	2.9199	53931000	0	17349000	4623700	31958000	0	1687500	2794800	1468400
Q9H6L5	Q9H6L5	Protein FAM134B	FAM134B	sp|Q9H6L5	2	2	2	0	1	0	2	0	1	0	2	0	1	0	2	7.6	54.68	0	7.7174	53901000	0	9539000	0	44362000	0	1033800	0	1631500
O14965	O14965	Aurora kinase A	AURKA	sp|O14965	2	2	2	0	2	2	2	0	2	2	2	0	2	2	2	8.9	45.809	0	7.6766	53900000	0	6802300	6151900	40945000	0	1175900	3122700	2118900
Q68CQ4	Q68CQ4	Digestive organ expansion factor homolog	DIEXF	sp|Q68CQ4	5	5	5	0	5	0	5	0	5	0	5	0	5	0	5	7.3	87.054	0	7.0297	53886000	0	24790000	0	29096000	0	1763100	0	1618700
P41236;Q6	P41236;Q6	Protein phosphatase inhibitor 2;Protein phosphat	PPP1R2;PP	sp|P41236	2	2	2	0	1	0	2	0	1	0	2	0	1	0	2	17.6	23.015	0	6.1751	53745000	0	14550000	0	39194000	0	2094500	0	800010
Q8WXA9	Q8WXA9	Splicing regulatory glutamine/lysine-rich protein 1	SREK1	sp|Q8WXA	2	2	2	0	2	0	0	0	2	0	0	0	2	0	0	7.3	59.38	0	17.193	53700000	0	53700000	0	0	0	4733600	0	0
Q8IZH2	Q8IZH2	5-3 exoribonuclease 1	XRN1	sp|Q8IZH2	4	4	4	0	1	3	4	0	1	3	4	0	1	3	4	3.5	194.1	0	5.2053	53576000	0	4742600	4089300	44744000	0	1613400	1762800	1630100
Q5VT66	Q5VT66	Mitochondrial amidoxime-reducing component 1	Mar-01	sp|Q5VT66	2	2	2	0	2	0	0	0	2	0	0	0	2	0	0	12.5	37.499	0	35.76	53383000	0	53383000	0	0	0	4705700	0	0
Q9Y5A7	Q9Y5A7	NEDD8 ultimate buster 1	NUB1	sp|Q9Y5A7	3	3	3	0	3	0	2	0	3	0	2	0	3	0	2	5.4	70.537	0	7.3302	53374000	0	21574000	0	31800000	0	1746800	0	1462700
Q9Y6A4	Q9Y6A4	Cilia- and flagella-associated protein 20	CFAP20	sp|Q9Y6A4	2	2	2	0	2	1	2	0	2	1	2	0	2	1	2	8.8	22.774	0.000948	3.1447	53114000	0	22255000	1749500	29110000	0	2165700	0	993250
Q9BV23	Q9BV23	Monoacylglycerol lipase ABHD6	ABHD6	sp|Q9BV23	2	2	2	0	0	0	2	0	0	0	2	0	0	0	2	8.3	38.33	0	5.9428	53070000	0	0	0	53070000	0	0	0	2182500
P34810	P34810	Macrosialin	CD68	sp|P34810	1	1	1	0	0	0	1	0	0	0	1	0	0	0	1	6.2	37.408	0	12.314	52995000	0	0	0	52995000	0	0	0	2179500
P98194	P98194	Calcium-transporting ATPase type 2C member 1	ATP2C1	sp|P98194	2	2	2	0	1	1	2	0	1	1	2	0	1	1	2	2.9	100.58	0	11.331	52988000	0	2620400	1323700	49044000	0	546220	875110	1716100
Q969E2	Q969E2	Secretory carrier-associated membrane protein 4	SCAMP4	sp|Q969E2	1	1	1	0	1	1	1	0	1	1	1	0	1	1	1	4.8	25.728	0.000952	3.2026	52853000	0	15837000	1532900	35483000	0	1396000	1030100	1459300
Q9C037	Q9C037	E3 ubiquitin-protein ligase TRIM4	TRIM4	sp|Q9C037	3	3	3	0	2	0	2	0	2	0	2	0	2	0	2	8	57.46	0	6.3839	52847000	0	35140000	0	17707000	0	3097600	0	0
Q9NVZ3	Q9NVZ3	Adaptin ear-binding coat-associated protein 2	NECAP2	sp|Q9NVZ3	2	2	2	0	1	0	2	0	1	0	2	0	1	0	2	12.5	28.338	0	6.3605	52827000	0	0	0	52827000	0	0	0	2172500
Q14331	Q14331	Protein FRG1	FRG1	sp|Q14331	1	1	1	0	1	1	1	0	1	1	1	0	1	1	1	5	29.172	0.006007	1.767	52814000	0	8143400	6937100	37733000	0	742870	5096200	1092200
Q86VX2	Q86VX2	COMM domain-containing protein 7	COMMD7	sp|Q86VX2	3	3	3	0	2	0	3	0	2	0	3	0	2	0	3	20	22.54	0	15.312	52766000	0	10609000	0	42157000	0	1169100	0	1499700
Q8IV38	Q8IV38	Ankyrin repeat and MYND domain-containing pro	ANKMY2	sp|Q8IV38	3	3	3	0	1	0	3	0	1	0	3	0	1	0	3	7.3	49.298	0	6.013	52674000	0	6218200	0	46456000	0	886950	0	1571700
O14972	O14972	Down syndrome critical region protein 3	DSCR3	sp|O14972	1	1	1	0	1	0	1	0	1	0	1	0	1	0	1	4	33.01	0.001174	3.0198	52596000	0	13161000	0	39435000	0	1160100	0	1621800
P16083	P16083	Ribosyldihydronicotinamide dehydrogenase [quin	NQO2	sp|P16083	2	2	2	0	2	0	1	0	2	0	1	0	2	0	1	19.5	25.918	0	4.5096	52532000	0	36063000	0	16469000	0	2655400	0	1200800
O75935	O75935	Dynactin subunit 3	DCTN3	sp|O75935	3	3	3	0	3	1	2	0	3	1	2	0	3	1	2	15.1	21.119	0.001152	2.6983	52512000	0	24726000	763960	27022000	0	1723500	0	1567400
Q9Y343	Q9Y343	Sorting nexin-24	SNX24	sp|Q9Y343	2	2	2	0	1	0	2	0	1	0	2	0	1	0	2	10.7	19.818	0	4.5114	52480000	0	4298400	0	48182000	0	879650	0	1480700
Q06546	Q06546	GA-binding protein alpha chain	GABPA	sp|Q06546	3	3	3	0	3	0	2	0	3	0	2	0	3	0	2	8.6	51.295	0	4.7392	52462000	0	16691000	0	35771000	0	1252100	0	1690300
Q9HCD5	Q9HCD5	Nuclear receptor coactivator 5	NCOA5	sp|Q9HCD	2	2	2	0	1	2	2	0	1	2	2	0	1	2	2	6.4	65.536	0	4.5325	52454000	0	7102900	5453800	39897000	0	1411000	2843700	1677100
O95070	O95070	Protein YIF1A	YIF1A	sp|O95070	1	1	1	0	1	1	1	0	1	1	1	0	1	1	1	4.1	32.011	0.000246	3.6489	52397000	0	3324000	1975900	47097000	0	857530	1184900	1515200
O14646	O14646	Chromodomain-helicase-DNA-binding protein 1	CHD1	sp|O14646	4	3	3	0	4	1	2	0	3	0	1	0	3	0	1	3.9	196.69	0	8.8164	52325000	0	39424000	0	12901000	0	3162400	0	843360
Q9UPN6	Q9UPN6	Protein SCAF8	SCAF8	sp|Q9UPN	6	3	3	0	4	1	6	0	1	0	3	0	1	0	3	5.9	140.52	0	7.5678	52309000	0	15382000	0	36927000	0	1619600	0	1254900
P16333;O4	P16333	Cytoplasmic protein NCK1	NCK1	sp|P16333	3	3	3	0	3	0	3	0	3	0	3	0	3	0	3	8.8	42.864	0.001172	2.9859	52300000	0	19479000	0	32821000	0	1715900	0	1350900
Q9UKR5	Q9UKR5	Probable ergosterol biosynthetic protein 28	C14orf1	sp|Q9UKR	1	1	1	0	1	1	1	0	1	1	1	0	1	1	1	6.4	15.864	0.00113	2.4329	52223000	0	19650000	1454600	31118000	0	1732100	977480	1279800
Q8IV04	Q8IV04	Carabin	TBC1D10C	sp|Q8IV04	1	1	1	1	0	1	0	1	0	1	0	1	0	1	0	2	49.712	0.00115	2.6756	52174000	8121200	0	44053000	0	8121200	0	29603000	0
Q9NWM8	Q9NWM8	Peptidyl-prolyl cis-trans isomerase FKBP14	FKBP14	sp|Q9NWM	2	2	2	0	2	0	2	0	2	0	2	0	2	0	2	14.2	24.171	0.001127	2.4123	52046000	0	24939000	0	27107000	0	2168400	0	1144700
P53667;P5	P53667	LIM domain kinase 1	LIMK1	sp|P53667	3	3	3	0	1	0	3	0	1	0	3	0	1	0	3	4.2	72.584	0.000719	3.3108	52027000	0	2769000	0	49258000	0	0	0	2025800
O43150	O43150	Arf-GAP with SH3 domain, ANK repeat and PH do	ASAP2	sp|O43150	4	4	3	0	1	0	4	0	1	0	4	0	0	0	3	6.4	111.65	0	6.969	52025000	0	2562000	0	49463000	0	335500	0	1924500
P63165;G2	P63165;G2	Small ubiquitin-related modifier 1;Small ubiquitin	SUMO1	sp|P63165	2	2	2	1	2	2	2	1	2	2	2	1	2	2	2	27.7	11.557	0	3.853	52007000	2127200	22938000	2084100	24858000	1334600	2011400	1407600	1818400
Q9BR61	Q9BR61	Acyl-CoA-binding domain-containing protein 6	ACBD6	sp|Q9BR61	3	3	3	0	2	0	2	0	2	0	2	0	2	0	2	14.2	31.15	0	6.6959	51782000	0	21933000	0	29849000	0	1429400	0	1731500
Q9H6F5	Q9H6F5	Coiled-coil domain-containing protein 86	CCDC86	sp|Q9H6F5	3	3	3	0	2	3	2	0	2	3	2	0	2	3	2	11.9	40.235	0	4.3443	51766000	0	6849100	12869000	32048000	0	934580	7505800	2129200
Q9UNQ2	Q9UNQ2	Probable dimethyladenosine transferase	DIMT1	sp|Q9UNC	2	2	2	1	1	0	1	1	1	0	1	1	1	0	1	6.4	35.236	0	4.3927	51764000	28990000	8909200	0	13865000	0	785340	0	570220
O95298;E9	O95298;E9	NADH dehydrogenase [ubiquinone] 1 subunit C2;	NDUFC2;N	sp|O95298	2	2	2	0	2	1	1	0	2	1	1	0	2	1	1	16	14.187	0.000484	3.4319	51695000	0	27172000	684460	23838000	0	2014500	740280	1080800
Q6DKJ4	Q6DKJ4	Nucleoredoxin	NXN	sp|Q6DKJ4	2	2	2	0	1	0	1	0	1	0	1	0	1	0	1	6.7	48.392	0.001151	2.6944	51620000	0	18220000	0	33400000	0	1606000	0	0
P78310	P78310	Coxsackievirus and adenovirus receptor	CXADR	sp|P78310	3	3	3	1	3	1	0	1	3	1	0	1	3	1	0	11.5	40.029	0	6.3039	51597000	1105000	49184000	1307700	0	2085600	2373800	1859900	0
P48506	P48506	Glutamate--cysteine ligase catalytic subunit	GCLC	sp|P48506	2	2	2	0	0	0	2	0	0	0	2	0	0	0	2	5.2	72.765	0	4.1342	51580000	0	0	0	51580000	0	0	0	2121200

P39880	P39880	Homeobox protein cut-like 1	CUX1	sp|P39880	3	3	3	0	3	2	1	0	3	2	1	0	3	2	1	2.9	164.19	0	8.1971	51484000	0	37372000	1298500	12813000	0	2119800	1401200	1172900
Q9H7L9	Q9H7L9	Sin3 histone deacetylase corepressor complex co	SUDS3	sp|Q9H7L9	3	3	3	0	3	0	2	0	3	0	2	0	3	0	2	13.1	38.136	0.000248	3.6965	51478000	0	24454000	0	27025000	0	2275800	0	991180
Q12894	Q12894	Interferon-related developmental regulator 2	IFRD2	sp|Q12894	3	3	3	0	2	0	3	0	2	0	3	0	2	0	3	7.3	54.813	0	6.6636	51370000	0	25455000	0	25915000	0	2231200	0	1078400
P17152	P17152	Transmembrane protein 11, mitochondrial	TMEM11	sp|P17152	2	2	2	0	2	1	1	0	2	1	1	0	2	1	1	12.5	21.541	0	5.8133	51356000	0	35067000	517170	15772000	0	1622800	989920	1474600
Q8N4L2	Q8N4L2	Type 2 phosphatidylinositol 4,5-bisphosphate 4-p	TMEM55A	sp|Q8N4L2	2	2	2	0	0	2	2	0	0	2	2	0	0	2	2	12.8	28.081	0	9.869	51321000	0	0	2568100	48753000	0	0	1732000	1998700
Q9P2B2	Q9P2B2	Prostaglandin F2 receptor negative regulator	PTGFRN	sp|Q9P2B2	4	4	4	0	2	0	4	0	2	0	4	0	2	0	4	4.7	98.555	0	9.8071	51292000	0	10044000	0	41248000	0	983920	0	1597800
Q9NVU7	Q9NVU7	Protein SDA1 homolog	SDAD1	sp|Q9NVU7	2	2	2	0	2	1	2	0	2	1	2	0	2	1	2	3.5	79.87	0.001155	2.7389	51180000	0	13748000	884540	36548000	0	1002100	1456700	850530
P30038	P30038	Delta-1-pyrroline-5-carboxylate dehydrogenase, r	ALDH4A1	sp|P30038	7	7	7	0	7	0	1	0	7	0	1	0	7	0	1	15.8	61.719	0	14.707	51166000	0	51166000	0	0	0	4510300	0	0
Q16678	Q16678	Cytochrome P450 1B1	CYP1B1	sp|Q16678	2	2	2	0	0	1	2	0	0	1	2	0	0	1	2	5.5	60.845	0	23.657	50816000	0	0	1395800	49420000	0	0	1884200	1086200
P09038	P09038	Fibroblast growth factor 2	FGF2	sp|P09038	2	2	2	0	0	0	2	0	0	0	2	0	0	0	2	5.9	30.77	0.001161	2.8256	50649000	0	0	0	50649000	0	0	0	2083000
Q99956	Q99956	Dual specificity protein phosphatase 9	DUSP9	sp|Q99956	2	2	2	0	2	0	0	0	2	0	0	0	2	0	0	6.5	41.867	0.001166	2.897	50575000	0	50575000	0	0	0	4458100	0	0
O95674	O95674	Phosphatidate cytidylyltransferase 2	CDS2	sp|O95674	2	2	2	0	2	0	2	0	2	0	2	0	2	0	2	8.5	51.417	0	4.2701	50566000	0	23407000	0	27159000	0	1963700	0	1216500
Q9HAB8	Q9HAB8	Phosphopantothenate--cysteine ligase	PPCS	sp|Q9HAB8	4	4	4	0	4	1	1	0	4	1	1	0	4	1	1	11.9	34.005	0	8.5294	50465000	0	43350000	1324300	5790700	0	3683700	835600	430060
Q16625	Q16625	Occludin	OCLN	sp|Q16625	4	4	4	0	4	0	0	0	4	0	0	0	4	0	0	11.7	59.143	0	6.5674	50380000	0	50380000	0	0	0	4440900	0	0
Q5BJD5	Q5BJD5	Transmembrane protein 41B	TMEM41B	sp|Q5BJD5	1	1	1	1	1	1	1	1	1	1	1	1	1	1	1	6.5	32.513	0	6.0566	50324000	565940	13194000	1384200	35181000	730110	1046600	936010	1393200
Q9NXX6	Q9NXX6	Non-structural maintenance of chromosomes ele	NSMCE4A	sp|Q9NXX6	5	5	5	0	5	0	0	0	5	0	0	0	5	0	0	19	44.301	0	9.4654	50267000	0	50267000	0	0	0	4431000	0	0
P62253	P62253	Ubiquitin-conjugating enzyme E2 G1;Ubiquitin-co	UBE2G1	sp|P62253	1	1	1	0	1	0	1	0	1	0	1	0	1	0	1	5.9	19.509	0	9.1025	50043000	0	22125000	0	27919000	0	1950300	0	1148200
Q9NVM4	Q9NVM4	Protein arginine N-methyltransferase 7	PRMT7	sp|Q9NVM4	2	2	2	0	0	0	2	0	0	0	2	0	0	0	2	5.5	78.458	0	9.397	50018000	0	0	0	50018000	0	0	0	2057000
Q8WZA0	Q8WZA0	Protein LZIC	LZIC	sp|Q8WZA0	2	2	2	0	2	0	2	0	2	0	2	0	2	0	2	7.9	21.494	0	3.8168	50001000	0	14858000	0	35142000	0	1307900	0	1447100
Q6P4A7	Q6P4A7	Sideroflexin-4	SFXN4	sp|Q6P4A7	2	2	2	0	2	2	1	0	2	2	1	0	2	2	1	8	37.998	0.001171	2.9737	49989000	0	29325000	3745600	16918000	0	2342300	2307600	1147900
Q15291	Q15291	Retinoblastoma-binding protein 5	RBBP5	sp|Q15291	3	3	3	0	3	1	2	0	3	1	2	0	3	1	2	11.2	59.152	0	5.5667	49942000	0	31496000	381260	18065000	0	2250400	448010	1077000
P55058	P55058	Phospholipid transfer protein	PLTP	sp|P55058	2	2	2	0	0	0	2	0	0	0	2	0	0	0	2	7.3	54.739	0	9.1389	49940000	0	0	0	49940000	0	0	0	2053800
O43924	O43924	Retinal rod rhodopsin-sensitive cGMP 3,5-cyclic p	PDE6D	sp|O43924	2	2	2	0	2	0	2	0	2	0	2	0	2	0	2	12.7	17.42	0	4.9013	49927000	0	14381000	0	35546000	0	1196000	0	1533500
Q9NY93	Q9NY93	Probable ATP-dependent RNA helicase DDX56	DDX56	sp|Q9NY93	3	3	3	1	0	3	3	1	0	3	3	1	0	3	3	7.5	61.589	0	7.8724	49867000	657370	0	7762400	41447000	2199000	0	4011400	1367800
O95229	O95229	ZW10 interactor	ZWINT	sp|O95229	2	2	2	0	2	0	2	0	2	0	2	0	2	0	2	17	31.293	0	9.7115	49718000	0	20688000	0	29030000	0	1801200	0	1216300
Q5VWJ9	Q5VWJ9	Sorting nexin-30	SNX30	sp|Q5VWJ9	1	1	1	0	0	0	1	0	0	0	1	0	0	0	1	4.3	49.676	0.007436	1.6648	49344000	0	0	0	49344000	0	0	0	2029300
O43772	O43772	Mitochondrial carnitine/acylcarnitine carrier prot	SLC25A20	sp|O43772	2	2	2	0	2	0	2	0	2	0	2	0	2	0	2	7	32.943	0	4.5082	49297000	0	10735000	0	38562000	0	903390	0	1628700
Q8N5C6	Q8N5C6	S1 RNA-binding domain-containing protein 1	SRBD1	sp|Q8N5C6	3	3	3	1	3	3	3	1	3	3	3	1	3	3	3	2.8	111.77	0	4.324	49191000	5669300	13544000	3683500	26295000	5268000	1672600	1863100	1616200
Q5UCC4	Q5UCC4	ER membrane protein complex subunit 10	EMC10	sp|Q5UCC4	1	1	1	0	1	0	1	0	1	0	1	0	1	0	1	6.5	27.347	0	7.9952	49168000	0	19930000	0	29238000	0	1756800	0	1202400
Q13625;Q5	Q13625	Apoptosis-stimulating of p53 protein 2	TP53BP2	sp|Q13625	4	4	4	0	2	0	4	0	2	0	4	0	2	0	4	5.2	125.61	0	8.6684	49151000	0	6314700	0	42836000	0	946810	0	1371500
Q9P1Y5	Q9P1Y5	Calmodulin-regulated spectrin-associated protein	CAMSAP3	sp|Q9P1Y5	3	2	2	0	3	1	1	0	2	1	1	0	2	1	1	2.8	134.75	0.00116	2.8249	49137000	0	8354200	2773200	38009000	0	343540	1993700	1825900
Q9BRF8	Q9BRF8	Serine/threonine-protein phosphatase CPPED1	CPPED1	sp|Q9BRF8	1	1	1	0	1	0	1	0	1	0	1	0	1	0	1	3.5	35.548	0.000949	3.1588	49058000	0	17070000	0	31988000	0	1504700	0	1315500
Q9BQP7	Q9BQP7	Mitochondrial genome maintenance exonuclease	MGME1	sp|Q9BQP7	2	2	2	0	2	0	2	0	2	0	2	0	2	0	2	8.1	39.42	0	3.9194	49020000	0	24049000	0	24971000	0	2123800	0	1023100
Q9Y4A5	Q9Y4A5	Transformation/transcription domain-associated	TRRAP	sp|Q9Y4A5	4	4	4	0	4	0	3	0	4	0	3	0	4	0	3	1.3	437.6	0	5.805	48941000	0	20537000	0	28404000	0	1554400	0	1424000
P61626	P61626	Lysozyme C	LYZ	sp|P61626	3	3	3	0	3	3	0	0	3	3	0	0	3	3	0	18.2	16.537	0	5.7159	48843000	0	31126000	17717000	0	0	2740700	11908000	0
Q9ULQ0	Q9ULQ0	Striatin-interacting protein 2	STRIP2	sp|Q9ULQ0	1	1	1	0	0	0	1	0	0	0	1	0	0	0	1	3	95.358	0	5.4949	48829000	0	0	0	48829000	0	0	0	2008100
Q92858	Q92858	Protein atonal homolog 1	ATOH1	sp|Q92858	1	1	1	0	0	1	0	0	0	1	0	0	0	1	0	3.1	38.16	0.008053	1.6296	48821000	0	0	48821000	0	0	0	32807000	0
Q9BQ48	Q9BQ48	39S ribosomal protein L34, mitochondrial	MRPL34	sp|Q9BQ48	2	2	2	0	2	1	2	0	2	1	2	0	2	1	2	23.9	10.165	0	6.3742	48702000	0	19088000	1525800	28087000	0	1304300	1761900	796880
Q9H3G5	Q9H3G5	Probable serine carboxypeptidase CPVL	CPVL	sp|Q9H3G5	3	3	3	0	3	1	0	0	3	1	0	0	3	1	0	9	54.163	0	11.069	48695000	0	43978000	4716100	0	0	3029300	4016600	0
P62487	P62487	DNA-directed RNA polymerase II subunit RPB7	POLR2G	sp|P62487	2	2	2	0	2	0	2	0	2	0	2	0	2	0	2	16.3	19.294	0	27.842	48618000	0	14235000	0	34383000	0	1267400	0	1401500
Q9BY67	Q9BY67	Cell adhesion molecule 1	CADM1	sp|Q9BY67	2	2	2	0	2	0	0	0	2	0	0	0	2	0	0	7.2	48.509	0	7.7036	48549000	0	48549000	0	0	0	4279600	0	0
Q9BZL1	Q9BZL1	Ubiquitin-like protein 5	UBL5	sp|Q9BZL1	1	1	1	0	0	0	1	0	0	0	1	0	0	0	1	12.3	8.5468	0.001132	2.4616	48503000	0	0	0	48503000	0	0	0	1994700
P03905	P03905	NADH-ubiquinone oxidoreductase chain 4	MT-ND4	sp|P03905	2	2	2	0	1	1	1	0	1	1	1	0	1	1	1	6.1	51.58	0	4.9856	48421000	0	16218000	1740200	30462000	0	1429600	1169400	0
P29084	P29084	Transcription initiation factor IIE subunit beta	GTF2E2	sp|P29084	2	2	2	0	2	0	2	0	2	0	2	0	2	0	2	10.7	33.043	0	4.1244	48173000	0	14674000	0	33499000	0	1188700	0	1482400
Q9HD34	Q9HD34	LYR motif-containing protein 4	LYRM4	sp|Q9HD34	1	1	1	0	1	1	1	0	1	1	1	0	1	1	1	15.4	10.758	0	4.5736	48074000	0	22037000	2182900	23855000	0	0	0	981040
Q9BU76	Q9BU76	Multiple myeloma tumor-associated protein 2	MMTAG2	sp|Q9BU76	3	3	3	1	2	2	1	1	2	2	1	1	2	2	1	14.1	29.411	0	9.938	47873000	666030	39667000	2994400	4546100	823430	3705200	1227700	605410
Q8N129	Q8N129	Protein canopy homolog 4	CNPY4	sp|Q8N129	2	2	2	0	1	0	2	0	1	0	2	0	1	0	2	8.9	28.309	0.000951	3.1967	47837000	0	10991000	0	36846000	0	1040900	0	1443300
Q7L775	Q7L775	EPM2A-interacting protein 1	EPM2AIP1	sp|Q7L775	3	3	3	0	0	0	3	0	0	0	3	0	0	0	3	7.9	70.369	0	11.565	47694000	0	0	0	47694000	0	0	0	1961500
Q8IUI8	Q8IUI8	Cytokine receptor-like factor 3	CRLF3	sp|Q8IUI8	3	3	3	0	3	0	3	0	3	0	3	0	3	0	3	7.7	49.765	0.001174	3.0229	47650000	0	20594000	0	27056000	0	1725100	0	1203000
P15848	P15848	Arylsulfatase B	ARSB	sp|P15848	1	1	1	0	0	0	1	0	0	0	1	0	0	0	1	2.6	59.687	0.001151	2.6816	47494000	0	0	0	47494000	0	0	0	1953200
Q8WVV4	Q8WVV4	Protein POF1B	POF1B	sp|Q8WVV4	5	5	5	0	1	5	2	0	1	5	2	0	1	5	2	9.2	68.064	0	9.4431	47421000	0	11299000	24603000	11519000	0	640540	16705000	656490
Q8WUA4	Q8WUA4	General transcription factor 3C polypeptide 2	GTF3C2	sp|Q8WUA4	2	2	2	1	2	2	0	1	2	2	0	1	2	2	0	4.5	100.68	0	4.2196	47369000	3380100	38112000	5877500	0	6107600	1525900	3055800	0
O15254	O15254	Peroxisomal acyl-coenzyme A oxidase 3	ACOX3	sp|O15254	1	1	1	0	0	0	1	0	0	0	1	0	0	0	1	1.6	77.628	0.000486	3.4757	47351000	0	0	0	47351000	0	0	0	1947300
Q96HN2	Q96HN2	Putative adenosylhomocysteinase 3	AHCYL2	sp|Q96HN2	4	1	1	0	3	1	4	0	0	0	1	0	0	0	1	8.7	66.72	0	5.6438	47281000	0	0	0	47281000	0	0	0	1944400
Q969X1	Q969X1	Protein lifeguard 3	TMBIM1	sp|Q969X1	2	2	2	0	0	0	2	0	0	0	2	0	0	0	2	4.8	34.607	0	7.7078	47230000	0	0	0	47230000	0	0	0	1942400
Q9UK76	Q9UK76	Hematological and neurological expressed 1 prot	HN1	sp|Q9UK76	2	2	2	1	2	0	2	1	2	0	2	1	2	0	2	21.4	16.014	0	4.6473	47214000	13651000	21232000	0	12332000	11795000	2438000	0	1796700
Q8IUR0	Q8IUR0	Trafficking protein particle complex subunit 5	TRAPPC5	sp|Q8IUR0	2	2	2	0	2	2	2	0	2	2	2	0	2	2	2	9	20.783	0.000721	3.3339	46958000	0	10430000	1885600	34643000	0	988130	1166600	1456400
P48060	P48060	Glioma pathogenesis-related protein 1	GLIPR1	sp|P48060	1	1	1	0	0	0	1	0	0	0	1	0	0	0	1	5.3	30.366	0	4.1691	46926000	0	0	0	46926000	0	0	0	1929800
Q7L099	Q7L099	Protein RUFY3	RUFY3	sp|Q7L099	4	3	3	0	1	0	4	0	0	0	3	0	0	0	3	11.7	52.964	0	6.5314	46779000	0	0	0	46779000	0	0	0	1923800
P69849;Q5	P69849;Q5	Nodal modulator 3;Nodal modulator 2	NOMO3;N	sp|P69849	20	1	1	0	17	1	18	0	1	0	1	0	1	0	1	24.4	134.13	0	7.7619	46756000	0	16359000	0	30396000	0	1442100	0	1250100
Q9H0R4	Q9H0R4	Haloacid dehalogenase-like hydrolase domain-con	HDHD2	sp|Q9H0R4	2	2	2	0	2	0	2	0	2	0	2	0	2	0	2	8.1	28.536	0	4.0487	46649000	0	22080000	0	24569000	0	1775300	0	1181400
Q66K14	Q66K14	TBC1 domain family member 9B	TBC1D9B	sp|Q66K14	4	4	4	0	2	0	4	0	2	0	4	0	2	0	4	3.9	140.52	0	7.899	46638000	0	6470500	0	40167000	0	1008400	0	1213900
O95905	O95905	Protein SGT1	ECD	sp|O95905	3	3	3	0	1	0	3	0	1	0	3	0	1	0	3	7.8	72.757	0	11.647	46634000	0	3538600	0	43096000	0	613680	0	1470600
Q86UV5	Q86UV5	Ubiquitin carboxyl-terminal hydrolase 48	USP48	sp|Q86UV5	3	3	3	0	1	0	3	0	1	0	3	0	1	0	3	5.3	119.03	0	7.6935	46627000	0	5738500	0	40889000	0	891920	0	1295500
Q16775	Q16775	Hydroxyacylglutathione hydrolase, mitochondrial	HAGH	sp|Q16775	4	4	4	0	4	0	0	0	4	0	0	0	4	0	0	15.3	33.805	0	6.6501	46565000	0	46565000	0	0	0	4104600	0	0
Q8WVK7	Q8WVK7	Spindle and kinetochore-associated protein 2	SKA2	sp|Q8WVK7	1	1	1	0	1	0	1	0	1	0	1	0	1	0	1	11.6	14.188	0	5.0579	46394000	0	29084000	0	17310000	0	2563700	0	711890
Q9Y3B9	Q9Y3B9	RRP15-like protein	RRP15	sp|Q9Y3B9	2	2	2	0	2	2	1	0	2	2	1	0	2	2	1	6.4	31.484	0.000248	3.6916	46342000	0	18066000	22738000	5538500	0	3940400	10900000	2259600
Q96IY1	Q96IY1	Kinetochore-associated protein NSL1 homolog	NSL1	sp|Q96IY1	1	1	1	0	1	0	1	0	1	0	1	0	1	0	1	6	32.162	0	4.2464	46313000	0	17921000	0	28392000	0	1579700	0	1167600
Q8N3R9	Q8N3R9	MAGUK p55 subfamily member 5	MPP5	sp|Q8N3R9	2	2	2	0	1	0	2	0	1	0	2	0	1	0	2	4	77.293	0	5.9637	46139000	0	5808400	0	40331000	0	0	0	1658600
Q8IXQ4	Q8IXQ4	GPALPP motifs-containing protein 1	GPALPP1	sp|Q8IXQ4	3	3	3	0	3	0	1	0	3	0	1	0	3	0	1	7.6	38.141	0	6.1344	46135000	0	41813000	0	4322100	0	3435300	0	428220
Q5TEJ8	Q5TEJ8	Protein THEMIS2	THEMIS2	sp|Q5TEJ8	2	2	2	0	0	0	2	0	0	0	2	0	0	0	2	6.1	72.048	0	6.9825	46093000	0	0	0	46093000	0	0	0	1895600
O60216;Q5	O60216;Q5	Double-strand-break repair protein rad21 homolo	RAD21;RAI	sp|O60216	2	2	2	0	2	0	0	0	2	0	0	0	2	0	0	3.3	71.689	0.001154	2.7308	46068000	0	46068000	0	0	0	4060900	0	0
P45984;P5	P45984;P5	Mitogen-activated protein kinase 9;Mitogen-activ	MAPK9;MA	sp|P45984	2	2	2	0	2	0	2	0	2	0	2	0	2	0	2	5.7	48.139	0	5.1949	45985000	0	23353000	0	22632000	0	1918800	0	1070500
Q9Y5L4	Q9Y5L4	Mitochondrial import inner membrane transloca	TIMM13	sp|Q9Y5L4	1	1	1	1	1	0	0	1	1	0	0	1	1	0	0	11.6	10.5	0.001153	2.7151	45972000	45972000	0	0	0	45972000	0	0	0
Q8TB36	Q8TB36	Ganglioside-induced differentiation-associated pr	GDAP1	sp|Q8TB36	1	1	1	0	1	1	1	0	1	1	1	0	1	1	1	4.2	41.345	0.000722	3.3459	45867000	0	12851000	1022000	31993000	0	1132800	686780	1315700
Q6GQQ9	Q6GQQ9	OTU domain-containing protein 7B	OTUD7B	sp|Q6GQQ9	2	2	2	0	2	0	1	0	2	0	1	0	2	0	1	3.4	92.525	0	5.8531	45862000	0	18692000	0	27170000	0	1647700	0	1117400
Q92574	Q92574	Hamartin	TSC1	sp|Q92574	3	3	3	0	3	0	1	0	3	0	1	0	3	0	1	4.9	129.77	0	12.772	45771000	0	26583000	0	19188000	0	1992700	0	1139800
Q9Y5T5	Q9Y5T5	Ubiquitin carboxyl-terminal hydrolase 16	USP16	sp|Q9Y5T5	4	4	4	0	3	1	3	0	3	1	3	0	3	1	3	6.2	93.569	0	6.9227	45672000	0	20470000	559310	24642000	0	1572200	904510	716950
Q96KX1	Q96KX1	Uncharacterized protein C4orf36	C4orf36	sp|Q96KX1	1	1	1	0	1	0	1	0	1	0	1	0	1	0	1	6	13.275	0.00433	1.8959	45591000	0	14959000	0	30632000	0	1318600	0	1259800
Q5SW96	Q5SW96	Low density lipoprotein receptor adapter protein	LDLRAP1	sp|Q5SW96	1	1	1	0	0	0	1	0	0	0	1	0	0	0	1	3.9	33.885	0.000485	3.4623	45307000	0	0	0	45307000	0	0	0	1863300
Q92889	Q92889	DNA repair endonuclease XPF	ERCC4	sp|Q92889	2	2	2	0	2	1	2	0	2	1	2	0	2	1	2	4.3	104.48	0.000247	3.6665	45247000	0	12783000	2077000	30387000	0	1124800	0	1251600
Q9BRT9	Q9BRT9	DNA replication complex GINS protein SLD5;DNA	GINS4	sp|Q9BRT9	1	1	1	0	1	0	1	0	1	0	1	0	1	0	1	8.5	26.047	0	6.0348	45223000	0	23570000	0	21653000	0	2077600	0	890500
Q86W42	Q86W42	THO complex subunit 6 homolog	THOC6	sp|Q86W42	2	2	2	0	2	0	0	0	2	0	0	0	2	0	0	7.3	37.535	0.000487	3.4902	45213000	0	45213000	0	0	0	3985500	0	0
Q7Z3T8	Q7Z3T8	Zinc finger FYVE domain-containing protein 16	ZFYVE16	sp|Q7Z3T8	4	4	4	0	3	0	4	0	3	0	4	0	3	0	4	4.2	168.9	0	16.775	45169000	0	11935000	0	33235000	0	924390	0	1494400
P52926	P52926	High mobility group protein HMGI-C	HMGA2	sp|P52926	2	2	2	0	0	2	2	0	0	2	2	0	0	2	2	33	11.832	0	11.622	45132000	0	0	4352500	40780000	0	0	2910100	1691900
P62070	P62070	Ras-related protein R-Ras2	RRAS2	sp|P62070	4	2	2	2	1	0	4	2	0	0	2	2	0	0	2	25	23.399	0	6.825	45122000	2126900	0	0	42995000	1936000	0	0	1959100
Q14790	Q14790	Caspase-8;Caspase-8 subunit p18;Caspase-8 subu	CASP8	sp|Q14790	3	3	3	0	1	0	3	0	1	0	3	0	1	0	3	6.3	55.391	0	8.5583	45114000	0	1480000	0	43634000	0	152150	0	1772800
Q9H3P2	Q9H3P2	Negative elongation factor A	NELFA	sp|Q9H3P2	2	2	2	0	2	0	1	0	2	0	1	0	2	0	1	9.1	57.276	0	6.4899	45088000	0	35362000	0	9726100	0	2561500	0	955620
O94854	O94854	Uncharacterized protein KIAA0754	KIAA0754	sp|O94854	2	2	2	0	0	2	2	0	0	2	2	0	0	2	2	4.7	135.15	0	15.519	44935000	0	0	3801200	41134000	0	0	2537800	1708300

Q9BZF3	Q9BZF3	Oxysterol-binding protein-related protein 6	OSBPL6	sp|Q9BZF3	2	2	2	0	2	1	2	0	2	1	2	0	2	1	2	2.9	106.3	0	8.5058	44800000	0	10949000	2395700	31455000	0	1025300	1869300	974120
Q6IQ22	Q6IQ22	Ras-related protein Rab-12	RAB12	sp|Q6IQ22	2	2	2	0	1	0	1	0	1	0	1	0	1	0	1	12.7	27.248	0.00095	3.1704	44629000	0	7124000	0	37505000	0	0	0	1542400
Q9UJ14	Q9UJ14	Gamma-glutamyltransferase 7;Gamma-glutamylt	GGT7	sp|Q9UJ14	2	2	2	0	1	0	2	0	1	0	2	0	1	0	2	4.7	70.466	0.008458	1.6078	44513000	0	12434000	0	32079000	0	1386200	0	1029100
Q9H5V9	Q9H5V9	UPF0428 protein CXorf56	CXorf56	sp|Q9H5V9	2	2	2	0	2	1	1	0	2	1	1	0	2	1	1	14.9	25.624	0	4.9239	44349000	0	29382000	2589800	12378000	0	2206100	2037400	595920
Q14657	Q14657	EKC/KEOPS complex subunit LAGE3	LAGE3	sp|Q14657	1	1	1	0	1	0	1	0	1	0	1	0	1	0	1	16.1	14.804	0.00114	2.5684	44325000	0	20385000	0	23940000	0	1796900	0	984560
Q9NP61	Q9NP61	ADP-ribosylation factor GTPase-activating protein	ARFGAP3	sp|Q9NP61	1	1	1	0	1	0	1	0	1	0	1	0	1	0	1	4.5	56.928	0	4.7929	44324000	0	7884600	0	36440000	0	695020	0	1498600
Q96BX8	Q96BX8	MOB kinase activator 3A	MOB3A	sp|Q96BX8	1	1	1	0	0	0	1	0	0	0	1	0	0	0	1	11.5	25.464	0.009255	1.5514	44294000	0	0	0	44294000	0	0	0	1821600
Q6NSJ5	Q6NSJ5	Volume-regulated anion channel subunit LRRC8E	LRRC8E	sp|Q6NSJ5	2	2	2	0	0	0	2	0	0	0	2	0	0	0	2	4.1	90.246	0.000249	3.7642	44286000	0	0	0	44286000	0	0	0	1821300
O60927	O60927	Protein phosphatase 1 regulatory subunit 11	PPP1R11	sp|O60927	2	2	2	0	2	0	2	0	2	0	2	0	2	0	2	32.5	13.952	0.000483	3.3944	44264000	0	19071000	0	25193000	0	1681300	0	1035800
Q9BQE5	Q9BQE5	Apolipoprotein L2	APOL2	sp|Q9BQE	4	4	4	0	0	0	4	0	0	0	4	0	0	0	4	13.9	37.092	0	12.021	44205000	0	0	0	44205000	0	0	0	1817900
P52756	P52756	RNA-binding protein 5	RBM5	sp|P52756	4	4	3	0	3	2	2	0	3	2	2	0	3	2	1	6	92.153	0	4.6307	44161000	0	16105000	6071800	21984000	0	1125000	4374900	0
Q96LI5;Q9	Q96LI5;Q9	CCR4-NOT transcription complex subunit 6-like;C(	CNOT6L;CI	sp|Q96LI5	2	2	2	0	1	1	2	0	1	1	2	0	1	1	2	3.2	63.001	0.000943	3.0691	44087000	0	4801700	1827200	37458000	0	0	1435200	1333100
P08651	P08651	Nuclear factor 1 C-type	NFIC	sp|P08651	3	3	3	0	2	1	3	0	2	1	3	0	2	1	3	7.1	55.674	0	7.4441	44070000	0	6500200	4343200	33226000	0	479830	3787900	590360
P86791;P8	P86791;P8	Vacuolar fusion protein CCZ1 homolog;Vacuolar f	CCZ1;CCZ1	sp|P86791	2	2	2	0	2	0	2	0	2	0	2	0	2	0	2	5.8	55.866	0	6.6583	44063000	0	12996000	0	31067000	0	1056900	0	1366300
Q03426	Q03426	Mevalonate kinase	MVK	sp|Q03426	2	2	2	0	2	0	1	0	2	0	1	0	2	0	1	8.8	42.45	0.006406	1.7166	44046000	0	20085000	0	23960000	0	1770500	0	0
Q8IV48	Q8IV48	3-5 exoribonuclease 1	ERI1	sp|Q8IV48	1	1	1	0	0	1	1	0	0	1	1	0	0	1	1	4.3	40.063	0	5.0757	44015000	0	0	6052900	37963000	0	0	4067500	1561200
Q9NYT0	Q9NYT0	Pleckstrin-2	PLEK2	sp|Q9NYT(	1	1	1	0	0	0	1	0	0	0	1	0	0	0	1	6.2	39.97	0	4.5093	43978000	0	0	0	43978000	0	0	0	1808600
Q9BRQ6	Q9BRQ6	MICOS complex subunit MIC25	CHCHD6	sp|Q9BRQ	3	3	3	1	3	0	1	1	3	0	1	1	3	0	1	22.1	26.457	0	11.277	43969000	605250	39615000	0	3748400	593400	3332400	0	325690
P13051	P13051	Uracil-DNA glycosylase	UNG	sp|P13051	2	2	2	0	1	0	2	0	1	0	2	0	1	0	2	16.6	34.645	0	6.4131	43913000	0	15755000	0	28158000	0	2068800	0	478000
Q96EA4	Q96EA4	Protein Spindly	SPDL1	sp|Q96EA4	2	2	2	0	2	0	1	0	2	0	1	0	2	0	1	5.1	70.171	0.000952	3.2122	43887000	0	27088000	0	16799000	0	1806300	0	1272300
Q8IVS2	Q8IVS2	Malonyl-CoA-acyl carrier protein transacylase, mi	MCAT	sp|Q8IVS2	2	2	2	0	2	2	1	0	2	2	1	0	2	2	1	6.9	42.961	0	5.5564	43843000	0	32968000	3032400	7843000	0	2911600	1488300	866510
P03915	P03915	NADH-ubiquinone oxidoreductase chain 5	MT-ND5	sp|P03915	2	2	2	0	2	0	2	0	2	0	2	0	2	0	2	4.6	67.026	0	5.1608	43796000	0	14672000	0	29124000	0	1304300	0	1186700
Q9Y624	Q9Y624	Junctional adhesion molecule A	F11R	sp|Q9Y624	3	3	3	0	3	0	1	0	3	0	1	0	3	0	1	14	32.583	0	4.0014	43574000	0	24816000	0	18758000	0	2055500	0	903440
P62380	P62380	TATA box-binding protein-like protein 1	TBPL1	sp|P62380	1	1	1	0	0	0	1	0	0	0	1	0	0	0	1	9.1	20.886	0.003901	1.919	43568000	0	0	0	43568000	0	0	0	1791800
Q6P1K8;Q:	Q6P1K8;Q:	General transcription factor IIH subunit 2-like pro	GTF2H2C;(	sp|Q6P1K8	1	1	1	0	1	1	1	0	1	1	1	0	1	1	1	2.3	44.452	0.006013	1.7751	43456000	0	7596700	2487000	33373000	0	786740	1800300	1126300
Q6ZVM7	Q6ZVM7	TOM1-like protein 2	TOM1L2	sp|Q6ZVM	1	1	1	0	0	0	1	0	0	0	1	0	0	0	1	5.3	55.556	0.008633	1.5682	43451000	0	0	0	43451000	0	0	0	1787000
Q96LD4	Q96LD4	Tripartite motif-containing protein 47	TRIM47	sp|Q96LD4	2	2	2	0	0	0	2	0	0	0	2	0	0	0	2	7.2	69.531	0	4.3119	43262000	0	0	0	43262000	0	0	0	1779200
Q92733	Q92733	Proline-rich protein PRCC	PRCC	sp|Q92733	4	4	4	0	4	0	0	0	4	0	0	0	4	0	0	17.3	52.417	0	26.697	43243000	0	43243000	0	0	0	3811900	0	0
Q96EI5	Q96EI5	Transcription elongation factor A protein-like 4	TCEAL4	sp|Q96EI5	3	3	3	0	3	3	3	0	3	3	3	0	3	3	3	20.5	24.647	0	15.463	43185000	0	9694500	2904900	30585000	0	1294700	1562600	1207100
O60427	O60427	Fatty acid desaturase 1	FADS1	sp|O60427	2	2	2	0	1	0	2	0	1	0	2	0	1	0	2	7	51.964	0	5.7169	42920000	0	4938600	0	37981000	0	0	0	1562000
Q9HCG8	Q9HCG8	Pre-mRNA-splicing factor CWC22 homolog	CWC22	sp|Q9HCG	4	4	4	0	4	3	3	0	4	3	3	0	4	3	3	6.1	105.47	0	7.3434	42910000	0	21221000	6071700	15617000	0	1546400	3868100	1178500
P23378	P23378	Glycine dehydrogenase (decarboxylating), mitoch	GLDC	sp|P23378	1	1	1	0	1	0	0	0	1	0	0	0	1	0	0	2.2	112.73	0	21.67	42658000	0	42658000	0	0	0	3760300	0	0
Q8N2K0	Q8N2K0	Monoacylglycerol lipase ABHD12	ABHD12	sp|Q8N2K(	2	2	2	0	0	1	2	0	0	1	2	0	0	1	2	5.3	45.096	0	4.9654	42593000	0	0	1034900	41558000	0	0	1001100	1403500
Q13309	Q13309	S-phase kinase-associated protein 2	SKP2	sp|Q13309	2	2	2	0	2	0	0	0	2	0	0	0	2	0	0	6.1	47.76	0	7.0818	42407000	0	42407000	0	0	0	3738200	0	0
O75832	O75832	26S proteasome non-ATPase regulatory subunit 1	PSMD10	sp|O75832	1	1	1	0	1	0	1	0	1	0	1	0	1	0	1	5.3	24.428	0.004535	1.8408	42355000	0	23257000	0	19098000	0	2050100	0	785420
P11117	P11117	Lysosomal acid phosphatase	ACP2	sp|P11117	2	2	2	0	1	0	2	0	1	0	2	0	1	0	2	5.9	48.344	0	4.2577	42259000	0	11399000	0	30859000	0	1004900	0	1269100
Q8IYS2	Q8IYS2	Uncharacterized protein KIAA2013	KIAA2013	sp|Q8IYS2	2	2	2	0	0	0	2	0	0	0	2	0	0	0	2	4.3	69.156	0	6.1518	42247000	0	0	0	42247000	0	0	0	1737400
Q9NUL7	Q9NUL7	Probable ATP-dependent RNA helicase DDX28	DDX28	sp|Q9NUL	2	2	2	0	1	0	2	0	1	0	2	0	1	0	2	8.3	59.58	0	8.9296	42235000	0	4108700	0	38126000	0	643440	0	1286700
Q6Y1H2	Q6Y1H2	Very-long-chain (3R)-3-hydroxyacyl-CoA dehydrat	HACD2	sp|Q6Y1H2	2	2	2	0	2	0	2	0	2	0	2	0	2	0	2	7.5	28.368	0.002661	2.1802	42182000	0	14370000	0	27812000	0	1257600	0	1152900
P53365	P53365	Arfaptin-2	ARFIP2	sp|P53365	2	2	2	0	2	0	1	0	2	0	1	0	2	0	1	7.9	37.855	0	5.5436	42159000	0	27345000	0	14814000	0	2410400	0	0
Q9UDR5	Q9UDR5	Alpha-aminoadipic semialdehyde synthase, mitoc	AASS	sp|Q9UDR	3	3	3	0	2	0	1	0	2	0	1	0	2	0	1	5.6	102.13	0	12.761	42129000	0	21553000	0	20576000	0	1899900	0	0
P28065	P28065	Proteasome subunit beta type-9	PSMB9	sp|P28065	1	1	1	0	0	0	1	0	0	0	1	0	0	0	1	6.8	23.264	0.000247	3.6643	42074000	0	0	0	42074000	0	0	0	1730300
Q9UJ68	Q9UJ68	Mitochondrial peptide methionine sulfoxide redu	MSRA	sp|Q9UJ68	3	3	3	0	1	0	3	0	1	0	3	0	1	0	3	16.6	26.132	0	3.9568	42039000	0	3997300	0	38042000	0	524480	0	1392400
P32970	P32970	CD70 antigen	CD70	sp|P32970	1	1	1	0	0	0	1	0	0	0	1	0	0	0	1	7.3	21.118	0	4.0215	41845000	0	0	0	41845000	0	0	0	1720900
O60879	O60879	Protein diaphanous homolog 2	DIAPH2	sp|O60879	3	3	3	0	2	0	2	0	2	0	2	0	2	0	2	4.4	125.57	0	12.524	41712000	0	11457000	0	30255000	0	1637200	0	617000
Q8N9T8	Q8N9T8	Protein KRI1 homolog	KRI1	sp|Q8N9T8	3	3	3	0	3	3	3	0	3	3	3	0	3	3	3	6	82.597	0	7.4869	41674000	0	10718000	6803400	24152000	0	866310	4574200	1069400
Q9BUH6	Q9BUH6	Protein PAXX	C9orf142	sp|Q9BUH	1	1	1	0	1	0	1	0	1	0	1	0	1	0	1	5.9	21.639	0.000249	3.7359	41543000	0	27188000	0	14355000	0	2396600	0	590360
Q96EH3	Q96EH3	Mitochondrial assembly of ribosomal large subun	MALSU1	sp|Q96EH3	3	3	3	0	2	0	2	0	2	0	2	0	2	0	2	28.6	26.17	0	10.176	41537000	0	14474000	0	27063000	0	1104000	0	1284800
O75970	O75970	Multiple PDZ domain protein	MPDZ	sp|O75970	2	2	2	0	2	1	2	0	2	1	2	0	2	1	2	2.1	221.62	0	4.2636	41533000	0	10893000	5143800	25495000	0	1303700	3388900	772800
Q5BJF2	Q5BJF2	Transmembrane protein 97	TMEM97	sp|Q5BJF2	1	1	1	0	1	1	1	0	1	1	1	0	1	1	1	4.5	20.848	0.003081	2.101	41495000	0	34628000	1078900	5787800	0	3338300	430480	246650
Q8IWZ8	Q8IWZ8	SURP and G-patch domain-containing protein 1	SUGP1	sp|Q8IWZ8	2	2	2	0	1	0	2	0	1	0	2	0	1	0	2	5.1	72.47	0.001126	2.4083	41455000	0	10984000	0	30471000	0	0	0	1253100
P54619	P54619	5-AMP-activated protein kinase subunit gamma-1	PRKAG1	sp|P54619	2	2	2	0	2	0	0	0	2	0	0	0	2	0	0	7.6	37.579	0	4.9016	41406000	0	41406000	0	0	0	3649900	0	0
Q9BV81	Q9BV81	ER membrane protein complex subunit 6	EMC6	sp|Q9BV81	1	1	1	0	1	0	1	0	1	0	1	0	1	0	1	10.9	12.017	0.00116	2.8128	41302000	0	14456000	0	26847000	0	1274300	0	1104100
Q9NWT6	Q9NWT6	Hypoxia-inducible factor 1-alpha inhibitor	HIF1AN	sp|Q9NWT	2	2	2	0	2	0	2	0	2	0	2	0	2	0	2	8.3	40.285	0	8.2974	41205000	0	10028000	0	31177000	0	852430	0	1313700
O95749	O95749	Geranylgeranyl pyrophosphate synthase	GGPS1	sp|O95749	2	2	2	1	2	0	1	1	2	0	1	1	2	0	1	6	34.87	0.003291	2.0815	41174000	614790	35603000	0	4955600	594620	2857100	0	505290
O15031	O15031	Plexin-B2	PLXNB2	sp|O15031	3	3	3	0	3	0	2	0	3	0	2	0	3	0	2	3.3	205.12	0.001148	2.6301	41041000	0	16946000	0	24095000	0	998560	0	1486100
Q9Y2R4	Q9Y2R4	Probable ATP-dependent RNA helicase DDX52	DDX52	sp|Q9Y2R4	1	1	1	0	1	1	1	0	1	1	1	0	1	1	1	2.3	67.497	0.003287	2.0638	40854000	0	9951100	7202500	23700000	0	732500	5187300	772150
Q9P0S3;Q!	Q9P0S3;Q!	ORM1-like protein 1;ORM1-like protein 2	ORMDL1;C	sp|Q9P0S3	3	3	2	0	0	0	3	0	0	0	3	0	0	0	2	28.8	17.371	0	6.8403	40834000	0	0	0	40834000	0	0	0	1679300
Q2VPK5	Q2VPK5	Cytoplasmic tRNA 2-thiolation protein 2	CTU2	sp|Q2VPK5	2	2	2	0	2	0	2	0	2	0	2	0	2	0	2	6.4	56.107	0	6.4666	40787000	0	10994000	0	29793000	0	976260	0	1218100
P20338	P20338	Ras-related protein Rab-4A	RAB4A	sp|P20338	2	2	2	0	2	0	1	0	2	0	1	0	2	0	1	12.4	24.389	0	7.5533	40706000	0	33157000	0	7548500	0	2661700	0	571540
Q8WXF7	Q8WXF7	Atlastin-1	ATL1	sp|Q8WXF	3	3	3	0	1	0	3	0	1	0	3	0	1	0	3	5.7	63.543	0	5.0968	40551000	0	2941600	0	37609000	0	0	0	1546700
Q03518	Q03518	Antigen peptide transporter 1	TAP1	sp|Q03518	1	1	1	0	1	0	1	0	1	0	1	0	1	0	1	3.7	87.217	0	15.021	40446000	0	6456800	0	33989000	0	569160	0	1397800
Q9H4L7	Q9H4L7	SWI/SNF-related matrix-associated actin-depende	SMARCAD:	sp|Q9H4L7	4	4	4	0	2	0	4	0	2	0	4	0	2	0	4	5.3	117.4	0	11.342	40438000	0	6661400	0	33776000	0	920460	0	1055800
Q13586	Q13586	Stromal interaction molecule 1	STIM1	sp|Q13586	2	2	2	0	2	1	1	0	2	1	1	0	2	1	1	6	77.422	0.000485	3.4469	40364000	0	14107000	1228800	25028000	0	738710	1187100	1172700
Q8NFC6	Q8NFC6	Biorientation of chromosomes in cell division prot	BOD1L1	sp|Q8NFC(	4	4	4	0	3	0	4	0	3	0	4	0	3	0	4	2.3	330.46	0	12.578	40162000	0	11427000	0	28735000	0	1115800	0	1073200
Q96FV2	Q96FV2	Secernin-2	SCRN2	sp|Q96FV2	1	1	1	0	1	0	1	0	1	0	1	0	1	0	1	4.7	46.596	0	5.6173	40115000	0	10846000	0	29268000	0	956100	0	1203700
Q8WZA9	Q8WZA9	Immunity-related GTPase family Q protein	IRGQ	sp|Q8WZA	3	3	3	0	2	0	1	0	2	0	1	0	2	0	1	7.1	62.717	0	6.3205	39997000	0	30575000	0	9421600	0	0	0	387470
Q86V48	Q86V48	Leucine zipper protein 1	LUZP1	sp|Q86V48	6	6	6	0	2	5	3	0	2	5	3	0	2	5	3	9.1	120.27	0	9.2406	39967000	0	6577900	19011000	14378000	0	827230	12518000	600820
O43516	O43516	WAS/WASL-interacting protein family member 1	WIPF1	sp|O43516	3	3	3	0	1	0	3	0	1	0	3	0	1	0	3	7.2	51.258	0	8.3838	39942000	0	659830	0	39282000	0	184240	0	1489400
Q96FV9	Q96FV9	THO complex subunit 1	THOC1	sp|Q96FV9	3	3	3	0	2	0	3	0	2	0	3	0	2	0	3	8.4	75.665	0	4.269	39859000	0	13739000	0	26120000	0	1234400	0	1050900
A6NDU8	A6NDU8	UPF0600 protein C5orf51	C5orf51	sp|A6NDU	1	1	1	0	0	0	1	0	0	0	1	0	0	0	1	9.9	33.62	0.000486	3.4675	39834000	0	0	0	39834000	0	0	0	1638200
Q96BR5	Q96BR5	Cytochrome c oxidase assembly factor 7	COA7	sp|Q96BR5	3	3	3	0	3	0	0	0	3	0	0	0	3	0	0	21.6	25.709	0	6.362	39820000	0	39820000	0	0	0	3510100	0	0
Q9Y5U2	Q9Y5U2	Protein TSSC4	TSSC4	sp|Q9Y5U2	2	2	2	0	2	2	2	0	2	2	2	0	2	2	2	6.7	34.325	0.000247	3.6699	39786000	0	19411000	1492700	18882000	0	1803700	896900	790020
Q5T749	Q5T749	Keratinocyte proline-rich protein	KPRP	sp|Q5T749	1	1	1	0	1	1	1	0	1	1	1	0	1	1	1	1.6	64.135	0.007439	1.669	39765000	0	10787000	24537000	4441000	0	353920	17113000	155110
Q9UIW2;Q	Q9UIW2;Q	Plexin-A1;Plexin-A4	PLXNA1;PL	sp|Q9UIW	2	2	2	0	0	0	2	0	0	0	2	0	0	0	2	1.5	211.06	0	6.2781	39693000	0	0	0	39693000	0	0	0	1632400
Q9H9Q4	Q9H9Q4	Non-homologous end-joining factor 1	NHEJ1	sp|Q9H9Q	2	2	2	0	1	0	1	0	1	0	1	0	1	0	1	11.7	33.337	0	10.027	39687000	0	26139000	0	13548000	0	2304100	0	0
Q8NE86	Q8NE86	Calcium uniporter protein, mitochondrial	MCU	sp|Q8NE8(	2	2	2	0	1	0	2	0	1	0	2	0	1	0	2	8	39.866	0	3.9001	39685000	0	3295700	0	36389000	0	708320	0	1078700
P07305	P07305	Histone H1.0;Histone H1.0, N-terminally processe	H1F0	sp|P07305	1	1	1	1	1	1	0	1	1	1	0	1	1	1	0	6.7	20.863	0	3.8873	39505000	27695000	8681900	3128100	0	23306000	836330	6420700	0
Q9H410	Q9H410	Kinetochore-associated protein DSN1 homolog	DSN1	sp|Q9H41(	2	2	2	1	2	0	1	1	2	0	1	1	2	0	1	10.7	40.067	0	5.775	39458000	1963200	18598000	0	18897000	2873400	684570	0	821840
Q8ND04	Q8ND04	Protein SMG8	SMG8	sp|Q8ND0	3	3	3	0	2	1	3	0	2	1	3	0	2	1	3	5.7	109.68	0	6.9177	39405000	0	6028200	257390	33119000	0	845390	0	1048100
Q9H2D1	Q9H2D1	Mitochondrial folate transporter/carrier	SLC25A32	sp|Q9H2D	1	1	1	0	0	0	1	0	0	0	1	0	0	0	1	7.9	35.407	0	5.6191	39400000	0	0	0	39400000	0	0	0	1620400
Q8NDI1	Q8NDI1	EH domain-binding protein 1	EHBP1	sp|Q8NDI1	4	4	4	0	1	0	4	0	1	0	4	0	1	0	4	6.1	140.02	0	11.132	39274000	0	2398600	0	36875000	0	0	0	1516500
P05165	P05165	Propionyl-CoA carboxylase alpha chain, mitochon	PCCA	sp|P05165	2	2	2	0	1	0	2	0	1	0	2	0	1	0	2	4.3	80.058	0.001156	2.7603	39263000	0	4371500	0	34891000	0	580130	0	1240200
P56962	P56962	Syntaxin-17	STX17	sp|P56962	1	1	1	0	1	1	1	0	1	1	1	0	1	1	1	6.6	33.403	0	11.417	39232000	0	17982000	1343300	19906000	0	1585100	902700	818650
O60825;Q:	O60825;Q:	6-phosphofructo-2-kinase/fructose-2,6-bisphosph	PFKFB2;PF	sp|O60825	2	1	1	2	1	0	1	1	1	0	1	1	1	0	1	4	58.476	0.002444	2.2096	39228000	21798000	5664300	0	11765000	21798000	499300	0	483860
Q8WVX9	Q8WVX9	Fatty acyl-CoA reductase 1	FAR1	sp|Q8WVX	2	2	2	0	2	0	1	0	2	0	1	0	2	0	1	5.2	59.356	0	4.5965	39210000	0	15052000	0	24158000	0	780380	0	1539900
O95376	O95376	E3 ubiquitin-protein ligase ARIH2	ARIH2	sp|O95376	1	1	1	0	1	0	1	0	1	0	1	0	1	0	1	3.4	57.818	0	5.4886	39205000	0	11348000	0	27857000	0	1000300	0	1145600
Q6UWP8	Q6UWP8	Suprabasin	SBSN	sp|Q6UWI	4	4	4	0	2	4	2	0	2	4	2	0	2	4	2	20.7	60.54	0	11.106	39201000	0	3518800	31151000	4531300	0	186690	21103000	140160
O96011	O96011	Peroxisomal membrane protein 11B	PEX11B	sp|O96011	3	3	3	0	3	1	1	0	3	1	1	0	3	1	1	12.4	28.431	0	5.9458	39152000	0	23217000	926550	15008000	0	1491700	852590	942150

Q9P291	Q9P291	Armadillo repeat-containing X-linked protein 1	ARMCX1	sp|Q9P291	3	3	3	0	0	0	3	0	0	0	3	0	0	0	3	8.4	49.18	0	7.5618	39077000	0	0	0	39077000	0	0	0	1607100
Q12802	Q12802	A-kinase anchor protein 13	AKAP13	sp|Q12802	4	4	4	0	1	0	4	0	1	0	4	0	1	0	4	2.1	307.55	0	17.629	38896000	0	2812400	0	36083000	0	406400	0	1325500
P46100	P46100	Transcriptional regulator ATRX	ATRX	sp|P46100	6	6	6	0	6	2	1	0	6	2	1	0	6	2	1	3.2	282.58	0	11.849	38868000	0	34207000	1899900	2761000	0	2857100	1322100	226470
P78381	P78381	UDP-galactose translocator	SLC35A2	sp|P78381	2	2	2	0	1	0	2	0	1	0	2	0	1	0	2	10.9	41.307	0	6.8599	38800000	0	3063500	0	35737000	0	446990	0	1292700
P53384	P53384	Cytosolic Fe-S cluster assembly factor NUBP1	NUBP1	sp|P53384	2	2	2	1	1	0	2	1	1	0	2	1	1	0	2	11.9	34.534	0	4.3869	38713000	8936400	4515600	0	25261000	6870100	2730800	0	772450
Q96IZ0	Q96IZ0	PRKC apoptosis WT1 regulator protein	PAWR	sp|Q96IZ0	4	4	4	0	4	2	0	0	4	2	0	0	4	2	0	16.5	36.567	0	8.389	38698000	0	35538000	3160300	0	0	2384300	2872100	0
Q02127	Q02127	Dihydroorotate dehydrogenase (quinone), mitoch	DHODH	sp|Q02127	3	3	3	0	3	1	1	0	3	1	1	0	3	1	1	10.6	42.867	0	6.8445	38520000	0	25972000	2071500	10477000	0	1400600	1581800	1130000
Q13685	Q13685	Angio-associated migratory cell protein	AAMP	sp|Q13685	3	3	3	0	1	0	3	0	1	0	3	0	1	0	3	9.9	46.75	0	18.002	38466000	0	4566100	0	33900000	0	1078000	0	718650
Q6UXH1	Q6UXH1	Cysteine-rich with EGF-like domain protein 2	CRELD2	sp|Q6UXH	3	3	3	0	3	0	3	0	3	0	3	0	3	0	3	8.5	38.191	0	6.5974	38354000	0	15314000	0	23040000	0	1233300	0	1064200
O95479	O95479	GDH/6PGL endoplasmic bifunctional protein;Gluc	H6PD	sp|O95479	3	3	3	0	0	1	3	0	0	1	3	0	0	1	3	4.8	88.892	0	9.6912	38325000	0	0	5698600	32627000	0	0	4658400	512840
Q96CM8	Q96CM8	Acyl-CoA synthetase family member 2, mitochonc	ACSF2	sp|Q96CM	2	2	2	0	0	0	2	0	0	0	2	0	0	0	2	6.2	68.124	0	13.4	38272000	0	0	0	38272000	0	0	0	1574000
Q6P1X5	Q6P1X5	Transcription initiation factor TFIID subunit 2	TAF2	sp|Q6P1X5	3	3	3	0	3	0	1	0	3	0	1	0	3	0	1	3.4	136.97	0	5.9	38163000	0	31357000	0	6805900	0	1320800	0	1723200
Q8NC96	Q8NC96	Adaptin ear-binding coat-associated protein 1	NECAP1	sp|Q8NC9	2	2	2	0	2	0	2	0	2	0	2	0	2	0	2	11.6	29.737	0.001142	2.5804	38160000	0	17890000	0	20271000	0	1700400	0	710180
Q9Y3C1	Q9Y3C1	Nucleolar protein 16	NOP16	sp|Q9Y3C1	3	3	3	0	3	1	3	0	3	1	3	0	3	1	3	14	21.188	0.000944	3.0872	38109000	0	18403000	2685400	17020000	0	1253500	2416900	456320
Q5TA45	Q5TA45	Integrator complex subunit 11	CPSF3L	sp|Q5TA45	2	2	2	0	1	0	2	0	1	0	2	0	1	0	2	4.7	67.662	0.001147	2.6236	37931000	0	9016600	0	28914000	0	794800	0	1189100
O14966;P5	O14966	Ras-related protein Rab-7L1	RAB29	sp|O14966	3	2	2	0	0	0	3	0	0	0	2	0	0	0	2	14.8	23.155	0	5.0503	37867000	0	0	0	37867000	0	0	0	1557300
Q9NUL3	Q9NUL3	Double-stranded RNA-binding protein Staufen ho	STAU2	sp|Q9NUL	4	2	2	1	4	4	4	1	2	2	2	1	2	2	2	7	62.608	0	5.9763	37849000	1059800	12721000	4404500	19664000	1682400	862150	2766500	638650
Q8NI99	Q8NI99	Angiopoietin-related protein 6	ANGPTL6	sp|Q8NI99	1	1	1	0	1	1	1	0	1	1	1	0	1	1	1	2.8	51.694	0.000486	3.4674	37639000	0	10168000	10653000	16818000	0	1122500	5816600	1807900
O14936	O14936	Peripheral plasma membrane protein CASK	CASK	sp|O14936	3	3	3	0	2	1	3	0	2	1	3	0	2	1	3	3.8	105.12	0.000248	3.7355	37596000	0	7044100	1025800	29526000	0	780810	1165000	578750
P43378	P43378	Tyrosine-protein phosphatase non-receptor type	PTPN9	sp|P43378	3	3	3	0	2	0	3	0	2	0	3	0	2	0	3	5.9	68.019	0	5.5392	37561000	0	5784200	0	31777000	0	576520	0	1240200
Q9NPI6	Q9NPI6	mRNA-decapping enzyme 1A	DCP1A	sp|Q9NPI6	3	3	3	1	2	0	3	1	2	0	3	1	2	0	3	6.5	63.278	0	5.497	37512000	279770	18093000	0	19139000	418430	1631500	0	611840
Q8NEB9	Q8NEB9	Phosphatidylinositol 3-kinase catalytic subunit typ	PIK3C3	sp|Q8NEB9	3	3	3	0	1	1	2	0	1	1	2	0	1	1	2	3.6	101.55	0	7.8074	37408000	0	3615600	3141900	30650000	0	847470	0	731750
Q9H0W9	Q9H0W9	Ester hydrolase C11orf54	C11orf54	sp|Q9H0W	2	2	2	0	2	0	0	0	2	0	0	0	2	0	0	12.1	35.117	0.001562	2.2664	37386000	0	37386000	0	0	0	3295500	0	0
Q9P0J7	Q9P0J7	E3 ubiquitin-protein ligase KCMF1	KCMF1	sp|Q9P0J7	2	2	2	0	2	0	2	0	2	0	2	0	2	0	2	10.2	41.945	0	6.2211	37312000	0	8278300	0	29034000	0	739040	0	1184700
O94888	O94888	UBX domain-containing protein 7	UBXN7	sp|O94888	2	2	2	0	2	0	0	0	2	0	0	0	2	0	0	6.3	54.862	0	6.8311	37312000	0	37312000	0	0	0	3289000	0	0
Q96LR5;Q9	Q96LR5;Q9	Ubiquitin-conjugating enzyme E2 E2;Ubiquitin-co	UBE2E2;UE	sp|Q96LR5	2	1	1	0	2	0	2	0	1	0	1	0	1	0	1	12.9	22.255	0	7.0748	37206000	0	14456000	0	22750000	0	1274300	0	935610
P30049	P30049	ATP synthase subunit delta, mitochondrial	ATP5D	sp|P30049	1	1	1	1	1	0	0	1	1	0	0	1	1	0	0	8.3	17.49	0	4.8655	37186000	5170600	32016000	0	0	5170600	2822200	0	0
Q86YV9	Q86YV9	Hermansky-Pudlak syndrome 6 protein	HPS6	sp|Q86YV9	2	2	2	0	1	0	2	0	1	0	2	0	1	0	2	3.2	82.974	0.000249	3.7491	37139000	0	9609700	0	27529000	0	1080700	0	898510
Q01484	Q01484	Ankyrin-2	ANK2	sp|Q01484	6	5	5	0	2	1	6	0	1	0	5	0	1	0	5	2	433.71	0	7.5752	37130000	0	3319000	0	33811000	0	584170	0	1098900
Q9UI14	Q9UI14	Prenylated Rab acceptor protein 1	RABAC1	sp|Q9UI14	1	1	1	0	1	0	1	0	1	0	1	0	1	0	1	7.6	20.648	0	4.8753	36894000	0	11405000	0	25489000	0	1005300	0	1048200
Q5VT25	Q5VT25	Serine/threonine-protein kinase MRCK alpha	CDC42BPA	sp|Q5VT25	5	4	4	0	2	3	3	0	2	2	2	0	2	2	2	4	197.3	0	9.1762	36846000	0	11729000	15400000	9716600	0	2422200	8960400	0
Q9Y296	Q9Y296	Trafficking protein particle complex subunit 4	TRAPPC4	sp|Q9Y296	2	2	2	0	1	0	2	0	1	0	2	0	1	0	2	11.4	24.34	0	5.6997	36834000	0	10719000	0	26115000	0	0	0	1074000
Q02153	Q02153	Guanylate cyclase soluble subunit beta-1	GUCY1B3	sp|Q02153	2	2	2	0	0	0	2	0	0	0	2	0	0	0	2	3.7	70.514	0	5.5469	36776000	0	0	0	36776000	0	0	0	1512400
O15164	O15164	Transcription intermediary factor 1-alpha	TRIM24	sp|O15164	3	3	3	0	2	0	3	0	2	0	3	0	2	0	3	4.7	116.83	0	4.2664	36655000	0	10067000	0	26588000	0	963970	0	1016900
P40818	P40818	Ubiquitin carboxyl-terminal hydrolase 8	USP8	sp|P40818	1	1	1	0	1	1	1	0	1	1	1	0	1	1	1	1.5	127.52	0	5.1666	36611000	0	9239200	2193600	25178000	0	814420	1474100	1035500
Q9GZT9;Q	Q9GZT9	Egl nine homolog 1	EGLN1	sp|Q9GZT9	3	3	3	0	3	1	1	0	3	1	1	0	3	1	1	5.6	46.02	0	4.8777	36589000	0	30849000	325390	5415100	0	2730200	214220	216190
P24844	P24844	Myosin regulatory light polypeptide 9	MYL9	sp|P24844	6	1	1	3	5	6	6	0	0	1	1	0	0	1	1	44.8	19.827	0	9.0883	36577000	0	0	2405500	34172000	0	0	1616500	1405300
Q9GZU8	Q9GZU8	Protein FAM192A	FAM192A	sp|Q9GZU	4	4	4	0	4	0	3	0	4	0	3	0	4	0	3	12.6	28.912	0	81.364	36473000	0	15229000	0	21244000	0	1378900	0	837210
Q8IZQ5	Q8IZQ5	Selenoprotein H	SELH	sp|Q8IZQ5	1	1	1	0	1	1	1	0	1	1	1	0	1	1	1	7.4	13.453	0.003083	2.1103	36411000	0	3390300	2851700	30169000	0	407740	2217300	830800
P42574	P42574	Caspase-3;Caspase-3 subunit p17;Caspase-3 subu	CASP3	sp|P42574	1	1	1	0	1	0	1	0	1	0	1	0	1	0	1	6.9	31.608	0.001158	2.7791	36372000	0	16498000	0	19874000	0	1454300	0	817330
O15321	O15321	Transmembrane 9 superfamily member 1	TM9SF1	sp|O15321	2	2	2	0	2	0	2	0	2	0	2	0	2	0	2	3.3	68.86	0.004545	1.8832	36347000	0	9058400	0	27288000	0	0	0	1122300
Q9NX24	Q9NX24	H/ACA ribonucleoprotein complex subunit 2	NHP2	sp|Q9NX24	1	1	1	0	1	1	1	0	1	1	1	0	1	1	1	12.4	17.201	0.001142	2.5789	36346000	0	11849000	7804800	16693000	0	1044400	5244800	686500
Q9HA65	Q9HA65	TBC1 domain family member 17	TBC1D17	sp|Q9HA65	3	3	3	0	2	0	3	0	2	0	3	0	2	0	3	5.4	72.727	0	6.8099	36257000	0	7041100	0	29216000	0	715640	0	1106500
Q9H4G4	Q9H4G4	Golgi-associated plant pathogenesis-related prote	GLIPR2	sp|Q9H4G	2	2	2	0	2	0	2	0	2	0	2	0	2	0	2	16.9	17.218	0.000483	3.3788	36251000	0	10600000	0	25650000	0	912750	0	1076600
Q96P63	Q96P63	Serpin B12	SERPINB12	sp|Q96P63	4	4	4	0	1	4	0	0	1	4	0	0	1	4	0	10.1	46.276	0	7.0826	36220000	0	6146900	30073000	0	0	689460	20061000	0
Q9UK59	Q9UK59	Lariat debranching enzyme	DBR1	sp|Q9UK59	2	2	2	0	1	1	2	0	1	1	2	0	1	1	2	5.7	61.554	0	5.712	36208000	0	0	1331200	34877000	0	0	1317500	1011500
O95825	O95825	Quinone oxidoreductase-like protein 1	CRYZL1	sp|O95825	1	1	1	0	1	0	0	0	1	0	0	0	1	0	0	6	38.696	0	13.654	36105000	0	36105000	0	0	0	3182600	0	0
Q6UVJ0	Q6UVJ0	Spindle assembly abnormal protein 6 homolog	SASS6	sp|Q6UVJ0	2	2	2	0	2	0	0	0	2	0	0	0	2	0	0	4.1	74.396	0	14.004	36102000	0	36102000	0	0	0	3182400	0	0
Q9BT22	Q9BT22	Chitobiosyldiphosphodolichol beta-mannosyltrans	ALG1	sp|Q9BT22	2	2	2	0	2	0	2	0	2	0	2	0	2	0	2	5.8	52.518	0	5.2625	36054000	0	20398000	0	15656000	0	1741700	0	700210
Q96GG9	Q96GG9	DCN1-like protein 1	DCUN1D1	sp|Q96GG	2	2	2	1	1	0	2	1	1	0	2	1	1	0	2	5.8	30.124	0.00115	2.6774	36040000	875160	5404800	0	29760000	1097200	809510	0	668810
Q96BP2	Q96BP2	Coiled-coil-helix-coiled-coil-helix domain-containi	CHCHD1	sp|Q96BP2	1	1	1	0	1	0	1	0	1	0	1	0	1	0	1	13.6	13.475	0	4.5624	36036000	0	23328000	0	12708000	0	2056400	0	522620
Q9H6X2;P5	Q9H6X2;P5	Anthrax toxin receptor 1;Anthrax toxin receptor 2	ANTXR1;AI	sp|Q9H6X2	2	2	2	0	0	0	2	0	0	0	2	0	0	0	2	3.7	62.788	0.000246	3.6153	35986000	0	0	0	35986000	0	0	0	1479900
P52943	P52943	Cysteine-rich protein 2	CRIP2	sp|P52943	1	1	1	0	1	0	1	0	1	0	1	0	1	0	1	15.4	22.492	0	5.6658	35936000	0	7592300	0	28344000	0	669250	0	1165700
Q96SZ5	Q96SZ5	2-aminoethanethiol dioxygenase	ADO	sp|Q96SZ5	4	4	4	1	4	1	1	1	4	1	1	1	4	1	1	14.8	29.751	0	5.5053	35825000	1290200	29746000	572550	4216300	1214400	1987800	742340	525820
P29372	P29372	DNA-3-methyladenine glycosylase	MPG	sp|P29372	3	3	3	0	2	0	1	0	2	0	1	0	2	0	1	16.1	32.868	0	7.1053	35780000	0	30921000	0	4859400	0	2725600	0	0
Q13153	Q13153	Serine/threonine-protein kinase PAK 1	PAK1	sp|Q13153	5	2	2	0	5	0	4	0	2	0	1	0	2	0	1	12.8	60.646	0.000487	3.4842	35732000	0	22257000	0	13476000	0	1961900	0	0
O14531	O14531	Dihydropyrimidinase-related protein 4	DPYSL4	sp|O14531	4	3	3	0	4	0	1	0	3	0	0	0	3	0	0	8	61.877	0.001178	3.0643	35636000	0	35636000	0	0	0	3141300	0	0
Q96MX6	Q96MX6	WD repeat-containing protein 92	WDR92	sp|Q96MX	2	2	2	0	2	0	0	0	2	0	0	0	2	0	0	10.1	39.74	0.009253	1.5512	35618000	0	35618000	0	0	0	3139700	0	0
P27448	P27448	MAP/microtubule affinity-regulating kinase 3	MARK3	sp|P27448	3	3	3	1	1	3	3	1	1	3	3	1	1	3	3	4.9	84.428	0	6.7422	35553000	654810	3330800	6711300	24856000	1901600	396010	3742400	440520
Q8WWH5	Q8WWH5	Probable tRNA pseudouridine synthase 1	TRUB1	sp|Q8WW	3	3	3	0	3	0	0	0	3	0	0	0	3	0	0	9.2	37.252	0	8.7362	35513000	0	35513000	0	0	0	3130500	0	0
Q9BVG9	Q9BVG9	Phosphatidylserine synthase 2	PTDSS2	sp|Q9BVG	2	2	2	0	1	1	2	0	1	1	2	0	1	1	2	4.7	56.252	0.000483	3.4035	35507000	0	9077600	1213400	25216000	0	644510	1186700	821300
Q6ZNB6	Q6ZNB6	NF-X1-type zinc finger protein NFXL1	NFXL1	sp|Q6ZNB	2	2	2	0	2	1	2	0	2	1	2	0	2	1	2	4.3	101.34	0.000718	3.2923	35488000	0	8039800	2722600	24726000	0	1381500	1621500	552100
Q8WVM0	Q8WVM0	Dimethyladenosine transferase 1, mitochondrial	TFB1M	sp|Q8WVM	2	2	2	0	0	1	2	0	0	1	2	0	0	1	2	8.7	39.542	0	13.852	35449000	0	0	2678800	32770000	0	0	2080800	1067000
Q9NVP2	Q9NVP2	Histone chaperone ASF1B	ASF1B	sp|Q9NVP	1	1	1	0	1	0	1	0	1	0	1	0	1	0	1	13.9	22.433	0.00135	2.3894	35410000	0	10763000	0	24647000	0	948770	0	1013600
Q02539	Q02539	Histone H1.1	HIST1H1A	sp|Q02539	5	2	2	2	3	2	5	0	0	0	2	0	0	0	2	24.7	21.842	0.001165	2.8855	35321000	0	0	0	35321000	0	0	0	1452600
P50452	P50452	Serpin B8	SERPINB8	sp|P50452	3	2	2	0	1	0	3	0	0	0	2	0	0	0	2	9.1	42.766	0	6.214	35300000	0	0	0	35300000	0	0	0	1451700
Q9NY61	Q9NY61	Protein AATF	AATF	sp|Q9NY61	2	2	2	0	0	2	2	0	0	2	2	0	0	2	2	8.4	63.132	0.000485	3.4474	35241000	0	0	10174000	25068000	0	0	6073600	1794000
Q9H6E4	Q9H6E4	Coiled-coil domain-containing protein 134	CCDC134	sp|Q9H6E4	2	2	2	0	2	0	2	0	2	0	2	0	2	0	2	8.7	26.56	0.001349	2.3845	35209000	0	13769000	0	21440000	0	1193300	0	902240
Q5ZPR3	Q5ZPR3	CD276 antigen	CD276	sp|Q5ZPR3	1	1	1	0	0	0	1	0	0	0	1	0	0	0	1	9.7	57.235	0	9.2322	35190000	0	0	0	35190000	0	0	0	1447200
Q9NVC6	Q9NVC6	Mediator of RNA polymerase II transcription subu	MED17	sp|Q9NVC	1	1	1	0	1	1	1	0	1	1	1	0	1	1	1	3.7	72.889	0.001136	2.5042	35183000	0	12155000	2685700	20342000	0	1095200	1557300	1060300
Q6NUQ1	Q6NUQ1	RAD50-interacting protein 1	RINT1	sp|Q6NUC	2	2	2	0	2	0	2	0	2	0	2	0	2	0	2	4.3	90.631	0	4.6128	35156000	0	23824000	0	11332000	0	2129200	0	436910
P22681	P22681	E3 ubiquitin-protein ligase CBL	CBL	sp|P22681	3	3	3	0	2	0	3	0	2	0	3	0	2	0	3	5.2	99.632	0.000246	3.6248	35086000	0	8970400	0	26116000	0	1049400	0	815330
Q9NVS2	Q9NVS2	28S ribosomal protein S18a, mitochondrial	MRPS18A	sp|Q9NVS2	2	2	2	0	2	0	1	0	2	0	1	0	2	0	1	12.8	22.184	0.000943	3.0703	35077000	0	21427000	0	13650000	0	1226000	0	1224100
Q9ULJ3	Q9ULJ3	Zinc finger and BTB domain-containing protein 21	ZBTB21	sp|Q9ULJ3	3	3	3	0	3	1	2	0	3	1	2	0	3	1	2	4.7	118.87	0	8.9466	34988000	0	16078000	1420300	17489000	0	1026400	1353500	711110
P07992	P07992	DNA excision repair protein ERCC-1	ERCC1	sp|P07992	1	1	1	0	1	0	1	0	1	0	1	0	1	0	1	7.1	32.562	0.007849	1.6447	34942000	0	7726900	0	27215000	0	681120	0	1119200
P10398	P10398	Serine/threonine-protein kinase A-Raf	ARAF	sp|P10398	2	2	2	0	1	0	2	0	1	0	2	0	1	0	2	5.1	67.585	0.000946	3.1081	34917000	0	3169800	0	31747000	0	653020	0	932010
Q9NWS8	Q9NWS8	Required for meiotic nuclear division protein 1 hc	RMND1	sp|Q9NWS	2	2	2	0	2	0	1	0	2	0	1	0	2	0	1	4.2	51.603	0	4.2134	34908000	0	16361000	0	18547000	0	757710	0	1447300
Q15118	Q15118	[Pyruvate dehydrogenase (acetyl-transferring)] ki	PDK1	sp|Q15118	1	1	1	0	1	0	0	0	1	0	0	0	1	0	0	2.5	49.244	0.00116	2.8111	34824000	0	34824000	0	0	0	3069700	0	0
Q969S0	Q969S0	UDP-xylose and UDP-N-acetylglucosamine transp	SLC35B4	sp|Q969S0	1	1	1	0	1	0	1	0	1	0	1	0	1	0	1	3	37.423	0.000944	3.0818	34793000	0	6439900	0	28353000	0	567670	0	1166100
Q09472;Q9	Q09472	Histone acetyltransferase p300	EP300	sp|Q09472	3	3	3	0	3	0	3	0	3	0	3	0	3	0	3	1.3	264.16	0	8.1905	34741000	0	11628000	0	23112000	0	1004900	0	970670
Q8NEC7	Q8NEC7	Glutathione S-transferase C-terminal domain-con	GSTCD	sp|Q8NEC7	1	1	1	0	1	0	1	0	1	0	1	0	1	0	1	1.4	71.078	0.001137	2.5131	34723000	0	7653200	0	27070000	0	674620	0	1113300
Q15418	Q15418	Ribosomal protein S6 kinase alpha-1	RPS6KA1	sp|Q15418	9	2	2	0	6	1	9	0	2	0	2	0	2	0	2	13.7	82.722	0	5.4095	34720000	0	10077000	0	24644000	0	876450	0	1025300
Q96SK2	Q96SK2	Transmembrane protein 209	TMEM209	sp|Q96SK2	3	3	3	0	0	0	3	0	0	0	3	0	0	0	3	7.3	62.921	0	6.4263	34712000	0	0	0	34712000	0	0	0	1427600
P34059	P34059	N-acetylgalactosamine-6-sulfatase	GALNS	sp|P34059	1	1	1	0	0	0	1	0	0	0	1	0	0	0	1	2.9	58.025	0	5.0221	34704000	0	0	0	34704000	0	0	0	1427200
P17568	P17568	NADH dehydrogenase [ubiquinone] 1 beta subcor	NDUFB7	sp|P17568	2	2	2	0	2	0	2	0	2	0	2	0	2	0	2	19.7	16.402	0	4.7431	34698000	0	14988000	0	19710000	0	1321200	0	810590
Q86TG7	Q86TG7	Retrotransposon-derived protein PEG10	PEG10	sp|Q86TG7	3	3	3	0	0	1	3	0	0	1	3	0	0	1	3	7.6	80.172	0	6.3833	34584000	0	0	1155400	33428000	0	0	1114600	1036600
A2NJV5;A0	A2NJV5;A0	Ig kappa chain V-II region RPMI 6410;Ig kappa cha	IGKV A18;I	sp|A2NJV5	1	1	1	1	0	1	0	1	0	1	0	1	0	1	0	10.8	13.085	0.001173	3.0135	34477000	26836000	0	7641400	0	26836000	0	5135000	0
Q969E8	Q969E8	Pre-rRNA-processing protein TSR2 homolog	TSR2	sp|Q969E8	1	1	1	0	1	0	0	0	1	0	0	0	1	0	0	6.3	20.894	0.001163	2.8477	34446000	0	34446000	0	0	0	3036400	0	0
Q6IBW4	Q6IBW4	Condensin-2 complex subunit H2	NCAPH2	sp|Q6IBW4	1	1	1	0	1	0	1	0	1	0	1	0	1	0	1	3.5	68.226	0.001348	2.3778	34425000	0	22612000	0	11814000	0	1993200	0	485850

Q9HAD4	Q9HAD4	WD repeat-containing protein 41	WDR41	sp|Q9HAD	1	1	1	0	1	0	1	0	1	0	1	0	1	0	1	3.5	51.727	0	3.8186	34310000	0	9266000	0	25044000	0	816790	0	1030000
P23443	P23443	Ribosomal protein S6 kinase beta-1	RPS6KB1	sp|P23443	1	1	1	0	1	0	1	0	1	0	1	0	1	0	1	3.4	59.139	0.000948	3.1458	34270000	0	20291000	0	13979000	0	1788600	0	574900
Q9Y639	Q9Y639	Neuroplastin	NPTN	sp|Q9Y639	1	1	1	0	1	0	1	0	1	0	1	0	1	0	1	4.3	44.387	0	6.8483	34256000	0	3574100	0	30682000	0	315050	0	1261800
O00422	O00422	Histone deacetylase complex subunit SAP18	SAP18	sp|O00422	2	2	2	0	2	0	0	0	2	0	0	0	2	0	0	20.3	17.561	0	13.12	34252000	0	34252000	0	0	0	3019300	0	0
Q53F19	Q53F19	Uncharacterized protein C17orf85	C17orf85	sp|Q53F19	2	2	2	0	2	1	1	0	2	1	1	0	2	1	1	4.2	70.592	0.001173	3.0154	34220000	0	24731000	2927800	6561000	0	1832700	1890500	694180
P49917	P49917	DNA ligase 4	LIG4	sp|P49917	2	2	2	0	0	0	2	0	0	0	2	0	0	0	2	2.4	103.97	0	4.3125	34200000	0	0	0	34200000	0	0	0	1406500
Q9BXB7	Q9BXB7	Spermatogenesis-associated protein 16	SPATA16	sp|Q9BXB7	1	1	1	0	1	0	1	0	1	0	1	0	1	0	1	1.8	65.263	0.008261	1.6209	34180000	0	10406000	0	23774000	0	917260	0	977730
O14734	O14734	Acyl-coenzyme A thioesterase 8	ACOT8	sp|O14734	1	1	1	0	0	0	1	0	0	0	1	0	0	0	1	5	35.914	0	5.3296	34099000	0	0	0	34099000	0	0	0	1402300
Q9Y618	Q9Y618	Nuclear receptor corepressor 2	NCOR2	sp|Q9Y618	4	4	4	0	2	1	4	0	2	1	4	0	2	1	4	2.3	273.65	0	4.5368	34093000	0	5515800	705870	27871000	0	0	0	1146200
Q9NYJ8	Q9NYJ8	TGF-beta-activated kinase 1 and MAP3K7-binding	TAB2	sp|Q9NYJ8	3	3	3	1	3	1	3	1	3	1	3	1	3	1	3	6.1	76.493	0	6.1123	34087000	1682300	7842100	3949600	20613000	2133800	774680	2680300	286610
Q15048	Q15048	Leucine-rich repeat-containing protein 14	LRRC14	sp|Q15048	1	1	1	0	1	1	1	0	1	1	1	0	1	1	1	1.4	54.513	0.008639	1.5763	34056000	0	16538000	0	17518000	0	1457800	0	720460
Q7Z4S6;O7	Q7Z4S6	Kinesin-like protein KIF21A	KIF21A	sp|Q7Z4S6	3	2	2	0	2	0	3	0	2	0	2	0	2	0	2	3	187.18	0	5.0769	33975000	0	13944000	0	20031000	0	1228500	0	824430
Q70Z53	Q70Z53	Protein FRA10AC1	FRA10AC1	sp|Q70Z53	1	1	1	0	1	1	1	0	1	1	1	0	1	1	1	4.4	37.548	0.002225	2.2355	33955000	0	15013000	3545700	15396000	0	1025700	2604100	709460
P39210	P39210	Protein Mpv17	MPV17	sp|P39210	2	2	2	0	0	0	2	0	0	0	2	0	0	0	2	17.6	19.733	0	35	33863000	0	0	0	33863000	0	0	0	1392600
P54802	P54802	Alpha-N-acetylglucosaminidase;Alpha-N-acetylglu	NAGLU	sp|P54802	1	1	1	0	0	1	1	0	0	1	1	0	0	1	1	3	82.265	0.004737	1.8111	33843000	0	0	9439100	24403000	0	0	6343100	1003600
Q96K21	Q96K21	Abscission/NoCut checkpoint regulator	ZFYVE19	sp|Q96K21	2	2	2	0	1	0	2	0	1	0	2	0	1	0	2	5.1	51.546	0	6.4677	33821000	0	2561000	0	31260000	0	341290	0	1170100
P18846;P1	P18846;P1	Cyclic AMP-dependent transcription factor ATF-1;	ATF1;CREE	sp|P18846	2	2	2	1	1	1	2	1	1	1	2	1	1	1	2	12.9	29.232	0.001133	2.4757	33776000	135230	19874000	1524200	12243000	0	0	0	503500
Q03154	Q03154	Aminoacylase-1	ACY1	sp|Q03154	2	2	2	0	1	0	2	0	1	0	2	0	1	0	2	4.9	45.884	0.000488	3.5195	33722000	0	4771300	0	28951000	0	0	0	1190600
Q92743	Q92743	Serine protease HTRA1	HTRA1	sp|Q92743	2	2	2	0	1	2	2	0	1	2	2	0	1	2	2	3.8	51.286	0	3.9794	33707000	0	1738900	5815900	26152000	0	364590	3727300	1045100
Q15633	Q15633	RISC-loading complex subunit TARBP2	TARBP2	sp|Q15633	2	2	2	0	2	1	2	0	2	1	2	0	2	1	2	9.3	39.039	0	10.111	33707000	0	13624000	3740300	16343000	0	1660200	2227800	498400
Q4ZIN3	Q4ZIN3	Membralin	TMEM259	sp|Q4ZIN3	1	1	1	0	1	0	1	0	1	0	1	0	1	0	1	2.9	67.888	0.001569	2.3386	33569000	0	2556400	0	31013000	0	225340	0	1275400
Q9ULW0	Q9ULW0	Targeting protein for Xklp2	TPX2	sp|Q9ULW	5	5	5	0	2	4	2	0	2	4	2	0	2	4	2	7.2	85.652	0	5.6068	33567000	0	6818500	10778000	15970000	0	968520	6773400	758700
Q9UP95	Q9UP95	Solute carrier family 12 member 4	SLC12A4	sp|Q9UP9	2	2	2	0	0	0	2	0	0	0	2	0	0	0	2	2.8	120.65	0	7.2262	33545000	0	0	0	33545000	0	0	0	1379600
P56559	P56559	ADP-ribosylation factor-like protein 4C	ARL4C	sp|P56559	1	1	1	0	0	0	1	0	0	0	1	0	0	0	1	7.8	21.487	0.000247	3.662	33503000	0	0	0	33503000	0	0	0	1377800
Q01804	Q01804	OTU domain-containing protein 4	OTUD4	sp|Q01804	2	2	2	0	2	2	2	0	2	2	2	0	2	2	2	2.5	124.04	0	6.5697	33466000	0	11416000	3939600	18110000	0	1078100	2165900	1154500
Q9UL62;Q9	Q9UL62;Q9	Short transient receptor potential channel 5;Shor	TRPC5;TRF	sp|Q9UL62	1	1	1	1	1	0	0	1	1	0	0	1	1	0	0	0.7	111.41	0.00946	1.5434	33427000	22012000	11415000	0	0	22012000	1006200	0	0
P53794	P53794	Sodium/myo-inositol cotransporter	SLC5A3	sp|P53794	2	2	2	0	0	0	2	0	0	0	2	0	0	0	2	6.1	79.693	0	3.8962	33418000	0	0	0	33418000	0	0	0	1374300
Q9Y6J9	Q9Y6J9	TAF6-like RNA polymerase II p300/CBP-associatec	TAF6L	sp|Q9Y6J9	2	2	2	0	2	0	1	0	2	0	1	0	2	0	1	6.3	67.814	0.000483	3.4031	33330000	0	25357000	0	7972900	0	1751600	0	811550
Q969S9	Q969S9	Ribosome-releasing factor 2, mitochondrial	GFM2	sp|Q969S9	2	2	2	0	2	0	1	0	2	0	1	0	2	0	1	2.6	86.6	0	4.9538	33258000	0	28479000	0	4778700	0	1581400	0	1125500
Q9H583	Q9H583	HEAT repeat-containing protein 1;HEAT repeat-cc	HEATR1	sp|Q9H583	1	1	1	0	1	0	1	0	1	0	1	0	1	0	1	0.5	242.37	0.003906	1.9363	33210000	0	8934400	0	24276000	0	0	0	998350
O75676	O75676	Ribosomal protein S6 kinase alpha-4	RPS6KA4	sp|O75676	3	3	3	0	2	1	3	0	2	1	3	0	2	1	3	4.8	85.605	0	8.3101	33136000	0	1320800	759270	31056000	0	242080	534030	1127800
P63096	P63096	Guanine nucleotide-binding protein G(i) subunit a	GNAI1	sp|P63096	4	1	1	0	2	2	4	0	1	0	1	0	1	0	1	14.1	40.361	0.000489	3.5355	33107000	0	9204700	0	23902000	0	811390	0	983000
P82914	P82914	28S ribosomal protein S15, mitochondrial	MRPS15	sp|P82914	1	1	1	0	1	1	1	0	1	1	1	0	1	1	1	4.7	29.842	0.005804	1.7839	33044000	0	13202000	1884900	17957000	0	1211700	1264200	692970
P61024	P61024	Cyclin-dependent kinases regulatory subunit 1	CKS1B	sp|P61024	2	2	2	0	2	0	1	0	2	0	1	0	2	0	1	32.9	9.6601	0	5.552	32887000	0	24201000	0	8685500	0	1627700	0	862830
Q9UPY8	Q9UPY8	Microtubule-associated protein RP/EB family mer	MAPRE3	sp|Q9UPY	3	2	2	0	1	0	3	0	0	0	2	0	0	0	2	15.3	31.982	0	6.8366	32840000	0	0	0	32840000	0	0	0	1350600
Q9H497	Q9H497	Torsin-3A	TOR3A	sp|Q9H497	1	1	1	0	0	0	1	0	0	0	1	0	0	0	1	6.8	46.198	0.000249	3.7819	32782000	0	0	0	32782000	0	0	0	1348200
Q8IZ21	Q8IZ21	Phosphatase and actin regulator 4	PHACTR4	sp|Q8IZ21	2	2	2	0	1	0	2	0	1	0	2	0	1	0	2	5.1	78.21	0	4.1179	32759000	0	13131000	0	19627000	0	1272600	0	692060
Q8TCY9	Q8TCY9	Up-regulator of cell proliferation	URGCP	sp|Q8TCY9	2	2	2	0	1	0	2	0	1	0	2	0	1	0	2	2.6	104.99	0	6.0628	32753000	0	7666700	0	25086000	0	866910	0	840600
Q8IY95	Q8IY95	Transmembrane protein 192	TMEM192	sp|Q8IY95	2	2	2	0	2	0	1	0	2	0	1	0	2	0	1	9.6	30.922	0	3.9224	32737000	0	16669000	0	16068000	0	1469400	0	0
Q9NTG7	Q9NTG7	NAD-dependent protein deacetylase sirtuin-3, mi	SIRT3	sp|Q9NTG	1	1	1	0	1	0	1	0	1	0	1	0	1	0	1	2.8	43.573	0.00072	3.329	32698000	0	10062000	0	22636000	0	886990	0	930900
Q96RN5	Q96RN5	Mediator of RNA polymerase II transcription subu	MED15	sp|Q96RN	2	2	2	1	1	1	2	1	1	1	2	1	1	1	2	3.9	86.753	0	9.233	32656000	1746800	5281600	755100	24872000	1839500	0	991430	446210
Q9UHR6	Q9UHR6	Zinc finger HIT domain-containing protein 2	ZNHIT2	sp|Q9UHR	2	2	2	0	2	0	2	0	2	0	2	0	2	0	2	5	42.883	0.001174	3.0288	32623000	0	15658000	0	16965000	0	1319100	0	758900
Q9NYB9	Q9NYB9	Abl interactor 2	ABI2	sp|Q9NYB	3	1	1	0	3	2	3	0	1	1	1	0	1	1	1	7.2	55.663	0.003903	1.9248	32609000	0	10304000	733990	21571000	0	781290	575740	931630
Q8NAV1	Q8NAV1	Pre-mRNA-splicing factor 38A	PRPF38A	sp|Q8NAV	2	2	2	1	2	0	1	1	2	0	1	1	2	0	1	9	37.476	0	4.6992	32590000	393170	24221000	0	7975600	0	2135000	0	0
O94885	O94885	SAM and SH3 domain-containing protein 1	SASH1	sp|O94885	2	2	2	0	0	0	2	0	0	0	2	0	0	0	2	2.2	136.65	0.000949	3.1557	32582000	0	0	0	32582000	0	0	0	1340000
Q8WVI0	Q8WVI0	Small integral membrane protein 4	SMIM4	sp|Q8WVI	1	1	1	0	1	0	1	0	1	0	1	0	1	0	1	15.7	8.6961	0.003699	1.9632	32575000	0	15989000	0	16586000	0	1409400	0	682130
Q15904	Q15904	V-type proton ATPase subunit S1	ATP6AP1	sp|Q15904	2	2	2	0	1	1	2	0	1	1	2	0	1	1	2	3.6	52.025	0.001139	2.5437	32518000	0	9028000	696720	22793000	0	983000	573030	645360
Q9NU23	Q9NU23	LYR motif-containing protein 2	LYRM2	sp|Q9NU2	2	2	2	0	2	0	0	0	2	0	0	0	2	0	0	21.6	10.449	0	5.2789	32452000	0	32452000	0	0	0	2860600	0	0
P29400	P29400	Collagen alpha-5(IV) chain	COL4A5	sp|P29400	1	1	1	0	1	0	1	0	1	0	1	0	1	0	1	0.5	161.04	0.003277	2.0344	32451000	0	14688000	0	17763000	0	1294800	0	730510
Q8N3X1	Q8N3X1	Formin-binding protein 4	FNBP4	sp|Q8N3X	3	3	3	0	3	0	2	0	3	0	2	0	3	0	2	3.4	110.26	0	7.5153	32428000	0	18953000	0	13475000	0	1351600	0	873260
Q9BSB4	Q9BSB4	Autophagy-related protein 101	ATG101	sp|Q9BSB4	2	2	2	0	1	1	2	0	1	1	2	0	1	1	2	12.8	25.002	0	6.4463	32350000	0	3820800	350500	28179000	0	0	0	1158900
O15226	O15226	NF-kappa-B-repressing factor	NKRF	sp|O15226	4	4	4	2	4	3	2	2	4	3	2	2	4	3	2	5.5	77.672	0	7.7898	32344000	2954100	18942000	4003200	6444300	2275100	2359500	1918400	1025900
Q9NQP4	Q9NQP4	Prefoldin subunit 4	PFDN4	sp|Q9NQP	1	1	1	0	1	0	1	0	1	0	1	0	1	0	1	9	15.314	0.000484	3.4079	32317000	0	10289000	0	22029000	0	906930	0	905940
P55040	P55040	GTP-binding protein GEM	GEM	sp|P55040	2	2	2	0	0	2	2	0	0	2	2	0	0	2	2	8.1	33.949	0	4.6906	32282000	0	0	3919400	28363000	0	0	2731300	1068900
Q9UMS0	Q9UMS0	NFU1 iron-sulfur cluster scaffold homolog, mitoch	NFU1	sp|Q9UMS	2	2	2	0	1	0	2	0	1	0	2	0	1	0	2	9.8	28.462	0	5.6679	32240000	0	4861600	0	27378000	0	550660	0	1003800
P10636	P10636	Microtubule-associated protein tau	MAPT	sp|P10636	2	2	2	0	2	0	0	0	2	0	0	0	2	0	0	4	78.927	0	4.3333	32172000	0	32172000	0	0	0	2836000	0	0
Q8NCE2	Q8NCE2	Myotubularin-related protein 14	MTMR14	sp|Q8NCE	1	1	1	0	0	0	1	0	0	0	1	0	0	0	1	2.9	72.203	0.001134	2.4828	32156000	0	0	0	32156000	0	0	0	1322400
Q9H2V7	Q9H2V7	Protein spinster homolog 1	SPNS1	sp|Q9H2V	2	2	2	0	1	1	2	0	1	1	2	0	1	1	2	4.7	56.629	0	6.2965	32118000	0	10913000	2806900	18398000	0	961990	1886200	756620
Q9NUB1	Q9NUB1	Acetyl-coenzyme A synthetase 2-like, mitochondr	ACSS1	sp|Q9NUB	4	4	4	0	4	0	0	0	4	0	0	0	4	0	0	10.2	74.856	0	11.584	32110000	0	32110000	0	0	0	2830500	0	0
P53634	P53634	Dipeptidyl peptidase 1;Dipeptidyl peptidase 1 exc	CTSC	sp|P53634	2	2	2	0	0	1	2	0	0	1	2	0	0	1	2	5	51.853	0	5.1534	31981000	0	0	264800	31716000	0	0	230230	1252000
Q08AD1	Q08AD1	Calmodulin-regulated spectrin-associated protein	CAMSAP2	sp|Q08AD	3	3	3	0	1	2	2	0	1	2	2	0	1	2	2	2.7	168.09	0	6.3243	31852000	0	1340200	4209100	26303000	0	455550	0	744300
P30047	P30047	GTP cyclohydrolase 1 feedback regulatory proteir	GCHFR	sp|P30047	1	1	1	0	1	0	0	0	1	0	0	0	1	0	0	29.8	9.6981	0	5.817	31828000	0	31828000	0	0	0	2805600	0	0
Q8NEM2	Q8NEM2	SHC SH2 domain-binding protein 1	SHCBP1	sp|Q8NEM	1	1	1	0	0	0	1	0	0	0	1	0	0	0	1	4.2	75.69	0	14.813	31812000	0	0	0	31812000	0	0	0	1308300
Q9P0V3	Q9P0V3	SH3 domain-binding protein 4	SH3BP4	sp|Q9P0V3	2	2	2	0	1	2	2	0	1	2	2	0	1	2	2	2.7	107.49	0	4.2914	31698000	0	1051800	4025500	26621000	0	700760	1954200	1237700
Q9BVL2	Q9BVL2	Nucleoporin p58/p45	NUPL1	sp|Q9BVL2	2	2	2	0	2	0	1	0	2	0	1	0	2	0	1	4.3	60.896	0.000246	3.6346	31647000	0	20961000	0	10686000	0	1847700	0	0
Q9NVA4	Q9NVA4	Transmembrane protein 184C	TMEM184	sp|Q9NVA	1	1	1	0	1	0	1	0	1	0	1	0	1	0	1	3	50.141	0	4.1381	31626000	0	5412800	0	26213000	0	477130	0	1078000
Q8WUD4	Q8WUD4	Coiled-coil domain-containing protein 12	CCDC12	sp|Q8WUD	3	3	3	0	3	2	1	0	3	2	1	0	3	2	1	24.1	19.181	0.001136	2.5123	31613000	0	25174000	783490	5654900	0	2044500	504310	429380
Q8TD30	Q8TD30	Alanine aminotransferase 2	GPT2	sp|Q8TD30	2	2	2	0	2	0	0	0	2	0	0	0	2	0	0	6.1	57.903	0	8.2757	31578000	0	31578000	0	0	0	2783600	0	0
Q8N201	Q8N201	Integrator complex subunit 1	INTS1	sp|Q8N201	1	1	1	0	0	0	1	0	0	0	1	0	0	0	1	0.8	244.29	0.001348	2.3768	31577000	0	0	0	31577000	0	0	0	1298600
O43765	O43765	Small glutamine-rich tetratricopeptide repeat-cor	SGTA	sp|O43765	3	3	3	0	3	1	0	0	3	1	0	0	3	1	0	9.9	34.063	0	6.78	31555000	0	29792000	1763700	0	0	1988900	1822400	0
Q9NNW7	Q9NNW7	Thioredoxin reductase 2, mitochondrial	TXNRD2	sp|Q9NNW	2	2	2	0	1	0	2	0	1	0	2	0	1	0	2	5	56.506	0	7.4134	31535000	0	6452100	0	25083000	0	1045800	0	554520
P05109	P05109	Protein S100-A8;Protein S100-A8, N-terminally pr	S100A8	sp|P05109	2	2	2	0	2	2	1	0	2	2	1	0	2	2	1	19.4	10.834	0.00025	3.7881	31400000	0	12684000	18716000	0	0	1124800	12570000	0
Q9H2P0	Q9H2P0	Activity-dependent neuroprotector homeobox pr	ADNP	sp|Q9H2P0	4	4	4	0	1	0	4	0	1	0	4	0	1	0	4	5.2	123.56	0	8.363	31395000	0	1569200	0	29825000	0	220700	0	1144200
Q96DZ1	Q96DZ1	Endoplasmic reticulum lectin 1	ERLEC1	sp|Q96DZ1	2	2	2	0	0	0	2	0	0	0	2	0	0	0	2	5	54.858	0	12.485	31391000	0	0	0	31391000	0	0	0	1291000
Q9H944	Q9H944	Mediator of RNA polymerase II transcription subu	MED20	sp|Q9H944	1	1	1	0	1	0	1	0	1	0	1	0	1	0	1	5.2	23.222	0.001169	2.9548	31364000	0	19282000	0	12082000	0	1699700	0	496880
P49914	P49914	5-formyltetrahydrofolate cyclo-ligase	MTHFS	sp|P49914	2	2	2	0	2	0	2	0	2	0	2	0	2	0	2	9.4	23.255	0.009642	1.5243	31355000	0	11045000	0	20310000	0	1039700	0	769190
Q8WX93	Q8WX93	Palladin	PALLD	sp|Q8WX9	2	2	2	0	1	0	2	0	1	0	2	0	1	0	2	2.7	150.56	0	4.1591	31286000	0	2260000	0	29026000	0	426560	0	966380
Q86X10	Q86X10	Ral GTPase-activating protein subunit beta	RALGAPB	sp|Q86X10	2	2	2	0	1	0	1	0	1	0	1	0	1	0	1	3.2	166.8	0.000482	3.3652	31286000	0	23462000	0	7824100	0	2068200	0	0
Q92604	Q92604	Acyl-CoA:lysophosphatidylglycerol acyltransferase	LPGAT1	sp|Q92604	1	1	1	0	0	0	1	0	0	0	1	0	0	0	1	3.2	43.089	0.006414	1.7271	31273000	0	0	0	31273000	0	0	0	1286100
O75191	O75191	Xylulose kinase	XYLB	sp|O75191	2	2	2	0	2	0	1	0	2	0	1	0	2	0	1	6.3	58.382	0	5.2868	31271000	0	21695000	0	9576800	0	1643800	0	662400
Q93050	Q93050	V-type proton ATPase 116 kDa subunit a isoform	ATP6V0A1	sp|Q93050	4	4	4	0	4	0	0	0	4	0	0	0	4	0	0	6.3	96.412	0	7.4578	31236000	0	31236000	0	0	0	2753400	0	0
Q9NXJ5	Q9NXJ5	Pyroglutamyl-peptidase 1	PGPEP1	sp|Q9NXJ5	1	1	1	0	0	0	1	0	0	0	1	0	0	0	1	7.7	23.138	0	4.9164	31136000	0	0	0	31136000	0	0	0	1280500
Q6ZS17	Q6ZS17	Protein FAM65A	FAM65A	sp|Q6ZS17	3	3	3	0	0	0	3	0	0	0	3	0	0	0	3	4.3	132.31	0	12.103	31118000	0	0	0	31118000	0	0	0	1279700
P20585	P20585	DNA mismatch repair protein Msh3	MSH3	sp|P20585	2	2	2	0	2	1	2	0	2	1	2	0	2	1	2	1.6	127.41	0.006423	1.7364	31075000	0	14765000	557090	15753000	0	975970	778530	569230
Q92536	Q92536	Y+L amino acid transporter 2	SLC7A6	sp|Q92536	2	1	1	0	2	0	1	0	1	0	1	0	1	0	1	7.2	56.827	0	4.0175	31068000	0	11916000	0	19152000	0	1050400	0	787630
Q9BZL4	Q9BZL4	Protein phosphatase 1 regulatory subunit 12C	PPP1R12C	sp|Q9BZL4	5	5	5	0	1	2	4	0	1	2	4	0	1	2	4	10	84.88	0	8.8428	31018000	0	1992900	2686600	26338000	0	489900	1314700	1259600
O75410	O75410	Transforming acidic coiled-coil-containing protein	TACC1	sp|O75410	2	2	2	0	1	1	2	0	1	1	2	0	1	1	2	2.4	87.793	0	4.1615	30970000	0	2281900	268650	28419000	0	374920	355190	820340
Q8WVD3	Q8WVD3	E3 ubiquitin-protein ligase RNF138	RNF138	sp|Q8WVD	2	2	2	0	2	0	2	0	2	0	2	0	2	0	2	13.9	28.192	0.004529	1.8263	30892000	0	19198000	0	11693000	0	1611400	0	561840
Q5T9L3	Q5T9L3	Protein wntless homolog	WLS	sp|Q5T9L3	2	2	2	0	1	0	2	0	1	0	2	0	1	0	2	4.4	62.253	0	6.5121	30745000	0	2918300	0	27826000	0	303540	0	1098100

P42357	P42357	Histidine ammonia-lyase	HAL	sp|P42357	5	5	5	1	0	5	0	1	0	5	0	1	0	5	0	10.4	72.697	0	9.6455	30742000	68118	0	30674000	0	68118	0	20613000	0
Q9Y6K0	Q9Y6K0	Choline/ethanolaminephosphotransferase 1	CEPT1	sp|Q9Y6K(	3	3	3	0	2	0	3	0	2	0	3	0	2	0	3	8.7	46.553	0	3.8256	30635000	0	9771100	0	20864000	0	1512100	0	207210
P62837	P62837	Ubiquitin-conjugating enzyme E2 D2	UBE2D2	sp|P62837	3	1	1	0	2	1	3	0	1	0	1	0	1	0	1	24.5	16.735	0.007447	1.687	30633000	0	13313000	0	17320000	0	1173500	0	712300
Q96EL3	Q96EL3	39S ribosomal protein L53, mitochondrial	MRPL53	sp|Q96EL3	1	1	1	0	0	0	1	0	0	0	1	0	0	0	1	16.1	12.107	0.000247	3.6591	30593000	0	0	0	30593000	0	0	0	1258100
Q9UQR0	Q9UQR0	Sex comb on midleg-like protein 2	SCML2	sp|Q9UQR	3	3	3	0	3	0	0	0	3	0	0	0	3	0	0	5.9	77.256	0	4.8195	30559000	0	30559000	0	0	0	2693700	0	0
Q9NXA8	Q9NXA8	NAD-dependent protein deacylase sirtuin-5, mito	SIRT5	sp|Q9NXA	2	2	2	0	2	0	1	0	2	0	1	0	2	0	1	10.6	33.881	0	7.5774	30455000	0	20567000	0	9888700	0	1508000	0	711620
O15439	O15439	Multidrug resistance-associated protein 4	ABCC4	sp|O15439	2	2	2	0	0	0	2	0	0	0	2	0	0	0	2	2	149.52	0	10.327	30433000	0	0	0	30433000	0	0	0	1251600
Q15014	Q15014	Mortality factor 4-like protein 2	MORF4L2	sp|Q15014	3	2	2	0	3	2	3	0	2	1	2	0	2	1	2	13.2	32.307	0.003912	1.9519	30367000	0	10739000	750520	18877000	0	663360	1148700	415240
Q9Y303	Q9Y303	Putative N-acetylglucosamine-6-phosphate deace	AMDHD2	sp|Q9Y303	1	1	1	0	0	0	1	0	0	0	1	0	0	0	1	2.9	43.747	0.003905	1.9302	30298000	0	0	0	30298000	0	0	0	1246000
Q13137	Q13137	Calcium-binding and coiled-coil domain-containin	CALCOCO2	sp|Q13137	3	3	3	0	2	0	2	0	2	0	2	0	2	0	2	9.9	52.253	0.000248	3.6956	30283000	0	9087000	0	21196000	0	636230	0	1036500
Q96PC5;Q!	Q96PC5;Q!	Melanoma inhibitory activity protein 2;cTAGE fam	MIA2;CTA(	sp|Q96PC!	5	5	5	0	3	0	4	0	3	0	4	0	3	0	4	4.7	159.83	0	6.4301	30282000	0	6422200	0	23860000	0	772180	0	775170
Q96CN9	Q96CN9	GRIP and coiled-coil domain-containing protein 1	GCC1	sp|Q96CN!	2	2	2	0	1	0	2	0	1	0	2	0	1	0	2	3.1	87.81	0	4.3118	30192000	0	4804300	0	25388000	0	0	0	1044100
P31327	P31327	Carbamoyl-phosphate synthase [ammonia], mito(	CPS1	sp|P31327	4	2	2	1	3	1	3	0	1	0	2	0	1	0	2	2.5	164.94	0	4.1655	30050000	0	1708000	0	28342000	0	182550	0	1133600
Q9Y6R0	Q9Y6R0	Numb-like protein	NUMBL	sp|Q9Y6R(	1	1	1	0	1	1	1	0	1	1	1	0	1	1	1	2	64.891	0.001138	2.5236	30015000	0	2387700	1888800	25739000	0	358890	881330	1298000
Q9UHB7	Q9UHB7	AF4/FMR2 family member 4	AFF4	sp|Q9UHB	3	3	3	0	3	0	2	0	3	0	2	0	3	0	2	4.2	127.46	0.00025	3.7999	29889000	0	14104000	0	15785000	0	1317200	0	575230
P52895	P52895	Aldo-keto reductase family 1 member C2	AKR1C2	sp|P52895	7	1	1	0	0	2	7	0	0	0	1	0	0	0	1	38.4	36.735	0.004742	1.819	29867000	0	0	0	29867000	0	0	0	1228300
Q16854	Q16854	Deoxyguanosine kinase, mitochondrial	DGUOK	sp|Q16854	1	1	1	0	1	0	1	0	1	0	1	0	1	0	1	9	32.055	0	5.5966	29827000	0	5447700	0	24379000	0	480210	0	1002600
Q12873	Q12873	Chromodomain-helicase-DNA-binding protein 3	CHD3	sp|Q12873	10	2	2	1	9	5	7	1	1	1	1	1	1	1	1	6.6	226.59	0.00846	1.608	29809000	2943500	10840000	1080100	14945000	0	955560	0	614630
P53350	P53350	Serine/threonine-protein kinase PLK1	PLK1	sp|P53350	2	2	2	0	1	2	2	0	1	2	2	0	1	2	2	5	68.254	0	4.17	29744000	0	4207900	2390200	23146000	0	500480	1528500	900090
Q8TAP6	Q8TAP6	Centrosomal protein of 76 kDa	CEP76	sp|Q8TAP(	1	1	1	1	0	0	0	1	0	0	0	1	0	0	0	1.2	74.413	0.007035	1.7018	29737000	29737000	0	0	0	29737000	0	0	0
Q96GM8	Q96GM8	Target of EGR1 protein 1	TOE1	sp|Q96GM	1	1	1	0	1	1	1	0	1	1	1	0	1	1	1	1.6	56.547	0.004536	1.8462	29653000	0	11152000	1314700	17186000	0	983070	883470	706780
Q5R3I4	Q5R3I4	Tetratricopeptide repeat protein 38	TTC38	sp|Q5R3I4	3	3	3	0	3	0	1	0	3	0	1	0	3	0	1	6.6	52.787	0	8.7592	29638000	0	22489000	0	7148500	0	1802100	0	474290
O43657	O43657	Tetraspanin-6	TSPAN6	sp|O43657	2	2	2	0	2	0	2	0	2	0	2	0	2	0	2	7.8	27.563	0.001569	2.3408	29590000	0	22423000	0	7166900	0	1730600	0	540720
Q8WUK0	Q8WUK0	Phosphatidylglycerophosphatase and protein-tyr(	PTPMT1	sp|Q8WUK	2	2	2	0	2	0	1	0	2	0	1	0	2	0	1	12.4	22.843	0	5.476	29538000	0	21229000	0	8309000	0	1871300	0	0
Q6ZXV5	Q6ZXV5	Transmembrane and TPR repeat-containing prote	TMTC3	sp|Q6ZXV!	1	1	1	0	1	0	1	0	1	0	1	0	1	0	1	1.1	104.01	0.004738	1.8117	29446000	0	5107900	0	24339000	0	450260	0	1000900
Q86X83	Q86X83	COMM domain-containing protein 2	COMMD2	sp|Q86X83	1	1	1	0	1	0	1	0	1	0	1	0	1	0	1	3.5	22.745	0.003904	1.9276	29369000	0	9557100	0	19812000	0	842450	0	814780
O95786	O95786	Probable ATP-dependent RNA helicase DDX58	DDX58	sp|O9578(	3	3	3	0	0	0	3	0	0	0	3	0	0	0	3	4	106.6	0	7.096	29365000	0	0	0	29365000	0	0	0	1207700
P41223	P41223	Protein BUD31 homolog	BUD31	sp|P41223	2	2	2	0	1	0	2	0	1	0	2	0	1	0	2	12.5	17	0	4.0055	29354000	0	22057000	0	7297200	0	0	0	300100
Q6ZMZ3	Q6ZMZ3	Nesprin-3	SYNE3	sp|Q6ZMZ	1	1	1	0	0	0	1	0	0	0	1	0	0	0	1	1.1	112.22	0.000247	3.6672	29347000	0	0	0	29347000	0	0	0	1206900
Q6ZSZ5	Q6ZSZ5	Rho guanine nucleotide exchange factor 18	ARHGEF18	sp|Q6ZSZ5	1	1	1	0	1	0	1	0	1	0	1	0	1	0	1	0.5	151.64	0.003078	2.0958	29240000	0	3063400	0	26177000	0	270040	0	1076500
Q9H4I3	Q9H4I3	TraB domain-containing protein	TRABD	sp|Q9H4I3	2	2	2	0	2	0	2	0	2	0	2	0	2	0	2	5.9	42.321	0	4.4725	29063000	0	8227900	0	20835000	0	713640	0	868500
Q15750	Q15750	TGF-beta-activated kinase 1 and MAP3K7-binding	TAB1	sp|Q1575(	4	4	4	0	3	0	2	0	3	0	2	0	3	0	2	11.1	54.643	0	14.588	29053000	0	21289000	0	7763400	0	1876600	0	0
Q32P44	Q32P44	Echinoderm microtubule-associated protein-like 3	EML3	sp|Q32P44	3	3	3	0	0	0	3	0	0	0	3	0	0	0	3	4.5	95.196	0	10.124	29011000	0	0	0	29011000	0	0	0	1193100
Q7Z6K3	Q7Z6K3	Protein prenyltransferase alpha subunit repeat-c(	PTAR1	sp|Q7Z6K3	1	1	1	0	1	0	1	0	1	0	1	0	1	0	1	2.5	46.405	0.009664	1.5364	28885000	0	13873000	0	15012000	0	1222900	0	617380
O60563	O60563	Cyclin-T1	CCNT1	sp|O60563	3	3	3	0	3	1	0	0	3	1	0	0	3	1	0	6.6	80.684	0	5.7069	28868000	0	24387000	4480600	0	0	1891200	3269400	0
O60282	O60282	Kinesin heavy chain isoform 5C	KIF5C	sp|O60282	10	3	3	0	6	5	10	0	0	0	3	0	0	0	3	14.5	109.49	0	13.073	28848000	0	0	0	28848000	0	0	0	1186400
Q9NXC5	Q9NXC5	WD repeat-containing protein mio	MIOS	sp|Q9NXC	4	4	4	0	2	0	3	0	2	0	3	0	2	0	3	5.6	98.583	0	5.8522	28767000	0	16150000	0	12616000	0	998920	0	943570
Q9H0H0	Q9H0H0	Integrator complex subunit 2	INTS2	sp|Q9H0H(	1	1	1	0	0	0	1	0	0	0	1	0	0	0	1	1	134.32	0.000248	3.7136	28707000	0	0	0	28707000	0	0	0	1180600
Q6PL24	Q6PL24	Protein TMED8	TMED8	sp|Q6PL24	2	2	2	0	2	0	0	0	2	0	0	0	2	0	0	10.8	35.74	0	3.8942	28510000	0	28510000	0	0	0	2513100	0	0
Q5BJH7	Q5BJH7	Protein YIF1B	YIF1B	sp|Q5BJH7	1	1	1	0	1	0	1	0	1	0	1	0	1	0	1	7.6	34.435	0	5.1848	28477000	0	10252000	0	18226000	0	903690	0	749540
Q9H4Z3	Q9H4Z3	Phosphorylated CTD-interacting factor 1	PCIF1	sp|Q9H4Z3	2	2	2	0	2	0	2	0	2	0	2	0	2	0	2	4.5	80.669	0	4.5863	28401000	0	10576000	0	17826000	0	928800	0	736530
Q13107	Q13107	Ubiquitin carboxyl-terminal hydrolase 4	USP4	sp|Q13107	2	2	2	0	0	0	2	0	0	0	2	0	0	0	2	3.5	108.56	0	5.5045	28392000	0	0	0	28392000	0	0	0	1167600
P48740;P0	P48740	Mannan-binding lectin serine protease 1;Mannan	MASP1	sp|P48740	4	4	4	0	0	4	0	0	0	4	0	0	0	4	0	8.7	79.246	0	9.9569	28336000	0	0	28336000	0	0	0	19041000	0
P08253	P08253	72 kDa type IV collagenase;PEX	MMP2	sp|P08253	1	1	1	0	0	0	1	0	0	0	1	0	0	0	1	1.8	73.881	0.000722	3.3555	28316000	0	0	0	28316000	0	0	0	1164500
Q9UDX5	Q9UDX5	Mitochondrial fission process protein 1	MTFP1	sp|Q9UDX	1	1	1	0	1	0	1	0	1	0	1	0	1	0	1	6.6	18.01	0.001166	2.9047	28250000	0	11089000	0	17161000	0	977510	0	705750
O95425	O95425	Supervillin	SVIL	sp|O95425	4	4	4	0	0	4	1	0	0	4	1	0	0	4	1	2.2	247.74	0	9.1843	28220000	0	0	15426000	12795000	0	0	9992600	899540
Q14249	Q14249	Endonuclease G, mitochondrial	ENDOG	sp|Q14249	1	1	1	0	0	0	1	0	0	0	1	0	0	0	1	5.7	32.62	0.000245	3.59	28170000	0	0	0	28170000	0	0	0	1158500
Q9NPF5	Q9NPF5	DNA methyltransferase 1-associated protein 1	DMAP1	sp|Q9NPF!	3	3	3	0	2	0	1	0	2	0	1	0	2	0	1	10.5	52.992	0	6.2435	28150000	0	28150000	0	0	0	2481400	0	0
O43808	O43808	Peroxisomal membrane protein PMP34	SLC25A17	sp|O43808	1	1	1	0	1	0	1	0	1	0	1	0	1	0	1	5.9	34.566	0	4.1378	28149000	0	12278000	0	15872000	0	1082300	0	652730
A2A3N6;Q	A2A3N6	Putative PIP5K1A and PSMD4-like protein	PIPSL	sp|A2A3N(	5	1	1	0	4	5	5	0	0	1	1	0	0	1	1	6	95.047	0	3.8418	28101000	0	0	5284300	22817000	0	0	3551000	938340
Q86XI2	Q86XI2	Condensin-2 complex subunit G2	NCAPG2	sp|Q86XI2	2	2	2	0	1	1	2	0	1	1	2	0	1	1	2	1.5	130.96	0.002663	2.1898	28000000	0	2445900	1555900	23998000	0	680070	0	522480
P00156	P00156	Cytochrome b	MT-CYB	sp|P00156	1	1	1	0	1	0	1	0	1	0	1	0	1	0	1	2.4	42.717	0.001565	2.2906	27813000	0	16817000	0	10996000	0	1482400	0	452210
A0A024RB	A0A024RB	Diphosphoinositol polyphosphate phosphohydrol	NUDT4;NU	sp|A0A024	2	2	2	0	2	0	1	0	2	0	1	0	2	0	1	12.7	20.434	0.000246	3.651	27810000	0	19767000	0	8043000	0	1742400	0	330770
Q13795	Q13795	ADP-ribosylation factor-related protein 1	ARFRP1	sp|Q13795	1	1	1	0	1	0	1	0	1	0	1	0	1	0	1	5	22.614	0.000486	3.4685	27722000	0	3957700	0	23764000	0	348870	0	977320
Q9Y680	Q9Y680	Peptidyl-prolyl cis-trans isomerase FKBP7	FKBP7	sp|Q9Y68(	1	1	1	0	0	0	1	0	0	0	1	0	0	0	1	5	25.794	0.008448	1.5967	27608000	0	0	0	27608000	0	0	0	1135400
Q9BT25	Q9BT25	HAUS augmin-like complex subunit 8	HAUS8	sp|Q9BT2!	1	1	1	0	1	1	1	0	1	1	1	0	1	1	1	5.4	44.857	0	4.721	27555000	0	18139000	1336200	8080200	0	1395900	1046100	387140
Q9Y3S2	Q9Y3S2	Zinc finger protein 330	ZNF330	sp|Q9Y3S2	2	2	2	0	2	0	2	0	2	0	2	0	2	0	2	10	36.201	0.000716	3.2676	27513000	0	6066500	0	21446000	0	534740	0	882010
Q96C24	Q96C24	Synaptotagmin-like protein 4	SYTL4	sp|Q96C24	1	1	1	0	0	0	1	0	0	0	1	0	0	0	1	3.4	76.023	0.000947	3.1194	27408000	0	0	0	27408000	0	0	0	1127200
Q16623	Q16623	Syntaxin-1A	STX1A	sp|Q16623	2	2	2	0	0	0	2	0	0	0	2	0	0	0	2	15.3	33.023	0	4.5962	27394000	0	0	0	27394000	0	0	0	1126600
P10109	P10109	Adrenodoxin, mitochondrial	FDX1	sp|P10109	1	1	1	0	1	0	1	0	1	0	1	0	1	0	1	11.4	19.393	0	10.193	27391000	0	19420000	0	7970900	0	1547400	0	492340
P63027	P63027	Vesicle-associated membrane protein 2	VAMP2	sp|P63027	3	1	1	1	3	0	3	0	1	0	1	0	1	0	1	34.5	12.663	0.000717	3.285	27282000	0	13381000	0	13901000	0	1183700	0	567520
Q9BRS8	Q9BRS8	La-related protein 6	LARP6	sp|Q9BRS8	3	3	3	0	0	0	3	0	0	0	3	0	0	0	3	12	54.736	0	6.4175	27247000	0	0	0	27247000	0	0	0	1120500
P29558;Q1	P29558;Q1	RNA-binding motif, single-stranded-interacting pr	RBMS1;RB	sp|P29558	1	1	1	0	1	1	1	0	1	1	1	0	1	1	1	2.5	44.505	0.003085	2.1191	27120000	0	6046800	3412300	17661000	0	499210	2470400	582750
Q9UKU7	Q9UKU7	Isobutyryl-CoA dehydrogenase, mitochondrial	ACAD8	sp|Q9UKU	2	2	2	0	2	1	0	0	2	1	0	0	2	1	0	4.3	45.069	0.00025	3.7917	27050000	0	14874000	12177000	0	0	900840	8593000	0
O60462	O60462	Neuropilin-2	NRP2	sp|O60462	3	3	3	0	0	0	3	0	0	0	3	0	0	0	3	4.7	104.83	0	10.285	27033000	0	0	0	27033000	0	0	0	1111800
Q13287	Q13287	N-myc-interactor	NMI	sp|Q13287	1	1	1	0	0	0	1	0	0	0	1	0	0	0	1	4.2	35.056	0.001162	2.8416	26981000	0	0	0	26981000	0	0	0	1109600
P49674;P4	P49674;P4	Casein kinase I isoform epsilon;Casein kinase I iso	CSNK1E;CS	sp|P49674	3	3	3	1	1	0	3	1	1	0	3	1	1	0	3	8.9	47.315	0.001163	2.8509	26905000	861690	1873500	0	24170000	0	0	0	993990
O43597	O43597	Protein sprouty homolog 2	SPRY2	sp|O43597	3	3	3	0	0	0	3	0	0	0	3	0	0	0	3	14.9	34.688	0	9.0353	26893000	0	0	0	26893000	0	0	0	1106000
P17050	P17050	Alpha-N-acetylgalactosaminidase	NAGA	sp|P17050	2	2	2	0	0	0	2	0	0	0	2	0	0	0	2	7.1	46.564	0	5.5199	26885000	0	0	0	26885000	0	0	0	1105600
O60504	O60504	Vinexin	SORBS3	sp|O60504	1	1	1	0	0	0	1	0	0	0	1	0	0	0	1	3.6	75.34	0.000719	3.3091	26875000	0	0	0	26875000	0	0	0	1105300
Q8NBN7	Q8NBN7	Retinol dehydrogenase 13	RDH13	sp|Q8NBN	2	2	2	0	2	0	1	0	2	0	1	0	2	0	1	5.4	35.932	0.001157	2.7648	26752000	0	14400000	0	12353000	0	1199700	0	577680
Q5JUR7	Q5JUR7	Testis-expressed sequence 30 protein	TEX30	sp|Q5JUR7	1	1	1	0	0	0	1	0	0	0	1	0	0	0	1	8.4	25.585	0	9.2084	26725000	0	0	0	26725000	0	0	0	1099100
Q5BKY9;Q	Q5BKY9	Protein FAM133B	FAM133B	sp|Q5BKY!	3	3	3	0	2	3	1	0	2	3	1	0	2	3	1	14.6	28.385	0.001177	3.0591	26705000	0	11548000	13340000	1817300	0	95491	9853500	107960
Q8NCL4	Q8NCL4	Polypeptide N-acetylgalactosaminyltransferase 6	GALNT6	sp|Q8NCL4	1	1	1	0	0	0	1	0	0	0	1	0	0	0	1	3.1	71.158	0.009656	1.5317	26675000	0	0	0	26675000	0	0	0	1097000
Q86TN4	Q86TN4	tRNA 2-phosphotransferase 1	TRPT1	sp|Q86TN4	1	1	1	0	0	0	1	0	0	0	1	0	0	0	1	5.9	27.742	0.003279	2.0413	26645000	0	0	0	26645000	0	0	0	1095800
Q16527	Q16527	Cysteine and glycine-rich protein 2	CSRP2	sp|Q16527	3	3	3	0	3	0	2	0	3	0	2	0	3	0	2	23.8	20.954	0	6.8801	26637000	0	16842000	0	9795300	0	1465900	0	421580
Q53S08;Q!	Q53S08;Q!	Ras-related protein Rab-6C	RAB6C	sp|Q53S08	1	1	1	0	1	0	0	0	1	0	0	0	1	0	0	8.7	28.242	0	21.178	26535000	0	26535000	0	0	0	2339000	0	0
Q06190	Q06190	Serine/threonine-protein phosphatase 2A regulat	PPP2R3A	sp|Q0619(	1	1	1	0	0	0	1	0	0	0	1	0	0	0	1	1.5	130.28	0.00454	1.8624	26522000	0	0	0	26522000	0	0	0	1090700
Q9BVV7	Q9BVV7	Mitochondrial import inner membrane transloca:	TIMM21	sp|Q9BVV	2	2	2	0	2	2	2	0	2	2	2	0	2	2	2	6.9	28.202	0.006001	1.756	26481000	0	8182300	1104700	17194000	0	730170	663700	776860
Q96GW9	Q96GW9	Methionine--tRNA ligase, mitochondrial	MARS2	sp|Q96GW	2	2	2	0	2	0	0	0	2	0	0	0	2	0	0	7.1	66.59	0	6.2435	26457000	0	26457000	0	0	0	2332200	0	0
Q8N490	Q8N490	Probable hydrolase PNKD	PNKD	sp|Q8N49(	1	1	1	0	1	0	0	0	1	0	0	0	1	0	0	3.9	42.875	0.002658	2.175	26447000	0	26447000	0	0	0	2331300	0	0
O94851	O94851	Protein-methionine sulfoxide oxidase MICAL2	MICAL2	sp|O94851	2	2	2	0	0	1	2	0	0	1	2	0	0	1	2	2	126.69	0.000716	3.2659	26426000	0	0	1894600	24532000	0	0	1466200	815830
Q9HB66	Q9HB66		MKKS	tr|Q9HB66	1	1	1	0	1	0	1	0	1	0	1	0	1	0	1	27	7.2634	0	4.568	26298000	0	10912000	0	15386000	0	961890	0	632750
P61266	P61266	Syntaxin-1B	STX1B	sp|P61266	2	2	2	0	0	0	2	0	0	0	2	0	0	0	2	10.1	33.244	0	7.767	26245000	0	0	0	26245000	0	0	0	1079300
Q86YR5	Q86YR5	G-protein-signaling modulator 1	GPSM1	sp|Q86YR!	2	2	2	0	2	0	1	0	2	0	1	0	2	0	1	5.3	74.509	0	7.2121	26162000	0	24004000	0	2158100	0	1828700	0	375980
Q9H0U9	Q9H0U9	Testis-specific Y-encoded-like protein 1	TSPYL1	sp|Q9H0U	1	1	1	0	0	1	1	0	0	1	1	0	0	1	1	4.6	49.192	0	12.407	26153000	0	0	4917200	21235000	0	0	3304300	873320
Q9C0E2	Q9C0E2	Exportin-4	XPO4	sp|Q9C0E2	2	2	2	0	2	0	1	0	2	0	1	0	2	0	1	2.4	130.14	0	5.1845	26136000	0	16336000	0	9799800	0	1079600	0	763450
Q9BQ69	Q9BQ69	O-acetyl-ADP-ribose deacetylase MACROD1	MACROD1	sp|Q9BQ6!	1	1	1	0	1	0	0	0	1	0	0	0	1	0	0	3.1	35.505	0.001175	3.0393	26108000	0	26108000	0	0	0	2301400	0	0
Q5TDH0	Q5TDH0	Protein DDI1 homolog 2	DDI2	sp|Q5TDH(	2	2	2	0	2	0	1	0	2	0	1	0	2	0	1	9.5	44.522	0.001152	2.7092	26001000	0	12827000	0	13174000	0	1130700	0	541770

Q5U5X0	Q5U5X0	Complex III assembly factor LYRM7	LYRM7	sp|Q5U5X	2	2	2	0	1	0	1	0	1	0	1	0	1	0	1	24	11.955	0	5.6615	25981000	0	25981000	0	0	0	2290200	0	0
P42695	P42695	Condensin-2 complex subunit D3	NCAPD3	sp|P42695	2	2	2	0	2	1	2	0	2	1	2	0	2	1	2	1.4	168.89	0	10.093	25944000	0	6579300	2132600	17232000	0	1131600	0	157070
Q9UL03	Q9UL03	Integrator complex subunit 6	INTS6	sp|Q9UL0	1	1	1	0	0	0	1	0	0	0	1	0	0	0	1	1.7	100.39	0.001348	2.377	25934000	0	0	0	25934000	0	0	0	1066500
O14672	O14672	Disintegrin and metalloproteinase domain-contai	ADAM10	sp|O1467	1	1	1	0	1	0	1	0	1	0	1	0	1	0	1	2	84.141	0.001564	2.2827	25865000	0	6359500	0	19505000	0	560580	0	802160
P55789	P55789	FAD-linked sulfhydryl oxidase ALR	GFER	sp|P55789	3	3	3	0	3	0	0	0	3	0	0	0	3	0	0	18.5	23.449	0	9.4418	25842000	0	25842000	0	0	0	2277900	0	0
Q15025	Q15025	TNFAIP3-interacting protein 1	TNIP1	sp|Q1502	2	2	2	0	1	1	2	0	1	1	2	0	1	1	2	4.1	71.863	0	6.2478	25808000	0	6278400	1560700	17969000	0	722650	994810	623750
O95758	O95758	Polypyrimidine tract-binding protein 3	PTBP3	sp|O9575	3	1	1	1	2	3	2	0	0	1	1	0	0	1	1	7.6	59.689	0.000485	3.4323	25759000	0	0	2576800	23182000	0	0	0	953390
Q5T0D9	Q5T0D9	Tumor protein p63-regulated gene 1-like protein	TPRG1L	sp|Q5T0D	2	2	2	0	0	0	2	0	0	0	2	0	0	0	2	16.9	30.211	0	6.2229	25756000	0	0	0	25756000	0	0	0	1059200
O00754	O00754	Lysosomal alpha-mannosidase;Lysosomal alpha-m	MAN2B1	sp|O0075	1	1	1	0	1	0	0	0	1	0	0	0	1	0	0	1.5	113.74	0	4.7819	25628000	0	25628000	0	0	0	2259100	0	0
P08922	P08922	Proto-oncogene tyrosine-protein kinase ROS	ROS1	sp|P08922	1	1	1	0	0	0	1	0	0	0	1	0	0	0	1	0.5	263.91	0.006424	1.7422	25567000	0	0	0	25567000	0	0	0	1051500
P31323	P31323	cAMP-dependent protein kinase type II-beta regu	PRKAR2B	sp|P31323	4	3	3	0	3	0	2	0	2	0	1	0	2	0	1	15.3	46.302	0	3.9218	25567000	0	25567000	0	0	0	2253700	0	0
Q9NSY1	Q9NSY1	BMP-2-inducible protein kinase	BMP2K	sp|Q9NSY	2	2	2	0	0	0	2	0	0	0	2	0	0	0	2	2.3	129.17	0	4.6159	25489000	0	0	0	25489000	0	0	0	1048300
Q9NWB6	Q9NWB6	Arginine and glutamate-rich protein 1	ARGLU1	sp|Q9NW	3	3	3	1	2	1	0	1	2	1	0	1	2	1	0	8.4	33.216	0	4.9981	25454000	1123600	20428000	3903000	0	1500800	1423500	0	0
O95834	O95834	Echinoderm microtubule-associated protein-like	EML2	sp|O9583	3	3	3	0	2	0	3	0	2	0	3	0	2	0	3	5.4	70.678	0	6.0759	25433000	0	4581700	0	20851000	0	470750	0	790620
Q9BRT6	Q9BRT6	Protein LLP homolog	LLPH	sp|Q9BRT	1	1	1	0	1	1	1	0	1	1	1	0	1	1	1	10.9	15.225	0.003079	2.0962	25412000	0	7097700	3176300	15138000	0	625660	2134400	622570
Q9H7B4	Q9H7B4	Histone-lysine N-methyltransferase SMYD3	SMYD3	sp|Q9H7B	2	2	2	0	2	0	2	0	2	0	2	0	2	0	2	6.5	49.097	0.00287	2.1345	25323000	0	8904000	0	16419000	0	795580	0	664550
Q9BUE6	Q9BUE6	Iron-sulfur cluster assembly 1 homolog, mitochon	ISCA1	sp|Q9BUE	1	1	1	0	1	0	1	0	1	0	1	0	1	0	1	9.3	14.179	0.000487	3.4823	25320000	0	6120600	0	19199000	0	539530	0	789570
Q99504	Q99504	Eyes absent homolog 3	EYA3	sp|Q9950	3	3	3	0	2	0	1	0	2	0	1	0	2	0	1	8	62.662	0.000247	3.6623	25258000	0	18886000	0	6372200	0	1664700	0	0
Q96QU8	Q96QU8	Exportin-6	XPO6	sp|Q96QU	1	1	1	0	1	0	1	0	1	0	1	0	1	0	1	1	128.88	0	5.1181	25244000	0	4431800	0	20812000	0	390660	0	855910
Q7Z3K3	Q7Z3K3	Pogo transposable element with ZNF domain	POGZ	sp|Q7Z3K	2	2	2	0	2	1	1	0	2	1	1	0	2	1	1	2.3	155.34	0	4.4897	25174000	0	10397000	947110	13830000	0	953730	600380	567560
P49711;Q8	P49711;Q8	Transcriptional repressor CTCF;Transcriptional re	CTCF;CTCF	sp|P49711	2	2	2	0	2	2	0	0	2	2	0	0	2	2	0	5.4	82.785	0	5.1067	25108000	0	14989000	10119000	0	0	1189600	6931600	0
P53611	P53611	Geranylgeranyl transferase type-2 subunit beta	RABGGTB	sp|P53611	2	2	2	0	1	0	2	0	1	0	2	0	1	0	2	3.3	36.924	0.000248	3.7001	25101000	0	1884900	0	23216000	0	468580	0	652350
Q96AA3	Q96AA3	Protein RFT1 homolog	RFT1	sp|Q96AA	2	2	2	0	1	0	2	0	1	0	2	0	1	0	2	4.3	60.335	0.000721	3.3349	25089000	0	6178400	0	18911000	0	834860	0	487470
P43007	P43007	Neutral amino acid transporter A	SLC1A4	sp|P43007	1	1	1	0	1	0	1	0	1	0	1	0	1	0	1	5.1	55.722	0.000247	3.6669	25076000	0	12102000	0	12974000	0	1066800	0	533550
O95295	O95295	SNARE-associated protein Snapin	SNAPIN	sp|O9529	2	2	2	0	2	0	1	0	2	0	1	0	2	0	1	22.8	14.874	0	11.46	25024000	0	18671000	0	6352900	0	1464900	0	442240
Q96F63	Q96F63	Coiled-coil domain-containing protein 97	CCDC97	sp|Q96F6	2	2	2	0	2	0	0	0	2	0	0	0	2	0	0	7.6	38.946	0	7.1463	25004000	0	25004000	0	0	0	2204100	0	0
Q8IX18	Q8IX18	Probable ATP-dependent RNA helicase DHX40	DHX40	sp|Q8IX18	1	1	1	0	1	0	1	0	1	0	1	0	1	0	1	1.5	88.559	0.001177	3.0577	24940000	0	5541800	0	19398000	0	488510	0	797740
O95985	O95985	DNA topoisomerase 3-beta-1	TOP3B	sp|O9598	3	3	2	2	2	1	2	2	2	1	2	1	1	0	1	4.8	96.661	0	7.6872	24893000	1068000	8251800	1616400	13957000	1126300	642230	1261400	425580
Q9Y605	Q9Y605	MORF4 family-associated protein 1	MRFAP1	sp|Q9Y60	1	1	1	0	1	0	1	0	1	0	1	0	1	0	1	18.9	14.649	0	7.3817	24831000	0	9339600	0	15491000	0	823280	0	637090
Q96LB3	Q96LB3	Intraflagellar transport protein 74 homolog	IFT74	sp|Q96LB	2	2	2	0	2	0	1	0	2	0	1	0	2	0	1	4.7	69.239	0	4.3803	24819000	0	15796000	0	9022600	0	902980	0	860510
P13987	P13987	CD59 glycoprotein	CD59	sp|P13987	1	1	1	0	0	1	1	0	0	1	1	0	0	1	1	6.2	14.177	0.003905	1.9317	24812000	0	0	1591300	23220000	0	0	1069400	954960
Q9UNS1	Q9UNS1	Protein timeless homolog	TIMELESS	sp|Q9UNS	4	4	4	0	3	1	2	0	3	1	2	0	3	1	2	4.6	138.66	0	6.8977	24792000	0	6216100	1918800	16657000	0	718710	1102500	701150
P38935	P38935	DNA-binding protein SMUBP-2	IGHMBP2	sp|P38935	2	2	2	1	1	2	2	1	1	2	2	1	1	2	2	2.9	109.15	0.000715	3.244	24697000	1324700	5128200	6321400	11923000	965030	563740	4503400	482850
Q96T60	Q96T60	Bifunctional polynucleotide phosphatase/kinase;F	PNKP	sp|Q96T6	2	2	2	0	1	0	2	0	1	0	2	0	1	0	2	6.3	57.076	0	4.8338	24690000	0	5438200	0	19252000	0	641450	0	629680
Q92925	Q92925	SWI/SNF-related matrix-associated actin-depende	SMARCD2	sp|Q9292	2	2	2	0	1	1	2	0	1	1	2	0	1	1	2	6.2	58.92	0	3.9343	24662000	0	4817000	878070	18967000	0	676220	640190	478290
Q58FG1	Q58FG1	Putative heat shock protein HSP 90-alpha A4	HSP90AA4	sp|Q58FG	2	1	1	1	2	1	2	0	1	0	1	0	1	0	1	5.3	47.711	0.001161	2.829	24588000	0	9909700	0	14678000	0	873530	0	603630
Q8IXI1	Q8IXI1	Mitochondrial Rho GTPase 2	RHOT2	sp|Q8IXI1|	2	2	2	0	1	0	1	0	1	0	1	0	1	0	1	4.2	68.117	0	5.1668	24556000	0	10489000	0	14067000	0	0	0	578530
Q13286	Q13286	Battenin	CLN3	sp|Q1328	1	1	1	0	0	0	1	0	0	0	1	0	0	0	1	3.4	47.622	0.001159	2.7908	24456000	0	0	0	24456000	0	0	0	1005800
Q9NUD5	Q9NUD5	Zinc finger CCHC domain-containing protein 3	ZCCHC3	sp|Q9NUD	2	2	2	0	2	0	0	0	2	0	0	0	2	0	0	11.7	43.547	0.000718	3.2937	24411000	0	24411000	0	0	0	2151800	0	0
O43426	O43426	Synaptojanin-1	SYNJ1	sp|O4342	2	2	2	0	0	0	2	0	0	0	2	0	0	0	2	1.8	173.1	0	5.1284	24352000	0	0	0	24352000	0	0	0	1001500
Q99808	Q99808	Equilibrative nucleoside transporter 1	SLC29A1	sp|Q9980	1	1	1	0	1	0	1	0	1	0	1	0	1	0	1	2	50.219	0.003275	2.0257	24349000	0	11242000	0	13107000	0	990960	0	539030
O14757	O14757	Serine/threonine-protein kinase Chk1	CHEK1	sp|O1475	2	2	2	0	2	0	2	0	2	0	2	0	2	0	2	5.7	54.433	0.000489	3.5329	24254000	0	12013000	0	12241000	0	984910	0	577470
O75448	O75448	Mediator of RNA polymerase II transcription subu	MED24	sp|O7544	1	1	1	0	0	1	1	0	0	1	1	0	0	1	1	1.6	110.3	0	6.1714	24235000	0	0	438340	23797000	0	0	294560	978660
Q9BWE0	Q9BWE0	Replication initiator 1	REPIN1	sp|Q9BWE	3	3	3	0	3	1	3	0	3	1	3	0	3	1	3	7.4	63.574	0.000488	3.5152	24228000	0	11513000	1610100	11105000	0	745740	1520000	287750
P01116	P01116	GTPase KRas;GTPase KRas, N-terminally processe	KRAS	sp|P01116	4	1	1	0	3	0	4	0	0	0	1	0	0	0	1	28	21.656	0.002663	2.1899	24201000	0	0	0	24201000	0	0	0	995280
Q9ULU8;Q	Q9ULU8;Q	Calcium-dependent secretion activator 1;Calcium	CADPS;CAI	sp|Q9ULU	2	2	2	0	0	0	2	0	0	0	2	0	0	0	2	2.4	152.78	0	7.5391	24137000	0	0	0	24137000	0	0	0	992660
Q13901	Q13901	Nuclear nucleic acid-binding protein C1D	C1D	sp|Q13901	1	1	1	0	1	0	1	0	1	0	1	0	1	0	1	7.1	16.019	0.001139	2.5576	24134000	0	14579000	0	9555900	0	1348700	0	329400
Q05707	Q05707	Collagen alpha-1(XIV) chain	COL14A1	sp|Q0570	2	2	2	0	2	0	0	0	2	0	0	0	2	0	0	1.5	193.51	0	5.1523	24036000	0	24036000	0	0	0	2118800	0	0
Q7L8L6	Q7L8L6	FAST kinase domain-containing protein 5	FASTKD5	sp|Q7L8L6	2	2	2	0	2	0	2	0	2	0	2	0	2	0	2	3.8	86.573	0	4.5984	24032000	0	9357300	0	14674000	0	858150	0	570180
Q8IXI2	Q8IXI2	Mitochondrial Rho GTPase 1	RHOT1	sp|Q8IXI2|	1	1	1	0	1	0	1	0	1	0	1	0	1	0	1	1.5	70.783	0.003077	2.0948	24029000	0	9735200	0	14294000	0	858150	0	587840
P55061	P55061	Bax inhibitor 1	TMBIM6	sp|P55061	1	1	1	0	1	0	1	0	1	0	1	0	1	0	1	3.4	26.537	0.002876	2.1609	24009000	0	6665600	0	17343000	0	587570	0	713260
P17706	P17706	Tyrosine-protein phosphatase non-receptor type	PTPN2	sp|P17706	2	2	2	0	2	1	2	0	2	1	2	0	2	1	2	5.1	48.473	0	5.4958	23987000	0	7468800	486940	16031000	0	603020	441320	600550
Q9BZA8;Q	Q9BZA8;Q	Protocadherin-11 Y-linked;Protocadherin-11 X-lin	PCDH11Y;F	sp|Q9BZA	2	2	2	0	2	0	1	0	2	0	1	0	2	0	1	1.3	146.77	0.00287	2.1324	23962000	0	17821000	0	6141800	0	1484300	0	339170
Q9H8Y5	Q9H8Y5	Ankyrin repeat and zinc finger domain-containing	ANKZF1	sp|Q9H8Y	2	2	2	0	1	0	1	0	1	0	1	0	1	0	1	3.3	80.926	0	4.1916	23955000	0	16118000	0	7837200	0	0	0	322310
Q9BZG8	Q9BZG8	Diphthamide biosynthesis protein 1	DPH1	sp|Q9BZG	3	3	3	0	1	0	2	0	1	0	2	0	1	0	2	6.8	48.805	0	4.3933	23951000	0	15117000	0	8834600	0	1332500	0	0
Q8IVH8	Q8IVH8	Mitogen-activated protein kinase kinase kinase ki	MAP4K3	sp|Q8IVH	2	2	2	0	0	0	2	0	0	0	2	0	0	0	2	6.6	101.31	0	4.4398	23744000	0	0	0	23744000	0	0	0	976470
O15084	O15084	Serine/threonine-protein phosphatase 6 regulato	ANKRD28	sp|O1508	1	1	1	0	1	1	1	0	1	1	1	0	1	1	1	0.9	112.96	0.006002	1.7572	23705000	0	8571000	896910	14237000	0	721510	644430	577790
Q8IWJ2	Q8IWJ2	GRIP and coiled-coil domain-containing protein 2	GCC2	sp|Q8IWJ	2	2	2	0	1	0	2	0	1	0	2	0	1	0	2	1.5	195.91	0.001167	2.9155	23700000	0	4848300	0	18852000	0	714540	0	488130
Q96II8	Q96II8	Leucine-rich repeat and calponin homology doma	LRCH3	sp|Q96II8|	3	3	3	0	2	1	3	0	2	1	3	0	2	1	3	4.8	86.082	0	9.5866	23505000	0	8027000	811840	14666000	0	616680	804240	435380
P25098;P3	P25098;P3	Beta-adrenergic receptor kinase 1;Beta-adrenerg	ADRBK1;AI	sp|P25098	2	2	2	0	1	0	2	0	1	0	2	0	1	0	2	2.3	79.573	0.000722	3.3516	23476000	0	4994200	0	18481000	0	594960	0	605340
Q15517	Q15517	Corneodesmosin	CDSN	sp|Q15517	2	2	2	0	1	2	0	0	1	2	0	0	1	2	0	5.1	51.522	0.000949	3.1575	23470000	0	7032000	16438000	0	0	766710	10900000	0
P30530	P30530	Tyrosine-protein kinase receptor UFO	AXL	sp|P30530	1	1	1	0	1	1	1	0	1	1	1	0	1	1	1	1.2	98.336	0.000948	3.132	23382000	0	3832300	735790	18814000	0	354850	429300	821840
Q96KQ7	Q96KQ7	Histone-lysine N-methyltransferase EHMT2	EHMT2	sp|Q96KQ	1	1	1	0	1	0	1	0	1	0	1	0	1	0	1	2.2	132.37	0.000489	3.5293	23374000	0	9441600	0	13932000	0	832270	0	572970
Q92805	Q92805	Golgin subfamily A member 1	GOLGA1	sp|Q9280	2	2	2	0	1	0	2	0	1	0	2	0	1	0	2	4.7	88.183	0	8.1057	23354000	0	1397000	0	21957000	0	361280	0	664840
Q96JH7	Q96JH7	Deubiquitinating protein VCIP135	VCPIP1	sp|Q96JH7	3	3	3	0	1	0	3	0	1	0	3	0	1	0	3	2.6	134.32	0	6.2367	23332000	0	5010200	0	18322000	0	551250	0	643890
P53602	P53602	Diphosphomevalonate decarboxylase	MVD	sp|P53602	2	2	2	0	0	0	2	0	0	0	2	0	0	0	2	5.8	43.404	0.00329	2.0765	23201000	0	0	0	23201000	0	0	0	954140
O60870	O60870	DNA/RNA-binding protein KIN17	KIN	sp|O6087	2	2	2	0	2	0	0	0	2	0	0	0	2	0	0	5.1	45.373	0	4.0694	23110000	0	23110000	0	0	0	2037100	0	0
Q6IQ49	Q6IQ49	Protein SDE2 homolog	SDE2	sp|Q6IQ4	2	2	2	0	2	1	0	0	2	1	0	0	2	1	0	6.4	49.741	0.001172	2.9749	23094000	0	22285000	809040	0	0	947490	1560600	0
Q9NWZ5	Q9NWZ5	Uridine-cytidine kinase-like 1	UCKL1	sp|Q9NW	1	1	1	0	1	0	1	0	1	0	1	0	1	0	1	1.6	61.14	0.003281	2.0504	23048000	0	9876200	0	13171000	0	870570	0	541680
Q9Y4U1	Q9Y4U1	Methylmalonic aciduria and homocystinuria type	MMACHC	sp|Q9Y4U	1	1	1	0	1	0	0	0	1	0	0	0	1	0	0	9.9	31.728	0.006829	1.7119	22987000	0	22987000	0	0	0	2026200	0	0
Q8NFG4	Q8NFG4	Folliculin	FLCN	sp|Q8NFG	2	2	2	0	0	0	2	0	0	0	2	0	0	0	2	4.1	64.473	0.001177	3.0559	22955000	0	0	0	22955000	0	0	0	944060
Q9HC21	Q9HC21	Mitochondrial thiamine pyrophosphate carrier	SLC25A19	sp|Q9HC2	2	2	2	0	2	0	1	0	2	0	1	0	2	0	1	5.6	35.511	0.006409	1.7233	22952000	0	16776000	0	6175800	0	0	0	253980
Q9H4L4	Q9H4L4	Sentrin-specific protease 3	SENP3	sp|Q9H4L	2	2	2	0	2	0	1	0	2	0	1	0	2	0	1	6.1	65.009	0.004533	1.8334	22923000	0	18583000	0	4339600	0	1532800	0	283720
Q9BQB6	Q9BQB6	Vitamin K epoxide reductase complex subunit 1	VKORC1	sp|Q9BQB	1	1	1	0	0	0	1	0	0	0	1	0	0	0	1	6.7	18.234	0.004734	1.8076	22912000	0	0	0	22912000	0	0	0	942260
P0C7V9;A6	P0C7V9;A6	Putative methyltransferase-like protein 15P1;Pro	METTL15P	sp|P0C7V	1	1	1	0	1	0	0	0	1	0	0	0	1	0	0	6.4	26.715	0	4.555	22758000	0	22758000	0	0	0	2006100	0	0
Q9BU61	Q9BU61	NADH dehydrogenase [ubiquinone] 1 alpha subcc	NDUFAF3	sp|Q9BU6	1	1	1	0	1	0	1	0	1	0	1	0	1	0	1	7.6	20.35	0	4.9984	22678000	0	10620000	0	12057000	0	936160	0	495870
Q9Y2H0	Q9Y2H0	Disks large-associated protein 4	DLGAP4	sp|Q9Y2H	2	2	2	0	0	0	2	0	0	0	2	0	0	0	2	3.8	108.01	0	6.3055	22633000	0	0	0	22633000	0	0	0	930790
Q8IXJ6	Q8IXJ6	NAD-dependent protein deacetylase sirtuin-2	SIRT2	sp|Q8IXJ6|	2	2	2	0	2	0	2	0	2	0	2	0	2	0	2	4.9	43.182	0.000247	3.689	22605000	0	10985000	0	11620000	0	1012900	0	433350
P18074	P18074	TFIIH basal transcription factor complex helicase	ERCC2	sp|P18074	1	1	1	0	1	0	0	0	1	0	0	0	1	0	0	3.3	86.908	0	4.7201	22583000	0	22583000	0	0	0	1990700	0	0
Q9BYX2	Q9BYX2	TBC1 domain family member 2A	TBC1D2	sp|Q9BYX	1	1	1	0	0	0	1	0	0	0	1	0	0	0	1	1.4	105.41	0	3.8499	22537000	0	0	0	22537000	0	0	0	926860
Q96CU9	Q96CU9	FAD-dependent oxidoreductase domain-containi	FOXRED1	sp|Q96CU	2	2	2	0	2	1	1	0	2	1	1	0	2	1	1	3.7	53.811	0.001151	2.6917	22429000	0	14079000	803420	7547200	0	928420	680370	482510
Q6PGP7	Q6PGP7	Tetratricopeptide repeat protein 37	TTC37	sp|Q6PGP	1	1	1	0	1	1	1	0	1	1	1	0	1	1	1	1.7	175.48	0.003279	2.0414	22398000	0	7273000	3758100	11367000	0	721740	2036200	876050
Q9BQG2	Q9BQG2	Peroxisomal NADH pyrophosphatase NUDT12	NUDT12	sp|Q9BQG	1	1	1	0	1	0	1	0	1	0	1	0	1	0	1	3.5	52.075	0.001131	2.4483	22393000	0	12315000	0	10078000	0	1085600	0	414480
Q96EF6	Q96EF6	F-box only protein 17	FBXO17	sp|Q96EF	1	1	1	0	1	0	1	0	1	0	1	0	1	0	1	5.4	31.479	0	4.8645	22392000	0	2773400	0	19618000	0	244480	0	806820
Q9BVL4	Q9BVL4	Selenoprotein O	SELO	sp|Q9BVL	1	1	1	0	0	0	1	0	0	0	1	0	0	0	1	1.9	73.488	0.001164	2.8586	22340000	0	0	0	22340000	0	0	0	918740
Q15527	Q15527	Surfeit locus protein 2	SURF2	sp|Q1552	1	1	1	0	1	0	1	0	1	0	1	0	1	0	1	8.6	29.647	0	4.8011	22329000	0	11431000	0	10898000	0	1007600	0	448210
Q9UGN5	Q9UGN5	Poly [ADP-ribose] polymerase 2	PARP2	sp|Q9UGN	2	2	2	0	1	0	1	0	1	0	1	0	1	0	1	4.5	66.205	0	4.138	22293000	0	17038000	0	5254800	0	1501900	0	0
O14657	O14657	Torsin-1B	TOR1B	sp|O1465	2	2	2	0	0	0	2	0	0	0	2	0	0	0	2	7.1	37.978	0.001178	3.0656	22186000	0	0	0	22186000	0	0	0	912400
Q7Z6K5	Q7Z6K5	Arpin	ARPIN	sp|Q7Z6K	2	2	2	0	2	0	1	0	2	0	1	0	2	0	1	10.2	24.943	0.000246	3.6467	22178000	0	22178000	0	0	0	1955000	0	0

P55212	P55212	Caspase-6;Caspase-6 subunit p18;Caspase-6 subu	CASP6	sp|P55212	2	2	2	0	1	0	2	0	1	0	2	0	1	0	2	6.5	33.31	0.001166	2.9048	22152000	0	8099200	0	14053000	0	898790	0	393080
C9JLW8	C9JLW8	Protein FAM195B	FAM195B	sp|C9JLW8	2	2	2	1	2	0	0	1	2	0	0	1	2	0	0	29.9	10.92	0	4.0315	22111000	1739100	20372000	0	0	2200600	1334200	0	0
O15047	O15047	Histone-lysine N-methyltransferase SETD1A	SETD1A	sp|O15047	2	2	2	0	2	0	1	0	2	0	1	0	2	0	1	1.8	186.03	0	5.0242	22052000	0	16549000	0	5503400	0	1296500	0	388580
Q9H4K7	Q9H4K7	Mitochondrial ribosome-associated GTPase 2	MTG2	sp|Q9H4K	3	3	3	0	0	3	2	0	0	3	2	0	0	3	2	9.1	43.955	0	6.0428	22044000	0	0	11791000	10253000	0	0	7595100	749910
Q8N8I0	Q8N8I0	Sterile alpha motif domain-containing protein 12	SAMD12	sp|Q8N8I	1	1	1	0	1	1	1	0	1	1	1	0	1	1	1	4	22.906	0.009839	1.507	22041000	0	1189000	4019100	16833000	0	382740	1942900	1172300
Q8NBM4	Q8NBM4	Ubiquitin-associated domain-containing protein 2	UBAC2	sp|Q8NBM	2	2	2	0	1	1	2	0	1	1	2	0	1	1	2	7.6	38.963	0.001165	2.8947	22016000	0	7011400	1108200	13896000	0	617020	702150	615090
Q96RT1	Q96RT1	Protein LAP2	ERBB2IP	sp|Q96RT	2	2	2	0	0	0	2	0	0	0	2	0	0	0	2	2.1	158.3	0.001564	2.279	21973000	0	0	0	21973000	0	0	0	903650
O95363	O95363	Phenylalanine--tRNA ligase, mitochondrial	FARS2	sp|O9536	1	1	1	0	1	0	1	0	1	0	1	0	1	0	1	2.7	52.356	0.009658	1.5327	21823000	0	12603000	0	9220100	0	0	0	379180
Q8IZA0	Q8IZA0	Dyslexia-associated protein KIAA0319-like protein	KIAA0319L	sp|Q8IZA0	2	2	2	0	2	0	1	0	2	0	1	0	2	0	1	1.6	115.66	0.008457	1.6075	21793000	0	8544400	0	13249000	0	590130	0	707910
P57764	P57764	Gasdermin-D	GSDMD	sp|P57764	2	2	2	0	0	0	2	0	0	0	2	0	0	0	2	5.6	52.8	0	8.8424	21789000	0	0	0	21789000	0	0	0	896100
Q4U2R6	Q4U2R6	39S ribosomal protein L51, mitochondrial	MRPL51	sp|Q4U2R	1	1	1	0	0	0	1	0	0	0	1	0	0	0	1	9.4	15.095	0.000249	3.7434	21771000	0	0	0	21771000	0	0	0	895360
P31641	P31641	Sodium- and chloride-dependent taurine transpo	SLC6A6	sp|P31641	2	2	2	0	0	0	2	0	0	0	2	0	0	0	2	2.9	69.829	0	4.6485	21759000	0	0	0	21759000	0	0	0	894840
A6NIH7	A6NIH7	Protein unc-119 homolog B	UNC119B	sp|A6NIH7	2	2	2	0	1	0	2	0	1	0	2	0	1	0	2	10.8	28.136	0.000716	3.2641	21685000	0	2897900	0	18787000	0	0	0	772630
Q9NZJ6	Q9NZJ6	Ubiquinone biosynthesis O-methyltransferase, mi	COQ3	sp|Q9NZJ6	1	1	1	0	1	0	0	0	1	0	0	0	1	0	0	4.3	41.054	0	6.3399	21614000	0	21614000	0	0	0	1905200	0	0
P52429	P52429	Diacylglycerol kinase epsilon	DGKE	sp|P52429	2	2	2	0	2	0	0	0	2	0	0	0	2	0	0	4.8	63.926	0	4.9941	21520000	0	21520000	0	0	0	1896900	0	0
Q15120	Q15120	[Pyruvate dehydrogenase (acetyl-transferring)] ki	PDK3	sp|Q1512	2	2	2	0	2	0	1	0	2	0	1	0	2	0	1	6.7	46.938	0.001168	2.9211	21518000	0	16099000	0	5418900	0	1079000	0	562970
Q9UER7	Q9UER7	Death domain-associated protein 6	DAXX	sp|Q9UER	3	3	3	0	3	2	0	0	3	2	0	0	3	2	0	8.2	81.372	0	4.1089	21456000	0	18172000	3284100	0	0	1328400	2480400	0
Q9NVV4	Q9NVV4	Poly(A) RNA polymerase, mitochondrial	MTPAP	sp|Q9NVV	1	1	1	0	1	0	0	0	1	0	0	0	1	0	0	4.1	66.171	0	4.9849	21409000	0	21409000	0	0	0	1887100	0	0
Q9H5J0	Q9H5J0	Zinc finger and BTB domain-containing protein 3	ZBTB3	sp|Q9H5J	2	2	2	0	0	2	0	0	0	2	0	0	0	2	0	12.5	61.827	0.000249	3.7536	21359000	0	0	21359000	0	0	0	14353000	0
Q8N5L8	Q8N5L8	Ribonuclease P protein subunit p25-like protein	RPP25L	sp|Q8N5L	1	1	1	0	1	1	1	0	1	1	1	0	1	1	1	5.5	17.631	0.006213	1.7474	21312000	0	10415000	1557400	9339400	0	918110	1046600	384090
Q9UPW5	Q9UPW5	Cytosolic carboxypeptidase 1	AGTPBP1	sp|Q9UPW	1	1	1	0	1	0	0	0	1	0	0	0	1	0	0	0.9	138.45	0.001171	2.9734	21235000	0	21235000	0	0	0	1871800	0	0
O75688	O75688	Protein phosphatase 1B	PPM1B	sp|O7568	2	1	1	0	2	0	2	0	1	0	1	0	1	0	1	5.2	52.642	0.008446	1.5961	21220000	0	2573200	0	18647000	0	0	0	766880
Q16773	Q16773	Kynurenine--oxoglutarate transaminase 1	CCBL1	sp|Q1677	1	1	1	0	1	0	1	0	1	0	1	0	1	0	1	3.1	47.875	0	5.3214	21139000	0	7567400	0	13572000	0	0	0	558140
Q9NUQ2	Q9NUQ2	1-acyl-sn-glycerol-3-phosphate acyltransferase ep	AGPAT5	sp|Q9NUC	1	1	1	0	1	1	1	0	1	1	1	0	1	1	1	3.3	42.072	0.000487	3.4952	21099000	0	3456700	1598500	16044000	0	453460	851420	733840
Q5T2N8	Q5T2N8	ATPase family AAA domain-containing protein 3C	ATAD3C	sp|Q5T2N	13	1	1	0	8	11	13	0	0	1	1	0	0	1	1	30.9	46.379	0.006009	1.7672	21083000	0	0	4301300	16782000	0	0	2890500	690150
Q96HV5	Q96HV5	Transmembrane protein 41A	TMEM41A	sp|Q96HV	1	1	1	0	1	0	1	0	1	0	1	0	1	0	1	4.5	29.665	0	15.775	21068000	0	21068000	0	0	0	1857100	0	0
Q96LJ7	Q96LJ7	Dehydrogenase/reductase SDR family member 1	DHRS1	sp|Q96LJ7	1	1	1	0	1	0	1	0	1	0	1	0	1	0	1	4.5	33.909	0	4.262	21030000	0	3298300	0	17732000	0	290750	0	729240
Q92508	Q92508	Piezo-type mechanosensitive ion channel compor	PIEZO1	sp|Q9250	2	2	2	0	0	0	2	0	0	0	2	0	0	0	2	1.8	286.79	0.003293	2.0861	20938000	0	0	0	20938000	0	0	0	861100
Q8N5C7	Q8N5C7	DTW domain-containing protein 1	DTWD1	sp|Q8N5C	2	2	2	0	0	0	2	0	0	0	2	0	0	0	2	8.2	35.248	0.000247	3.6674	20897000	0	0	0	20897000	0	0	0	859410
Q9NZQ3	Q9NZQ3	NCK-interacting protein with SH3 domain	NCKIPSD	sp|Q9NZQ	2	2	2	0	1	0	2	0	1	0	2	0	1	0	2	4.6	78.959	0.000247	3.6747	20872000	0	2428200	0	18444000	0	570300	0	402260
P21953	P21953	2-oxoisovalerate dehydrogenase subunit beta, mi	BCKDHB	sp|P21953	2	2	2	0	2	0	0	0	2	0	0	0	2	0	0	8.9	43.122	0	9.2041	20868000	0	20868000	0	0	0	1839500	0	0
Q14409;P3	Q14409;P3	Putative glycerol kinase 3;Glycerol kinase	GK3P;GK	sp|Q1440	2	2	2	1	2	0	2	1	2	0	2	1	2	0	2	3.6	60.597	0.008453	1.599	20838000	380370	12247000	0	8210500	0	1152400	0	264820
P49454	P49454	Centromere protein F	CENPF	sp|P49454	3	3	3	0	0	0	3	0	0	0	3	0	0	0	3	1.2	357.52	0	5.4918	20836000	0	0	0	20836000	0	0	0	856900
Q96C00	Q96C00	Zinc finger and BTB domain-containing protein 9	ZBTB9	sp|Q96C0	5	5	5	1	0	5	0	1	0	5	0	1	0	5	0	16.7	50.602	0	10.95	20816000	1136800	0	19679000	0	0	0	13224000	0
Q96JG6	Q96JG6	Coiled-coil domain-containing protein 132	CCDC132	sp|Q96JG	1	1	1	0	1	0	1	0	1	0	1	0	1	0	1	1.2	111.17	0.001164	2.8749	20788000	0	3785700	0	17002000	0	333700	0	699230
Q9NQ29	Q9NQ29	Putative RNA-binding protein Luc7-like 1	LUC7L	sp|Q9NQ2	7	2	2	1	6	4	5	0	2	1	1	0	2	1	1	18.1	43.727	0.00072	3.3254	20704000	0	12308000	642360	7753800	0	977090	461620	396750
Q96AJ9	Q96AJ9	Vesicle transport through interaction with t-SNAR	VTI1A	sp|Q96AJ9	2	2	2	0	0	0	2	0	0	0	2	0	0	0	2	12	25.217	0.000717	3.2828	20696000	0	0	0	20696000	0	0	0	851150
Q9H7S9;Q	Q9H7S9;Q	Zinc finger protein 703;Zinc finger protein 503	ZNF703;ZN	sp|Q9H7S	1	1	1	0	1	0	1	0	1	0	1	0	1	0	1	2	58.221	0.000717	3.2774	20520000	0	15562000	0	4958400	0	1371800	0	203920
O94875	O94875	Sorbin and SH3 domain-containing protein 2	SORBS2	sp|O9487	2	2	2	0	0	2	1	0	0	2	1	0	0	2	1	3.7	124.11	0	4.5614	20503000	0	0	12782000	7720500	0	0	8177000	730040
Q9UPM8	Q9UPM8	AP-4 complex subunit epsilon-1	AP4E1	sp|Q9UPM	2	2	2	0	0	0	2	0	0	0	2	0	0	0	2	2.2	127.29	0	12.928	20411000	0	0	0	20411000	0	0	0	839400
Q6P4I2	Q6P4I2	WD repeat-containing protein 73	WDR73	sp|Q6P4I2	1	1	1	0	1	0	1	0	1	0	1	0	1	0	1	2.6	41.685	0.003914	1.9553	20405000	0	7939600	0	12465000	0	699860	0	512650
O75143	O75143	Autophagy-related protein 13	ATG13	sp|O7514	2	2	2	0	1	0	2	0	1	0	2	0	1	0	2	6.2	56.571	0	3.8295	20393000	0	3695000	0	16698000	0	419520	0	592900
Q7L1V2	Q7L1V2	Vacuolar fusion protein MON1 homolog B	MON1B	sp|Q7L1V2	1	1	1	0	1	0	1	0	1	0	1	0	1	0	1	5.5	59.217	0.001148	2.6447	20364000	0	4500400	0	15864000	0	396700	0	652420
P49848	P49848	Transcription initiation factor TFIID subunit 6	TAF6	sp|P49848	1	1	1	0	1	1	1	0	1	1	1	0	1	1	1	2.4	72.668	0	4.0868	20345000	0	5671200	1301600	13372000	0	592110	783230	549220
Q9P2X0	Q9P2X0	Dolichol-phosphate mannosyltransferase subunit	DPM3	sp|Q9P2X	1	1	1	0	0	0	1	0	0	0	1	0	0	0	1	10.9	10.094	0.000722	3.3559	20337000	0	0	0	20337000	0	0	0	836390
O14524	O14524	Transmembrane protein 194A	TMEM194	sp|O1452	1	1	1	0	0	0	1	0	0	0	1	0	0	0	1	1.8	50.639	0.004539	1.8615	20297000	0	0	0	20297000	0	0	0	834720
Q6PI78	Q6PI78	Transmembrane protein 65	TMEM65	sp|Q6PI78	2	2	2	0	2	0	1	0	2	0	1	0	2	0	1	8.8	25.498	0	4.3618	20266000	0	14644000	0	5622400	0	1059000	0	462990
P42771;P4	P42771;P4	Cyclin-dependent kinase inhibitor 2A;Cyclin-deper	CDKN2A;C	sp|P42771	2	2	2	0	2	0	0	0	2	0	0	0	2	0	0	18.6	16.532	0	5.1514	20219000	0	20219000	0	0	0	1782300	0	0
Q9Y5W7	Q9Y5W7	Sorting nexin-14	SNX14	sp|Q9Y5W	1	1	1	0	0	0	1	0	0	0	1	0	0	0	1	1	110.18	0.000719	3.3239	20063000	0	0	0	20063000	0	0	0	825110
Q96DT6	Q96DT6	Cysteine protease ATG4C	ATG4C	sp|Q96DT	2	2	2	0	2	0	1	0	2	0	1	0	2	0	1	6.6	52.497	0	6.4911	19965000	0	8958800	0	11006000	0	727920	0	514430
Q9BTY2	Q9BTY2	Plasma alpha-L-fucosidase	FUCA2	sp|Q9BTY	1	1	1	0	0	0	1	0	0	0	1	0	0	0	1	2.4	54.066	0.008455	1.6004	19862000	0	0	0	19862000	0	0	0	816830
Q08357	Q08357	Sodium-dependent phosphate transporter 2	SLC20A2	sp|Q0835	1	1	1	0	1	0	1	0	1	0	1	0	1	0	1	4.6	70.392	0.000489	3.5366	19743000	0	6113500	0	13630000	0	538900	0	560530
P19532	P19532	Transcription factor E3	TFE3	sp|P19532	2	2	2	0	0	0	2	0	0	0	2	0	0	0	2	9.7	61.52	0.000248	3.6995	19645000	0	0	0	19645000	0	0	0	807900
P15735	P15735	Phosphorylase b kinase gamma catalytic chain, liv	PHKG2	sp|P15735	2	2	2	0	2	0	2	0	2	0	2	0	2	0	2	4.7	46.442	0.003078	2.0952	19615000	0	8382400	0	11233000	0	0	0	461950
Q14562	Q14562	ATP-dependent RNA helicase DHX8	DHX8	sp|Q1456	5	3	3	2	5	2	2	1	3	1	0	1	3	1	0	6.1	139.31	0	6.3881	19554000	943220	18044000	567000	0	1293400	825770	795560	0
O75155	O75155	Cullin-associated NEDD8-dissociated protein 2	CAND2	sp|O7515	7	3	3	0	6	3	4	0	3	0	0	0	3	0	0	6.3	135.25	0	5.4422	19554000	0	19554000	0	0	0	1723600	0	0
Q9BT67	Q9BT67	NEDD4 family-interacting protein 1	NDFIP1	sp|Q9BT6	1	1	1	0	0	0	1	0	0	0	1	0	0	0	1	12.7	24.899	0.000717	3.2739	19553000	0	0	0	19553000	0	0	0	804140
Q86WC4	Q86WC4	Osteopetrosis-associated transmembrane proteir	OSTM1	sp|Q86WC	1	1	1	0	1	0	1	0	1	0	1	0	1	0	1	3.3	37.256	0.001783	2.257	19540000	0	5554200	0	13986000	0	489600	0	575180
Q9Y5B8	Q9Y5B8	Nucleoside diphosphate kinase 7	NME7	sp|Q9Y5B	1	1	1	0	0	0	1	0	0	0	1	0	0	0	1	3.7	42.491	0	4.8226	19523000	0	0	0	19523000	0	0	0	802880
O75179	O75179	Ankyrin repeat domain-containing protein 17	ANKRD17	sp|O7517	4	2	2	0	3	0	3	0	1	0	1	0	1	0	1	2.5	274.25	0	7.309	19490000	0	5630100	0	13859000	0	0	0	569970
O60921	O60921	Checkpoint protein HUS1	HUS1	sp|O60921	1	1	1	0	0	0	1	0	0	0	1	0	0	0	1	7.1	31.691	0.003698	1.9631	19455000	0	0	0	19455000	0	0	0	800110
Q8NB49	Q8NB49	Phospholipid-transporting ATPase IG	ATP11C	sp|Q8NB4	1	1	1	0	1	0	0	0	1	0	0	0	1	0	0	1.5	129.48	0.003492	2.0182	19453000	0	19453000	0	0	0	1714800	0	0
Q15051	Q15051	IQ calmodulin-binding motif-containing protein 1	IQCB1	sp|Q15051	1	1	1	0	1	0	0	0	1	0	0	0	1	0	0	2.7	68.928	0.000721	3.3313	19437000	0	19437000	0	0	0	1713400	0	0
Q68D91	Q68D91	Metallo-beta-lactamase domain-containing prote	MBLAC2	sp|Q68D9	2	2	2	0	2	0	0	0	2	0	0	0	2	0	0	7.5	31.371	0	5.0727	19378000	0	19378000	0	0	0	1708100	0	0
Q9NUM4	Q9NUM4	Transmembrane protein 106B	TMEM106	sp|Q9NUM	2	2	2	0	0	0	2	0	0	0	2	0	0	0	2	9.9	31.127	0	3.8525	19328000	0	0	0	19328000	0	0	0	794890
P38435	P38435	Vitamin K-dependent gamma-carboxylase	GGCX	sp|P38435	1	1	1	0	0	0	1	0	0	0	1	0	0	0	1	1.8	87.56	0.000249	3.7811	19326000	0	0	0	19326000	0	0	0	794790
Q8NEW0	Q8NEW0	Zinc transporter 7	SLC30A7	sp|Q8NEW	1	1	1	0	0	0	1	0	0	0	1	0	0	0	1	4.3	41.625	0.007429	1.657	19224000	0	0	0	19224000	0	0	0	790600
Q8N357	Q8N357	Solute carrier family 35 member F6	SLC35F6	sp|Q8N35	2	2	2	0	1	1	2	0	1	1	2	0	1	1	2	5.1	40.214	0.001154	2.7244	19190000	0	1248100	453230	17489000	0	207420	341400	585010
Q86UL3	Q86UL3	Glycerol-3-phosphate acyltransferase 4	AGPAT6	sp|Q86UL	1	1	1	0	1	1	1	0	1	1	1	0	1	1	1	2.6	52.07	0.000489	3.5506	19051000	0	4986200	1292900	12772000	0	439520	868830	525250
O60333	O60333	Kinesin-like protein KIF1B	KIF1B	sp|O6033	5	3	2	0	2	2	5	0	0	0	3	0	0	0	2	3.6	204.47	0	7.8576	18993000	0	0	0	18993000	0	0	0	781110
P68871;P0	P68871;P0	Hemoglobin subunit beta;LVV-hemorphin-7;Spinc	HBB;HBD	sp|P68871	3	2	2	1	2	3	2	0	1	2	1	0	1	2	1	22.4	15.998	0	4.8633	18981000	0	652990	5485100	12843000	0	405390	2023400	1842900
Q9BX68	Q9BX68	Histidine triad nucleotide-binding protein 2, mito	HINT2	sp|Q9BX6	2	2	2	1	0	0	2	1	0	0	2	1	0	0	2	22.1	17.162	0	22.972	18980000	646470	0	0	18334000	646470	0	0	753980
Q6NXR4	Q6NXR4	TELO2-interacting protein 2	TTI2	sp|Q6NXR	2	2	2	0	1	0	2	0	1	0	2	0	1	0	2	7.5	56.914	0	4.5441	18977000	0	1908900	0	17068000	0	291580	0	578620
O15554	O15554	Intermediate conductance calcium-activated pota	KCNN4	sp|O1555	2	2	2	0	0	0	2	0	0	0	2	0	0	0	2	6.3	47.695	0	5.7965	18967000	0	0	0	18967000	0	0	0	780020
Q92625	Q92625	Ankyrin repeat and SAM domain-containing prote	ANKS1A	sp|Q9262	2	2	2	0	2	0	1	0	2	0	1	0	2	0	1	2.2	123.11	0	4.888	18893000	0	13580000	0	5313600	0	1197000	0	0
Q7L7X3	Q7L7X3	Serine/threonine-protein kinase TAO1	TAOK1	sp|Q7L7X	3	2	2	0	1	1	3	0	0	1	2	0	0	1	2	3.2	116.07	0.000489	3.5396	18890000	0	0	1386400	17504000	0	0	0	719850
P00813	P00813	Adenosine deaminase	ADA	sp|P00813	2	2	2	0	2	0	1	0	2	0	1	0	2	0	1	11.6	40.764	0.00117	2.9608	18817000	0	13593000	0	5223300	0	887210	0	525830
Q92997	Q92997	Segment polarity protein dishevelled homolog DV	DVL3	sp|Q9299	2	2	1	1	1	0	2	1	1	0	2	1	0	0	1	3.4	78.054	0	7.2441	18794000	1369200	4543600	0	12881000	0	467150	0	463110
O14893	O14893	Gem-associated protein 2	GEMIN2	sp|O1489	1	1	1	0	1	0	0	0	1	0	0	0	1	0	0	4.3	31.585	0.000245	3.5851	18769000	0	18769000	0	0	0	1654400	0	0
P22670	P22670	MHC class II regulatory factor RFX1	RFX1	sp|P22670	1	1	1	0	1	0	1	0	1	0	1	0	1	0	1	2.9	104.76	0.000246	3.6152	18707000	0	4659600	0	14048000	0	410740	0	577720
Q9ULK4	Q9ULK4	Mediator of RNA polymerase II transcription subu	MED23	sp|Q9ULK	2	2	2	0	0	0	2	0	0	0	2	0	0	0	2	1.8	156.47	0	4.7497	18557000	0	0	0	18557000	0	0	0	763170
Q15057	Q15057	Arf-GAP with coiled-coil, ANK repeat and PH dom	ACAP2	sp|Q1505	2	2	2	0	1	0	2	0	1	0	2	0	1	0	2	4.2	88.028	0	4.6066	18536000	0	1250600	0	17286000	0	305310	0	515820
P46020	P46020	Phosphorylase b kinase regulatory subunit alpha,	PHKA1	sp|P46020	2	2	2	0	1	1	1	0	1	1	1	0	1	1	1	2.9	137.31	0.003701	1.967	18515000	0	8625600	1836000	8053200	0	0	1233800	331190
Q9ULH1	Q9ULH1	Arf-GAP with SH3 domain, ANK repeat and PH do	ASAP1	sp|Q9ULH	4	3	3	0	2	0	4	0	1	0	3	0	1	0	3	5.5	125.5	0.000484	3.4216	18484000	0	3401800	0	15083000	0	0	0	620280
P15151	P15151	Poliovirus receptor	PVR	sp|P15151	1	1	1	0	0	0	1	0	0	0	1	0	0	0	1	2.9	45.302	0.007243	1.6964	18419000	0	0	0	18419000	0	0	0	757500
Q9UPY3	Q9UPY3	Endoribonuclease Dicer	DICER1	sp|Q9UPY	2	2	2	0	1	1	1	0	1	1	1	0	1	1	1	1.8	218.68	0	7.4369	18377000	0	3557800	619890	14199000	0	313610	416570	0
Q96C23	Q96C23	Aldose 1-epimerase	GALM	sp|Q96C2	1	1	1	0	0	0	1	0	0	0	1	0	0	0	1	4.1	37.765	0.002657	2.1718	18355000	0	0	0	18355000	0	0	0	754870
Q9BQ04	Q9BQ04	RNA-binding protein 4B	RBM4B	sp|Q9BQ0	8	1	1	2	7	5	6	0	1	0	1	0	1	0	1	22.6	40.149	0	5.5725	18306000	0	8261000	0	10045000	0	728190	0	413110
Q86UK7	Q86UK7	Zinc finger protein 598	ZNF598	sp|Q86UK	2	2	2	0	2	0	0	0	2	0	0	0	2	0	0	3.2	98.636	0.001129	2.4183	18102000	0	18102000	0	0	0	1595700	0	0

Q9UHR4	Q9UHR4	Brain-specific angiogenesis inhibitor 1-associated	BAIAP2L1	sp|Q9UHR	3	3	3	0	3	0	0	0	3	0	0	0	3	0	0	8.8	56.882	0	5.0564	17973000	0	17973000	0	0	0	1584300	0	0
P25490	P25490	Transcriptional repressor protein YY1	YY1	sp|P25490	2	2	2	0	2	1	1	0	2	1	1	0	2	1	1	6.8	44.712	0	6.3675	17954000	0	9724100	1147100	7082400	0	944820	603480	370960
Q9BWD3;(	Q9BWD3;(	Protein FAM127B;Protein FAM127C	FAM127B;	sp|Q9BWD	2	2	2	0	2	0	2	0	2	0	2	0	2	0	2	14.2	13.188	0.002869	2.1252	17924000	0	3663900	0	14260000	0	321630	0	587820
P05067	P05067	Amyloid beta A4 protein;N-APP;Soluble APP-alph	APP	sp|P05067	1	1	1	0	1	1	1	0	1	1	1	0	1	1	1	1.6	86.942	0	4.5207	17893000	0	2052200	601200	15240000	0	210550	314640	686470
Q9H1Z4	Q9H1Z4	WD repeat-containing protein 13	WDR13	sp|Q9H1Z	2	2	2	0	1	1	1	0	1	1	1	0	1	1	1	6.6	53.695	0.001143	2.5953	17880000	0	6799300	1163800	9917100	0	599350	782060	0
Q9UNH6	Q9UNH6	Sorting nexin-7	SNX7	sp|Q9UNH	1	1	1	0	1	0	1	0	1	0	1	0	1	0	1	2.8	45.302	0.00114	2.5662	17874000	0	6167300	0	11707000	0	0	0	481440
P55081	P55081	Microfibrillar-associated protein 1	MFAP1	sp|P55081	2	2	2	0	1	0	2	0	1	0	2	0	1	0	2	6.4	51.958	0.008437	1.5859	17872000	0	6072800	0	11799000	0	800610	0	219950
P20248	P20248	Cyclin-A2	CCNA2	sp|P20248	1	1	1	0	1	1	0	0	1	1	0	0	1	1	0	3.7	48.55	0	5.3335	17773000	0	15042000	2730500	0	0	1326000	1834900	0
Q6UUV9	Q6UUV9	CREB-regulated transcription coactivator 1	CRTC1	sp|Q6UUV	2	2	2	0	1	0	2	0	1	0	2	0	1	0	2	5	67.299	0	5.3637	17770000	0	12773000	0	4996700	0	1125900	0	205490
Q96EU7	Q96EU7	C1GALT1-specific chaperone 1	C1GALT1C	sp|Q96EU	1	1	1	0	0	0	1	0	0	0	1	0	0	0	1	6.6	36.382	0.001156	2.7587	17674000	0	0	0	17674000	0	0	0	726850
Q8TBE7	Q8TBE7	Solute carrier family 35 member G2	SLC35G2	sp|Q8TBE	2	2	2	0	0	0	2	0	0	0	2	0	0	0	2	14.6	46.421	0	4.3599	17649000	0	0	0	17649000	0	0	0	725820
Q9NZ63	Q9NZ63	Uncharacterized protein C9orf78	C9orf78	sp|Q9NZ6	1	1	1	0	1	0	1	0	1	0	1	0	1	0	1	3.1	33.688	0.002446	2.2191	17634000	0	8737800	0	8896000	0	770220	0	365850
Q99081	Q99081	Transcription factor 12	TCF12	sp|Q99081	2	2	2	0	1	0	1	0	1	0	1	0	1	0	1	4	72.964	0	3.857	17625000	0	10907000	0	6717500	0	0	0	276260
Q96GQ7	Q96GQ7	Probable ATP-dependent RNA helicase DDX27	DDX27	sp|Q96GQ	1	1	1	0	0	1	1	0	0	1	1	0	0	1	1	1.8	89.834	0	4.286	17578000	0	0	2801700	14776000	0	0	1882700	607680
Q9Y3B6	Q9Y3B6	ER membrane protein complex subunit 9	EMC9	sp|Q9Y3B	1	1	1	0	0	0	1	0	0	0	1	0	0	0	1	6.2	23.061	0	3.9491	17560000	0	0	0	17560000	0	0	0	722170
Q5F1R6	Q5F1R6	DnaJ homolog subfamily C member 21	DNAJC21	sp|Q5F1R	1	1	1	0	1	1	0	0	1	1	0	0	1	1	0	3	62.027	0	5.3319	17546000	0	12817000	4729300	0	0	1129800	3178000	0
Q9BRG1	Q9BRG1	Vacuolar protein-sorting-associated protein 25	VPS25	sp|Q9BRG	4	4	4	0	4	0	0	0	4	0	0	0	4	0	0	29.5	20.747	0	7.4267	17524000	0	17524000	0	0	0	1544700	0	0
Q9UKV5	Q9UKV5	E3 ubiquitin-protein ligase AMFR	AMFR	sp|Q9UKV	1	1	1	0	0	0	1	0	0	0	1	0	0	0	1	4.7	72.995	0	14.881	17492000	0	0	0	17492000	0	0	0	719370
P36021	P36021	Monocarboxylate transporter 8	SLC16A2	sp|P36021	2	2	2	0	0	1	2	0	0	1	2	0	0	1	2	4.5	59.511	0	4.4729	17454000	0	0	1017100	16437000	0	0	683510	675970
Q99704	Q99704	Docking protein 1	DOK1	sp|Q99704	1	1	1	0	0	0	1	0	0	0	1	0	0	0	1	5.6	52.391	0.00114	2.5621	17350000	0	0	0	17350000	0	0	0	713510
Q9HC36	Q9HC36	rRNA methyltransferase 3, mitochondrial	RNMTL1	sp|Q9HC3	2	2	2	0	2	1	1	0	2	1	1	0	2	1	1	5.2	47.019	0.001134	2.4866	17349000	0	8957100	1803500	6588200	0	521420	1214000	537040
Q8WVF1	Q8WVF1	Protein OSCP1	OSCP1	sp|Q8WVF	1	1	1	0	1	0	1	0	1	0	1	0	1	0	1	4.4	44.586	0	3.8478	17224000	0	6708300	0	10516000	0	591330	0	432480
Q9UHA3	Q9UHA3	Probable ribosome biogenesis protein RLP24	RSL24D1	sp|Q9UHA	1	1	1	0	1	1	0	0	1	1	0	0	1	1	0	7.4	19.621	0.007041	1.7096	17219000	0	11137000	6081900	0	0	981710	4087000	0
Q9Y3D0	Q9Y3D0	Mitotic spindle-associated MMXD complex subun	FAM96B	sp|Q9Y3D	1	1	1	0	0	0	1	0	0	0	1	0	0	0	1	13.5	17.663	0.002874	2.1519	17157000	0	0	0	17157000	0	0	0	705580
Q9UMZ2	Q9UMZ2	Synergin gamma	SYNRG	sp|Q9UMZ	2	2	2	0	0	0	2	0	0	0	2	0	0	0	2	3	140.65	0	14.39	17118000	0	0	0	17118000	0	0	0	704000
Q12986	Q12986	Transcriptional repressor NF-X1	NFX1	sp|Q1298	2	2	2	0	2	1	2	0	2	1	2	0	2	1	2	2.9	124.39	0	5.8178	17109000	0	5246600	1527000	10335000	0	525820	1061300	326560
Q9HAF1	Q9HAF1	Chromatin modification-related protein MEAF6	MEAF6	sp|Q9HAF	1	1	1	0	1	0	0	0	1	0	0	0	1	0	0	7.9	21.635	0	5.5277	17106000	0	17106000	0	0	0	1507900	0	0
Q96K19	Q96K19	E3 ubiquitin-protein ligase RNF170	RNF170	sp|Q96K1	1	1	1	0	1	0	1	0	1	0	1	0	1	0	1	5.4	29.814	0	4.995	17058000	0	4719000	0	12339000	0	415970	0	507470
Q9NVH6	Q9NVH6	Trimethyllysine dioxygenase, mitochondrial	TMLHE	sp|Q9NVH	1	1	1	0	1	0	0	0	1	0	0	0	1	0	0	3.8	49.517	0.00025	3.8018	17037000	0	17037000	0	0	0	1501800	0	0
Q13472	Q13472	DNA topoisomerase 3-alpha	TOP3A	sp|Q13472	2	1	1	1	2	1	1	0	1	0	0	0	1	0	0	2.4	112.37	0.000951	3.1881	17032000	0	17032000	0	0	0	1501300	0	0
Q13541	Q13541	Eukaryotic translation initiation factor 4E-binding	EIF4EBP1	sp|Q13541	1	1	1	0	1	0	0	0	1	0	0	0	1	0	0	22	12.58	0.001173	3.0164	17003000	0	17003000	0	0	0	1498800	0	0
P08123	P08123	Collagen alpha-2(I) chain	COL1A2	sp|P08123	1	1	1	0	1	1	1	0	1	1	1	0	1	1	1	1.3	129.31	0.00157	2.3482	16980000	0	2177700	883620	13919000	0	260480	458120	639570
Q9UHL4	Q9UHL4	Dipeptidyl peptidase 2	DPP7	sp|Q9UHL	2	2	2	0	0	0	2	0	0	0	2	0	0	0	2	4.3	54.341	0.001148	2.6456	16961000	0	0	0	16961000	0	0	0	697550
Q9NRW1	Q9NRW1	Ras-related protein Rab-6B	RAB6B	sp|Q9NRW	5	2	2	1	5	1	2	0	2	0	0	0	2	0	0	33.7	23.461	0	6.7358	16946000	0	16946000	0	0	0	1493700	0	0
P82921	P82921	28S ribosomal protein S21, mitochondrial	MRPS21	sp|P82921	1	1	1	0	1	0	1	0	1	0	1	0	1	0	1	16.1	10.688	0	6.7393	16928000	0	16928000	0	0	0	1492200	0	0
Q8IW35	Q8IW35	Centrosomal protein of 97 kDa	CEP97	sp|Q8IW3	2	2	2	0	2	0	0	0	2	0	0	0	2	0	0	4.5	96.98	0.001567	2.3096	16913000	0	16913000	0	0	0	1490800	0	0
P09493	P09493	Tropomyosin alpha-1 chain	TPM1	sp|P09493	8	1	1	1	7	5	7	0	1	0	0	0	1	0	0	18.7	32.708	0.001158	2.7811	16888000	0	16888000	0	0	0	1488600	0	0
Q7Z7N9	Q7Z7N9	Transmembrane protein 179B	TMEM179	sp|Q7Z7N	1	1	1	0	1	0	1	0	1	0	1	0	1	0	1	7.3	23.55	0.003913	1.9538	16863000	0	5807400	0	11056000	0	511920	0	454670
O60238	O60238	BCL2/adenovirus E1B 19 kDa protein-interacting p	BNIP3L	sp|O6023	1	1	1	0	0	0	1	0	0	0	1	0	0	0	1	10	23.93	0.007437	1.6686	16756000	0	0	0	16756000	0	0	0	689100
P49427	P49427	Ubiquitin-conjugating enzyme E2 R1	CDC34	sp|P49427	2	2	2	0	1	0	2	0	1	0	2	0	1	0	2	7.2	26.736	0.00308	2.098	16744000	0	2858400	0	13885000	0	0	0	571040
Q9H3Q1	Q9H3Q1	Cdc42 effector protein 4	CDC42EP4	sp|Q9H3Q	1	1	1	0	0	0	1	0	0	0	1	0	0	0	1	5.3	37.979	0.004542	1.8751	16661000	0	0	0	16661000	0	0	0	685200
Q9BZQ6	Q9BZQ6	ER degradation-enhancing alpha-mannosidase-lik	EDEM3	sp|Q9BZQ	1	1	1	0	0	0	1	0	0	0	1	0	0	0	1	2.3	104.66	0.006421	1.7362	16652000	0	0	0	16652000	0	0	0	684810
Q9NWW5	Q9NWW5	Ceroid-lipofuscinosis neuronal protein 6	CLN6	sp|Q9NW	1	1	1	0	1	0	1	0	1	0	1	0	1	0	1	3.5	35.919	0	3.8535	16640000	0	6945000	0	9695400	0	612200	0	398730
Q9NQ89	Q9NQ89	Protein C12orf4	C12orf4	sp|Q9NQ8	1	1	1	0	0	0	1	0	0	0	1	0	0	0	1	4.3	63.8	0.000715	3.2332	16605000	0	0	0	16605000	0	0	0	682890
Q9BVC5	Q9BVC5	Ashwin	C2orf49	sp|Q9BVC	1	1	1	0	1	0	1	0	1	0	1	0	1	0	1	5.6	25.858	0.004538	1.8585	16597000	0	6767000	0	9829800	0	596500	0	404260
Q99717;Q	Q99717;Q	Mothers against decapentaplegic homolog 5;Mot	SMAD5;SM	sp|Q99717	1	1	1	0	1	0	0	0	1	0	0	0	1	0	0	5.2	52.258	0.003288	2.0745	16502000	0	16502000	0	0	0	1454600	0	0
Q6IPR3	Q6IPR3	tRNA wybutosine-synthesizing protein 3 homolog	TYW3	sp|Q6IPR3	1	1	1	0	1	0	1	0	1	0	1	0	1	0	1	6.9	29.794	0	5.3093	16386000	0	7973600	0	8412100	0	702870	0	345950
Q14BN4	Q14BN4	Sarcolemmal membrane-associated protein	SLMAP	sp|Q14BN	2	2	2	0	2	0	1	0	2	0	1	0	2	0	1	3	95.197	0	4.7795	16357000	0	7752500	0	8604900	0	585200	0	452050
Q7L5Y9	Q7L5Y9	Macrophage erythroblast attacher	MAEA	sp|Q7L5Y	1	1	1	0	1	0	1	0	1	0	1	0	1	0	1	2.3	45.287	0.001165	2.8824	16342000	0	5869400	0	10473000	0	517380	0	430690
Q9NR77	Q9NR77	Peroxisomal membrane protein 2	PXMP2	sp|Q9NR7	1	1	1	0	1	0	1	0	1	0	1	0	1	0	1	5.1	22.252	0.002005	2.2419	16322000	0	4538200	0	11784000	0	400040	0	484620
Q13506	Q13506	NGFI-A-binding protein 1	NAB1	sp|Q1350	1	1	1	0	0	1	1	0	0	1	1	0	0	1	1	3.3	54.401	0.000247	3.6704	16318000	0	0	1930400	14387000	0	0	1297200	591680
Q562E7	Q562E7	WD repeat-containing protein 81	WDR81	sp|Q562E	2	2	2	0	0	0	2	0	0	0	2	0	0	0	2	2	211.69	0.000249	3.7589	16277000	0	0	0	16277000	0	0	0	669410
Q9Y6X4	Q9Y6X4	Soluble lamin-associated protein of 75 kDa	FAM169A	sp|Q9Y6X	2	2	2	0	2	0	0	0	2	0	0	0	2	0	0	6	74.954	0	6.115	16213000	0	16213000	0	0	0	1429200	0	0
Q6PK04	Q6PK04	Coiled-coil domain-containing protein 137	CCDC137	sp|Q6PK0	2	2	2	0	0	1	2	0	0	1	2	0	0	1	2	10	33.231	0	3.947	16186000	0	0	1692900	14493000	0	0	1501000	232650
Q96G74	Q96G74	OTU domain-containing protein 5	OTUD5	sp|Q96G7	2	2	2	0	2	0	0	0	2	0	0	0	2	0	0	6.1	60.625	0	5.4727	16135000	0	16135000	0	0	0	1422300	0	0
O43286	O43286	Beta-1,4-galactosyltransferase 5	B4GALT5	sp|O4328	1	1	1	0	0	0	1	0	0	0	1	0	0	0	1	2.1	45.118	0.008469	1.6162	16088000	0	0	0	16088000	0	0	0	661650
Q96QA5	Q96QA5	Gasdermin-A	GSDMA	sp|Q96QA	2	2	2	0	1	2	0	0	1	2	0	0	1	2	0	3.8	49.364	0	4.3036	16068000	0	759970	15308000	0	0	109600	10245000	0
A0A1B0GT	A0A1B0GTU1			sp|A0A1B	2	1	1	0	2	0	2	0	1	0	1	0	1	0	1	5	88.936	0.004532	1.8313	16038000	0	12090000	0	3948200	0	1065700	0	162370
O75486	O75486	Transcription initiation protein SPT3 homolog	SUPT3H	sp|O7548	1	1	1	0	1	1	1	0	1	1	1	0	1	1	1	3.8	35.792	0.00025	3.8078	16037000	0	4879000	978390	10180000	0	425720	600940	479540
Q9P2K8	Q9P2K8	Eukaryotic translation initiation factor 2-alpha kin	EIF2AK4	sp|Q9P2K	1	1	1	0	0	0	1	0	0	0	1	0	0	0	1	1	186.91	0	5.6857	15957000	0	0	0	15957000	0	0	0	656250
P58557	P58557	Putative ribonuclease	YBEY	sp|P58557	2	2	2	0	1	0	2	0	1	0	2	0	1	0	2	13.8	19.298	0.000486	3.4693	15924000	0	3382200	0	12541000	0	602770	0	211140
Q96P11	Q96P11	Probable 28S rRNA (cytosine-C(5))-methyltransfe	NSUN5	sp|Q96P1	1	1	1	0	1	0	0	0	1	0	0	0	1	0	0	2.3	46.691	0.002657	2.1722	15909000	0	15909000	0	0	0	1402300	0	0
Q7RTS9	Q7RTS9	Dymeclin	DYM	sp|Q7RTS	1	1	1	0	0	0	1	0	0	0	1	0	0	0	1	1.5	75.935	0.001172	3.0003	15874000	0	0	0	15874000	0	0	0	652840
Q8TBY8	Q8TBY8	Polyamine-modulated factor 1-binding protein 1	PMFBP1	sp|Q8TBY	1	1	1	0	1	0	1	0	1	0	1	0	1	0	1	0.7	117.49	0.008466	1.6096	15806000	0	5709900	0	10096000	0	503320	0	415190
Q5VW38	Q5VW38	Protein GPR107	GPR107	sp|Q5VW3	1	1	1	0	1	0	1	0	1	0	1	0	1	0	1	1.8	66.99	0.001127	2.4099	15804000	0	6962800	0	8841700	0	613760	0	363620
Q8N8R5	Q8N8R5	UPF0565 protein C2orf69	C2orf69	sp|Q8N8R	1	1	1	0	1	0	0	0	1	0	0	0	1	0	0	4.2	43.448	0.003911	1.9518	15788000	0	15788000	0	0	0	1391700	0	0
Q9HAS0	Q9HAS0	Protein Njmu-R1	C17orf75	sp|Q9HAS	1	1	1	0	1	1	1	0	1	1	1	0	1	1	1	2.8	44.621	0.000245	3.5831	15689000	0	4886100	458920	10344000	0	430700	308390	425420
Q9BSC4	Q9BSC4	Nucleolar protein 10	NOL10	sp|Q9BSC	1	1	1	0	1	0	1	0	1	0	1	0	1	0	1	1.2	80.301	0.008451	1.5974	15671000	0	4237200	0	11434000	0	373500	0	470230
A0A1B0GV	A0A1B0GV	TRPM8 channel-associated factor 2	TCAF2	tr|A0A1B0	1	1	1	0	0	1	1	0	0	1	1	0	0	1	1	1.3	78.793	0.006005	1.763	15547000	0	0	928020	14619000	0	0	0	601210
Q9ULQ1	Q9ULQ1	Two pore calcium channel protein 1	TPCN1	sp|Q9ULQ	1	1	1	0	0	1	1	0	0	1	1	0	0	1	1	1.5	94.146	0.000248	3.7107	15526000	0	0	7087300	8438600	0	0	4762700	347040
Q00587	Q00587	Cdc42 effector protein 1	CDC42EP1	sp|Q00587	2	2	2	0	2	1	0	0	2	1	0	0	2	1	0	6.9	40.294	0.001146	2.6159	15514000	0	15126000	388200	0	0	1333300	0	0
Q8NFH5	Q8NFH5	Nucleoporin NUP53	NUP35	sp|Q8NFH	1	1	1	0	1	0	1	0	1	0	1	0	1	0	1	3.7	34.773	0.007455	1.6935	15507000	0	2755400	0	12751000	0	242890	0	524400
Q5VVQ6	Q5VVQ6	Ubiquitin thioesterase OTU1	YOD1	sp|Q5VVQ	2	2	2	0	2	0	0	0	2	0	0	0	2	0	0	10.9	38.322	0	18.78	15483000	0	15483000	0	0	0	1364800	0	0
Q9H1Y0	Q9H1Y0	Autophagy protein 5	ATG5	sp|Q9H1Y	1	1	1	0	1	0	1	0	1	0	1	0	1	0	1	3.3	32.447	0.007245	1.6966	15439000	0	5978800	0	9459800	0	527020	0	389040
Q5MIZ7	Q5MIZ7	Serine/threonine-protein phosphatase 4 regulato	SMEK2	sp|Q5MIZ	4	1	1	0	2	1	4	0	0	0	1	0	0	0	1	5.9	97.457	0	11.161	15396000	0	0	0	15396000	0	0	0	633180
O94763	O94763	Unconventional prefoldin RPB5 interactor 1	URI1	sp|O9476	1	1	1	0	1	0	1	0	1	0	1	0	1	0	1	1.7	59.832	0.001126	2.4001	15329000	0	7198200	0	8130500	0	634520	0	334370
Q969Q5	Q969Q5	Ras-related protein Rab-24	RAB24	sp|Q969Q	1	1	1	0	1	0	0	0	1	0	0	0	1	0	0	5.4	23.124	0.008643	1.5793	15280000	0	15280000	0	0	0	1346900	0	0
Q8WXH0	Q8WXH0	Nesprin-2	SYNE2	sp|Q8WXH	1	1	1	0	0	0	1	0	0	0	1	0	0	0	1	0.1	796.43	0.000483	3.3762	15269000	0	0	0	15269000	0	0	0	627940
Q9BQI0	Q9BQI0	Allograft inflammatory factor 1-like	AIF1L	sp|Q9BQI	1	1	1	0	1	0	0	0	1	0	0	0	1	0	0	6	17.068	0.007846	1.644	15223000	0	15223000	0	0	0	1341900	0	0
Q96T23	Q96T23	Remodeling and spacing factor 1	RSF1	sp|Q96T2	2	2	2	0	2	0	0	0	2	0	0	0	2	0	0	2.2	163.82	0	4.2155	15217000	0	15217000	0	0	0	1341400	0	0
Q9BW19	Q9BW19	Kinesin-like protein KIFC1	KIFC1	sp|Q9BW1	2	2	2	1	2	0	2	1	2	0	2	1	2	0	2	4	73.747	0	3.9649	15189000	1392300	6016100	0	7780800	1370600	650450	0	221470
Q9NX00	Q9NX00	Transmembrane protein 160	TMEM160	sp|Q9NX0	2	2	2	0	2	2	0	0	2	2	0	0	2	2	0	9	19.657	0.000486	3.466	15143000	0	7468300	7675000	0	0	656040	5159900	0
Q6QNY0	Q6QNY0	Biogenesis of lysosome-related organelles comple	BLOC1S3	sp|Q6QNY	1	1	1	0	1	0	1	0	1	0	1	0	1	0	1	9.4	21.255	0.002224	2.226	15077000	0	7386900	0	7690600	0	651150	0	316280
Q13416	Q13416	Origin recognition complex subunit 2	ORC2	sp|Q1341	2	2	2	0	2	0	0	0	2	0	0	0	2	0	0	4.2	65.971	0.001129	2.425	15042000	0	15042000	0	0	0	1326000	0	0
Q8NI36	Q8NI36	WD repeat-containing protein 36	WDR36	sp|Q8NI36	1	1	1	0	0	0	1	0	0	0	1	0	0	0	1	2.8	105.32	0.001138	2.5228	15037000	0	0	0	15037000	0	0	0	618410
Q9P2X3	Q9P2X3	Protein IMPACT	IMPACT	sp|Q9P2X	1	1	1	0	1	0	0	0	1	0	0	0	1	0	0	3.1	36.476	0.001567	2.3155	14963000	0	14963000	0	0	0	1319000	0	0
Q9NS91	Q9NS91	E3 ubiquitin-protein ligase RAD18	RAD18	sp|Q9NS9	1	1	1	0	1	0	1	0	1	0	1	0	1	0	1	1.6	56.222	0.004537	1.8487	14958000	0	6273600	0	8684500	0	553010	0	357150
Q6BDS2	Q6BDS2	UHRF1-binding protein 1	UHRF1BP1	sp|Q6BDS	1	1	1	0	1	0	0	0	1	0	0	0	1	0	0	0.9	159.48	0.000245	3.5932	14898000	0	14898000	0	0	0	1313300	0	0
Q9BTX7	Q9BTX7	Alpha-tocopherol transfer protein-like	TTPAL	sp|Q9BTX	1	1	1	0	0	0	1	0	0	0	1	0	0	0	1	7.9	38.515	0.001784	2.2591	14881000	0	0	0	14881000	0	0	0	611970

Q12884	Q12884	Prolyl endopeptidase FAP;Antiplasmin-cleaving er	FAP	sp|Q1288	1	1	1	0	0	0	1	0	0	0	1	0	0	0	1	2.5	87.711	0	8.9072	14862000	0	0	0	14862000	0	0	0	611190
Q6VN20	Q6VN20	Ran-binding protein 10	RANBP10	sp|Q6VN2	1	1	1	0	1	0	1	0	1	0	1	0	1	0	1	2.3	67.256	0	4.7606	14792000	0	4001300	0	10791000	0	352710	0	443790
O95210	O95210	Starch-binding domain-containing protein 1	STBD1	sp|O9521	1	1	1	0	0	0	1	0	0	0	1	0	0	0	1	4.2	39.007	0.000246	3.6332	14779000	0	0	0	14779000	0	0	0	607810
Q96Q45	Q96Q45	Transmembrane protein 237	TMEM237	sp|Q96Q4	1	1	1	0	0	0	1	0	0	0	1	0	0	0	1	4.2	45.525	0	4.7736	14762000	0	0	0	14762000	0	0	0	607090
Q9H6U8	Q9H6U8	Alpha-1,2-mannosyltransferase ALG9	ALG9	sp|Q9H6U	2	2	2	0	1	0	1	0	1	0	1	0	1	0	1	6.1	69.863	0.001133	2.4788	14719000	0	4707900	0	10011000	0	414990	0	0
P32780	P32780	General transcription factor IIH subunit 1	GTF2H1	sp|P32780	1	1	1	0	0	0	1	0	0	0	1	0	0	0	1	1.6	62.031	0.001563	2.275	14640000	0	0	0	14640000	0	0	0	602100
P50336	P50336	Protoporphyrinogen oxidase	PPOX	sp|P50336	1	1	1	0	0	0	1	0	0	0	1	0	0	0	1	3.6	50.765	0	5.0627	14608000	0	0	0	14608000	0	0	0	600770
Q9Y653	Q9Y653	G-protein coupled receptor 56;GPR56 N-terminal	GPR56	sp|Q9Y65	1	1	1	0	0	0	1	0	0	0	1	0	0	0	1	1.7	77.737	0.001155	2.7441	14547000	0	0	0	14547000	0	0	0	598260
Q53S33	Q53S33	BolA-like protein 3	BOLA3	sp|Q53S3	1	1	1	0	1	0	1	0	1	0	1	0	1	0	1	15.9	12.114	0.001153	2.7107	14523000	0	5685200	0	8838200	0	501140	0	363480
O14545	O14545	TRAF-type zinc finger domain-containing protein	TRAFD1	sp|O1454	2	2	2	0	2	1	2	0	2	1	2	0	2	1	2	3.8	64.84	0	4.1329	14469000	0	7685500	298860	6485000	0	640760	283230	221010
Q9P2Q2	Q9P2Q2	FERM domain-containing protein 4A	FRMD4A	sp|Q9P2Q	1	1	1	0	0	1	1	0	0	1	1	0	0	1	1	1.3	115.46	0.00117	2.963	14463000	0	0	1931100	12531000	0	0	1297700	515360
Q8NHV4	Q8NHV4	Protein NEDD1	NEDD1	sp|Q8NHV	1	1	1	0	1	0	1	0	1	0	1	0	1	0	1	2.7	71.965	0.001161	2.829	14459000	0	4975100	0	9484200	0	438550	0	390040
Q9Y3Q3	Q9Y3Q3	Transmembrane emp24 domain-containing prote	TMED3	sp|Q9Y3Q	1	1	1	0	1	0	1	0	1	0	1	0	1	0	1	4.6	24.777	0.007441	1.6754	14446000	0	4421100	0	10025000	0	0	0	412280
Q6ZSJ8	Q6ZSJ8	Uncharacterized protein C1orf122	C1orf122	sp|Q6ZSJ8	1	1	1	0	1	0	0	0	1	0	0	0	1	0	0	10	11.471	0.001164	2.8599	14444000	0	14444000	0	0	0	1273300	0	0
B3EWF7	B3EWF7	Laforin, isoform 9	EPM2A	sp|B3EWF	1	1	1	0	1	1	1	0	1	1	1	0	1	1	1	3.5	35.169	0.000487	3.4998	14314000	0	9628700	482970	4202100	0	924080	244000	178050
Q99735	Q99735	Microsomal glutathione S-transferase 2	MGST2	sp|Q9973	1	1	1	0	1	0	0	0	1	0	0	0	1	0	0	9.5	16.62	0.00328	2.0432	14311000	0	14311000	0	0	0	1261500	0	0
Q1ED39	Q1ED39	Lysine-rich nucleolar protein 1	KNOP1	sp|Q1ED3	2	2	2	0	2	2	1	0	2	2	1	0	2	2	1	5.5	51.588	0.001157	2.7728	14152000	0	7989200	2511500	3650900	0	809590	1442900	289650
Q8IW45	Q8IW45	ATP-dependent (S)-NAD(P)H-hydrate dehydratase	CARKD	sp|Q8IW4	1	1	1	0	1	0	1	0	1	0	1	0	1	0	1	3.7	36.576	0.00095	3.168	14115000	0	4118600	0	9996200	0	363050	0	411100
Q9UM13	Q9UM13	Anaphase-promoting complex subunit 10	ANAPC10	sp|Q9UM1	3	3	3	0	1	3	0	0	1	3	0	0	1	3	0	23.8	21.252	0.000487	3.485	14108000	0	2881000	11227000	0	0	505960	7292500	0
O15541	O15541	RING finger protein 113A	RNF113A	sp|O15541	1	1	1	0	1	0	1	0	1	0	1	0	1	0	1	3.8	38.787	0.001135	2.4889	14097000	0	8154200	0	5942700	0	718790	0	244400
Q9Y448	Q9Y448	Small kinetochore-associated protein	KNSTRN	sp|Q9Y44	2	2	2	0	1	0	2	0	1	0	2	0	1	0	2	7	35.438	0	5.4828	14050000	0	1749900	0	12300000	0	0	0	505840
Q8TEB9	Q8TEB9	Rhomboid-related protein 4	RHBDD1	sp|Q8TEB	1	1	1	0	0	0	1	0	0	0	1	0	0	0	1	5.4	35.823	0	6.7581	14041000	0	0	0	14041000	0	0	0	577460
Q96BN8	Q96BN8	Ubiquitin thioesterase otulin	OTULIN	sp|Q96BN	2	2	2	0	2	0	0	0	2	0	0	0	2	0	0	6	40.262	0	4.0024	14014000	0	14014000	0	0	0	1235300	0	0
Q5T8D3	Q5T8D3	Acyl-CoA-binding domain-containing protein 5	ACBD5	sp|Q5T8D	1	1	1	0	0	0	1	0	0	0	1	0	0	0	1	1.9	60.091	0.001347	2.3712	14004000	0	0	0	14004000	0	0	0	575940
O75530	O75530	Polycomb protein EED	EED	sp|O7553	2	2	2	0	2	0	2	0	2	0	2	0	2	0	2	6.3	50.197	0.001785	2.2645	13964000	0	7651900	0	6312500	0	527530	0	406580
Q96BN2	Q96BN2	Transcriptional adapter 1	TADA1	sp|Q96BN	2	2	2	0	2	0	0	0	2	0	0	0	2	0	0	6.3	37.381	0.001141	2.5752	13918000	0	13918000	0	0	0	1226800	0	0
Q9NZ43	Q9NZ43	Vesicle transport protein USE1	USE1	sp|Q9NZ4	1	1	1	0	0	0	1	0	0	0	1	0	0	0	1	4.2	29.371	0.00113	2.4347	13889000	0	0	0	13889000	0	0	0	571180
Q6ZRQ5	Q6ZRQ5	Protein MMS22-like	MMS22L	sp|Q6ZRQ	1	1	1	0	1	0	1	0	1	0	1	0	1	0	1	0.9	142.32	0.001562	2.2702	13881000	0	4277400	0	9604100	0	0	0	394970
Q8N138	Q8N138	ORM1-like protein 3	ORMDL3	sp|Q8N13	2	1	1	0	1	0	2	0	1	0	1	0	1	0	1	17	17.494	0	4.0137	13821000	0	3281800	0	10539000	0	289290	0	433430
Q96RF0	Q96RF0	Sorting nexin-18	SNX18	sp|Q96RF	2	2	2	0	0	0	2	0	0	0	2	0	0	0	2	4.6	68.894	0	4.0755	13796000	0	0	0	13796000	0	0	0	567390
P13686	P13686	Tartrate-resistant acid phosphatase type 5	ACP5	sp|P13686	1	1	1	0	0	0	1	0	0	0	1	0	0	0	1	3.7	36.598	0.001157	2.777	13787000	0	0	0	13787000	0	0	0	566990
Q9Y619	Q9Y619	Mitochondrial ornithine transporter 1	SLC25A15	sp|Q9Y61	1	1	1	0	1	0	1	0	1	0	1	0	1	0	1	4	32.736	0.008462	1.608	13768000	0	0	0	13768000	0	0	0	566210
Q6AI08	Q6AI08	HEAT repeat-containing protein 6	HEATR6	sp|Q6AI08	3	3	3	0	3	0	0	0	3	0	0	0	3	0	0	3.5	128.78	0	17.421	13728000	0	13728000	0	0	0	1210100	0	0
Q7Z5H3	Q7Z5H3	Rho GTPase-activating protein 22	ARHGAP22	sp|Q7Z5H	3	3	3	0	0	2	1	0	0	2	1	0	0	2	1	6.9	76.778	0.00025	3.7936	13726000	0	0	5057600	8668500	0	0	3398700	0
P51606	P51606	N-acylglucosamine 2-epimerase	RENBP	sp|P51606	1	1	1	0	0	0	1	0	0	0	1	0	0	0	1	3.3	48.83	0.000245	3.5953	13699000	0	0	0	13699000	0	0	0	563370
P01594;P0	P01594;P0	Ig kappa chain V-I region AU;Ig kappa chain V-I region AG		sp|P01594	1	1	1	0	0	1	0	0	0	1	0	0	0	1	0	13.7	12.848	0.000723	3.3571	13699000	0	0	13699000	0	0	0	9205400	0
Q8IZ69	Q8IZ69	tRNA (uracil-5-)-methyltransferase homolog A	TRMT2A	sp|Q8IZ69	2	2	2	0	2	1	1	0	2	1	1	0	2	1	1	3.5	68.725	0.00516	1.7911	13692000	0	5375200	970950	7346000	0	409740	691470	327180
Q9NY12	Q9NY12	H/ACA ribonucleoprotein complex subunit 1	GAR1	sp|Q9NY1	2	2	2	0	1	2	1	0	1	2	1	0	1	2	1	7.4	22.348	0.000246	3.6423	13671000	0	3080400	2867700	7723100	0	586690	1563000	366560
Q9BRS2	Q9BRS2	Serine/threonine-protein kinase RIO1	RIOK1	sp|Q9BRS	2	2	2	0	2	0	0	0	2	0	0	0	2	0	0	3.9	65.582	0.001783	2.2517	13543000	0	13543000	0	0	0	1193800	0	0
Q4VCS5;Q8	Q4VCS5;Q8	Angiomotin;Angiomotin-like protein 1	AMOT;AM	sp|Q4VCS	2	2	2	0	2	0	0	0	2	0	0	0	2	0	0	1.7	118.08	0.003076	2.0926	13478000	0	13478000	0	0	0	1188100	0	0
Q9UBS8	Q9UBS8	E3 ubiquitin-protein ligase RNF14	RNF14	sp|Q9UBS	2	2	2	0	2	0	1	0	2	0	1	0	2	0	1	8.6	53.837	0.001565	2.2993	13384000	0	8815000	0	4568900	0	488080	0	476850
P54646	P54646	5-AMP-activated protein kinase catalytic subunit	PRKAA2	sp|P54646	2	1	1	0	2	0	1	0	1	0	0	0	1	0	0	8.9	62.319	0.001145	2.6125	13348000	0	13348000	0	0	0	1176600	0	0
Q96C11	Q96C11	FGGY carbohydrate kinase domain-containing pro	FGGY	sp|Q96C1	1	1	1	0	0	0	1	0	0	0	1	0	0	0	1	2.5	59.992	0.001137	2.518	13320000	0	0	0	13320000	0	0	0	547800
Q7Z4F1	Q7Z4F1	Low-density lipoprotein receptor-related protein	LRP10	sp|Q7Z4F1	1	1	1	0	0	0	1	0	0	0	1	0	0	0	1	2.9	76.193	0	4.0868	13140000	0	0	0	13140000	0	0	0	540400
Q2VPB7	Q2VPB7	AP-5 complex subunit beta-1	AP5B1	sp|Q2VPB	1	1	1	0	0	0	1	0	0	0	1	0	0	0	1	1.4	93.948	0.001143	2.5879	13038000	0	0	0	13038000	0	0	0	536200
Q96GY0	Q96GY0	Zinc finger C2HC domain-containing protein 1A	ZC2HC1A	sp|Q96GY	2	2	2	0	1	2	2	0	1	2	2	0	1	2	2	7.1	35.092	0.000714	3.2308	13009000	0	1626300	1424900	9958100	0	341490	734730	434220
P0CG08;B7	P0CG08;B7	Golgi pH regulator B;Golgi pH regulator A	GPR89B;GI	sp|P0CG0	1	1	1	0	1	0	0	0	1	0	0	0	1	0	0	5.9	52.916	0.008439	1.5905	12987000	0	12987000	0	0	0	1144800	0	0
Q9UJK0	Q9UJK0	Ribosome biogenesis protein TSR3 homolog	TSR3	sp|Q9UJK	2	2	2	0	0	0	2	0	0	0	2	0	0	0	2	9	33.596	0.000245	3.5665	12982000	0	0	0	12982000	0	0	0	533890
Q9H6Y2	Q9H6Y2	WD repeat-containing protein 55	WDR55	sp|Q9H6Y	2	2	2	0	0	1	1	0	0	1	1	0	0	1	1	4.7	42.07	0.004736	1.8091	12969000	0	0	863220	12106000	0	0	580080	0
O43759	O43759	Synaptogyrin-1	SYNGR1	sp|O4375	1	1	1	0	1	0	0	0	1	0	0	0	1	0	0	5.2	25.455	0.002875	2.1577	12939000	0	12939000	0	0	0	1140500	0	0
Q9H1A3	Q9H1A3	Methyltransferase-like protein 9	METTL9	sp|Q9H1A	1	1	1	0	0	0	1	0	0	0	1	0	0	0	1	6.3	36.536	0	4.5056	12889000	0	0	0	12889000	0	0	0	530090
Q00653	Q00653	Nuclear factor NF-kappa-B p100 subunit;Nuclear f	NFKB2	sp|Q0065	1	1	1	0	0	0	1	0	0	0	1	0	0	0	1	2.1	96.748	0.007847	1.6442	12875000	0	0	0	12875000	0	0	0	529480
Q96JC1	Q96JC1	Vam6/Vps39-like protein	VPS39	sp|Q96JC1	1	1	1	0	1	0	1	0	1	0	1	0	1	0	1	1.8	101.81	0.000482	3.3723	12736000	0	5548200	0	7187600	0	489070	0	295590
O94788	O94788	Retinal dehydrogenase 2	ALDH1A2	sp|O9478	4	2	2	0	4	1	2	0	2	0	0	0	2	0	0	9.3	56.723	0.001128	2.4172	12730000	0	12730000	0	0	0	1122100	0	0
Q15274	Q15274	Nicotinate-nucleotide pyrophosphorylase [carbox	QPRT	sp|Q1527	2	2	2	0	2	0	0	0	2	0	0	0	2	0	0	7.4	30.845	0.001145	2.6071	12715000	0	12715000	0	0	0	1120900	0	0
P38432	P38432	Coilin	COIL	sp|P38432	1	1	1	0	1	0	1	0	1	0	1	0	1	0	1	2.8	62.608	0	4.4076	12680000	0	6242700	0	6437400	0	550290	0	264740
O60669	O60669	Monocarboxylate transporter 2	SLC16A7	sp|O6066	1	1	1	0	0	1	1	0	0	1	1	0	0	1	1	2.3	52.2	0.000245	3.5984	12676000	0	0	808890	11867000	0	0	543570	488030
P35367	P35367	Histamine H1 receptor	HRH1	sp|P35367	1	1	1	0	0	0	1	0	0	0	1	0	0	0	1	5.7	55.784	0.00025	3.8034	12661000	0	0	0	12661000	0	0	0	520700
Q86UA6	Q86UA6	RPA-interacting protein	RPAIN	sp|Q86UA	2	2	2	0	2	0	1	0	2	0	1	0	2	0	1	6.8	24.77	0.002872	2.1479	12649000	0	8138400	0	4511000	0	717390	0	0
Q96ES7	Q96ES7	SAGA-associated factor 29 homolog	CCDC101	sp|Q96ES	1	1	1	0	1	0	0	0	1	0	0	0	1	0	0	4.4	33.238	0.003491	2.0089	12567000	0	12567000	0	0	0	1107700	0	0
Q86V85	Q86V85	Integral membrane protein GPR180	GPR180	sp|Q86V8	1	1	1	0	1	0	1	0	1	0	1	0	1	0	1	3.4	49.395	0.000718	3.2953	12502000	0	4095000	0	8406700	0	360970	0	345730
Q8TDM6	Q8TDM6	Disks large homolog 5	DLG5	sp|Q8TDM	3	3	3	0	0	3	0	0	0	3	0	0	0	3	0	2	213.87	0	4.367	12437000	0	0	12437000	0	0	0	8357900	0
Q9BT88	Q9BT88	Synaptotagmin-11	SYT11	sp|Q9BT8	2	2	2	0	1	2	0	0	1	2	0	0	1	2	0	4.9	48.296	0	5.9587	12364000	0	8478300	3886100	0	0	1986100	1372800	0
Q8TAP9	Q8TAP9	M-phase-specific PLK1-interacting protein	MPLKIP	sp|Q8TAP	2	2	2	0	2	1	1	0	2	1	1	0	2	1	1	19	19.147	0.000944	3.0803	12342000	0	8890900	524440	2926900	0	729910	332360	194240
Q96RU3	Q96RU3	Formin-binding protein 1	FNBP1	sp|Q96RU	1	1	1	0	1	0	1	0	1	0	1	0	1	0	1	1.6	71.306	0.003294	2.0866	12318000	0	5872900	0	6445100	0	517690	0	265060
Q9UBL6	Q9UBL6	Copine-7	CPNE7	sp|Q9UBL	2	1	1	0	1	1	2	0	0	0	1	0	0	0	1	3.8	70.293	0.003084	2.1184	12290000	0	0	0	12290000	0	0	0	505440
Q49A26	Q49A26	Putative oxidoreductase GLYR1	GLYR1	sp|Q49A2	1	1	1	0	0	1	1	0	0	1	1	0	0	1	1	3.1	60.547	0.001162	2.8395	12203000	0	0	2391300	9811200	0	0	1606900	403490
O75943	O75943	Cell cycle checkpoint protein RAD17	RAD17	sp|O7594	1	1	1	0	0	0	1	0	0	0	1	0	0	0	1	1.2	77.054	0.003284	2.062	12144000	0	0	0	12144000	0	0	0	499430
P52564	P52564	Dual specificity mitogen-activated protein kinase	MAP2K6	sp|P52564	3	2	2	0	3	0	1	0	2	0	0	0	2	0	0	10.8	37.492	0.001146	2.6206	12014000	0	12014000	0	0	0	1059100	0	0
A4D1P6	A4D1P6	WD repeat-containing protein 91	WDR91	sp|A4D1P	1	1	1	0	1	0	1	0	1	0	1	0	1	0	1	2.4	83.343	0.001129	2.4184	11982000	0	3411200	0	8571100	0	0	0	352490
Q9NUM3	Q9NUM3	Zinc transporter ZIP9	SLC39A9	sp|Q9NUN	1	1	1	0	0	1	1	0	0	1	1	0	0	1	1	4.9	32.251	0.001154	2.725	11939000	0	0	373910	11565000	0	0	251260	475620
Q8TED0	Q8TED0	U3 small nucleolar RNA-associated protein 15 hor	UTP15	sp|Q8TED	1	1	1	0	1	1	1	0	1	1	1	0	1	1	1	1.9	58.414	0.001126	2.4068	11922000	0	2790100	847740	8284400	0	318780	485160	352390
Q8IXW5	Q8IXW5	Putative RNA polymerase II subunit B1 CTD phosp	RPAP2	sp|Q8IXW	2	2	2	0	2	0	0	0	2	0	0	0	2	0	0	6	69.508	0	4.2191	11890000	0	11890000	0	0	0	1048000	0	0
Q9UK73	Q9UK73	Protein fem-1 homolog B	FEM1B	sp|Q9UK7	1	1	1	0	0	0	1	0	0	0	1	0	0	0	1	1.8	70.263	0.000486	3.4774	11861000	0	0	0	11861000	0	0	0	487790
P11274	P11274	Breakpoint cluster region protein	BCR	sp|P11274	3	2	2	0	3	0	2	0	2	0	1	0	2	0	1	2.8	142.82	0.000951	3.1925	11802000	0	8074100	0	3728200	0	711720	0	0
Q8NEZ2	Q8NEZ2	Vacuolar protein sorting-associated protein 37A	VPS37A	sp|Q8NEZ	1	1	1	0	0	0	1	0	0	0	1	0	0	0	1	3.3	44.314	0.006014	1.776	11691000	0	0	0	11691000	0	0	0	480810
O43353	O43353	Receptor-interacting serine/threonine-protein kir	RIPK2	sp|O4335	1	1	1	0	0	0	1	0	0	0	1	0	0	0	1	1.9	61.194	0.001143	2.5876	11683000	0	0	0	11683000	0	0	0	480480
O15212	O15212	Prefoldin subunit 6	PFDN6	sp|O1521	1	1	1	0	1	1	1	0	1	1	1	0	1	1	1	6.2	14.582	0.008637	1.5715	11670000	0	5401200	592330	5676500	0	476110	398040	233450
Q53FT3	Q53FT3	Protein Hikeshi	C11orf73	sp|Q53FT	1	1	1	0	1	0	1	0	1	0	1	0	1	0	1	6.6	21.627	0.001133	2.4773	11640000	0	5236900	0	6403100	0	461630	0	263330
O14772	O14772	Fucose-1-phosphate guanylyltransferase	FPGT	sp|O1477	2	2	2	0	2	0	0	0	2	0	0	0	2	0	0	4.4	68.009	0.003084	2.1183	11503000	0	11503000	0	0	0	1013900	0	0
Q8N5A5	Q8N5A5	Zinc finger CCCH-type with G patch domain-conta	ZGPAT	sp|Q8N5A	1	1	1	0	1	0	1	0	1	0	1	0	1	0	1	3.4	57.358	0.000249	3.7871	11368000	0	2947600	0	8420300	0	259820	0	346290
Q96PV6	Q96PV6	Leukocyte receptor cluster member 8	LENG8	sp|Q96PV	1	1	1	0	0	0	1	0	0	0	1	0	0	0	1	1.6	88.156	0.000488	3.5014	11348000	0	0	0	11348000	0	0	0	466700
Q9Y6M5	Q9Y6M5	Zinc transporter 1	SLC30A1	sp|Q9Y6M	1	1	1	0	0	0	1	0	0	0	1	0	0	0	1	2.4	55.299	0.006405	1.7163	11301000	0	0	0	11301000	0	0	0	464760
Q8N3P4	Q8N3P4	Vacuolar protein sorting-associated protein 8 hon	VPS8	sp|Q8N3P	1	1	1	0	0	0	1	0	0	0	1	0	0	0	1	0.8	161.75	0.001566	2.305	11234000	0	0	0	11234000	0	0	0	462000
Q9H173	Q9H173	Nucleotide exchange factor SIL1	SIL1	sp|Q9H17	1	1	1	1	1	0	1	1	1	0	1	1	1	0	1	2.8	52.084	0.001168	2.929	11212000	1769900	4578900	0	4863100	1769900	403630	0	200000
Q9NXG6	Q9NXG6	Transmembrane prolyl 4-hydroxylase	P4HTM	sp|Q9NXG	1	1	1	0	0	0	1	0	0	0	1	0	0	0	1	2.8	56.66	0.004743	1.8226	11194000	0	0	0	11194000	0	0	0	460340
Q96B02	Q96B02	Ubiquitin-conjugating enzyme E2 W	UBE2W	sp|Q96B0	1	1	1	0	0	0	1	0	0	0	1	0	0	0	1	11.9	17.331	0.00113	2.4404	11186000	0	0	0	11186000	0	0	0	460020
Q969Y2	Q969Y2	tRNA modification GTPase GTPBP3, mitochondria	GTPBP3	sp|Q969Y	2	2	2	0	2	0	0	0	2	0	0	0	2	0	0	5.9	52.058	0	4.2244	11148000	0	11148000	0	0	0	982650	0	0
O43826	O43826	Glucose-6-phosphate translocase	SLC37A4	sp|O4382	2	2	2	0	2	0	1	0	2	0	1	0	2	0	1	5.4	46.36	0.000247	3.678	11109000	0	7423200	0	3686100	0	654350	0	151590

P54252	P54252	Ataxin-3	ATXN3	sp|P54252	1	1	1	0	0	0	1	0	0	0	1	0	0	0	1	5.5	41.25	0.001144	2.5997	11076000	0	0	0	11076000	0	0	0	455510
Q9Y248	Q9Y248	DNA replication complex GINS protein PSF2	GINS2	sp|Q9Y248	1	1	1	0	1	0	0	0	1	0	0	0	1	0	0	6.5	21.427	0.001149	2.6562	11049000	0	11049000	0	0	0	973970	0	0
Q14966	Q14966	Zinc finger protein 638	ZNF638	sp|Q14966	2	2	2	0	1	2	1	0	1	2	1	0	1	2	1	1.1	220.62	0.001351	2.3927	10991000	0	3109200	4123500	3757900	0	0	2771000	0
Q9Y2S2	Q9Y2S2	Lambda-crystallin homolog	CRYL1	sp|Q9Y2S2	1	1	1	0	1	0	1	0	1	0	1	0	1	0	1	3.4	35.419	0.008054	1.6377	10990000	0	2581100	0	8409200	0	0	0	345830
Q9H6U6	Q9H6U6	Breast carcinoma-amplified sequence 3	BCAS3	sp|Q9H6U	1	1	1	0	1	0	0	0	1	0	0	0	1	0	0	2.7	101.24	0.000246	3.6171	10978000	0	10978000	0	0	0	967720	0	0
Q5HYI7	Q5HYI7	Metaxin-3	MTX3	sp|Q5HYI7	1	1	1	0	1	0	0	0	1	0	0	0	1	0	0	5.8	35.093	0.00966	1.5328	10956000	0	10956000	0	0	0	965750	0	0
Q49A17	Q49A17	Polypeptide N-acetylgalactosaminyltransferase-lik	GALNTL6	sp|Q49A17	1	1	1	0	1	0	0	0	1	0	0	0	1	0	0	1.3	69.787	0.004541	1.8629	10898000	0	10898000	0	0	0	960600	0	0
A5PLN9	A5PLN9	Trafficking protein particle complex subunit 13	TRAPPC13	sp|A5PLN9	1	1	1	0	0	0	1	0	0	0	1	0	0	0	1	7.9	46.523	0.000249	3.7419	10879000	0	0	0	10879000	0	0	0	447390
Q15814	Q15814	Tubulin-specific chaperone C	TBCC	sp|Q15814	1	1	1	0	1	0	0	0	1	0	0	0	1	0	0	6.1	39.248	0.003282	2.0552	10839000	0	10839000	0	0	0	955470	0	0
Q9NZN5	Q9NZN5	Rho guanine nucleotide exchange factor 12	ARHGEF12	sp|Q9NZN	2	2	2	0	2	0	0	0	2	0	0	0	2	0	0	1.8	173.23	0.007444	1.6791	10819000	0	10819000	0	0	0	953720	0	0
Q5SRE7	Q5SRE7	Phytanoyl-CoA dioxygenase domain-containing pr	PHYHD1	sp|Q5SRE7	2	2	2	0	2	0	0	0	2	0	0	0	2	0	0	10.3	32.41	0	6.1433	10787000	0	10787000	0	0	0	950880	0	0
P01112	P01112	GTPase HRas;GTPase HRas, N-terminally processe	HRAS	sp|P01112	6	1	1	0	3	0	6	0	0	0	1	0	0	0	1	34.4	21.298	0.001131	2.4491	10702000	0	0	0	10702000	0	0	0	440140
O75864	O75864	Protein phosphatase 1 regulatory subunit 37	PPP1R37	sp|O75864	1	1	1	0	0	0	1	0	0	0	1	0	0	0	1	2.5	74.766	0	5.1269	10657000	0	0	0	10657000	0	0	0	438280
Q9BSE5	Q9BSE5	Agmatinase, mitochondrial	AGMAT	sp|Q9BSE5	1	1	1	0	1	0	0	0	1	0	0	0	1	0	0	8.5	37.66	0.002666	2.2008	10657000	0	10657000	0	0	0	939440	0	0
Q99943	Q99943	1-acyl-sn-glycerol-3-phosphate acyltransferase alp	AGPAT1	sp|Q99943	1	1	1	0	1	0	0	0	1	0	0	0	1	0	0	7.4	31.716	0.001163	2.8505	10622000	0	10622000	0	0	0	936320	0	0
Q8WVQ1	Q8WVQ1	Soluble calcium-activated nucleotidase 1	CANT1	sp|Q8WVQ	1	1	1	0	0	0	1	0	0	0	1	0	0	0	1	4.5	44.839	0.001155	2.7525	10604000	0	0	0	10604000	0	0	0	436090
O43414	O43414	ERI1 exoribonuclease 3	ERI3	sp|O43414	2	2	2	0	1	1	1	0	1	1	1	0	1	1	1	4.7	37.238	0.00845	1.5972	10591000	0	0	692230	9898800	0	0	465170	407090
O43301	O43301	Heat shock 70 kDa protein 12A	HSPA12A	sp|O43301	1	1	1	0	1	0	0	0	1	0	0	0	1	0	0	2.1	74.977	0	4.4095	10590000	0	10590000	0	0	0	933540	0	0
Q969S2	Q969S2	Endonuclease 8-like 2	NEIL2	sp|Q969S2	1	1	1	0	0	0	1	0	0	0	1	0	0	0	1	5.1	36.826	0	4.9087	10581000	0	0	0	10581000	0	0	0	435130
Q9UKT5	Q9UKT5	F-box only protein 4	FBXO4	sp|Q9UKT5	1	1	1	0	1	0	1	0	1	0	1	0	1	0	1	3.1	44.136	0.009666	1.5399	10547000	0	3852200	0	6694300	0	339560	0	275310
Q9H300	Q9H300	Presenilins-associated rhomboid-like protein, mit	PARL	sp|Q9H300	1	1	1	0	0	1	1	0	0	1	1	0	0	1	1	2.4	42.19	0.003089	2.121	10525000	0	0	1233800	9291300	0	0	829100	382110
P26374	P26374	Rab proteins geranylgeranyltransferase compone	CHML	sp|P26374	1	1	1	0	0	0	1	0	0	0	1	0	0	0	1	2.9	74.071	0	4.018	10494000	0	0	0	10494000	0	0	0	431560
Q9H0G5	Q9H0G5	Nuclear speckle splicing regulatory protein 1	NSRP1	sp|Q9H0G	1	1	1	0	1	1	1	0	1	1	1	0	1	1	1	2.7	66.389	0.000484	3.4056	10462000	0	2771100	1214100	6476800	0	244270	815860	266360
P05090	P05090	Apolipoprotein D	APOD	sp|P05090	2	2	2	1	0	2	0	1	0	2	0	1	0	2	0	9.5	21.275	0.001571	2.3533	10392000	486090	0	9906300	0	2839700	0	4303400	0
Q96JQ2	Q96JQ2	Calmin	CLMN	sp|Q96JQ2	1	1	1	0	0	0	1	0	0	0	1	0	0	0	1	1.7	111.65	0.000718	3.2875	10378000	0	0	0	10378000	0	0	0	426800
Q9NUP7	Q9NUP7	tRNA:m(4)X modification enzyme TRM13 homolo	TRMT13	sp|Q9NUP	1	1	1	0	1	0	1	0	1	0	1	0	1	0	1	3.5	54.246	0.007445	1.6849	10357000	0	4336400	0	6021000	0	382250	0	247620
Q9NWH9	Q9NWH9	SAFB-like transcription modulator	SLTM	sp|Q9NWH	2	2	2	0	0	1	1	0	0	1	1	0	0	1	1	1.9	117.15	0	12.238	10355000	0	0	2954000	7400800	0	0	0	304360
O75379	O75379	Vesicle-associated membrane protein 4	VAMP4	sp|O75379	1	1	1	0	1	0	1	0	1	0	1	0	1	0	1	9.9	16.397	0.007442	1.6755	10342000	0	3723900	0	6618500	0	328260	0	272190
Q6PJ69	Q6PJ69	Tripartite motif-containing protein 65	TRIM65	sp|Q6PJ69	2	2	2	0	2	0	2	0	2	0	2	0	2	0	2	4.4	57.353	0	7.2112	10338000	0	4831900	0	5506200	0	425930	0	226450
Q96QG7	Q96QG7	Myotubularin-related protein 9	MTMR9	sp|Q96QG	1	1	1	0	1	0	1	0	1	0	1	0	1	0	1	1.5	63.461	0.002659	2.1771	10314000	0	5182200	0	5131900	0	456800	0	211050
P01040	P01040	Cystatin-A;Cystatin-A, N-terminally processed	CSTA	sp|P01040	2	2	2	0	1	2	0	0	1	2	0	0	1	2	0	25.5	11.006	0.000486	3.4784	10288000	0	2045900	8242600	0	0	509120	5210200	0
Q14574	Q14574	Desmocollin-3	DSC3	sp|Q14574	2	2	2	0	0	2	0	0	0	2	0	0	0	2	0	2.6	99.968	0.001141	2.5693	10248000	0	0	10248000	0	0	0	6886800	0
Q9UJC5	Q9UJC5	SH3 domain-binding glutamic acid-rich-like protei	SH3BGRL2	sp|Q9UJC5	1	1	1	0	1	0	0	0	1	0	0	0	1	0	0	13.1	12.326	0.002665	2.1933	10211000	0	10211000	0	0	0	900070	0	0
O43159	O43159	Ribosomal RNA-processing protein 8	RRP8	sp|O43159	1	1	1	0	1	1	1	0	1	1	1	0	1	1	1	1.8	50.714	0.003278	2.0395	10204000	0	1366700	1837700	6999400	0	120470	1234900	287850
Q9H7E9	Q9H7E9	UPF0488 protein C8orf33	C8orf33	sp|Q9H7E9	2	2	2	0	2	1	0	0	2	1	0	0	2	1	0	9.2	24.992	0.001144	2.5973	10188000	0	9497500	690260	0	0	570380	730670	0
Q8N573	Q8N573	Oxidation resistance protein 1	OXR1	sp|Q8N573	2	2	2	0	1	0	2	0	1	0	2	0	1	0	2	3.8	97.969	0	13.18	10145000	0	3251600	0	6893600	0	286620	0	283510
P34741	P34741	Syndecan-2	SDC2	sp|P34741	2	2	2	0	2	0	0	0	2	0	0	0	2	0	0	11.4	22.16	0	3.8359	10119000	0	10119000	0	0	0	892020	0	0
Q96EP0	Q96EP0	E3 ubiquitin-protein ligase RNF31	RNF31	sp|Q96EP0	1	1	1	0	0	0	1	0	0	0	1	0	0	0	1	2	119.65	0.000721	3.3324	10055000	0	0	0	10055000	0	0	0	413500
Q9UDY4	Q9UDY4	DnaJ homolog subfamily B member 4	DNAJB4	sp|Q9UDY	1	1	1	0	1	0	1	0	1	0	1	0	1	0	1	3	37.806	0.008644	1.5794	10055000	0	3118700	0	6935800	0	0	0	285240
Q9BQ51	Q9BQ51	Programmed cell death 1 ligand 2	PDCD1LG2	sp|Q9BQ5	1	1	1	0	0	1	1	0	0	1	1	0	0	1	1	4.4	30.957	0.005803	1.7839	10041000	0	0	966860	9074500	0	0	649720	373190
Q53TN4	Q53TN4	Cytochrome b reductase 1	CYBRD1	sp|Q53TN4	1	1	1	0	0	0	1	0	0	0	1	0	0	0	1	3.5	31.641	0.002658	2.1722	10022000	0	0	0	10022000	0	0	0	412170
O43741	O43741	5-AMP-activated protein kinase subunit beta-2	PRKAB2	sp|O43741	2	2	2	0	2	0	0	0	2	0	0	0	2	0	0	15.8	30.302	0.001147	2.6243	9987000	0	9987000	0	0	0	880350	0	0
Q9H6W3	Q9H6W3	Bifunctional lysine-specific demethylase and histic	NO66	sp|Q9H6W	2	2	2	0	0	2	1	0	0	2	1	0	0	2	1	3.7	71.085	0.001176	3.0511	9971300	0	0	5520400	4450900	0	0	3543000	349790
Q6P3X3	Q6P3X3	Tetratricopeptide repeat protein 27	TTC27	sp|Q6P3X3	2	2	2	0	2	0	0	0	2	0	0	0	2	0	0	2.7	96.631	0.000482	3.3697	9966100	0	9966100	0	0	0	878500	0	0
Q9NV35	Q9NV35	Probable 8-oxo-dGTP diphosphatase NUDT15	NUDT15	sp|Q9NV35	1	1	1	0	1	0	0	0	1	0	0	0	1	0	0	6.7	18.609	0.001568	2.3244	9958300	0	9958300	0	0	0	877810	0	0
Q7Z5R6	Q7Z5R6	Amyloid beta A4 precursor protein-binding family	APBB1IP	sp|Q7Z5R6	1	1	1	0	0	0	1	0	0	0	1	0	0	0	1	3	73.182	0.00135	2.3925	9887800	0	0	0	9887800	0	0	0	406640
P51843	P51843	Nuclear receptor subfamily 0 group B member 1	NR0B1	sp|P51843	1	1	1	0	0	0	1	0	0	0	1	0	0	0	1	3.4	51.717	0	4.4081	9870800	0	0	0	9870800	0	0	0	405940
Q9BRX5	Q9BRX5	DNA replication complex GINS protein PSF3	GINS3	sp|Q9BRX5	2	2	2	0	2	0	0	0	2	0	0	0	2	0	0	15.7	24.534	0	4.3813	9866600	0	9866600	0	0	0	869730	0	0
Q9H8H2	Q9H8H2	Probable ATP-dependent RNA helicase DDX31	DDX31	sp|Q9H8H	1	1	1	0	0	1	0	0	0	1	0	0	0	1	0	2	94.086	0.007038	1.7057	9864700	0	0	9864700	0	0	0	6629000	0
O43572	O43572	A-kinase anchor protein 10, mitochondrial	AKAP10	sp|O43572	2	2	2	0	0	0	2	0	0	0	2	0	0	0	2	4.8	73.817	0	5.643	9855500	0	0	0	9855500	0	0	0	405310
Q9H4I9	Q9H4I9	Essential MCU regulator, mitochondrial	SMDT1	sp|Q9H4I9	1	1	1	0	1	0	1	0	1	0	1	0	1	0	1	6.5	11.441	0.006412	1.7245	9842600	0	2868400	0	6974200	0	252840	0	286820
Q96MG8	Q96MG8	Protein-L-isoaspartate O-methyltransferase doma	PCMTD1	sp|Q96MG	2	2	2	0	0	2	0	0	0	2	0	0	0	2	0	7.3	40.675	0.000248	3.6942	9830000	0	0	9830000	0	0	0	6605700	0
O76075	O76075	DNA fragmentation factor subunit beta	DFFB	sp|O76075	1	1	1	0	1	0	0	0	1	0	0	0	1	0	0	4.1	39.109	0.003488	1.9882	9823600	0	9823600	0	0	0	865940	0	0
Q9BQL6	Q9BQL6	Fermitin family homolog 1	FERMT1	sp|Q9BQL6	3	2	2	0	1	0	3	0	0	0	2	0	0	0	2	4.3	77.436	0.001132	2.4636	9762800	0	0	0	9762800	0	0	0	401500
O43164	O43164	E3 ubiquitin-protein ligase Praja-2	PJA2	sp|O43164	1	1	1	0	1	0	1	0	1	0	1	0	1	0	1	2.4	78.213	0.000718	3.3007	9751800	0	1658800	0	8093000	0	146230	0	332830
Q6NT16	Q6NT16	MFS-type transporter SLC18B1	SLC18B1	sp|Q6NT16	1	1	1	0	1	0	0	0	1	0	0	0	1	0	0	2.4	48.868	0.001784	2.257	9739400	0	9739400	0	0	0	858520	0	0
Q05655	Q05655	Protein kinase C delta type;Protein kinase C delta	PRKCD	sp|Q05655	1	1	1	0	1	0	0	0	1	0	0	0	1	0	0	1.8	77.504	0.000246	3.614	9730400	0	9730400	0	0	0	857730	0	0
Q9NPH0	Q9NPH0	Lysophosphatidic acid phosphatase type 6	ACP6	sp|Q9NPH	1	1	1	0	1	0	0	0	1	0	0	0	1	0	0	4.9	48.886	0.001133	2.4795	9705600	0	9705600	0	0	0	855540	0	0
Q07820	Q07820	Induced myeloid leukemia cell differentiation pro	MCL1	sp|Q07820	1	1	1	0	0	0	1	0	0	0	1	0	0	0	1	2.3	37.337	0.0058	1.7797	9702400	0	0	0	9702400	0	0	0	399020
Q8WV07	Q8WV07	Oral cancer-overexpressed protein 1	ORAOV1	sp|Q8WV0	1	1	1	0	0	1	1	0	0	1	1	0	0	1	1	11.7	15.354	0	5.627	9657400	0	0	994000	8663400	0	0	667960	356290
Q9H7F4	Q9H7F4	Transmembrane protein 185B	TMEM185	sp|Q9H7F4	1	1	1	0	0	0	1	0	0	0	1	0	0	0	1	7.7	40.599	0.003486	1.9824	9584200	0	0	0	9584200	0	0	0	394160
P05423	P05423	DNA-directed RNA polymerase III subunit RPC4	POLR3D	sp|P05423	1	1	1	0	1	0	1	0	1	0	1	0	1	0	1	2.3	44.395	0.002877	2.1641	9584200	0	6965900	0	2618300	0	614040	0	107680
Q9C0B7	Q9C0B7	Transport and Golgi organization protein 6 homol	TANGO6	sp|Q9C0B7	1	1	1	0	0	0	1	0	0	0	1	0	0	0	1	1.6	120.75	0.001156	2.7609	9519100	0	0	0	9519100	0	0	0	391480
Q96EX1	Q96EX1	Small integral membrane protein 12	SMIM12	sp|Q96EX1	1	1	1	0	1	0	1	0	1	0	1	0	1	0	1	9.8	10.799	0.002874	2.153	9465200	0	3257900	0	6207300	0	287180	0	255280
P36897	P36897	TGF-beta receptor type-1	TGFBR1	sp|P36897	1	1	1	0	1	0	1	0	1	0	1	0	1	0	1	3	55.959	0	4.6119	9434500	0	1792300	0	7642300	0	157990	0	314290
Q8IX05	Q8IX05	CD302 antigen	CD302	sp|Q8IX05	1	1	1	0	0	0	1	0	0	0	1	0	0	0	1	8.2	26.182	0.001161	2.8371	9344800	0	0	0	9344800	0	0	0	384310
Q8TCG2	Q8TCG2	Phosphatidylinositol 4-kinase type 2-beta	PI4K2B	sp|Q8TCG	1	1	1	0	0	0	1	0	0	0	1	0	0	0	1	4.8	54.744	0.003284	2.0582	9325600	0	0	0	9325600	0	0	0	383520
Q12955	Q12955	Ankyrin-3	ANK3	sp|Q12955	3	3	3	0	3	0	0	0	3	0	0	0	3	0	0	0.8	480.4	0.001167	2.9093	9325100	0	9325100	0	0	0	822000	0	0
Q8NCM8	Q8NCM8	Cytoplasmic dynein 2 heavy chain 1	DYNC2H1	sp|Q8NCM	1	1	1	0	1	0	0	0	1	0	0	0	1	0	0	0.2	492.62	0.003076	2.0929	9322700	0	9322700	0	0	0	821780	0	0
P29590	P29590	Protein PML	PML	sp|P29590	1	1	1	0	1	0	1	0	1	0	1	0	1	0	1	1.8	97.55	0.001147	2.6299	9295100	0	2434900	0	6860200	0	214630	0	282130
Q5VU43	Q5VU43	Myomegalin	PDE4DIP	sp|Q5VU4	1	1	1	0	0	0	1	0	0	0	1	0	0	0	1	0.6	265.1	0.001156	2.7587	9244200	0	0	0	9244200	0	0	0	380180
Q9P2W1	Q9P2W1	Homologous-pairing protein 2 homolog	PSMC3IP	sp|Q9P2W	3	3	3	0	3	0	0	0	3	0	0	0	3	0	0	22.1	24.906	0.000488	3.5217	9239600	0	9239600	0	0	0	814460	0	0
Q9NUJ3	Q9NUJ3	T-complex protein 11-like protein 1	TCP11L1	sp|Q9NUJ	1	1	1	0	0	0	1	0	0	0	1	0	0	0	1	2	57.034	0.002871	2.1405	9174200	0	0	0	9174200	0	0	0	377300
Q14807	Q14807	Kinesin-like protein KIF22	KIF22	sp|Q14807	2	2	2	0	2	0	0	0	2	0	0	0	2	0	0	4.1	73.261	0.001127	2.416	8979900	0	8979900	0	0	0	791570	0	0
O15379	O15379	Histone deacetylase 3	HDAC3	sp|O15379	2	1	1	1	2	0	1	0	1	0	1	0	1	0	1	5.4	48.847	0.000485	3.4453	8965200	0	2852200	0	6113000	0	251420	0	251400
Q9BSL1	Q9BSL1	Ubiquitin-associated domain-containing protein 1	UBAC1	sp|Q9BSL1	1	1	1	0	1	0	0	0	1	0	0	0	1	0	0	4.7	45.338	0.000943	3.0708	8929400	0	8929400	0	0	0	787120	0	0
P25445	P25445	Tumor necrosis factor receptor superfamily mem	FAS	sp|P25445	1	1	1	0	0	0	1	0	0	0	1	0	0	0	1	3.6	37.732	0.000483	3.3916	8892800	0	0	0	8892800	0	0	0	365720
Q86VN1	Q86VN1	Vacuolar protein-sorting-associated protein 36	VPS36	sp|Q86VN	2	2	2	0	2	0	1	0	2	0	1	0	2	0	1	7	43.816	0	4.9764	8865600	0	8865600	0	0	0	781490	0	0
Q14692	Q14692	Ribosome biogenesis protein BMS1 homolog	BMS1	sp|Q14692	2	2	2	0	1	1	1	0	1	1	1	0	1	1	1	2	145.81	0.003902	1.9196	8779100	0	5666100	3113000	0	0	0	2092000	0
A3KN83;Q	A3KN83;Q	Protein strawberry notch homolog 1;Protein stra	SBNO1;SBI	sp|A3KN8	2	2	2	0	2	0	1	0	2	0	1	0	2	0	1	1.9	154.31	0	4.1348	8729900	0	5659300	0	3070600	0	424240	0	200900
Q96BY7;Q	Q96BY7;Q	Autophagy-related protein 2 homolog B;Autopha	ATG2B;AT	sp|Q96BY	2	2	2	0	0	0	2	0	0	0	2	0	0	0	2	0.9	232.76	0.003487	1.9832	8688800	0	0	0	8688800	0	0	0	357330
Q5R372	Q5R372	Rab GTPase-activating protein 1-like	RABGAP1L	sp|Q5R372	1	1	1	0	1	0	1	0	1	0	1	0	1	0	1	1.5	92.512	0.001166	2.8994	8631000	0	2181600	0	6449400	0	192310	0	265240
Q8TDI0	Q8TDI0	Chromodomain-helicase-DNA-binding protein 5	CHD5	sp|Q8TDI0	10	1	1	0	10	5	8	0	1	0	0	0	0	0	0	5.9	223.05	0.00683	1.714	8568900	0	8568900	0	0	0	755340	0	0
Q9P1Z2	Q9P1Z2	Calcium-binding and coiled-coil domain-containin	CALCOCO1	sp|Q9P1Z2	2	2	2	0	1	0	1	0	1	0	1	0	1	0	1	3.2	77.335	0	5.3919	8506800	0	0	0	8506800	0	0	0	349850
O95864	O95864	Fatty acid desaturase 2	FADS2	sp|O95864	2	2	2	0	1	1	2	0	1	1	2	0	1	1	2	4.1	52.259	0	4.5973	8492000	0	2401400	343550	5747000	0	215580	225140	238180
Q5VZL5;Q	Q5VZL5	Zinc finger MYM-type protein 4	ZMYM4	sp|Q5VZL5	3	3	3	0	3	0	0	0	3	0	0	0	3	0	0	2.1	172.79	0.001563	2.2781	8455700	0	8455700	0	0	0	745360	0	0
Q99707	Q99707	Methionine synthase	MTR	sp|Q99707	1	1	1	0	0	0	1	0	0	0	1	0	0	0	1	0.6	140.53	0.008464	1.6092	8435800	0	0	0	8435800	0	0	0	346930
Q8N5I4	Q8N5I4	Dehydrogenase/reductase SDR family member or	DHRSX	sp|Q8N5I4	1	1	1	0	0	0	1	0	0	0	1	0	0	0	1	5.2	36.443	0.000246	3.6369	8371600	0	0	0	8371600	0	0	0	344290
Q9H967	Q9H967	WD repeat-containing protein 76	WDR76	sp|Q9H967	1	1	1	0	0	1	1	0	0	1	1	0	0	1	1	2.9	69.768	0.000722	3.3518	8334700	0	0	1142500	7192200	0	0	767780	295780

Q9H3F6	Q9H3F6	BTB/POZ domain-containing adapter for CUL3-me	KCTD10	sp|Q9H3F6	1	1	1	0	0	0	1	0	0	0	1	0	0	0	1	4.2	35.432	0.002662	2.1873	8318700	0	0	0	8318700	0	0	0	342110
Q9BXW6	Q9BXW6	Oxysterol-binding protein-related protein 1	OSBPL1A	sp|Q9BXW	1	1	1	0	1	0	1	0	1	0	1	0	1	0	1	0.9	108.47	0.009652	1.5281	8284300	0	4029300	0	4255000	0	355180	0	174990
Q6KC79	Q6KC79	Nipped-B-like protein	NIPBL	sp|Q6KC79	3	3	3	0	3	0	0	0	3	0	0	0	3	0	0	1.5	316.05	0	4.9478	8229200	0	8229200	0	0	0	725400	0	0
Q15007	Q15007	Pre-mRNA-splicing regulator WTAP	WTAP	sp|Q15007	1	1	1	1	1	0	0	1	1	0	0	1	1	0	0	3.3	44.243	0.000248	3.6916	8178800	1465400	6713500	0	0	1465400	591790	0	0
P50548	P50548	ETS domain-containing transcription factor ERF	ERF	sp|P50548	1	1	1	0	0	0	1	0	0	0	1	0	0	0	1	2.6	58.702	0.000484	3.4209	8141000	0	0	0	8141000	0	0	0	334800
Q9H6B4	Q9H6B4	CXADR-like membrane protein	CLMP	sp|Q9H6B4	1	1	1	0	0	0	1	0	0	0	1	0	0	0	1	3.5	41.28	0.001158	2.7873	8044000	0	0	0	8044000	0	0	0	330820
Q9NVM6	Q9NVM6	DnaJ homolog subfamily C member 17	DNAJC17	sp|Q9NVM	1	1	1	0	0	0	1	0	0	0	1	0	0	0	1	5.9	34.687	0	5.3153	8029700	0	0	0	8029700	0	0	0	330230
Q9ULE4	Q9ULE4	Protein FAM184B	FAM184B	sp|Q9ULE4	1	1	1	1	0	0	0	1	0	0	0	1	0	0	0	0.9	121.04	0.008632	1.5669	7973100	7973100	0	0	0	7973100	0	0	0
Q16610	Q16610	Extracellular matrix protein 1	ECM1	sp|Q16610	2	2	2	0	0	2	0	0	0	2	0	0	0	2	0	5	60.673	0	3.8354	7853100	0	0	7853100	0	0	0	5277200	0
Q9H4B0	Q9H4B0	Probable tRNA N6-adenosine threonylcarbamoylt	OSGEPL1	sp|Q9H4B0	1	1	1	0	0	0	1	0	0	0	1	0	0	0	1	3.1	45.122	0.009252	1.5476	7815400	0	0	0	7815400	0	0	0	321410
Q00534	Q00534	Cyclin-dependent kinase 6	CDK6	sp|Q00534	2	1	1	0	2	1	1	0	1	0	0	0	1	0	0	6.7	36.938	0	3.9956	7705500	0	7705500	0	0	0	679230	0	0
Q96JI7	Q96JI7	Spatacsin	SPG11	sp|Q96JI7|	1	1	1	0	0	0	1	0	0	0	1	0	0	0	1	0.6	278.86	0.001349	2.3853	7623500	0	0	0	7623500	0	0	0	313520
Q5TF21	Q5TF21	Protein SOGA3	SOGA3	sp|Q5TF21	2	1	1	0	0	1	2	0	0	0	1	0	0	0	1	2.3	103.2	0.00095	3.1881	7622200	0	0	0	7622200	0	0	0	313470
Q6DKK2	Q6DKK2	Tetratricopeptide repeat protein 19, mitochondri	TTC19	sp|Q6DKK2	1	1	1	0	1	0	0	0	1	0	0	0	1	0	0	3.2	42.456	0.000483	3.4008	7572500	0	7572500	0	0	0	667510	0	0
P28749	P28749	Retinoblastoma-like protein 1	RBL1	sp|P28749	1	1	1	0	0	0	1	0	0	0	1	0	0	0	1	1.5	120.85	0	5.1441	7550700	0	0	0	7550700	0	0	0	310530
Q9BQE4	Q9BQE4	Selenoprotein S	VIMP	sp|Q9BQE4	1	1	1	0	0	0	1	0	0	0	1	0	0	0	1	5.8	21.163	0.006419	1.7326	7538600	0	0	0	7538600	0	0	0	310030
Q5TGY1	Q5TGY1	Transmembrane and coiled-coil domain-containir	TMCO4	sp|Q5TGY1	1	1	1	0	0	0	1	0	0	0	1	0	0	0	1	4.4	67.909	0.003911	1.9485	7511000	0	0	0	7511000	0	0	0	308890
P22735	P22735	Protein-glutamine gamma-glutamyltransferase K	TGM1	sp|P22735	3	3	3	0	0	3	0	0	0	3	0	0	0	3	0	3.5	89.786	0	3.8901	7457200	0	0	7457200	0	0	0	5011200	0
P36222	P36222	Chitinase-3-like protein 1	CHI3L1	sp|P36222	1	1	1	0	0	0	1	0	0	0	1	0	0	0	1	6	42.625	0.000487	3.4908	7426900	0	0	0	7426900	0	0	0	305440
P27482	P27482	Calmodulin-like protein 3	CALML3	sp|P27482	2	2	2	0	0	2	0	0	0	2	0	0	0	2	0	15.4	16.891	0.00157	2.341	7379000	0	0	7379000	0	0	0	4958700	0
P07476	P07476	Involucrin	IVL	sp|P07476	2	2	2	1	0	2	0	1	0	2	0	1	0	2	0	5.1	68.478	0	5.4117	7377100	1250500	0	6126600	0	2175800	0	3191800	0
Q14164	Q14164	Inhibitor of nuclear factor kappa-B kinase subunit	IKBKE	sp|Q14164	3	3	3	0	1	3	0	0	1	3	0	0	1	3	0	4.2	80.461	0	5.598	7366100	0	1610700	5755400	0	0	1132100	2877500	0
Q8NHS3	Q8NHS3	Major facilitator superfamily domain-containing p	MFSD8	sp|Q8NHS	1	1	1	0	0	0	1	0	0	0	1	0	0	0	1	3.1	57.627	0.001567	2.3124	7365500	0	0	0	7365500	0	0	0	302910
Q8TF71	Q8TF71	Monocarboxylate transporter 10	SLC16A10	sp|Q8TF71	1	1	1	0	1	0	0	0	1	0	0	0	1	0	0	3.3	55.492	0.00072	3.3276	7359600	0	7359600	0	0	0	648740	0	0
Q9BPX7	Q9BPX7	UPF0415 protein C7orf25	C7orf25	sp|Q9BPX7	1	1	1	1	0	1	1	1	0	1	1	1	0	1	1	2.6	46.45	0.004332	1.9002	7305400	1640500	0	1290800	4374200	1476000	0	1073300	138480
Q9NQY0	Q9NQY0	Bridging integrator 3	BIN3	sp|Q9NQY	1	1	1	0	0	0	1	0	0	0	1	0	0	0	1	3.2	29.665	0.008056	1.6392	7265300	0	0	0	7265300	0	0	0	298790
P04196	P04196	Histidine-rich glycoprotein	HRG	sp|P04196	1	1	1	0	0	1	0	0	0	1	0	0	0	1	0	1.7	59.578	0.001147	2.6234	7261200	0	0	7261200	0	0	0	4879500	0
P51451	P51451	Tyrosine-protein kinase Blk	BLK	sp|P51451	1	1	1	0	0	0	1	0	0	0	1	0	0	0	1	2.6	57.706	0.000717	3.2725	7222900	0	0	0	7222900	0	0	0	297050
O15155	O15155	BET1 homolog	BET1	sp|O15155	1	1	1	0	1	0	0	0	1	0	0	0	1	0	0	9.3	13.289	0.000945	3.0888	7210800	0	7210800	0	0	0	635620	0	0
Q8IU60	Q8IU60	m7GpppN-mRNA hydrolase	DCP2	sp|Q8IU60	1	1	1	0	1	0	0	0	1	0	0	0	1	0	0	2.1	48.423	0.009654	1.529	7192000	0	7192000	0	0	0	633970	0	0
O15156	O15156	Zinc finger and BTB domain-containing protein 7E	ZBTB7B	sp|O15156	1	1	1	0	0	1	0	0	0	1	0	0	0	1	0	4.8	58.026	0.001347	2.3666	7168900	0	0	7168900	0	0	0	4817500	0
Q9Y2K2	Q9Y2K2	Serine/threonine-protein kinase SIK3	SIK3	sp|Q9Y2K2	1	1	1	0	0	0	1	0	0	0	1	0	0	0	1	1.8	144.85	0	5.145	7165300	0	0	0	7165300	0	0	0	294680
Q92503	Q92503	SEC14-like protein 1	SEC14L1	sp|Q92503	1	1	1	0	0	0	1	0	0	0	1	0	0	0	1	2.1	81.249	0.000246	3.647	7040900	0	0	0	7040900	0	0	0	289560
O15381	O15381	Nuclear valosin-containing protein-like	NVL	sp|O15381	1	1	1	0	1	0	0	0	1	0	0	0	1	0	0	1.8	95.05	0.00391	1.9453	6958400	0	6958400	0	0	0	613380	0	0
Q12899	Q12899	Tripartite motif-containing protein 26	TRIM26	sp|Q12899	1	1	1	0	0	1	1	0	0	1	1	0	0	1	1	2	62.165	0.00641	1.7244	6914000	0	0	1318300	5595700	0	0	885870	230130
Q92968	Q92968	Peroxisomal membrane protein PEX13	PEX13	sp|Q92968	1	1	1	0	1	0	1	0	1	0	1	0	1	0	1	2.5	44.129	0.001155	2.7422	6899800	0	1185300	0	5714400	0	104490	0	235010
O14682	O14682	Ectoderm-neural cortex protein 1	ENC1	sp|O14682	1	1	1	0	0	0	1	0	0	0	1	0	0	0	1	2	66.129	0.001173	3.0089	6847900	0	0	0	6847900	0	0	0	281630
Q9NSV4	Q9NSV4	Protein diaphanous homolog 3	DIAPH3	sp|Q9NSV4	1	1	1	0	1	0	0	0	1	0	0	0	1	0	0	1.9	136.92	0.004733	1.807	6839200	0	6839200	0	0	0	602870	0	0
P54098	P54098	DNA polymerase subunit gamma-1	POLG	sp|P54098	1	1	1	0	1	0	0	0	1	0	0	0	1	0	0	1.1	139.56	0.003289	2.0754	6821800	0	6821800	0	0	0	601330	0	0
Q14676	Q14676	Mediator of DNA damage checkpoint protein 1	MDC1	sp|Q14676	1	1	1	0	0	0	1	0	0	0	1	0	0	0	1	1.2	226.66	0.003487	1.9834	6812200	0	0	0	6812200	0	0	0	280150
P43235	P43235	Cathepsin K	CTSK	sp|P43235	1	1	1	0	0	1	1	0	0	1	1	0	0	1	1	2.1	36.966	0.006416	1.7298	6780700	0	0	1357300	5423400	0	0	912110	223040
O75554	O75554	WW domain-binding protein 4	WBP4	sp|O75554	1	1	1	0	1	0	0	0	1	0	0	0	1	0	0	2.9	42.506	0.002872	2.1415	6762000	0	6762000	0	0	0	596070	0	0
P01034	P01034	Cystatin-C	CST3	sp|P01034	1	1	1	0	1	0	0	0	1	0	0	0	1	0	0	7.5	15.799	0.001128	2.4162	6760400	0	6760400	0	0	0	595920	0	0
Q02040	Q02040	A-kinase anchor protein 17A	AKAP17A	sp|Q02040	1	1	1	0	1	1	0	0	1	1	0	0	1	1	0	1.2	80.735	0.006214	1.7532	6725900	0	5528500	1197400	0	0	487330	804660	0
Q5VSL9	Q5VSL9	Striatin-interacting protein 1	STRIP1	sp|Q5VSL9	1	1	1	0	0	0	1	0	0	0	1	0	0	0	1	2.7	95.575	0.000949	3.1471	6698500	0	0	0	6698500	0	0	0	275480
P35754	P35754	Glutaredoxin-1	GLRX	sp|P35754	2	2	2	0	0	2	0	0	0	2	0	0	0	2	0	37.7	11.776	0.00025	3.7999	6616300	0	0	6616300	0	0	0	4446200	0
O60518	O60518	Ran-binding protein 6	RANBP6	sp|O60518	3	1	1	0	2	3	3	0	0	1	1	0	0	1	1	2.9	124.71	0.003287	2.0716	6614600	0	0	256540	6358000	0	0	172390	261480
Q96FZ2	Q96FZ2	Embryonic stem cell-specific 5-hydroxymethylcyto	HMCES	sp|Q96FZ2	1	1	1	0	1	1	0	0	1	1	0	0	1	1	0	3.4	40.574	0.000718	3.3029	6589700	0	6399400	190350	0	0	564100	127910	0
Q9UKG9	Q9UKG9	Peroxisomal carnitine O-octanoyltransferase	CROT	sp|Q9UKG	1	1	1	0	1	0	1	0	1	0	1	0	1	0	1	2.5	70.178	0.001347	2.3657	6527300	0	1326000	0	5201300	0	116890	0	213910
Q8TEU7	Q8TEU7	Rap guanine nucleotide exchange factor 6	RAPGEF6	sp|Q8TEU	1	1	1	0	1	0	0	0	1	0	0	0	1	0	0	1.2	179.42	0.00025	3.7945	6523100	0	6523100	0	0	0	575010	0	0
Q9Y2G8	Q9Y2G8	DnaJ homolog subfamily C member 16	DNAJC16	sp|Q9Y2G8	1	1	1	0	0	0	1	0	0	0	1	0	0	0	1	1.7	90.59	0.003908	1.938	6482200	0	0	0	6482200	0	0	0	266580
Q9P2G1	Q9P2G1	Ankyrin repeat and IBR domain-containing protei	ANKIB1	sp|Q9P2G1	1	1	1	0	0	0	1	0	0	0	1	0	0	0	1	1.8	122	0	4.7091	6400700	0	0	0	6400700	0	0	0	263230
Q6JQN1	Q6JQN1	Acyl-CoA dehydrogenase family member 10	ACAD10	sp|Q6JQN1	1	1	1	0	0	0	1	0	0	0	1	0	0	0	1	1.5	118.83	0.008467	1.6111	6353700	0	0	0	6353700	0	0	0	261300
Q8N7R7	Q8N7R7	Cyclin-Y-like protein 1	CCNYL1	sp|Q8N7R7	1	1	1	0	0	0	1	0	0	0	1	0	0	0	1	2.2	40.705	0.00601	1.7689	6331900	0	0	0	6331900	0	0	0	260410
Q6P1R4	Q6P1R4	tRNA-dihydrouridine(16/17) synthase [NAD(P)(+)]	DUS1L	sp|Q6P1R4	1	1	1	0	1	0	0	0	1	0	0	0	1	0	0	3	53.23	0.000947	3.1293	6276100	0	6276100	0	0	0	553230	0	0
Q8TBK6	Q8TBK6	Zinc finger CCHC domain-containing protein 10	ZCCHC10	sp|Q8TBK6	1	1	1	0	1	0	0	0	1	0	0	0	1	0	0	7.3	20.966	0.000247	3.6597	6171300	0	6171300	0	0	0	543990	0	0
Q96DF8	Q96DF8	Protein DGCR14	DGCR14	sp|Q96DF8	1	1	1	0	1	0	0	0	1	0	0	0	1	0	0	1.9	52.567	0.004735	1.8088	6106600	0	6106600	0	0	0	538290	0	0
O00635	O00635	E3 ubiquitin-protein ligase TRIM38	TRIM38	sp|O00635	1	1	1	0	0	0	1	0	0	0	1	0	0	0	1	2.4	53.415	0.000483	3.3964	6055200	0	0	0	6055200	0	0	0	249030
Q01432	Q01432	AMP deaminase 3	AMPD3	sp|Q01432	1	1	1	0	0	0	1	0	0	0	1	0	0	0	1	0.9	88.811	0.008635	1.5687	6048400	0	0	0	6048400	0	0	0	248740
Q9Y3A4	Q9Y3A4	Ribosomal RNA-processing protein 7 homolog A	RRP7A	sp|Q9Y3A4	3	3	3	0	0	2	2	0	0	2	2	0	0	2	2	15	32.334	0.000248	3.706	6037400	0	0	2176800	3860600	0	0	1462800	0
Q9UJX6	Q9UJX6	Anaphase-promoting complex subunit 2	ANAPC2	sp|Q9UJX6	1	1	1	0	0	0	1	0	0	0	1	0	0	0	1	1.1	93.827	0.007851	1.6502	6012800	0	0	0	6012800	0	0	0	247280
O76024	O76024	Wolframin	WFS1	sp|O76024	2	2	2	0	1	0	1	0	1	0	1	0	1	0	1	2.9	100.29	0.009841	1.5074	6004300	0	3531500	0	2472900	0	0	0	101700
P78332	P78332	RNA-binding protein 6	RBM6	sp|P78332	3	2	2	0	0	2	1	0	0	2	0	0	0	2	0	2.9	128.64	0.006425	1.7423	5970400	0	0	5970400	0	0	0	4012100	0
P18440	P18440	Arylamine N-acetyltransferase 1	NAT1	sp|P18440	1	1	1	0	0	0	1	0	0	0	1	0	0	0	1	3.1	33.898	0.001143	2.5897	5956100	0	0	0	5956100	0	0	0	244950
Q14687	Q14687	Genetic suppressor element 1	GSE1	sp|Q14687	1	1	1	0	1	0	0	0	1	0	0	0	1	0	0	1.2	136.16	0.000951	3.1898	5911700	0	5911700	0	0	0	521110	0	0
P61244	P61244	Protein max	MAX	sp|P61244	1	1	1	0	1	0	1	0	1	0	1	0	1	0	1	8.1	18.275	0.008051	1.626	5888100	0	2323600	0	3564600	0	204820	0	146600
Q56A73	Q56A73	Spindlin-4	SPIN4	sp|Q56A73	1	1	1	0	0	1	0	0	0	1	0	0	0	1	0	3.2	28.66	0.004731	1.8007	5861800	0	0	5861800	0	0	0	3939100	0
Q8N302	Q8N302	Angiogenic factor with G patch and FHA domains	AGGF1	sp|Q8N302	2	2	2	1	0	2	0	1	0	2	0	1	0	2	0	2.7	80.976	0	3.9258	5847200	325220	0	5522000	0	564470	0	3471500	0
Q86WQ0	Q86WQ0	Nuclear receptor 2C2-associated protein	NR2C2AP	sp|Q86WC	1	1	1	0	0	0	1	0	0	0	1	0	0	0	1	8.6	15.876	0	3.9755	5802300	0	0	0	5802300	0	0	0	238620
Q9NYL4	Q9NYL4	Peptidyl-prolyl cis-trans isomerase FKBP11	FKBP11	sp|Q9NYL4	1	1	1	0	0	0	1	0	0	0	1	0	0	0	1	5	22.18	0.001136	2.5015	5781400	0	0	0	5781400	0	0	0	237760
Q9H871	Q9H871	Protein RMD5 homolog A	RMND5A	sp|Q9H871	1	1	1	0	1	0	0	0	1	0	0	0	1	0	0	3.1	43.992	0.00745	1.6881	5764000	0	5764000	0	0	0	508090	0	0
O95319	O95319	CUGBP Elav-like family member 2	CELF2	sp|O95319	1	1	1	0	1	0	0	0	1	0	0	0	1	0	0	2.8	54.284	0.007453	1.6907	5745900	0	5745900	0	0	0	506490	0	0
O60507	O60507	Protein-tyrosine sulfotransferase 1	TPST1	sp|O60507	1	1	1	0	0	0	1	0	0	0	1	0	0	0	1	4.9	42.188	0.000245	3.5986	5640500	0	0	0	5640500	0	0	0	231970
P04259;CC	P04259	Keratin, type II cytoskeletal 6B	KRT6B	sp|P04259	33	1	1	5	21	32	14	0	0	1	0	0	0	1	0	45.7	60.066	0.009644	1.5261	5606400	0	0	5606400	0	0	0	3767500	0
Q9UPN4	Q9UPN4	Centrosomal protein of 131 kDa	CEP131	sp|Q9UPN	2	2	2	0	0	0	2	0	0	0	2	0	0	0	2	2.1	122.15	0.002664	2.1915	5543200	0	0	0	5543200	0	0	0	227970
Q9BT09	Q9BT09	Protein canopy homolog 3	CNPY3	sp|Q9BT09	1	1	1	0	1	0	0	0	1	0	0	0	1	0	0	4.7	30.748	0.00095	3.1839	5534600	0	5534600	0	0	0	487870	0	0
Q9H467	Q9H467	CUE domain-containing protein 2	CUEDC2	sp|Q9H467	1	1	1	0	1	0	0	0	1	0	0	0	1	0	0	5.6	32.009	0.00642	1.7341	5506800	0	5506800	0	0	0	485420	0	0
Q86X29	Q86X29	Lipolysis-stimulated lipoprotein receptor	LSR	sp|Q86X29	1	1	1	0	1	0	0	0	1	0	0	0	1	0	0	1.7	71.438	0.001783	2.257	5442200	0	5442200	0	0	0	479720	0	0
Q13835	Q13835	Plakophilin-1	PKP1	sp|Q13835	2	2	2	0	1	2	0	0	1	2	0	0	1	2	0	2	82.86	0.002225	2.2323	5416600	0	1094500	4322100	0	0	393730	2607200	0
Q9H1J1	Q9H1J1	Regulator of nonsense transcripts 3A	UPF3A	sp|Q9H1J1	2	2	2	0	0	2	0	0	0	2	0	0	0	2	0	4.4	54.696	0.002656	2.1717	5294600	0	0	5294600	0	0	0	3558000	0
Q9HAU0	Q9HAU0	Pleckstrin homology domain-containing family A r	PLEKHA5	sp|Q9HAU	2	2	2	0	0	1	1	0	0	1	1	0	0	1	1	2.8	127.46	0.000947	3.1154	5200800	0	0	2501400	2699400	0	0	1680900	0
Q30154	Q30154	HLA class II histocompatibility antigen, DR beta 5 c	HLA-DRB5	sp|Q30154	3	1	1	0	0	1	3	0	0	0	1	0	0	0	1	19.5	30.056	0.002227	2.2415	5177700	0	0	0	5177700	0	0	0	212940
Q8TEV9	Q8TEV9	Smith-Magenis syndrome chromosomal region ca	SMCR8	sp|Q8TEV9	1	1	1	0	0	0	1	0	0	0	1	0	0	0	1	1.2	105.02	0.00114	2.5604	5156300	0	0	0	5156300	0	0	0	212060
P60880	P60880	Synaptosomal-associated protein 25	SNAP25	sp|P60880	1	1	1	0	0	0	1	0	0	0	1	0	0	0	1	7.3	23.315	0.00115	2.676	5100000	0	0	0	5100000	0	0	0	209740
O95619	O95619	YEATS domain-containing protein 4	YEATS4	sp|O95619	1	1	1	0	1	0	1	0	1	0	1	0	1	0	1	4.8	26.499	0.003088	2.1209	5036600	0	2419700	0	2616800	0	213300	0	107620
Q96D09	Q96D09	G-protein coupled receptor-associated sorting pr	GPRASP2	sp|Q96D09	1	1	1	0	1	0	0	0	1	0	0	0	1	0	0	2.1	93.772	0	3.9971	5029400	0	5029400	0	0	0	443330	0	0
Q96PZ2	Q96PZ2	Protein FAM111A	FAM111A	sp|Q96PZ2	2	2	2	0	0	1	1	0	0	1	1	0	0	1	1	4.4	70.195	0.000715	3.2629	5016200	0	0	5016200	0	0	0	3370900	0
P37198	P37198	Nuclear pore glycoprotein p62	NUP62	sp|P37198	1	1	1	0	0	0	1	0	0	0	1	0	0	0	1	1.9	53.254	0.000249	3.7808	5001600	0	0	0	5001600	0	0	0	205690
Q12933	Q12933	TNF receptor-associated factor 2	TRAF2	sp|Q12933	1	1	1	0	1	0	0	0	1	0	0	0	1	0	0	4	55.859	0.000719	3.3135	4920400	0	4920400	0	0	0	433730	0	0

O43318	O43318	Mitogen-activated protein kinase kinase kinase 7	MAP3K7	sp|O43318	2	2	2	0	1	0	1	0	1	0	1	0	1	0	1	6.1	67.195	0	6.2976	4883000	0	4883000	0	0	0	430430	0	0
O14681	O14681	Etoposide-induced protein 2.4 homolog	EI24	sp|O14681	2	2	2	0	2	0	1	0	2	0	1	0	2	0	1	4.4	38.964	0.00116	2.8167	4831500	0	2235400	0	2596100	0	197050	0	106760
Q96GM5	Q96GM5	SWI/SNF-related matrix-associated actin-depende	SMARCD1	sp|Q96GM	1	1	1	0	0	0	1	0	0	0	1	0	0	0	1	2.5	58.232	0.004739	1.8124	4781000	0	0	0	4781000	0	0	0	196620
Q6NXT6	Q6NXT6	Transmembrane anterior posterior transformatio	TAPT1	sp|Q6NXT	1	1	1	0	0	0	1	0	0	0	1	0	0	0	1	3.9	64.259	0.000948	3.1371	4618200	0	0	0	4618200	0	0	0	189920
P17482	P17482	Homeobox protein Hox-B9	HOXB9	sp|P17482	1	1	1	0	1	0	0	0	1	0	0	0	1	0	0	5.6	28.058	0.001127	2.4092	4614200	0	4614200	0	0	0	406740	0	0
Q9NPJ6	Q9NPJ6	Mediator of RNA polymerase II transcription subu	MED4	sp|Q9NPJ6	1	1	1	0	1	0	0	0	1	0	0	0	1	0	0	3.3	29.745	0.001149	2.668	4599200	0	4599200	0	0	0	405420	0	0
Q96NX9;Q	Q96NX9;Q	Dachshund homolog 2;Dachshund homolog 1	DACH2;DA	sp|Q96NX	1	1	1	0	1	0	0	0	1	0	0	0	1	0	0	1.8	65.322	0.000946	3.1126	4563300	0	4563300	0	0	0	402250	0	0
Q9HCY8	Q9HCY8	Protein S100-A14	S100A14	sp|Q9HCY	1	1	1	0	0	1	0	0	0	1	0	0	0	1	0	10.6	11.662	0.003277	2.0351	4519000	0	0	4519000	0	0	0	3036800	0
P60006	P60006	Anaphase-promoting complex subunit 15	ANAPC15	sp|P60006	1	1	1	0	0	1	1	0	0	1	1	0	0	1	1	8.3	14.281	0.001132	2.4552	4507400	0	0	3538700	968670	0	0	2378000	39837
Q12983	Q12983	BCL2/adenovirus E1B 19 kDa protein-interacting p	BNIP3	sp|Q12983	1	1	1	0	0	0	1	0	0	0	1	0	0	0	1	4.2	27.832	0.002226	2.2359	4461200	0	0	0	4461200	0	0	0	183470
O75528	O75528	Transcriptional adapter 3	TADA3	sp|O75528	1	1	1	0	1	0	0	0	1	0	0	0	1	0	0	2.5	48.902	0.001568	2.3323	4410200	0	4410200	0	0	0	388760	0	0
P07948;P0	P07948;P0	Tyrosine-protein kinase Lyn;Tyrosine-protein kina	LYN;HCK	sp|P07948	2	1	1	0	2	1	1	0	1	0	0	0	1	0	0	3.5	58.573	0.008444	1.5949	4399100	0	4399100	0	0	0	387780	0	0
Q5BKX5	Q5BKX5	UPF0692 protein C19orf54	C19orf54	sp|Q5BKX	1	1	1	0	1	0	0	0	1	0	0	0	1	0	0	2.8	37.778	0.004534	1.8335	4202500	0	4202500	0	0	0	370440	0	0
Q96RU2	Q96RU2	Ubiquitin carboxyl-terminal hydrolase 28	USP28	sp|Q96RU	1	1	1	0	1	0	0	0	1	0	0	0	1	0	0	1.1	122.49	0.001152	2.702	4186400	0	4186400	0	0	0	369030	0	0
Q9BVS5	Q9BVS5	tRNA (adenine(58)-N(1))-methyltransferase, mito	TRMT61B	sp|Q9BVS	1	1	1	0	1	0	0	0	1	0	0	0	1	0	0	2.5	52.965	0.007852	1.6518	4156000	0	4156000	0	0	0	366350	0	0
Q96HJ9	Q96HJ9	UPF0562 protein C7orf55	C7orf55	sp|Q96HJ	1	1	1	0	1	0	0	0	1	0	0	0	1	0	0	7.1	12.749	0.009646	1.5261	4154300	0	4154300	0	0	0	366200	0	0
P01833	P01833	Polymeric immunoglobulin receptor;Secretory co	PIGR	sp|P01833	1	1	1	0	0	1	0	0	0	1	0	0	0	1	0	2.5	83.283	0.001146	2.6226	4145700	0	0	4145700	0	0	0	2785900	0
Q02338	Q02338	D-beta-hydroxybutyrate dehydrogenase, mitochc	BDH1	sp|Q02338	1	1	1	0	1	0	0	0	1	0	0	0	1	0	0	2	38.157	0.007452	1.69	4065600	0	4065600	0	0	0	358380	0	0
Q9BXS4	Q9BXS4	Transmembrane protein 59	TMEM59	sp|Q9BXS	1	1	1	1	0	0	1	1	0	0	1	1	0	0	1	4.6	36.223	0.007039	1.7067	4003000	215090	0	0	3787900	215090	0	0	155780
Q9BVJ7	Q9BVJ7	Dual specificity protein phosphatase 23	DUSP23	sp|Q9BVJ7	1	1	1	0	0	0	1	0	0	0	1	0	0	0	1	5.3	16.588	0.00349	2.0028	3959500	0	0	0	3959500	0	0	0	162840
Q2TAM9	Q2TAM9	Tumor suppressor candidate gene 1 protein	TUSC1	sp|Q2TAM	1	1	1	0	0	1	0	0	0	1	0	0	0	1	0	6.6	23.39	0.003087	2.1209	3889600	0	0	3889600	0	0	0	2613800	0
P52657	P52657	Transcription initiation factor IIA subunit 2	GTF2A2	sp|P52657	1	1	1	0	1	0	1	0	1	0	1	0	1	0	1	6.4	12.457	0.008471	1.6184	3660200	0	0	0	3660200	0	0	0	150530
Q8N4C8	Q8N4C8	Misshapen-like kinase 1	MINK1	sp|Q8N4C	5	1	1	1	2	2	5	0	0	0	1	0	0	0	1	4.1	149.82	0.008442	1.5944	3410000	0	0	0	3410000	0	0	0	140240
O60279	O60279	Sushi domain-containing protein 5	SUSD5	sp|O6027	1	1	1	0	1	0	0	0	1	0	0	0	1	0	0	1.9	68.02	0.007448	1.6874	3362100	0	3362100	0	0	0	296370	0	0
O60551	O60551	Glycylpeptide N-tetradecanoyltransferase 2	NMT2	sp|O60551	3	1	1	0	3	1	2	0	1	0	0	0	1	0	0	10.4	56.98	0.00884	1.5611	3204700	0	3204700	0	0	0	282490	0	0
Q9BPX6	Q9BPX6	Calcium uptake protein 1, mitochondrial	MICU1	sp|Q9BPX	1	1	1	0	0	1	0	0	0	1	0	0	0	1	0	3.2	54.351	0.003295	2.089	3170100	0	0	3170100	0	0	0	2130300	0
Q9UL40	Q9UL40	Zinc finger protein 346	ZNF346	sp|Q9UL4	1	1	1	0	1	0	0	0	1	0	0	0	1	0	0	4.4	32.932	0	3.9506	3083700	0	3083700	0	0	0	271820	0	0
P20226;Q6	P20226;Q6	TATA-box-binding protein;TATA box-binding prot	TBP;TBPL2	sp|P20226	1	1	1	0	1	0	0	0	1	0	0	0	1	0	0	2.9	37.698	0.009668	1.5428	2899700	0	2899700	0	0	0	255610	0	0
Q8N2M8	Q8N2M8	CLK4-associating serine/arginine rich protein	CLASRP	sp|Q8N2M	1	1	1	0	1	0	0	0	1	0	0	0	1	0	0	2.1	77.16	0.001141	2.5706	2817000	0	2817000	0	0	0	248310	0	0
Q96N21	Q96N21	AP-4 complex accessory subunit tepsin	ENTHD2	sp|Q96N2	1	1	1	0	0	0	1	0	0	0	1	0	0	0	1	3.2	55.136	0.001135	2.4934	2659200	0	0	0	2659200	0	0	0	109360
Q9UI43	Q9UI43	rRNA methyltransferase 2, mitochondrial	FTSJ2	sp|Q9UI43	1	1	1	0	1	1	0	0	1	1	0	0	1	1	0	3.3	27.423	0.009259	1.5527	2527900	0	2253600	274310	0	0	198650	184340	0
O43293	O43293	Death-associated protein kinase 3	DAPK3	sp|O4329	1	1	1	0	0	1	0	0	0	1	0	0	0	1	0	2.9	52.535	0.000249	3.7408	2467400	0	0	2467400	0	0	0	1658100	0
Q9UIG0	Q9UIG0	Tyrosine-protein kinase BAZ1B	BAZ1B	sp|Q9UIG	1	1	1	0	0	1	0	0	0	1	0	0	0	1	0	0.7	170.9	0.003489	1.9971	2436600	0	0	2436600	0	0	0	1637400	0
Q6ZVX7	Q6ZVX7	F-box only protein 50	NCCRP1	sp|Q6ZVX	1	1	1	0	0	1	0	0	0	1	0	0	0	1	0	4	30.847	0.003276	2.032	2349000	0	0	2349000	0	0	0	1578500	0
Q6IPR1	Q6IPR1	LYR motif-containing protein 5	LYRM5	sp|Q6IPR1	1	1	1	0	1	0	0	0	1	0	0	0	1	0	0	7.8	10.864	0.007434	1.6628	2024200	0	2024200	0	0	0	178430	0	0
Q9HA92	Q9HA92	Radical S-adenosyl methionine domain-containing	RSAD1	sp|Q9HA9	1	1	1	0	1	0	0	0	1	0	0	0	1	0	0	2.5	48.713	0.009257	1.5526	1913500	0	1913500	0	0	0	168680	0	0
Q9NZM5	Q9NZM5	Glioma tumor suppressor candidate region gene :	GLTSCR2	sp|Q9NZM	1	1	1	0	0	1	0	0	0	1	0	0	0	1	0	3.8	54.389	0.009849	1.5234	1746000	0	0	1746000	0	0	0	1173300	0
O43240	O43240	Kallikrein-10	KLK10	sp|O4324	2	2	2	0	0	2	0	0	0	2	0	0	0	2	0	8	30.17	0.003484	1.9711	1660800	0	0	1660800	0	0	0	1116100	0
Q8TDX5	Q8TDX5	2-amino-3-carboxymuconate-6-semialdehyde dec	ACMSD	sp|Q8TDX	1	1	1	0	1	0	1	0	1	0	1	0	1	0	1	2.7	38.035	0.009847	1.5233	1181800	0	395970	0	785800	0	0	0	32316

Majority protein IDs	Protein names	Gene names	Intensity	log(2)-fold change U87_ADCY8-IP/U87 lysate
Q9Y4L1	Hypoxia up-regulated protein 1	HYOU1	7.467E+09	-8.87881
P49588	Alanine--tRNA ligase, cytoplasmic	AARS	8.462E+09	-8.36187
O43747	AP-1 complex subunit gamma-1	AP1G1	1.28E+09	-8.3128
P53992	Protein transport protein Sec24C	SEC24C	1.932E+09	-8.21981
P13804	Electron transfer flavoprotein subunit alpha, mitoc	ETFA	4.986E+09	-8.06995
Q05193	Dynamin-1	DNM1	1.824E+09	-8.02666
P40121	Macrophage-capping protein	CAPG	3.009E+09	-7.66806
P68036	Ubiquitin-conjugating enzyme E2 L3	UBE2L3	7.738E+09	-7.66321
Q99798	Aconitate hydratase, mitochondrial	ACO2	6.428E+09	-7.611
P04181	Ornithine aminotransferase, mitochondrial;Ornithi	OAT	6.178E+09	-7.56763
P10620	Microsomal glutathione S-transferase 1	MGST1	982570000	-7.50635
P06132	Uroporphyrinogen decarboxylase	UROD	2.109E+09	-7.3472
P30566	Adenylosuccinate lyase	ADSL	3.312E+09	-7.30375
P00374	Dihydrofolate reductase	DHFR	1.187E+09	-7.26164
Q5GLZ8	Probable E3 ubiquitin-protein ligase HERC4	HERC4	975740000	-7.19624
P30043	Flavin reductase (NADPH)	BLVRB	2.424E+09	-7.10379
P52788	Spermine synthase	SMS	4.883E+09	-7.08105
P31939	Bifunctional purine biosynthesis protein PURH;Phc	ATIC	1.541E+10	-7.08031
P60983	Glia maturation factor beta	GMFB	1.394E+09	-7.03485
Q16401	26S proteasome non-ATPase regulatory subunit 5	PSMD5	1.463E+09	-7.0334
Q15836	Vesicle-associated membrane protein 3	VAMP3	673180000	-7.02115
P30520	Adenylosuccinate synthetase isozyme 2	ADSS	1.655E+09	-6.95994
P00966	Argininosuccinate synthase	ASS1	1.803E+09	-6.94823
P15559	NAD(P)H dehydrogenase [quinone] 1	NQO1	1.539E+09	-6.92101
Q9H2G2	STE20-like serine/threonine-protein kinase	SLK	1.897E+09	-6.90117
P49915	GMP synthase [glutamine-hydrolyzing]	GMPS	5.426E+09	-6.86586
Q9UJU6	Drebrin-like protein	DBNL	1.136E+09	-6.81375
A0AVT1	Ubiquitin-like modifier-activating enzyme 6	UBA6	2.688E+09	-6.81198
P28838	Cytosol aminopeptidase	LAP3	3.612E+09	-6.72426
Q15785	Mitochondrial import receptor subunit TOM34	TOMM34	1.807E+09	-6.71404
P11413	Glucose-6-phosphate 1-dehydrogenase	G6PD	5.248E+09	-6.6959
P43487	Ran-specific GTPase-activating protein	RANBP1	6.556E+09	-6.53688
P50502;Q8	Hsc70-interacting protein;Putative protein FAM10/	ST13;ST13P5;ST13	5.354E+09	-6.52716
P07741	Adenine phosphoribosyltransferase	APRT	2.333E+09	-6.51584
P17174	Aspartate aminotransferase, cytoplasmic	GOT1	5.906E+09	-6.50304
Q16881	Thioredoxin reductase 1, cytoplasmic	TXNRD1	4.306E+09	-6.48057

Q9Y6B6	GTP-binding protein SAR1b	SAR1B	699040000	-6.43399
P42765	3-ketoacyl-CoA thiolase, mitochondrial	ACAA2	1.365E+09	-6.37695
Q8IWE2	Protein NOXP20	FAM114A1	930990000	-6.32318
Q96BY6	Dedicator of cytokinesis protein 10	DOCK10	417390000	-6.3088
P06744	Glucose-6-phosphate isomerase	GPI	1.993E+10	-6.30266
Q12907	Vesicular integral-membrane protein VIP36	LMAN2	2.058E+09	-6.21797
P11766	Alcohol dehydrogenase class-3	ADH5	2.522E+09	-6.20967
Q15155	Nodal modulator 1	NOMO1	1.235E+09	-6.20602
P14314	Glucosidase 2 subunit beta	PRKCSH	5.008E+09	-6.17097
Q9ULC4;A0	Malignant T-cell-amplified sequence 1	MCTS1	1.775E+09	-6.13116
Q99536	Synaptic vesicle membrane protein VAT-1 homolog	VAT1	3.276E+09	-6.12824
Q12965	Unconventional myosin-Ie	MYO1E	1.146E+09	-6.10634
Q13617	Cullin-2	CUL2	1.243E+09	-6.08137
P31930	Cytochrome b-c1 complex subunit 1, mitochondria	UQCRC1	4.279E+09	-6.0769
O60493	Sorting nexin-3	SNX3	1.203E+09	-6.06579
Q96AE4	Far upstream element-binding protein 1	FUBP1	5.44E+09	-6.05275
Q7L576	Cytoplasmic FMR1-interacting protein 1	CYFIP1	2.39E+09	-6.05164
O60443	Non-syndromic hearing impairment protein 5	DFNA5	776300000	-5.99801
Q93045	Stathmin-2	STMN2	554940000	-5.97886
P21912	Succinate dehydrogenase [ubiquinone] iron-sulfur	SDHB	897290000	-5.97779
P49591	Serine--tRNA ligase, cytoplasmic	SARS	4.458E+09	-5.96919
Q9BS26	Endoplasmic reticulum resident protein 44	ERP44	1.227E+09	-5.96402
O00764	Pyridoxal kinase	PDXK	2.674E+09	-5.94925
P04818	Thymidylate synthase	TYMS	1.338E+09	-5.93748
P50395	Rab GDP dissociation inhibitor beta	GDI2	1.994E+10	-5.93288
P18669	Phosphoglycerate mutase 1	PGAM1	2.602E+10	-5.92665
Q16719	Kynureninase	KYNU	5.437E+09	-5.91348
Q92783	Signal transducing adapter molecule 1	STAM	826190000	-5.90976
P37802	Transgelin-2	TAGLN2	1.087E+10	-5.89213
P04632	Calpain small subunit 1	CAPNS1	1.701E+09	-5.88005
Q92890	Ubiquitin fusion degradation protein 1 homolog	UFD1L	881570000	-5.87193
O94915	Protein furry homolog-like	FRYL	427850000	-5.87187
P30041	Peroxiredoxin-6	PRDX6	1.716E+10	-5.8699
P23381	Tryptophan--tRNA ligase, cytoplasmic;T1-TrpRS;T2	WARS	4.377E+09	-5.80152
P12111	Collagen alpha-3(VI) chain	COL6A3	3.735E+09	-5.78699
P48637	Glutathione synthetase	GSS	2.745E+09	-5.77231
P41567	Eukaryotic translation initiation factor 1	EIF1	5.709E+09	-5.74382
Q15631	Translin	TSN	3.06E+09	-5.72499
P37268	Squalene synthase	FDFT1	211630000	-5.69598
P47895	Aldehyde dehydrogenase family 1 member A3	ALDH1A3	3.767E+09	-5.69082
Q13217	DnaJ homolog subfamily C member 3	DNAJC3	811060000	-5.66959
O14786	Neuropilin-1	NRP1	1.045E+09	-5.66309
O95747	Serine/threonine-protein kinase OSR1	OXSR1	1.413E+09	-5.66303
Q9HCJ6	Synaptic vesicle membrane protein VAT-1 homolog	VAT1L	1.053E+09	-5.61033
O15144	Actin-related protein 2/3 complex subunit 2	ARPC2	4.326E+09	-5.56927
P06737	Glycogen phosphorylase, liver form	PYGL	2.834E+09	-5.56067
Q9H299	SH3 domain-binding glutamic acid-rich-like protein	SH3BGRL3	2.792E+09	-5.54732
Q12765	Secernin-1	SCRN1	822210000	-5.5381
Q9P0V9	Septin-10	Sep-10	275630000	-5.5188
Q15181	Inorganic pyrophosphatase	PPA1	1.062E+10	-5.51229

P50552	Vasodilator-stimulated phosphoprotein	VASP	852320000	-5.48646
Q96G03	Phosphoglucomutase-2	PGM2	852710000	-5.48498
P29401	Transketolase	TKT	1.925E+10	-5.43525
P23526	Adenosylhomocysteinase	AHCY	1.277E+10	-5.43371
O00186	Syntaxin-binding protein 3	STXBP3	431360000	-5.42454
Q6IN85	Serine/threonine-protein phosphatase 4 regulator	SMEK1	567910000	-5.41697
Q04828	Aldo-keto reductase family 1 member C1	AKR1C1	6.001E+09	-5.4083
P09601	Heme oxygenase 1	HMOX1	1.131E+09	-5.40791
P11216	Glycogen phosphorylase, brain form	PYGB	2.41E+09	-5.39404
Q9NTX5	Ethylmalonyl-CoA decarboxylase	ECHDC1	1.383E+09	-5.38822
P53396	ATP-citrate synthase	ACLY	3.427E+10	-5.38082
O00193	Small acidic protein	SMAP	629130000	-5.37521
O75937	DnaJ homolog subfamily C member 8	DNAJC8	2.324E+09	-5.34409
P30085	UMP-CMP kinase	CMPK1	2.244E+09	-5.34119
Q14320	Protein FAM50A	FAM50A	836330000	-5.3406
P08133	Annexin A6	ANXA6	6.974E+09	-5.33632
Q92945	Far upstream element-binding protein 2	KHSRP	4.442E+09	-5.33605
P15531	Nucleoside diphosphate kinase A	NME1	8.223E+09	-5.33167
P61011	Signal recognition particle 54 kDa protein	SRP54	2.449E+09	-5.32282
P41250	Glycine--tRNA ligase	GARS	7.214E+09	-5.322
O75131	Copine-3	CPNE3	1.285E+09	-5.3164
P61081	NEDD8-conjugating enzyme Ubc12	UBE2M	1.016E+09	-5.30307
P52209	6-phosphogluconate dehydrogenase, decarboxylat	PGD	9.187E+09	-5.30284
P48739	Phosphatidylinositol transfer protein beta isoform	PITPNB	1.217E+09	-5.30061
Q15382	GTP-binding protein Rheb	RHEB	597380000	-5.29531
Q9H3N1	Thioredoxin-related transmembrane protein 1	TMX1	853690000	-5.293
P30044	Peroxiredoxin-5, mitochondrial	PRDX5	3.426E+09	-5.2765
Q9NT62	Ubiquitin-like-conjugating enzyme ATG3	ATG3	1.185E+09	-5.25295
P22314	Ubiquitin-like modifier-activating enzyme 1	UBA1	2.496E+10	-5.2476
O00194	Ras-related protein Rab-27B	RAB27B	828430000	-5.23619
P62820	Ras-related protein Rab-1A	RAB1A	1.047E+10	-5.2212
P11310	Medium-chain specific acyl-CoA dehydrogenase, m	ACADM	2.09E+09	-5.21114
P46926	Glucosamine-6-phosphate isomerase 1	GNPDA1	1.481E+09	-5.20604
P11177	Pyruvate dehydrogenase E1 component subunit be	PDHB	4.025E+09	-5.20138
Q8N3F8	MICAL-like protein 1	MICALL1	532840000	-5.19236
P19174	1-phosphatidylinositol 4,5-bisphosphate phosphoc	PLCG1	397030000	-5.17757
P18206	Vinculin	VCL	1.316E+10	-5.15376
P09429;B2I	High mobility group protein B1;Putative high mobi	HMGB1;HMGB1P1	3.998E+09	-5.11481
P16949	Stathmin	STMN1	2.775E+09	-5.11409
Q92688	Acidic leucine-rich nuclear phosphoprotein 32 fam	ANP32B	2.191E+09	-5.06884
P00558	Phosphoglycerate kinase 1	PGK1	4.354E+10	-5.06519
Q96AY3	Peptidyl-prolyl cis-trans isomerase FKBP10	FKBP10	641940000	-5.05921
Q14108	Lysosome membrane protein 2	SCARB2	2.129E+09	-5.05342
P17301	Integrin alpha-2	ITGA2	730190000	-5.05272
O60610	Protein diaphanous homolog 1	DIAPH1	1.262E+09	-5.03886
P07195	L-lactate dehydrogenase B chain	LDHB	6.087E+10	-5.02861
P20810	Calpastatin	CAST	2.858E+09	-5.0283
P31948	Stress-induced-phosphoprotein 1	STIP1	1.365E+10	-5.0189
Q00535	Cyclin-dependent-like kinase 5	CDK5	904870000	-4.9743
P14550	Alcohol dehydrogenase [NADP(+)]	AKR1A1	3.088E+09	-4.96225

O00499	Myc box-dependent-interacting protein 1	BIN1	563560000	-4.95715
P36507	Dual specificity mitogen-activated protein kinase k	MAP2K2	1.24E+09	-4.92513
O15118	Niemann-Pick C1 protein	NPC1	1.545E+09	-4.92369
Q13439	Golgin subfamily A member 4	GOLGA4	370350000	-4.91854
P48960	CD97 antigen;CD97 antigen subunit alpha;CD97 an	CD97	397850000	-4.89356
Q15185	Prostaglandin E synthase 3	PTGES3	1.949E+09	-4.8777
Q8N766	ER membrane protein complex subunit 1	EMC1	1.478E+09	-4.86999
Q9NY33	Dipeptidyl peptidase 3	DPP3	2.286E+09	-4.83205
Q16576	Histone-binding protein RBBP7	RBBP7	1.535E+09	-4.83044
Q9NR31	GTP-binding protein SAR1a	SAR1A	3.509E+09	-4.82834
Q15691	Microtubule-associated protein RP/EB family mem	MAPRE1	5.456E+09	-4.82389
P51148	Ras-related protein Rab-5C	RAB5C	4.365E+09	-4.8125
Q9NRW7	Vacuolar protein sorting-associated protein 45	VPS45	982200000	-4.81038
P09972	Fructose-bisphosphate aldolase C	ALDOC	6.455E+09	-4.80305
P20020	Plasma membrane calcium-transporting ATPase 1	ATP2B1	1.1E+09	-4.78968
Q8IWW6	Rho GTPase-activating protein 12	ARHGAP12	114990000	-4.77578
P07737	Profilin-1	PFN1	3.953E+10	-4.77432
O60701	UDP-glucose 6-dehydrogenase	UGDH	2.427E+09	-4.77428
Q9UBB4	Ataxin-10	ATXN10	946180000	-4.74587
P23919	Thymidylate kinase	DTYMK	1.358E+09	-4.73761
P04083	Annexin A1	ANXA1	1.146E+11	-4.73363
O43865	Putative adenosylhomocysteinase 2	AHCYL1	395110000	-4.72158
Q01518	Adenylyl cyclase-associated protein 1	CAP1	1.082E+10	-4.71224
O00429	Dynamin-1-like protein	DNM1L	3.955E+09	-4.71217
P16070	CD44 antigen	CD44	3.429E+09	-4.70581
P26885	Peptidyl-prolyl cis-trans isomerase FKBP2	FKBP2	936440000	-4.69129
Q6IBS0	Twinfilin-2	TWF2	3.029E+09	-4.68635
Q13637	Ras-related protein Rab-32	RAB32	483930000	-4.67668
Q9NZM1	Myoferlin	MYOF	4.735E+09	-4.6746
Q08257	Quinone oxidoreductase	CRYZ	1.604E+09	-4.67431
Q8NBJ5	Procollagen galactosyltransferase 1	COLGALT1	689010000	-4.67425
O94804	Serine/threonine-protein kinase 10	STK10	154860000	-4.66536
Q13098	COP9 signalosome complex subunit 1	GPS1	1.389E+09	-4.65456
P55145	Mesencephalic astrocyte-derived neurotrophic fac	MANF	1.698E+09	-4.64859
O00469	Procollagen-lysine,2-oxoglutarate 5-dioxygenase 2	PLOD2	1.211E+09	-4.63555
Q96I99	Succinyl-CoA ligase [GDP-forming] subunit beta, m	SUCLG2	2.209E+09	-4.62718
Q8WUM4	Programmed cell death 6-interacting protein	PDCD6IP	7.906E+09	-4.6212
O76003	Glutaredoxin-3	GLRX3	5.337E+09	-4.59833
P36871	Phosphoglucomutase-1	PGM1	4.057E+09	-4.59084
P30048	Thioredoxin-dependent peroxide reductase, mitoc	PRDX3	2.892E+09	-4.58698
P99999	Cytochrome c	CYCS	8.665E+09	-4.57955
P04080	Cystatin-B	CSTB	3.623E+09	-4.56674
Q9H2M9	Rab3 GTPase-activating protein non-catalytic subu	RAB3GAP2	522760000	-4.56535
Q99615	DnaJ homolog subfamily C member 7	DNAJC7	1.322E+09	-4.56179
Q8TEX9	Importin-4	IPO4	3.558E+09	-4.55321
P48735	Isocitrate dehydrogenase [NADP], mitochondrial	IDH2	2.251E+09	-4.54768
P38606	V-type proton ATPase catalytic subunit A	ATP6V1A	5.357E+09	-4.54341
P46734	Dual specificity mitogen-activated protein kinase k	MAP2K3	370110000	-4.53675
O60869	Endothelial differentiation-related factor 1	EDF1	760850000	-4.53417
P61923	Coatomer subunit zeta-1	COPZ1	1.009E+09	-4.52907

P50570	Dynamin-2	DNM2	3.451E+09	-4.5274
P49755	Transmembrane emp24 domain-containing protei	TMED10	5.648E+09	-4.5265
P10768	S-formylglutathione hydrolase	ESD	1.984E+09	-4.51296
P08134	Rho-related GTP-binding protein RhoC	RHOC	95490000	-4.51256
Q7Z6Z7	E3 ubiquitin-protein ligase HUWE1	HUWE1	3.315E+09	-4.5095
P30101	Protein disulfide-isomerase A3	PDIA3	2.708E+10	-4.48902
P60174	Triosephosphate isomerase	TPI1	8.067E+10	-4.48844
O75663	TIP41-like protein	TIPRL	1.456E+09	-4.48349
Q15056	Eukaryotic translation initiation factor 4H	EIF4H	4.547E+09	-4.48021
Q12792	Twinfilin-1	TWF1	1.458E+09	-4.47528
O15145	Actin-related protein 2/3 complex subunit 3	ARPC3	2.052E+09	-4.46813
Q14232	Translation initiation factor eIF-2B subunit alpha	EIF2B1	844020000	-4.46227
Q9BT78	COP9 signalosome complex subunit 4	COPS4	973670000	-4.45671
P33121	Long-chain-fatty-acid--CoA ligase 1	ACSL1	386230000	-4.45537
P17812	CTP synthase 1	CTPS1	6.296E+09	-4.45501
O75390	Citrate synthase, mitochondrial	CS	1.426E+10	-4.4406
P00338	L-lactate dehydrogenase A chain	LDHA	9.252E+10	-4.43821
Q8WW12	PEST proteolytic signal-containing nuclear protein	PCNP	617310000	-4.42502
Q13492	Phosphatidylinositol-binding clathrin assembly pro	PICALM	601810000	-4.4213
Q07960	Rho GTPase-activating protein 1	ARHGAP1	3.902E+09	-4.42007
P51812	Ribosomal protein S6 kinase alpha-3	RPS6KA3	1.173E+09	-4.41837
P61158	Actin-related protein 3	ACTR3	5.246E+09	-4.40197
P15121	Aldose reductase	AKR1B1	8.691E+09	-4.39534
P09622	Dihydrolipoyl dehydrogenase, mitochondrial	DLD	2.112E+09	-4.39425
Q15758	Neutral amino acid transporter B(0)	SLC1A5	1.837E+09	-4.37978
P19367	Hexokinase-1	HK1	1.217E+10	-4.37424
P14625	Endoplasmin	HSP90B1	4.314E+10	-4.36557
Q9HDC9	Adipocyte plasma membrane-associated protein	APMAP	1.362E+09	-4.34251
Q9UBT2	SUMO-activating enzyme subunit 2	UBA2	3.422E+09	-4.33941
P08758	Annexin A5	ANXA5	5.775E+10	-4.33089
Q9NR45	Sialic acid synthase	NANS	1.781E+09	-4.32673
P35237	Serpin B6	SERPINB6	1.618E+09	-4.32501
P37837	Transaldolase	TALDO1	3.515E+09	-4.32388
Q13177	Serine/threonine-protein kinase PAK 2;PAK-2p27;F	PAK2	1.475E+09	-4.314
P49321	Nuclear autoantigenic sperm protein	NASP	4.607E+09	-4.30439
Q70E73	Ras-associated and pleckstrin homology domains-c	RAPH1	222330000	-4.30406
P29144	Tripeptidyl-peptidase 2	TPP2	1.104E+09	-4.30023
P33991	DNA replication licensing factor MCM4	MCM4	7.06E+09	-4.29078
Q9UNZ2	NSFL1 cofactor p47	NSFL1C	912730000	-4.28765
O00299	Chloride intracellular channel protein 1	CLIC1	8.35E+09	-4.25185
Q9H3S7	Tyrosine-protein phosphatase non-receptor type 2	PTPN23	715300000	-4.24738
P00491	Purine nucleoside phosphorylase	PNP	5.641E+09	-4.24285
Q16836	Hydroxyacyl-coenzyme A dehydrogenase, mitocho	HADH	2.802E+09	-4.24259
P13797	Plastin-3	PLS3	1.108E+10	-4.23319
O75717	WD repeat and HMG-box DNA-binding protein 1	WDHD1	822040000	-4.23254
P54920	Alpha-soluble NSF attachment protein	NAPA	1.475E+09	-4.22981
Q8TC12	Retinol dehydrogenase 11	RDH11	1.085E+09	-4.22768
P51149	Ras-related protein Rab-7a	RAB7A	5.376E+09	-4.2232
P07355;A6I	Annexin A2;Putative annexin A2-like protein	ANXA2;ANXA2P2	6.979E+10	-4.21844
P06703	Protein S100-A6	S100A6	469620000	-4.21837

P54819	Adenylate kinase 2, mitochondrial;Adenylate kinas	AK2	3.032E+09	-4.20613
P23921	Ribonucleoside-diphosphate reductase large subu	RRM1	1.75E+09	-4.20428
P13693	Translationally-controlled tumor protein	TPT1	8.013E+09	-4.20385
Q13620	Cullin-4B	CUL4B	1.882E+09	-4.19271
Q9HC35	Echinoderm microtubule-associated protein-like 4	EML4	799120000	-4.18886
P22102	Trifunctional purine biosynthetic protein adenosin	GART	8.464E+09	-4.18484
P40926	Malate dehydrogenase, mitochondrial	MDH2	2.487E+10	-4.1747
P40925	Malate dehydrogenase, cytoplasmic	MDH1	8.917E+09	-4.16784
O15498	Synaptobrevin homolog YKT6	YKT6	583660000	-4.16748
P46939	Utrophin	UTRN	535060000	-4.16267
Q12931	Heat shock protein 75 kDa, mitochondrial	TRAP1	4.923E+09	-4.16255
Q13057	Bifunctional coenzyme A synthase;Phosphopantetl	COASY	1.125E+09	-4.14733
O43681	ATPase ASNA1	ASNA1	1.872E+09	-4.14513
Q99714	3-hydroxyacyl-CoA dehydrogenase type-2	HSD17B10	8.081E+09	-4.14173
Q92879	CUGBP Elav-like family member 1	CELF1	649190000	-4.13826
Q16555	Dihydropyrimidinase-related protein 2	DPYSL2	9.799E+09	-4.12942
Q01105	Protein SET	SET	1.15E+10	-4.12471
O00116	Alkyldihydroxyacetonephosphate synthase, peroxi	AGPS	1.141E+09	-4.12441
Q9P2R3	Rabankyrin-5	ANKFY1	918880000	-4.12122
P61201	COP9 signalosome complex subunit 2	COPS2	1.978E+09	-4.1177
O14745	Na(+)/H(+) exchange regulatory cofactor NHE-RF1	SLC9A3R1	403760000	-4.11566
Q14195	Dihydropyrimidinase-related protein 3	DPYSL3	2.334E+09	-4.0995
P21281	V-type proton ATPase subunit B, brain isoform	ATP6V1B2	2.418E+09	-4.08246
Q9P2I0	Cleavage and polyadenylation specificity factor sul	CPSF2	414220000	-4.07408
Q92520	Protein FAM3C	FAM3C	621480000	-4.06964
P62937	Peptidyl-prolyl cis-trans isomerase A;Peptidyl-prol	PPIA	5.398E+10	-4.06841
Q96HE7	ERO1-like protein alpha	ERO1L	3.228E+09	-4.06431
P45974	Ubiquitin carboxyl-terminal hydrolase 5	USP5	6.731E+09	-4.05964
Q9H553	Alpha-1,3/1,6-mannosyltransferase ALG2	ALG2	228400000	-4.05523
P41214	Eukaryotic translation initiation factor 2D	EIF2D	462520000	-4.05226
Q9H3K6	BolA-like protein 2	BOLA2	922480000	-4.05141
Q9NX40	OCIA domain-containing protein 1	OCIAD1	744330000	-4.05044
P52565	Rho GDP-dissociation inhibitor 1	ARHGDIA	1.175E+10	-4.04593
O14976	Cyclin-G-associated kinase	GAK	285730000	-4.0265
P51610	Host cell factor 1;HCF N-terminal chain 1;HCF N-te	HCFC1	1.579E+09	-4.02442
P13667	Protein disulfide-isomerase A4	PDIA4	1.553E+10	-4.01938
Q96KP4	Cytosolic non-specific dipeptidase	CNDP2	1.721E+09	-4.01513
P26639	Threonine--tRNA ligase, cytoplasmic	TARS	6.358E+09	-4.0147
P46109	Crk-like protein	CRKL	1.551E+09	-4.01014
Q9Y2V2	Calcium-regulated heat stable protein 1	CARHSP1	636030000	-4.0048
Q6UB35	Monofunctional C1-tetrahydrofolate synthase, mit	MTHFD1L	1.035E+09	-4.0012
Q14847	LIM and SH3 domain protein 1	LASP1	2.142E+09	-3.98465
Q9H0U4	Ras-related protein Rab-1B	RAB1B	2.498E+09	-3.98299
Q86XL3	Ankyrin repeat and LEM domain-containing protei	ANKLE2	173000000	-3.982
P49748	Very long-chain specific acyl-CoA dehydrogenase,	ACADVL	3.157E+09	-3.98102
P39748	Flap endonuclease 1	FEN1	4.392E+09	-3.97409
Q86VP6	Cullin-associated NEDD8-dissociated protein 1	CAND1	9.525E+09	-3.94897
Q9BSF4	Uncharacterized protein C19orf52	C19orf52	302400000	-3.9443
P05556	Integrin beta-1	ITGB1	5.518E+09	-3.932
P14618	Pyruvate kinase PKM	PKM	2.043E+11	-3.92699

O75521	Enoyl-CoA delta isomerase 2, mitochondrial	ECI2	1.013E+09	-3.92571
Q96EK9	Protein KTI12 homolog	KTI12	206690000	-3.91627
Q6DD88	Atlastin-3	ATL3	2.858E+09	-3.91529
P27361	Mitogen-activated protein kinase 3	MAPK3	1.699E+09	-3.91279
Q9Y3B3	Transmembrane emp24 domain-containing protei	TMED7	163510000	-3.90994
Q709C8	Vacuolar protein sorting-associated protein 13C	VPS13C	143710000	-3.90364
Q9UBF2	Coatomer subunit gamma-2	COPG2	1.093E+09	-3.90278
Q9NQW6	Actin-binding protein anillin	ANLN	169360000	-3.88975
P04075	Fructose-bisphosphate aldolase A	ALDOA	5.843E+10	-3.88923
P07099	Epoxide hydrolase 1	EPHX1	2.001E+09	-3.88224
O60763	General vesicular transport factor p115	USO1	3.61E+09	-3.86852
Q13085	Acetyl-CoA carboxylase 1;Biotin carboxylase	ACACA	508400000	-3.86044
Q93008	Probable ubiquitin carboxyl-terminal hydrolase FA	USP9X	1.516E+09	-3.85834
Q5VYK3	Proteasome-associated protein ECM29 homolog	ECM29	3.122E+09	-3.85314
Q9BZZ5	Apoptosis inhibitor 5	API5	1.818E+09	-3.85103
Q9Y490	Talin-1	TLN1	4.636E+10	-3.85041
P60903	Protein S100-A10	S100A10	904620000	-3.84814
P54578	Ubiquitin carboxyl-terminal hydrolase 14	USP14	2.43E+09	-3.83602
P61604	10 kDa heat shock protein, mitochondrial	HSPE1	4.234E+09	-3.8312
P34932	Heat shock 70 kDa protein 4	HSPA4	1.613E+10	-3.81425
Q14697	Neutral alpha-glucosidase AB	GANAB	1.779E+10	-3.81384
P22307	Non-specific lipid-transfer protein	SCP2	753750000	-3.80753
P36776	Lon protease homolog, mitochondrial	LONP1	8.144E+09	-3.7876
P13861	cAMP-dependent protein kinase type II-alpha regu	PRKAR2A	1.83E+09	-3.77454
Q07954	Prolow-density lipoprotein receptor-related protei	LRP1	3.399E+09	-3.77177
Q13033	Striatin-3	STRN3	449430000	-3.76837
Q96AC1	Fermitin family homolog 2	FERMT2	1.312E+09	-3.76831
P18754	Regulator of chromosome condensation	RCC1	628420000	-3.76705
P24928	DNA-directed RNA polymerase II subunit RPB1	POLR2A	350190000	-3.72101
Q9UI09	NADH dehydrogenase [ubiquinone] 1 alpha subcor	NDUFA12	429480000	-3.71784
Q02790	Peptidyl-prolyl cis-trans isomerase FKBP4;Peptidyl-	FKBP4	4.696E+09	-3.71465
Q16831	Uridine phosphorylase 1	UPP1	188990000	-3.70934
O94826	Mitochondrial import receptor subunit TOM70	TOMM70A	1.737E+09	-3.70411
Q96KB5	Lymphokine-activated killer T-cell-originated prote	PBK	276310000	-3.70159
Q99829	Copine-1	CPNE1	2.896E+09	-3.66665
Q9UBW8	COP9 signalosome complex subunit 7a	COPS7A	758500000	-3.66545
P60953	Cell division control protein 42 homolog	CDC42	1.196E+09	-3.66395
O14964	Hepatocyte growth factor-regulated tyrosine kinas	HGS	815260000	-3.66178
P51452	Dual specificity protein phosphatase 3	DUSP3	822690000	-3.65793
P00505	Aspartate aminotransferase, mitochondrial	GOT2	5.033E+09	-3.65437
Q15942	Zyxin	ZYX	2.511E+09	-3.65319
P35613	Basigin	BSG	3.045E+09	-3.6503
P17931	Galectin-3	LGALS3	160130000	-3.64963
Q01085	Nucleolysin TIAR	TIAL1	603170000	-3.64962
P06733	Alpha-enolase	ENO1	1.599E+11	-3.64063
Q00688	Peptidyl-prolyl cis-trans isomerase FKBP3	FKBP3	1.2E+09	-3.6328
Q9Y696	Chloride intracellular channel protein 4	CLIC4	3.143E+09	-3.63266
P54727	UV excision repair protein RAD23 homolog B	RAD23B	1.892E+09	-3.63194
P15170	Eukaryotic peptide chain release factor GTP-bindin	GSPT1	2.632E+09	-3.63126
Q9BUL8	Programmed cell death protein 10	PDCD10	782200000	-3.62834

Q6UWE0	E3 ubiquitin-protein ligase LRSAM1	LRSAM1	217520000	-3.62804
Q9H1E3	Nuclear ubiquitous casein and cyclin-dependent ki	NUCKS1	1.291E+09	-3.62784
P49023	Paxillin	PXN	1.915E+09	-3.6267
Q05682	Caldesmon	CALD1	1.492E+09	-3.62199
Q16658	Fascin	FSCN1	4.039E+09	-3.61437
O43156	TELO2-interacting protein 1 homolog	TTI1	288310000	-3.60957
Q92905	COP9 signalosome complex subunit 5	COPS5	1.015E+09	-3.597
P54886	Delta-1-pyrroline-5-carboxylate synthase;Glutama	ALDH18A1	2.718E+09	-3.58144
P21283	V-type proton ATPase subunit C 1	ATP6V1C1	935790000	-3.57845
P61077	Ubiquitin-conjugating enzyme E2 D3	UBE2D3	719010000	-3.5746
P50851	Lipopolysaccharide-responsive and beige-like anch	LRBA	307710000	-3.57065
O00410	Importin-5	IPO5	1.608E+10	-3.5706
P07900	Heat shock protein HSP 90-alpha	HSP90AA1	1.158E+11	-3.56944
Q15437	Protein transport protein Sec23B	SEC23B	754660000	-3.56437
P12110	Collagen alpha-2(VI) chain	COL6A2	411710000	-3.56349
Q04917	14-3-3 protein eta	YWHAH	3.102E+09	-3.55367
Q7Z460	CLIP-associating protein 1	CLASP1	267260000	-3.55123
P22392;O6	Nucleoside diphosphate kinase B;Putative nucleosi	NME2;NME2P1	2.413E+10	-3.55119
P01911	HLA class II histocompatibility antigen, DRB1-15 be	HLA-DRB1	387830000	-3.54149
P34897	Serine hydroxymethyltransferase, mitochondrial	SHMT2	6.545E+09	-3.53403
Q96QK1	Vacuolar protein sorting-associated protein 35	VPS35	5.34E+09	-3.51499
P11717	Cation-independent mannose-6-phosphate recept	IGF2R	911840000	-3.50321
Q9NYA1	Sphingosine kinase 1	SPHK1	96197000	-3.49605
Q3KQV9	UDP-N-acetylhexosamine pyrophosphorylase-like		UAP1L1	1.15E+09	-3.47845
P30086	Phosphatidylethanolamine-binding protein 1;Hipp	PEBP1	8.565E+09	-3.4688
P31946	14-3-3 protein beta/alpha;14-3-3 protein beta/alpl	YWHAB	1.001E+10	-3.4528
P08473	Neprilysin	MME	181880000	-3.45134
O95197	Reticulon-3	RTN3	429120000	-3.44708
Q16851	UTP--glucose-1-phosphate uridylyltransferase	UGP2	2.5E+09	-3.4379
Q92734	Protein TFG	TFG	1.056E+09	-3.4336
Q9NUP9	Protein lin-7 homolog C	LIN7C	199710000	-3.4326
Q9Y2B0	Protein canopy homolog 2	CNPY2	1.089E+09	-3.43254
P48509	CD151 antigen	CD151	85623000	-3.42826
Q9BXJ9	N-alpha-acetyltransferase 15, NatA auxiliary subun	NAA15	3.332E+09	-3.4262
Q15365	Poly(rC)-binding protein 1	PCBP1	1.4E+10	-3.41873
O95456	Proteasome assembly chaperone 1	PSMG1	497360000	-3.41864
Q9BSJ8	Extended synaptotagmin-1	ESYT1	5.149E+09	-3.40915
P26006	Integrin alpha-3;Integrin alpha-3 heavy chain;Integ	ITGA3	3.172E+09	-3.40762
O43719	HIV Tat-specific factor 1	HTATSF1	768340000	-3.40605
Q15084	Protein disulfide-isomerase A6	PDIA6	1.397E+10	-3.4041
P12004	Proliferating cell nuclear antigen	PCNA	7.717E+09	-3.39364
Q04323	UBX domain-containing protein 1	UBXN1	1.111E+09	-3.39269
Q8N1G4	Leucine-rich repeat-containing protein 47	LRRC47	624940000	-3.38966
O00232	26S proteasome non-ATPase regulatory subunit 12	PSMD12	7.525E+09	-3.37452
O15143	Actin-related protein 2/3 complex subunit 1B	ARPC1B	1.234E+09	-3.37413
Q9NVD7	Alpha-parvin	PARVA	693800000	-3.37376
P13639	Elongation factor 2	EEF2	1.124E+11	-3.36408
O95433	Activator of 90 kDa heat shock protein ATPase hon	AHSA1	7.859E+09	-3.36086
Q9HA77	Probable cysteine--tRNA ligase, mitochondrial	CARS2	726290000	-3.35853
P50454	Serpin H1	SERPINH1	6.178E+09	-3.35636

P61026	Ras-related protein Rab-10	RAB10	1.961E+09	-3.34233
P08243	Asparagine synthetase [glutamine-hydrolyzing]	ASNS	1.932E+09	-3.33939
Q9BSH4	Translational activator of cytochrome c oxidase 1	TACO1	823850000	-3.33878
P27797	Calreticulin	CALR	3.09E+10	-3.33278
Q14C86	GTPase-activating protein and VPS9 domain-conta	GAPVD1	1.111E+09	-3.31002
P49189	4-trimethylaminobutyraldehyde dehydrogenase	ALDH9A1	804540000	-3.30978
Q96C19	EF-hand domain-containing protein D2	EFHD2	697240000	-3.30788
P46459	Vesicle-fusing ATPase	NSF	2.724E+09	-3.30651
P13073	Cytochrome c oxidase subunit 4 isoform 1, mitocho	COX4I1	2.03E+09	-3.29809
O75116	Rho-associated protein kinase 2	ROCK2	482810000	-3.2961
P27348	14-3-3 protein theta	YWHAQ	6.911E+09	-3.29423
Q7Z3B4	Nucleoporin p54	NUP54	330960000	-3.28797
Q14247	Src substrate cortactin	CTTN	5.813E+09	-3.28778
Q9UNS2	COP9 signalosome complex subunit 3	COPS3	1.39E+09	-3.28769
Q99832	T-complex protein 1 subunit eta	CCT7	1.805E+10	-3.27878
P13473	Lysosome-associated membrane glycoprotein 2	LAMP2	1.466E+09	-3.27688
Q2M2I8	AP2-associated protein kinase 1	AAK1	283270000	-3.27452
P00492	Hypoxanthine-guanine phosphoribosyltransferase	HPRT1	5.031E+09	-3.27374
P22234	Multifunctional protein ADE2;Phosphoribosylamin	PAICS	9.656E+09	-3.26723
P10155	60 kDa SS-A/Ro ribonucleoprotein	TROVE2	1.224E+09	-3.26712
Q9BVK6	Transmembrane emp24 domain-containing protei	TMED9	1.273E+09	-3.26621
Q96TA1	Niban-like protein 1	FAM129B	7.402E+09	-3.26496
Q9BZQ8	Protein Niban	FAM129A	3.284E+09	-3.2616
P61758	Prefoldin subunit 3	VBP1	2.1E+09	-3.25849
P27824	Calnexin	CANX	1.215E+10	-3.25764
Q9Y6D5	Brefeldin A-inhibited guanine nucleotide-exchange	ARFGEF2	449220000	-3.25274
P61163	Alpha-centractin	ACTR1A	6.007E+09	-3.24707
Q9HB71	Calcyclin-binding protein	CACYBP	5.409E+09	-3.24545
O95801	Tetratricopeptide repeat protein 4	TTC4	436710000	-3.24207
O43432	Eukaryotic translation initiation factor 4 gamma 3	EIF4G3	342700000	-3.24165
P61019	Ras-related protein Rab-2A	RAB2A	2.573E+09	-3.23828
Q00765	Receptor expression-enhancing protein 5	REEP5	510290000	-3.23823
P49419	Alpha-aminoadipic semialdehyde dehydrogenase	ALDH7A1	1.889E+09	-3.22945
Q9H4A3	Serine/threonine-protein kinase WNK1	WNK1	261730000	-3.22821
Q13526	Peptidyl-prolyl cis-trans isomerase NIMA-interactir	PIN1	744220000	-3.22436
P62826	GTP-binding nuclear protein Ran	RAN	1.095E+10	-3.22231
P39687	Acidic leucine-rich nuclear phosphoprotein 32 fam	ANP32A	2.368E+09	-3.21397
O75915	PRA1 family protein 3	ARL6IP5	2.568E+09	-3.21345
Q8NBS9	Thioredoxin domain-containing protein 5	TXNDC5	3.814E+09	-3.20303
P35658	Nuclear pore complex protein Nup214	NUP214	358270000	-3.19995
P54709	Sodium/potassium-transporting ATPase subunit be	ATP1B3	3.26E+09	-3.19926
P50995	Annexin A11	ANXA11	519530000	-3.19693
P61088;Q5	Ubiquitin-conjugating enzyme E2 N;Putative ubiqu	UBE2N;UBE2NL	687430000	-3.19643
Q9NTK5	Obg-like ATPase 1	OLA1	5.392E+09	-3.19336
Q15642	Cdc42-interacting protein 4	TRIP10	622610000	-3.18586
Q86VS8	Protein Hook homolog 3	HOOK3	505230000	-3.18107
P13798	Acylamino-acid-releasing enzyme	APEH	2.097E+09	-3.17422
Q7KZF4	Staphylococcal nuclease domain-containing protei	SND1	1.317E+10	-3.17234
O95299	NADH dehydrogenase [ubiquinone] 1 alpha subcor	NDUFA10	1.47E+09	-3.15975
Q56VL3	OCIA domain-containing protein 2	OCIAD2	1.463E+09	-3.15742

Q9Y2W2	WW domain-binding protein 11	WBP11	335590000	-3.15513
P20337	Ras-related protein Rab-3B	RAB3B	1.043E+09	-3.15365
P61586	Transforming protein RhoA	RHOA	2.585E+09	-3.15324
P84077;P61	ADP-ribosylation factor 1;ADP-ribosylation factor 3	ARF1;ARF3	1.547E+10	-3.14319
Q15366	Poly(rC)-binding protein 2	PCBP2	8.295E+09	-3.14102
P08754	Guanine nucleotide-binding protein G(k) subunit a	GNAI3	1.473E+09	-3.13951
P07237	Protein disulfide-isomerase	P4HB	2.8E+10	-3.13384
O15355	Protein phosphatase 1G	PPM1G	2.117E+09	-3.12565
P55809	Succinyl-CoA:3-ketoacid coenzyme A transferase 1	OXCT1	2.458E+09	-3.12403
Q16799	Reticulon-1	RTN1	313620000	-3.12044
Q9NYU2	UDP-glucose:glycoprotein glucosyltransferase 1	UGGT1	3.67E+09	-3.10907
O75083	WD repeat-containing protein 1	WDR1	9.679E+09	-3.10899
P08238	Heat shock protein HSP 90-beta	HSP90AB1	6.076E+10	-3.10136
O43847	Nardilysin	NRD1	701660000	-3.08654
P38117	Electron transfer flavoprotein subunit beta	ETFB	2.395E+09	-3.08046
P15153	Ras-related C3 botulinum toxin substrate 2	RAC2	1.047E+09	-3.07747
O00154	Cytosolic acyl coenzyme A thioester hydrolase	ACOT7	1.366E+09	-3.066
Q13838	Spliceosome RNA helicase DDX39B	DDX39B	4.81E+09	-3.06181
Q9NWV4	UPF0587 protein C1orf123	C1orf123	507210000	-3.05969
P12081	Histidine--tRNA ligase, cytoplasmic	HARS	3.604E+09	-3.05955
Q9NSE4	Isoleucine--tRNA ligase, mitochondrial	IARS2	1.296E+09	-3.04686
Q5JRX3	Presequence protease, mitochondrial	PITRM1	1.432E+09	-3.04668
Q96FW1	Ubiquitin thioesterase OTUB1	OTUB1	2.188E+09	-3.04443
P00441	Superoxide dismutase [Cu-Zn]	SOD1	2.919E+09	-3.04423
Q86V21	Acetoacetyl-CoA synthetase	AACS	161670000	-3.0425
Q01813	ATP-dependent 6-phosphofructokinase, platelet ty	PFKP	7.242E+09	-3.03493
Q9NQC3	Reticulon-4	RTN4	9.273E+09	-3.0319
O60502	Protein O-GlcNAcase	MGEA5	617710000	-3.02967
P08195	4F2 cell-surface antigen heavy chain	SLC3A2	1.812E+09	-3.02929
Q9Y2L1	Exosome complex exonuclease RRP44	DIS3	2.033E+09	-3.02317
P61160	Actin-related protein 2	ACTR2	3.611E+09	-3.01803
Q92973	Transportin-1	TNPO1	3.247E+09	-3.0157
Q9H2U2	Inorganic pyrophosphatase 2, mitochondrial	PPA2	1.152E+09	-3.01543
P61106	Ras-related protein Rab-14	RAB14	1.226E+09	-3.01502
Q7L5N1	COP9 signalosome complex subunit 6	COPS6	970130000	-3.01423
Q15006	ER membrane protein complex subunit 2	EMC2	936910000	-3.00722
Q9BQ52	Zinc phosphodiesterase ELAC protein 2	ELAC2	774340000	-2.99696
Q8TCT9	Minor histocompatibility antigen H13	HM13	1.703E+09	-2.99045
P33316	Deoxyuridine 5-triphosphate nucleotidohydrolase,	DUT	4.022E+09	-2.98642
O75306	NADH dehydrogenase [ubiquinone] iron-sulfur pro	NDUFS2	1.612E+09	-2.98605
Q15907;P6	Ras-related protein Rab-11B;Ras-related protein R	RAB11B;RAB11A	2.22E+09	-2.98084
P36551	Oxygen-dependent coproporphyrinogen-III oxidase	CPOX	977030000	-2.97767
O60499	Syntaxin-10	STX10	161540000	-2.97667
P28340	DNA polymerase delta catalytic subunit	POLD1	635050000	-2.9756
Q9UKF6	Cleavage and polyadenylation specificity factor sul	CPSF3	1.023E+09	-2.96998
Q9Y5X1	Sorting nexin-9	SNX9	388290000	-2.95908
Q9P289	Serine/threonine-protein kinase 26	STK26	761070000	-2.95624
Q92544	Transmembrane 9 superfamily member 4	TM9SF4	1.445E+09	-2.94598
P08559	Pyruvate dehydrogenase E1 component subunit al	PDHA1	2.225E+09	-2.93253
P18564	Integrin beta-6	ITGB6	459750000	-2.93021

Q08378	Golgin subfamily A member 3	GOLGA3	413040000	-2.9265
P84085	ADP-ribosylation factor 5	ARF5	7.25E+09	-2.92396
Q8IZP0	Abl interactor 1	ABI1	456600000	-2.92367
Q8IVF2	Protein AHNAK2	AHNAK2	1.783E+10	-2.91172
Q9Y2A7	Nck-associated protein 1	NCKAP1	1.242E+09	-2.9058
Q9Y570	Protein phosphatase methylesterase 1	PPME1	619340000	-2.90362
O15397	Importin-8	IPO8	359510000	-2.90222
O95716	Ras-related protein Rab-3D	RAB3D	200160000	-2.8986
O43837	Isocitrate dehydrogenase [NAD] subunit beta, mito	IDH3B	1.75E+09	-2.89145
Q92598	Heat shock protein 105 kDa	HSPH1	6.705E+09	-2.89131
Q13464	Rho-associated protein kinase 1	ROCK1	178150000	-2.88576
O00471	Exocyst complex component 5	EXOC5	357890000	-2.88444
O96005	Cleft lip and palate transmembrane protein 1	CLPTM1	448310000	-2.87405
Q9UKK9	ADP-sugar pyrophosphatase	NUDT5	2.154E+09	-2.87352
P31947	14-3-3 protein sigma	SFN	1.395E+09	-2.8715
P08648	Integrin alpha-5;Integrin alpha-5 heavy chain;Integ	ITGA5	1.314E+09	-2.86756
P61221	ATP-binding cassette sub-family E member 1	ABCE1	3.726E+09	-2.86695
O60749	Sorting nexin-2	SNX2	1.373E+09	-2.86605
Q9NV70	Exocyst complex component 1	EXOC1	746440000	-2.86184
Q99497	Protein deglycase DJ-1	PARK7	5.959E+09	-2.85716
Q15008	26S proteasome non-ATPase regulatory subunit 6	PSMD6	4.478E+09	-2.85704
Q15436	Protein transport protein Sec23A	SEC23A	3.532E+09	-2.85345
Q9BYC5	Alpha-(1,6)-fucosyltransferase	FUT8	696830000	-2.84959
Q15404	Ras suppressor protein 1	RSU1	2.096E+09	-2.84754
Q13509	Tubulin beta-3 chain	TUBB3	9.973E+09	-2.84446
Q96P70	Importin-9	IPO9	1.291E+09	-2.83393
Q14643	Inositol 1,4,5-trisphosphate receptor type 1	ITPR1	671700000	-2.83172
P50990	T-complex protein 1 subunit theta	CCT8	1.683E+10	-2.82585
Q15020	Squamous cell carcinoma antigen recognized by T-	SART3	633770000	-2.82246
P60842	Eukaryotic initiation factor 4A-I	EIF4A1	4.519E+10	-2.82122
Q9UQ80	Proliferation-associated protein 2G4	PA2G4	5.475E+09	-2.81945
Q9UIA9	Exportin-7	XPO7	500690000	-2.81701
Q7Z2W9	39S ribosomal protein L21, mitochondrial	MRPL21	618800000	-2.81472
Q13907	Isopentenyl-diphosphate Delta-isomerase 1	IDI1	741040000	-2.81434
Q9NZL9	Methionine adenosyltransferase 2 subunit beta	MAT2B	1.021E+09	-2.80891
P48444	Coatomer subunit delta	ARCN1	3.371E+09	-2.80748
P17612;P22	cAMP-dependent protein kinase catalytic subunit a	PRKACA;PRKACB	565780000	-2.8036
O15511	Actin-related protein 2/3 complex subunit 5	ARPC5	1.093E+09	-2.79912
O15460	Prolyl 4-hydroxylase subunit alpha-2	P4HA2	1.614E+09	-2.78365
P36405	ADP-ribosylation factor-like protein 3	ARL3	1.948E+09	-2.78197
O75436	Vacuolar protein sorting-associated protein 26A	VPS26A	1.694E+09	-2.77979
Q9BTW9	Tubulin-specific chaperone D	TBCD	1.011E+09	-2.77916
Q9UKS6	Protein kinase C and casein kinase substrate in neu	PACSIN3	128130000	-2.77798
Q16181	Septin-7	Sep-07	3.075E+09	-2.77591
Q14974	Importin subunit beta-1	KPNB1	2.508E+10	-2.77395
Q9BZG1	Ras-related protein Rab-34	RAB34	266300000	-2.76812
Q9NQW7	Xaa-Pro aminopeptidase 1	XPNPEP1	1.498E+09	-2.765
Q92575	UBX domain-containing protein 4	UBXN4	274270000	-2.76291
P56545	C-terminal-binding protein 2	CTBP2	540190000	-2.75672
Q96S44	TP53-regulating kinase	TP53RK	397330000	-2.75499

P12931	Proto-oncogene tyrosine-protein kinase Src	SRC	189270000	-2.75012
Q9P0L0	Vesicle-associated membrane protein-associated p	VAPA	2.359E+09	-2.74909
Q6ZSR9	Uncharacterized protein FLJ45252		165230000	-2.74468
Q3V6T2	Girdin	CCDC88A	68424000	-2.74262
Q9Y678	Coatomer subunit gamma-1	COPG1	4.188E+09	-2.74114
P59998	Actin-related protein 2/3 complex subunit 4	ARPC4	2.825E+09	-2.73419
P26583	High mobility group protein B2	HMGB2	1.512E+09	-2.73243
P62877	E3 ubiquitin-protein ligase RBX1;E3 ubiquitin-prote	RBX1	324230000	-2.72691
P63104	14-3-3 protein zeta/delta	YWHAZ	2.642E+10	-2.72465
Q9UKY7	Protein CDV3 homolog	CDV3	1.939E+09	-2.72345
P48643	T-complex protein 1 subunit epsilon	CCT5	2.94E+10	-2.72206
Q9Y6E0	Serine/threonine-protein kinase 24;Serine/threoni	STK24	348100000	-2.72126
Q9Y266	Nuclear migration protein nudC	NUDC	3.886E+09	-2.71223
P26640	Valine--tRNA ligase	VARS	4.847E+09	-2.7112
P13674	Prolyl 4-hydroxylase subunit alpha-1	P4HA1	984730000	-2.70873
Q9NS69	Mitochondrial import receptor subunit TOM22 hor	TOMM22	919630000	-2.70569
P17980	26S protease regulatory subunit 6A	PSMC3	8.852E+09	-2.69982
O60488	Long-chain-fatty-acid--CoA ligase 4	ACSL4	2.113E+09	-2.69435
P28074	Proteasome subunit beta type-5	PSMB5	3.029E+09	-2.68887
P17987	T-complex protein 1 subunit alpha	TCP1	1.97E+10	-2.66472
P51570	Galactokinase	GALK1	1.018E+09	-2.66051
P04406	Glyceraldehyde-3-phosphate dehydrogenase	GAPDH	2.828E+11	-2.65682
P15311	Ezrin	EZR	2.636E+09	-2.65522
P61289	Proteasome activator complex subunit 3	PSME3	3.762E+09	-2.65374
Q9Y547	Intraflagellar transport protein 25 homolog	HSPB11	330070000	-2.65253
Q15738	Sterol-4-alpha-carboxylate 3-dehydrogenase, deca	NSDHL	2.071E+09	-2.64808
Q9BTE6	Alanyl-tRNA editing protein Aarsd1	AARSD1	229040000	-2.64655
Q9UPN7	Serine/threonine-protein phosphatase 6 regulator	PPP6R1	303470000	-2.644
P12268	Inosine-5-monophosphate dehydrogenase 2	IMPDH2	9.798E+09	-2.64127
Q13557	Calcium/calmodulin-dependent protein kinase typ	CAMK2D	1.917E+09	-2.63935
P50991	T-complex protein 1 subunit delta	CCT4	2.055E+10	-2.63775
Q99733	Nucleosome assembly protein 1-like 4	NAP1L4	2.747E+09	-2.63668
Q9BXS5	AP-1 complex subunit mu-1	AP1M1	1.339E+09	-2.63386
Q9Y3E0	Vesicle transport protein GOT1B	GOLT1B	388560000	-2.63278
P14324	Farnesyl pyrophosphate synthase	FDPS	792170000	-2.62787
P51153	Ras-related protein Rab-13	RAB13	938640000	-2.62706
P43304	Glycerol-3-phosphate dehydrogenase, mitochondr	GPD2	2.753E+09	-2.62584
Q96JB5	CDK5 regulatory subunit-associated protein 3	CDK5RAP3	407180000	-2.625
P15309	Prostatic acid phosphatase;PAPf39	ACPP	78529000	-2.618
Q14566	DNA replication licensing factor MCM6	MCM6	5.937E+09	-2.61573
Q13535	Serine/threonine-protein kinase ATR	ATR	93981000	-2.60783
P42224	Signal transducer and activator of transcription 1-a	STAT1	2.005E+09	-2.60613
P53582	Methionine aminopeptidase 1	METAP1	168640000	-2.604
Q16543	Hsp90 co-chaperone Cdc37;Hsp90 co-chaperone C	CDC37	5.135E+09	-2.60307
Q9Y5K5	Ubiquitin carboxyl-terminal hydrolase isozyme L5	UCHL5	1.255E+09	-2.60211
Q6UW68	Transmembrane protein 205	TMEM205	400720000	-2.59905
P12955	Xaa-Pro dipeptidase	PEPD	902380000	-2.58646
P09104	Gamma-enolase	ENO2	4.918E+09	-2.58336
P20340	Ras-related protein Rab-6A	RAB6A	2.454E+09	-2.57727
P62495	Eukaryotic peptide chain release factor subunit 1	ETF1	2.821E+09	-2.57397

O14530	Thioredoxin domain-containing protein 9	TXNDC9	209090000	-2.56812
P55084	Trifunctional enzyme subunit beta, mitochondrial;3	HADHB	4.022E+09	-2.56537
Q9Y450	HBS1-like protein	HBS1L	764520000	-2.55913
Q9UJZ1	Stomatin-like protein 2, mitochondrial	STOML2	2.951E+09	-2.55605
P00403	Cytochrome c oxidase subunit 2	MT-CO2	4.155E+09	-2.54838
Q9C0C9	E2/E3 hybrid ubiquitin-protein ligase UBE2O	UBE2O	1.126E+09	-2.54389
P17655	Calpain-2 catalytic subunit	CAPN2	1.174E+10	-2.53928
P49327	Fatty acid synthase;[Acyl-carrier-protein] S-acetylt	FASN	3.037E+10	-2.53761
Q9Y3A5	Ribosome maturation protein SBDS	SBDS	534290000	-2.53677
P08240	Signal recognition particle receptor subunit alpha	SRPR	678140000	-2.53386
P08237	ATP-dependent 6-phosphofructokinase, muscle ty	PFKM	2.205E+09	-2.526
Q9Y2Q3	Glutathione S-transferase kappa 1	GSTK1	991040000	-2.5238
Q92947	Glutaryl-CoA dehydrogenase, mitochondrial	GCDH	330040000	-2.52006
O43708	Maleylacetoacetate isomerase	GSTZ1	560620000	-2.51799
Q06210	Glutamine--fructose-6-phosphate aminotransferas	GFPT1	2.354E+09	-2.51786
P78371	T-complex protein 1 subunit beta	CCT2	3.661E+10	-2.51244
P32322	Pyrroline-5-carboxylate reductase 1, mitochondria	PYCR1	492320000	-2.51003
Q15125	3-beta-hydroxysteroid-Delta(8),Delta(7)-isomerase	EBP	994480000	-2.50684
P36404	ADP-ribosylation factor-like protein 2	ARL2	587170000	-2.50581
Q9HC38	Glyoxalase domain-containing protein 4	GLOD4	2.575E+09	-2.50262
Q58FF8	Putative heat shock protein HSP 90-beta 2	HSP90AB2P	3.706E+09	-2.50096
P62330	ADP-ribosylation factor 6	ARF6	894040000	-2.49757
Q6YN16	Hydroxysteroid dehydrogenase-like protein 2	HSDL2	1.307E+09	-2.49411
O60256	Phosphoribosyl pyrophosphate synthase-associate	PRPSAP2	739940000	-2.48324
Q9UHD8	Septin-9	Sep-09	2.763E+09	-2.47905
P62258	14-3-3 protein epsilon	YWHAE	3.56E+10	-2.47762
P24752	Acetyl-CoA acetyltransferase, mitochondrial	ACAT1	4.656E+09	-2.4766
Q92922	SWI/SNF complex subunit SMARCC1	SMARCC1	295370000	-2.47472
O95163	Elongator complex protein 1	IKBKAP	960260000	-2.47012
P67812	Signal peptidase complex catalytic subunit SEC11A	SEC11A	834860000	-2.46698
Q16531	DNA damage-binding protein 1	DDB1	5.704E+09	-2.46461
P46060	Ran GTPase-activating protein 1	RANGAP1	6.803E+09	-2.46414
P26038	Moesin	MSN	1.745E+10	-2.45913
Q9HAV4	Exportin-5	XPO5	1.623E+09	-2.45865
Q06323	Proteasome activator complex subunit 1	PSME1	2.531E+09	-2.45597
P53618	Coatomer subunit beta	COPB1	7.497E+09	-2.44801
P53634	Dipeptidyl peptidase 1;Dipeptidyl peptidase 1 excl	CTSC	31981000	-2.44309
Q9NVJ2	ADP-ribosylation factor-like protein 8B	ARL8B	1.109E+09	-2.44192
Q16878	Cysteine dioxygenase type 1	CDO1	118520000	-2.42662
Q96RQ1	Endoplasmic reticulum-Golgi intermediate compar	ERGIC2	239990000	-2.42652
Q9NTZ6	RNA-binding protein 12	RBM12	917440000	-2.4246
Q9UL63	Muskelin	MKLN1	370160000	-2.42248
P21796	Voltage-dependent anion-selective channel protei	VDAC1	7.212E+09	-2.42117
Q8NFQ8	Torsin-1A-interacting protein 2	TOR1AIP2	262390000	-2.41949
P04843	Dolichyl-diphosphooligosaccharide--protein glycos	RPN1	1.022E+10	-2.41613
O75874	Isocitrate dehydrogenase [NADP] cytoplasmic	IDH1	2.827E+09	-2.40978
Q15643	Thyroid receptor-interacting protein 11	TRIP11	316530000	-2.40917
P06454	Prothymosin alpha;Prothymosin alpha, N-terminal	PTMA	673670000	-2.39319
P02786	Transferrin receptor protein 1;Transferrin recepto	TFRC	9.354E+09	-2.39236
P23284	Peptidyl-prolyl cis-trans isomerase B	PPIB	7.013E+09	-2.38466

P30153	Serine/threonine-protein phosphatase 2A 65 kDa r	PPP2R1A	8.295E+09	-2.38437
P27816	Microtubule-associated protein 4	MAP4	5.575E+09	-2.38318
Q96EE3	Nucleoporin SEH1	SEH1L	234430000	-2.3831
O76094	Signal recognition particle subunit SRP72	SRP72	2.266E+09	-2.38071
P30040	Endoplasmic reticulum resident protein 29	ERP29	2.232E+09	-2.37981
Q9NYL2	Mitogen-activated protein kinase kinase kinase ML	ZAK	576330000	-2.37978
P18085	ADP-ribosylation factor 4	ARF4	3.103E+09	-2.37966
P49589	Cysteine--tRNA ligase, cytoplasmic	CARS	598850000	-2.37858
Q7L1Q6	Basic leucine zipper and W2 domain-containing pro	BZW1	3.586E+09	-2.37603
Q9UNH7	Sorting nexin-6;Sorting nexin-6, N-terminally proce	SNX6	627370000	-2.3716
O43175	D-3-phosphoglycerate dehydrogenase	PHGDH	1.101E+10	-2.36781
P63241;Q6	Eukaryotic translation initiation factor 5A-1;Eukary	EIF5A;EIF5AL1;EIF5	1.593E+10	-2.36698
O00151	PDZ and LIM domain protein 1	PDLIM1	1.997E+09	-2.36569
Q9ULT8	E3 ubiquitin-protein ligase HECTD1	HECTD1	549720000	-2.36451
P48556	26S proteasome non-ATPase regulatory subunit 8	PSMD8	1.882E+09	-2.36238
O76031	ATP-dependent Clp protease ATP-binding subunit c	CLPX	379990000	-2.36076
Q9P0J0	NADH dehydrogenase [ubiquinone] 1 alpha subcon	NDUFA13	745000000	-2.35451
Q92888	Rho guanine nucleotide exchange factor 1	ARHGEF1	570410000	-2.35325
P04179	Superoxide dismutase [Mn], mitochondrial	SOD2	2.546E+09	-2.35124
Q13596	Sorting nexin-1	SNX1	632300000	-2.34275
O75962	Triple functional domain protein	TRIO	633220000	-2.34112
P14406	Cytochrome c oxidase subunit 7A2, mitochondrial	COX7A2	619690000	-2.3314
O94903	Proline synthase co-transcribed bacterial homolog	PROSC	662610000	-2.3242
P00367;P49	Glutamate dehydrogenase 1, mitochondrial;Glutar	GLUD1;GLUD2	3.664E+09	-2.31884
O43242	26S proteasome non-ATPase regulatory subunit 3	PSMD3	6.594E+09	-2.3185
Q7L311	Armadillo repeat-containing X-linked protein 2	ARMCX2	126350000	-2.31383
Q9UH99	SUN domain-containing protein 2	SUN2	435990000	-2.31345
P00533	Epidermal growth factor receptor	EGFR	613290000	-2.31294
P09525	Annexin A4	ANXA4	2.248E+09	-2.31228
P11586	C-1-tetrahydrofolate synthase, cytoplasmic;Methy	MTHFD1	2.048E+10	-2.31124
Q15819	Ubiquitin-conjugating enzyme E2 variant 2	UBE2V2	1.996E+09	-2.30942
Q9BTE3	Mini-chromosome maintenance complex-binding p	MCMBP	448110000	-2.30839
Q13200	26S proteasome non-ATPase regulatory subunit 2	PSMD2	1.359E+10	-2.30782
Q9BVG4	Protein PBDC1	PBDC1	370110000	-2.30721
Q96JJ3	Engulfment and cell motility protein 2	ELMO2	301460000	-2.30598
Q92538	Golgi-specific brefeldin A-resistance guanine nucle	GBF1	1.087E+09	-2.29233
Q9GZQ8;A6	Microtubule-associated proteins 1A/1B light chain	MAP1LC3B;MAP1L	512260000	-2.28479
P40939	Trifunctional enzyme subunit alpha, mitochondrial	HADHA	8.541E+09	-2.2837
Q99460	26S proteasome non-ATPase regulatory subunit 1	PSMD1	7.21E+09	-2.28318
Q9UBT7	Alpha-catulin	CTNNAL1	133700000	-2.2827
P55786	Puromycin-sensitive aminopeptidase	NPEPPS	7.054E+09	-2.27441
Q00610	Clathrin heavy chain 1	CLTC	7.501E+10	-2.26991
P14927	Cytochrome b-c1 complex subunit 7	UQCRB	386690000	-2.26746
Q08AF3	Schlafen family member 5	SLFN5	415960000	-2.26438
P35244	Replication protein A 14 kDa subunit	RPA3	389430000	-2.25988
Q9ULA0	Aspartyl aminopeptidase	DNPEP	1.107E+09	-2.25734
P05120	Plasminogen activator inhibitor 2	SERPINB2	982320000	-2.24831
Q9UL46	Proteasome activator complex subunit 2	PSME2	5.77E+09	-2.24562
Q9Y4R8	Telomere length regulation protein TEL2 homolog	TELO2	90010000	-2.24493
O95292	Vesicle-associated membrane protein-associated p	VAPB	1.12E+09	-2.2403

P40227	T-complex protein 1 subunit zeta	CCT6A	1.809E+10	-2.24019
P32455	Interferon-induced guanylate-binding protein 1	GBP1	296530000	-2.2313
P50213	Isocitrate dehydrogenase [NAD] subunit alpha, mit	IDH3A	2.907E+09	-2.22585
P05204;Q1	Non-histone chromosomal protein HMG-17;High n	HMGN2;HMGN3	357670000	-2.22007
Q99436	Proteasome subunit beta type-7	PSMB7	1.153E+09	-2.21523
P60981	Destrin	DSTN	893400000	-2.2089
P00390	Glutathione reductase, mitochondrial	GSR	781040000	-2.20794
P78559	Microtubule-associated protein 1A;MAP1A heavy c	MAP1A	4.141E+09	-2.20311
O43592	Exportin-T	XPOT	1.586E+09	-2.20043
Q9NWV8	BRISC and BRCA1-A complex member 1	BABAM1	176990000	-2.19776
Q9Y6D6	Brefeldin A-inhibited guanine nucleotide-exchange	ARFGEF1	96572000	-2.19518
O14662	Syntaxin-16	STX16	214470000	-2.19336
P49821	NADH dehydrogenase [ubiquinone] flavoprotein 1,	NDUFV1	1.04E+09	-2.19029
P55010	Eukaryotic translation initiation factor 5	EIF5	3.912E+09	-2.18982
P63000;P6(	Ras-related C3 botulinum toxin substrate 1;Ras-rel	RAC1;RAC3	1.6E+09	-2.18579
P49257	Protein ERGIC-53	LMAN1	1.195E+09	-2.18368
Q9NZ32	Actin-related protein 10	ACTR10	495030000	-2.18167
Q13011	Delta(3,5)-Delta(2,4)-dienoyl-CoA isomerase, mito	ECH1	1.417E+09	-2.17846
Q68E01	Integrator complex subunit 3	INTS3	311470000	-2.17816
P29373	Cellular retinoic acid-binding protein 2	CRABP2	295050000	-2.17777
Q06124	Tyrosine-protein phosphatase non-receptor type 1	PTPN11	836950000	-2.16652
P28331	NADH-ubiquinone oxidoreductase 75 kDa subunit,	NDUFS1	2.448E+09	-2.15827
Q13555	Calcium/calmodulin-dependent protein kinase typ	CAMK2G	166000000	-2.15283
Q2TAA2	Isoamyl acetate-hydrolyzing esterase 1 homolog	IAH1	607880000	-2.15009
Q9Y3Z3	Deoxynucleoside triphosphate triphosphohydrolas	SAMHD1	589020000	-2.14924
P49736	DNA replication licensing factor MCM2	MCM2	6.489E+09	-2.14322
Q5VTR2	E3 ubiquitin-protein ligase BRE1A	RNF20	612800000	-2.13499
P61981	14-3-3 protein gamma;14-3-3 protein gamma, N-te	YWHAG	5.555E+09	-2.12024
P21964	Catechol O-methyltransferase	COMT	1.136E+09	-2.11798
P22612	cAMP-dependent protein kinase catalytic subunit g	PRKACG	323640000	-2.11534
O75694	Nuclear pore complex protein Nup155	NUP155	1.189E+09	-2.11239
P55060	Exportin-2	CSE1L	1.732E+10	-2.11038
P04062	Glucosylceramidase	GBA	1.104E+09	-2.1101
O14880	Microsomal glutathione S-transferase 3	MGST3	836680000	-2.10943
P07711	Cathepsin L1;Cathepsin L1 heavy chain;Cathepsin L	CTSL	703190000	-2.10908
Q9BV44	THUMP domain-containing protein 3	THUMPD3	353860000	-2.10743
Q9H4M9	EH domain-containing protein 1	EHD1	8.361E+09	-2.10172
P55735	Protein SEC13 homolog	SEC13	2.086E+09	-2.09399
Q13148	TAR DNA-binding protein 43	TARDBP	2.45E+09	-2.09378
P11802	Cyclin-dependent kinase 4	CDK4	289160000	-2.09366
Q8WX92	Negative elongation factor B	NELFB	378020000	-2.09233
Q8IX04	Ubiquitin-conjugating enzyme E2 variant 3	UEVLD	383990000	-2.09069
Q9UNM6	26S proteasome non-ATPase regulatory subunit 13	PSMD13	5.02E+09	-2.08955
Q14318	Peptidyl-prolyl cis-trans isomerase FKBP8	FKBP8	504510000	-2.08927
P80723	Brain acid soluble protein 1	BASP1	759190000	-2.08137
P24539	ATP synthase F(0) complex subunit B1, mitochondi	ATP5F1	2.312E+09	-2.08121
P35606	Coatomer subunit beta	COPB2	3.417E+09	-2.07625
Q9NXG2	THUMP domain-containing protein 1	THUMPD1	466900000	-2.07021
O43776	Asparagine--tRNA ligase, cytoplasmic	NARS	1.925E+09	-2.06993
P46940	Ras GTPase-activating-like protein IQGAP1	IQGAP1	2.149E+10	-2.06792

Q15363	Transmembrane emp24 domain-containing protei	TMED2	2.621E+09	-2.06314
P33176	Kinesin-1 heavy chain	KIF5B	7.131E+09	-2.06012
P56537	Eukaryotic translation initiation factor 6	EIF6	1.649E+09	-2.05006
O15427	Monocarboxylate transporter 4	SLC16A3	130030000	-2.04618
Q9Y5L0	Transportin-3	TNPO3	2.073E+09	-2.04571
O60568	Procollagen-lysine,2-oxoglutarate 5-dioxygenase 3	PLOD3	342480000	-2.0417
Q9NYY8	FAST kinase domain-containing protein 2	FASTKD2	258590000	-2.02783
P13995	Bifunctional methylenetetrahydrofolate dehydroge	MTHFD2	519920000	-2.02469
P27695	DNA-(apurinic or apyrimidinic site) lyase;DNA-(apu	APEX1	3.206E+09	-2.02428
P21291	Cysteine and glycine-rich protein 1	CSRP1	1.071E+09	-2.02161
P22059	Oxysterol-binding protein 1	OSBP	389920000	-2.01984
P23528	Cofilin-1	CFL1	6.881E+10	-2.01968
P04439	HLA class I histocompatibility antigen, A-3 alpha ch	HLA-A	1.234E+09	-2.0159
P31949	Protein S100-A11;Protein S100-A11, N-terminally p	S100A11	778390000	-2.00585
Q9NQX3	Gephyrin;Molybdopterin adenylyltransferase;Moly	GPHN	225790000	-2.00306
Q9Y6E2	Basic leucine zipper and W2 domain-containing pro	BZW2	900260000	-1.99954
O75964	ATP synthase subunit g, mitochondrial	ATP5L	1.018E+09	-1.99925
P45880	Voltage-dependent anion-selective channel protei	VDAC2	3.459E+09	-1.99137
Q8WUM0	Nuclear pore complex protein Nup133	NUP133	301040000	-1.99032
Q53EP0	Fibronectin type III domain-containing protein 3B	FNDC3B	74397000	-1.9881
Q969Z0	Protein TBRG4	TBRG4	358160000	-1.98444
Q16880	2-hydroxyacylsphingosine 1-beta-galactosyltransfe	UGT8	346440000	-1.98348
O75369	Filamin-B	FLNB	8.305E+09	-1.98164
Q9HAV7	GrpE protein homolog 1, mitochondrial	GRPEL1	671310000	-1.97418
P42704	Leucine-rich PPR motif-containing protein, mitocho	LRPPRC	2.009E+10	-1.96888
P26641	Elongation factor 1-gamma	EEF1G	3.381E+10	-1.96857
P06576	ATP synthase subunit beta, mitochondrial	ATP5B	5.514E+10	-1.96536
P31153	S-adenosylmethionine synthase isoform type-2	MAT2A	1.792E+09	-1.9637
Q96P16	Regulation of nuclear pre-mRNA domain-containin	RPRD1A	844270000	-1.95158
P43246	DNA mismatch repair protein Msh2	MSH2	1.992E+09	-1.95154
Q9Y4Z0	U6 snRNA-associated Sm-like protein LSm4	LSM4	1.263E+09	-1.95146
O60841	Eukaryotic translation initiation factor 5B	EIF5B	2.965E+09	-1.94983
P13010	X-ray repair cross-complementing protein 5	XRCC5	1.928E+10	-1.9496
P68104;Q5	Elongation factor 1-alpha 1;Putative elongation fac	EEF1A1;EEF1A1P5	1.274E+11	-1.94932
O75396	Vesicle-trafficking protein SEC22b	SEC22B	2.616E+09	-1.94645
Q29RF7	Sister chromatid cohesion protein PDS5 homolog A	PDS5A	729270000	-1.94006
P53621	Coatomer subunit alpha;Xenin;Proxenin	COPA	9.566E+09	-1.93794
Q00341	Vigilin	HDLBP	6.246E+09	-1.93553
P13489	Ribonuclease inhibitor	RNH1	804630000	-1.935
O43396	Thioredoxin-like protein 1	TXNL1	563030000	-1.92979
Q9BPX3	Condensin complex subunit 3	NCAPG	1.487E+09	-1.9281
Q96CN7	Isochorismatase domain-containing protein 1	ISOC1	403880000	-1.92408
Q96DH6	RNA-binding protein Musashi homolog 2	MSI2	463510000	-1.92363
Q15043	Zinc transporter ZIP14	SLC39A14	354730000	-1.9219
P35232	Prohibitin	PHB	1.123E+10	-1.92026
P78406	mRNA export factor	RAE1	550240000	-1.91548
Q13442	28 kDa heat- and acid-stable phosphoprotein	PDAP1	1.327E+09	-1.91518
Q9H845	Acyl-CoA dehydrogenase family member 9, mitoch	ACAD9	1.915E+09	-1.91423
P53597	Succinyl-CoA ligase [ADP/GDP-forming] subunit alp	SUCLG1	854560000	-1.90581
P21980	Protein-glutamine gamma-glutamyltransferase 2	TGM2	1.162E+09	-1.90541

P07858	Cathepsin B;Cathepsin B light chain;Cathepsin B he	CTSB	2.124E+09	-1.90064
P25787	Proteasome subunit alpha type-2	PSMA2	6.36E+09	-1.89746
O00267	Transcription elongation factor SPT5	SUPT5H	360960000	-1.89546
P63010	AP-2 complex subunit beta	AP2B1	7.397E+09	-1.89245
P49368	T-complex protein 1 subunit gamma	CCT3	2.618E+10	-1.89195
Q9Y4K1	Absent in melanoma 1 protein	AIM1	1.033E+09	-1.89152
P43034	Platelet-activating factor acetylhydrolase IB subuni	PAFAH1B1	968990000	-1.88813
O60313	Dynamin-like 120 kDa protein, mitochondrial;Dyna	OPA1	1.399E+09	-1.88592
Q9UI12	V-type proton ATPase subunit H	ATP6V1H	1.364E+09	-1.88075
P49411	Elongation factor Tu, mitochondrial	TUFM	1.116E+10	-1.87802
Q92797	Symplekin	SYMPK	529540000	-1.87278
P41227	N-alpha-acetyltransferase 10	NAA10	901260000	-1.86924
Q13951	Core-binding factor subunit beta	CBFB	198250000	-1.8682
P51858	Hepatoma-derived growth factor	HDGF	4.186E+09	-1.86256
P11047	Laminin subunit gamma-1	LAMC1	305470000	-1.86173
Q9NRF9	DNA polymerase epsilon subunit 3	POLE3	739170000	-1.84537
P35080	Profilin-2	PFN2	1.292E+09	-1.84057
Q9Y6G9	Cytoplasmic dynein 1 light intermediate chain 1	DYNC1LI1	912260000	-1.83921
P17858	ATP-dependent 6-phosphofructokinase, liver type	PFKL	1.244E+09	-1.83823
P49721	Proteasome subunit beta type-2	PSMB2	2.154E+09	-1.8364
P11172	Uridine 5-monophosphate synthase;Orotate phosp	UMPS	1.375E+09	-1.83074
P61966	AP-1 complex subunit sigma-1A	AP1S1	132690000	-1.83072
O75534	Cold shock domain-containing protein E1	CSDE1	2.559E+09	-1.82656
Q99567	Nuclear pore complex protein Nup88	NUP88	153100000	-1.82334
Q8N183	Mimitin, mitochondrial	NDUFAF2	502500000	-1.81954
Q9NP72	Ras-related protein Rab-18	RAB18	945160000	-1.8186
O14744	Protein arginine N-methyltransferase 5;Protein arg	PRMT5	2.154E+09	-1.81551
Q9NVA2	Septin-11	Sep-11	1.934E+09	-1.80876
Q9P003	Protein cornichon homolog 4	CNIH4	371040000	-1.80857
P52630	Signal transducer and activator of transcription 2	STAT2	221270000	-1.80396
O75822	Eukaryotic translation initiation factor 3 subunit J	EIF3J	2.127E+09	-1.80349
P29966	Myristoylated alanine-rich C-kinase substrate	MARCKS	1.064E+09	-1.80312
Q15637	Splicing factor 1	SF1	2.589E+09	-1.80306
Q9H4A4	Aminopeptidase B	RNPEP	946840000	-1.80286
Q9UHB9	Signal recognition particle subunit SRP68	SRP68	4.058E+09	-1.79927
P22033	Methylmalonyl-CoA mutase, mitochondrial	MUT	578980000	-1.79833
P01130	Low-density lipoprotein receptor	LDLR	388310000	-1.79604
Q9NQT8	Kinesin-like protein KIF13B	KIF13B	157010000	-1.79053
P48047	ATP synthase subunit O, mitochondrial	ATP5O	4.917E+09	-1.78897
P21589	5-nucleotidase	NT5E	2.672E+09	-1.78696
P28066	Proteasome subunit alpha type-5	PSMA5	2.894E+09	-1.78515
Q02224	Centromere-associated protein E	CENPE	5.317E+10	-1.78244
Q9UQN3	Charged multivesicular body protein 2b	CHMP2B	86022000	-1.7767
Q13418	Integrin-linked protein kinase	ILK	2.258E+09	-1.77591
P03891	NADH-ubiquinone oxidoreductase chain 2	MT-ND2	105490000	-1.77528
Q9BPX5	Actin-related protein 2/3 complex subunit 5-like pr	ARPC5L	512640000	-1.77249
Q9Y2X7	ARF GTPase-activating protein GIT1	GIT1	313050000	-1.77076
Q96QD8	Sodium-coupled neutral amino acid transporter 2	SLC38A2	310880000	-1.77074
P55263	Adenosine kinase	ADK	1.126E+09	-1.77042
Q9BXP5	Serrate RNA effector molecule homolog	SRRT	1.146E+09	-1.76989

Q92896	Golgi apparatus protein 1	GLG1	149830000	-1.76922
Q9Y285	Phenylalanine--tRNA ligase alpha subunit	FARSA	3.432E+09	-1.76342
P25685	DnaJ homolog subfamily B member 1	DNAJB1	963510000	-1.75776
Q9Y508	E3 ubiquitin-protein ligase RNF114	RNF114	284150000	-1.75099
Q8TCE6;Q6	Protein FAM45A;Protein FAM45B	FAM45A;FAM45B	222500000	-1.74655
Q9NP74	Palmdelphin	PALMD	707870000	-1.73315
O75448	Mediator of RNA polymerase II transcription subur	MED24	24235000	-1.73225
O60343	TBC1 domain family member 4	TBC1D4	192300000	-1.72363
Q9BWM7	Sideroflexin-3	SFXN3	1.088E+09	-1.7136
P29692	Elongation factor 1-delta	EEF1D	1.226E+10	-1.71083
P61006	Ras-related protein Rab-8A	RAB8A	346850000	-1.71053
Q02952	A-kinase anchor protein 12	AKAP12	4.704E+09	-1.70761
Q9NSD9	Phenylalanine--tRNA ligase beta subunit	FARSB	3.042E+09	-1.70637
P33993	DNA replication licensing factor MCM7	MCM7	1.058E+10	-1.7042
O94925	Glutaminase kidney isoform, mitochondrial	GLS	1.286E+09	-1.70338
Q6IA69	Glutamine-dependent NAD(+) synthetase	NADSYN1	176670000	-1.70031
Q9UHQ4	B-cell receptor-associated protein 29	BCAP29	62420000	-1.69903
Q8NBJ7	Sulfatase-modifying factor 2	SUMF2	533440000	-1.69828
O00217	NADH dehydrogenase [ubiquinone] iron-sulfur pro	NDUFS8	241430000	-1.69649
P61009	Signal peptidase complex subunit 3	SPCS3	431310000	-1.69498
P60660	Myosin light polypeptide 6	MYL6	3.753E+09	-1.69238
Q9Y5M8	Signal recognition particle receptor subunit beta	SRPRB	1.021E+09	-1.69158
P78344	Eukaryotic translation initiation factor 4 gamma 2	EIF4G2	5.43E+09	-1.69111
P18031	Tyrosine-protein phosphatase non-receptor type 1	PTPN1	1.517E+09	-1.68965
Q969G3	SWI/SNF-related matrix-associated actin-depende	SMARCE1	339020000	-1.68606
O00442	RNA 3-terminal phosphate cyclase	RTCA	829450000	-1.68506
Q96JJ7	Protein disulfide-isomerase TMX3	TMX3	205040000	-1.68376
O43684	Mitotic checkpoint protein BUB3	BUB3	1.333E+09	-1.68288
Q9BYD1	39S ribosomal protein L13, mitochondrial	MRPL13	468660000	-1.67625
P49593	Protein phosphatase 1F	PPM1F	721680000	-1.67583
Q9BTV4	Transmembrane protein 43	TMEM43	823780000	-1.67101
Q9HCN8	Stromal cell-derived factor 2-like protein 1	SDF2L1	359200000	-1.67
P36969	Phospholipid hydroperoxide glutathione peroxidas	GPX4	168500000	-1.66955
Q13330	Metastasis-associated protein MTA1	MTA1	304200000	-1.66832
Q99584	Protein S100-A13	S100A13	158360000	-1.66699
P61224;A6I	Ras-related protein Rap-1b;Ras-related protein Ra	RAP1B	3.023E+09	-1.66508
Q9BY44	Eukaryotic translation initiation factor 2A;Eukaryot	EIF2A	1.209E+09	-1.66469
P43686	26S protease regulatory subunit 6B	PSMC4	8.491E+09	-1.66186
Q06830	Peroxiredoxin-1	PRDX1	2.814E+10	-1.65621
O43447	Peptidyl-prolyl cis-trans isomerase H	PPIH	344680000	-1.65051
O95373	Importin-7	IPO7	9.644E+09	-1.64651
O43148	mRNA cap guanine-N7 methyltransferase	RNMT	210080000	-1.64363
O43181	NADH dehydrogenase [ubiquinone] iron-sulfur pro	NDUFS4	193380000	-1.64056
Q8NCW5	NAD(P)H-hydrate epimerase	APOA1BP	443210000	-1.6366
P30622	CAP-Gly domain-containing linker protein 1	CLIP1	470880000	-1.63546
Q86W92	Liprin-beta-1	PPFIBP1	2.189E+09	-1.62997
Q9UBB6	Neurochondrin	NCDN	166960000	-1.62723
P10809	60 kDa heat shock protein, mitochondrial	HSPD1	9.791E+10	-1.62687
Q14254	Flotillin-2	FLOT2	229050000	-1.62454
P43490	Nicotinamide phosphoribosyltransferase	NAMPT	3.982E+10	-1.62453

Q9H490	Phosphatidylinositol glycan anchor biosynthesis cla	PIGU	142880000	-1.61646
O95573	Long-chain-fatty-acid--CoA ligase 3	ACSL3	832180000	-1.61567
Q9Y5S9	RNA-binding protein 8A	RBM8A	2.214E+09	-1.60908
Q9Y394	Dehydrogenase/reductase SDR family member 7	DHRS7	341320000	-1.60727
Q15417	Calponin-3	CNN3	768310000	-1.60661
O75947	ATP synthase subunit d, mitochondrial	ATP5H	824030000	-1.60507
P60891	Ribose-phosphate pyrophosphokinase 1	PRPS1	1.699E+09	-1.6029
P04899	Guanine nucleotide-binding protein G(i) subunit al	GNAI2	1.473E+09	-1.60164
P09132	Signal recognition particle 19 kDa protein	SRP19	81537000	-1.59729
P62195	26S protease regulatory subunit 8	PSMC5	7.439E+09	-1.59546
Q9H7D7	WD repeat-containing protein 26	WDR26	472650000	-1.59122
O00505	Importin subunit alpha-4	KPNA3	618830000	-1.59094
Q96HC4	PDZ and LIM domain protein 5	PDLIM5	965670000	-1.59075
Q8TCS8	Polyribonucleotide nucleotidyltransferase 1, mitoc	PNPT1	728700000	-1.59019
Q8NFH3	Nucleoporin Nup43	NUP43	131640000	-1.57837
O95782	AP-2 complex subunit alpha-1	AP2A1	3.991E+09	-1.57741
Q8TCU6	Phosphatidylinositol 3,4,5-trisphosphate-depende	PREX1	72325000	-1.57418
O00303	Eukaryotic translation initiation factor 3 subunit F	EIF3F	2.47E+09	-1.57402
Q9H223	EH domain-containing protein 4	EHD4	404350000	-1.57132
Q92620	Pre-mRNA-splicing factor ATP-dependent RNA heli	DHX38	502660000	-1.56494
Q12888	Tumor suppressor p53-binding protein 1	TP53BP1	692970000	-1.55906
P63151	Serine/threonine-protein phosphatase 2A 55 kDa r	PPP2R2A	1.515E+09	-1.55789
P49354	Protein farnesyltransferase/geranylgeranyltransfe	FNTA	450250000	-1.5569
P25705	ATP synthase subunit alpha, mitochondrial	ATP5A1	3.074E+10	-1.55385
Q9UKK3	Poly [ADP-ribose] polymerase 4	PARP4	294110000	-1.54875
Q8N0X7	Spartin	SPG20	344060000	-1.54343
Q96FQ6	Protein S100-A16	S100A16	160490000	-1.54135
Q5T4S7	E3 ubiquitin-protein ligase UBR4	UBR4	2.29E+09	-1.54048
Q14789	Golgin subfamily B member 1	GOLGB1	588940000	-1.53843
P33992	DNA replication licensing factor MCM5	MCM5	7.97E+09	-1.53618
O00487	26S proteasome non-ATPase regulatory subunit 14	PSMD14	1.924E+09	-1.53331
Q02750	Dual specificity mitogen-activated protein kinase k	MAP2K1	1.345E+09	-1.53021
Q8TC07	TBC1 domain family member 15	TBC1D15	327520000	-1.52846
Q9Y4I1	Unconventional myosin-Va	MYO5A	592000000	-1.52769
P28072	Proteasome subunit beta type-6	PSMB6	3.1E+09	-1.51729
Q09161	Nuclear cap-binding protein subunit 1	NCBP1	476290000	-1.51676
Q8TAT6	Nuclear protein localization protein 4 homolog	NPLOC4	1.486E+09	-1.51549
Q9BQA1	Methylosome protein 50	WDR77	1.239E+09	-1.51452
P62879	Guanine nucleotide-binding protein G(I)/G(S)/G(T)	GNB2	2.134E+09	-1.5057
Q8IVD9	NudC domain-containing protein 3	NUDCD3	287140000	-1.50409
O95757	Heat shock 70 kDa protein 4L	HSPA4L	1.486E+09	-1.50269
P35611	Alpha-adducin	ADD1	571720000	-1.5016
Q9BT17	Mitochondrial ribosome-associated GTPase 1	MTG1	179930000	-1.49688
Q96N66	Lysophospholipid acyltransferase 7	MBOAT7	432750000	-1.49107
P49757	Protein numb homolog	NUMB	170960000	-1.4875
O75348;O9	V-type proton ATPase subunit G 1;V-type proton A	ATP6V1G1;ATP6V1	828740000	-1.48401
Q14764	Major vault protein	MVP	2.223E+10	-1.48242
Q96SU4	Oxysterol-binding protein-related protein 9	OSBPL9	235090000	-1.4785
Q99720	Sigma non-opioid intracellular receptor 1	SIGMAR1	636840000	-1.47127
P50613	Cyclin-dependent kinase 7	CDK7	91912000	-1.47093

O75326	Semaphorin-7A	SEMA7A	225510000	-1.47082
Q9NW15	Anoctamin-10	ANO10	167200000	-1.46919
Q99816	Tumor susceptibility gene 101 protein	TSG101	429540000	-1.46539
Q9HD45	Transmembrane 9 superfamily member 3	TM9SF3	360840000	-1.46085
O94822	E3 ubiquitin-protein ligase listerin	LTN1	400680000	-1.45866
P06396	Gelsolin	GSN	3.036E+09	-1.45757
P19387	DNA-directed RNA polymerase II subunit RPB3	POLR2C	610600000	-1.45634
Q9UQE7	Structural maintenance of chromosomes protein 3	SMC3	1.569E+09	-1.45565
O14981	TATA-binding protein-associated factor 172	BTAF1	80294000	-1.45489
Q9BZF1	Oxysterol-binding protein-related protein 8	OSBPL8	354000000	-1.45367
P05023	Sodium/potassium-transporting ATPase subunit al	ATP1A1	1.087E+10	-1.45364
A1L0T0	Acetolactate synthase-like protein	ILVBL	700610000	-1.4526
P13807	Glycogen [starch] synthase, muscle	GYS1	644430000	-1.44969
P40222	Alpha-taxilin	TXLNA	1.707E+09	-1.44479
P20073	Annexin A7	ANXA7	990030000	-1.44432
Q969V3	Nicalin	NCLN	474270000	-1.44056
P10644	cAMP-dependent protein kinase type I-alpha regul	PRKAR1A	1.94E+09	-1.4359
P09382	Galectin-1	LGALS1	3.803E+09	-1.43471
Q8WVM8	Sec1 family domain-containing protein 1	SCFD1	2.376E+09	-1.43452
P63172	Dynein light chain Tctex-type 1	DYNLT1	432260000	-1.42046
P62993	Growth factor receptor-bound protein 2	GRB2	649820000	-1.41773
Q8IZL8	Proline-, glutamic acid- and leucine-rich protein 1	PELP1	260620000	-1.41768
Q15645	Pachytene checkpoint protein 2 homolog	TRIP13	1.278E+09	-1.4014
Q9HB40	Retinoid-inducible serine carboxypeptidase	SCPEP1	92351000	-1.39921
P36542	ATP synthase subunit gamma, mitochondrial	ATP5C1	2.433E+09	-1.39879
P46108	Adapter molecule crk	CRK	484610000	-1.39557
Q13895	Bystin	BYSL	181850000	-1.39139
O95202	LETM1 and EF-hand domain-containing protein 1, r	LETM1	1.381E+09	-1.39092
Q9UKM7	Endoplasmic reticulum mannosyl-oligosaccharide 1	MAN1B1	140360000	-1.38517
O00148	ATP-dependent RNA helicase DDX39A	DDX39A	1.535E+09	-1.38199
A6NDG6	Phosphoglycolate phosphatase	PGP	251790000	-1.37809
Q8NBX0	Saccharopine dehydrogenase-like oxidoreductase	SCCPDH	537070000	-1.37614
Q9Y2S7	Polymerase delta-interacting protein 2	POLDIP2	1.114E+09	-1.37525
O75380	NADH dehydrogenase [ubiquinone] iron-sulfur pro	NDUFS6	76361000	-1.37523
Q9NZN4	EH domain-containing protein 2	EHD2	278050000	-1.37305
Q14315	Filamin-C	FLNC	4.838E+10	-1.37162
P35998	26S protease regulatory subunit 7	PSMC2	7.249E+09	-1.36965
Q14738	Serine/threonine-protein phosphatase 2A 56 kDa r	PPP2R5D	462860000	-1.36535
P55072	Transitional endoplasmic reticulum ATPase	VCP	2.639E+10	-1.36255
Q13190	Syntaxin-5	STX5	394730000	-1.36239
P00846	ATP synthase subunit a	MT-ATP6	329710000	-1.35866
P49720	Proteasome subunit beta type-3	PSMB3	3.679E+09	-1.35587
P24666	Low molecular weight phosphotyrosine protein ph	ACP1	446340000	-1.35319
Q9UBQ5	Eukaryotic translation initiation factor 3 subunit K	EIF3K	603760000	-1.35201
Q9H3U1	Protein unc-45 homolog A	UNC45A	1.657E+09	-1.35078
Q969T3	Sorting nexin-21	SNX21	64898000	-1.3499
P30419	Glycylpeptide N-tetradecanoyltransferase 1	NMT1	1.623E+09	-1.34756
O94808	Glutamine--fructose-6-phosphate aminotransferas	GFPT2	7.714E+09	-1.3465
P56192	Methionine--tRNA ligase, cytoplasmic	MARS	4.332E+09	-1.34183
P24534	Elongation factor 1-beta	EEF1B2	1.41E+10	-1.33977

P05121	Plasminogen activator inhibitor 1	SERPINE1	581490000	-1.33496
P51571	Translocon-associated protein subunit delta	SSR4	1.63E+09	-1.33247
Q9Y3F4	Serine-threonine kinase receptor-associated prote	STRAP	4.026E+09	-1.32314
Q6UX53	Methyltransferase-like protein 7B	METTL7B	101710000	-1.32256
P28161	Glutathione S-transferase Mu 2	GSTM2	749940000	-1.3213
P11166	Solute carrier family 2, facilitated glucose transpor	SLC2A1	608160000	-1.31866
P39656	Dolichyl-diphosphooligosaccharide--protein glycos	DDOST	6.111E+09	-1.31865
P53999	Activated RNA polymerase II transcriptional coacti	SUB1	4.001E+09	-1.31845
P35249	Replication factor C subunit 4	RFC4	1.094E+09	-1.30711
P11234	Ras-related protein Ral-B	RALB	572140000	-1.30486
P12956	X-ray repair cross-complementing protein 6	XRCC6	2.501E+10	-1.29753
P21333	Filamin-A	FLNA	4.907E+10	-1.29647
P62333	26S protease regulatory subunit 10B	PSMC6	8.175E+09	-1.29595
Q8IYT4	Katanin p60 ATPase-containing subunit A-like 2	KATNAL2	818470000	-1.29459
Q9BY32	Inosine triphosphate pyrophosphatase	ITPA	297250000	-1.2938
P11279	Lysosome-associated membrane glycoprotein 1	LAMP1	1.405E+09	-1.29202
Q14677	Clathrin interactor 1	CLINT1	438760000	-1.29134
Q96A33	Coiled-coil domain-containing protein 47	CCDC47	1.154E+09	-1.29048
Q9Y305	Acyl-coenzyme A thioesterase 9, mitochondrial	ACOT9	491360000	-1.28937
Q07866	Kinesin light chain 1	KLC1	2.275E+09	-1.28846
Q16222	UDP-N-acetylhexosamine pyrophosphorylase;UDP	UAP1	272040000	-1.28544
O60711	Leupaxin	LPXN	865400000	-1.28537
Q9NZJ7	Mitochondrial carrier homolog 1	MTCH1	71462000	-1.28405
P25205	DNA replication licensing factor MCM3	MCM3	7.633E+09	-1.2828
Q92621	Nuclear pore complex protein Nup205	NUP205	982040000	-1.27856
Q6P2E9	Enhancer of mRNA-decapping protein 4	EDC4	1.815E+09	-1.27488
O95817	BAG family molecular chaperone regulator 3	BAG3	235720000	-1.27344
P10515	Dihydrolipoyllysine-residue acetyltransferase comp	DLAT	1.405E+09	-1.26922
P50416	Carnitine O-palmitoyltransferase 1, liver isoform	CPT1A	1.128E+09	-1.26804
Q15386	Ubiquitin-protein ligase E3C	UBE3C	323820000	-1.26593
Q15291	Retinoblastoma-binding protein 5	RBBP5	49942000	-1.26542
Q9UGP8	Translocation protein SEC63 homolog	SEC63	703700000	-1.2654
P62714;P67	Serine/threonine-protein phosphatase 2A catalytic	PPP2CB;PPP2CA	2.709E+09	-1.26383
P35269	General transcription factor IIF subunit 1	GTF2F1	440630000	-1.26242
P11908	Ribose-phosphate pyrophosphokinase 2	PRPS2	553760000	-1.26115
P63244	Guanine nucleotide-binding protein subunit beta-2	GNB2L1	1.17E+10	-1.25673
Q96A26	Protein FAM162A	FAM162A	464820000	-1.25377
Q15392	Delta(24)-sterol reductase	DHCR24	110280000	-1.2506
Q9UJ70	N-acetyl-D-glucosamine kinase	NAGK	1.142E+09	-1.25052
Q9BW92	Threonine--tRNA ligase, mitochondrial	TARS2	549220000	-1.24968
Q99543	DnaJ homolog subfamily C member 2;DnaJ homolo	DNAJC2	233340000	-1.24543
Q15021	Condensin complex subunit 1	NCAPD2	2.171E+09	-1.24019
Q8N3D4	EH domain-binding protein 1-like protein 1	EHBP1L1	481250000	-1.23691
Q9Y4W6	AFG3-like protein 2	AFG3L2	1.053E+09	-1.2359
Q9ULE6	Paladin	PALD1	742540000	-1.23479
Q9Y3B8	Oligoribonuclease, mitochondrial	REXO2	417390000	-1.23476
Q9UI30	Multifunctional methyltransferase subunit TRM11	TRMT112	377670000	-1.23443
Q96IX5	Up-regulated during skeletal muscle growth protei	USMG5	560880000	-1.23414
P09012	U1 small nuclear ribonucleoprotein A	SNRPA	1.216E+09	-1.2338
O00231	26S proteasome non-ATPase regulatory subunit 11	PSMD11	6.042E+09	-1.2278

Q9NRG9	Aladin	AAAS	183210000	-1.22557
P61421	V-type proton ATPase subunit d 1	ATP6V0D1	61538000	-1.21781
Q53H96	Pyrroline-5-carboxylate reductase 3	PYCRL	135600000	-1.21087
O75616	GTPase Era, mitochondrial	ERAL1	225930000	-1.20878
O75410	Transforming acidic coiled-coil-containing protein :	TACC1	30970000	-1.20763
Q16537	Serine/threonine-protein phosphatase 2A 56 kDa r	PPP2R5E	735650000	-1.20275
Q9UII2	ATPase inhibitor, mitochondrial	ATPIF1	469840000	-1.20033
Q9Y316	Protein MEMO1	MEMO1	907900000	-1.19883
P07686	Beta-hexosaminidase subunit beta;Beta-hexosami	HEXB	2.035E+09	-1.19778
P27708	CAD protein;Glutamine-dependent carbamoyl-pho	CAD	1.021E+10	-1.1953
P00387	NADH-cytochrome b5 reductase 3;NADH-cytochro	CYB5R3	4.473E+09	-1.18965
P51970	NADH dehydrogenase [ubiquinone] 1 alpha subcor	NDUFA8	1.013E+09	-1.18904
P49406	39S ribosomal protein L19, mitochondrial	MRPL19	332080000	-1.1882
P51665	26S proteasome non-ATPase regulatory subunit 7	PSMD7	3.118E+09	-1.1875
O60684;O1	Importin subunit alpha-7;Importin subunit alpha-6	KPNA6;KPNA5	981460000	-1.18035
Q658Y4	Protein FAM91A1	FAM91A1	291070000	-1.17854
O75531	Barrier-to-autointegration factor;Barrier-to-autoin	BANF1	506540000	-1.17771
Q8N6M0	OTU domain-containing protein 6B	OTUD6B	134120000	-1.17625
Q92609	TBC1 domain family member 5	TBC1D5	377400000	-1.17428
Q09028	Histone-binding protein RBBP4	RBBP4	3.909E+09	-1.17247
Q9BR76	Coronin-1B	CORO1B	2.267E+09	-1.1704
P13796	Plastin-2	LCP1	180620000	-1.16877
Q9BUK6	Protein misato homolog 1	MSTO1	120820000	-1.1665
Q99623	Prohibitin-2	PHB2	1.203E+10	-1.16438
P53680	AP-2 complex subunit sigma	AP2S1	279760000	-1.16239
O43172	U4/U6 small nuclear ribonucleoprotein Prp4	PRPF4	290800000	-1.16194
Q99470	Stromal cell-derived factor 2	SDF2	117310000	-1.16074
Q14204	Cytoplasmic dynein 1 heavy chain 1	DYNC1H1	6.145E+10	-1.15714
Q9NXH9	tRNA (guanine(26)-N(2))-dimethyltransferase	TRMT1	263570000	-1.1543
Q9GZM5	Protein YIPF3;Protein YIPF3, 36 kDa form III	YIPF3	135020000	-1.15395
P43155	Carnitine O-acetyltransferase	CRAT	162650000	-1.1523
P05455	Lupus La protein	SSB	7.417E+09	-1.15159
P47985;P0(	Cytochrome b-c1 complex subunit Rieske, mitocho	UQCRFS1;UQCRFS	1.667E+09	-1.14961
P28070	Proteasome subunit beta type-4	PSMB4	5.123E+09	-1.14216
P20618	Proteasome subunit beta type-1	PSMB1	4.893E+09	-1.14204
O00264	Membrane-associated progesterone receptor com	PGRMC1	402490000	-1.14135
Q6RW13	Type-1 angiotensin II receptor-associated protein	AGTRAP	215630000	-1.14078
Q15003	Condensin complex subunit 2	NCAPH	669940000	-1.14052
Q16795	NADH dehydrogenase [ubiquinone] 1 alpha subcor	NDUFA9	1.577E+09	-1.13751
Q9NP97	Dynein light chain roadblock-type 1	DYNLRB1	273770000	-1.13402
O14980	Exportin-1	XPO1	9.044E+09	-1.13226
Q96RE7	Nucleus accumbens-associated protein 1	NACC1	76208000	-1.12807
P05067	Amyloid beta A4 protein;N-APP;Soluble APP-alpha	APP	17893000	-1.12549
P50748	Kinetochore-associated protein 1	KNTC1	163140000	-1.1192
Q6Y7W6	PERQ amino acid-rich with GYF domain-containing	GIGYF2	862790000	-1.11726
P51572	B-cell receptor-associated protein 31	BCAP31	2.206E+09	-1.11599
Q9HCC0	Methylcrotonoyl-CoA carboxylase beta chain, mito	MCCC2	644180000	-1.11594
Q9Y5S2	Serine/threonine-protein kinase MRCK beta	CDC42BPB	441920000	-1.11121
Q8TEA8	D-tyrosyl-tRNA(Tyr) deacylase 1	DTD1	147180000	-1.11016
P48059	LIM and senescent cell antigen-like-containing dom	LIMS1	194750000	-1.10556

Q7L014	Probable ATP-dependent RNA helicase DDX46	DDX46	1.456E+09	-1.10209
Q9BW27	Nuclear pore complex protein Nup85	NUP85	193100000	-1.10117
P53367	Arfaptin-1	ARFIP1	484560000	-1.09674
Q9BRP1	Programmed cell death protein 2-like	PDCD2L	250780000	-1.0948
O75251	NADH dehydrogenase [ubiquinone] iron-sulfur pro	NDUFS7	453570000	-1.09372
Q5H9R7	Serine/threonine-protein phosphatase 6 regulator	PPP6R3	894000000	-1.09022
P12109	Collagen alpha-1(VI) chain	COL6A1	206900000	-1.08627
Q9NXF1	Testis-expressed sequence 10 protein	TEX10	220760000	-1.0859
P04350	Tubulin beta-4A chain	TUBB4A	1.144E+11	-1.08578
P25788	Proteasome subunit alpha type-3	PSMA3	2.605E+09	-1.07872
O75676	Ribosomal protein S6 kinase alpha-4	RPS6KA4	33136000	-1.07852
P01889	HLA class I histocompatibility antigen, B-7 alpha ch	HLA-B	110970000	-1.071
P23588	Eukaryotic translation initiation factor 4B	EIF4B	1.883E+09	-1.06798
Q9H6T3	RNA polymerase II-associated protein 3	RPAP3	460720000	-1.06476
Q8WWY3	U4/U6 small nuclear ribonucleoprotein Prp31	PRPF31	776620000	-1.05886
P60900	Proteasome subunit alpha type-6	PSMA6	2.622E+09	-1.05511
Q9Y312	Protein AAR2 homolog	AAR2	352830000	-1.05071
P40616	ADP-ribosylation factor-like protein 1	ARL1	1.185E+09	-1.04897
Q9UNF0	Protein kinase C and casein kinase substrate in neu	PACSIN2	693090000	-1.04678
Q92696	Geranylgeranyl transferase type-2 subunit alpha	RABGGTA	794020000	-1.04428
Q99805	Transmembrane 9 superfamily member 2	TM9SF2	596700000	-1.04086
P52789	Hexokinase-2	HK2	1.881E+09	-1.04031
Q9UHG3	Prenylcysteine oxidase 1	PCYOX1	492910000	-1.03597
Q9BZK7	F-box-like/WD repeat-containing protein TBL1XR1	TBL1XR1	212050000	-1.03545
Q99538	Legumain	LGMN	417410000	-1.03476
P49770	Translation initiation factor eIF-2B subunit beta	EIF2B2	869490000	-1.03303
Q10471	Polypeptide N-acetylgalactosaminyltransferase 2;F	GALNT2	722040000	-1.03267
Q15276	Rab GTPase-binding effector protein 1	RABEP1	247920000	-1.03262
Q99439	Calponin-2	CNN2	708990000	-1.02541
P51659	Peroxisomal multifunctional enzyme type 2;(3R)-hy	HSD17B4	2.172E+09	-1.01585
O15270	Serine palmitoyltransferase 2	SPTLC2	318320000	-1.01336
P62308;A8I	Small nuclear ribonucleoprotein G;Putative small n	SNRPG;SNRPGP15	1.257E+09	-1.00563
Q09666	Neuroblast differentiation-associated protein AHN	AHNAK	5.59E+10	-1.00372
Q02218	2-oxoglutarate dehydrogenase, mitochondrial	OGDH	2.393E+09	-1.0026
P46063	ATP-dependent DNA helicase Q1	RECQL	2.237E+09	-1.00117
Q14728	Major facilitator superfamily domain-containing pr	MFSD10	57950000	-1.00051
Q9UIC8	Leucine carboxyl methyltransferase 1	LCMT1	432390000	-0.99714
P54652	Heat shock-related 70 kDa protein 2	HSPA2	151020000	-0.996
Q6NUK1	Calcium-binding mitochondrial carrier protein SCaI	SLC25A24	525930000	-0.99141
Q13671	Ras and Rab interactor 1	RIN1	413180000	-0.99107
P63167;Q9	Dynein light chain 1, cytoplasmic;Dynein light chair	DYNLL1;DYNLL2	353500000	-0.9773
Q969F1	General transcription factor 3C polypeptide 6	GTF3C6	163910000	-0.97262
P98194	Calcium-transporting ATPase type 2C member 1	ATP2C1	52988000	-0.9716
P61764	Syntaxin-binding protein 1	STXBP1	1.256E+09	-0.97059
Q96M27	Protein PRRC1	PRRC1	562590000	-0.97
Q9NPQ8	Synembryn-A	RIC8A	895440000	-0.96871
O14818	Proteasome subunit alpha type-7	PSMA7	5.864E+09	-0.96639
Q04637	Eukaryotic translation initiation factor 4 gamma 1	EIF4G1	1.483E+10	-0.9659
Q10469	Alpha-1,6-mannosyl-glycoprotein 2-beta-N-acetylg	MGAT2	99790000	-0.96413
Q53H12	Acylglycerol kinase, mitochondrial	AGK	2.526E+09	-0.96346

Q9BRJ2	39S ribosomal protein L45, mitochondrial	MRPL45	345630000	-0.95093
P25789	Proteasome subunit alpha type-4	PSMA4	2.268E+09	-0.94598
Q9GZT3	SRA stem-loop-interacting RNA-binding protein, m	SLIRP	911780000	-0.94487
Q96JM3	Chromosome alignment-maintaining phosphoprot	CHAMP1	115000000	-0.94175
Q9NR46	Endophilin-B2	SH3GLB2	167710000	-0.94082
Q16186	Proteasomal ubiquitin receptor ADRM1	ADRM1	906140000	-0.93866
Q8TB36	Ganglioside-induced differentiation-associated pro	GDAP1	45867000	-0.93791
P30530	Tyrosine-protein kinase receptor UFO	AXL	23382000	-0.93687
Q99570	Phosphoinositide 3-kinase regulatory subunit 4	PIK3R4	155800000	-0.93658
O75351	Vacuolar protein sorting-associated protein 4B	VPS4B	134110000	-0.93131
P35221	Catenin alpha-1	CTNNA1	467260000	-0.92679
Q9NVA1	Ubiquinol-cytochrome-c reductase complex assem	UQCC1	176970000	-0.92628
P56385	ATP synthase subunit e, mitochondrial	ATP5I	336080000	-0.92297
O43819	Protein SCO2 homolog, mitochondrial	SCO2	155470000	-0.92139
Q9NUM3	Zinc transporter ZIP9	SLC39A9	11939000	-0.92063
Q7L523;Q5	Ras-related GTP-binding protein A;Ras-related GTF	RRAGA;RRAGB	225050000	-0.91974
Q5J8M3	ER membrane protein complex subunit 4	EMC4	167140000	-0.91579
Q14203	Dynactin subunit 1	DCTN1	4.44E+09	-0.9152
P05198	Eukaryotic translation initiation factor 2 subunit 1	EIF2S1	7.071E+09	-0.90274
Q15334	Lethal(2) giant larvae protein homolog 1	LLGL1	109390000	-0.90155
Q05048	Cleavage stimulation factor subunit 1	CSTF1	327190000	-0.90055
O60885	Bromodomain-containing protein 4	BRD4	121780000	-0.89623
Q9GZS3	WD repeat-containing protein 61;WD repeat-conta	WDR61	414510000	-0.89214
P57737	Coronin-7	CORO7	363520000	-0.89212
P07384	Calpain-1 catalytic subunit	CAPN1	4.366E+09	-0.89118
P62273	40S ribosomal protein S29	RPS29	885610000	-0.88854
Q7L2H7	Eukaryotic translation initiation factor 3 subunit M	EIF3M	4.94E+09	-0.88803
Q9NQG5	Regulation of nuclear pre-mRNA domain-containin	RPRD1B	1.003E+09	-0.88367
Q9BRT2	Ubiquinol-cytochrome-c reductase complex assem	UQCC2	255830000	-0.88275
Q9Y6C9	Mitochondrial carrier homolog 2	MTCH2	766960000	-0.8827
Q9Y512	Sorting and assembly machinery component 50 ho	SAMM50	308540000	-0.88207
Q969X5	Endoplasmic reticulum-Golgi intermediate compar	ERGIC1	500180000	-0.8792
Q71U36;P0	Tubulin alpha-1A chain;Tubulin alpha-3E chain	TUBA1A;TUBA3E	806080000	-0.8759
Q9H061	Transmembrane protein 126A	TMEM126A	146330000	-0.87269
Q9Y2V7	Conserved oligomeric Golgi complex subunit 6	COG6	192940000	-0.87263
Q9P258	Protein RCC2	RCC2	3.25E+09	-0.86616
Q9P2J5	Leucine--tRNA ligase, cytoplasmic	LARS	9.655E+09	-0.86428
Q9UG63	ATP-binding cassette sub-family F member 2	ABCF2	1.547E+09	-0.86353
P52292	Importin subunit alpha-1	KPNA2	3.897E+09	-0.86341
Q9NQ48	Leucine zipper transcription factor-like protein 1	LZTFL1	291170000	-0.85933
Q9NYB0	Telomeric repeat-binding factor 2-interacting prot	TERF2IP	231540000	-0.85461
Q969G5	Protein kinase C delta-binding protein	PRKCDBP	347540000	-0.85298
Q9Y2R5	28S ribosomal protein S17, mitochondrial	MRPS17	172280000	-0.85274
P35241	Radixin	RDX	757130000	-0.8518
P98179	RNA-binding protein 3	RBM3	1.708E+09	-0.84942
Q13438	Protein OS-9	OS9	322080000	-0.84824
P60059	Protein transport protein Sec61 subunit gamma	SEC61G	691370000	-0.84756
O75489	NADH dehydrogenase [ubiquinone] iron-sulfur pro	NDUFS3	2.482E+09	-0.84117
O15258	Protein RER1	RER1	757860000	-0.8404
Q13322	Growth factor receptor-bound protein 10	GRB10	121080000	-0.8397

Q13409	Cytoplasmic dynein 1 intermediate chain 2	DYNC1I2		2.388E+09	-0.83482
Q14103	Heterogeneous nuclear ribonucleoprotein D0	HNRNPD		6.653E+09	-0.83432
Q9BTE1	Dynactin subunit 5	DCTN5		140700000	-0.83112
Q8WWM7	Ataxin-2-like protein	ATXN2L		1.068E+09	-0.82653
Q6PJG6	BRCA1-associated ATM activator 1	BRAT1		143650000	-0.81782
O14828	Secretory carrier-associated membrane protein 3	SCAMP3		1.007E+09	-0.81678
Q9UEW8	STE20/SPS1-related proline-alanine-rich protein ki	STK39		327540000	-0.81429
Q9Y6M7	Sodium bicarbonate cotransporter 3	SLC4A7		566650000	-0.81404
P23368	NAD-dependent malic enzyme, mitochondrial	ME2		868270000	-0.81114
P08865	40S ribosomal protein SA	RPSA		9.036E+09	-0.80996
Q9UJW0	Dynactin subunit 4	DCTN4		358390000	-0.80922
Q14919	Dr1-associated corepressor	DRAP1		342740000	-0.80886
Q96FX7	tRNA (adenine(58)-N(1))-methyltransferase catalyt	TRMT61A		151010000	-0.80859
Q92599	Septin-8		Sep-08	119650000	-0.80851
O96000	NADH dehydrogenase [ubiquinone] 1 beta subcom	NDUFB10		428090000	-0.79828
P62310	U6 snRNA-associated Sm-like protein LSm3	LSM3		68822000	-0.79736
Q96A65	Exocyst complex component 4	EXOC4		624150000	-0.79656
Q5VWZ2	Lysophospholipase-like protein 1	LYPLAL1		269010000	-0.79517
Q13405	39S ribosomal protein L49, mitochondrial	MRPL49		79903000	-0.79157
Q9UHV9	Prefoldin subunit 2	PFDN2		359220000	-0.78868
Q9Y4P1	Cysteine protease ATG4B	ATG4B		305210000	-0.78808
P08574	Cytochrome c1, heme protein, mitochondrial	CYC1		1.038E+09	-0.78602
P55036	26S proteasome non-ATPase regulatory subunit 4	PSMD4		3.036E+09	-0.78321
P30533	Alpha-2-macroglobulin receptor-associated protei	LRPAP1		517300000	-0.78028
Q8N357	Solute carrier family 35 member F6	SLC35F6		19190000	-0.777
P35610	Sterol O-acyltransferase 1	SOAT1		1.045E+09	-0.77133
O14579	Coatomer subunit epsilon	COPE		1.321E+09	-0.77043
Q13131	5-AMP-activated protein kinase catalytic subunit a	PRKAA1		312440000	-0.76867
Q6P996	Pyridoxal-dependent decarboxylase domain-conta	PDXDC1		444390000	-0.76518
P42285	Superkiller viralicidic activity 2-like 2	SKIV2L2		1.322E+09	-0.76418
Q96EP5	DAZ-associated protein 1	DAZAP1		1.375E+09	-0.7623
P51532	Transcription activator BRG1	SMARCA4		628490000	-0.76121
P07602	Prosaposin;Saposin-A;Saposin-B-Val;Saposin-B;Sap	PSAP		54197000	-0.76059
Q9UPN3	Microtubule-actin cross-linking factor 1, isoforms 1	MACF1		1.535E+09	-0.759
P35579	Myosin-9	MYH9		1.703E+11	-0.75817
P07437	Tubulin beta chain	TUBB		4.728E+10	-0.75744
P15927	Replication protein A 32 kDa subunit	RPA2		1.275E+09	-0.7563
Q9Y6A9	Signal peptidase complex subunit 1	SPCS1		203200000	-0.75452
O14617	AP-3 complex subunit delta-1	AP3D1		2.524E+09	-0.75223
P55209	Nucleosome assembly protein 1-like 1	NAP1L1		1.631E+10	-0.74721
Q7L0Y3	Mitochondrial ribonuclease P protein 1	TRMT10C		508700000	-0.74569
P60709	Actin, cytoplasmic 1;Actin, cytoplasmic 1, N-termir	ACTB		4.922E+09	-0.74466
Q9NQ50	39S ribosomal protein L40, mitochondrial	MRPL40		351320000	-0.73701
Q86Y56	Dynein assembly factor 5, axonemal	DNAAF5		993780000	-0.73021
Q9GZP4	PITH domain-containing protein 1	PITHD1		290560000	-0.72965
Q3ZCQ8	Mitochondrial import inner membrane translocase	TIMM50		1.588E+09	-0.72864
Q9BYD3	39S ribosomal protein L4, mitochondrial	MRPL4		911790000	-0.72785
Q9UBD5	Origin recognition complex subunit 3	ORC3		69871000	-0.72361
Q9UPT5	Exocyst complex component 7	EXOC7		275330000	-0.7231
Q9Y4J8	Dystrobrevin alpha	DTNA		126520000	-0.72247

Q9H444	Charged multivesicular body protein 4b	CHMP4B	918940000	-0.71895
Q8WVV9	Heterogeneous nuclear ribonucleoprotein L-like	HNRNPLL	127870000	-0.7177
Q13347	Eukaryotic translation initiation factor 3 subunit I	EIF3I	4.481E+09	-0.71694
P04920	Anion exchange protein 2	SLC4A2	127900000	-0.71659
Q14573	Inositol 1,4,5-trisphosphate receptor type 3	ITPR3	63540000	-0.71536
Q9UL15	BAG family molecular chaperone regulator 5	BAG5	898050000	-0.71506
Q9C0E8	Protein lunapark	LNP	134220000	-0.71153
O14776	Transcription elongation regulator 1	TCERG1	342100000	-0.7089
P50281	Matrix metalloproteinase-14	MMP14	465020000	-0.70675
O60645	Exocyst complex component 3	EXOC3	223830000	-0.70227
Q9UDW1	Cytochrome b-c1 complex subunit 9	UQCR10	762950000	-0.7008
Q86Y39	NADH dehydrogenase [ubiquinone] 1 alpha subcor	NDUFA11	82936000	-0.69782
Q96IU4	Alpha/beta hydrolase domain-containing protein 1	ABHD14B	160690000	-0.69601
Q9HBI1	Beta-parvin	PARVB	535790000	-0.69487
Q9NYB9	Abl interactor 2	ABI2	32609000	-0.69434
Q99653	Calcineurin B homologous protein 1	CHP1	215150000	-0.69395
Q14152	Eukaryotic translation initiation factor 3 subunit A	EIF3A	1.543E+10	-0.68617
Q9BVC6	Transmembrane protein 109	TMEM109	749950000	-0.68472
Q9UM00	Transmembrane and coiled-coil domain-containing	TMCO1	805420000	-0.68307
P25786	Proteasome subunit alpha type-1	PSMA1	3.47E+09	-0.67758
P26358	DNA (cytosine-5)-methyltransferase 1	DNMT1	1.396E+09	-0.67653
Q92572	AP-3 complex subunit sigma-1	AP3S1	143820000	-0.67504
P61619	Protein transport protein Sec61 subunit alpha isof	SEC61A1	809340000	-0.66951
Q9Y262	Eukaryotic translation initiation factor 3 subunit L	EIF3L	7.312E+09	-0.66587
Q13257	Mitotic spindle assembly checkpoint protein MAD2	MAD2L1	296840000	-0.665
O95831	Apoptosis-inducing factor 1, mitochondrial	AIFM1	3.25E+09	-0.66436
Q08J23	tRNA (cytosine(34)-C(5))-methyltransferase	NSUN2	2.259E+09	-0.66144
P51159	Ras-related protein Rab-27A	RAB27A	126500000	-0.66013
Q96CW1	AP-2 complex subunit mu	AP2M1	1.323E+09	-0.65993
Q9Y294	Histone chaperone ASF1A	ASF1A	148540000	-0.6597
P67936	Tropomyosin alpha-4 chain	TPM4	6.52E+09	-0.65748
P19022	Cadherin-2	CDH2	250970000	-0.65671
Q8IZ07	Ankyrin repeat domain-containing protein 13A	ANKRD13A	97311000	-0.65399
O60664	Perilipin-3	PLIN3	3.562E+09	-0.65376
O00203	AP-3 complex subunit beta-1	AP3B1	969820000	-0.65186
Q53H82	Beta-lactamase-like protein 2	LACTB2	247420000	-0.65059
Q93009	Ubiquitin carboxyl-terminal hydrolase 7	USP7	2.946E+09	-0.65027
O00743	Serine/threonine-protein phosphatase 6 catalytic s	PPP6C	419040000	-0.64981
Q9UBI6	Guanine nucleotide-binding protein G(I)/G(S)/G(O)	GNG12	164320000	-0.64721
Q05209	Tyrosine-protein phosphatase non-receptor type 1	PTPN12	93081000	-0.64116
Q00325	Phosphate carrier protein, mitochondrial	SLC25A3	4.509E+09	-0.63973
Q9Y4C2	TRPM8 channel-associated factor 1	TCAF1	485220000	-0.63867
P09488;Q0	Glutathione S-transferase Mu 1;Glutathione S-tran	GSTM1;GSTM4	117030000	-0.63505
P06730	Eukaryotic translation initiation factor 4E	EIF4E	1.683E+09	-0.63463
Q6NYC1	Bifunctional arginine demethylase and lysyl-hydro	JMJD6	631670000	-0.63163
Q9HBL7	Plasminogen receptor (KT)	PLGRKT	71877000	-0.62891
Q96T76	MMS19 nucleotide excision repair protein homolo	MMS19	1.046E+09	-0.62295
Q99613;B5	Eukaryotic translation initiation factor 3 subunit C;	EIF3C;EIF3CL	1.141E+10	-0.62181
Q9Y2T2	AP-3 complex subunit mu-1	AP3M1	199780000	-0.6216
P52434	DNA-directed RNA polymerases I, II, and III subunit	POLR2H	126020000	-0.6215

Q9H9B4	Sideroflexin-1	SFXN1	3.699E+09	-0.61977
Q96K17	Transcription factor BTF3 homolog 4	BTF3L4	961250000	-0.61938
Q8WVJ2	NudC domain-containing protein 2	NUDCD2	285100000	-0.61746
O95104	Splicing factor, arginine/serine-rich 15	SCAF4	705060000	-0.61633
P62136	Serine/threonine-protein phosphatase PP1-alpha c	PPP1CA	2.508E+09	-0.61584
Q14669	E3 ubiquitin-protein ligase TRIP12	TRIP12	1.022E+09	-0.6132
O43670	BUB3-interacting and GLEBS motif-containing prot	ZNF207	531130000	-0.6127
Q96ST3	Paired amphipathic helix protein Sin3a	SIN3A	249840000	-0.61043
O75909	Cyclin-K	CCNK	289580000	-0.60797
P53990	IST1 homolog	IST1	495620000	-0.60636
O60518	Ran-binding protein 6	RANBP6	6614600	-0.60102
Q8TED1	Probable glutathione peroxidase 8	GPX8	78499000	-0.59971
Q8N5K1	CDGSH iron-sulfur domain-containing protein 2	CISD2	529050000	-0.59873
Q9BYI3	Hyccin	FAM126A	124130000	-0.59686
Q66K74	Microtubule-associated protein 1S;MAP1S heavy c	MAP1S	622560000	-0.59679
Q9Y5K6	CD2-associated protein	CD2AP	583460000	-0.59475
O00159	Unconventional myosin-Ic	MYO1C	3.372E+09	-0.59201
P20700	Lamin-B1	LMNB1	590050000	-0.58567
O60884	DnaJ homolog subfamily A member 2	DNAJA2	1.342E+09	-0.58235
Q5VIR6	Vacuolar protein sorting-associated protein 53 hor	VPS53	296350000	-0.5802
Q8TCJ2	Dolichyl-diphosphooligosaccharide--protein glycos	STT3B	990060000	-0.5791
Q9NYP7	Elongation of very long chain fatty acids protein 5	ELOVL5	428300000	-0.5789
O95071	E3 ubiquitin-protein ligase UBR5	UBR5	241830000	-0.57871
Q16566	Calcium/calmodulin-dependent protein kinase typ	CAMK4	64102000	-0.57858
Q6P1M0	Long-chain fatty acid transport protein 4	SLC27A4	486420000	-0.57774
P17152	Transmembrane protein 11, mitochondrial	TMEM11	51356000	-0.57494
P56134	ATP synthase subunit f, mitochondrial	ATP5J2	2.404E+09	-0.57414
Q5BJD5	Transmembrane protein 41B	TMEM41B	50324000	-0.57381
Q9H857	5-nucleotidase domain-containing protein 2	NT5DC2	985060000	-0.57085
P62191	26S protease regulatory subunit 4	PSMC1	9.102E+09	-0.56029
P09211	Glutathione S-transferase P	GSTP1	1.821E+10	-0.55912
Q9Y6R0	Numb-like protein	NUMBL	30015000	-0.55854
Q96GD0	Pyridoxal phosphate phosphatase	PDXP	231700000	-0.55837
P82650	28S ribosomal protein S22, mitochondrial	MRPS22	581160000	-0.55637
Q9NW64	Pre-mRNA-splicing factor RBM22	RBM22	87189000	-0.55564
Q9H2K8	Serine/threonine-protein kinase TAO3	TAOK3	60317000	-0.553
P62314	Small nuclear ribonucleoprotein Sm D1	SNRPD1	1.448E+09	-0.55169
Q8NB16	Mixed lineage kinase domain-like protein	MLKL	69997000	-0.54903
Q5JVF3	PCI domain-containing protein 2	PCID2	495190000	-0.54872
A6NHL2	Tubulin alpha chain-like 3	TUBAL3	227070000	-0.54716
Q9BSR8	Protein YIPF4	YIPF4	103420000	-0.5469
O95298;E9	NADH dehydrogenase [ubiquinone] 1 subunit C2;N	NDUFC2;NDUFC2-	51695000	-0.54596
P09669	Cytochrome c oxidase subunit 6C	COX6C	588050000	-0.54423
P82979	SAP domain-containing ribonucleoprotein	SARNP	862100000	-0.54301
O94776	Metastasis-associated protein MTA2	MTA2	688070000	-0.53941
Q5TZA2	Rootletin	CROCC	169810000	-0.537
Q8N983	39S ribosomal protein L43, mitochondrial	MRPL43	487470000	-0.53599
Q14008	Cytoskeleton-associated protein 5	CKAP5	3.098E+09	-0.53314
Q96CS3	FAS-associated factor 2	FAF2	2.07E+09	-0.52805
P78527	DNA-dependent protein kinase catalytic subunit	PRKDC	2.851E+10	-0.52642

Q9UHI6	Probable ATP-dependent RNA helicase DDX20	DDX20	835820000	-0.52381
Q15654	Thyroid receptor-interacting protein 6	TRIP6	766270000	-0.5178
P0CW20;P(	LIM and senescent cell antigen-like-containing dom	LIMS3L;LIMS3	104330000	-0.51706
Q13547	Histone deacetylase 1	HDAC1	1.069E+09	-0.51282
P30084	Enoyl-CoA hydratase, mitochondrial	ECHS1	2.874E+09	-0.50699
Q9Y6D9	Mitotic spindle assembly checkpoint protein MAD1	MAD1L1	119180000	-0.50631
Q969E2	Secretory carrier-associated membrane protein 4	SCAMP4	52853000	-0.50249
Q9H9A5	CCR4-NOT transcription complex subunit 10	CNOT10	124390000	-0.50192
Q10570	Cleavage and polyadenylation specificity factor sub	CPSF1	88119000	-0.50038
Q9P032	NADH dehydrogenase [ubiquinone] 1 alpha subcor	NDUFAF4	556780000	-0.50009
Q9UJS0	Calcium-binding mitochondrial carrier protein Aral	SLC25A13	2.749E+09	-0.49888
Q9UKY1	Zinc fingers and homeoboxes protein 1	ZHX1	90546000	-0.49533
P12270	Nucleoprotein TPR	TPR	2.062E+09	-0.49431
O95477	ATP-binding cassette sub-family A member 1	ABCA1	158420000	-0.49409
Q9BZX2	Uridine-cytidine kinase 2	UCK2	955610000	-0.49277
O15260	Surfeit locus protein 4	SURF4	2.573E+09	-0.49028
Q8N2K0	Monoacylglycerol lipase ABHD12	ABHD12	42593000	-0.48744
P10619	Lysosomal protective protein;Lysosomal protective	CTSA	371910000	-0.48707
Q92769	Histone deacetylase 2	HDAC2	280440000	-0.4831
P08123	Collagen alpha-2(I) chain	COL1A2	16980000	-0.48138
P30536	Translocator protein	TSPO	137650000	-0.47935
Q13505	Metaxin-1	MTX1	205580000	-0.478
P60228	Eukaryotic translation initiation factor 3 subunit E	EIF3E	8.167E+09	-0.47677
P68366	Tubulin alpha-4A chain	TUBA4A	1.64E+09	-0.47505
Q9Y277	Voltage-dependent anion-selective channel protei	VDAC3	745490000	-0.47182
Q96H79	Zinc finger CCCH-type antiviral protein 1-like	ZC3HAV1L	141760000	-0.47121
P14635	G2/mitotic-specific cyclin-B1	CCNB1	218510000	-0.46879
P0DP25;P0DP24;P0DP23			2.176E+09	-0.46858
Q9BYD2	39S ribosomal protein L9, mitochondrial	MRPL9	410630000	-0.46761
P41091;Q2	Eukaryotic translation initiation factor 2 subunit 3;	EIF2S3;EIF2S3L	4.057E+09	-0.46658
Q9HAS0	Protein Njmu-R1	C17orf75	15689000	-0.46413
Q9H4L5	Oxysterol-binding protein-related protein 3	OSBPL3	149510000	-0.46294
O96019	Actin-like protein 6A	ACTL6A	2.01E+09	-0.46097
Q9BYN8	28S ribosomal protein S26, mitochondrial	MRPS26	96019000	-0.46064
P53007	Tricarboxylate transport protein, mitochondrial	SLC25A1	815120000	-0.45929
Q92542	Nicastrin	NCSTN	96031000	-0.45438
P68363	Tubulin alpha-1B chain	TUBA1B	1.345E+11	-0.44816
Q9Y6K9	NF-kappa-B essential modulator	IKBKG	140310000	-0.44717
Q9HA64	Ketosamine-3-kinase	FN3KRP	223840000	-0.44652
P17706	Tyrosine-protein phosphatase non-receptor type 2	PTPN2	23987000	-0.44446
P24941	Cyclin-dependent kinase 2	CDK2	720310000	-0.44112
P62633	Cellular nucleic acid-binding protein	CNBP	213870000	-0.43737
Q9UJA5	tRNA (adenine(58)-N(1))-methyltransferase non-ca	TRMT6	476320000	-0.43518
O15066	Kinesin-like protein KIF3B;Kinesin-like protein KIF3	KIF3B	61433000	-0.43497
Q9NUQ7	Ufm1-specific protease 2	UFSP2	210180000	-0.43419
Q9H9J2	39S ribosomal protein L44, mitochondrial	MRPL44	472510000	-0.43363
Q13185	Chromobox protein homolog 3	CBX3	2.579E+09	-0.43286
P09543	2,3-cyclic-nucleotide 3-phosphodiesterase	CNP	538610000	-0.43072
P08579	U2 small nuclear ribonucleoprotein B	SNRPB2	277830000	-0.42826
P29218	Inositol monophosphatase 1	IMPA1	176990000	-0.42763

Q7Z406	Myosin-14	MYH14	1.801E+09	-0.42667
Q12824	SWI/SNF-related matrix-associated actin-depende	SMARCB1	76485000	-0.42401
Q9P016	Thymocyte nuclear protein 1	THYN1	140960000	-0.41997
Q05086	Ubiquitin-protein ligase E3A	UBE3A	202570000	-0.41794
Q8TB61	Adenosine 3-phospho 5-phosphosulfate transport	SLC35B2	71155000	-0.41322
P55884	Eukaryotic translation initiation factor 3 subunit B	EIF3B	8.996E+09	-0.41148
Q9UBX3	Mitochondrial dicarboxylate carrier	SLC25A10	393440000	-0.41004
Q9H078	Caseinolytic peptidase B protein homolog	CLPB	227110000	-0.40965
P38919	Eukaryotic initiation factor 4A-III;Eukaryotic initiati	EIF4A3	2.052E+09	-0.40936
Q9C0C2	182 kDa tankyrase-1-binding protein	TNKS1BP1	1.015E+09	-0.40716
Q96ST2	Protein IWS1 homolog	IWS1	236920000	-0.40198
P19105;O1	Myosin regulatory light chain 12A;Myosin regulato	MYL12A;MYL12B	1.918E+10	-0.40011
P13498	Cytochrome b-245 light chain	CYBA	70693000	-0.39721
P41208	Centrin-2	CETN2	523270000	-0.39522
P56945	Breast cancer anti-estrogen resistance protein 1	BCAR1	104870000	-0.39383
Q9ULX3	RNA-binding protein NOB1	NOB1	210630000	-0.38968
P63261	Actin, cytoplasmic 2;Actin, cytoplasmic 2, N-termir	ACTG1	2.255E+11	-0.38915
Q9UKR5	Probable ergosterol biosynthetic protein 28	C14orf1	52223000	-0.38878
Q9NRX1	RNA-binding protein PNO1	PNO1	227950000	-0.38495
Q9Y496	Kinesin-like protein KIF3A	KIF3A	304400000	-0.38368
Q9BRX2	Protein pelota homolog	PELO	514190000	-0.37976
O95602	DNA-directed RNA polymerase I subunit RPA1	POLR1A	191990000	-0.37617
Q5HYI8	Rab-like protein 3	RABL3	147920000	-0.37608
P61978	Heterogeneous nuclear ribonucleoprotein K	HNRNPK	3.646E+10	-0.3742
P49841	Glycogen synthase kinase-3 beta	GSK3B	358430000	-0.37259
Q5VTB9	E3 ubiquitin-protein ligase RNF220	RNF220	103990000	-0.37186
P22695	Cytochrome b-c1 complex subunit 2, mitochondria	UQCRC2	5.404E+09	-0.36949
P14868	Aspartate--tRNA ligase, cytoplasmic	DARS	7.016E+09	-0.36944
P63165;G2	Small ubiquitin-related modifier 1;Small ubiquitin-r	SUMO1	52007000	-0.36943
P12236	ADP/ATP translocase 3;ADP/ATP translocase 3, N-t	SLC25A6	2.428E+09	-0.36872
P09874	Poly [ADP-ribose] polymerase 1	PARP1	1.171E+10	-0.36696
P47756	F-actin-capping protein subunit beta	CAPZB	2.577E+09	-0.36646
Q14839	Chromodomain-helicase-DNA-binding protein 4	CHD4	4.387E+09	-0.36552
P23193	Transcription elongation factor A protein 1	TCEA1	634660000	-0.3655
Q14980	Nuclear mitotic apparatus protein 1	NUMA1	1.067E+09	-0.3648
Q15370	Transcription elongation factor B polypeptide 2	TCEB2	152270000	-0.36465
P47755	F-actin-capping protein subunit alpha-2	CAPZA2	1.3E+09	-0.36414
Q9NPL8	Complex I assembly factor TIMMDC1, mitochondri	TIMMDC1	147050000	-0.3632
O75340	Programmed cell death protein 6	PDCD6	310280000	-0.36309
Q9Y3E7	Charged multivesicular body protein 3	CHMP3	143890000	-0.35972
P36873	Serine/threonine-protein phosphatase PP1-gamm	PPP1CC	553900000	-0.35683
Q5JTV8	Torsin-1A-interacting protein 1	TOR1AIP1	331850000	-0.35588
O95070	Protein YIF1A	YIF1A	52397000	-0.35474
O14773	Tripeptidyl-peptidase 1	TPP1	972960000	-0.34988
O95139	NADH dehydrogenase [ubiquinone] 1 beta subcom	NDUFB6	358090000	-0.34847
Q9BXF6	Rab11 family-interacting protein 5	RAB11FIP5	274350000	-0.34031
Q99986	Serine/threonine-protein kinase VRK1	VRK1	771730000	-0.33869
Q13263	Transcription intermediary factor 1-beta	TRIM28	9.309E+09	-0.32866
Q96GA3	Protein LTV1 homolog	LTV1	260980000	-0.32781
Q6P1N0	Coiled-coil and C2 domain-containing protein 1A	CC2D1A	114160000	-0.32479

P54136	Arginine--tRNA ligase, cytoplasmic	RARS	6.552E+09	-0.32289
Q9NWU5	39S ribosomal protein L22, mitochondrial	MRPL22	155990000	-0.3206
Q8IUR0	Trafficking protein particle complex subunit 5	TRAPPC5	46958000	-0.3201
Q96DV4	39S ribosomal protein L38, mitochondrial	MRPL38	269710000	-0.31328
Q8TAF3	WD repeat-containing protein 48	WDR48	98615000	-0.30872
Q9NZ45	CDGSH iron-sulfur domain-containing protein 1	CISD1	392380000	-0.30832
Q16630	Cleavage and polyadenylation specificity factor sul	CPSF6	518040000	-0.30824
Q9NUQ3	Gamma-taxilin	TXLNG	148000000	-0.30754
O15372	Eukaryotic translation initiation factor 3 subunit H	EIF3H	4.246E+09	-0.29519
Q01844	RNA-binding protein EWS	EWSR1	1.267E+09	-0.29471
Q9UHX1	Poly(U)-binding-splicing factor PUF60	PUF60	3.175E+09	-0.28963
Q5JWF2	Guanine nucleotide-binding protein G(s) subunit al	GNAS	379450000	-0.28915
Q9UEY8	Gamma-adducin	ADD3	138000000	-0.28513
O95347	Structural maintenance of chromosomes protein 2	SMC2	1.8E+09	-0.28329
P82933	28S ribosomal protein S9, mitochondrial	MRPS9	666090000	-0.28245
Q9UH62	Armadillo repeat-containing X-linked protein 3	ARMCX3	266080000	-0.27822
Q12874	Splicing factor 3A subunit 3	SF3A3	1.8E+09	-0.2777
Q14192	Four and a half LIM domains protein 2	FHL2	73498000	-0.2767
Q9NUJ1	Mycophenolic acid acyl-glucuronide esterase, mito	ABHD10	782830000	-0.2747
Q9UNF1	Melanoma-associated antigen D2	MAGED2	970340000	-0.27326
Q96G46	tRNA-dihydrouridine(47) synthase [NAD(P)(+)]-like	DUS3L	175510000	-0.26874
Q15459	Splicing factor 3A subunit 1	SF3A1	2.293E+09	-0.26659
A0FGR8	Extended synaptotagmin-2	ESYT2	407720000	-0.26483
P46821	Microtubule-associated protein 1B;MAP1B heavy c	MAP1B	2.04E+10	-0.25631
P00918	Carbonic anhydrase 2	CA2	9.259E+09	-0.25516
O95721	Synaptosomal-associated protein 29	SNAP29	121260000	-0.24988
Q96N67	Dedicator of cytokinesis protein 7	DOCK7	578360000	-0.24949
Q5XKP0	Protein QIL1	QIL1	424740000	-0.24718
Q15393	Splicing factor 3B subunit 3	SF3B3	3.625E+09	-0.24565
Q86TS9	39S ribosomal protein L52, mitochondrial	MRPL52	230170000	-0.24554
Q14157	Ubiquitin-associated protein 2-like	UBAP2L	1.633E+09	-0.24388
O75477	Erlin-1	ERLIN1	811730000	-0.24367
Q9BUF5	Tubulin beta-6 chain	TUBB6	3.079E+10	-0.24332
O15344	E3 ubiquitin-protein ligase Midline-1	MID1	74837000	-0.23936
Q99700	Ataxin-2	ATXN2	77915000	-0.23861
P82673	28S ribosomal protein S35, mitochondrial	MRPS35	674810000	-0.23809
Q96GC9	Vacuole membrane protein 1	VMP1	344770000	-0.2363
P53985	Monocarboxylate transporter 1	SLC16A1	289720000	-0.23519
Q13084	39S ribosomal protein L28, mitochondrial	MRPL28	745430000	-0.23425
Q8IWZ3	Ankyrin repeat and KH domain-containing protein	ANKHD1	123510000	-0.2333
P46736	Lys-63-specific deubiquitinase BRCC36	BRCC3	102790000	-0.23215
Q96IJ6	Mannose-1-phosphate guanyltransferase alpha	GMPPA	440950000	-0.23113
Q9UFW8	CGG triplet repeat-binding protein 1	CGGBP1	96795000	-0.22964
P50579	Methionine aminopeptidase 2	METAP2	378070000	-0.22838
Q9BVV7	Mitochondrial import inner membrane translocase	TIMM21	26481000	-0.22712
Q92820	Gamma-glutamyl hydrolase	GGH	878470000	-0.22084
Q9Y2D5	A-kinase anchor protein 2	AKAP2	694120000	-0.21754
O00461	Golgi integral membrane protein 4	GOLIM4	80961000	-0.21697
Q9BRK5	45 kDa calcium-binding protein	SDF4	352710000	-0.21602
Q8N5M9	Protein jagunal homolog 1	JAGN1	71983000	-0.21498

Q02978	Mitochondrial 2-oxoglutarate/malate carrier prote	SLC25A11	1.777E+09	-0.21309
Q96G23	Ceramide synthase 2	CERS2	404470000	-0.21112
Q5VTU8;P5	ATP synthase subunit epsilon-like protein, mitocho	ATP5EP2;ATP5E	365130000	-0.20933
Q15555	Microtubule-associated protein RP/EB family mem	MAPRE2	558680000	-0.20833
Q8N4L2	Type 2 phosphatidylinositol 4,5-bisphosphate 4-ph	TMEM55A	51321000	-0.20662
Q9UBU8	Mortality factor 4-like protein 1	MORF4L1	130700000	-0.2014
O43674	NADH dehydrogenase [ubiquinone] 1 beta subcom	NDUFB5	311020000	-0.19776
Q9UBS4	DnaJ homolog subfamily B member 11	DNAJB11	998410000	-0.19477
O15371	Eukaryotic translation initiation factor 3 subunit D	EIF3D	4.05E+09	-0.19464
Q9Y2U8	Inner nuclear membrane protein Man1	LEMD3	104580000	-0.1923
O14925	Mitochondrial import inner membrane translocase	TIMM23	656790000	-0.19141
Q9Y295	Developmentally-regulated GTP-binding protein 1	DRG1	1.812E+09	-0.19121
P27694	Replication protein A 70 kDa DNA-binding subunit;	RPA1	3.376E+09	-0.19041
Q9HC07	Transmembrane protein 165	TMEM165	454410000	-0.18735
Q13459	Unconventional myosin-IXb	MYO9B	318310000	-0.18422
P68371	Tubulin beta-4B chain	TUBB4B	3.904E+10	-0.1773
Q9H270	Vacuolar protein sorting-associated protein 11 hor	VPS11	201940000	-0.17484
Q15904	V-type proton ATPase subunit S1	ATP6AP1	32518000	-0.17149
P57088	Transmembrane protein 33	TMEM33	652860000	-0.17082
Q86U44	N6-adenosine-methyltransferase 70 kDa subunit	METTL3	183640000	-0.17025
O43809	Cleavage and polyadenylation specificity factor sul	NUDT21	2.069E+09	-0.17006
Q14558	Phosphoribosyl pyrophosphate synthase-associate	PRPSAP1	1.089E+09	-0.16874
Q9NR56	Muscleblind-like protein 1	MBNL1	269530000	-0.16839
P27449	V-type proton ATPase 16 kDa proteolipid subunit	ATP6V0C	125100000	-0.16531
Q99575	Ribonucleases P/MRP protein subunit POP1	POP1	262160000	-0.16421
P63220	40S ribosomal protein S21	RPS21	1.94E+09	-0.16272
Q9UI10	Translation initiation factor eIF-2B subunit delta	EIF2B4	302810000	-0.16116
Q9NUV7	Serine palmitoyltransferase 3	SPTLC3	270800000	-0.15883
Q01650;Q9	Large neutral amino acids transporter small subuni	SLC7A5;SLC7A7	76030000	-0.15828
P16615	Sarcoplasmic/endoplasmic reticulum calcium ATPa	ATP2A2	5.335E+09	-0.15706
Q96D46	60S ribosomal export protein NMD3	NMD3	220660000	-0.15611
Q8ND24	RING finger protein 214	RNF214	93484000	-0.15574
Q6IAN0	Dehydrogenase/reductase SDR family member 7B	DHRS7B	395880000	-0.15568
Q8WXX5	DnaJ homolog subfamily C member 9	DNAJC9	1.543E+09	-0.1556
P05141	ADP/ATP translocase 2;ADP/ATP translocase 2, N-t	SLC25A5	1.874E+10	-0.15343
P61020	Ras-related protein Rab-5B	RAB5B	607700000	-0.14884
P04049	RAF proto-oncogene serine/threonine-protein kina	RAF1	56114000	-0.14656
P16402	Histone H1.3	HIST1H1D	69777000	-0.14433
O96011	Peroxisomal membrane protein 11B	PEX11B	39152000	-0.1441
P23258;Q9	Tubulin gamma-1 chain;Tubulin gamma-2 chain	TUBG1;TUBG2	1.538E+09	-0.14396
P42025	Beta-centractin	ACTR1B	445620000	-0.13773
Q147X3	N-alpha-acetyltransferase 30	NAA30	127810000	-0.13586
Q96C36	Pyrroline-5-carboxylate reductase 2	PYCR2	664190000	-0.1355
Q9BYC8	39S ribosomal protein L32, mitochondrial	MRPL32	128500000	-0.12824
Q5T653	39S ribosomal protein L2, mitochondrial	MRPL2	364650000	-0.1265
Q32MZ4	Leucine-rich repeat flightless-interacting protein 1	LRRFIP1	658090000	-0.12278
Q13144	Translation initiation factor eIF-2B subunit epsilon	EIF2B5	298330000	-0.11572
Q5T8P6	RNA-binding protein 26	RBM26	349310000	-0.11497
P14735	Insulin-degrading enzyme	IDE	1.558E+09	-0.11448
Q15019	Septin-2	Sep-02	6.328E+09	-0.11144

Q2M1P5	Kinesin-like protein KIF7	KIF7	68399000	-0.11132
Q9Y679	Ancient ubiquitous protein 1	AUP1	331850000	-0.10753
Q15293	Reticulocalbin-1	RCN1	5.85E+09	-0.10718
Q96CW5	Gamma-tubulin complex component 3	TUBGCP3	191260000	-0.10254
Q6UXN9	WD repeat-containing protein 82	WDR82	200480000	-0.09981
Q9P015	39S ribosomal protein L15, mitochondrial	MRPL15	368060000	-0.09978
Q15582	Transforming growth factor-beta-induced protein i	TGFBI	75583000	-0.09818
P78362	SRSF protein kinase 2;SRSF protein kinase 2 N-tern	SRPK2	185520000	-0.0974
P56556	NADH dehydrogenase [ubiquinone] 1 alpha subcor	NDUFA6	253750000	-0.09659
Q9NTJ3	Structural maintenance of chromosomes protein 4	SMC4	1.932E+09	-0.09525
P36915	Guanine nucleotide-binding protein-like 1	GNL1	143820000	-0.09344
Q92979	Ribosomal RNA small subunit methyltransferase N	EMG1	143800000	-0.09222
P46977	Dolichyl-diphosphooligosaccharide--protein glycos	STT3A	640840000	-0.09073
Q9HCK8;Q8	Chromodomain-helicase-DNA-binding protein 8;Ch	CHD8;CHD6;CHD9,	86603000	-0.0903
P78346	Ribonuclease P protein subunit p30	RPP30	652530000	-0.08745
Q16891	MICOS complex subunit MIC60	IMMT	3.645E+09	-0.08606
O75821	Eukaryotic translation initiation factor 3 subunit G	EIF3G	4.885E+09	-0.08577
O15269	Serine palmitoyltransferase 1	SPTLC1	465650000	-0.08489
O43795	Unconventional myosin-Ib	MYO1B	974380000	-0.0823
O95864	Fatty acid desaturase 2	FADS2	8492000	-0.08123
Q6P9B6	TLD domain-containing protein 1	TLDC1	213800000	-0.08044
Q9NYK5	39S ribosomal protein L39, mitochondrial	MRPL39	748490000	-0.08008
P51648	Fatty aldehyde dehydrogenase	ALDH3A2	248660000	-0.07728
P82909	28S ribosomal protein S36, mitochondrial	MRPS36	146090000	-0.06803
Q9HCU5	Prolactin regulatory element-binding protein	PREB	135310000	-0.06796
Q8N1F7	Nuclear pore complex protein Nup93	NUP93	1.222E+09	-0.06367
O75382	Tripartite motif-containing protein 3	TRIM3	81569000	-0.06347
Q99442	Translocation protein SEC62	SEC62	63872000	-0.06152
P08621	U1 small nuclear ribonucleoprotein 70 kDa	SNRNP70	1.369E+09	-0.06142
O94874	E3 UFM1-protein ligase 1	UFL1	733860000	-0.05902
P51692;P42	Signal transducer and activator of transcription 5B,	STAT5B;STAT5A	128520000	-0.05645
Q13885	Tubulin beta-2A chain	TUBB2A	104290000	-0.05624
Q9BQ70	Transcription factor 25	TCF25	107690000	-0.05373
Q15046	Lysine--tRNA ligase	KARS	3.634E+09	-0.04742
P23634	Plasma membrane calcium-transporting ATPase 4	ATP2B4	206520000	-0.04732
Q01970	1-phosphatidylinositol 4,5-bisphosphate phosphoc	PLCB3	507450000	-0.04338
P28288	ATP-binding cassette sub-family D member 3	ABCD3	494610000	-0.03813
Q14258	E3 ubiquitin/ISG15 ligase TRIM25	TRIM25	1.287E+09	-0.03727
Q92785	Zinc finger protein ubi-d4	DPF2	176610000	-0.03661
Q7Z4Q2	HEAT repeat-containing protein 3	HEATR3	238770000	-0.03413
Q9BYD6	39S ribosomal protein L1, mitochondrial	MRPL1	321840000	-0.03197
P47897	Glutamine--tRNA ligase	QARS	4.875E+09	-0.03063
Q99729	Heterogeneous nuclear ribonucleoprotein A/B	HNRNPAB	2.197E+09	-0.02694
Q96S59	Ran-binding protein 9	RANBP9	189180000	-0.02523
Q96EY7	Pentatricopeptide repeat domain-containing prote	PTCD3	890960000	-0.02192
Q9BSD7	Cancer-related nucleoside-triphosphatase	NTPCR	1.13E+09	-0.02186
Q2NL82	Pre-rRNA-processing protein TSR1 homolog	TSR1	581040000	-0.02079
P00749	Urokinase-type plasminogen activator;Urokinase-t	PLAU	87085000	-0.02034
P35222	Catenin beta-1	CTNNB1	640890000	-0.01968
Q15054	DNA polymerase delta subunit 3	POLD3	181890000	-0.01876

P62306	Small nuclear ribonucleoprotein F	SNRPF	1.225E+09	-0.01798
Q8TEQ6	Gem-associated protein 5	GEMIN5	1.089E+09	-0.01701
Q9GZT9	Egl nine homolog 1	EGLN1	36589000	-0.01321
Q9UHR5	SAP30-binding protein	SAP30BP	127220000	-0.0125
O43852	Calumenin	CALU	1.64E+10	-0.00697
O43399	Tumor protein D54	TPD52L2	3.442E+09	-0.00442
Q13356	Peptidyl-prolyl cis-trans isomerase-like 2	PPIL2	95187000	-0.00298
Q6SZW1	Sterile alpha and TIR motif-containing protein 1	SARM1	153580000	-0.00023
P11142	Heat shock cognate 71 kDa protein	HSPA8	8.872E+10	0
Q9H2H8	Peptidyl-prolyl cis-trans isomerase-like 3	PPIL3	78558000	0.00233
P07814	Bifunctional glutamate/proline--tRNA ligase;Glutar	EPRS	1.083E+10	0.002857
Q9BSJ2	Gamma-tubulin complex component 2	TUBGCP2	689920000	0.003049
P10599	Thioredoxin	TXN	4.082E+09	0.003085
P14678;P63	Small nuclear ribonucleoprotein-associated protein	SNRPB;SNRPN	485590000	0.006971
P06493	Cyclin-dependent kinase 1	CDK1	1.724E+09	0.008334
P43353;P48	Aldehyde dehydrogenase family 3 member B1;Ald	ALDH3B1;ALDH3B2	82997000	0.009136
Q9BQ95	Evolutionarily conserved signaling intermediate in	ECSIT	202390000	0.012925
Q9Y399	28S ribosomal protein S2, mitochondrial	MRPS2	204230000	0.015817
P36021	Monocarboxylate transporter 8	SLC16A2	17454000	0.016003
Q13586	Stromal interaction molecule 1	STIM1	40364000	0.017607
P04844	Dolichyl-diphosphooligosaccharide--protein glycos	RPN2	1.173E+10	0.018131
Q9UKL0	REST corepressor 1	RCOR1	109680000	0.019727
P55011	Solute carrier family 12 member 2	SLC12A2	115700000	0.019894
O75533	Splicing factor 3B subunit 1	SF3B1	6.509E+09	0.020293
O00767	Acyl-CoA desaturase	SCD	593100000	0.020936
Q9UBM7	7-dehydrocholesterol reductase	DHCR7	321540000	0.023142
O75494	Serine/arginine-rich splicing factor 10	SRSF10	96612000	0.025093
Q96GC5	39S ribosomal protein L48, mitochondrial	MRPL48	141880000	0.025188
Q9H2D6	TRIO and F-actin-binding protein	TRIOBP	490920000	0.025741
Q15286	Ras-related protein Rab-35	RAB35	185210000	0.029778
Q07666	KH domain-containing, RNA-binding, signal transdu	KHDRBS1	2.969E+09	0.03049
Q9UNL2	Translocon-associated protein subunit gamma	SSR3	710150000	0.031257
Q6PJT7	Zinc finger CCCH domain-containing protein 14	ZC3H14	144210000	0.035303
Q52LJ0	Protein FAM98B	FAM98B	714250000	0.037091
O95239;Q2	Chromosome-associated kinesin KIF4A;Chromosor	KIF4A;KIF4B	87682000	0.039669
Q92616	Translational activator GCN1	GCN1L1	1.004E+10	0.043285
Q9Y6W5	Wiskott-Aldrich syndrome protein family member	WASF2	137170000	0.046219
P60468	Protein transport protein Sec61 subunit beta	SEC61B	699400000	0.046722
Q9BZE1	39S ribosomal protein L37, mitochondrial	MRPL37	760070000	0.049878
Q8IUF8	Bifunctional lysine-specific demethylase and histid	MINA	175780000	0.049976
Q2TAY7	WD40 repeat-containing protein SMU1;WD40 repe	SMU1	236510000	0.054667
Q8N6R0	Methyltransferase-like protein 13	METTL13	213270000	0.056608
Q13162	Peroxiredoxin-4	PRDX4	1.595E+09	0.058624
O75607	Nucleoplasmin-3	NPM3	505630000	0.059551
O43237	Cytoplasmic dynein 1 light intermediate chain 2	DYNC1LI2	544630000	0.05981
Q8TCG1	Protein CIP2A	KIAA1524	132680000	0.061242
P32119	Peroxiredoxin-2	PRDX2	5.967E+09	0.06172
Q9BZL4	Protein phosphatase 1 regulatory subunit 12C	PPP1R12C	31018000	0.061768
Q15746	Myosin light chain kinase, smooth muscle;Myosin l	MYLK	99616000	0.063422
Q9NZ01	Very-long-chain enoyl-CoA reductase	TECR	758950000	0.065268

Q8WYA6	Beta-catenin-like protein 1	CTNNBL1	413770000	0.069216
Q96F86	Enhancer of mRNA-decapping protein 3	EDC3	531400000	0.070551
Q96KP1	Exocyst complex component 2	EXOC2	366170000	0.071916
Q04837	Single-stranded DNA-binding protein, mitochondri	SSBP1	6.797E+09	0.073097
Q14671	Pumilio homolog 1	PUM1	245600000	0.073356
P12277	Creatine kinase B-type	CKB	4.855E+10	0.073786
P82932	28S ribosomal protein S6, mitochondrial	MRPS6	105440000	0.074689
Q9NYV4	Cyclin-dependent kinase 12	CDK12	103350000	0.078597
P55042	GTP-binding protein RAD	RRAD	73327000	0.07981
Q7Z3K3	Pogo transposable element with ZNF domain	POGZ	25174000	0.081103
Q9H2U1	ATP-dependent RNA helicase DHX36	DHX36	239660000	0.081158
O43598	2-deoxynucleoside 5-phosphate N-hydrolase 1	DNPH1	736260000	0.0859
Q9HC98;Q8	Serine/threonine-protein kinase Nek6;Serine/thre	NEK6;NEK7	58343000	0.087328
Q15018	BRISC complex subunit Abro1	FAM175B	181620000	0.092177
P46934	E3 ubiquitin-protein ligase NEDD4	NEDD4	300400000	0.093327
Q9UN37	Vacuolar protein sorting-associated protein 4A	VPS4A	267460000	0.094823
Q9Y6N5	Sulfide:quinone oxidoreductase, mitochondrial	SQRDL	2.501E+09	0.097632
Q12981	Vesicle transport protein SEC20	BNIP1	88523000	0.098091
Q7LGA3	Heparan sulfate 2-O-sulfotransferase 1	HS2ST1	61930000	0.100614
Q9NSC5	Homer protein homolog 3	HOMER3	152230000	0.101184
P23508	Colorectal mutant cancer protein	MCC	57362000	0.10466
Q86TG7	Retrotransposon-derived protein PEG10	PEG10	34584000	0.104667
Q9Y291	28S ribosomal protein S33, mitochondrial	MRPS33	59758000	0.105191
Q96LI5;Q9U	CCR4-NOT transcription complex subunit 6-like;CC	CNOT6L;CNOT6	44087000	0.106467
Q8IZH2	5-3 exoribonuclease 1	XRN1	53576000	0.112908
Q969S3	Zinc finger protein 622	ZNF622	258970000	0.113459
Q13049	E3 ubiquitin-protein ligase TRIM32	TRIM32	76439000	0.115384
Q96EC8	Protein YIPF6	YIPF6	63437000	0.11593
Q8NEZ5	F-box only protein 22	FBXO22	289590000	0.117603
Q12905	Interleukin enhancer-binding factor 2	ILF2	5.884E+09	0.120179
Q9BTE7	DCN1-like protein 5	DCUN1D5	372960000	0.121789
Q53GQ0	Very-long-chain 3-oxoacyl-CoA reductase	HSD17B12	2.059E+09	0.123177
O00483	Cytochrome c oxidase subunit NDUFA4	NDUFA4	91204000	0.125049
Q9P1Y5	Calmodulin-regulated spectrin-associated protein :	CAMSAP3	49137000	0.126841
Q13155	Aminoacyl tRNA synthase complex-interacting mul	AIMP2	1.591E+09	0.12704
O14497	AT-rich interactive domain-containing protein 1A	ARID1A	86199000	0.128558
Q12756	Kinesin-like protein KIF1A	KIF1A	282290000	0.133131
P68871;P02	Hemoglobin subunit beta;LVV-hemorphin-7;Spinor	HBB;HBD	18981000	0.134804
Q12789	General transcription factor 3C polypeptide 1	GTF3C1	162440000	0.13496
Q86XP3	ATP-dependent RNA helicase DDX42	DDX42	1.464E+09	0.14033
Q9NR50	Translation initiation factor eIF-2B subunit gamma	EIF2B3	364450000	0.140664
P56962	Syntaxin-17	STX17	39232000	0.141
O75165	DnaJ homolog subfamily C member 13	DNAJC13	2.56E+09	0.14288
Q8WXI9	Transcriptional repressor p66-beta	GATAD2B	146720000	0.144185
O95168	NADH dehydrogenase [ubiquinone] 1 beta subcom	NDUFB4	276960000	0.145091
Q9NWB7	Intraflagellar transport protein 57 homolog	IFT57	66833000	0.145289
Q9BRT8;Q8	COBW domain-containing protein 1;COBW domair	CBWD1;CBWD2;CI	68803000	0.147882
Q8WUY1	Protein THEM6	THEM6	69957000	0.148491
Q1KMD3	Heterogeneous nuclear ribonucleoprotein U-like p	HNRNPUL2	498660000	0.148845
Q9H7D0	Dedicator of cytokinesis protein 5	DOCK5	104290000	0.151901

P62140	Serine/threonine-protein phosphatase PP1-beta ca	PPP1CB	539550000	0.153922
O60669	Monocarboxylate transporter 2	SLC16A7	12676000	0.155496
P61964	WD repeat-containing protein 5	WDR5	146660000	0.156039
O15084	Serine/threonine-protein phosphatase 6 regulator	ANKRD28	23705000	0.157478
O43324	Eukaryotic translation elongation factor 1 epsilon-1	EEF1E1	1.38E+09	0.158776
P41252	Isoleucine--tRNA ligase, cytoplasmic	IARS	9.194E+09	0.15988
Q969M3	Protein YIPF5	YIPF5	86174000	0.163277
P13987	CD59 glycoprotein	CD59	24812000	0.163289
Q9NVE7	Pantothenate kinase 4	PANK4	73476000	0.163877
Q86UP2	Kinectin	KTN1	6.198E+09	0.166898
Q5VTL8	Pre-mRNA-splicing factor 38B	PRPF38B	71214000	0.169238
O95249	Golgi SNAP receptor complex member 1	GOSR1	366040000	0.170561
Q96A72	Protein mago nashi homolog 2	MAGOHB	1.025E+09	0.171117
P13716	Delta-aminolevulinic acid dehydratase	ALAD	96300000	0.174083
Q9Y4W2	Ribosomal biogenesis protein LAS1L	LAS1L	181270000	0.175946
Q92643	GPI-anchor transamidase	PIGK	100510000	0.179343
Q9Y5U2	Protein TSSC4	TSSC4	39786000	0.183058
P20290	Transcription factor BTF3	BTF3	5.331E+09	0.183835
Q9Y625;O7	Glypican-6;Secreted glypican-6;Glypican-4;Secrete	GPC6;GPC4	111070000	0.184365
Q9BV36	Melanophilin	MLPH	629360000	0.184494
Q9NX63	MICOS complex subunit MIC19	CHCHD3	630570000	0.184705
Q12979	Active breakpoint cluster region-related protein	ABR	887870000	0.190289
Q8NBM4	Ubiquitin-associated domain-containing protein 2	UBAC2	22016000	0.190982
Q9BUN8	Derlin-1	DERL1	180430000	0.192277
O43414	ERI1 exoribonuclease 3	ERI3	10591000	0.19241
Q9HD26	Golgi-associated PDZ and coiled-coil motif-containi	GOPC	143850000	0.194185
P43307	Translocon-associated protein subunit alpha	SSR1	1.518E+09	0.194638
Q8TAQ2	SWI/SNF complex subunit SMARCC2	SMARCC2	839300000	0.194916
Q86TB9	Protein PAT1 homolog 1	PATL1	293090000	0.196749
P24844	Myosin regulatory light polypeptide 9	MYL9	36577000	0.201995
Q8N5C6	S1 RNA-binding domain-containing protein 1	SRBD1	49191000	0.205099
Q7Z3U7	Protein MON2 homolog	MON2	178990000	0.209932
Q9BTC0	Death-inducer obliterator 1	DIDO1	245970000	0.210127
Q9P0M9	39S ribosomal protein L27, mitochondrial	MRPL27	76636000	0.211203
Q9NUQ2	1-acyl-sn-glycerol-3-phosphate acyltransferase eps	AGPAT5	21099000	0.214405
Q9NQ29	Putative RNA-binding protein Luc7-like 1	LUC7L	20704000	0.218476
O94842	TOX high mobility group box family member 4	TOX4	72030000	0.223874
Q15796	Mothers against decapentaplegic homolog 2	SMAD2	56231000	0.226352
Q8TCC3	39S ribosomal protein L30, mitochondrial	MRPL30	79109000	0.226918
O43491	Band 4.1-like protein 2	EPB41L2	1.451E+09	0.231699
Q8NI27	THO complex subunit 2	THOC2	352960000	0.231814
Q8WUD4	Coiled-coil domain-containing protein 12	CCDC12	31613000	0.232056
P11233	Ras-related protein Ral-A	RALA	165820000	0.234069
Q9BYC9	39S ribosomal protein L20, mitochondrial	MRPL20	93487000	0.234375
P55196	Afadin	MLLT4	229250000	0.23639
Q9BWF3	RNA-binding protein 4	RBM4	671560000	0.236834
O75643	U5 small nuclear ribonucleoprotein 200 kDa helica	SNRNP200	5.588E+09	0.239924
Q9Y5B9	FACT complex subunit SPT16	SUPT16H	2.262E+09	0.243462
P35914	Hydroxymethylglutaryl-CoA lyase, mitochondrial	HMGCL	164320000	0.243716
P52907	F-actin-capping protein subunit alpha-1	CAPZA1	5.323E+09	0.244085

P26368	Splicing factor U2AF 65 kDa subunit	U2AF2	3.977E+09	0.245679
Q8WU90	Zinc finger CCCH domain-containing protein 15	ZC3H15	723550000	0.246748
P06753	Tropomyosin alpha-3 chain	TPM3	2.414E+09	0.246939
Q12800;Q9	Alpha-globin transcription factor CP2;Upstream-bi	TFCP2;UBP1	73841000	0.247211
P51398	28S ribosomal protein S29, mitochondrial	DAP3	1.006E+09	0.248893
Q16666	Gamma-interferon-inducible protein 16	IFI16	1.562E+09	0.251305
Q05519	Serine/arginine-rich splicing factor 11	SRSF11	971000000	0.251311
P62304	Small nuclear ribonucleoprotein E	SNRPE	3.29E+09	0.255697
Q7Z7F7	39S ribosomal protein L55, mitochondrial	MRPL55	204160000	0.256493
P39880	Homeobox protein cut-like 1	CUX1	51484000	0.256583
O75122	CLIP-associating protein 2	CLASP2	418420000	0.257143
Q9H0E2	Toll-interacting protein	TOLLIP	92495000	0.261523
Q53GS9	U4/U6.U5 tri-snRNP-associated protein 2	USP39	533050000	0.266017
P35659	Protein DEK	DEK	600070000	0.266057
P36957	Dihydrolipoyllysine-residue succinyltransferase co	DLST	3.121E+09	0.267433
P62249	40S ribosomal protein S16	RPS16	1.055E+10	0.279927
Q658P3	Metalloreductase STEAP3	STEAP3	647670000	0.280145
Q13045	Protein flightless-1 homolog	FLII	1.09E+09	0.283
Q8WTS6	Histone-lysine N-methyltransferase SETD7	SETD7	255440000	0.284154
Q96A35	39S ribosomal protein L24, mitochondrial	MRPL24	644630000	0.287971
O14578	Citron Rho-interacting kinase	CIT	226400000	0.297284
Q15629	Translocating chain-associated membrane protein	TRAM1	244500000	0.300428
Q9BWU0	Kanadaptin	SLC4A1AP	292880000	0.301303
Q9BY43	Charged multivesicular body protein 4a	CHMP4A	162390000	0.303621
Q13435	Splicing factor 3B subunit 2	SF3B2	4.647E+09	0.318682
Q15070	Mitochondrial inner membrane protein OXA1L	OXA1L	134400000	0.318851
Q96GM8	Target of EGR1 protein 1	TOE1	29653000	0.32192
Q9Y265	RuvB-like 1	RUVBL1	7.703E+09	0.323251
Q9BQE3	Tubulin alpha-1C chain	TUBA1C	765790000	0.325477
O75486	Transcription initiation protein SPT3 homolog	SUPT3H	16037000	0.32557
Q13595	Transformer-2 protein homolog alpha	TRA2A	142960000	0.325753
Q8N684	Cleavage and polyadenylation specificity factor su	CPSF7	288720000	0.326753
Q13501	Sequestosome-1	SQSTM1	3.799E+09	0.332934
O75746	Calcium-binding mitochondrial carrier protein Aral	SLC25A12	1.77E+09	0.333362
Q9Y5T5	Ubiquitin carboxyl-terminal hydrolase 16	USP16	45672000	0.335264
Q8IY17	Neuropathy target esterase	PNPLA6	444200000	0.337238
Q8NI35	InaD-like protein	INADL	726730000	0.338355
O43505	Beta-1,4-glucuronyltransferase 1	B4GAT1	86443000	0.340936
Q8N1F8	Serine/threonine-protein kinase 11-interacting pro	STK11IP	2.756E+09	0.341304
P35250	Replication factor C subunit 2	RFC2	729740000	0.343155
Q9NR12	PDZ and LIM domain protein 7	PDLIM7	485970000	0.344966
Q15648	Mediator of RNA polymerase II transcription subur	MED1	88365000	0.346093
Q9Y3D3	28S ribosomal protein S16, mitochondrial	MRPS16	159440000	0.350861
Q03001	Dystonin	DST	1.18E+09	0.35171
Q96DG6	Carboxymethylenebutenolidase homolog	CMBL	197870000	0.353102
Q08AM6	Protein VAC14 homolog	VAC14	716810000	0.355408
P19388	DNA-directed RNA polymerases I, II, and III subunit	POLR2E	430560000	0.355735
Q9BRZ2	E3 ubiquitin-protein ligase TRIM56	TRIM56	72639000	0.356588
Q8TB03	Uncharacterized protein CXorf38	CXorf38	99978000	0.357336
P49959	Double-strand break repair protein MRE11A	MRE11A	694270000	0.357764

O14545	TRAF-type zinc finger domain-containing protein 1	TRAFD1	14469000	0.357862
Q9UJ83	2-hydroxyacyl-CoA lyase 1	HACL1	54762000	0.363213
P26599	Polypyrimidine tract-binding protein 1	PTBP1	1.549E+10	0.364957
P26196	Probable ATP-dependent RNA helicase DDX6	DDX6	2.696E+09	0.365619
Q96KR1	Zinc finger RNA-binding protein	ZFR	252560000	0.366941
Q13523	Serine/threonine-protein kinase PRP4 homolog	PRPF4B	96229000	0.369045
Q96EI5	Transcription elongation factor A protein-like 4	TCEAL4	43185000	0.372403
Q92552	28S ribosomal protein S27, mitochondrial	MRPS27	980460000	0.373155
P62081	40S ribosomal protein S7	RPS7	2.549E+10	0.374186
O43264	Centromere/kinetochore protein zw10 homolog	ZW10	993530000	0.378832
Q8NC51	Plasminogen activator inhibitor 1 RNA-binding pro	SERBP1	5.04E+09	0.378841
Q9UK59	Lariat debranching enzyme	DBR1	36208000	0.381307
O14949	Cytochrome b-c1 complex subunit 8	UQCRQ	268150000	0.381906
Q9UKA4	A-kinase anchor protein 11	AKAP11	115130000	0.387516
Q13561	Dynactin subunit 2	DCTN2	576820000	0.39474
Q8WUM9	Sodium-dependent phosphate transporter 1	SLC20A1	84448000	0.394794
O75439	Mitochondrial-processing peptidase subunit beta	PMPCB	1.34E+09	0.395445
P23396	40S ribosomal protein S3	RPS3	1.483E+10	0.397245
Q9GZL7	Ribosome biogenesis protein WDR12	WDR12	158700000	0.402704
Q9BRP8	Partner of Y14 and mago	WIBG	192860000	0.404227
P42345	Serine/threonine-protein kinase mTOR	MTOR	387280000	0.411472
O60828	Polyglutamine-binding protein 1	PQBP1	199540000	0.413086
Q53EU6	Glycerol-3-phosphate acyltransferase 3	AGPAT9	729180000	0.414685
Q08379	Golgin subfamily A member 2	GOLGA2	684240000	0.415704
Q9UMS4	Pre-mRNA-processing factor 19	PRPF19	2.471E+09	0.417389
P35251	Replication factor C subunit 1	RFC1	269590000	0.420183
Q92925	SWI/SNF-related matrix-associated actin-depende	SMARCD2	24662000	0.420615
Q9Y2Q9	28S ribosomal protein S28, mitochondrial	MRPS28	208530000	0.423718
O75691	Small subunit processome component 20 homolog	UTP20	62189000	0.424195
Q9Y676	28S ribosomal protein S18b, mitochondrial	MRPS18B	273460000	0.425345
Q9NP92	28S ribosomal protein S30, mitochondrial	MRPS30	207890000	0.42805
P15880	40S ribosomal protein S2	RPS2	1.087E+10	0.430681
P07339	Cathepsin D;Cathepsin D light chain;Cathepsin D h	CTSD	4.752E+09	0.433022
Q5SSJ5	Heterochromatin protein 1-binding protein 3	HP1BP3	651250000	0.438024
P82664	28S ribosomal protein S10, mitochondrial	MRPS10	251960000	0.442191
O94766	Galactosylgalactosylxylosylprotein 3-beta-glucuron	B3GAT3	190410000	0.448316
Q9C0D9	Ethanolaminephosphotransferase 1	EPT1	210790000	0.449317
P20585	DNA mismatch repair protein Msh3	MSH3	31075000	0.451741
Q13112	Chromatin assembly factor 1 subunit B	CHAF1B	53953000	0.453537
Q14739	Lamin-B receptor	LBR	2.02E+09	0.454597
B3EWF7	Laforin, isoform 9	EPM2A	14314000	0.454599
P10909	Clusterin;Clusterin beta chain;Clusterin alpha chair	CLU	188040000	0.458427
Q96DI7	U5 small nuclear ribonucleoprotein 40 kDa protein	SNRNP40	88232000	0.458902
Q8TED0	U3 small nucleolar RNA-associated protein 15 hom	UTP15	11922000	0.461288
Q6P6C2	RNA demethylase ALKBH5	ALKBH5	160360000	0.46913
Q96SB4	SRSF protein kinase 1	SRPK1	330160000	0.46921
Q92665	28S ribosomal protein S31, mitochondrial	MRPS31	651400000	0.471328
Q8N163	Cell cycle and apoptosis regulator protein 2	CCAR2	2.375E+09	0.471855
Q9BZF9	Uveal autoantigen with coiled-coil domains and an	UACA	83085000	0.471965
O43143	Pre-mRNA-splicing factor ATP-dependent RNA heli	DHX15	3.198E+09	0.474758

Q9UJF2	Ras GTPase-activating protein nGAP	RASAL2	198810000	0.475227
Q9H000	Probable E3 ubiquitin-protein ligase makorin-2	MKRN2	142670000	0.478487
Q9H5Q4	Dimethyladenosine transferase 2, mitochondrial	TFB2M	90429000	0.483828
Q9NU22	Midasin	MDN1	295710000	0.484875
Q02127	Dihydroorotate dehydrogenase (quinone), mitocho	DHODH	38520000	0.485244
P02751	Fibronectin;Anastellin;Ugl-Y1;Ugl-Y2;Ugl-Y3	FN1	206490000	0.487964
Q86U38	Nucleolar protein 9	NOP9	303900000	0.488936
P62753	40S ribosomal protein S6	RPS6	6.378E+09	0.491069
P84090	Enhancer of rudimentary homolog	ERH	87359000	0.493792
P11387	DNA topoisomerase 1	TOP1	1.214E+09	0.493837
P17480	Nucleolar transcription factor 1	UBTF	100980000	0.493974
Q96CU9	FAD-dependent oxidoreductase domain-containin	FOXRED1	22429000	0.495761
Q96SZ5	2-aminoethanethiol dioxygenase	ADO	35825000	0.497511
P04150	Glucocorticoid receptor	NR3C1	71182000	0.499385
Q5T1M5	FK506-binding protein 15	FKBP15	279200000	0.499802
O94905	Erlin-2	ERLIN2	2.405E+09	0.501336
Q08170	Serine/arginine-rich splicing factor 4	SRSF4	228970000	0.501349
Q92900	Regulator of nonsense transcripts 1	UPF1	2.714E+09	0.502688
Q13724	Mannosyl-oligosaccharide glucosidase	MOGS	886930000	0.502916
Q7Z4V5	Hepatoma-derived growth factor-related protein 2	HDGFRP2	390340000	0.508452
O43813	LanC-like protein 1	LANCL1	89273000	0.5089
Q9Y315	Deoxyribose-phosphate aldolase	DERA	383270000	0.509454
P40818	Ubiquitin carboxyl-terminal hydrolase 8	USP8	36611000	0.509507
Q14011	Cold-inducible RNA-binding protein	CIRBP	1.075E+09	0.511253
P49848	Transcription initiation factor TFIID subunit 6	TAF6	20345000	0.512052
Q9H0V9	VIP36-like protein	LMAN2L	149210000	0.514992
Q9NZN8	CCR4-NOT transcription complex subunit 2	CNOT2	149450000	0.515778
P09661	U2 small nuclear ribonucleoprotein A	SNRPA1	1.092E+09	0.515932
P48681	Nestin	NES	96651000	0.520091
P62318	Small nuclear ribonucleoprotein Sm D3	SNRPD3	2.324E+09	0.520425
O60716	Catenin delta-1	CTNND1	1.439E+09	0.52625
Q7L2J0	7SK snRNA methylphosphate capping enzyme	MEPCE	209190000	0.527703
Q9BVG9	Phosphatidylserine synthase 2	PTDSS2	35507000	0.530974
Q9Y3D9	28S ribosomal protein S23, mitochondrial	MRPS23	601650000	0.536876
Q9NRZ9	Lymphoid-specific helicase	HELLS	107640000	0.537506
Q6P2Q9	Pre-mRNA-processing-splicing factor 8	PRPF8	5.404E+09	0.538299
Q5K651	Sterile alpha motif domain-containing protein 9	SAMD9	74682000	0.545288
P49458	Signal recognition particle 9 kDa protein	SRP9	568690000	0.547339
Q14161	ARF GTPase-activating protein GIT2	GIT2	220850000	0.548603
O14654	Insulin receptor substrate 4	IRS4	504230000	0.549058
Q96JB2	Conserved oligomeric Golgi complex subunit 3	COG3	180810000	0.550232
Q9NQT4	Exosome complex component RRP46	EXOSC5	93830000	0.551923
P52701	DNA mismatch repair protein Msh6	MSH6	883280000	0.552186
Q27J81	Inverted formin-2	INF2	4.077E+09	0.552291
P62847	40S ribosomal protein S24	RPS24	5.757E+09	0.552971
Q9NVC6	Mediator of RNA polymerase II transcription subur	MED17	35183000	0.554574
Q9GZR7	ATP-dependent RNA helicase DDX24	DDX24	299840000	0.555225
O60762	Dolichol-phosphate mannosyltransferase subunit 1	DPM1	1.27E+09	0.555457
O14965	Aurora kinase A	AURKA	53900000	0.559478
Q9H936;Q9	Mitochondrial glutamate carrier 1;Mitochondrial g	SLC25A22;SLC25A1	340620000	0.562175

O00541	Pescadillo homolog	PES1	364750000	0.562798
Q9P2E9	Ribosome-binding protein 1	RRBP1	3.145E+09	0.568883
Q96K37	Solute carrier family 35 member E1	SLC35E1	109410000	0.570864
O94854	Uncharacterized protein KIAA0754	KIAA0754	44935000	0.571017
P46783	40S ribosomal protein S10	RPS10	1.126E+10	0.573355
P17066	Heat shock 70 kDa protein 6	HSPA6	4.967E+09	0.574245
Q00839	Heterogeneous nuclear ribonucleoprotein U	HNRNPU	1.122E+10	0.580217
O76071	Probable cytosolic iron-sulfur protein assembly pro	CIAO1	116490000	0.580593
Q71RC2	La-related protein 4	LARP4	345160000	0.580754
Q96PY5	Formin-like protein 2	FMNL2	148860000	0.584086
P09234	U1 small nuclear ribonucleoprotein C	SNRPC	154140000	0.58494
Q96QC0	Serine/threonine-protein phosphatase 1 regulator	PPP1R10	132450000	0.586143
Q02880	DNA topoisomerase 2-beta	TOP2B	59563000	0.587101
Q8WZ42	Titin	TTN	120700000	0.59588
P23434	Glycine cleavage system H protein, mitochondrial	GCSH	236380000	0.599168
Q9BV38	WD repeat-containing protein 18	WDR18	160910000	0.599572
A8MXV4	Nucleoside diphosphate-linked moiety X motif 19,	NUDT19	144700000	0.60625
P49773	Histidine triad nucleotide-binding protein 1	HINT1	183660000	0.606921
O94832	Unconventional myosin-Id	MYO1D	78182000	0.608515
Q6NUQ4	Transmembrane protein 214	TMEM214	72558000	0.611468
O15042	U2 snRNP-associated SURP motif-containing prote	U2SURP	1.388E+09	0.612681
Q16637	Survival motor neuron protein	SMN1	505760000	0.612939
P22830	Ferrochelatase, mitochondrial	FECH	120800000	0.614861
P08708	40S ribosomal protein S17	RPS17	8.64E+09	0.616781
E9PAV3	Nascent polypeptide-associated complex subunit a	NACA	5.279E+09	0.617465
Q9BVA1	Tubulin beta-2B chain	TUBB2B	436410000	0.619044
Q8IXM3	39S ribosomal protein L41, mitochondrial	MRPL41	544360000	0.621081
Q9BU23	Lipase maturation factor 2	LMF2	189310000	0.626637
Q9BX40	Protein LSM14 homolog B	LSM14B	204730000	0.631238
Q96I24	Far upstream element-binding protein 3	FUBP3	1.418E+09	0.631296
P62701	40S ribosomal protein S4, X isoform	RPS4X	7.362E+09	0.635856
Q9BTA9	WW domain-containing adapter protein with coile	WAC	68332000	0.63595
P47813;O1	Eukaryotic translation initiation factor 1A, X-chrom	EIF1AX;EIF1AY	1.048E+09	0.641371
O95816	BAG family molecular chaperone regulator 2	BAG2	1.215E+09	0.64889
Q16540	39S ribosomal protein L23, mitochondrial	MRPL23	165100000	0.652331
Q9UNS1	Protein timeless homolog	TIMELESS	24792000	0.652984
P57678	Gem-associated protein 4	GEMIN4	198180000	0.655112
Q9P0V3	SH3 domain-binding protein 4	SH3BP4	31698000	0.658916
Q9Y3B4	Splicing factor 3B subunit 6	SF3B6	1.1E+09	0.66213
A5YKK6	CCR4-NOT transcription complex subunit 1	CNOT1	1.282E+09	0.664484
Q14956	Transmembrane glycoprotein NMB	GPNMB	301740000	0.666107
O95819	Mitogen-activated protein kinase kinase kinase kin	MAP4K4	402610000	0.667438
P45973	Chromobox protein homolog 5	CBX5	167090000	0.66934
Q7Z478	ATP-dependent RNA helicase DHX29	DHX29	496930000	0.669789
Q9NXW2	DnaJ homolog subfamily B member 12	DNAJB12	86619000	0.671577
Q9BQ67	Glutamate-rich WD repeat-containing protein 1	GRWD1	683310000	0.671712
Q15025	TNFAIP3-interacting protein 1	TNIP1	25808000	0.673453
Q9Y613	FH1/FH2 domain-containing protein 1	FHOD1	1.354E+09	0.674962
Q9H1I8	Activating signal cointegrator 1 complex subunit 2	ASCC2	189960000	0.675611
Q6P1K8;Q1	General transcription factor IIH subunit 2-like prot	GTF2H2C;GTF2H2	43456000	0.676646

P09651;A0/	Heterogeneous nuclear ribonucleoprotein A1;Hete	HNRNPA1;HNRNP/	1.73E+10	0.677818
Q9NX20	39S ribosomal protein L16, mitochondrial	MRPL16	176670000	0.679185
P19525	Interferon-induced, double-stranded RNA-activate	EIF2AK2	669730000	0.681014
O75886	Signal transducing adapter molecule 2	STAM2	111210000	0.681907
Q9UKD2	mRNA turnover protein 4 homolog	MRTO4	426980000	0.68207
Q9P270	SLAIN motif-containing protein 2	SLAIN2	122740000	0.682668
Q15424;Q1	Scaffold attachment factor B1;Scaffold attachment	SAFB;SAFB2	271230000	0.683485
Q8WUA2	Peptidyl-prolyl cis-trans isomerase-like 4	PPIL4	225970000	0.68573
P61247	40S ribosomal protein S3a	RPS3A	1.106E+10	0.686747
Q9H0U3	Magnesium transporter protein 1	MAGT1	200940000	0.689677
Q92499	ATP-dependent RNA helicase DDX1	DDX1	6.46E+09	0.691776
O14979	Heterogeneous nuclear ribonucleoprotein D-like	HNRNPDL	2.164E+09	0.698079
P25490	Transcriptional repressor protein YY1	YY1	17954000	0.702042
Q8WY22	BRI3-binding protein	BRI3BP	101980000	0.704784
Q8NE71	ATP-binding cassette sub-family F member 1	ABCF1	942500000	0.706561
Q92615	La-related protein 4B	LARP4B	355910000	0.711487
O75818	Ribonuclease P protein subunit p40	RPP40	78412000	0.715344
Q8IXB1	DnaJ homolog subfamily C member 10	DNAJC10	305500000	0.716749
P30876	DNA-directed RNA polymerase II subunit RPB2	POLR2B	371910000	0.718847
Q96HY6	DDRGK domain-containing protein 1	DDRGK1	1.269E+09	0.720575
Q7Z3C6	Autophagy-related protein 9A	ATG9A	56892000	0.725825
Q86UL3	Glycerol-3-phosphate acyltransferase 4	AGPAT6	19051000	0.72607
P55769	NHP2-like protein 1;NHP2-like protein 1, N-termin	NHP2L1	177550000	0.726481
P49792	E3 SUMO-protein ligase RanBP2	RANBP2	1.231E+09	0.726977
Q5VYS8	Terminal uridylyltransferase 7	ZCCHC6	117630000	0.727055
Q8N8I0	Sterile alpha motif domain-containing protein 12	SAMD12	22041000	0.72887
Q03135	Caveolin-1	CAV1	1.157E+09	0.729116
Q14137	Ribosome biogenesis protein BOP1	BOP1	202860000	0.729632
Q01082	Spectrin beta chain, non-erythrocytic 1	SPTBN1	3.212E+09	0.73184
Q8N3C0	Activating signal cointegrator 1 complex subunit 3	ASCC3	301510000	0.73207
Q9Y426	C2 domain-containing protein 2	C2CD2	109030000	0.733711
Q9NV56	MRG/MORF4L-binding protein	MRGBP	159840000	0.740677
O60524	Nuclear export mediator factor NEMF	NEMF	153030000	0.741016
Q9UQB8	Brain-specific angiogenesis inhibitor 1-associated p	BAIAP2	526930000	0.748484
Q12959	Disks large homolog 1	DLG1	83219000	0.750364
Q9UQ88;P2	Cyclin-dependent kinase 11A;Cyclin-dependent kir	CDK11A;CDK11B	322870000	0.757386
Q96GY0	Zinc finger C2HC domain-containing protein 1A	ZC2HC1A	13009000	0.758788
Q96P47	Arf-GAP with GTPase, ANK repeat and PH domain-	AGAP3	270170000	0.760501
Q8TDB6	E3 ubiquitin-protein ligase DTX3L	DTX3L	137680000	0.761083
Q9HCD5	Nuclear receptor coactivator 5	NCOA5	52454000	0.761801
Q9UPT8	Zinc finger CCCH domain-containing protein 4	ZC3H4	244620000	0.763196
P53350	Serine/threonine-protein kinase PLK1	PLK1	29744000	0.763975
O43615	Mitochondrial import inner membrane translocase	TIMM44	1.615E+09	0.765486
Q96T88	E3 ubiquitin-protein ligase UHRF1	UHRF1	233600000	0.766202
O43294	Transforming growth factor beta-1-induced transc	TGFB1I1	116890000	0.766666
Q7RTV0	PHD finger-like domain-containing protein 5A	PHF5A	62919000	0.769627
O15212	Prefoldin subunit 6	PFDN6	11670000	0.7698
Q6P1L8	39S ribosomal protein L14, mitochondrial	MRPL14	160760000	0.770234
O00560	Syntenin-1	SDCBP	5.529E+09	0.774857
Q8TAP9	M-phase-specific PLK1-interacting protein	MPLKIP	12342000	0.774906

Q9HD33	39S ribosomal protein L47, mitochondrial	MRPL47	312860000	0.776009
Q9NVU7	Protein SDA1 homolog	SDAD1	51180000	0.77627
Q9Y5X2	Sorting nexin-8	SNX8	257890000	0.778089
Q9NRW3	DNA dC->dU-editing enzyme APOBEC-3C	APOBEC3C	63858000	0.778176
Q8IVS2	Malonyl-CoA-acyl carrier protein transacylase, mit	MCAT	43843000	0.780377
P52926	High mobility group protein HMGI-C	HMGA2	45132000	0.782424
Q86YP4	Transcriptional repressor p66-alpha	GATAD2A	185400000	0.791729
P12235	ADP/ATP translocase 1	SLC25A4	204460000	0.793341
Q16678	Cytochrome P450 1B1	CYP1B1	50816000	0.794662
P82675	28S ribosomal protein S5, mitochondrial	MRPS5	332360000	0.797628
Q9NZI8	Insulin-like growth factor 2 mRNA-binding protein	IGF2BP1	2.614E+09	0.798274
Q9BQ51	Programmed cell death 1 ligand 2	PDCD1LG2	10041000	0.799908
Q15029	116 kDa U5 small nuclear ribonucleoprotein comp	EFTUD2	3.743E+09	0.800285
O95714	E3 ubiquitin-protein ligase HERC2	HERC2	94183000	0.800734
Q5BJF2	Transmembrane protein 97	TMEM97	41495000	0.803481
Q8WXF1	Paraspeckle component 1	PSPC1	806580000	0.804507
Q9Y241	HIG1 domain family member 1A, mitochondrial	HIGD1A	154430000	0.805485
Q14683	Structural maintenance of chromosomes protein 1	SMC1A	1.446E+09	0.808594
O15228	Dihydroxyacetone phosphate acyltransferase	GNPAT	174320000	0.810774
Q07021	Complement component 1 Q subcomponent-bindi	C1QBP	1.026E+10	0.812299
Q9GZZ1	N-alpha-acetyltransferase 50	NAA50	701550000	0.812819
Q9UBU9	Nuclear RNA export factor 1	NXF1	101180000	0.81542
Q08380	Galectin-3-binding protein	LGALS3BP	321180000	0.818679
Q92562	Polyphosphoinositide phosphatase	FIG4	119200000	0.820409
Q7Z739	YTH domain-containing family protein 3	YTHDF3	367350000	0.821047
Q08945	FACT complex subunit SSRP1	SSRP1	2.775E+09	0.825678
Q99661	Kinesin-like protein KIF2C	KIF2C	147970000	0.826453
Q9ULX6	A-kinase anchor protein 8-like	AKAP8L	225010000	0.829964
O43709	Probable 18S rRNA (guanine-N(7))-methyltransfera	WBSCR22	198070000	0.831586
Q15678	Tyrosine-protein phosphatase non-receptor type 1	PTPN14	113380000	0.835004
O75323	Protein NipSnap homolog 2	GBAS	334890000	0.836613
O75940	Survival of motor neuron-related-splicing factor 3C	SMNDC1	228050000	0.839784
Q96TA2	ATP-dependent zinc metalloprotease YME1L1	YME1L1	328400000	0.84216
O94851	Protein-methionine sulfoxide oxidase MICAL2	MICAL2	26426000	0.845741
Q16629	Serine/arginine-rich splicing factor 7	SRSF7	921840000	0.846405
Q9NRX2	39S ribosomal protein L17, mitochondrial	MRPL17	129060000	0.849019
Q15554	Telomeric repeat-binding factor 2	TERF2	101400000	0.850083
Q9UDT6	CAP-Gly domain-containing linker protein 2	CLIP2	77215000	0.854975
P62269	40S ribosomal protein S18	RPS18	7.677E+09	0.856353
Q71UM5	40S ribosomal protein S27-like	RPS27L	213860000	0.857797
P82663	28S ribosomal protein S25, mitochondrial	MRPS25	112850000	0.85953
P82914	28S ribosomal protein S15, mitochondrial	MRPS15	33044000	0.86736
O60264	SWI/SNF-related matrix-associated actin-depende	SMARCA5	461740000	0.870307
P22626	Heterogeneous nuclear ribonucleoproteins A2/B1	HNRNPA2B1	1.253E+10	0.872017
P0DMV9;P(	Heat shock 70 kDa protein 1B;Heat shock 70 kDa p	HSPA1B;HSPA1A	8.492E+09	0.877286
P49916	DNA ligase 3	LIG3	126370000	0.878439
P25398	40S ribosomal protein S12	RPS12	3.19E+09	0.878544
P18510	Interleukin-1 receptor antagonist protein	IL1RN	76861000	0.880739
Q96II8	Leucine-rich repeat and calponin homology domai	LRCH3	23505000	0.885351
O43390	Heterogeneous nuclear ribonucleoprotein R	HNRNPR	2.05E+09	0.894483

A4D1E9	GTP-binding protein 10	GTPBP10	214310000	0.897076
O15226	NF-kappa-B-repressing factor	NKRF	32344000	0.903013
P78357	Contactin-associated protein 1	CNTNAP1	66707000	0.905964
Q8WV07	Oral cancer-overexpressed protein 1	ORAOV1	9657400	0.90671
Q59GN2;P6	Putative 60S ribosomal protein L39-like 5;60S ribos	RPL39P5;RPL39	607150000	0.907525
Q01804	OTU domain-containing protein 4	OTUD4	33466000	0.907698
P67870	Casein kinase II subunit beta	CSNK2B	180480000	0.910153
Q9Y230	RuvB-like 2	RUVBL2	6.419E+09	0.910272
Q9Y4P3	Transducin beta-like protein 2	TBL2	521540000	0.914825
P68032;P6	Actin, alpha cardiac muscle 1;Actin, gamma-enteri	ACTC1;ACTG2;ACT	1.501E+10	0.916583
O60506	Heterogeneous nuclear ribonucleoprotein Q	SYNCRIP	7.764E+09	0.920463
Q9P0K7	Ankycorbin	RAI14	705020000	0.923324
Q9BZH6	WD repeat-containing protein 11	WDR11	1.681E+10	0.926348
Q96JP5	E3 ubiquitin-protein ligase ZFP91	ZFP91	53931000	0.9285
Q9ULJ3	Zinc finger and BTB domain-containing protein 21	ZBTB21	34988000	0.92855
Q8TAE8	Growth arrest and DNA damage-inducible proteins	GADD45GIP1	391660000	0.929114
Q06265	Exosome complex component RRP45	EXOSC9	283010000	0.931822
Q8NFW8	N-acylneuraminate cytidylyltransferase	CMAS	226690000	0.93478
P08047	Transcription factor Sp1	SP1	71856000	0.935165
Q9NNW5	WD repeat-containing protein 6	WDR6	157180000	0.936907
Q9Y383	Putative RNA-binding protein Luc7-like 2	LUC7L2	1.445E+09	0.93835
Q9BZF3	Oxysterol-binding protein-related protein 6	OSBPL6	44800000	0.940327
Q01780	Exosome component 10	EXOSC10	1.008E+09	0.943534
Q13310	Polyadenylate-binding protein 4	PABPC4	7.68E+09	0.953529
Q9BQ61	Uncharacterized protein C19orf43	C19orf43	255520000	0.957452
Q15149	Plectin	PLEC	2.414E+10	0.958169
P0DN76;Q0	Splicing factor U2AF 35 kDa subunit	U2AF1	1.519E+09	0.958187
Q9HAB8	Phosphopantothenate--cysteine ligase	PPCS	50465000	0.958275
Q14527	Helicase-like transcription factor	HLTF	685690000	0.958982
Q9BUQ8	Probable ATP-dependent RNA helicase DDX23	DDX23	779590000	0.961194
Q8WVM0	Dimethyladenosine transferase 1, mitochondrial	TFB1M	35449000	0.963578
P61962	DDB1- and CUL4-associated factor 7	DCAF7	206850000	0.967538
Q9NPD3	Exosome complex component RRP41	EXOSC4	466830000	0.969352
Q9NZC9	SWI/SNF-related matrix-associated actin-depende	SMARCAL1	60703000	0.97321
O15160	DNA-directed RNA polymerases I and III subunit RI	POLR1C	558130000	0.97647
P49585	Choline-phosphate cytidylyltransferase A	PCYT1A	289150000	0.977874
Q9H814	Phosphorylated adapter RNA export protein	PHAX	222510000	0.979633
Q8NHQ9	ATP-dependent RNA helicase DDX55	DDX55	74998000	0.981616
P19784	Casein kinase II subunit alpha	CSNK2A2	689940000	0.988722
Q63ZY3	KN motif and ankyrin repeat domain-containing pr	KANK2	271930000	0.988904
P54132	Bloom syndrome protein	BLM	215420000	0.991686
P60866	40S ribosomal protein S20	RPS20	3.004E+09	0.996944
Q96T51	RUN and FYVE domain-containing protein 1	RUFY1	124830000	1.0001
P52597	Heterogeneous nuclear ribonucleoprotein F;Heter	HNRNPF	8.29E+09	1.001032
O75955	Flotillin-1	FLOT1	109560000	1.002555
Q01433	AMP deaminase 2	AMPD2	347780000	1.004184
P46782	40S ribosomal protein S5;40S ribosomal protein S5	RPS5	4.905E+09	1.005341
Q9Y281	Cofilin-2	CFL2	1.703E+09	1.006067
Q6P4A7	Sideroflexin-4	SFXN4	49989000	1.007396
O14936	Peripheral plasma membrane protein CASK	CASK	37596000	1.009318

O43707	Alpha-actinin-4	ACTN4	3.557E+10	1.010711
P62857	40S ribosomal protein S28	RPS28	4.091E+09	1.011966
P62277	40S ribosomal protein S13	RPS13	1.246E+10	1.015632
P55795	Heterogeneous nuclear ribonucleoprotein H2	HNRNPH2	585410000	1.017496
Q9BU76	Multiple myeloma tumor-associated protein 2	MMTAG2	47873000	1.019974
Q15773	Myeloid leukemia factor 2	MLF2	153350000	1.020283
Q8N7H5	RNA polymerase II-associated factor 1 homolog	PAF1	437170000	1.02214
Q96DA6	Mitochondrial import inner membrane translocase	DNAJC19	80346000	1.026338
Q5VST9	Obscurin	OBSCN	157910000	1.027306
P46781	40S ribosomal protein S9	RPS9	8.704E+09	1.030497
Q7L4I2	Arginine/serine-rich coiled-coil protein 2	RSRC2	99955000	1.030901
P62263	40S ribosomal protein S14	RPS14	5.188E+09	1.031954
Q9UKN8	General transcription factor 3C polypeptide 4	GTF3C4	191740000	1.032266
Q9Y3B7	39S ribosomal protein L11, mitochondrial	MRPL11	489980000	1.039048
P40429	60S ribosomal protein L13a	RPL13A	4.411E+09	1.045471
P62888	60S ribosomal protein L30	RPL30	1.364E+09	1.048377
Q9Y3T9	Nucleolar complex protein 2 homolog	NOC2L	163110000	1.063079
O94906	Pre-mRNA-processing factor 6	PRPF6	512140000	1.065968
P53814	Smoothelin	SMTN	145310000	1.067906
Q6VY07	Phosphofurin acidic cluster sorting protein 1	PACS1	149400000	1.068676
P32969	60S ribosomal protein L9	RPL9	4.339E+09	1.06871
Q8IX12	Cell division cycle and apoptosis regulator protein	CCAR1	685230000	1.069881
Q8TEM1	Nuclear pore membrane glycoprotein 210	NUP210	191260000	1.072787
P31942	Heterogeneous nuclear ribonucleoprotein H3	HNRNPH3	475640000	1.073575
Q02809	Procollagen-lysine,2-oxoglutarate 5-dioxygenase 1	PLOD1	1.909E+09	1.076222
P40938	Replication factor C subunit 3	RFC3	127420000	1.078121
Q9NUQ6	SPATS2-like protein	SPATS2L	591420000	1.078267
Q8IZ69	tRNA (uracil-5-)-methyltransferase homolog A	TRMT2A	13692000	1.079582
Q16698	2,4-dienoyl-CoA reductase, mitochondrial	DECR1	1.162E+09	1.082528
P49750	YLP motif-containing protein 1	YLPM1	140880000	1.083348
Q9P035	Very-long-chain (3R)-3-hydroxyacyl-CoA dehydrata	HACD3	1.124E+09	1.084261
Q15397	Pumilio domain-containing protein KIAA0020	KIAA0020	277990000	1.084563
P31943	Heterogeneous nuclear ribonucleoprotein H;Heter	HNRNPH1	1.243E+10	1.086206
Q8IWR0	Zinc finger CCCH domain-containing protein 7A	ZC3H7A	76355000	1.090613
O95232	Luc7-like protein 3	LUC7L3	914350000	1.09148
P62266	40S ribosomal protein S23	RPS23	3.811E+09	1.093203
Q92600	Cell differentiation protein RCD1 homolog	RQCD1	111750000	1.093624
Q9NP77	RNA polymerase II subunit A C-terminal domain ph	SSU72	147580000	1.094641
Q63HN8	E3 ubiquitin-protein ligase RNF213	RNF213	815560000	1.094918
Q13510	Acid ceramidase;Acid ceramidase subunit alpha;Ac	ASAH1	312150000	1.095143
P27169	Serum paraoxonase/arylesterase 1	PON1	998990000	1.104322
Q13136	Liprin-alpha-1	PPFIA1	236950000	1.108729
Q8IVF6;A2A	Ankyrin repeat domain-containing protein 18A;Ank	ANKRD18A;ANKRD	88802000	1.113731
O75190	DnaJ homolog subfamily B member 6	DNAJB6	54069000	1.114686
Q9H300	Presenilins-associated rhomboid-like protein, mito	PARL	10525000	1.117558
P10586	Receptor-type tyrosine-protein phosphatase F	PTPRF	240210000	1.118174
L0R819		ASNSD1	59013000	1.122526
P62851	40S ribosomal protein S25	RPS25	3.848E+09	1.122879
P49756	RNA-binding protein 25	RBM25	470160000	1.123605
Q86U42	Polyadenylate-binding protein 2	PABPN1	539110000	1.130493

Q13506	NGFI-A-binding protein 1	NAB1	16318000	1.132512
P20042	Eukaryotic translation initiation factor 2 subunit 2	EIF2S2	2.126E+09	1.13571
Q15154	Pericentriolar material 1 protein	PCM1	78540000	1.138427
Q9Y3C6	Peptidyl-prolyl cis-trans isomerase-like 1	PPIL1	220480000	1.140217
Q5T5Y3	Calmodulin-regulated spectrin-associated protein 1	CAMSAP1	65891000	1.143195
Q8TBA6	Golgin subfamily A member 5	GOLGA5	240620000	1.144136
Q9BQ48	39S ribosomal protein L34, mitochondrial	MRPL34	48702000	1.144698
P62241	40S ribosomal protein S8	RPS8	9.492E+09	1.14906
Q96RN5	Mediator of RNA polymerase II transcription subur	MED15	32656000	1.151788
P39019	40S ribosomal protein S19	RPS19	5.118E+09	1.157726
Q70UQ0	Inhibitor of nuclear factor kappa-B kinase-interacti	IKBIP	235090000	1.158791
P62910	60S ribosomal protein L32	RPL32	1.652E+09	1.162486
Q15024	Exosome complex component RRP42	EXOSC7	438630000	1.164926
Q9BUJ2	Heterogeneous nuclear ribonucleoprotein U-like p	HNRNPUL1	1.112E+09	1.16847
Q02241	Kinesin-like protein KIF23	KIF23	213200000	1.168939
O43663	Protein regulator of cytokinesis 1	PRC1	103160000	1.169713
Q13428	Treacle protein	TCOF1	1.206E+09	1.172514
Q9HC36	rRNA methyltransferase 3, mitochondrial	RNMTL1	17349000	1.176667
P63173	60S ribosomal protein L38	RPL38	2.754E+09	1.177054
P50750	Cyclin-dependent kinase 9	CDK9	81233000	1.178724
P05386	60S acidic ribosomal protein P1	RPLP1	920250000	1.180979
Q3MHD2	Protein LSM12 homolog	LSM12	333910000	1.181517
P17096	High mobility group protein HMG-I/HMG-Y	HMGA1	631800000	1.182772
P15407	Fos-related antigen 1	FOSL1	342520000	1.202298
O00425	Insulin-like growth factor 2 mRNA-binding protein	IGF2BP3	2.101E+09	1.204431
Q4KMP7	TBC1 domain family member 10B	TBC1D10B	375920000	1.216563
Q6PGP7	Tetratricopeptide repeat protein 37	TTC37	22398000	1.216794
P52298	Nuclear cap-binding protein subunit 2	NCBP2	83777000	1.216911
P62829	60S ribosomal protein L23	RPL23	6.107E+09	1.224802
Q12904	Aminoacyl tRNA synthase complex-interacting mul	AIMP1	2.561E+09	1.228862
Q96B26	Exosome complex component RRP43	EXOSC8	246300000	1.22916
P68400;Q8	Casein kinase II subunit alpha;Casein kinase II subu	CSNK2A1;CSNK2A3	2.97E+09	1.234484
Q9H7B2	Ribosome production factor 2 homolog	RPF2	168080000	1.235272
Q8WTT2	Nucleolar complex protein 3 homolog	NOC3L	242540000	1.237576
Q8WVC0	RNA polymerase-associated protein LEO1	LEO1	133780000	1.239686
Q8IVF7	Formin-like protein 3	FMNL3	133620000	1.240173
Q12849	G-rich sequence factor 1	GRSF1	1.857E+09	1.243472
P37108	Signal recognition particle 14 kDa protein	SRP14	3.741E+09	1.243823
P11940	Polyadenylate-binding protein 1	PABPC1	6.306E+09	1.24516
Q8IXK0	Polyhomeotic-like protein 2	PHC2	160860000	1.247517
P62854;Q5	40S ribosomal protein S26;Putative 40S ribosomal	RPS26;RPS26P11	4.491E+09	1.261526
Q02878	60S ribosomal protein L6	RPL6	1.762E+10	1.266484
Q92541	RNA polymerase-associated protein RTF1 homolog	RTF1	157290000	1.268302
Q5C9Z4	Nucleolar MIF4G domain-containing protein 1	NOM1	67136000	1.268607
Q9BPW8	Protein NipSnap homolog 1	NIPSNAP1	875720000	1.270396
Q8IWX8	Calcium homeostasis endoplasmic reticulum prote	CHERP	370580000	1.272149
Q8WUR7	UPF0235 protein C15orf40	C15orf40	88847000	1.274057
Q8TDD1	ATP-dependent RNA helicase DDX54	DDX54	249900000	1.277142
P42677	40S ribosomal protein S27	RPS27	3.118E+09	1.277237
Q96E11	Ribosome-recycling factor, mitochondrial	MRRF	385970000	1.277879

Q71UI9;P0(	Histone H2A.V;Histone H2A.Z	H2AFV;H2AFZ	143080000	1.281585
O43854	EGF-like repeat and discoidin I-like domain-contair	EDIL3	165570000	1.28309
P48634	Protein PRRC2A	PRRC2A	118760000	1.284336
O60573	Eukaryotic translation initiation factor 4E type 2	EIF4E2	140810000	1.28574
P31689	DnaJ homolog subfamily A member 1	DNAJA1	2.7E+09	1.291544
P36578	60S ribosomal protein L4	RPL4	1.709E+10	1.292637
Q6PKG0	La-related protein 1	LARP1	3.036E+09	1.292996
P05412	Transcription factor AP-1	JUN	112160000	1.293987
O95772	MLN64 N-terminal domain homolog	STARD3NL	87298000	1.296267
Q86WJ1	Chromodomain-helicase-DNA-binding protein 1-lik	CHD1L	276820000	1.299758
P14866	Heterogeneous nuclear ribonucleoprotein L	HNRNPL	3.926E+09	1.303265
Q08211	ATP-dependent RNA helicase A	DHX9	1.191E+10	1.303814
Q6GMV2	SET and MYND domain-containing protein 5	SMYD5	86866000	1.307374
Q7Z2T5	TRMT1-like protein	TRMT1L	285680000	1.317332
Q9H2V7	Protein spinster homolog 1	SPNS1	32118000	1.317842
Q96HS1	Serine/threonine-protein phosphatase PGAM5, mi	PGAM5	714960000	1.318897
P40937	Replication factor C subunit 5	RFC5	483420000	1.319118
Q15650	Activating signal cointegrator 1	TRIP4	119060000	1.321874
Q92974	Rho guanine nucleotide exchange factor 2	ARHGEF2	2.159E+09	1.322967
P39023	60S ribosomal protein L3	RPL3	1.946E+10	1.329359
Q8NB90	Spermatogenesis-associated protein 5	SPATA5	222620000	1.332113
Q9P2Q2	FERM domain-containing protein 4A	FRMD4A	14463000	1.332304
Q92522	Histone H1x	H1FX	271100000	1.333814
Q13151	Heterogeneous nuclear ribonucleoprotein A0	HNRNPA0	1.95E+09	1.334532
Q9Y3I0	tRNA-splicing ligase RtcB homolog	RTCB	7.139E+09	1.337494
Q9BYJ9	YTH domain-containing family protein 1	YTHDF1	63845000	1.339374
O43920	NADH dehydrogenase [ubiquinone] iron-sulfur pro	NDUFS5	198650000	1.340446
Q9Y5Q9	General transcription factor 3C polypeptide 3	GTF3C3	147380000	1.351088
P37840	Alpha-synuclein	SNCA	75892000	1.352185
P55040	GTP-binding protein GEM	GEM	32282000	1.353461
Q5JTH9	RRP12-like protein	RRP12	625930000	1.361612
P06748	Nucleophosmin	NPM1	5.208E+10	1.363908
Q13243	Serine/arginine-rich splicing factor 5	SRSF5	553920000	1.369459
P05388;Q8	60S acidic ribosomal protein P0;60S acidic ribosom	RPLP0;RPLP0P6	2.39E+10	1.370851
O00139	Kinesin-like protein KIF2A	KIF2A	117730000	1.371674
Q9BQG0	Myb-binding protein 1A	MYBBP1A	2.636E+09	1.373538
P62424	60S ribosomal protein L7a	RPL7A	8.67E+09	1.373928
Q9UGR2	Zinc finger CCCH domain-containing protein 7B	ZC3H7B	82174000	1.375008
Q9H967	WD repeat-containing protein 76	WDR76	8334700	1.376168
Q8IV48	3-5 exoribonuclease 1	ERI1	44015000	1.381487
Q8WVK2	U4/U6.U5 small nuclear ribonucleoprotein 27 kDa	SNRNP27	121220000	1.383314
P82930	28S ribosomal protein S34, mitochondrial	MRPS34	227780000	1.384534
O00458	Interferon-related developmental regulator 1	IFRD1	102870000	1.388142
Q01130	Serine/arginine-rich splicing factor 2	SRSF2	2.607E+09	1.388991
O00571;O1	ATP-dependent RNA helicase DDX3X;ATP-depende	DDX3X;DDX3Y	7.758E+09	1.39025
Q13283	Ras GTPase-activating protein-binding protein 1	G3BP1	3.085E+09	1.392788
Q14257	Reticulocalbin-2	RCN2	1.417E+09	1.393893
O75475	PC4 and SFRS1-interacting protein	PSIP1	207600000	1.39712
O00411	DNA-directed RNA polymerase, mitochondrial	POLRMT	93146000	1.39864
Q13247	Serine/arginine-rich splicing factor 6	SRSF6	1.494E+09	1.401985

Q9NVH2	Integrator complex subunit 7	INTS7	356710000	1.404655
P35637	RNA-binding protein FUS	FUS	4.006E+09	1.407659
Q9P2N5	RNA-binding protein 27	RBM27	118370000	1.410563
Q4G0J3	La-related protein 7	LARP7	308580000	1.410765
P19338	Nucleolin	NCL	4.079E+10	1.412346
P49247	Ribose-5-phosphate isomerase	RPIA	131680000	1.413147
Q8IZQ5	Selenoprotein H	SELH	36411000	1.416231
Q9BZI7	Regulator of nonsense transcripts 3B	UPF3B	165910000	1.417004
Q96PK6	RNA-binding protein 14	RBM14	601180000	1.424478
Q12906	Interleukin enhancer-binding factor 3	ILF3	4.019E+09	1.426801
Q86V81	THO complex subunit 4	ALYREF	2.775E+09	1.427439
Q96EY1	DnaJ homolog subfamily A member 3, mitochondr	DNAJA3	160860000	1.428221
Q9BT25	HAUS augmin-like complex subunit 8	HAUS8	27555000	1.434093
Q9Y3B2	Exosome complex component CSL4	EXOSC1	98952000	1.437494
Q01469	Fatty acid-binding protein, epidermal	FABP5	415110000	1.439445
Q9UKX7	Nuclear pore complex protein Nup50	NUP50	298380000	1.442473
Q53F19	Uncharacterized protein C17orf85	C17orf85	34220000	1.445386
Q8N5L8	Ribonuclease P protein subunit p25-like protein	RPP25L	21312000	1.446194
Q15388	Mitochondrial import receptor subunit TOM20 hor	TOMM20	813860000	1.448229
Q02543	60S ribosomal protein L18a	RPL18A	1.496E+09	1.448982
P84103	Serine/arginine-rich splicing factor 3	SRSF3	2.338E+09	1.449052
O75381	Peroxisomal membrane protein PEX14	PEX14	103090000	1.453404
Q6NZI2	Polymerase I and transcript release factor	PTRF	4.066E+09	1.455749
Q99878;Q9	Histone H2A type 1-J;Histone H2A type 1-H;Histon	HIST1H2AJ;HIST1H	5.304E+09	1.463319
Q9NR30	Nucleolar RNA helicase 2	DDX21	3.812E+09	1.465359
Q15014	Mortality factor 4-like protein 2	MORF4L2	30367000	1.467985
O94992	Protein HEXIM1	HEXIM1	230180000	1.470791
Q14498	RNA-binding protein 39	RBM39	1.645E+09	1.471426
Q9NVI7	ATPase family AAA domain-containing protein 3A	ATAD3A	4.493E+09	1.474105
O60832	H/ACA ribonucleoprotein complex subunit 4	DKC1	627870000	1.475417
Q9NXV6	CDKN2A-interacting protein	CDKN2AIP	74912000	1.477876
P11021	78 kDa glucose-regulated protein	HSPA5	7.681E+10	1.481666
P11182	Lipoamide acyltransferase component of branchec	DBT	248550000	1.488717
Q9UKV3	Apoptotic chromatin condensation inducer in the r	ACIN1	257290000	1.492855
Q9H974	Queuine tRNA-ribosyltransferase subunit QTRTD1	QTRTD1	154480000	1.494345
Q9BVP2	Guanine nucleotide-binding protein-like 3	GNL3	704740000	1.49454
Q92614	Unconventional myosin-XVIIIa	MYO18A	2.503E+09	1.494677
Q7Z2W4	Zinc finger CCCH-type antiviral protein 1	ZC3HAV1	775250000	1.49794
Q969Q0	60S ribosomal protein L36a-like	RPL36AL	656190000	1.5066
Q9Y6M1	Insulin-like growth factor 2 mRNA-binding protein	IGF2BP2	186310000	1.510495
Q9BYG3	MKI67 FHA domain-interacting nucleolar phospho	NIFK	68258000	1.512342
P22087	rRNA 2-O-methyltransferase fibrillarin	FBL	524280000	1.516266
Q86VM9	Zinc finger CCCH domain-containing protein 18	ZC3H18	80775000	1.518234
P16401	Histone H1.5	HIST1H1B	814940000	1.518441
Q9Y3D7	Mitochondrial import inner membrane translocase	PAM16	166960000	1.523455
Q9H9E3	Conserved oligomeric Golgi complex subunit 4	COG4	108330000	1.527916
P46776	60S ribosomal protein L27a	RPL27A	4.693E+09	1.528077
O75175	CCR4-NOT transcription complex subunit 3	CNOT3	289130000	1.528913
Q13123	Protein Red	IK	133170000	1.529535
P78345	Ribonuclease P protein subunit p38	RPP38	1.374E+09	1.532443

O95399	Urotensin-2	UTS2	1.392E+09	1.539075
Q9H6S0	Probable ATP-dependent RNA helicase YTHDC2	YTHDC2	115520000	1.54335
P06280	Alpha-galactosidase A	GLA	189240000	1.550423
P62841	40S ribosomal protein S15	RPS15	5.716E+09	1.550496
Q9NY93	Probable ATP-dependent RNA helicase DDX56	DDX56	49867000	1.552249
Q6ZNB6	NF-X1-type zinc finger protein NFXL1	NFXL1	35488000	1.554328
P61313	60S ribosomal protein L15	RPL15	7.591E+09	1.558885
O95985	DNA topoisomerase 3-beta-1	TOP3B	24893000	1.567524
P84098	60S ribosomal protein L19	RPL19	3.749E+09	1.568491
P27635	60S ribosomal protein L10	RPL10	4.509E+09	1.569859
P18077	60S ribosomal protein L35a	RPL35A	2.948E+09	1.570657
Q9Y221	60S ribosome subunit biogenesis protein NIP7 hom	NIP7	149830000	1.573236
Q9BVQ7	Spermatogenesis-associated protein 5-like protein	SPATA5L1	69673000	1.587049
P62906	60S ribosomal protein L10a	RPL10A	5.98E+09	1.588096
Q9NQT5	Exosome complex component RRP40	EXOSC3	523530000	1.588164
Q9H0S4	Probable ATP-dependent RNA helicase DDX47	DDX47	131140000	1.596081
Q9NQ55	Suppressor of SWI4 1 homolog	PPAN	60524000	1.602194
Q16643	Drebrin	DBN1	779510000	1.603104
P46976	Glycogenin-1	GYG1	378460000	1.606121
O00165	HCLS1-associated protein X-1	HAX1	82233000	1.607437
Q06587	E3 ubiquitin-protein ligase RING1	RING1	60640000	1.608745
Q9H0G5	Nuclear speckle splicing regulatory protein 1	NSRP1	10462000	1.614944
O75400	Pre-mRNA-processing factor 40 homolog A	PRPF40A	606700000	1.621157
Q15717	ELAV-like protein 1	ELAVL1	1.224E+09	1.623771
Q8IWS0	PHD finger protein 6	PHF6	179950000	1.627442
Q9H089	Large subunit GTPase 1 homolog	LSG1	143070000	1.630966
Q96GQ7	Probable ATP-dependent RNA helicase DDX27	DDX27	17578000	1.631419
Q9BPZ3	Polyadenylate-binding protein-interacting protein	PAIP2	112790000	1.632203
Q9UHB6	LIM domain and actin-binding protein 1	LIMA1	579770000	1.641872
Q8ND56	Protein LSM14 homolog A	LSM14A	144070000	1.667877
Q7L2E3	Putative ATP-dependent RNA helicase DHX30	DHX30	1.773E+09	1.668949
P46013	Antigen KI-67	MKI67	191130000	1.670039
Q9Y2R9	28S ribosomal protein S7, mitochondrial	MRPS7	191700000	1.673429
P46779	60S ribosomal protein L28	RPL28	3.312E+09	1.673453
Q9BVJ6;Q5	U3 small nucleolar RNA-associated protein 14 hom	UTP14A;UTP14C	81051000	1.673703
Q9NZB2	Constitutive coactivator of PPAR-gamma-like prote	FAM120A	688420000	1.674042
Q7L5L3	Glycerophosphodiester phosphodiesterase domain	GDPD3	56581000	1.675387
P62244	40S ribosomal protein S15a	RPS15A	6.676E+09	1.67555
Q96ER9	Coiled-coil domain-containing protein 51	CCDC51	95265000	1.681879
Q8NI99	Angiopoietin-related protein 6	ANGPTL6	37639000	1.685861
Q12986	Transcriptional repressor NF-X1	NFX1	17109000	1.700413
P62913	60S ribosomal protein L11	RPL11	5.103E+09	1.700474
Q7Z417	Nuclear fragile X mental retardation-interacting pr	NUFIP2	1.277E+09	1.701115
Q12797	Aspartyl/asparaginyl beta-hydroxylase	ASPH	4.745E+09	1.703332
Q14978	Nucleolar and coiled-body phosphoprotein 1	NOLC1	922850000	1.710831
Q9H0H5	Rac GTPase-activating protein 1	RACGAP1	119890000	1.714232
Q9HCG8	Pre-mRNA-splicing factor CWC22 homolog	CWC22	42910000	1.714673
P19447	TFIIH basal transcription factor complex helicase X	ERCC3	61417000	1.715379
Q07065	Cytoskeleton-associated protein 4	CKAP4	5.679E+09	1.715508
P62280	40S ribosomal protein S11	RPS11	2.503E+09	1.718004

Q9UM22	Mammalian ependymin-related protein 1	EPDR1	81229000	1.71815
Q9BVI4	Nucleolar complex protein 4 homolog	NOC4L	137830000	1.729998
P25440	Bromodomain-containing protein 2	BRD2	130680000	1.730606
Q8IYB3	Serine/arginine repetitive matrix protein 1	SRRM1	813750000	1.733505
Q7KZI7	Serine/threonine-protein kinase MARK2	MARK2	68647000	1.736209
O00567	Nucleolar protein 56	NOP56	314910000	1.736266
Q86UE4	Protein LYRIC	MTDH	1.313E+09	1.743593
O75592	E3 ubiquitin-protein ligase MYCBP2	MYCBP2	224190000	1.748209
Q13868	Exosome complex component RRP4	EXOSC2	704830000	1.757996
Q9NY61	Protein AATF	AATF	35241000	1.759372
Q15075	Early endosome antigen 1	EEA1	267880000	1.759436
Q9NYL9	Tropomodulin-3	TMOD3	598610000	1.761358
Q6UN15	Pre-mRNA 3-end-processing factor FIP1	FIP1L1	197090000	1.76423
Q5T9A4	ATPase family AAA domain-containing protein 3B	ATAD3B	1.48E+09	1.76449
Q15233	Non-POU domain-containing octamer-binding prot	NONO	6.375E+09	1.766732
Q9Y224	UPF0568 protein C14orf166	C14orf166	1.036E+09	1.768875
Q8TER5	Rho guanine nucleotide exchange factor 40	ARHGEF40	89788000	1.770013
Q9H5V9	UPF0428 protein CXorf56	CXorf56	44349000	1.773539
Q9BRT6	Protein LLP homolog	LLPH	25412000	1.777523
P46777	60S ribosomal protein L5	RPL5	6.617E+09	1.778659
Q13242	Serine/arginine-rich splicing factor 9	SRSF9	243730000	1.786017
Q96AG4	Leucine-rich repeat-containing protein 59	LRRC59	7.91E+09	1.789229
Q15287	RNA-binding protein with serine-rich domain 1	RNPS1	133450000	1.793498
Q8IUD2	ELKS/Rab6-interacting/CAST family member 1	ERC1	200420000	1.798395
Q9UJV9	Probable ATP-dependent RNA helicase DDX41	DDX41	113950000	1.814365
Q9H6F5	Coiled-coil domain-containing protein 86	CCDC86	51766000	1.817694
Q16778;P3	Histone H2B type 2-E;Histone H2B type 1-B;Histon	HIST2H2BE;HIST1H	83871000	1.821616
P26373	60S ribosomal protein L13	RPL13	7.588E+09	1.82279
P62995	Transformer-2 protein homolog beta	TRA2B	573130000	1.8248
Q99880;Q9	Histone H2B type 1-L;Histone H2B type 1-M;Histor	HIST1H2BL;HIST1H	7.155E+09	1.826912
Q92743	Serine protease HTRA1	HTRA1	33707000	1.83449
Q5RKV6	Exosome complex component MTR3	EXOSC6	670340000	1.840378
Q9NYF8	Bcl-2-associated transcription factor 1	BCLAF1	205250000	1.843807
Q5SW79	Centrosomal protein of 170 kDa	CEP170	1.088E+09	1.844717
O15446	DNA-directed RNA polymerase I subunit RPA34	CD3EAP	100940000	1.845651
Q9UEU0	Vesicle transport through interaction with t-SNARE	VTI1B	136390000	1.860543
Q9BW60	Elongation of very long chain fatty acids protein 1	ELOVL1	101320000	1.861143
P38159;Q9	RNA-binding motif protein, X chromosome;RNA-bi	RBMX;RBMXL1	1.257E+09	1.863475
O75569	Interferon-inducible double-stranded RNA-depend	PRKRA	206000000	1.86355
Q5UIP0	Telomere-associated protein RIF1	RIF1	374590000	1.866992
P09496	Clathrin light chain A	CLTA	97553000	1.867394
Q6P5R6	60S ribosomal protein L22-like 1	RPL22L1	2.142E+09	1.871462
Q07020	60S ribosomal protein L18	RPL18	8.067E+09	1.875762
Q70Z53	Protein FRA10AC1	FRA10AC1	33955000	1.875992
P52272	Heterogeneous nuclear ribonucleoprotein M	HNRNPM	1.056E+10	1.878543
O00206	Toll-like receptor 4	TLR4	282320000	1.889157
P46020	Phosphorylase b kinase regulatory subunit alpha, s	PHKA1	18515000	1.897378
P46778	60S ribosomal protein L21	RPL21	4.223E+09	1.89904
P23246	Splicing factor, proline- and glutamine-rich	SFPQ	8.996E+09	1.900787
P62750	60S ribosomal protein L23a	RPL23A	5.112E+09	1.902039

P11441	Ubiquitin-like protein 4A	UBL4A	74810000	1.902051
O43633	Charged multivesicular body protein 2a	CHMP2A	177310000	1.902239
Q92841	Probable ATP-dependent RNA helicase DDX17	DDX17	4.257E+09	1.908861
P49790	Nuclear pore complex protein Nup153	NUP153	98715000	1.919322
P47914	60S ribosomal protein L29	RPL29	2.036E+09	1.919351
Q9H0U9	Testis-specific Y-encoded-like protein 1	TSPYL1	26153000	1.919762
A2A3N6	Putative PIP5K1A and PSMD4-like protein	PIPSL	28101000	1.920043
P18124	60S ribosomal protein L7	RPL7	1.65E+10	1.927603
Q7RTP6	Protein-methionine sulfoxide oxidase MICAL3	MICAL3	96987000	1.932067
P02545	Prelamin-A/C;Lamin-A/C	LMNA	7.284E+09	1.932803
P30050	60S ribosomal protein L12	RPL12	7.702E+09	1.936532
Q12899	Tripartite motif-containing protein 26	TRIM26	6914000	1.944646
P18621	60S ribosomal protein L17	RPL17	4.905E+09	1.947942
Q53EZ4	Centrosomal protein of 55 kDa	CEP55	75609000	1.951609
P22061	Protein-L-isoaspartate(D-aspartate) O-methyltrans	PCMT1	4.282E+09	1.953089
P50914	60S ribosomal protein L14	RPL14	4.442E+09	1.954075
Q9H6R4	Nucleolar protein 6	NOL6	144160000	1.967239
Q6VMQ6	Activating transcription factor 7-interacting proteir	ATF7IP	62654000	1.976028
P17844	Probable ATP-dependent RNA helicase DDX5	DDX5	4.942E+09	1.98294
Q49A26	Putative oxidoreductase GLYR1	GLYR1	12203000	1.993675
Q9Y5Q8	General transcription factor 3C polypeptide 5	GTF3C5	140260000	1.994333
Q9NW13	RNA-binding protein 28	RBM28	378510000	1.994744
P62316	Small nuclear ribonucleoprotein Sm D2	SNRPD2	2.223E+09	1.998774
Q96C57	Uncharacterized protein C12orf43	C12orf43	116370000	1.999298
Q15646	2-5-oligoadenylate synthase-like protein	OASL	126610000	2.014627
Q96I25	Splicing factor 45	RBM17	234770000	2.019956
P43235	Cathepsin K	CTSK	6780700	2.031905
P61353	60S ribosomal protein L27	RPL27	5.148E+09	2.032346
P56182	Ribosomal RNA processing protein 1 homolog A	RRP1	158410000	2.035959
Q9NVP1	ATP-dependent RNA helicase DDX18	DDX18	356890000	2.037424
Q14690	Protein RRP5 homolog	PDCD11	150570000	2.044461
Q9BQ39	ATP-dependent RNA helicase DDX50	DDX50	220820000	2.044498
P35268	60S ribosomal protein L22	RPL22	2.128E+09	2.047401
Q9Y4C8	Probable RNA-binding protein 19	RBM19	60235000	2.059061
P61254	60S ribosomal protein L26	RPL26	2.37E+09	2.060317
Q8N999	Uncharacterized protein C12orf29	C12orf29	75137000	2.065913
Q5T2N8	ATPase family AAA domain-containing protein 3C	ATAD3C	21083000	2.066337
Q9H0A0	N-acetyltransferase 10	NAT10	848800000	2.071503
Q96QR8	Transcriptional activator protein Pur-beta	PURB	197350000	2.077594
P29558;Q1	RNA-binding motif, single-stranded-interacting prc	RBMS1;RBMS2	27120000	2.083796
P16989	Y-box-binding protein 3	YBX3	1.338E+09	2.088213
Q9NY12	H/ACA ribonucleoprotein complex subunit 1	GAR1	13671000	2.092197
Q9BY77	Polymerase delta-interacting protein 3	POLDIP3	224000000	2.095232
Q8N9T8	Protein KRI1 homolog	KRI1	41674000	2.096718
O43159	Ribosomal RNA-processing protein 8	RRP8	10204000	2.101005
P51991	Heterogeneous nuclear ribonucleoprotein A3	HNRNPA3	3.838E+09	2.10146
Q3KQU3	MAP7 domain-containing protein 1	MAP7D1	285250000	2.103333
P62805	Histone H4	HIST1H4A	2.755E+09	2.104813
Q9NTJ5	Phosphatidylinositide phosphatase SAC1	SACM1L	4.729E+09	2.106547
Q9NUL3	Double-stranded RNA-binding protein Staufen hon	STAU2	37849000	2.114965

E9PRG8	Uncharacterized protein C11orf98	C11orf98	119080000	2.126787
P49207	60S ribosomal protein L34	RPL34	2.397E+09	2.127032
O75970	Multiple PDZ domain protein	MPDZ	41533000	2.13265
Q9HAU5	Regulator of nonsense transcripts 2	UPF2	149610000	2.136502
P84243;Q7	Histone H3.3;Histone H3.2;Histone H3.1t;Histone H	H3F3A;HIST2H3A;H	777340000	2.136603
Q9Y6H1;Q5	Coiled-coil-helix-coiled-coil-helix domain-containin	CHCHD2;CHCHD2F	501520000	2.14848
Q96BW9	Phosphatidate cytidylyltransferase, mitochondrial	TAMM41	67225000	2.151275
Q9H0D6	5-3 exoribonuclease 2	XRN2	1.138E+09	2.153174
Q15633	RISC-loading complex subunit TARBP2	TARBP2	33707000	2.160244
P04792	Heat shock protein beta-1	HSPB1	5.474E+09	2.1619
P04040	Catalase	CAT	5.266E+10	2.165107
Q03701	CCAAT/enhancer-binding protein zeta	CEBPZ	194210000	2.186718
P16403	Histone H1.2	HIST1H1C	1.97E+09	2.190734
O95400	CD2 antigen cytoplasmic tail-binding protein 2	CD2BP2	522400000	2.191367
Q9Y3U8	60S ribosomal protein L36	RPL36	1.056E+09	2.199109
P62899	60S ribosomal protein L31	RPL31	5.922E+09	2.203712
Q9Y3Y2	Chromatin target of PRMT1 protein	CHTOP	134110000	2.214762
Q6FI81	Anamorsin	CIAPIN1	1.928E+09	2.216503
Q9Y5A9	YTH domain-containing family protein 2	YTHDF2	328710000	2.220688
Q96CT7	Coiled-coil domain-containing protein 124	CCDC124	838180000	2.220925
Q14331	Protein FRG1	FRG1	52814000	2.222185
P19838	Nuclear factor NF-kappa-B p105 subunit;Nuclear fa	NFKB1	112560000	2.224215
P98175	RNA-binding protein 10	RBM10	222850000	2.232014
P42766	60S ribosomal protein L35	RPL35	3.87E+09	2.232353
P42166	Lamina-associated polypeptide 2, isoform alpha;Th	TMPO	1.617E+09	2.234158
P62917	60S ribosomal protein L8	RPL8	5.478E+09	2.238649
O43251;Q9	RNA binding protein fox-1 homolog 2;RNA binding	RBFOX2;RBFOX1	81029000	2.242927
P62987;P0(	Ubiquitin-60S ribosomal protein L40;Ubiquitin;60S	UBA52;UBB;UBC	2.857E+09	2.246078
P78316	Nucleolar protein 14	NOP14	104310000	2.252969
O75223	Gamma-glutamylcyclotransferase	GGCT	352380000	2.256376
Q9Y3B9	RRP15-like protein	RRP15	46342000	2.270189
Q9Y2W1	Thyroid hormone receptor-associated protein 3	THRAP3	367810000	2.283598
O76021	Ribosomal L1 domain-containing protein 1	RSL1D1	1.229E+09	2.305123
P67809	Nuclease-sensitive element-binding protein 1	YBX1	8.578E+09	2.312258
Q86XZ4	Spermatogenesis-associated serine-rich protein 2	SPATS2	181590000	2.315778
Q1ED39	Lysine-rich nucleolar protein 1	KNOP1	14152000	2.316589
Q14444	Caprin-1	CAPRIN1	2.902E+09	2.316818
Q9HCE1	Putative helicase MOV-10	MOV10	800250000	2.324659
O43464	Serine protease HTRA2, mitochondrial	HTRA2	289920000	2.329951
P29323	Ephrin type-B receptor 2	EPHB2	223440000	2.330609
Q5T3I0	G patch domain-containing protein 4	GPATCH4	163190000	2.333574
Q14684	Ribosomal RNA processing protein 1 homolog B	RRP1B	323790000	2.335986
O95793	Double-stranded RNA-binding protein Staufen hon	STAU1	999300000	2.340561
P83731	60S ribosomal protein L24	RPL24	4.644E+09	2.366572
Q14694	Ubiquitin carboxyl-terminal hydrolase 10	USP10	273210000	2.369507
Q9UJX5	Anaphase-promoting complex subunit 4	ANAPC4	271080000	2.398884
Q07955	Serine/arginine-rich splicing factor 1	SRSF1	1.768E+09	2.40063
Q9BWE0	Replication initiator 1	REPIN1	24228000	2.401183
Q9Y3C1	Nucleolar protein 16	NOP16	38109000	2.40504
O75330	Hyaluronan mediated motility receptor	HMMR	77731000	2.415949

Q5T5P2	Sickle tail protein homolog	KIAA1217	431770000	2.419373
P05387	60S acidic ribosomal protein P2	RPLP2	2.359E+09	2.422696
P38646	Stress-70 protein, mitochondrial	HSPA9	4.481E+10	2.427241
Q9UKJ3	G patch domain-containing protein 8	GPATCH8	127020000	2.4332
Q9Y2X3	Nucleolar protein 58	NOP58	413700000	2.435316
Q9BUB5	MAP kinase-interacting serine/threonine-protein k	MKNK1	201220000	2.442631
P14373	Zinc finger protein RFP	TRIM27	54081000	2.46315
Q00577	Transcriptional activator protein Pur-alpha	PURA	650860000	2.476588
Q9BZE4	Nucleolar GTP-binding protein 1	GTPBP4	619090000	2.48072
P46087	Probable 28S rRNA (cytosine(4447)-C(5))-methyltra	NOP2	829490000	2.49056
P46100	Transcriptional regulator ATRX	ATRX	38868000	2.545439
Q9Y2I8	WD repeat-containing protein 37	WDR37	106850000	2.550504
Q13573	SNW domain-containing protein 1	SNW1	489040000	2.59127
P11388	DNA topoisomerase 2-alpha	TOP2A	104730000	2.593561
P55265	Double-stranded RNA-specific adenosine deamina	ADAR	251870000	2.635287
Q9UJX3	Anaphase-promoting complex subunit 7	ANAPC7	244660000	2.643758
Q14651	Plastin-1	PLS1	131500000	2.651843
P54802	Alpha-N-acetylglucosaminidase;Alpha-N-acetylgluc	NAGLU	33843000	2.660004
Q9UN86	Ras GTPase-activating protein-binding protein 2	G3BP2	735880000	2.66095
Q8IX01	SURP and G-patch domain-containing protein 2	SUGP2	257790000	2.670349
Q9ULV4	Coronin-1C	CORO1C	4.689E+09	2.671374
P08651	Nuclear factor 1 C-type	NFIC	44070000	2.681731
Q00059	Transcription factor A, mitochondrial	TFAM	145540000	2.683724
Q6PK04	Coiled-coil domain-containing protein 137	CCDC137	16186000	2.689691
O43290	U4/U6.U5 tri-snRNP-associated protein 1	SART1	415690000	2.714025
P48729	Casein kinase I isoform alpha	CSNK1A1	395000000	2.726248
Q9BRJ6	Uncharacterized protein C7orf50	C7orf50	330040000	2.733649
Q9Y2R4	Probable ATP-dependent RNA helicase DDX52	DDX52	40854000	2.748031
Q8NCA5	Protein FAM98A	FAM98A	527470000	2.806683
P61513	60S ribosomal protein L37a	RPL37A	613010000	2.829505
Q99459	Cell division cycle 5-like protein	CDC5L	959710000	2.83403
A6NHR9	Structural maintenance of chromosomes flexible h	SMCHD1	344230000	2.83872
Q9NX58	Cell growth-regulating nucleolar protein	LYAR	796950000	2.853744
Q99848	Probable rRNA-processing protein EBP2	EBNA1BP2	302800000	2.861214
P50402	Emerin	EMD	833910000	2.87117
P43243	Matrin-3	MATR3	3.584E+09	2.882443
Q66PJ3	ADP-ribosylation factor-like protein 6-interacting p	ARL6IP4	171120000	2.890182
O00178	GTP-binding protein 1	GTPBP1	615700000	2.907024
Q13867	Bleomycin hydrolase	BLMH	1.11E+09	2.907276
Q6WKZ4	Rab11 family-interacting protein 1	RAB11FIP1	108000000	2.922746
Q9NX24	H/ACA ribonucleoprotein complex subunit 2	NHP2	36346000	2.933556
Q9UJX4	Anaphase-promoting complex subunit 5	ANAPC5	173370000	2.950585
Q5MNZ9	WD repeat domain phosphoinositide-interacting p	WIPI1	450750000	2.951847
Q9BPX7	UPF0415 protein C7orf25	C7orf25	7305400	2.954304
Q9Y520	Protein PRRC2C	PRRC2C	1.413E+09	3.002486
Q9BVS4	Serine/threonine-protein kinase RIO2	RIOK2	173680000	3.00561
Q8TDN6	Ribosome biogenesis protein BRX1 homolog	BRIX1	577480000	3.00689
Q9UKG1	DCC-interacting protein 13-alpha	APPL1	550860000	3.011066
O14974	Protein phosphatase 1 regulatory subunit 12A	PPP1R12A	445400000	3.022295
Q15050	Ribosome biogenesis regulatory protein homolog	RRS1	90407000	3.02443

P51114	Fragile X mental retardation syndrome-related pro	FXR1	2.212E+09	3.030866
P08670	Vimentin	VIM	1.338E+10	3.042313
O94964	Protein SOGA1;N-terminal form;C-terminal 80 kDa	SOGA1	121870000	3.043707
Q9H1A4	Anaphase-promoting complex subunit 1	ANAPC1	477610000	3.064478
Q9UHD2	Serine/threonine-protein kinase TBK1	TBK1	664530000	3.068334
Q9Y608	Leucine-rich repeat flightless-interacting protein 2	LRRFIP2	138350000	3.086521
P27448	MAP/microtubule affinity-regulating kinase 3	MARK3	35553000	3.086684
P16152	Carbonyl reductase [NADPH] 1	CBR1	8.961E+09	3.103467
Q96KG9	N-terminal kinase-like protein	SCYL1	1.199E+09	3.113649
P07910;A0	Heterogeneous nuclear ribonucleoproteins C1/C2;	HNRNPC;HNRNPCI	5.595E+09	3.123294
Q92804	TATA-binding protein-associated factor 2N	TAF15	491530000	3.132379
Q9ULW0	Targeting protein for Xklp2	TPX2	33567000	3.158279
P42696	RNA-binding protein 34	RBM34	72035000	3.160683
Q9HAN9	Nicotinamide/nicotinic acid mononucleotide aden	NMNAT1	184810000	3.168322
O95479	GDH/6PGL endoplasmic bifunctional protein;Gluco	H6PD	38325000	3.183254
Q8WVF5	BTB/POZ domain-containing protein KCTD4	KCTD4	99151000	3.199171
Q06787	Fragile X mental retardation protein 1	FMR1	403840000	3.213306
P38935	DNA-binding protein SMUBP-2	IGHMBP2	24697000	3.221368
Q9NYJ8	TGF-beta-activated kinase 1 and MAP3K7-binding	TAB2	34087000	3.225234
Q6WCQ1	Myosin phosphatase Rho-interacting protein	MPRIP	1.097E+09	3.265974
P12814	Alpha-actinin-1	ACTN1	9.639E+09	3.299655
Q9H4K7	Mitochondrial ribosome-associated GTPase 2	MTG2	22044000	3.34028
Q9H6W3	Bifunctional lysine-specific demethylase and histid	NO66	9971300	3.340411
P51116	Fragile X mental retardation syndrome-related pro	FXR2	750390000	3.356977
O95453	Poly(A)-specific ribonuclease PARN	PARN	149900000	3.365007
Q9UDY2	Tight junction protein ZO-2	TJP2	2.318E+09	3.455966
O95425	Supervillin	SVIL	28220000	3.473601
O94875	Sorbin and SH3 domain-containing protein 2	SORBS2	20503000	3.485524
Q13813	Spectrin alpha chain, non-erythrocytic 1	SPTAN1	5.045E+09	3.493301
Q8IY81	pre-rRNA processing protein FTSJ3	FTSJ3	479070000	3.512954
Q7Z6B7	SLIT-ROBO Rho GTPase-activating protein 1	SRGAP1	94381000	3.562448
Q99873	Protein arginine N-methyltransferase 1	PRMT1	4.722E+09	3.701188
Q9H307	Pinin	PNN	448800000	3.7256
Q9ULQ1	Two pore calcium channel protein 1	TPCN1	15526000	3.778606
Q9UKM9	RNA-binding protein Raly	RALY	270190000	3.808557
Q96CX2	BTB/POZ domain-containing protein KCTD12	KCTD12	2.153E+09	3.813539
P30260	Cell division cycle protein 27 homolog	CDC27	539910000	3.849701
P83111	Serine beta-lactamase-like protein LACTB, mitocho	LACTB	191610000	3.86001
O75145	Liprin-alpha-3	PPFIA3	252740000	3.956597
P35580	Myosin-10	MYH10	8.469E+09	3.991994
Q7Z4L5	Tetratricopeptide repeat protein 21B	TTC21B	114170000	4.046948
O75044	SLIT-ROBO Rho GTPase-activating protein 2	SRGAP2	124910000	4.05977
Q8N806	Putative E3 ubiquitin-protein ligase UBR7	UBR7	1.104E+09	4.132703
O75828	Carbonyl reductase [NADPH] 3	CBR3	2.042E+09	4.135369
Q8IY37	Probable ATP-dependent RNA helicase DHX37	DHX37	62890000	4.168421
Q69YQ0	Cytospin-A	SPECC1L	401500000	4.222877
Q5M775	Cytospin-B	SPECC1	86691000	4.242875
P78347	General transcription factor II-I	GTF2I	9.015E+09	4.270077
Q9UQ35	Serine/arginine repetitive matrix protein 2	SRRM2	1.778E+09	4.311073
Q8TD55	Pleckstrin homology domain-containing family O m	PLEKHO2	206790000	4.34207

Q9BQS8	FYVE and coiled-coil domain-containing protein 1	FYCO1	460050000	4.360571
Q86V48	Leucine zipper protein 1	LUZP1	39967000	4.380927
P29508;P48	Serpin B3;Serpin B4	SERPINB3;SERPINE	113980000	4.389839
Q9NVR0	Kelch-like protein 11	KLHL11	189620000	4.396562
O95218	Zinc finger Ran-binding domain-containing protein	ZRANB2	427660000	4.439041
Q07157	Tight junction protein ZO-1	TJP1	631030000	4.50253
Q8WVV4	Protein POF1B	POF1B	47421000	4.669363
P83881	60S ribosomal protein L36a	RPL36A	215150000	4.732743
Q9UI42	Carboxypeptidase A4	CPA4	68129000	4.754822
P17302	Gap junction alpha-1 protein	GJA1	233450000	4.799272
Q7L5D6	Golgi to ER traffic protein 4 homolog	GET4	941620000	4.851023
P83916	Chromobox protein homolog 1	CBX1	259210000	4.933376
A6ND36	Protein FAM83G	FAM83G	371690000	5.298068
Q9BRK4	Leucine zipper putative tumor suppressor 2	LZTS2	75210000	5.33998
Q8IUR6	CREB3 regulatory factor	CREBRF	67438000	5.365834
Q8TAA9	Vang-like protein 1	VANGL1	82151000	5.509439
Q9UQ03	Coronin-2B	CORO2B	82264000	5.608199
P81605	Dermcidin;Survival-promoting peptide;DCD-1	DCD	670380000	5.685468
P46379	Large proline-rich protein BAG6	BAG6	3.846E+09	5.806973
P60006	Anaphase-promoting complex subunit 15	ANAPC15	4507400	5.899496
Q9UJX2	Cell division cycle protein 23 homolog	CDC23	138960000	6.033636
P35908	Keratin, type II cytoskeletal 2 epidermal	KRT2	2.912E+10	6.156368
Q5BKY9	Protein FAM133B	FAM133B	26705000	6.512067
O60783	28S ribosomal protein S14, mitochondrial	MRPS14	591540000	6.521307
Q02413	Desmoglein-1	DSG1	409370000	6.62297
P14923	Junction plakoglobin	JUP	305790000	6.714096
P15924	Desmoplakin	DSP	1.471E+09	6.74599
P00736	Complement C1r subcomponent;Complement C1r	C1R	116490000	6.77378
Q5T749	Keratinocyte proline-rich protein	KPRP	39765000	6.785657
Q13042	Cell division cycle protein 16 homolog	CDC16	375580000	6.858132
Q9Y4B5	Microtubule cross-linking factor 1	MTCL1	59784000	7.232892
Q6UWP8	Suprabasin	SBSN	39201000	7.23423
Q92844	TRAF family member-associated NF-kappa-B activa	TANK	105820000	7.447244
P06312	Ig kappa chain V-IV region	IGKV4-1	140440000	7.736079
Q08554	Desmocollin-1	DSC1	111390000	7.771449
P28799	Granulins;Acrogranin;Paragranulin;Granulin-1;Grar	GRN	326620000	8.112904
Q9NZT1	Calmodulin-like protein 5	CALML5	624150000	8.227778
P31944	Caspase-14;Caspase-14 subunit p17, mature form;	CASP14	331480000	8.816134
Q8IWB9	Testis-expressed sequence 2 protein	TEX2	427770000	9.149067
P40145	Adenylate cyclase type 8	ADCY8	497780000	9.775896
P19474	E3 ubiquitin-protein ligase TRIM21	TRIM21	800570000	10.72909
Q7Z2W7	Transient receptor potential cation channel subfan	TRPM8	629030000	10.96603
P25311	Zinc-alpha-2-glycoprotein	AZGP1	275930000	11.34477
P01857;P01	Ig gamma-1 chain C region;Ig gamma-3 chain C reg	IGHG1;IGHG3	3.028E+10	16.25345
P07942	Laminin subunit beta-1	LAMB1	4.635E+10	INFINITE
Q6NSI4	Uncharacterized protein CXorf57	CXorf57	9.631E+09	INFINITE
Q96QH2	PML-RARA-regulated adapter molecule 1	PRAM1	9.546E+09	INFINITE
A8K2U0	Alpha-2-macroglobulin-like protein 1	A2ML1	8.067E+09	INFINITE
P07954	Fumarate hydratase, mitochondrial	FH	7.554E+09	INFINITE
P54577	Tyrosine--tRNA ligase, cytoplasmic;Tyrosine--tRNA	YARS	6.953E+09	INFINITE

P78417	Glutathione S-transferase omega-1	GSTO1	5.237E+09	INFINITE
O15067	Phosphoribosylformylglycinamidine synthase	PFAS	3.507E+09	INFINITE
Q02818	Nucleobindin-1	NUCB1	3.332E+09	INFINITE
Q9P287	BRCA2 and CDKN1A-interacting protein	BCCIP	3.182E+09	INFINITE
Q9Y617	Phosphoserine aminotransferase	PSAT1	3.099E+09	INFINITE
P09936	Ubiquitin carboxyl-terminal hydrolase isozyme L1	UCHL1	2.887E+09	INFINITE
P09960	Leukotriene A-4 hydrolase	LTA4H	2.772E+09	INFINITE
Q9UBE0	SUMO-activating enzyme subunit 1;SUMO-activati	SAE1	2.551E+09	INFINITE
Q04446	1,4-alpha-glucan-branching enzyme	GBE1	2.463E+09	INFINITE
O94979	Protein transport protein Sec31A	SEC31A	2.436E+09	INFINITE
Q9BTT0	Acidic leucine-rich nuclear phosphoprotein 32 fam	ANP32E	2.414E+09	INFINITE
P01834	Ig kappa chain C region	IGKC	2.388E+09	INFINITE
Q15435	Protein phosphatase 1 regulatory subunit 7	PPP1R7	2.358E+09	INFINITE
Q14166	Tubulin--tyrosine ligase-like protein 12	TTLL12	2.342E+09	INFINITE
P21399	Cytoplasmic aconitate hydratase	ACO1	2.327E+09	INFINITE
Q92597	Protein NDRG1	NDRG1	2.174E+09	INFINITE
A6NJG6	Arginine-fifty homeobox	ARGFX	2.113E+09	INFINITE
Q15257	Serine/threonine-protein phosphatase 2A activato	PPP2R4	2.073E+09	INFINITE
P31150	Rab GDP dissociation inhibitor alpha	GDI1	2.018E+09	INFINITE
O96008	Mitochondrial import receptor subunit TOM40 hor	TOMM40	1.914E+09	INFINITE
Q15813	Tubulin-specific chaperone E	TBCE	1.89E+09	INFINITE
P53004	Biliverdin reductase A	BLVRA	1.816E+09	INFINITE
Q9UBQ7	Glyoxylate reductase/hydroxypyruvate reductase	GRHPR	1.783E+09	INFINITE
O75153	Clustered mitochondria protein homolog	CLUH	1.781E+09	INFINITE
P30837	Aldehyde dehydrogenase X, mitochondrial	ALDH1B1	1.716E+09	INFINITE
P31040	Succinate dehydrogenase [ubiquinone] flavoprotei	SDHA	1.631E+09	INFINITE
O75347	Tubulin-specific chaperone A	TBCA	1.629E+09	INFINITE
P0DN79;P3	Cystathionine beta-synthase	CBS	1.598E+09	INFINITE
P16435	NADPH--cytochrome P450 reductase	POR	1.582E+09	INFINITE
Q9NUU7	ATP-dependent RNA helicase DDX19A	DDX19A	1.573E+09	INFINITE
Q99598	Translin-associated protein X	TSNAX	1.555E+09	INFINITE
O60271	C-Jun-amino-terminal kinase-interacting protein 4	SPAG9	1.539E+09	INFINITE
P19623	Spermidine synthase	SRM	1.511E+09	INFINITE
P05091	Aldehyde dehydrogenase, mitochondrial	ALDH2	1.503E+09	INFINITE
Q14019	Coactosin-like protein	COTL1	1.492E+09	INFINITE
Q13126	S-methyl-5-thioadenosine phosphorylase	MTAP	1.489E+09	INFINITE
Q14914	Prostaglandin reductase 1	PTGR1	1.48E+09	INFINITE
Q10567	AP-1 complex subunit beta-1	AP1B1	1.445E+09	INFINITE
O14929	Histone acetyltransferase type B catalytic subunit	HAT1	1.407E+09	INFINITE
P49753;Q8	Acyl-coenzyme A thioesterase 2, mitochondrial;Ac	ACOT2;ACOT1	1.388E+09	INFINITE
P48147	Prolyl endopeptidase	PREP	1.372E+09	INFINITE
Q86UE8	Serine/threonine-protein kinase tousled-like 2	TLK2	1.319E+09	INFINITE
Q9BRX8	Redox-regulatory protein FAM213A	FAM213A	1.318E+09	INFINITE
Q9HB07	UPF0160 protein MYG1, mitochondrial	C12orf10	1.298E+09	INFINITE
Q9BWD1	Acetyl-CoA acetyltransferase, cytosolic	ACAT2	1.284E+09	INFINITE
Q04760	Lactoylglutathione lyase	GLO1	1.272E+09	INFINITE
Q9Y237	Peptidyl-prolyl cis-trans isomerase NIMA-interactir	PIN4	1.235E+09	INFINITE
Q8IY67	Ribonucleoprotein PTB-binding 1	RAVER1	1.227E+09	INFINITE
Q6PIU2	Neutral cholesterol ester hydrolase 1	NCEH1	1.222E+09	INFINITE
P00568	Adenylate kinase isoenzyme 1	AK1	1.222E+09	INFINITE

Q96RP9	Elongation factor G, mitochondrial	GFM1	1.206E+09	INFINITE
P15586	N-acetylglucosamine-6-sulfatase	GNS	1.189E+09	INFINITE
P49903	Selenide, water dikinase 1	SEPHS1	1.17E+09	INFINITE
Q9NRV9	Heme-binding protein 1	HEBP1	1.152E+09	INFINITE
P0DPI2;A0A0B4J2D5			1.138E+09	INFINITE
P17252	Protein kinase C alpha type	PRKCA	1.137E+09	INFINITE
O94973	AP-2 complex subunit alpha-2	AP2A2	1.101E+09	INFINITE
Q96PE2	Rho guanine nucleotide exchange factor 17	ARHGEF17	1.1E+09	INFINITE
Q9H7Z7	Prostaglandin E synthase 2;Prostaglandin E syntha:	PTGES2	1.098E+09	INFINITE
Q8TBC4	NEDD8-activating enzyme E1 catalytic subunit	UBA3	1.071E+09	INFINITE
P52888	Thimet oligopeptidase	THOP1	1.059E+09	INFINITE
P14174	Macrophage migration inhibitory factor	MIF	1.05E+09	INFINITE
P36543	V-type proton ATPase subunit E 1	ATP6V1E1	1.03E+09	INFINITE
Q99685	Monoglyceride lipase	MGLL	1.021E+09	INFINITE
Q9BV73	Centrosome-associated protein CEP250	CEP250	1.008E+09	INFINITE
Q13618	Cullin-3	CUL3	996970000	INFINITE
O95352	Ubiquitin-like modifier-activating enzyme ATG7	ATG7	995700000	INFINITE
Q8IYD1	Eukaryotic peptide chain release factor GTP-bindin	GSPT2	984070000	INFINITE
P28482	Mitogen-activated protein kinase 1	MAPK1	979640000	INFINITE
Q9NUQ9	Protein FAM49B	FAM49B	977310000	INFINITE
P54687	Branched-chain-amino-acid aminotransferase, cyto	BCAT1	967410000	INFINITE
P43897	Elongation factor Ts, mitochondrial	TSFM	963280000	INFINITE
Q14696	LDLR chaperone MESD	MESDC2	959920000	INFINITE
Q99961	Endophilin-A2	SH3GL1	955200000	INFINITE
Q9Y6Y8	SEC23-interacting protein	SEC23IP	933440000	INFINITE
O75844	CAAX prenyl protease 1 homolog	ZMPSTE24	933020000	INFINITE
P08397	Porphobilinogen deaminase	HMBS	920680000	INFINITE
Q14376	UDP-glucose 4-epimerase	GALE	907950000	INFINITE
Q00796	Sorbitol dehydrogenase	SORD	903380000	INFINITE
P30405	Peptidyl-prolyl cis-trans isomerase F, mitochondria	PPIF	877660000	INFINITE
P0DOY3;P0	Ig lambda-6 chain C region;Ig lambda-7 chain C reg	IGLC6;IGLC7	861550000	INFINITE
Q96EM0	Trans-3-hydroxy-L-proline dehydratase	L3HYPDH	859470000	INFINITE
Q9Y3E5	Peptidyl-tRNA hydrolase 2, mitochondrial	PTRH2	850160000	INFINITE
Q13616	Cullin-1	CUL1	850000000	INFINITE
P40763	Signal transducer and activator of transcription 3	STAT3	844330000	INFINITE
P31350	Ribonucleoside-diphosphate reductase subunit M2	RRM2	829910000	INFINITE
Q9H3P7	Golgi resident protein GCP60	ACBD3	828020000	INFINITE
P09417	Dihydropteridine reductase	QDPR	817150000	INFINITE
O95394	Phosphoacetylglucosamine mutase	PGM3	816260000	INFINITE
Q969H8	Myeloid-derived growth factor	MYDGF	814330000	INFINITE
Q9NZL4	Hsp70-binding protein 1	HSPBP1	810110000	INFINITE
Q9NX46	Poly(ADP-ribose) glycohydrolase ARH3	ADPRHL2	804080000	INFINITE
Q9BV20	Methylthioribose-1-phosphate isomerase	MRI1	802890000	INFINITE
Q9Y5X3	Sorting nexin-5	SNX5	792320000	INFINITE
Q6NVY1	3-hydroxyisobutyryl-CoA hydrolase, mitochondrial	HIBCH	785790000	INFINITE
Q9UHY1	Nuclear receptor-binding protein	NRBP1	783290000	INFINITE
Q13564	NEDD8-activating enzyme E1 regulatory subunit	NAE1	778560000	INFINITE
Q9H993	Protein-glutamate O-methyltransferase	ARMT1	775450000	INFINITE
Q9GZY8	Mitochondrial fission factor	MFF	771480000	INFINITE
Q9UHY7	Enolase-phosphatase E1	ENOPH1	771210000	INFINITE

P49006	MARCKS-related protein	MARCKSL1	770110000	INFINITE
O43768	Alpha-endosulfine	ENSA	766190000	INFINITE
Q15126	Phosphomevalonate kinase	PMVK	765470000	INFINITE
Q9H488	GDP-fucose protein O-fucosyltransferase 1	POFUT1	764430000	INFINITE
Q4J6C6	Prolyl endopeptidase-like	PREPL	761340000	INFINITE
P20645	Cation-dependent mannose-6-phosphate receptor	M6PR	742080000	INFINITE
Q13423	NAD(P) transhydrogenase, mitochondrial	NNT	739320000	INFINITE
P49643	DNA primase large subunit	PRIM2	739000000	INFINITE
Q9Y333	U6 snRNA-associated Sm-like protein LSm2	LSM2	729540000	INFINITE
P61956	Small ubiquitin-related modifier 2	SUMO2	726360000	INFINITE
Q99426	Tubulin-folding cofactor B	TBCB	724240000	INFINITE
Q9BV86	N-terminal Xaa-Pro-Lys N-methyltransferase 1;N-te	NTMT1	719410000	INFINITE
Q9NZ08	Endoplasmic reticulum aminopeptidase 1	ERAP1	710780000	INFINITE
P08962	CD63 antigen	CD63	706900000	INFINITE
P20936	Ras GTPase-activating protein 1	RASA1	703500000	INFINITE
P35270	Sepiapterin reductase	SPR	701150000	INFINITE
O14737	Programmed cell death protein 5	PDCD5	700040000	INFINITE
Q9Y2Z4	Tyrosine--tRNA ligase, mitochondrial	YARS2	695400000	INFINITE
Q9GZZ9	Ubiquitin-like modifier-activating enzyme 5	UBA5	690780000	INFINITE
Q9Y2Z0	Suppressor of G2 allele of SKP1 homolog	SUGT1	686790000	INFINITE
P53041	Serine/threonine-protein phosphatase 5	PPP5C	677340000	INFINITE
P52306	Rap1 GTPase-GDP dissociation stimulator 1	RAP1GDS1	676760000	INFINITE
Q53EL6	Programmed cell death protein 4	PDCD4	667700000	INFINITE
O15347	High mobility group protein B3	HMGB3	664180000	INFINITE
P12532	Creatine kinase U-type, mitochondrial	CKMT1A	663290000	INFINITE
Q14CX7	N-alpha-acetyltransferase 25, NatB auxiliary subun	NAA25	661860000	INFINITE
P48449	Lanosterol synthase	LSS	655180000	INFINITE
Q9H5K3	Protein O-mannose kinase	POMK	649040000	INFINITE
P55039	Developmentally-regulated GTP-binding protein 2	DRG2	647350000	INFINITE
Q08752	Peptidyl-prolyl cis-trans isomerase D	PPID	645220000	INFINITE
P30519	Heme oxygenase 2	HMOX2	631530000	INFINITE
O43583	Density-regulated protein	DENR	622950000	INFINITE
Q9UHD1	Cysteine and histidine-rich domain-containing prot	CHORDC1	622640000	INFINITE
Q6PI48	Aspartate--tRNA ligase, mitochondrial	DARS2	620090000	INFINITE
Q9BXW7	Cat eye syndrome critical region protein 5	CECR5	619970000	INFINITE
Q06203	Amidophosphoribosyltransferase	PPAT	614120000	INFINITE
Q9UGV2	Protein NDRG3	NDRG3	613280000	INFINITE
P01111	GTPase NRas	NRAS	612830000	INFINITE
Q96EK6	Glucosamine 6-phosphate N-acetyltransferase	GNPNAT1	609860000	INFINITE
Q9Y5K8	V-type proton ATPase subunit D	ATP6V1D	608050000	INFINITE
O00170	AH receptor-interacting protein	AIP	605310000	INFINITE
O95861	3(2),5-bisphosphate nucleotidase 1	BPNT1	602410000	INFINITE
Q9H4A6	Golgi phosphoprotein 3	GOLPH3	595150000	INFINITE
P54105	Methylosome subunit pICln	CLNS1A	594880000	INFINITE
Q15005	Signal peptidase complex subunit 2	SPCS2	593310000	INFINITE
Q15165	Serum paraoxonase/arylesterase 2	PON2	590890000	INFINITE
P35573	Glycogen debranching enzyme;4-alpha-glucanotra	AGL	588330000	INFINITE
P31937	3-hydroxyisobutyrate dehydrogenase, mitochondr	HIBADH	587950000	INFINITE
Q5TFE4	5-nucleotidase domain-containing protein 1	NT5DC1	586900000	INFINITE
P34949	Mannose-6-phosphate isomerase	MPI	586480000	INFINITE

Q58FF6	Putative heat shock protein HSP 90-beta 4	HSP90AB4P	583960000	INFINITE
O95336	6-phosphogluconolactonase	PGLS	581600000	INFINITE
Q8N8S7	Protein enabled homolog	ENAH	581280000	INFINITE
Q15428	Splicing factor 3A subunit 2	SF3A2	579850000	INFINITE
Q16539	Mitogen-activated protein kinase 14	MAPK14	578690000	INFINITE
P11498	Pyruvate carboxylase, mitochondrial	PC	578430000	INFINITE
Q3ZCM7	Tubulin beta-8 chain	TUBB8	578350000	INFINITE
Q10713	Mitochondrial-processing peptidase subunit alpha	PMPCA	576760000	INFINITE
Q9UH65	Switch-associated protein 70	SWAP70	575680000	INFINITE
Q8N3U4	Cohesin subunit SA-2	STAG2	574540000	INFINITE
Q9NRN7	L-aminoadipate-semialdehyde dehydrogenase-pho	AASDHPPT	572390000	INFINITE
P25325	3-mercaptopyruvate sulfurtransferase	MPST	571740000	INFINITE
Q68EM7	Rho GTPase-activating protein 17	ARHGAP17	563800000	INFINITE
Q9NVS9	Pyridoxine-5-phosphate oxidase	PNPO	561290000	INFINITE
Q13619	Cullin-4A	CUL4A	556820000	INFINITE
Q9BRR6	ADP-dependent glucokinase	ADPGK	555550000	INFINITE
Q9NQH7	Probable Xaa-Pro aminopeptidase 3	XPNPEP3	555220000	INFINITE
P61086	Ubiquitin-conjugating enzyme E2 K	UBE2K	553990000	INFINITE
Q9HB90;Q9	Ras-related GTP-binding protein C;Ras-related GTP	RRAGC;RRAGD	548950000	INFINITE
O14908	PDZ domain-containing protein GIPC1	GIPC1	545630000	INFINITE
Q9H8S9;Q7	MOB kinase activator 1A;MOB kinase activator 1B	MOB1A;MOB1B	539610000	INFINITE
Q9H6Z4	Ran-binding protein 3	RANBP3	538930000	INFINITE
Q6P587	Acylpyruvase FAHD1, mitochondrial	FAHD1	538140000	INFINITE
Q9GZT8	NIF3-like protein 1	NIF3L1	534540000	INFINITE
P10301	Ras-related protein R-Ras	RRAS	531200000	INFINITE
Q2M389	WASH complex subunit 7	KIAA1033	530400000	INFINITE
Q8NBF2	NHL repeat-containing protein 2	NHLRC2	530190000	INFINITE
Q9Y3A6	Transmembrane emp24 domain-containing protein	TMED5	528590000	INFINITE
P21266	Glutathione S-transferase Mu 3	GSTM3	524820000	INFINITE
P53701	Cytochrome c-type heme lyase	HCCS	523010000	INFINITE
Q86X55	Histone-arginine methyltransferase CARM1	CARM1	522240000	INFINITE
Q8WUX9	Charged multivesicular body protein 7	CHMP7	518150000	INFINITE
P41240	Tyrosine-protein kinase CSK	CSK	516070000	INFINITE
A0A0B4J1Y9;A0A0B4J1V6;A0A0B4J1V0		IGHV3-72;IGHV3-7	515680000	INFINITE
Q9Y376	Calcium-binding protein 39	CAB39	510150000	INFINITE
Q9H9A6	Leucine-rich repeat-containing protein 40	LRRC40	508930000	INFINITE
Q9C0B1	Alpha-ketoglutarate-dependent dioxygenase FTO	FTO	508920000	INFINITE
P16930	Fumarylacetoacetase	FAH	506400000	INFINITE
Q641Q2;Q9	WASH complex subunit FAM21A;WASH complex su	FAM21A;FAM21C	505730000	INFINITE
P62873	Guanine nucleotide-binding protein G(I)/G(S)/G(T)	GNB1	503770000	INFINITE
Q92878	DNA repair protein RAD50	RAD50	498830000	INFINITE
P51553	Isocitrate dehydrogenase [NAD] subunit gamma, m	IDH3G	496490000	INFINITE
P01859	Ig gamma-2 chain C region	IGHG2	489920000	INFINITE
Q96Q11	CCA tRNA nucleotidyltransferase 1, mitochondrial	TRNT1	486290000	INFINITE
Q16204	Coiled-coil domain-containing protein 6	CCDC6	485600000	INFINITE
Q15036	Sorting nexin-17	SNX17	483420000	INFINITE
Q9UI26	Importin-11	IPO11	477470000	INFINITE
Q16822	Phosphoenolpyruvate carboxykinase [GTP], mitoch	PCK2	476160000	INFINITE
Q8N6T3	ADP-ribosylation factor GTPase-activating protein	ARFGAP1	474780000	INFINITE
Q9Y673	Dolichyl-phosphate beta-glucosyltransferase	ALG5	472590000	INFINITE

Q4V328	GRIP1-associated protein 1	GRIPAP1	469100000	INFINITE
O95983	Methyl-CpG-binding domain protein 3	MBD3	467690000	INFINITE
Q7Z4H8	KDEL motif-containing protein 2	KDELC2	465650000	INFINITE
Q9P0I2	ER membrane protein complex subunit 3	EMC3	465210000	INFINITE
O15173	Membrane-associated progesterone receptor com	PGRMC2	464440000	INFINITE
Q9NUQ8	ATP-binding cassette sub-family F member 3	ABCF3	463290000	INFINITE
O00273	DNA fragmentation factor subunit alpha	DFFA	460810000	INFINITE
O00629	Importin subunit alpha-3	KPNA4	458400000	INFINITE
Q9Y5Y2	Cytosolic Fe-S cluster assembly factor NUBP2	NUBP2	457800000	INFINITE
Q9UMY4	Sorting nexin-12	SNX12	453290000	INFINITE
Q9NP79	Vacuolar protein sorting-associated protein VTA1 h	VTA1	451380000	INFINITE
Q9NPH2	Inositol-3-phosphate synthase 1	ISYNA1	451080000	INFINITE
Q9UIJ7	GTP:AMP phosphotransferase AK3, mitochondrial	AK3	449490000	INFINITE
P07203	Glutathione peroxidase 1	GPX1	447610000	INFINITE
P52594	Arf-GAP domain and FG repeat-containing protein	AGFG1	446850000	INFINITE
Q15427	Splicing factor 3B subunit 4	SF3B4	443590000	INFINITE
Q16513	Serine/threonine-protein kinase N2	PKN2	442750000	INFINITE
Q9UNN8	Endothelial protein C receptor	PROCR	441270000	INFINITE
Q14165	Malectin	MLEC	441100000	INFINITE
P30046;A6I	D-dopachrome decarboxylase;D-dopachrome deca	DDT;DDTL	439940000	INFINITE
Q7KZ85	Transcription elongation factor SPT6	SUPT6H	439470000	INFINITE
Q13404	Ubiquitin-conjugating enzyme E2 variant 1	UBE2V1	439120000	INFINITE
O15121	Sphingolipid delta(4)-desaturase DES1	DEGS1	436980000	INFINITE
Q86UX7	Fermitin family homolog 3	FERMT3	435780000	INFINITE
Q9UBV2	Protein sel-1 homolog 1	SEL1L	435100000	INFINITE
P03886	NADH-ubiquinone oxidoreductase chain 1	MT-ND1	433410000	INFINITE
Q9Y4E8	Ubiquitin carboxyl-terminal hydrolase 15	USP15	432640000	INFINITE
Q8IZ83	Aldehyde dehydrogenase family 16 member A1	ALDH16A1	431350000	INFINITE
Q9H0A8	COMM domain-containing protein 4	COMMD4	429890000	INFINITE
Q13630	GDP-L-fucose synthase	TSTA3	428510000	INFINITE
Q9H9Q2	COP9 signalosome complex subunit 7b	COPS7B	428110000	INFINITE
O14562	Ubiquitin domain-containing protein UBFD1	UBFD1	426770000	INFINITE
Q9H3K2	Growth hormone-inducible transmembrane protei	GHITM	426500000	INFINITE
Q9NWY4	UPF0609 protein C4orf27	C4orf27	426340000	INFINITE
Q96EK5	KIF1-binding protein	KIAA1279	426270000	INFINITE
O43252	Bifunctional 3-phosphoadenosine 5-phosphosulfat	PAPSS1	425650000	INFINITE
P45954	Short/branched chain specific acyl-CoA dehydroge	ACADSB	424130000	INFINITE
Q13158	FAS-associated death domain protein	FADD	420110000	INFINITE
Q9NRF8	CTP synthase 2	CTPS2	419820000	INFINITE
Q5VW32	BRO1 domain-containing protein BROX	BROX	419510000	INFINITE
Q9P1F3	Costars family protein ABRACL	ABRACL	418290000	INFINITE
Q53T59	HCLS1-binding protein 3	HS1BP3	417600000	INFINITE
P18858	DNA ligase 1	LIG1	417240000	INFINITE
Q6ZRP7	Sulfhydryl oxidase 2	QSOX2	413670000	INFINITE
Q9H2W6	39S ribosomal protein L46, mitochondrial	MRPL46	412190000	INFINITE
Q7Z2Z2	Elongation factor Tu GTP-binding domain-containi	EFTUD1	410540000	INFINITE
P20339	Ras-related protein Rab-5A	RAB5A	407780000	INFINITE
Q86TI2	Dipeptidyl peptidase 9	DPP9	407340000	INFINITE
O95777	U6 snRNA-associated Sm-like protein LSm8	LSM8	406190000	INFINITE
Q9BRA2	Thioredoxin domain-containing protein 17	TXNDC17	403860000	INFINITE

O60739	Eukaryotic translation initiation factor 1b	EIF1B	400990000	INFINITE
P09110	3-ketoacyl-CoA thiolase, peroxisomal	ACAA1	400750000	INFINITE
O15027	Protein transport protein Sec16A	SEC16A	397210000	INFINITE
P30825	High affinity cationic amino acid transporter 1	SLC7A1	393680000	INFINITE
Q9BXL7	Caspase recruitment domain-containing protein 11	CARD11	391560000	INFINITE
O95340	Bifunctional 3-phosphoadenosine 5-phosphosulfat	PAPSS2	391300000	INFINITE
Q8WVY7	Ubiquitin-like domain-containing CTD phosphatase	UBLCP1	388120000	INFINITE
P52732	Kinesin-like protein KIF11	KIF11	384080000	INFINITE
Q96GA7	Serine dehydratase-like	SDSL	382970000	INFINITE
Q96DB5	Regulator of microtubule dynamics protein 1	RMDN1	381910000	INFINITE
Q01581	Hydroxymethylglutaryl-CoA synthase, cytoplasmic	HMGCS1	381280000	INFINITE
P30740	Leukocyte elastase inhibitor	SERPINB1	380330000	INFINITE
Q9BTU6	Phosphatidylinositol 4-kinase type 2-alpha	PI4K2A	379690000	INFINITE
Q9Y371	Endophilin-B1	SH3GLB1	379160000	INFINITE
Q9Y5P6	Mannose-1-phosphate guanyltransferase beta	GMPPB	378390000	INFINITE
Q5T6V5	UPF0553 protein C9orf64	C9orf64	378040000	INFINITE
Q9H8Y8	Golgi reassembly-stacking protein 2	GORASP2	376450000	INFINITE
P0C0L4;P0(	Complement C4-A;Complement C4 beta chain;Con	C4A;C4B	375840000	INFINITE
P50583	Bis(5-nucleosyl)-tetraphosphatase [asymmetrical]	NUDT2	375090000	INFINITE
Q8NFH4	Nucleoporin Nup37	NUP37	373640000	INFINITE
P26440	Isovaleryl-CoA dehydrogenase, mitochondrial	IVD	373500000	INFINITE
Q32P41	tRNA (guanine(37)-N1)-methyltransferase	TRMT5	373420000	INFINITE
O43488	Aflatoxin B1 aldehyde reductase member 2	AKR7A2	373060000	INFINITE
P68402	Platelet-activating factor acetylhydrolase IB subuni	PAFAH1B2	372230000	INFINITE
Q8N335	Glycerol-3-phosphate dehydrogenase 1-like protei	GPD1L	371640000	INFINITE
Q00169	Phosphatidylinositol transfer protein alpha isoform	PITPNA	368940000	INFINITE
Q9NXR7	BRCA1-A complex subunit BRE	BRE	368930000	INFINITE
Q15031	Probable leucine--tRNA ligase, mitochondrial	LARS2	365540000	INFINITE
Q9UJY4	ADP-ribosylation factor-binding protein GGA2	GGA2	365370000	INFINITE
Q96GK7;Q(	Fumarylacetoacetate hydrolase domain-containing	FAHD2A;FAHD2B	365020000	INFINITE
P27144	Adenylate kinase 4, mitochondrial	AK4	364720000	INFINITE
Q9UHA4	Ragulator complex protein LAMTOR3	LAMTOR3	364600000	INFINITE
Q16850	Lanosterol 14-alpha demethylase	CYP51A1	362850000	INFINITE
Q6UW02	Cytochrome P450 20A1	CYP20A1	361710000	INFINITE
Q92882	Osteoclast-stimulating factor 1	OSTF1	361410000	INFINITE
O60678	Protein arginine N-methyltransferase 3	PRMT3	360690000	INFINITE
Q9UNE7	E3 ubiquitin-protein ligase CHIP	STUB1	360080000	INFINITE
O14967	Calmegin	CLGN	359490000	INFINITE
P05166	Propionyl-CoA carboxylase beta chain, mitochondr	PCCB	358800000	INFINITE
Q8NHH9	Atlastin-2	ATL2	358730000	INFINITE
Q8TD19	Serine/threonine-protein kinase Nek9	NEK9	358260000	INFINITE
P84095	Rho-related GTP-binding protein RhoG	RHOG	357960000	INFINITE
Q4VC31	Coiled-coil domain-containing protein 58	CCDC58	354620000	INFINITE
Q86Y82	Syntaxin-12	STX12	354440000	INFINITE
Q14181	DNA polymerase alpha subunit B	POLA2	353870000	INFINITE
Q9NRY5	Protein FAM114A2	FAM114A2	352800000	INFINITE
Q9Y3L5	Ras-related protein Rap-2c	RAP2C	352440000	INFINITE
P63279	SUMO-conjugating enzyme UBC9	UBE2I	352000000	INFINITE
Q2TAL8	Glutamine-rich protein 1	QRICH1	351510000	INFINITE
Q7Z4W1	L-xylulose reductase	DCXR	346600000	INFINITE

Q15042	Rab3 GTPase-activating protein catalytic subunit	RAB3GAP1	344660000	INFINITE
O95571	Persulfide dioxygenase ETHE1, mitochondrial	ETHE1	344030000	INFINITE
P19404	NADH dehydrogenase [ubiquinone] flavoprotein 2,	NDUFV2	342880000	INFINITE
Q5JRA6	Melanoma inhibitory activity protein 3	MIA3	342020000	INFINITE
Q6UVK1	Chondroitin sulfate proteoglycan 4	CSPG4	340510000	INFINITE
Q69YN2	CWF19-like protein 1	CWF19L1	340410000	INFINITE
O95486	Protein transport protein Sec24A	SEC24A	338010000	INFINITE
Q9H910	Hematological and neurological expressed 1-like p	HN1L	337480000	INFINITE
Q32P28	Prolyl 3-hydroxylase 1	LEPRE1	336350000	INFINITE
Q3LXA3	Bifunctional ATP-dependent dihydroxyacetone kin	DAK	335930000	INFINITE
Q9UBP6	tRNA (guanine-N(7)-)-methyltransferase	METTL1	335640000	INFINITE
P27707	Deoxycytidine kinase	DCK	333710000	INFINITE
Q5JTZ9	Alanine--tRNA ligase, mitochondrial	AARS2	333550000	INFINITE
Q0VDF9	Heat shock 70 kDa protein 14	HSPA14	332490000	INFINITE
P82970	High mobility group nucleosome-binding domain-c	HMGN5	331260000	INFINITE
Q8NF37	Lysophosphatidylcholine acyltransferase 1	LPCAT1	330770000	INFINITE
Q13478	Interleukin-18 receptor 1	IL18R1	330730000	INFINITE
Q9UHQ9	NADH-cytochrome b5 reductase 1	CYB5R1	330250000	INFINITE
Q14155	Rho guanine nucleotide exchange factor 7	ARHGEF7	329780000	INFINITE
P49902	Cytosolic purine 5-nucleotidase	NT5C2	329010000	INFINITE
P49366	Deoxyhypusine synthase	DHPS	326590000	INFINITE
O15126	Secretory carrier-associated membrane protein 1	SCAMP1	326310000	INFINITE
Q9BYT8	Neurolysin, mitochondrial	NLN	325950000	INFINITE
Q6L8Q7	2,5-phosphodiesterase 12	PDE12	325190000	INFINITE
P80303	Nucleobindin-2;Nesfatin-1	NUCB2	324740000	INFINITE
Q9NZT2	Opioid growth factor receptor	OGFR	324590000	INFINITE
Q8IYI6	Exocyst complex component 8	EXOC8	322950000	INFINITE
P49642	DNA primase small subunit	PRIM1	322510000	INFINITE
O95487	Protein transport protein Sec24B	SEC24B	321350000	INFINITE
Q6NXE6	Armadillo repeat-containing protein 6	ARMC6	319360000	INFINITE
Q9H2P9	Diphthine synthase	DPH5	319050000	INFINITE
P98082	Disabled homolog 2	DAB2	318230000	INFINITE
Q96B97	SH3 domain-containing kinase-binding protein 1	SH3KBP1	318010000	INFINITE
Q9HD20	Manganese-transporting ATPase 13A1	ATP13A1	317340000	INFINITE
O00401	Neural Wiskott-Aldrich syndrome protein	WASL	316260000	INFINITE
Q12846	Syntaxin-4	STX4	315240000	INFINITE
Q8TF05	Serine/threonine-protein phosphatase 4 regulator	PPP4R1	309600000	INFINITE
Q9NQ88	Fructose-2,6-bisphosphatase TIGAR	TIGAR	308080000	INFINITE
Q9H9T3	Elongator complex protein 3	ELP3	307860000	INFINITE
Q9BW91	ADP-ribose pyrophosphatase, mitochondrial	NUDT9	304270000	INFINITE
Q9H832	Ubiquitin-conjugating enzyme E2 Z	UBE2Z	303490000	INFINITE
Q9NVI1	Fanconi anemia group I protein	FANCI	303170000	INFINITE
Q9NPA0	ER membrane protein complex subunit 7	EMC7	302710000	INFINITE
O60826	Coiled-coil domain-containing protein 22	CCDC22	302120000	INFINITE
Q96RQ3	Methylcrotonoyl-CoA carboxylase subunit alpha, m	MCCC1	298770000	INFINITE
O94760	N(G),N(G)-dimethylarginine dimethylaminohydrola	DDAH1	298310000	INFINITE
Q92747	Actin-related protein 2/3 complex subunit 1A	ARPC1A	294980000	INFINITE
Q13451	Peptidyl-prolyl cis-trans isomerase FKBP5;Peptidyl-	FKBP5	294790000	INFINITE
Q96PZ0	Pseudouridylate synthase 7 homolog	PUS7	294240000	INFINITE
Q16706	Alpha-mannosidase 2	MAN2A1	293320000	INFINITE

Q9HBM1	Kinetochore protein Spc25	SPC25	293010000	INFINITE
Q9ULC3	Ras-related protein Rab-23	RAB23	291190000	INFINITE
Q969T9	WW domain-binding protein 2	WBP2	291160000	INFINITE
Q96PD2	Discoidin, CUB and LCCL domain-containing protei	DCBLD2	290810000	INFINITE
Q96A49	Synapse-associated protein 1	SYAP1	288860000	INFINITE
Q92530	Proteasome inhibitor PI31 subunit	PSMF1	288040000	INFINITE
Q3YEC7	Rab-like protein 6	RABL6	287960000	INFINITE
Q96C86	m7GpppX diphosphatase	DCPS	287560000	INFINITE
Q8WW59	SPRY domain-containing protein 4	SPRYD4	287300000	INFINITE
Q16762	Thiosulfate sulfurtransferase	TST	285650000	INFINITE
Q9UBQ0	Vacuolar protein sorting-associated protein 29	VPS29	285100000	INFINITE
Q9UBC2	Epidermal growth factor receptor substrate 15-like	EPS15L1	284970000	INFINITE
P37235	Hippocalcin-like protein 1	HPCAL1	284780000	INFINITE
Q6P179	Endoplasmic reticulum aminopeptidase 2	ERAP2	284620000	INFINITE
O95273	Cyclin-D1-binding protein 1	CCNDBP1	283670000	INFINITE
P51151	Ras-related protein Rab-9A	RAB9A	280370000	INFINITE
Q04206	Transcription factor p65	RELA	277340000	INFINITE
P13984	General transcription factor IIF subunit 2	GTF2F2	275730000	INFINITE
P10321;P01	HLA class I histocompatibility antigen, Cw-7 alpha c	HLA-C;HLA-H	274980000	INFINITE
Q8IWB7	WD repeat and FYVE domain-containing protein 1	WDFY1	274940000	INFINITE
O95630	STAM-binding protein	STAMBP	274070000	INFINITE
Q9NS87	Kinesin-like protein KIF15	KIF15	274040000	INFINITE
Q9Y6M9	NADH dehydrogenase [ubiquinone] 1 beta subcom	NDUFB9	273630000	INFINITE
O75352	Mannose-P-dolichol utilization defect 1 protein	MPDU1	273270000	INFINITE
Q9NQR4	Omega-amidase NIT2	NIT2	272180000	INFINITE
O43676	NADH dehydrogenase [ubiquinone] 1 beta subcom	NDUFB3	272020000	INFINITE
Q7Z4H3	HD domain-containing protein 2	HDDC2	271770000	INFINITE
Q9HCN4	GPN-loop GTPase 1	GPN1	271740000	INFINITE
O14787	Transportin-2	TNPO2	271670000	INFINITE
Q9H9P8	L-2-hydroxyglutarate dehydrogenase, mitochondri	L2HGDH	270410000	INFINITE
Q9Y320	Thioredoxin-related transmembrane protein 2	TMX2	269410000	INFINITE
P15374	Ubiquitin carboxyl-terminal hydrolase isozyme L3	UCHL3	269340000	INFINITE
Q9P0S9	Transmembrane protein 14C	TMEM14C	268900000	INFINITE
P62942	Peptidyl-prolyl cis-trans isomerase FKBP1A	FKBP1A	265980000	INFINITE
Q96S66	Chloride channel CLIC-like protein 1	CLCC1	264440000	INFINITE
Q16740	ATP-dependent Clp protease proteolytic subunit, r	CLPP	264170000	INFINITE
Q02252	Methylmalonate-semialdehyde dehydrogenase [ac	ALDH6A1	263720000	INFINITE
Q12996	Cleavage stimulation factor subunit 3	CSTF3	262780000	INFINITE
Q9NVV0	Trimeric intracellular cation channel type B	TMEM38B	261880000	INFINITE
Q92917	G patch domain and KOW motifs-containing protei	GPKOW	261350000	INFINITE
Q6PD62	RNA polymerase-associated protein CTR9 homolog	CTR9	261010000	INFINITE
P48507	Glutamate--cysteine ligase regulatory subunit	GCLM	258870000	INFINITE
Q9NR19	Acetyl-coenzyme A synthetase, cytoplasmic	ACSS2	257820000	INFINITE
Q13043	Serine/threonine-protein kinase 4;Serine/threonin	STK4	256110000	INFINITE
Q9GZP9	Derlin-2	DERL2	255390000	INFINITE
Q13363	C-terminal-binding protein 1	CTBP1	254810000	INFINITE
Q9Y697	Cysteine desulfurase, mitochondrial	NFS1	253100000	INFINITE
Q14160	Protein scribble homolog	SCRIB	253010000	INFINITE
O15091	Mitochondrial ribonuclease P protein 3	KIAA0391	246830000	INFINITE
P56211	cAMP-regulated phosphoprotein 19	ARPP19	246370000	INFINITE

Q9NY27	Serine/threonine-protein phosphatase 4 regulator	PPP4R2	245710000	INFINITE
Q9Y6I3	Epsin-1	EPN1	245280000	INFINITE
P78330	Phosphoserine phosphatase	PSPH	245210000	INFINITE
Q9H2J4	Phosducin-like protein 3	PDCL3	245100000	INFINITE
Q9H0R6	Glutamyl-tRNA(Gln) amidotransferase subunit A, n	QRSL1	245000000	INFINITE
Q04941	Proteolipid protein 2	PLP2	244800000	INFINITE
Q6XZF7	Dynamin-binding protein	DNMBP	244660000	INFINITE
Q9NRX4	14 kDa phosphohistidine phosphatase	PHPT1	244650000	INFINITE
O43169	Cytochrome b5 type B	CYB5B	243160000	INFINITE
Q9NZD8	Maspardin	SPG21	242990000	INFINITE
O94855	Protein transport protein Sec24D	SEC24D	242630000	INFINITE
P05089	Arginase-1	ARG1	242350000	INFINITE
Q8NA56	Tetratricopeptide repeat protein 29	TTC29	242190000	INFINITE
Q9Y6Y0	Influenza virus NS1A-binding protein	IVNS1ABP	241230000	INFINITE
Q9UPU5	Ubiquitin carboxyl-terminal hydrolase 24	USP24	240270000	INFINITE
Q0VDG4	Secernin-3	SCRN3	240080000	INFINITE
Q08209	Serine/threonine-protein phosphatase 2B catalytic	PPP3CA	239840000	INFINITE
Q12929	Epidermal growth factor receptor kinase substrate	EPS8	239130000	INFINITE
P60033	CD81 antigen	CD81	239040000	INFINITE
P61129	Zinc finger CCCH domain-containing protein 6	ZC3H6	238630000	INFINITE
Q9BW83	Intraflagellar transport protein 27 homolog	IFT27	236860000	INFINITE
Q8IYB8	ATP-dependent RNA helicase SUPV3L1, mitochond	SUPV3L1	236800000	INFINITE
Q96L92	Sorting nexin-27	SNX27	236290000	INFINITE
O95155	Ubiquitin conjugation factor E4 B	UBE4B	235710000	INFINITE
Q96J02	E3 ubiquitin-protein ligase Itchy homolog	ITCH	233930000	INFINITE
O95563	Mitochondrial pyruvate carrier 2	MPC2	233880000	INFINITE
Q14653	Interferon regulatory factor 3	IRF3	233790000	INFINITE
Q8NFV4	Alpha/beta hydrolase domain-containing protein 1	ABHD11	231790000	INFINITE
Q9Y3D6	Mitochondrial fission 1 protein	FIS1	231220000	INFINITE
Q5TC12	ATP synthase mitochondrial F1 complex assembly	ATPAF1	229600000	INFINITE
A0A0G2JM	HLA class II histocompatibility antigen, DR alpha ch	HLA-DRA;HLA-DQ/	229500000	INFINITE
Q9NUY8	TBC1 domain family member 23	TBC1D23	228790000	INFINITE
P42892	Endothelin-converting enzyme 1	ECE1	228320000	INFINITE
P22570	NADPH:adrenodoxin oxidoreductase, mitochondri	FDXR	228140000	INFINITE
Q9H074	Polyadenylate-binding protein-interacting protein	PAIP1	228050000	INFINITE
Q8NHP8	Putative phospholipase B-like 2;Putative phospholi	PLBD2	227360000	INFINITE
Q9BXK5	Bcl-2-like protein 13	BCL2L13	224980000	INFINITE
Q4KWH8	1-phosphatidylinositol 4,5-bisphosphate phosphoc	PLCH1	224580000	INFINITE
Q92990	Glomulin	GLMN	224310000	INFINITE
P26232	Catenin alpha-2	CTNNA2	223800000	INFINITE
P56378	6.8 kDa mitochondrial proteolipid	MP68	222930000	INFINITE
Q7Z7A4	PX domain-containing protein kinase-like protein	PXK	222730000	INFINITE
Q96EY8	Cob(I)yrinic acid a,c-diamide adenosyltransferase,	MMAB	222440000	INFINITE
O14777	Kinetochore protein NDC80 homolog	NDC80	221780000	INFINITE
Q5T6F2	Ubiquitin-associated protein 2	UBAP2	221630000	INFINITE
Q8IVL6	Prolyl 3-hydroxylase 3	LEPREL2	221040000	INFINITE
P11171	Protein 4.1	EPB41	220480000	INFINITE
Q9Y606	tRNA pseudouridine synthase A, mitochondrial	PUS1	220300000	INFINITE
Q14240	Eukaryotic initiation factor 4A-II;Eukaryotic initiatic	EIF4A2	219370000	INFINITE
Q12769	Nuclear pore complex protein Nup160	NUP160	219330000	INFINITE

P07947;P0(	Tyrosine-protein kinase Yes;Tyrosine-protein kinas	YES1;FYN	219140000	INFINITE
Q12972	Nuclear inhibitor of protein phosphatase 1;Activat	PPP1R8	216890000	INFINITE
O60784	Target of Myb protein 1	TOM1	216430000	INFINITE
Q9H267	Vacuolar protein sorting-associated protein 33B	VPS33B	216340000	INFINITE
Q8N0U8	Vitamin K epoxide reductase complex subunit 1-lik	VKORC1L1	216150000	INFINITE
Q9UFN0	Protein NipSnap homolog 3A	NIPSNAP3A	215400000	INFINITE
Q676U5	Autophagy-related protein 16-1	ATG16L1	214720000	INFINITE
P56589	Peroxisomal biogenesis factor 3	PEX3	214710000	INFINITE
Q9P2R7	Succinyl-CoA ligase [ADP-forming] subunit beta, m	SUCLA2	214530000	INFINITE
Q07812	Apoptosis regulator BAX	BAX	214440000	INFINITE
Q5SRE5	Nucleoporin NUP188 homolog	NUP188	214170000	INFINITE
Q9BXB4	Oxysterol-binding protein-related protein 11	OSBPL11	214090000	INFINITE
Q9P2T1	GMP reductase 2	GMPR2	213780000	INFINITE
Q96K76	Ubiquitin carboxyl-terminal hydrolase 47	USP47	213650000	INFINITE
Q96RS6	NudC domain-containing protein 1	NUDCD1	213580000	INFINITE
Q13636	Ras-related protein Rab-31	RAB31	213370000	INFINITE
Q5T0F9	Coiled-coil and C2 domain-containing protein 1B	CC2D1B	213090000	INFINITE
Q9BQC3	Diphthamide biosynthesis protein 2	DPH2	212590000	INFINITE
Q96C90	Protein phosphatase 1 regulatory subunit 14B	PPP1R14B	211130000	INFINITE
Q5T2E6	UPF0668 protein C10orf76	C10orf76	210900000	INFINITE
Q9BWH6	RNA polymerase II-associated protein 1	RPAP1	210770000	INFINITE
P29992	Guanine nucleotide-binding protein subunit alpha-	GNA11	210320000	INFINITE
Q8WWC4	Uncharacterized protein C2orf47, mitochondrial	C2orf47	210280000	INFINITE
Q92466	DNA damage-binding protein 2	DDB2	210240000	INFINITE
P48723	Heat shock 70 kDa protein 13	HSPA13	210050000	INFINITE
P51809	Vesicle-associated membrane protein 7	VAMP7	206460000	INFINITE
Q9BPU6	Dihydropyrimidinase-related protein 5	DPYSL5	206220000	INFINITE
P60520	Gamma-aminobutyric acid receptor-associated prc	GABARAPL2	205800000	INFINITE
Q6NZ67;Q6	Mitotic-spindle organizing protein 2B;Mitotic-spinc	MZT2B;MZT2A	205450000	INFINITE
Q13325	Interferon-induced protein with tetratricopeptide	IFIT5	205210000	INFINITE
Q14997	Proteasome activator complex subunit 4	PSME4	204530000	INFINITE
O00291	Huntingtin-interacting protein 1	HIP1	203710000	INFINITE
P46199	Translation initiation factor IF-2, mitochondrial	MTIF2	203520000	INFINITE
Q92692	Nectin-2	PVRL2	202250000	INFINITE
P49418	Amphiphysin	AMPH	201920000	INFINITE
Q6P1J9	Parafibromin	CDC73	200240000	INFINITE
Q7KZN9	Cytochrome c oxidase assembly protein COX15 hoi	COX15	200230000	INFINITE
O75368	SH3 domain-binding glutamic acid-rich-like protein	SH3BGRL	199980000	INFINITE
P62312	U6 snRNA-associated Sm-like protein LSm6	LSM6	199740000	INFINITE
O15127	Secretory carrier-associated membrane protein 2	SCAMP2	199460000	INFINITE
O14920	Inhibitor of nuclear factor kappa-B kinase subunit l	IKBKB	198900000	INFINITE
Q96D71	RalBP1-associated Eps domain-containing protein :	REPS1	198200000	INFINITE
Q12768	WASH complex subunit strumpellin	KIAA0196	198030000	INFINITE
Q16563	Synaptophysin-like protein 1	SYPL1	198020000	INFINITE
P38571	Lysosomal acid lipase/cholesteryl ester hydrolase	LIPA	197390000	INFINITE
Q96KA5	Cleft lip and palate transmembrane protein 1-like (	CLPTM1L	197320000	INFINITE
Q96PU8	Protein quaking	QKI	196460000	INFINITE
P61225	Ras-related protein Rap-2b	RAP2B	196160000	INFINITE
Q14139	Ubiquitin conjugation factor E4 A	UBE4A	195860000	INFINITE
Q16644	MAP kinase-activated protein kinase 3	MAPKAPK3	195850000	INFINITE

Q8N668	COMM domain-containing protein 1	COMMD1	194470000	INFINITE
Q9Y5P4	Collagen type IV alpha-3-binding protein	COL4A3BP	194250000	INFINITE
Q8TCD5	5(3)-deoxyribonucleotidase, cytosolic type	NT5C	193690000	INFINITE
P04637	Cellular tumor antigen p53	TP53	192030000	INFINITE
P42126	Enoyl-CoA delta isomerase 1, mitochondrial	ECI1	191880000	INFINITE
Q13425	Beta-2-syntrophin	SNTB2	191740000	INFINITE
P42330	Aldo-keto reductase family 1 member C3	AKR1C3	190580000	INFINITE
Q8IXH7	Negative elongation factor C/D	NELFCD	189980000	INFINITE
Q9HD42	Charged multivesicular body protein 1a	CHMP1A	189710000	INFINITE
Q9Y263	Phospholipase A-2-activating protein	PLAA	188960000	INFINITE
Q9GZT4	Serine racemase	SRR	188660000	INFINITE
P43003	Excitatory amino acid transporter 1	SLC1A3	188540000	INFINITE
Q9BUI4	DNA-directed RNA polymerase III subunit RPC3	POLR3C	187850000	INFINITE
Q9UNI6	Dual specificity protein phosphatase 12	DUSP12	187690000	INFINITE
P51003	Poly(A) polymerase alpha	PAPOLA	187330000	INFINITE
Q5T447	E3 ubiquitin-protein ligase HECTD3	HECTD3	187220000	INFINITE
Q13576	Ras GTPase-activating-like protein IQGAP2	IQGAP2	186860000	INFINITE
P40123	Adenylyl cyclase-associated protein 2	CAP2	186070000	INFINITE
Q6P1N9	Putative deoxyribonuclease TATDN1	TATDN1	185780000	INFINITE
P57740	Nuclear pore complex protein Nup107	NUP107	185730000	INFINITE
Q6UXV4	MICOS complex subunit MIC27	APOOL	183810000	INFINITE
O95302	Peptidyl-prolyl cis-trans isomerase FKBP9	FKBP9	183600000	INFINITE
Q7Z7H5	Transmembrane emp24 domain-containing protei	TMED4	183390000	INFINITE
Q9NR09	Baculoviral IAP repeat-containing protein 6	BIRC6	183210000	INFINITE
Q9P265	Disco-interacting protein 2 homolog B	DIP2B	182660000	INFINITE
O95372	Acyl-protein thioesterase 2	LYPLA2	181530000	INFINITE
P23786	Carnitine O-palmitoyltransferase 2, mitochondrial	CPT2	180300000	INFINITE
P29353	SHC-transforming protein 1	SHC1	180240000	INFINITE
Q99614	Tetratricopeptide repeat protein 1	TTC1	180190000	INFINITE
P60510	Serine/threonine-protein phosphatase 4 catalytic s	PPP4C	179580000	INFINITE
P06756	Integrin alpha-V;Integrin alpha-V heavy chain;Integ	ITGAV	179450000	INFINITE
P16219	Short-chain specific acyl-CoA dehydrogenase, mito	ACADS	177800000	INFINITE
Q5T440	Putative transferase CAF17, mitochondrial	IBA57	177440000	INFINITE
Q7LBC6	Lysine-specific demethylase 3B	KDM3B	177310000	INFINITE
Q9Y3I1	F-box only protein 7	FBXO7	177250000	INFINITE
P57076	UPF0769 protein C21orf59	C21orf59	177110000	INFINITE
Q9P2W9	Syntaxin-18	STX18	175020000	INFINITE
Q8WZ82	Ovarian cancer-associated gene 2 protein	OVCA2	174640000	INFINITE
Q8WU79	Stromal membrane-associated protein 2	SMAP2	174360000	INFINITE
P82094	TATA element modulatory factor	TMF1	173310000	INFINITE
P14859	POU domain, class 2, transcription factor 1	POU2F1	173180000	INFINITE
O95219	Sorting nexin-4	SNX4	173050000	INFINITE
P07311	Acylphosphatase-1	ACYP1	171420000	INFINITE
Q96ER3	Protein SAAL1	SAAL1	171160000	INFINITE
Q8N6H7	ADP-ribosylation factor GTPase-activating protein 2	ARFGAP2	170790000	INFINITE
Q9NRL2	Bromodomain adjacent to zinc finger domain prote	BAZ1A	170330000	INFINITE
O75880	Protein SCO1 homolog, mitochondrial	SCO1	168330000	INFINITE
P63313	Thymosin beta-10	TMSB10	168030000	INFINITE
Q04721	Neurogenic locus notch homolog protein 2;Notch 2	NOTCH2	167750000	INFINITE
Q9BXR0	Queuine tRNA-ribosyltransferase	QTRT1	167390000	INFINITE

O14957	Cytochrome b-c1 complex subunit 10	UQCR11	167360000	INFINITE
P80297;P02	Metallothionein-1X;Metallothionein-2;Metallothio	MT1X;MT2A;MT1(	166950000	INFINITE
Q9UIQ6	Leucyl-cystinyl aminopeptidase;Leucyl-cystinyl ami	LNPEP	166940000	INFINITE
Q9UGI8	Testin	TES	166800000	INFINITE
Q99595	Mitochondrial import inner membrane translocase	TIMM17A	166790000	INFINITE
P49005	DNA polymerase delta subunit 2	POLD2	166570000	INFINITE
Q8NBU5	ATPase family AAA domain-containing protein 1	ATAD1	165800000	INFINITE
O75718	Cartilage-associated protein	CRTAP	165730000	INFINITE
P31749	RAC-alpha serine/threonine-protein kinase	AKT1	165610000	INFINITE
Q9H0B6	Kinesin light chain 2	KLC2	165430000	INFINITE
P26572	Alpha-1,3-mannosyl-glycoprotein 2-beta-N-acetylg	MGAT1	165080000	INFINITE
Q8N543	Prolyl 3-hydroxylase OGFOD1	OGFOD1	164680000	INFINITE
Q96I15	Selenocysteine lyase	SCLY	164150000	INFINITE
O95140	Mitofusin-2	MFN2	164100000	INFINITE
O15400	Syntaxin-7	STX7	164070000	INFINITE
Q9Y6I9	Testis-expressed sequence 264 protein	TEX264	163780000	INFINITE
P31151	Protein S100-A7	S100A7	163760000	INFINITE
O75792	Ribonuclease H2 subunit A	RNASEH2A	163540000	INFINITE
Q9Y3A3	MOB-like protein phocein	MOB4	163530000	INFINITE
Q6S8J3;A5A	POTE ankyrin domain family member E;POTE anky	POTEE;POTEF;POT	163310000	INFINITE
P52294	Importin subunit alpha-5;Importin subunit alpha-5	KPNA1	163090000	INFINITE
Q3SXM5	Inactive hydroxysteroid dehydrogenase-like protei	HSDL1	162330000	INFINITE
Q8NBQ5	Estradiol 17-beta-dehydrogenase 11	HSD17B11	161870000	INFINITE
Q15041	ADP-ribosylation factor-like protein 6-interacting p	ARL6IP1	161710000	INFINITE
Q93034	Cullin-5	CUL5	161240000	INFINITE
Q16774	Guanylate kinase	GUK1	160980000	INFINITE
O60306	Intron-binding protein aquarius	AQR	160570000	INFINITE
Q9UJY5	ADP-ribosylation factor-binding protein GGA1	GGA1	160570000	INFINITE
P33908	Mannosyl-oligosaccharide 1,2-alpha-mannosidase	MAN1A1	158690000	INFINITE
Q9UK45	U6 snRNA-associated Sm-like protein LSm7	LSM7	158490000	INFINITE
Q9BUB7	Transmembrane protein 70, mitochondrial	TMEM70	158460000	INFINITE
P62256	Ubiquitin-conjugating enzyme E2 H	UBE2H	158430000	INFINITE
P10253	Lysosomal alpha-glucosidase;76 kDa lysosomal alp	GAA	158430000	INFINITE
A0A0G2JRQ6			158410000	INFINITE
Q96S52	GPI transamidase component PIG-S	PIGS	158320000	INFINITE
P98170	E3 ubiquitin-protein ligase XIAP	XIAP	157970000	INFINITE
Q9UNP9	Peptidyl-prolyl cis-trans isomerase E	PPIE	157890000	INFINITE
Q9UP83	Conserved oligomeric Golgi complex subunit 5	COG5	157510000	INFINITE
Q08188	Protein-glutamine gamma-glutamyltransferase E;P	TGM3	157500000	INFINITE
O43913	Origin recognition complex subunit 5	ORC5	157220000	INFINITE
P09884	DNA polymerase alpha catalytic subunit	POLA1	156870000	INFINITE
P51948	CDK-activating kinase assembly factor MAT1	MNAT1	156660000	INFINITE
Q9Y2D4	Exocyst complex component 6B	EXOC6B	156520000	INFINITE
Q9HBH5	Retinol dehydrogenase 14	RDH14	155960000	INFINITE
O43149	Zinc finger ZZ-type and EF-hand domain-containing	ZZEF1	155720000	INFINITE
Q14149	MORC family CW-type zinc finger protein 3	MORC3	155530000	INFINITE
Q16134	Electron transfer flavoprotein-ubiquinone oxidore	ETFDH	155170000	INFINITE
Q9Y276	Mitochondrial chaperone BCS1	BCS1L	154870000	INFINITE
Q6YP21	Kynurenine--oxoglutarate transaminase 3	CCBL2	154870000	INFINITE
Q9Y2I1	Nischarin	NISCH	154690000	INFINITE

Q8IZ81	ELMO domain-containing protein 2	ELMOD2	154590000	INFINITE
Q9BZV1	UBX domain-containing protein 6	UBXN6	154140000	INFINITE
P34896	Serine hydroxymethyltransferase, cytosolic	SHMT1	153900000	INFINITE
Q99797	Mitochondrial intermediate peptidase	MIPEP	153320000	INFINITE
Q9NPF4	Probable tRNA N6-adenosine threonylcarbamoyltr	OSGEP	153000000	INFINITE
Q9Y4E6	WD repeat-containing protein 7	WDR7	152960000	INFINITE
Q9NRG0	Chromatin accessibility complex protein 1	CHRAC1	152410000	INFINITE
Q00013	55 kDa erythrocyte membrane protein	MPP1	152220000	INFINITE
Q8WUH6	Transmembrane protein 263	TMEM263	152190000	INFINITE
P42226	Signal transducer and activator of transcription 6	STAT6	151950000	INFINITE
Q96F07	Cytoplasmic FMR1-interacting protein 2	CYFIP2	151720000	INFINITE
P48426	Phosphatidylinositol 5-phosphate 4-kinase type-2 a	PIP4K2A	151700000	INFINITE
Q99627	COP9 signalosome complex subunit 8	COPS8	151540000	INFINITE
P42566	Epidermal growth factor receptor substrate 15	EPS15	151440000	INFINITE
Q3SY69	Mitochondrial 10-formyltetrahydrofolate dehydrog	ALDH1L2	151350000	INFINITE
Q8N5M4	Tetratricopeptide repeat protein 9C	TTC9C	151300000	INFINITE
P62834	Ras-related protein Rap-1A	RAP1A	151240000	INFINITE
Q9Y6A5	Transforming acidic coiled-coil-containing protein 3	TACC3	150870000	INFINITE
Q9NRG1	Phosphoribosyltransferase domain-containing prot	PRTFDC1	150180000	INFINITE
Q8N8N7	Prostaglandin reductase 2	PTGR2	149520000	INFINITE
O75608	Acyl-protein thioesterase 1	LYPLA1	149200000	INFINITE
Q9BV57	1,2-dihydroxy-3-keto-5-methylthiopentene dioxyge	ADI1	148040000	INFINITE
Q13017	Rho GTPase-activating protein 5	ARHGAP5	147900000	INFINITE
O14653	Golgi SNAP receptor complex member 2	GOSR2	147590000	INFINITE
Q8NEU8	DCC-interacting protein 13-beta	APPL2	147380000	INFINITE
O14548	Cytochrome c oxidase subunit 7A-related protein,	COX7A2L	146880000	INFINITE
Q14197	Peptidyl-tRNA hydrolase ICT1, mitochondrial	ICT1	146440000	INFINITE
Q14126	Desmoglein-2	DSG2	145890000	INFINITE
P49356	Protein farnesyltransferase subunit beta	FNTB	145460000	INFINITE
P16278	Beta-galactosidase	GLB1	145460000	INFINITE
Q9NX74	tRNA-dihydrouridine(20) synthase [NAD(P)+]-like	DUS2	145180000	INFINITE
P12694	2-oxoisovalerate dehydrogenase subunit alpha, mi	BCKDHA	145140000	INFINITE
Q6UWP7	Lysocardiolipin acyltransferase 1	LCLAT1	143920000	INFINITE
Q6PD74	Alpha- and gamma-adaptin-binding protein p34	AAGAB	143200000	INFINITE
Q9UEE9	Craniofacial development protein 1	CFDP1	142930000	INFINITE
O00625	Pirin	PIR	142680000	INFINITE
Q15121	Astrocytic phosphoprotein PEA-15	PEA15	142470000	INFINITE
Q9NZZ3	Charged multivesicular body protein 5	CHMP5	141930000	INFINITE
P27487	Dipeptidyl peptidase 4;Dipeptidyl peptidase 4 mem	DPP4	141330000	INFINITE
Q9UGP4	LIM domain-containing protein 1	LIMD1	141060000	INFINITE
Q15833	Syntaxin-binding protein 2	STXBP2	140990000	INFINITE
Q8WWV3	Reticulon-4-interacting protein 1, mitochondrial	RTN4IP1	140470000	INFINITE
Q9H0C8	Integrin-linked kinase-associated serine/threonine	ILKAP	140150000	INFINITE
Q9BSV6	tRNA-splicing endonuclease subunit Sen34	TSEN34	139980000	INFINITE
Q6IA86	Elongator complex protein 2	ELP2	139910000	INFINITE
Q15102	Platelet-activating factor acetylhydrolase IB subuni	PAFAH1B3	139860000	INFINITE
Q6IAA8	Ragulator complex protein LAMTOR1	LAMTOR1	139740000	INFINITE
P52948	Nuclear pore complex protein Nup98-Nup96;Nucle	NUP98	139650000	INFINITE
Q05397	Focal adhesion kinase 1	PTK2	139290000	INFINITE
Q9NVH1	DnaJ homolog subfamily C member 11	DNAJC11	139200000	INFINITE

Q9UHJ6	Sedoheptulokinase	SHPK		139140000	INFINITE
Q9NPD8	Ubiquitin-conjugating enzyme E2 T	UBE2T		138640000	INFINITE
O43617	Trafficking protein particle complex subunit 3	TRAPPC3		138550000	INFINITE
Q96EY4	Translation machinery-associated protein 16	TMA16		138460000	INFINITE
P36406	E3 ubiquitin-protein ligase TRIM23	TRIM23		138450000	INFINITE
Q8N5M1	ATP synthase mitochondrial F1 complex assembly	ATPAF2		138430000	INFINITE
O75431	Metaxin-2	MTX2		138200000	INFINITE
O43752	Syntaxin-6	STX6		138160000	INFINITE
P04183	Thymidine kinase, cytosolic	TK1		137370000	INFINITE
Q9UID3	Vacuolar protein sorting-associated protein 51 hor	VPS51		136890000	INFINITE
P45877	Peptidyl-prolyl cis-trans isomerase C	PPIC		136880000	INFINITE
P49459	Ubiquitin-conjugating enzyme E2 A	UBE2A		136730000	INFINITE
Q9H4A5	Golgi phosphoprotein 3-like	GOLPH3L		136720000	INFINITE
O43395	U4/U6 small nuclear ribonucleoprotein Prp3	PRPF3		136500000	INFINITE
P20336	Ras-related protein Rab-3A	RAB3A		136460000	INFINITE
O95182	NADH dehydrogenase [ubiquinone] 1 alpha subcor	NDUFA7		136320000	INFINITE
Q9NXV2	BTB/POZ domain-containing protein KCTD5	KCTD5		136020000	INFINITE
A0MZ66	Shootin-1	KIAA1598		135980000	INFINITE
Q8NCN5	Pyruvate dehydrogenase phosphatase regulatory s	PDPR		135960000	INFINITE
Q7Z3J2	UPF0505 protein C16orf62	C16orf62		135780000	INFINITE
O15382	Branched-chain-amino-acid aminotransferase, mit	BCAT2		135370000	INFINITE
O14656	Torsin-1A	TOR1A		135230000	INFINITE
Q96GQ5	RUS1 family protein C16orf58	C16orf58		135200000	INFINITE
Q9P253	Vacuolar protein sorting-associated protein 18 hor	VPS18		134200000	INFINITE
Q2NKX8	DNA excision repair protein ERCC-6-like	ERCC6L		134110000	INFINITE
Q9UL25	Ras-related protein Rab-21	RAB21		133900000	INFINITE
O75150	E3 ubiquitin-protein ligase BRE1B	RNF40		133810000	INFINITE
Q96S55	ATPase WRNIP1	WRNIP1		133700000	INFINITE
Q9BUT1	3-hydroxybutyrate dehydrogenase type 2	BDH2		133600000	INFINITE
Q9P260	LisH domain and HEAT repeat-containing protein K	KIAA1468		133450000	INFINITE
P42356	Phosphatidylinositol 4-kinase alpha	PI4KA		133290000	INFINITE
P10606	Cytochrome c oxidase subunit 5B, mitochondrial	COX5B		133130000	INFINITE
Q9H1B7	Interferon regulatory factor 2-binding protein-like	IRF2BPL		133010000	INFINITE
Q9NS86	LanC-like protein 2	LANCL2		132230000	INFINITE
P27701	CD82 antigen	CD82		132170000	INFINITE
Q6P161	39S ribosomal protein L54, mitochondrial	MRPL54		132160000	INFINITE
Q9BT73	Proteasome assembly chaperone 3	PSMG3		132020000	INFINITE
O60231	Putative pre-mRNA-splicing factor ATP-dependent	DHX16		131940000	INFINITE
P06702	Protein S100-A9	S100A9		131930000	INFINITE
Q9NX47	E3 ubiquitin-protein ligase MARCH5		Mar-05	131280000	INFINITE
Q9Y6N1	Cytochrome c oxidase assembly protein COX11, mi	COX11		130710000	INFINITE
Q9BY42	Protein RTF2 homolog	RTFDC1		130700000	INFINITE
P04424	Argininosuccinate lyase	ASL		130650000	INFINITE
O94966	Ubiquitin carboxyl-terminal hydrolase 19	USP19		129760000	INFINITE
P31751	RAC-beta serine/threonine-protein kinase	AKT2		129170000	INFINITE
Q8TBX8	Phosphatidylinositol 5-phosphate 4-kinase type-2	PIP4K2C		128950000	INFINITE
P55327	Tumor protein D52	TPD52		128350000	INFINITE
Q9UNW1	Multiple inositol polyphosphate phosphatase 1	MINPP1		128230000	INFINITE
Q5VT52	Regulation of nuclear pre-mRNA domain-containin	RPRD2		128180000	INFINITE
P08581	Hepatocyte growth factor receptor	MET		128100000	INFINITE

Q15398	Disks large-associated protein 5	DLGAP5	128080000	INFINITE
Q9H446	RWD domain-containing protein 1	RWDD1	127980000	INFINITE
P42858	Huntingtin	HTT	127370000	INFINITE
P78540	Arginase-2, mitochondrial	ARG2	127180000	INFINITE
O00233	26S proteasome non-ATPase regulatory subunit 9	PSMD9	126970000	INFINITE
Q01415	N-acetylgalactosamine kinase	GALK2	126860000	INFINITE
Q16763	Ubiquitin-conjugating enzyme E2 S	UBE2S	126550000	INFINITE
Q8N9N8	Probable RNA-binding protein EIF1AD	EIF1AD	125860000	INFINITE
P24386	Rab proteins geranylgeranyltransferase componen	CHM	125170000	INFINITE
Q9NVX2	Notchless protein homolog 1	NLE1	125140000	INFINITE
A3KMH1	von Willebrand factor A domain-containing proteir	VWA8	125090000	INFINITE
Q9UQ16	Dynamin-3	DNM3	124900000	INFINITE
O14735	CDP-diacylglycerol--inositol 3-phosphatidyltransfer	CDIPT	124730000	INFINITE
Q5JSH3	WD repeat-containing protein 44	WDR44	124540000	INFINITE
Q9NRK6	ATP-binding cassette sub-family B member 10, mit	ABCB10	124490000	INFINITE
Q15139	Serine/threonine-protein kinase D1	PRKD1	124230000	INFINITE
Q14691	DNA replication complex GINS protein PSF1	GINS1	124230000	INFINITE
Q9H269	Vacuolar protein sorting-associated protein 16 hor	VPS16	124090000	INFINITE
O60830	Mitochondrial import inner membrane translocase	TIMM17B	123760000	INFINITE
Q9NVT9	Armadillo repeat-containing protein 1	ARMC1	123630000	INFINITE
Q13641	Trophoblast glycoprotein	TPBG	123230000	INFINITE
Q9NXR1;Q9	Nuclear distribution protein nudE homolog 1;Nucle	NDE1;NDEL1	123140000	INFINITE
Q86TU7	Histone-lysine N-methyltransferase setd3	SETD3	123020000	INFINITE
A0A024R1F	Translation machinery-associated protein 7	hCG_2014768;TM/	122890000	INFINITE
O95881	Thioredoxin domain-containing protein 12	TXNDC12	122570000	INFINITE
C4AMC7;Q	Putative WAS protein family homolog 3;WAS prote	WASH3P;WASH2P	122430000	INFINITE
Q8TDQ7	Glucosamine-6-phosphate isomerase 2	GNPDA2	122410000	INFINITE
O75976	Carboxypeptidase D	CPD	122120000	INFINITE
Q9BYV8	Centrosomal protein of 41 kDa	CEP41	121420000	INFINITE
P40692	DNA mismatch repair protein Mlh1	MLH1	121290000	INFINITE
Q9BZE9	Tether containing UBX domain for GLUT4	ASPSCR1	120840000	INFINITE
Q13740	CD166 antigen	ALCAM	120740000	INFINITE
Q8IUR7	Armadillo repeat-containing protein 8	ARMC8	120390000	INFINITE
Q9GZN8	UPF0687 protein C20orf27	C20orf27	120370000	INFINITE
Q8TD16	Protein bicaudal D homolog 2	BICD2	120090000	INFINITE
O75496	Geminin	GMNN	120060000	INFINITE
P61916	Epididymal secretory protein E1	NPC2	119960000	INFINITE
Q9Y314	Nitric oxide synthase-interacting protein	NOSIP	119660000	INFINITE
Q96CP2	FLYWCH family member 2	FLYWCH2	119520000	INFINITE
Q0VGL1	Ragulator complex protein LAMTOR4;Ragulator co	LAMTOR4	118980000	INFINITE
Q8TDZ2	Protein-methionine sulfoxide oxidase MICAL1	MICAL1	118880000	INFINITE
Q8WTW3	Conserved oligomeric Golgi complex subunit 1	COG1	118680000	INFINITE
P78318	Immunoglobulin-binding protein 1	IGBP1	118680000	INFINITE
Q9Y4K4	Mitogen-activated protein kinase kinase kinase kin	MAP4K5	118470000	INFINITE
Q86UY8	5-nucleotidase domain-containing protein 3	NT5DC3	118460000	INFINITE
O43660	Pleiotropic regulator 1	PLRG1	118260000	INFINITE
Q92636	Protein FAN	NSMAF	117950000	INFINITE
Q9UM54	Unconventional myosin-VI	MYO6	117670000	INFINITE
O75794	Cell division cycle protein 123 homolog	CDC123	117610000	INFINITE
Q5SY16	Polynucleotide 5-hydroxyl-kinase NOL9	NOL9	117450000	INFINITE

Q9UBN6	Tumor necrosis factor receptor superfamily memb	TNFRSF10D	117170000	INFINITE
O43678	NADH dehydrogenase [ubiquinone] 1 alpha subcor	NDUFA2	116610000	INFINITE
Q05639	Elongation factor 1-alpha 2	EEF1A2	116070000	INFINITE
Q96G28	Cilia- and flagella-associated protein 36	CFAP36	115840000	INFINITE
Q93052	Lipoma-preferred partner	LPP	115670000	INFINITE
P20839	Inosine-5-monophosphate dehydrogenase 1	IMPDH1	115600000	INFINITE
Q9NVG8	TBC1 domain family member 13	TBC1D13	114900000	INFINITE
Q86SZ2	Trafficking protein particle complex subunit 6B	TRAPPC6B	114700000	INFINITE
P40306	Proteasome subunit beta type-10	PSMB10	114680000	INFINITE
Q8N122	Regulatory-associated protein of mTOR	RPTOR	114340000	INFINITE
Q96P48	Arf-GAP with Rho-GAP domain, ANK repeat and PH	ARAP1	113570000	INFINITE
Q9Y3P9	Rab GTPase-activating protein 1	RABGAP1	113520000	INFINITE
P83436	Conserved oligomeric Golgi complex subunit 7	COG7	113290000	INFINITE
P28062	Proteasome subunit beta type-8	PSMB8	113040000	INFINITE
P05106	Integrin beta-3	ITGB3	113000000	INFINITE
Q9P0J1	[Pyruvate dehydrogenase [acetyl-transferring]]-ph	PDP1	112770000	INFINITE
Q7Z434	Mitochondrial antiviral-signaling protein	MAVS	112720000	INFINITE
O00330	Pyruvate dehydrogenase protein X component, mi	PDHX	112670000	INFINITE
Q7Z422	SUZ domain-containing protein 1	SZRD1	112350000	INFINITE
Q9H3H3	UPF0696 protein C11orf68	C11orf68	112260000	INFINITE
P32929	Cystathionine gamma-lyase	CTH	111910000	INFINITE
P29083	General transcription factor IIE subunit 1	GTF2E1	111870000	INFINITE
Q14344	Guanine nucleotide-binding protein subunit alpha-	GNA13	111710000	INFINITE
Q96BW5	Phosphotriesterase-related protein	PTER	111480000	INFINITE
P18583	Protein SON	SON	111440000	INFINITE
Q9UBB9	Tuftelin-interacting protein 11	TFIP11	111360000	INFINITE
Q9NW82	WD repeat-containing protein 70	WDR70	110540000	INFINITE
P61970	Nuclear transport factor 2	NUTF2	108670000	INFINITE
O15020	Spectrin beta chain, non-erythrocytic 2	SPTBN2	108620000	INFINITE
O60637	Tetraspanin-3	TSPAN3	108100000	INFINITE
Q8N108	Mesoderm induction early response protein 1	MIER1	107990000	INFINITE
O75127	Pentatricopeptide repeat-containing protein 1, mit	PTCD1	107280000	INFINITE
Q9UL45	Biogenesis of lysosome-related organelles complex	BLOC1S6	107090000	INFINITE
Q12840	Kinesin heavy chain isoform 5A	KIF5A	106390000	INFINITE
Q5JPH6	Probable glutamate--tRNA ligase, mitochondrial	EARS2	106330000	INFINITE
Q8N1G2	Cap-specific mRNA (nucleoside-2-O-)-methyltransf	CMTR1	106270000	INFINITE
Q9Y2J2	Band 4.1-like protein 3;Band 4.1-like protein 3, N-t	EPB41L3	106110000	INFINITE
Q9HBK9	Arsenite methyltransferase	AS3MT	105570000	INFINITE
Q7Z3E5	LisH domain-containing protein ARMC9	ARMC9	105040000	INFINITE
P13591	Neural cell adhesion molecule 1	NCAM1	104670000	INFINITE
Q8NBN3	Transmembrane protein 87A	TMEM87A	104480000	INFINITE
Q6UW63	KDEL motif-containing protein 1	KDELC1	104270000	INFINITE
O95208	Epsin-2	EPN2	104120000	INFINITE
Q9BUR5	Apolipoprotein O	APOO	103650000	INFINITE
P16383	GC-rich sequence DNA-binding factor 2	GCFC2	103570000	INFINITE
Q13445	Transmembrane emp24 domain-containing protein	TMED1	103560000	INFINITE
P51580	Thiopurine S-methyltransferase	TPMT	103450000	INFINITE
A0A0C4DH67;A0A0C4DH69;A0A075B6S5		IGKV1-8;IGKV1-9;I	103400000	INFINITE
Q00403	Transcription initiation factor IIB	GTF2B	103330000	INFINITE
O75600	2-amino-3-ketobutyrate coenzyme A ligase, mitoch	GCAT	103300000	INFINITE

P50897	Palmitoyl-protein thioesterase 1	PPT1	103200000	INFINITE
Q9UPN9	E3 ubiquitin-protein ligase TRIM33	TRIM33	102970000	INFINITE
P62328	Thymosin beta-4;Hematopoietic system regulatory	TMSB4X	102870000	INFINITE
Q08722	Leukocyte surface antigen CD47	CD47	102830000	INFINITE
P54725	UV excision repair protein RAD23 homolog A	RAD23A	102560000	INFINITE
Q9H7F0	Probable cation-transporting ATPase 13A3	ATP13A3	102450000	INFINITE
P51784	Ubiquitin carboxyl-terminal hydrolase 11	USP11	102450000	INFINITE
O43347	RNA-binding protein Musashi homolog 1	MSI1	102010000	INFINITE
Q9NV88	Integrator complex subunit 9	INTS9	101840000	INFINITE
Q6A1A2;O1	Putative 3-phosphoinositide-dependent protein ki	PDPK2P;PDPK1	101660000	INFINITE
Q9NX62	Inositol monophosphatase 3	IMPAD1	101280000	INFINITE
Q6P3W7	SCY1-like protein 2	SCYL2	101250000	INFINITE
Q96FZ7	Charged multivesicular body protein 6	CHMP6	101190000	INFINITE
Q8N9F7	Glycerophosphodiester phosphodiesterase domair	GDPD1	101090000	INFINITE
O60934	Nibrin	NBN	100910000	INFINITE
P51965	Ubiquitin-conjugating enzyme E2 E1	UBE2E1	100710000	INFINITE
Q14627	Interleukin-13 receptor subunit alpha-2	IL13RA2	100640000	INFINITE
P51649	Succinate-semialdehyde dehydrogenase, mitochor	ALDH5A1	100630000	INFINITE
Q96I51	Williams-Beuren syndrome chromosomal region 1	WBSCR16	100310000	INFINITE
P09001	39S ribosomal protein L3, mitochondrial	MRPL3	99514000	INFINITE
O96013	Serine/threonine-protein kinase PAK 4	PAK4	99374000	INFINITE
O14618	Copper chaperone for superoxide dismutase	CCS	99228000	INFINITE
Q96H20	Vacuolar-sorting protein SNF8	SNF8	99069000	INFINITE
P61803	Dolichyl-diphosphooligosaccharide--protein glycos	DAD1	98915000	INFINITE
Q7L5Y1	Mitochondrial enolase superfamily member 1	ENOSF1	98633000	INFINITE
O95167	NADH dehydrogenase [ubiquinone] 1 alpha subcor	NDUFA3	98381000	INFINITE
Q9Y2R0	Cytochrome c oxidase assembly factor 3 homolog,	COA3	98363000	INFINITE
Q6UX04	Peptidyl-prolyl cis-trans isomerase CWC27 homolo	CWC27	98295000	INFINITE
Q86YN1	Dolichyldiphosphatase 1	DOLPP1	98295000	INFINITE
Q9BVA0	Katanin p80 WD40 repeat-containing subunit B1	KATNB1	98145000	INFINITE
Q969U7	Proteasome assembly chaperone 2	PSMG2	97794000	INFINITE
Q9BTX1	Nucleoporin NDC1	NDC1	97763000	INFINITE
Q07864	DNA polymerase epsilon catalytic subunit A	POLE	97614000	INFINITE
Q9H788	SH2 domain-containing protein 4A	SH2D4A	97596000	INFINITE
Q96CV9	Optineurin	OPTN	97525000	INFINITE
Q03252	Lamin-B2	LMNB2	97463000	INFINITE
O75879	Glutamyl-tRNA(Gln) amidotransferase subunit B, n	GATB	97462000	INFINITE
Q8TB52	F-box only protein 30	FBXO30	97386000	INFINITE
Q8WXE9	Stonin-2	STON2	97258000	INFINITE
O00423	Echinoderm microtubule-associated protein-like 1	EML1	96816000	INFINITE
O95260	Arginyl-tRNA--protein transferase 1	ATE1	96780000	INFINITE
Q5TBB1	Ribonuclease H2 subunit B	RNASEH2B	96560000	INFINITE
Q9H0P0	Cytosolic 5-nucleotidase 3A	NT5C3A	96496000	INFINITE
Q8NCG7	Sn1-specific diacylglycerol lipase beta	DAGLB	96476000	INFINITE
A1A4S6	Rho GTPase-activating protein 10	ARHGAP10	96470000	INFINITE
Q7L592	NADH dehydrogenase [ubiquinone] complex I, ass	NDUFAF7	95927000	INFINITE
Q15628	Tumor necrosis factor receptor type 1-associated [	TRADD	95900000	INFINITE
Q96BP3	Peptidylprolyl isomerase domain and WD repeat-c	PPWD1	95866000	INFINITE
Q9UDX4;O	SEC14-like protein 3;SEC14-like protein 2	SEC14L3;SEC14L2	95841000	INFINITE
Q969N2	GPI transamidase component PIG-T	PIGT	95806000	INFINITE

Q9BVM2	Protein DPCD	DPCD	95688000	INFINITE
Q96EY5	Multivesicular body subunit 12A	MVB12A	95628000	INFINITE
A1X283	SH3 and PX domain-containing protein 2B	SH3PXD2B	95420000	INFINITE
O15056	Synaptojanin-2	SYNJ2	95262000	INFINITE
Q86WB0	Nuclear-interacting partner of ALK	ZC3HC1	95220000	INFINITE
P52758	Ribonuclease UK114	HRSP12	95169000	INFINITE
Q9Y3C8	Ubiquitin-fold modifier-conjugating enzyme 1	UFC1	95168000	INFINITE
Q8IWV7	E3 ubiquitin-protein ligase UBR1	UBR1	95021000	INFINITE
Q86X76	Nitrilase homolog 1	NIT1	95016000	INFINITE
Q99447	Ethanolamine-phosphate cytidylyltransferase	PCYT2	94826000	INFINITE
Q96BJ3	Axin interactor, dorsalization-associated protein	AIDA	94780000	INFINITE
Q8NB37	Parkinson disease 7 domain-containing protein 1	PDDC1	94425000	INFINITE
Q53HC9	Protein TSSC1	TSSC1	93741000	INFINITE
Q9H9C1	Spermatogenesis-defective protein 39 homolog	VIPAS39	93410000	INFINITE
P00414	Cytochrome c oxidase subunit 3	MT-CO3	93362000	INFINITE
O75884	Putative hydrolase RBBP9	RBBP9	93254000	INFINITE
Q8IYS1	Peptidase M20 domain-containing protein 2	PM20D2	93235000	INFINITE
P06865	Beta-hexosaminidase subunit alpha	HEXA	93004000	INFINITE
Q15526	Surfeit locus protein 1	SURF1	92939000	INFINITE
Q9H5N1	Rab GTPase-binding effector protein 2	RABEP2	92858000	INFINITE
P33527	Multidrug resistance-associated protein 1	ABCC1	92755000	INFINITE
Q8TF66	Leucine-rich repeat-containing protein 15	LRRC15	92388000	INFINITE
O15305	Phosphomannomutase 2	PMM2	92052000	INFINITE
Q9HBM6;Q	Transcription initiation factor TFIID subunit 9B;Tra	TAF9B;TAF9	91895000	INFINITE
Q9HCS7	Pre-mRNA-splicing factor SYF1	XAB2	91792000	INFINITE
Q9BTZ2;P0	Dehydrogenase/reductase SDR family member 4;P	DHRS4;DHRS4L1	91507000	INFINITE
O14933	Ubiquitin/ISG15-conjugating enzyme E2 L6	UBE2L6	91153000	INFINITE
O15294	UDP-N-acetylglucosamine--peptide N-acetylglucos	OGT	91055000	INFINITE
Q15843	NEDD8	NEDD8	90901000	INFINITE
O14802	DNA-directed RNA polymerase III subunit RPC1	POLR3A	90876000	INFINITE
P78356	Phosphatidylinositol 5-phosphate 4-kinase type-2 l	PIP4K2B	90266000	INFINITE
Q5VZE5	N-alpha-acetyltransferase 35, NatC auxiliary subun	NAA35	89965000	INFINITE
Q8IYQ7	Threonine synthase-like 1	THNSL1	89667000	INFINITE
O95671	N-acetylserotonin O-methyltransferase-like protei	ASMTL	89379000	INFINITE
P80217	Interferon-induced 35 kDa protein	IFI35	89159000	INFINITE
Q9UFC0	Leucine-rich repeat and WD repeat-containing pro	LRWD1	89072000	INFINITE
Q9NRP0	Oligosaccharyltransferase complex subunit OSTC	OSTC	88912000	INFINITE
O75449	Katanin p60 ATPase-containing subunit A1	KATNA1	88811000	INFINITE
Q9NUT2	ATP-binding cassette sub-family B member 8, mito	ABCB8	88515000	INFINITE
Q96SZ6	CDK5 regulatory subunit-associated protein 1	CDK5RAP1	88104000	INFINITE
O75629	Protein CREG1	CREG1	88056000	INFINITE
Q9H0W8	Protein SMG9	SMG9	87720000	INFINITE
Q9UMR2	ATP-dependent RNA helicase DDX19B	DDX19B	87581000	INFINITE
Q8IV08	Phospholipase D3	PLD3	87501000	INFINITE
P57105	Synaptojanin-2-binding protein	SYNJ2BP	87431000	INFINITE
Q9C040	Tripartite motif-containing protein 2	TRIM2	87402000	INFINITE
P18615	Negative elongation factor E	NELFE	87370000	INFINITE
P61769	Beta-2-microglobulin;Beta-2-microglobulin form pl	B2M	87301000	INFINITE
Q9UGJ0	5-AMP-activated protein kinase subunit gamma-2	PRKAG2	86938000	INFINITE
O95470	Sphingosine-1-phosphate lyase 1	SGPL1	86381000	INFINITE

P42575	Caspase-2;Caspase-2 subunit p18;Caspase-2 subur	CASP2	86292000	INFINITE
Q92930	Ras-related protein Rab-8B	RAB8B	86241000	INFINITE
Q8N2G8	GH3 domain-containing protein	GHDC	85828000	INFINITE
Q9Y223	Bifunctional UDP-N-acetylglucosamine 2-epimeras	GNE	85535000	INFINITE
P0DSN7;A0A075B6S9		IGKV1D-37	85253000	INFINITE
P47929	Galectin-7	LGALS7	85160000	INFINITE
Q8ND76	Cyclin-Y	CCNY	85005000	INFINITE
Q7Z5K2	Wings apart-like protein homolog	WAPAL	84931000	INFINITE
Q9NSK0	Kinesin light chain 4	KLC4	84892000	INFINITE
Q96J01	THO complex subunit 3	THOC3	84841000	INFINITE
O43402	ER membrane protein complex subunit 8	EMC8	84749000	INFINITE
Q86XI8	Uncharacterized protein C19orf68	C19orf68	84623000	INFINITE
Q96BH1	E3 ubiquitin-protein ligase RNF25	RNF25	84552000	INFINITE
Q7Z5L9	Interferon regulatory factor 2-binding protein 2	IRF2BP2	84505000	INFINITE
O43929	Origin recognition complex subunit 4	ORC4	84038000	INFINITE
P22413	Ectonucleotide pyrophosphatase/phosphodiestera	ENPP1	83942000	INFINITE
Q4G0N4	NAD kinase 2, mitochondrial	NADK2	83782000	INFINITE
Q96Q15	Serine/threonine-protein kinase SMG1	SMG1	83693000	INFINITE
Q96EV2	RNA-binding protein 33	RBM33	83618000	INFINITE
Q7Z4G4	tRNA (guanine(10)-N2)-methyltransferase homolo	TRMT11	83497000	INFINITE
P05026	Sodium/potassium-transporting ATPase subunit be	ATP1B1	83448000	INFINITE
Q9HD15	Steroid receptor RNA activator 1	SRA1	83281000	INFINITE
Q9BWS9	Chitinase domain-containing protein 1	CHID1	83252000	INFINITE
O43815	Striatin	STRN	83225000	INFINITE
Q86SF2	N-acetylgalactosaminyltransferase 7	GALNT7	83200000	INFINITE
P23025	DNA repair protein complementing XP-A cells	XPA	83175000	INFINITE
O15431	High affinity copper uptake protein 1	SLC31A1	83083000	INFINITE
Q9BZJ0	Crooked neck-like protein 1	CRNKL1	83049000	INFINITE
P23497	Nuclear autoantigen Sp-100	SP100	82991000	INFINITE
Q8N4V1	Membrane magnesium transporter 1	MMGT1	82853000	INFINITE
Q9Y4P8	WD repeat domain phosphoinositide-interacting p	WIPI2	82186000	INFINITE
P48163	NADP-dependent malic enzyme	ME1	81929000	INFINITE
Q96PU5	E3 ubiquitin-protein ligase NEDD4-like	NEDD4L	81576000	INFINITE
Q9H201	Epsin-3	EPN3	81546000	INFINITE
Q8NBF6	Late secretory pathway protein AVL9 homolog	AVL9	81496000	INFINITE
Q13362	Serine/threonine-protein phosphatase 2A 56 kDa r	PPP2R5C	81234000	INFINITE
Q969G6	Riboflavin kinase	RFK	81209000	INFINITE
Q4G176	Acyl-CoA synthetase family member 3, mitochondr	ACSF3	80713000	INFINITE
Q8NBI5	Solute carrier family 43 member 3	SLC43A3	80548000	INFINITE
Q13642	Four and a half LIM domains protein 1	FHL1	80439000	INFINITE
Q8N4P3	Guanosine-3,5-bis(diphosphate) 3-pyrophosphohy	HDDC3	80305000	INFINITE
Q4G0F5	Vacuolar protein sorting-associated protein 26B	VPS26B	80231000	INFINITE
Q6NYC8	Phostensin	PPP1R18	79719000	INFINITE
Q14141	Septin-6	Sep-06	79643000	INFINITE
Q6P158	Putative ATP-dependent RNA helicase DHX57	DHX57	79457000	INFINITE
Q16877;P1	6-phosphofructo-2-kinase/fructose-2,6-bisphosph	PFKFB4;PFKFB1	79420000	INFINITE
Q9Y4X5	E3 ubiquitin-protein ligase ARIH1	ARIH1	79193000	INFINITE
Q86U90	YrdC domain-containing protein, mitochondrial	YRDC	78935000	INFINITE
Q92504	Zinc transporter SLC39A7	SLC39A7	78807000	INFINITE
Q9BXW9	Fanconi anemia group D2 protein	FANCD2	78644000	INFINITE

Q9UNN5	FAS-associated factor 1	FAF1	78610000	INFINITE
Q96AB3	Isochorismatase domain-containing protein 2, mitc	ISOC2	78440000	INFINITE
Q13610	Periodic tryptophan protein 1 homolog	PWP1	78376000	INFINITE
Q13188	Serine/threonine-protein kinase 3;Serine/threonin	STK3	78173000	INFINITE
Q7LG56	Ribonucleoside-diphosphate reductase subunit M2	RRM2B	77539000	INFINITE
Q9ULH7	MKL/myocardin-like protein 2	MKL2	77527000	INFINITE
P07738	Bisphosphoglycerate mutase	BPGM	77387000	INFINITE
A6NCN2	Putative keratin-87 protein	KRT87P	77219000	INFINITE
Q4G148	Glucoside xylosyltransferase 1	GXYLT1	76886000	INFINITE
Q99836	Myeloid differentiation primary response protein I	MYD88	76779000	INFINITE
P85037	Forkhead box protein K1	FOXK1	76750000	INFINITE
Q9H6V9	UPF0554 protein C2orf43	C2orf43	76402000	INFINITE
P35555	Fibrillin-1	FBN1	76333000	INFINITE
O75027	ATP-binding cassette sub-family B member 7, mito	ABCB7	76093000	INFINITE
Q9Y2W6	Tudor and KH domain-containing protein	TDRKH	75673000	INFINITE
O43567	E3 ubiquitin-protein ligase RNF13	RNF13	75537000	INFINITE
Q9NRL3	Striatin-4	STRN4	75519000	INFINITE
P27105	Erythrocyte band 7 integral membrane protein	STOM	75177000	INFINITE
Q7Z4T9	Protein MAATS1	MAATS1	75135000	INFINITE
Q9BTD8	RNA-binding protein 42	RBM42	74980000	INFINITE
P49768	Presenilin-1;Presenilin-1 NTF subunit;Presenilin-1 (	PSEN1	74683000	INFINITE
Q9Y6G3	39S ribosomal protein L42, mitochondrial	MRPL42	74212000	INFINITE
P14649	Myosin light chain 6B	MYL6B	73912000	INFINITE
Q9NP81	Serine--tRNA ligase, mitochondrial	SARS2	73912000	INFINITE
Q9UJC3	Protein Hook homolog 1	HOOK1	73872000	INFINITE
Q96AX1	Vacuolar protein sorting-associated protein 33A	VPS33A	73778000	INFINITE
Q9H1D9	DNA-directed RNA polymerase III subunit RPC6	POLR3F	73639000	INFINITE
P48651	Phosphatidylserine synthase 1	PTDSS1	73464000	INFINITE
Q12974;Q9	Protein tyrosine phosphatase type IVA 2;Protein ty	PTP4A2;PTP4A1	73256000	INFINITE
Q99747	Gamma-soluble NSF attachment protein	NAPG	73197000	INFINITE
Q9NZN3	EH domain-containing protein 3	EHD3	72911000	INFINITE
Q6NW29	RWD domain-containing protein 4	RWDD4	72880000	INFINITE
P51617	Interleukin-1 receptor-associated kinase 1	IRAK1	72695000	INFINITE
P36639	7,8-dihydro-8-oxoguanine triphosphatase	NUDT1	72437000	INFINITE
Q9UBL3	Set1/Ash2 histone methyltransferase complex sub	ASH2L	72429000	INFINITE
O95166;Q9	Gamma-aminobutyric acid receptor-associated prc	GABARAP;GABAR/	72330000	INFINITE
Q9BUR4	Telomerase Cajal body protein 1	WRAP53	72077000	INFINITE
B9A064;P0	Immunoglobulin lambda-like polypeptide 5;Ig laml	IGLL5;IGLC1	71964000	INFINITE
Q8WXD5	Gem-associated protein 6	GEMIN6	71900000	INFINITE
P18887	DNA repair protein XRCC1	XRCC1	71772000	INFINITE
Q9BWQ6	Protein YIPF2	YIPF2	71606000	INFINITE
P58107	Epiplakin	EPPK1	71557000	INFINITE
Q16718	NADH dehydrogenase [ubiquinone] 1 alpha subcoi	NDUFA5	71298000	INFINITE
Q04726;Q0	Transducin-like enhancer protein 3;Transducin-like	TLE3;TLE2;TLE1;TL	71289000	INFINITE
P04114	Apolipoprotein B-100;Apolipoprotein B-48	APOB	71280000	INFINITE
Q5SWX8	Protein odr-4 homolog	ODR4	71048000	INFINITE
P56377	AP-1 complex subunit sigma-2	AP1S2	71026000	INFINITE
Q9Y2H6	Fibronectin type-III domain-containing protein 3A	FNDC3A	70833000	INFINITE
Q8N111	Cell cycle exit and neuronal differentiation protein	CEND1	70660000	INFINITE
P49840	Glycogen synthase kinase-3 alpha	GSK3A	70408000	INFINITE

Q9NPJ3	Acyl-coenzyme A thioesterase 13;Acyl-coenzyme A	ACOT13	70371000	INFINITE
Q9Y4B6	Protein VPRBP	VPRBP	70309000	INFINITE
Q99996	A-kinase anchor protein 9	AKAP9	69838000	INFINITE
O15075	Serine/threonine-protein kinase DCLK1	DCLK1	69822000	INFINITE
O75419	Cell division control protein 45 homolog	CDC45	69603000	INFINITE
P23511	Nuclear transcription factor Y subunit alpha	NFYA	69564000	INFINITE
P49590	Probable histidine--tRNA ligase, mitochondrial	HARS2	69479000	INFINITE
Q13488	V-type proton ATPase 116 kDa subunit a isoform 3	TCIRG1	69444000	INFINITE
Q96I59	Probable asparagine--tRNA ligase, mitochondrial	NARS2	69422000	INFINITE
P41743	Protein kinase C iota type	PRKCI	69356000	INFINITE
O43493	Trans-Golgi network integral membrane protein 2	TGOLN2	68967000	INFINITE
Q01658	Protein Dr1	DR1	68760000	INFINITE
Q8NI60	Atypical kinase ADCK3, mitochondrial	ADCK3	68683000	INFINITE
Q7Z7K0	COX assembly mitochondrial protein homolog	CMC1	68666000	INFINITE
Q9H3Z4	DnaJ homolog subfamily C member 5	DNAJC5	68482000	INFINITE
P49815	Tuberin	TSC2	68334000	INFINITE
Q96HY7	Probable 2-oxoglutarate dehydrogenase E1 compo	DHTKD1	68155000	INFINITE
Q8NBJ4	Golgi membrane protein 1	GOLM1	68001000	INFINITE
Q13232	Nucleoside diphosphate kinase 3	NME3	67936000	INFINITE
P24821	Tenascin	TNC	67667000	INFINITE
Q9BZD4	Kinetochore protein Nuf2	NUF2	67452000	INFINITE
Q6YHK3	CD109 antigen	CD109	67365000	INFINITE
P04233	HLA class II histocompatibility antigen gamma chai	CD74	67339000	INFINITE
Q9P2D3	HEAT repeat-containing protein 5B	HEATR5B	67338000	INFINITE
Q9Y2L5	Trafficking protein particle complex subunit 8	TRAPPC8	67194000	INFINITE
O14874	[3-methyl-2-oxobutanoate dehydrogenase [lipoam	BCKDK	67094000	INFINITE
Q8WVT3	Trafficking protein particle complex subunit 12	TRAPPC12	66667000	INFINITE
Q8NBT2	Kinetochore protein Spc24	SPC24	66548000	INFINITE
Q5HYK3	2-methoxy-6-polyprenyl-1,4-benzoquinol methylas	COQ5	66527000	INFINITE
P11169;Q8	Solute carrier family 2, facilitated glucose transpor	SLC2A3;SLC2A14	66437000	INFINITE
Q86VR2	Protein FAM134C	FAM134C	66419000	INFINITE
Q5RI15	Cytochrome c oxidase protein 20 homolog	COX20	66343000	INFINITE
P30154	Serine/threonine-protein phosphatase 2A 65 kDa r	PPP2R1B	66107000	INFINITE
Q8NB46	Serine/threonine-protein phosphatase 6 regulator	ANKRD52	66015000	INFINITE
Q96MW1	Coiled-coil domain-containing protein 43	CCDC43	65993000	INFINITE
Q9C0F1	Centrosomal protein of 44 kDa	CEP44	65754000	INFINITE
Q68CQ7	Glycosyltransferase 8 domain-containing protein 1	GLT8D1	65621000	INFINITE
Q9Y3R5	Protein dopey-2	DOPEY2	65598000	INFINITE
Q86TP1	Protein prune homolog	PRUNE	65360000	INFINITE
P15291	Beta-1,4-galactosyltransferase 1;Lactose synthase	B4GALT1	65290000	INFINITE
Q9P013	Spliceosome-associated protein CWC15 homolog	CWC15	65233000	INFINITE
Q13546	Receptor-interacting serine/threonine-protein kina	RIPK1	65181000	INFINITE
P29317	Ephrin type-A receptor 2	EPHA2	65029000	INFINITE
P52735	Guanine nucleotide exchange factor VAV2	VAV2	64982000	INFINITE
Q92845	Kinesin-associated protein 3	KIFAP3	64979000	INFINITE
Q8IZ73	RNA pseudouridylate synthase domain-containing	RPUSD2	64827000	INFINITE
Q15052	Rho guanine nucleotide exchange factor 6	ARHGEF6	64811000	INFINITE
Q9UJ41	Rab5 GDP/GTP exchange factor	RABGEF1	64695000	INFINITE
Q6NUM9	All-trans-retinol 13,14-reductase	RETSAT	64656000	INFINITE
P51808	Dynein light chain Tctex-type 3	DYNLT3	64319000	INFINITE

Q15067	Peroxisomal acyl-coenzyme A oxidase 1	ACOX1	64215000	INFINITE
Q96SY0	von Willebrand factor A domain-containing protein	VWA9	63783000	INFINITE
P49069	Calcium signal-modulating cyclophilin ligand	CAMLG	63477000	INFINITE
Q9Y2Z9	Ubiquinone biosynthesis monooxygenase COQ6, m	COQ6	63278000	INFINITE
Q9UNX4	WD repeat-containing protein 3	WDR3	63250000	INFINITE
Q96GX9	Methylthioribulose-1-phosphate dehydratase	APIP	63180000	INFINITE
Q8TBQ9	Protein kish-A	TMEM167A	63044000	INFINITE
Q7Z6M1	Rab9 effector protein with kelch motifs	RABEPK	62886000	INFINITE
Q9BV79	Trans-2-enoyl-CoA reductase, mitochondrial	MECR	62878000	INFINITE
Q9BPY3	Protein FAM118B	FAM118B	62845000	INFINITE
O75152	Zinc finger CCCH domain-containing protein 11A	ZC3H11A	62657000	INFINITE
O95379	Tumor necrosis factor alpha-induced protein 8	TNFAIP8	62230000	INFINITE
Q9H900	Protein zwilch homolog	ZWILCH	61880000	INFINITE
Q9C035	Tripartite motif-containing protein 5	TRIM5	61801000	INFINITE
Q70CQ2	Ubiquitin carboxyl-terminal hydrolase 34	USP34	61413000	INFINITE
Q53RT3	Retroviral-like aspartic protease 1	ASPRV1	61342000	INFINITE
Q9Y282	Endoplasmic reticulum-Golgi intermediate compar	ERGIC3	61210000	INFINITE
Q86VI3	Ras GTPase-activating-like protein IQGAP3	IQGAP3	61163000	INFINITE
Q5EBL4	RILP-like protein 1	RILPL1	60913000	INFINITE
Q9GZS1	DNA-directed RNA polymerase I subunit RPA49	POLR1E	60779000	INFINITE
Q96BM9	ADP-ribosylation factor-like protein 8A	ARL8A	60648000	INFINITE
Q8WUA7	TBC1 domain family member 22A	TBC1D22A	60616000	INFINITE
Q9UQ53	Alpha-1,3-mannosyl-glycoprotein 4-beta-N-acetylg	MGAT4B	60601000	INFINITE
P49754	Vacuolar protein sorting-associated protein 41 hor	VPS41	60537000	INFINITE
P35813	Protein phosphatase 1A	PPM1A	60479000	INFINITE
Q6PID6	Tetratricopeptide repeat protein 33	TTC33	60392000	INFINITE
Q8N392	Rho GTPase-activating protein 18	ARHGAP18	60151000	INFINITE
Q96TC7	Regulator of microtubule dynamics protein 3	RMDN3	60147000	INFINITE
Q8WWI5	Choline transporter-like protein 1	SLC44A1	60130000	INFINITE
Q7L7V1	Putative pre-mRNA-splicing factor ATP-dependent	DHX32	60122000	INFINITE
Q9BY41	Histone deacetylase 8	HDAC8	60102000	INFINITE
Q9UGJ1	Gamma-tubulin complex component 4	TUBGCP4	60093000	INFINITE
Q9H330	Transmembrane protein 245	TMEM245	60093000	INFINITE
Q14012	Calcium/calmodulin-dependent protein kinase typ	CAMK1	60062000	INFINITE
P05114	Non-histone chromosomal protein HMG-14	HMGN1	60032000	INFINITE
O15111	Inhibitor of nuclear factor kappa-B kinase subunit a	CHUK	59892000	INFINITE
Q9Y5Z4	Heme-binding protein 2	HEBP2	59825000	INFINITE
Q7L266	Isoaspartyl peptidase/L-asparaginase;Isoaspartyl p	ASRGL1	59478000	INFINITE
Q5T160	Probable arginine--tRNA ligase, mitochondrial	RARS2	59328000	INFINITE
Q9NUW8	Tyrosyl-DNA phosphodiesterase 1	TDP1	59213000	INFINITE
Q9Y587	AP-4 complex subunit sigma-1	AP4S1	59033000	INFINITE
P57081	tRNA (guanine-N(7)-)-methyltransferase non-catal	WDR4	59014000	INFINITE
Q9NXE4	Sphingomyelin phosphodiesterase 4	SMPD4	58946000	INFINITE
Q7Z7E8	Ubiquitin-conjugating enzyme E2 Q1	UBE2Q1	58872000	INFINITE
Q9P2B4	CTTNBP2 N-terminal-like protein	CTTNBP2NL	58474000	INFINITE
Q9NRY4	Rho GTPase-activating protein 35	ARHGAP35	58469000	INFINITE
Q8IWA4	Mitofusin-1	MFN1	58395000	INFINITE
Q9NZW5	MAGUK p55 subfamily member 6	MPP6	58301000	INFINITE
O95677	Eyes absent homolog 4	EYA4	58229000	INFINITE
Q92667	A-kinase anchor protein 1, mitochondrial	AKAP1	58215000	INFINITE

O75391	Sperm-associated antigen 7	SPAG7	58208000	INFINITE
Q9Y4K3	TNF receptor-associated factor 6	TRAF6	58195000	INFINITE
Q8IVM0	Coiled-coil domain-containing protein 50	CCDC50	58085000	INFINITE
O60341	Lysine-specific histone demethylase 1A	KDM1A	58035000	INFINITE
Q9UKZ1	CCR4-NOT transcription complex subunit 11	CNOT11	58026000	INFINITE
Q92791	Synaptonemal complex protein SC65	LEPREL4	57913000	INFINITE
Q13823	Nucleolar GTP-binding protein 2	GNL2	57876000	INFINITE
Q15369	Transcription elongation factor B polypeptide 1	TCEB1	57777000	INFINITE
Q6ZRS2	Helicase SRCAP	SRCAP	57692000	INFINITE
Q96DE0	U8 snoRNA-decapping enzyme	NUDT16	57519000	INFINITE
Q9NZ52	ADP-ribosylation factor-binding protein GGA3	GGA3	57473000	INFINITE
P01024	Complement C3;Complement C3 beta chain;C3-be	C3	57438000	INFINITE
Q86WR0	Coiled-coil domain-containing protein 25	CCDC25	56916000	INFINITE
P06400	Retinoblastoma-associated protein	RB1	56848000	INFINITE
Q9NWU1	3-oxoacyl-[acyl-carrier-protein] synthase, mitochoı	OXSM	56836000	INFINITE
Q9NWS0	PIH1 domain-containing protein 1	PIH1D1	56769000	INFINITE
P58546	Myotrophin	MTPN	56507000	INFINITE
P15104	Glutamine synthetase	GLUL	56467000	INFINITE
O95684	FGFR1 oncogene partner	FGFR1OP	56345000	INFINITE
O95149	Snurportin-1	SNUPN	56333000	INFINITE
P47712	Cytosolic phospholipase A2;Phospholipase A2;Lysc	PLA2G4A	56287000	INFINITE
O95807	Transmembrane protein 50A	TMEM50A	56251000	INFINITE
P42785	Lysosomal Pro-X carboxypeptidase	PRCP	56133000	INFINITE
Q96F85	CB1 cannabinoid receptor-interacting protein 1	CNRIP1	56015000	INFINITE
O43677	NADH dehydrogenase [ubiquinone] 1 subunit C1, r	NDUFC1	55906000	INFINITE
Q8TEY7	Ubiquitin carboxyl-terminal hydrolase 33	USP33	55870000	INFINITE
Q6NZY4	Zinc finger CCHC domain-containing protein 8	ZCCHC8	55729000	INFINITE
Q8WVC6	Dephospho-CoA kinase domain-containing protein	DCAKD	55641000	INFINITE
Q9NTI5	Sister chromatid cohesion protein PDS5 homolog E	PDS5B	55370000	INFINITE
Q12893	Transmembrane protein 115	TMEM115	54630000	INFINITE
O00592	Podocalyxin	PODXL	54522000	INFINITE
O15013	Rho guanine nucleotide exchange factor 10	ARHGEF10	54459000	INFINITE
Q8NFF5	FAD synthase;Molybdenum cofactor biosynthesis ı	FLAD1	54128000	INFINITE
Q9H1E5	Thioredoxin-related transmembrane protein 4	TMX4	54102000	INFINITE
Q86W56	Poly(ADP-ribose) glycohydrolase	PARG	53950000	INFINITE
Q9H6L5	Protein FAM134B	FAM134B	53901000	INFINITE
Q68CQ4	Digestive organ expansion factor homolog	DIEXF	53886000	INFINITE
P41236;Q6	Protein phosphatase inhibitor 2;Protein phosphata	PPP1R2;PPP1R2P3	53745000	INFINITE
Q8WXA9	Splicing regulatory glutamine/lysine-rich protein 1	SREK1	53700000	INFINITE
Q5VT66	Mitochondrial amidoxime-reducing component 1	Mar-01	53383000	INFINITE
Q9Y5A7	NEDD8 ultimate buster 1	NUB1	53374000	INFINITE
Q9Y6A4	Cilia- and flagella-associated protein 20	CFAP20	53114000	INFINITE
Q9BV23	Monoacylglycerol lipase ABHD6	ABHD6	53070000	INFINITE
P34810	Macrosialin	CD68	52995000	INFINITE
Q9C037	E3 ubiquitin-protein ligase TRIM4	TRIM4	52847000	INFINITE
Q9NVZ3	Adaptin ear-binding coat-associated protein 2	NECAP2	52827000	INFINITE
Q86VX2	COMM domain-containing protein 7	COMMD7	52766000	INFINITE
Q8IV38	Ankyrin repeat and MYND domain-containing prot	ANKMY2	52674000	INFINITE
O14972	Down syndrome critical region protein 3	DSCR3	52596000	INFINITE
P16083	Ribosyldihydronicotinamide dehydrogenase [quinc	NQO2	52532000	INFINITE

O75935	Dynactin subunit 3	DCTN3	52512000	INFINITE
Q9Y343	Sorting nexin-24	SNX24	52480000	INFINITE
Q06546	GA-binding protein alpha chain	GABPA	52462000	INFINITE
O14646	Chromodomain-helicase-DNA-binding protein 1	CHD1	52325000	INFINITE
Q9UPN6	Protein SCAF8	SCAF8	52309000	INFINITE
P16333	Cytoplasmic protein NCK1	NCK1	52300000	INFINITE
Q8IV04	Carabin	TBC1D10C	52174000	INFINITE
Q9NWM8	Peptidyl-prolyl cis-trans isomerase FKBP14	FKBP14	52046000	INFINITE
P53667	LIM domain kinase 1	LIMK1	52027000	INFINITE
O43150	Arf-GAP with SH3 domain, ANK repeat and PH don	ASAP2	52025000	INFINITE
Q9BR61	Acyl-CoA-binding domain-containing protein 6	ACBD6	51782000	INFINITE
Q9UNQ2	Probable dimethyladenosine transferase	DIMT1	51764000	INFINITE
Q6DKJ4	Nucleoredoxin	NXN	51620000	INFINITE
P78310	Coxsackievirus and adenovirus receptor	CXADR	51597000	INFINITE
P48506	Glutamate--cysteine ligase catalytic subunit	GCLC	51580000	INFINITE
Q9H7L9	Sin3 histone deacetylase corepressor complex con	SUDS3	51478000	INFINITE
Q12894	Interferon-related developmental regulator 2	IFRD2	51370000	INFINITE
Q9P2B2	Prostaglandin F2 receptor negative regulator	PTGFRN	51292000	INFINITE
P30038	Delta-1-pyrroline-5-carboxylate dehydrogenase, m	ALDH4A1	51166000	INFINITE
P09038	Fibroblast growth factor 2	FGF2	50649000	INFINITE
Q99956	Dual specificity protein phosphatase 9	DUSP9	50575000	INFINITE
O95674	Phosphatidate cytidylyltransferase 2	CDS2	50566000	INFINITE
Q16625	Occludin	OCLN	50380000	INFINITE
Q9NXX6	Non-structural maintenance of chromosomes elen	NSMCE4A	50267000	INFINITE
P62253	Ubiquitin-conjugating enzyme E2 G1;Ubiquitin-con	UBE2G1	50043000	INFINITE
Q9NVM4	Protein arginine N-methyltransferase 7	PRMT7	50018000	INFINITE
Q8WZA0	Protein LZIC	LZIC	50001000	INFINITE
P55058	Phospholipid transfer protein	PLTP	49940000	INFINITE
O43924	Retinal rod rhodopsin-sensitive cGMP 3,5-cyclic ph	PDE6D	49927000	INFINITE
O95229	ZW10 interactor	ZWINT	49718000	INFINITE
Q5VWJ9	Sorting nexin-30	SNX30	49344000	INFINITE
O43772	Mitochondrial carnitine/acylcarnitine carrier prote	SLC25A20	49297000	INFINITE
Q5UCC4	ER membrane protein complex subunit 10	EMC10	49168000	INFINITE
Q13625	Apoptosis-stimulating of p53 protein 2	TP53BP2	49151000	INFINITE
Q9BRF8	Serine/threonine-protein phosphatase CPPED1	CPPED1	49058000	INFINITE
Q9BQP7	Mitochondrial genome maintenance exonuclease :	MGME1	49020000	INFINITE
Q9Y4A5	Transformation/transcription domain-associated p	TRRAP	48941000	INFINITE
P61626	Lysozyme C	LYZ	48843000	INFINITE
Q9ULQ0	Striatin-interacting protein 2	STRIP2	48829000	INFINITE
Q92858	Protein atonal homolog 1	ATOH1	48821000	INFINITE
Q9H3G5	Probable serine carboxypeptidase CPVL	CPVL	48695000	INFINITE
P62487	DNA-directed RNA polymerase II subunit RPB7	POLR2G	48618000	INFINITE
Q9BY67	Cell adhesion molecule 1	CADM1	48549000	INFINITE
Q9BZL1	Ubiquitin-like protein 5	UBL5	48503000	INFINITE
P03905	NADH-ubiquinone oxidoreductase chain 4	MT-ND4	48421000	INFINITE
P29084	Transcription initiation factor IIE subunit beta	GTF2E2	48173000	INFINITE
Q9HD34	LYR motif-containing protein 4	LYRM4	48074000	INFINITE
Q8N129	Protein canopy homolog 4	CNPY4	47837000	INFINITE
Q7L775	EPM2A-interacting protein 1	EPM2AIP1	47694000	INFINITE
Q8IUI8	Cytokine receptor-like factor 3	CRLF3	47650000	INFINITE

P15848	Arylsulfatase B	ARSB	47494000	INFINITE
Q8WUA4	General transcription factor 3C polypeptide 2	GTF3C2	47369000	INFINITE
O15254	Peroxisomal acyl-coenzyme A oxidase 3	ACOX3	47351000	INFINITE
Q96HN2	Putative adenosylhomocysteinase 3	AHCYL2	47281000	INFINITE
Q969X1	Protein lifeguard 3	TMBIM1	47230000	INFINITE
Q9UK76	Hematological and neurological expressed 1 protei	HN1	47214000	INFINITE
P48060	Glioma pathogenesis-related protein 1	GLIPR1	46926000	INFINITE
Q7L099	Protein RUFY3	RUFY3	46779000	INFINITE
P69849;Q5	Nodal modulator 3;Nodal modulator 2	NOMO3;NOMO2	46756000	INFINITE
Q9H0R4	Haloacid dehalogenase-like hydrolase domain-con	HDHD2	46649000	INFINITE
Q66K14	TBC1 domain family member 9B	TBC1D9B	46638000	INFINITE
O95905	Protein SGT1	ECD	46634000	INFINITE
Q86UV5	Ubiquitin carboxyl-terminal hydrolase 48	USP48	46627000	INFINITE
Q16775	Hydroxyacylglutathione hydrolase, mitochondrial	HAGH	46565000	INFINITE
Q8WVK7	Spindle and kinetochore-associated protein 2	SKA2	46394000	INFINITE
Q96IY1	Kinetochore-associated protein NSL1 homolog	NSL1	46313000	INFINITE
Q8N3R9	MAGUK p55 subfamily member 5	MPP5	46139000	INFINITE
Q8IXQ4	GPALPP motifs-containing protein 1	GPALPP1	46135000	INFINITE
Q5TEJ8	Protein THEMIS2	THEMIS2	46093000	INFINITE
O60216;Q9	Double-strand-break repair protein rad21 homolog	RAD21;RAD21L1	46068000	INFINITE
P45984;P5	Mitogen-activated protein kinase 9;Mitogen-activa	MAPK9;MAPK10	45985000	INFINITE
Q9Y5L4	Mitochondrial import inner membrane translocase	TIMM13	45972000	INFINITE
Q6GQQ9	OTU domain-containing protein 7B	OTUD7B	45862000	INFINITE
Q92574	Hamartin	TSC1	45771000	INFINITE
Q96KX1	Uncharacterized protein C4orf36	C4orf36	45591000	INFINITE
Q5SW96	Low density lipoprotein receptor adapter protein 1	LDLRAP1	45307000	INFINITE
Q92889	DNA repair endonuclease XPF	ERCC4	45247000	INFINITE
Q9BRT9	DNA replication complex GINS protein SLD5;DNA r	GINS4	45223000	INFINITE
Q86W42	THO complex subunit 6 homolog	THOC6	45213000	INFINITE
Q7Z3T8	Zinc finger FYVE domain-containing protein 16	ZFYVE16	45169000	INFINITE
P62070	Ras-related protein R-Ras2	RRAS2	45122000	INFINITE
Q14790	Caspase-8;Caspase-8 subunit p18;Caspase-8 subur	CASP8	45114000	INFINITE
Q9H3P2	Negative elongation factor A	NELFA	45088000	INFINITE
Q6IQ22	Ras-related protein Rab-12	RAB12	44629000	INFINITE
Q9UJ14	Gamma-glutamyltransferase 7;Gamma-glutamyltra	GGT7	44513000	INFINITE
Q14657	EKC/KEOPS complex subunit LAGE3	LAGE3	44325000	INFINITE
Q9NP61	ADP-ribosylation factor GTPase-activating protein	ARFGAP3	44324000	INFINITE
Q96BX8	MOB kinase activator 3A	MOB3A	44294000	INFINITE
Q6NSJ5	Volume-regulated anion channel subunit LRRC8E	LRRC8E	44286000	INFINITE
O60927	Protein phosphatase 1 regulatory subunit 11	PPP1R11	44264000	INFINITE
Q9BQE5	Apolipoprotein L2	APOL2	44205000	INFINITE
P52756	RNA-binding protein 5	RBM5	44161000	INFINITE
P86791;P8	Vacuolar fusion protein CCZ1 homolog;Vacuolar fu	CCZ1;CCZ1B	44063000	INFINITE
Q03426	Mevalonate kinase	MVK	44046000	INFINITE
Q9NYT0	Pleckstrin-2	PLEK2	43978000	INFINITE
Q9BRQ6	MICOS complex subunit MIC25	CHCHD6	43969000	INFINITE
P13051	Uracil-DNA glycosylase	UNG	43913000	INFINITE
Q96EA4	Protein Spindly	SPDL1	43887000	INFINITE
P03915	NADH-ubiquinone oxidoreductase chain 5	MT-ND5	43796000	INFINITE
Q9Y624	Junctional adhesion molecule A	F11R	43574000	INFINITE

P62380	TATA box-binding protein-like protein 1	TBPL1	43568000	INFINITE
Q6ZVM7	TOM1-like protein 2	TOM1L2	43451000	INFINITE
Q96LD4	Tripartite motif-containing protein 47	TRIM47	43262000	INFINITE
Q92733	Proline-rich protein PRCC	PRCC	43243000	INFINITE
O60427	Fatty acid desaturase 1	FADS1	42920000	INFINITE
P23378	Glycine dehydrogenase (decarboxylating), mitocho	GLDC	42658000	INFINITE
Q13309	S-phase kinase-associated protein 2	SKP2	42407000	INFINITE
O75832	26S proteasome non-ATPase regulatory subunit 10	PSMD10	42355000	INFINITE
P11117	Lysosomal acid phosphatase	ACP2	42259000	INFINITE
Q8IYS2	Uncharacterized protein KIAA2013	KIAA2013	42247000	INFINITE
Q9NUL7	Probable ATP-dependent RNA helicase DDX28	DDX28	42235000	INFINITE
Q6Y1H2	Very-long-chain (3R)-3-hydroxyacyl-CoA dehydrata	HACD2	42182000	INFINITE
P53365	Arfaptin-2	ARFIP2	42159000	INFINITE
Q9UDR5	Alpha-aminoadipic semialdehyde synthase, mitoch	AASS	42129000	INFINITE
P28065	Proteasome subunit beta type-9	PSMB9	42074000	INFINITE
Q9UJ68	Mitochondrial peptide methionine sulfoxide reduc	MSRA	42039000	INFINITE
P32970	CD70 antigen	CD70	41845000	INFINITE
O60879	Protein diaphanous homolog 2	DIAPH2	41712000	INFINITE
Q9BUH6	Protein PAXX	C9orf142	41543000	INFINITE
Q96EH3	Mitochondrial assembly of ribosomal large subunit	MALSU1	41537000	INFINITE
Q8IWZ8	SURP and G-patch domain-containing protein 1	SUGP1	41455000	INFINITE
P54619	5-AMP-activated protein kinase subunit gamma-1	PRKAG1	41406000	INFINITE
Q9BV81	ER membrane protein complex subunit 6	EMC6	41302000	INFINITE
Q9NWT6	Hypoxia-inducible factor 1-alpha inhibitor	HIF1AN	41205000	INFINITE
O95749	Geranylgeranyl pyrophosphate synthase	GGPS1	41174000	INFINITE
O15031	Plexin-B2	PLXNB2	41041000	INFINITE
Q9P0S3;Q5	ORM1-like protein 1;ORM1-like protein 2	ORMDL1;ORMDL2	40834000	INFINITE
Q2VPK5	Cytoplasmic tRNA 2-thiolation protein 2	CTU2	40787000	INFINITE
P20338	Ras-related protein Rab-4A	RAB4A	40706000	INFINITE
Q8WXF7	Atlastin-1	ATL1	40551000	INFINITE
Q03518	Antigen peptide transporter 1	TAP1	40446000	INFINITE
Q9H4L7	SWI/SNF-related matrix-associated actin-depende	SMARCAD1	40438000	INFINITE
Q8NFC6	Biorientation of chromosomes in cell division prote	BOD1L1	40162000	INFINITE
Q96FV2	Secernin-2	SCRN2	40115000	INFINITE
Q8WZA9	Immunity-related GTPase family Q protein	IRGQ	39997000	INFINITE
O43516	WAS/WASL-interacting protein family member 1	WIPF1	39942000	INFINITE
Q96FV9	THO complex subunit 1	THOC1	39859000	INFINITE
A6NDU8	UPF0600 protein C5orf51	C5orf51	39834000	INFINITE
Q96BR5	Cytochrome c oxidase assembly factor 7	COA7	39820000	INFINITE
Q9UIW2;Q9	Plexin-A1;Plexin-A4	PLXNA1;PLXNA4	39693000	INFINITE
Q9H9Q4	Non-homologous end-joining factor 1	NHEJ1	39687000	INFINITE
Q8NE86	Calcium uniporter protein, mitochondrial	MCU	39685000	INFINITE
P07305	Histone H1.0;Histone H1.0, N-terminally processec	H1F0	39505000	INFINITE
Q9H410	Kinetochore-associated protein DSN1 homolog	DSN1	39458000	INFINITE
Q8ND04	Protein SMG8	SMG8	39405000	INFINITE
Q9H2D1	Mitochondrial folate transporter/carrier	SLC25A32	39400000	INFINITE
Q8NDI1	EH domain-binding protein 1	EHBP1	39274000	INFINITE
P05165	Propionyl-CoA carboxylase alpha chain, mitochond	PCCA	39263000	INFINITE
O60825;Q1	6-phosphofructo-2-kinase/fructose-2,6-bisphospha	PFKFB2;PFKFB3	39228000	INFINITE
Q8WVX9	Fatty acyl-CoA reductase 1	FAR1	39210000	INFINITE

O95376	E3 ubiquitin-protein ligase ARIH2	ARIH2	39205000	INFINITE
Q9P291	Armadillo repeat-containing X-linked protein 1	ARMCX1	39077000	INFINITE
Q12802	A-kinase anchor protein 13	AKAP13	38896000	INFINITE
P78381	UDP-galactose translocator	SLC35A2	38800000	INFINITE
P53384	Cytosolic Fe-S cluster assembly factor NUBP1	NUBP1	38713000	INFINITE
Q96IZ0	PRKC apoptosis WT1 regulator protein	PAWR	38698000	INFINITE
Q13685	Angio-associated migratory cell protein	AAMP	38466000	INFINITE
Q6UXH1	Cysteine-rich with EGF-like domain protein 2	CRELD2	38354000	INFINITE
Q96CM8	Acyl-CoA synthetase family member 2, mitochondı	ACSF2	38272000	INFINITE
Q6P1X5	Transcription initiation factor TFIID subunit 2	TAF2	38163000	INFINITE
Q8NC96	Adaptin ear-binding coat-associated protein 1	NECAP1	38160000	INFINITE
Q5TA45	Integrator complex subunit 11	CPSF3L	37931000	INFINITE
O14966	Ras-related protein Rab-7L1	RAB29	37867000	INFINITE
P43378	Tyrosine-protein phosphatase non-receptor type 9	PTPN9	37561000	INFINITE
Q9NPI6	mRNA-decapping enzyme 1A	DCP1A	37512000	INFINITE
Q8NEB9	Phosphatidylinositol 3-kinase catalytic subunit type	PIK3C3	37408000	INFINITE
Q9H0W9	Ester hydrolase C11orf54	C11orf54	37386000	INFINITE
Q9P0J7	E3 ubiquitin-protein ligase KCMF1	KCMF1	37312000	INFINITE
O94888	UBX domain-containing protein 7	UBXN7	37312000	INFINITE
Q96LR5;Q9	Ubiquitin-conjugating enzyme E2 E2;Ubiquitin-con	UBE2E2;UBE2E3	37206000	INFINITE
P30049	ATP synthase subunit delta, mitochondrial	ATP5D	37186000	INFINITE
Q86YV9	Hermansky-Pudlak syndrome 6 protein	HPS6	37139000	INFINITE
Q01484	Ankyrin-2	ANK2	37130000	INFINITE
Q9UI14	Prenylated Rab acceptor protein 1	RABAC1	36894000	INFINITE
Q5VT25	Serine/threonine-protein kinase MRCK alpha	CDC42BPA	36846000	INFINITE
Q9Y296	Trafficking protein particle complex subunit 4	TRAPPC4	36834000	INFINITE
Q02153	Guanylate cyclase soluble subunit beta-1	GUCY1B3	36776000	INFINITE
O15164	Transcription intermediary factor 1-alpha	TRIM24	36655000	INFINITE
Q9GZU8	Protein FAM192A	FAM192A	36473000	INFINITE
P42574	Caspase-3;Caspase-3 subunit p17;Caspase-3 subur	CASP3	36372000	INFINITE
O15321	Transmembrane 9 superfamily member 1	TM9SF1	36347000	INFINITE
Q9HA65	TBC1 domain family member 17	TBC1D17	36257000	INFINITE
Q9H4G4	Golgi-associated plant pathogenesis-related protei	GLIPR2	36251000	INFINITE
Q96P63	Serpin B12	SERPINB12	36220000	INFINITE
O95825	Quinone oxidoreductase-like protein 1	CRYZL1	36105000	INFINITE
Q6UVJ0	Spindle assembly abnormal protein 6 homolog	SASS6	36102000	INFINITE
Q9BT22	Chitobiosyldiphosphodolichol beta-mannosyltrans	ALG1	36054000	INFINITE
Q96GG9	DCN1-like protein 1	DCUN1D1	36040000	INFINITE
Q96BP2	Coiled-coil-helix-coiled-coil-helix domain-containin	CHCHD1	36036000	INFINITE
Q9H6X2;P5	Anthrax toxin receptor 1;Anthrax toxin receptor 2	ANTXR1;ANTXR2	35986000	INFINITE
P52943	Cysteine-rich protein 2	CRIP2	35936000	INFINITE
P29372	DNA-3-methyladenine glycosylase	MPG	35780000	INFINITE
Q13153	Serine/threonine-protein kinase PAK 1	PAK1	35732000	INFINITE
O14531	Dihydropyrimidinase-related protein 4	DPYSL4	35636000	INFINITE
Q96MX6	WD repeat-containing protein 92	WDR92	35618000	INFINITE
Q8WWH5	Probable tRNA pseudouridine synthase 1	TRUB1	35513000	INFINITE
Q9NVP2	Histone chaperone ASF1B	ASF1B	35410000	INFINITE
Q02539	Histone H1.1	HIST1H1A	35321000	INFINITE
P50452	Serpin B8	SERPINB8	35300000	INFINITE
Q9H6E4	Coiled-coil domain-containing protein 134	CCDC134	35209000	INFINITE

Q5ZPR3	CD276 antigen	CD276	35190000	INFINITE
Q6NUQ1	RAD50-interacting protein 1	RINT1	35156000	INFINITE
P22681	E3 ubiquitin-protein ligase CBL	CBL	35086000	INFINITE
Q9NVS2	28S ribosomal protein S18a, mitochondrial	MRPS18A	35077000	INFINITE
P07992	DNA excision repair protein ERCC-1	ERCC1	34942000	INFINITE
P10398	Serine/threonine-protein kinase A-Raf	ARAF	34917000	INFINITE
Q9NWS8	Required for meiotic nuclear division protein 1 hor	RMND1	34908000	INFINITE
Q15118	[Pyruvate dehydrogenase (acetyl-transferring)] kin	PDK1	34824000	INFINITE
Q969S0	UDP-xylose and UDP-N-acetylglucosamine transpo	SLC35B4	34793000	INFINITE
Q09472	Histone acetyltransferase p300	EP300	34741000	INFINITE
Q8NEC7	Glutathione S-transferase C-terminal domain-cont	GSTCD	34723000	INFINITE
Q15418	Ribosomal protein S6 kinase alpha-1	RPS6KA1	34720000	INFINITE
Q96SK2	Transmembrane protein 209	TMEM209	34712000	INFINITE
P34059	N-acetylgalactosamine-6-sulfatase	GALNS	34704000	INFINITE
P17568	NADH dehydrogenase [ubiquinone] 1 beta subcom	NDUFB7	34698000	INFINITE
A2NJV5;A0	Ig kappa chain V-II region RPMI 6410;Ig kappa chai	IGKV A18;IGKV2D-	34477000	INFINITE
Q969E8	Pre-rRNA-processing protein TSR2 homolog	TSR2	34446000	INFINITE
Q6IBW4	Condensin-2 complex subunit H2	NCAPH2	34425000	INFINITE
Q9HAD4	WD repeat-containing protein 41	WDR41	34310000	INFINITE
P23443	Ribosomal protein S6 kinase beta-1	RPS6KB1	34270000	INFINITE
Q9Y639	Neuroplastin	NPTN	34256000	INFINITE
O00422	Histone deacetylase complex subunit SAP18	SAP18	34252000	INFINITE
P49917	DNA ligase 4	LIG4	34200000	INFINITE
Q9BXB7	Spermatogenesis-associated protein 16	SPATA16	34180000	INFINITE
O14734	Acyl-coenzyme A thioesterase 8	ACOT8	34099000	INFINITE
Q9Y618	Nuclear receptor corepressor 2	NCOR2	34093000	INFINITE
Q15048	Leucine-rich repeat-containing protein 14	LRRC14	34056000	INFINITE
Q7Z4S6	Kinesin-like protein KIF21A	KIF21A	33975000	INFINITE
P39210	Protein Mpv17	MPV17	33863000	INFINITE
Q96K21	Abscission/NoCut checkpoint regulator	ZFYVE19	33821000	INFINITE
P18846;P16	Cyclic AMP-dependent transcription factor ATF-1;C	ATF1;CREB1	33776000	INFINITE
Q03154	Aminoacylase-1	ACY1	33722000	INFINITE
Q4ZIN3	Membralin	TMEM259	33569000	INFINITE
Q9UP95	Solute carrier family 12 member 4	SLC12A4	33545000	INFINITE
P56559	ADP-ribosylation factor-like protein 4C	ARL4C	33503000	INFINITE
Q9UL62;Q9	Short transient receptor potential channel 5;Short	TRPC5;TRPC4	33427000	INFINITE
P53794	Sodium/myo-inositol cotransporter	SLC5A3	33418000	INFINITE
Q9Y6J9	TAF6-like RNA polymerase II p300/CBP-associated	TAF6L	33330000	INFINITE
Q969S9	Ribosome-releasing factor 2, mitochondrial	GFM2	33258000	INFINITE
Q9H583	HEAT repeat-containing protein 1;HEAT repeat-cor	HEATR1	33210000	INFINITE
P63096	Guanine nucleotide-binding protein G(i) subunit al	GNAI1	33107000	INFINITE
P61024	Cyclin-dependent kinases regulatory subunit 1	CKS1B	32887000	INFINITE
Q9UPY8	Microtubule-associated protein RP/EB family mem	MAPRE3	32840000	INFINITE
Q9H497	Torsin-3A	TOR3A	32782000	INFINITE
Q8IZ21	Phosphatase and actin regulator 4	PHACTR4	32759000	INFINITE
Q8TCY9	Up-regulator of cell proliferation	URGCP	32753000	INFINITE
Q8IY95	Transmembrane protein 192	TMEM192	32737000	INFINITE
Q9NTG7	NAD-dependent protein deacetylase sirtuin-3, mit	SIRT3	32698000	INFINITE
Q9UHR6	Zinc finger HIT domain-containing protein 2	ZNHIT2	32623000	INFINITE
Q8NAV1	Pre-mRNA-splicing factor 38A	PRPF38A	32590000	INFINITE

O94885	SAM and SH3 domain-containing protein 1	SASH1	32582000	INFINITE
Q8WVI0	Small integral membrane protein 4	SMIM4	32575000	INFINITE
Q9NU23	LYR motif-containing protein 2	LYRM2	32452000	INFINITE
P29400	Collagen alpha-5(IV) chain	COL4A5	32451000	INFINITE
Q8N3X1	Formin-binding protein 4	FNBP4	32428000	INFINITE
Q9BSB4	Autophagy-related protein 101	ATG101	32350000	INFINITE
Q9NQP4	Prefoldin subunit 4	PFDN4	32317000	INFINITE
Q9UMS0	NFU1 iron-sulfur cluster scaffold homolog, mitoch	NFU1	32240000	INFINITE
P10636	Microtubule-associated protein tau	MAPT	32172000	INFINITE
Q8NCE2	Myotubularin-related protein 14	MTMR14	32156000	INFINITE
Q9NUB1	Acetyl-coenzyme A synthetase 2-like, mitochondri	ACSS1	32110000	INFINITE
Q08AD1	Calmodulin-regulated spectrin-associated protein	CAMSAP2	31852000	INFINITE
P30047	GTP cyclohydrolase 1 feedback regulatory protein	GCHFR	31828000	INFINITE
Q8NEM2	SHC SH2 domain-binding protein 1	SHCBP1	31812000	INFINITE
Q9BVL2	Nucleoporin p58/p45	NUPL1	31647000	INFINITE
Q9NVA4	Transmembrane protein 184C	TMEM184C	31626000	INFINITE
Q8TD30	Alanine aminotransferase 2	GPT2	31578000	INFINITE
Q8N201	Integrator complex subunit 1	INTS1	31577000	INFINITE
O43765	Small glutamine-rich tetratricopeptide repeat-cont	SGTA	31555000	INFINITE
Q9NNW7	Thioredoxin reductase 2, mitochondrial	TXNRD2	31535000	INFINITE
P05109	Protein S100-A8;Protein S100-A8, N-terminally pro	S100A8	31400000	INFINITE
Q9H2P0	Activity-dependent neuroprotector homeobox pro	ADNP	31395000	INFINITE
Q96DZ1	Endoplasmic reticulum lectin 1	ERLEC1	31391000	INFINITE
Q9H944	Mediator of RNA polymerase II transcription subur	MED20	31364000	INFINITE
P49914	5-formyltetrahydrofolate cyclo-ligase	MTHFS	31355000	INFINITE
Q8WX93	Palladin	PALLD	31286000	INFINITE
Q86X10	Ral GTPase-activating protein subunit beta	RALGAPB	31286000	INFINITE
Q92604	Acyl-CoA:lysophosphatidylglycerol acyltransferase	LPGAT1	31273000	INFINITE
O75191	Xylulose kinase	XYLB	31271000	INFINITE
Q93050	V-type proton ATPase 116 kDa subunit a isoform 1	ATP6V0A1	31236000	INFINITE
Q9NXJ5	Pyroglutamyl-peptidase 1	PGPEP1	31136000	INFINITE
Q6ZS17	Protein FAM65A	FAM65A	31118000	INFINITE
Q92536	Y+L amino acid transporter 2	SLC7A6	31068000	INFINITE
Q8WVD3	E3 ubiquitin-protein ligase RNF138	RNF138	30892000	INFINITE
Q5T9L3	Protein wntless homolog	WLS	30745000	INFINITE
P42357	Histidine ammonia-lyase	HAL	30742000	INFINITE
Q9Y6K0	Choline/ethanolaminephosphotransferase 1	CEPT1	30635000	INFINITE
P62837	Ubiquitin-conjugating enzyme E2 D2	UBE2D2	30633000	INFINITE
Q96EL3	39S ribosomal protein L53, mitochondrial	MRPL53	30593000	INFINITE
Q9UQR0	Sex comb on midleg-like protein 2	SCML2	30559000	INFINITE
Q9NXA8	NAD-dependent protein deacylase sirtuin-5, mitoc	SIRT5	30455000	INFINITE
O15439	Multidrug resistance-associated protein 4	ABCC4	30433000	INFINITE
Q9Y303	Putative N-acetylglucosamine-6-phosphate deacet	AMDHD2	30298000	INFINITE
Q13137	Calcium-binding and coiled-coil domain-containing	CALCOCO2	30283000	INFINITE
Q96PC5;Q9	Melanoma inhibitory activity protein 2;cTAGE fami	MIA2;CTAGE1	30282000	INFINITE
Q96CN9	GRIP and coiled-coil domain-containing protein 1	GCC1	30192000	INFINITE
P31327	Carbamoyl-phosphate synthase [ammonia], mitocl	CPS1	30050000	INFINITE
Q9UHB7	AF4/FMR2 family member 4	AFF4	29889000	INFINITE
P52895	Aldo-keto reductase family 1 member C2	AKR1C2	29867000	INFINITE
Q16854	Deoxyguanosine kinase, mitochondrial	DGUOK	29827000	INFINITE

Q12873	Chromodomain-helicase-DNA-binding protein 3	CHD3	29809000	INFINITE
Q8TAP6	Centrosomal protein of 76 kDa	CEP76	29737000	INFINITE
Q5R3I4	Tetratricopeptide repeat protein 38	TTC38	29638000	INFINITE
O43657	Tetraspanin-6	TSPAN6	29590000	INFINITE
Q8WUK0	Phosphatidylglycerophosphatase and protein-tyro:	PTPMT1	29538000	INFINITE
Q6ZXV5	Transmembrane and TPR repeat-containing protei	TMTC3	29446000	INFINITE
Q86X83	COMM domain-containing protein 2	COMMD2	29369000	INFINITE
O95786	Probable ATP-dependent RNA helicase DDX58	DDX58	29365000	INFINITE
P41223	Protein BUD31 homolog	BUD31	29354000	INFINITE
Q6ZMZ3	Nesprin-3	SYNE3	29347000	INFINITE
Q6ZSZ5	Rho guanine nucleotide exchange factor 18	ARHGEF18	29240000	INFINITE
Q9H4I3	TraB domain-containing protein	TRABD	29063000	INFINITE
Q15750	TGF-beta-activated kinase 1 and MAP3K7-binding		TAB1	29053000	INFINITE
Q32P44	Echinoderm microtubule-associated protein-like 3	EML3	29011000	INFINITE
Q7Z6K3	Protein prenyltransferase alpha subunit repeat-co	PTAR1	28885000	INFINITE
O60563	Cyclin-T1	CCNT1	28868000	INFINITE
O60282	Kinesin heavy chain isoform 5C	KIF5C	28848000	INFINITE
Q9NXC5	WD repeat-containing protein mio	MIOS	28767000	INFINITE
Q9H0H0	Integrator complex subunit 2	INTS2	28707000	INFINITE
Q6PL24	Protein TMED8	TMED8	28510000	INFINITE
Q5BJH7	Protein YIF1B	YIF1B	28477000	INFINITE
Q9H4Z3	Phosphorylated CTD-interacting factor 1	PCIF1	28401000	INFINITE
Q13107	Ubiquitin carboxyl-terminal hydrolase 4	USP4	28392000	INFINITE
P48740	Mannan-binding lectin serine protease 1;Mannan-	MASP1	28336000	INFINITE
P08253	72 kDa type IV collagenase;PEX	MMP2	28316000	INFINITE
Q9UDX5	Mitochondrial fission process protein 1	MTFP1	28250000	INFINITE
Q14249	Endonuclease G, mitochondrial	ENDOG	28170000	INFINITE
Q9NPF5	DNA methyltransferase 1-associated protein 1	DMAP1	28150000	INFINITE
O43808	Peroxisomal membrane protein PMP34	SLC25A17	28149000	INFINITE
Q86XI2	Condensin-2 complex subunit G2	NCAPG2	28000000	INFINITE
P00156	Cytochrome b	MT-CYB	27813000	INFINITE
A0A024RB(	Diphosphoinositol polyphosphate phosphohydrola	NUDT4;NUDT11;N	27810000	INFINITE
Q13795	ADP-ribosylation factor-related protein 1	ARFRP1	27722000	INFINITE
Q9Y680	Peptidyl-prolyl cis-trans isomerase FKBP7	FKBP7	27608000	INFINITE
Q9Y3S2	Zinc finger protein 330	ZNF330	27513000	INFINITE
Q96C24	Synaptotagmin-like protein 4	SYTL4	27408000	INFINITE
Q16623	Syntaxin-1A	STX1A	27394000	INFINITE
P10109	Adrenodoxin, mitochondrial	FDX1	27391000	INFINITE
P63027	Vesicle-associated membrane protein 2	VAMP2	27282000	INFINITE
Q9BRS8	La-related protein 6	LARP6	27247000	INFINITE
Q9UKU7	Isobutyryl-CoA dehydrogenase, mitochondrial	ACAD8	27050000	INFINITE
O60462	Neuropilin-2	NRP2	27033000	INFINITE
Q13287	N-myc-interactor	NMI	26981000	INFINITE
P49674;P4{	Casein kinase I isoform epsilon;Casein kinase I isof	CSNK1E;CSNK1D	26905000	INFINITE
O43597	Protein sprouty homolog 2	SPRY2	26893000	INFINITE
P17050	Alpha-N-acetylgalactosaminidase	NAGA	26885000	INFINITE
O60504	Vinexin	SORBS3	26875000	INFINITE
Q8NBN7	Retinol dehydrogenase 13	RDH13	26752000	INFINITE
Q5JUR7	Testis-expressed sequence 30 protein	TEX30	26725000	INFINITE
Q8NCL4	Polypeptide N-acetylgalactosaminyltransferase 6	GALNT6	26675000	INFINITE

Q86TN4	tRNA 2-phosphotransferase 1	TRPT1	26645000	INFINITE
Q16527	Cysteine and glycine-rich protein 2	CSRP2	26637000	INFINITE
Q53S08;Q9	Ras-related protein Rab-6C	RAB6C	26535000	INFINITE
Q06190	Serine/threonine-protein phosphatase 2A regulatc	PPP2R3A	26522000	INFINITE
Q96GW9	Methionine--tRNA ligase, mitochondrial	MARS2	26457000	INFINITE
Q8N490	Probable hydrolase PNKD	PNKD	26447000	INFINITE
Q9HB66		MKKS	26298000	INFINITE
P61266	Syntaxin-1B	STX1B	26245000	INFINITE
Q86YR5	G-protein-signaling modulator 1	GPSM1	26162000	INFINITE
Q9C0E2	Exportin-4	XPO4	26136000	INFINITE
Q9BQ69	O-acetyl-ADP-ribose deacetylase MACROD1	MACROD1	26108000	INFINITE
Q5TDH0	Protein DDI1 homolog 2	DDI2	26001000	INFINITE
Q5U5X0	Complex III assembly factor LYRM7	LYRM7	25981000	INFINITE
P42695	Condensin-2 complex subunit D3	NCAPD3	25944000	INFINITE
Q9UL03	Integrator complex subunit 6	INTS6	25934000	INFINITE
O14672	Disintegrin and metalloproteinase domain-contain	ADAM10	25865000	INFINITE
P55789	FAD-linked sulfhydryl oxidase ALR	GFER	25842000	INFINITE
O95758	Polypyrimidine tract-binding protein 3	PTBP3	25759000	INFINITE
Q5T0D9	Tumor protein p63-regulated gene 1-like protein	TPRG1L	25756000	INFINITE
O00754	Lysosomal alpha-mannosidase;Lysosomal alpha-m	MAN2B1	25628000	INFINITE
P08922	Proto-oncogene tyrosine-protein kinase ROS	ROS1	25567000	INFINITE
P31323	cAMP-dependent protein kinase type II-beta regul	PRKAR2B	25567000	INFINITE
Q9NSY1	BMP-2-inducible protein kinase	BMP2K	25489000	INFINITE
Q9NWB6	Arginine and glutamate-rich protein 1	ARGLU1	25454000	INFINITE
O95834	Echinoderm microtubule-associated protein-like 2	EML2	25433000	INFINITE
Q9H7B4	Histone-lysine N-methyltransferase SMYD3	SMYD3	25323000	INFINITE
Q9BUE6	Iron-sulfur cluster assembly 1 homolog, mitochonc	ISCA1	25320000	INFINITE
Q99504	Eyes absent homolog 3	EYA3	25258000	INFINITE
Q96QU8	Exportin-6	XPO6	25244000	INFINITE
P49711;Q8	Transcriptional repressor CTCF;Transcriptional rep	CTCF;CTCFL	25108000	INFINITE
P53611	Geranylgeranyl transferase type-2 subunit beta	RABGGTB	25101000	INFINITE
Q96AA3	Protein RFT1 homolog	RFT1	25089000	INFINITE
P43007	Neutral amino acid transporter A	SLC1A4	25076000	INFINITE
O95295	SNARE-associated protein Snapin	SNAPIN	25024000	INFINITE
Q96F63	Coiled-coil domain-containing protein 97	CCDC97	25004000	INFINITE
Q8IX18	Probable ATP-dependent RNA helicase DHX40	DHX40	24940000	INFINITE
Q9Y605	MORF4 family-associated protein 1	MRFAP1	24831000	INFINITE
Q96LB3	Intraflagellar transport protein 74 homolog	IFT74	24819000	INFINITE
Q96T60	Bifunctional polynucleotide phosphatase/kinase;P	PNKP	24690000	INFINITE
Q58FG1	Putative heat shock protein HSP 90-alpha A4	HSP90AA4P	24588000	INFINITE
Q8IXI1	Mitochondrial Rho GTPase 2	RHOT2	24556000	INFINITE
Q13286	Battenin	CLN3	24456000	INFINITE
Q9NUD5	Zinc finger CCHC domain-containing protein 3	ZCCHC3	24411000	INFINITE
O43426	Synaptojanin-1	SYNJ1	24352000	INFINITE
Q99808	Equilibrative nucleoside transporter 1	SLC29A1	24349000	INFINITE
O14757	Serine/threonine-protein kinase Chk1	CHEK1	24254000	INFINITE
P01116	GTPase KRas;GTPase KRas, N-terminally processed	KRAS	24201000	INFINITE
Q9ULU8;Q8	Calcium-dependent secretion activator 1;Calcium-(	CADPS;CADPS2	24137000	INFINITE
Q13901	Nuclear nucleic acid-binding protein C1D	C1D	24134000	INFINITE
Q05707	Collagen alpha-1(XIV) chain	COL14A1	24036000	INFINITE

Q7L8L6	FAST kinase domain-containing protein 5	FASTKD5	24032000	INFINITE
Q8IXI2	Mitochondrial Rho GTPase 1	RHOT1	24029000	INFINITE
P55061	Bax inhibitor 1	TMBIM6	24009000	INFINITE
Q9BZA8;Q9	Protocadherin-11 Y-linked;Protocadherin-11 X-link	PCDH11Y;PCDH11	23962000	INFINITE
Q9H8Y5	Ankyrin repeat and zinc finger domain-containing	ANKZF1	23955000	INFINITE
Q9BZG8	Diphthamide biosynthesis protein 1	DPH1	23951000	INFINITE
Q8IVH8	Mitogen-activated protein kinase kinase kinase kin	MAP4K3	23744000	INFINITE
Q8IWJ2	GRIP and coiled-coil domain-containing protein 2	GCC2	23700000	INFINITE
P25098;P35	Beta-adrenergic receptor kinase 1;Beta-adrenergic	ADRBK1;ADRBK2	23476000	INFINITE
Q15517	Corneodesmosin	CDSN	23470000	INFINITE
Q96KQ7	Histone-lysine N-methyltransferase EHMT2	EHMT2	23374000	INFINITE
Q92805	Golgin subfamily A member 1	GOLGA1	23354000	INFINITE
Q96JH7	Deubiquitinating protein VCIP135	VCPIP1	23332000	INFINITE
P53602	Diphosphomevalonate decarboxylase	MVD	23201000	INFINITE
O60870	DNA/RNA-binding protein KIN17	KIN	23110000	INFINITE
Q6IQ49	Protein SDE2 homolog	SDE2	23094000	INFINITE
Q9NWZ5	Uridine-cytidine kinase-like 1	UCKL1	23048000	INFINITE
Q9Y4U1	Methylmalonic aciduria and homocystinuria type C	MMACHC	22987000	INFINITE
Q8NFG4	Folliculin	FLCN	22955000	INFINITE
Q9HC21	Mitochondrial thiamine pyrophosphate carrier	SLC25A19	22952000	INFINITE
Q9H4L4	Sentrin-specific protease 3	SENP3	22923000	INFINITE
Q9BQB6	Vitamin K epoxide reductase complex subunit 1	VKORC1	22912000	INFINITE
P0C7V9;A6	Putative methyltransferase-like protein 15P1;Prob	METTL15P1;METTL	22758000	INFINITE
Q9BU61	NADH dehydrogenase [ubiquinone] 1 alpha subcor	NDUFAF3	22678000	INFINITE
Q9Y2H0	Disks large-associated protein 4	DLGAP4	22633000	INFINITE
Q8IXJ6	NAD-dependent protein deacetylase sirtuin-2	SIRT2	22605000	INFINITE
P18074	TFIIH basal transcription factor complex helicase X	ERCC2	22583000	INFINITE
Q9BYX2	TBC1 domain family member 2A	TBC1D2	22537000	INFINITE
Q9BQG2	Peroxisomal NADH pyrophosphatase NUDT12	NUDT12	22393000	INFINITE
Q96EF6	F-box only protein 17	FBXO17	22392000	INFINITE
Q9BVL4	Selenoprotein O	SELO	22340000	INFINITE
Q15527	Surfeit locus protein 2	SURF2	22329000	INFINITE
Q9UGN5	Poly [ADP-ribose] polymerase 2	PARP2	22293000	INFINITE
O14657	Torsin-1B	TOR1B	22186000	INFINITE
Q7Z6K5	Arpin	ARPIN	22178000	INFINITE
P55212	Caspase-6;Caspase-6 subunit p18;Caspase-6 subur	CASP6	22152000	INFINITE
C9JLW8	Protein FAM195B	FAM195B	22111000	INFINITE
O15047	Histone-lysine N-methyltransferase SETD1A	SETD1A	22052000	INFINITE
Q96RT1	Protein LAP2	ERBB2IP	21973000	INFINITE
O95363	Phenylalanine--tRNA ligase, mitochondrial	FARS2	21823000	INFINITE
Q8IZA0	Dyslexia-associated protein KIAA0319-like protein	KIAA0319L	21793000	INFINITE
P57764	Gasdermin-D	GSDMD	21789000	INFINITE
Q4U2R6	39S ribosomal protein L51, mitochondrial	MRPL51	21771000	INFINITE
P31641	Sodium- and chloride-dependent taurine transport	SLC6A6	21759000	INFINITE
A6NIH7	Protein unc-119 homolog B	UNC119B	21685000	INFINITE
Q9NZJ6	Ubiquinone biosynthesis O-methyltransferase, mit	COQ3	21614000	INFINITE
P52429	Diacylglycerol kinase epsilon	DGKE	21520000	INFINITE
Q15120	[Pyruvate dehydrogenase (acetyl-transferring)] kin	PDK3	21518000	INFINITE
Q9UER7	Death domain-associated protein 6	DAXX	21456000	INFINITE
Q9NVV4	Poly(A) RNA polymerase, mitochondrial	MTPAP	21409000	INFINITE

Q9H5J0	Zinc finger and BTB domain-containing protein 3	ZBTB3	21359000	INFINITE
Q9UPW5	Cytosolic carboxypeptidase 1	AGTPBP1	21235000	INFINITE
O75688	Protein phosphatase 1B	PPM1B	21220000	INFINITE
Q16773	Kynurenine--oxoglutarate transaminase 1	CCBL1	21139000	INFINITE
Q96HV5	Transmembrane protein 41A	TMEM41A	21068000	INFINITE
Q96LJ7	Dehydrogenase/reductase SDR family member 1	DHRS1	21030000	INFINITE
Q92508	Piezo-type mechanosensitive ion channel compon	PIEZO1	20938000	INFINITE
Q8N5C7	DTW domain-containing protein 1	DTWD1	20897000	INFINITE
Q9NZQ3	NCK-interacting protein with SH3 domain	NCKIPSD	20872000	INFINITE
P21953	2-oxoisovalerate dehydrogenase subunit beta, mit	BCKDHB	20868000	INFINITE
Q14409;P3	Putative glycerol kinase 3;Glycerol kinase	GK3P;GK	20838000	INFINITE
P49454	Centromere protein F	CENPF	20836000	INFINITE
Q96C00	Zinc finger and BTB domain-containing protein 9	ZBTB9	20816000	INFINITE
Q96JG6	Coiled-coil domain-containing protein 132	CCDC132	20788000	INFINITE
Q96AJ9	Vesicle transport through interaction with t-SNARE	VTI1A	20696000	INFINITE
Q9H7S9;Q9	Zinc finger protein 703;Zinc finger protein 503	ZNF703;ZNF503	20520000	INFINITE
Q9UPM8	AP-4 complex subunit epsilon-1	AP4E1	20411000	INFINITE
Q6P4I2	WD repeat-containing protein 73	WDR73	20405000	INFINITE
O75143	Autophagy-related protein 13	ATG13	20393000	INFINITE
Q7L1V2	Vacuolar fusion protein MON1 homolog B	MON1B	20364000	INFINITE
Q9P2X0	Dolichol-phosphate mannosyltransferase subunit 3	DPM3	20337000	INFINITE
O14524	Transmembrane protein 194A	TMEM194A	20297000	INFINITE
Q6PI78	Transmembrane protein 65	TMEM65	20266000	INFINITE
P42771;P42	Cyclin-dependent kinase inhibitor 2A;Cyclin-depen	CDKN2A;CDKN2B	20219000	INFINITE
Q9Y5W7	Sorting nexin-14	SNX14	20063000	INFINITE
Q96DT6	Cysteine protease ATG4C	ATG4C	19965000	INFINITE
Q9BTY2	Plasma alpha-L-fucosidase	FUCA2	19862000	INFINITE
Q08357	Sodium-dependent phosphate transporter 2	SLC20A2	19743000	INFINITE
P19532	Transcription factor E3	TFE3	19645000	INFINITE
P15735	Phosphorylase b kinase gamma catalytic chain, live	PHKG2	19615000	INFINITE
Q14562	ATP-dependent RNA helicase DHX8	DHX8	19554000	INFINITE
O75155	Cullin-associated NEDD8-dissociated protein 2	CAND2	19554000	INFINITE
Q9BT67	NEDD4 family-interacting protein 1	NDFIP1	19553000	INFINITE
Q86WC4	Osteopetrosis-associated transmembrane protein	OSTM1	19540000	INFINITE
Q9Y5B8	Nucleoside diphosphate kinase 7	NME7	19523000	INFINITE
O75179	Ankyrin repeat domain-containing protein 17	ANKRD17	19490000	INFINITE
O60921	Checkpoint protein HUS1	HUS1	19455000	INFINITE
Q8NB49	Phospholipid-transporting ATPase IG	ATP11C	19453000	INFINITE
Q15051	IQ calmodulin-binding motif-containing protein 1	IQCB1	19437000	INFINITE
Q68D91	Metallo-beta-lactamase domain-containing protein	MBLAC2	19378000	INFINITE
Q9NUM4	Transmembrane protein 106B	TMEM106B	19328000	INFINITE
P38435	Vitamin K-dependent gamma-carboxylase	GGCX	19326000	INFINITE
Q8NEW0	Zinc transporter 7	SLC30A7	19224000	INFINITE
O60333	Kinesin-like protein KIF1B	KIF1B	18993000	INFINITE
Q9BX68	Histidine triad nucleotide-binding protein 2, mitocl	HINT2	18980000	INFINITE
Q6NXR4	TELO2-interacting protein 2	TTI2	18977000	INFINITE
O15554	Intermediate conductance calcium-activated potas	KCNN4	18967000	INFINITE
Q92625	Ankyrin repeat and SAM domain-containing protei	ANKS1A	18893000	INFINITE
Q7L7X3	Serine/threonine-protein kinase TAO1	TAOK1	18890000	INFINITE
P00813	Adenosine deaminase	ADA	18817000	INFINITE

Q92997	Segment polarity protein dishevelled homolog DVL	DVL3	18794000	INFINITE
O14893	Gem-associated protein 2	GEMIN2	18769000	INFINITE
P22670	MHC class II regulatory factor RFX1	RFX1	18707000	INFINITE
Q9ULK4	Mediator of RNA polymerase II transcription subur	MED23	18557000	INFINITE
Q15057	Arf-GAP with coiled-coil, ANK repeat and PH doma	ACAP2	18536000	INFINITE
Q9ULH1	Arf-GAP with SH3 domain, ANK repeat and PH dom	ASAP1	18484000	INFINITE
P15151	Poliovirus receptor	PVR	18419000	INFINITE
Q9UPY3	Endoribonuclease Dicer	DICER1	18377000	INFINITE
Q96C23	Aldose 1-epimerase	GALM	18355000	INFINITE
Q9BQ04	RNA-binding protein 4B	RBM4B	18306000	INFINITE
Q86UK7	Zinc finger protein 598	ZNF598	18102000	INFINITE
Q9UHR4	Brain-specific angiogenesis inhibitor 1-associated p	BAIAP2L1	17973000	INFINITE
Q9BWD3;C	Protein FAM127B;Protein FAM127C	FAM127B;FAM127	17924000	INFINITE
Q9H1Z4	WD repeat-containing protein 13	WDR13	17880000	INFINITE
Q9UNH6	Sorting nexin-7	SNX7	17874000	INFINITE
P55081	Microfibrillar-associated protein 1	MFAP1	17872000	INFINITE
P20248	Cyclin-A2	CCNA2	17773000	INFINITE
Q6UUV9	CREB-regulated transcription coactivator 1	CRTC1	17770000	INFINITE
Q96EU7	C1GALT1-specific chaperone 1	C1GALT1C1	17674000	INFINITE
Q8TBE7	Solute carrier family 35 member G2	SLC35G2	17649000	INFINITE
Q9NZ63	Uncharacterized protein C9orf78	C9orf78	17634000	INFINITE
Q99081	Transcription factor 12	TCF12	17625000	INFINITE
Q9Y3B6	ER membrane protein complex subunit 9	EMC9	17560000	INFINITE
Q5F1R6	DnaJ homolog subfamily C member 21	DNAJC21	17546000	INFINITE
Q9BRG1	Vacuolar protein-sorting-associated protein 25	VPS25	17524000	INFINITE
Q9UKV5	E3 ubiquitin-protein ligase AMFR	AMFR	17492000	INFINITE
Q99704	Docking protein 1	DOK1	17350000	INFINITE
Q8WVF1	Protein OSCP1	OSCP1	17224000	INFINITE
Q9UHA3	Probable ribosome biogenesis protein RLP24	RSL24D1	17219000	INFINITE
Q9Y3D0	Mitotic spindle-associated MMXD complex subunit	FAM96B	17157000	INFINITE
Q9UMZ2	Synergin gamma	SYNRG	17118000	INFINITE
Q9HAF1	Chromatin modification-related protein MEAF6	MEAF6	17106000	INFINITE
Q96K19	E3 ubiquitin-protein ligase RNF170	RNF170	17058000	INFINITE
Q9NVH6	Trimethyllysine dioxygenase, mitochondrial	TMLHE	17037000	INFINITE
Q13472	DNA topoisomerase 3-alpha	TOP3A	17032000	INFINITE
Q13541	Eukaryotic translation initiation factor 4E-binding p	EIF4EBP1	17003000	INFINITE
Q9UHL4	Dipeptidyl peptidase 2	DPP7	16961000	INFINITE
Q9NRW1	Ras-related protein Rab-6B	RAB6B	16946000	INFINITE
P82921	28S ribosomal protein S21, mitochondrial	MRPS21	16928000	INFINITE
Q8IW35	Centrosomal protein of 97 kDa	CEP97	16913000	INFINITE
P09493	Tropomyosin alpha-1 chain	TPM1	16888000	INFINITE
Q7Z7N9	Transmembrane protein 179B	TMEM179B	16863000	INFINITE
O60238	BCL2/adenovirus E1B 19 kDa protein-interacting pr	BNIP3L	16756000	INFINITE
P49427	Ubiquitin-conjugating enzyme E2 R1	CDC34	16744000	INFINITE
Q9H3Q1	Cdc42 effector protein 4	CDC42EP4	16661000	INFINITE
Q9BZQ6	ER degradation-enhancing alpha-mannosidase-like	EDEM3	16652000	INFINITE
Q9NWW5	Ceroid-lipofuscinosis neuronal protein 6	CLN6	16640000	INFINITE
Q9NQ89	Protein C12orf4	C12orf4	16605000	INFINITE
Q9BVC5	Ashwin	C2orf49	16597000	INFINITE
Q99717;Q1	Mothers against decapentaplegic homolog 5;Moth	SMAD5;SMAD1	16502000	INFINITE

Q6IPR3	tRNA wybutosine-synthesizing protein 3 homolog	TYW3	16386000	INFINITE
Q14BN4	Sarcolemmal membrane-associated protein	SLMAP	16357000	INFINITE
Q7L5Y9	Macrophage erythroblast attacher	MAEA	16342000	INFINITE
Q9NR77	Peroxisomal membrane protein 2	PXMP2	16322000	INFINITE
Q562E7	WD repeat-containing protein 81	WDR81	16277000	INFINITE
Q9Y6X4	Soluble lamin-associated protein of 75 kDa	FAM169A	16213000	INFINITE
Q96G74	OTU domain-containing protein 5	OTUD5	16135000	INFINITE
O43286	Beta-1,4-galactosyltransferase 5	B4GALT5	16088000	INFINITE
Q96QA5	Gasdermin-A	GSDMA	16068000	INFINITE
A0A1B0GTU1			16038000	INFINITE
Q9P2K8	Eukaryotic translation initiation factor 2-alpha kina	EIF2AK4	15957000	INFINITE
P58557	Putative ribonuclease	YBEY	15924000	INFINITE
Q96P11	Probable 28S rRNA (cytosine-C(5))-methyltransfera	NSUN5	15909000	INFINITE
Q7RTS9	Dymeclin	DYM	15874000	INFINITE
Q8TBY8	Polyamine-modulated factor 1-binding protein 1	PMFBP1	15806000	INFINITE
Q5VW38	Protein GPR107	GPR107	15804000	INFINITE
Q8N8R5	UPF0565 protein C2orf69	C2orf69	15788000	INFINITE
Q9BSC4	Nucleolar protein 10	NOL10	15671000	INFINITE
A0A1B0GV	TRPM8 channel-associated factor 2	TCAF2	15547000	INFINITE
Q00587	Cdc42 effector protein 1	CDC42EP1	15514000	INFINITE
Q8NFH5	Nucleoporin NUP53	NUP35	15507000	INFINITE
Q5VVQ6	Ubiquitin thioesterase OTU1	YOD1	15483000	INFINITE
Q9H1Y0	Autophagy protein 5	ATG5	15439000	INFINITE
Q5MIZ7	Serine/threonine-protein phosphatase 4 regulator	SMEK2	15396000	INFINITE
O94763	Unconventional prefoldin RPB5 interactor 1	URI1	15329000	INFINITE
Q969Q5	Ras-related protein Rab-24	RAB24	15280000	INFINITE
Q8WXH0	Nesprin-2	SYNE2	15269000	INFINITE
Q9BQI0	Allograft inflammatory factor 1-like	AIF1L	15223000	INFINITE
Q96T23	Remodeling and spacing factor 1	RSF1	15217000	INFINITE
Q9BW19	Kinesin-like protein KIFC1	KIFC1	15189000	INFINITE
Q9NX00	Transmembrane protein 160	TMEM160	15143000	INFINITE
Q6QNY0	Biogenesis of lysosome-related organelles comple	BLOC1S3	15077000	INFINITE
Q13416	Origin recognition complex subunit 2	ORC2	15042000	INFINITE
Q8NI36	WD repeat-containing protein 36	WDR36	15037000	INFINITE
Q9P2X3	Protein IMPACT	IMPACT	14963000	INFINITE
Q9NS91	E3 ubiquitin-protein ligase RAD18	RAD18	14958000	INFINITE
Q6BDS2	UHRF1-binding protein 1	UHRF1BP1	14898000	INFINITE
Q9BTX7	Alpha-tocopherol transfer protein-like	TTPAL	14881000	INFINITE
Q12884	Prolyl endopeptidase FAP;Antiplasmin-cleaving en	FAP	14862000	INFINITE
Q6VN20	Ran-binding protein 10	RANBP10	14792000	INFINITE
O95210	Starch-binding domain-containing protein 1	STBD1	14779000	INFINITE
Q96Q45	Transmembrane protein 237	TMEM237	14762000	INFINITE
Q9H6U8	Alpha-1,2-mannosyltransferase ALG9	ALG9	14719000	INFINITE
P32780	General transcription factor IIH subunit 1	GTF2H1	14640000	INFINITE
P50336	Protoporphyrinogen oxidase	PPOX	14608000	INFINITE
Q9Y653	G-protein coupled receptor 56;GPR56 N-terminal f	GPR56	14547000	INFINITE
Q53S33	BolA-like protein 3	BOLA3	14523000	INFINITE
Q8NHV4	Protein NEDD1	NEDD1	14459000	INFINITE
Q9Y3Q3	Transmembrane emp24 domain-containing protei	TMED3	14446000	INFINITE
Q6ZSJ8	Uncharacterized protein C1orf122	C1orf122	14444000	INFINITE

Q99735	Microsomal glutathione S-transferase 2	MGST2	14311000	INFINITE
Q8IW45	ATP-dependent (S)-NAD(P)H-hydrate dehydratase	CARKD	14115000	INFINITE
Q9UM13	Anaphase-promoting complex subunit 10	ANAPC10	14108000	INFINITE
O15541	RING finger protein 113A	RNF113A	14097000	INFINITE
Q9Y448	Small kinetochore-associated protein	KNSTRN	14050000	INFINITE
Q8TEB9	Rhomboid-related protein 4	RHBDD1	14041000	INFINITE
Q96BN8	Ubiquitin thioesterase otulin	OTULIN	14014000	INFINITE
Q5T8D3	Acyl-CoA-binding domain-containing protein 5	ACBD5	14004000	INFINITE
O75530	Polycomb protein EED	EED	13964000	INFINITE
Q96BN2	Transcriptional adapter 1	TADA1	13918000	INFINITE
Q9NZ43	Vesicle transport protein USE1	USE1	13889000	INFINITE
Q6ZRQ5	Protein MMS22-like	MMS22L	13881000	INFINITE
Q8N138	ORM1-like protein 3	ORMDL3	13821000	INFINITE
Q96RF0	Sorting nexin-18	SNX18	13796000	INFINITE
P13686	Tartrate-resistant acid phosphatase type 5	ACP5	13787000	INFINITE
Q9Y619	Mitochondrial ornithine transporter 1	SLC25A15	13768000	INFINITE
Q6AI08	HEAT repeat-containing protein 6	HEATR6	13728000	INFINITE
Q7Z5H3	Rho GTPase-activating protein 22	ARHGAP22	13726000	INFINITE
P51606	N-acylglucosamine 2-epimerase	RENBP	13699000	INFINITE
P01594;P0	Ig kappa chain V-I region AU;Ig kappa chain V-I region AG		13699000	INFINITE
Q9BRS2	Serine/threonine-protein kinase RIO1	RIOK1	13543000	INFINITE
Q4VCS5;Q8	Angiomotin;Angiomotin-like protein 1	AMOT;AMOTL1	13478000	INFINITE
Q9UBS8	E3 ubiquitin-protein ligase RNF14	RNF14	13384000	INFINITE
P54646	5-AMP-activated protein kinase catalytic subunit a	PRKAA2	13348000	INFINITE
Q96C11	FGGY carbohydrate kinase domain-containing prot	FGGY	13320000	INFINITE
Q7Z4F1	Low-density lipoprotein receptor-related protein 1	LRP10	13140000	INFINITE
Q2VPB7	AP-5 complex subunit beta-1	AP5B1	13038000	INFINITE
P0CG08;B7	Golgi pH regulator B;Golgi pH regulator A	GPR89B;GPR89A	12987000	INFINITE
Q9UJK0	Ribosome biogenesis protein TSR3 homolog	TSR3	12982000	INFINITE
Q9H6Y2	WD repeat-containing protein 55	WDR55	12969000	INFINITE
O43759	Synaptogyrin-1	SYNGR1	12939000	INFINITE
Q9H1A3	Methyltransferase-like protein 9	METTL9	12889000	INFINITE
Q00653	Nuclear factor NF-kappa-B p100 subunit;Nuclear fa	NFKB2	12875000	INFINITE
Q96JC1	Vam6/Vps39-like protein	VPS39	12736000	INFINITE
O94788	Retinal dehydrogenase 2	ALDH1A2	12730000	INFINITE
Q15274	Nicotinate-nucleotide pyrophosphorylase [carboxy	QPRT	12715000	INFINITE
P38432	Coilin	COIL	12680000	INFINITE
P35367	Histamine H1 receptor	HRH1	12661000	INFINITE
Q86UA6	RPA-interacting protein	RPAIN	12649000	INFINITE
Q96ES7	SAGA-associated factor 29 homolog	CCDC101	12567000	INFINITE
Q86V85	Integral membrane protein GPR180	GPR180	12502000	INFINITE
Q8TDM6	Disks large homolog 5	DLG5	12437000	INFINITE
Q9BT88	Synaptotagmin-11	SYT11	12364000	INFINITE
Q96RU3	Formin-binding protein 1	FNBP1	12318000	INFINITE
Q9UBL6	Copine-7	CPNE7	12290000	INFINITE
O75943	Cell cycle checkpoint protein RAD17	RAD17	12144000	INFINITE
P52564	Dual specificity mitogen-activated protein kinase k	MAP2K6	12014000	INFINITE
A4D1P6	WD repeat-containing protein 91	WDR91	11982000	INFINITE
Q8IXW5	Putative RNA polymerase II subunit B1 CTD phospl	RPAP2	11890000	INFINITE
Q9UK73	Protein fem-1 homolog B	FEM1B	11861000	INFINITE

P11274	Breakpoint cluster region protein	BCR	11802000	INFINITE
Q8NEZ2	Vacuolar protein sorting-associated protein 37A	VPS37A	11691000	INFINITE
O43353	Receptor-interacting serine/threonine-protein kina	RIPK2	11683000	INFINITE
Q53FT3	Protein Hikeshi	C11orf73	11640000	INFINITE
O14772	Fucose-1-phosphate guanylyltransferase	FPGT	11503000	INFINITE
Q8N5A5	Zinc finger CCCH-type with G patch domain-contai	ZGPAT	11368000	INFINITE
Q96PV6	Leukocyte receptor cluster member 8	LENG8	11348000	INFINITE
Q9Y6M5	Zinc transporter 1	SLC30A1	11301000	INFINITE
Q8N3P4	Vacuolar protein sorting-associated protein 8 hom	VPS8	11234000	INFINITE
Q9H173	Nucleotide exchange factor SIL1	SIL1	11212000	INFINITE
Q9NXG6	Transmembrane prolyl 4-hydroxylase	P4HTM	11194000	INFINITE
Q96B02	Ubiquitin-conjugating enzyme E2 W	UBE2W	11186000	INFINITE
Q969Y2	tRNA modification GTPase GTPBP3, mitochondrial	GTPBP3	11148000	INFINITE
O43826	Glucose-6-phosphate translocase	SLC37A4	11109000	INFINITE
P54252	Ataxin-3	ATXN3	11076000	INFINITE
Q9Y248	DNA replication complex GINS protein PSF2	GINS2	11049000	INFINITE
Q14966	Zinc finger protein 638	ZNF638	10991000	INFINITE
Q9Y2S2	Lambda-crystallin homolog	CRYL1	10990000	INFINITE
Q9H6U6	Breast carcinoma-amplified sequence 3	BCAS3	10978000	INFINITE
Q5HYI7	Metaxin-3	MTX3	10956000	INFINITE
Q49A17	Polypeptide N-acetylgalactosaminyltransferase-like	GALNTL6	10898000	INFINITE
A5PLN9	Trafficking protein particle complex subunit 13	TRAPPC13	10879000	INFINITE
Q15814	Tubulin-specific chaperone C	TBCC	10839000	INFINITE
Q9NZN5	Rho guanine nucleotide exchange factor 12	ARHGEF12	10819000	INFINITE
Q5SRE7	Phytanoyl-CoA dioxygenase domain-containing pro	PHYHD1	10787000	INFINITE
P01112	GTPase HRas;GTPase HRas, N-terminally processed	HRAS	10702000	INFINITE
O75864	Protein phosphatase 1 regulatory subunit 37	PPP1R37	10657000	INFINITE
Q9BSE5	Agmatinase, mitochondrial	AGMAT	10657000	INFINITE
Q99943	1-acyl-sn-glycerol-3-phosphate acyltransferase alp	AGPAT1	10622000	INFINITE
Q8WVQ1	Soluble calcium-activated nucleotidase 1	CANT1	10604000	INFINITE
O43301	Heat shock 70 kDa protein 12A	HSPA12A	10590000	INFINITE
Q969S2	Endonuclease 8-like 2	NEIL2	10581000	INFINITE
Q9UKT5	F-box only protein 4	FBXO4	10547000	INFINITE
P26374	Rab proteins geranylgeranyltransferase componen	CHML	10494000	INFINITE
P05090	Apolipoprotein D	APOD	10392000	INFINITE
Q96JQ2	Calmin	CLMN	10378000	INFINITE
Q9NUP7	tRNA:m(4)X modification enzyme TRM13 homolog	TRMT13	10357000	INFINITE
Q9NWH9	SAFB-like transcription modulator	SLTM	10355000	INFINITE
O75379	Vesicle-associated membrane protein 4	VAMP4	10342000	INFINITE
Q6PJ69	Tripartite motif-containing protein 65	TRIM65	10338000	INFINITE
Q96QG7	Myotubularin-related protein 9	MTMR9	10314000	INFINITE
P01040	Cystatin-A;Cystatin-A, N-terminally processed	CSTA	10288000	INFINITE
Q14574	Desmocollin-3	DSC3	10248000	INFINITE
Q9UJC5	SH3 domain-binding glutamic acid-rich-like protein	SH3BGRL2	10211000	INFINITE
Q9H7E9	UPF0488 protein C8orf33	C8orf33	10188000	INFINITE
Q8N573	Oxidation resistance protein 1	OXR1	10145000	INFINITE
P34741	Syndecan-2	SDC2	10119000	INFINITE
Q96EP0	E3 ubiquitin-protein ligase RNF31	RNF31	10055000	INFINITE
Q9UDY4	DnaJ homolog subfamily B member 4	DNAJB4	10055000	INFINITE
Q53TN4	Cytochrome b reductase 1	CYBRD1	10022000	INFINITE

O43741	5-AMP-activated protein kinase subunit beta-2	PRKAB2	9987000	INFINITE
Q6P3X3	Tetratricopeptide repeat protein 27	TTC27	9966100	INFINITE
Q9NV35	Probable 8-oxo-dGTP diphosphatase NUDT15	NUDT15	9958300	INFINITE
Q7Z5R6	Amyloid beta A4 precursor protein-binding family I	APBB1IP	9887800	INFINITE
P51843	Nuclear receptor subfamily 0 group B member 1	NR0B1	9870800	INFINITE
Q9BRX5	DNA replication complex GINS protein PSF3	GINS3	9866600	INFINITE
Q9H8H2	Probable ATP-dependent RNA helicase DDX31	DDX31	9864700	INFINITE
O43572	A-kinase anchor protein 10, mitochondrial	AKAP10	9855500	INFINITE
Q9H4I9	Essential MCU regulator, mitochondrial	SMDT1	9842600	INFINITE
Q96MG8	Protein-L-isoaspartate O-methyltransferase domai	PCMTD1	9830000	INFINITE
O76075	DNA fragmentation factor subunit beta	DFFB	9823600	INFINITE
Q9BQL6	Fermitin family homolog 1	FERMT1	9762800	INFINITE
O43164	E3 ubiquitin-protein ligase Praja-2	PJA2	9751800	INFINITE
Q6NT16	MFS-type transporter SLC18B1	SLC18B1	9739400	INFINITE
Q05655	Protein kinase C delta type;Protein kinase C delta t	PRKCD	9730400	INFINITE
Q9NPH0	Lysophosphatidic acid phosphatase type 6	ACP6	9705600	INFINITE
Q07820	Induced myeloid leukemia cell differentiation prot	MCL1	9702400	INFINITE
Q9H7F4	Transmembrane protein 185B	TMEM185B	9584200	INFINITE
P05423	DNA-directed RNA polymerase III subunit RPC4	POLR3D	9584200	INFINITE
Q9C0B7	Transport and Golgi organization protein 6 homolo	TANGO6	9519100	INFINITE
Q96EX1	Small integral membrane protein 12	SMIM12	9465200	INFINITE
P36897	TGF-beta receptor type-1	TGFBR1	9434500	INFINITE
Q8IX05	CD302 antigen	CD302	9344800	INFINITE
Q8TCG2	Phosphatidylinositol 4-kinase type 2-beta	PI4K2B	9325600	INFINITE
Q12955	Ankyrin-3	ANK3	9325100	INFINITE
Q8NCM8	Cytoplasmic dynein 2 heavy chain 1	DYNC2H1	9322700	INFINITE
P29590	Protein PML	PML	9295100	INFINITE
Q5VU43	Myomegalin	PDE4DIP	9244200	INFINITE
Q9P2W1	Homologous-pairing protein 2 homolog	PSMC3IP	9239600	INFINITE
Q9NUJ3	T-complex protein 11-like protein 1	TCP11L1	9174200	INFINITE
Q14807	Kinesin-like protein KIF22	KIF22	8979900	INFINITE
O15379	Histone deacetylase 3	HDAC3	8965200	INFINITE
Q9BSL1	Ubiquitin-associated domain-containing protein 1	UBAC1	8929400	INFINITE
P25445	Tumor necrosis factor receptor superfamily memb	FAS	8892800	INFINITE
Q86VN1	Vacuolar protein-sorting-associated protein 36	VPS36	8865600	INFINITE
Q14692	Ribosome biogenesis protein BMS1 homolog	BMS1	8779100	INFINITE
A3KN83;Q9	Protein strawberry notch homolog 1;Protein straw	SBNO1;SBNO2	8729900	INFINITE
Q96BY7;Q2	Autophagy-related protein 2 homolog B;Autophag	ATG2B;ATG2A	8688800	INFINITE
Q5R372	Rab GTPase-activating protein 1-like	RABGAP1L	8631000	INFINITE
Q8TDI0	Chromodomain-helicase-DNA-binding protein 5	CHD5	8568900	INFINITE
Q9P1Z2	Calcium-binding and coiled-coil domain-containing	CALCOCO1	8506800	INFINITE
Q5VZL5	Zinc finger MYM-type protein 4	ZMYM4	8455700	INFINITE
Q99707	Methionine synthase	MTR	8435800	INFINITE
Q8N5I4	Dehydrogenase/reductase SDR family member on	DHRSX	8371600	INFINITE
Q9H3F6	BTB/POZ domain-containing adapter for CUL3-me	KCTD10	8318700	INFINITE
Q9BXW6	Oxysterol-binding protein-related protein 1	OSBPL1A	8284300	INFINITE
Q6KC79	Nipped-B-like protein	NIPBL	8229200	INFINITE
Q15007	Pre-mRNA-splicing regulator WTAP	WTAP	8178800	INFINITE
P50548	ETS domain-containing transcription factor ERF	ERF	8141000	INFINITE
Q9H6B4	CXADR-like membrane protein	CLMP	8044000	INFINITE

Q9NVM6	DnaJ homolog subfamily C member 17	DNAJC17	8029700	INFINITE
Q9ULE4	Protein FAM184B	FAM184B	7973100	INFINITE
Q16610	Extracellular matrix protein 1	ECM1	7853100	INFINITE
Q9H4B0	Probable tRNA N6-adenosine threonylcarbamoyltr	OSGEPL1	7815400	INFINITE
Q00534	Cyclin-dependent kinase 6	CDK6	7705500	INFINITE
Q96JI7	Spatacsin	SPG11	7623500	INFINITE
Q5TF21	Protein SOGA3	SOGA3	7622200	INFINITE
Q6DKK2	Tetratricopeptide repeat protein 19, mitochondria	TTC19	7572500	INFINITE
P28749	Retinoblastoma-like protein 1	RBL1	7550700	INFINITE
Q9BQE4	Selenoprotein S	VIMP	7538600	INFINITE
Q5TGY1	Transmembrane and coiled-coil domain-containing	TMCO4	7511000	INFINITE
P22735	Protein-glutamine gamma-glutamyltransferase K	TGM1	7457200	INFINITE
P36222	Chitinase-3-like protein 1	CHI3L1	7426900	INFINITE
P27482	Calmodulin-like protein 3	CALML3	7379000	INFINITE
P07476	Involucrin	IVL	7377100	INFINITE
Q14164	Inhibitor of nuclear factor kappa-B kinase subunit ε	IKBKE	7366100	INFINITE
Q8NHS3	Major facilitator superfamily domain-containing pr	MFSD8	7365500	INFINITE
Q8TF71	Monocarboxylate transporter 10	SLC16A10	7359600	INFINITE
Q9NQY0	Bridging integrator 3	BIN3	7265300	INFINITE
P04196	Histidine-rich glycoprotein	HRG	7261200	INFINITE
P51451	Tyrosine-protein kinase Blk	BLK	7222900	INFINITE
O15155	BET1 homolog	BET1	7210800	INFINITE
Q8IU60	m7GpppN-mRNA hydrolase	DCP2	7192000	INFINITE
O15156	Zinc finger and BTB domain-containing protein 7B	ZBTB7B	7168900	INFINITE
Q9Y2K2	Serine/threonine-protein kinase SIK3	SIK3	7165300	INFINITE
Q92503	SEC14-like protein 1	SEC14L1	7040900	INFINITE
O15381	Nuclear valosin-containing protein-like	NVL	6958400	INFINITE
Q92968	Peroxisomal membrane protein PEX13	PEX13	6899800	INFINITE
O14682	Ectoderm-neural cortex protein 1	ENC1	6847900	INFINITE
Q9NSV4	Protein diaphanous homolog 3	DIAPH3	6839200	INFINITE
P54098	DNA polymerase subunit gamma-1	POLG	6821800	INFINITE
Q14676	Mediator of DNA damage checkpoint protein 1	MDC1	6812200	INFINITE
O75554	WW domain-binding protein 4	WBP4	6762000	INFINITE
P01034	Cystatin-C	CST3	6760400	INFINITE
Q02040	A-kinase anchor protein 17A	AKAP17A	6725900	INFINITE
Q5VSL9	Striatin-interacting protein 1	STRIP1	6698500	INFINITE
P35754	Glutaredoxin-1	GLRX	6616300	INFINITE
Q96FZ2	Embryonic stem cell-specific 5-hydroxymethylcyto:	HMCES	6589700	INFINITE
Q9UKG9	Peroxisomal carnitine O-octanoyltransferase	CROT	6527300	INFINITE
Q8TEU7	Rap guanine nucleotide exchange factor 6	RAPGEF6	6523100	INFINITE
Q9Y2G8	DnaJ homolog subfamily C member 16	DNAJC16	6482200	INFINITE
Q9P2G1	Ankyrin repeat and IBR domain-containing protein	ANKIB1	6400700	INFINITE
Q6JQN1	Acyl-CoA dehydrogenase family member 10	ACAD10	6353700	INFINITE
Q8N7R7	Cyclin-Y-like protein 1	CCNYL1	6331900	INFINITE
Q6P1R4	tRNA-dihydrouridine(16/17) synthase [NAD(P)(+)]-	DUS1L	6276100	INFINITE
Q8TBK6	Zinc finger CCHC domain-containing protein 10	ZCCHC10	6171300	INFINITE
Q96DF8	Protein DGCR14	DGCR14	6106600	INFINITE
O00635	E3 ubiquitin-protein ligase TRIM38	TRIM38	6055200	INFINITE
Q01432	AMP deaminase 3	AMPD3	6048400	INFINITE
Q9Y3A4	Ribosomal RNA-processing protein 7 homolog A	RRP7A	6037400	INFINITE

Q9UJX6	Anaphase-promoting complex subunit 2	ANAPC2	6012800	INFINITE
O76024	Wolframin	WFS1	6004300	INFINITE
P78332	RNA-binding protein 6	RBM6	5970400	INFINITE
P18440	Arylamine N-acetyltransferase 1	NAT1	5956100	INFINITE
Q14687	Genetic suppressor element 1	GSE1	5911700	INFINITE
P61244	Protein max	MAX	5888100	INFINITE
Q56A73	Spindlin-4	SPIN4	5861800	INFINITE
Q8N302	Angiogenic factor with G patch and FHA domains 1	AGGF1	5847200	INFINITE
Q86WQ0	Nuclear receptor 2C2-associated protein	NR2C2AP	5802300	INFINITE
Q9NYL4	Peptidyl-prolyl cis-trans isomerase FKBP11	FKBP11	5781400	INFINITE
Q9H871	Protein RMD5 homolog A	RMND5A	5764000	INFINITE
O95319	CUGBP Elav-like family member 2	CELF2	5745900	INFINITE
O60507	Protein-tyrosine sulfotransferase 1	TPST1	5640500	INFINITE
P04259	Keratin, type II cytoskeletal 6B	KRT6B	5606400	INFINITE
Q9UPN4	Centrosomal protein of 131 kDa	CEP131	5543200	INFINITE
Q9BT09	Protein canopy homolog 3	CNPY3	5534600	INFINITE
Q9H467	CUE domain-containing protein 2	CUEDC2	5506800	INFINITE
Q86X29	Lipolysis-stimulated lipoprotein receptor	LSR	5442200	INFINITE
Q13835	Plakophilin-1	PKP1	5416600	INFINITE
Q9H1J1	Regulator of nonsense transcripts 3A	UPF3A	5294600	INFINITE
Q9HAU0	Pleckstrin homology domain-containing family A m	PLEKHA5	5200800	INFINITE
Q30154	HLA class II histocompatibility antigen, DR beta 5 c	HLA-DRB5	5177700	INFINITE
Q8TEV9	Smith-Magenis syndrome chromosomal region car	SMCR8	5156300	INFINITE
P60880	Synaptosomal-associated protein 25	SNAP25	5100000	INFINITE
O95619	YEATS domain-containing protein 4	YEATS4	5036600	INFINITE
Q96D09	G-protein coupled receptor-associated sorting pro	GPRASP2	5029400	INFINITE
Q96PZ2	Protein FAM111A	FAM111A	5016200	INFINITE
P37198	Nuclear pore glycoprotein p62	NUP62	5001600	INFINITE
Q12933	TNF receptor-associated factor 2	TRAF2	4920400	INFINITE
O43318	Mitogen-activated protein kinase kinase kinase 7	MAP3K7	4883000	INFINITE
O14681	Etoposide-induced protein 2.4 homolog	EI24	4831500	INFINITE
Q96GM5	SWI/SNF-related matrix-associated actin-depende	SMARCD1	4781000	INFINITE
Q6NXT6	Transmembrane anterior posterior transformation	TAPT1	4618200	INFINITE
P17482	Homeobox protein Hox-B9	HOXB9	4614200	INFINITE
Q9NPJ6	Mediator of RNA polymerase II transcription subur	MED4	4599200	INFINITE
Q96NX9;Q9	Dachshund homolog 2;Dachshund homolog 1	DACH2;DACH1	4563300	INFINITE
Q9HCY8	Protein S100-A14	S100A14	4519000	INFINITE
Q12983	BCL2/adenovirus E1B 19 kDa protein-interacting p	BNIP3	4461200	INFINITE
O75528	Transcriptional adapter 3	TADA3	4410200	INFINITE
P07948;P08	Tyrosine-protein kinase Lyn;Tyrosine-protein kinas	LYN;HCK	4399100	INFINITE
Q5BKX5	UPF0692 protein C19orf54	C19orf54	4202500	INFINITE
Q96RU2	Ubiquitin carboxyl-terminal hydrolase 28	USP28	4186400	INFINITE
Q9BVS5	tRNA (adenine(58)-N(1))-methyltransferase, mitoc	TRMT61B	4156000	INFINITE
Q96HJ9	UPF0562 protein C7orf55	C7orf55	4154300	INFINITE
P01833	Polymeric immunoglobulin receptor;Secretory com	PIGR	4145700	INFINITE
Q02338	D-beta-hydroxybutyrate dehydrogenase, mitochor	BDH1	4065600	INFINITE
Q9BXS4	Transmembrane protein 59	TMEM59	4003000	INFINITE
Q9BVJ7	Dual specificity protein phosphatase 23	DUSP23	3959500	INFINITE
Q2TAM9	Tumor suppressor candidate gene 1 protein	TUSC1	3889600	INFINITE
P52657	Transcription initiation factor IIA subunit 2	GTF2A2	3660200	INFINITE

Q8N4C8	Misshapen-like kinase 1	MINK1	3410000	INFINITE
O60279	Sushi domain-containing protein 5	SUSD5	3362100	INFINITE
O60551	Glycylpeptide N-tetradecanoyltransferase 2	NMT2	3204700	INFINITE
Q9BPX6	Calcium uptake protein 1, mitochondrial	MICU1	3170100	INFINITE
Q9UL40	Zinc finger protein 346	ZNF346	3083700	INFINITE
P20226;Q6	TATA-box-binding protein;TATA box-binding prote	TBP;TBPL2	2899700	INFINITE
Q8N2M8	CLK4-associating serine/arginine rich protein	CLASRP	2817000	INFINITE
Q96N21	AP-4 complex accessory subunit tepsin	ENTHD2	2659200	INFINITE
Q9UI43	rRNA methyltransferase 2, mitochondrial	FTSJ2	2527900	INFINITE
O43293	Death-associated protein kinase 3	DAPK3	2467400	INFINITE
Q9UIG0	Tyrosine-protein kinase BAZ1B	BAZ1B	2436600	INFINITE
Q6ZVX7	F-box only protein 50	NCCRP1	2349000	INFINITE
Q6IPR1	LYR motif-containing protein 5	LYRM5	2024200	INFINITE
Q9HA92	Radical S-adenosyl methionine domain-containing	RSAD1	1913500	INFINITE
Q9NZM5	Glioma tumor suppressor candidate region gene 2	GLTSCR2	1746000	INFINITE
O43240	Kallikrein-10	KLK10	1660800	INFINITE
Q8TDX5	2-amino-3-carboxymuconate-6-semialdehyde deca	ACMSD	1181800	INFINITE
O00287	Regulatory factor X-associated protein	RFXAP	0	
O14569	Cytochrome b561 domain-containing protein 2	CYB561D2	0	
O15116	U6 snRNA-associated Sm-like protein LSm1	LSM1	0	
O15357	Phosphatidylinositol 3,4,5-trisphosphate 5-phosph	INPPL1	0	
O43548	Protein-glutamine gamma-glutamyltransferase 5	TGM5	0	
O60331	Phosphatidylinositol 4-phosphate 5-kinase type-1	PIP5K1C	0	
O75376	Nuclear receptor corepressor 1	NCOR1	0	
P10412	Histone H1.4	HIST1H1E	0	
P32856	Syntaxin-2	STX2	0	
P41226	Ubiquitin-like modifier-activating enzyme 7	UBA7	0	
P41229	Lysine-specific demethylase 5C	KDM5C	0	
P49137	MAP kinase-activated protein kinase 2	MAPKAPK2	0	
P57768	Sorting nexin-16	SNX16	0	
Q07352	Zinc finger protein 36, C3H1 type-like 1	ZFP36L1	0	
Q53GL0	Pleckstrin homology domain-containing family O m	PLEKHO1	0	
Q53LP3	Ankyrin repeat domain-containing protein SOWAH	SOWAHC	0	
Q5SQN1	Synaptosomal-associated protein 47	SNAP47	0	
Q5SR56	Hippocampus abundant transcript-like protein 1	HIATL1	0	
Q5T1V6	Probable ATP-dependent RNA helicase DDX59	DDX59	0	
Q5T750	Skin-specific protein 32	XP32	0	
Q6B0K9	Hemoglobin subunit mu	HBM	0	
Q6P1Q9	Methyltransferase-like protein 2B	METTL2B	0	
Q86VQ3	Thioredoxin domain-containing protein 2	TXNDC2	0	
Q8IVV2	Lipoxygenase homology domain-containing protei	LOXHD1	0	
Q8IW75	Serpin A12	SERPINA12	0	
Q8IWT6	Volume-regulated anion channel subunit LRRC8A	LRRC8A	0	
Q92685	Dol-P-Man:Man(5)GlcNAc(2)-PP-Dol alpha-1,3-mar	ALG3	0	
Q96S94	Cyclin-L2	CCNL2	0	
Q9BUL5	PHD finger protein 23	PHF23	0	
Q9UL33	Trafficking protein particle complex subunit 2-like	TRAPPC2L	0	
Q9UNK0	Syntaxin-8	STX8	0	

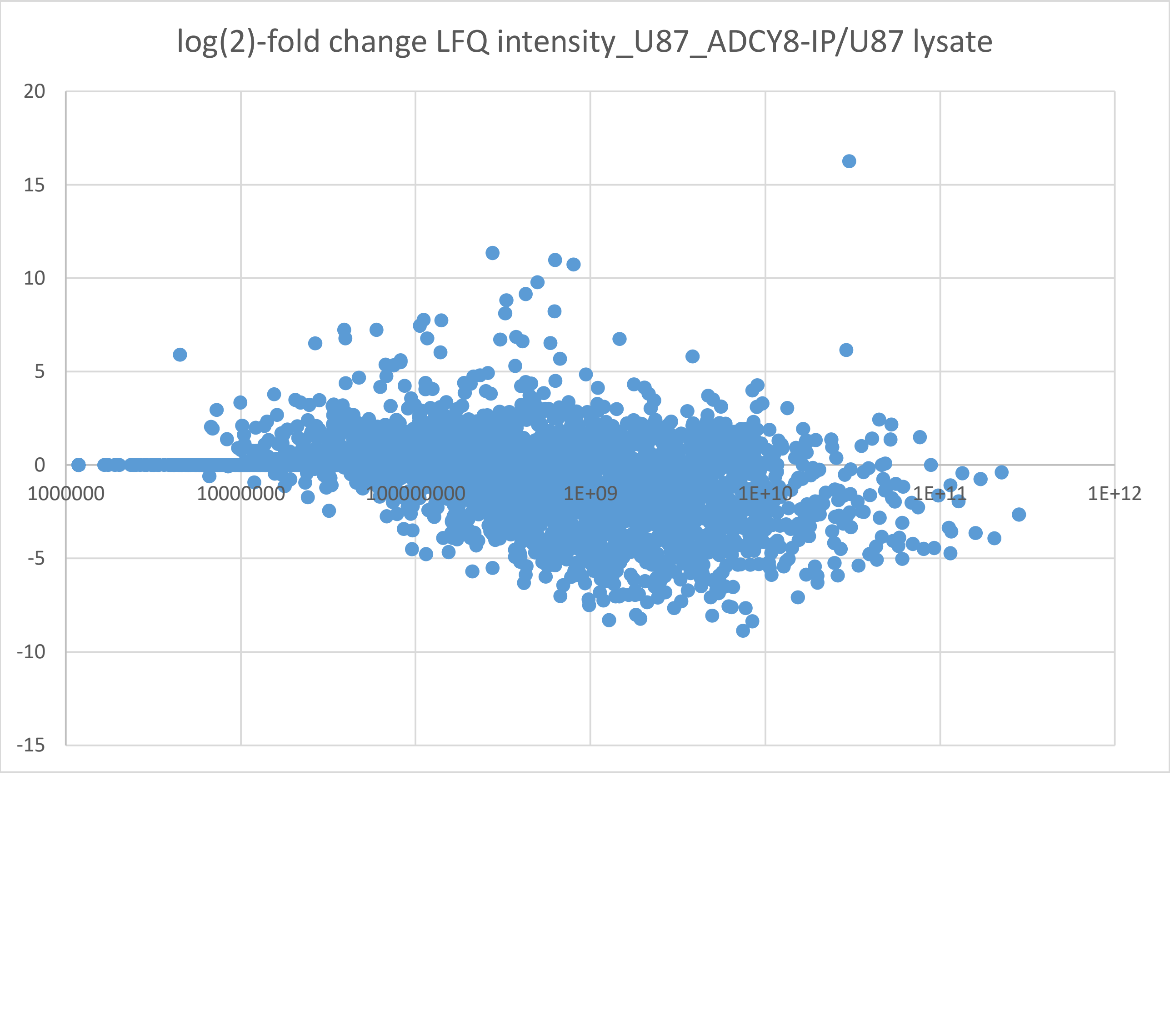

log(2)-fold change LFQ intensity_U87_ADCY8-IP/U87 lysate
20
15
10
5
0
-5
-10
-15
1000000
10000000
100000000
1E+09
1E+10
1E+11
1E+12
